# First X-ray Crystal Structure Characterization, Computational Studies, and Improved Synthetic Route to the Bioactive 5-Arylimino-1,3,4-thiadiazole Derivatives

**DOI:** 10.3390/ijms24043759

**Published:** 2023-02-13

**Authors:** Ziad Moussa, Alejandro Perez Paz, Zaher M. A. Judeh, Ahmed Alzamly, Haythem A. Saadeh, Basim H. Asghar, Sara Alsaedi, Bayan Masoud, Salama Almeqbaali, Saeda Estwani, Amna Aljaberi, Munirah M. Al-Rooqi, Saleh A. Ahmed

**Affiliations:** 1 Department of Chemistry, College of Science, United Arab Emirates University, Al Ain P.O. Box 15551, United Arab Emirates; 2 School of Chemistry, Chemical Engineering and Biotechnology, Nanyang Technological University, 62 Nanyang Drive, N1.2–B1-14, Singapore 637459, Singapore; 3 Department of Chemistry, School of Science, The University of Jordan, Amman 11942, Jordan; 4 Department of Chemistry, Faculty of Applied Sciences, Umm Al-Qura University, Makkah 21955, Saudi Arabia; 5 Department of Chemistry, Faculty of Science, Assiut University, Assiut 71516, Egypt

**Keywords:** isothiocyanate, *N*-arylcyanothioformamide, hydrazonoyl chloride, 1,3,4-thiadiazole, density functional theory

## Abstract

*N*-arylcyanothioformamides are useful coupling components for building key chemical intermediates and biologically active molecules in an expedited and efficient manner. Similarly, substituted (*Z*)-2-oxo-*N*-phenylpropanehydrazonoyl chlorides have been utilized in numerous one-step heteroannulation reactions to assemble the structural core of several different types of heterocyclic compounds. Herein, we demonstrate the effectiveness of the reaction of *N*-arylcyanothioformamides with various substituted (*Z*)-2-oxo-*N*-phenylpropanehydrazonoyl chlorides to produce, stereoselectively and regioselectively, a range of 5-arylimino-1,3,4-thiadiazole derivatives decorated with a multitude of functional groups on both aromatic rings. The synthetic methodology features mild room-temperature conditions, large substrate scope, wide array of functional groups on both reactants, and good to high reaction yields. The products were isolated by gravity filtration in all cases and structures were confirmed by multinuclear NMR spectroscopy and high accuracy mass spectral analysis. Proof of molecular structure of the isolated 5-arylimino-1,3,4-thiadiazole regioisomer was obtained for the first time by single-crystal X-ray diffraction analysis. Crystal-structure determination was carried out on (*Z*)-1-(5-((3-fluorophenyl)imino)-4-(4-iodophenyl)-4,5-dihydro-1,3,4-thiadiazol-2-yl)ethan-1-one and (*Z*)-1-(4-phenyl-5-(*p*-tolylimino)-4,5-dihydro-1,3,4-thiadiazol-2-yl)ethan-1-one. Similarly, the tautomeric structures of the *N*-arylcyanothioformamides and (*Z*)-geometries of the 2-oxo-*N*-phenylpropanehydrazonoyl chloride coupling partners were proven by X-ray diffraction studies. As representative examples, crystal-structure determination was carried out on (4-ethoxyphenyl)carbamothioyl cyanide and *(Z)-N*-(2,3-difluorophenyl)-2-oxopropanehydrazonoyl chloride. Density functional theory calculations at the B3LYP-D4/def2-TZVP level were carried out to rationalize the observed experimental findings.

## 1. Introduction

The 1,3,4-thiadiazole nucleus is highly important and one of the most well-recognized heterocyclic scaffolds, comprising a common and integral feature of various natural products and pharmaceutical agents and is applied widely in agricultural and materials chemistry [1]. The five-membered heterocyclic ring contains two nitrogen atoms and a sulfur atom. There are quite a few isomers of 1,3,4-thiadiazole including 1,2,3-thiadiazole, 1,2,4-thiadiazole, and 1,2,5-thiadiazole, although the 1,3,4-thiadiazole system remains the most investigated because it possesses multiple possible reactive sites and displays wider chemical properties. For instance, it is highly aromatic and weakly basic, and, while it is stable in aqueous acidic solutions, it is base-sensitive and can undergo ring cleavage under basic conditions [2]. Additionally, the ring is electron deficient owing to the electron-withdrawing characteristic of the two nitrogen atoms and unsusceptible to electrophilic substitution but reactive toward nucleophilic attack. Notably, activation of the 2′ or 5′ position of this ring by introducing appropriate substitutions renders these sites readily reactive, producing various derivatives. The 1,3,4-thiadiazoles show a wide spectrum of bioactivities including antifungal [3], antihypertensive [4], antimicrobial [5], anticonvulsants [6], antituberculosis [7], antidepressant and anxiolytic [8], antioxidant [9], anti-inflammatory [10], and anticancer activity [11].

**Figure 1:**
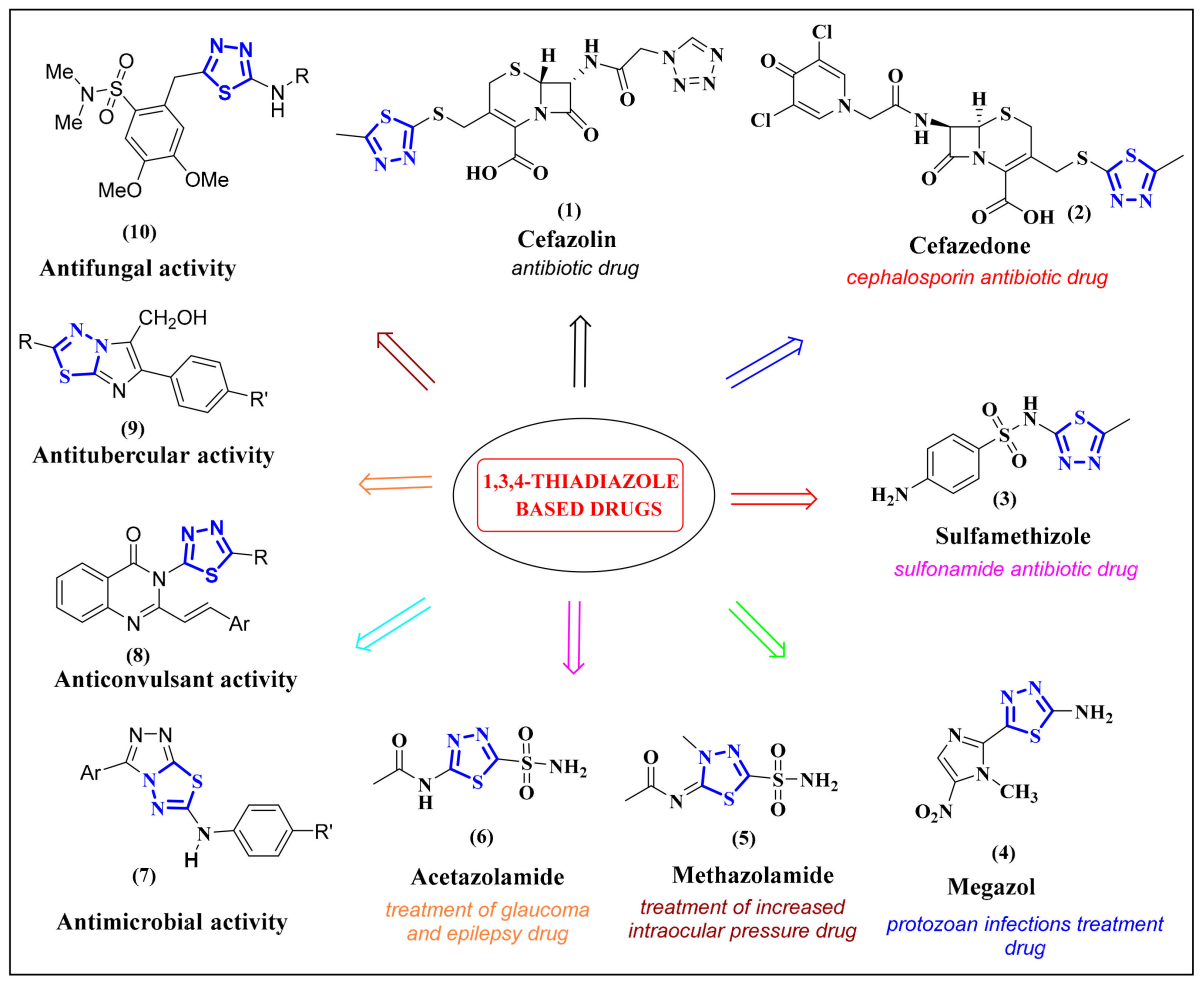
Examples of the 1,3,4-thiadiazole motif in several drugs and promising potential agents and its usefulness in chemical and bioactive agents.

To highlight the versatile utility of 1,3,4-thiadiazoles, several commercial drugs and bioactive compounds performing various biological and chemical functions have been summarized in Figure 1 (**1**–**10**). The broad-spectrum prescription drugs Cefazolin (**1**), Cefazedone (**2**), and the sulfonamide Sulfamethizole (**3**) all serve as very effective antibiotics. While Cefazolin and Cefazedone inactivate penicillin-binding proteins active in the final stages of assembling the bacterial cell wall, Sulfamethizole competes with *p*-aminobenzoic acid for the bacterial enzyme dihydropteroate synthase, thereby disrupting folic acid synthesis, ultimately leading to cell death. Megazol (**4**) is a 1,3,4-thiadiazole based drug that treats certain protozoan infections and is particularly effective against T. brucei and T. cruzi, the cause of African sleeping sickness, and Chagas disease, respectively. Whereas Methazolamide (**5**) and Acetazolamide (**6**) are both indicated in the treatment of increased intraocular pressure in glaucoma, the latter is also used to treat epilepsy. Compounds **7**–**10** are also promising bioactive heterocycles useful as antimicrobials (**7** and **10**), antitubercular (**9**) and anticonvulsant (**8**) agents.

**Scheme 1:**
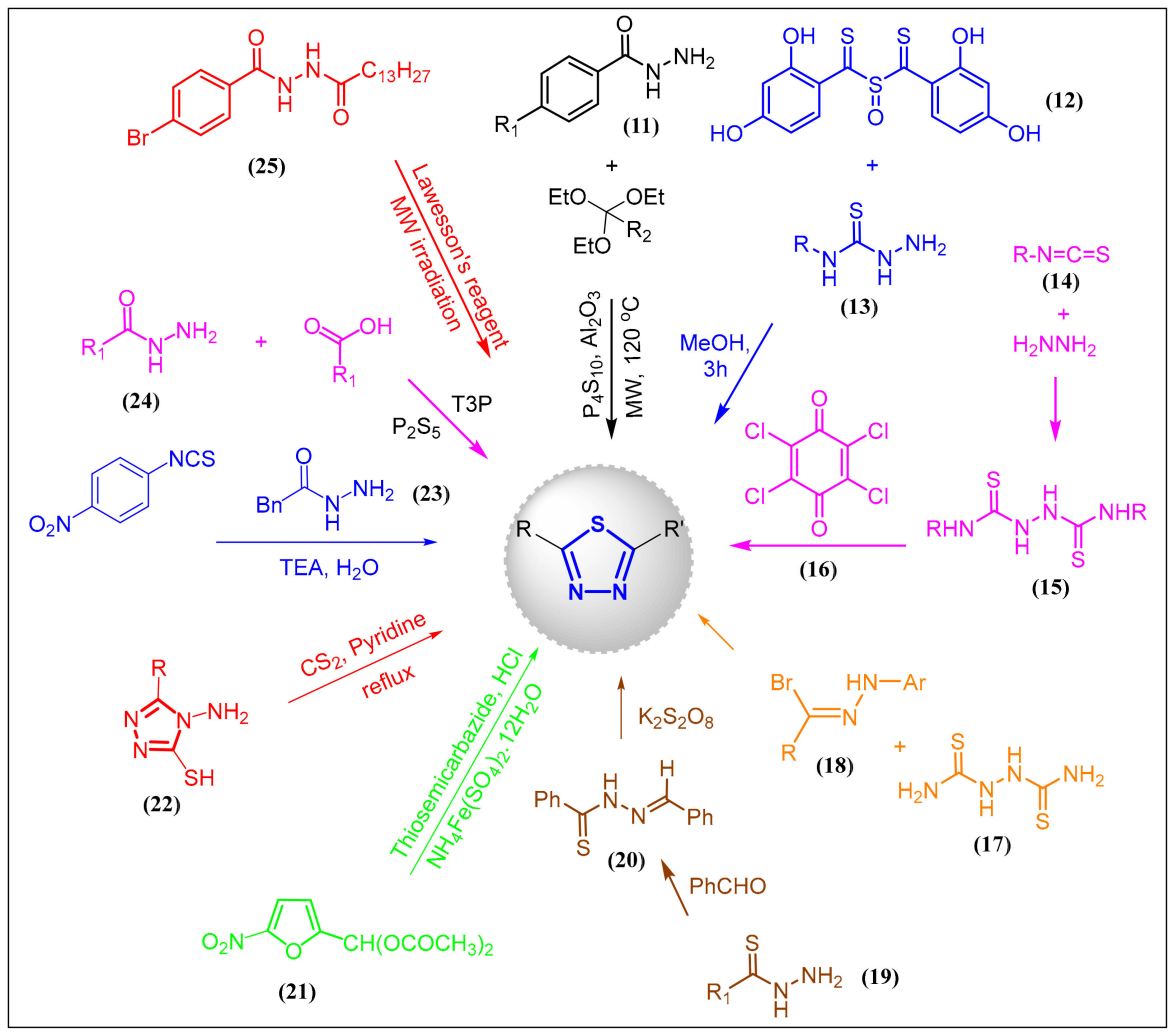
General preparation of the 1,3,4-thiadiazole nucleus from various routes and reactants.

Several types of one-pot syntheses of 1,3,4-thiadiazoles have been described in the past decades using a range of starting materials such as **11**–**25**, although some approaches are still performed under harsh conditions while others have been synthetically improved (Scheme 1). Thus, 1,3,4-thiadiazoles may be synthesized directly by Augustine’s method from acid hydrazides (**24**) and carboxylic acids using propylphosphonic anhydride (T3P) and either P_2_S_5_ or Lawesson’s reagent for thionation [12]. Polshettiwar et al. described the synthesis of 1,3,4-thiadiazoles from various aromatic heterocyclic acid hydrazides **11** and triethyl orthoformate under microwave irradiation [13]. The reaction is catalyzed by Nafion NR50 and phosphorus pentasulfide in alumina (P_4_S_10_/Al_2_O_3_). Cyclization of *N*,*N*′-diacylhydrazines is a very popular and convenient method to prepare 1,3,4-thiadiazoles. As such, the synthesis of 1,3,4-thiadiazoles from diacylhydrazines has been described starting by *N*-acylation of 4-bromobenzohydrazide with myristoyl chloride to produce unsymmetrical *N*,*N*′-diacylhydrazine **25**, which may be cyclized into the thiadiazole with a yield of 91% in 13 min using microwave irradiation and Lawesson’s reagent [1]. Treatment of sulfinyl-bis((2,4-dihydroxyphenyl)methanethione) (**12**) with substituted thiosemicarbazides **13** in methanol also affords 1,3,4-thiadiazoles and features *N*-substitution with an amine group at the 5-position [14]. Bithioureas **15**, prepared from hydrazine and isothiocyanates **14**, also act as precursors to 1,3,4-thiadiazoles when reacted with 2,3,5,6-tetrachlorocyclohexa-2,5-diene-1,4-dione (**16**), although yields range between 12 and 26% [15]. In the latter cases, reaction between the acid hydrazide and thionating agent, isothiocyanate or dithiocarbamates is always a multi-step process. Of note, isothiocyanates have been reported to directly couple with acid hydrazides such as **23** to afford 2-substituted-1,3,4-thiadiazole in the presence of water and triethylamine [16]. This reaction only proceeds under reflux conditions. The 1,3,4-thiadiazole nucleus can also be generated by the reaction of thiocarbazides **17** with hydrazonoyl halide **18** as reported by Sayed et al. [17]. Fararr et al. described the synthesis of 2,5-disubstituted-1,3,4-thiadiazoles by treating thiobenzhydrazide **19** with benzaldehyde to produce the thiohydrazide derivative **20**, followed by oxidation with potassium persulfate [18]. Many syntheses of 1,3,4-thiadiazoles originate from thiosemicarbazides, where, for instance, 5-nitro-2-furfurilidene diacetate (**21**) reacts with thiosemicarbazide and metal oxidant to generate the thiadiazole [19]. Reaction of 4-amino-5-mercapto-1,2,4-triazoles (**22**) with carbon disulfide in refluxing pyridine also renders 1,3,4-thiadiazoles as products [20].

## 2. Results and Discussions

### 2.1. Chemistry

**Scheme 2:**
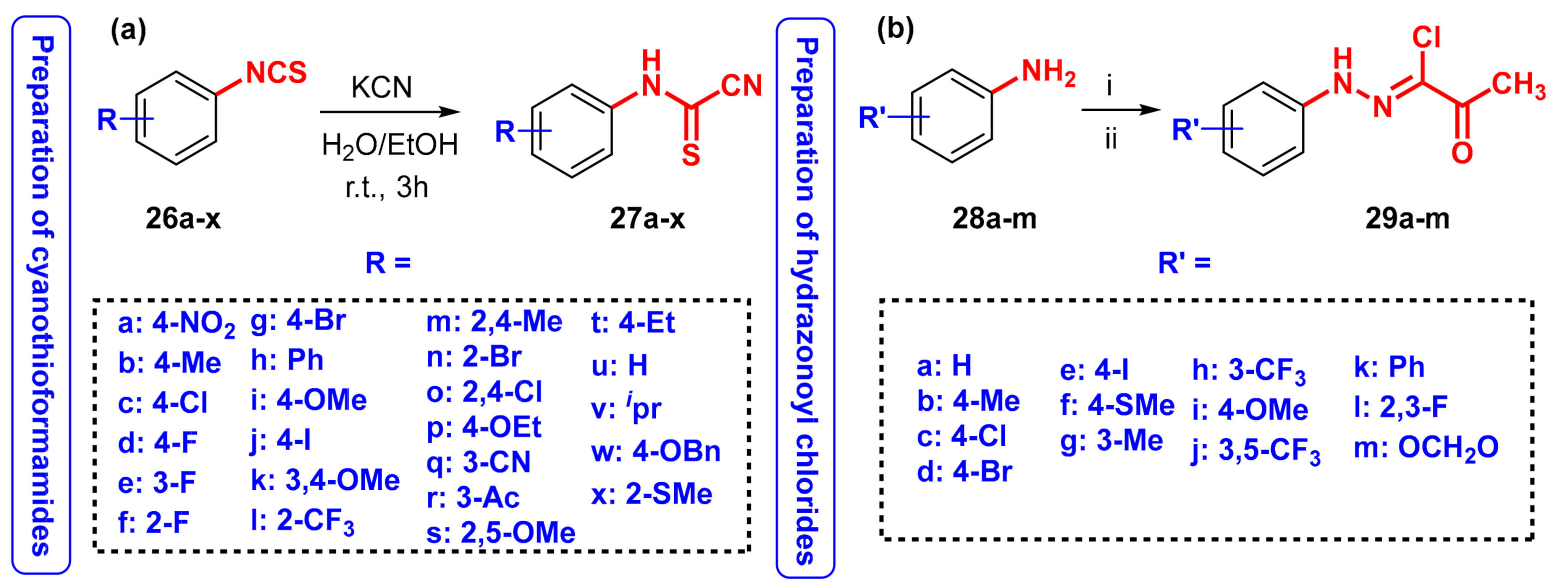
(**a**) Preparation of variously substituted *N*-arylcyanothioformamides **27a**–**x** and (**b**) hydrazonoyl chlorides **29a**–**m**. Reagents and conditions: (i) HCl/NaNO_2_/H_2_O/0–5 °C; (ii) 3-chloropentane-2,4-dione, CH_3_COONa/EtOH/0–5 °C.

Although there are several methods in the literature for the synthesis of 1,3,4-thiadiazoles as described earlier, there are no reliable protocols to prepare 5-arylimino-1,3,4-thiadiazoles and sporadic examples of these species have been reported as minor or side products. A literature search has shown that isothiocyanates [21] or *N*-arycyanothioformamides [22,23,24,25,26,27,28,29] could in principle serve as potential starting materials and sources of sulfur. However, although the former (RN=C=S) has been reported to produce 5-arylimino-1,3,4-thiadiazoles under reflux conditions (61 °C) and long reaction times (10–12 h) [21], in our hands, the same reaction failed to produce any products and the isothiocyanates remained unreactive at room temperature. As an alternative to isothiocyanates, the corresponding carbamodithioic acid derivatives were reported to generate 5-arylimino-1,3,4-thiadiazoles at room temperature when reacted with hydrazonoyl halides [30,31]. Although effective, the major drawback of this approach is the production of methanethiol which is hazardous and exhibits a putrid smell. Thus, for these reasons, we aimed to develop a novel alternative preparation protocol that offers broad substrate scope, high efficiency, and mild conditions that preclude the use of unsuitable reagents, long reaction time, and elevated reflux temperature. Mahran has also produced a few examples of 5-arylimino-1,3,4-thiadiazoles, although the use of elevated temperature and sodium ethoxide as base, which can partially destroy both starting materials by substituting the chloride or cyanide, is undesirable [32]. The suggested regiochemical (reaction via *N* or *S*) and stereochemical (*E*/*Z*) outcome of the reaction was not proven and the mechanism was also partly erroneous and not supported by any chemical or computational data. In this context, *N*-arylcyanothioformamides proved appealing as starting materials for the preparation of 1,3,4-thiadiazoles, although more extensive characterization and computational studies are warranted to provide convincing structural and mechanistic proof. Thus, pursuing this idea, several variously substituted *N*-arylcyanothioformamides **27a–x** (Scheme 2a) were prepared on 20 mmol scale from commercial isothiocyanates **26a–x** and potassium cyanide in water-ethanol in good yields (65–88%) (Scheme 2). The physical and spectral data of **27a–x** matched those reported in the literature [33]. Preparation of the hydrazonoyl chlorides coupling partners **29a–m** (Scheme 2b) was carried out according to literature methods described previously [34,35]. Accordingly, variously substituted aromatic amines **28a–m** were diazotized in 6 M hydrochloric acid with sodium nitrite and the resulting diazonium chloride salts were neutralized with sodium acetate solution, followed by the addition of 3-chloropentane-2,4-dione. In all cases, the resulting solid was collected by gravity filtration and used without further purification. All new and known hydrazonoyl chlorides were fully characterized by melting point, standard spectroscopic and analytical techniques, as well as ^1^H and ^13^C NMR spectrometry. It is noted that in all cases the NMR spectral data of the prepared known hydrazonoyl chlorides could not be compared and matched to anything described in the literature because none of the related published works described any NMR data or included spectra in the Supplementary Materials Sections [36,37,38,39,40,41,42,43]. In addition, melting point values and IR spectra were only available for three known compounds (**29f**, **29h**, **29i**) [39,41]. Therefore, this work documents for the first time all the relevant characterization data and corresponding spectra of the hydrazonoyl chlorides used herein (see the Experimental Section 3.5 and Supplementary Materials S3–S89). 

#### 2.1.1. Crystal-Structure Determination of the Hydrazonoyl Chlorides

**Figure 2:**
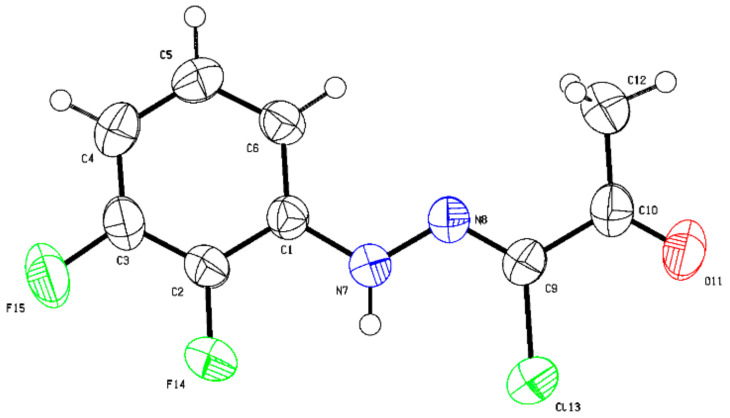
The crystal structure of *(Z)-N*-(2,3-difluorophenyl)-2-oxopropanehydrazonoyl chloride (**29l**) (Deposition Number **2179719**). For bond distances (Å) and angles (deg), see Supplementary Materials Section.

**Figure 3:**
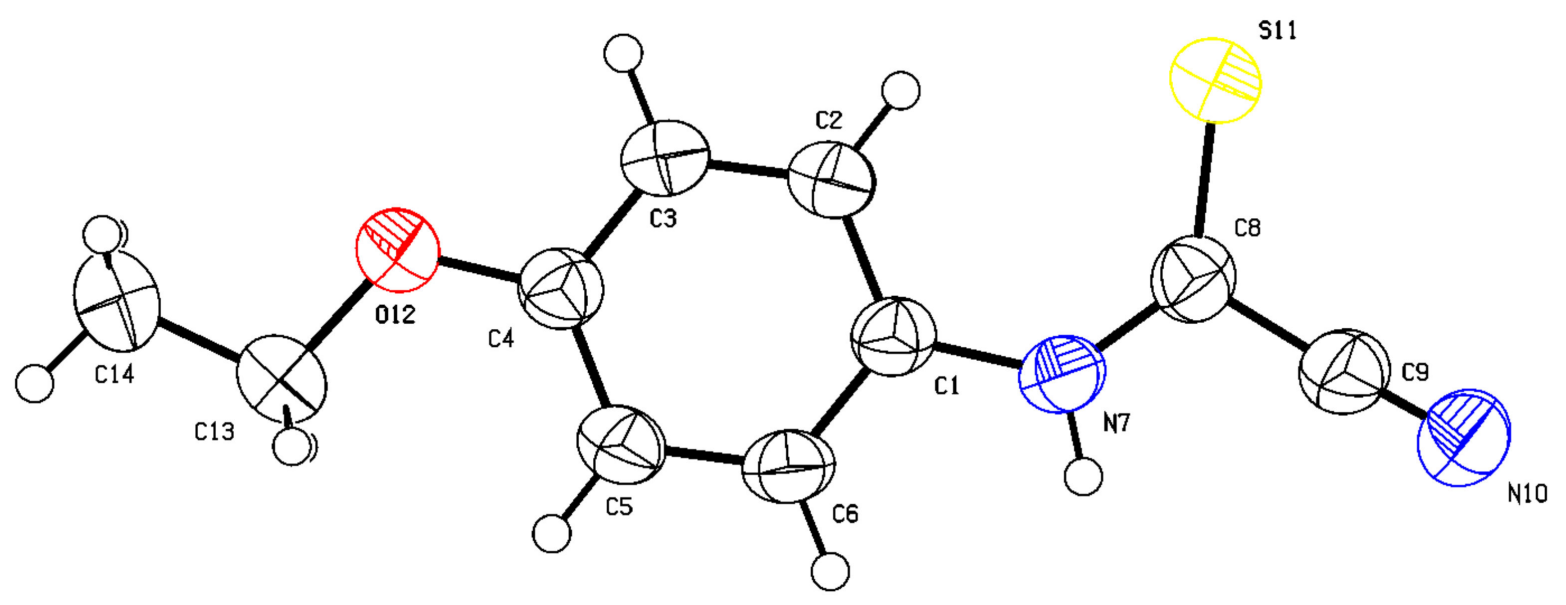
The crystal structure of (4-ethoxyphenyl)carbamothioyl cyanide (**27p**) (Deposition Number **2179720**). For bond distances (Å) and angles (deg), see Supplementary Materials Section.

In addition to the standard spectroscopic techniques used to characterize the starting materials, crystal-structure determination by means of single-crystal X-ray diffraction was carried out on *(Z)-N*-(2,3-difluorophenyl)-2-oxopropanehydrazonoyl chloride (**29l**) (Figure 2) (see Supplementary Materials Section S459–S462) and (4-ethoxyphenyl)carbamothioyl cyanide (**27p**) (Figure 3) (see Supplementary Materials Section S463–S466) as typical representative molecules of the starting materials. 

The hydrazonoyl chloride (Figure 2) features a near planar C_1_-N(H)-N(7)-C(Cl) unit (dihedral angle = 0.8(3)°) where the phenyl ring is almost coplanar with it (where the C(6)-C(1)-N(7)-N(8) dihedral angle = 2.6(3)° and F(14)-C(2)-C(1)-N(7) dihedral angle = −1.2(3)°). The acetyl fragment (where the N(8)-C(9)-C(10)-C(12) dihedral angle = 1.6(3)°) also adopts a linear co-planar arrangement with respect to the rest of the molecule. Another notable feature is the Z-geometry around the N(8)-C(9) bond where an angle of 120.07(16)°, typical of trigonal planar molecular geometry, is observed for N(8)-C(9)-C(10). Similarly, a bond angle of 120.50(15)° is observed for C(9)-N(8)-N(7), providing evidence to support the anticipated bent molecular geometry around N_8_. Likewise, N_7_ displays a trigonal planar geometry where the N(8)-N(7)-C(1) is 119.55 (14)°. The N(7)-N(8) bond length of 1.331 (2) Å is much shorter than the typical N-N single bond (1.47 Å). The preceding bond angles and lengths data indicate that the molecule exhibits extended conjugation and zigzag conformation. 

All cyanothioformamides used herein were prepared according to our published procedure form various substituted isothiocyanates and potassium cyanide (Scheme 2a) [33]. These reactants were partially characterized by standard 1D NMR spectroscopy (^1^H and ^13^C) and the obtained spectral data matched those reported earlier [33]. One interesting structural aspect of *N*-arylcyanothioformamides is that they exist as tautomeric mixtures in solution (arylcarbamoyl cyanide and arylcarbonocyanidimidothioic acid) [23]. We were the first to report this inherent property [23] based on the presence of two different sets of NMR signals stemming from the tautomeric mixture in the ^1^H and ^13^C NMR spectra of these species. To date, no conclusive evidence has been presented to ascertain the structure of the major tautomer in solution. The implication of the existence of tautomers on the regiochemical outcome is important since such species may react via the nitrogen or sulfur atom, leading to different heterocycles. Structural elucidation of (4-ethoxyphenyl)carbamothioyl cyanide (**27p**) by single crystal X-ray crystallography, as a representative example of the arylcyanothioformamide starting materials, is shown in Figure 3. Clearly, the compound appears as one tautomer in the solid state, indicating the thione as the preferred form. The nitrile function, originating from potassium cyanide, is clearly visible and exhibits a typical linear bond angle of 177.0(4)° for N(10)-C(9)-C(8) and a bond length C(9)-N(10) = 1.128(4) Å and consistent with a triple bond. The cyanothioformamide (Figure 3) also features a near planar C(1)-N(H)-C(CN) unit (dihedral angle = −178.0 (3)°) and a relatively coplanar phenyl ring (where the C(2)-C(1)-N(7)-C(8) dihedral angle = 2.3 (5)° and C_1_-N(H)-C(S) dihedral angle = 0.8(5)°). Interestingly, the ethoxy fragment (where the C(5)-C(4)-O(12)-C(13) dihedral angle = −2.2(5)°) also adopts a linear co-planar arrangement with respect to the rest of the molecule. The foregoing bond angles and lengths confirm that the molecule displays extended conjugation and zigzag conformation which facilitates conversion between the N/S tautomers in solution. Another noteworthy structural element is the anticipated wide bond angle around the C(1)-N(7)-C(8) bonds (131.3(2)°) necessary to minimize crowding and non-bonded interaction between the phenyl and thione. Such angle is more typical of trigonal planar molecular geometry and sp^2^ hybridized nitrogen (N7) atom. Similarly, a wide bond angle of 132.4(2)° is observed for N(7)-C(8)-S(11) to further reduce torsional strain and decrease special proximity (intramolecular distance) with the phenyl group. The remaining complementary, yet distorted, angles around C(8) are 112.4(2)° and 115.3(2)° support the expected trigonal planar molecular geometry around C(8). 

#### 2.1.2. Preparation of 5-Arylimino-1,3,4-thiadiazoles

**Scheme 3:**
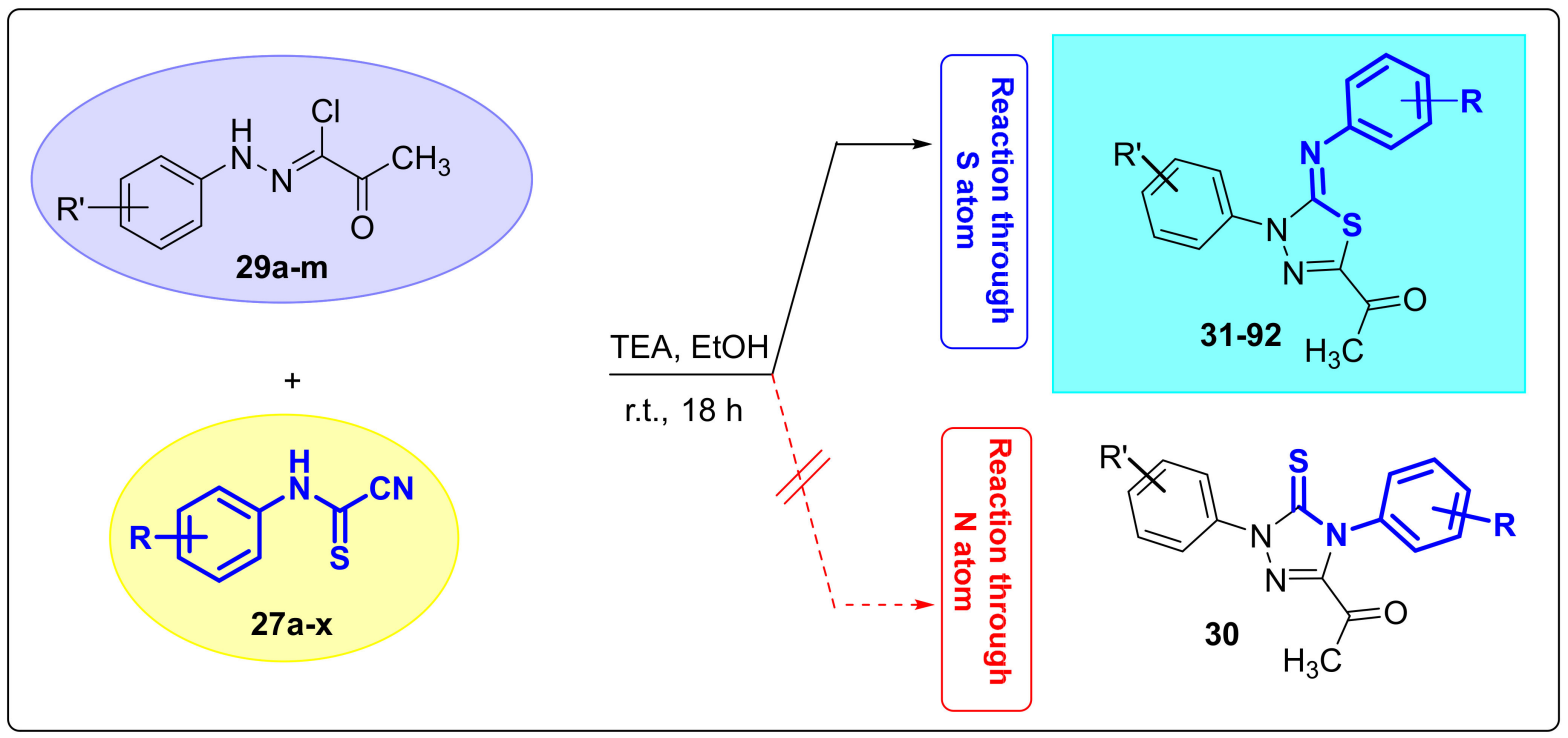
Synthetic approach to prepare different derivatives of variously substituted 5-arylimino-1,3,4-thiadiazoles **31**–**92**.

At the onset of our work, we were intending to find improved conditions to prepare highly pure 5-arylimino-1,3,4-thiadiazoles without resorting to purification by column chromatography. Thus, using (4-nitrophenyl)carbamothioyl cyanide (**27a**) as the least nucleophilic reactant, due to the highly negative mesomeric effect (-M) of NO_2_, and (*Z*)-2-oxo-*N*-phenylpropanehydrazonoyl chloride (**29a**) as an unsubstituted model substrate (Scheme 3), we explored several reaction conditions to determine the optimal conditions for best yields and purity of products. Initially, an equimolar amount of **27a** and **29a** was treated with two molar equivalents of triethylamine as base and the reaction mixture stirred overnight at ambient temperature using a series of solvents including DMF, DMSO, and acetonitrile. Unfortunately, TLC analysis indicated proximity of several spots, corresponding to starting materials and side products, alongside the desired product. Further, the product could not be precipitated out from the reaction solvent. Pleasingly, when ethanol was used instead, almost immediate precipitation of product in copious quantity was observed. In this case, (*Z*)-1-(5-((4-nitrophenyl)imino)-4-phenyl-4,5-dihydro-1,3,4-thiadiazol-2-yl)ethan-1-one (**31**) precipitated from the reaction mixture and was isolated by filtration using Whatman filter paper in 77% yield. No trace of the alternative regioisomer **30** was observed at the completion of reaction. Although in general the reaction was near complete within the first two hours, as indicated by the disappearance of most of the starting materials by TLC and aliquot NMR analysis, it was left stirring gently for further 16 h to collect more precipitate. No signs of decomposition were observed with longer stirring times, highlighting the robustness of the product. Although protic solvents usually impede reactivity via the nitrogen atom due to strong hydrogen bonding and promote a reaction pathway involving cyclization via the more polarized and larger sulfur anion, it appears that switching to aprotic solvents like DMSO or DMF has no impact on the regiochemical outcome of the reaction, nor could it induce reactivity via the nitrogen atom as would have been expected. Therefore, a single regioisomer formed at the end of the reaction regardless of solvent used. 

**Figure 4:**
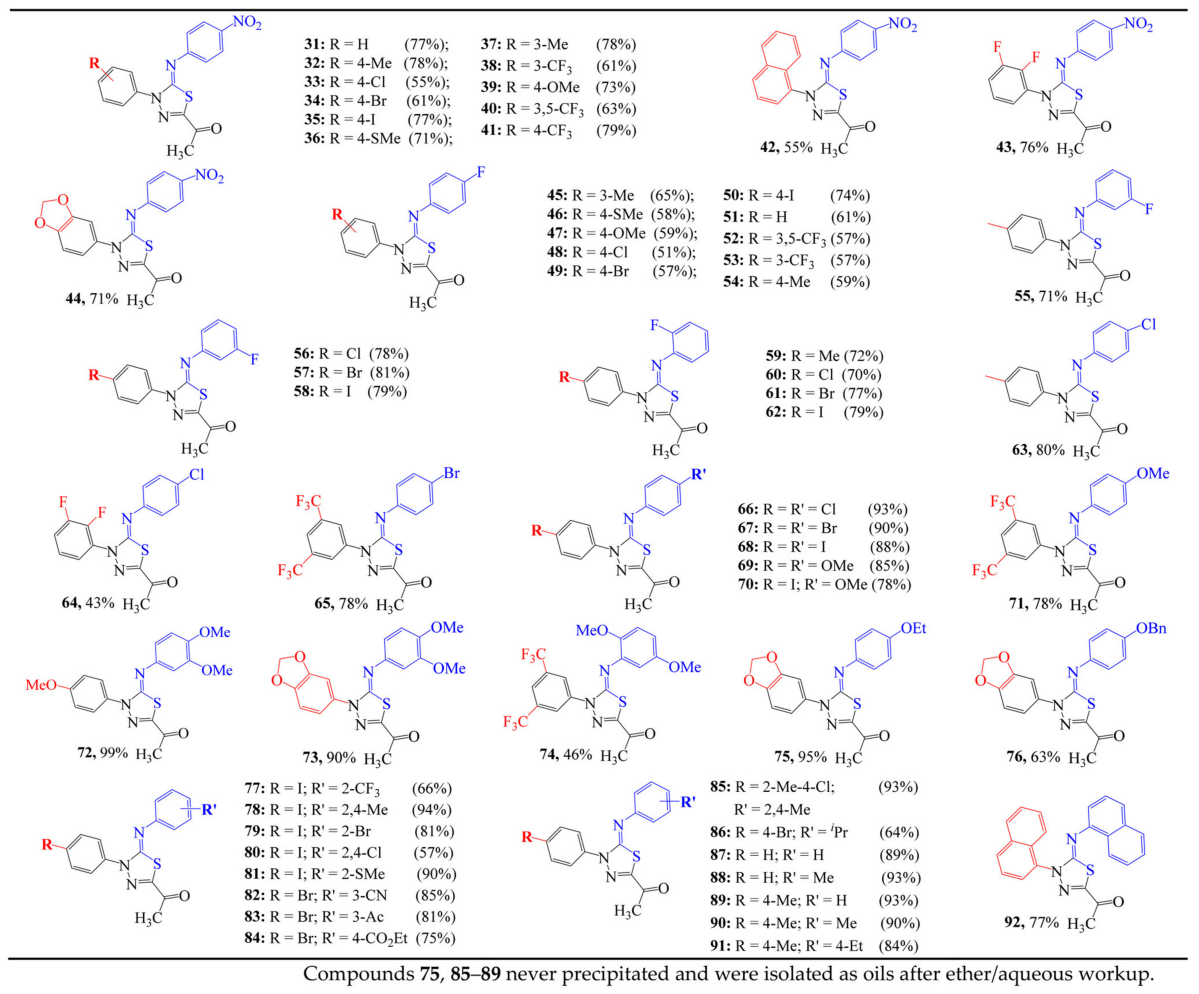
Preparation of variously substituted 1,3,4-thiadiazole derivatives **31**–**92**.

With the optimized reaction conditions in hand (1:1:2 equimolar amounts of the *N*-arylcyanothioformamide, hydrazonoyl chloride, and TEA, rt, 18 h), the generality of this synthetic protocol was initially evaluated using the least nucleophilic *N*-arylcyanothioformamide (**27a**) with a variety of substituted hydrazonoyl chloride derivatives (**29**). The latter were decorated with substituents or heterocyclic rings capable of displaying positive or negative mesomeric (+M, −M) and inductive (+I and −I) effects to examine the impact on regioselectivity, reactivity, and yield. In addition, being able to install any substituents at free will is critical for providing a library of compounds for bioactivity and SAR studies. The desired nitrated thiadiazoles **31**–**44** were obtained in 55–79% yield. Clear enhancement in yield was observed as the halogen group was changed from 4-chloro (**33**: 55%) to 4-bromo (**34**: 61%) to 4-iodo (**35**: 77%); meanwhile, the unsubstituted (**29a**) and methyl substituted hydrazonoyl chlorides (**29b** and **29g**) generated the nitrated products **31**, **32**, and **37** in improved yields (77–78%). Using +M groups such as 4-methoxy (**29j**), 1,3-dioxolane (**29m**), or 4-thiomethyl (**29f**) caused slight reduction in product yields (73–71%), whereas 3-CF_3_ (**29h**) or disubstituted 3,5-bis(trifluoromethyl) (**29j**) reactants diminished yields to 61–63%. Notably, 4-CF_3_ or 2,3-F substitutions were favorable (**41**: 79% yield; **43**: 76% yield). Switching to *N*-(4-fluorophenyl)cyanothioformamide (**27f**) as the reactant, the general yields of the *p*-fluorinated thiadiazoles **45**–**54** were lower (51–74%) than those obtained with the nitro analog **27a**. Polyfluorinated products such as **52** and **53** were only isolated in 57% yield because they are highly soluble in the ethanol solvent. Indeed, filtration of the precipitated solid from the reactions of the fluorinated products and ^1^H NMR analysis of the remaining mother liquor revealed the thiadiazoles as the sole products still present in solution. Therefore, the isolated yields herein do not reflect actual reaction yield, but rather the amount isolated by filtration of the precipitate formed. In fact, ^1^H NMR analysis of the crude products in most entries (Figure 4) showed complete and clean conversion. Though, *meta*- and *ortho*-fluorinated products **55**–**62** were isolated in better yields than their 4-fluoro analogs **45**–**54** (67–79%).

Next, we demonstrated the ease of preparing products with similar substituents on both aromatic groups. Accordingly, dichloro-(**66**), dibromo-(**67**), diiodo-(**68**), dimethoxy-(**69**), dimethyl-(**91**), and dinaphthyl-(**92**) substituted thiadiazoles were easily prepared from the appropriate reactants. Further, mono- and poly-substituted thiadiazoles with any combinations of -M, +M, -I, and +I groups on both aromatic rings could also be prepared by various permutations of the reactants. Thus, various alkoxylated (**69**, **72**, **73**, **76**), halogenated (**48**–**50**, **52**, **53**, **56**–**58**, **60**–**62**, **64**–**68**, **77**, **79**, and **80**), alkylated (**90**, **91**), and several other products functionalized with a thiomethyl (**81**), nitrile (**82**), 3,4-methylenedioxy (**44**, **73**, **75**, **76**), ketone (**83**), ester (**84**), isopropyl (**86**), and benzyloxy (**76**) groups have been successfully prepared. Analogs with hydrophobic groups (**87**–**92**), which satisfy the demand for facile penetration through cell membranes, were readily prepared. In total, 18 functional groups were installed in various combinations and in reasonable yields. Finally, we challenged our methodology using sterically hindered substrates. Pleasingly, the desired products **74**, **77**–**81**, and **85** were cleanly produced in moderate (**74**: 46%; **77**: 66%; **80**: 57%) to surprisingly high yields in cases of *ortho*-methyl-(**78**) (94%), *ortho*-bromo-(**79**) (81%), and *ortho*-dimethyl-(**85**) (93%). The synthesis of 5-arylimino-1,3,4-thiadiazoles was amenable to scale-up to gram quantities as demonstrated by the synthesis of **35**, **61,** and **90**, on a large scale from **27a/29e**, **27f/29d**, and **27b/29b** (10 mmol scale). The products were isolated in 74%, 75%, and 87%, respectively, with yields comparable to those obtained during small scale preparation. Regarding reactivity, alkoxylated *N*-arylcyanothioformamides and hydrazonoyl chlorides proved the slowest substrates to yield any appreciative amounts of precipitate in the first two hours. This is not surprising since the alkoxy groups render the N-H group of such reactants less acidic and slower to react with the added base. Interestingly though, this did not impact the overall yield of the reactions producing alkoxylated derivatives which, in fact, were obtained in high yield (**72**: 99%; **75**: 95%; **73**: 90%) except for the sterically hindered ortho-methoxylated product **74** (46%). Generally, though, the reactivity of the *N*-arylcyanothioformamide substrates was not impacted to any significant extent by the nature or size of substituent in the *ortho* position of the aromatic ring. For instance, 2-fluoro substituted *N*-arylcyanothioformamide **27f** generated products in 67–79% yield and the 2-bromo **27n** and thiomethyl **27x** analogs produced even larger yields (81% and 90%, respectively). Notably, the highest yielding arylcyanothioformamide substrates were unsubstituted and those substituted with alkyl groups. Unsubstituted arylcyanothioformamide afforded **87** in 89% yield whereas that substituted with 4-methyl, 4-methyl or 4-ethyl generated products in 84–93% yield, respectively. Even in cases where **27** substrates are *ortho*-alkylated and highly substituted, a yield of 94% and 93% was obtained for the resulting products **78** and **85**, respectively, suggesting that steric factors were subjugated by the presence of alkyl groups. Switching to *ortho*-trifluoromethylated substitution (**27l**) was relatively detrimental to the yield (**77**: 66%) whereas keeping the alkyl group even on heteroatoms (e.g., thiomethyl **27l**) maintained the high yield (**81**: 90%). 

The resulting light yellow/off-white/brown/grey/green/orange products could be isolated cleanly by gravity filtration as solids without the need for flash chromatography and are very stable at room temperature. However, compounds **75**, **85**–**89** were isolated as oils, though they did not require any purification. All products were amenable to storage at ambient temperature for months. High-resolution mass spectrometry (HRMS) and NMR measurements corroborated the suggested structures **31**–**92** (Figure 4). 

#### 2.1.3. 1D/2D NMR Structural Analysis 

The chemical structures of all compounds (**31**–**92**) (Figure 4) and formation of the key thiadiazole bonds and ring were confirmed and fully characterized by standard spectroscopic and analytical techniques (mp, IR, 1D NMR, and HRMS). In addition, 2D homonuclear and heteronuclear NMR (^1^H-^1^H-gDQFCOSY, ^1^H-^13^C-gHSQC, ^1^H-^13^C-gHMBC) was performed on all examples to trace the ^1^H-^1^H and ^1^H-^13^C connectivity and identify the chemical shifts of each carbon and proton (see Supplementary Materials Section S90–S458). The physical and spectral data of known chemical compounds matched those reported in the literature (see Section 3.5 and Supplementary Materials Sections). The most distinctive IR signals of the thiadiazole products are those of the C=O and C=N groups which appear around 1678–1697 and 1613–1643 cm^−1^, respectively. 

**Figure 5:**
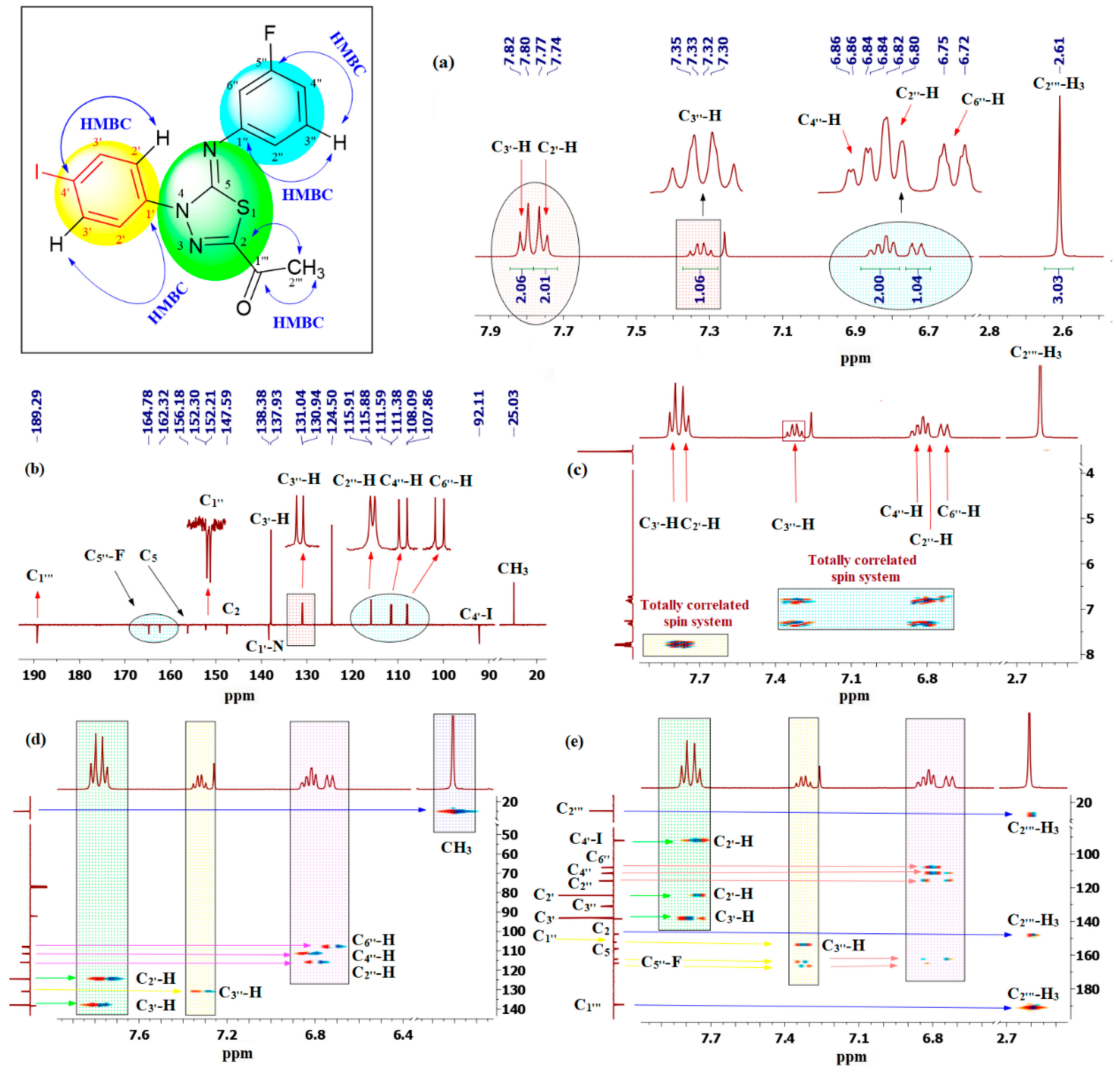
Truncated 1D and 2D NMR spectra of 1,3,4-thiadiazole **58**: (**a**) ^1^H-NMR; (**b**) ^13^C-CRAPT NMR; (**c**) ^1^H-^1^H-gDQFCOSY NMR; (**d**) ^1^H-^13^C-gHSQC NMR; (**e**) ^1^H-^13^C-gHMBC NMR.

Next, using (*Z*)-1-(5-((3-fluorophenyl)imino)-4-(4-iodophenyl)-4,5-dihydro-1,3,4-thiadiazol-2-yl)ethan-1-one (**58**) as a representative model example, the relevant NMR spectra that were used for structural elucidation and chemical shift assignments are shown in Figure 5. Examination of the ^13^C-CRAPT NMR spectrum (Figure 5, spectrum b) established the presence of the expected 14 signals (six aromatic CH’s, four phenyl quaternary carbons, one C=O carbon, one acyl methyl, and two heterocyclic quaternary carbons), which is consistent with two carbons being magnetically equivalent. The most prominent feature of the ^13^C-CRAPT NMR of **58** is the presence of six doublets corresponding to the 4-fluorophenyl ring carbons which are spin-coupled with the fluorine atom. This led to a quick identification and matching of the observed chemical shifts of 4-fluorophenyl with the respective carbon position on the 4-fluorophenyl ring. Thus, based on the magnitude of the coupling constants, as anticipated, the C_5″_, which is directly bonded to the F atom, was the most deshielded doublet (δ 163.6) and exhibited the largest coupling constant (d, *J* = 246.0 Hz). On the contrary, the para C_2″_ atom (δ 115.9), which is the farthest doublet from the fluorine atom, exhibited the smallest coupling constant (d, *J* = 3.0 Hz). The quaternary C_1″_ carbon, which corresponds to the only doublet with a negative phase, was surprisingly highly deshielded, resonating at δ 152.3 (d, *J* = 9.0 Hz). As expected, the two flanking *ortho* carbon atoms C_6″_ (δ 108.0) and C_4″_ (δ 111.4) exhibited the largest coupling constants among the methine groups (d, *J* = 23.0, 21.0 Hz, respectively), whereas the meta carbon C_3″_ resonated further downfield at δ 131.0 and displayed much lower long-range coupling constant (*J* = 10.0 Hz). The four fluorophenyl protons H_3″_, H_4″_, H_2″_, and H_6″_ (δ (ppm) 7.32, 6.84, 6.81, and 6.73 ppm, respectively) (Figure 5a), which correlate with the same spin system in the ^1^H-^1^H**-**gDQFCOSY spectrum (Figure 5, spectrum c), were matched to C_3″_ (δ 131.0), C_4″_ (δ 111.5), C_2″_ (δ 115.9), and C_6″_ (δ 108.0), respectively, based on the ^1^H-^13^C-gHSQC spectrum (Figure 5d). The two remaining methine aromatic signals in the HSQC with positive phase and double the intensity at δ (ppm) 137.9 and 124.5 ppm were traced to the iodophenyl H_3′_ (δ 7.81) and H_2′_ (δ 7.76) doublets, respectively, based on strong correlation cross peaks observed in the ^1^H-^13^C-gHSQC spectrum (Figure 5d). Clearly, C_3′_H and C_2′_H comprise one spin system, as further supported by ^1^H-^1^H**-**gDQFCOSY which shows them as a totally correlated system (4-contour red square in the aromatic region (Figure 5c). The scalar coupling between the vicinal C_3′_H and C_2′_H is 8.8 Hz. Identification of the distinctive signal of C_4′_-I (δ 92.1) confirmed the preceding assignment of the iodophenyl methines. Particularly, H_2′_ shows strong correlation cross peak (^3^*J*) in the ^1^H-^13^C-gHMBC spectrum (Figure 5e) with C_4′_-I. On the other hand, H_3′_ shows strong HMBC correlation contour (^3^*J*) with C_1′_, completing the assignment and matching of the iodophenyl ring chemical shift values. 

**Figure 6:**
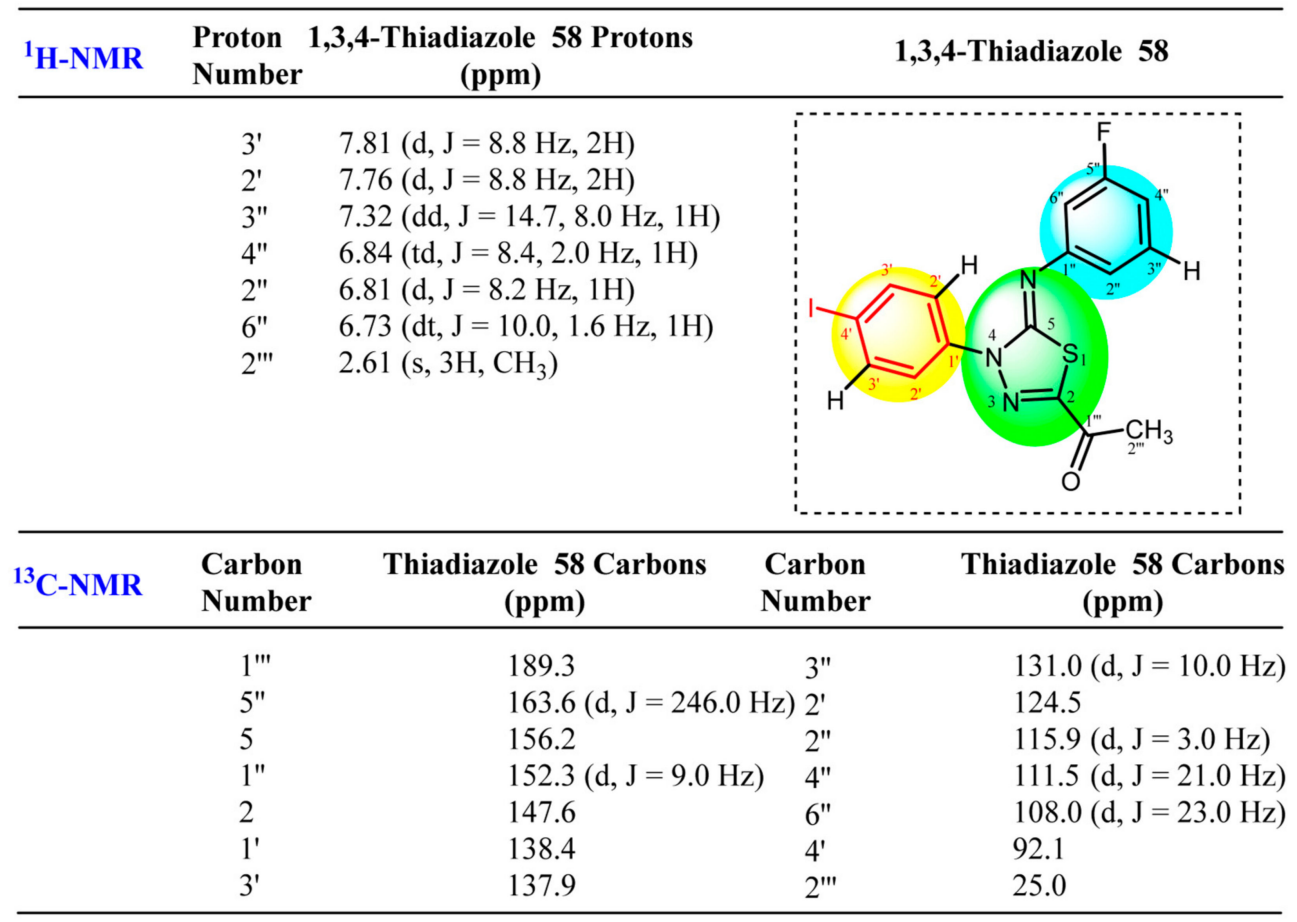
Assigned ^1^H- and ^13^C-NMR chemical shifts of 1,3,4-thiadiazole **58**.

Having completely assigned the chemical shift values to the protons of the two independent spin systems of the fluorophenyl and iodophenyl, the quaternary carbons were identified through the ^1^H-^13^C-gHMBC spectrum (Figure 5e). The methyl (C_2′′′_-H_3_; ^1^H NMR *δ* 2.61 ppm) group tethered to C_2_ of the thiadiazole ring (Figure 5) offered the only entry point to provide unambiguous matching of the observed chemical shifts of the heterocycle quaternary centers in the ^13^C-CRAPT NMR to the appropriate structural positions. In this regard, the ^1^H-^13^C-gHMBC NMR spectrum (Figure 5e) shows two strong long-range correlation contours between the C_2′′′_ methyl protons (*δ* (ppm) 2.61 ppm) and the most deshielded ketone (C=O) carbon C_1′′′_ (*δ* (ppm) 189.3 ppm) as well as C_2_ (*δ* (ppm) 147.6 ppm). Interestingly, the latter correlation proves the formation of the new thiadiazole N=C_2_-S quaternary center and is an indication of a successful heterocyclization reaction between the hydrazonoyl chloride and *N*-arycyanothioformamide starting material. Lastly, recognition of C_2_ prompted the identification of C_5_ as the signal at *δ* 156.2 ppm. Notably, this was the only signal that showed no HMBC long-range coupling, as would be expected for such an isolated carbon far removed from all protons. The complete assigned ^1^H- and ^13^C-NMR chemical shifts of 1,3,4-thiadiazole **58** are shown in Figure 6. 

#### 2.1.4. Crystal-Structure Determination of the 5-Arylimino-1,3,4-thiadiazoles

**Figure 7:**
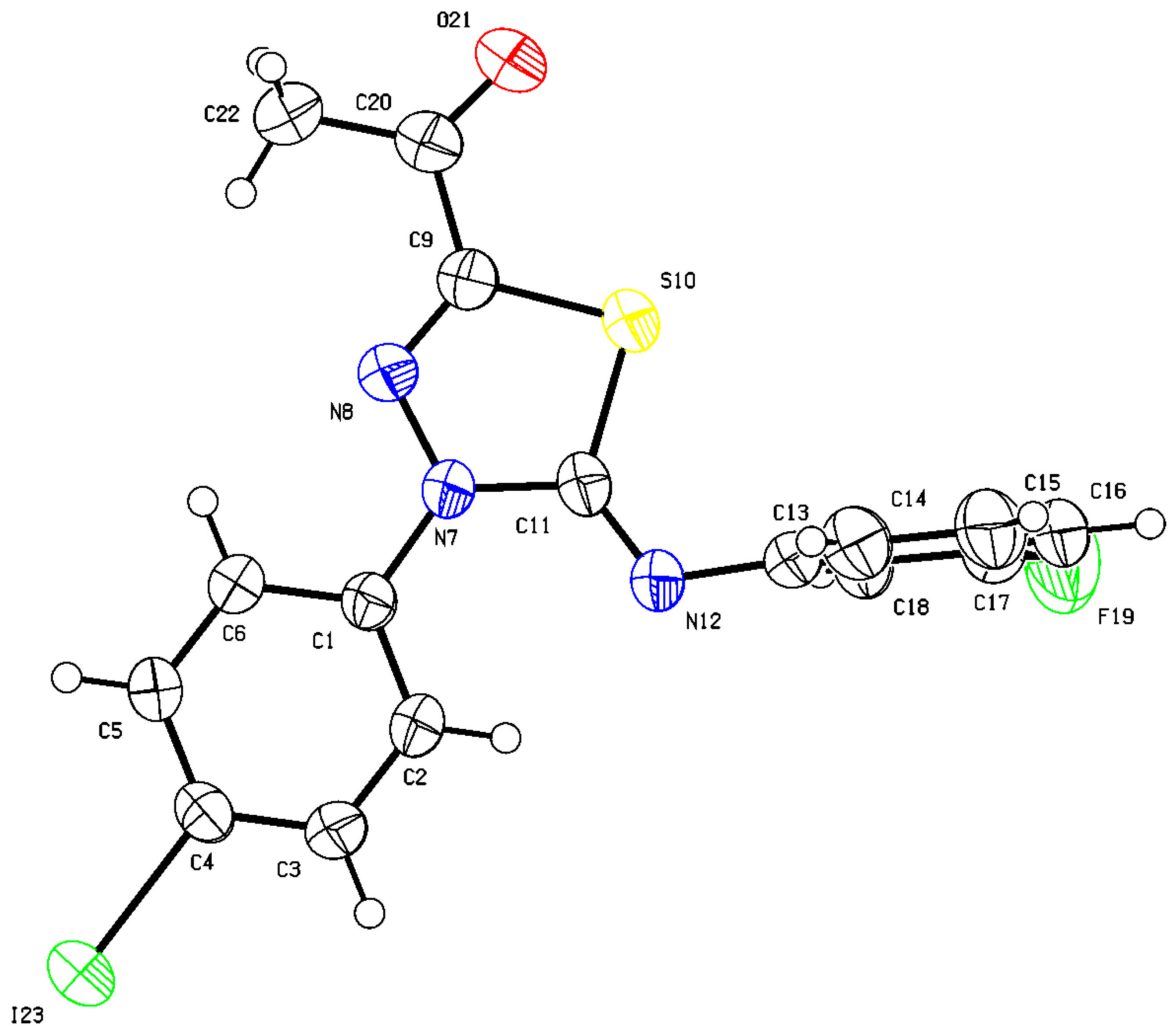
Thermal ellipsoid plots of (*Z*)-1-(5-((3-fluorophenyl)imino)-4-(4-iodophenyl)-4,5-dihydro-1,3,4-thiadiazol-2-yl)ethan-1-one (**58**) with ellipsoids drawn at 50% probability level (Deposition Number **2178549**). For bond distances (Å) and angles (deg), see Supplementary Materials Section.

Although 1D and 2D NMR analysis provided strong evidence supporting the formation of the thiadiazole scaffold, it is not sufficient to distinguish the two possible regioisomeric products and conclusively determine the correct structure of the obtained product. Pleasingly, we were able to grow crystals suitable for X-ray diffraction analysis. Thus, structural verification of **58** was also carried out by single crystal X-ray crystallography (Figure 7) (see Supplementary Materials Section S467–S471). 

Clearly, thiadiazol **58** comprises a 5-membered 1,3,4-thiadiazole with sulfur incorporated into the heterocyclic ring and a C_11_=N_7_ group characterized by short bond length of N(7)-C(11) = 1.409(4) Å (X-ray numbering) and typical trigonal planar geometry for N(8)-C(9)-S(10) = 116.3(3)° and N(12)-C(11)-N(7) = 125.2(3)°, though a much strained internal angle of 107.8(3)° was observed for N(7)-C(11)-S(10). The presence of vicinal substituents on the heterocyclic ring compresses the internal angles to relieve crowding and increase the complementary external angles to better accommodate the bulky groups. As expected, the C_9_=N_8_ bond length of N(8)-C(9) = 1.275(5)° is shorter than that of C(11)-N(7) = 1.409(4)°, indicating π bond character and typical trigonal planar geometry and sp^2^ hybridization based on the C(9)-N(8)-N(7) = 112.1° bond angle. The C(11)-N(7) displays a bond shorter than the typical C-N single bond due to strong resonance participation of N(7) with the attached 4-iodophenyl, N(7) and especially the iminoaryl group. Notably, the strong flow of electrons from N(7) to the iminoaryl group impacted the chemical shift of C(11) to a very significant extent. For instance, with 4-Cl, 4-NO_2_, and naphthyl (as electron withdrawing groups or electron sink) substituents on the iminoaryl group, the ^13^C chemical shift values of C(11) (X-ray numbering) are 157.6, 157.1, and 158.4 ppm, respectively. However, with electron donating substituents such as OMe, the chemical shift value is 153.9 ppm since the positive mesomeric effect of the methoxy counteracts the flow of electrons. Similarly, the chemical shift of C_11_ is significantly impacted by the iminoaryl substituent since the sulfur is capable of conjugation with the iminoaryl group as well. Thus, when the substituent is the electron-withdrawing 4-NO_2_ group, as an example, the chemical shift value of C_11_ is 149.2 ppm, and 146.3 ppm when the substituent is the electron-donating benzyloxy group (BnO). Both sulfur bonds, S(10)-C(11) = 1.771(4)° and S(10)-C(9) = 1.744(4)°, exhibit typical single bond length, suggesting that they are not strongly conjugated with the flanking π systems conceivably due to poor orbital overlap as evident by the internal strained angle of 88.21(17)° for C(9)-S(10)-C(11). Similary, N(7)-N(8) = 1.361(4)° is suggestive of a single bond. Consequently, the 5-arylimino-1,3,4-thiadiazole ring lacks true aromaticity but enjoys greater extended conjugation and resonance stabilization than the alternative regioisomer **30**, thus favoring its formation. The densely substituted thiadiazole ring X-ray clearly shows intact 3-fluorophenyl)imino group, the C9 acyl pendant and the N7-4-iodophenyl which features a long C-I bond (I(23)-C(4) = 2.102(3)°). More importantly, the stereochemistry of the observed regioisomer **58** has been established as *Z* around the iminophenyl double bond. It features a near planar 1,3,4-thiadiazole ring (dihedral angle: N(7)-N(8)-C(9)-S(10) = 1.0(4)°; C9-S(10)-C(11)-N(7) = 3.6(3)°) where the 4-iodophenyl substituent is roughly coplanar with it (where the C(6)-C(1)-N(7)-N(8) dihedral angle = −6.5(5)°. The acetyl fragment (where the N(8)-C(9)-C(20)-C(22) dihedral angle = −0.3(6)°) also assumes a linear co-planar arrangement with respect to the thiadiazole and 4-iodophenyl rings. Another notable feature is the Z-geometry around the N(12)-C(11) bond where an angle of 118.7(3)°, typical of trigonal planar molecular geometry, is observed for C(11)-N(12)-C(13) and a bond length typical of C=N is noted for N(12)-C(11) = 1.258(5)°. Similarly, a bond angle of 125.2(3)° is observed for N(12)-C(11)-N(7), providing evidence to support the anticipated bent molecular geometry around N(12). The observed Z-geometry creates crowding with the heterocyclic ring, forcing the 3-fluorophenyl ring to adopt an orthogonal orientation with respect to the rest of the molecule (where the C(11)-N(12)-C(13)-C(14) dihedral angle = 91.9(5)°). Of note, the *trans* diastereomer does not form due to steric factors related to the congested environment created by the neighboring N-aryl group which fosters unfavorable non-bonding interactions. Accordingly, the reported synthesis of 5-arylimino-1,3,4-thiadiazoles is exclusively regio- and diastereoselective. 

**Figure 8:**
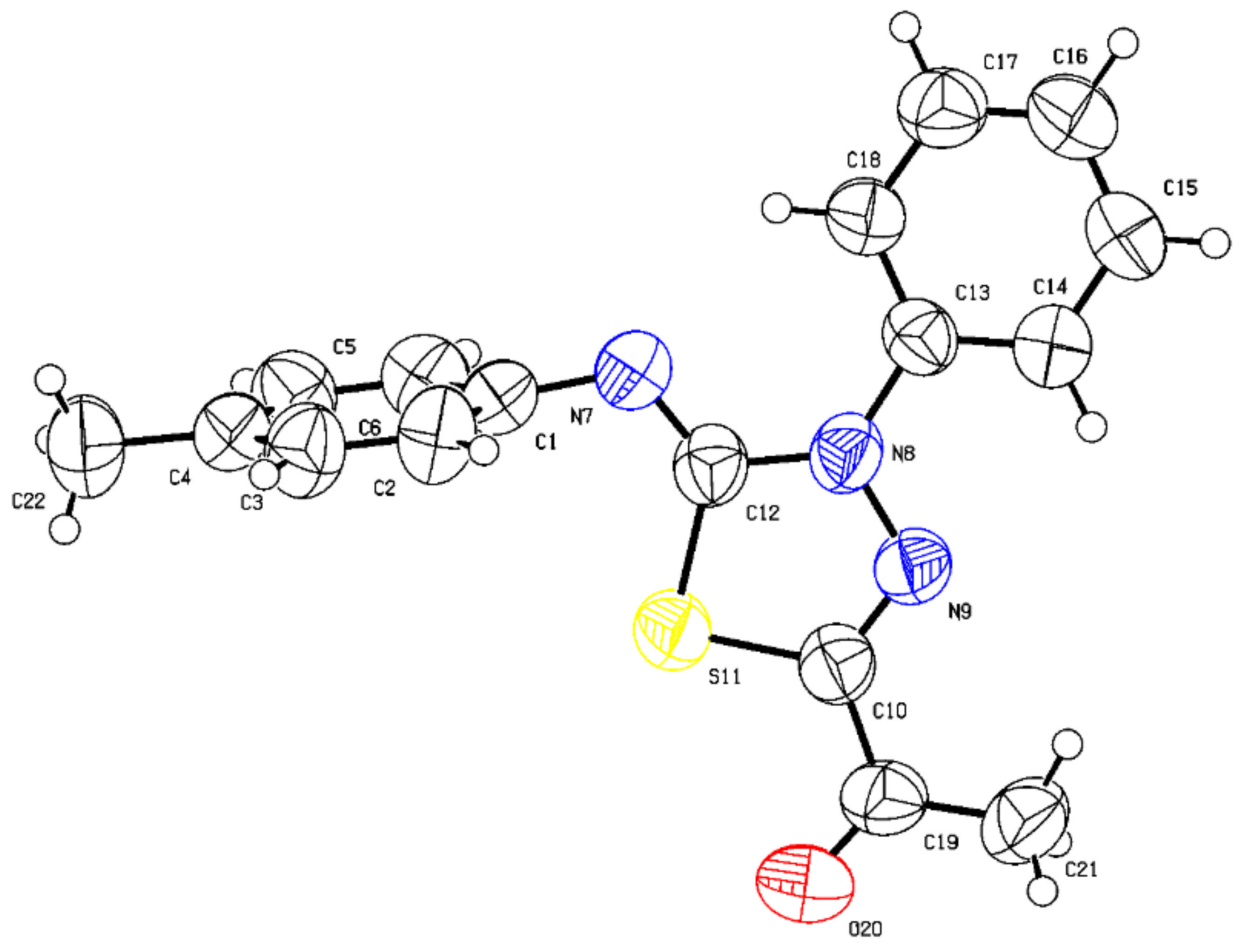
Thermal ellipsoid plots of (*Z*)-1-(4-phenyl-5-(*p*-tolylimino)-4,5-dihydro-1,3,4-thiadiazol-2-yl)ethan-1-one (**88**) with ellipsoids drawn at 50% probability level (Deposition Number **2178551**). For bond distances (Å) and angles (deg), see the Supplementary Materials Section S472–S476.

The structure of (*Z*)-1-(4-phenyl-5-(*p*-tolylimino)-4,5-dihydro-1,3,4-thiadiazol-2-yl)ethan-1-one (**88**) (Figure 8) was also proven by single crystal X-ray crystallography, adding further proof to support the consistent stereochemistry observed for the reported regio- and diastereoselective reaction, suggesting that the substituents on the aryl groups of both starting materials have no bearing on the stereochemical outcome of the reaction. The substituents mostly impacted the speed of reactions and how fast it reached completion (*vide infra*). In comparison, the bond angles and lengths of the thiadiazole ring of (*Z*)-1-(4-phenyl-5-(*p*-tolylimino)-4,5-dihydro-1,3,4-thiadiazol-2-yl)ethan-1-one are very similar to those observed for (*Z*)-1-(5-((3-fluorophenyl)imino)-4-(4-iodophenyl)-4,5-dihydro-1,3,4-thiadiazol-2-yl)ethan-1-one and are shown in Figure 8. The bond lengths of the aromatic C=C bonds such as C(13)-C(14) = 1.379(6) exhibit typical values know for these rings. The *p*-tolylimino and phenyl groups are both intact and the latter is oriented at 80.6(6)° (C_12_-N_7_-C_1_-C_2_) with respect to the rest of the molecule in which all groups are nearly co-planar to minimize non-bonded repulsion interactions. 

#### 2.1.5. Proposed Mechanism of Formation of the 5-Arylimino-1,3,4-thiadiazoles

**Scheme 4:**
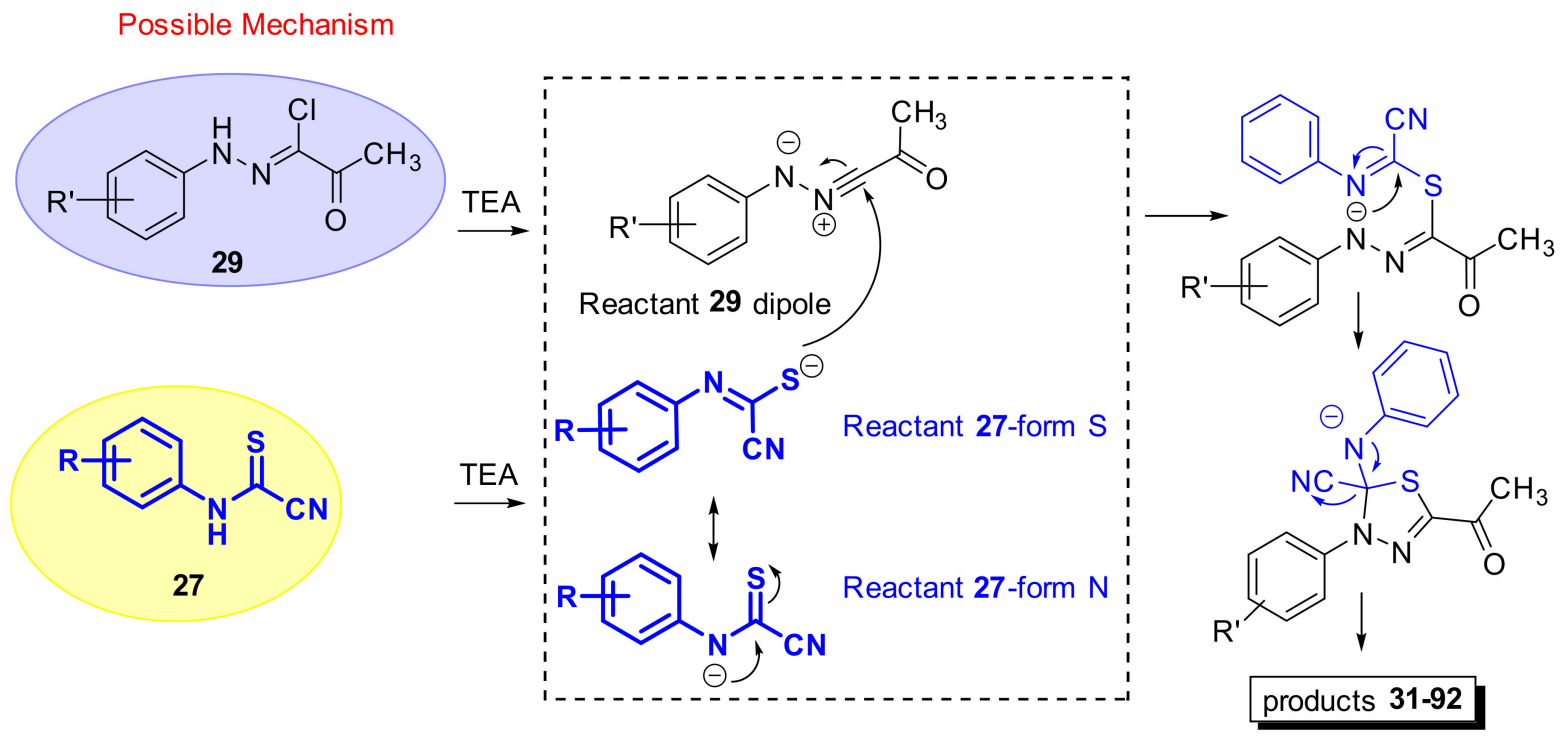
Proposed mechanism for the formation of substituted 5-arylimino-1,3,4-thiadiazoles from *N*-arylcyanothioformamide. The thioamide tautomerism of deprotonated reactant **27** is indicated by the double arrow in the middle panel.

Using phenylcarbamothioyl cyanide (**27a**) as a representative example, the proposed mechanism of heterocyclization is shown in Scheme 4. Mechanistically, the cyanothioformanilide precursor **27a**, which is a mixture of tautomers in solution, undergoes immediate deprotonation by the triethylamine base, to afford the corresponding anion as a triethylammonium salt. This intermediate has been characterized by ^1^H and ^13^C NMR in various solvents. ^1^H NMR analysis of the precursor phenylcarbamothioyl cyanide (**27a**) in CDCl_3_ indicated a tautomeric ratio of 71:31. Interestingly, almost the same resonance ratio was observed for the triethylammonium phenylcarbamothioyl cyanide (**27a**) salt in CDCl_3_ (69:31), although a much more drastic shift in ratio was obtained in different deuterated solvents. For instance, the ratio of the same salts in MeOD was 90:10 and 98:2 in DMSO-d6, indicating preference towards the formation of one major resonance intermediate. To further probe the impact of solvent on the regiochemical outcome, the reaction between **27a** and **29a** was performed in DMSO to test the effect of switching between protic and aprotic polar solvents. The same product **31** was obtained in a similar yield, although longer time was required, suggesting that the electronics of the substrate overcome solvent control. Therefore, although hydrogen bonding between ethanol and the nitrogen anion of **27a** (reactant **27**-form N) promotes reaction at the sulfur atom through reactant **27**-form S, using DMSO does not seem to foster any reactivity at the nitrogen atom. Next, acting as a nucleophile and reacting through the sulfur anion resonance form, reactant **27**-form S attacks the electrophilic carbon of reactant **29** dipole to generate the key S-C bond responsible for the exclusive formation of one regioisomeric product. Cyclization ensues nucleophilic attack of the dipole nitrogen anion onto the imine carbon, followed by elimination of cyanide ion. The reactivity of the electrophilic carbon of reactant **29** dipole towards sulfur [44,45] and nitrogen [46] anions has been observed previously, providing indirect evidence for the second step in our mechanism involving the formation of the C-S bond. In the next section, we confirm our proposed mechanism via computational studies.

### 2.2. Computational Results

To rationalize the experimental findings and confirm the proposed mechanism of Scheme 4, we conducted density functional theory (DFT) calculations at the B3LYP-D4/def2-TZVP level in ethanol as implicit solvent using the ORCA software [47] (see SI, S477–S479 for more computational details). The atomic charges, frontier molecular orbitals, and some DFT global descriptors were computed to rationalize the regioselectivity and identify the most susceptible sites in the molecules for the reaction. Finally, the possible role of the solvent in blocking one of the reaction pathways has been addressed. The discussion in the following sections concerns the deprotonated form of **27** and the dipole generated from **29** (reactant **29** dipole) with the substituents R = CH_3_ and R’ = H, respectively, and the first intermediate reaction, see Scheme 4.

#### 2.2.1. Charge Population and Bond Order Analysis

The calculated atomic charges (in units of *e*) for reactant **29** dipole and deprotonated **27** using different definitions are displayed in Table 1 and Table 2, respectively, along with the corresponding molecular representation of the reactants with atom labels.

The calculated charges from Table 1 suggest that the bonds N3-N2=C5 in **29** zwitterion are markedly polarized. Only the Mulliken and ADCH definitions seem to capture the correct zwitterionic character for the azo group in **29** zwitterion with positive and negative partial charges of similar magnitude on N2 and N3 atoms, respectively. The Mayer bond orders are 1.37, 2.30, and 1.11 for the azo group (N2-N3), the N2=C5 and N3-C4 bonds, respectively. The C5 atom in **29** zwitterion has a large positive Bader charge (0.742*e*) which signals its susceptibility to a nucleophilic attack by the anionic form of **27**. Indeed, the calculated average molecular electrostatic potential (MEP) values on the local surface of C5, N2, N3 atoms are 4.16573, 9.24631, and −12.36919 kcal/mol, respectively, which confirm that the C5 atom is one of the most susceptible sites for nucleophilic attack.

For reactant **27** anion, we note that NPA, Bader and ADCH charges (Table 2) get the correct partial charge (positive) on the nitrile carbon C18. The Mayer bond order for the nitrile group (C18N17) is 3.057, consistent with a triple bond. More importantly, the S1 and N2 sites have similar NPA charges (~−0.4*e*) consistent with a thioamide tautomerism [^−^N-C=S <=> N=C-S^−^], while Bader charges overemphasize the anionic character of the N2 atom. The Mayer bond orders for the thioamide group are in between the single and double bond with calculated values 1.405 and 1.659 for S1-C4 and N2-C4, respectively, consistent with the resonance pair of Lewis structures of the tautomerism. The bond order for the C4-C18 bond is 0.942, consistent with a single C-C bond. The ADCH method also gives similar charges (~−0.6*e*) for S1 and N2 atoms. The static picture that emerges from this simple charge population analysis is that for **27** anion the S1 and N2 sites have nearly equivalent atomic charges. Thus, one would expect that the reaction could take place via nucleophilic attack of either site to the azo compound. Thus, we need a grid-based DFT approach to further investigate the regioselectivity of the reaction, which will be considered next.

#### 2.2.2. Some Grid-Based DFT Quantities

The average local ionization energy (ALIE, denoted by Ī) [48] confirms that sulfur S1 is the most vulnerable site in anionic **27** for electrophilic attack by **29** dipole. The minima of the ALIE on the van der Waals (vdW) surface (defined as the isosurface of electron density with ρ = 0.001 a.u.) indicate the susceptible sites for electrophilic or radical attack since these regions are where the electrons are more weakly bounded. For anionic **27**, in principle two possible atoms are susceptible for electrophilic attack (S1 and N2) but our ALIE analysis confirms that S1 with Ī = 7.094492 eV is in fact the preferred site as it is the global minimum and lower than the ALIE value for N2 (Ī = 8.746194 eV). 

For completeness, we also calculated the average local electron affinity (LEA) [49] in **29** zwitterion to determine the sites more susceptible for nucleophilic attack by **27** anion. The site with more positive value of LEA represents the regions where accepting an electron is most favorable. According to the LEA analysis of **29** zwitterion, the C5 atom has the highest value of LEA (−22.2831 eV) while the N2 atom has the lowest value of LEA (−31.43293 eV). This is consistent with the preferential attack by the sulfur atom of **27** anion to the C5 site of **29** zwitterion, both “soft” sites, in agreement with the HSAB principle of Pearson.

#### 2.2.3. Frontier Molecular Orbitals (FMO)

**Figure 9:**
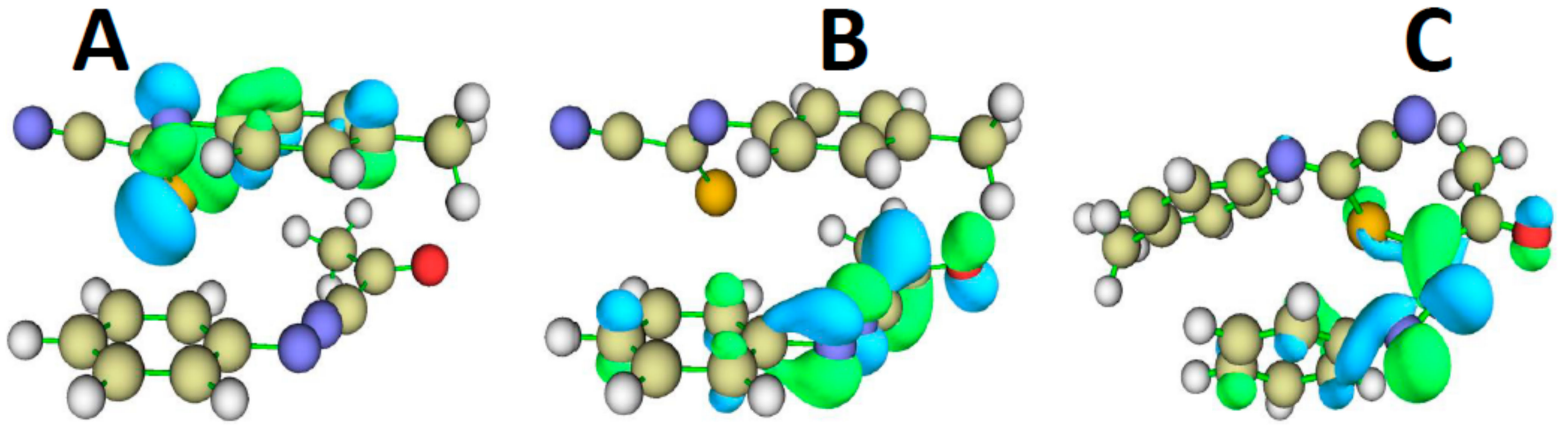
Isosurface representation of the Frontier Molecular Orbitals (FMO) for HOMO (**A**) and LUMO (**B**) for the unreacted complex of **29** (bottom molecule) with **27** (top molecule). The HOMO for the intermediate state is shown in (**C**). The level of theory is B3LYP-D4/def2-TZVP. The isosurface value is +/− 0.05 a.u., with the green and blue regions corresponding to the positive and negative signs of the FMO, respectively. Color code for atoms: grey = C, blue = N, red = O, yellow = S, and white = H.

**Figure 10:**
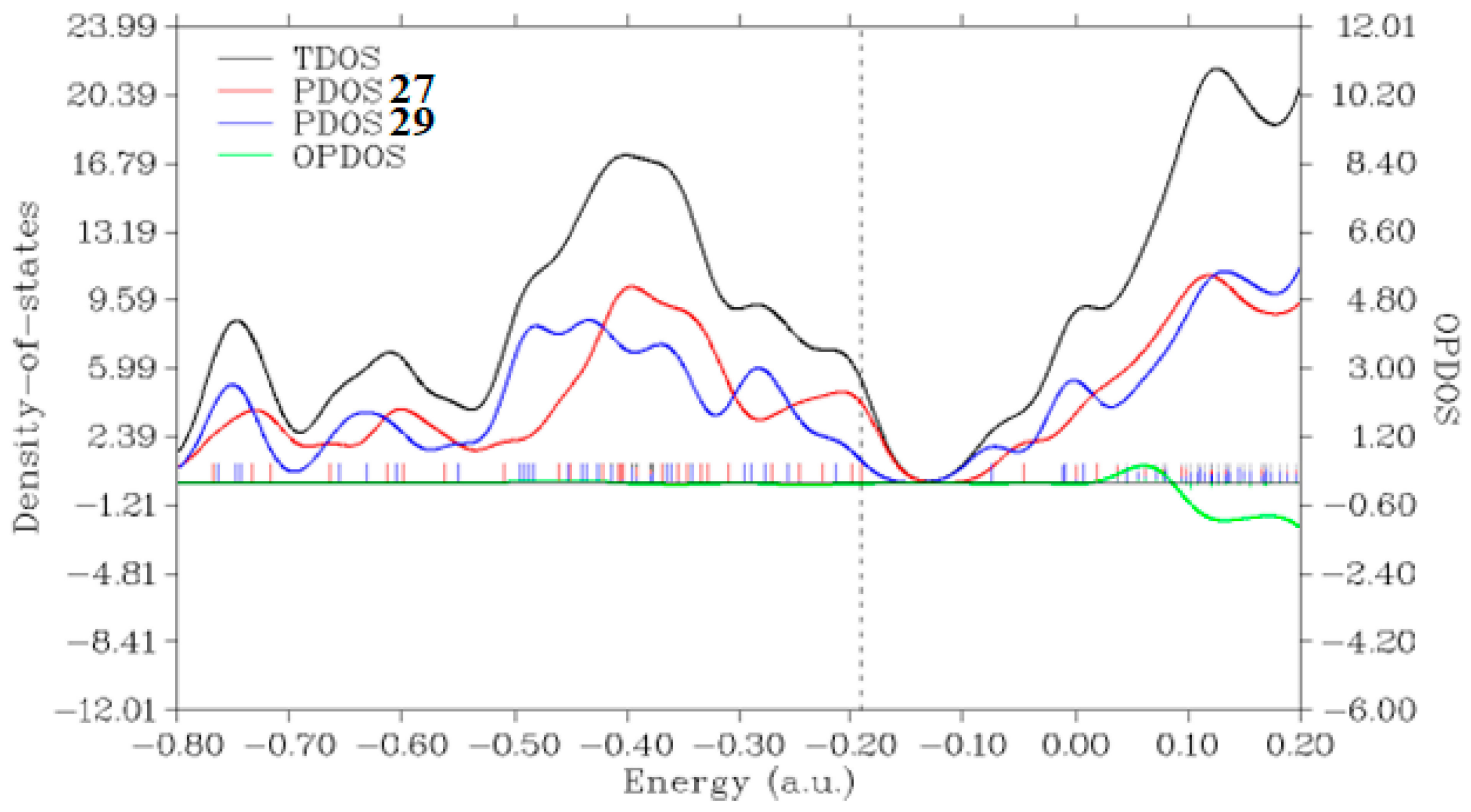
Electronic Density of States (DOS) of the unreacted complex of **29** with **27** at the B3LYP-D4/def2-TZVP level in implicit ethanol as solvent. Total DOS (black) and projected DOS on the reactant **29** (blue) and **27** (red) molecules. The overlap population DOS (OPDOS) is given by the green curve which shows the negligible overlap between MOs below the LUMO level. The vertical dashed line indicates the energy position of the HOMO. The eigenvalues of the Kohn–Sham are indicated by the vertical spikes. Energy units are in hartree and DOS is in hartree^−1^.

The Frontier Molecular Orbitals (FMO) were computed on the unreacted complex made of **27** anion with **29** and are displayed in Figure 9A,B. The HOMO is clearly localized on anionic **27** (A) whereas the LUMO is localized on the **29** zwitterion (B). Upon reaction, we expected a transfer of electronic charge from the anionic **27** moiety to the **29**-zwitterion reactant. This picture is confirmed in Figure 9C corresponding to the HOMO of the reaction intermediate, see Scheme 4. A projected density of states (PDOS) plot (Figure 10) on the unreacted complex further confirms this finding by showing the localization of HOMO (vertical dashes) on the **27** moiety (red line) and the LUMO on **29** fragment (blue). The nearly non-interacting character of the reactants in the complex is confirmed by the negligible overlap population DOS (OPDOS, green curve) between MOs below the LUMO level.

#### 2.2.4. Conceptual DFT Global Descriptors

Table 3 shows some conceptual DFT global descriptors derived from the frontier MO eigenvalues at the B3LYP-D4/def2-TZVP level. The electronic chemical potential, defined approximately as the simple average of FMO eigenvalues, μ = (E_HOMO_ + E_LUMO_)/2, is a global index that can give an idea of the electron flow between reactant species. According to Table 3, the chemical potential of reactant **27** anion (μ = −3.203 eV) is higher than reactant **29** zwitterion (−4.068 eV), which means that, upon addition, electrons flow from the former to the latter. Reactant **29** zwitterion is confirmed to act as a stronger electrophile due to its larger value of the global electrophilicity index ω = μ^2^/(2η) compared to **27** anion. The value of ω for reactants **29** dipole and **27** is 2.103 and 1.234 eV, respectively. 

The global nucleophilicity index *N* = E_HOMO_ − E_HOMO_ (TCE) is defined as the HOMO energy relative to the HOMO energy of the reference compound tetracyanoethylene (TCE), with all energies in eV, and both computed at the same level of theory [50]. This index was introduced in Ref. [50] and served to classify organic molecules as strong (*N* > 3.00 eV), moderate (2.00 eV < *N* < 3.00 eV), and marginal nucleophiles (*N* < 2.00 eV). From Table 3, we see that reactant **27** anion (*N* = 3.454 eV) is a significant stronger nucleophile than reactant **29** (*N* = 2.702 eV). 

#### 2.2.5. Possible Role of the Solvent

**Figure 11:**
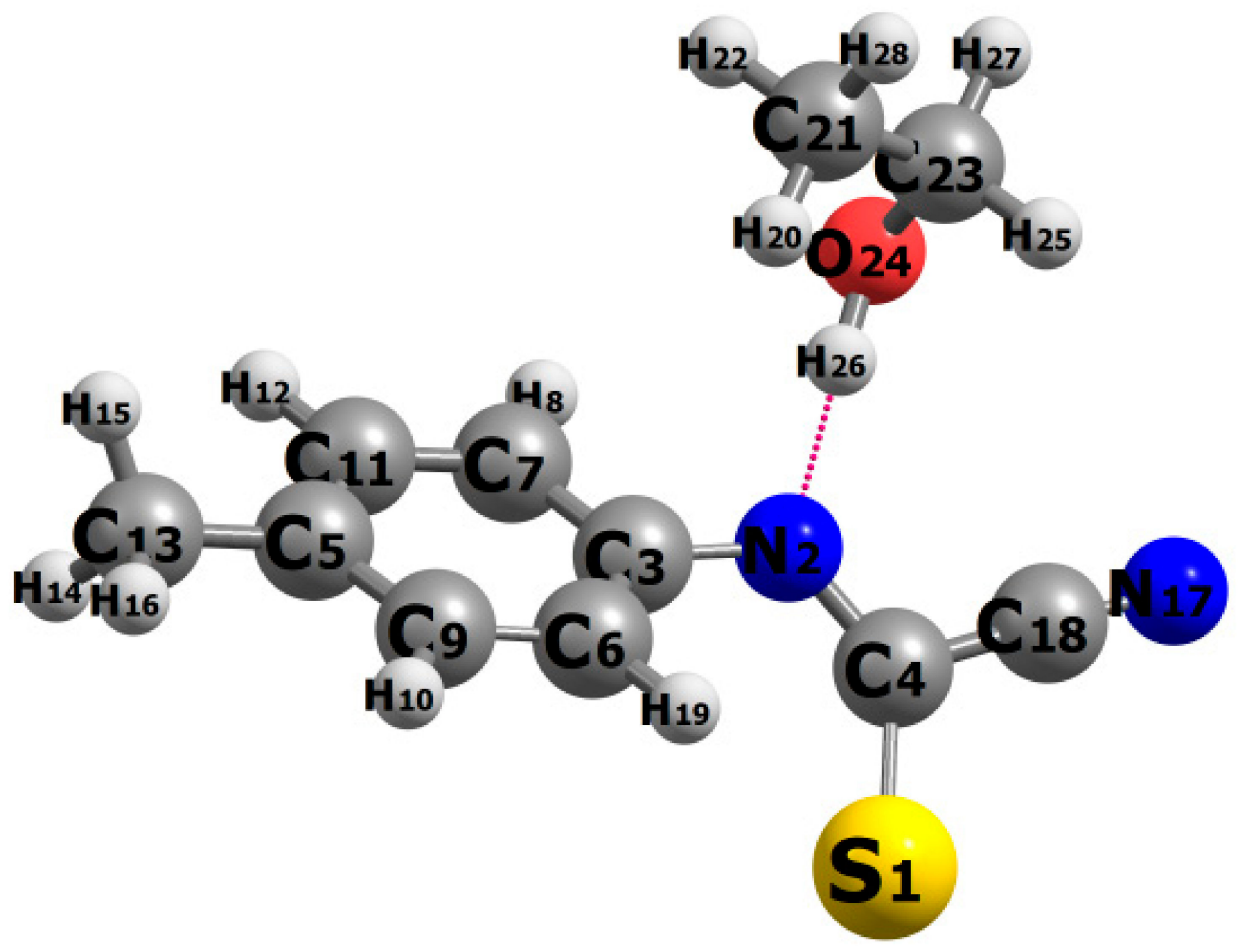
Relaxed structure of the hydrogen-bonded complex between deprotonated **27** (with R = CH_3_) at the bottom with an explicit ethanol molecule above. The violet dashes between H26 and N2 atoms indicate the intermolecular hydrogen bond. Color code for atoms: grey = C, blue = N, red = O, yellow = S, and white = H.

Although the above analysis (population analysis, FMO, global descriptors) gives considerable insight into the regioselectivity of the reaction, it totally neglects the possible effect of the solvent, which was described implicitly so far. The solvent may play an important role due to the strong polar character of the reactants involved (e.g., the calculated electric dipole moment of **29** zwitterion is 2.20 Debye) and the presence of various hydrogen bonding acceptor sites. In this final section, we preliminarily investigate the role of the solvent, and, to this end, we consider an explicit ethanol molecule in close proximity to the N2 and S1 sites of deprotonated **27**. Figure 11 shows the relaxed geometry of the system formed by an explicit ethanol molecule hydrogen bonded to the N2 atom of anionic **27** (H26∙∙∙N2, violet dashes). The intermolecular interaction energy was estimated to be about 7.81 kcal/mol and the optimized distance H26∙∙∙N2 was 1.7661 Å. Sulfur sites are known to be poor proton acceptors and indeed our geometry optimizations failed to coordinate the ethanol molecule with the S1 sulfur site. Thus, it seems likely that polar and protic solvents (such as ethanol, used here) block and reduce the nucleophilicity of the N2 site and, as a result, the reactivity is directed preferentially via the (unsolvated and freely available) sulfur site S1 in **27** towards the reactant **29**.

**Scheme 5:**
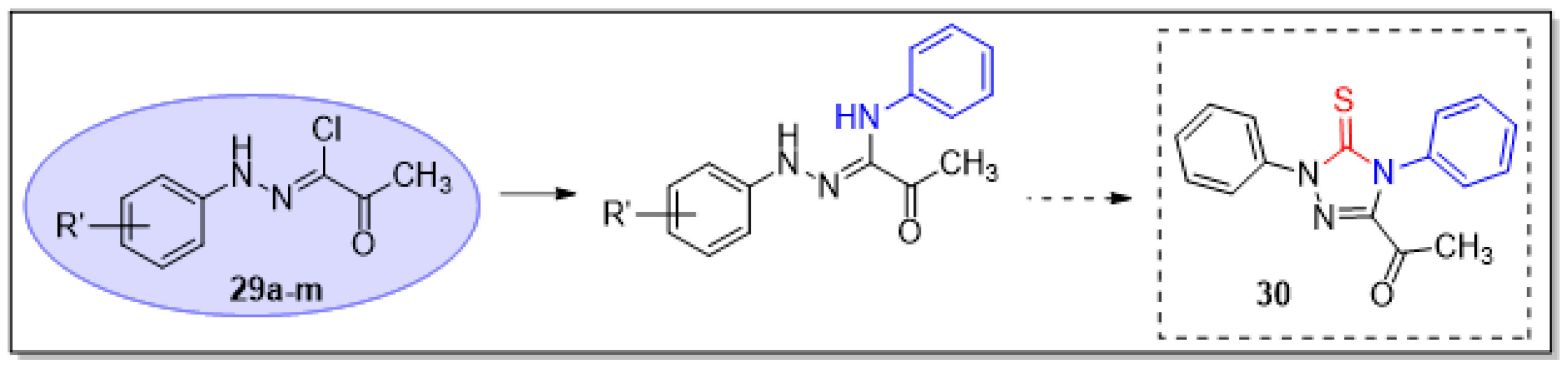
Proposed synthetic approach to prepare different derivatives of variously substituted 5-thioxo-1,2,4-triazoles.

Finally, there are no known or reported general literature methods to prepare the 5-thioxo-1,2,4-triazole regioisomer **30** (Scheme 5), which will not only serve as potentially bioactive sulfur heterocycles, but they could also be converted to the selenium analogs for comparison and further development. Thus, 1,1′-Thiocarbonyldiimidazole (TCDI) could convert variously substituted (*Z*)-2-oxo-*N*,*N*′-diphenylpropanehydrazonamide to **30** which could potentially be reacted with Woollins’ reagent to afford the selenium analogs. 

## 3. Materials and Methods

### 3.1. General Information

All chemical reactions were performed with magnetic stirring in oven-dried glassware. All chemical reagents and reaction solvents were used as received from MilliporeSigma without any further purification. Analytical thin-layer chromatography (TLC) used to track the progress of reactions or test purity of isolated products was performed on precoated silica gel plates (HSGF 254) and visualized under UV irradiation at λ = 254 nm. ^1^H and ^13^C NMR spectra were recorded in DMSO-d_6_ on a Varian 400 MHz NMR spectrometer. The NMR chemical shifts (δ (ppm)) are reported in parts per million (ppm) relative to the residual solvent peak (^1^H-NMR δ (ppm) 2.50 for DMSO-d_6_; δ (ppm) 39.52 for DMSO-d_6_). The following abbreviations were used to explain NMR peak multiplicities: br s = broad signal, s = singlet, d = doublet, t = triplet, and m = multiplet. IR spectra were recorded using a Thermo Nicolet Nexus 470 FT-IR. High-resolution mass analyses (HRMS) were obtained using a Waters Q-TOF Premier mass spectrometer (electrospray ionization (ESI)). Melting points were measured using a capillary melting point apparatus (MEL-TEMP) in degrees Celsius (°C) and are uncorrected. Single-crystal X-ray diffraction data (SC-XRD) were collected using a Rigaku Oxford Diffraction XtaLAB Synergy-S, equipped with two microfocus PhotonJet-S sources, Cu Kα radiation (λ = 1.54184 Å) and Mo Kα radiation (λ = 0.71073 Å), and a HyPix-6000HE hybrid photon counting detector. Data were processed and analyzed using CrysAlisPro software package, where data refinement was accomplished by Olex2. For computational details and further references, see Supplementary Materials Section.

### 3.2. General Procedure for the Preparation of N-Arylcyanothioformamide ***27a***–***x***

All *N-*Arylcyanothioformamide **27a**–**x** used herein were prepared according to our published procedure form various substituted isothiocyanates and potassium cyanide (Scheme 2a) [26]. These reactants were partially characterized by standard 1D NMR spectroscopy (^1^H and ^13^C) and the obtained spectral data matched those reported earlier [32]. 

### 3.3. General Procedure for the Preparation of (Z)-N-(Aryl)-2-Oxopropanehydrazonoyl Chlorides ***29a***–***m***

The aniline (10 mmol) in 250 mL round bottom flask immersed in an ice-water bath was treated with a cold solution of 6*M* HCl (6 mL) and stirred for 10 min, followed by the addition of 15–20 mL of cold water in cases where a very thick slurry formed during the acidification process. The resulting hydrochoride salt was diazotized with a solution of sodium nitrite (0.70 g, 10 mmol) in water (10 mL) to produce coloured solutions ranging from bright yellow to orange to brown, depending on the aniline substrate used. Immediate neutralization with 50 mL chilled ethnolic solution of sodium acetate trihydrate (1.36 g, 10 mmol) was carried out by dropwise addition of 50 mL ethanolic solution of 3-chloro-2,4-pentanedione (1.34 g, 10 mmol) over 20 min. The ice-water bath was removed and the reation was allowed to stir at room temperature for another 3 h. Water (100 mL) was added and the resulting solid was collected by vaccuum filtration using sintered glass funnel and washed with cold water and petroleum ether and dried in an oven (55–57 °C) for several hours to afford *(Z)-N*-(aryl)-2-oxopropanehydrazonoyl chlorides **29a**–**m** in the described yields. NMR analysis confirmed the purity of the hydrazonoyl chlorides which were used in the next step without further purification.

### 3.4. General Synthesis of 1,3,4-Thiadiazole Derivatives ***31***–***92***

In a well-ventilated fume hood, a stirred solution of the *N*-arylcyanothioformamide **27** (1.0 mmol) and hydrazonoyl chloride **29** (1.0 mmol) in ethanol (10 mL) was treated with Et_3_N (279 µL, 2.0 mmol) and the mixture stirred for 18 h at room temperature. The formed precipitate was filtered using Whatman filter paper, washed with minimum amount of ethanol, and air-dried to afford the desired 1,3,4-thiadiazole products **31**–**74**, **76**–**84**, and **90**–**92** as solid flakes. Compounds **75** and **85**–**89** never precipitated and were isolated as oils after ether/aqueous workup. In these cases, the reaction mixture was poured into 30 mL of ether and the ether layer was washed with saturated NaCl solution (2 × 30 mL), water (1 × 30 mL), dried (Na_2_SO_4_), and concentrated *in vacuo* to afford **75**, and **85**–**89** as clean oils which did not require any further purification as indicated by NMR analysis.

### 3.5. Experimental Data

The yields, m.p., and spectral data of compounds (**29a**–**m** and **31**–**92**) are shown below, and the corresponding spectra have been included in the Supplementary Materials Section:

**(*Z*)-2-oxo-*N*-phenylpropanehydrazonoyl chloride (29a)** [36,37]**.**

Bright yellow solid (85% yield); mp 138–139 °C; IR (KBr) 3253 (NH), 1686 (C=O), 1603 (C=N-N), 1360, 1286, 1232, 1023, 954, 846, 752, 687, 603, 552, 493 cm^−1^; ^1^H NMR (CDCl_3_, 400 MHz) δ 8.49 (broad s, 1H, NH), 7.37 (t, *J* = 8.4 Hz, 2H, Ar-H), 7.24 (d, *J* = 8.4 Hz, 2H, Ar-H), 7.08 (t, *J* = 7.6 Hz, 1H, Ar-H), 2.57 (s, 3H, C(O)-**CH_3_**); ^13^C NMR (CDCl_3_, 100 MHz) δ 188.4 (C=O), 141.2 (C-N), 129.6 (2 × CH), 125.1 (C=N-N), 123.5 (CH), 114.4 (2 × CH), 25.3 (C(O)-**C**H_3_); HRMS (ESI^+^): *m*/*z* [M + H]^+^ calcd for C_9_H_10_ClN_2_O: 197.0482; found: 197.0497.

**(*Z*)-2-oxo-*N*-(*p*-tolyl)propanehydrazonoyl chloride (29b)** [37]**.**

Yellow solid (88% yield); mp 146–148 °C; IR (KBr) 3246 (NH), 1688 (C=O), 1543 (C=N-N), 1355, 1303, 1236, 1024, 935, 817, 630, 589, 509, 490 cm^−1^; ^1^H NMR (CDCl_3_, 400 MHz) δ 8.44 (broad s, 1H, NH), 7.16 (d, *J* = 8.4 Hz, 2H, Ar-H), 7.13 (d, *J* = 8.4 Hz, 2H, Ar-H), 2.56 (s, 3H, C(O)-**CH_3_**), 2.33 (s, 3H, CH_3-p-tolyl_); ^13^C NMR (CDCl_3_, 100 MHz) δ 188.3 (C=O), 139.0 (ArC_q_), 133.2 (C-N), 130.0 (2 × CH), 124.6 (C=N-N), 114.4 (2 × CH), 25.2 (C(O)-**C**H_3_), 20.7 (Ar-**C**H_3_); HRMS (ESI^+^): *m*/*z* [M + H]^+^ calcd for C_10_H_12_ClN_2_O: 211.0638; found: 211.0627.

***(Z)-N*-(4-chlorophenyl)-2-oxopropanehydrazonoyl chloride (29c)** [37]**.**

Yellow solid (87% yield); mp 174–175 °C; IR (KBr) 3249 (NH), 1693 (C=O), 1598 (C=N-N), 1404, 1357, 1270, 1240, 1085, 1008, 933, 831, 728, 649, 629, 584, 500 cm^−1^; ^1^H NMR (DMSO-d_6_, 400 MHz) δ 10.77 (broad s, 1H, NH), 7.42 (d, *J* = 9.2 Hz, 2H, Ar-H), 7.37 (d, *J* = 9.2 Hz, 2H, Ar-H), 2.46 (s, 3H, CH_3_); ^13^C NMR (DMSO-d_6_, 100 MHz) δ 188.0 (C=O), 141.6 (C-N), 129.2 (2 × CH), 126.4 (C=N-N), 123.5 (C-Cl), 116.4 (2 × CH), 25.4 (C(O)-**C**H_3_); HRMS (ESI^+^): *m*/*z* [M + H]^+^ calcd for C_9_H_9_Cl_2_N_2_O: 231.0092; found: 231.0100.

***(Z)-N*-(4-bromophenyl)-2-oxopropanehydrazonoyl chloride (29d)** [37]**.**

Bright yellow flakes (98% yield); mp 164–166 °C; IR (KBr) 3248 (NH), 1687 (C=O), 1595 (C=N-N), 1543, 1481, 1357, 1225, 1071, 1027, 935, 828, 578, 504 cm^−1^; ^1^H NMR (CDCl_3_, 400 MHz) δ 8.45 (broad s, 1H, NH), 7.46 (d, *J* = 8.8 Hz, 2H, Ar-H), 7.12 (d, *J* = 8.8 Hz, 2H, Ar-H), 2.56 (s, 3H, CH_3_); ^13^C NMR (CDCl_3_, 100 MHz) δ 188.2 (C=O), 140.4 (C-N), 132.5 (2 × CH), 125.9 (C=N-N), 116.0 (2 × CH), 115.9 (C-Br), 25.3 (C(O)-**C**H_3_); HRMS (ESI^+^): *m*/*z* [M + H]^+^ calcd for C_9_H_9_BrClN_2_O: 274.9587; found: 274.9579.

***(Z)-N*-(4-iodophenyl)-2-oxopropanehydrazonoyl chloride (29e)** [38]**.**

Light grey flakes (84% yield); mp 168–169 °C; IR (KBr) 3244 (NH), 1681 (C=O), 1590 (C=N-N), 1537, 1499, 1357, 1221, 1176, 1025, 935, 825 cm^−1^; ^1^H NMR (CDCl_3_, 400 MHz) δ 8.43 (broad s, 1H, NH), 7.65 (d, *J* = 8.8 Hz, 2H, Ar-H), 7.00 (d, *J* = 8.8 Hz, 2H, Ar-H), 2.56 (s, 3H, CH_3_); ^13^C NMR (CDCl_3_, 100 MHz) δ 188.2 (C=O), 141.1 (C-N), 138.4 (2 × CH), 126.0 (C=N-N), 116.4 (2 × CH), 86.1 (C-I), 25.3 (C(O)-**C**H_3_); HRMS (ESI^+^): *m*/*z* [M + H]^+^ calcd for C_9_H_9_IClN_2_O: 322.9448; found: 322.9940.

***(Z)-N*-(4-(methylthio)phenyl)-2-oxopropanehydrazonoyl chloride (29f)** [39]**.**

Green solid (73% yield); lit. mp 109–110 °C [39]; mp 109–110 °C; IR (KBr) 3237 (NH), 1677 (C=O), 1599 (C=N-N), 1357, 1400, 1233, 1023, 824, 644, 582, 505, 491 cm^−1^; ^1^H NMR (CDCl_3_, 400 MHz) δ 8.47 (broad s, 1H, NH), 7.28 (d, *J* = 8.8 Hz, 2H, Ar-H), 7.17 (d, *J* = 8.8 Hz, 2H, Ar-H), 2.55 (s, 3H, C(O)-**CH_3_**), 2.48 (s, 3H, CH_3-methylthio_); ^13^C NMR (CDCl_3_, 100 MHz) δ 188.2 (C=O), 139.2 (ArC_q_), 132.7 (C-N), 128.8 (2 × CH), 125.2 (C=N-N), 115.1 (2 × CH), 25.3 (C(O)-**C**H_3_), 16.9 (Ar-S**C**H_3_); HRMS (ESI^+^): *m*/*z* [M + H]^+^ calcd for C_10_H_12_ClN_2_OS: 243.0359; found: 243.0371.

**(*Z*)-** **2-oxo-*N*-(*m*-tolyl)propanehydrazonoyl chloride (29g) [40]** **.**

Off-white solid (85% yield); mp 148–149 °C; IR (KBr) 3259 (NH), 1680 (C=O), 1614 (C=N-N), 1552, 1160, 1027, 940, 894, 770, 690, 610, 493 cm^−1^; ^1^H NMR (CDCl_3_, 400 MHz) δ 8.43 (broad s, 1H, NH), 7.25 (t, *J* = 8.0 Hz, 1H, Ar-H), 7.07–7.01 (m, 2H, Ar-H), 6.90 (d, *J* = 7.2 Hz, 1H, Ar-H), 2.57 (s, 3H, C(O)-**CH_3_**), 2.38 (s, 3H, CH_3-m-tolyl_); ^13^C NMR (CDCl_3_, 100 MHz) δ 188.4 (C=O), 141.2 (ArC_q_), 139.6 (C-N), 129.4 (CH), 125.0 (C=N-N), 124.4 (CH), 115.0 (CH), 111.6 (CH), 25.3 (C(O)-**C**H_3_), 21.5 (Ar-**C**H_3_); HRMS (ESI^+^): *m*/*z* [M + H]^+^ calcd for C_10_H_12_ClN_2_O: 211.0638; found: 211.0627.

**(*Z*)-2-oxo-*N*-(3-(trifluoromethyl)phenyl)propanehydrazonoyl chloride (29h)** [41]**.**

Bright yellow solid (91% yield); lit. mp 125 °C [41]; mp 167–168 °C; IR (KBr) 3236 (NH), 1672 (C=O), 1621 (C=N-N), 1600, 1490, 1419, 1359, 943, 886, 790, 720, 698, 653, 557, 500 cm^−1^; ^1^H NMR (CDCl_3_, 400 MHz) δ 8.58 (broad s, 1H, NH), 7.51–7.45 (m, 2H, Ar-H), 7.41 (d, *J* = 8.4 Hz, 1H, Ar-H), 7.32 (d, *J* = 7.6 Hz, 1H, Ar-H), 2.59 (s, 3H, C(O)-**CH_3_**); ^13^C NMR (CDCl_3_, 100 MHz) δ 188.3 (C=O), 141.8 (C-N), 132.1 (q, *J* = 32.0 Hz, **C-**CF_3_), 130.2 (CH), 126.5 (C=N-N), 123.7 (q, *J* = 272.0 Hz, CF_3_), 119.9 (q, *J* = 4.0 Hz, CH), 117.5 (CH), 111.2 (q, *J* = 4.0 Hz, CH), 25.4 (C(O**C**H_3_); HRMS (ESI^+^): *m*/*z* [M + H]^+^ calcd for C_10_H_9_ClF_3_N_2_O: 265.0356; found: 265.0345.

***(Z)-N*-(4-methoxyphenyl)-2-oxopropanehydrazonoyl chloride (29i)** [41]**.**

Green solid (82% yield); lit. mp 118 °C [41]; mp 120–122 °C; IR (KBr) 3245 (NH), 1681 (C=O), 1541 (C=N-N), 1298, 1219, 1024, 828, 647, 594 cm^−1^; ^1^H NMR (CDCl_3_, 400 MHz) δ 8.42 (broad s, 1H, NH), 7.17 (d, *J* = 8.8 Hz, 2H, Ar-H), 6.91 (d, *J* = 8.8 Hz, 2H, Ar-H), 3.80 (s, 3H, OCH_3_), 2.54 (s, 3H, CH_3_); ^13^C NMR (CDCl_3_, 100 MHz) δ 188.3 (C=O), 156.1 (C-O), 135.0 (C-N), 124.2 (C=N-N), 115.7 (2 × CH), 114.8 (2 × CH), 55.6 (OMe), 25.2 (C(O)-**C**H_3_); HRMS (ESI^+^): *m*/*z* [M + H]^+^ calcd for C_10_H_12_ClN_2_O_2_: 227.0587; found: 227.0595.

***(Z)-N*-(3,5-bis(trifluoromethyl)phenyl)-2-oxopropanehydrazonoyl chloride (29j)** [42]**.**

Off-white solid (81% yield); mp 179–181 °C; IR (KBr) 3257 (NH), 1684 (C=O), 1620 (C=N-N), 1557, 1475, 1379, 1291, 1189, 1030, 956, 920, 887, 703, 613, 498 cm^−1^; ^1^H NMR (DMSO-d_6_, 400 MHz) δ 11.15 (broad s, 1H, NH), 7.97 (s, 2H, Ar-H), 7.66 (s, 1H, Ar-H), 2.53 (s, 3H, C(O)-**CH_3_**); ^13^C NMR (CDCl_3_, 100 MHz) δ 188.0 (C=O), 144.4 (C-N), 131.4 (q, *J* = 33.0 Hz, **C-**CF_3_), 126.0 (C=N-N), 123.32 (q, *J* = 271.0 Hz, CF_3_), 114.8 (sept, *J* = 4.0 Hz, CH), 114.6 (q, *J* = 4.0 Hz, 2 × CH), 25.3 (C(O**C**H_3_); HRMS (ESI^+^): *m*/*z* [M + H]^+^ calcd for C_11_H_8_ClF_6_N_2_O: 333.0229; found: 333.039.

***(Z)-N*-(naphthalen-1-yl)-2-oxopropanehydrazonoyl chloride (29k)** [37]**.**

Dark brown solid (61% yield); mp 91–93 °C; IR (KBr) 3348 (NH), 1700 (C=O), 1596 (C=N-N), 1556, 1400, 1220, 1202, 1007, 935, 853, 764, 680, 605, 540, 458 cm^−1^; ^1^H NMR (CDCl_3_, 400 MHz) δ 9.04 (broad s, 1H, NH), 7.89 (t, *J* = 8.0 Hz, 2H, Ar-H), 7.65–7.46 (m, 5H, Ar-H), 2.63 (s, 3H, C(O)-**CH_3_**); ^13^C NMR (CDCl_3_, 100 MHz) δ 188.3 (C=O), 135.8 (C-N), 134.1 (q), 129.0 (CH), 126.9 (C=N-N), 126.4 (CH), 126.3 (CH), 126.1 (CH), 123.7 (CH), 122.4 (q), 118.8 (CH), 110.7 (CH), 25.4 (C(O)-**C**H_3_); HRMS (ESI^+^): *m*/*z* [M + H]^+^ calcd for C_13_H_12_ClN_2_O: 247.0638; found: 247.0626.

***(Z)-N*** **-(2,3-difluorophenyl)-2-oxopropanehydrazonoyl chloride (29l).**

Bright yellow solid (65% yield); mp 112–114 °C; IR (KBr) 3338 (NH), 1709 (C=O), 1632 (C=N-N), 1567, 1528, 1454, 1361, 1302, 1251, 1188, 1036, 983, 928, 818, 928, 781, 703, 600, 580, 450 cm^−1^; ^1^H NMR (CDCl_3_, 400 MHz) δ 8.53 (broad s, 1H, NH), 7.31 (dd, *J* = 8.4, 6.8 Hz, 1H, Ar-H), 7.13–7.05 (m, 1H, Ar-H), 6.90–6.80 (m, 1H, Ar-H), 2.57 (s, 3H, C(O)-CH_3_); ^13^C NMR (CDCl_3_, 100 MHz) δ 188.1 (C=O), 150.7 (dd, *J* = 246.0, 10.0 Hz, **C-**F), 131.6 (dd, *J* = 7.0, 3.0 Hz, C-N), 128.1 (C=N-N), 124.6 (dd, *J* = 8.0, 5.0 Hz, CH), 110.8 (d, *J* = 17.0 Hz, CH), 110.6 (d, *J* = 3.0 Hz, CH), 25.3 (C(O)-**C**H_3_); HRMS (ESI^+^): *m*/*z* [M + H]^+^ calcd for C_9_H_8_ClF_2_N_2_O: 233.0293; found: 233.0301.

***(Z)-N*-(benzo[d][1,3]dioxol-5-yl)-2-oxopropanehydrazonoyl chloride (29m)** [43]**.**

Brown solid (72% yield); mp 135–137 °C; IR (KBr) 3245 (NH), 1681 (C=O), 1538 (C=N-N), 1363, 1306, 1263, 1039, 922, 848, 814, 700, 632, 581, 508 cm^−1^; ^1^H NMR (CDCl_3_, 400 MHz) δ 8.40 (broad s, 1H, NH), 6.87 (d, *J* = 1.2 Hz, 1H, Ar-H), 6.77 (d, *J* = 8.4 Hz, 1H, Ar-H), 6.60 (dd, *J* = 8.4, 1.2 Hz, 1H, Ar-H), 5.97 (s, 2H, OCH_2_), 2.54 (s, 3H, CH_3_); ^13^C NMR (CDCl_3_, 100 MHz) δ 188.2 (C=O), 148.7 (C-O), 143.9 (C-O), 136.5 (C-N), 124.4 (C=N-N), 108.6 (CH), 107.1 (CH), 101.4 (OCH_2_), 96.8 (CH), 25.2 (C(O)-**C**H_3_); HRMS (ESI^+^): *m*/*z* [M + H]^+^ calcd for C_10_H_10_ClN_2_O_3_: 241.0380; found: 241.0391.

**(*Z*)-1-(5-((4-nitrophenyl)imino)-4-phenyl-4,5-dihydro-1,3,4-thiadiazol-2-yl)ethan-1-one (31).**

Yellow solid (77% yield); mp 167 °C; IR (KBr) 1687 (C=O), 1618 (C=N), 1570, 1537, 1503, 1483, 1365, 1328, 1276, 1253, 1138, 1100, 948, 863, 840, 756, 734, 714, 686, 616, 598, 561, 524, 495 cm^−1^; ^1^H NMR (CDCl_3_, 400 MHz) δ (ppm) 8.25 (d, *J* = 8.8 Hz, 2H, Ar-H_p-nitrophenyl_), 7.91 (d, *J* = 7.6 Hz, 2H, Ar-H_ph_), 7.54 (t, *J* = 7.6 Hz, 2H, Ar-H_ph_), 7.42 (t, *J* = 7.6 Hz, 1H, Ar-H_ph_), 7.13 (d, *J* = 8.8 Hz, 2H, Ar-H_p-nitrophenyl_), 2.63 (s, 3H, CH_3_); ^13^C NMR (CDCl_3_, 100 MHz) δ (ppm) 189.2 (C=O), 157.1 (N=C-N), 156.4 (**C**-NO_2_), 147.4 (C=N-N), 144.0 (C-N_p-nitrophenyl_), 138.2 (C-N_ph_), 129.0 (2 × CH_ph_), 128.0 (CH_ph_), 125.7 (2 × CH_p-nitrophenyl_), 123.4 (2 × CH_ph_), 121.1 (2 × CH_p-nitrophenyl_), 25.0 (C(O)-**C**H_3_); HRMS (ESI^+^): *m*/*z* [M + H]^+^ calcd for C_16_H_13_N_4_O_3_S: 341.0708; found: 341.0717.

**(*Z*)-1-(5-((4-nitrophenyl)imino)-4-(*p*-tolyl)-4,5-dihydro-1,3,4-thiadiazol-2-yl)ethan-1-one (32).**

Yellow solid (78% yield); mp 146 °C; IR (KBr) 1685 (C=O), 1618 (C=N), 1572, 1534 (C=S), 1508, 1335, 1280, 1257, 1144, 1103, 1057, 946, 862, 842, 810, 785, 753, 727, 695, 617, 595, 550, 522, 498 cm^−1^; ^1^H NMR (CDCl_3_, 400 MHz) δ (ppm) 8.24 (d, *J* = 8.8 Hz, 2H, Ar-H_p-nitrophenyl_), 7.74 (d, *J* = 8.4 Hz, 2H, Ar-H_p-tolyl_), 7.32 (d, *J* = 8.4 Hz, 2H, Ar-H_p-tolyl_), 7.13 (d, *J* = 8.8 Hz, 2H, Ar-H_p-nitrophenyl_), 2.62 (s, 3H, CH_3_), 2.43 (s, 3H, CH_3_, Ar-CH_3_); ^13^C NMR (CDCl_3_, 100 MHz) δ (ppm) 189.2 (C=O), 157.3 (N=C-N), 156.5 (**C**-NO_2_), 147.2 (C=N-N), 143.9 (C-N_p-nitrophenyl_), 138.3 (C-N_p-tolyl_), 135.7 (Ar**C_q_**-Me), 129.6 (2 × CH_p-tolyl_), 125.7 (2 × CH_p-nitrophenyl_), 123.6 (2 × CH_p-tolyl_), 121.1 (2 × CH_p-nitrophenyl_), 25.1 (C(O)-**C**H_3_), 21.2 (*p*-tolyl-**C**H_3_); HRMS (ESI^+^): *m*/*z* [M + H]^+^ calcd for C_17_H_15_N_4_O_3_S: 355.0865; found: 355.0873.

**(*Z*)-1-(4-(4-chlorophenyl)-5-((4-nitrophenyl)imino)-4,5-dihydro-1,3,4-thiadiazol-2-yl)ethan-1-one (33).**

Yellow solid (55% yield); mp 184–186 °C; IR (KBr) 1685 (C=O), 1619 (C=N), 1567, 1538 (C=S), 1504, 1484, 1328, 1283, 1254, 1143, 1093, 1010, 950, 865, 830, 757, 742, 699, 617, 597, 522, 503, 477 cm^−1^; ^1^H NMR (CDCl_3_, 400 MHz) δ (ppm) 8.26 (d, *J* = 8.4 Hz, 2H, Ar-H_p-nitrophenyl_), 7.90 (d, *J* = 8.8 Hz, 2H, Ar-H_p-chlorophenyl_), 7.48 (d, *J* = 8.8 Hz, 2H, Ar-H_p-chlorophenyl_), 7.14 (d, *J* = 8.4 Hz, 2H, Ar-H_p-nitrophenyl_), 2.63 (s, 3H, CH_3_); ^13^C NMR (CDCl_3_, 100 MHz) δ (ppm) 189.1 (C=O), 156.9 (N=C-N), 156.1 (**C**-NO_2_), 147.8 (C=N-N), 144.1 (C-N_p-nitrophenyl_), 136.8 (C-N_p-nitrophenyl_), 133.4 (C-Cl), 129.1 (2 × CH_p-chlorophenyl_), 125.6 (2 × CH_p-nitrophenyl_), 124.4 (2 × CH_p-chlorophenyl_), 121.1 (2 × CH_p-nitrophenyl_), 24.9 (C(O)-**C**H_3_); HRMS (ESI^+^): *m*/*z* [M + H]^+^ calcd for C_16_H_12_ClN_4_O_3_S: 375.0319; found: 375.0311.

**(*Z*)-1-(4-(4-bromophenyl)-5-((4-nitrophenyl)imino)-4,5-dihydro-1,3,4-thiadiazol-2-yl)ethan-1-one (34).**

Yellow solid (61% yield); mp 192–194 °C; IR (KBr) 1684 (C=O), 1618 (C=N), 1560, 1538 (C=S), 1504, 1480, 1330, 1281, 1254, 1142, 1101, 1073, 1007, 949, 864, 840, 828, 757, 738, 697, 617, 598, 523, 499, 418 cm^−1^; ^1^H NMR (CDCl_3_, 400 MHz) δ (ppm) 8.26 (d, *J* = 8.8 Hz, 2H, Ar-H_p-nitrophenyl_), 7.85 (d, *J* = 8.4 Hz, 2H, Ar-H_p-bromophenyl_), 7.63 (d, *J* = 8.4 Hz, 2H, Ar-H_p-bromophenyl_), 7.14 (d, *J* = 8.8 Hz, 2H, Ar-H_p-nitrophenyl_), 2.63 (s, 3H, CH_3_); ^13^C NMR (CDCl_3_, 100 MHz) δ (ppm) 189.1 (C=O), 156.8 (N=C-N), 156.1 (**C**-NO_2_), 147.9 (C=N-N), 144.1 (C-N_p-nitrophenyl_), 137.3 (C-N_p-bromophenyl_), 132.1 (2 × CH_p-bromophenyl_), 125.8 (2 × CH_p-nitrophenyl_), 124.7 (2 × CH_p-bromophenyl_), 121.4 (C-Br), 121.1 (2 × CH_p-nitrophenyl_), 25.1 (C(O)-**C**H_3_); HRMS (ESI^+^): *m*/*z* [M + H]^+^ calcd for C_16_H_12_BrN_4_O_3_S: 418.9813; found: 418.9824.

**(*Z*)-1-(4-(4-iodophenyl)-5-((4-nitrophenyl)imino)-4,5-dihydro-1,3,4-thiadiazol-2-yl)ethan-1-one (35).**

Light brown solid (77% yield); mp 198–200 °C; IR (KBr) IR (KBr) 1688 (C=O), 1633 (C=N), 1575, 1537 (C=S), 1509, 1480, 1370, 1343, 1280, 1253, 1147, 1096, 1003, 943, 865, 840, 817, 755, 733, 698, 618, 592, 519, 497 cm^−1^; ^1^H NMR (CDCl_3_, 400 MHz) δ (ppm) 8.26 (d, *J* = 8.8 Hz, 2H, Ar-H_p-nitrophenyl_), 7.83 (d, *J* = 8.4 Hz, 2H, Ar-H_p-iodophenyl_), 7.72 (d, *J* = 8.4 Hz, 2H, Ar-H_p-iodophenyl_), 7.13 (d, *J* = 8.8 Hz, 2H, Ar-H_p-nitrophenyl_), 2.62 (s, 3H, CH_3_); ^13^C NMR (CDCl_3_, 100 MHz) δ (ppm) 189.1 (C=O), 156.8 (N=C-N), 156.1 (**C**-NO_2_), 147.9 (C=N-N), 144.1 (C-N_p-nitrophenyl_), 138.1 (2 × CH_p-iodophenyl_), 138.0 (C-N_p-nitrophenyl_), 125.8 (2 × CH_p-nitrophenyl_), 124.8 (2 × CH_p-iodophenyl_), 121.1 (2 × CH_p-nitrophenyl_), 92.7 (C-I), 25.1 (C(O)-**C**H_3_); HRMS (ESI^+^): *m*/*z* [M + H]^+^ calcd for C_16_H_12_IN_4_O_3_S: 466.9675; found: 466.9666.

**(*Z*)-1-(4-(4-(methylthio)phenyl)-5-((4-nitrophenyl)imino)-4,5-dihydro-1,3,4-thiadiazol-2-yl)ethan-1-one (36).**

Bright yellow (71% yield); mp 154–156 °C; IR (KBr) 1692 (C=O), 1614 (C=N), 1595, 1570 (C=S), 1513, 1486, 1367, 1341, 1287, 1254, 1148, 1107, 1089, 1013, 945, 864, 840, 813, 756, 697, 619, 596, 521 cm^−1^; ^1^H NMR (CDCl_3_, 400 MHz) δ (ppm) 8.25 (d, *J* = 8.8 Hz, 2H, Ar-H_p-nitrophenyl_), 7.82 (d, *J* = 8.4 Hz, 2H, Ar-H_p-methylthiophenyl_), 7.36 (d, *J* = 8.4 Hz, 2H, Ar-H_p-methylthiophenyl_), 7.14 (d, *J* = 8.8 Hz, 2H, Ar-H_p-nitrophenyl_), 2.62 (s, 3H, C(O)**C**H_3_), 2.53 (s, 3H, CH_3_, S-CH_3_); ^13^C NMR (CDCl_3_, 100 MHz) δ (ppm) 189.1 (C=O), 157.1 (N=C-N), 156.3 (**C**-NO_2_), 147.4 (C=N-N), 144.0 (C-N_p-nitrophenyl_), 139.0 (Ar**C_q_**-SMe), 135.1 (C-N_p-methylthiophenyl_), 126.4 (2 × CH_p-methylthiophenyl_), 125.7 (2 × CH_p-nitrophenyl_), 123.8 (2 × CH_p-methylthiophenyl_), 121.1 (2 × CH_p-nitrophenyl_), 25.1 (C(O)-**C**H_3_), 15.6 (S-**C**H_3_); HRMS (ESI^+^): *m*/*z* [M + H]^+^ calcd for C_17_H_15_N_4_O_3_S_2_: 387.0586; found: 387.0574.

**(*Z*)-1-(5-((4-nitrophenyl)imino)-4-(m-tolyl)-4,5-dihydro-1,3,4-thiadiazol-2-yl)ethan-1-one (37).**

Bright yellow solid (78% yield); mp 154–156 °C; IR (KBr) 1687 (C=O), 1623 (C=N), 1581, 1537 (C=S), 1341, 1294, 1259, 1145, 1104, 1061, 947, 857, 840, 776, 753, 714, 698, 683, 616, 592, 567, 518, 496, 441 cm^−1^; ^1^H NMR (CDCl_3_, 400 MHz) δ (ppm) 8.24 (d, *J* = 8.8 Hz, 2H, Ar-H_p-nitrophenyl_), 7.68 (d, *J* = 8.4 Hz, 1H, Ar-H_m-tolyl_), 7.65 (s, 1H, Ar-H_m-tolyl_), 7.41 (t, *J* = 8.0 Hz, 1H, Ar-H_m-tolyl_), 7.23 (d, *J* = 8.4 Hz, 1H, Ar-H_m-tolyl_), 7.14 (d, *J* = 8.8 Hz, 2H, Ar-H_p-nitrophenyl_), 2.62 (s, 3H, CH_3_), 2.46 (s, 3H, CH_3_, Ar-CH_3_); ^13^C NMR (CDCl_3_, 100 MHz) δ (ppm) 189.2 (C=O), 157.3 (N=C-N), 156.5 (**C**-NO_2_), 147.3 (C=N-N), 143.9 (C-N_p-nitrophenyl_), 139.2 (Ar**C_q_**-Me), 138.0 (C-N_m-tolyl_), 129.0 (CH_m-tolyl_), 128.8 (CH_m-tolyl_), 125.7 (2 × CH_p-nitrophenyl_), 124.2 (CH_m-tolyl_), 121.1 (2 × CH_p-nitrophenyl_), 120.8 (CH_m-tolyl_), 25.1 (C(O)-**C**H_3_), 21.5 (m-tolyl-**C**H_3_); HRMS (ESI^+^): *m*/*z* [M + H]^+^ calcd for C_17_H_15_N_4_O_3_S: 355.0865; found: 355.0877.

**(*Z*)-1-(5-((4-nitrophenyl)imino)-4-(3-(trifluoromethyl)phenyl)-4,5-dihydro-1,3,4-thiadiazol-2-yl)ethan-1-one (38).**

Yellow solid (61% yield); mp 185–186 °C; IR (KBr) 1689 (C=O), 1625 (C=N), 1596, 1574 (C=S), 1543, 1507, 1485, 1452, 1331, 1309, 1252, 1182, 1141, 1108, 1072, 948, 896, 857, 838, 801, 781, 756, 734, 716, 695, 667, 616, 593, 559, 525, 495 cm^−1^; ^1^H NMR (CDCl_3_, 400 MHz) δ (ppm) 8.27 (d, *J* = 8.8 Hz, 2H, Ar-H_p-nitrophenyl_), 8.25–8.19 (m, 2H, Ar-H_m-(trifluoromethyl)phenyl_), 7.69–7.64 (m, 2H, Ar-H_m-(trifluoromethyl)phenyl_), 7.15 (d, *J* = 8.8 Hz, 2H, Ar-H_p-nitrophenyl_), 2.66 (s, 3H, CH_3_); ^13^C NMR (CDCl_3_, 100 MHz) δ (ppm) 189.1 (C=O), 156.7 (N=C-N), 155.9 (**C**-NO_2_), 148.3 (C=N-N), 144.2 (C-N_p-nitrophenyl_), 138.8 (**C**-N_m-(trifluoromethyl)phenyl_), 131.5 (q, *J* = 32.9 Hz, Ar**C_q_**-CF_3_), 129.6 (s, CH_p-(trifluoromethyl)phenyl_), 126.1 (q, *J* = 1.0 Hz, CH_m-(trifluoromethyl)phenyl_), 125.8 (2 × CH_p-nitrophenyl_), 124.3 (q, *J* = 3.7 Hz, CH_m-(trifluoromethyl)phenyl_), 123.5 (q, *J* = 270.8 Hz, **C**F_3_), 121.1 (2 × CH_p-nitrophenyl_), 120.0 (q, *J* = 7.6 Hz, CH_m-(trifluoromethyl)phenyl_), 25.1 (C(O)-**C**H_3_); HRMS (ESI^+^): *m*/*z* [M + H]^+^ calcd for C_17_H_12_F_3_N_4_O_3_S: 409.0582; found: 409.0594.

**(*Z*)-1-(4-(4-methoxyphenyl)-5-((4-nitrophenyl)imino)-4,5-dihydro-1,3,4-thiadiazol-2-yl)ethan-1-one (39).**

Bright yellow solid (73% yield); mp 173–174 °C; IR (KBr) 1693 (C=O), 1613 (C=N), 1575, 1509, 1370, 1342, 1254, 1181, 1147, 1102, 1026, 947, 868, 840, 754, 729, 696, 621, 598, 551, 521 cm^−1^; ^1^H NMR (CDCl_3_, 400 MHz) δ (ppm) 8.24 (d, *J* = 8.8 Hz, 2H, Ar-H_p-nitrophenyl_), 7.75 (d, *J* = 9.2 Hz, 2H, Ar-H_p-methoxyphenyl_), 7.13 (d, *J* = 8.8 Hz, 2H, Ar-H_p-nitrophenyl_), 7.03 (d, *J* = 9.2 Hz, 2H, Ar-H_p-methoxyphenyl_), 3.87 (s, 3H, OCH_3_), 2.61 (s, 3H, C(O)**C**H_3_); ^13^C NMR (CDCl_3_, 100 MHz) δ (ppm) 189.1 (C=O), 159.1 (C-O), 157.5 (N=C-N), 156.5 (**C**-NO_2_), 147.0 (C=N-N), 143.8 (C-N_p-nitrophenyl_), 131.0 (C-N_p-methoxyphenyl_), 125.7 (2 × CH_p-nitrophenyl_), 125.4 (2 × CH_p-methoxyphenyl_), 121.1 (2 × CH_p-nitrophenyl_), 114.2 (2 × CH_p-methoxyphenyl_), 55.6 (OCH_3_), 25.0 (C(O)-**C**H_3_); HRMS (ESI^+^): *m*/*z* [M + H]^+^ calcd for C_17_H_15_N_4_O_4_S: 371.0814; found: 371.0821.

**(*Z*)-1-(4-(3,5-bis(trifluoromethyl)phenyl)-5-((4-nitrophenyl)imino)-4,5-dihydro-1,3,4-thiadiazol-2-yl)ethan-1-one (40).**

Off white solid (63% yield); mp 181–184 °C; IR (KBr) 1698 (C=O), 1624 (C=N), 1600, 1583, 1516 (C=S), 1468, 1390, 1345, 1317, 1287, 1246, 1176, 1134, 1103, 914, 898, 857, 649, 774, 727, 701, 684, 659, 591, 559, 513 cm^−1^; ^1^H NMR (CDCl_3_, 400 MHz) δ (ppm) 8.58 (s, 2H, Ar-H_(trifluoromethyl)phenyl_), 8.29 (d, *J* = 8.8 Hz, 2H, Ar-H_p-nitrophenyl_), 7.88 (s, 1H, Ar-H_(trifluoromethyl)phenyl_), 7.17 (d, *J* = 8.8 Hz, 2H, Ar-H_p-nitrophenyl_), 2.69 (s, 3H, CH_3_, COCH_3_); ^13^C NMR (CDCl_3_, 100 MHz) δ (ppm) 188.9 (C=O), 156.3 (N=C-N), 155.3 (**C**-NO_2_), 149.2 (C=N-N), 144.5 (C-N_p-nitrophenyl_), 139.6 (**C**-N_(trifluoromethyl)phenyl_), 132.5 (q, *J* = 33.8 Hz, Ar**C_q_**-CF_3_), 125.8 2 × CH_p-nitrophenyl_), 122.8 (q, *J* = 272.0 Hz, **C**F_3_), 122.5 (q, *J* = 3.7 Hz, 2 × CH_p-(trifluoromethyl)phenyl_), 121.1 (2 × CH_p-nitrophenyl_), 120.8 (sept, *J* = 3.7 Hz, CH_(trifluoromethyl)phenyl_), 25.2 (C(O)-**C**H_3_); HRMS (ESI^+^): *m*/*z* [M + H]^+^ calcd for C_18_H_11_F_6_N_4_O_3_S: 477.0456; found: 477.0444.

**(*Z*)-1-(5-((4-nitrophenyl)imino)-4-(4-(trifluoromethyl)phenyl)-4,5-dihydro-1,3,4-thiadiazol-2-yl)ethan-1-one (41).**

Bright yellow solid (79% yield); mp 194 °C; IR (KBr) 1690 (C=O), 1630 (C=N), 1574, 1542 (C=S), 1506, 1424, 1334, 1276, 1254, 1192, 1165, 1144, 1108, 1070, 1013, 949, 866, 844, 771, 756, 733, 697, 664, 617, 593, 523, 496, 430 cm^−1^; ^1^H NMR (CDCl_3_, 400 MHz) δ (ppm) 8.27 (d, *J* = 8.8 Hz, 2H, Ar-H_p-nitrophenyl_), 8.16 (d, *J* = 8.4 Hz, 2H, Ar-H_p-(trifluoromethyl)phenyl_), 7.77 (d, *J* = 8.4 Hz, 2H, Ar-H_p-(trifluoromethyl)phenyl_), 7.15 (d, *J* = 8.8 Hz, 2H, Ar-H_p-nitrophenyl_), 2.65 (s, 3H, CH_3_); ^13^C NMR (CDCl_3_, 100 MHz) δ (ppm) 189.1 (C=O), 156.7 (N=C-N), 155.9 (**C**-NO_2_), 148.4 (C=N-N), 144.3 (C-N_p-nitrophenyl_), 141.1 (**C**-N_p-(trifluoromethyl)phenyl_), 129.4 (q, *J* = 32.9 Hz, Ar**C_q_**-CF_3_), 126.2 (q, *J* = 3.8 Hz, 2 × CH_p-(trifluoromethyl)phenyl_), 125.8 (2 × CH_p-nitrophenyl_), 123.6 (q, *J* = 270.8 Hz, **C**F_3_), 122.9 (2 × CH_p-(trifluoromethyl)phenyl_), 121.1 (2 × CH_p-nitrophenyl_), 25.1 (C(O)-**C**H_3_); HRMS (ESI^+^): *m*/*z* [M + H]^+^ calcd for C_17_H_12_F_3_N_4_O_3_S: 409.0582; found: 409.0591.

**(*Z*)-1-(4-(naphthalen-1-yl)-5-((4-nitrophenyl)imino)-4,5-dihydro-1,3,4-thiadiazol-2-yl)ethan-1-one (42).**

Dark brown solid (55% yield); mp 162 °C; IR (KBr) 1687 (C=O), 1618 (C=N), 1566, 1505 (C=S), 1397, 1333, 1266, 1151, 1108, 943, 853, 840, 806, 772, 697, 591 cm^−1^; ^1^H NMR (CDCl_3_, 400 MHz) δ (ppm) 8.19 (d, *J* = 8.8 Hz, 2H, Ar-H_p-nitrophenyl_), 8.06 (d, *J* = 8.4 Hz, 1H, Ar-H_naphthyl_), 8.02–7.97 (m, 1H, Ar-H_naphthyl_), 7.76–7.58 (m, 5H, Ar-H_naphthyl_), 7.06 (d, *J* = 8.8 Hz, 2H, Ar-H_p-nitrophenyl_), 2.58 (s, 3H, CH_3_); ^13^C NMR (CDCl_3_, 100 MHz) δ (ppm) 189.3 (C=O), 158.4 (N=C-N), 156.4 (**C**-NO_2_), 147.8 (C=N-N), 143.9 (C-N_p-nitrophenyl_), 134.5 (**C**-N_naphthyl_), 133.9 (**C**_naphthyl_), 130.7 (CH_naphthyl_), 129.3 (**C**_naphthyl_), 128.7 (CH_naphthyl_), 127.6 (CH_naphthyl_), 126.9 (CH_naphthyl_), 126.2 (CH_naphthyl_), 125.5 (2 × CH_p-nitrophenyl_), 125.3 (CH_naphthyl_), 122.3 (CH_naphthyl_), 121.1 (2 × CH_p-nitrophenyl_), 25.1 (C(O)-**C**H_3_); HRMS (ESI^+^): *m*/*z* [M + H]^+^ calcd for C_20_H_15_N_4_O_3_S: 391.0865; found: 391.0876.

**(*Z*)-1-(4-(2,3-difluorophenyl)-5-((4-nitrophenyl)imino)-4,5-dihydro-1,3,4-thiadiazol-2-yl)ethan-1-one (43).**

Bright yellow solid (76% yield); mp 118 °C; IR (KBr) 1685 (C=O), 1636 (C=N), 1598, 1578, 1513 (C=S), 1478, 1374, 1339, 1291, 1263, 1189, 1153, 1130, 1109, 1069, 968, 941, 858, 839, 796, 784, 753, 712, 697, 621, 574, 525, 499, 474 cm^−1^; ^1^H NMR (CDCl_3_, 400 MHz) δ (ppm) 8.23 (d, *J* = 8.4 Hz, 2H, Ar-H_p-nitrophenyl_), 7.45–7.25 (m, 3H, Ar-H_2,3-difluorophenyl_), 7.11 (d, *J* = 8.4 Hz, 2H, Ar-H_p-nitrophenyl_), 2.59 (s, 3H, CH_3_); ^13^C NMR (CDCl_3_, 100 MHz) δ (ppm) 189.1 (C=O), 156.7 (N=C-N), 155.8 (**C**-NO_2_), 151.1 (dd, *J* = 250.0, 11.0 Hz, **C**-F), 148.7 (C=N-N), 146.1 (dd, *J* = 256.0, 14.0 Hz, **C**-F), 144.1 (C-N_p-nitrophenyl_), 127.0 (dd, *J* = 9.0, 2.5 Hz, C-N_2,3-difluorophenyl_), 125.6 (2 × CH_p-nitrophenyl_), 124.1 (dd, *J* = 7.0, 5.0 Hz, Ar-CH_2,3-difluorophenyl_), 123.5 (d, *J* = 4.0 Hz, Ar-CH_2,3-difluorophenyl_), 121.1 (2 × CH_p-nitrophenyl_), 118.6 (d, *J* = 17.0 Hz, Ar-CH_2,3-difluorophenyl_), 25.1 (C(O)-**C**H_3_); HRMS (ESI^+^): *m*/*z* [M + H]^+^ calcd for C_16_H_11_F_2_N_4_O_3_S: 377.0520; found: 377.0525.

**(*Z*)-1-(4-(benzo[d]**[1,3]**dioxol-5-yl)-5-((4-nitrophenyl)imino)-4,5-dihydro-1,3,4-thiadiazol-2-yl)ethan-1-one (44).**

Light brown solid (71% yield); mp 174 °C; IR (KBr) 1686 (C=O), 1625 (C=N), 1583, 1541 (C=S), 1508, 1485, 1342, 1274, 1226, 1138, 1109, 1039, 938, 919, 860, 840, 809, 754, 714, 698, 617, 590, 551, 517, 490 cm^−1^; ^1^H NMR (CDCl_3_, 400 MHz) δ (ppm) 8.22 (d, *J* = 8.4 Hz, 2H, Ar-H_p-nitrophenyl_), 7.36–7.30 (m, 2H, Ar-H_benzo[d][1,3]dioxole_), 7.12 (d, *J* = 8.4 Hz, 2H, Ar-H_p-nitrophenyl_), 6.91 (d, *J* = 8.8 Hz, 1H, Ar-H_benzo[d][1,3]__dioxole_), 6.06 (s, 2H, OCH_2_), 2.60 (s, 3H, CH_3_); ^13^C NMR (CDCl_3_, 100 MHz) δ (ppm) 189.1 (C=O), 157.4 (N=C-N), 156.4 (**C**-NO_2_), 147.8 (C=N-N), 147.3 (C-O), 147.0 (**C**-O), 143.9 (C-N_p-nitrophenyl_), 131.9 (**C**-N_benzo[d][1,3]dioxole_), 125.7 (2 × CH_p-nitrophenyl_), 121.1 (2 × CH_p-nitrophenyl_), 117.8 (CH_benzo[d][1,3]dioxole_), 108.1 (CH_benzo[d][1,3]dioxole_), 105.6 (CH_benzo[d][1,3]dioxole_), 101.9 (CH_2_), 25.0 (C(O)-**C**H_3_); HRMS (ESI^+^): *m*/*z* [M + H]^+^ calcd for C_17_H_13_N_4_O_5_S: 385.0607; found: 385.0614.

**(*Z*)-1-(5-((4-fluorophenyl)imino)-4-(m-tolyl)-4,5-dihydro-1,3,4-thiadiazol-2-yl)ethan-1-one (45).**

Yellow solid (65% yield); mp 112–114 °C; IR (KBr) 1686 (C=O), 1628 (C=N), 1583, 1538 (C=S), 1498, 1488, 1367, 1337, 1294, 1274, 1243, 1222, 1205, 1144, 1091, 1059, 943, 903, 836, 780, 717, 675, 638, 596, 575, 505 cm^−1^; ^1^H NMR (CDCl_3_, 400 MHz) δ (ppm) 7.72 (d, *J* = 8.4 Hz, 1H, Ar-H_m-tolyl_), 7.70 (s, 1H, Ar-H_m-tolyl_), 7.38 (t, *J* = 7.6 Hz, 1H, Ar-H_m-tolyl_), 7.19 (d, *J* = 7.6 Hz, 1H, Ar-H_m-tolyl_), 7.10–7.02 (m, 2H, Ar-H_p-fluorophenyl_), 7.01–6.95 (m, 2H, Ar-H_p-fluorophenyl_), 2.60 (s, 3H, CH_3_), 2.44 (s, 3H, CH_3_, Ar-CH_3_); ^13^C NMR (CDCl_3_, 100 MHz) δ (ppm) 189.5 (C=O), 159.6 (d, *J* = 241.0 Hz, C-F), 156.7 (N=C-N), 147.2 (d, *J* = 2.0 Hz, **C**-N_p-fluorophenyl_), 146.7 (C=N-N), 139.0 (Ar**C_q_**-Me), 138.4 (C-N_m-tolyl_), 128.7 (CH_m-tolyl_), 128.5 (CH_m-tolyl_), 123.9 (CH_m-tolyl_), 121.9 (d, *J* = 8.0 Hz, 2 × CH_p-fluorophenyl_), 120.5 (CH_m-tolyl_), 116.4 (d, *J* = 22.0 Hz, 2 × CH_p-fluorophenyl_), 25.0 (C(O)-**C**H_3_), 21.5 (m-tolyl-**C**H_3_); HRMS (ESI^+^): *m*/*z* [M + H]^+^ calcd for C_17_H_15_FN_3_OS: 328.0920; found: 328.0916.

**(*Z*)-1-(5-((4-fluorophenyl)imino)-4-(4-(methylthio)phenyl)-4,5-dihydro-1,3,4-thiadiazol-2-yl)ethan-1-one (46).**

Bright yellow solid (58% yield); mp 99 °C; IR (KBr) 1692 (C=O), 1620 (C=N), 1532 (C=S), 1501, 1367, 1285, 1244, 1214, 1150, 1109, 1091, 1058, 1012, 944, 854, 814, 677, 596, 580, 502, 473 cm^−1^; ^1^H NMR (CDCl_3_, 400 MHz) δ (ppm) 7.86 (d, *J* = 8.4 Hz, 2H, Ar-H_p-methylthio_), 7.35 (d, *J* = 8.4 Hz, 2H, Ar-H_p-methylthio_), 7.09–7.02 (m, 2H, Ar-H_p-fluorophenyl_), 7.01–6.95 (m, 2H, Ar-H_p-fluorophenyl_), 2.60 (s, 3H, CH_3_), 2.52 (s, 3H, CH_3_, Ar-SCH_3_); ^13^C NMR (CDCl_3_, 100 MHz) δ (ppm) 189.4 (C=O), 159.7 (d, *J* = 242.0 Hz, C-F), 156.5 (N=C-N), 147.0 (d, *J* = 2.0 Hz, **C**-N_p-fluorophenyl_), 146.9 (C=N-N), 138.2 (Ar**C_q_**-SMe), 135.7 (C-N_p-methylthio_), 126.6 (2 × CH_p-methylthio_), 123.5 (2 × CH_p-methylthio_), 121.9 (d, *J* = 8.0 Hz, 2 × CH_p-fluorophenyl_), 116.4 (d, *J* = 22.0 Hz, 2 × CH_p-fluorophenyl_), 25.0 (C(O)-**C**H_3_), 15.8 (S-**C**H_3_); HRMS (ESI^+^): *m*/*z* [M + H]^+^ calcd for C_17_H_15_FN_3_OS_2_: 360.0641; found: 360.0635.

**(*Z*)-1-(5-((4-fluorophenyl)imino)-4-(4-methoxyphenyl)-4,5-dihydro-1,3,4-thiadiazol-2-yl)ethan-1-one (47).**

Bright yellow solid (59% yield); mp 154 °C; IR (KBr) 1692 (C=O), 1620 (C=N), 1590, 1533 (C=S), 1501, 1368, 1282, 1253, 1215, 1150, 1032, 944, 823, 726, 677, 598, 559 cm^−1^; ^1^H NMR (CDCl_3_, 400 MHz) δ (ppm) 7.77 (d, *J* = 8.8 Hz, 2H, Ar-H_p-methoxyphenyl_), 7.09–6.95 (m, 6H, 2xAr-H_p-methoxyphenyl_ and 4xAr-H_p-fluorophenyl_), 3.86 (s, 3H, OCH_3_), 2.59 (s, 3H, CH_3_);^13^C NMR (CDCl_3_, 100 MHz) δ (ppm) 189.4 (C=O), 158.9 (C-O), 159.6 (d, *J* = 242.0 Hz, C-F), 157.1 (N=C-N), 147.1 (d, *J* = 2.0 Hz, **C**-N_p-fluorophenyl_), 146.5 (C=N-N), 131.5 (C-N_p-methoxyphenyl_), 125.1 (2 × CH_p-methoxyphenyl_), 122.0 (d, *J* = 8.0 Hz, 2 × CH_p-fluorophenyl_), 116.4 (d, *J* = 23.0 Hz, 2 × CH_p-fluorophenyl_), 114.1 (2 × CH_p-methoxyphenyl_), 55.5 (O-CH_3_), 25.0 (C(O)-**C**H_3_; HRMS (ESI^+^): *m*/*z* [M + H]^+^ calcd for C_17_H_15_FN_3_O_2_S: 344.0869; found: 344.0877.

**(*Z*)-1-(4-(4-chlorophenyl)-5-((4-fluorophenyl)imino)-4,5-dihydro-1,3,4-thiadiazol-2-yl)ethan-1-one (48)**. 

Bright yellow solid (51% yield); mp 116 °C; IR (KBr) 1681 (C=O), 1636 (C=N), 1586, 1531 (C=S), 1504, 1486, 1363, 1283, 1273, 1247, 1228, 1214, 1147, 1092, 1058, 1011, 948, 849, 826, 795, 674, 593, 503, 476 cm^−1^; ^1^H NMR (CDCl_3_, 400 MHz) δ (ppm) 7.95 (d, *J* = 8.8 Hz, 2H, Ar-H_p-chlorophenyl_), 7.46 (d, *J* = 8.8 Hz, 2H, Ar-H_p-chlorophenyl_), 7.10–7.02 (m, 2H, Ar-H_p-fluorophenyl_), 7.01–6.94 (m, 2H, Ar-H_p-fluorophenyl_), 2.61 (s, 3H, CH_3_); ^13^C NMR (CDCl_3_, 100 MHz) δ (ppm) 189.3 (C=O), 159.7 (d, *J* = 242.0 Hz, C-F), 156.1 (N=C-N), 147.3 (C=N-N), 146.9 (d, *J* = 2.0 Hz, **C**-N_p-fluorophenyl_), 137.2 (C-N_p-chlorophenyl_), 132.8 (C-Cl), 129.0 (2 × CH_p-chlorophenyl_), 124.1 (2 × CH_p-chlorophenyl_), 121.8 (d, *J* = 8.0 Hz, 2 × CH_p-fluorophenyl_), 116.5 (d, *J* = 23.0 Hz, 2 × CH_p-fluorophenyl_), 25.0 (C(O)-**C**H_3_); HRMS (ESI^+^): *m*/*z* [M + H]^+^ calcd for C_16_H_12_FClN_3_OS: 348.0374; found: 348.0385.

**(*Z*)-1-(4-(4-bromophenyl)-5-((4-fluorophenyl)imino)-4,5-dihydro-1,3,4-thiadiazol-2-yl)ethan-1-one (49).**

Bright yellow solid (57% yield); mp 122–124 °C; IR (KBr) 1681 (C=O), 1635 (C=N), 1581, 1530 (C=S), 1483, 1364, 1282, 1247, 1228, 1215, 1147, 1091, 1071, 1058, 1008, 947, 848, 822, 794, 737, 673, 641, 593, 577, 526, 508, 420 cm^−1^; ^1^H NMR (CDCl_3_, 400 MHz) δ (ppm) 7.90 (d, *J* = 8.8 Hz, 2H, Ar-H_p-bromophenyl_), 7.60 (d, *J* = 8.8 Hz, 2H, Ar-H_p-bromophenyl_), 7.10–7.02 (m, 2H, Ar-H_p-fluorophenyl_), 7.01–6.94 (m, 2H, Ar-H_p-fluorophenyl_), 2.60 (s, 3H, CH_3_); ^13^C NMR (CDCl_3_, 100 MHz) δ (ppm) 189.3 (C=O), 159.8 (d, *J* = 242.0 Hz, C-F), 156.1 (N=C-N), 147.4 (C=N-N), 146.8 (d, *J* = 2.0 Hz, **C**-N_p-fluorophenyl_), 137.7 (C-N_p-bromophenyl_), 132.0 (2 × CH_p-bromophenyl_), 124.3 (2 × CH_p-bromophenyl_), 121.8 (d, *J* = 8.0 Hz, 2 × CH_p-fluorophenyl_), 120.7 (C-Br), 116.5 (d, *J* = 23.0 Hz, 2 × CH_p-fluorophenyl_), 25.0 (C(O)-**C**H_3_); HRMS (ESI^+^): *m*/*z* [M + H]^+^ calcd for C_16_H_12_FBrN_3_OS: 391.9868; found: 391.9879.

**(*Z*)-1-(5-((4-fluorophenyl)imino)-4-(4-iodophenyl)-4,5-dihydro-1,3,4-thiadiazol-2-yl)ethan-1-one (50).**

Light brown solid (74% yield); mp 115 °C; IR (KBr) 1687 (C=O), 1619 (C=N), 1600, 1579, 1532 (C=S), 1500, 1482, 1362, 1336, 1279, 1243, 1217, 1142, 1093, 1055, 1006, 944, 852, 819, 735, 678, 640, 596, 577, 508 cm^−1^; ^1^H NMR (CDCl_3_, 400 MHz) δ (ppm) 7.81 (d, *J* = 8.8 Hz, 2H, Ar-H_p-iodophenyl_), 7.76 (d, *J* = 8.8 Hz, 2H, Ar-H_p-iodophenyl_), 7.10–7.03 (m, 2H, Ar-H_p-fluorophenyl_), 7.01–6.94 (m, 2H, Ar-H_p-fluorophenyl_), 2.60 (s, 3H, CH_3_); ^13^C NMR (CDCl_3_, 100 MHz) δ (ppm) 189.4 (C=O), 159.7 (d, *J* = 242.0 Hz, C-F), 156.0 (N=C-N), 147.4 (C=N-N), 146.9 (d, *J* = 2.0 Hz, **C**-N_p-fluorophenyl_), 138.5 (C-N_p-iodophenyl_), 137.9 (2 × CH_p-iodophenyl_), 124.5 (2 × CH_p-iodophenyl_), 121.8 (d, *J* = 8.0 Hz, 2 × CH_p-fluorophenyl_), 116.5 (d, *J* = 23.0 Hz, 2 × CH_p-fluorophenyl_), 92.0 (C-I), 25.0 (C(O)-**C**H_3_); HRMS (ESI^+^): *m*/*z* [M + H]^+^ calcd for C_16_H_12_FIN_3_OS: 439.9730; found: 439.9721.

**(*Z*)-1-(5-((4-fluorophenyl)imino)-4-phenyl-4,5-dihydro-1,3,4-thiadiazol-2-yl)ethan-1-one (51).**

Bright yellow solid (61% yield); mp 118–121 °C; IR (KBr) 1687 (C=O), 1633 (C=N), 1588, 1529 (C=S), 1502, 1368, 1338, 1267, 1227, 1150, 1092, 1057, 943, 870, 846, 829, 763, 727, 691, 671, 640, 593, 570, 515, 505 cm^−1^; ^1^H NMR (CDCl_3_, 400 MHz) δ (ppm) 7.95 (d, *J* = 8.0 Hz, 2H, Ar-H_phenyl_), 7.51 (t, *J* = 7.6 Hz, 2H, Ar-H_p-phenyl_), 7.38 (t, *J* = 7.6 Hz, 1H, Ar-H_phenyl_), 7.09–7.02 (m, 2H, Ar-H_p-fluorophenyl_), 7.01–6.97 (m, 2H, Ar-H_p-fluorophenyl_), 2.61 (s, 3H, CH_3_); ^13^C NMR (CDCl_3_, 100 MHz) δ (ppm) 189.5 (C=O), 159.7 (d, *J* = 241.0 Hz, C-F), 156.7 (N=C-N), 147.1 (C=N-N), 147.0 (d, *J* = 2.0 Hz, **C**-N_p-fluorophenyl_), 138.6 (C-N_phenyl_), 129.0 (2 × CH_phenyl_), 127.6 (CH_phenyl_), 123.2 (2 × CH_phenyl_), 121.9 (d, *J* = 8.0 Hz, 2 × CH_p-fluorophenyl_), 116.4 (d, *J* = 22.0 Hz, 2 × CH_p-fluorophenyl_), 25.0 (C(O)-**C**H_3_); HRMS (ESI^+^): *m*/*z* [M + H]^+^ calcd for C_16_H_13_FN_3_OS: 314.0763; found: 314.0770.

**(*Z*)-1-(4-(3,5-bis(trifluoromethyl)phenyl)-5-((4-fluorophenyl)imino)-4,5-dihydro-1,3,4-thiadiazol-2-yl)ethan-1-one (52).**

Bright yellow solid (57% yield); mp 140–141 °C; IR (KBr) 1692 (C=O), 1632 (C=N), 1545 (C=S), 1505, 1466, 1390, 1366, 1317, 1283, 1239, 1174, 1133, 896, 839, 770, 726, 701, 685, 594, 511, 421 cm^−1^; ^1^H NMR (CDCl_3_, 400 MHz) δ (ppm) 8.65 (s, 2H, Ar-H_(trifluoromethyl)phenyl_), 7.84 (s, 1H, Ar-H_(trifluoromethyl)phenyl_), 7.13–7.05 (m, 2H, Ar-H_p-fluorophenyl_), 7.04–6.98 (m, 2H, Ar-H_p-fluorophenyl_), 2.67 (s, 3H, CH_3_, COCH_3_); ^13^C NMR (CDCl_3_, 100 MHz) δ (ppm) 189.2 (C=O), 160.0 (d, *J* = 243.0 Hz, C-F), 155.1 (d, *J* = 2.0 Hz, N=C-N), 148.7 (C=N-N), 146.1 (d, *J* = 3.0 Hz, **C**-N_p-fluorophenyl_), 140.1 (**C**-N_(trifluoromethyl)phenyl_), 132.3 (q, *J* = 34.0 Hz, Ar**C_q_**-CF_3_), 122.9 (q, *J* = 271.0 Hz, **C**F_3_), 122.0 (q, *J* = 4.0 Hz, 2 × CH_p-(trifluoromethyl)phenyl_), 121.8 (d, *J* = 8.0 Hz, 2 × CH_p-fluorophenyl_), 120.1 (sept, *J* = 4.0 Hz, CH_(trifluoromethyl)phenyl_), 116.6 (d, *J* = 23.0 Hz, 2 × CH_p-fluorophenyl_), 25.2 (C(O)-**C**H_3_); HRMS (ESI^+^): *m*/*z* [M + H]^+^ calcd for C_18_H_11_F_7_N_3_OS: 450.0511; found: 450.0500.

**(*Z*)-1-(5-((4-fluorophenyl)imino)-4-(3-(trifluoromethyl)phenyl)-4,5-dihydro-1,3,4-thiadiazol-2-yl)ethan-1-one (53).**

Off white solid (57% yield); mp 114–115 °C; IR (KBr) 1694 (C=O), 1623 (C=N), 1589, 1548 (C=S), 1497, 1457, 1347, 1321, 1293, 1216, 1169, 1128, 1073, 839, 798, 730, 697, 678, 574 cm^−1^; ^1^H NMR (CDCl_3_, 400 MHz) δ (ppm) 8.32–8.26 (m, 2H, Ar-H_p-(trifluoromethyl)phenyl_), 7.64–7.58 (m, 2H, Ar-H_m-(trifluoromethyl)phenyl_), 7.12–7.03 (m, 2H, Ar-H_p-fluorophenyl_), 7.02–6.95 (m, 2H, Ar-H_p-fluorophenyl_), 2.63 (s, 3H, CH_3_); ^13^C NMR (CDCl_3_, 100 MHz) δ (ppm) 189.4 (C=O), 159.8 (d, *J* = 242.0 Hz, C-F), 155.8 (N=C-N), 147.8 (C=N-N), 146.7 (d, *J* = 2.0 Hz, **C**-N_p-fluorophenyl_), 139.2 (**C**-N_p-(trifluoromethyl)phenyl_), 131.4 (q, *J* = 33.0 Hz, Ar**C_q_**-CF_3_), 129.5 (s, CH_m-(trifluoromethyl)phenyl_), 125.7 (q, *J* = 1.0 Hz, CH_p-(trifluoromethyl)phenyl_), 123.7 (q, *J* = 4.0 Hz, CH_m-(trifluoromethyl)phenyl_), 123.6 (q, *J* = 271.0 Hz, **C**F_3_), 121.8 (d, *J* = 8.0 Hz, 2 × CH_p-fluorophenyl_), 119.5 (q, *J* = 4.0 Hz, CH_p-(trifluoromethyl)phenyl_), 116.5 (d, *J* = 22.0 Hz, 2 × CH_p-fluorophenyl_), 25.1 (C(O)-**C**H_3_); HRMS (ESI^+^): *m*/*z* [M + H]^+^ calcd for C_17_H_12_F_4_N_3_OS: 382.0637; found: 382.0624.

**(*Z*)-1-(5-((4-fluorophenyl)imino)-4-(*p*-tolyl)-4,5-dihydro-1,3,4-thiadiazol-2-yl)ethan-1-one (54).**

Bright yellow solid (59% yield); mp 142–144 °C; IR (KBr) 1688 (C=O), 1620 (C=N), 1532 (C=S), 1501, 1367, 1339, 1280, 1247, 1214, 1145, 1094, 1059, 945, 856, 828, 811, 723, 676, 634, 598, 553, 524, 505 cm^−1^; ^1^H NMR (CDCl_3_, 400 MHz) δ (ppm) 7.77 (d, *J* = 8.4 Hz, 2H, Ar-H_p-tolyl_), 7.30 (d, *J* = 8.4 Hz, 2H, Ar-H_p-tolyl_), 7.09–7.02 (m, 2H, Ar-H_p-fluorophenyl_), 7.01–6.95 (m, 2H, Ar-H_p-flurorophenyl_), 2.59 (s, 3H, CH_3_), 2.41 (s, 3H, CH_3_, Ar-CH_3_); ^13^C NMR (CDCl_3_, 100 MHz) δ (ppm) 189.5 (C=O), 159.6 (d, *J* = 242.0 Hz, C-F), 156.8 (N=C-N), 147.2 (d, *J* = 2.0 Hz, **C**-N_p-fluorophenyl_), 146.6 (C=N-N), 137.7 (Ar**C_q_**-Me), 136.0 (C-N_p-tolyl_), 129.5 (2 × CH_p-tolyl_), 123.3 (2 × CH_p-tolyl_), 121.9 (d, *J* = 8.0 Hz, 2 × CH_p-fluorophenyl_), 116.4 (d, *J* = 23.0 Hz, 2 × CH_p-fluorophenyl_), 25.0 (C(O)-**C**H_3_), 21.1 (*p*-tolyl-**C**H_3_); HRMS (ESI^+^): *m*/*z* [M + H]^+^ calcd for C_17_H_15_FN_3_OS: 328.0920; found: 328.0929.

**(*Z*)-1-(5-((3-fluorophenyl)imino)-4-(*p*-tolyl)-4,5-dihydro-1,3,4-thiadiazol-2-yl)ethan-1-one (55).**

Yellow oil (71% yield); IR (KBr) IR (KBr) 1687 (C=O), 1636 (C=N), 1602, 1578 (C=S), 1529, 1506, 1480, 1368, 1281, 1259, 1175, 1155, 1126, 1097, 1059, 961, 944, 857, 813, 771, 690, 599, 557, 515, 496, 448 cm^−1^; ^1^H NMR (CDCl_3_, 400 MHz) δ (ppm) 7.77 (d, *J* = 8.4 Hz, 2H, Ar-H_p-tolyl_), 7.34–7.27 (m, 3H, 2xAr-H_p-tolyl_ and 1xAr-H_m-fluorophenyl_), 6.86–6.78 (m, 2H, Ar-H_m-fluorophenyl_), 6.77–6.72 (m, 1H, Ar-H_m-fluorophenyl_), 2.60 (s, 3H, CH_3_), 2.41 (s, 3H, CH_3_, Ar-CH_3_); ^13^C NMR (CDCl_3_, 100 MHz) δ (ppm) 189.5 (C=O), 163.6 (d, *J* = 245.0 Hz, C-F), 157.0 (N=C-N), 152.6 (d, *J* = 9.0 Hz, **C**-N_m-fluorophenyl_), 147.0 (C=N-N), 137.9 (Ar**C_q_**-Me), 136.0 (C-N_p-tolyl_), 130.9 (d, *J* = 9.0 Hz, CH_m-fluorophenyl_), 129.6 (2 × CH_p-tolyl_), 123.4 (2 × CH_p-tolyl_), 116.1 (d, *J* = 3.0 Hz, CH_m-fluorophenyl_), 111.3 (d, *J* = 22.0 Hz, CH_m-fluorophenyl_), 108.2 (d, *J* = 22.0 Hz, CH_m-fluorophenyl_), 25.0 (C(O)-**C**H_3_), 21.2 (*p*-tolyl-**C**H_3_); HRMS (ESI^+^): *m*/*z* [M + H]^+^ calcd for C_17_H_15_FN_3_OS: 328.0920; found: 328.0909.

**(*Z*)-1-(4-(4-chlorophenyl)-5-((3-fluorophenyl)imino)-4,5-dihydro-1,3,4-thiadiazol-2-yl)ethan-1-one (56).**

Bright yellow solid (78% yield); mp 153–155 °C; IR (KBr) 1682 (C=O), 1643 (C=N), 1606, 1582 (C=S), 1526, 1487, 1443, 1361, 1307, 1288, 1260, 1176, 1156, 1129, 1092, 1056, 1012, 962, 880, 826, 771, 693, 589, 517, 495, 477, 442 cm^−1^; ^1^H NMR (CDCl_3_, 400 MHz) δ (ppm) 7.94 (d, *J* = 8.8 Hz, 2H, Ar-H_p-chlorophenyl_), 7.46 (d, *J* = 8.8 Hz, 2H, Ar-H_p-chlorophenyl_), 7.37–7.28 (m, 1H, Ar-H_m-fluorophenyl_), 6.88–6.79 (m, 2H, Ar-H_m-fluorophenyl_), 6.76–6.71 (m, 1H, Ar-H_m-fluorophenyl_), 2.61 (s, 3H, CH_3_); ^13^C NMR (CDCl_3_, 100 MHz) δ (ppm) 189.3 (C=O), 163.5 (d, *J* = 246.0 Hz, C-F), 156.3 (N=C-N), 152.3 (d, *J* = 10.0 Hz, **C**-N_m-fluorophenyl_), 147.5 (C=N-N), 137.1 (C-N_p-chlorophenyl_), 132.8 (C-Cl), 131.0 (d, *J* = 10.0 Hz, CH_m-fluorophenyl_), 129.0 (2 × CH_p-chlorophenyl_), 124.1 (2 × CH_p-chlorophenyl_), 115.9 (d, *J* = 3.0 Hz, CH_m-fluorophenyl_), 111.4 (d, *J* = 21.0 Hz, CH_m-fluorophenyl_), 108.0 (d, *J* = 20.0 Hz, CH_m-fluorophenyl_), 25.0 (C(O)-**C**H_3_); HRMS (ESI^+^): *m*/*z* [M + H]^+^ calcd for C_16_H_12_FClN_3_OS: 348.0374; found: 348.0362.

**(*Z*)-1-(4-(4-bromophenyl)-5-((3-fluorophenyl)imino)-4,5-dihydro-1,3,4-thiadiazol-2-yl)ethan-1-one (57).**

Bright yellow solid (71% yield); mp 129–130 °C; IR (KBr) 1688 (C=O), 1650 (C=N), 1605, 1578, 1533 (C=S), 1478, 1361, 1339, 1280, 1260, 1168, 1153, 1125, 1099, 1053, 1004, 956, 861, 827, 785, 773, 688, 584, 517, 500, 422 cm^−1^; ^1^H NMR (CDCl_3_, 400 MHz) δ (ppm) 7.89 (d, *J* = 8.8 Hz, 2H, Ar-H_p-bromophenyl_), 7.61 (d, *J* = 8.8 Hz, 2H, Ar-H_p-bromophenyl_), 7.37–7.28 (m, 1H, Ar-H_m-fluorophenyl_), 6.88–6.78 (m, 2H, Ar-H_m-fluorophenyl_), 6.77–6.71 (m, 1H, Ar-H_m-fluorophenyl_), 2.61 (s, 3H, CH_3_); ^13^C NMR (CDCl_3_, 100 MHz) δ (ppm) 189.3 (C=O), 163.6 (d, *J* = 246.0 Hz, C-F), 156.2 (N=C-N), 152.3 (d, *J* = 9.0 Hz, **C**-N_m-fluorophenyl_), 147.5 (C=N-N), 137.6 (C-N_p-bromophenyl_), 132.0 (2 × CH_p-bromophenyl_), 131.0 (d, *J* = 9.0 Hz, CH_m-fluorophenyl_), 124.3 (2 × CH_p-bromophenyl_), 120.8 (C-Br), 115.9 (d, *J* = 3.0 Hz, CH_m-fluorophenyl_), 111.5 (d, *J* = 21.0 Hz, CH_m-fluorophenyl_), 108.0 (d, *J* = 20.0 Hz, CH_m-fluorophenyl_), 25.0 (C(O)-**C**H_3_); HRMS (ESI^+^): *m*/*z* [M + H]^+^ calcd for C_16_H_12_FBrN_3_OS: 391.9868; found: 391.9875.

**(*Z*)-1-(5-((3-fluorophenyl)imino)-4-(4-iodophenyl)-4,5-dihydro-1,3,4-thiadiazol-2-yl)ethan-1-one (58).**

Light brown solid (69% yield); mp 145–147 °C; IR (KBr) 1691 (C=O), 1640 (C=N), 1603, 1577, 1534 (C=S), 1477, 1360, 1337, 1277, 1262, 1170, 1151, 1126, 1098, 1054, 999, 957, 871, 832, 817, 778, 700, 584, 517, 450 cm^−1^; ^1^H NMR (CDCl_3_, 400 MHz) (ppm) 7.81 (d, *J* = 8.8 Hz, 2H, Ar-H_p-iodophenyl_), 7.76 (d, *J* = 8.8 Hz, 2H, Ar-H_p-iodophenyl_), 7.32 (dd, *J* = 14.7, 8.0 Hz, 1H, Ar-H_m-fluorophenyl_), 6.84 td, J = 8.4, 2.0 Hz, 1H, Ar-H_m-fluorophenyl_), 6.81 (d, J = 8.2 Hz, 1H, Ar-H_m-fluorophenyl_), 6.73 (dt, J = 10.0, 1.6 Hz, 1H, Ar-H_m-fluorophenyl_), 2.61 (s, 3H, CH_3_); ^13^C NMR (CDCl_3_, 100 MHz) δ (ppm) 189.3 (C=O), 163.6 (d, *J* = 246.0 Hz, C-F), 156.2 (N=C-N), 152.3 (d, *J* = 9.0 Hz, **C**-N_m-fluorophenyl_), 147.6 (C=N-N), 138.4 (C-N_p-iodophenyl_), 137.9 (2 × CH_p-iodophenyl_), 131.0 (d, *J* = 10.0 Hz, CH_m-fluorophenyl_), 124.5 (2 × CH_p-iodophenyl_), 115.9 (d, *J* = 3.0 Hz, CH_m-fluorophenyl_), 111.5 (d, *J* = 21.0 Hz, CH_m-fluorophenyl_), 108.0 (d, *J* = 23.0 Hz, CH_m-fluorophenyl_), 92.1 (C-I), 25.0 (C(O)-**C**H_3_); HRMS (ESI^+^): *m*/*z* [M + H]^+^ calcd for C_16_H_12_FIN_3_OS: 439.9730; found: 439.9743.

**(*Z*)-1-(5-((2-fluorophenyl)imino)-4-(p-tolyl)-4,5-dihydro-1,3,4-thiadiazol-2-yl)ethan-1-one (59).**

Bright yellow solid (72% yield); mp 83 °C; IR (KBr) 1684 (C=O), 1635 (C=N), 1522 (C=S), 1492, 1206, 1145, 1101, 1059, 943, 817, 790, 746, 685, 599, 547, 516 cm^−1^; ^1^H NMR (CDCl_3_, 400 MHz) δ (ppm) 7.81 (d, *J* = 8.4 Hz, 2H, Ar-H_p-tolyl_), 7.30 (d, *J* = 8.4 Hz, 2H, Ar-H_p-tolyl_), 7.16–7.01 (m, 4H, Ar-H_o-fluorophenyl_), 2.60 (s, 3H, CH_3_), 2.41 (s, 3H, CH_3_, Ar-CH_3_); ^13^C NMR (CDCl_3_, 100 MHz) δ (ppm) 189.4 (C=O), 158.3 (N=C-N), 153.9 (d, *J* = 245.0 Hz, C-F), 147.4 (C=N-N), 138.8 (d, *J* = 12.0 Hz, **C**-N_o-fluorophenyl_), 137.8 (Ar**C_q_**-Me), 136.0 (C-N_p-tolyl_), 129.5 (2 × CH_p-tolyl_), 125.5 (d, *J* = 7.0 Hz, CH_o-fluorophenyl_), 124.9 (d, *J* = 4.0 Hz, CH_m-fluorophenyl_), 123.3 (2 × CH_p-tolyl_), 122.1 (CH_o-fluorophenyl_), 116.5 (d, *J* = 19.0 Hz, CH_o-fluorophenyl_), 25.0 (C(O)-**C**H_3_), 21.1 (*p*-tolyl-**C**H_3_); HRMS (ESI^+^): *m*/*z* [M + H]^+^ calcd for C_17_H_15_FN_3_OS: 328.0920; found: 328.0906.

**(*Z*)-1-(4-(4-chlorophenyl)-5-((2-fluorophenyl)imino)-4,5-dihydro-1,3,4-thiadiazol-2-yl)ethan-1-one (60).**

Bright yellow solid (70% yield); mp 108 °C; IR (KBr) 1689 (C=O), 1632 (C=N), 1533 (C=S), 1486, 1364, 1338, 1281, 1254, 1205, 1143, 1092, 1056, 1008, 944, 835, 759, 696, 590, 477 cm^−1^; ^1^H NMR (CDCl_3_, 400 MHz) δ (ppm) 7.99 (d, *J* = 8.8 Hz, 2H, Ar-H_p-chlorophenyl_), 7.46 (d, *J* = 8.8 Hz, 2H, Ar-H_p-chlorophenyl_), 7.18–7.08 (m, 3H, Ar-H_o-fluorophenyl_), 7.07–7.00 (m, 1H, Ar-H_o-fluorophenyl_), 2.61 (s, 3H, CH_3_); ^13^C NMR (CDCl_3_, 100 MHz) δ (ppm) 189.2 (C=O), 157.6 (N=C-N), 153.8 (d, *J* = 246.0 Hz, C-F), 147.9 (C=N-N), 138.4 (d, *J* = 12.0 Hz, **C**-N_o-fluorophenyl_), 137.1 (C-N_p-chlorophenyl_), 132.8 (C-Cl), 129.0 (2 × CH_p-chlorophenyl_), 125.6 (d, *J* = 7.0 Hz, CH_o-fluorophenyl_), 124.9 (d, *J* = 4.0 Hz, CH_m-fluorophenyl_), 124.1 (2 × CH_p-chlorophenyl_), 121.8 (d, *J* = 4.0 Hz, CH_o-fluorophenyl_), 116.6 (d, *J* = 20.0 Hz, CH_o-fluorophenyl_), 25.0 (C(O)-**C**H_3_); HRMS (ESI^+^): *m*/*z* [M + H]^+^ calcd for C_16_H_12_FClN_3_OS: 348.0374; found: 348.0380.

**(*Z*)-1-(4-(4-bromophenyl)-5-((2-fluorophenyl)imino)-4,5-dihydro-1,3,4-thiadiazol-2-yl)ethan-1-one (61)**. 

Light green solid (77% yield); mp 153–154 °C; IR (KBr) 1686 (C=O), 1641 (C=N), 1604, 1580, 1525 (C=S), 1481, 1361, 1340, 1282, 1260, 1175, 1155, 1128, 1094, 1055, 1008, 961, 879, 823, 784, 771, 689, 638, 586, 516, 492 cm^−1^; ^1^H NMR (CDCl_3_, 400 MHz) δ (ppm) 7.93 (d, *J* = 8.8 Hz, 2H, Ar-H_p-bromophenyl_), 7.61 (d, *J* = 8.8 Hz, 2H, Ar-H_p-bromophenyl_), 7.18–7.08 (m, 3H, Ar-H_o-fluorophenyl_), 7.07–7.00 (m, 1H, Ar-H_o-fluorophenyl_), 2.61 (s, 3H, CH_3_); ^13^C NMR (CDCl_3_, 100 MHz) δ (ppm) 189.2 (C=O), 157.5 (N=C-N), 153.8 (d, *J* = 246.0 Hz, C-F), 148.0 (C=N-N), 138.3 (d, *J* = 12.0 Hz, **C**-N_o-fluorophenyl_), 137.6 (C-N_p-bromophenyl_), 132.0 (2 × CH_p-bromophenyl_), 125.7 (d, *J* = 8.0 Hz, CH_o-fluorophenyl_), 124.9 (d, *J* = 4.0 Hz, CH_m-fluorophenyl_), 124.3 (2 × CH_p-bromophenyl_), 121.8 (d, *J* = 4.0 Hz, CH_o-fluorophenyl_), 120.8 (C-Br), 116.6 (d, *J* = 19.0 Hz, CH_o-fluorophenyl_), 25.0 (C(O)-**C**H_3_); HRMS (ESI^+^): *m*/*z* [M + H]^+^ calcd for C_16_H_12_FBrN_3_OS: 391.9868; found: 391.9875.

**(*Z*)-1-(4-(4-iodophenyl)-5-((2-fluorophenyl)imino)-4,5-dihydro-1,3,4-thiadiazol-2-yl)ethan-1-one (62).**

Light brown solid (79% yield); mp 143 °C; IR (KBr) 1687 (C=O), 1633 (C=N), 1577, 1532 (C=S), 1481, 1361, 1336, 1281, 1205, 1141, 1098, 1055, 1000, 933, 832, 813, 790, 759, 690, 587, 516, 450 cm^−1^; ^1^H NMR (CDCl_3_, 400 MHz) δ (ppm) 7.81 (broad singlet of coalesced quartet, 4H, Ar-H_p-iodophenyl_), 7.18–7.08 (m, 3H, Ar-H_o-fluorophenyl_), 7.07–7.00 (m, 1H, Ar-H_o-fluorophenyl_), 2.60 (s, 3H, CH_3_); ^13^C NMR (CDCl_3_, 100 MHz) δ (ppm) 189.2 (C=O), 157.4 (N=C-N), 153.8 (d, *J* = 246.0 Hz, C-F), 148.0 (C=N-N), 138.4 (d, *J* = 12.0 Hz, **C**-N_o-fluorophenyl_), 138.3 (C-N_p-iodophenyl_), 137.9 (2 × CH_p-iodophenyl_), 125.6 (d, *J* = 8.0 Hz, CH_o-fluorophenyl_), 124.9 (d, *J* = 3.0 Hz, CH_m-fluorophenyl_), 124.5 (2 × CH_p-iodophenyl_), 121.7 (CH_o-fluorophenyl_), 116.6 (d, *J* = 20.0 Hz, CH_o-fluorophenyl_), 92.1 (C-I), 25.0 (C(O)-**C**H_3_); HRMS (ESI^+^): *m*/*z* [M + H]^+^ calcd for C_16_H_12_FClN_3_OS: 348.0374; found: 348.0380.

**(*Z*)-1-(5-((4-chlorophenyl)imino)-4-(*p*-tolyl)-4,5-dihydro-1,3,4-thiadiazol-2-yl)ethan-1-one (63).**

Bright yellow solid (80% yield); mp 171–173 °C; IR (KBr) 1688 (C=O), 1618 (C=N), 1584, 1536 (C=S), 1504, 1484, 1368, 1337, 1292, 1247, 1144, 1099, 1059, 1010, 947, 853, 813, 718, 640, 625, 597, 548, 523, 499 cm^−1^; ^1^H NMR (CDCl_3_, 400 MHz) δ (ppm) 7.76 (d, *J* = 8.0 Hz, 2H, Ar-H_p-tolyl_), 7.31 (d, *J* = 8.8 Hz, 2H, Ar-H_p-chlorophenyl_), 7.30 (d, *J* = 8.0 Hz, 2H, Ar-H_p-tolyl_), 6.96 (d, *J* = 8.8 Hz, 2H, Ar-H_p-chlorophenyl_), 2.59 (s, 3H, CH_3_, COCH_3_), 2.41 (s, 3H, CH_3_, Ar-CH_3_); ^13^C NMR (CDCl_3_, 100 MHz) δ (ppm) 189.4 (C=O), 156.7 (N=C-N), 149.5 (**C**-N_p-chlorophenyl_), 146.7 (C=N-N), 137.8 (Ar**C_q_**-Me), 136.0 (C-N_p-tolyl_), 129.7 (2 × CH_p-tolyl_), 129.5 (2 × CH_p-chlorophenyl_), 129.4 (C-Cl), 123.3 (2 × CH_p-tolyl_), 121.9 (2 × CH_p-chlorophenyl_), 25.0 (C(O)-**C**H_3_), 21.1 (p-tolyl-**C**H_3_); HRMS (ESI^+^): *m*/*z* [M + H]^+^ calcd for C_17_H_15_ClN_3_OS: 344.0624; found: 344.0633.

**(*Z*)-1-(5-((4-chlorophenyl)imino)-4-(2,3-difluorophenyl)-4,5-dihydro-1,3,4-thiadiazol-2-yl)ethan-1-one (64).**

Light brown solid (43% yield); mp 87–89 °C; IR (KBr) 1681 (C=O), 1607 (C=N), 1580, 1521 (C=S), 1504, 1481, 1370, 1297, 1271, 1254, 1184, 1152, 1134, 1095, 1061, 1006, 969, 937, 841, 822, 797, 780, 733, 710, 652, 626, 599, 576, 565, 519, 505, 480 cm^−1^; ^1^H NMR (CDCl_3_, 400 MHz) δ (ppm) 7.44–7.39 (m, 1H, Ar-H_2,3-difluorophenyl_), 7.36–7.22 (m, 2H, Ar-H_2,3-difluorophenyl_), 7.30 (d, *J* = 8.8 Hz, 2H, Ar-H_p-chlorophenyl_), 6.94 (d, *J* = 8.8 Hz, 2H, Ar-H_p-chlorophenyl_), 2.57 (s, 3H, CH_3_); ^13^C NMR (CDCl_3_, 100 MHz) δ (ppm) 189.3 (C=O), 155.8 (N=C-N), 151.1 (dd, *J* = 250.0, 11.0 Hz, **C**-F), 148.8 (C-N_p-chlorophenyl_), 148.3 (C=N-N), 146.1 (dd, *J* = 256.0, 14.0 Hz, **C**-F), 129.8 (C-Cl), 129.7 (2 × CH_p-chlorophenyl_), 127.3 (dd, *J* = 9.0, 2.0 Hz, C-N_2,3-difluorophenyl_), 124.0 (dd, *J* = 7.0, 5.0 Hz, Ar-CH_2,3-difluorophenyl_), 123.4 (d, *J* = 4.0 Hz, Ar-CH_2,3-difluorophenyl_), 121.9 (2 × CH_p-chlorophenyl_), 118.2 (d, *J* = 17.0 Hz, Ar-CH_2,3-difluorophenyl_), 25.0 (C(O)-**C**H_3_); HRMS (ESI^+^): *m*/*z* [M + H]^+^ calcd for C_16_H_11_ClF_2_N_3_OS: 366.0279; found: 366.0291.

**(*Z*)-1-(4-(3,5-bis(trifluoromethyl)phenyl)-5-((4-bromophenyl)imino)-4,5-dihydro-1,3,4-thiadiazol-2-yl)ethan-1-one (65).**

Bright yellow solid (78% yield); mp 177–178 °C; IR (KBr) 1692 (C=O), 1643 (C=N), 1613, 1582, 1540 (C=S), 1467, 1390, 1318, 1288, 1238, 1182, 1137, 901, 830, 724, 704, 683, 589, 551, 496, 420 cm^−1^; ^1^H NMR (CDCl_3_, 400 MHz) δ (ppm) 8.63 (s, 2H, Ar-H_(trifluoromethyl)phenyl_), 7.84 (s, 1H, Ar-H_(trifluoromethyl)phenyl_), 7.51 (d, *J* = 8.8 Hz, 2H, Ar-H_p-bromophenyl_), 6.93 (d, *J* = 8.8 Hz, 2H, Ar-H_p-bromophenyl_), 2.66 (s, 3H, CH_3_, COCH_3_); ^13^C NMR (CDCl_3_, 100 MHz) δ (ppm) 189.1 (C=O), 155.2 (N=C-N), 148.9 (C=N-N), 148.8 (**C**-N_p-bromophenyl_), 140.0 (**C**-N_(trifluoromethyl)phenyl_), 132.9 (2 × CH_p-bromophenyl_), 132.4 (q, *J* = 33.7 Hz, Ar**C_q_**-CF_3_), 122.9 (q, *J* = 272.0 Hz, **C**F_3_), 122.2 (2 × CH_p-bromophenyl_), 122.1 (q, *J* = 3.5 Hz, 2 × CH_p-(trifluoromethyl)phenyl_), 120.3 (sept, *J* = 3.7 Hz, CH_(trifluoromethyl)phenyl_), 118.1 (C-Br), 25.2 (C(O)-**C**H_3_); HRMS (ESI^+^): *m*/*z* [M + H]^+^ calcd for C_18_H_11_BrF_6_N_3_OS: 509.9710; found: 509.9700.

**(*Z*)-1-(4-(4-chlorophenyl)-5-((4-chlorophenyl)imino)-4,5-dihydro-1,3,4-thiadiazol-2-yl)ethan-1-one (66).**

Bright yellow solid (93% yield); mp 161 °C; IR (KBr) 1697 (C=O), 1618 (C=N), 1583 (C=S), 1543, 1484, 1367, 1275, 1246, 1142, 1096, 1058, 1012, 866, 826 690, 595 cm^−1^; ^1^H NMR (CDCl_3_, 400 MHz) δ (ppm) 7.93 (d, *J* = 8.8 Hz, 2H, Ar-H_p-chlorophenyl_), 7.46 (d, *J* = 8.8 Hz, 2H, Ar-H_p-chlorophenyl_), 7.33 (d, *J* = 8.8 Hz, 2H, Ar-H_p-chlorophenyl_), 6.96 (d, *J* = 8.8 Hz, 2H, Ar-H_p-chlorophenyl_), 2.61 (s, 3H, CH_3_); ^13^C NMR (CDCl_3_, 100 MHz) δ (ppm) 189.3 (C=O), 156.1 (N=C-N), 149.2 (C-N), 147.4 (C=N-N), 137.1 (C-N), 132.9 (C-Cl), 129.8 (2 × CH), 129.7 (C-Cl), 129.0 (2 × CH), 124.1 (2 × CH), 121.8 (2 × CH), 25.0 (C(O)-**C**H_3_); HRMS (ESI^+^): *m*/*z* [M + H]^+^ calcd for C_16_H_12_Cl_2_N_3_OS: 364.0078; found: 364.0091.

**(*Z*)-1-(4-(4-bromophenyl)-5-((4-bromophenyl)imino)-4,5-dihydro-1,3,4-thiadiazol-2-yl)ethan-1-one (67).**

Bright yellow solid (90% yield); mp 147 °C; IR (KBr) 1654 (C=O), 1615 (C=N), 1592 (C=S), 1515, 1497, 1458, 1415, 1391, 1275, 1151, 1129, 1072, 877, 817, 769, 734, 714, 694, 672, 614, 554, 513, 453 cm^−1^; ^1^H NMR (CDCl_3_, 400 MHz) δ (ppm) 7.88 (d, *J* = 8.8 Hz, 2H, Ar-H_p-bromophenyl_), 7.60 (d, *J* = 8.8 Hz, 2H, Ar-H_p-bromophenyl_), 7.47 (d, *J* = 8.8 Hz, 2H, Ar-H_p-bromophenyl_), 6.90 (d, *J* = 8.8 Hz, 2H, Ar-H_p-bromophenyl_), 2.60 (s, 3H, CH_3_); ^13^C NMR (CDCl_3_, 100 MHz) δ (ppm) 189.3 (C=O), 156.0 (N=C-N), 149.6 (C-N), 147.5 (C=N-N), 137.6 (C-N), 132.8 (2 × CH), 132.0 (2 × CH), 124.4 (2 × CH), 122.2 (2 × CH), 120.8 (C-Br), 117.5 (C-Br), 25.0 (C(O)-**C**H_3_); HRMS (ESI^+^): *m*/*z* [M + H]^+^ calcd for C_16_H_12_Br_2_N_3_OS: 451.9068; found: 451.9059.

**(*Z*)-1-(4-(4-iodophenyl)-5-((4-iodophenyl)imino)-4,5-dihydro-1,3,4-thiadiazol-2-yl)ethan-1-one (68).**

Light yellow solid (88% yield); mp 198 °C; IR (KBr) 1691 (C=O), 1615 (C=N), 1573 (C=S), 1538, 1477, 1365, 1337, 1278, 1242, 1142, 1099, 1057, 1004, 947, 862, 847, 819, 712, 591 cm^−1^; ^1^H NMR (CDCl_3_, 400 MHz) δ (ppm) 7.80 (d, *J* = 8.8 Hz, 2H, Ar-H_p-iodophenyl_), 7.74 (d, *J* = 8.8 Hz, 2H, Ar-H_p-iodophenyl_), 7.66 (d, *J* = 8.4 Hz, 2H, Ar-H_p-iodophenyl_), 6.78 (d, *J* = 8.4 Hz, 2H, Ar-H_p-iodophenyl_), 2.60 (s, 3H, CH_3_); ^13^C NMR (CDCl_3_, 100 MHz) δ (ppm) 189.3 (C=O), 155.8 (N=C-N), 150.3 (C-N_p-iodophenyl_), 147.5 (C=N-N), 138.7 (2 × CH_p-iodophenyl_), 138.4 (C-N_p-iodophenyl_), 138.0 (2 × CH_p-iodophenyl_), 124.5 (2 × CH_p-iodophenyl_), 122.6 (2 × CH_p-iodophenyl_), 92.1 (C-I), 88.3 (C-I), 25.0 (C(O)-**C**H_3_); HRMS (ESI^+^): *m*/*z* [M + H]^+^ calcd for C_16_H_12_I_2_N_3_OS: 547.8791; found: 547.8800.

**(*Z*)-1-(4-(4-methoxyphenyl)-5-((4-methoxyphenyl)imino)-4,5-dihydro-1,3,4-thiadiazol-2-yl)ethan-1-one (69).**

Yellow solid (73% yield); mp 111–113 °C; IR (KBr) 1682 (C=O), 1623 (C=N), 1602, 1530 (C=S), 1504, 1442, 1368, 1277, 1251, 1176, 1150, 1097, 1036, 947, 820, 725, 682, 631, 595, 560, 520, cm^−1^; ^1^H NMR (CDCl_3_, 400 MHz) δ (ppm) 7.81 (d, *J* = 8.8 Hz, 2H, Ar-H_p-methoxyphenyl_), 7.00 (d, *J* = 8.8 Hz, 2H, Ar-H_p-methoxyphenyl_), 6.98 (d, *J* = 8.4 Hz, 2H, Ar-H_p-methoxyphenyl_), 6.89 (d, *J* = 8.4 Hz, 2H, Ar-H_p-methoxyphenyl_), 3.85 (s, 3H, OCH_3_), 3.80 (s, 3H, OCH_3_), 2.58 (s, 3H, CH_3_, COCH_3_); ^13^C NMR (CDCl_3_, 100 MHz) δ (ppm) 189.5 (C=O), 158.7 (C-O), 156.5 (C-O), 156.2 (N=C-N), 146.3 (C=N-N), 144.2 (C-N_p-methoxyphenyl_), 131.7 (**C**-N_p-methoxyphenyl_), 125.0 (2 × CH_p-methoxyphenyl_), 121.6 (2 × CH_p-methoxyphenyl_), 114.8 (2 × CH_p-methoxyphenyl_), 114.1 (2 × CH_p-methoxyphenyl_), 55.5 (O-CH_3_), 55.4 (O-CH_3_), 24.9 (C(O)-**C**H_3_); HRMS (ESI^+^): *m*/*z* [M + H]^+^ calcd for C_18_H_18_N_3_O_3_S: 356.1069; found: 356.1078.

**(*Z*)-1-(4-(4-iodophenyl)-5-((4-methoxyphenyl)imino)-4,5-dihydro-1,3,4-thiadiazol-2-yl)ethan-1-one (70).**

Yellow solid (89% yield); mp 139 °C; IR (KBr) 1682 (C=O), 1629 (C=N), 1578 (C=S), 1528, 1505, 1483, 1370, 1345, 1269, 1239, 1175, 1156, 1068, 1033, 1004, 944, 829, 639, 594, 517 cm^−1^; ^1^H NMR (CDCl_3_, 400 MHz) δ (ppm) 7.79 (broad singlet of coalesced quartet, 4H, Ar-H_p-iodophenyl_), 6.97 (d, *J* = 8.8 Hz, 2H, Ar-H_p-iodophenyl_), 6.90 (d, *J* = 8.8 Hz, 2H, Ar-H_p-methoxyphenyl_), 6.91 (d, *J* = 8.8 Hz, 2H, Ar-H_p-methoxyphenyl_), 3.80 (s, 3H, OCH_3_), 2.59 (s, 3H, CH_3_, COCH_3_); ^13^C NMR (CDCl_3_, 100 MHz) δ (ppm) 189.4 (C=O), 156.7 (C-O), 155.2 (N=C-N), 147.3 (C=N-N), 143.8 (**C**-N_p-methoxyphenyl_), 138.6 (**C**-N_p-iodophenyl_), 137.9 (2 × CH_p-iodophenyl_), 124.4 (2 × CH_p-iodophenyl_), 121.4 (2 × CH_p-methoxyphenyl_), 114.9 (2 × CH_p-methoxyphenyl_), 91.8 (C-I), 55.5 (OCH_3_), 25.0 (C(O)-**C**H_3_); HRMS (ESI^+^): *m*/*z* [M + H]^+^ calcd for C_17_H_15_IN_3_O_2_S: 451.9930; found: 451.9921.

**(*Z*)-1-(4-(3,5-bis(trifluoromethyl)phenyl)-5-((4-methoxyphenyl)imino)-4,5-dihydro-1,3,4-thiadiazol-2-yl)ethan-1-one (71).**

Bright yellow solid (94% yield); mp 165 °C; IR (KBr) 1691 (C=O), 1644 (C=N), 1613, 1541 (C=S), 1508, 1466, 1391, 1369, 1317, 1290, 1255, 1181, 1167, 1132, 1099, 1034, 900, 838, 808, 770, 725, 704, 685, 594, 576, 527, 512, 421 cm^−1^; ^1^H NMR (CDCl_3_, 400 MHz) δ (ppm) 8.68 (s, 2H, Ar-H_(trifluoromethyl)phenyl_), 7.82 (s, 1H, Ar-H_(trifluoromethyl)phenyl_), 7.01 (d, *J* = 8.8 Hz, 2H, Ar-H_p-methoxyphenyl_), 6.94 (d, *J* = 8.8 Hz, 2H, Ar-H_p-methoxyphenyl_), 3.82 (s, 3H, OCH_3_), 2.65 (s, 3H, CH_3_, COCH_3_); ^13^C NMR (CDCl_3_, 100 MHz) δ (ppm) 189.3 (C=O), 157.0 (C-O), 153.9 (N=C-N), 148.6 (C=N-N), 143.0 (**C**-N_p-methoxyphenyl_), 140.3 (**C**-N_(trifluoromethyl)phenyl_), 132.3 (q, *J* = 34.0 Hz, Ar**C_q_**-CF_3_), 123.0 (q, *J* = 271.0 Hz, **C**F_3_), 121.9 (q, *J* = 3.5 Hz, 2 × CH_p-(trifluoromethyl)phenyl_), 121.5 (2 × CH_p-methoxyphenyl_), 119.9 (sept, *J* = 3.8 Hz, CH_(trifluoromethyl)phenyl_), 114.9 (2 × CH_p-methoxyphenyl_), 55.5 (O-CH_3_), 25.2 (C(O)-**C**H_3_); HRMS (ESI^+^): *m*/*z* [M + H]^+^ calcd for C_19_H_14_F_6_N_3_O_2_S: 462.0711; found: 462.0701.

**(*Z*)-1-(5-((3,4-dimethoxyphenyl)imino)-4-(4-methoxyphenyl)-4,5-dihydro-1,3,4-thiadiazol-2-yl)ethan-1-one (72).**

Light orange solid (77% yield); mp 132 °C; IR (KBr) 1684 (C=O), 1630 (C=N), 1518, 1547 (C=S), 1372, 1331, 1282, 1175, 1135, 1029, 944, 828, 763, 603, 518 cm^−1^; ^1^H NMR (CDCl_3_, 400 MHz) δ (ppm) 7.80 (d, *J* = 9.2 Hz, 2H, Ar-H_p-methoxyphenyl_), 7.01 (d, *J* = 9.2 Hz, 2H, Ar-H_p-methoxyphenyl_), 6.84 (d, *J* = 8.8 Hz, 1H, Ar-H_(3,4-dimethoxyphenyl)_), 6.62 (dd, *J* = 8.8, 2.4 Hz, 1H, Ar-H_(3,4-dimethoxyphenyl)_), 6.59 (d, *J* = 2.4 Hz, 1H, Ar-H_(3,4-dimethoxyphenyl)_), 3.87 (s, 3H, OCH_3_), 3.85 (s, 3H, OCH_3_), 3.84 (s, 3H, OCH_3_), 2.58 (s, 3H, CH_3_); ^13^C NMR (CDCl_3_, 100 MHz) δ (ppm) 189.5 (C=O), 158.8 (C-O_p-methoxyphenyl_), 156.5 (N=C-N), 149.6 (C-O_(3,4-dimethoxyphenyl)_), 146.5 (C=N-N), 146.0 (C-O_(3,4-dimethoxyphenyl)_), 144.4 (C-N_(3,4-dimethoxyphenyl_), 131.6 (**C**-N_p-methoxyphenyl_), 125.1 (2 × CH_p-methoxyphenyl_), 114.1 (2 × CH_p-methoxyphenyl_), 111.7 (CH_(3,4-dimethoxyphenyl)_), 111.2 (CH_(3,4-dimethoxyphenyl)_), 105.3 (CH_(3,4-dimethoxyphenyl)_), 56.0 (O**C**H_3_), 55.8 (O**C**H_3_), 55.5 (O**C**H_3_), 25.0 (C(O)-**C**H_3_); HRMS (ESI^+^): *m*/*z* [M + H]^+^ calcd for C_19_H_20_N_3_O_4_S: 386.1175; found: 386.1169.

**(*Z*)-1-(4-(benzo[d][1,3]dioxol-5-yl)-5-((3,4-dimethoxyphenyl)imino)-4,5-dihydro-1,3,4-thiadiazol-2-yl)ethan-1-one (73).**

Brown solid (90% yield); mp 164 °C; IR (KBr) 1678 (C=O), 1626 (C=N), 1577, 1513 (C=S), 1486, 1365, 1323, 1229, 1176, 1127, 1036, 937, 847, 825, 771, 736, 664, 607, 559 cm^−1^; ^1^H NMR (CDCl_3_, 400 MHz) δ (ppm) 7.42–7.34 (m, 2H, Ar-H), 6.89 (d, *J* = 8.4 Hz, 1H, Ar-H), 6.84 (d, *J* = 8.4 Hz, 1H, Ar-H), 6.63–6.56 (m, 2H, Ar-H), 6.03 (OCH_2_), 3.86 (s, 3H, OCH_3_), 3.84 (s, 3H, OCH_3_), 2.57 (s, 3H, CH_3_); ^13^C NMR (CDCl_3_, 100 MHz) δ (ppm) 189.5 (C=O), 156.1 (N=C-N), 149.6 (C-N_(3,4-dimethoxyphenyl_), 147.7 (C-O), 146.9 (C-O), 146.4 (C=N-N), 145.9 (C-O), 144.5 (C-O), 132.5 (C-N_4-(benzo[d][1,3]dioxol_), 117.5 (CH_(benzo[d][1,3]dioxol_), 111.7 (CH_(3,4-dimethoxyphenyl_), 111.1 (CH_(3,4-dimethoxyphenyl_), 108.0 (CH_(benzo[d][1,3]dioxol_), 105.4 (CH_(benzo[d][1,3]dioxol_), 105.2 (CH_(3,4-dimethoxyphenyl_), 101.8 (O**C**H_2-(benzo[d][1,3]dioxol_), 60.0 (O**C**H_3_), 55.8 (O**C**H_3_), 24.9 (C(O)-**C**H_3_); HRMS (ESI^+^): *m*/*z* [M + H]^+^ calcd for C_19_H_18_N_3_O_5_S: 400.0967; found: 400.0979.

**(*Z*)-1-(4-(3,5-bis(trifluoromethyl)phenyl)-5-((2,5-dimethoxyphenyl)imino)-4,5-dihydro-1,3,4-thiadiazol-2-yl)ethan-1-one (74).**

Bright yellow solid (46% yield); mp 125 °C; IR (KBr) 1690 (C=O), 1632 (C=N), 1582, 1540 (C=S), 1500, 1389, 1277, 1200, 1127, 1028, 940, 895, 852, 787, 699, 584 cm^−1^; ^1^H NMR (CDCl_3_, 400 MHz) δ (ppm) 8.75 (s, 2H, Ar-H_(trifluoromethyl)phenyl_), 7.82 (s, 1H, Ar-H_(trifluoromethyl)phenyl_), 6.91 (d, *J* = 8.8 Hz, 1H, Ar-H_2,5-dimethoxyphenyl_), 6.68 (dd, *J* = 8.8, 3.2 Hz, 1H, Ar-H_2,5-dimethoxyphenyl_), 6.60 (d, *J* = 3.2 Hz, H, Ar-H_2,5-dimethoxyphenyl_), 3.80 (s, 3H, OCH_3_), 3.77 (s, 3H, OCH_3_), 2.66 (s, 3H, CH_3_, COCH_3_); ^13^C NMR (CDCl_3_, 100 MHz) δ (ppm) 189.3 (C=O), 155.2 (N=C-N), 154.2 (C-O), 149.4 (C=N-N), 145.0 (C-O), 140.3 (**C**-N_(trifluoromethyl)phenyl_), 139.2 (**C**-N_2,5-dimethoxyphenyl_), 132.2 (q, *J* = 33.6 Hz, Ar**C_q_**-CF_3_), 123.0 (q, *J* = 271.0 Hz, **C**F_3_), 122.1 (q, *J* = 3.5 Hz, 2 × CH_p-(trifluoromethyl)phenyl_), 121.5 (2 × CH_2,5-dimethoxyphenyl_), 120.0 (sept, *J* = 3.8 Hz, CH_(trifluoromethyl)phenyl_), 113.4 (2 × CH_2,5-dimethoxyphenyl_), 110.3 (2 × CH_2,5-dimethoxyphenyl_), 106.6 (2 × CH_2,5-dimethoxyphenyl_), 56.3 (O-CH_3_), 55.7 (O-CH_3_), 25.2 (C(O)-**C**H_3_); HRMS (ESI^+^): *m*/*z* [M + H]^+^ calcd for C_20_H_16_F_6_N_3_O_3_S: 492.0817; found: 492.0805.

**(*Z*)-1-(4-(benzo[d][1,3]dioxol-5-yl)-5-((4-ethoxyphenyl)imino)-4,5-dihydro-1,3,4-thiadiazol-2-yl)ethan-1-one (75).**

Yellow brownish oil (95% yield); IR (KBr) 1690 (C=O), 1623 (C=N), 1504 (C=S), 1487, 1227, 1142, 1043, 942, 558 cm^−1^; ^1^H NMR (CDCl_3_, 400 MHz) δ (ppm) 7.42–7.37 (m, 2H, Ar-H_4-benzyloxy)phenyl_), 6.95 (d, *J* = 8.8 Hz, 2H, Ar-H_4-ethoxyphenyl_), 6.89 (d, *J* = 8.4 Hz, 1H, Ar-H_4-benzyloxy)phenyl_), 6.88 (d, *J* = 8.8 Hz, 2H, Ar-H_4-ethoxyphenyl_), 6.03 (OCH_2_), 4.01 (q, *J* = 6.8 Hz, 2H, OCH_2_), 2.57 (s, 3H, CH_3_), 1.41 (t, *J* = 6.8 Hz, 3H, OCH_2_**CH_3_**); ^13^C NMR (CDCl_3_, 100 MHz) δ (ppm) 189.5 (C=O), 155.8 (C-O), 155.7 (N=C-N), 147.7 (C-N_4-(benzyloxy)phenyl_), 146.8 (C-O), 146.3 (C=N-N), 144.1 (C-O), 132.7 (C-N_4-(benzo[d][1,3]dioxol_), 121.4 (2 × CH), 117.3 (CH_(benzo[d][1,3]dioxol_), 115.3 (2 × CH), 108.0 (CH_(benzo[d][1,3]dioxol_), 105.4 (CH_(benzo[d][1,3]dioxol_), 101.7 (O**C**H_2-(benzo[d][1,3]dioxol_), 63.6 (O**C**H_2_), 24.9 (C(O)-**C**H_3_), 14.8 (OCH_2_**CH_3_**); HRMS (ESI^+^): *m*/*z* [M + H]^+^ calcd for C_19_H_18_N_3_O_4_S: 384.1018; found: 384.1009.

**(*Z*)-1-(4-(benzo[d][1,3]dioxol-5-yl)-5-((4-(benzyloxy)phenyl)imino)-4,5-dihydro-1,3,4-thiadiazol-2-yl)ethan-1-one (76).**

Dark yellow solid (63% yield); mp 122 °C; IR (KBr) 1684 (C=O), 1636 (C=N), 1581, 1528 (C=S), 1500, 1369, 1283, 1222, 1168, 1137, 1104, 1033, 936, 843, 700, 555, 518 cm^−1^; ^1^H NMR (CDCl_3_, 400 MHz) δ (ppm) 7.50–7.30 (m, 7H, Ar-H), 7.03–6.86 (m, 5H, Ar-H), 6.04 (OCH_2_), 5.05 (OCH_2_), 2.58 (s, 3H, CH_3_); ^13^C NMR (CDCl_3_, 100 MHz) δ (ppm) 189.4 (C=O), 155.9 (N=C-N), 155.7 (C-O), 147.7 (C-N_4-(benzyloxy)phenyl_), 146.8 (C-O), 146.3 (C=N-N), 144.3 (C-O), 136.9 (Ar**C_q_**-Bn), 132.6 (C-N_4-(benzo[d][1,3]dioxol_), 128.5 (2 × CH), 127.9 (CH), 127.5 (2 × CH), 121.5 (2 × CH), 117.4 (CH_(benzo[d][1,3]dioxol_), 115.8 (2 × CH), 108.0 (CH_(benzo[d][1,3]dioxol_), 105.4 (CH_(benzo[d][1,3]dioxol_), 101.7 (O**C**H_2-(benzo[d][1,3]dioxol_), 70.2 (O**C**H_2_Bn), 24.9 (C(O)-**C**H_3_); HRMS (ESI^+^): *m*/*z* [M + H]^+^ calcd for C_24_H_20_N_3_O_4_S: 446.1175; found: 446.1163.

**(*Z*)-1-(4-(4-iodophenyl)-5-((2-(trifluoromethyl)phenyl)imino)-4,5-dihydro-1,3,4-thiadiazol-2-yl)ethan-1-one (77).**

Dark yellow solid (66% yield); mp 134–137 °C; IR (KBr) 1688 (C=O), 1622 (C=N), 1573, 1537 (C=S), 1483, 1450, 1364, 1338, 1314, 1281, 1114, 1055, 1004, 942, 844, 819, 759, 704, 646, 585, 450 cm^−1^; ^1^H NMR (CDCl_3_, 400 MHz) δ (ppm) 7.82 (d, *J* = 8.8 Hz, 2H, Ar-H_p-iodophenyl_), 7.78 (d, *J* = 8.8 Hz, 2H, Ar-H_p-iodophenyl_), 7.68 (d, *J* = 7.6 Hz, 1H, Ar-H_m-(trifluoromethyl)phenyl_), 7.54 (t, *J* = 7.6 Hz, 1H, Ar-H_m-(trifluoromethyl)phenyl_), 7.21 (t, *J* = 8.0 Hz, 1H, Ar-H_m-(trifluoromethyl)phenyl_), 7.08 (d, *J* = 8.0 Hz, 1H, Ar-H_m-(trifluoromethyl)phenyl_), 2.61 (s, 3H, CH_3_); ^13^C NMR (CDCl_3_, 100 MHz) δ (ppm) 189.3 (C=O), 156.6 (N=C-N), 148.9 (**C**-N*_o_*_-(trifluoromethyl)phenyl_), 147.9 (C=N-N), 138.2 (C-N_p-iodophenyl_), 138.0 (2 × CH_p-iodophenyl_), 133.7 (CH*_o_*_-(trifluoromethyl)phenyl_), 127.0 (q, *J* = 5.0 Hz, CH*_o_*_-(trifluoromethyl)phenyl_), 124.6 (2 × CH_p-iodophenyl_), 124.3 (CH*_o_*_-(trifluoromethyl)phenyl_), 123.8 (q, *J* = 272.0 Hz, **C**F_3_), 122.5 (q, *J* = 30.0 Hz, Ar**C_q_**-CF_3_), 119.7 (CH*_o_*_-(trifluoromethyl)phenyl_), 92.4 (C-I), 25.0 (C(O)-**C**H_3_); HRMS (ESI^+^): *m*/*z* [M + H]^+^ calcd for C_17_H_12_IF_3_N_3_OS: 489.9698; found: 489.9709.

**(*Z*)-1-(5-((2,4-dimethylphenyl)imino)-4-(4-iodophenyl)-4,5-dihydro-1,3,4-thiadiazol-2-yl)ethan-1-one (78).**

Dark yellow solid (94% yield); mp 181 °C; IR (KBr) 1691 (C=O), 1635 (C=N), 1581, 1532 (C=S), 1482, 1368, 1271, 1227, 1157, 1056, 1003, 942, 849, 817, 675, 579 cm^−1^; ^1^H NMR (CDCl_3_, 400 MHz) δ (ppm) 7.86 (d, *J* = 8.8 Hz, 2H, Ar-H_p-iodophenyl_), 7.80 (d, *J* = 8.8 Hz, 2H, Ar-H_p-iodophenyl_), 7.05 (s, 1H, Ar-H_2,4-dimethylphenyl_), 7.01 (d, *J* = 8.0 Hz, 1H, Ar-H_2,4-dimethylphenyl_), 6.83 (d, *J* = 8.0 Hz, 1H, Ar-H_2,4-dimethylphenyl_), 2.60 (s, 3H, CH_3_), 2.31 (s, 3H, CH_3_, Ar-CH_3_), 2.16 (s, 3H, CH_3_, Ar-CH_3_); ^13^C NMR (CDCl_3_, 100 MHz) δ (ppm) 189.4 (C=O), 155.0 (N=C-N), 147.7 (C=N-N), 147.1 (C-N_2,4-dimethylphenyl_), 138.7 (C-N*_p_*_-iodophenyl_), 137.9 (2 × CH_p-iodophenyl_), 134.4 (Ar**C_q_**-Me), 131.7 (CH_2,4-dimethylphenyl_), 129.8 (Ar**C_q_**-Me), 127.8 (2 × CH_p-iodophenyl_), 124.2 (CH_2,4-dimethylphenyl_), 118.0 (CH_2,4-dimethylphenyl_), 91.6 (C-I), 25.0 (C(O)-**C**H_3_), 20.9 (**C**H_3_), 17.8 (**C**H_3_); HRMS (ESI^+^): *m*/*z* [M + H]^+^ calcd for C_18_H_17_IN_3_OS: 450.0137; found: 450.0148.

**(*Z*)-1-(5-((2-bromophenyl)imino)-4-(4-iodophenyl)-4,5-dihydro-1,3,4-thiadiazol-2-yl)ethan-1-one (79).**

Yellow solid (81% yield); mp 201 °C; IR (KBr) 1693 (C=O), 1621 (C=N), 1574, 1534 (C=S), 1460, 1362, 1336, 1280, 1237, 1148, 1096, 1054, 1024, 939, 842, 816, 752, 708, 582, 537, 514, 498, 444, 415 cm^−1^; ^1^H NMR (CDCl_3_, 400 MHz) δ (ppm) 7.88 (d, *J* = 8.4 Hz, 2H, Ar-H_p-iodophenyl_), 7.82 (d, *J* = 8.4 Hz, 2H, Ar-H_p-iodophenyl_), 7.63 (d, *J* = 8.4 Hz, 1H, Ar-H*_o_*_-bromophenyl_), 7.31 (t, *J* = 8.4 Hz, 1H, Ar-H*_o_*_-bromophenyl_), 7.05–6.96 (m, 2H, Ar-H*_o_*_-bromophenyl_), 2.61 (s, 3H, CH_3_); ^13^C NMR (CDCl_3_, 100 MHz) δ (ppm) 189.2 (C=O), 156.9 (N=C-N), 149.2 (C-N*_o_*_-bromophenyl_), 148.0 (C=N-N), 138.3 (C-N*_p_*_-iodophenyl_), 138.0 (2 × CH_p-iodophenyl_), 133.5 (CH*_o_*_-bromophenyl_), 128.9 (CH*_o_*_-bromophenyl_), 125.9 (CH*_o_*_-bromophenyl_), 124.7 (2 × CH_p-iodophenyl_), 120.1 (CH*_o_*_-bromophenyl_), 117.1 (C-Br), 92.3 (C-I), 25.0 (C(O)-**C**H_3_); HRMS (ESI^+^): *m*/*z* [M + H]^+^ calcd for C_16_H_12_IBrN_3_OS: 499.8929; found: 499.8943.

**(*Z*)-1-(5-((2,4-dichlorophenyl)imino)-4-(4-iodophenyl)-4,5-dihydro-1,3,4-thiadiazol-2-yl)ethan-1-one (80).**

Light grey solid (57% yield); mp 206–207 °C; IR (KBr) 1694 (C=O), 1631 (C=N), 1578, 1538 (C=S), 1483, 1366, 1337, 1280, 1262, 1240, 1148, 1099, 1055, 1003, 944, 871, 823, 806, 678, 599, 497 cm^−1^; ^1^H NMR (CDCl_3_, 400 MHz) δ (ppm) 7.82 (broad singlet of coalesced quartet, 4H, Ar-H_p-iodophenyl_), 7.46 (d, *J* = 2.4 Hz, 1H, Ar-H_2,4-dichlorophenyl_), 7.24 (dd, *J* = 8.8, 2.0 Hz, 1H, Ar-H_2,4-dichlorophenyl_), 6.95 (d, *J* = 8.4 Hz, 1H, Ar-H_2,4-dichlorophenyl_), 2.61 (s, 3H, CH_3_); ^13^C NMR (CDCl_3_, 100 MHz) δ (ppm) 189.1 (C=O), 157.3 (N=C-N), 148.1 (C=N-N), 146.4 (C-N_2,4-dichlorophenyl_), 138.2 (C-N*_p_*_-iodophenyl_), 138.0 (2 × CH_p-iodophenyl_), 130.2 (CH_2,4-dichlorophenyl_), 130.0 (C-Cl), 128.3 (CH_2,4-dichlorophenyl_), 127.7 (C-Cl), 124.7 (2 × CH_p-iodophenyl_), 121.2 (CH_2,4-dichlorophenyl_), 92.4 (C-I), 25.0 (C(O)-**C**H_3_); HRMS (ESI^+^): *m*/*z* [M + H]^+^ calcd for C_16_H_11_Cl_2_IN_3_OS: 489.9045; found: 489.9056.

**(*Z*)-1-(4-(4-iodophenyl)-5-((2-(methylthio)phenyl)imino)-4,5-dihydro-1,3,4-thiadiazol-2-yl)ethan-1-one (81).**

Yellow solid (90% yield); mp 156 °C; IR (KBr) 1691 (C=O), 1635 (C=N), 1570, 1529 (C=S), 1482, 1365, 1280, 1147, 1067, 940, 819, 745, 669, 615, 587, 535, 496, 437 cm^−1^; ^1^H NMR (CDCl_3_, 400 MHz) δ (ppm) 7.90 (d, *J* = 8.4 Hz, 2H, Ar-H*_p_*_-iodophenyl_), 7.82 (d, *J* = 8.4 Hz, 2H, Ar-H_p-iodophenyl_), 7.22–7.11 (m, 3H, Ar-H*_o_*_-(methylthio)phenyl_), 6.99–6.94 (m, 1H, Ar-H*_o_*_-(methylthio)phenyl_), 2.60 (s, 3H, CH_3_), 2.42 (s, 3H, CH_3_, S-CH_3_); ^13^C NMR (CDCl_3_, 100 MHz) δ (ppm) 189.4 (C=O), 156.2 (N=C-N), 147.8 (C=N-N), 147.5 (C-N*_p_*_-methylthiophenyl_), 138.4 (C-N*_p_*_-iodophenyl_), 137.9 (2 × CH*_p_*_-iodophenyl_), 132.4 (Ar**C_q_**-SMe), 125.5 (CH*_o_*_-(methylthio)phenyl_), 125.3 (CH*_o_*_-(methylthio)phenyl_), 124.8 (CH*_o_*_-(methylthio)phenyl_), 124.7 (2 × CH*_p_*_-iodophenyl_), 117.7 (CH*_o_*_-(methylthio)phenyl_), 92.1 (C-I), 25.0 (C(O)-**C**H_3_), 14.5 (S-**C**H_3_); HRMS (ESI^+^): *m*/*z* [M + H]^+^ calcd for C_17_H_15_IN_3_OS_2_: 467.9701; found: 467.9711.

**(*Z*)-3-((5-acetyl-3-(4-bromophenyl)-1,3,4-thiadiazol-2(3H)-ylidene)amino)benzonitrile (82).**

Bright yellow solid (85% yield); mp 194 °C; IR (KBr) 1683 (C=O), 1641 (C=N), 1581, 1538 (C=S), 1483, 1366, 1338, 1284, 1258, 1169, 1135, 1096, 1074, 1054, 1004, 943, 903, 825, 794, 760, 689, 588, 519, 474, 422 cm^−1^; ^1^H NMR (CDCl_3_, 400 MHz) δ (ppm) 7.86 (d, *J* = 8.8 Hz, 2H, Ar-H_p-bromophenyl_), 7.61 (d, *J* = 8.8 Hz, 2H, Ar-H_p-bromophenyl_), 7.47 (t, *J* = 8.0 Hz, 1H, Ar-H_m-cyanophenyl_), 7.40 (d, *J* = 7.6 Hz, 1H, Ar-H_m-cyanophenyl_), 7.29 (s, Ar-H_m-cyanophenyl_), 7.25 (d, *J* = 7.2 Hz, 1H, Ar-H_m-cyanophenyl_), 2.62 (s, 3H, CH_3_); ^13^C NMR (CDCl_3_, 100 MHz) δ (ppm) 189.1 (C=O), 157.1 (N=C-N), 151.3 (**C**-N_m-cyanophenyl_), 147.6 (C=N-N), 137.4 (C-N_p-bromophenyl_), 132.0 (2 × CH_p-bromophenyl_), 130.8 (CH_m-cyanophenyl_), 128.0 (CH_m-cyanophenyl_), 125.1 (CH_m-cyanophenyl_), 124.5 (2 × CH_p-bromophenyl_), 124.3 (CH_m-cyanophenyl_), 121.1 (C-Br), 118.4 (**C**N), 113.6 (**C**-CN), 25.0 (C(O)-**C**H_3_); HRMS (ESI^+^): *m*/*z* [M + H]^+^ calcd for C_17_H_12_BrN_4_OS: 398.9915; found: 398.9906.

**(*Z*)-1-(3-((5-acetyl-3-(4-bromophenyl)-1,3,4-thiadiazol-2(3H)-ylidene)amino)phenyl)ethan-1-one (83).**

Bright yellow solid (81% yield); mp 122–125 °C; IR (KBr) 1688 (C=O), 1634 (C=N), 1577, 1534 (C=S), 1484, 1426, 1359, 1280, 1213, 1143, 1055, 1007, 947, 893, 826, 751, 693, 585, 498 cm^−1^; ^1^H NMR (CDCl_3_, 400 MHz) δ (ppm) 7.90 (d, *J* = 8.8 Hz, 2H, Ar-H_p-bromophenyl_), 7.74–7.70 (m, 1H, Ar-H_5-acetylphenyl_), 7.64–7.58 (m, 3H, 2Ar-H_p-bromophenyl_ and 1Ar-H_5-acetylphenyl_), 7.47 (t, *J* = 8.0 Hz, 1H, Ar-H_5-acetylphenyl_), 7.24–7.20 (m, 1H, Ar-H_5-acetylphenyl_), 2.61 (s, 3H, CH_3_), 2.60 (s, 3H, CH_3_); ^13^C NMR (CDCl_3_, 100 MHz) δ (ppm) 197.6 (C=O), 189.3 (C=O), 156.5 (N=C-N), 151.1 (C-N_5-acetylphenyl_), 147.5 (C=N-N), 138.6 (Ar**C_q_**-Ac), 137.6 (C-N_p-bromophenyl_), 132.0 (2 × CH_p-bromophenyl_), 130.1 (CH_5-acetylphenyl_), 125.1 (CH_5-acetylphenyl_), 124.6 (CH_5-acetylphenyl_), 124.4 (2 × CH_p-bromophenyl_), 120.9 (C-Br), 120.4 (CH_5-acetylphenyl_), 26.7 (C(O)-**C**H_3_), 25.0 (C(O)-**C**H_3_); HRMS (ESI^+^): *m*/*z* [M + H]^+^ calcd for C_18_H_15_BrN_3_O_2_S: 416.0068; found: 416.0080.

**Ethyl (*Z*)-4-((5-acetyl-3-(4-bromophenyl)-1,3,4-thiadiazol-2(3H)-ylidene)amino)benzoate (84).**

Bright yellow solid (75% yield); mp 123–124 °C; IR (KBr) 1717 (ester C=O), 1685 (C=O), 1637 (C=N), 1599, 1519 (C=S), 1485, 1364, 1281, 1172, 1150, 1102, 1056, 1010, 942, 864, 820, 770, 703, 593, 500 cm^−1^; ^1^H NMR (CDCl_3_, 400 MHz) δ (ppm) 8.06 (d, *J* = 8.0 Hz, 2H, Ar-H_ethylbenzoate_), 7.89 (d, *J* = 8.8 Hz, 2H, Ar-H_p-bromophenyl_), 7.61 (d, *J* = 8.8 Hz, 2H, Ar-H_p-bromophenyl_), 7.06 (d, *J* = 8.0 Hz, 2H, Ar-H_ethylbenzoate_), 4.37 (q, *J* = 7.2 Hz, 2H, OCH_2_), 2.61 (s, 3H, CH_3_), 1.39 (t, *J* = 7.2 Hz, 3H, OCH_2_**CH_3_**); ^13^C NMR (CDCl_3_, 100 MHz) δ (ppm) 189.2 (C=O), 166.1 (C=O), 156.1 (N=C-N), 154.5 (C-N_ethylbenzoate_), 147.7 (C=N-N), 137.6 (C-N_p-bromophenyl_), 132.0 (2 × CH_p-bromophenyl_), 131.5 (2 × CH_ethylbenzoate_), 126.6 (Ar**C**_q-_COOEt), 124.5 (2 × CH_p-bromophenyl_) 120.9 (C-Br), 120.3 (2 × CH_ethylbenzoate_), 60.9 (O**C**H_2_), 25.1 (C(O)-**C**H_3_), 14.3 (OCH_2_**CH_3_**); HRMS (ESI^+^): *m*/*z* [M + H]^+^ calcd for C_19_H_17_BrN_3_O_3_S: 446.0174; found: 446.0163.

**(*Z*)-1-(4-(4-chloro-2-methylphenyl)-5-((2,4-dimethylphenyl)imino)-4,5-dihydro-1,3,4-thiadiazol-2-yl)ethan-1-one (85).**

Yellow oil (93% yield); IR (KBr) 1691 (C=O), 1630 (C=N), 1513 (C=S), 1486, 1365, 1284, 1252, 1203, 1162, 1101, 1055, 942, 890, 584 cm^−1^; ^1^H NMR (CDCl_3_, 400 MHz) δ (ppm) 7.44 (d, *J* = 8.4 Hz, 1H, Ar-H_4-chloro-2-methylphenyl_), 7.05 (d, 1H, *J* = 2.0 Hz, Ar-H_4-chloro-2-methylphenyl_), 7.35 (dd, *J* = 8.4, 2.4 Hz, 1H, Ar-H_4-chloro-2-methylphenyl_), 7.02 (s, 1H, Ar-H_2,4-dimethylphenyl_), 7.01 (d, *J* = 8.0 Hz, 1H, Ar-H_2,4-dimethylphenyl_), 6.81 (d, *J* = 8.0 Hz, 1H, Ar-H_2,4-dimethylphenyl_), 2.55 (s, 3H, CH_3_), 2.37 (s, 3H, CH_3_, Ar-CH_3_), 2.30 (s, 3H, CH_3_, Ar-CH_3_), 2.10 (s, 3H, CH_3_, Ar-CH_3_); ^13^C NMR (CDCl_3_, 100 MHz) δ (ppm) 189.3 (C=O), 155.8 (N=C-N), 147.4 (C=N-N), 147.3 (C-N_2,4-dimethylphenyl_), 137.8 (Ar**C_q_**-Me), 135.6 (C-N_4-chloro-2-methylphenyl_), 135.2 (C-Cl_4-chloro-2-methylphenyl_), 133.9 (Ar**C_q_**-Me), 131.5 (CH_2,4-dimethylphenyl_), 131.2 (CH_4-chloro-2-methylphenyl_), 129.6 (Ar**C_q_**-Me), 129.1 (CH_4-chloro-2-methylphenyl_), 127.5 (CH_2,4-dimethylphenyl_), 127.0 (CH_4-chloro-2-methylphenyl_), 118.0 (CH_2,4-dimethylphenyl_), 24.8 (C(O)-**C**H_3_), 20.8 (**C**H_3_), 18.0 (**C**H_3_), 17.6 (**C**H_3_); HRMS (ESI^+^): *m*/*z* [M + H]^+^ calcd for C_19_H_19_ClN_3_OS: 372.0937; found: 372.0923.

**(*Z*)-1-(4-(4-bromophenyl)-5-((4-isopropylphenyl)imino)-4,5-dihydro-1,3,4-thiadiazol-2-yl)ethan-1-one (86).**

Yellow brownish oil (64% yield); IR (KBr) 1693 (C=O), 1613 (C=N), 1547, 1481, 1363, 1323, 1263, 1244, 1135, 1088, 1072, 1044, 1008, 939, 825, 809, 588, 534, 505, 458 cm^−1^; ^1^H NMR (CDCl_3_, 400 MHz) δ (ppm) 7.94 (d, *J* = 8.8 Hz, 2H, Ar-H_p-bromophenyl_), 7.56 (d, *J* = 8.8 Hz, 2H, Ar-H_p-bromophenyl_), 8.26 (d, *J* = 8.0 Hz, 2H, Ar-H_p-isopropylphenyl_), 7.14 (d, *J* = 8.0 Hz, 2H, Ar-H_p-isopropylphenyl_), 2.90 (sept, *J* = 6.8 Hz, 1H, CH_3_C**H**CH_3_), 2.60 (s, 3H, CH_3_), 1.25 (d, *J* = 6.8 Hz, 6H, C**H_3_**CHC**H_3_**); ^13^C NMR (CDCl_3_, 100 MHz) δ (ppm) 189.5 (C=O), 154.9 (N=C-N), 148.3 (C-N_p-isopropylphenyl_), 147.2 (C=N-N), 145.3 (Ar**C_q_**-*^i^*Pr), 137.9 (C-N_p-bromophenyl_), 131.8 (2 × CH_p-bromophenyl_), 127.6 (2 × CH_p-isopropylphenyl_), 124.1 (2 × CH_p-bromophenyl_), 120.3 (C-Br), 120.1 (2 × CH_p-isopropylphenyl_), 33.6 (CH_3_**CH**CH_3_), 25.0 (C(O)-**C**H_3_), 24.0 (**CH_3_**CH**CH_3_**); HRMS (ESI^+^): *m*/*z* [M + H]^+^ calcd for C_19_H_19_BrN_3_OS: 416.0432; found: 416.0419.

**(*Z*)-1-(4-phenyl-5-(phenylimino)-4,5-dihydro-1,3,4-thiadiazol-2-yl)ethan-1-one (87)** [31]**.**

Yellow oil (89% yield); IR (KBr) 1680 (C=O), 1635 (C=N), 1544, 1529 (C=S), 1359, 1339 cm^−1^; ^1^H NMR (CDCl_3_, 400 MHz) δ (ppm) 8.01 (d, *J* = 7.6 Hz, 2H, Ar-H_phenyl_), 7.51 (t, *J* = 8.0 Hz, 2H, Ar-H_phenyl_), 7.38 (t, *J* = 7.6 Hz, 2H, Ar-H_phenyl_), 7.35 (t, *J* = 7.6 Hz, 1H, Ar-H_phenyl_), 7.14 (t, *J* = 7.6 Hz, 1H, Ar-H_phenyl_), 7.06 (d, *J* = 8.4 Hz, 2H, Ar-H_phenyl_), 2.61 (s, 3H, CH_3_, COCH_3_); ^13^C NMR (CDCl_3_, 100 MHz) δ (ppm) 189.5 (C=O), 155.9 (N=C-N), 151.0 (**C**-N_phenyl_), 146.8 (C=N-N), 138.7 (**C**-N_phenyl_), 129.7 (2 × CH_phenyl_), 128.8 (2 × CH_phenyl_), 127.3 (CH_phenyl_), 124.5 (CH_phenyl_), 122.9 (2 × CH_phenyl_), 120.3 (2 × CH_phenyl_), 24.9 (C(O)-**C**H_3_), 21.1 (Ar-**C**H_3_); HRMS (ESI^+^): *m*/*z* [M + H]^+^ calcd for C_16_H_14_N_3_OS: 296.0858; found: 296.0845.

**(*Z*)-1-(4-phenyl-5-(*p*-tolylimino)-4,5-dihydro-1,3,4-thiadiazol-2-yl)ethan-1-one (88)**. 

Yellow oil (93% yield); IR (KBr) 1687 (C=O), 1618 (C=N), 1566, 1505 (C=S), 1399, 1339 cm^−1^; ^1^H NMR (CDCl_3_, 400 MHz) δ (ppm) 8.00 (d, *J* = 8.4 Hz, 2H, Ar-H_phenyl_), 7.50 (t, *J* = 8.0 Hz, 2H, Ar-H_phenyl_), 7.36 (t, *J* = 8.0 Hz, 1H, Ar-H_phenyl_), 7.18 (d, *J* = 8.4 Hz, 2H, Ar-H_p-tolyl_), 6.96 (d, *J* = 8.4 Hz, 2H, Ar-H_p-tolyl_), 2.61 (s, 3H, CH_3_, COCH_3_), 2.35 (s, 3H, CH_3_, Ar-CH_3_); ^13^C NMR (CDCl_3_, 100 MHz) δ (ppm) 189.5 (C=O), 155.5 (N=C-N), 148.5 (C-N_p-tolyl_), 146.8 (C=N-N), 138.8 (**C**-N_phenyl_), 134.0 (Ar**C_q_**-Me), 130.2 (2 × CH_p-tolyl_), 128.8 (2 × CH_phenyl_), 127.2 (CH_phenyl_), 122.9 (2 × CH_phenyl_), 120.1 (2 × CH_p-tolyl_), 24.9 (C(O)-**C**H_3_), 20.9 (Ar-**C**H_3_); HRMS (ESI^+^): *m*/*z* [M + H]^+^ calcd for C_17_H_16_N_3_OS: 310.1014; found: 310.1003.

**(*Z*)-1-(5-(phenylimino)-4-(*p*-tolyl)-4,5-dihydro-1,3,4-thiadiazol-2-yl)ethan-1-one (89)**. 

Yellow oil (93% yield); IR (KBr) 1689 (C=O), 1625 (C=N), 1546, 1515 (C=S), 1379, 1349 cm^−1^; ^1^H NMR (CDCl_3_, 400 MHz) δ (ppm) 7.84 (d, *J* = 8.8 Hz, 2H, Ar-H_p-tolyl_), 7.37 (t, *J* = 8.0 Hz, 2H, Ar-H_phenyl_), 7.30 (d, *J* = 8.8 Hz, 2H, Ar-H_p-tolyl_), 7.13 (t, *J* = 7.6 Hz, 1H, Ar-H_phenyl_), 7.04 (d, *J* = 8.4 Hz, 2H, Ar-H_phenyl_), 2.60 (s, 3H, CH_3_, COCH_3_), 2.42 (s, 3H, CH_3_, Ar-CH_3_); ^13^C NMR (CDCl_3_, 100 MHz) δ (ppm) 189.5 (C=O), 156.1 (N=C-N), 151.1 (**C**-N_phenyl_), 146.5 (C=N-N), 137.4 (Ar**C_q_**-Me), 136.2 (C-N_p-tolyl_), 129.6 (2 × CH_p-tolyl_), 129.4 (2 × CH_phenyl_), 124.4 (CH_phenyl_), 123.1 (2 × CH_p-tolyl_), 120.4 (2 × CH_phenyl_), 24.9 (C(O)-**C**H_3_), 21.1 (Ar-**C**H_3_); HRMS (ESI^+^): *m*/*z* [M + H]^+^ calcd for C_17_H_16_N_3_OS: 310.1014; found: 310.1007.

**(*Z*)-1-(4-(*p*-tolyl)-5-(*p*-tolylimino)-4,5-dihydro-1,3,4-thiadiazol-2-yl)ethan-1-one (90)**. 

Bright yellow solid (90% yield); mp 139 °C; IR (KBr) 1689 (C=O), 1625 (C=N), 1602, 1530 (C=S), 1504, 1367, 1339, 1272, 1247, 1145, 1100, 1059, 1017, 946, 854, 814, 724, 679, 597, 551, 498 cm^−1^; ^1^H NMR (CDCl_3_, 400 MHz) δ (ppm) 7.80 (d, *J* = 8.4 Hz, 2H, Ar-H_p-tolyl_), 7.29 (d, *J* = 8.4 Hz, 2H, Ar-H_p-tolyl_), 7.16 (d, *J* = 8.0 Hz, 2H, Ar-H_p-tolyl_), 6.93 (d, *J* = 8.0 Hz, 2H, Ar-H_p-tolyl_), 2.59 (s, 3H, CH_3_, COCH_3_), 2.41 (s, 3H, CH_3_, Ar-CH_3_), 2.33 (s, 3H, CH_3_, Ar-CH_3_); ^13^C NMR (CDCl_3_, 100 MHz) δ (ppm) 189.6 (C=O), 155.9 (N=C-N), 148.5 (C-N_p-tolyl_), 146.6 (C=N-N), 137.4 (Ar**C_q_**-Me), 136.3 (**C**-N_p-tolyl_), 134.0 (Ar**C_q_**-Me), 130.2 (2 × CH_p-tolyl_), 129.5 (2 × CH_p-tolyl_), 123.2 (2 × CH_p-tolyl_), 120.2 (2 × CH_p-tolyl_), 24.9 (C(O)-**C**H_3_), 21.1 (Ar-**C**H_3_), 21.0 (Ar-**C**H_3_); HRMS (ESI^+^): *m*/*z* [M + H]^+^ calcd for C_18_H_18_N_3_OS: 324.1171; found: 324.1180.

**(*Z*)-1-(5-((4-ethylphenyl)imino)-4-(*p*-tolyl)-4,5-dihydro-1,3,4-thiadiazol-2-yl)ethan-1-one (91).**

Yellow solid (84% yield); mp 84 °C; IR (KBr) 1684 (C=O), 1624 (C=N), 1600, 1530 (C=S), 1504, 1363, 1291, 1278, 1244, 1148, 1055, 944, 856, 817, 725, 675, 597, 551 cm^−1^; ^1^H NMR (CDCl_3_, 400 MHz) δ (ppm) 7.85 (d, *J* = 8.4 Hz, 2H, Ar-H_p-tolyl_), 7.30 (d, *J* = 8.4 Hz, 2H, Ar-H_p-tolyl_), 7.21 (d, *J* = 8.0 Hz, 2H, Ar-H_p-ethylphenyl_), 6.98 (d, *J* = 8.0 Hz, 2H, Ar-H_p-ethylphenyl_), 2.65 (q, *J* = 7.6 Hz, 2H, **CH_2_**CH_3_), 2.60 (s, 3H, CH_3_, COCH_3_), 2.42 (s, 3H, CH_3_, Ar-CH_3_), 1.26 (t, *J* = 7.6 Hz, 3H, CH_2_**CH_3_**); ^13^C NMR (CDCl_3_, 100 MHz) δ (ppm) 189.5 (C=O), 155.6 (N=C-N), 148.7 (C-N_p-ethylphenyl_), 146.4 (C=N-N), 140.3 (Ar**C_q_**-Et), 137.3 (Ar**C_q_**-Me), 136.3 (**C**-N_p-tolyl_), 129.4 (2 × CH_p-tolyl_), 128.9 (2 × CH_p-ethylphenyl_), 123.0 (2 × CH_p-tolyl_), 120.2 (2 × CH_p-ethylphenyl_), 28.3 (**C**H_2_CH_3_), 24.9 (C(O)-**C**H_3_), 21.1 (Ar-**C**H_3_), 15.6 (CH_2_**C**H_3_); HRMS (ESI^+^): *m*/*z* [M + H]^+^ calcd for C_19_H_20_N_3_OS: 338.1327; found: 338.1319.

**(*Z*)-1-(4-(naphthalen-1-yl)-5-(naphthalen-1-ylimino)-4,5-dihydro-1,3,4-thiadiazol-2-yl)ethan-1-one (92).**

Brown solid (77% yield); IR (KBr) 1686 (C=O), 1620 (C=N), 1571, 1509 (C=S), 1394, 1275, 1181, 1141, 1065, 1013, 943, 584 cm^−1^; ^1^H NMR (CDCl_3_, 400 MHz) δ (ppm) 8.07 (d, *J* = 8.8 Hz, 1H, Ar-H_naphthyl_), 8.06 (d, *J* = 8.4 Hz, 1H, Ar-H_naphthyl_), 7.91–7.85 (m, 2H, Ar-H_naphthyl_), 7.78 (d, *J* = 8.0 Hz, 1H, Ar-H_naphthyl_), 7.74 (d, *J* = 8.4 Hz, 1H, Ar-H_naphthyl_), 7.69–7.58 (m, 4H, Ar-H_naphthyl_), 7.47–7.40 (m, 2H, Ar-H_naphthyl_), 7.32–7.27 (m, 1H, Ar-H_naphthyl_), 7.17 (d, *J* = 7.6 Hz, 1H, Ar-H_naphthyl_), 2.57 (s, 3H, CH_3_); ^13^C NMR (CDCl_3_, 100 MHz) δ (ppm) 189.7 (C=O), 157.4 (N=C-N), 147.6 (C=N-N), 147.5 (C-N_naphthyl_), 134.6 (**C**-N_naphthyl_), 134.5 (**C**_naphthyl_), 134.3 (**C**_naphthyl_), 130.4 (CH_naphthyl_), 129.7 (**C**_naphthyl_), 128.6 (CH_naphthyl_), 127.9 (**C**_naphthyl_), 127.7 (CH_naphthyl_), 127.4 (CH_naphthyl_), 126.8 (CH_naphthyl_), 126.3 (CH_naphthyl_), 126.2 (CH_naphthyl_), 126.0 (CH_naphthyl_), 125.5 (CH_naphthyl_), 125.3 (CH_naphthyl_), 124.5 (CH_naphthyl_), 123.3 (CH_naphthyl_), 122.7 (CH_naphthyl_), 113.2 (CH_naphthyl_), 25.0 (C(O)-**C**H_3_); HRMS (ESI^+^): *m*/*z* [M + H]^+^ calcd for C_24_H_18_N_3_OS: 396.1171; found: 396.1160.

## 4. Conclusions

In conclusion, the triethylamine mediated reaction of *N*-arylcyanothioformamide **27** and hydrazonoyl chloride **29** for the synthesis of 5-arylimino-1,3,4-thiadiazoles at room temperature has been successfully demonstrated. The reaction accepts a range of functional groups on both reactants including the typical halides, alkoxides, esters, ketones, cyano, nitro, thiomethyl, naphthyl, trifluoromethyl, and alkyl functions and affords a broad scope of products. Proof of the *Z*-stereochemistry and molecular structure of the isolated regioisomer was obtained by single-crystal X-ray diffraction analysis. Density functional theory calculations at the B3LYP-D4/def2-TZVP level in implicit solvent (ethanol) were carried out to support the suggested mechanism and experimental observations in reference to the stereo- and regiochemical outcome. It is expected that the current synthetic technique could become candidate for the rapid synthesis of 5-arylimino-1,3,4-thiadiazole libraries because it is exclusively stereo- and regioselective, practical, scalable, uses a simple reagent system, and offers mild reaction conditions.

## Figures and Tables

**Table 1:** Calculated atomic charges (in units of *e*) for reactant **29** zwitterion computed at the B3LYP-D4/def2-TZVP level of theory in implicit ethanol as solvent. A ball-and-stick representation (grey color = C atoms, blue = N, red = O, white = H) with atom labels is shown.

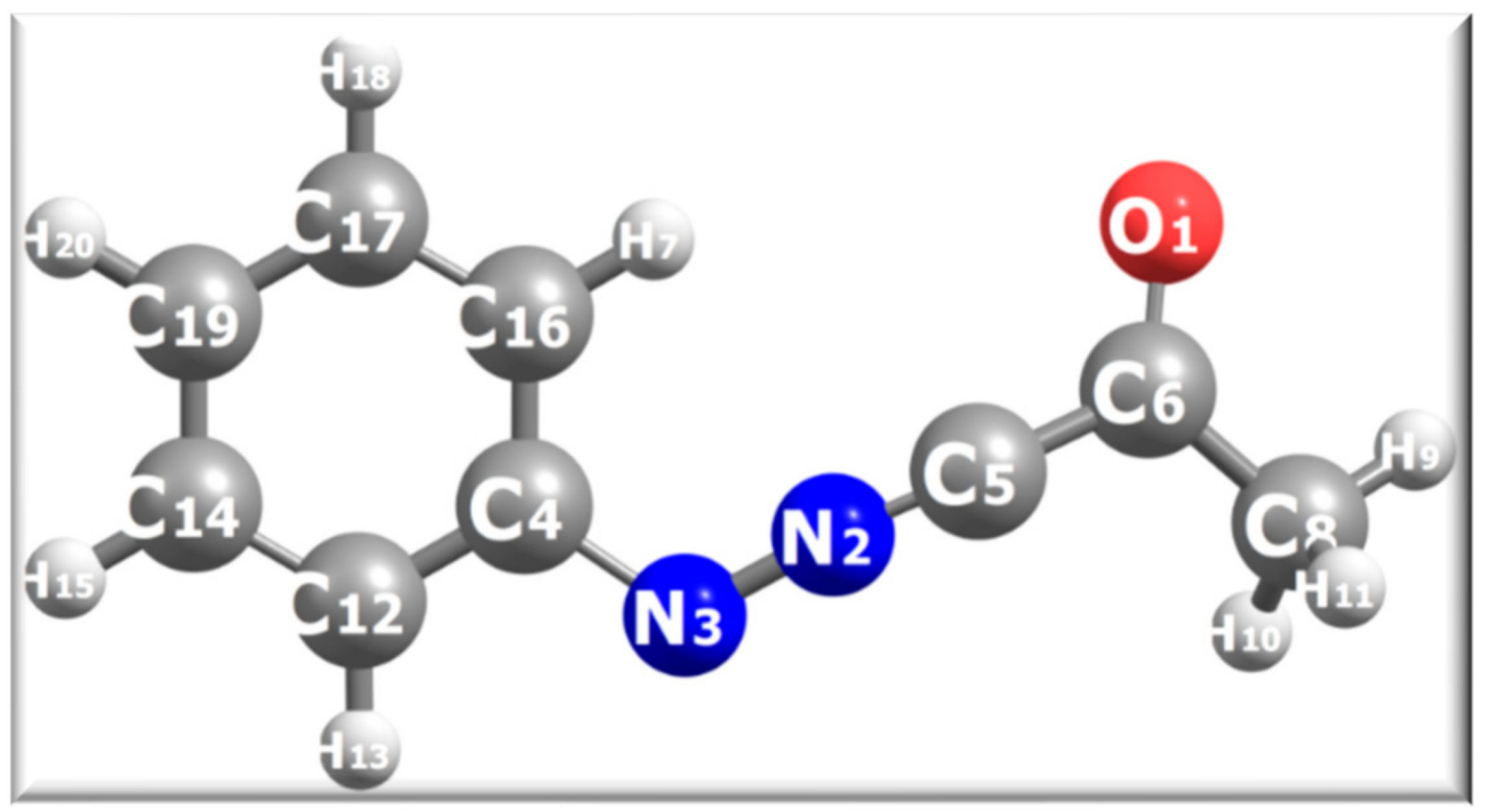					
Atom Labels in Reactant 29 (azo Zwitterion)	Mulliken	Löwdin	NPA	Bader	ADCH
O1	−0.401	0.068	−0.574	−1.167	−0.392
N2	0.242	0.245	0.007	−0.876	0.278
N3	−0.273	0.050	−0.262	−0.422	−0.322
C4	0.262	−0.232	0.084	0.321	0.086
C5	−0.060	−0.081	0.167	0.742	−0.100
C6	0.294	−0.366	0.484	1.096	0.234
H7	0.155	0.156	0.230	0.068	0.182
C8	−0.356	−0.233	−0.691	−0.024	−0.224
H9	0.145	0.139	0.241	0.064	0.124
H10	0.159	0.149	0.248	0.070	0.140
H11	0.165	0.154	0.257	0.075	0.134
C12	−0.257	−0.114	−0.217	−0.037	−0.113
H13	0.139	0.155	0.231	0.073	0.133
C14	−0.118	−0.141	−0.208	−0.039	−0.122
H15	0.151	0.153	0.226	0.061	0.144
C16	−0.253	−0.119	−0.248	−0.042	−0.217
C17	−0.129	−0.139	−0.202	−0.034	−0.123
H18	0.153	0.153	0.226	0.059	0.144
C19	−0.167	−0.147	−0.225	−0.047	−0.126
H20	0.150	0.151	0.225	0.057	0.141

**Table 2:** Calculated atomic charges for the deprotonated form of reactant **27** computed at the B3LYP-D4/def2-TZVP level of theory in implicit ethanol as solvent. A ball-and-stick model (grey color = C atoms, blue = N, yellow = S, white = H) of the molecule with atom labels is shown.

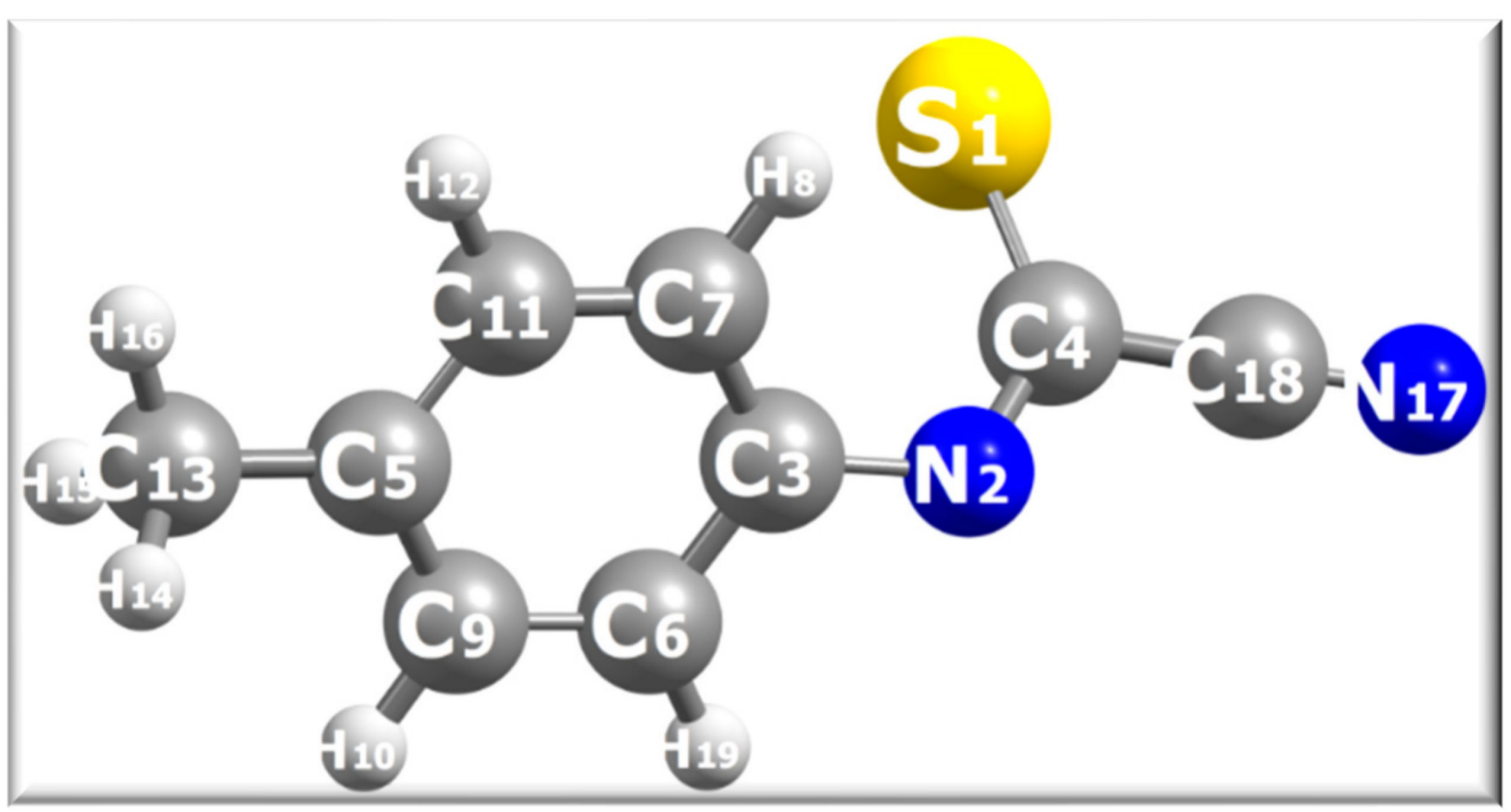					
Atom Labels in Reactant 27	Mulliken	Löwdin	NPA	Bader	ADCH
S1	−0.581	−0.030	−0.402	−0.318	−0.666
N2	−0.289	0.105	−0.490	−1.165	−0.571
C3	0.236	−0.238	0.110	0.345	0.085
C4	0.218	−0.622	−0.041	0.523	0.571
C5	0.133	−0.187	−0.042	−0.023	−0.031
C6	−0.272	−0.132	−0.236	−0.057	−0.109
C7	−0.248	−0.131	−0.231	−0.049	−0.282
H8	0.123	0.149	0.222	0.051	0.148
C9	−0.200	−0.129	−0.214	−0.059	−0.172
H10	0.133	0.151	0.215	0.041	0.138
C11	−0.208	−0.134	−0.218	−0.056	−0.051
H12	0.135	0.150	0.215	0.039	0.134
C13	−0.383	−0.260	−0.620	0.001	−0.246
H14	0.130	0.130	0.217	0.019	0.098
H15	0.130	0.129	0.217	0.018	0.098
H16	0.131	0.128	0.215	0.015	0.097
N17	−0.113	−0.026	−0.429	−1.307	−0.450
C18	−0.196	−0.203	0.294	0.934	0.090
H19	0.120	0.149	0.220	0.048	0.121

**Table 3:** Global reactivity descriptors of the individual reactants calculated at the B3YLP-D4/def2-TZVP in implicit ethanol as solvent. Energy units are in eV. The E_HOMO_ for the reference compound tetracyanoethylene (TCE) is −8.736 eV, computed at the same level.

Global Reactivity Descriptors	Reactant 29 (Zwitterion)	Reactant 27 Anion
E_HOMO_ (eV)	−6.035	−5.2828
E_LUMO_ (eV)	−2.101	−1.124
HOMO-LUMO gap (eV) or chemical hardness η = E_LUMO_ − E_HOMO_	3.934	4.1588
chemical potential (μ, eV)	−4.068	−3.203
global electrophilicity (ω, eV)	2.103	1.234
global nucleophilicity (*N,* eV)	2.702	3.454

## Data Availability

Samples of the compounds are available from the authors.

## Supplementary Materials

The following supporting information can be downloaded at: https://www.mdpi.com/article/10.3390/ijms24043759/s1.
