# Supplementary material for: First X-ray Crystal Structure Characterization, Computational Studies, and Improved Synthetic Route to the Bioactive 5-Arylimino-1,3,4-thiadiazole Derivatives
Source: Int J Mol Sci. 2023 Feb 13;24(4):3759. doi: 10.3390/ijms24043759 (PMC9965731; doi:10.3390/ijms24043759)
Supplement: Supplementary file 1 [file ijms-24-03759-s001.zip › ijms-2186185-supplementary.pdf]

Supplementary Materials for

First X-Ray Crystal Structure Characterization Computations Studies, and Improved Synthetic Route to the Bioactive 5-Arylimino-1,3,4-Thiadiazole Derivatives

Ziad Moussa 1,*, Alejandro Perez Paz 1, Zaher M. A. Judeh 2, Ahmed Alzamly 1, Haythem A. Saadeh 3,
Basim H. Asghar 4, Sara Alsaedi 1, Bayan Masoud 1, Salama Almeqbaali 1, Saeda Estwani 1, Amna Aljaberi 1,
Munirah M. Al-Rooqi 4 and Saleh A. Ahmed 4,5,*

1 Department of Chemistry, College of Science, United Arab Emirates University,
Al Ain P.O. Box 15551, United Arab Emirates; aperez@uaeu.ac.ae (A.P.P.); ahmed.alzamly@uaeu.ac.ae (A.A.); 201809448@uaeu.ac.ae (S.A.); 201950253@uaeu.ac.ae (B.M.); 201908280@uaeu.ac.ae (S.A.); 201950248@uaeu.ac.ae (S.E.); 201705734@uaeu.ac.ae (A.A.)
2 School of Chemistry, Chemical Engineering and Biotechnology, Nanyang Technological University,
62 Nanyang Drive, N1.2–B1-14, Singapore 637459, Singapore; zaher@ntu.edu.sg
3 Department of Chemistry, School of Science, The University of Jordan, Amman 11942, Jordan;
h.saadeh@ju.edu.jo
4 Department of Chemistry, Faculty of Applied Sciences, Umm Al-Qura University,
Makkah 21955, Saudi Arabia; bhasghar@uqu.edu.sa (B.H.A.); munira900@hotmail.com (M.M.A.-R.)
5 Department of Chemistry, Faculty of Science, Assiut University, 71516 Assiut, Egypt
* Correspondence: zmoussa@uaeu.ac.ae (Z.M.); saahmed@uqu.edu.sa (S.A.A.); Tel.: +971-509695321 (Z.M.)

**Table of Contents**	S2
1D and 2D NMR spectra of **29a-m**	S3-S89
1D and 2D NMR spectra of **31-92**	S90-S458
Crystal data for (Z)-N-(2,3-difluorophenyl)-2-oxopropanehydrazonoyl chloride	S459-S462
Crystal data for (4-ethoxyphenyl)carbamothioyl cyanide	S463-S466
Crystal data for (Z)-1-(5-((3-fluorophenyl)imino)-4-(4-iodophenyl)-4,5-dihydro-1,3,4-thiadiazol-2-yl)ethan-1-one	S467-S471
Crystal data for (Z)-1-(4-phenyl-5-(p-tolylimino)-4,5-dihydro-1,3,4-thiadiazol-2-yl)ethan-1-one	S472-S476
Computational Details	S477-S479

General Information. Reactions were conducted with magnetic stirring in air-dried glassware. All reagents and reaction solvents were used as received without any further purification. Analytical thin-layer chromatography (TLC) was used to follow the progress of reactions and was carried out on precoated silica gel plates (HSGF 254) and visualized under UV irradiation (254 nm). ^{1}H and ^{13}C NMR spectra were recorded in DMSO-d_6 or $CDCl_3$ on a Bruker DPX 300 and 75 MHz NMR spectrometer and on a Varian 400 and 100 MHz NMR spectrometer. The NMR chemical shifts (δ) are reported in parts per million (ppm) relative to the residual solvent peak (^{1}H-NMR δ 7.26 for $CDCl_3$, δ 2.50 for DMSO-d_6; ^{13}C-NMR δ 77.0 for $CDCl_3$, δ 39.52 for DMSO-d_6).

^{1}H NMR ($CDCl_3$) spectrum of (Z)-2-oxo-N-phenylpropanehydrazonoyl chloride (**29a**)

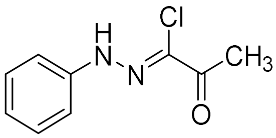

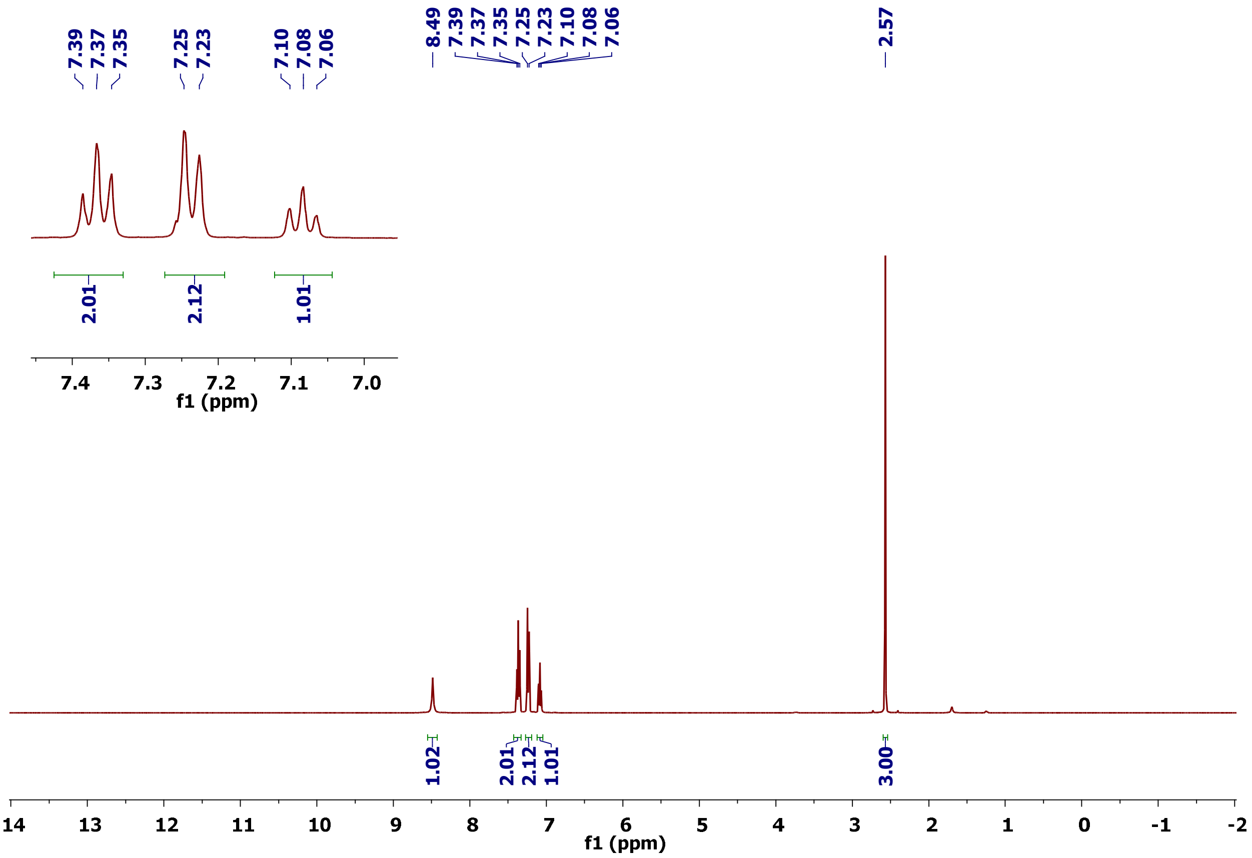

^{13}C NMR ($CDCl_3$) spectrum of (Z)-2-oxo-N-phenylpropanehydrazonoyl chloride

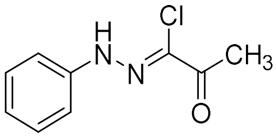

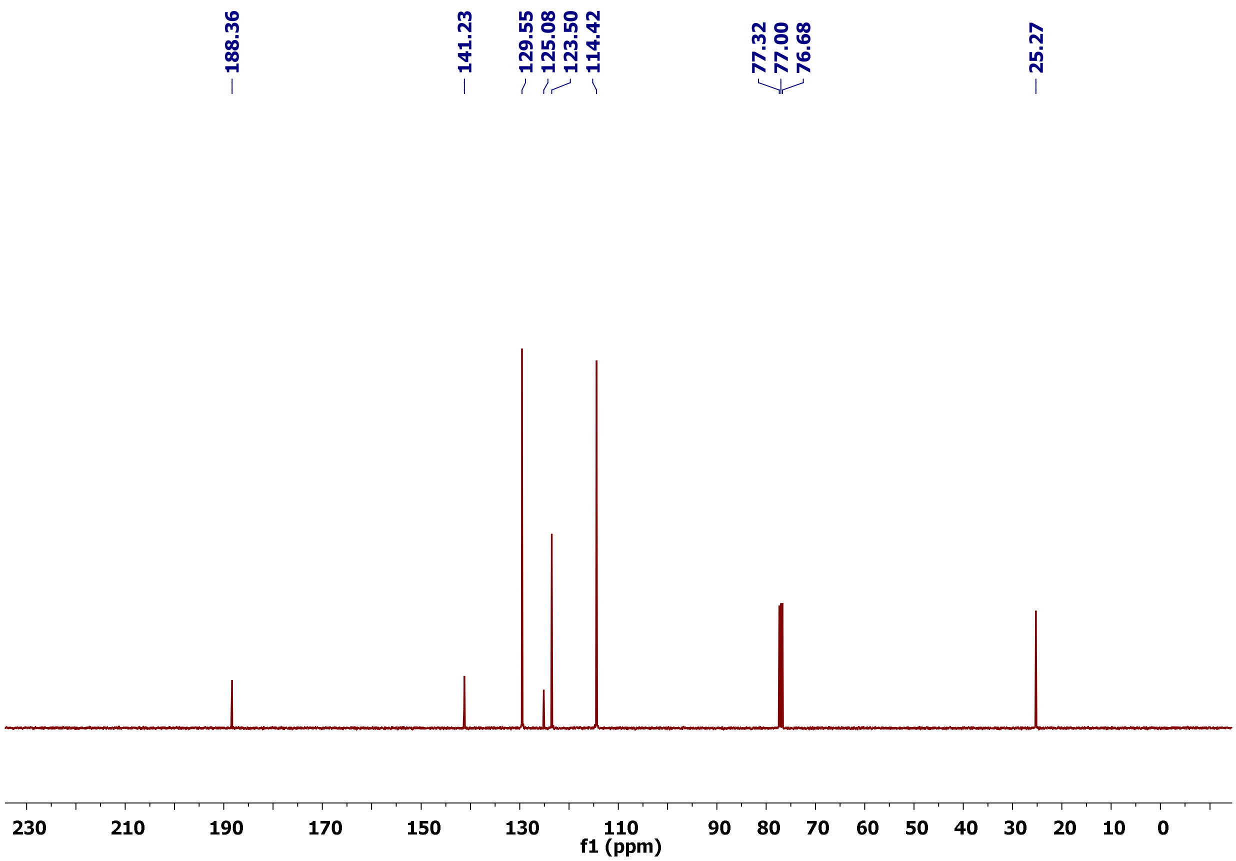

^{13}C CRAPT NMR ($CDCl_3$) spectrum of (Z)-2-oxo-N-phenylpropanehydrazonoyl chloride

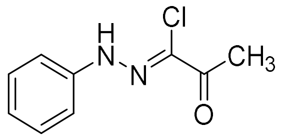

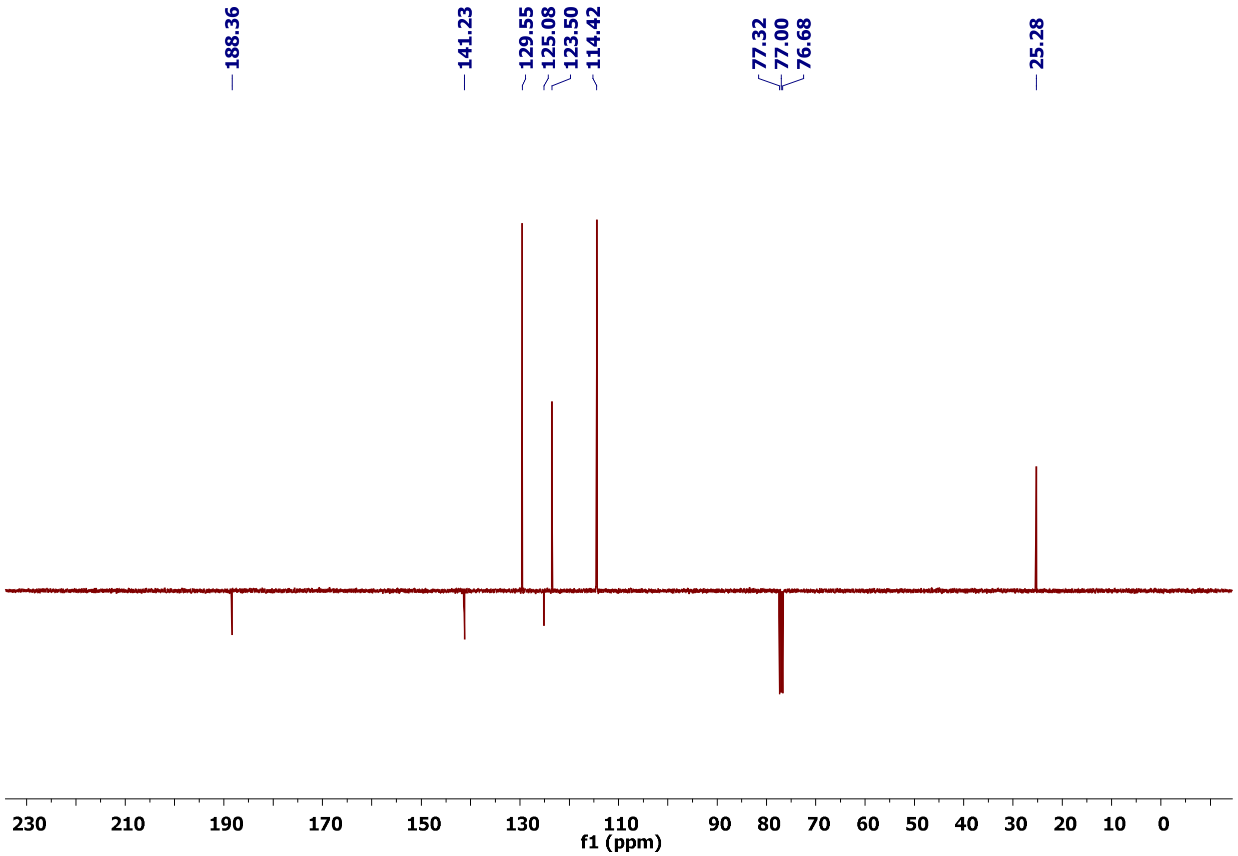

^1H-^1H-gDQCOSY NMR ($CDCl_3$) spectrum of (Z)-2-oxo-N-phenylpropanehydrazonoyl chloride

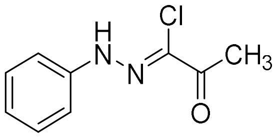

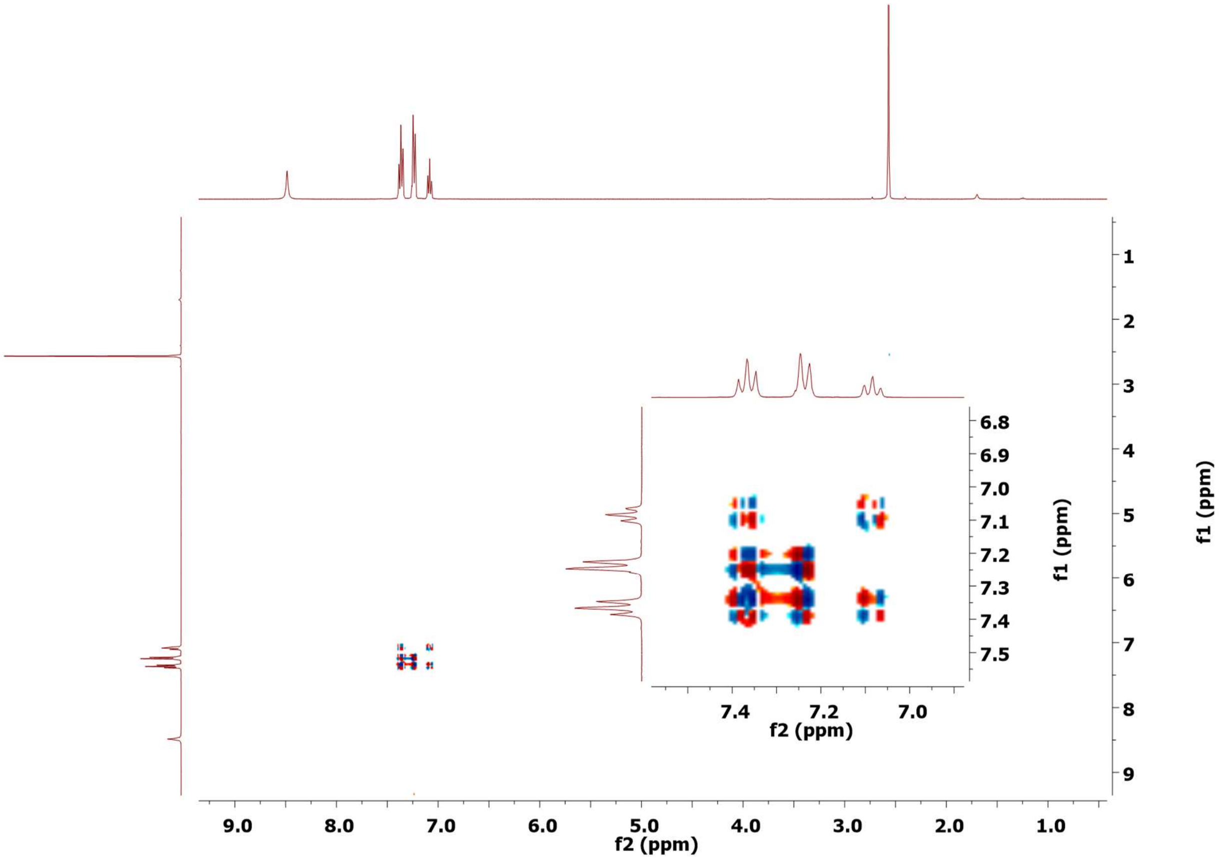

^{1}H-^{13}C-HSQC NMR ($CDCl_3$) spectrum of (Z)-2-oxo-N-phenylpropanehydrazonoyl chloride

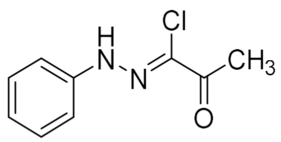

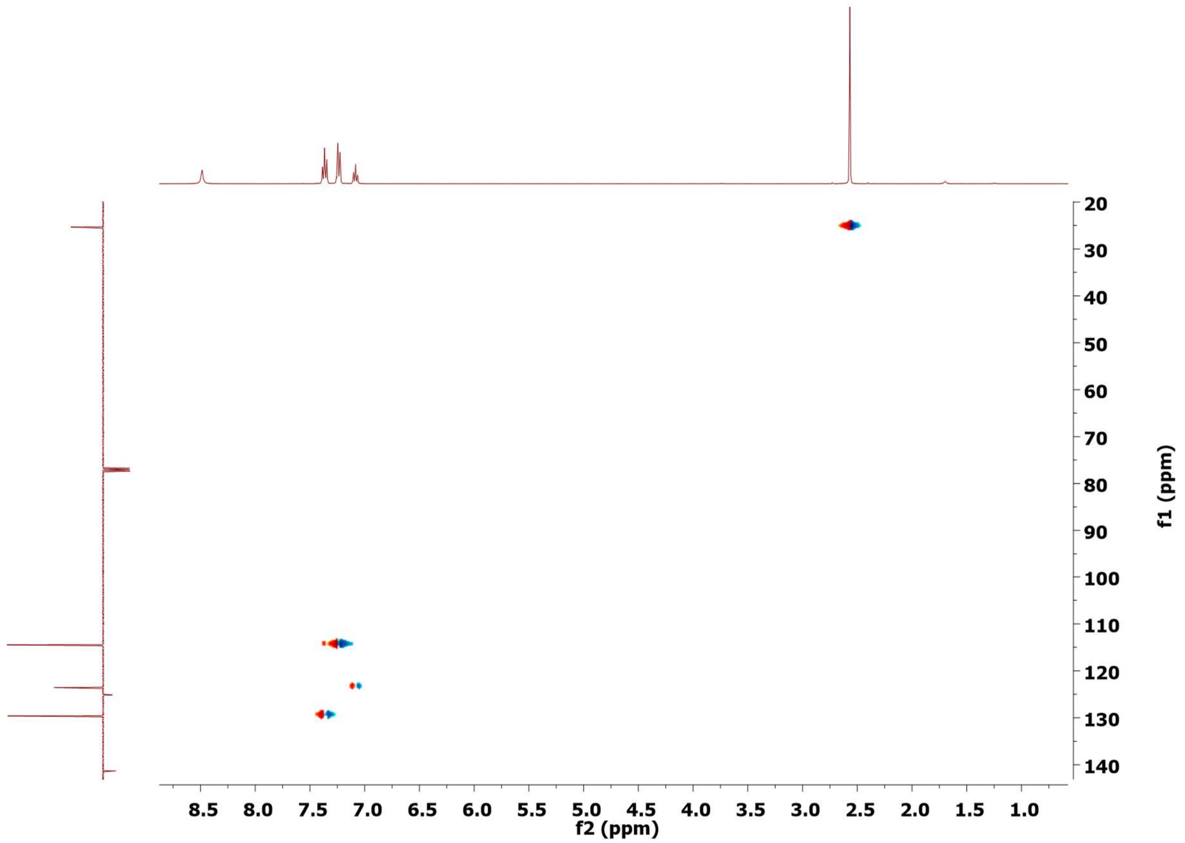

^{1}H-^{13}C-gHMBC NMR ($CDCl_3$) spectrum of (Z)-2-oxo-N-phenylpropanehydrazonoyl chloride

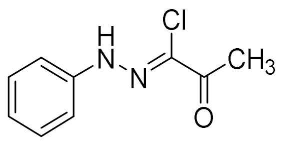

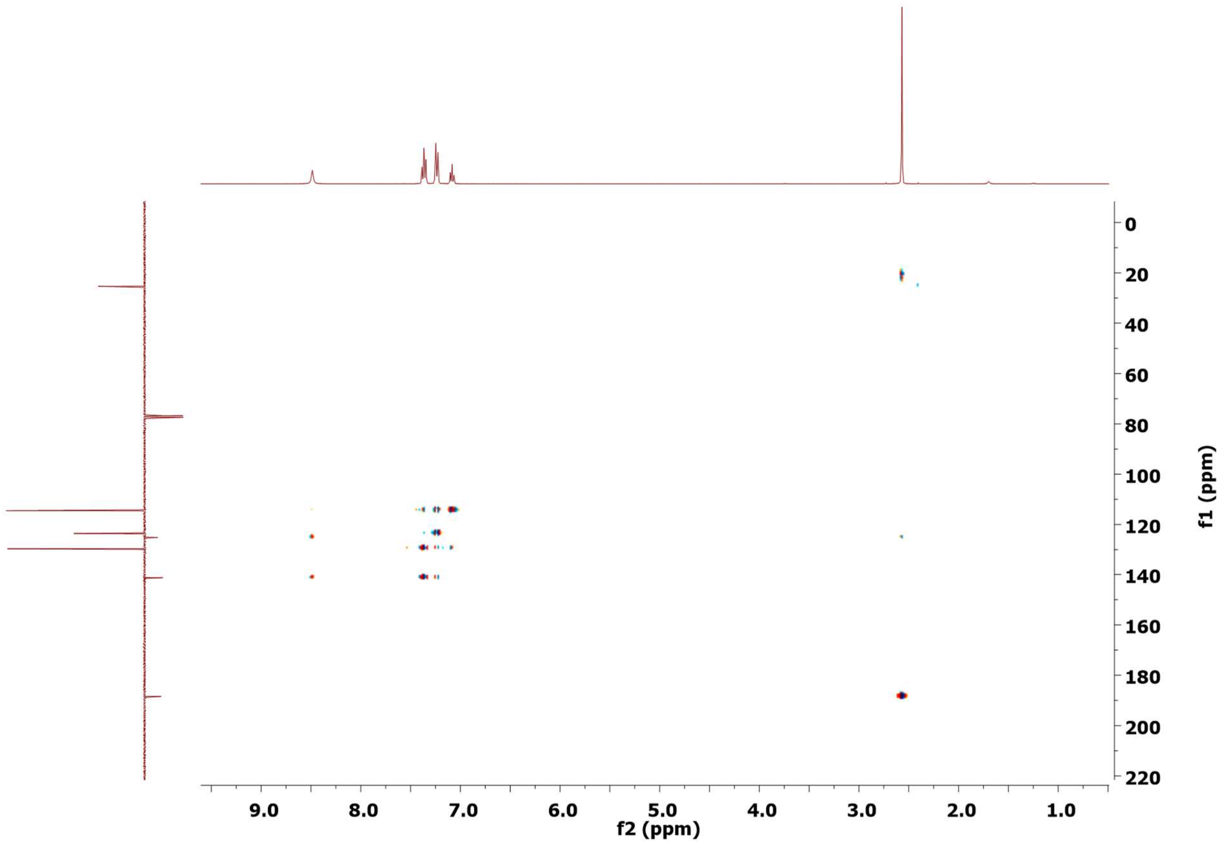

IR spectrum of (Z)-2-oxo-N-phenylpropanehydrazonoyl chloride

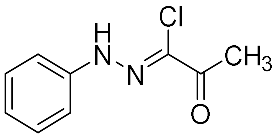

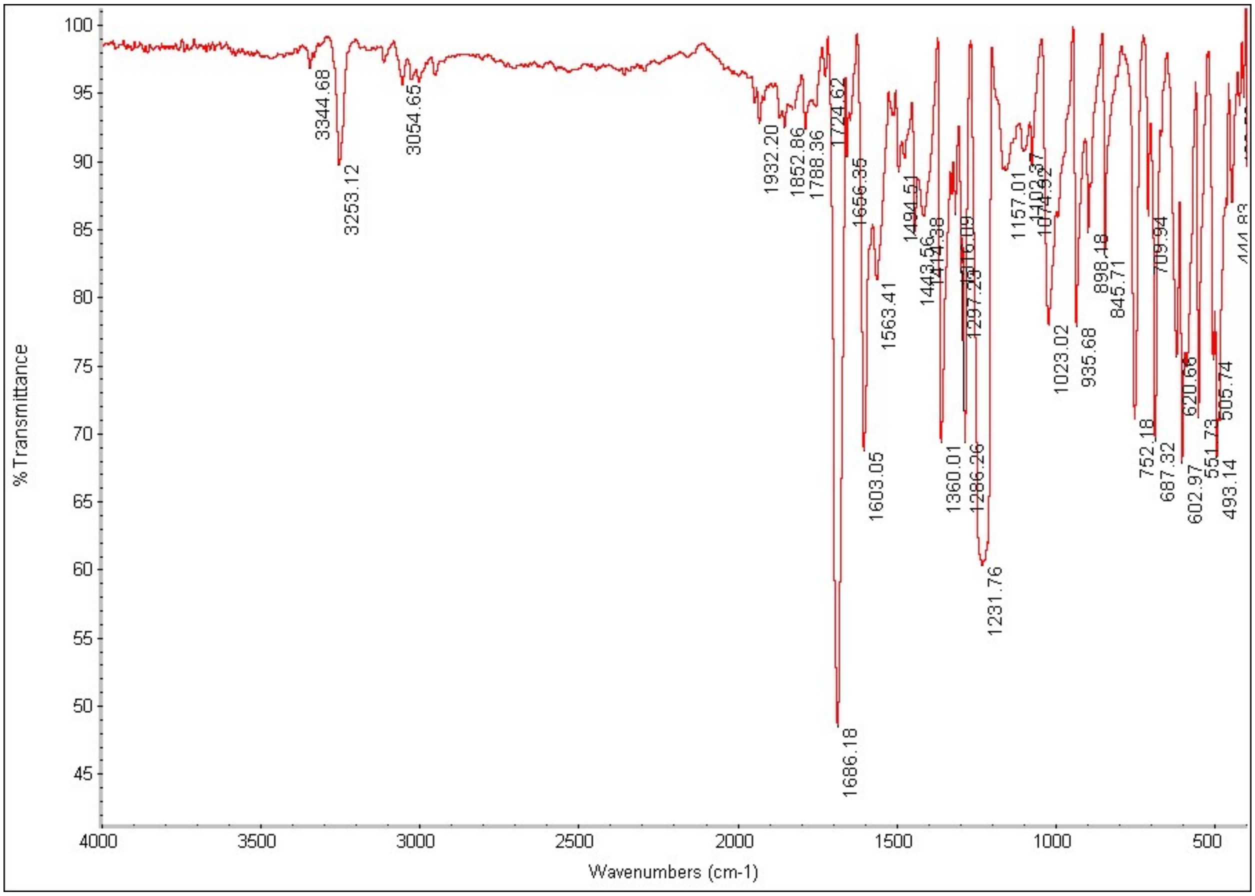

^{1}H NMR ($CDCl_3$) spectrum of (Z)-2-oxo-N-(p-tolyl)propanehydrazonoyl chloride (**29b**)

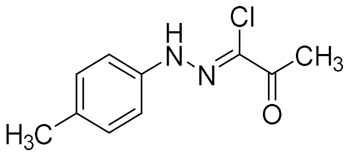

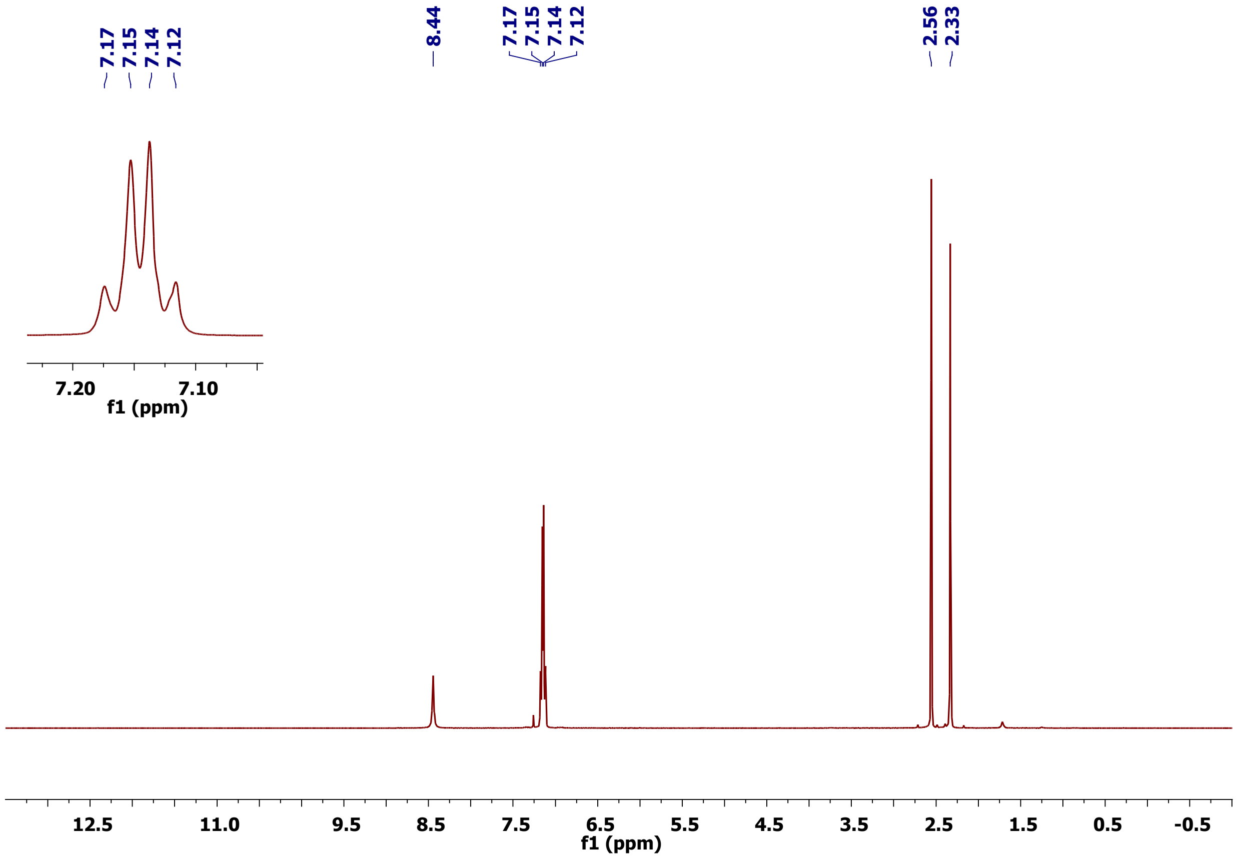

^{13}C NMR ($CDCl_3$) spectrum of (Z)-2-oxo-N-(p-tolyl)propanehydrazonoyl chloride

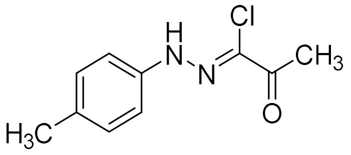

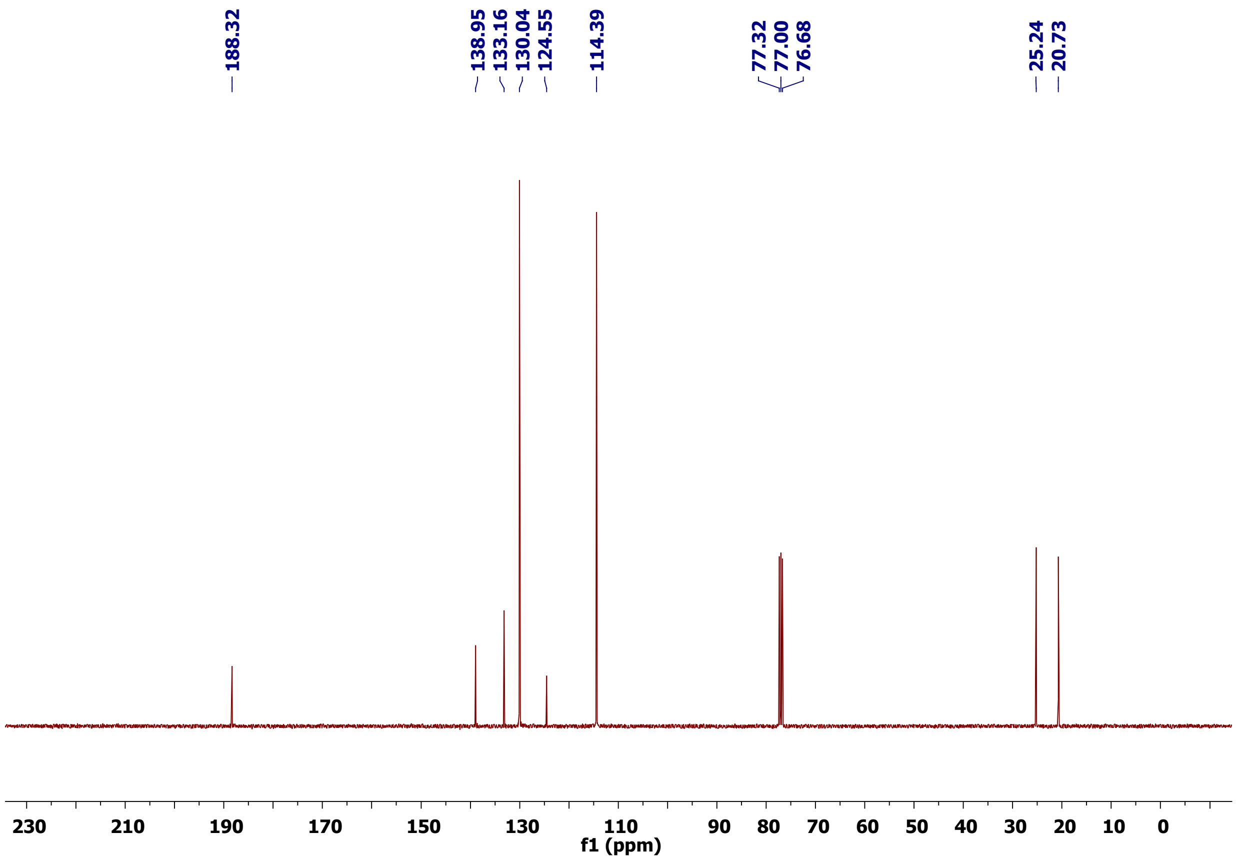

^{13}C CRAPT NMR ($CDCl_3$) spectrum of (Z)-2-oxo-N-(p-tolyl)propanehydrazonoyl chloride

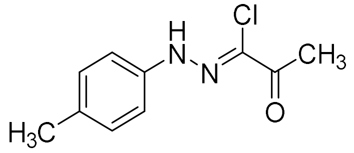

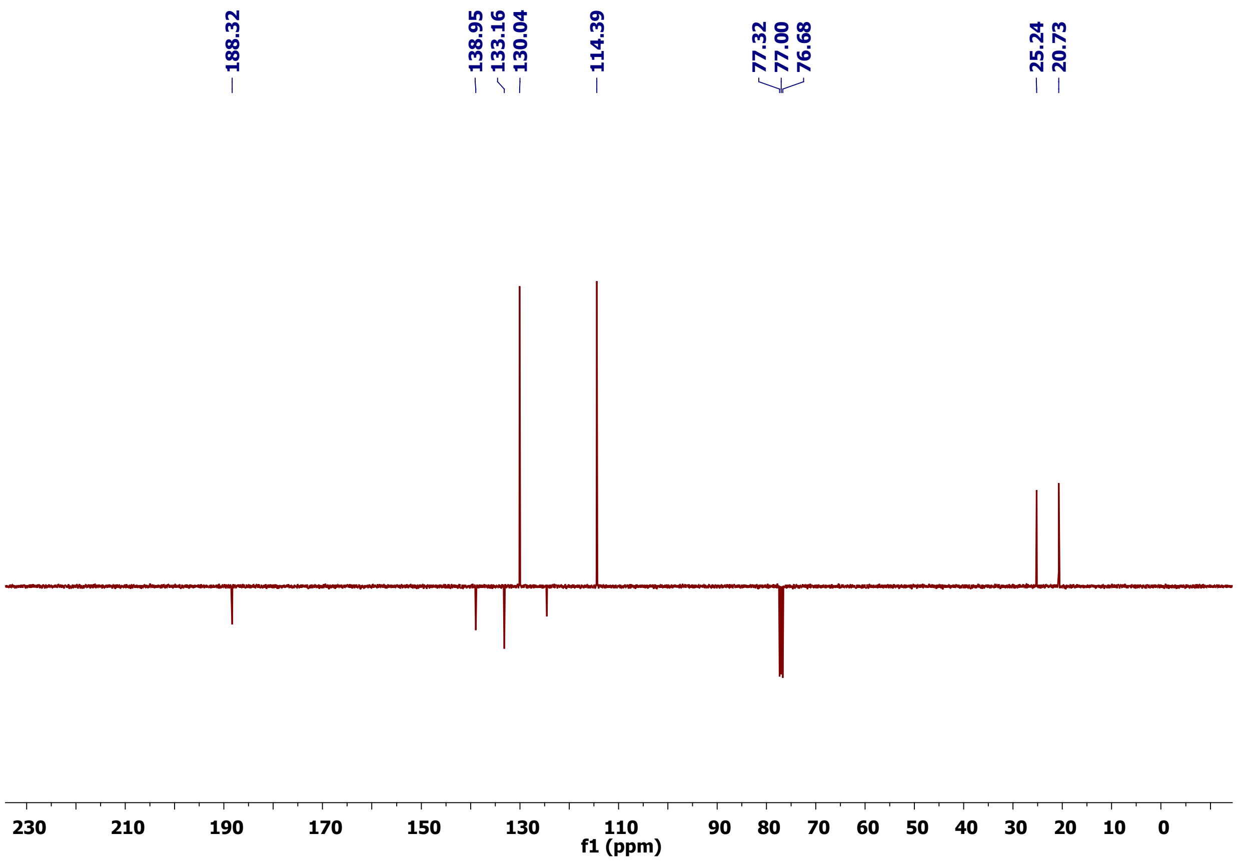

^{1}H-^{1}H-gDQCOSY NMR ($CDCl_3$) spectrum of (Z)-2-oxo-N-(p-tolyl)propanehydrazonoyl chloride

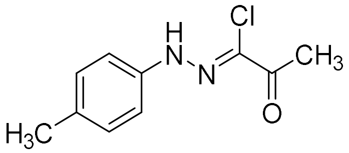

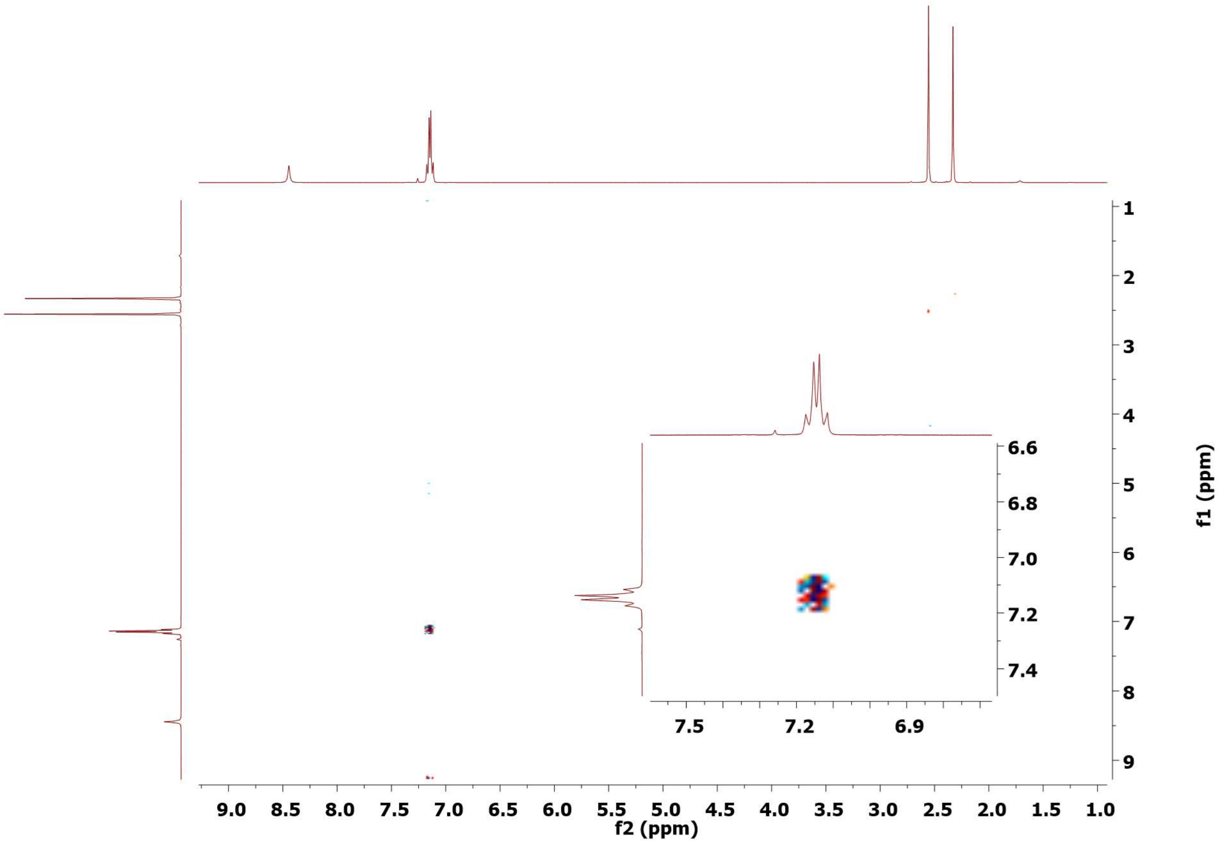

^{1}H-^{13}C-HSQC NMR ($CDCl_3$) spectrum of (Z)-2-oxo-N-(p-tolyl)propanehydrazonoyl chloride

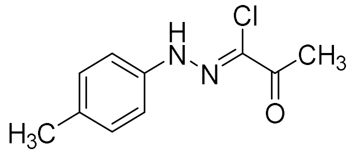

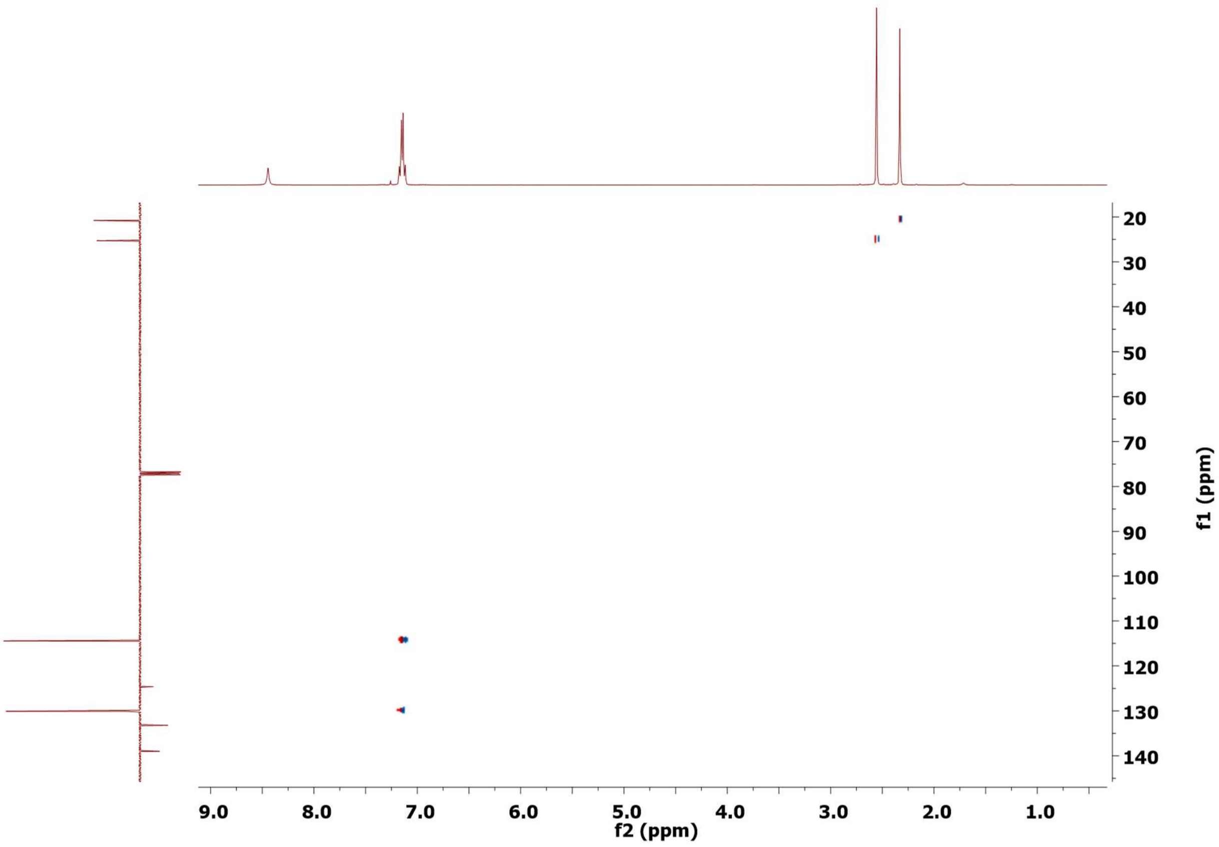

^{1}H-^{13}C-gHMBC NMR ($CDCl_3$) spectrum of (Z)-2-oxo-N-(p-tolyl)propanehydrazonoyl chloride

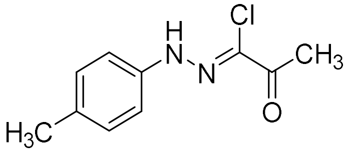

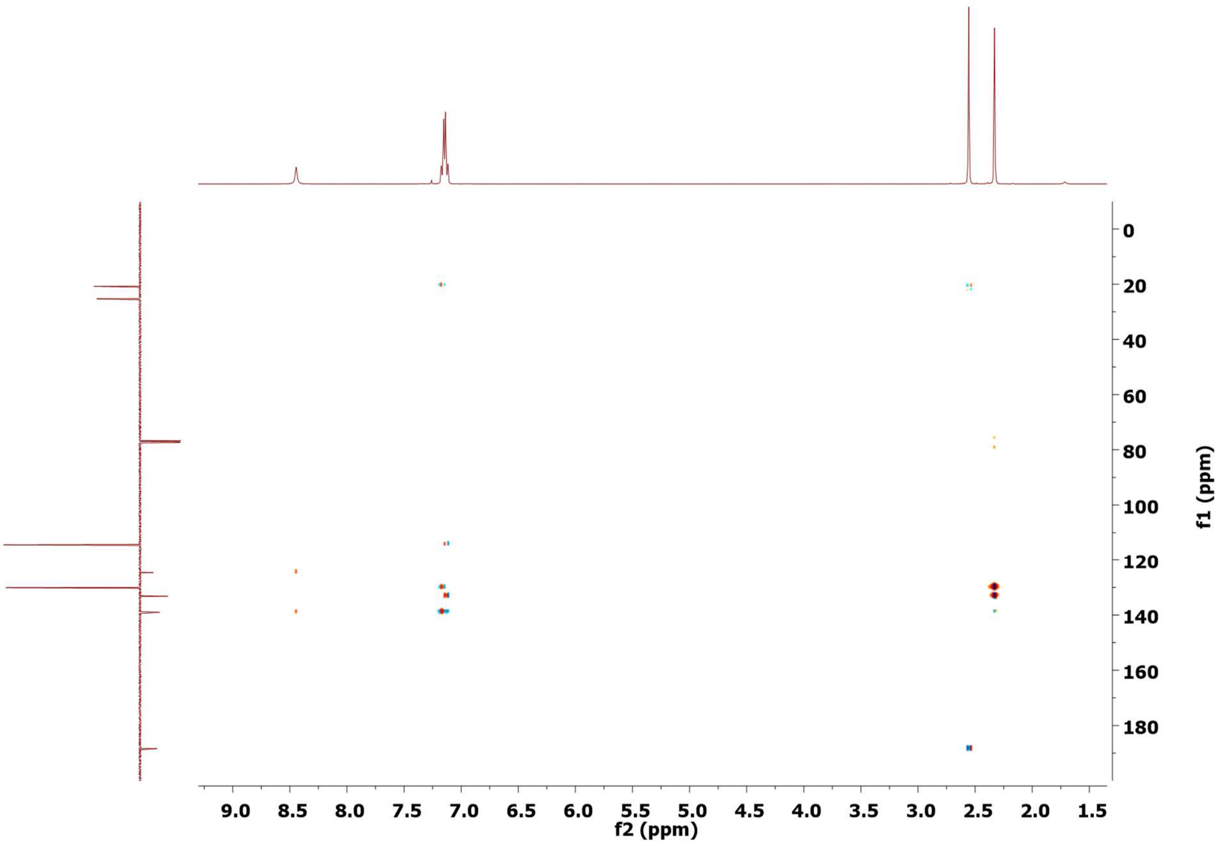

IR spectrum of (Z)-2-oxo-N-(p-tolyl)propanehydrazonoyl chloride

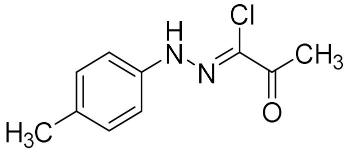

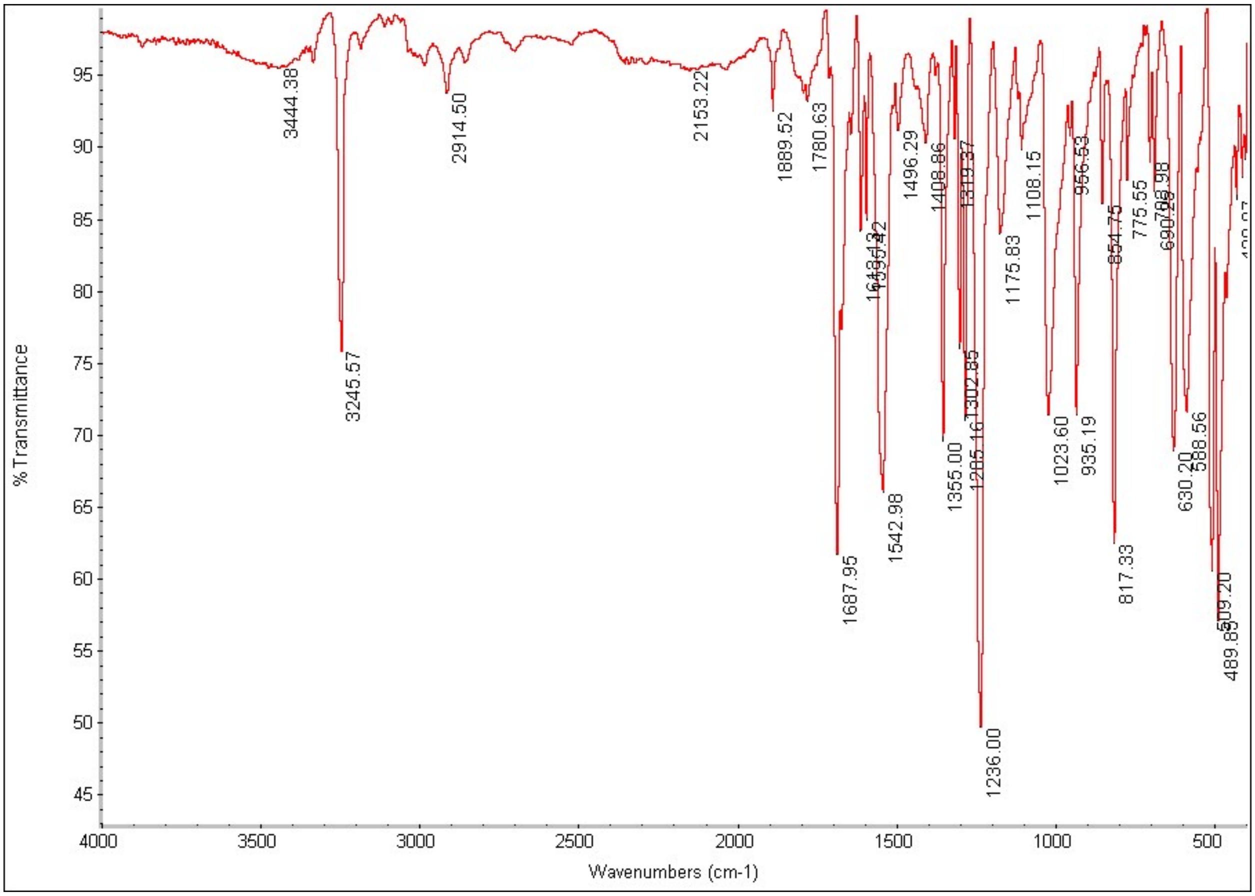

^{1}H NMR (DMSO-d_6) spectrum of (Z)-N-(4-chlorophenyl)-2-oxopropanehydrazonoyl chloride (**29c**)

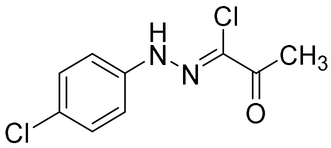

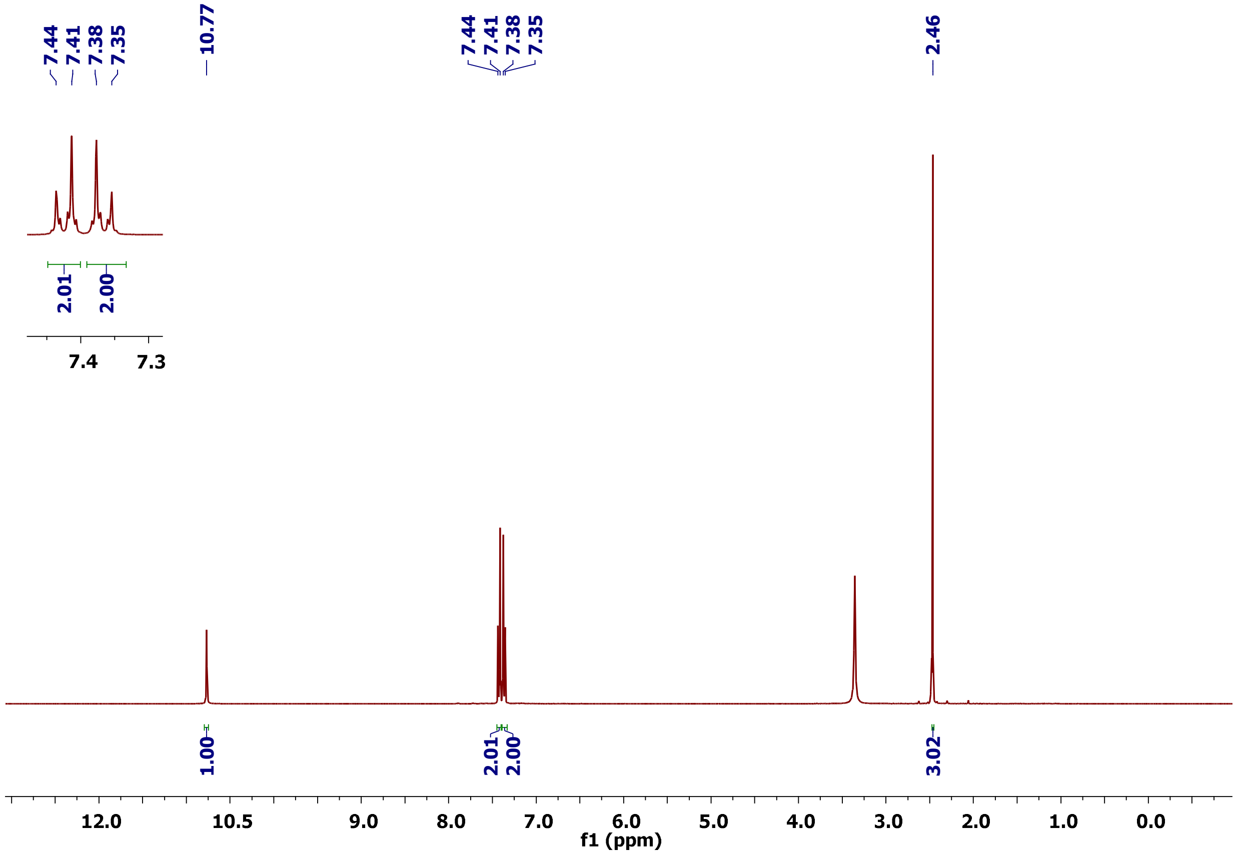

^{13}C NMR (DMSO-d_6) spectrum of (Z)-N-(4-chlorophenyl)-2-oxopropanehydrazonoyl chloride

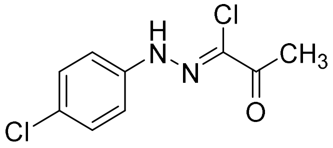

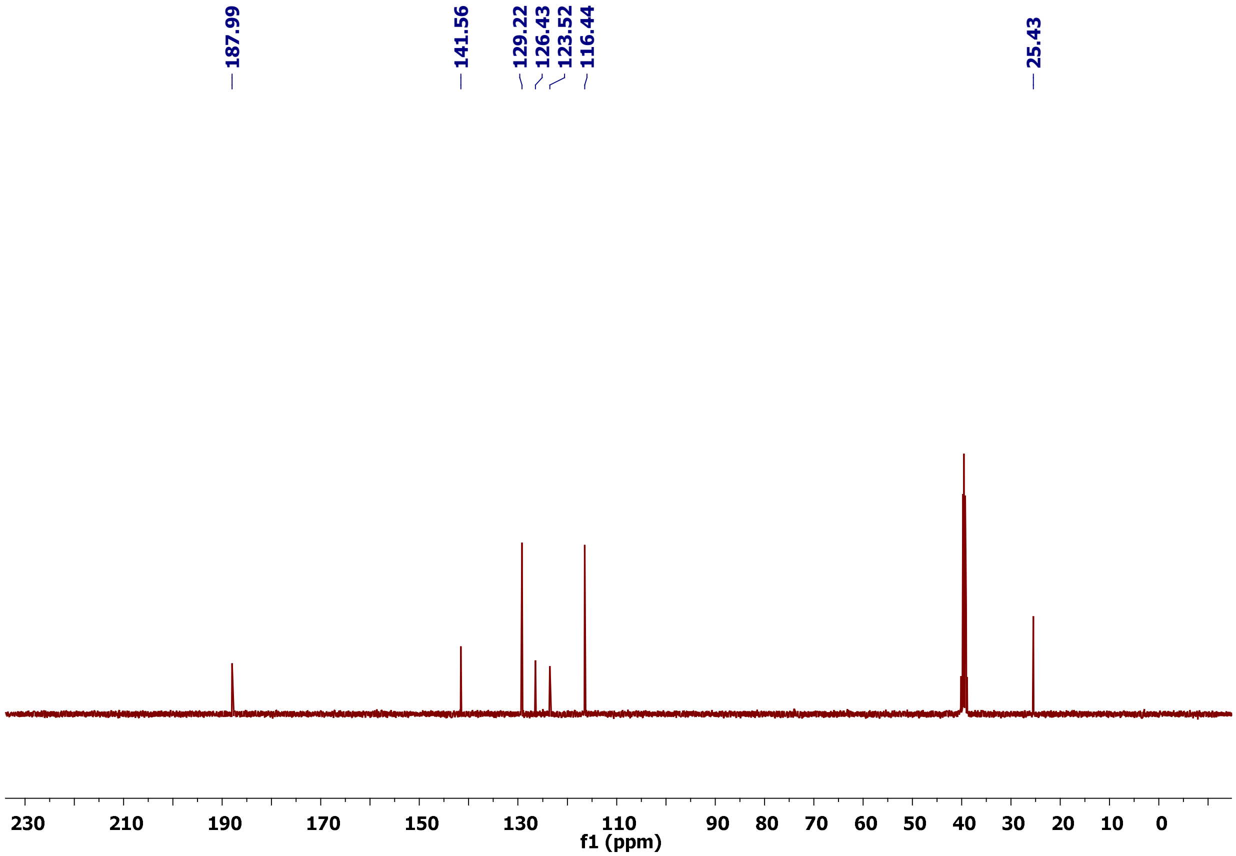

^{13}C CRAPT NMR (DMSO-d_6) spectrum of (Z)-N-(4-chlorophenyl)-2-oxopropanehydrazonoyl chloride

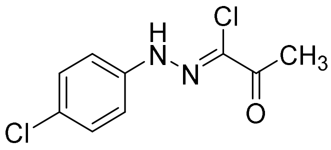

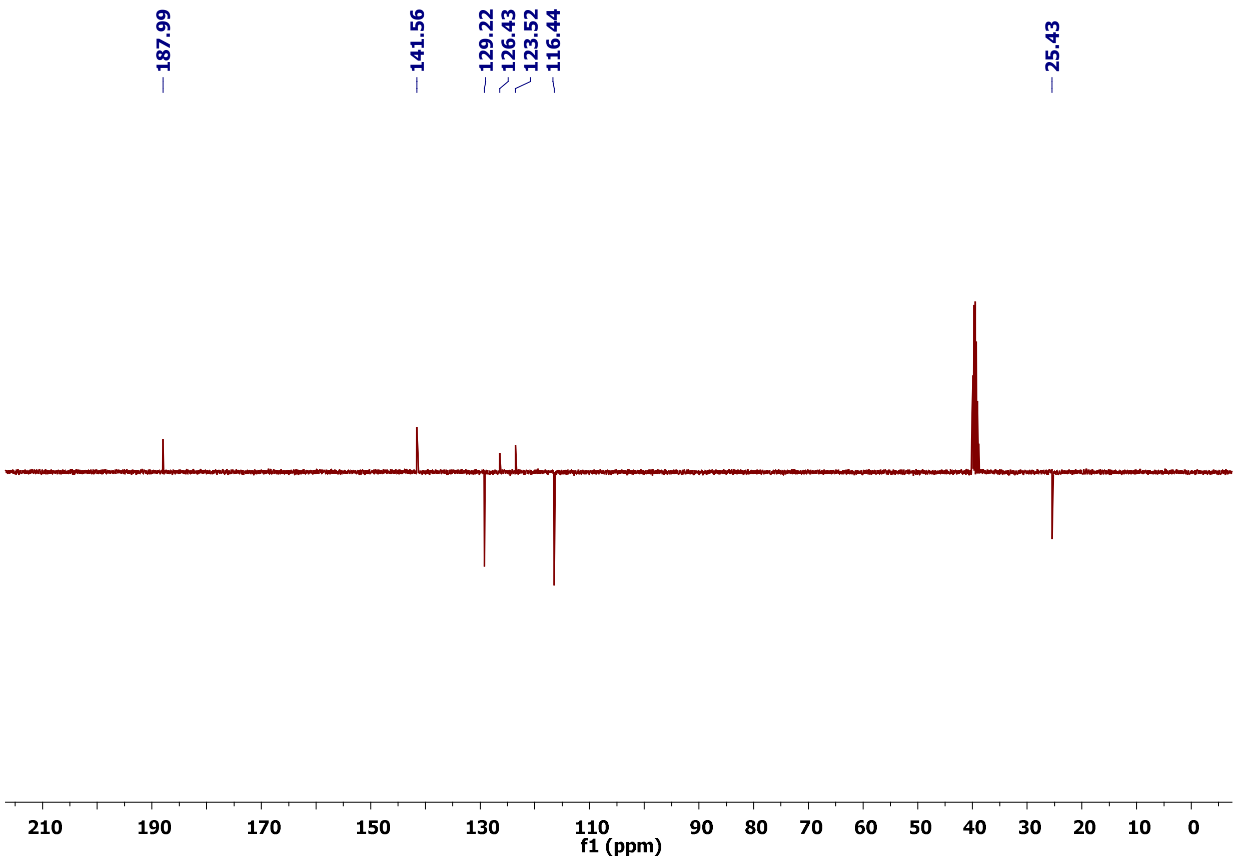

IR spectrum of (Z)-N-(4-chlorophenyl)-2-oxopropanehydrazonoyl chloride

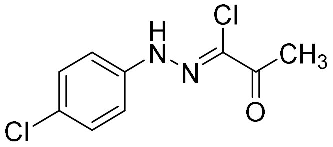

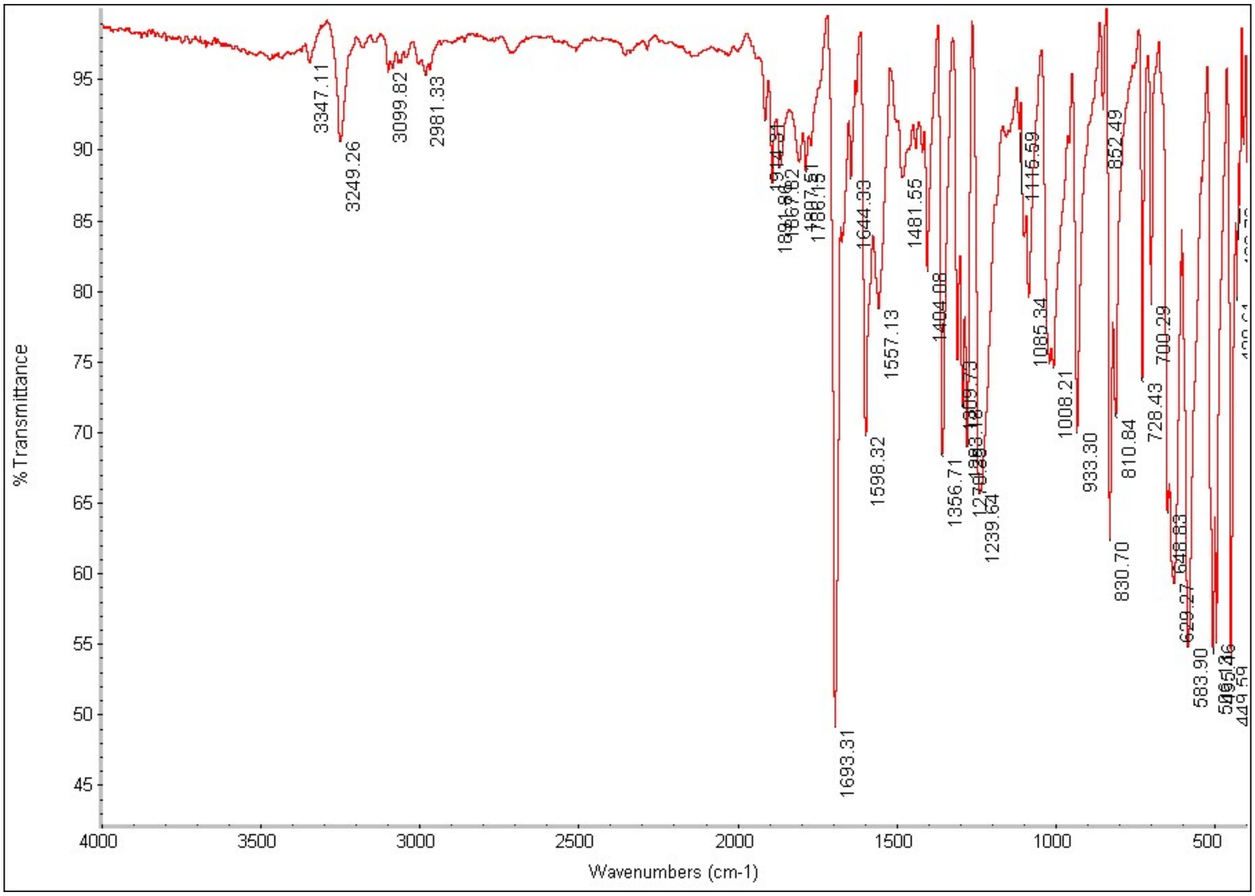

^{1}H NMR ($CDCl_3$) spectrum of (Z)-N-(4-bromophenyl)-2-oxopropanehydrazonoyl chloride (**29d**)

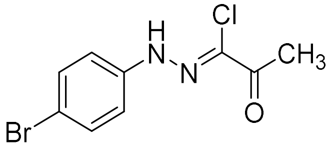

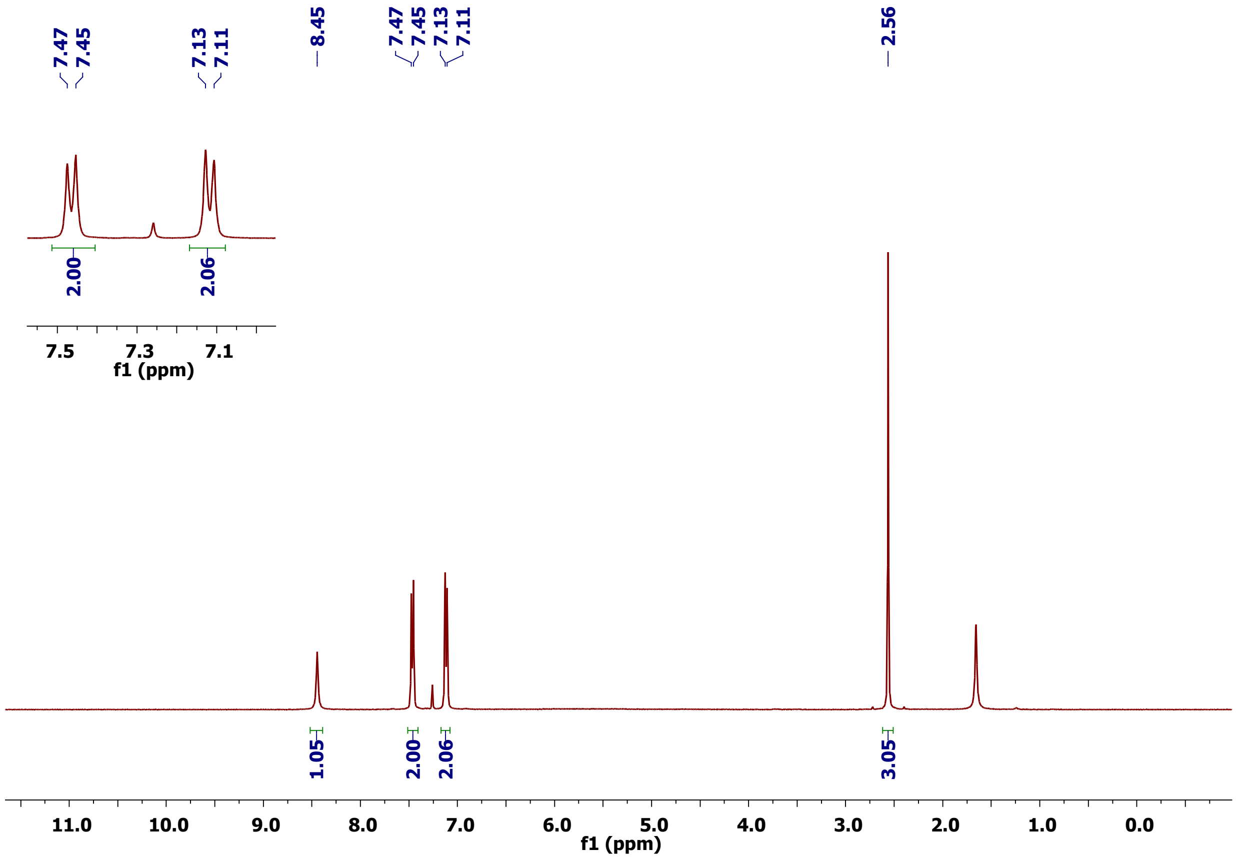

^{13}C NMR ($CDCl_3$) spectrum of (Z)-N-(4-bromophenyl)-2-oxopropanehydrazonoyl chloride

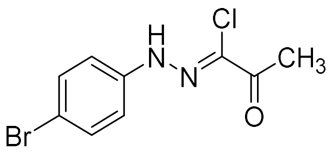

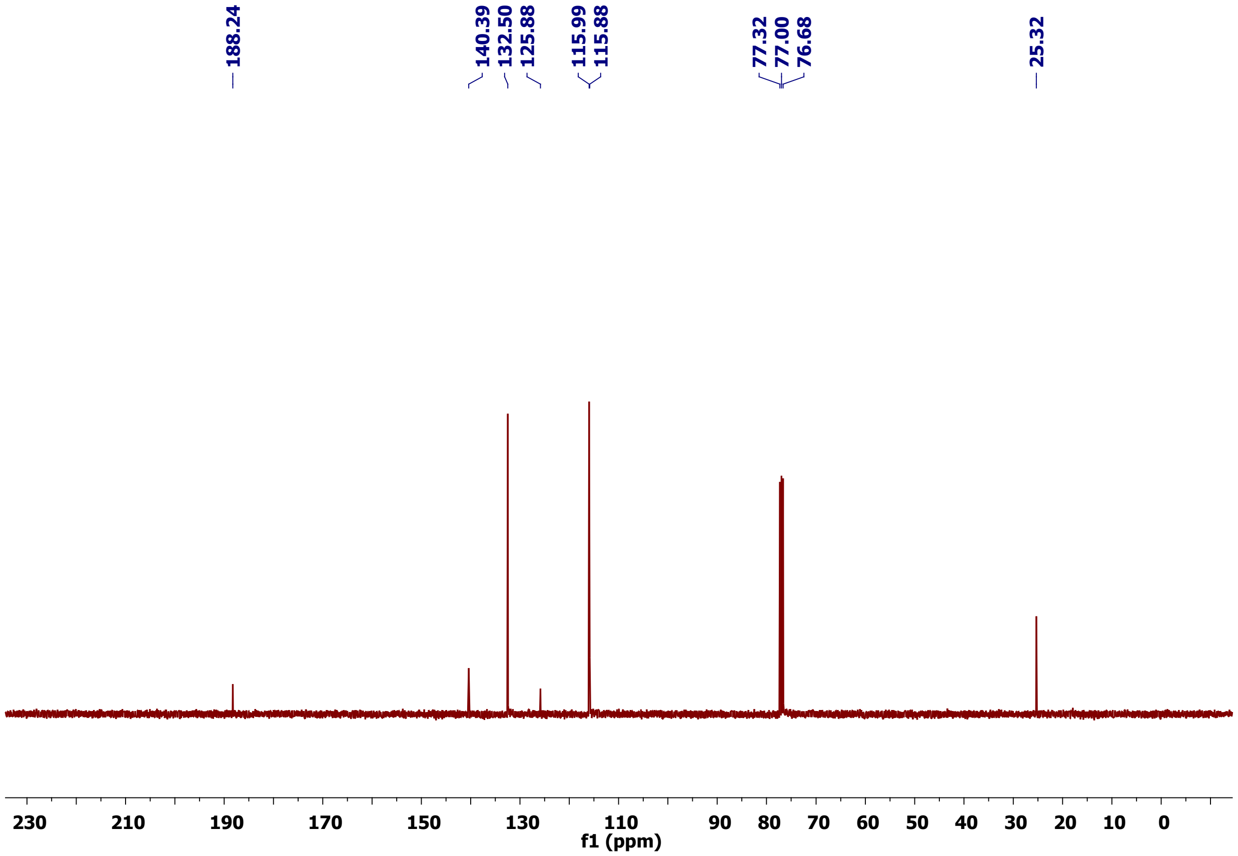

^{13}C CRAPT NMR ($CDCl_3$) spectrum of (Z)-N-(4-bromophenyl)-2-oxopropanehydrazonoyl chloride

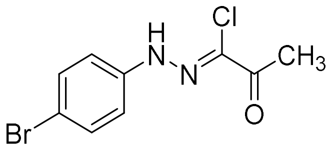

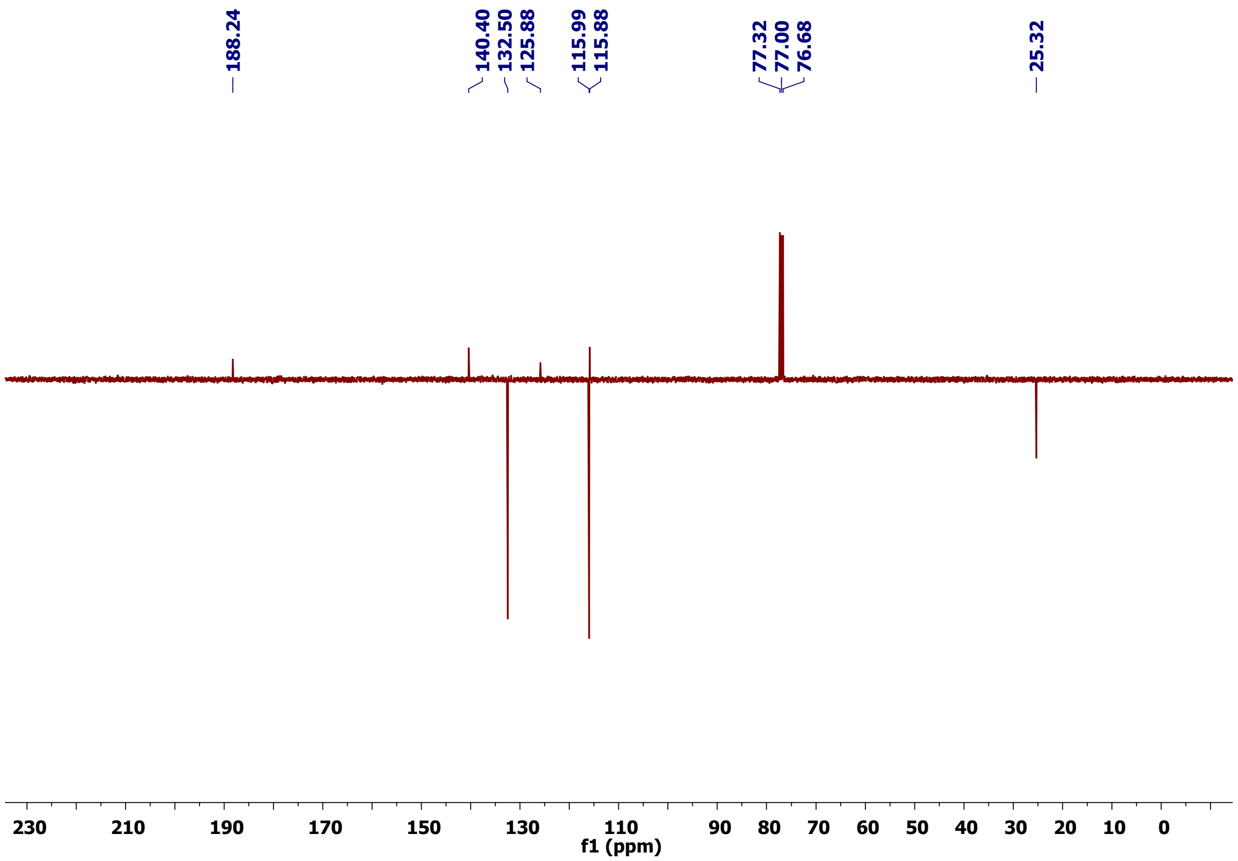

^1H-^{13}C-HSQC NMR ($CDCl_3$) spectrum of (Z)-N-(4-bromophenyl)-2-oxopropanehydrazonoyl chloride

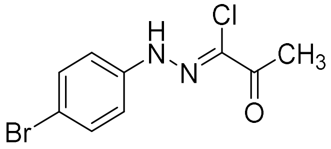

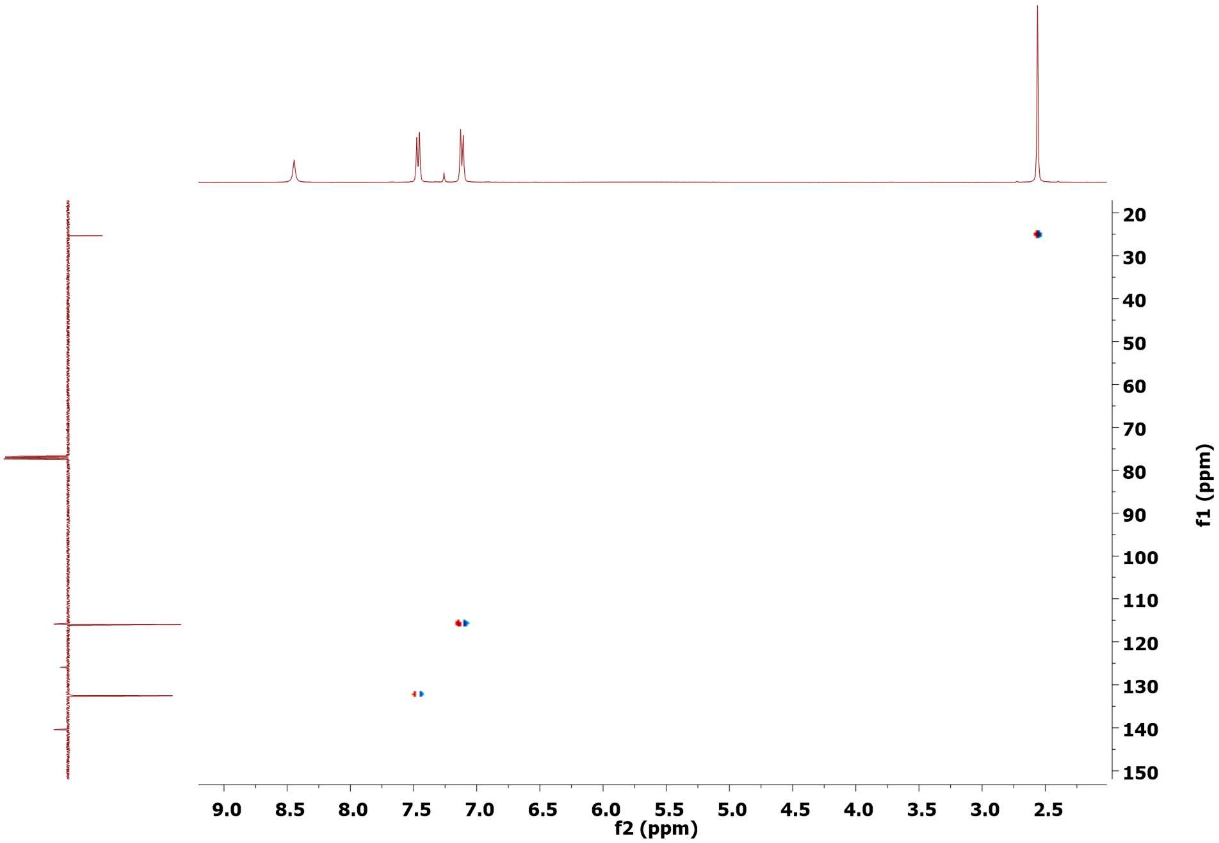

^{1}H-^{13}C-gHMBC NMR ($CDCl_3$) spectrum of (Z)-N-(4-bromophenyl)-2-oxopropanehydrazonoyl chloride

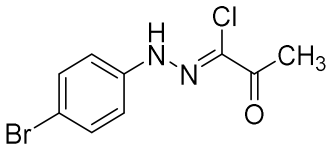

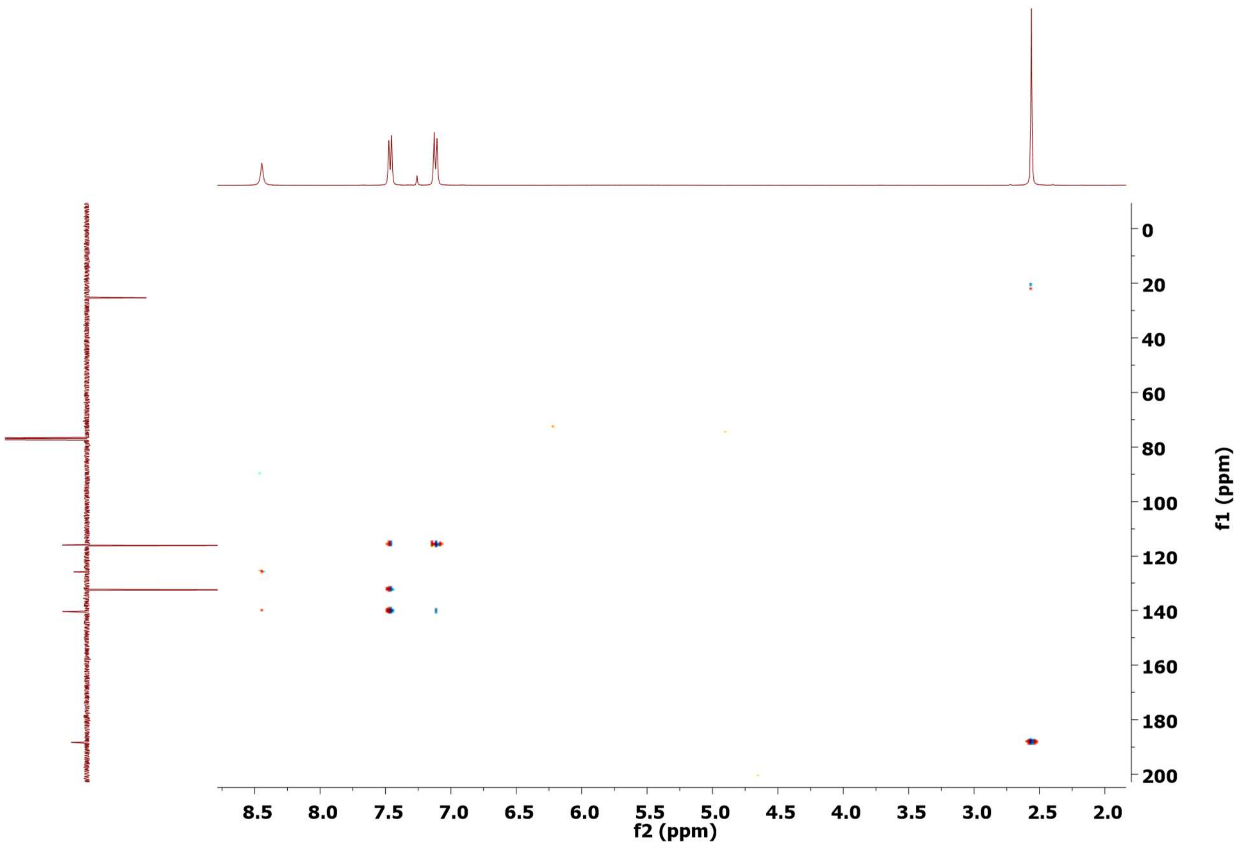

IR spectrum of (Z)-N-(4-bromophenyl)-2-oxopropanehydrazonoyl chloride

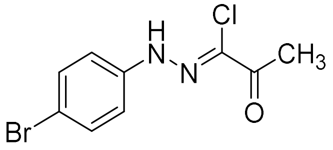

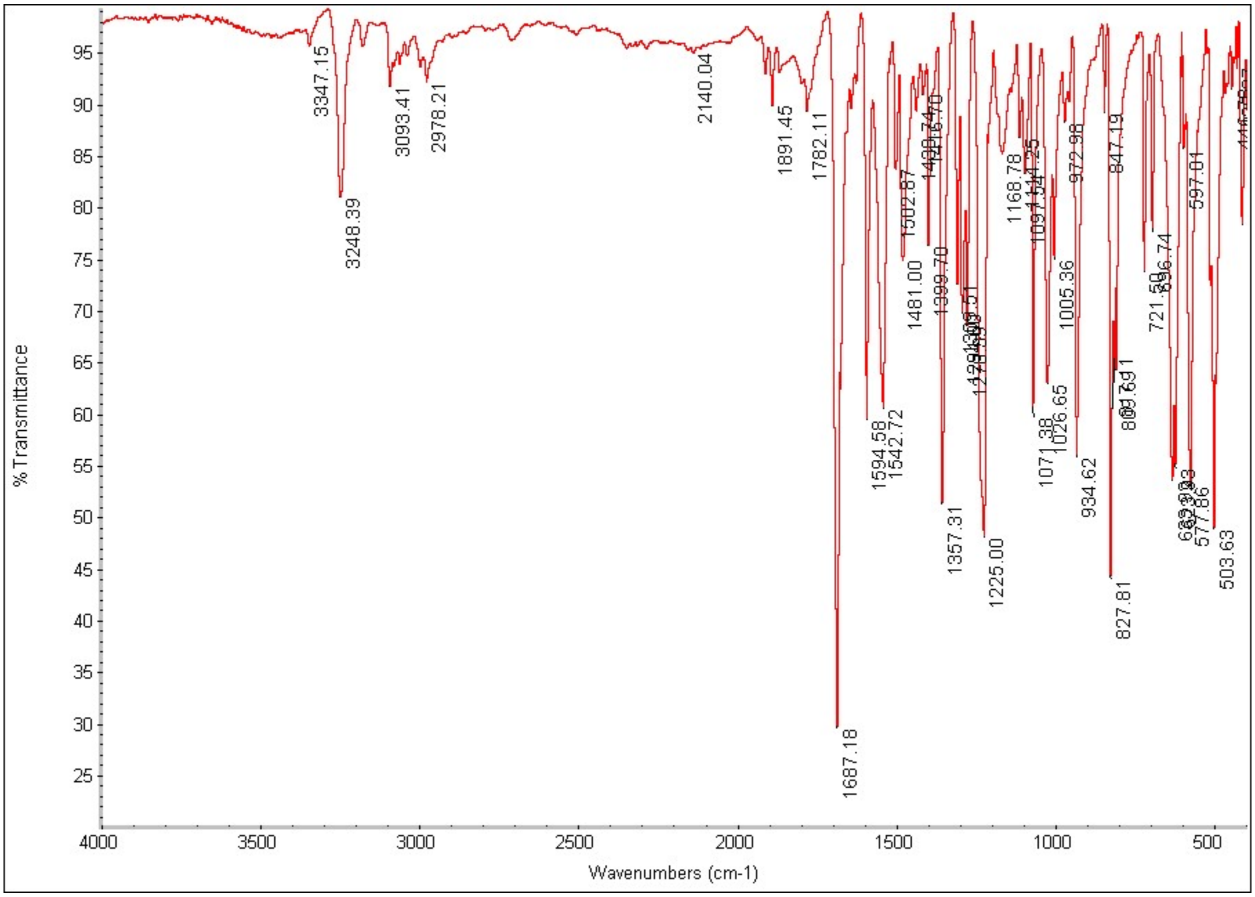

^{1}H NMR ($CDCl_3$) spectrum of (Z)-N-(4-iodophenyl)-2-oxopropanehydrazonoyl chloride (**29e**)

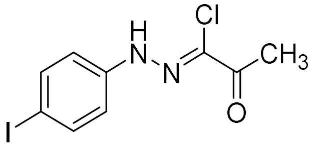

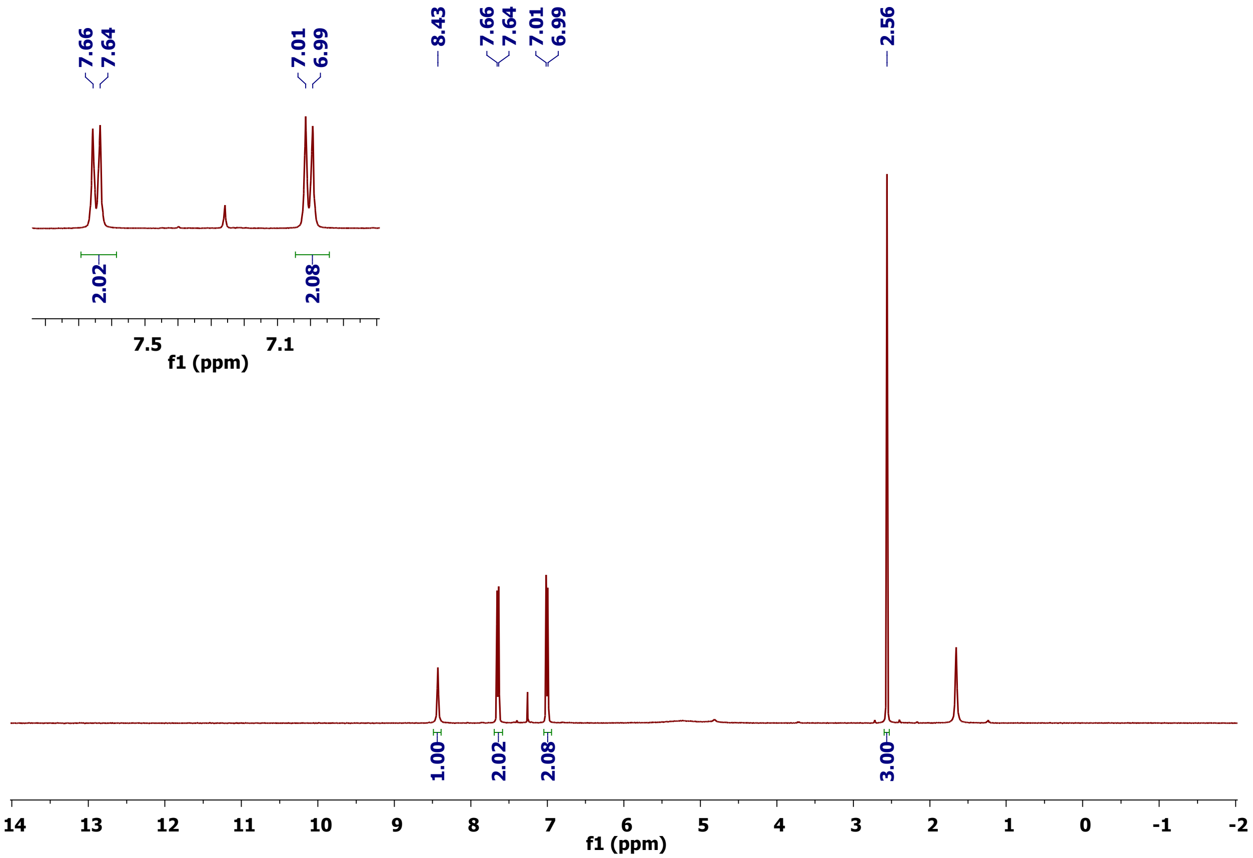

^{13}C NMR ($CDCl_3$) spectrum of (Z)-N-(4-iodophenyl)-2-oxopropanehydrazonoyl chloride

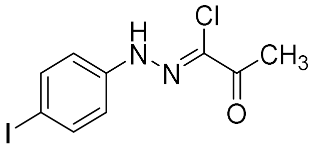

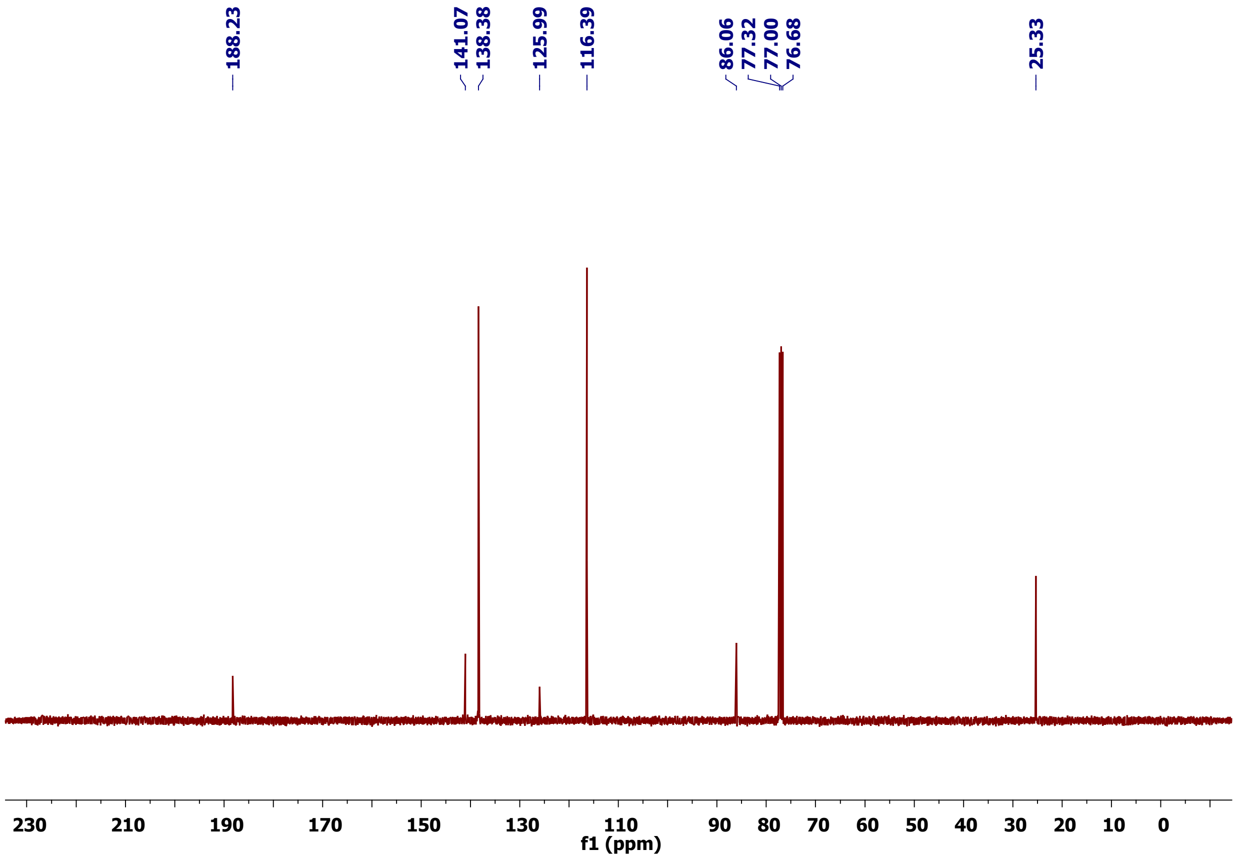

^{13}C CRAPT NMR ($CDCl_3$) spectrum of (Z)-N-(4-iodophenyl)-2-oxopropanehydrazonoyl chloride

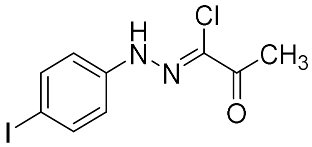

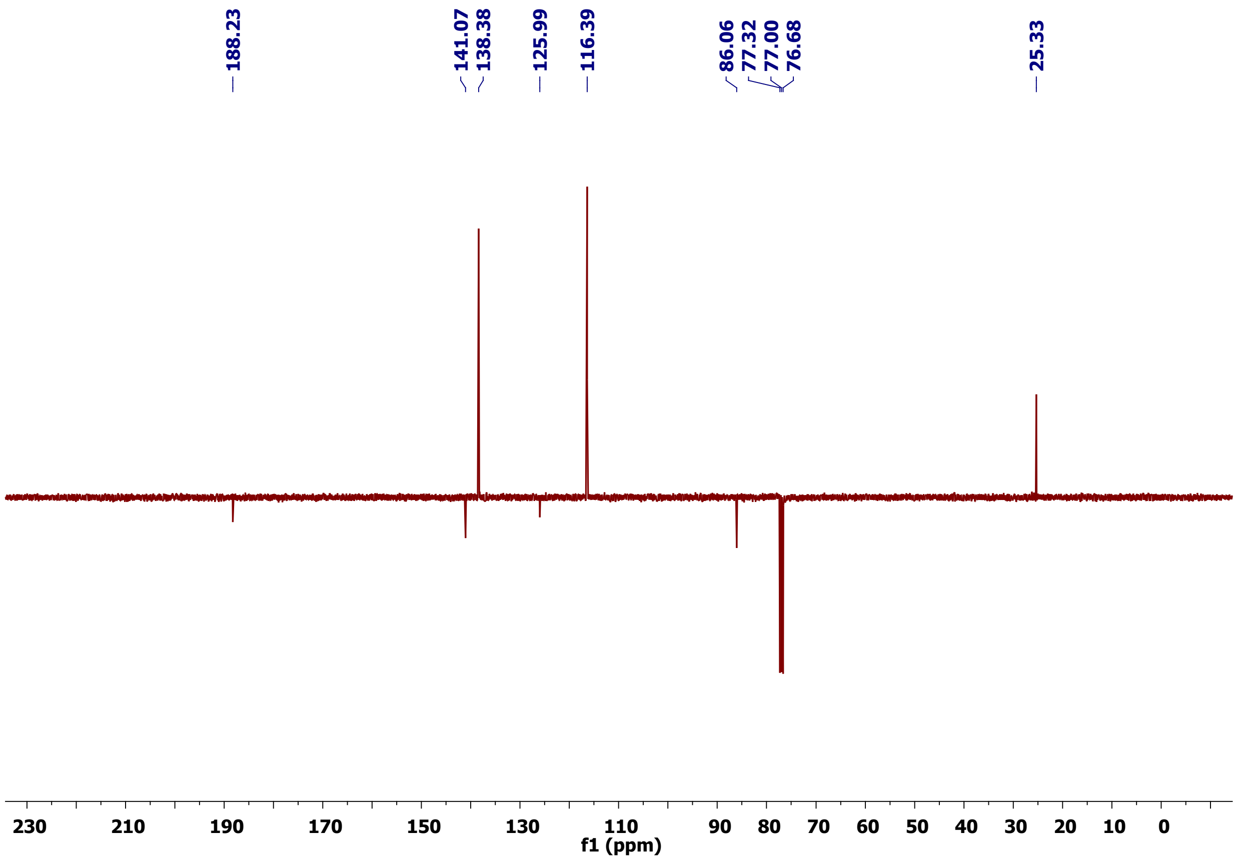

^{1}H-^{1}H-gDQCOSY NMR ($CDCl_3$) spectrum of (Z)-N-(4-iodophenyl)-2-oxopropanehydrazonoyl chloride

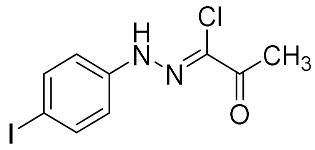

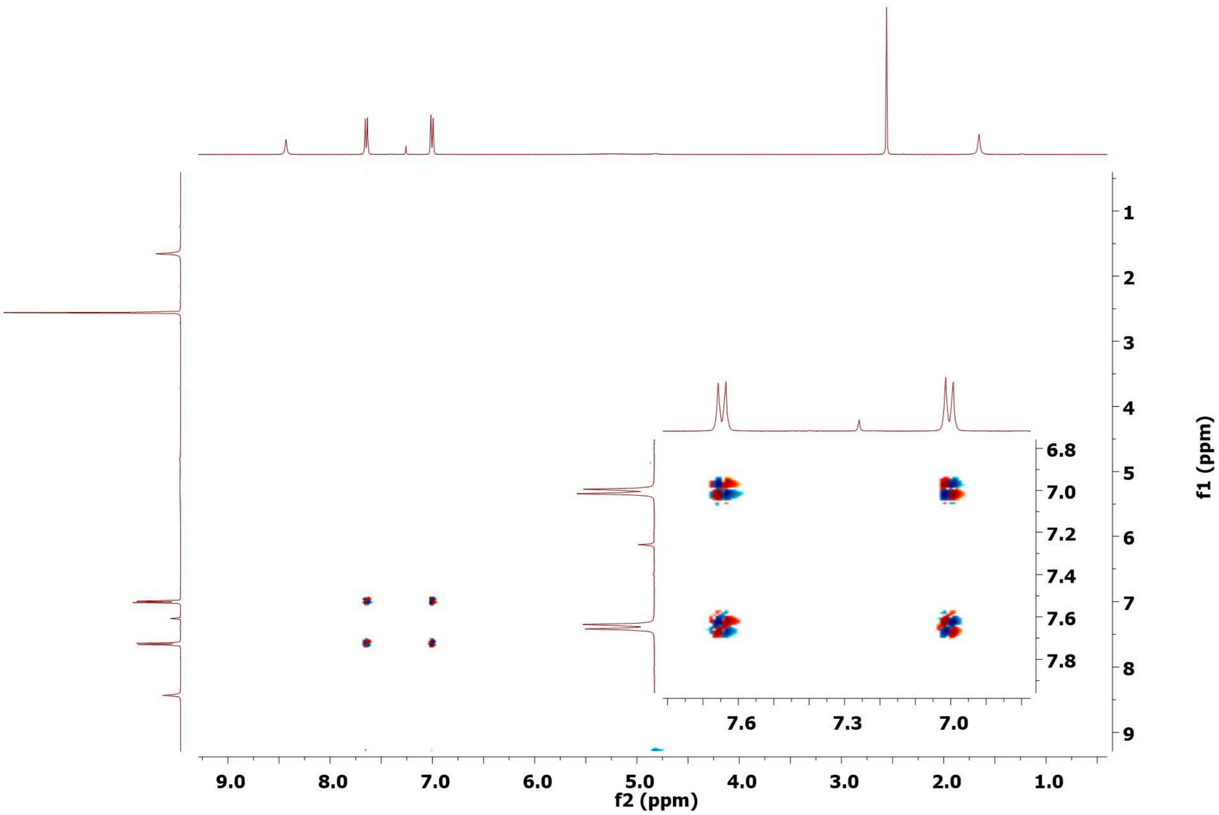

^{1}H-^{13}C-HSQC NMR ($CDCl_3$) spectrum of (Z)-N-(4-iodophenyl)-2-oxopropanehydrazonoyl chloride

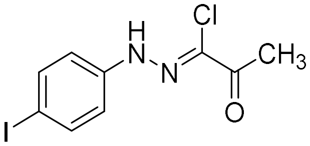

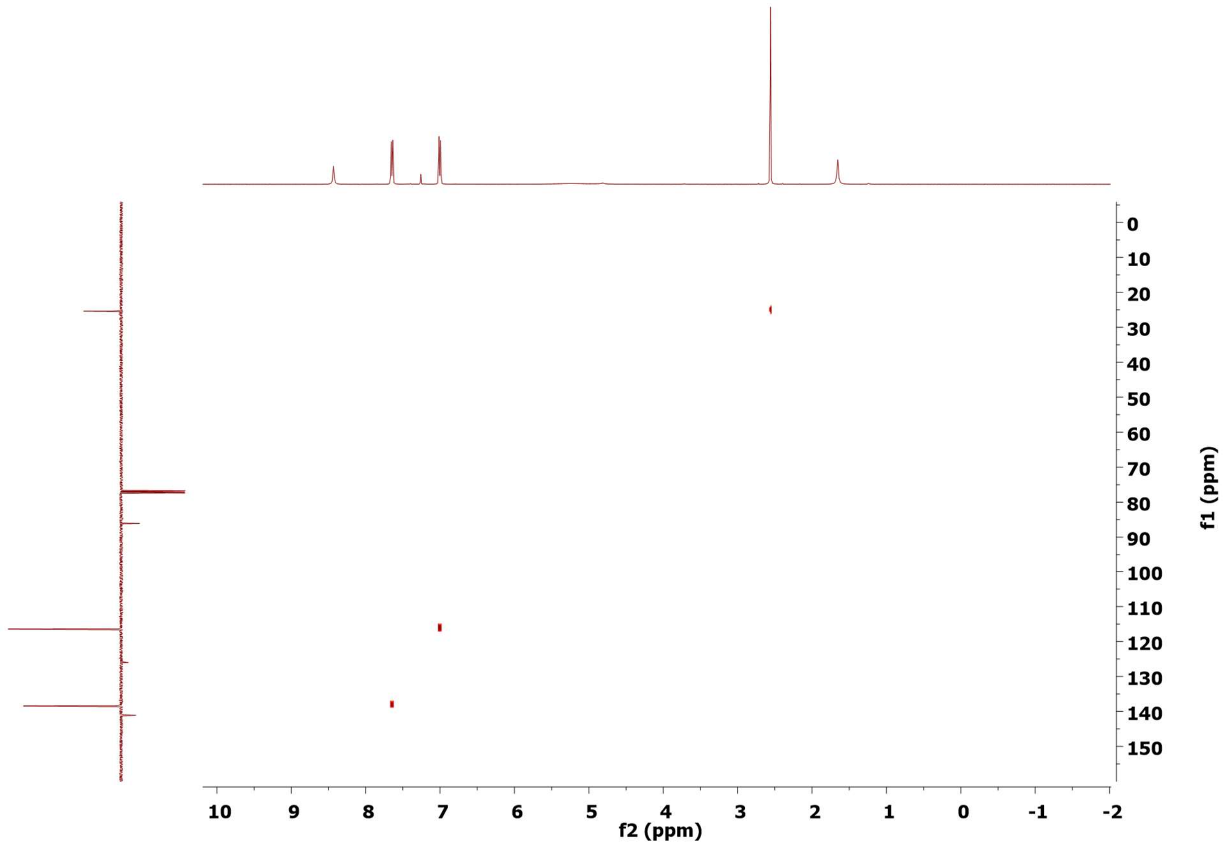

^{1}H-^{13}C-gHMBC NMR ($CDCl_3$) spectrum of (Z)-N-(4-iodophenyl)-2-oxopropanehydrazonoyl chloride

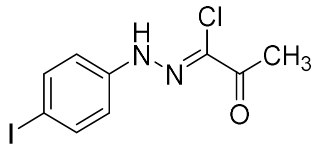

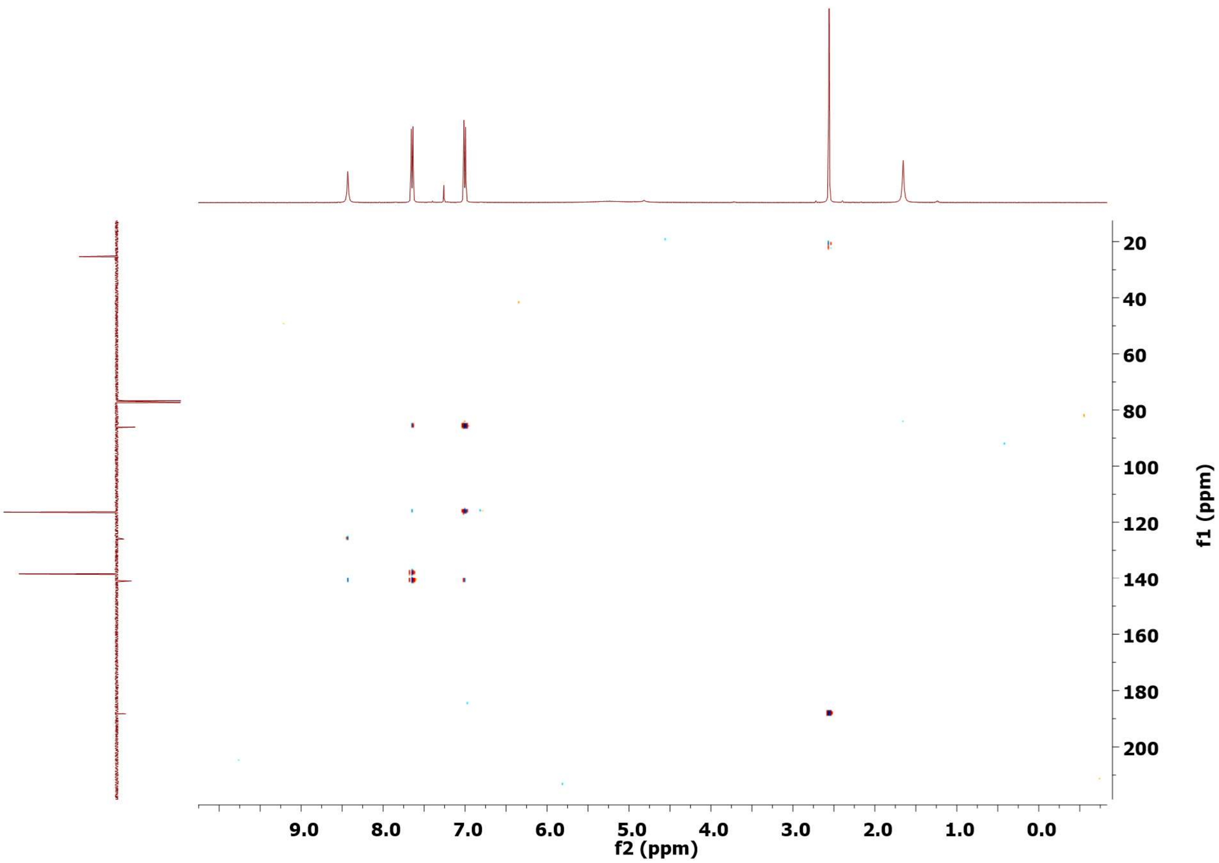

IR spectrum of (Z)-N-(4-iodophenyl)-2-oxopropanehydrazonoyl chloride

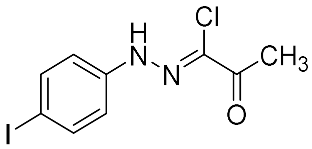

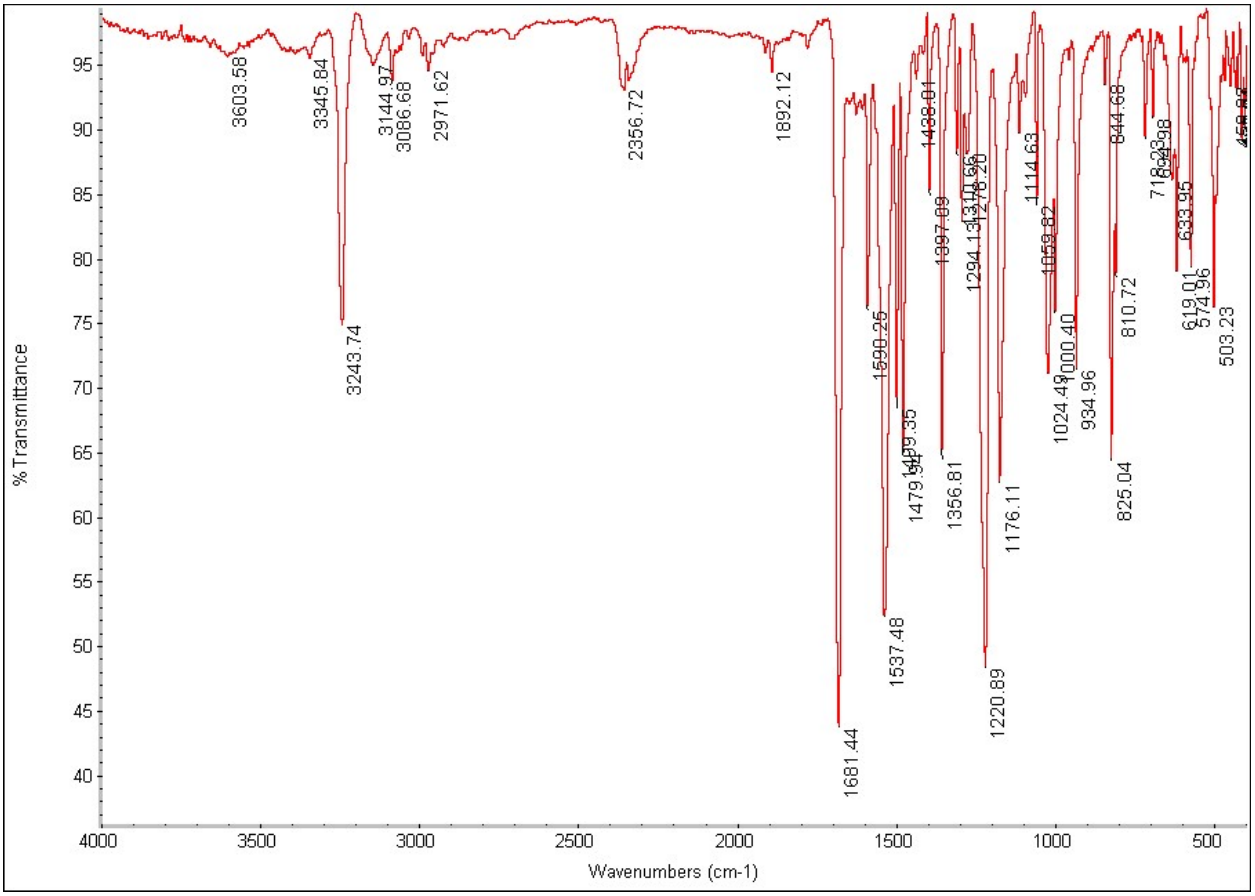

^{1}H NMR ($CDCl_3$) spectrum of (Z)-N-(4-(methylthio)phenyl)-2-oxopropanehydrazonoyl chloride **(29f)**

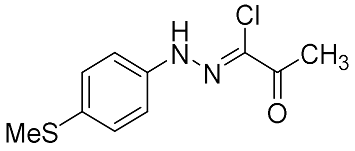

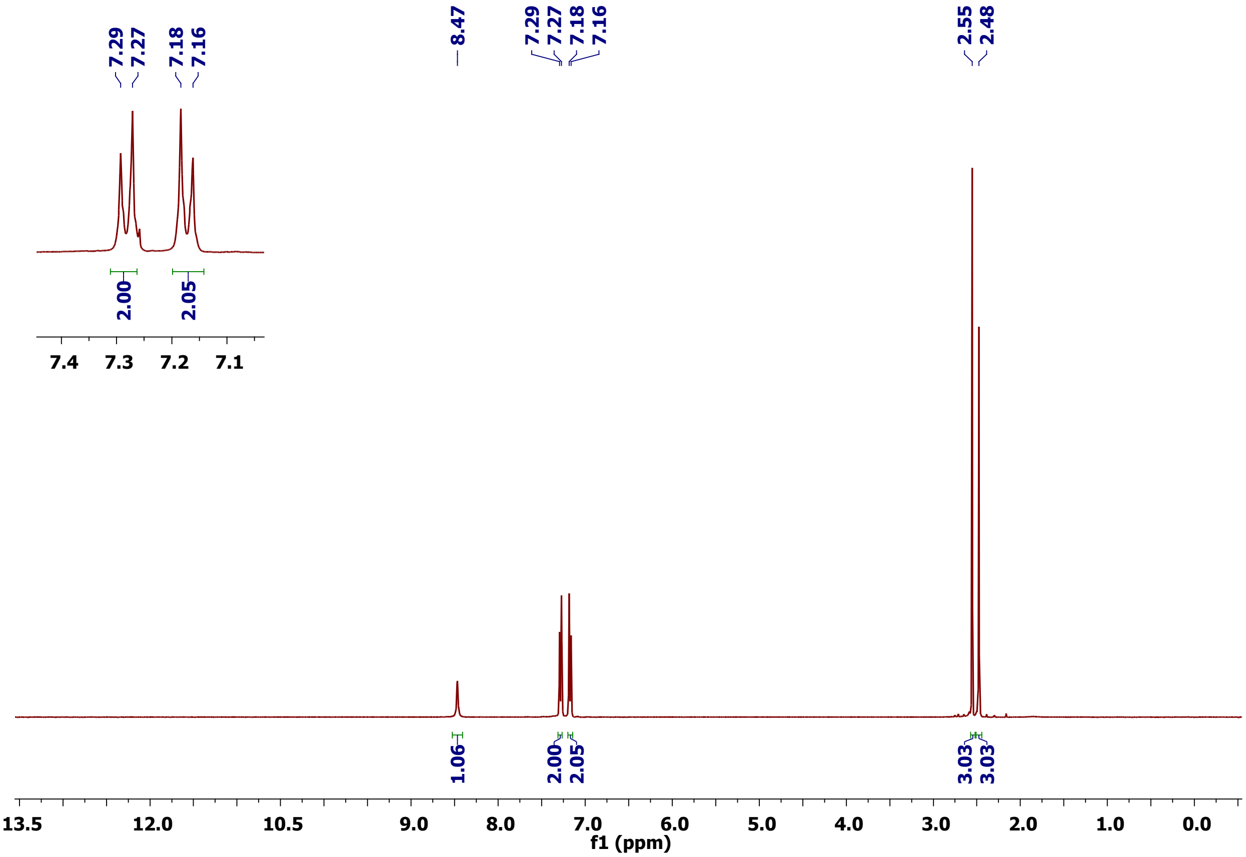

^{13}C NMR ($CDCl_3$) spectrum of (Z)-N-(4-(methylthio)phenyl)-2-oxopropanehydrazonoyl chloride

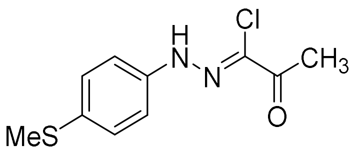

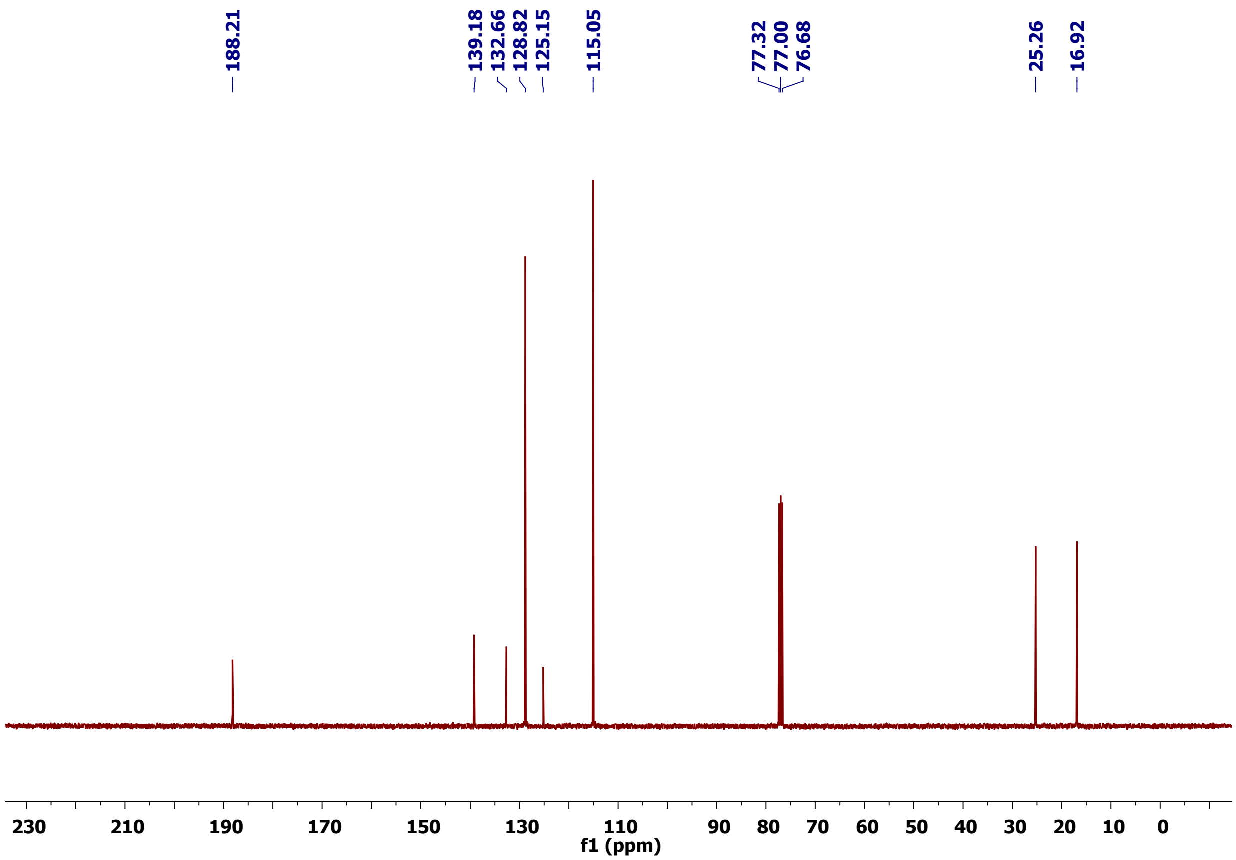

^{13}C CRAPT NMR ($CDCl_3$) spectrum of (Z)-N-(4-(methylthio)phenyl)-2-oxopropanehydrazonoyl chloride

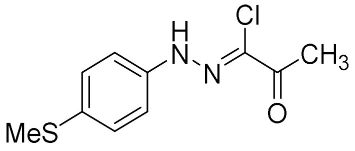

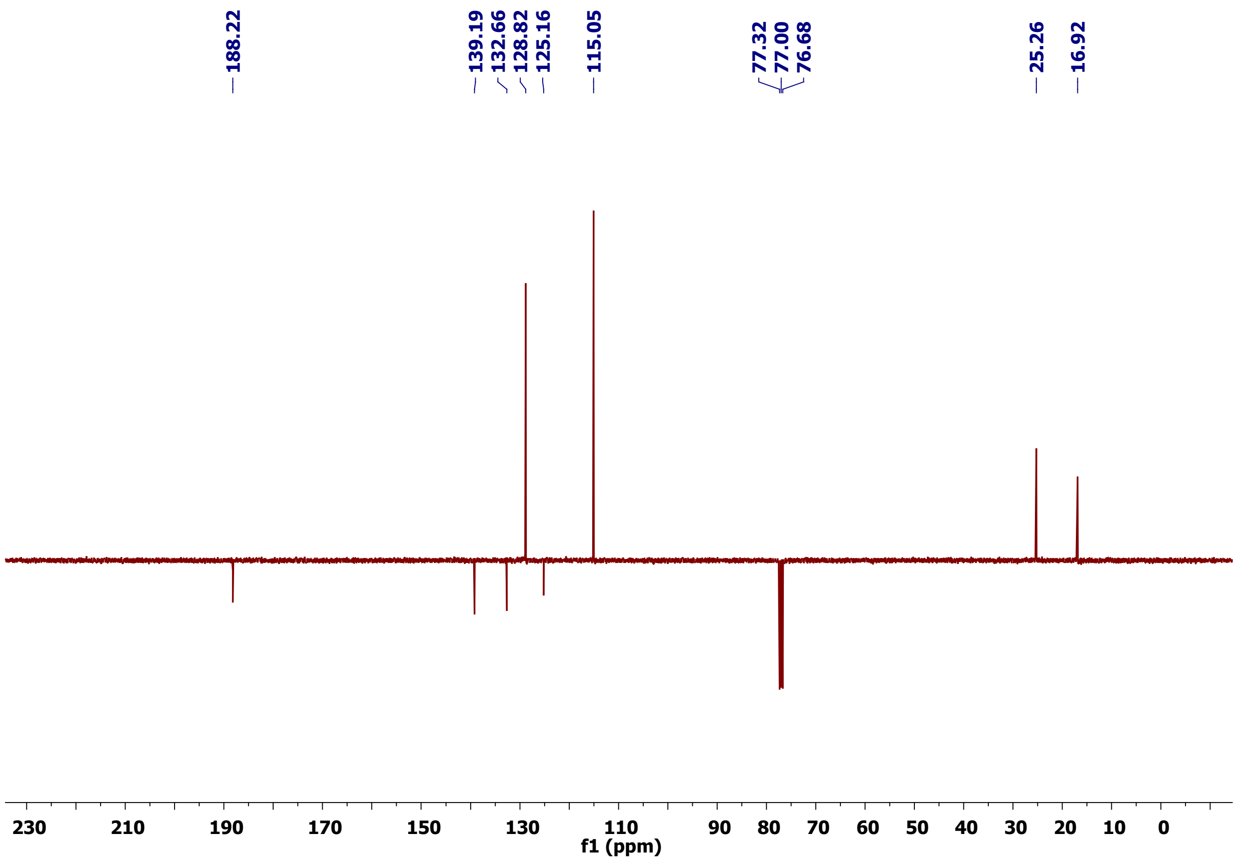

^{1}H-^{1}H-gDQCOSY NMR ($CDCl_3$) spectrum of (Z)-N-(4-(methylthio)phenyl)-2-oxopropanehydrazonoyl chloride

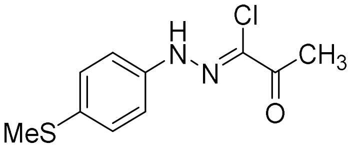

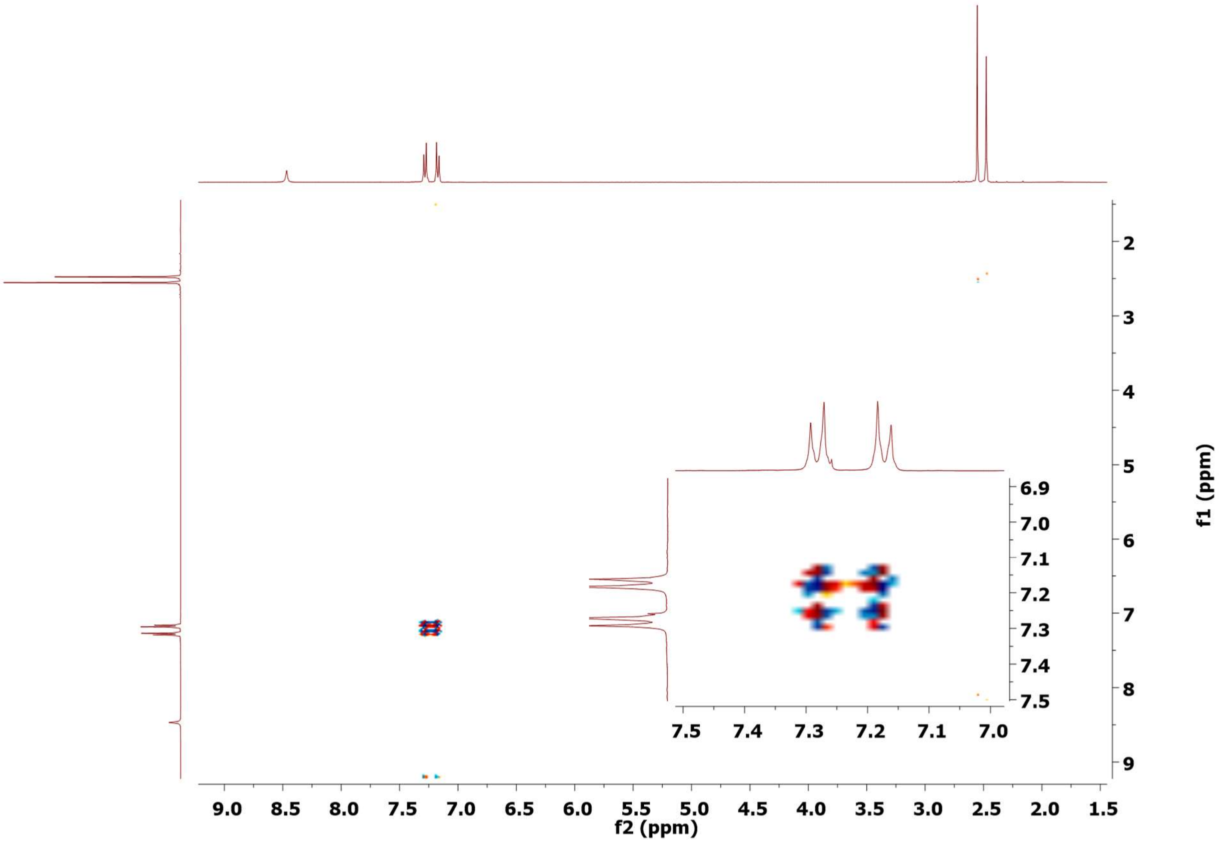

^{1}H-^{13}C-HSQC NMR ($CDCl_3$) spectrum of (Z)-N-(4-(methylthio)phenyl)-2-oxopropanehydrazonoyl chloride

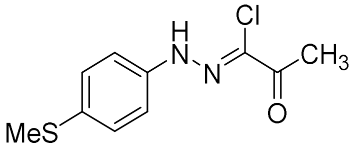

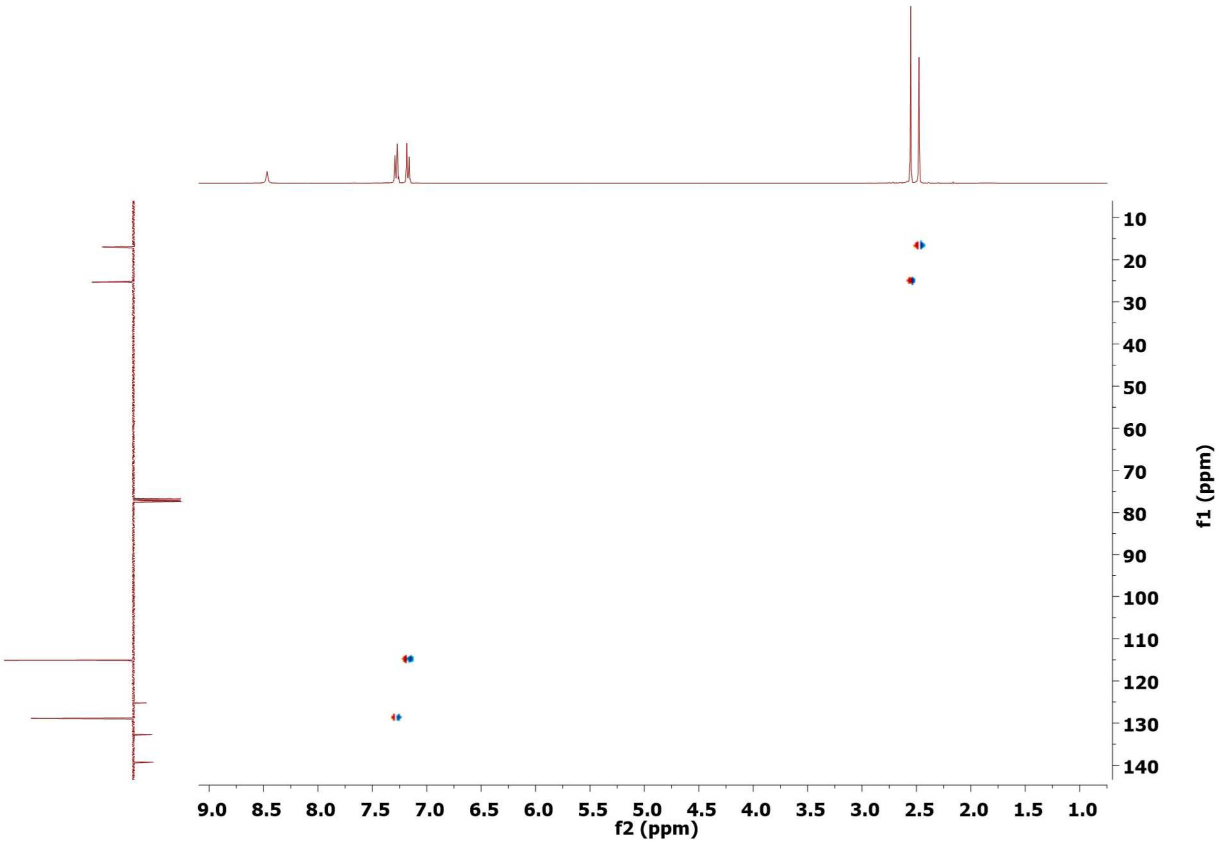

^{1}H-^{13}C-gHMBC NMR ($CDCl_3$) spectrum of (Z)-N-(4-(methylthio)phenyl)-2-oxopropanehydrazonoyl chloride

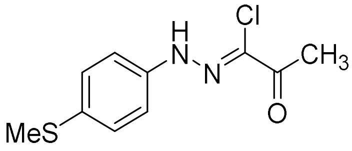

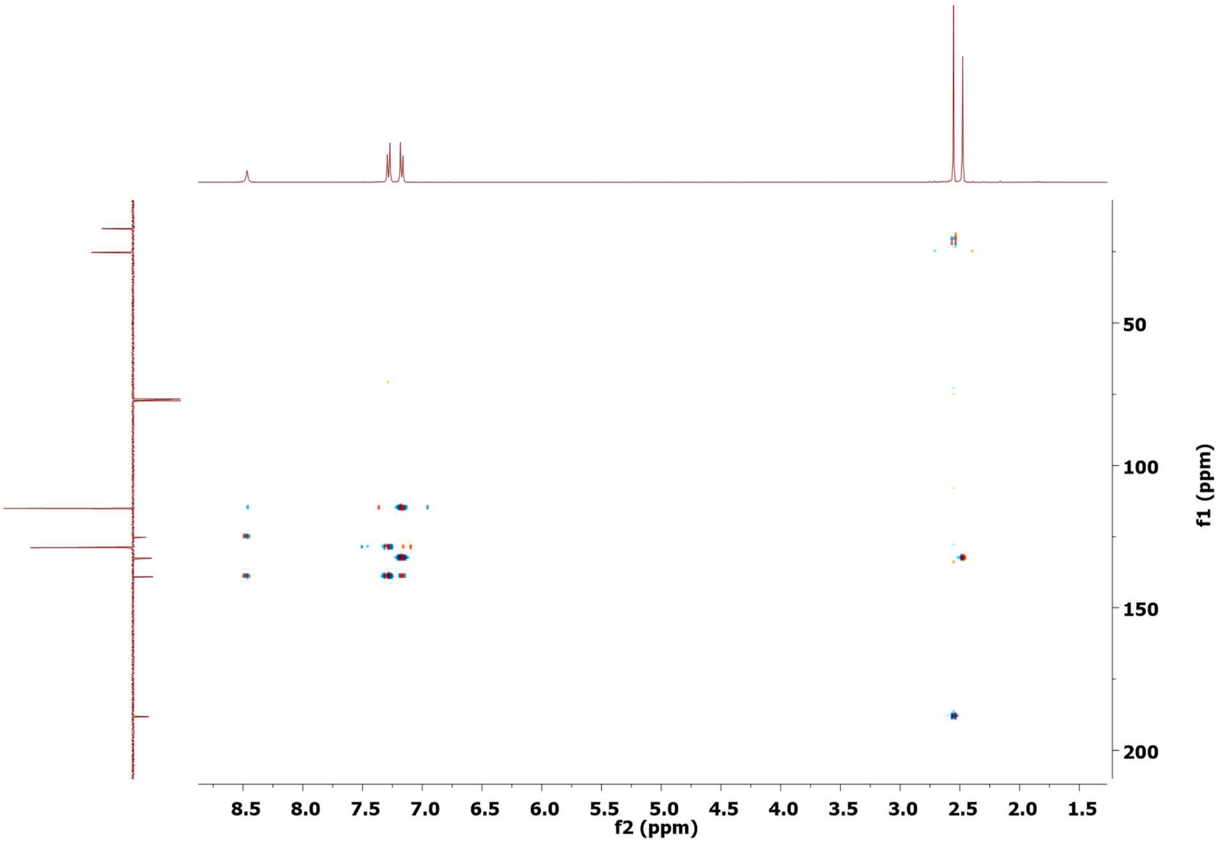

IR spectrum of (Z)-N-(4-(methylthio)phenyl)-2-oxopropanehydrazonoyl chloride

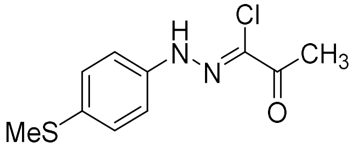

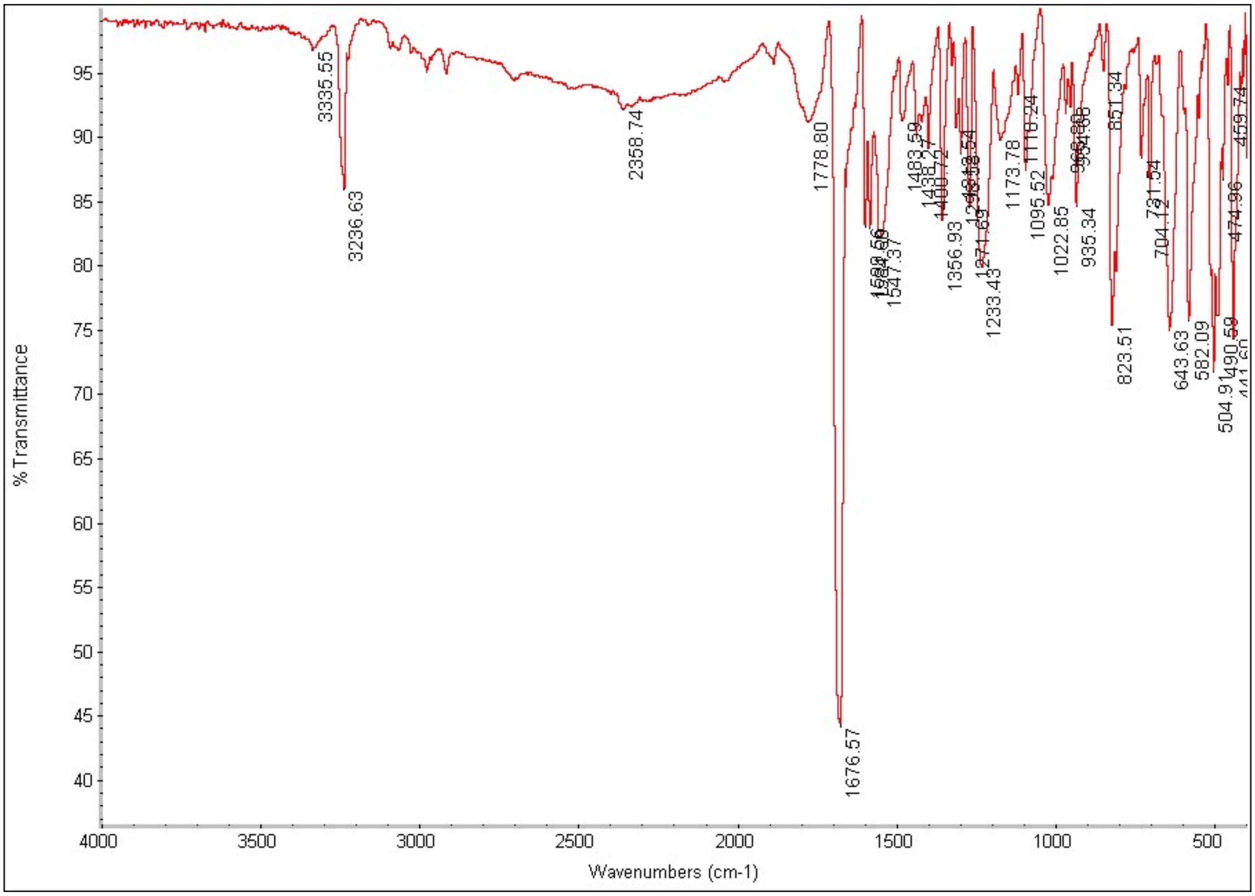

^{1}H NMR ($CDCl_3$) spectrum of (Z)-2-oxo-N-(m-tolyl)propanehydrazonoyl chloride (**29g**)

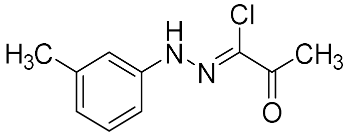

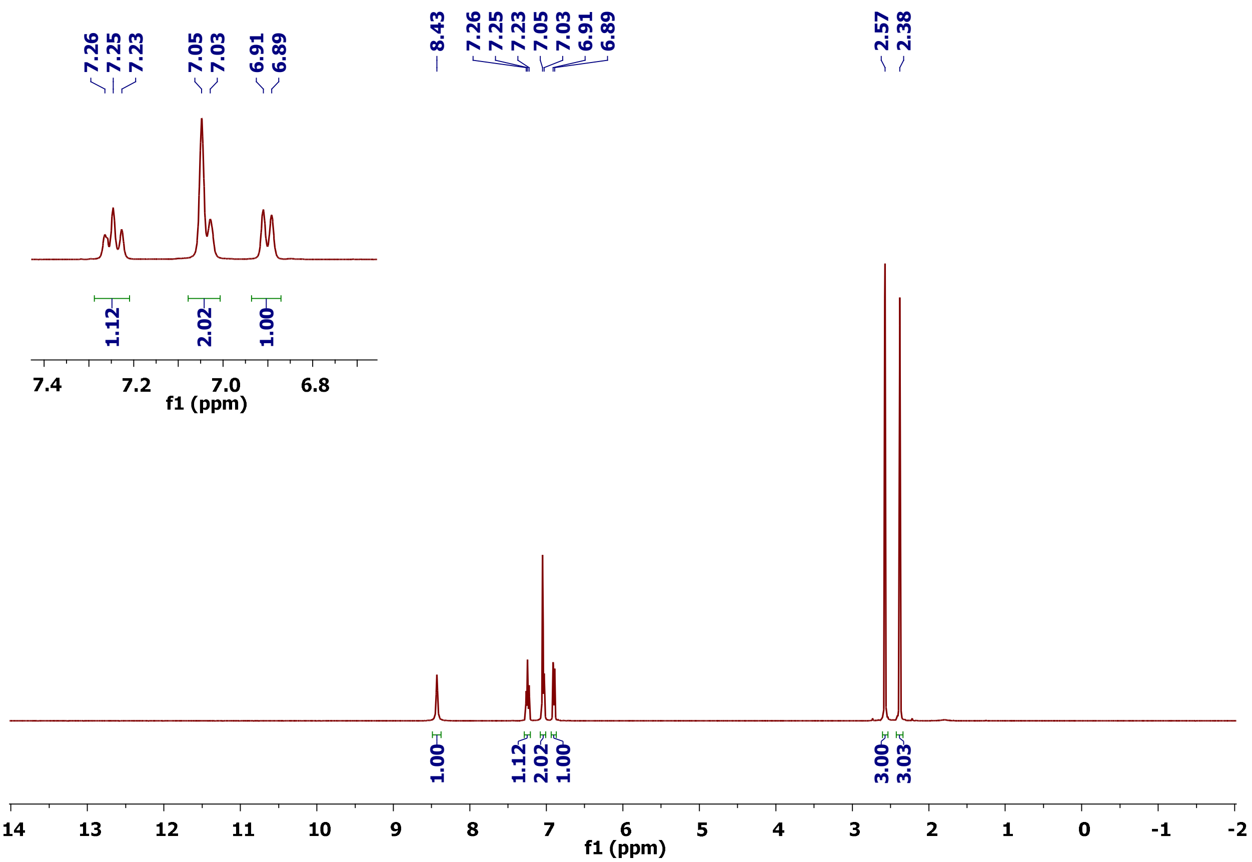

^{13}C NMR ($CDCl_3$) spectrum of (Z)-2-oxo-N-(m-tolyl)propanehydrazonoyl chloride

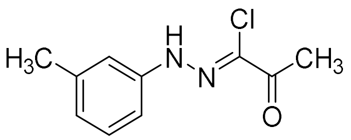

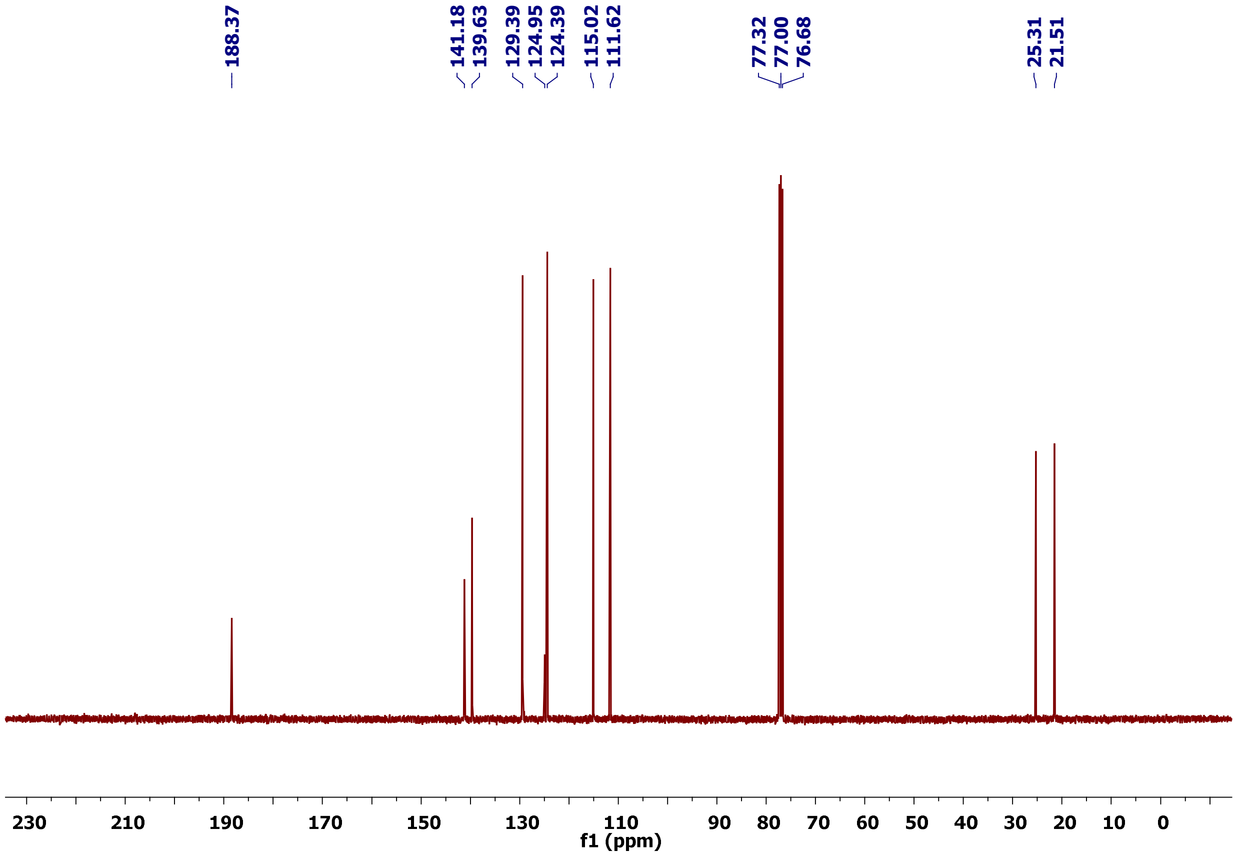

^{13}C CRAPT NMR ($CDCl_3$) spectrum of (Z)-2-oxo-N-(m-tolyl)propanehydrazonoyl chloride

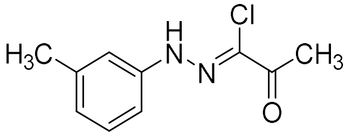

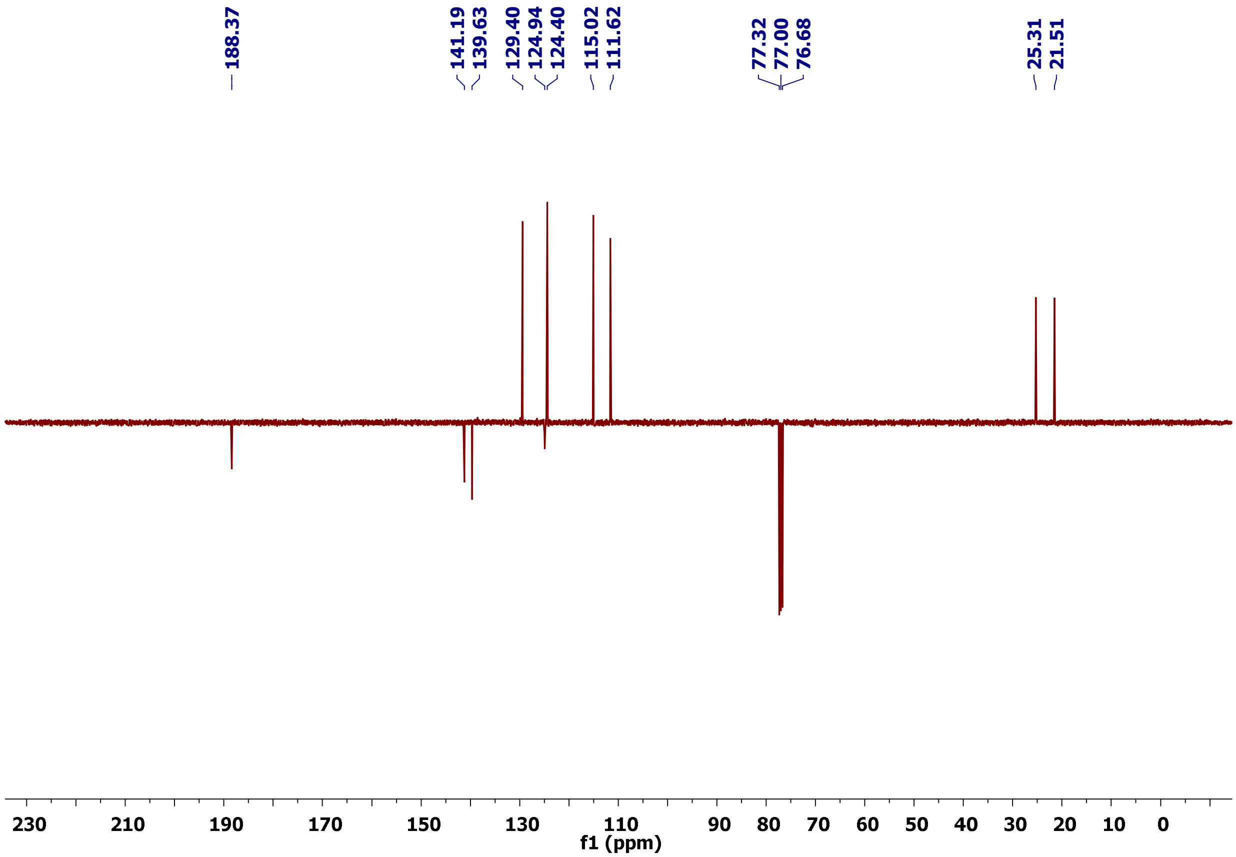

^{1}H-^{1}H-gDQCOSY NMR ($CDCl_3$) spectrum of (Z)-2-oxo-N-(m-tolyl)propanehydrazonoyl chloride

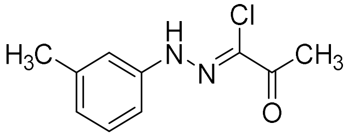

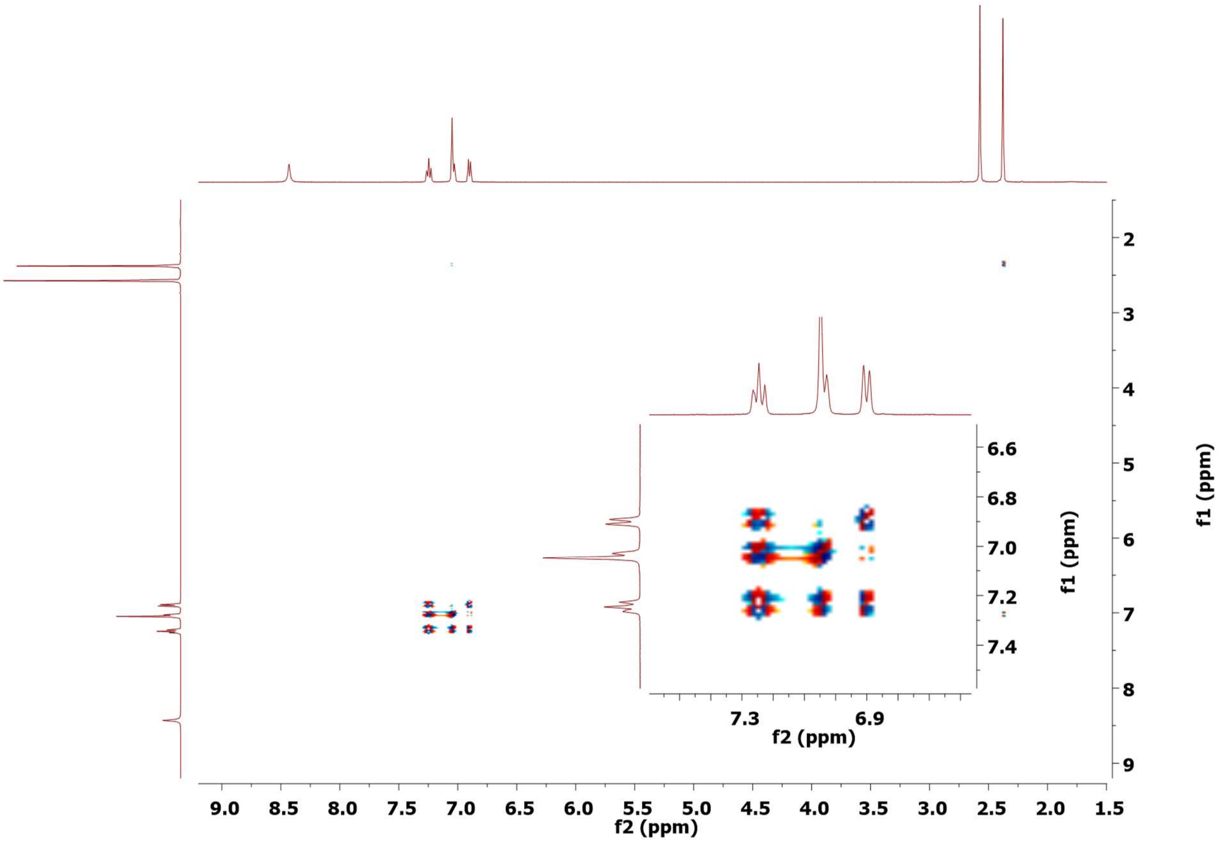

^{1}H-^{13}C-HSQC NMR ($CDCl_3$) spectrum of (Z)-2-oxo-N-(m-tolyl)propanehydrazonoyl chloride

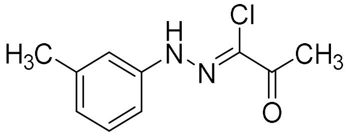

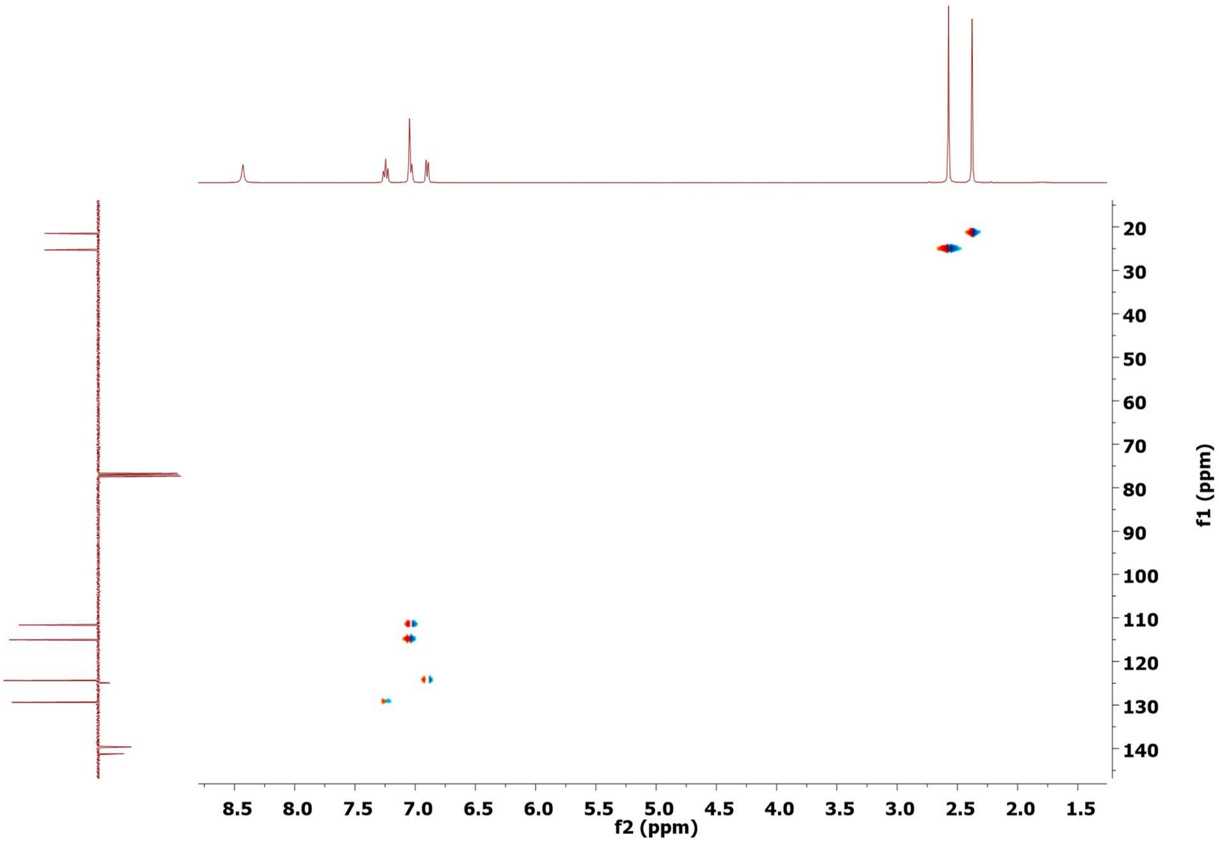

^{1}H-^{13}C-gHMBC NMR ($CDCl_3$) spectrum of (Z)-2-oxo-N-(m-tolyl)propanehydrazonoyl chloride

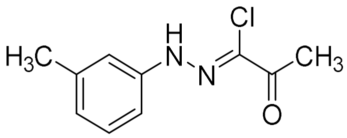

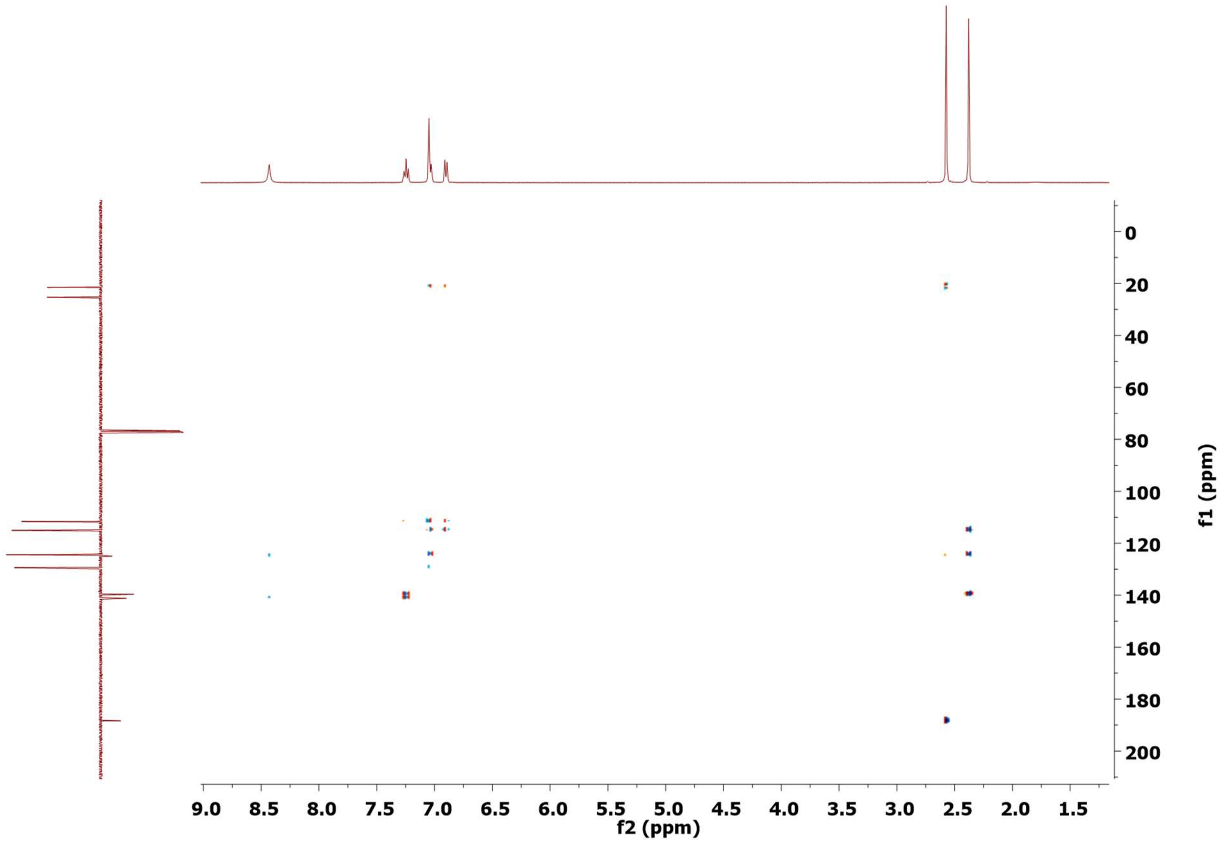

IR spectrum of (Z)-2-oxo-N-(m-tolyl)propanehydrazonoyl chloride

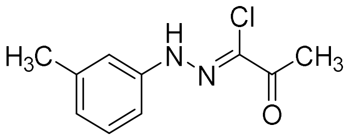

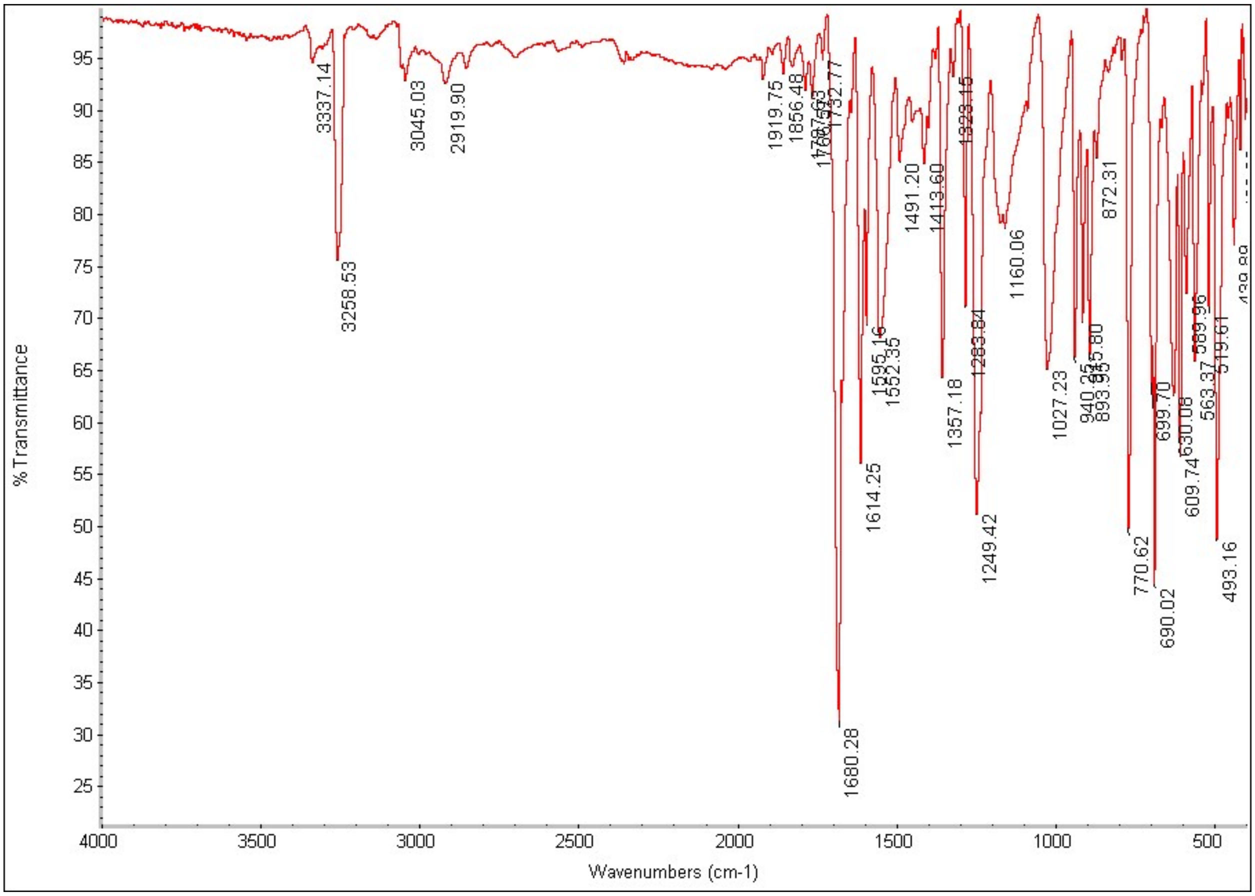

^{1}H NMR ($CDCl_3$) spectrum of (Z)-2-oxo-N-(3-(trifluoromethyl)phenyl)propanehydrazonoyl chloride (**29h**)

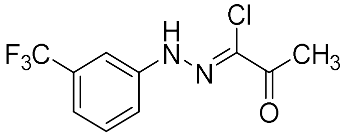

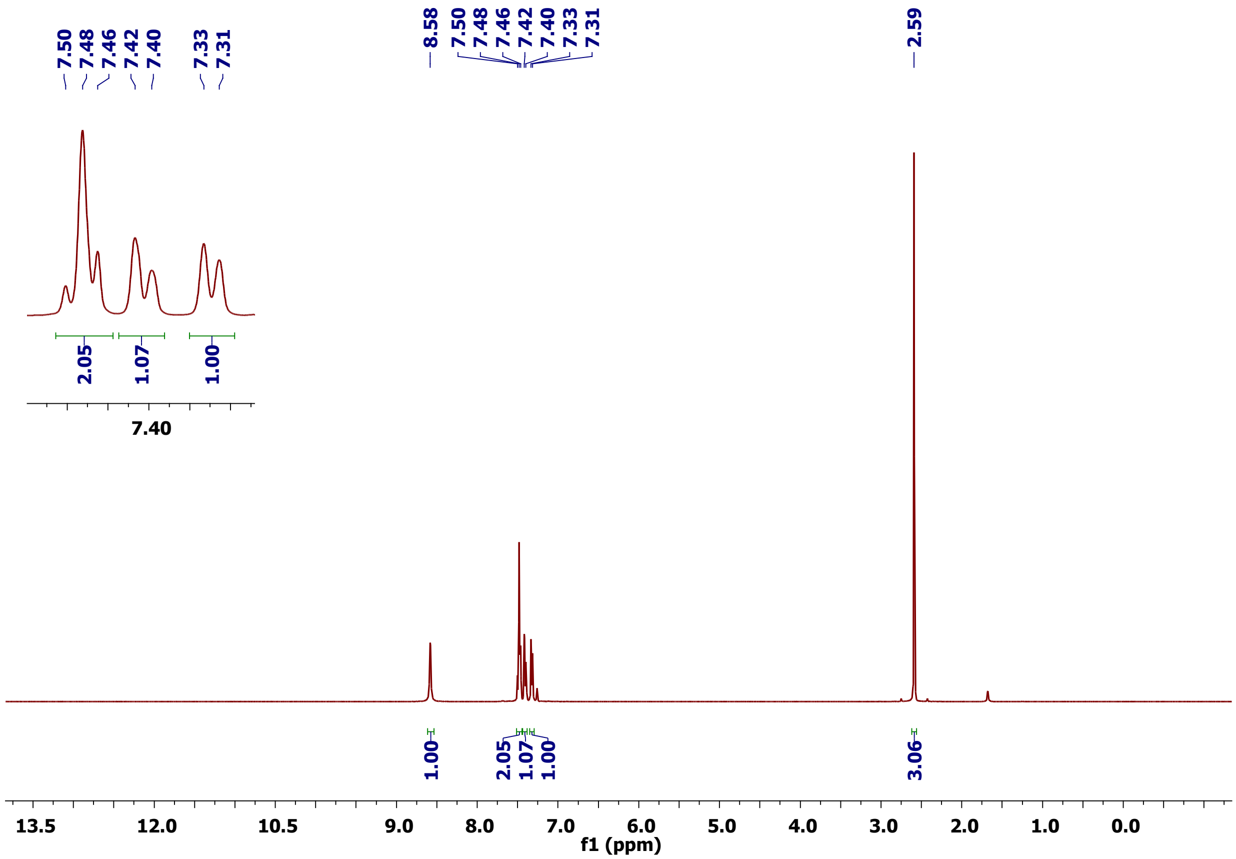

^{13}C NMR ($CDCl_3$) spectrum of (Z)-2-oxo-N-(3-(trifluoromethyl)phenyl)propanehydrazonoyl chloride

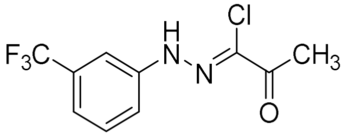

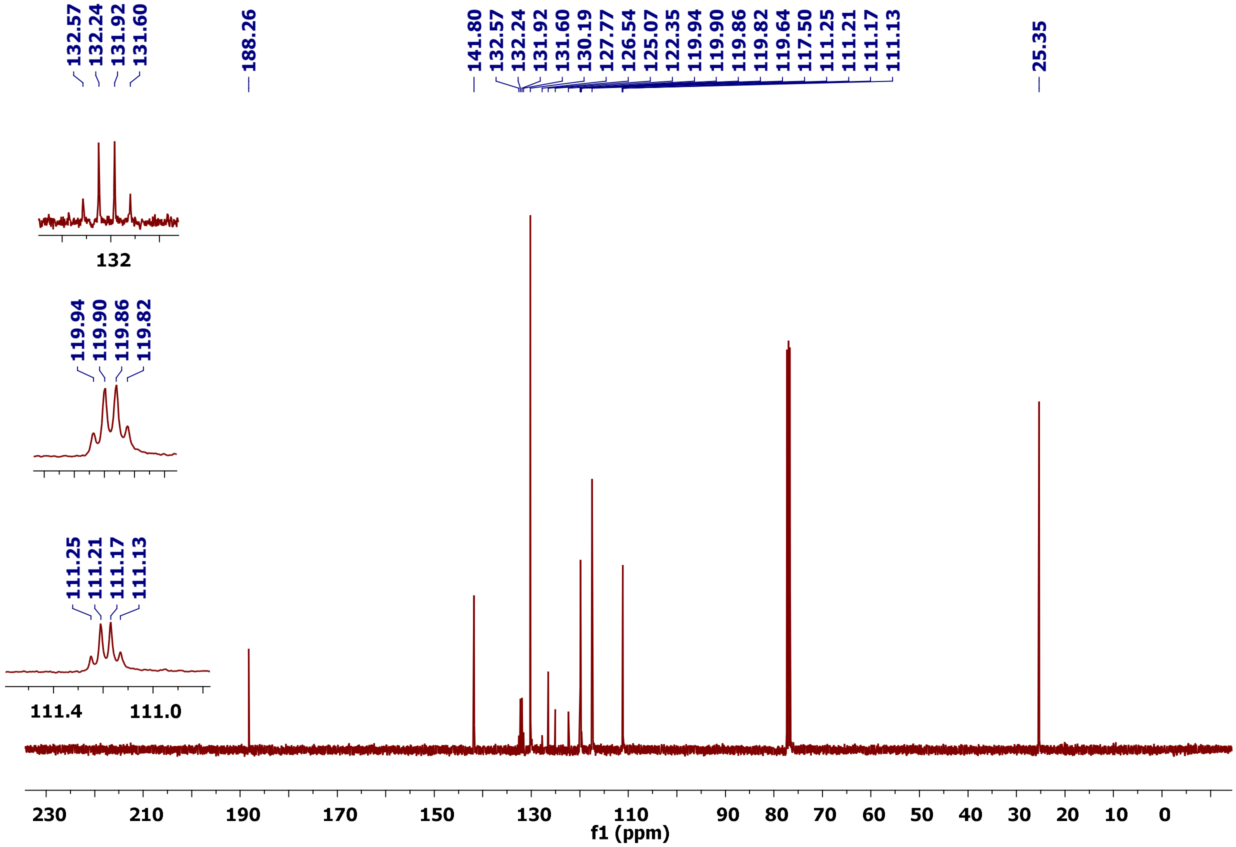

^{13}C CRAPT NMR ($CDCl_3$) spectrum of (Z)-2-oxo-N-(3-(trifluoromethyl)phenyl)propanehydrazonoyl chloride

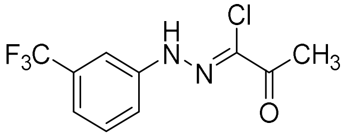

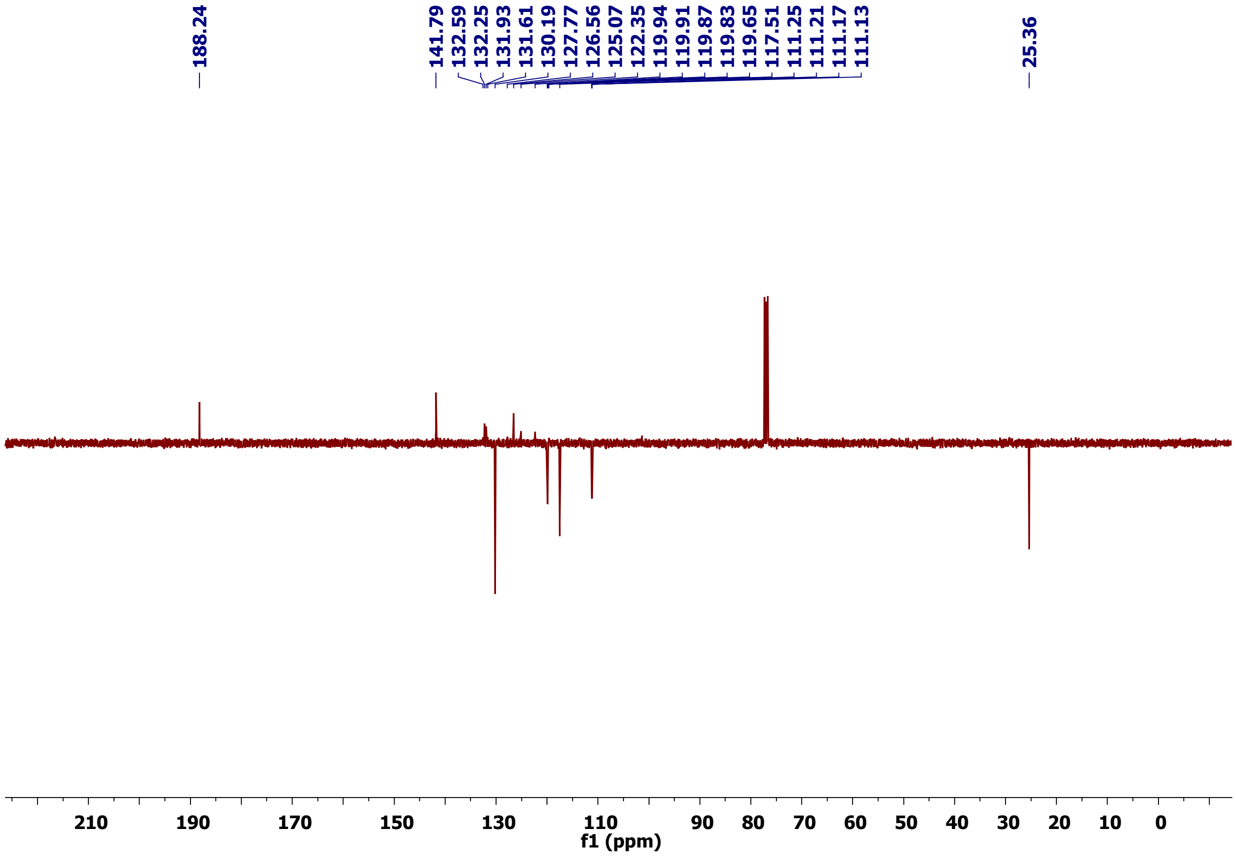

^{1}H-^{1}H-gDQCOSY NMR ($CDCl_3$) spectrum of (Z)-2-oxo-N-(3-(trifluoromethyl)phenyl)propanehydrazonoyl chloride

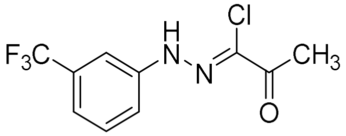

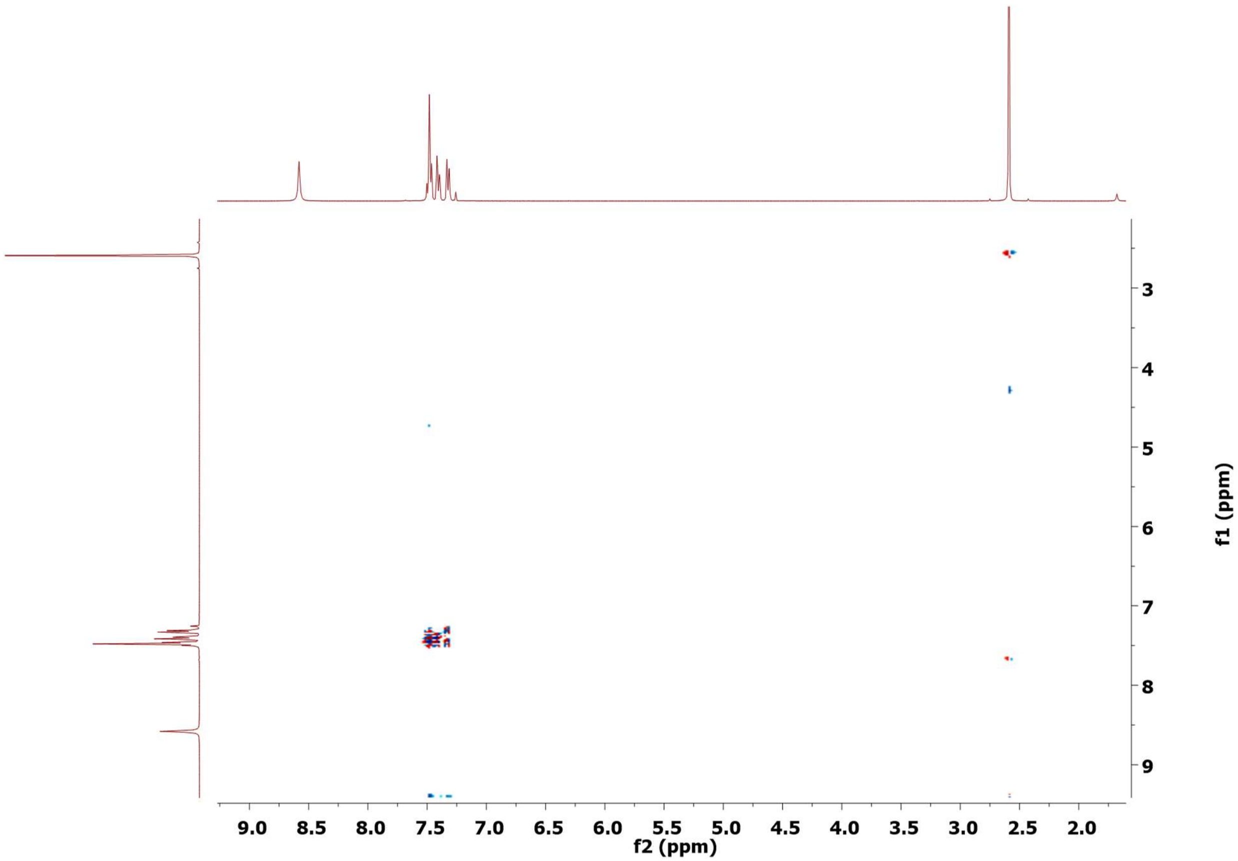

^1H-^{13}C-HSQC NMR ($CDCl_3$) spectrum of (Z)-2-oxo-N-(3-(trifluoromethyl)phenyl)propanehydrazonoyl chloride

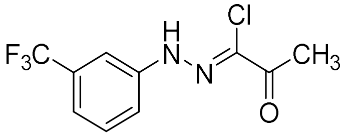

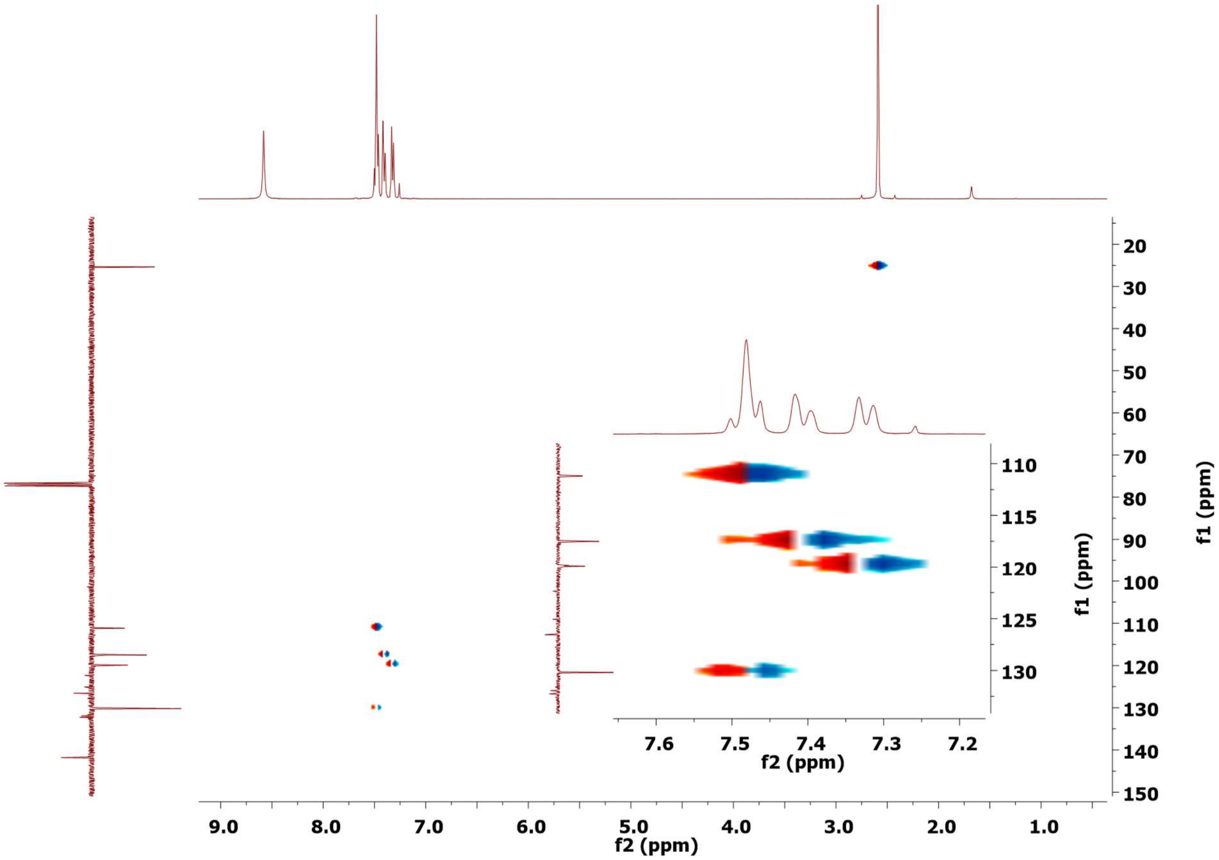

^1H-^{13}C-gHMBC NMR ($CDCl_3$) spectrum of (Z)-2-oxo-N-(3-(trifluoromethyl)phenyl)propanehydrazonoyl chloride

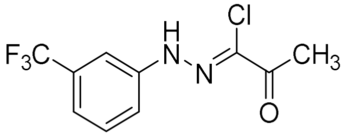

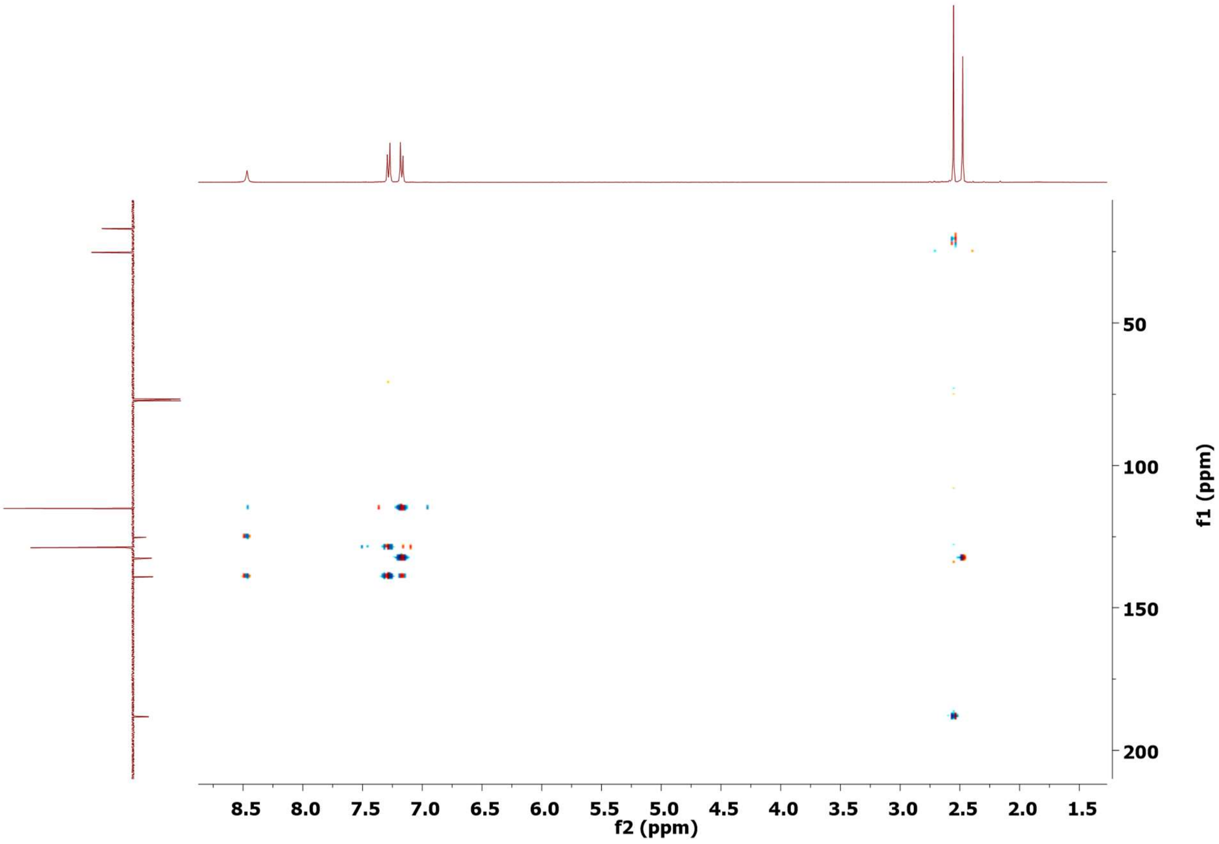

IR spectrum of (Z)-2-oxo-N-(3-(trifluoromethyl)phenyl)propanehydrazonoyl chloride

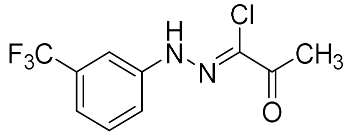

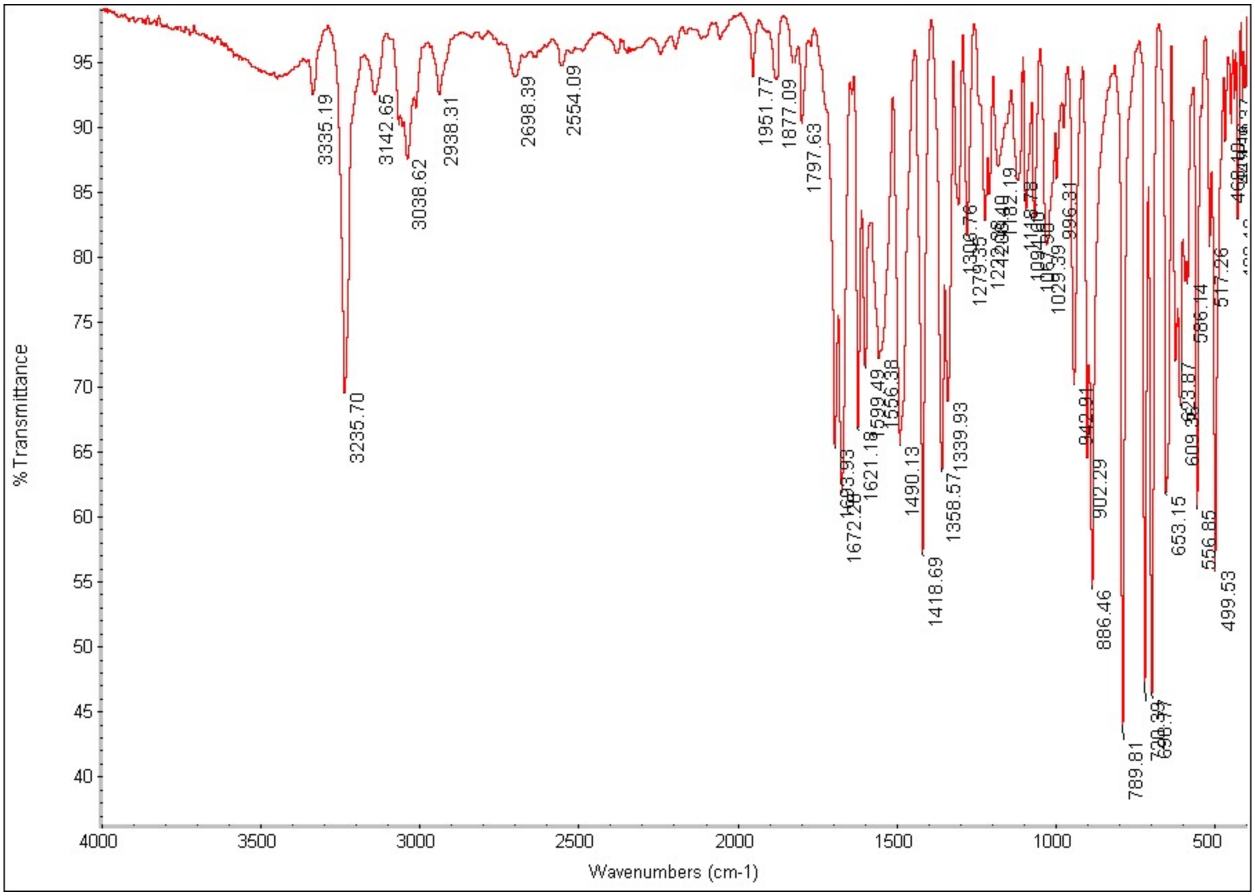

^{1}H NMR ($CDCl_3$) spectrum of (Z)-N-(4-methoxyphenyl)-2-oxopropanehydrazonoyl chloride (**29i**)

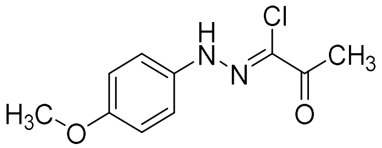

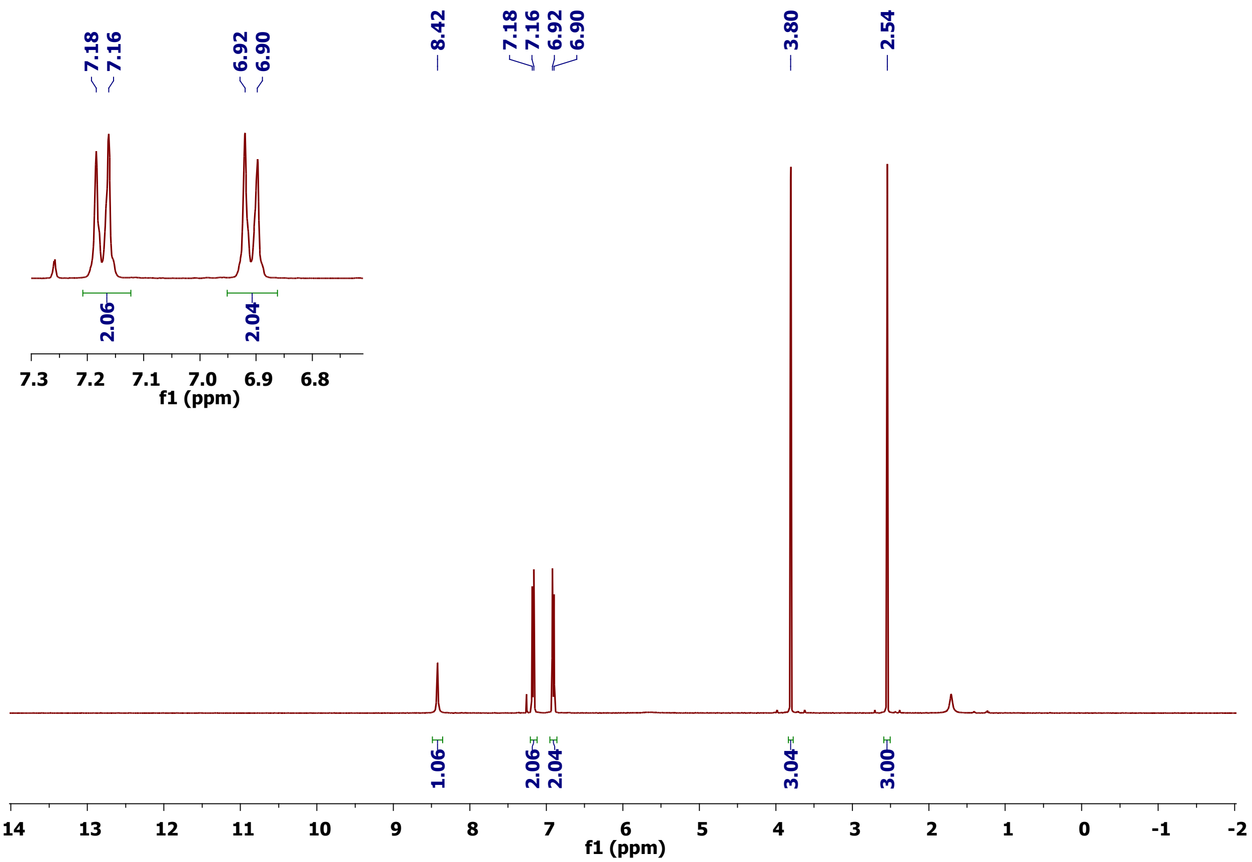

^{13}C NMR ($CDCl_3$) spectrum of (Z)-N-(4-methoxyphenyl)-2-oxopropanehydrazonoyl chloride

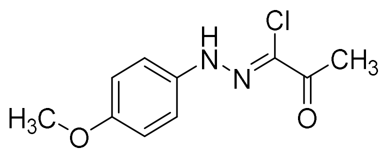

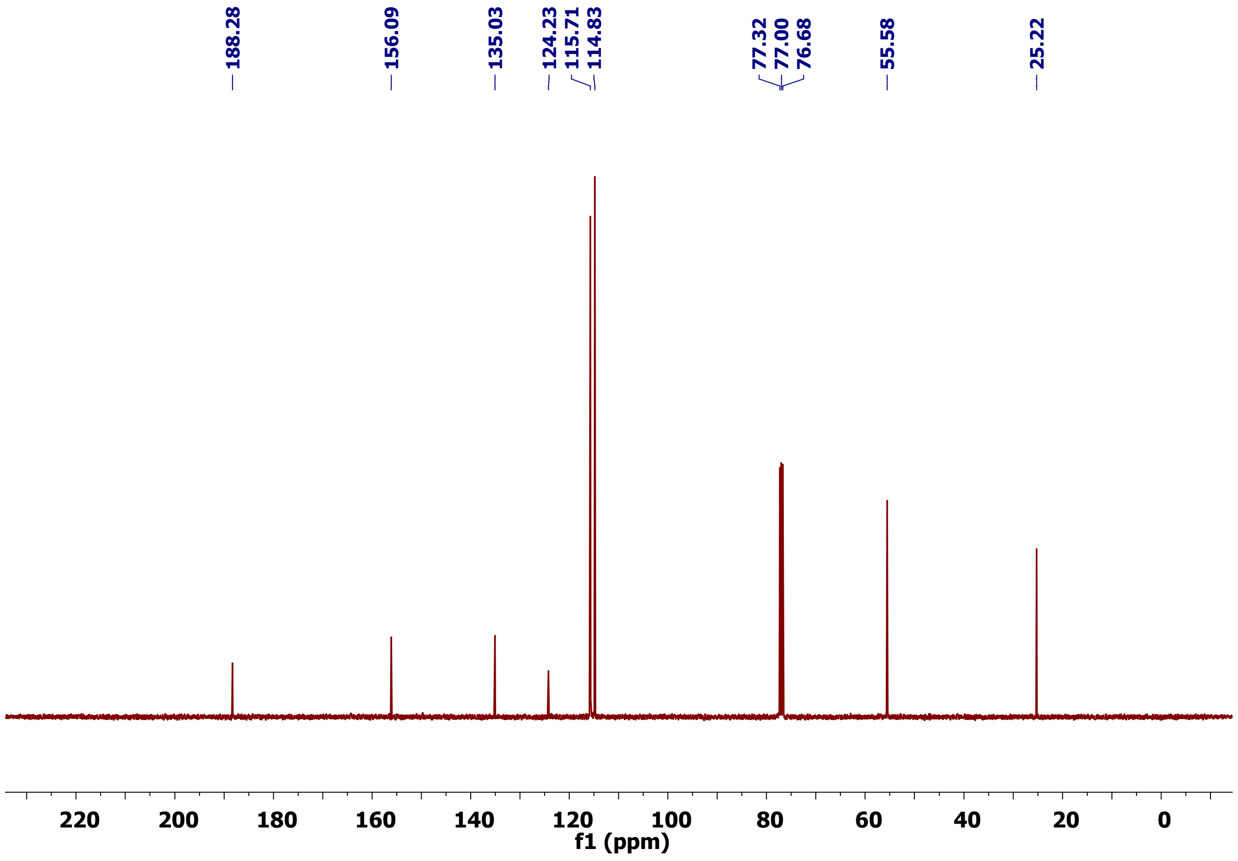

^{13}C CRAPT NMR ($CDCl_3$) spectrum of (Z)-N-(4-methoxyphenyl)-2-oxopropanehydrazonoyl chloride

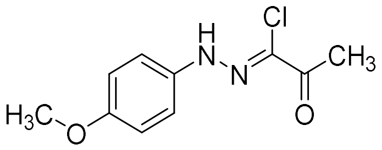

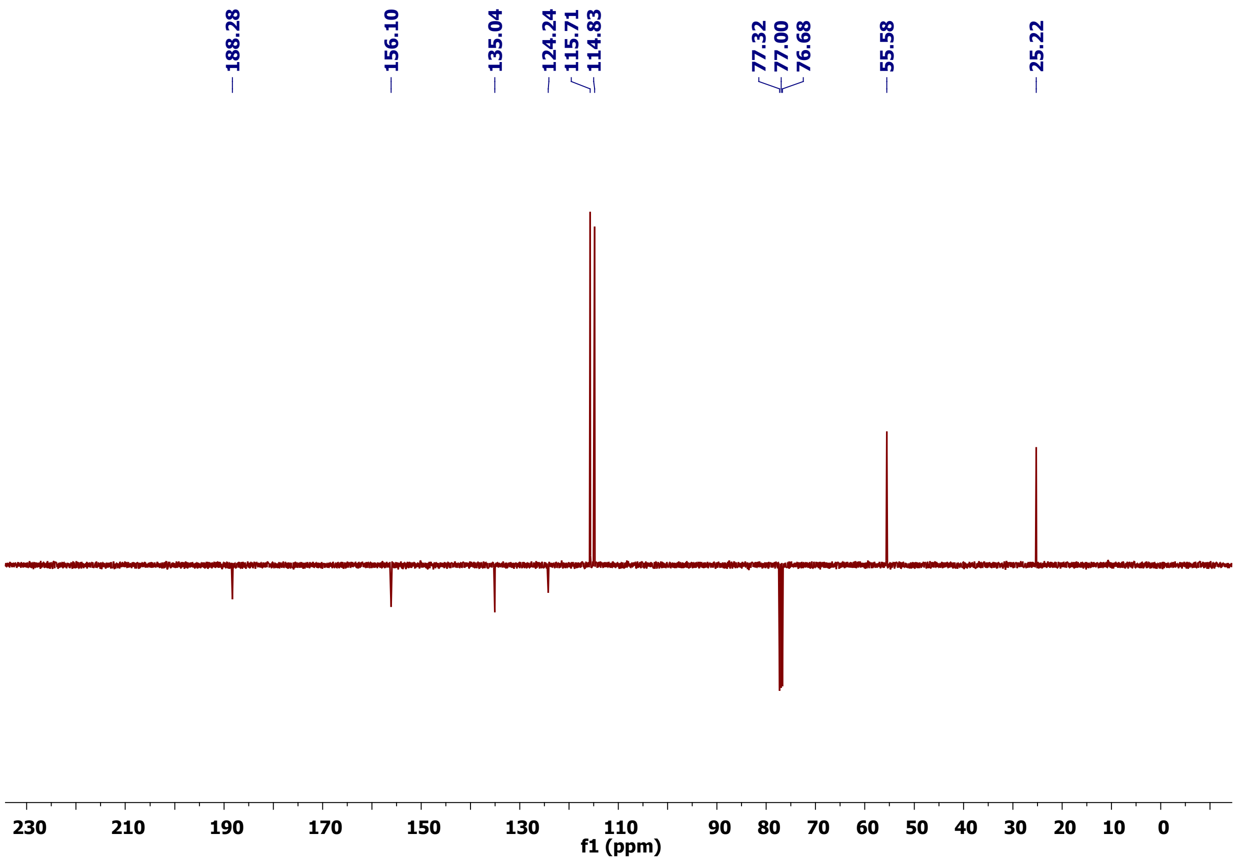

^{1}H-^{1}H-gDQCOSY NMR ($CDCl_3$) spectrum of (Z)-N-(4-methoxyphenyl)-2-oxopropanehydrazonoyl chloride

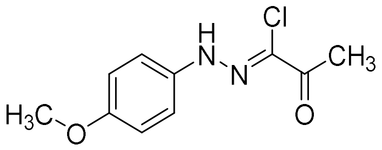

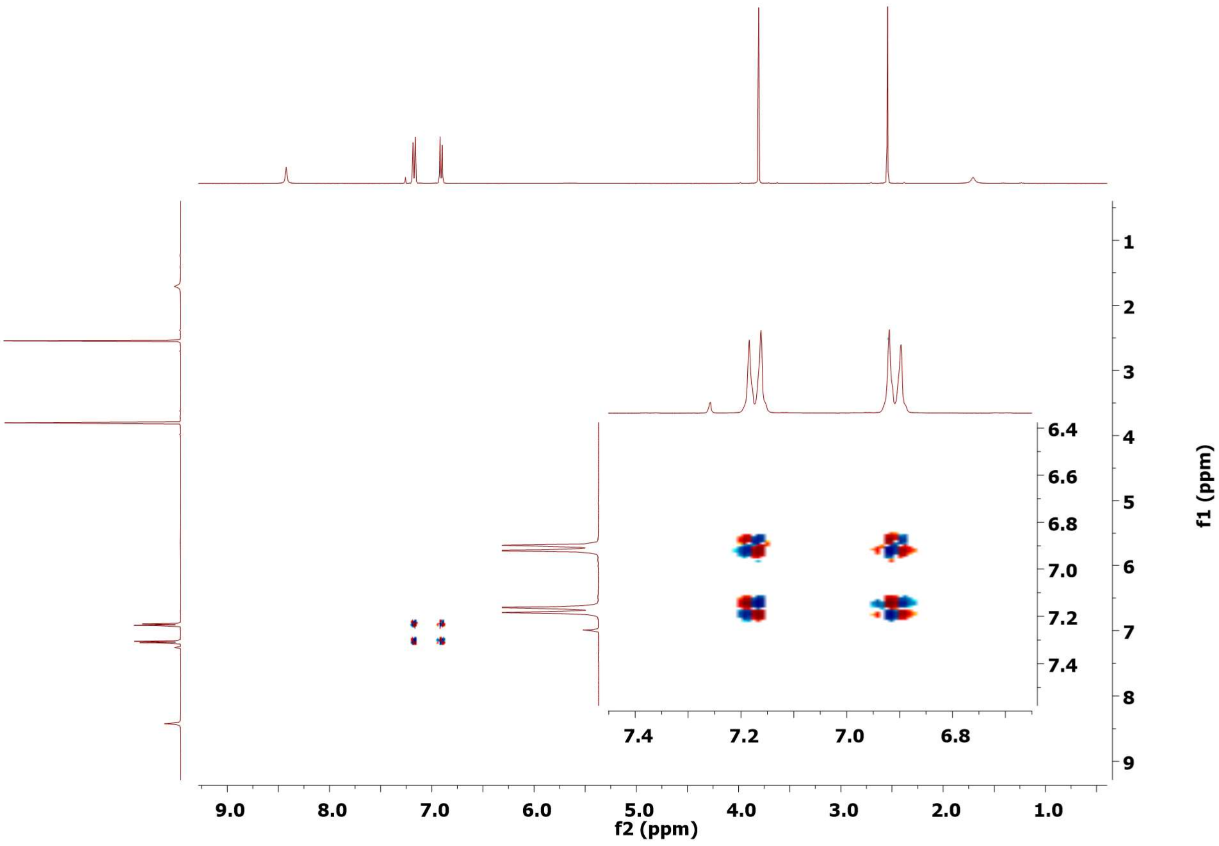

^{1}H-^{13}C-HSQC NMR ($CDCl_3$) spectrum of (Z)-N-(4-methoxyphenyl)-2-oxopropanehydrazonoyl chloride

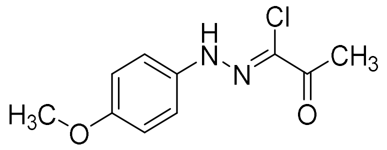

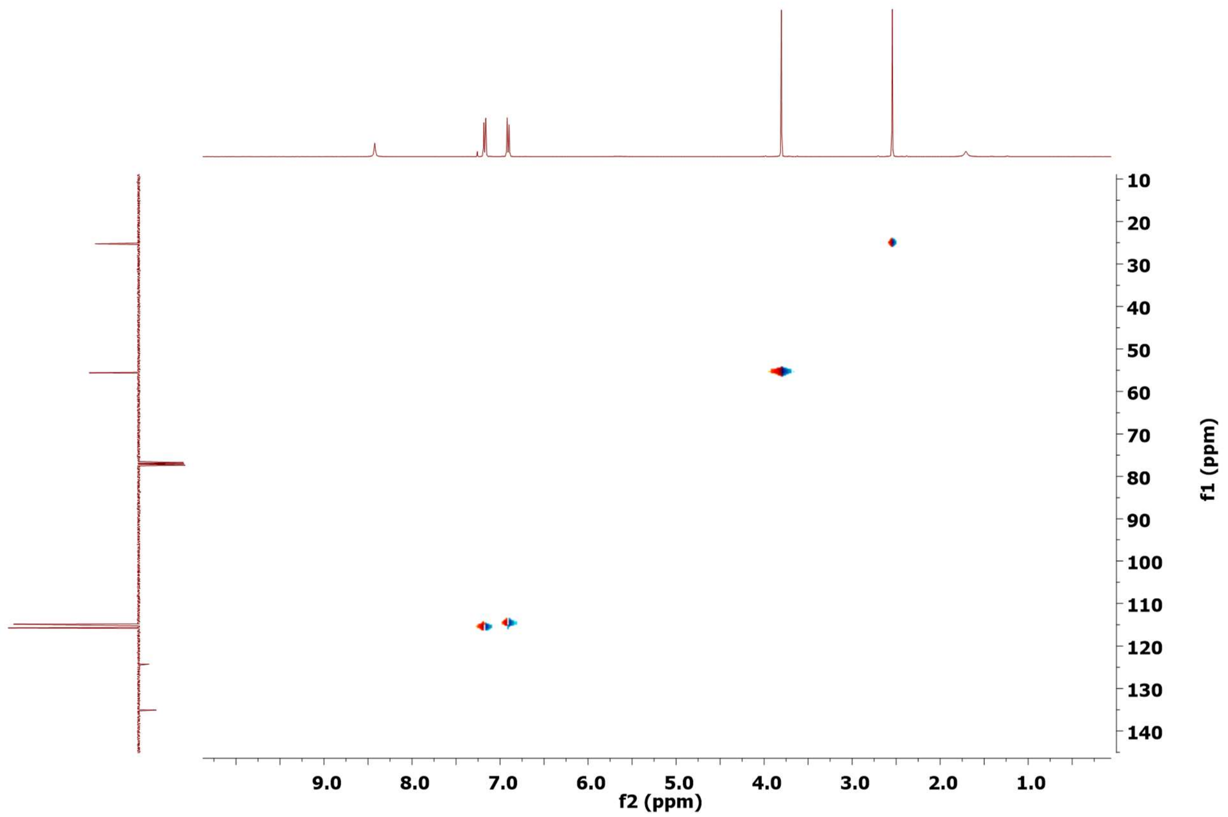

^{1}H-^{13}C-gHMBC NMR ($CDCl_3$) spectrum of (Z)-N-(4-methoxyphenyl)-2-oxopropanehydrazonoyl chloride

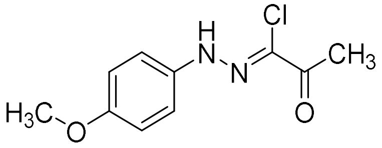

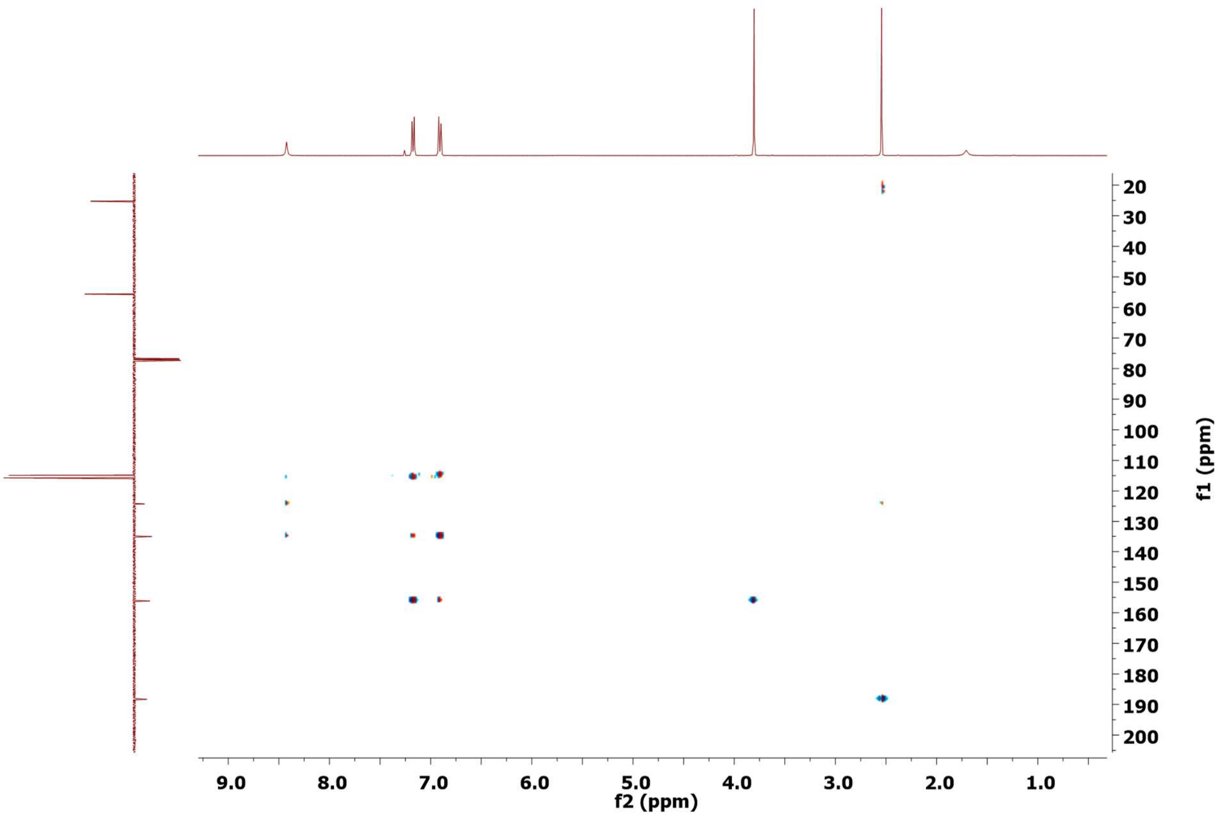

IR spectrum of (Z)-N-(4-methoxyphenyl)-2-oxopropanehydrazonoyl chloride

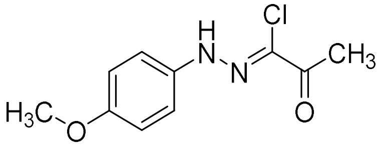

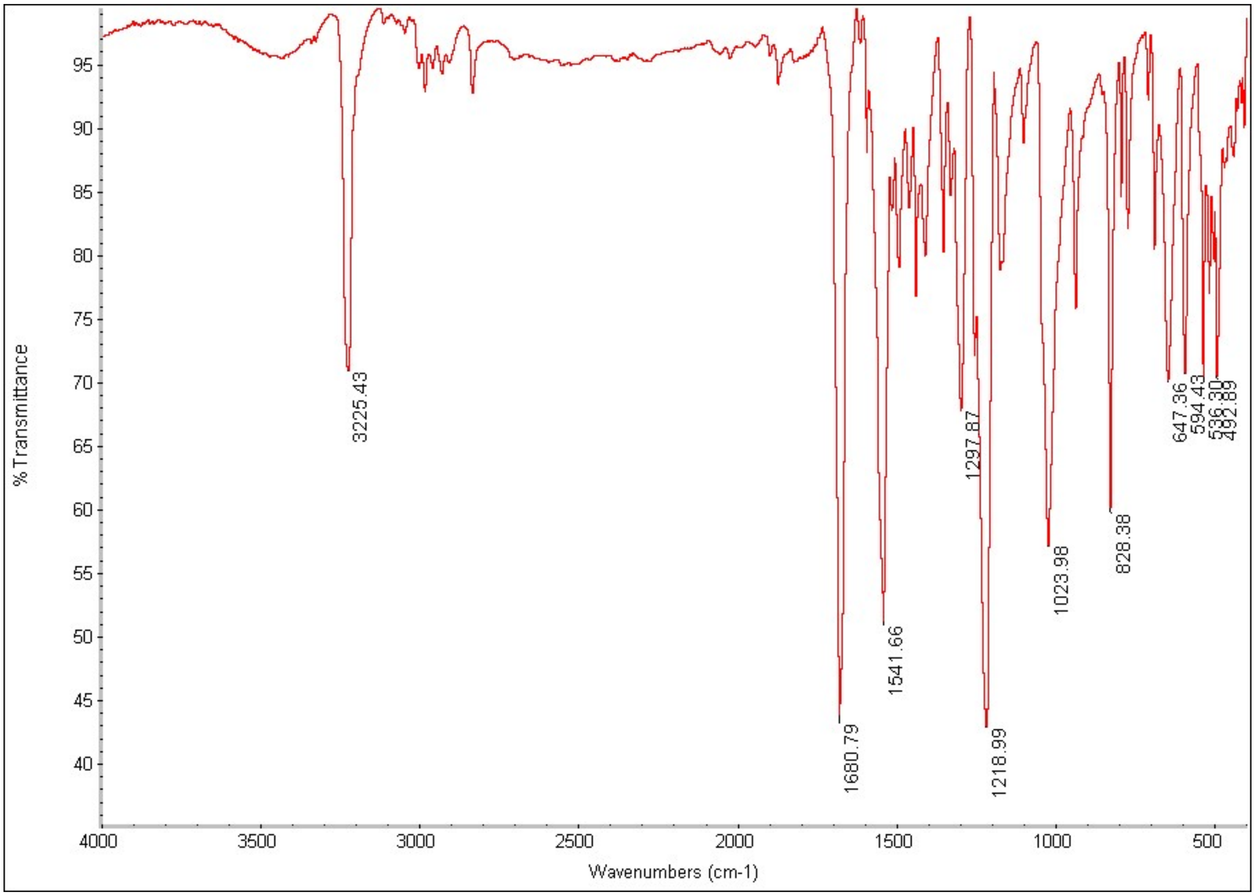

^{1}H NMR (DMSO-d$_6$) spectrum of (Z)-N-(3,5-bis(trifluoromethyl)phenyl)-2-oxopropanehydrazonoyl chloride (**29j**)

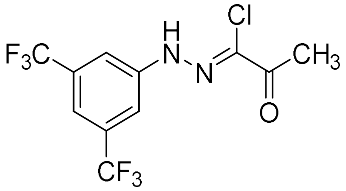

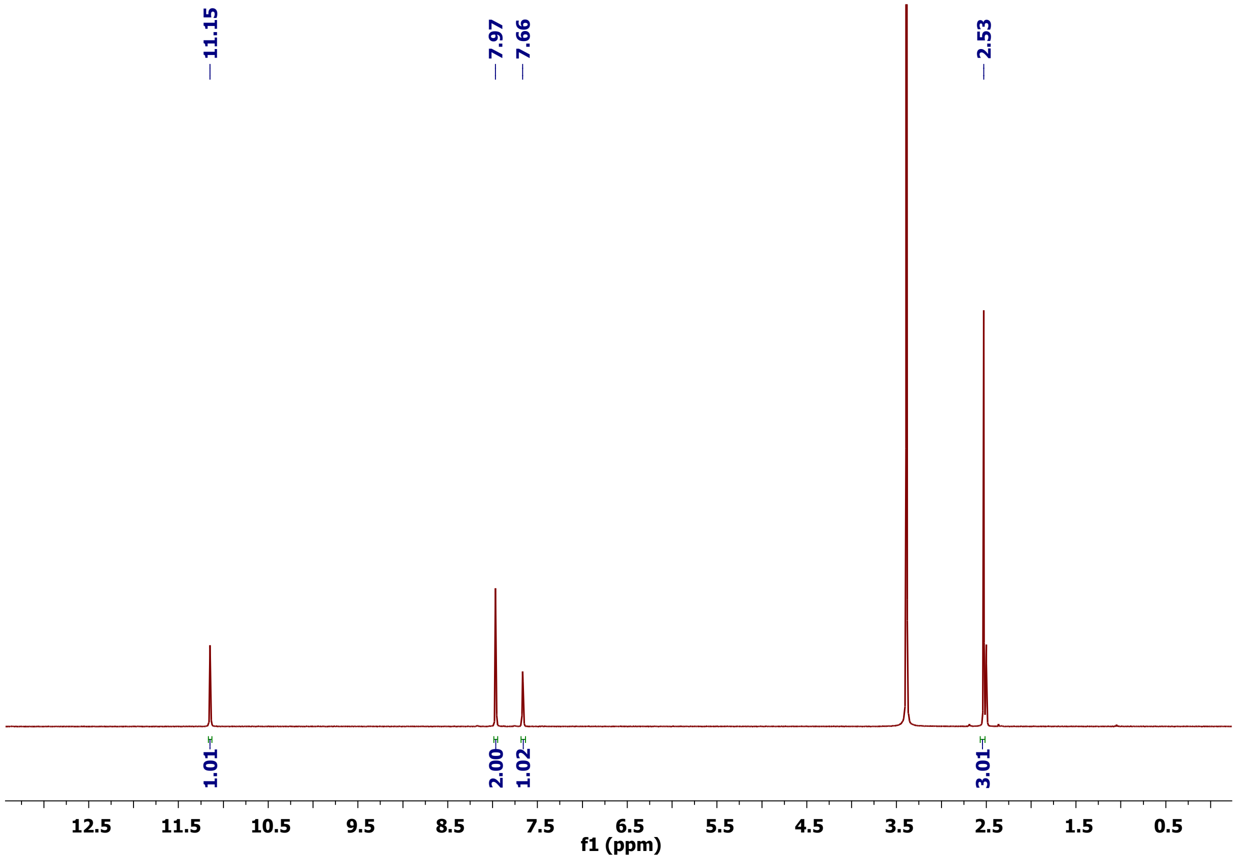

^{13}C NMR (DMSO-d_6) spectrum of (Z)-N-(3,5-bis(trifluoromethyl)phenyl)-2-oxopropanehydrazonoyl chloride

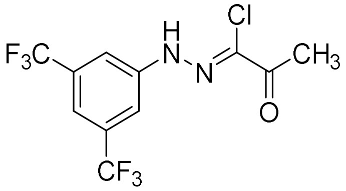

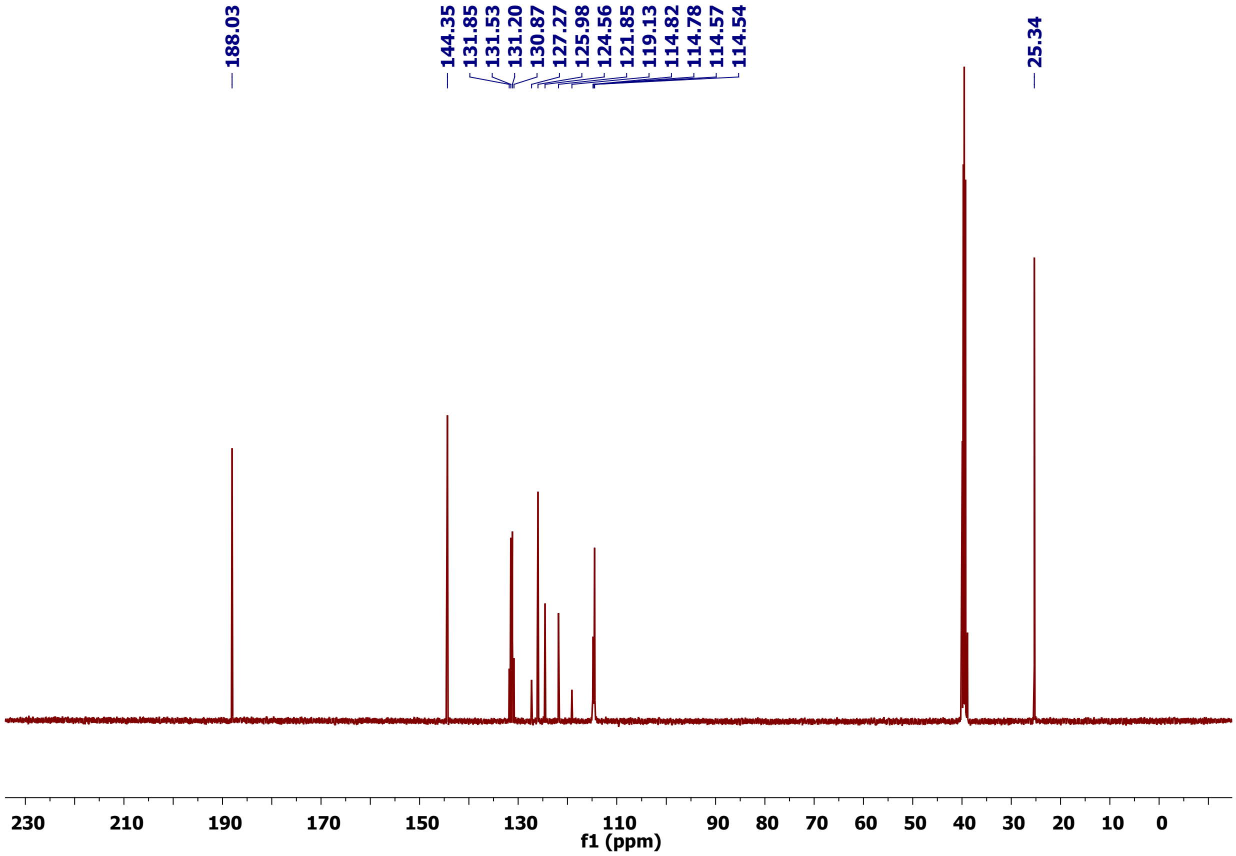

^{13}C CRAPT NMR (DMSO-d_6) spectrum of (Z)-N-(3,5-bis(trifluoromethyl)phenyl)-2-oxopropanehydrazonoyl chloride

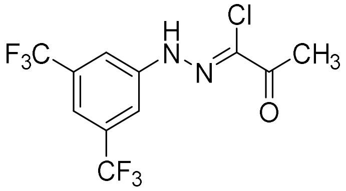

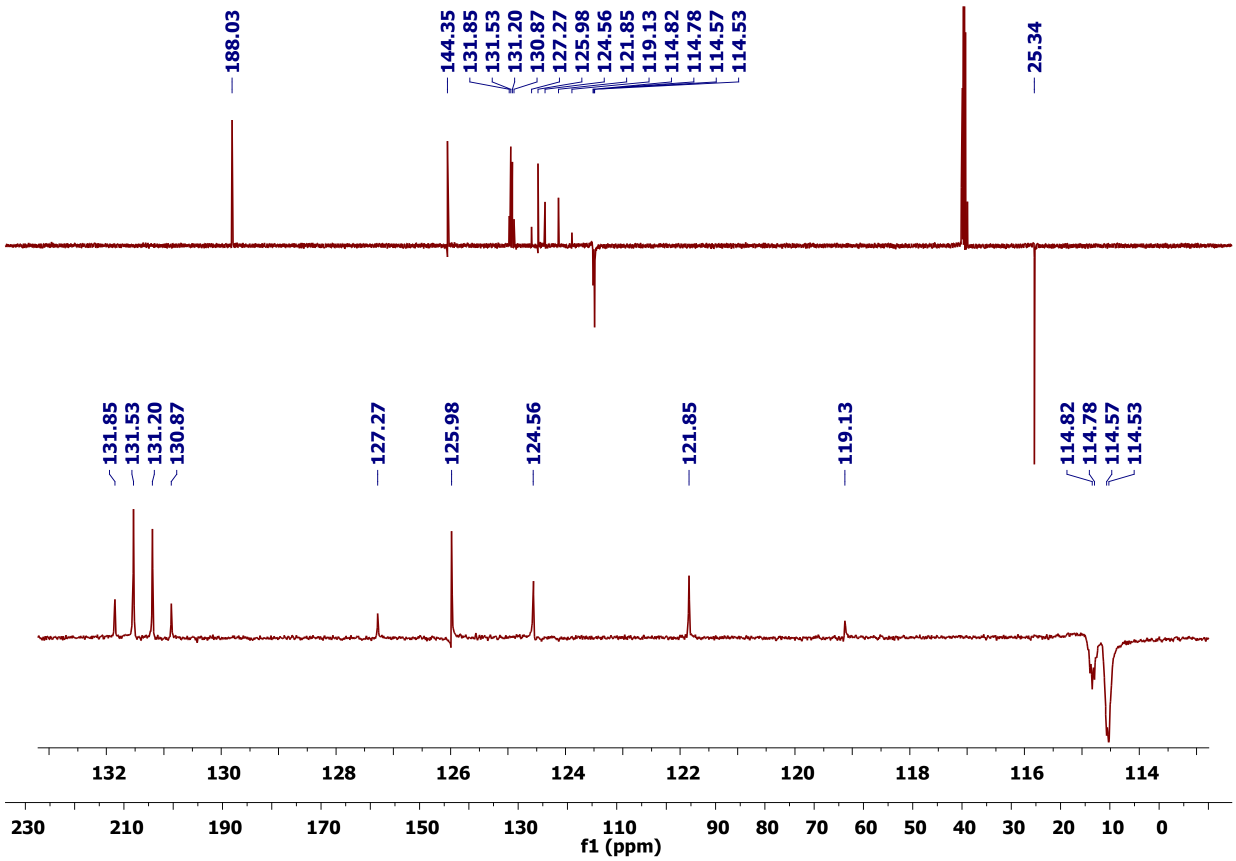

^{1}H-^{1}H-gDQCOSY NMR (DMSO-d_6) spectrum of (Z)-N-(3,5-bis(trifluoromethyl)phenyl)-2-oxopropanehydrazonoyl chloride

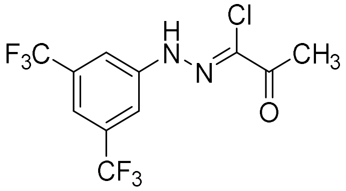

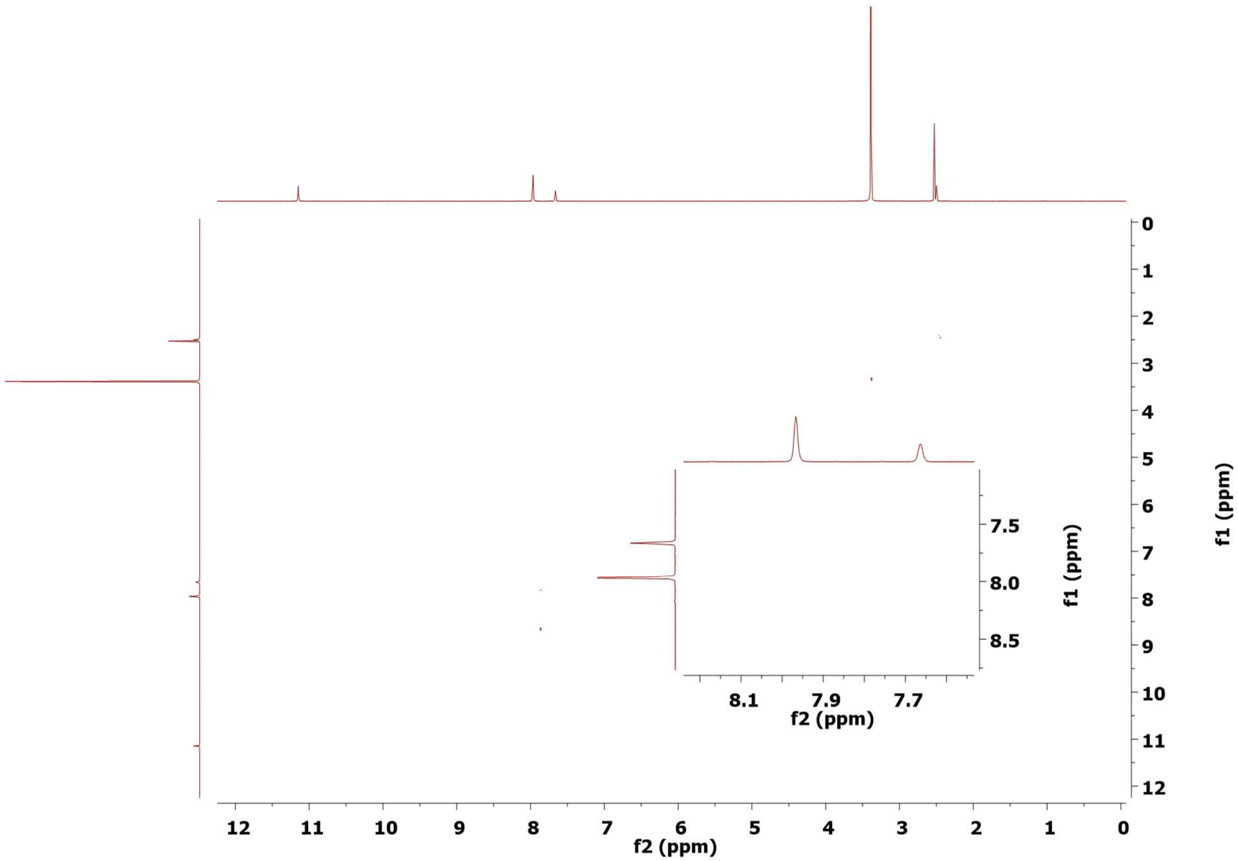

^{1}H-^{13}C-HSQC NMR (DMSO-d$_6$) spectrum of (Z)-N-(3,5-bis(trifluoromethyl)phenyl)-2-oxopropanehydrazonoyl chloride

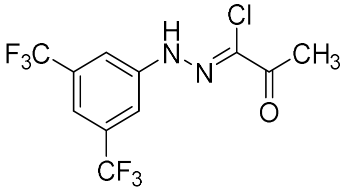

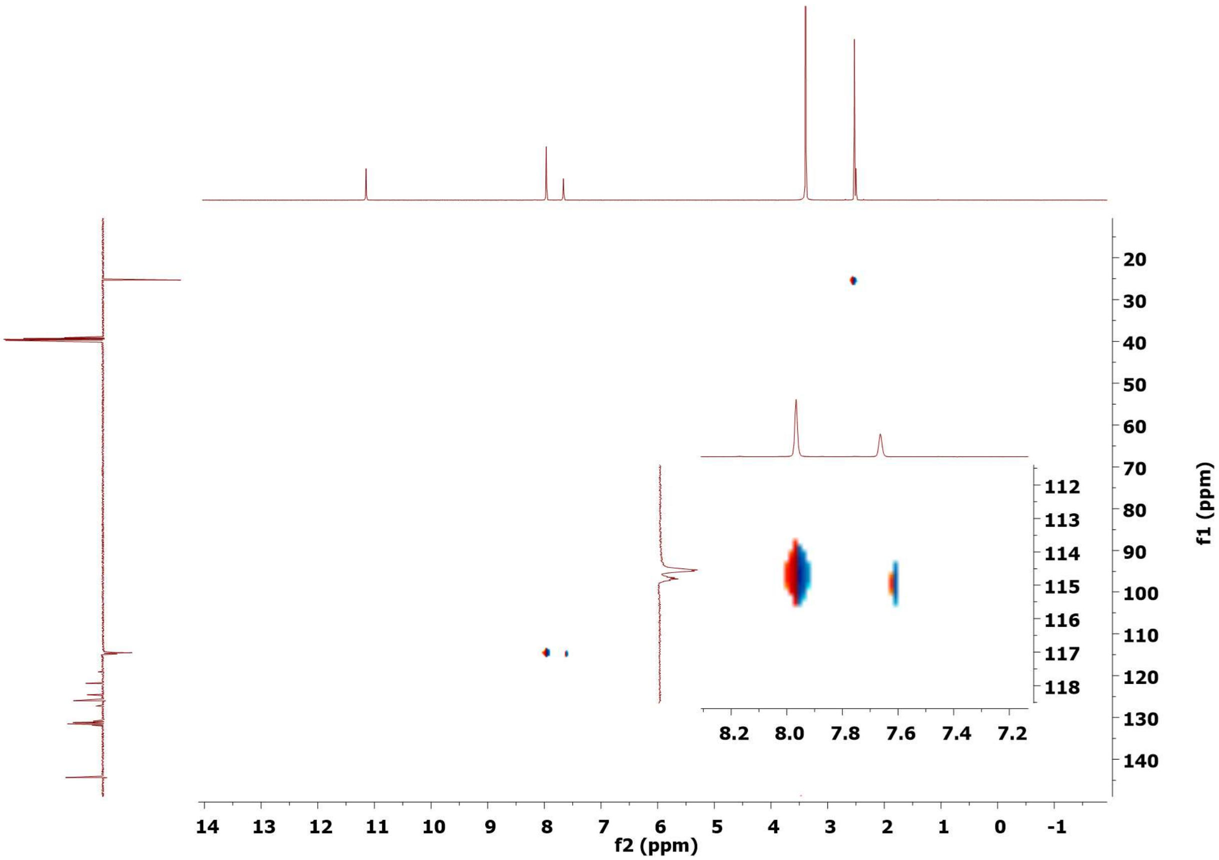

^{1}H-^{13}C-gHMBC NMR (DMSO-d_6) spectrum of (Z)-N-(3,5-bis(trifluoromethyl)phenyl)-2-oxopropanehydrazonoyl chloride

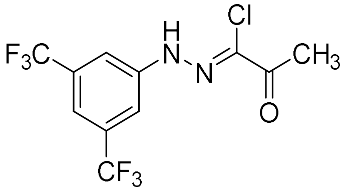

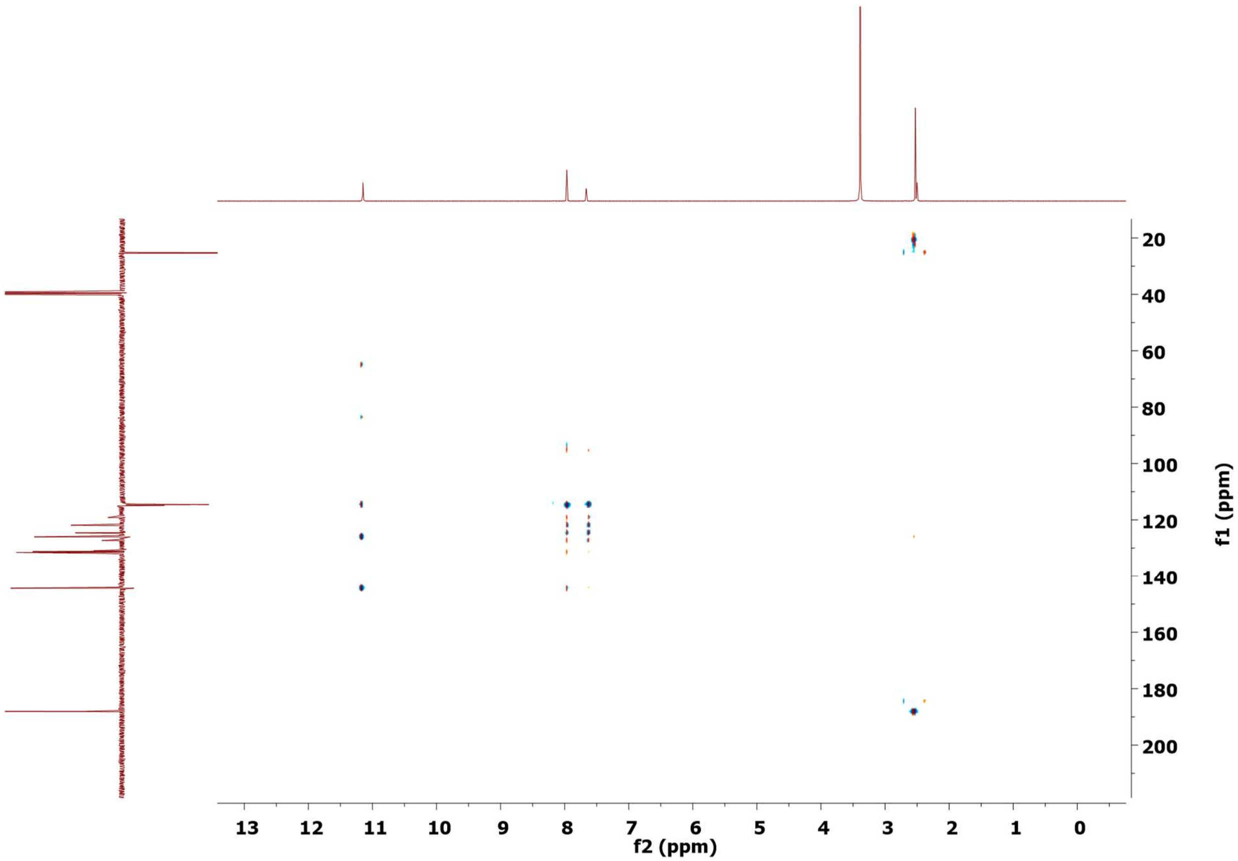

IR spectrum of (Z)-N-(3,5-bis(trifluoromethyl)phenyl)-2-oxopropanehydrazonoyl chloride

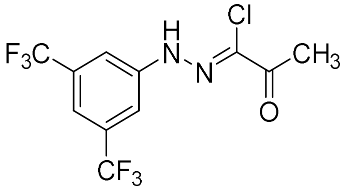

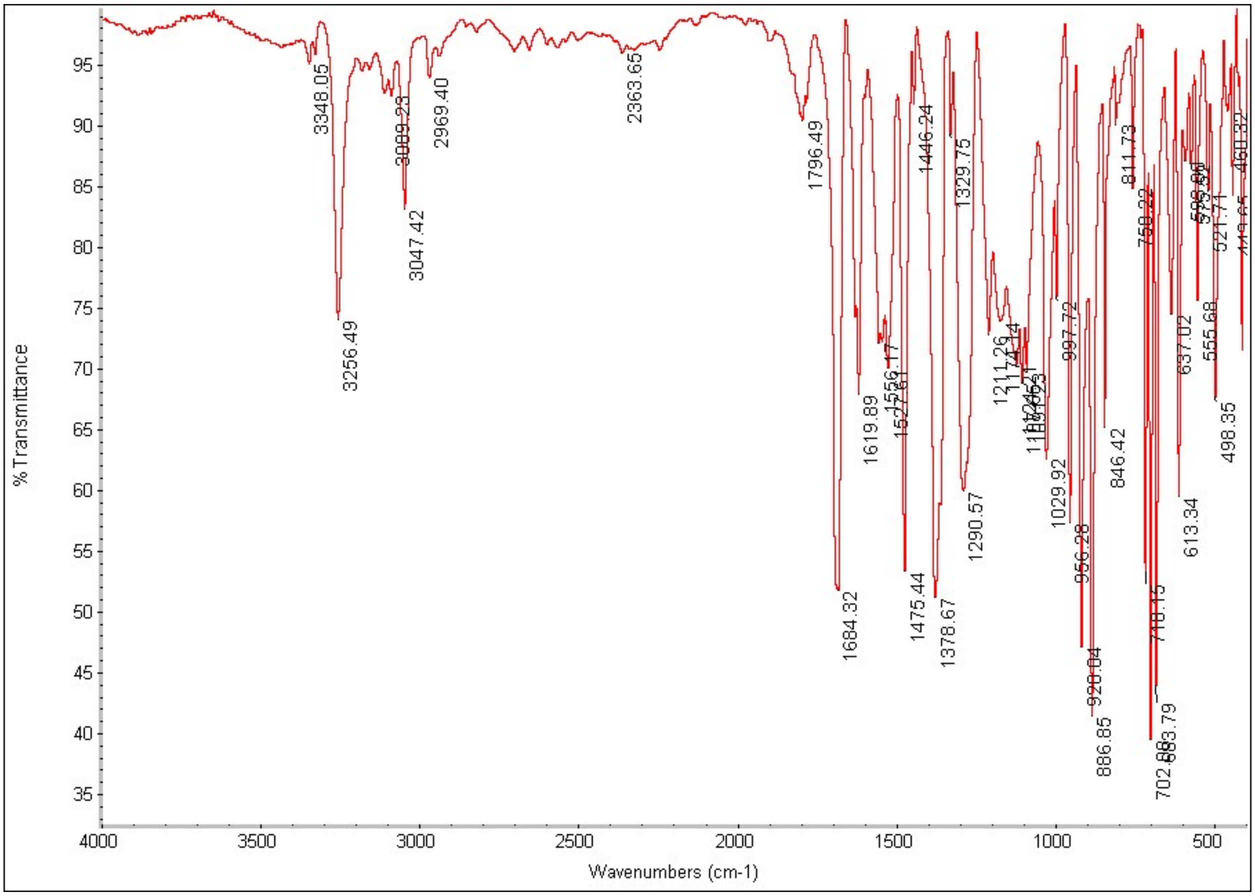

^{1}H NMR ($CDCl_3$) spectrum of (Z)-N-(naphthalen-1-yl)-2-oxopropanehydrazonoyl chloride (**29k**)

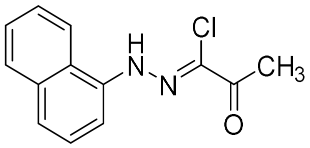

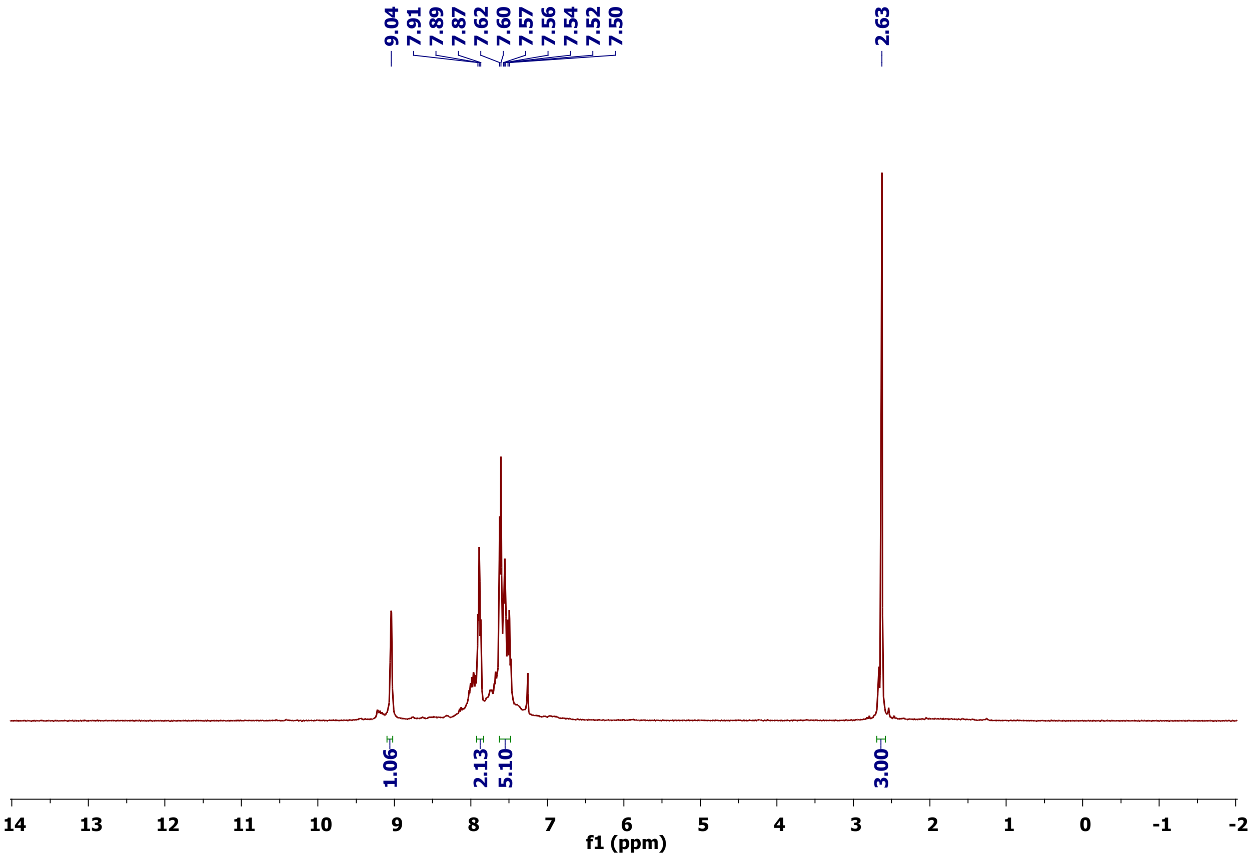

^{13}C NMR ($CDCl_3$) spectrum of (Z)-N-(naphthalen-1-yl)-2-oxopropanehydrazonoyl chloride

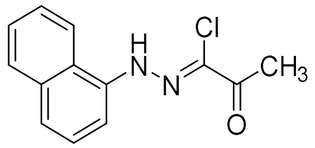

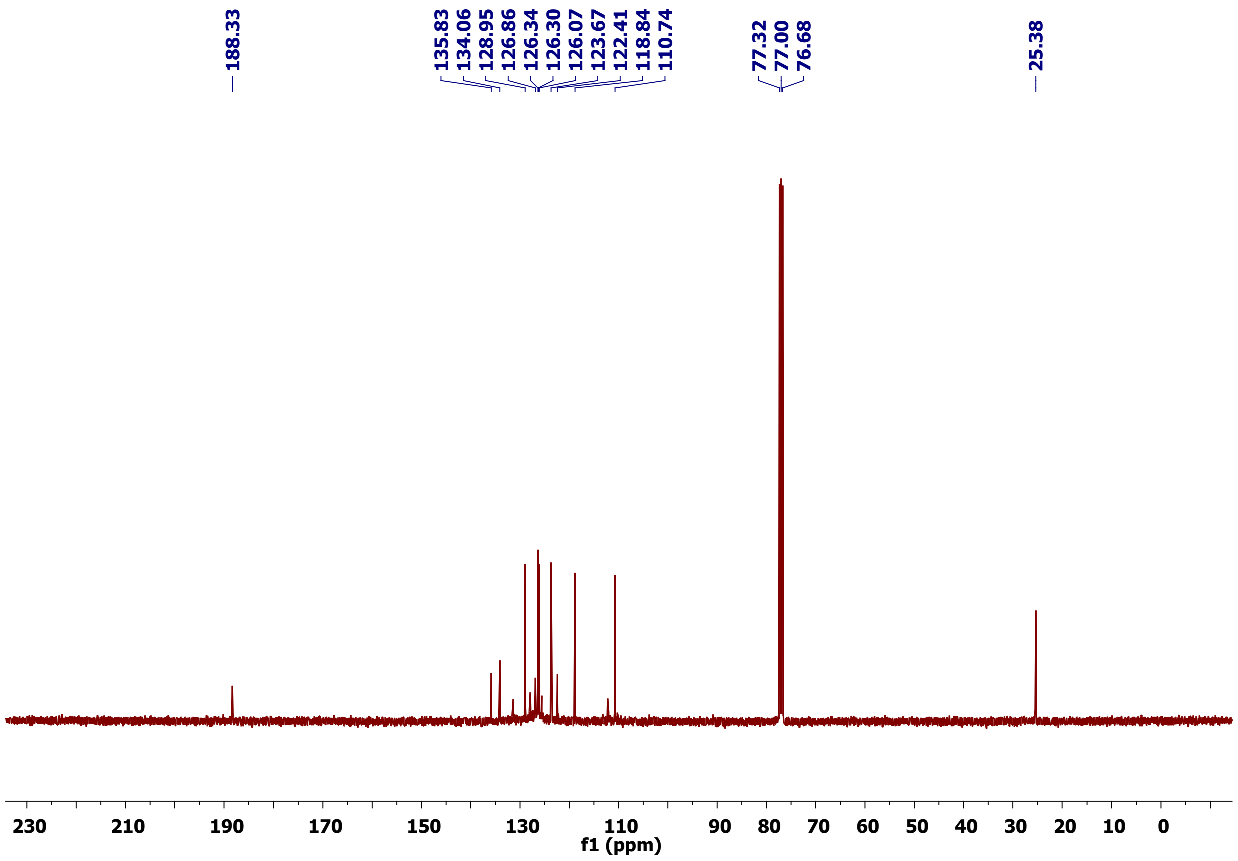

^{13}C CRAPT NMR ($CDCl_3$) spectrum of (Z)-N-(naphthalen-1-yl)-2-oxopropanehydrazonoyl chloride

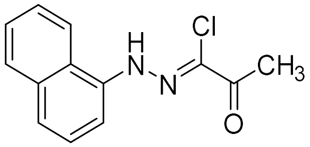

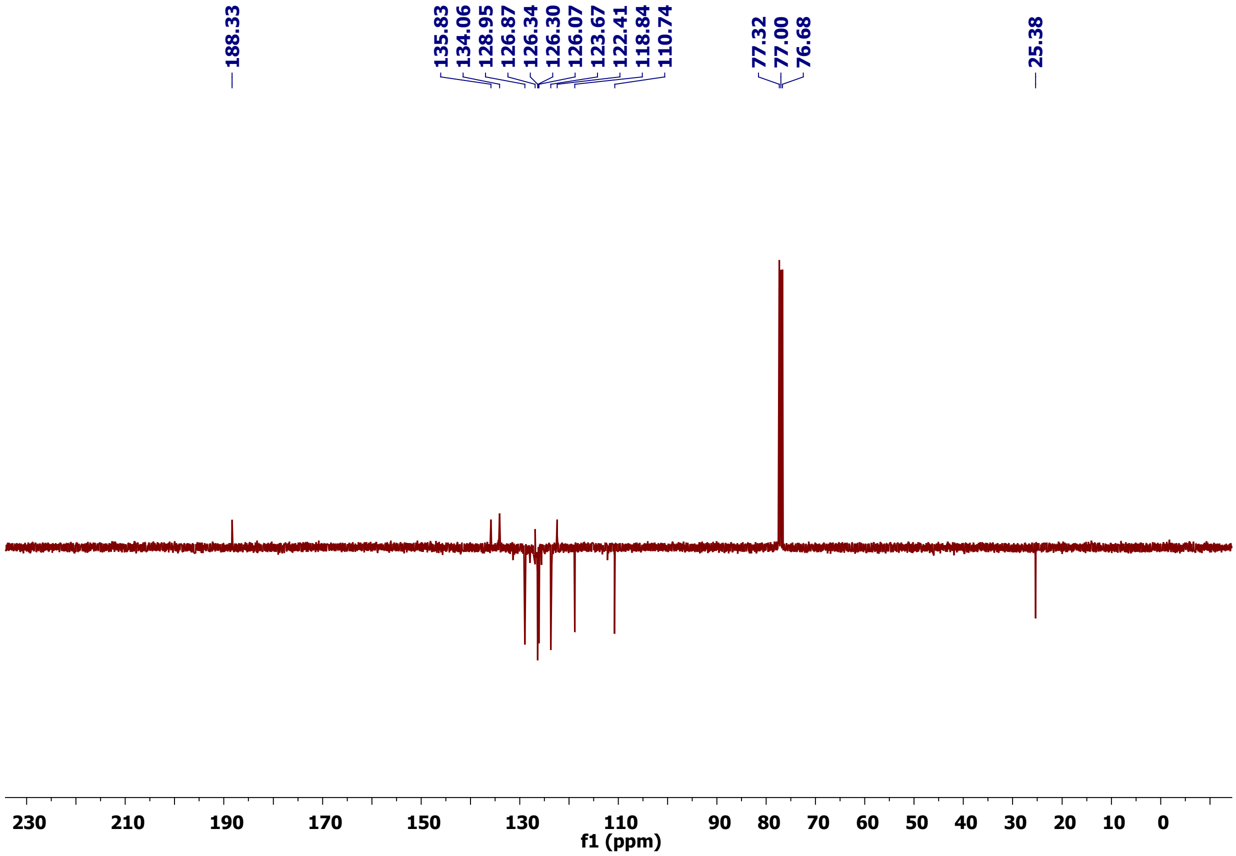

^{1}H-^{1}H-gDQCOSY NMR ($CDCl_3$) spectrum of (Z)-N-(naphthalen-1-yl)-2-oxopropanehydrazonoyl chloride

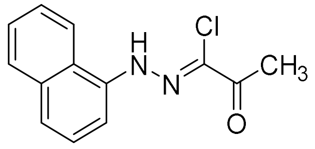

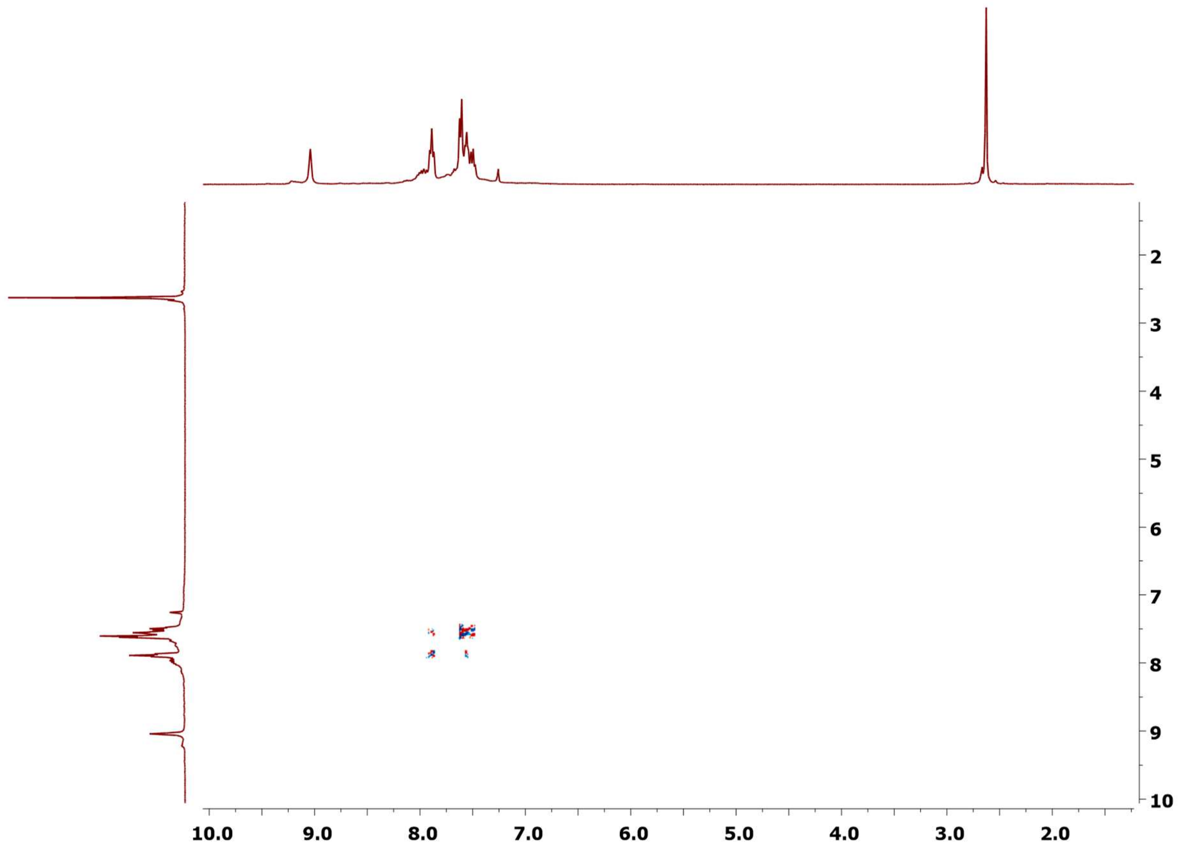

^{1}H-^{13}C-HSQC NMR ($CDCl_3$) spectrum of (Z)-N-(naphthalen-1-yl)-2-oxopropanehydrazonoyl chloride

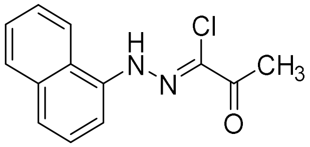

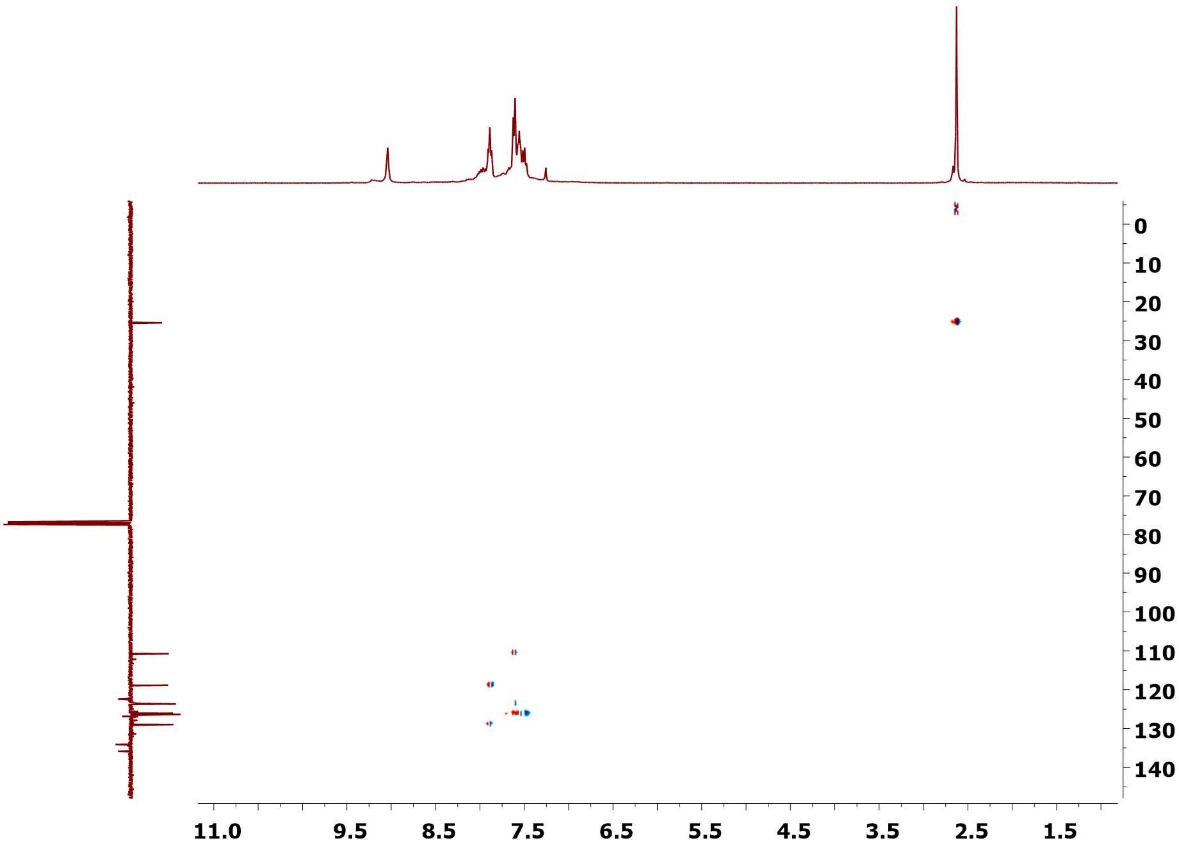

^{1}H-^{13}C-gHMBC NMR ($CDCl_3$) spectrum of (Z)-N-(naphthalen-1-yl)-2-oxopropanehydrazonoyl chloride

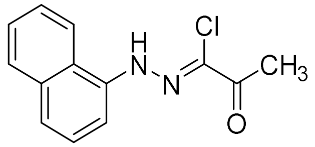

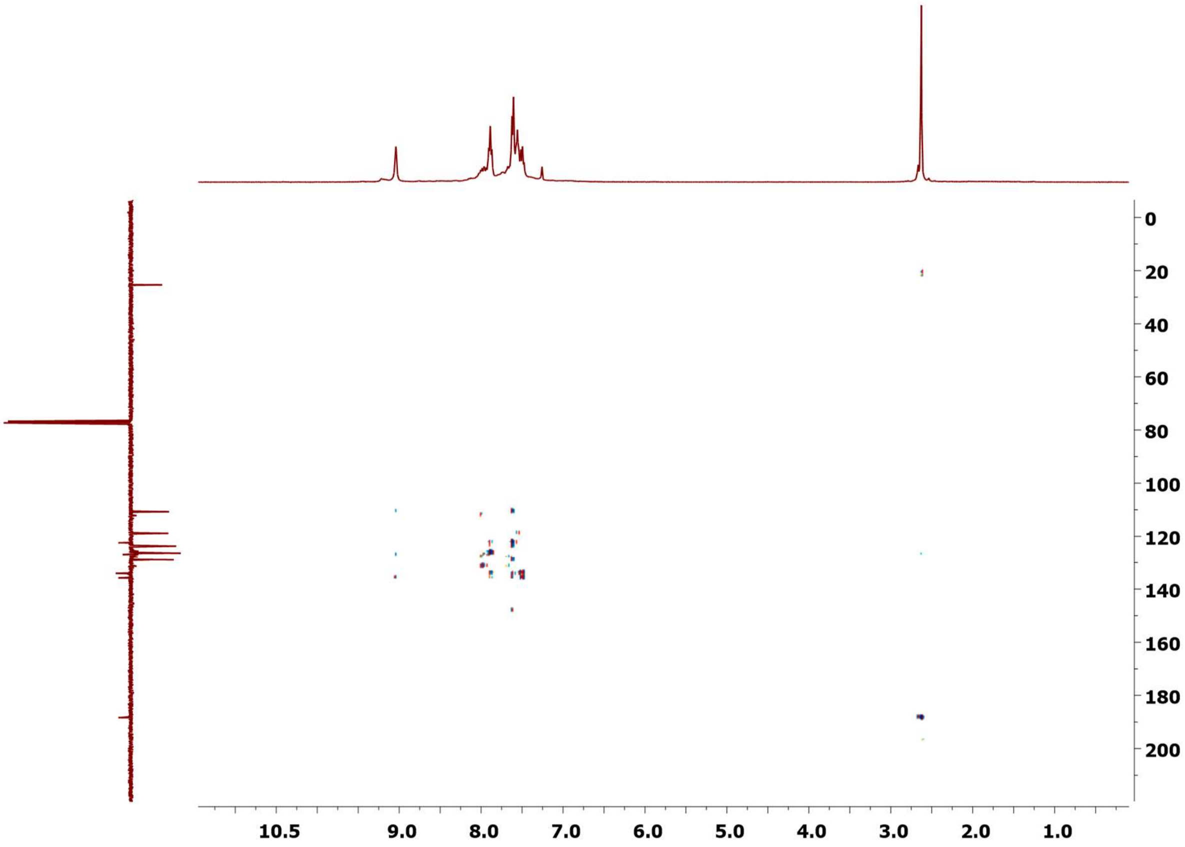

IR spectrum of (Z)-N-(naphthalen-1-yl)-2-oxopropanehydrazonoyl chloride

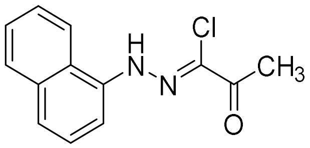

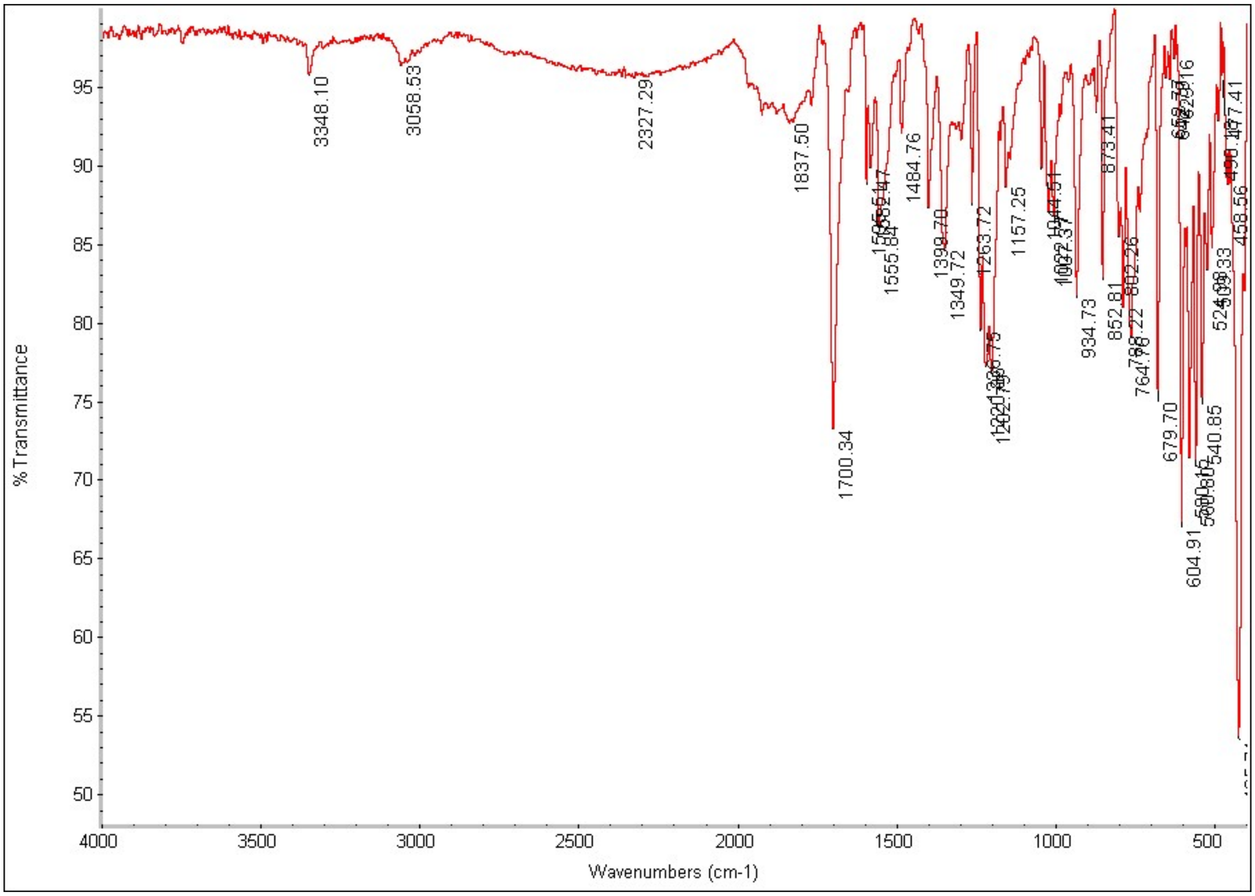

^{1}H NMR ($CDCl_3$) spectrum of (Z)-N-(2,3-difluorophenyl)-2-oxopropanehydrazonoyl chloride (**29l**)

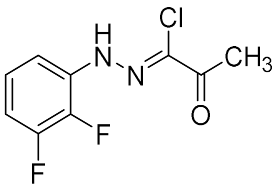

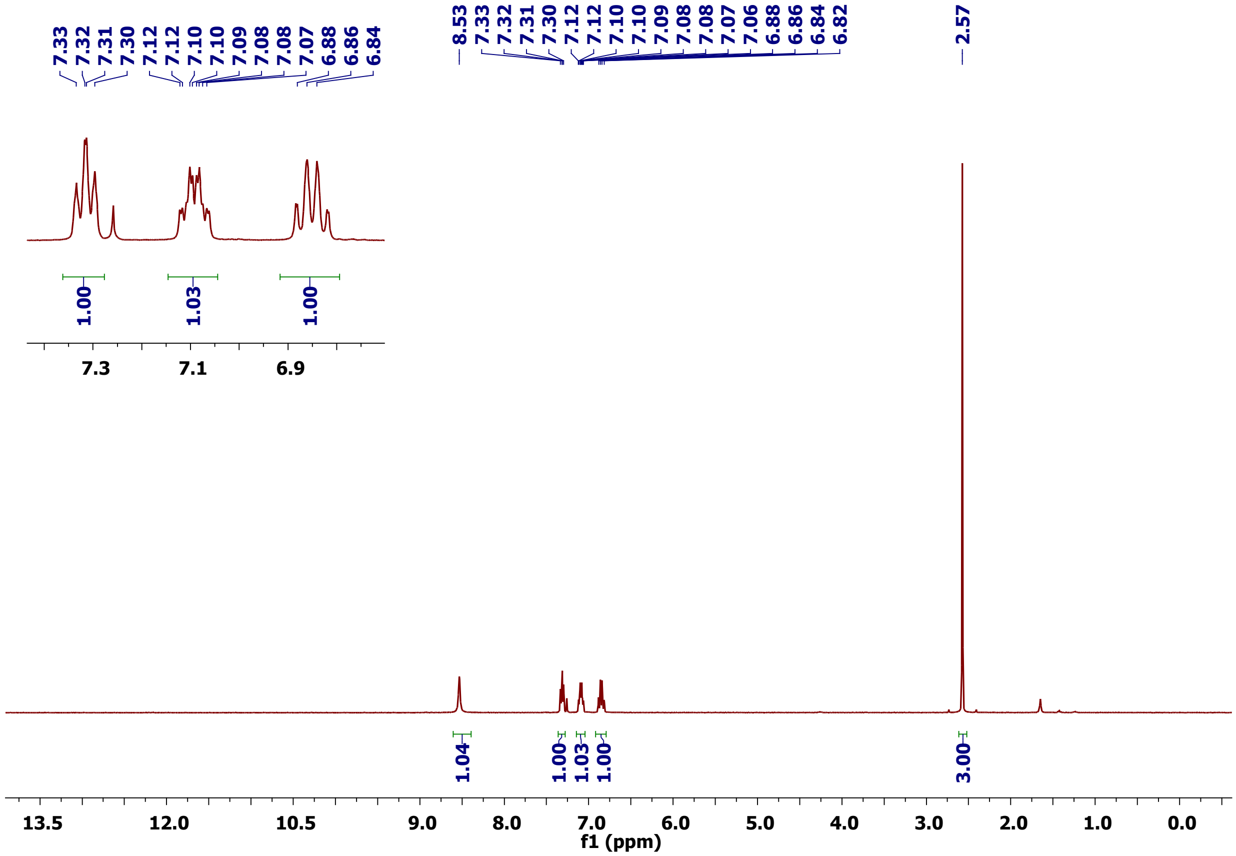

^{13}C NMR (CDCl$_3$) spectrum of (Z)-N-(2,3-difluorophenyl)-2-oxopropanehydrazonoyl chloride

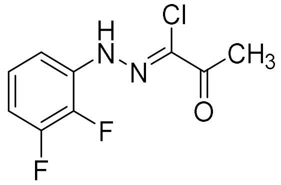

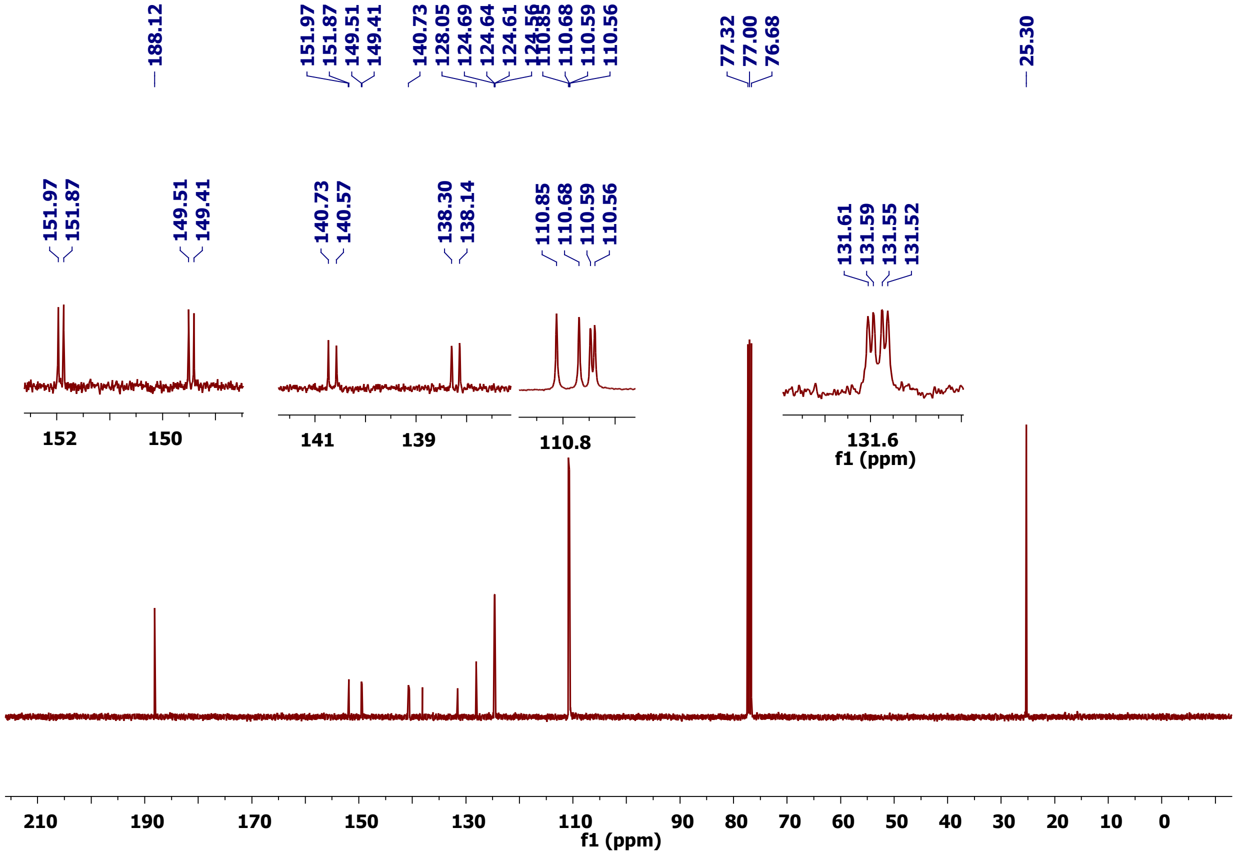

^{13}C CRAPT NMR ($CDCl_3$) spectrum of (Z)-N-(2,3-difluorophenyl)-2-oxopropanehydrazonoyl chloride

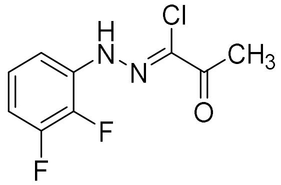

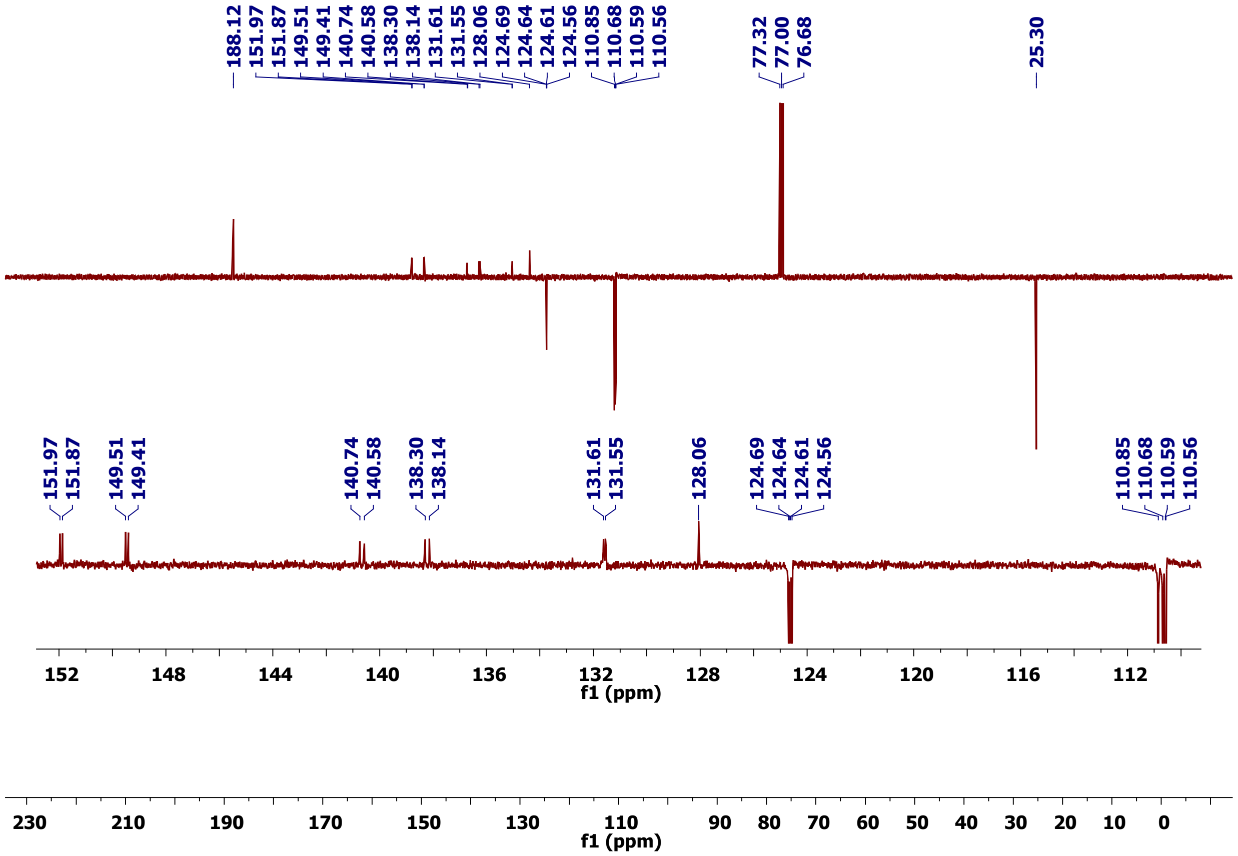

^{1}H-^{1}H-gDQCOSY NMR ($CDCl_3$) spectrum of (Z)-N-(2,3-difluorophenyl)-2-oxopropanehydrazonoyl chloride

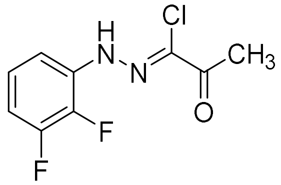

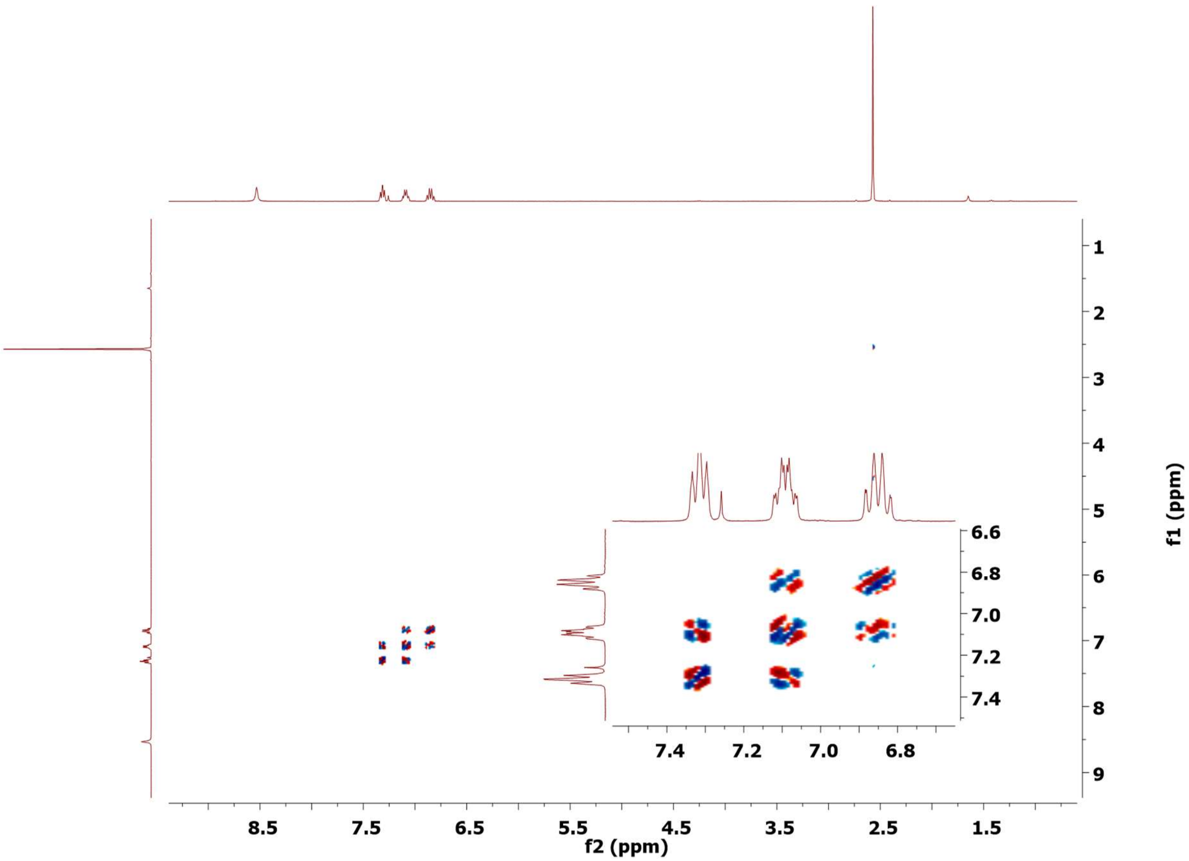

^{1}H-^{13}C-HSQC NMR ($CDCl_3$) spectrum of (Z)-N-(2,3-difluorophenyl)-2-oxopropanehydrazonoyl chloride

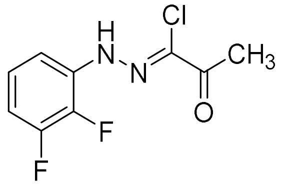

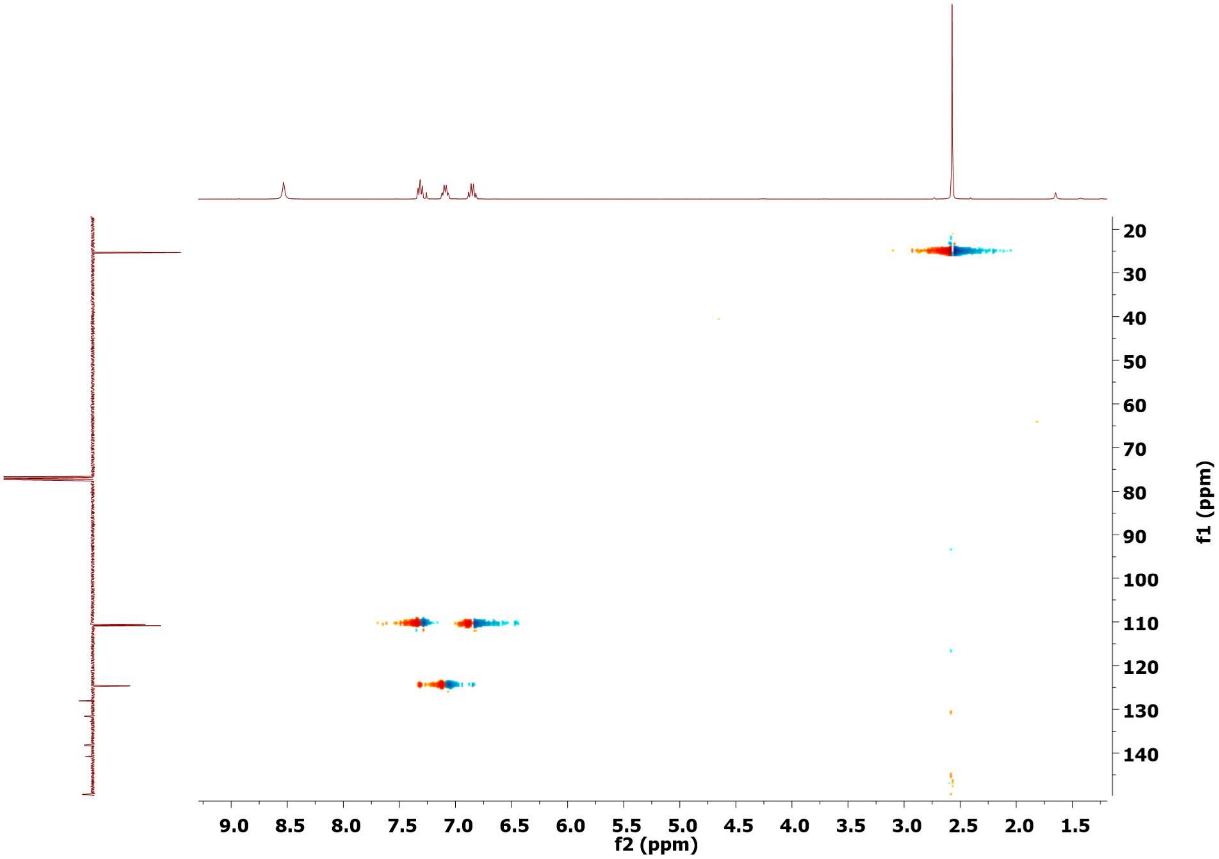

^{1}H-^{13}C-gHMBC NMR ($CDCl_3$) spectrum of (Z)-N-(2,3-difluorophenyl)-2-oxopropanehydrazonoyl chloride

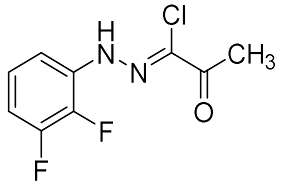

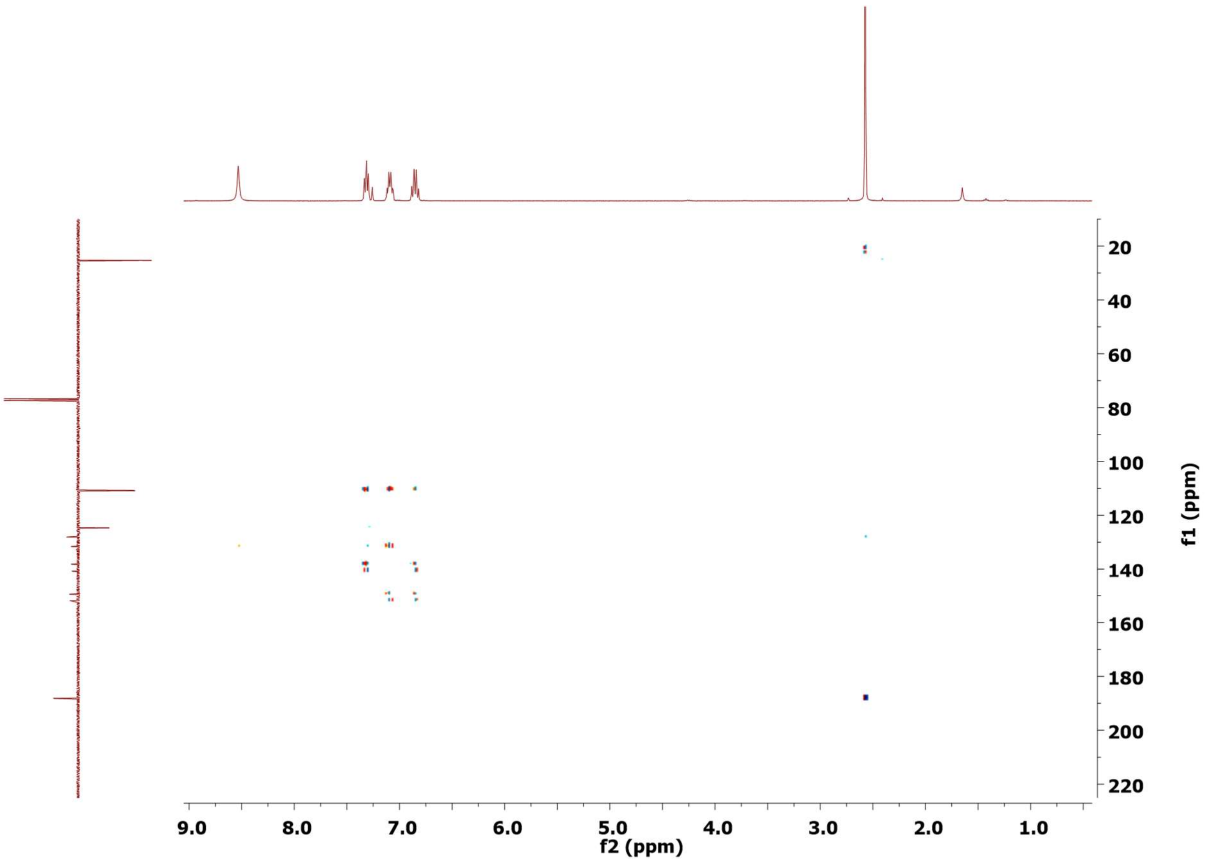

IR spectrum of (Z)-N-(2,3-difluorophenyl)-2-oxopropanehydrazonoyl chloride

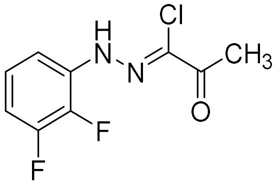

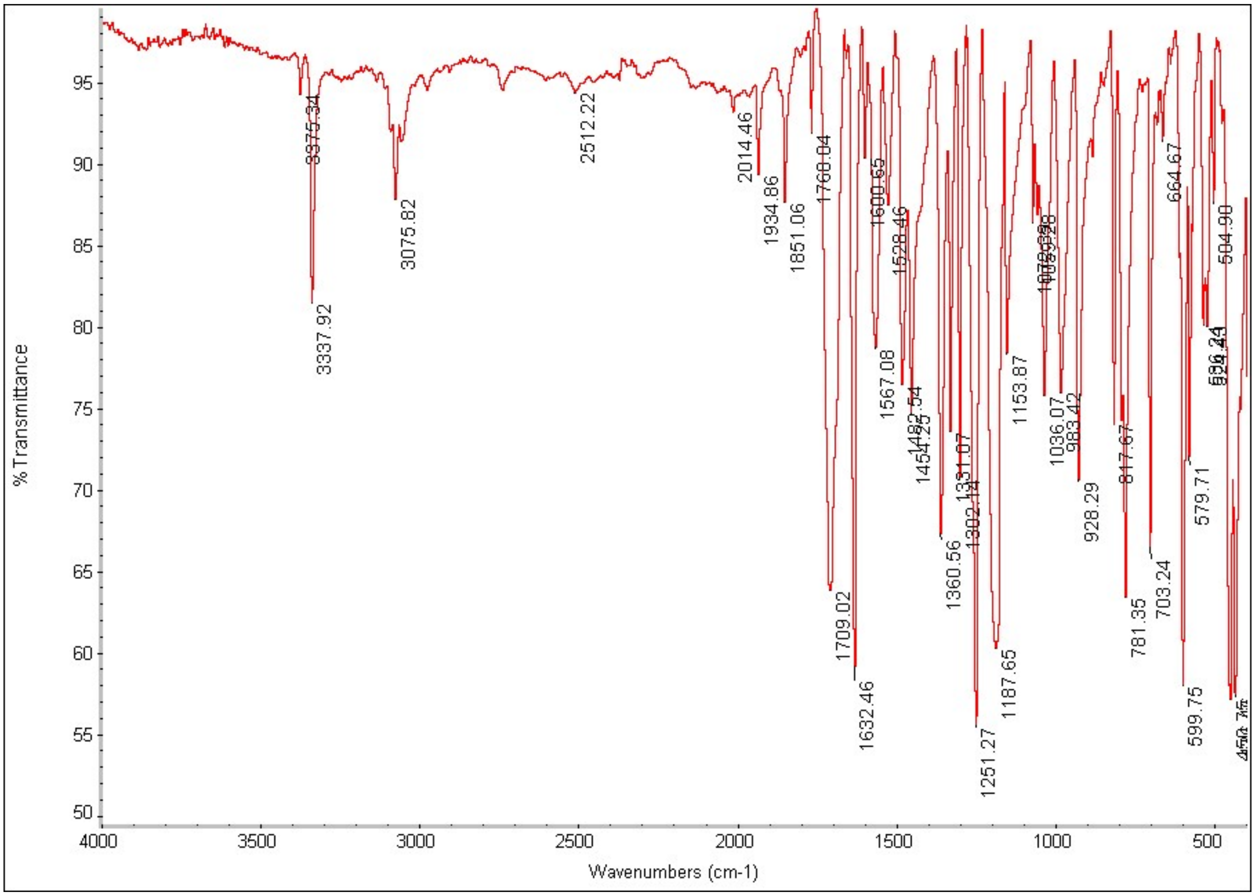

^{1}H NMR ($CDCl_3$) spectrum of (Z)-N-(benzo[d][1,3]dioxol-5-yl)-2-oxopropanehydrazonoyl chloride (**29m**)

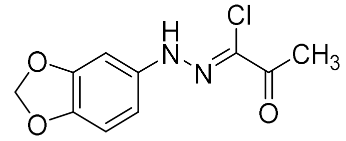

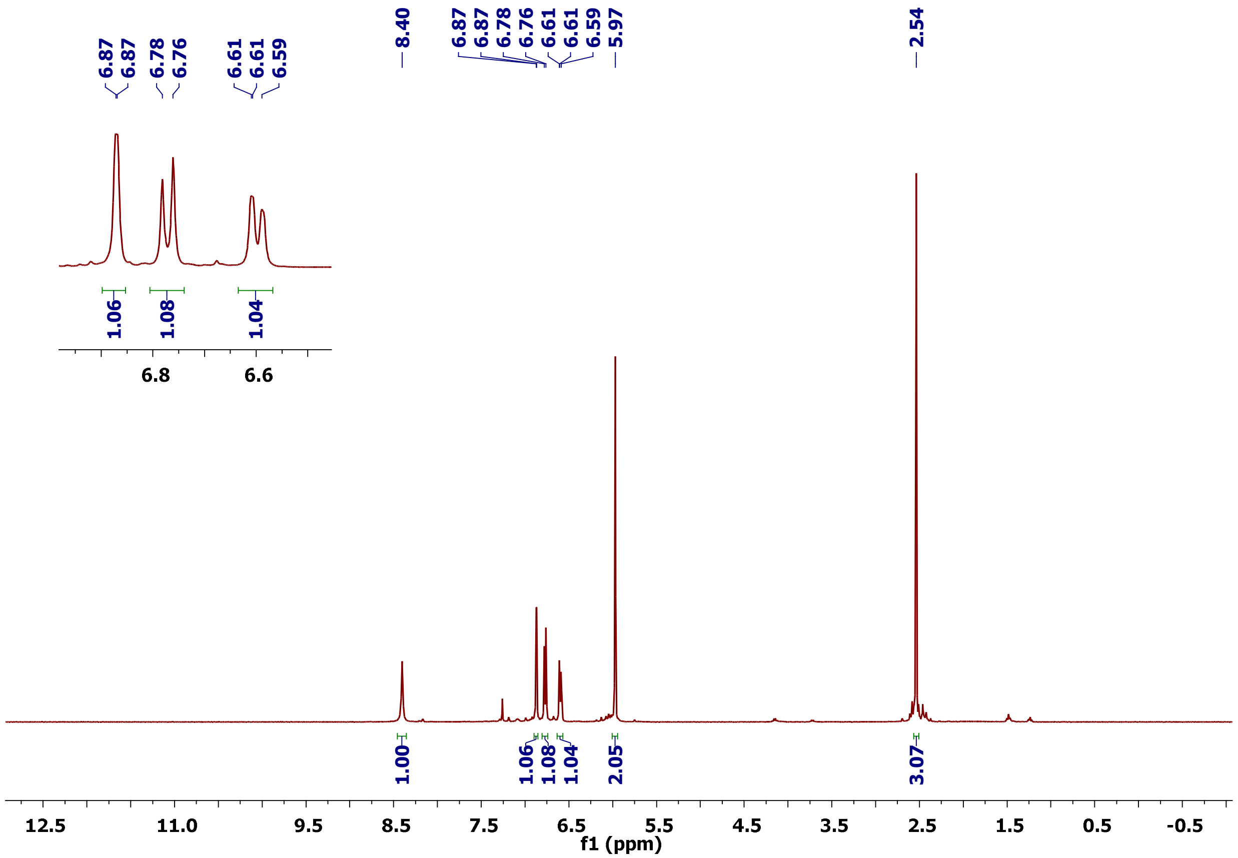

^{13}C NMR ($CDCl_3$) spectrum of (Z)-N-(benzo[d][1,3]dioxol-5-yl)-2-oxopropanehydrazonoyl chloride

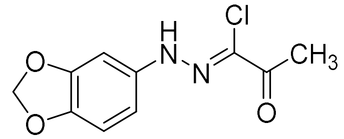

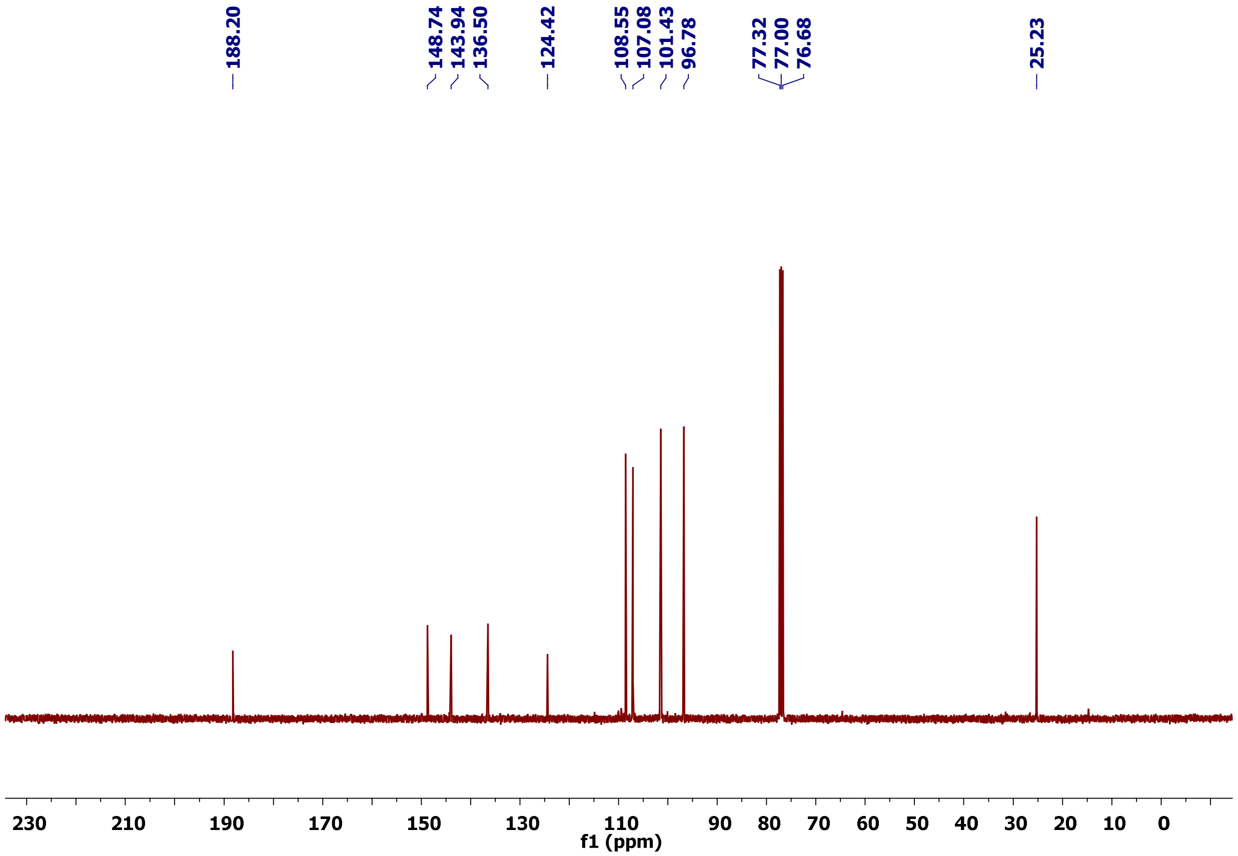

^{13}C CRAPT NMR ($CDCl_3$) spectrum of (Z)-N-(benzo[d][1,3]dioxol-5-yl)-2-oxopropanehydrazonoyl chloride

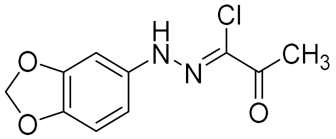

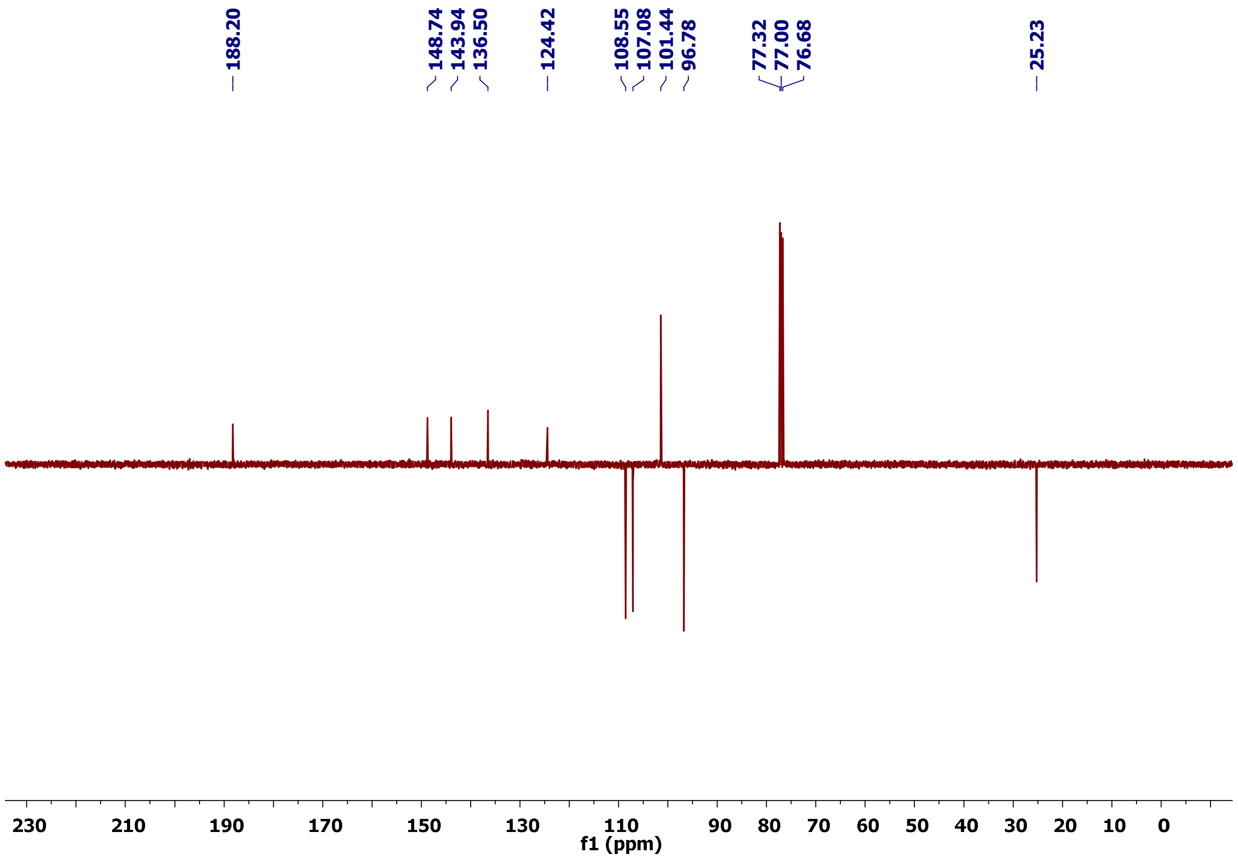

^{1}H-^{1}H-gDQCOSY NMR ($CDCl_3$) spectrum of (Z)-N-(benzo[d][1,3]dioxol-5-yl)-2-oxopropanehydrazonoyl chloride

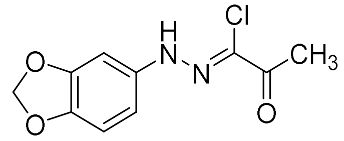

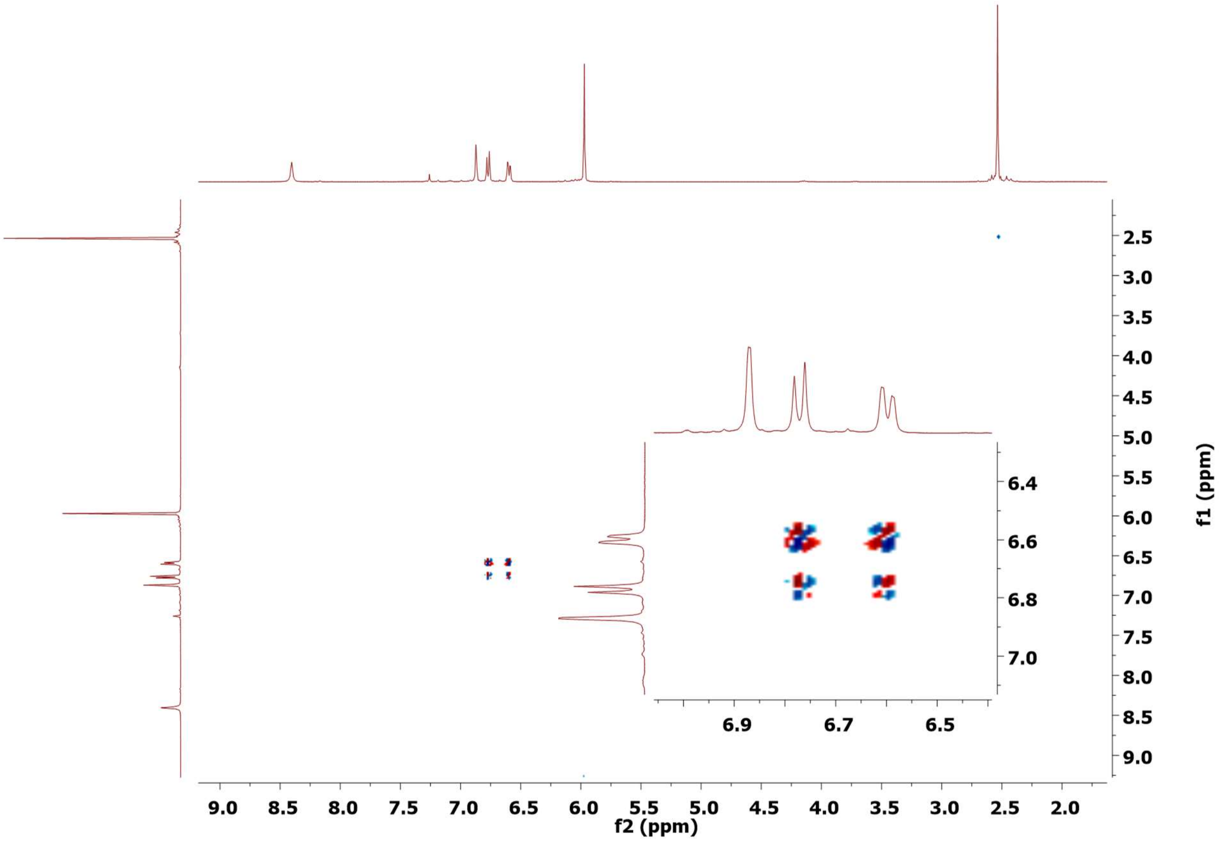

^{1}H-^{13}C-HSQC NMR ($CDCl_3$) spectrum of (Z)-N-(benzo[d][1,3]dioxol-5-yl)-2-oxopropanehydrazonoyl chloride

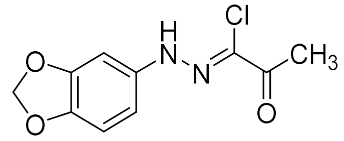

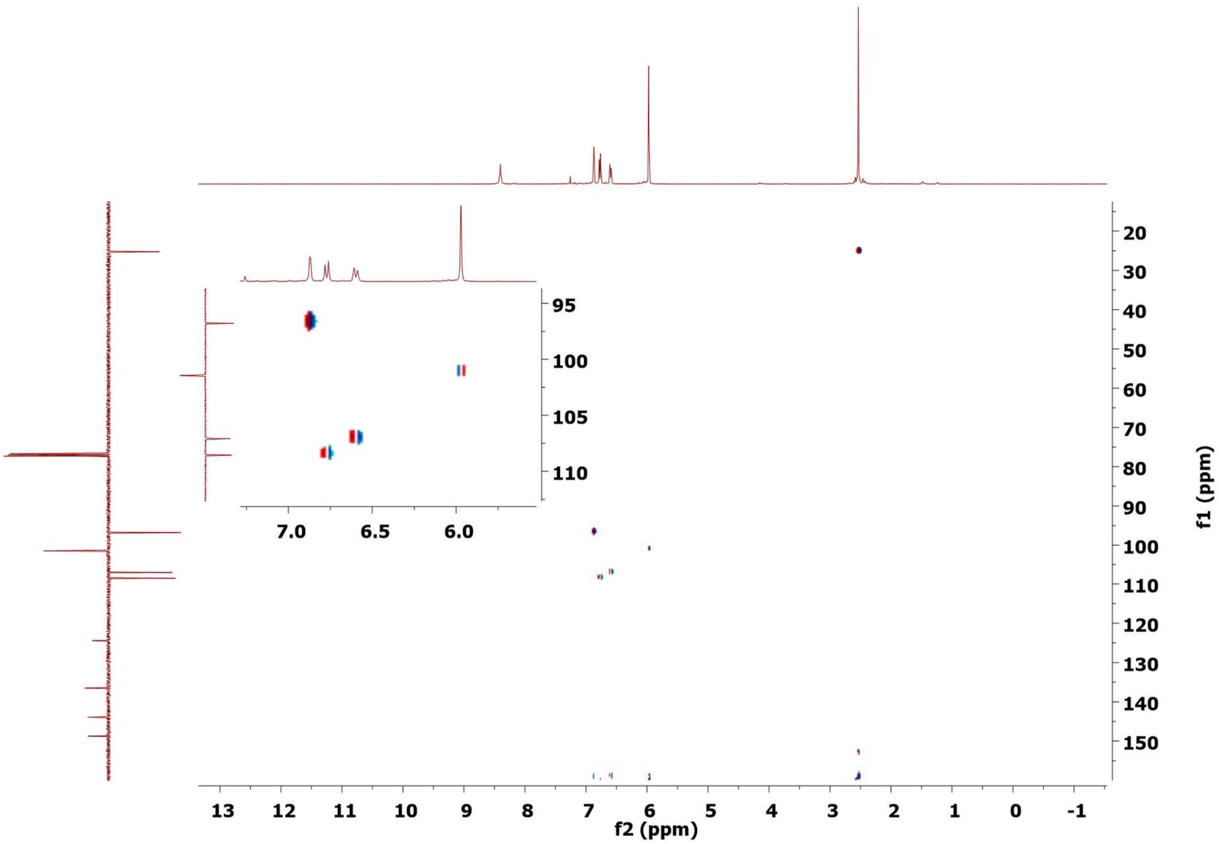

^{1}H-^{13}C-gHMBC NMR ($CDCl_3$) spectrum of (Z)-N-(benzo[d][1,3]dioxol-5-yl)-2-oxopropanehydrazonoyl chloride

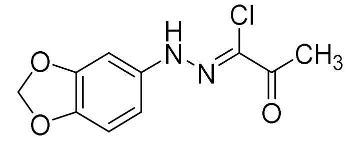

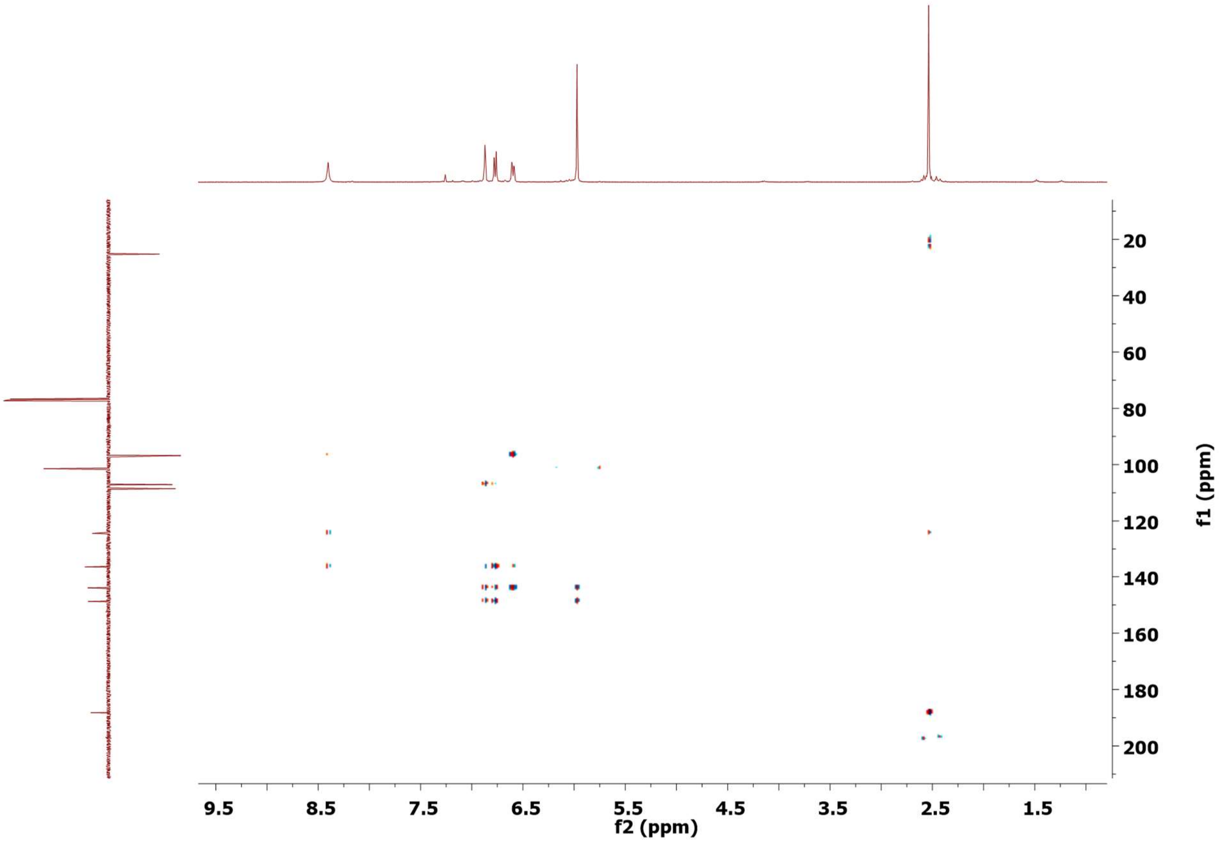

IR spectrum of (Z)-N-(benzo[d][1,3]dioxol-5-yl)-2-oxopropanehydrazonoyl chloride

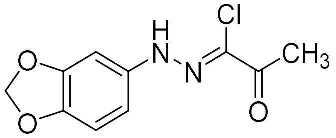

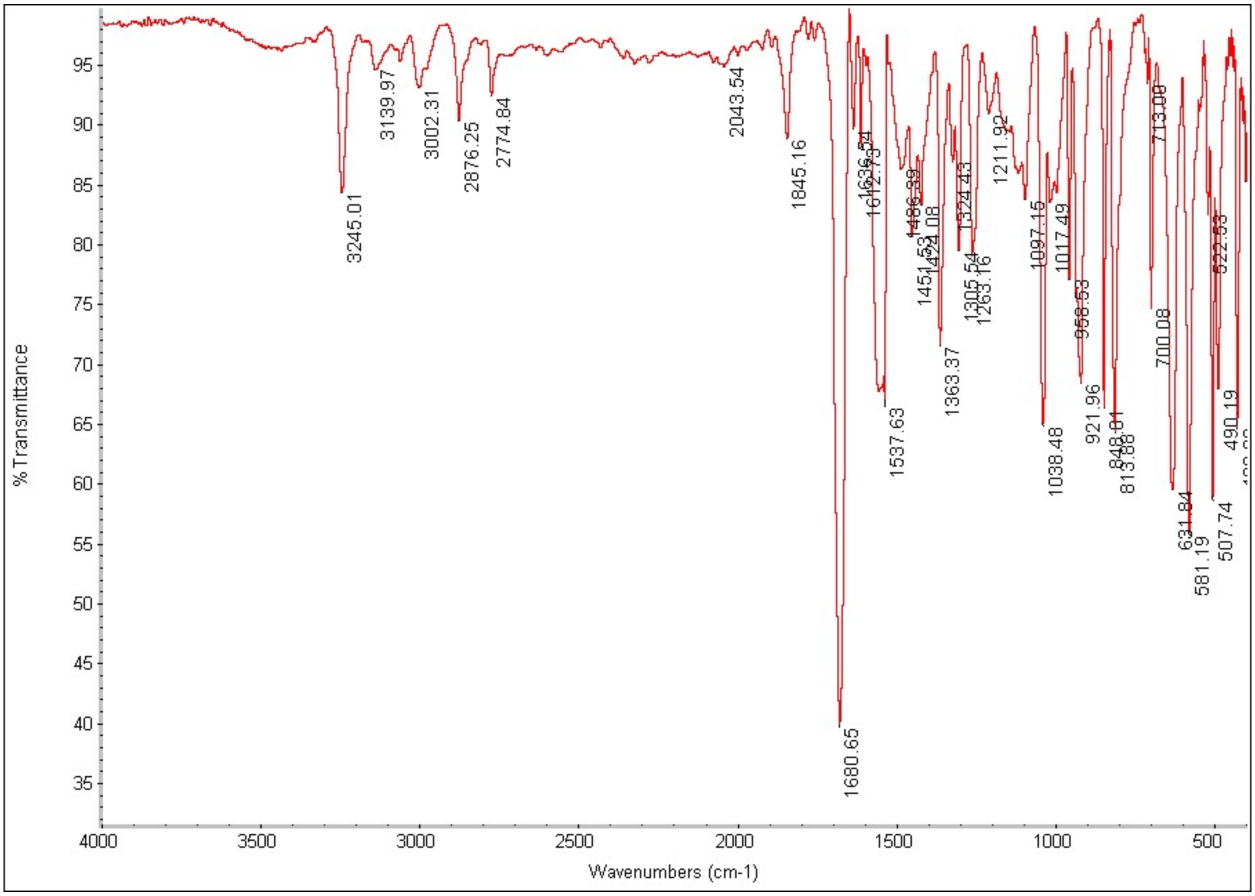

^{1}H NMR ($CDCl_3$) spectrum of (Z)-1-(4-(4-chlorophenyl)-5-((4-chlorophenyl)imino)-4,5-dihydro-1,3,4-thiadiazol-2-yl)ethan-1-one

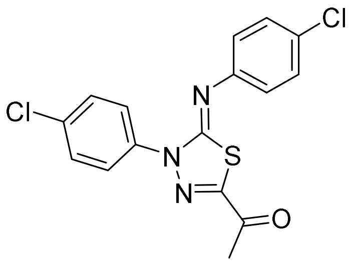

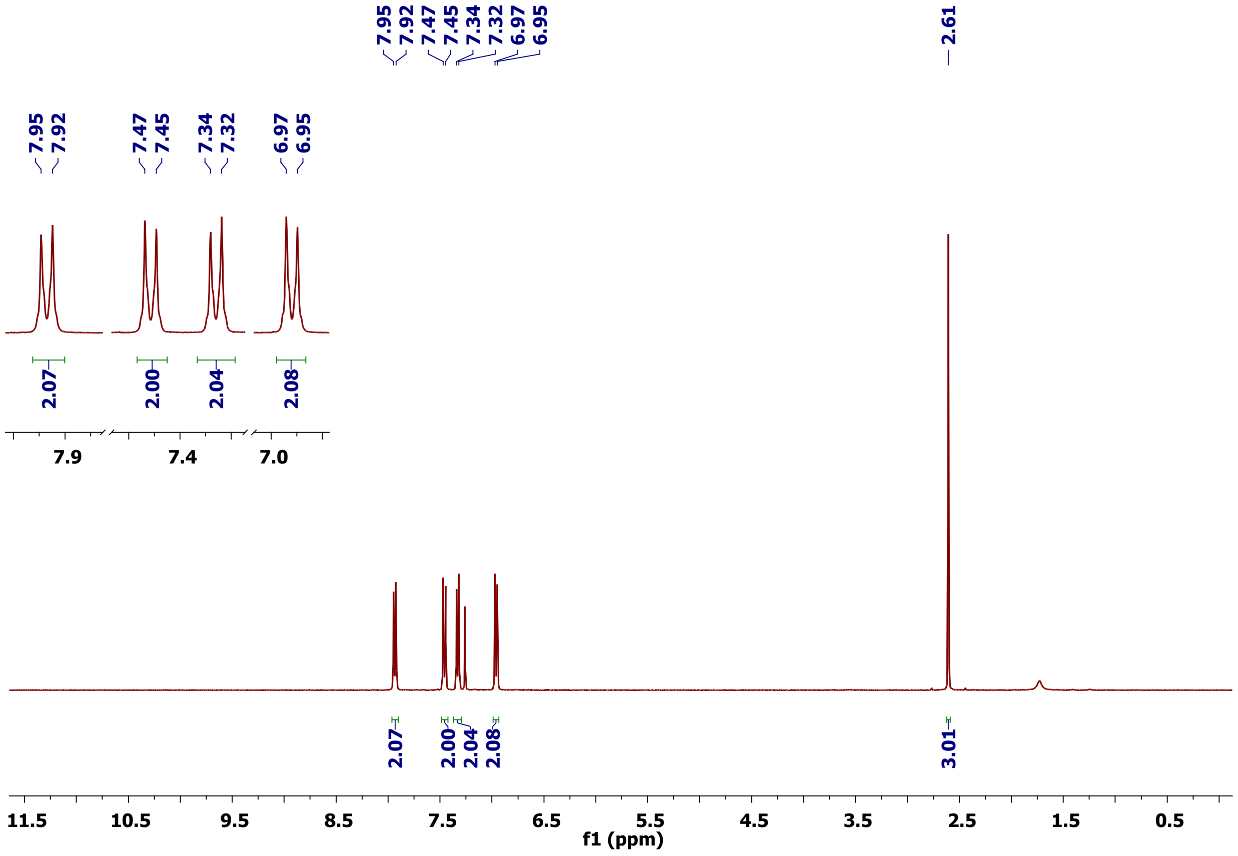

^{13}C NMR ($CDCl_3$) spectrum of (Z)-1-(4-(4-chlorophenyl)-5-((4-chlorophenyl)imino)-4,5-dihydro-1,3,4-thiadiazol-2-yl)ethan-1-one

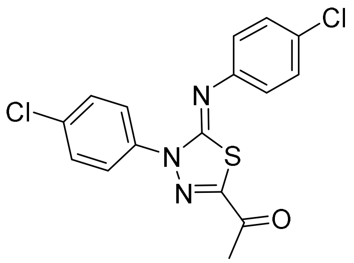

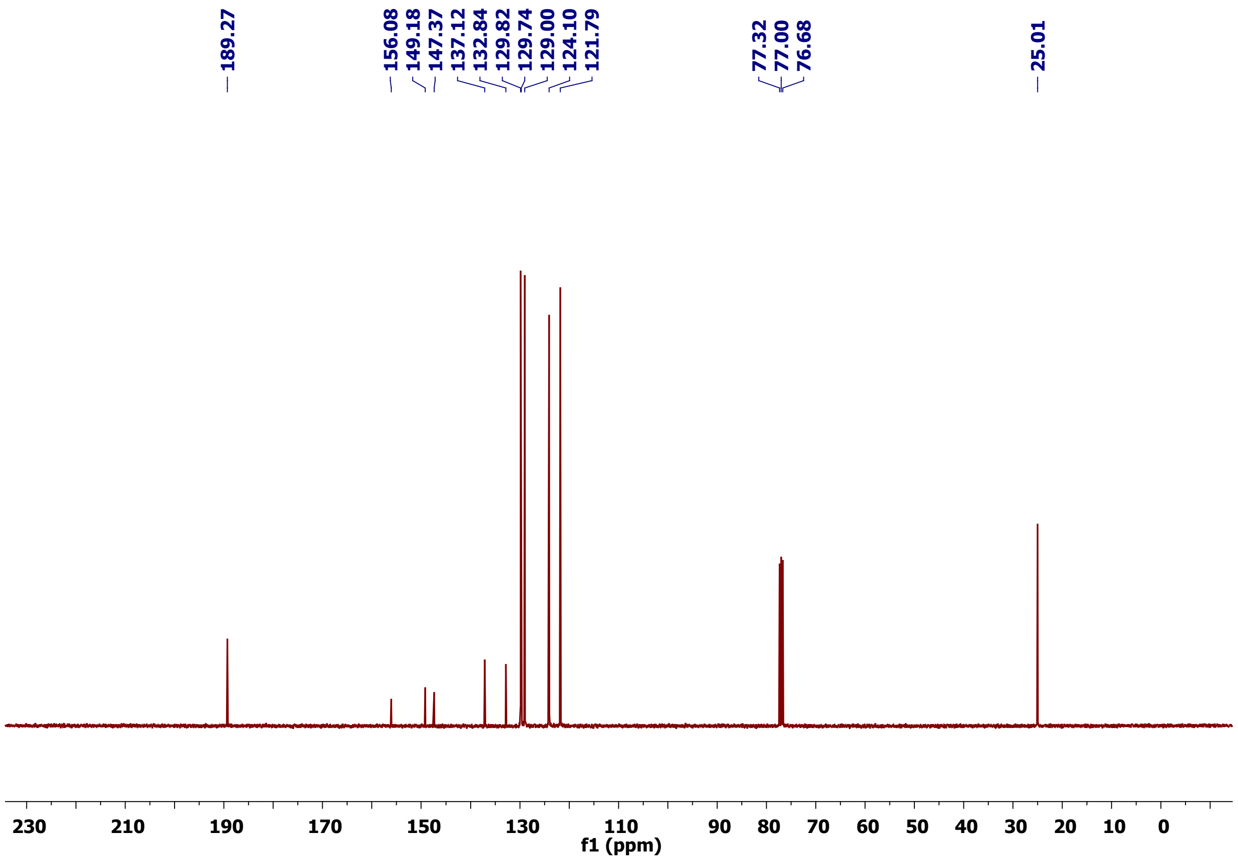

^{13}C CRAPT NMR ($CDCl_3$) spectrum of (Z)-1-(4-(4-chlorophenyl)-5-((4-chlorophenyl)imino)-4,5-dihydro-1,3,4-thiadiazol-2-yl)ethan-1-one

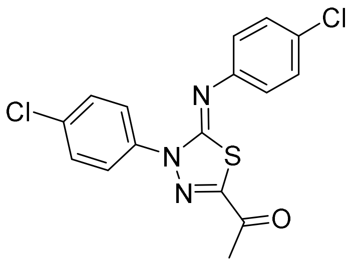

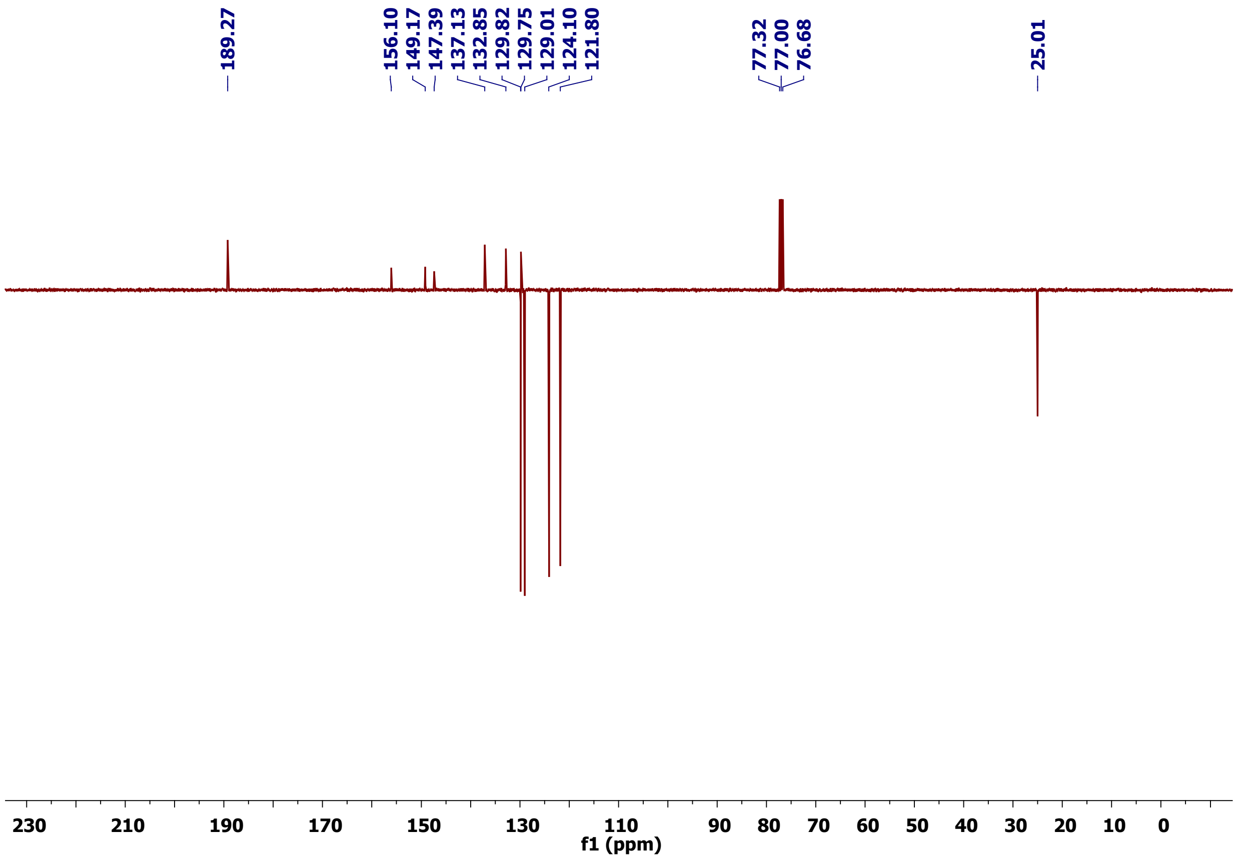

^{1}H-^{1}H gDQCOSY NMR ($CDCl_3$) spectrum of (Z)-1-(4-(4-chlorophenyl)-5-((4-chlorophenyl)imino)-4,5-dihydro-1,3,4-thiadiazol-2-yl)ethan-1-one

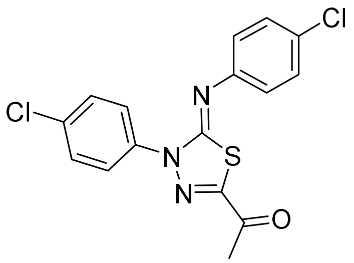

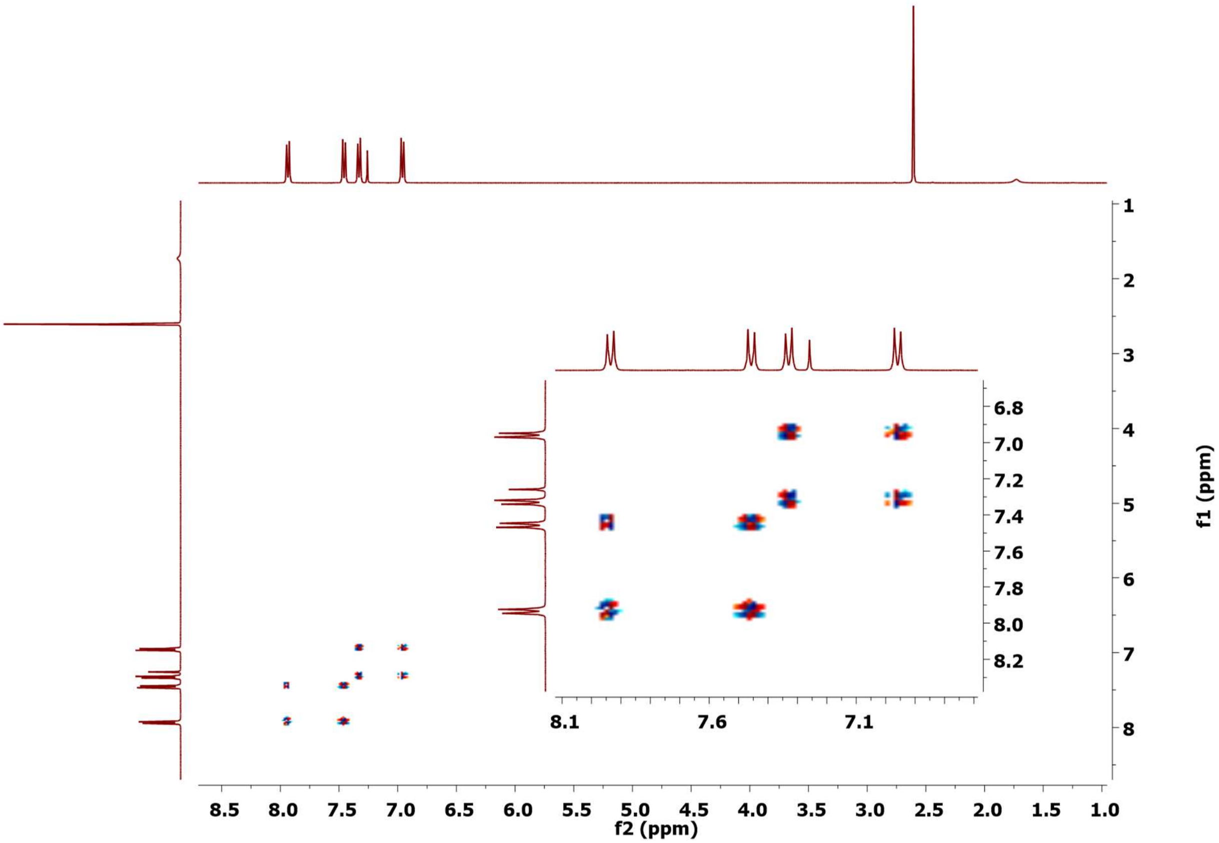

^1H-^{13}C-HSQC NMR ($CDCl_3$) spectrum of (Z)-1-(4-(4-chlorophenyl)-5-((4-chlorophenyl)imino)-4,5-dihydro-1,3,4-thiadiazol-2-yl)ethan-1-one

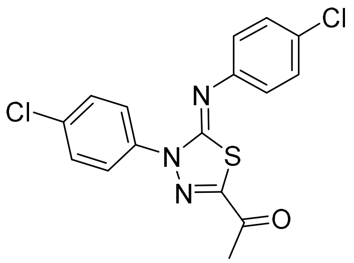

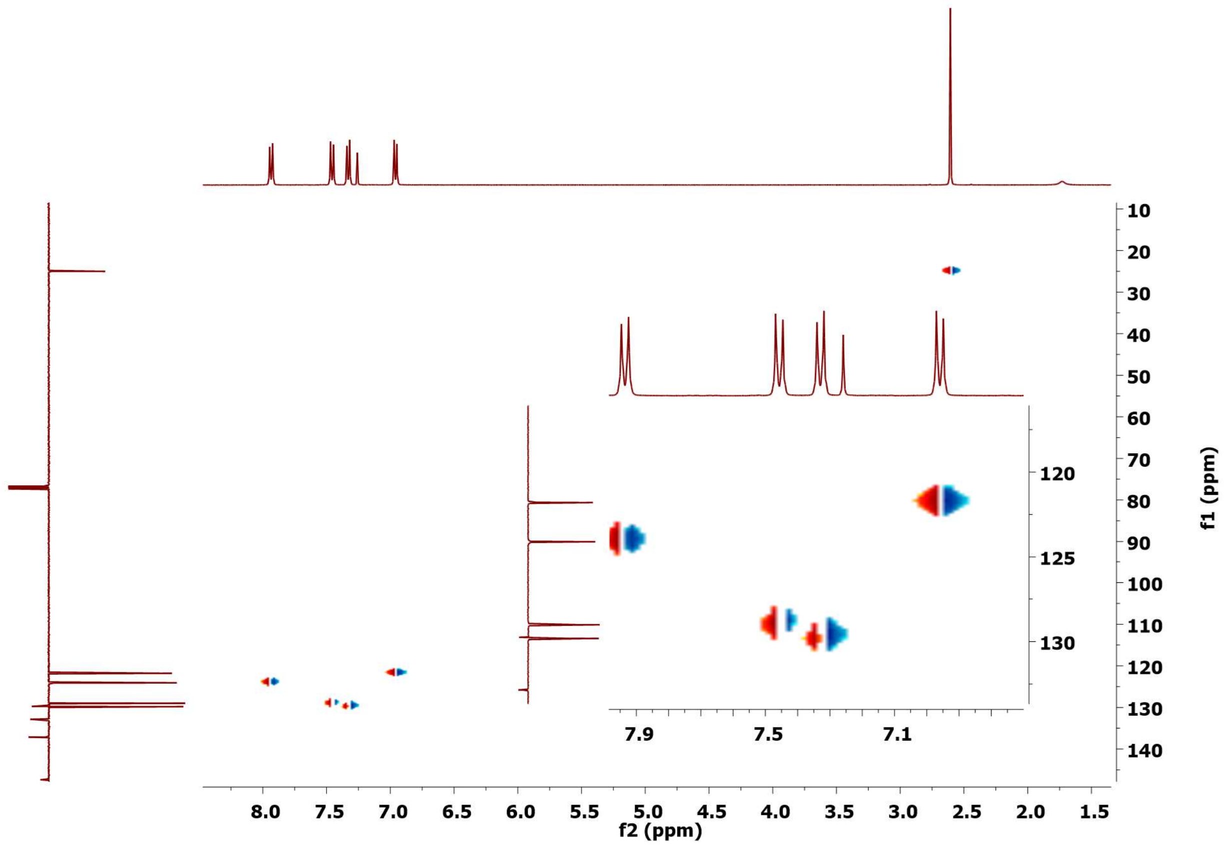

^{1}H-^{13}C-gHMBC NMR ($CDCl_3$) spectrum of (Z)-1-(4-(4-chlorophenyl)-5-((4-chlorophenyl)imino)-4,5-dihydro-1,3,4-thiadiazol-2-yl)ethan-1-one

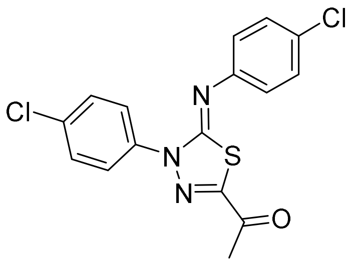

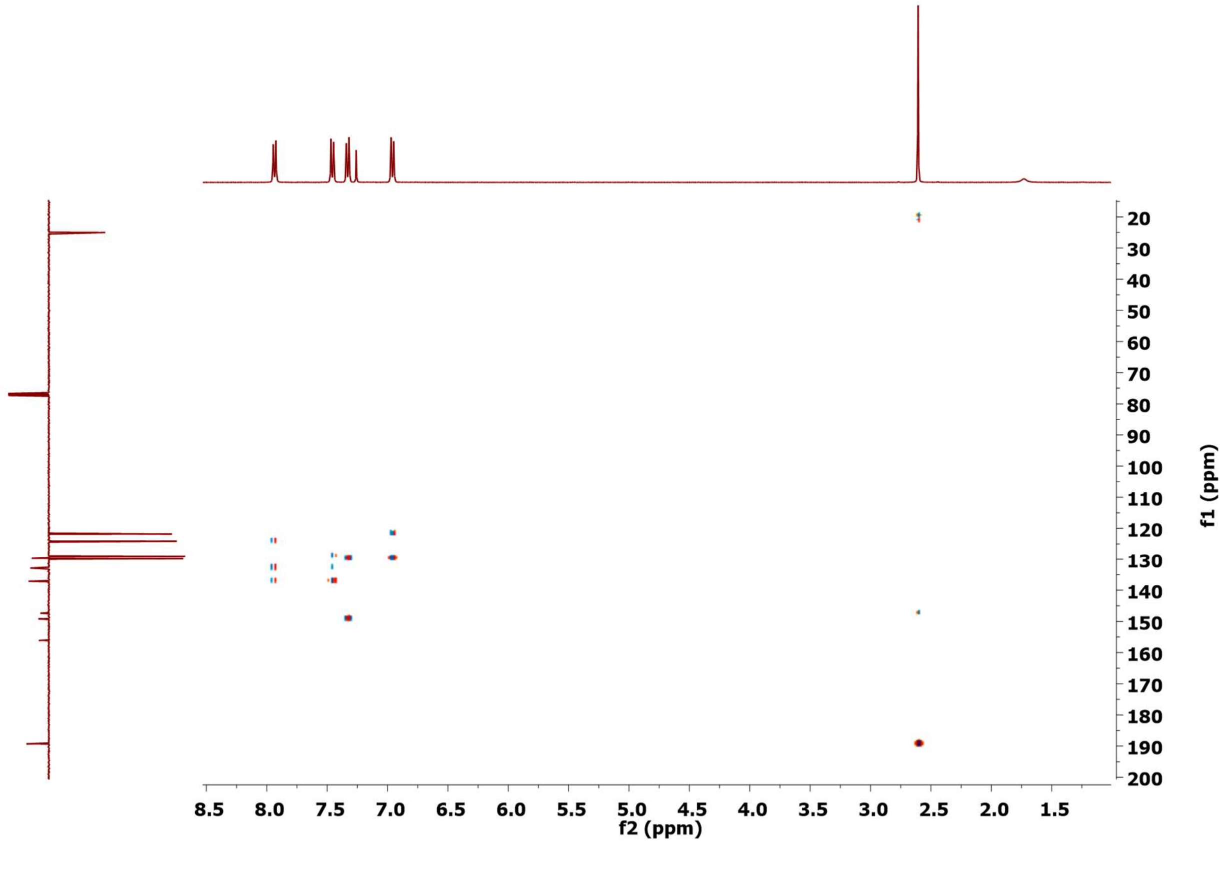

^{1}H NMR ($CDCl_3$) spectrum of (Z)-1-(4-(4-bromophenyl)-5-((4-bromophenyl)imino)-4,5-dihydro-1,3,4-thiadiazol-2-yl)ethan-1-one

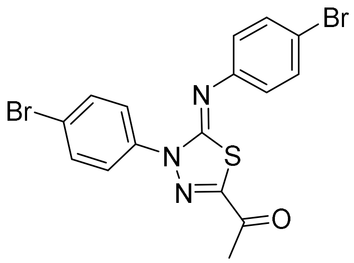

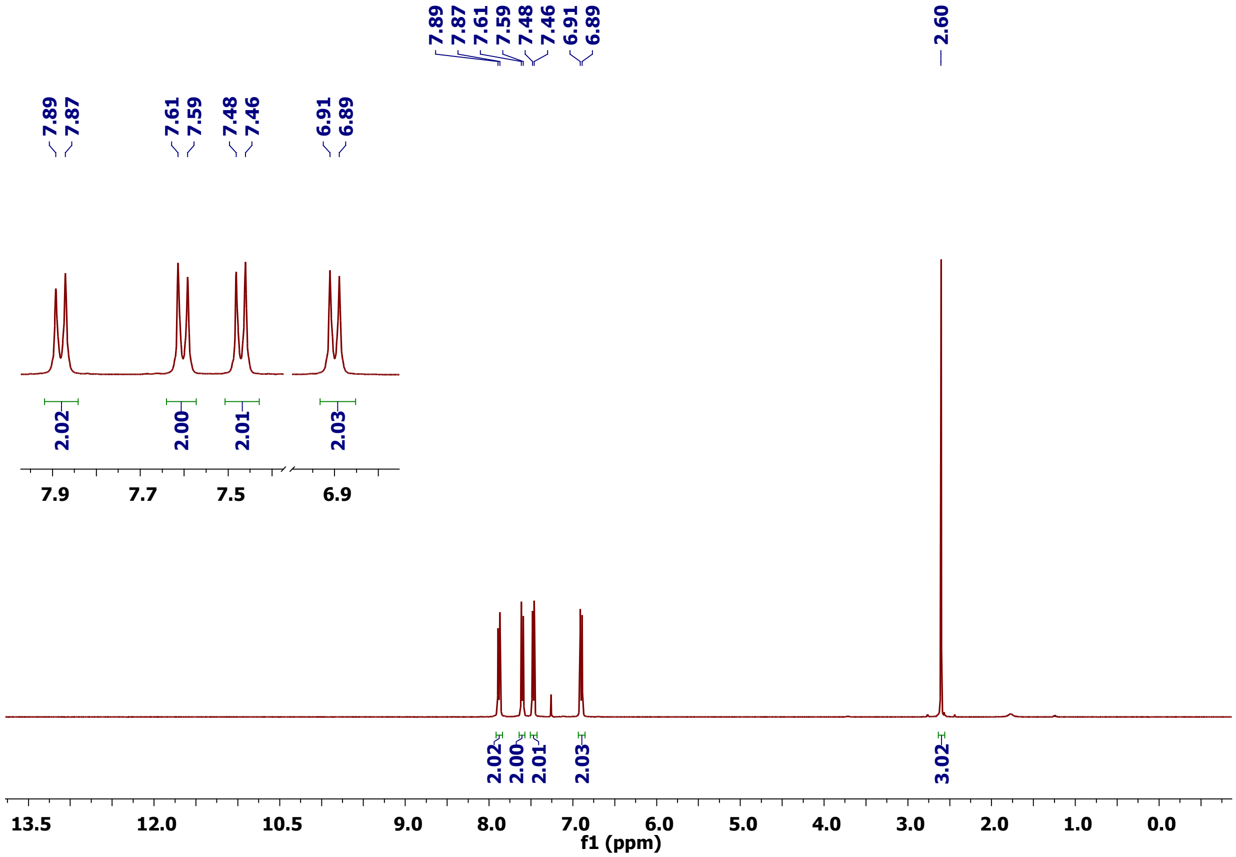

^{13}C NMR ($CDCl_3$) spectrum of (Z)-1-(4-(4-bromophenyl)-5-((4-bromophenyl)imino)-4,5-dihydro-1,3,4-thiadiazol-2-yl)ethan-1-one

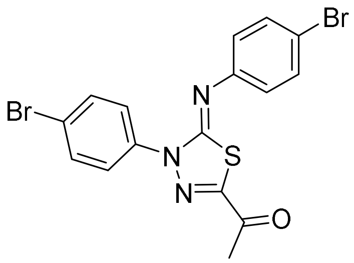

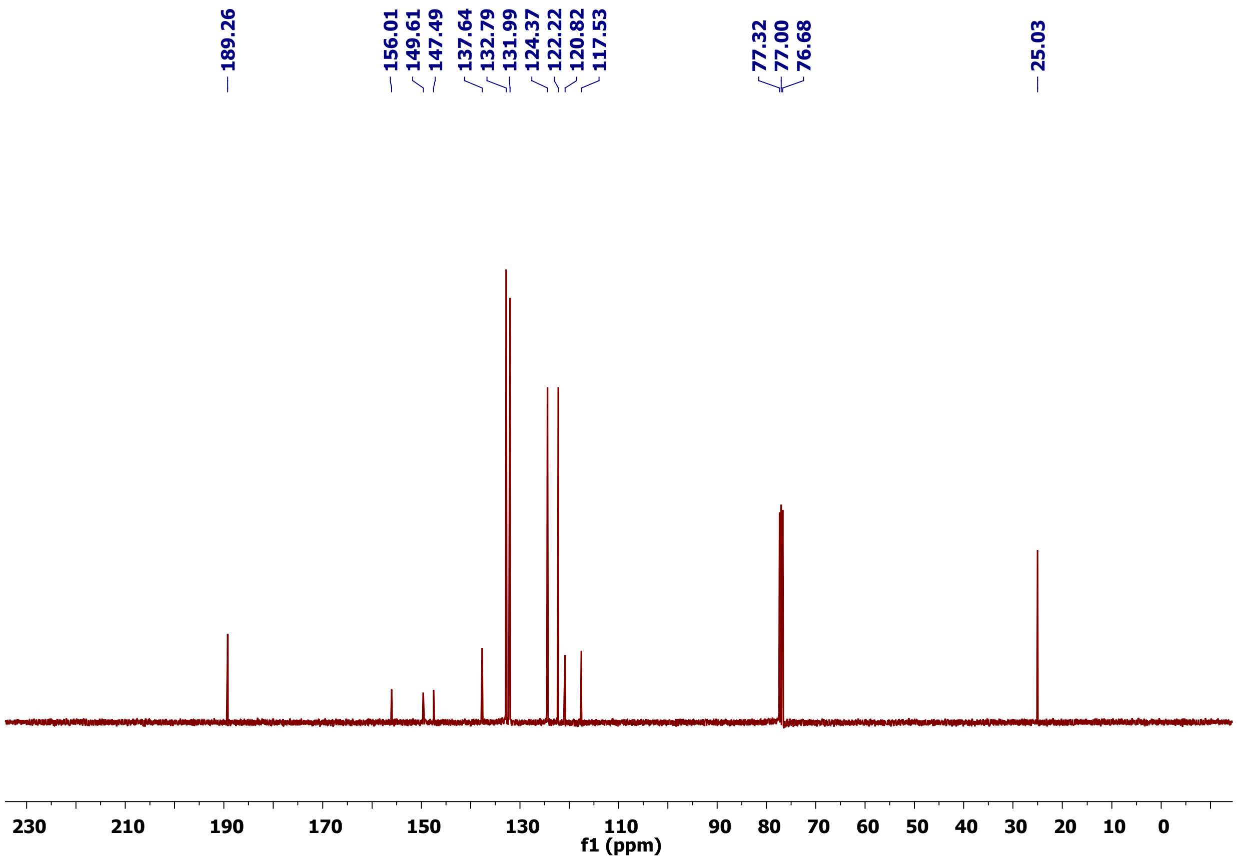

^{13}C CRAPT NMR ($CDCl_3$) spectrum of (Z)-1-(4-(4-bromophenyl)-5-((4-bromophenyl)imino)-4,5-dihydro-1,3,4-thiadiazol-2-yl)ethan-1-one

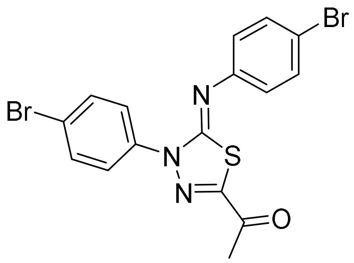

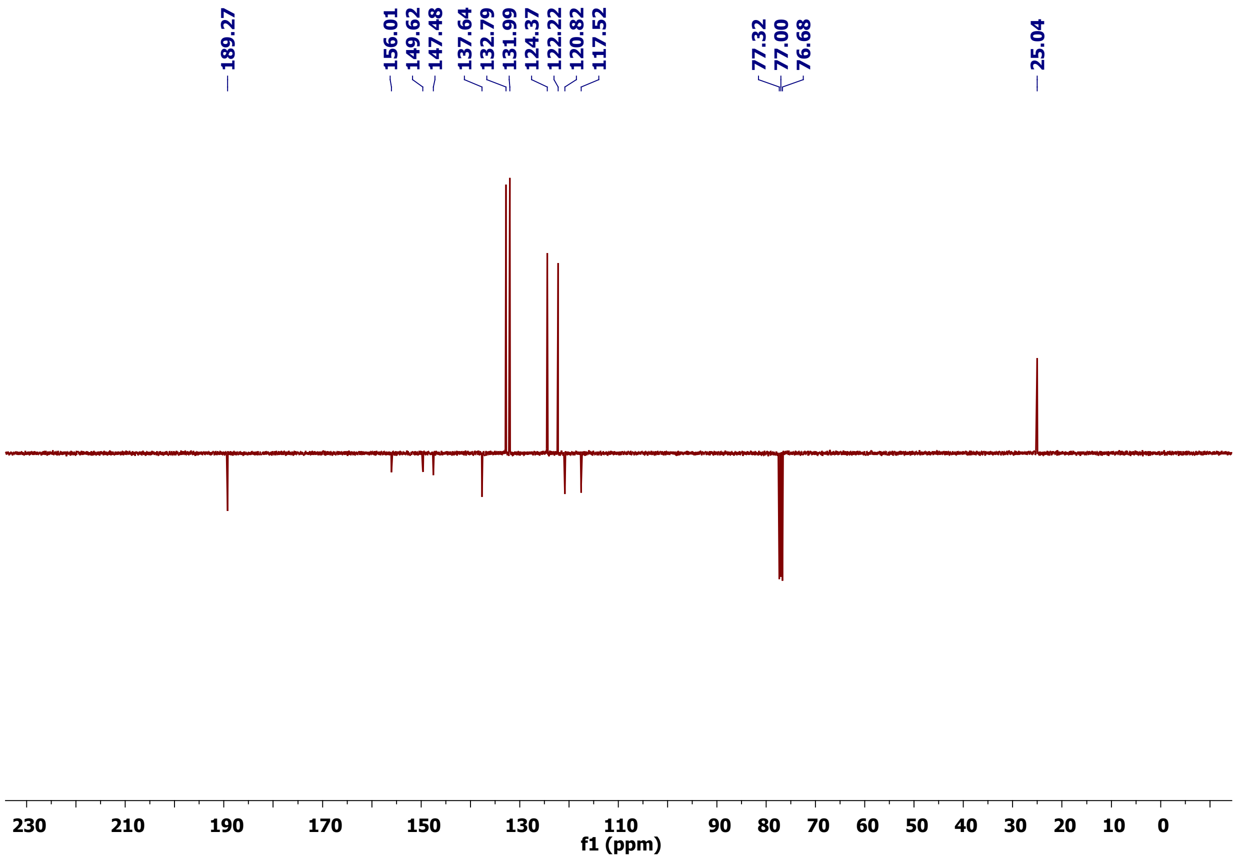

^1H-^1H gDQCOSY NMR ($CDCl_3$) spectrum of (Z)-1-(4-(4-bromophenyl)-5-((4-bromophenyl)imino)-4,5-dihydro-1,3,4-thiadiazol-2-yl)ethan-1-one

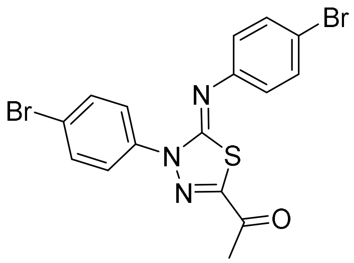

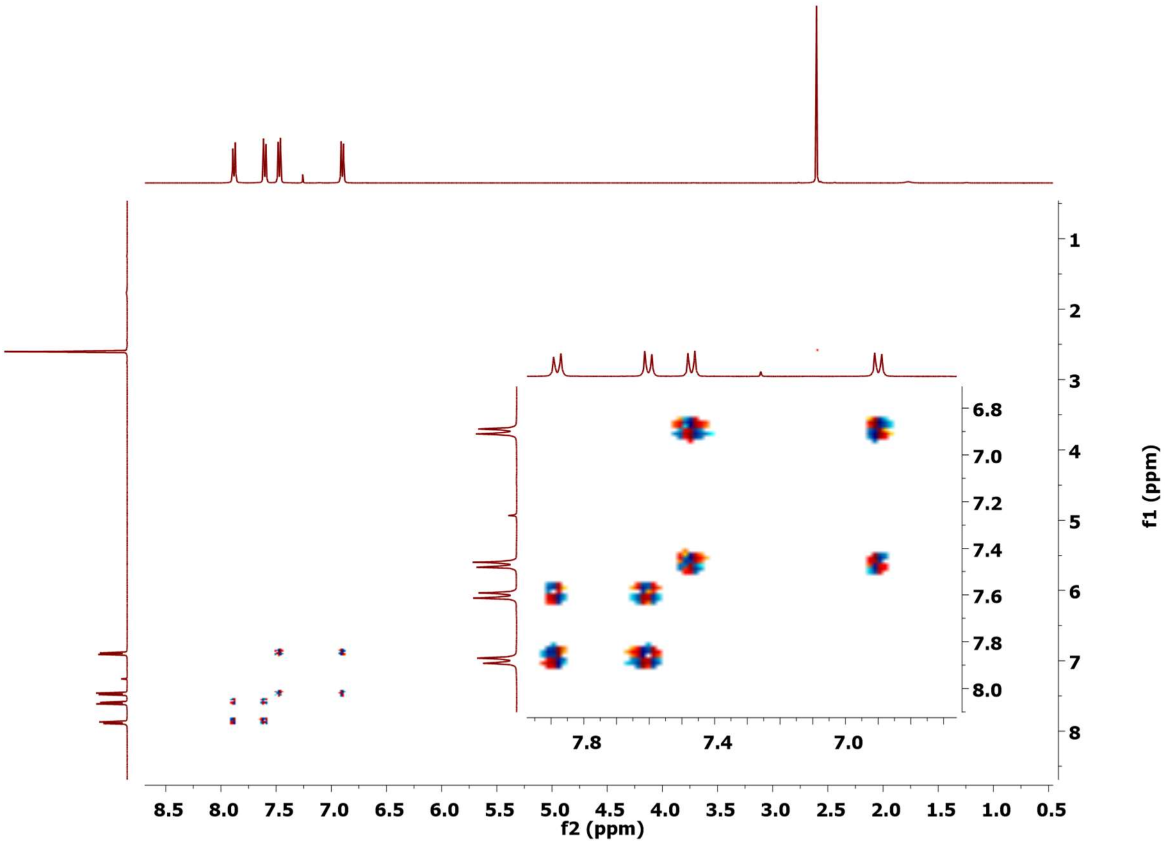

^{1}H-^{13}C-HSQC NMR ($CDCl_3$) spectrum of (Z)-1-(4-(4-bromophenyl)-5-((4-bromophenyl)imino)-4,5-dihydro-1,3,4-thiadiazol-2-yl)ethan-1-one

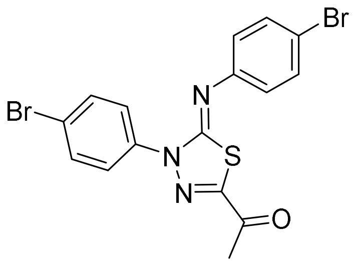

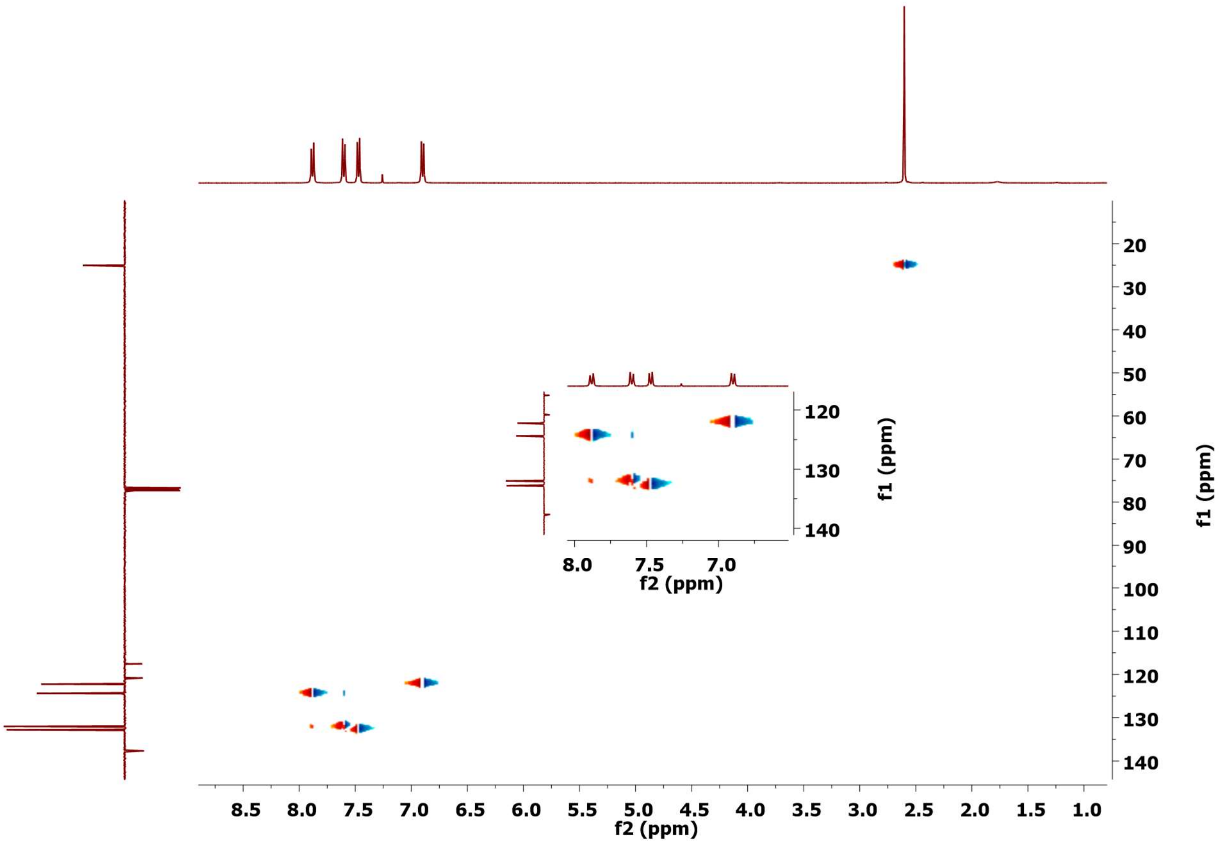

^{1}H-^{13}C-gHMBC NMR ($CDCl_3$) spectrum of (Z)-1-(4-(4-bromophenyl)-5-((4-bromophenyl)imino)-4,5-dihydro-1,3,4-thiadiazol-2-yl)ethan-1-one

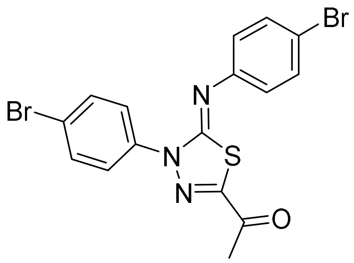

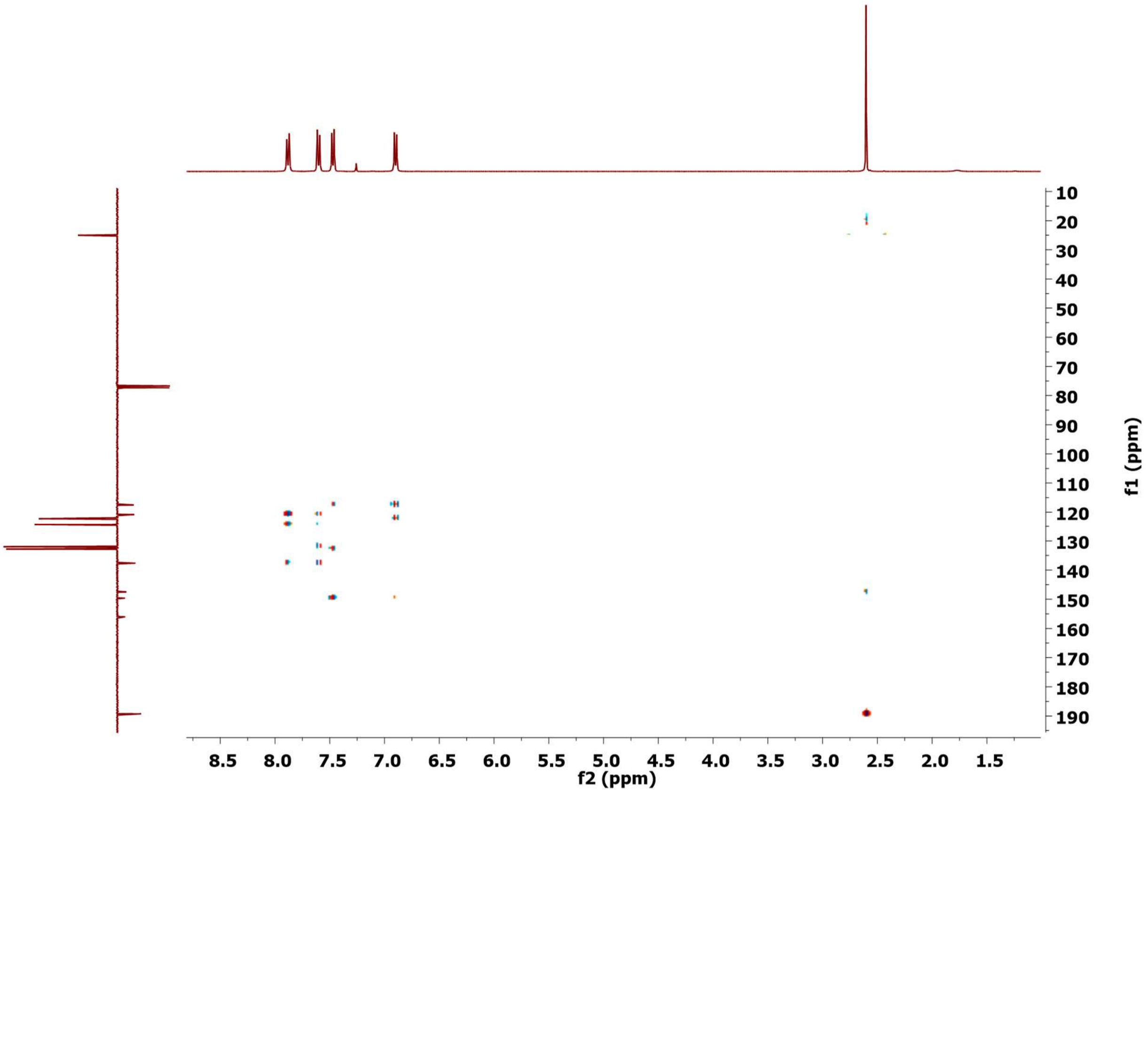

^{1}H NMR ($CDCl_3$) spectrum of (Z)-1-(4-(4-iodophenyl)-5-((4-iodophenyl)imino)-4,5-dihydro-1,3,4-thiadiazol-2-yl)ethan-1-one

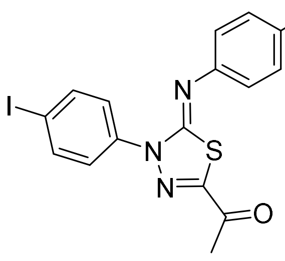

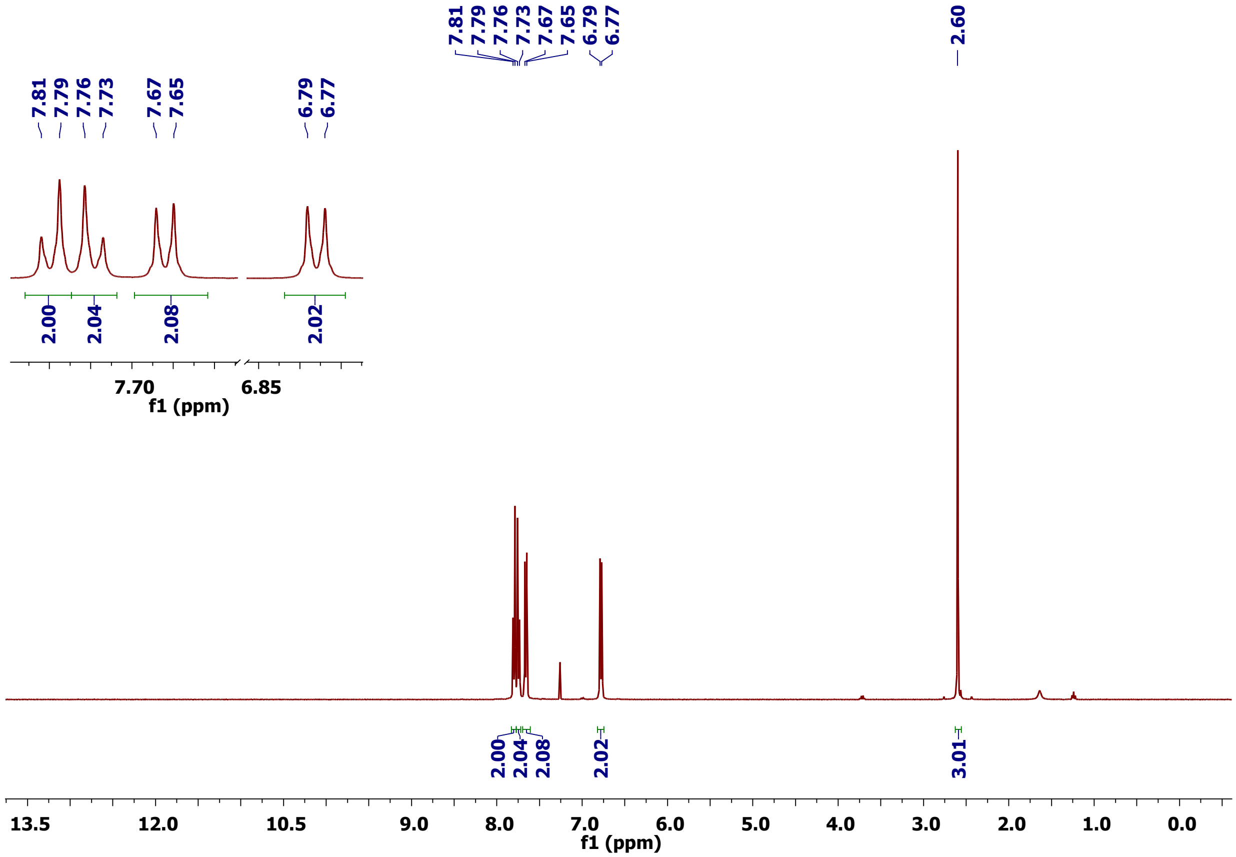

^{13}C NMR ($CDCl_3$) spectrum of (Z)-1-(4-(4-iodophenyl)-5-((4-iodophenyl)imino)-4,5-dihydro-1,3,4-thiadiazol-2-yl)ethan-1-one

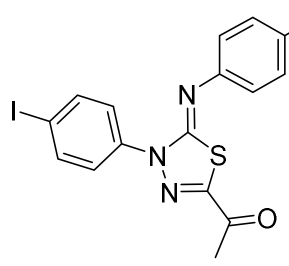

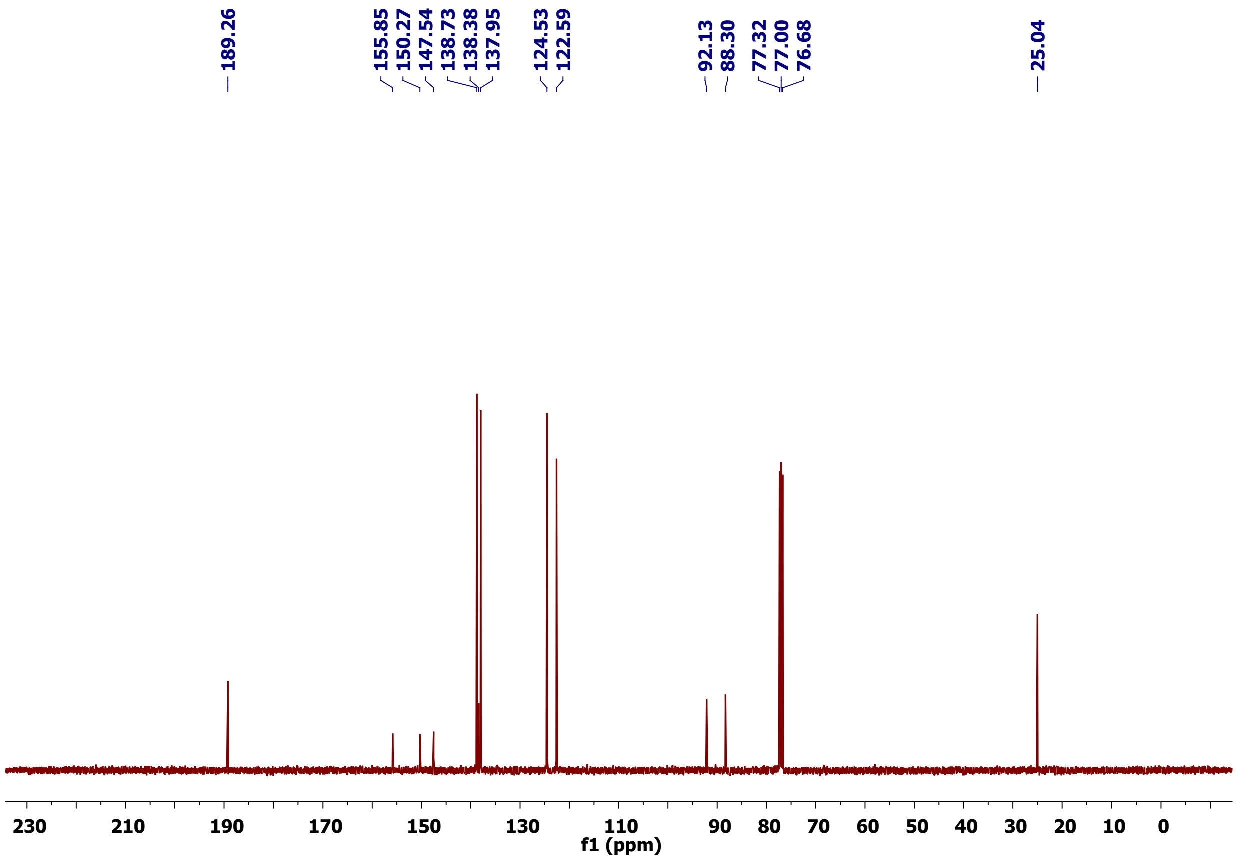

^{13}C CRAPT NMR ($CDCl_3$) spectrum of (Z)-1-(4-(4-iodophenyl)-5-((4-iodophenyl)imino)-4,5-dihydro-1,3,4-thiadiazol-2-yl)ethan-1-one

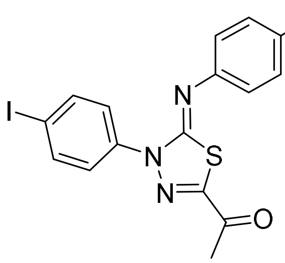

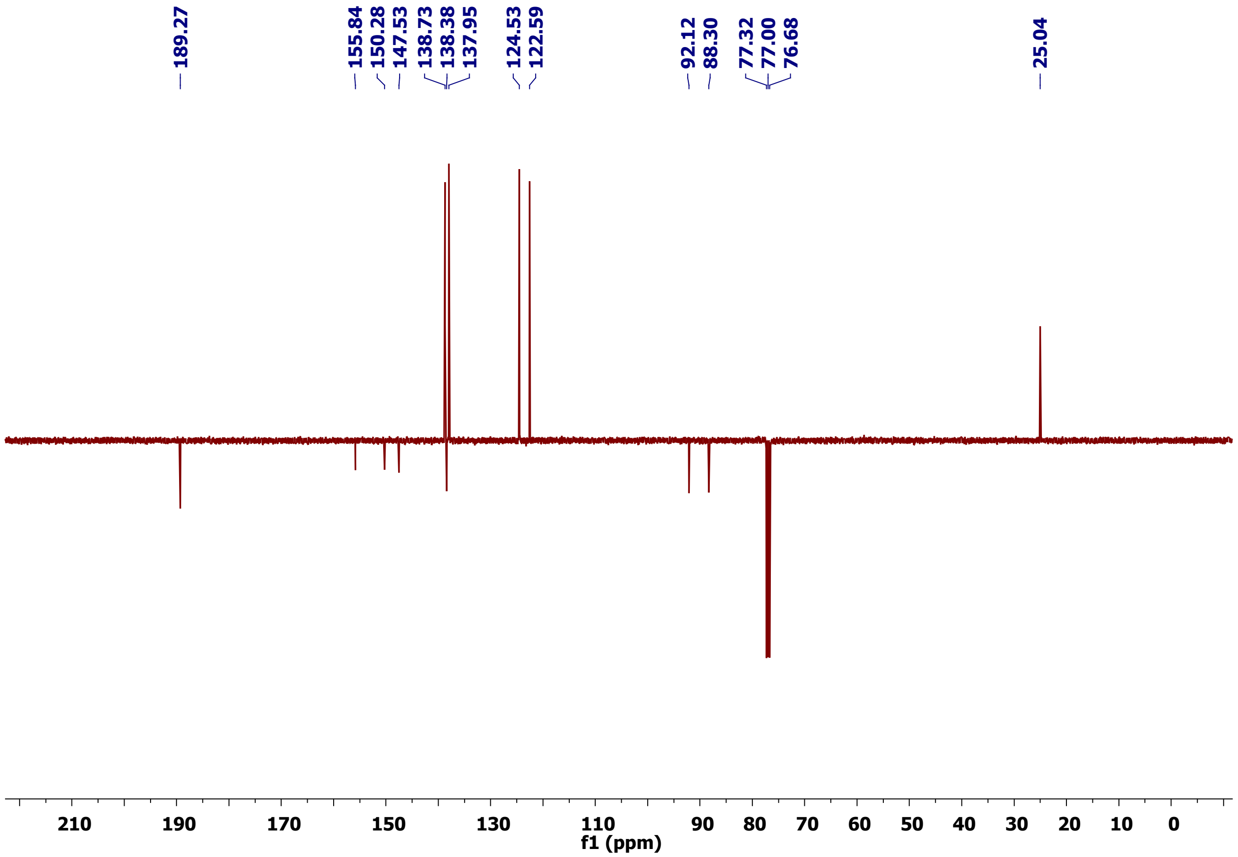

^{1}H-^{1}H gDQCOSY NMR ($CDCl_3$) spectrum of (Z)-1-(4-(4-iodophenyl)-5-((4-iodophenyl)imino)-4,5-dihydro-1,3,4-thiadiazol-2-yl)ethan-1-one

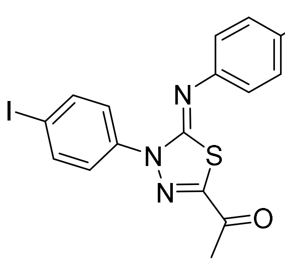

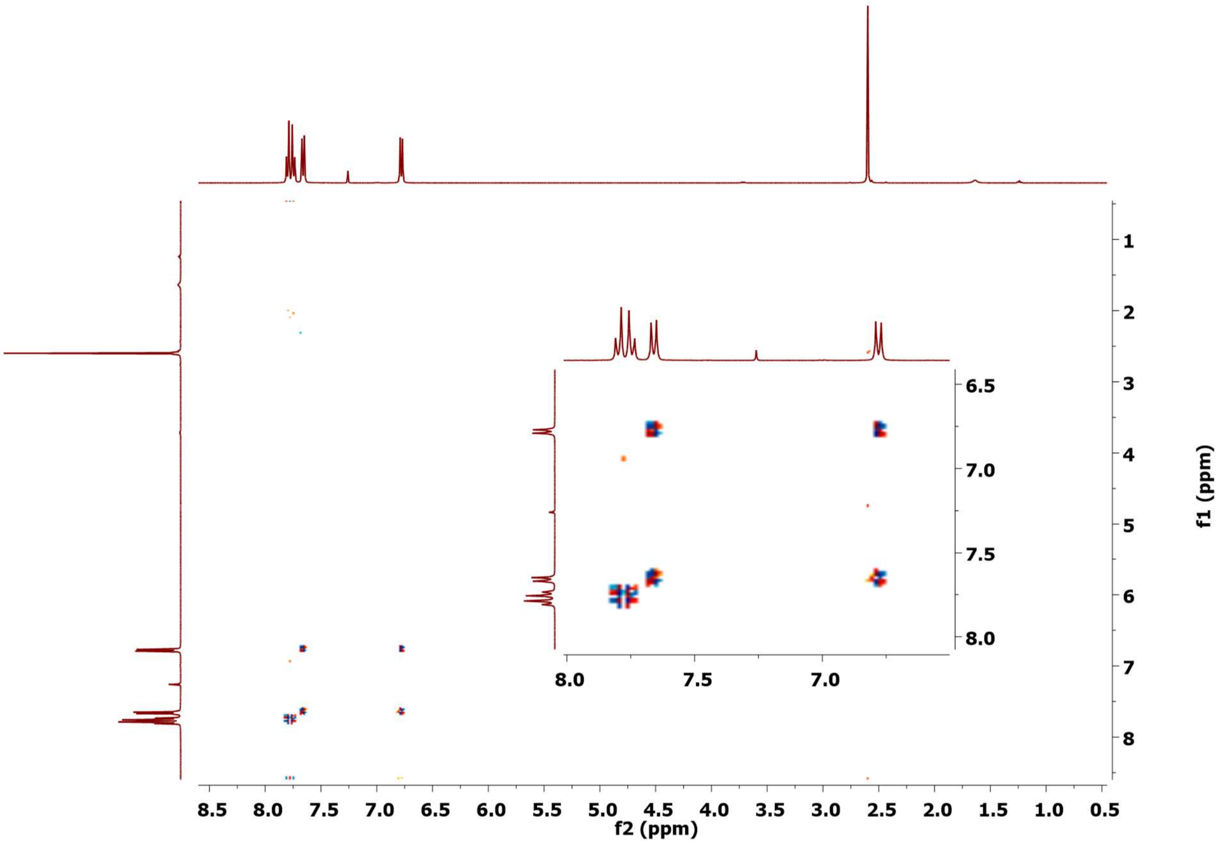

^{1}H-^{13}C-HSQC NMR ($CDCl_3$) spectrum of (Z)-1-(4-(4-iodophenyl)-5-((4-iodophenyl)imino)-4,5-dihydro-1,3,4-thiadiazol-2-yl)ethan-1-one

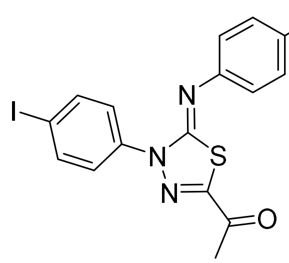

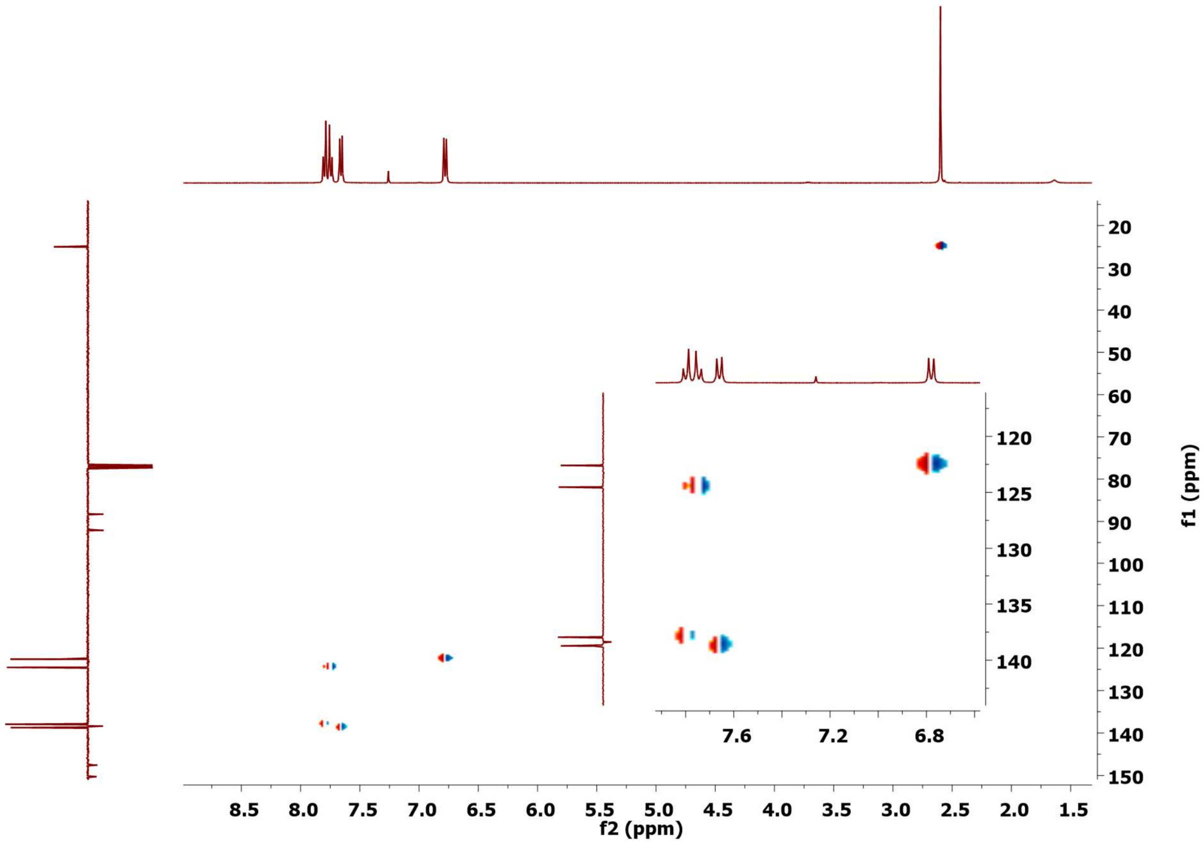

^{1}H-^{13}C-gHMBC NMR ($CDCl_3$) spectrum of (Z)-1-(4-(4-iodophenyl)-5-((4-iodophenyl)imino)-4,5-dihydro-1,3,4-thiadiazol-2-yl)ethan-1-one

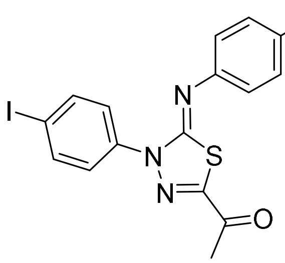

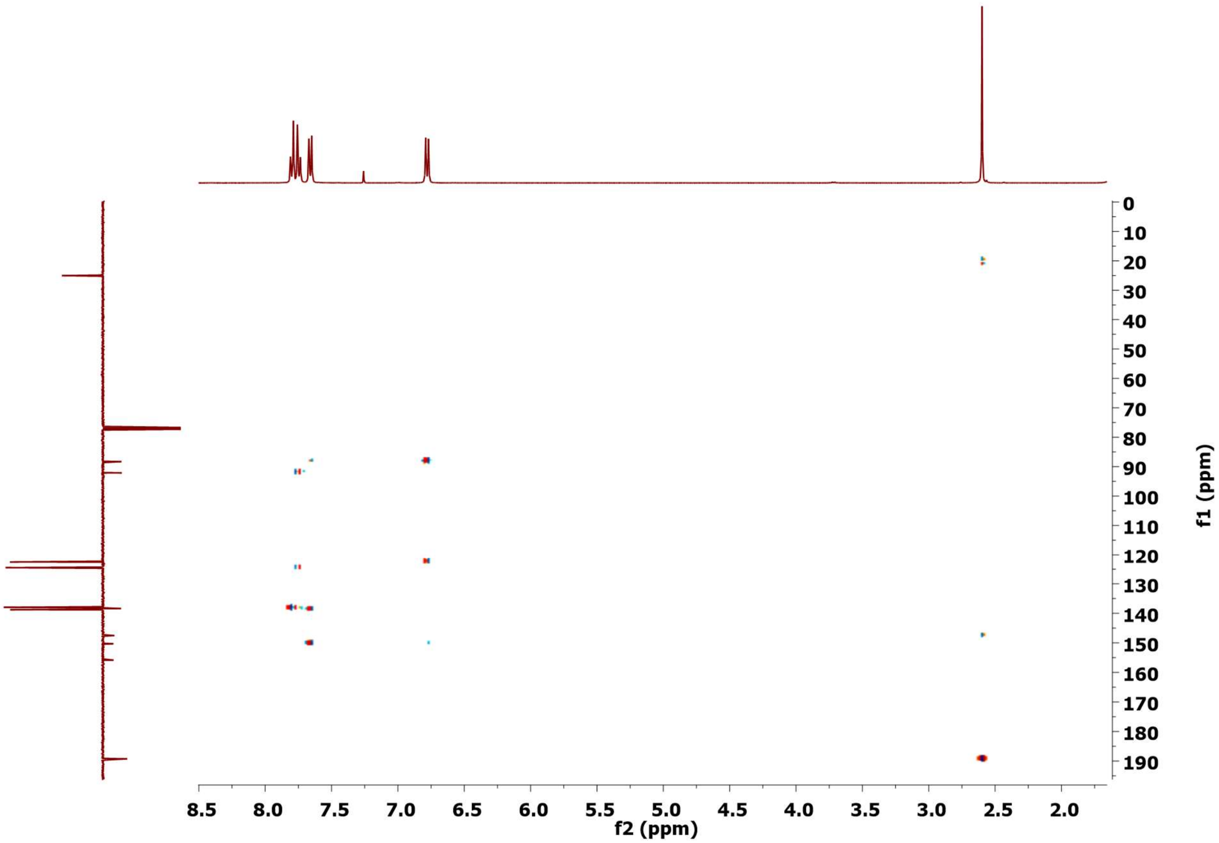

^{1}H NMR ($CDCl_3$) spectrum of (Z)-1-(4-(4-iodophenyl)-5-((4-methoxyphenyl)imino)-4,5-dihydro-1,3,4-thiadiazol-2-yl)ethan-1-one

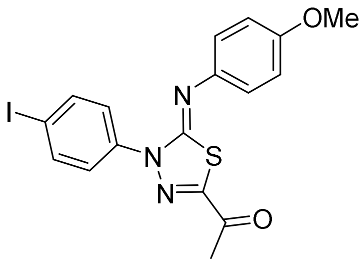

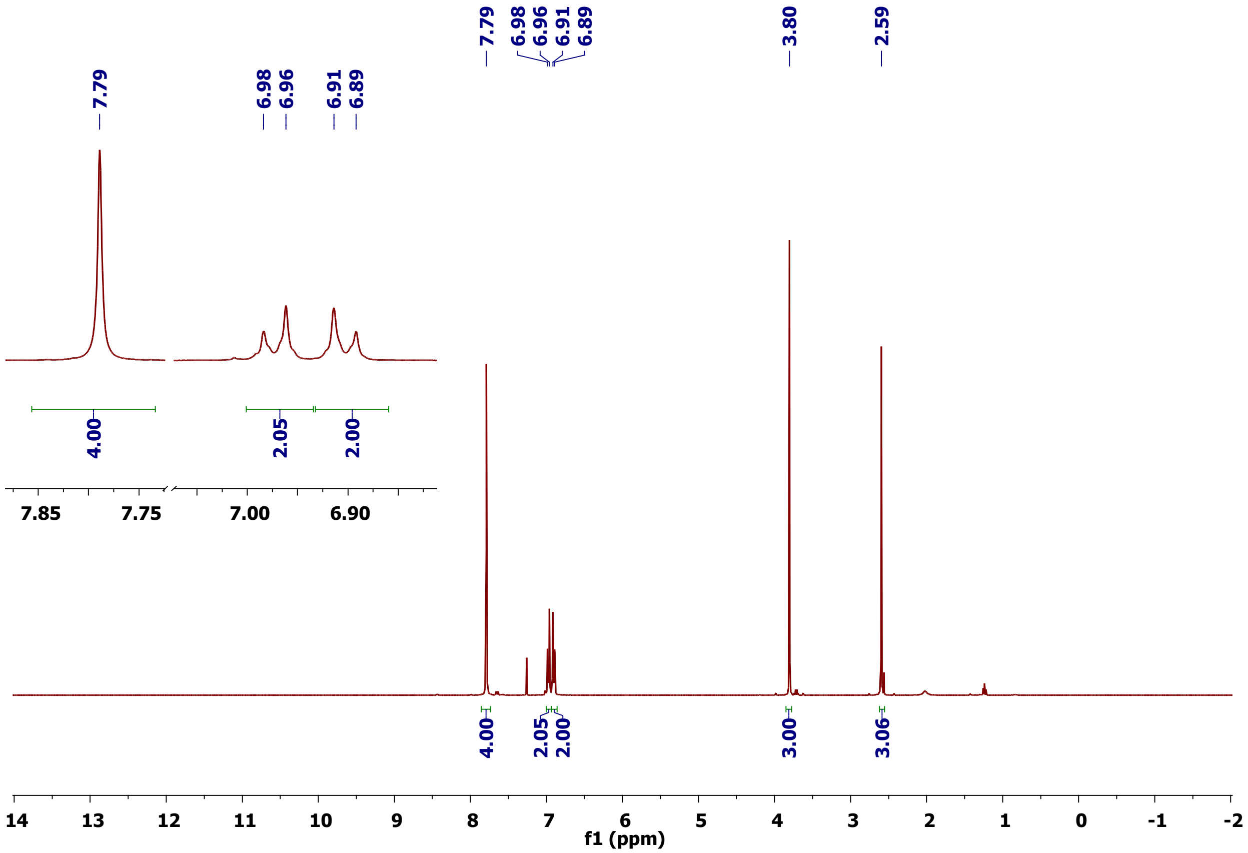

^{13}C NMR ($CDCl_3$) spectrum of (Z)-1-(4-(4-iodophenyl)-5-((4-methoxyphenyl)imino)-4,5-dihydro-1,3,4-thiadiazol-2-yl)ethan-1-one

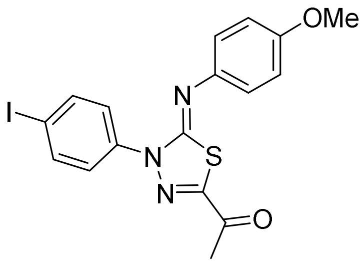

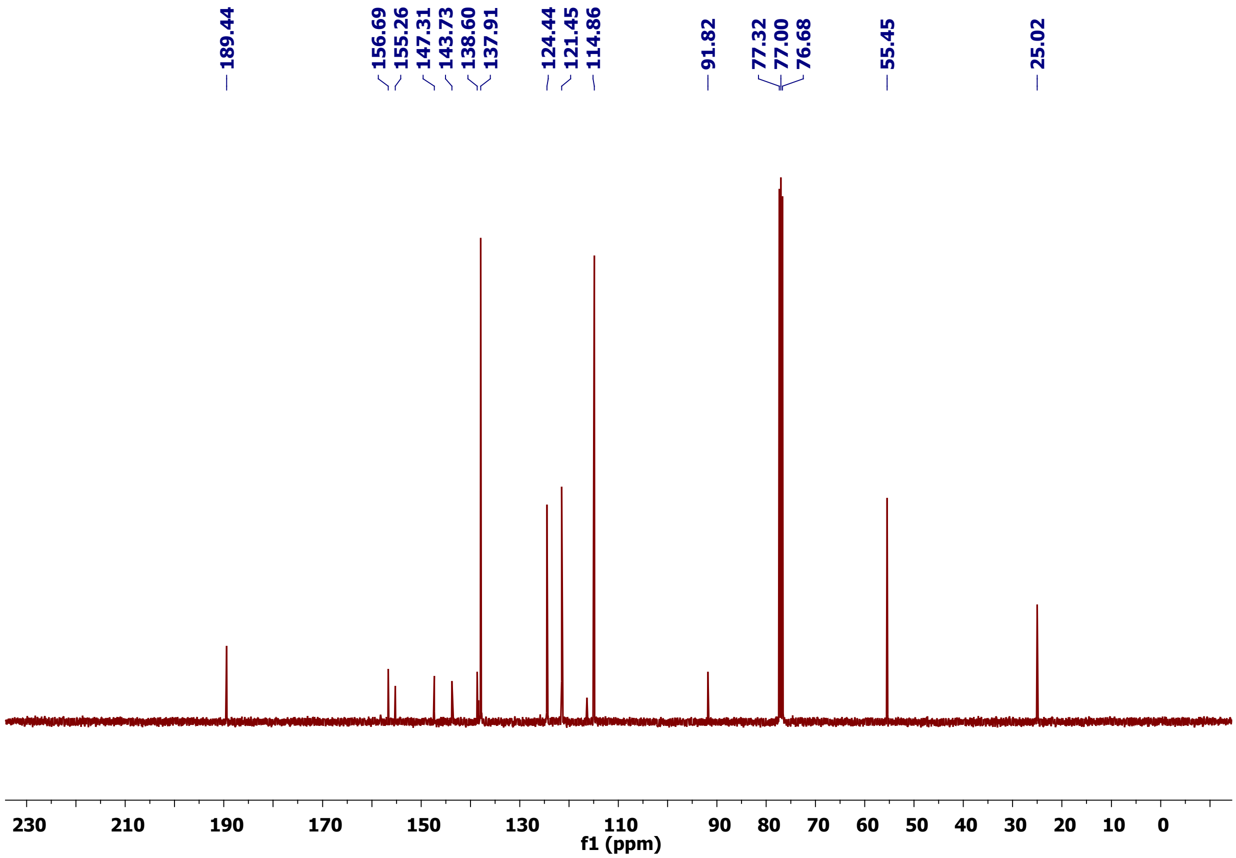

^{13}C CRAPT NMR ($CDCl_3$) spectrum of (Z)-1-(4-(4-iodophenyl)-5-((4-methoxyphenyl)imino)-4,5-dihydro-1,3,4-thiadiazol-2-yl)ethan-1-one

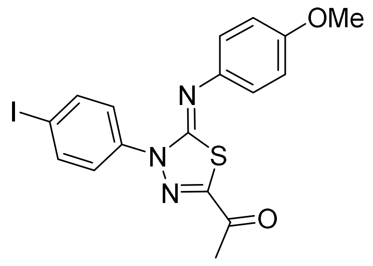

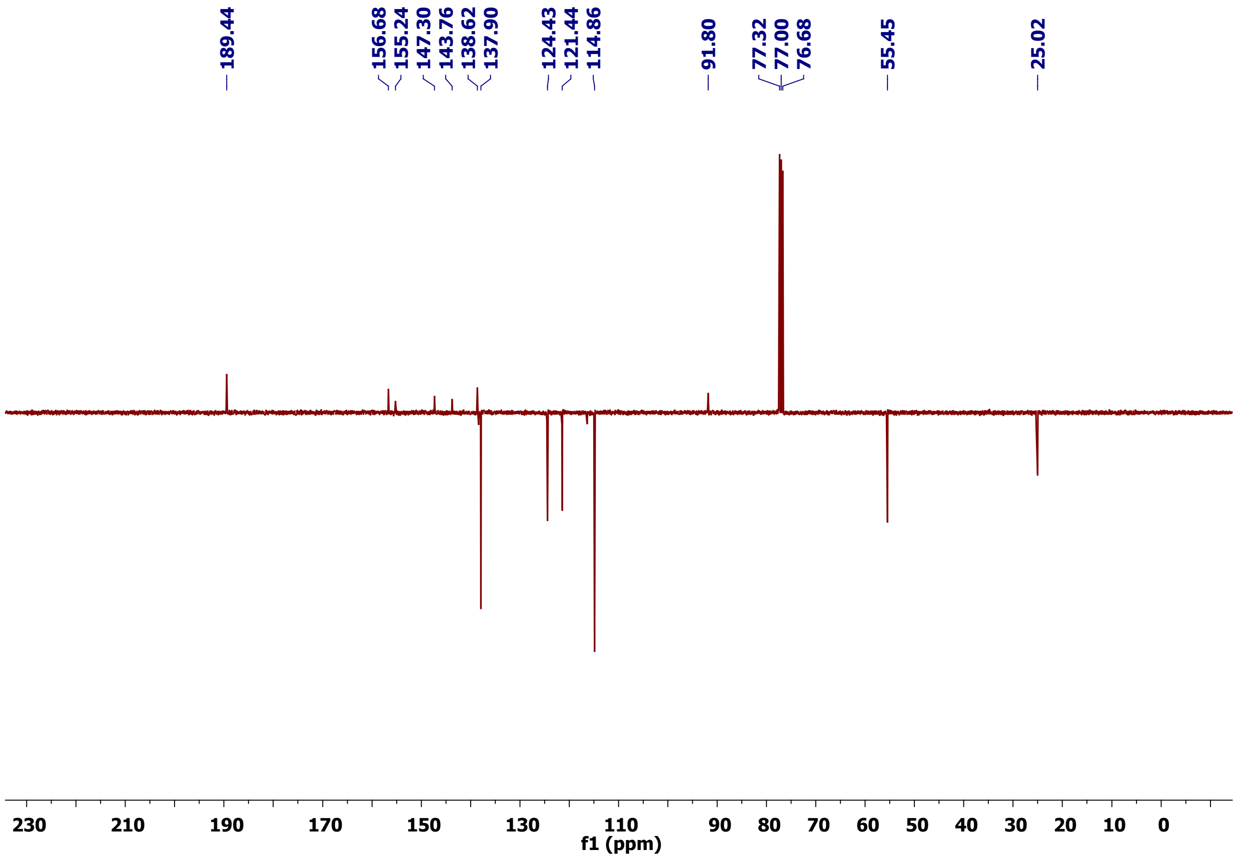

^{1}H-^{1}H gDQCOSY NMR ($CDCl_3$) spectrum of (Z)-1-(4-(4-iodophenyl)-5-((4-methoxyphenyl)imino)-4,5-dihydro-1,3,4-thiadiazol-2-yl)ethan-1-one

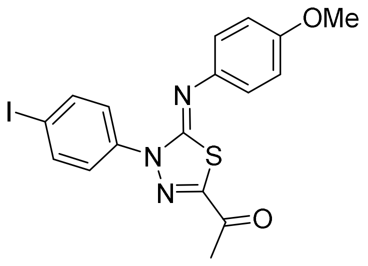

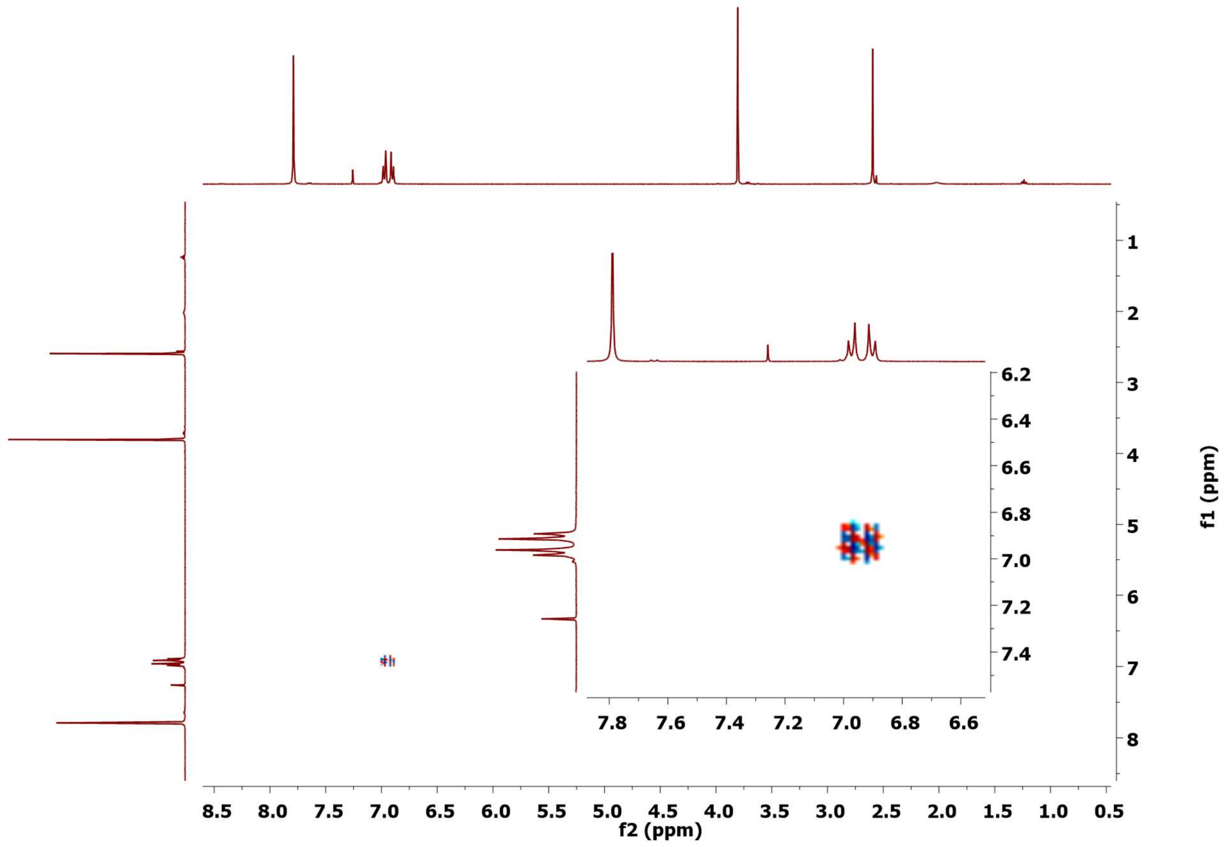

^{1}H-^{13}C-HSQC NMR ($CDCl_3$) spectrum of (Z)-1-(4-(4-iodophenyl)-5-((4-methoxyphenyl)imino)-4,5-dihydro-1,3,4-thiadiazol-2-yl)ethan-1-one

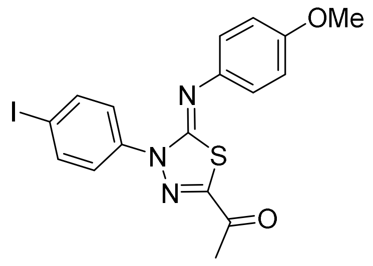

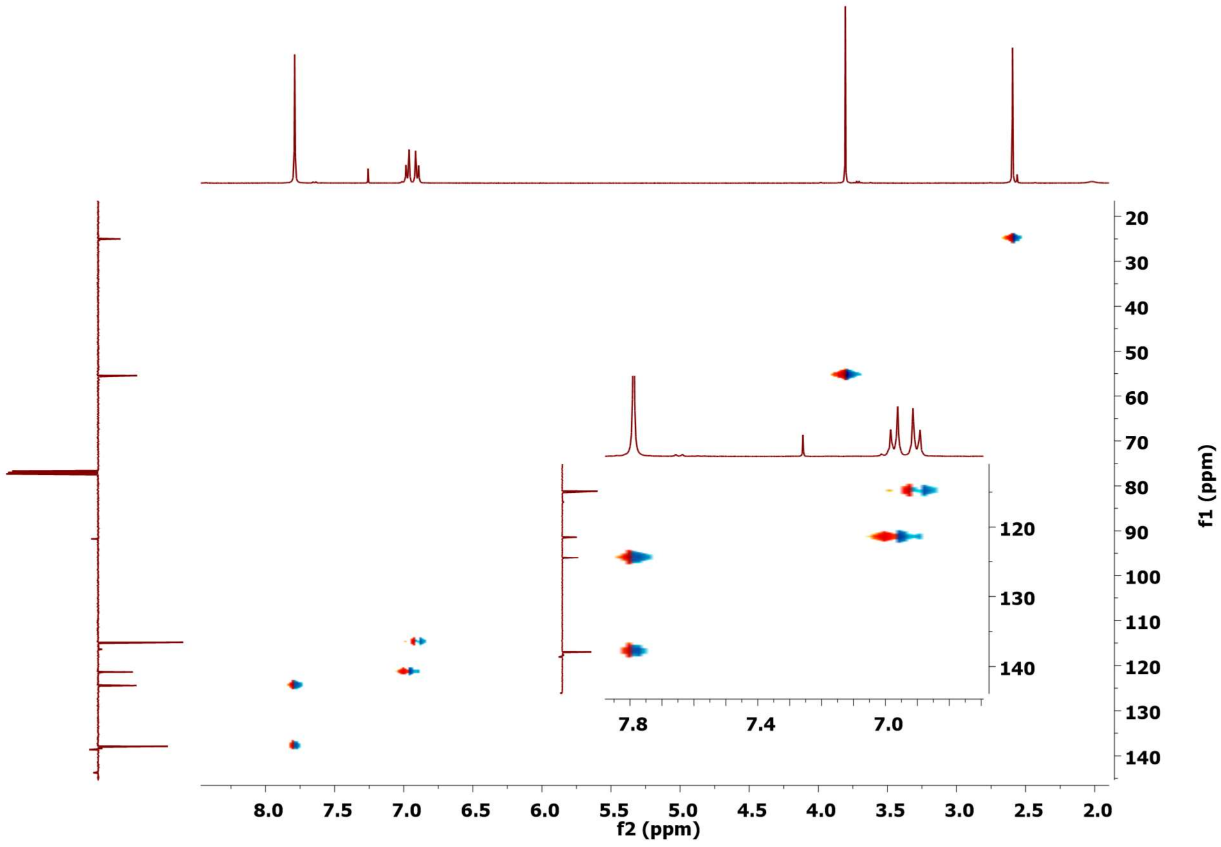

^{1}H-^{13}C-gHMBC NMR ($CDCl_3$) spectrum of (Z)-1-(4-(4-iodophenyl)-5-((4-methoxyphenyl)imino)-4,5-dihydro-1,3,4-thiadiazol-2-yl)ethan-1-one

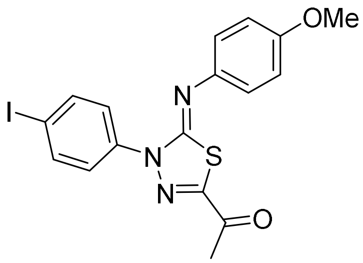

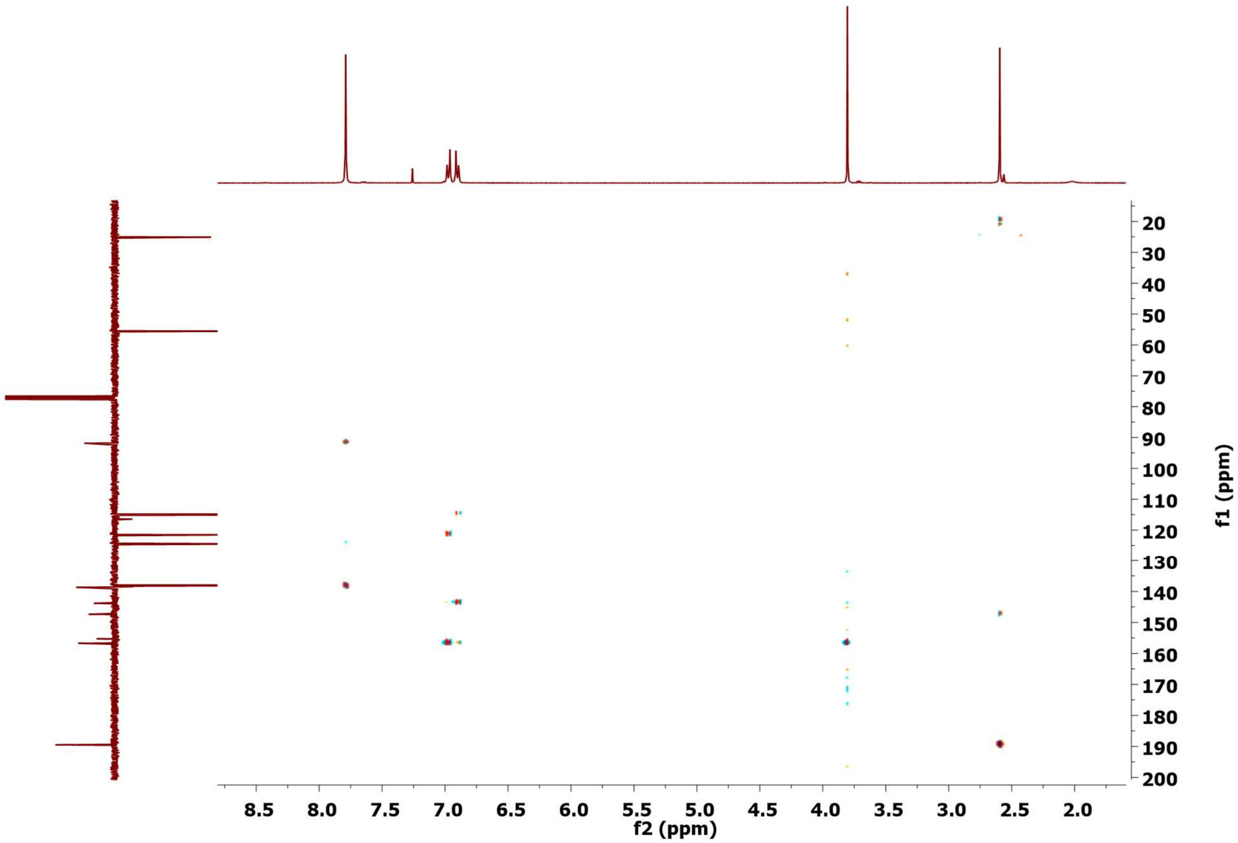

^{1}H NMR ($CDCl_3$) spectrum of (Z)-1-(4-(4-iodophenyl)-5-((4-methoxyphenyl)imino)-4,5-dihydro-1,3,4-thiadiazol-2-yl)ethan-1-one

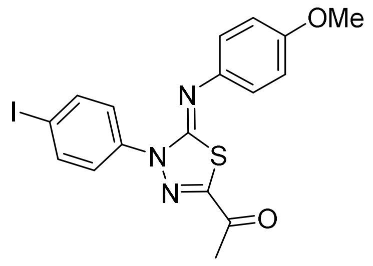

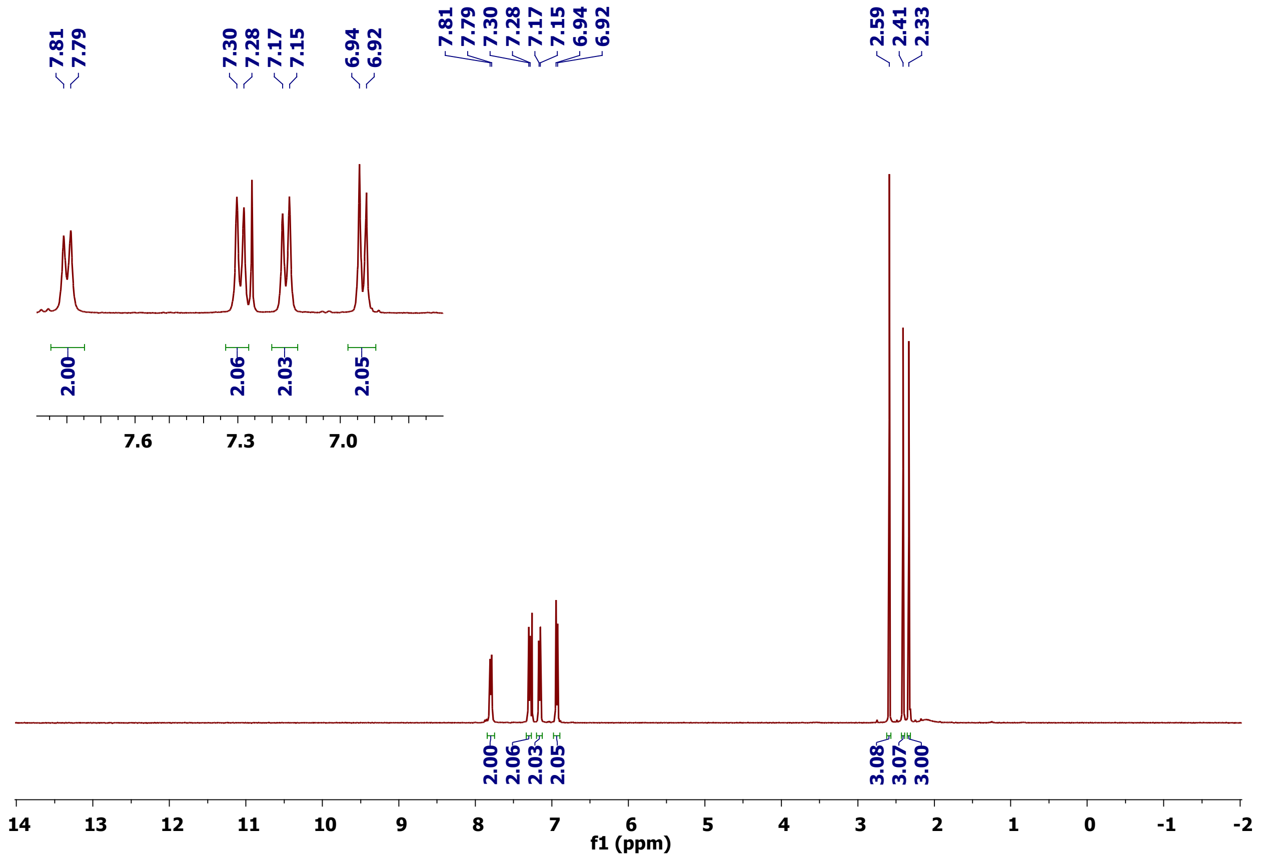

^{13}C NMR ($CDCl_3$) spectrum of (Z)-1-(4-(p-tolyl)-5-(p-tolylimino)-4,5-dihydro-1,3,4-thiadiazol-2-yl)ethan-1-one

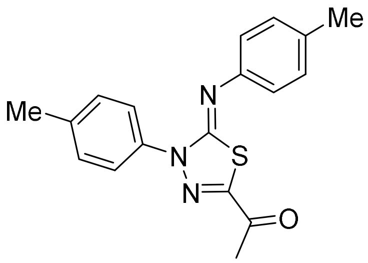

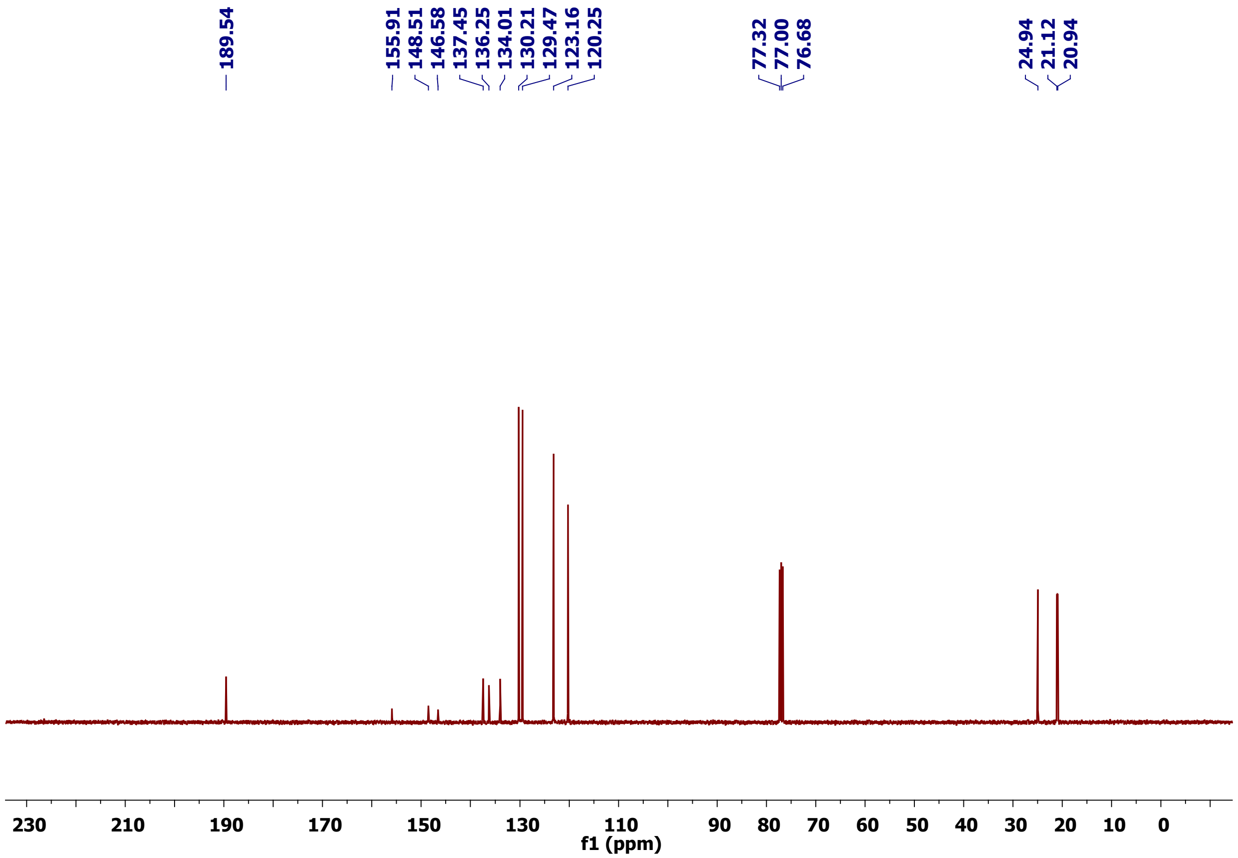

^{13}C CRAPT NMR ($CDCl_3$) spectrum of (Z)-1-(4-(p-tolyl)-5-(p-tolylimino)-4,5-dihydro-1,3,4-thiadiazol-2-yl)ethan-1-one

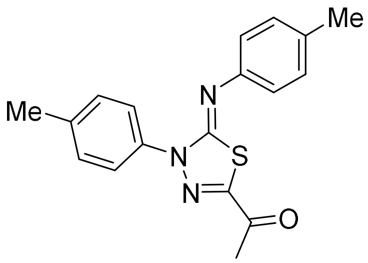

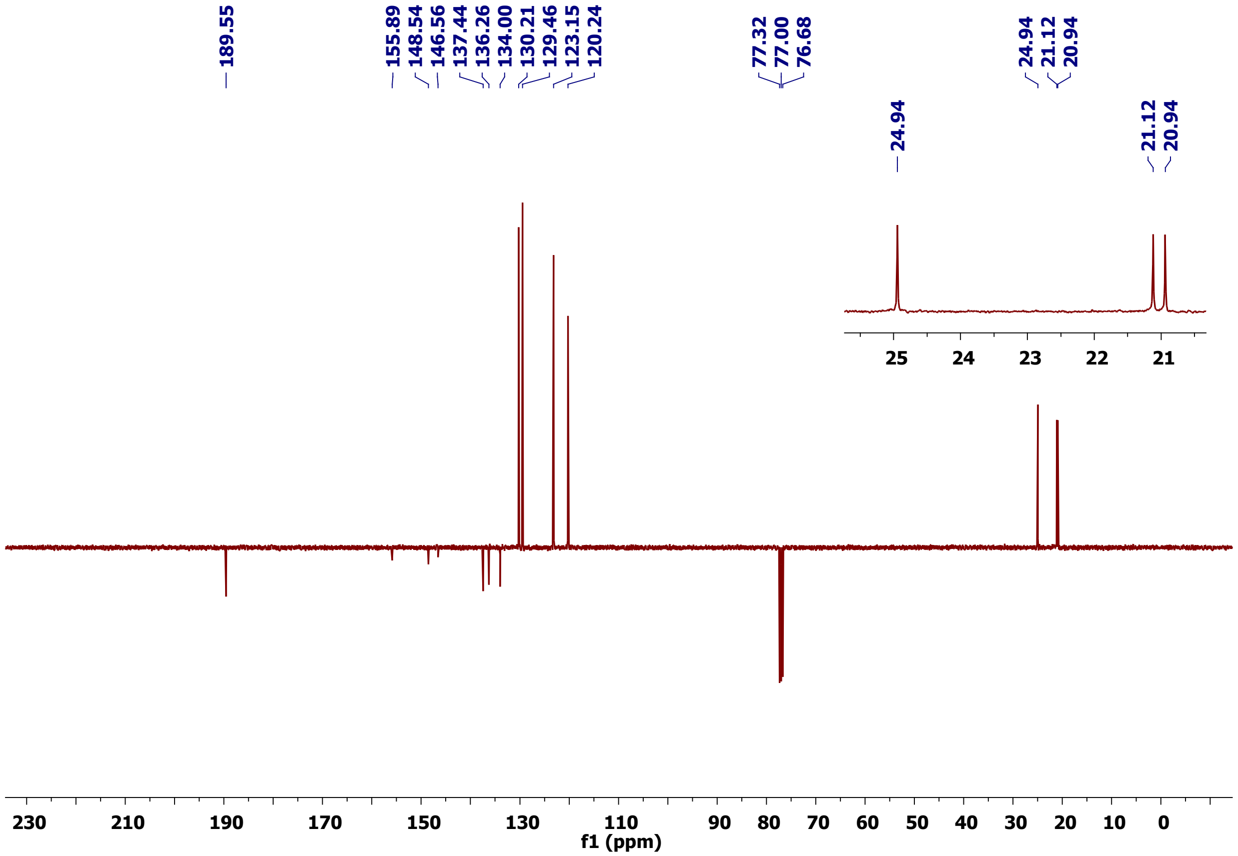

^{1}H-^{1}H gDQCOSY NMR ($CDCl_3$) spectrum of (Z)-1-(4-(p-tolyl)-5-(p-tolylimino)-4,5-dihydro-1,3,4-thiadiazol-2-yl)ethan-1-one

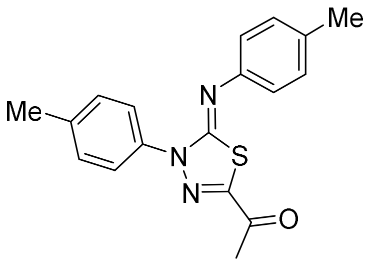

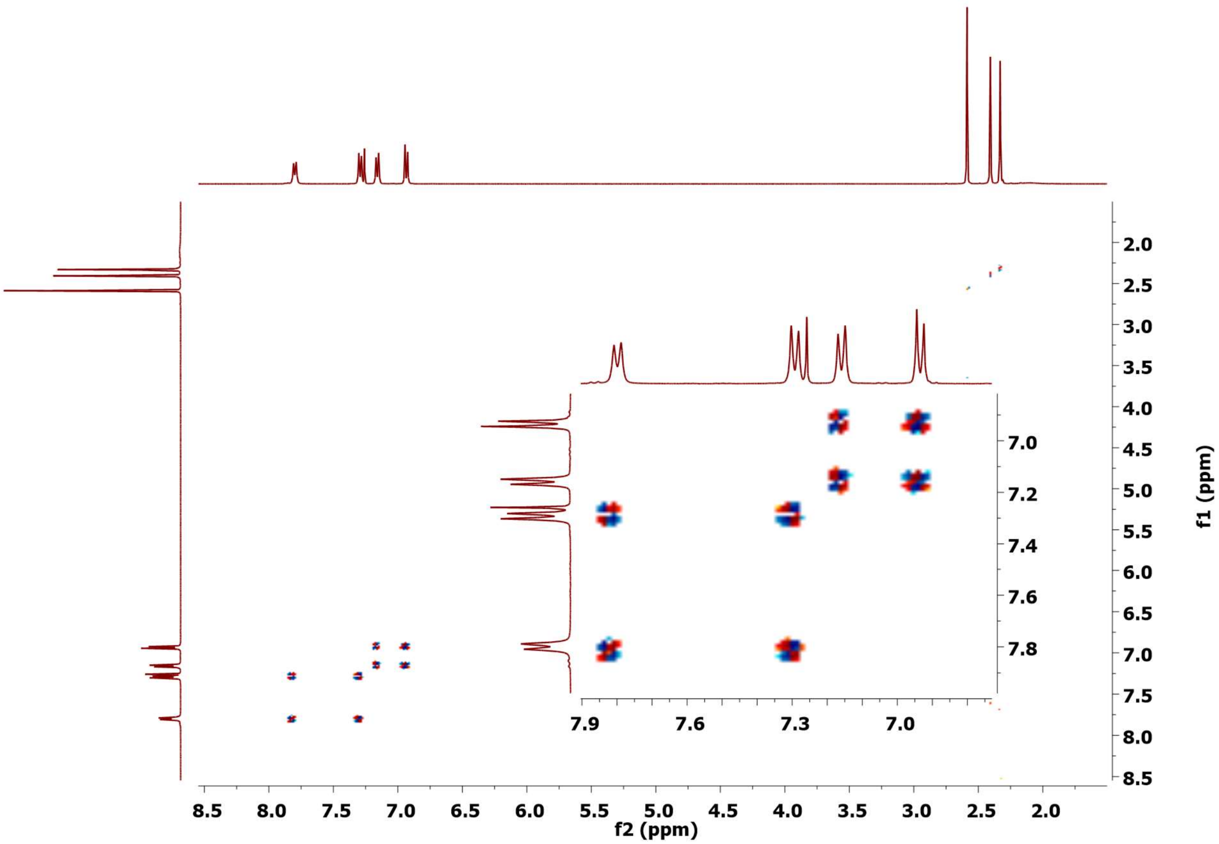

^{1}H-^{13}C-HSQC NMR ($CDCl_3$) spectrum of (Z)-1-(4-(p-tolyl)-5-(p-tolylimino)-4,5-dihydro-1,3,4-thiadiazol-2-yl)ethan-1-one

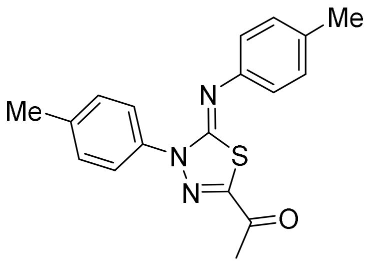

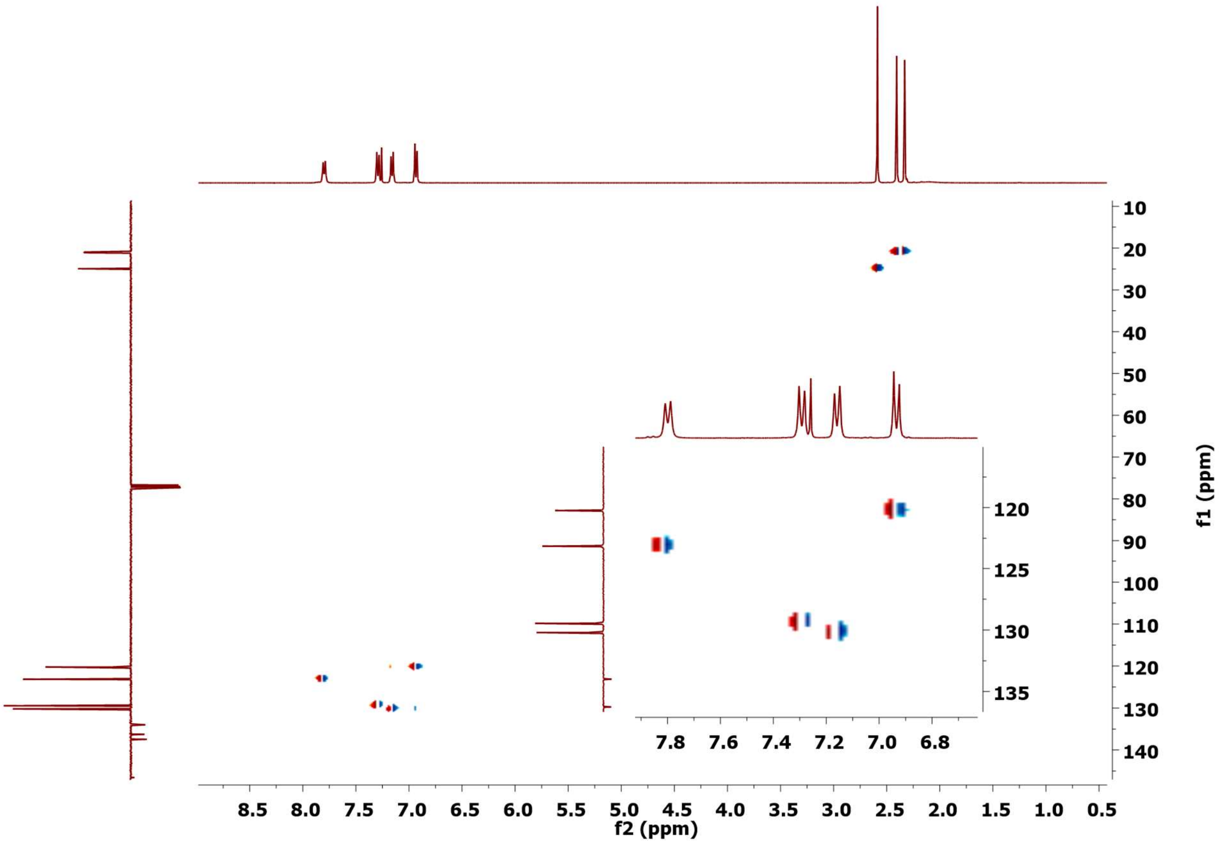

^{1}H-^{13}C-gHMBC NMR ($CDCl_3$) spectrum of (Z)-1-(4-(p-tolyl)-5-(p-tolylimino)-4,5-dihydro-1,3,4-thiadiazol-2-yl)ethan-1-one

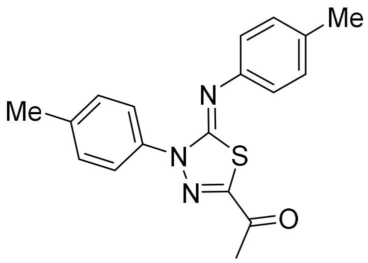

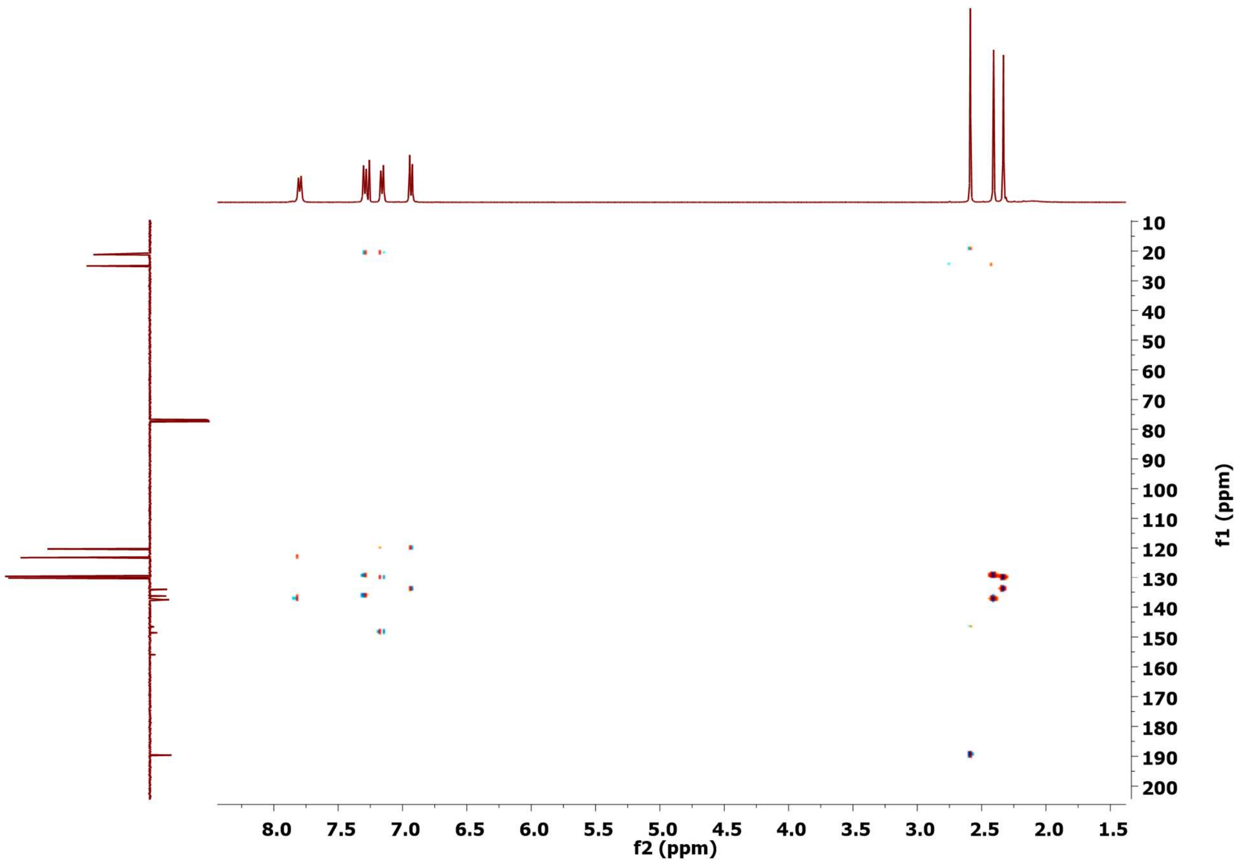

^{1}H NMR ($CDCl_3$) spectrum of (Z)-1-(4-(4-methoxyphenyl)-5-((4-methoxyphenyl)imino)-4,5-dihydro-1,3,4-thiadiazol-2-yl)ethan-1-one

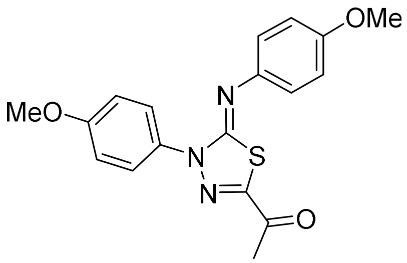

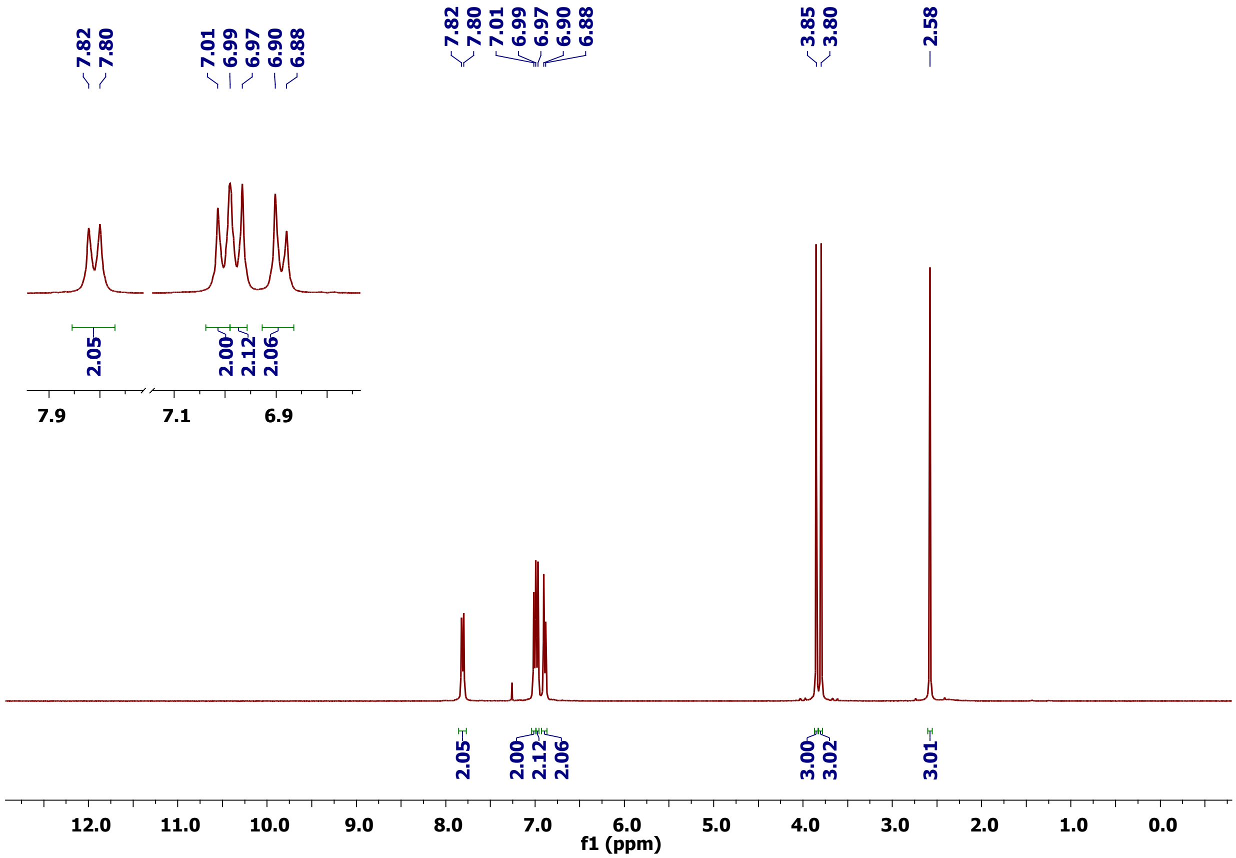

^{13}C NMR ($CDCl_3$) spectrum of (Z)-1-(4-(4-methoxyphenyl)-5-((4-methoxyphenyl)imino)-4,5-dihydro-1,3,4-thiadiazol-2-yl)ethan-1-one

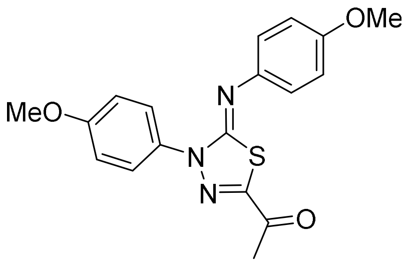

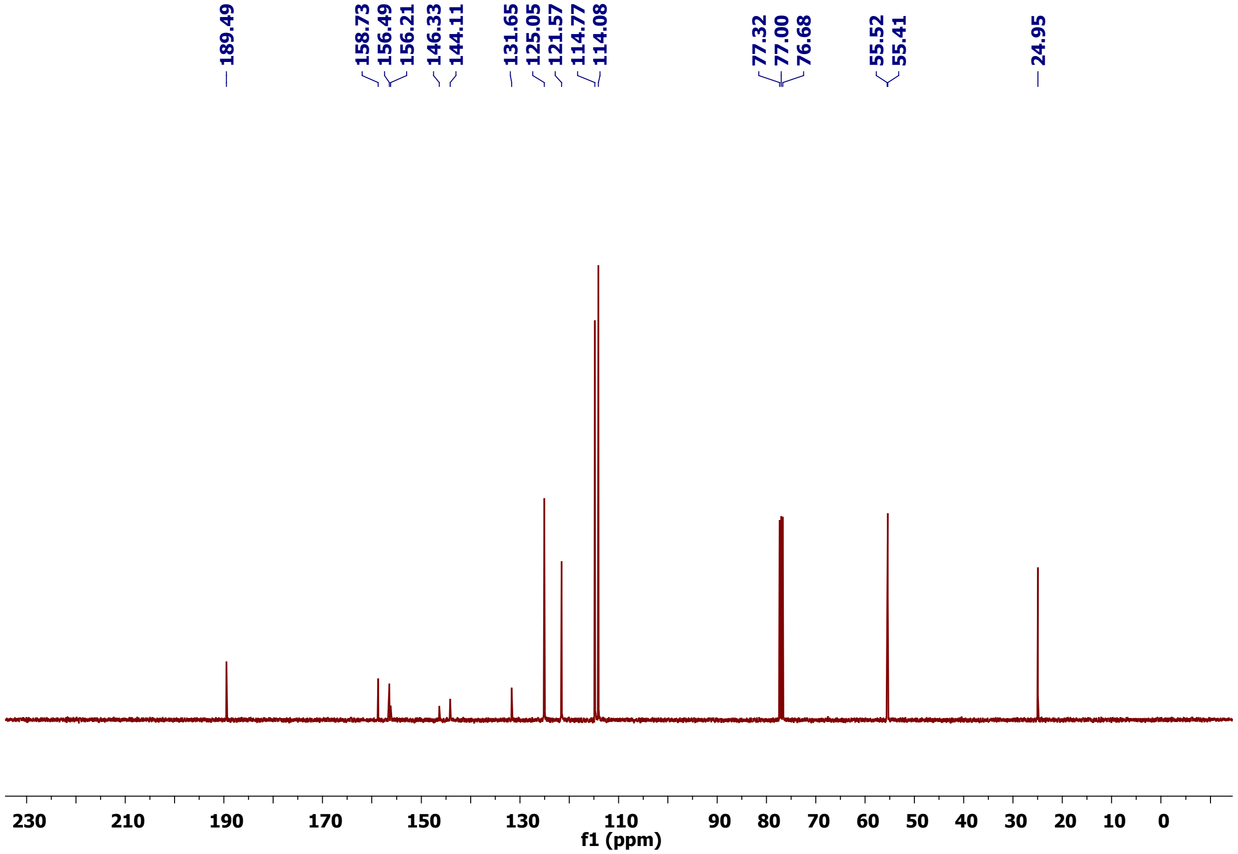

^{13}C CRAPT NMR ($CDCl_3$) spectrum of (Z)-1-(4-(4-methoxyphenyl)-5-((4-methoxyphenyl)imino)-4,5-dihydro-1,3,4-thiadiazol-2-yl)ethan-1-one

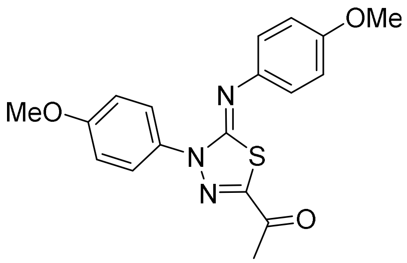

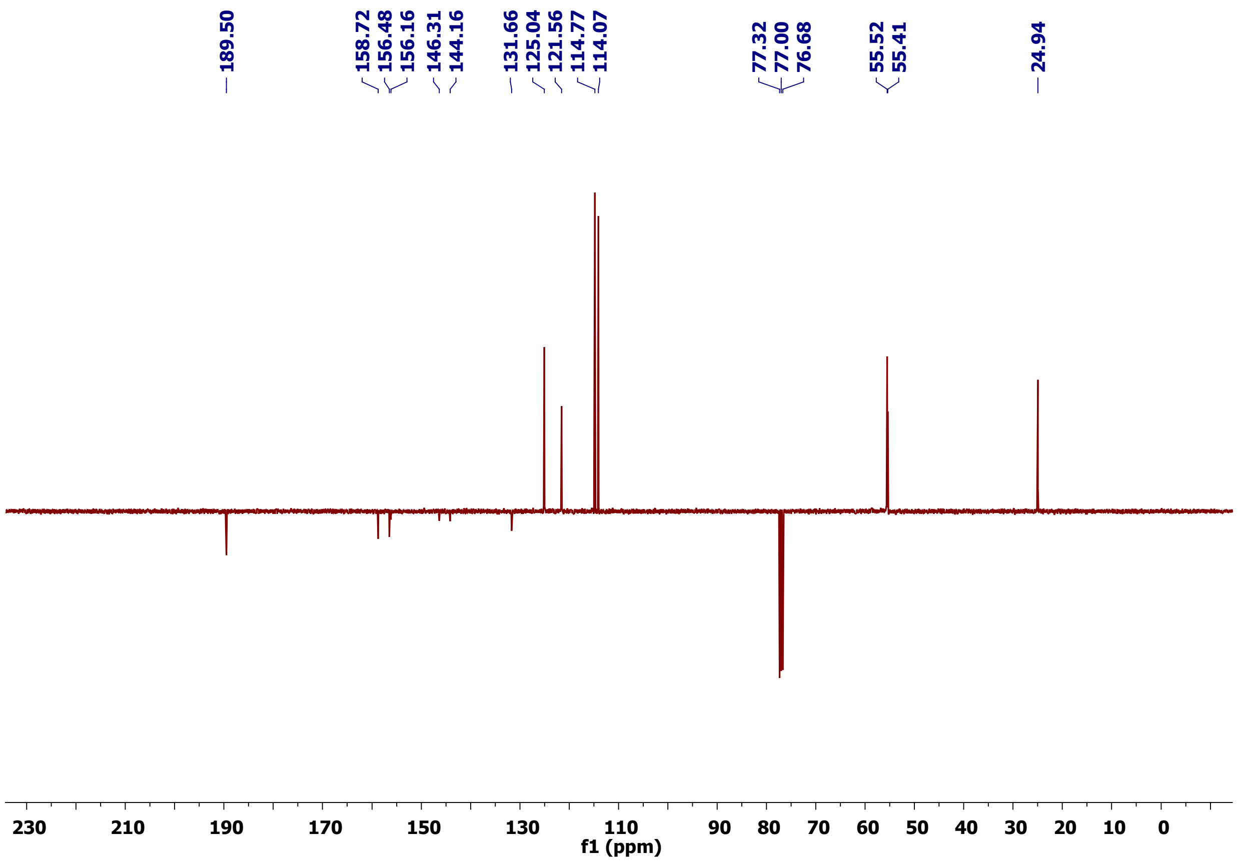

^1H-^1H gDQCOSY NMR ($CDCl_3$) spectrum of (Z)-1-(4-(4-methoxyphenyl)-5-((4-methoxyphenyl)imino)-4,5-dihydro-1,3,4-thiadiazol-2-yl)ethan-1-one

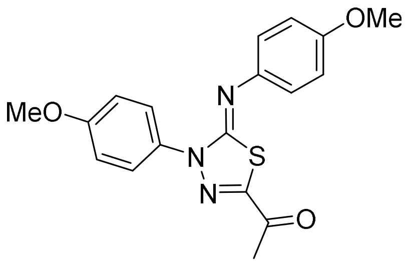

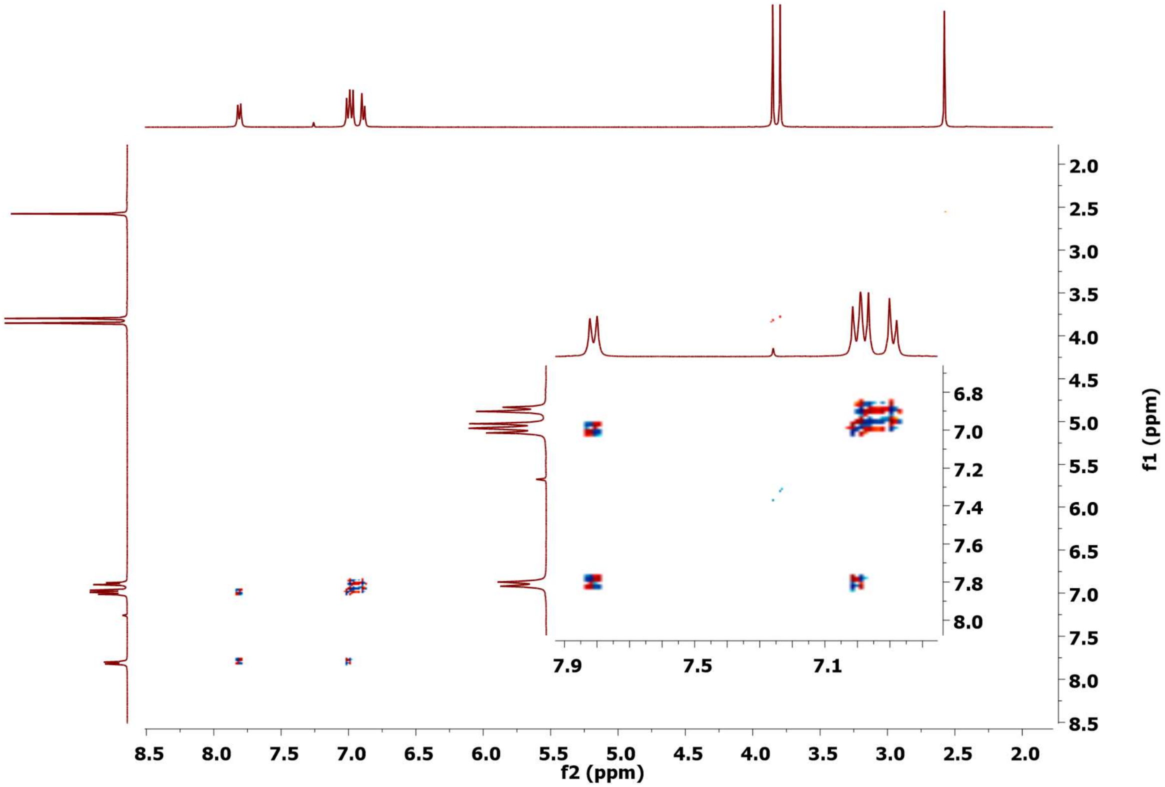

^1H-^{13}C-HSQC NMR ($CDCl_3$) spectrum of (Z)-1-(4-(4-methoxyphenyl)-5-((4-methoxyphenyl)imino)-4,5-dihydro-1,3,4-thiadiazol-2-yl)ethan-1-one

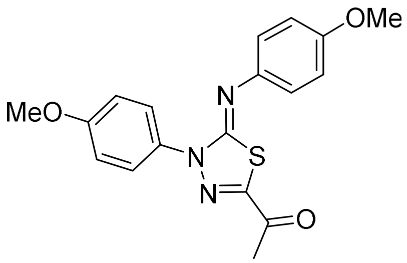

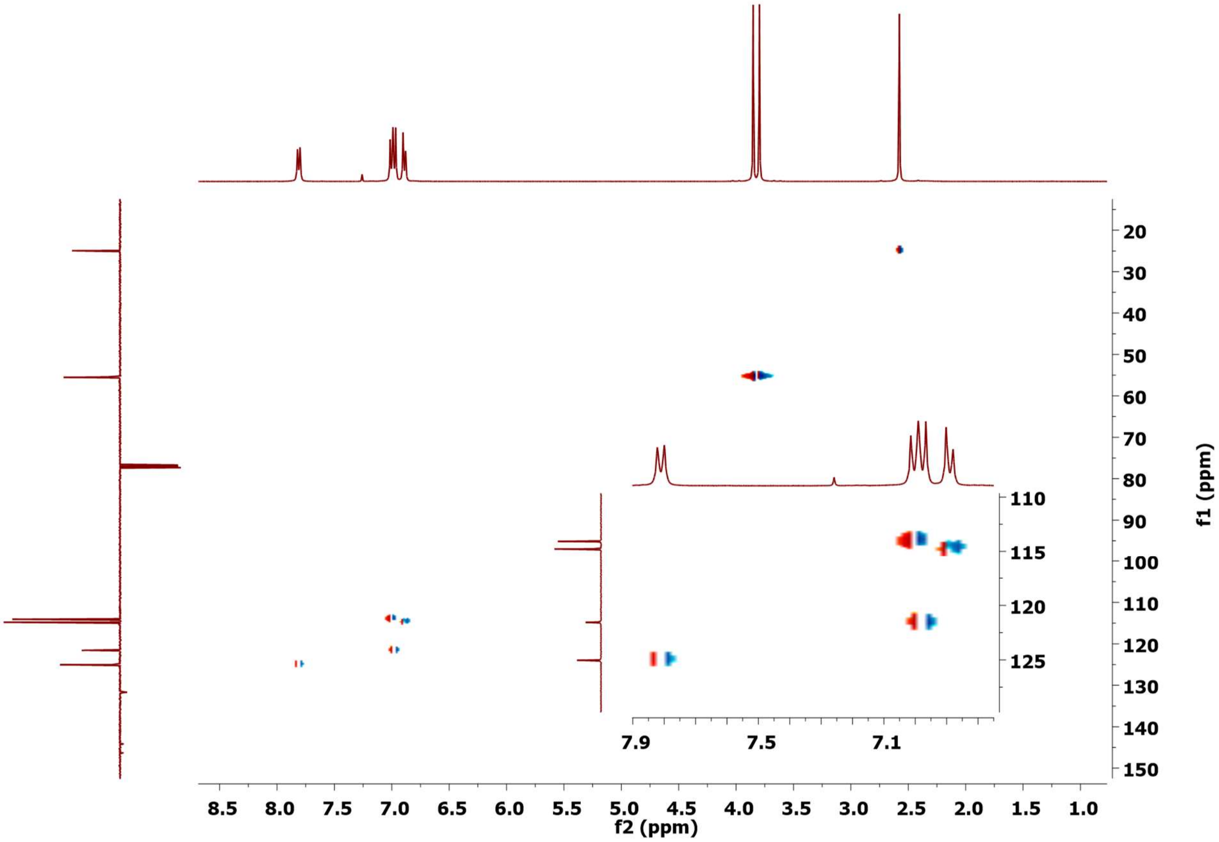

^{1}H-^{13}C-gHMBC NMR ($CDCl_3$) spectrum of (Z)-1-(4-(4-methoxyphenyl)-5-((4-methoxyphenyl)imino)-4,5-dihydro-1,3,4-thiadiazol-2-yl)ethan-1-one

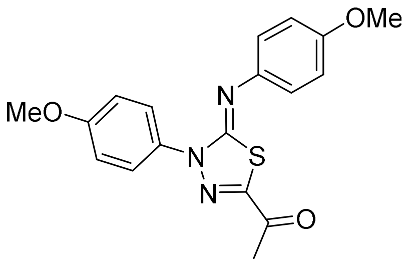

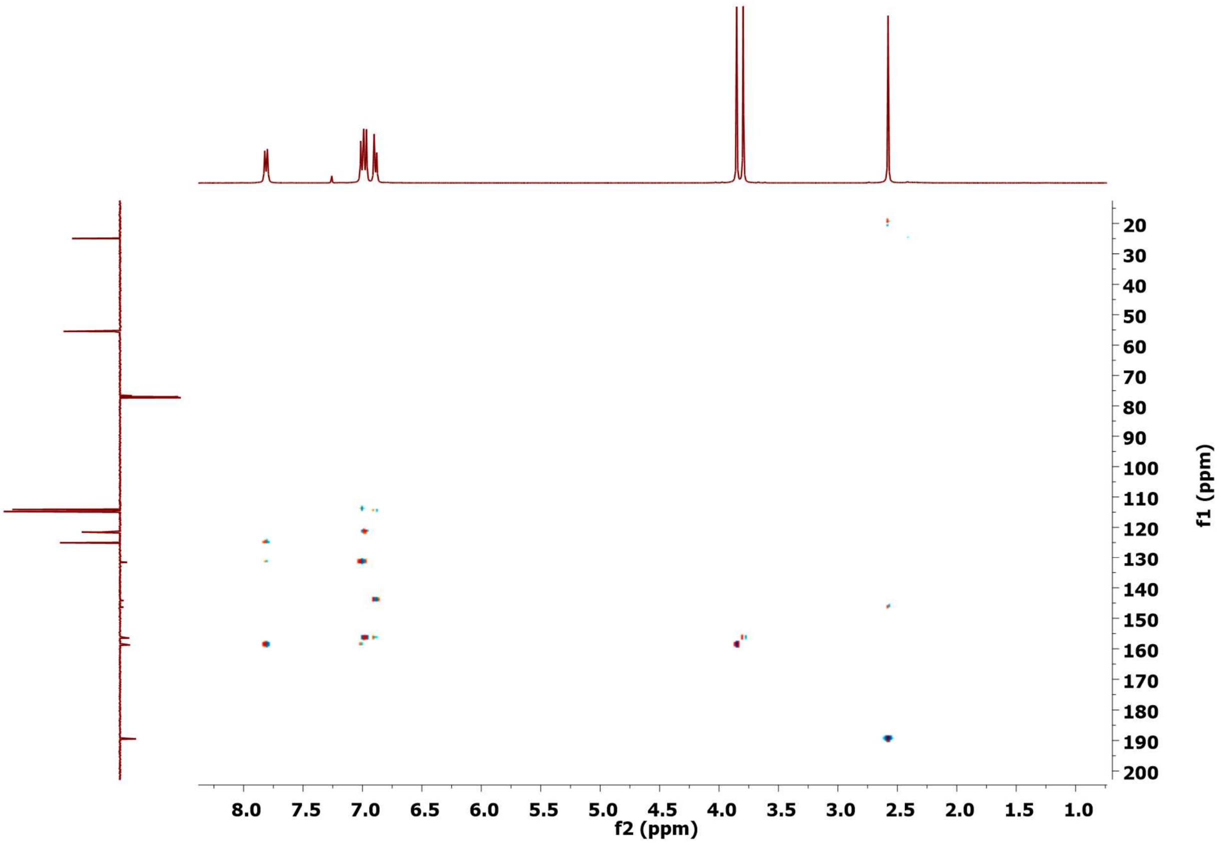

^{1}H NMR ($CDCl_3$) spectrum of (Z)-1-(5-((4-chlorophenyl)imino)-4-(p-tolyl)-4,5-dihydro-1,3,4-thiadiazol-2-yl)ethan-1-one

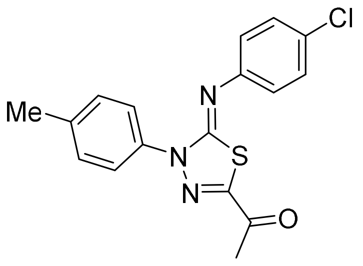

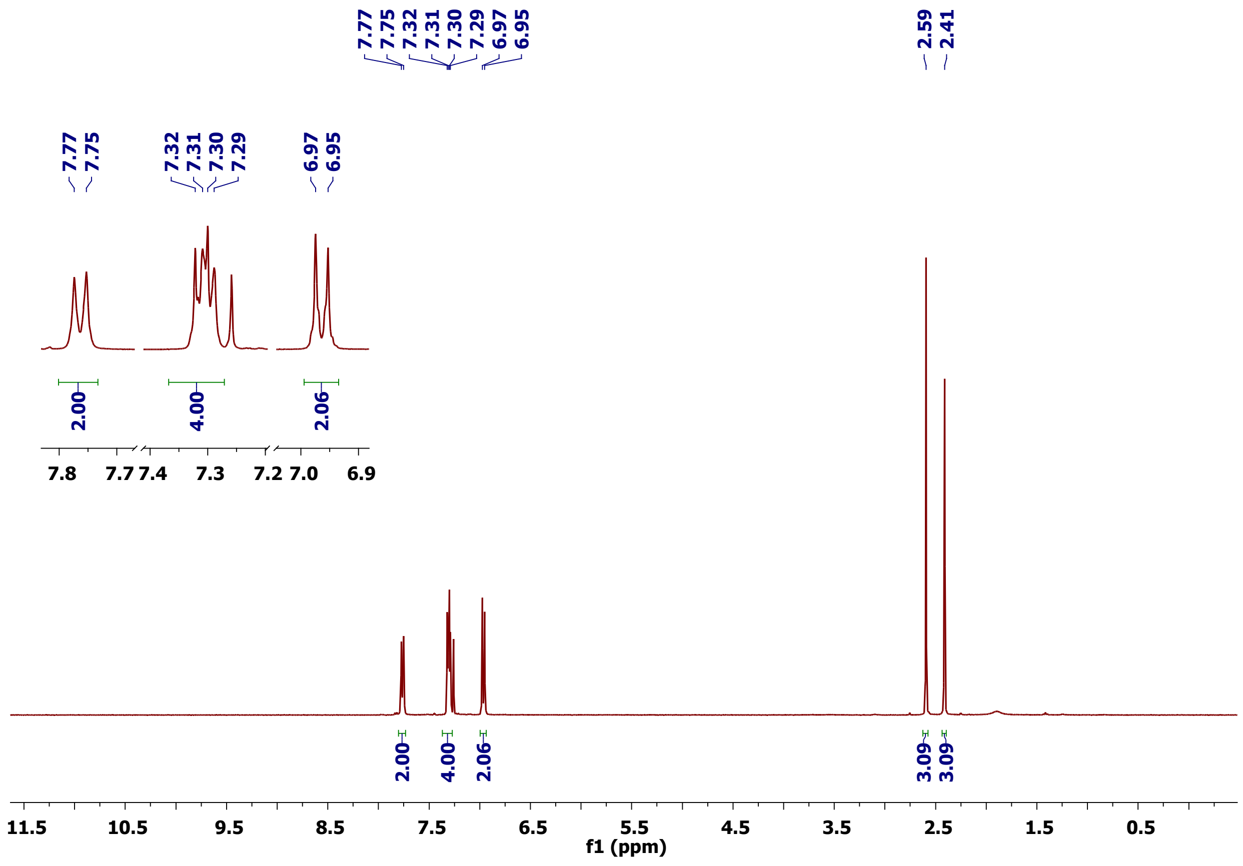

^{13}C NMR ($CDCl_3$) spectrum of (Z)-1-(5-((4-chlorophenyl)imino)-4-(p-tolyl)-4,5-dihydro-1,3,4-thiadiazol-2-yl)ethan-1-one

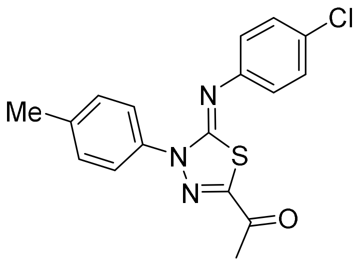

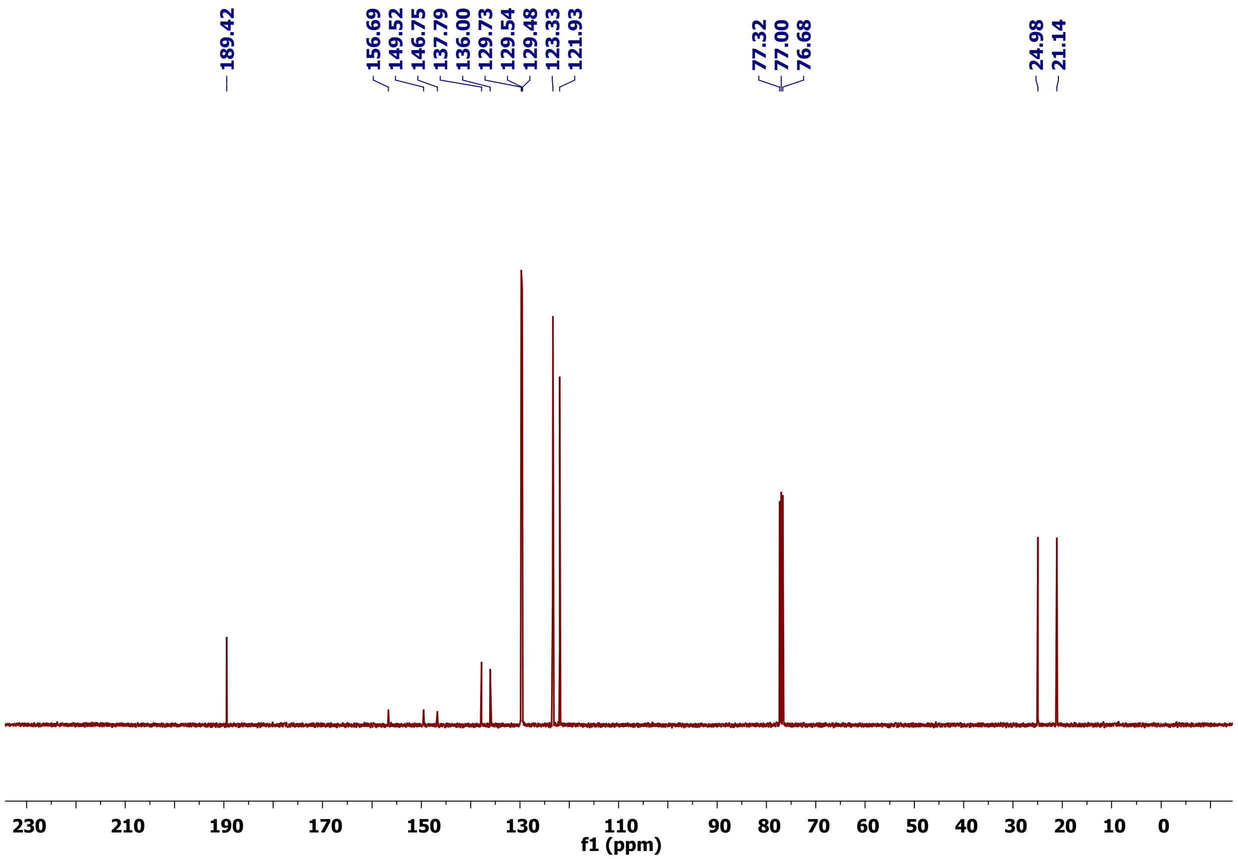

^{13}C CRAPT NMR ($CDCl_3$) spectrum of (Z)-1-(5-((4-chlorophenyl)imino)-4-(p-tolyl)-4,5-dihydro-1,3,4-thiadiazol-2-yl)ethan-1-one

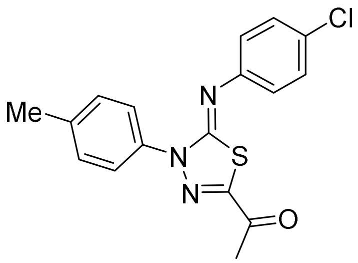

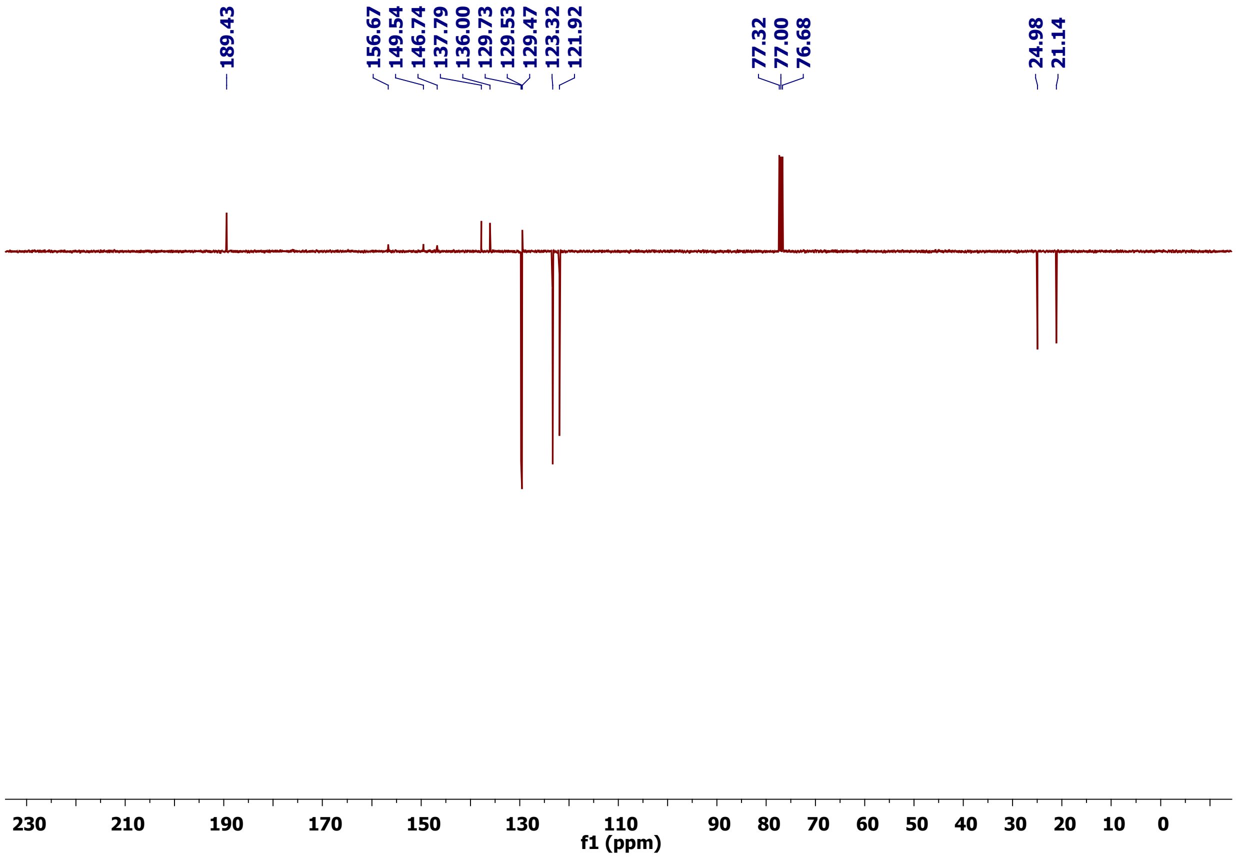

^1H-^1H gDQCOSY NMR ($CDCl_3$) spectrum of (Z)-1-(5-((4-chlorophenyl)imino)-4-(p-tolyl)-4,5-dihydro-1,3,4-thiadiazol-2-yl)ethan-1-one

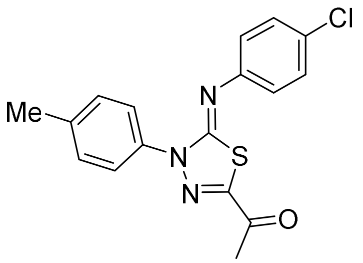

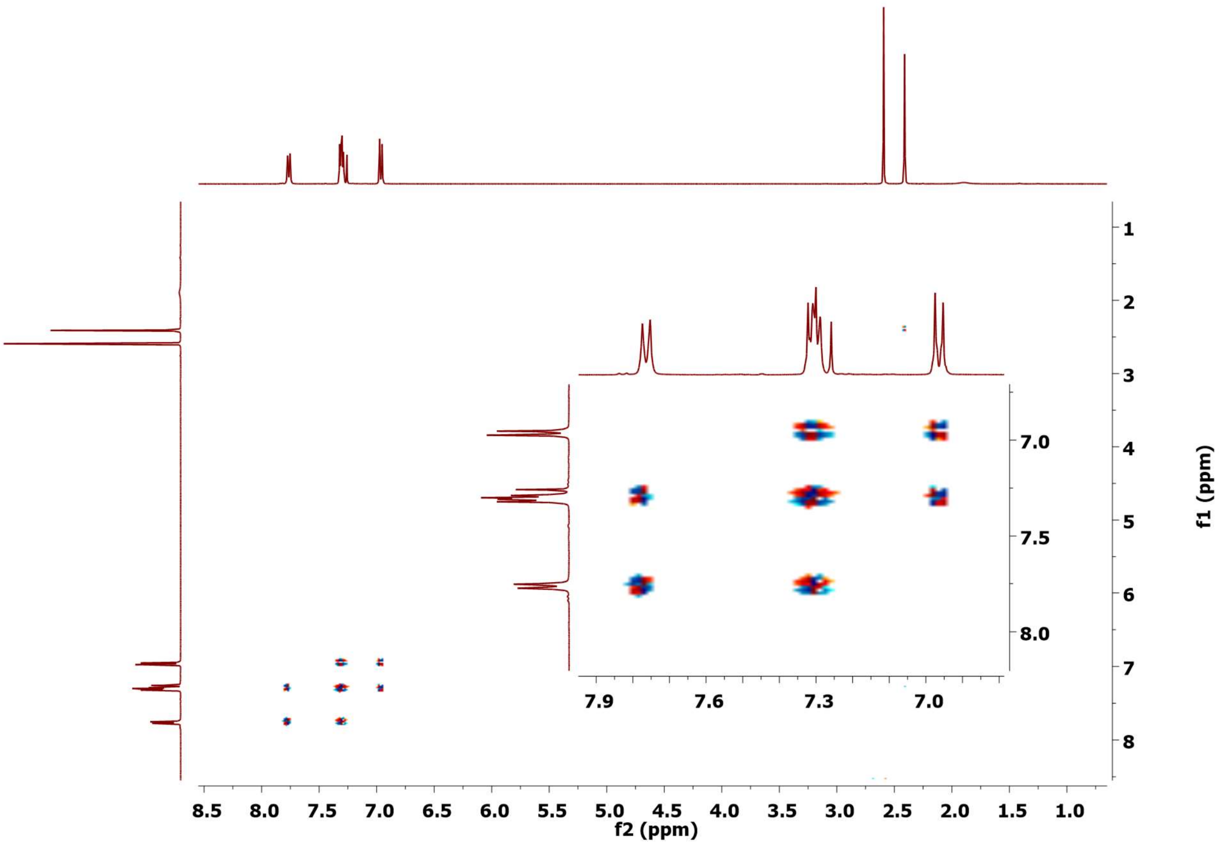

^{1}H-^{13}C-HSQC NMR ($CDCl_3$) spectrum of (Z)-1-(5-((4-chlorophenyl)imino)-4-(p-tolyl)-4,5-dihydro-1,3,4-thiadiazol-2-yl)ethan-1-one

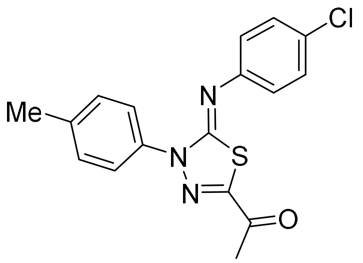

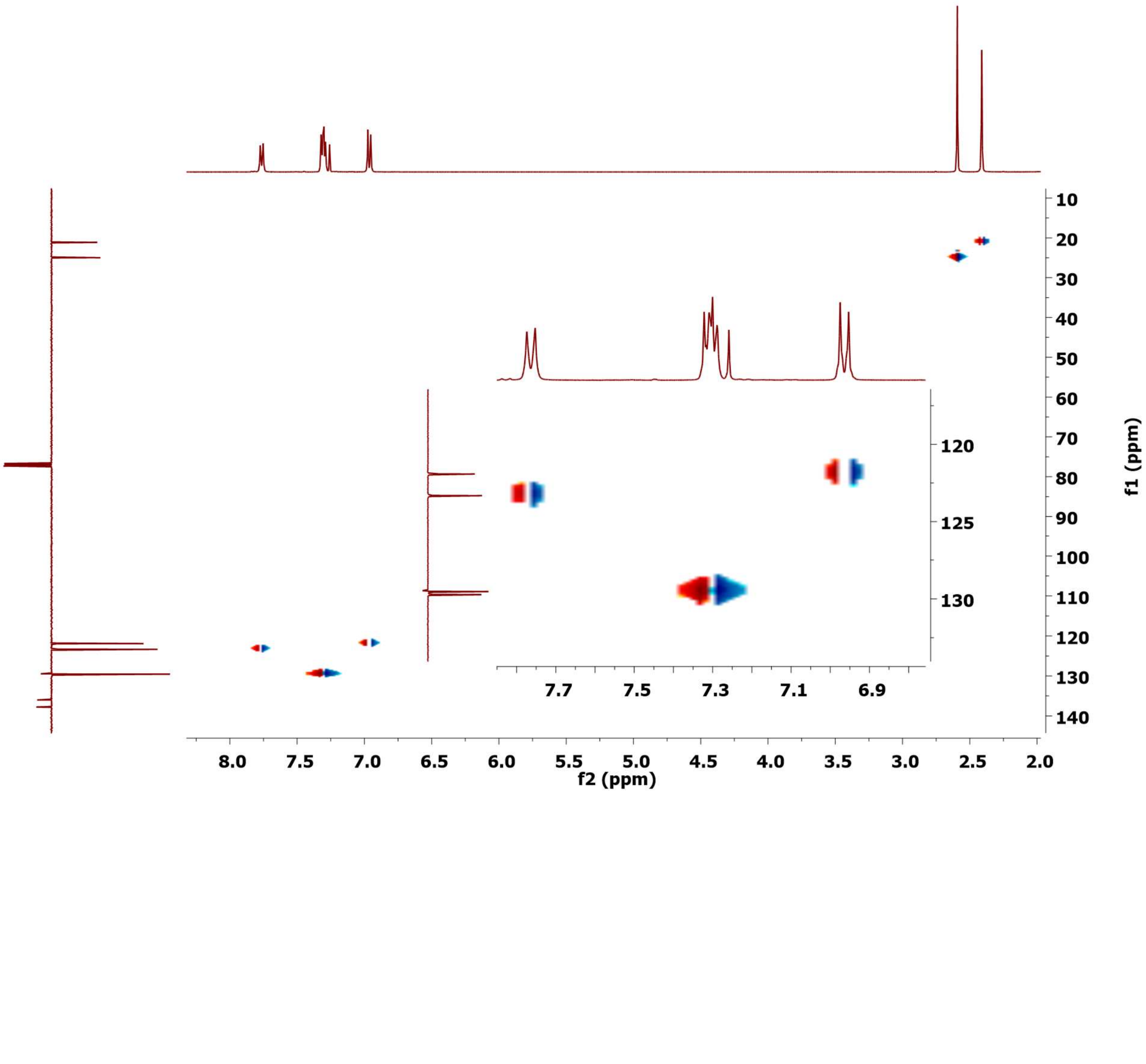

^1H-^{13}C-gHMBC NMR ($CDCl_3$) spectrum of (Z)-1-(5-((4-chlorophenyl)imino)-4-(p-tolyl)-4,5-dihydro-1,3,4-thiadiazol-2-yl)ethan-1-one

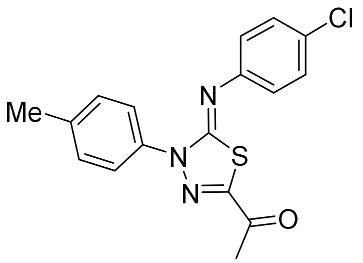

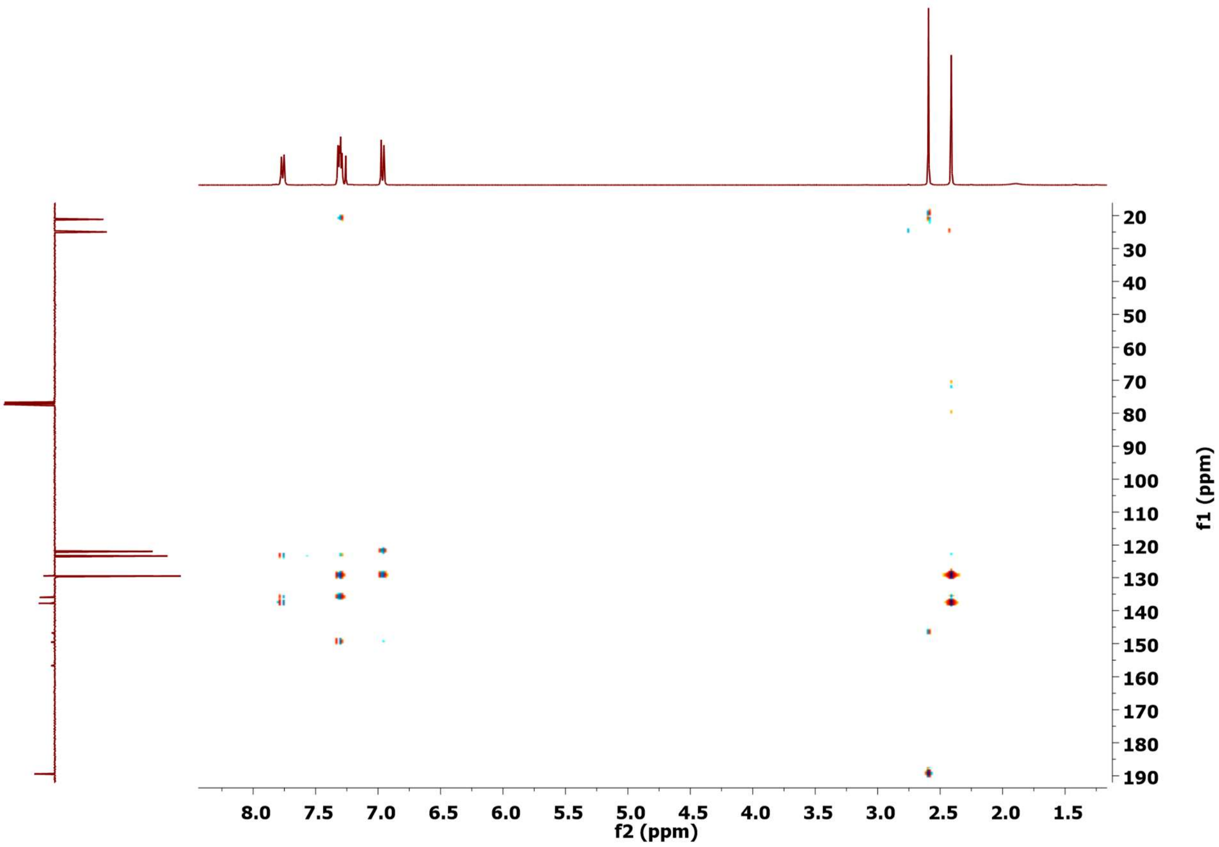

^{1}H NMR ($CDCl_3$) spectrum of (Z)-1-(4-(4-chlorophenyl)-5-((4-nitrophenyl)imino)-4,5-dihydro-1,3,4-thiadiazol-2-yl)ethan-1-one

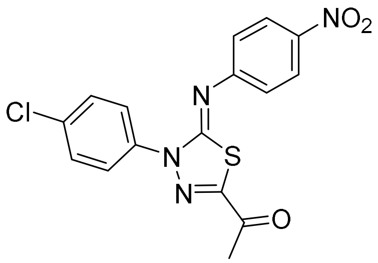

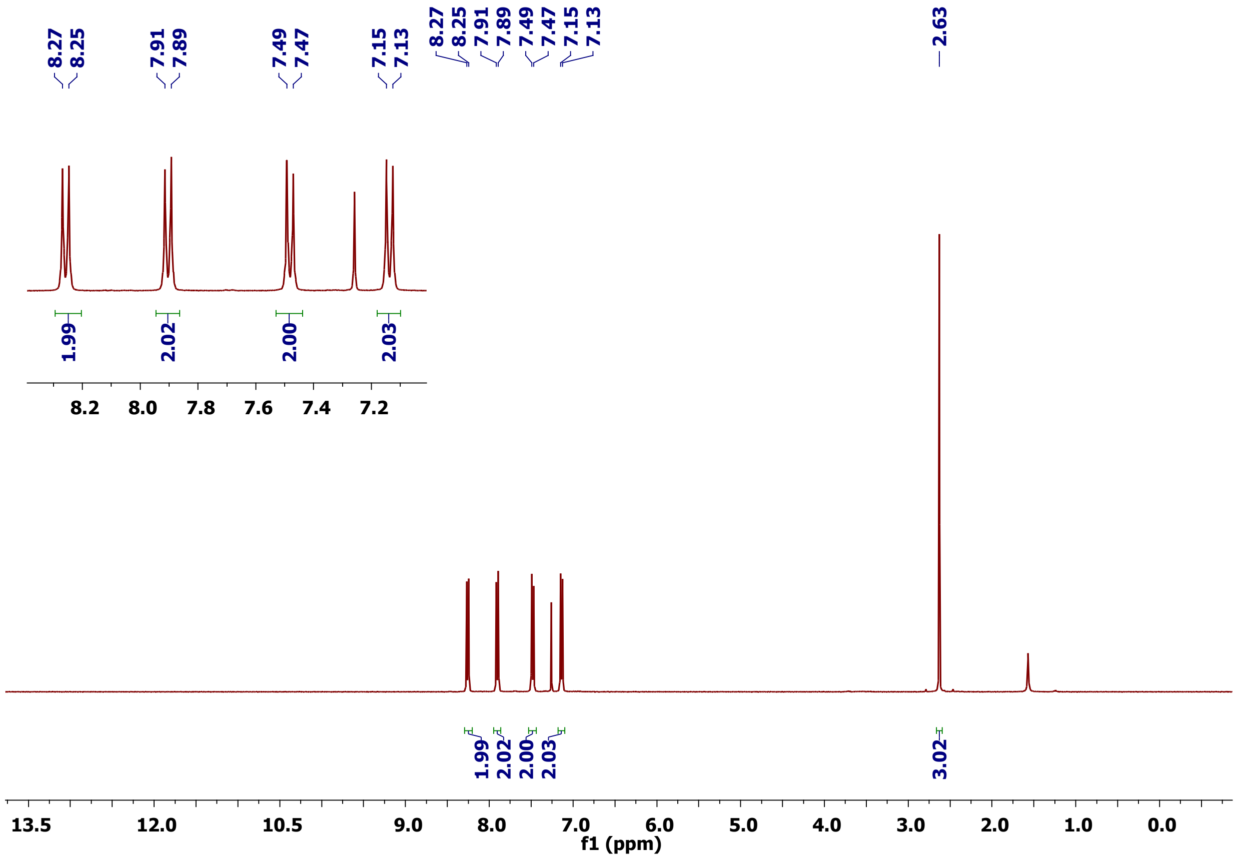

^{13}C NMR ($CDCl_3$) spectrum of (Z)-1-(4-(4-chlorophenyl)-5-((4-nitrophenyl)imino)-4,5-dihydro-1,3,4-thiadiazol-2-yl)ethan-1-one

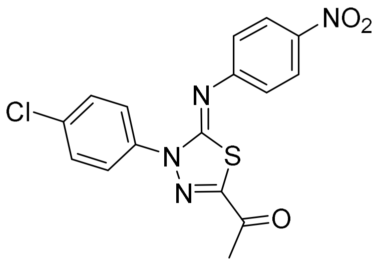

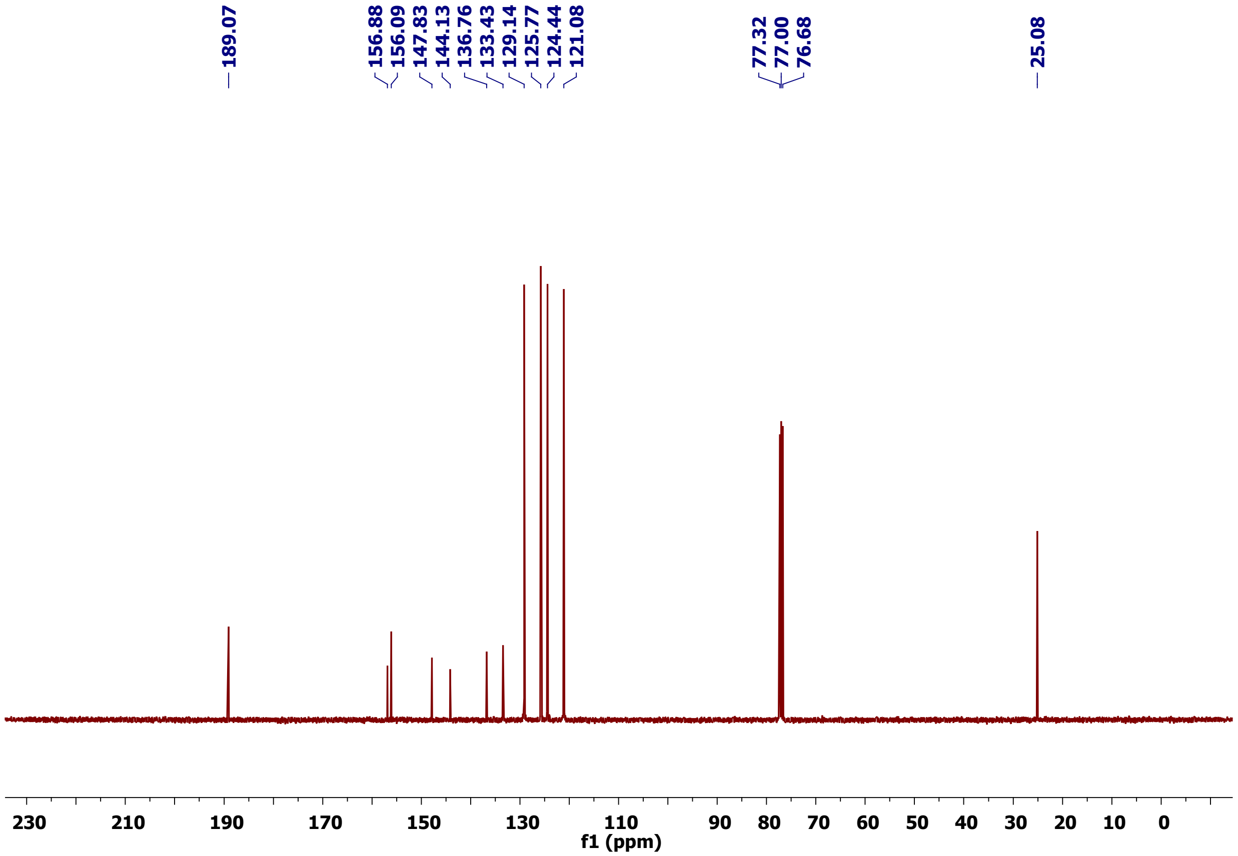

^{13}C CRAPT NMR ($CDCl_3$) spectrum of (Z)-1-(4-(4-chlorophenyl)-5-((4-nitrophenyl)imino)-4,5-dihydro-1,3,4-thiadiazol-2-yl)ethan-1-one

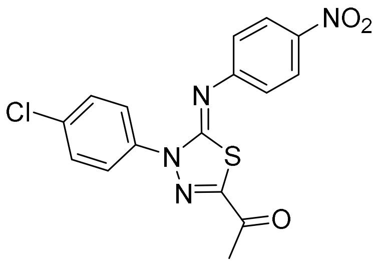

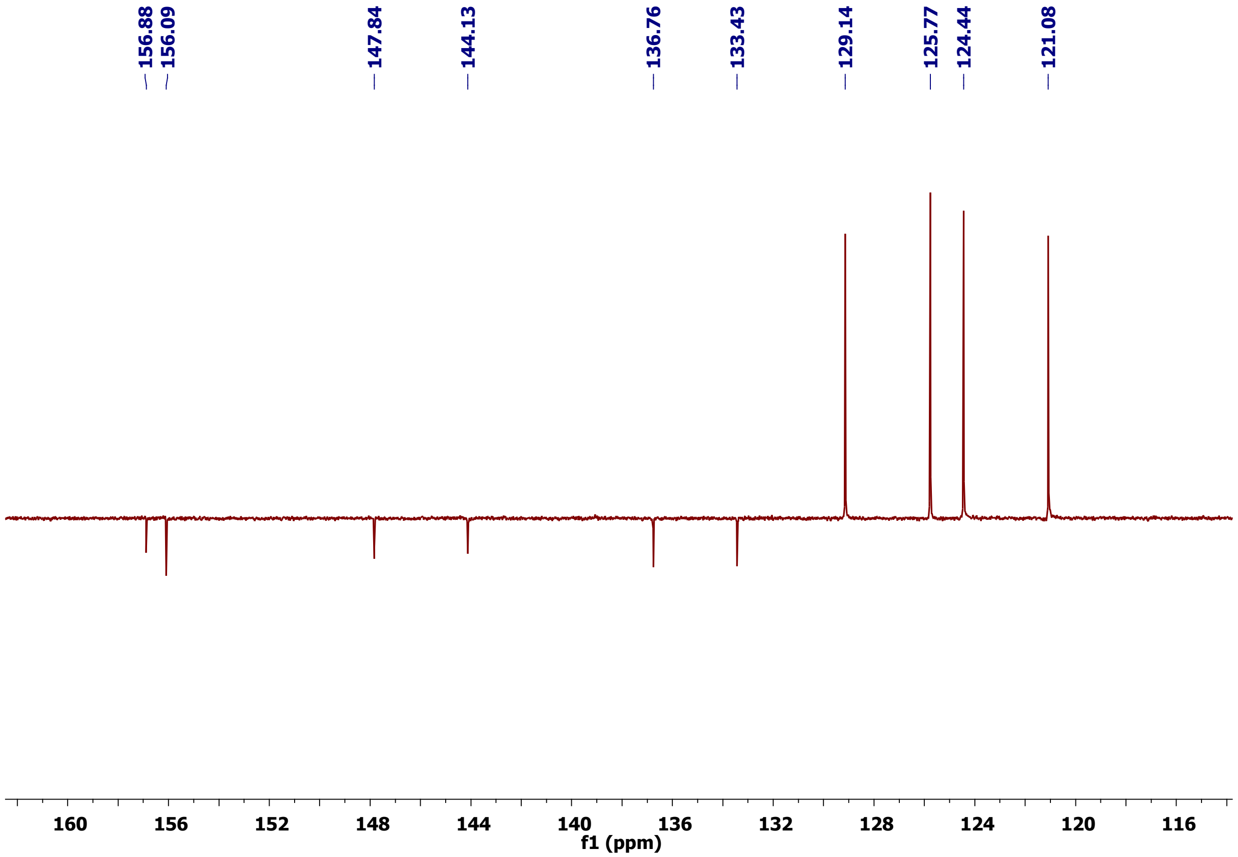

^{1}H-^{1}H gDQCOSY NMR ($CDCl_3$) spectrum of (Z)-1-(4-(4-chlorophenyl)-5-((4-nitrophenyl)imino)-4,5-dihydro-1,3,4-thiadiazol-2-yl)ethan-1-one

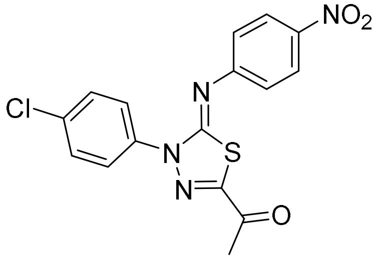

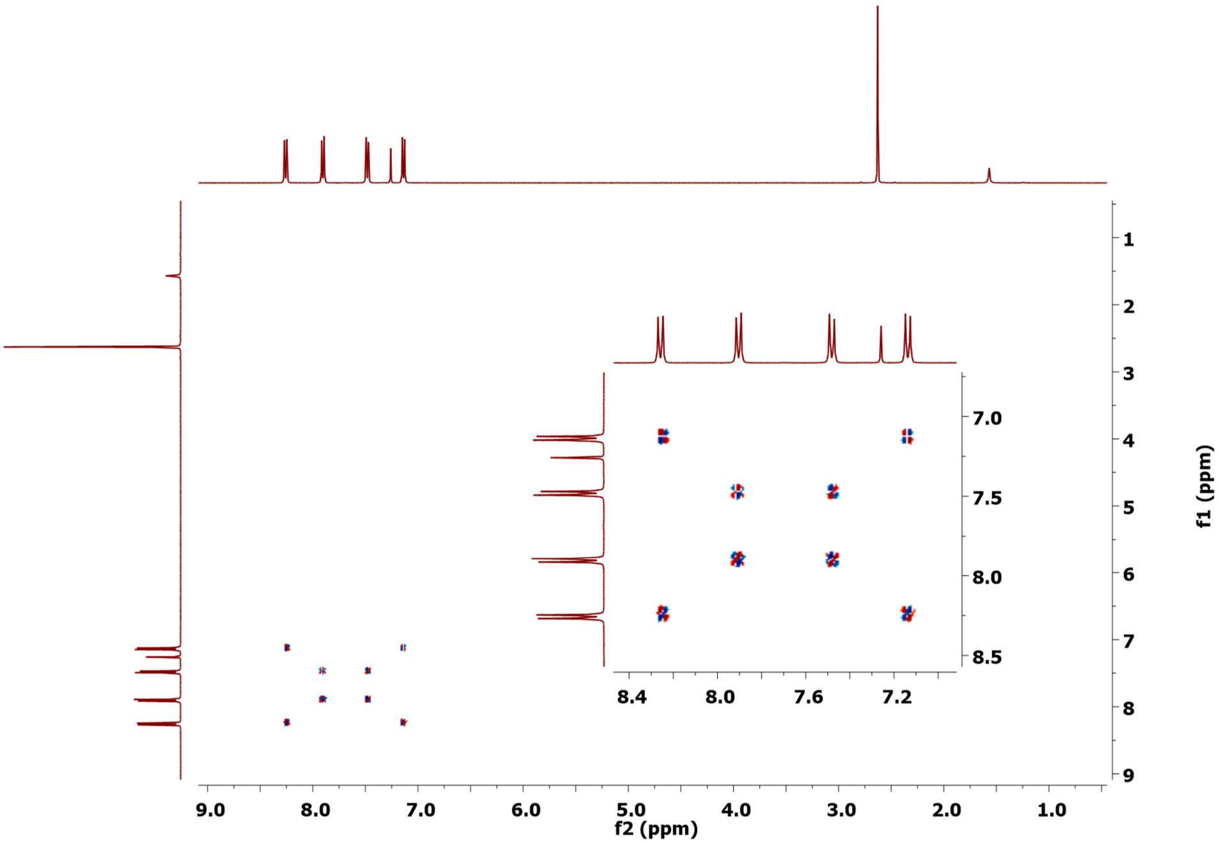

^{1}H-^{13}C-HSQC NMR ($CDCl_3$) spectrum of (Z)-1-(4-(4-chlorophenyl)-5-((4-nitrophenyl)imino)-4,5-dihydro-1,3,4-thiadiazol-2-yl)ethan-1-one

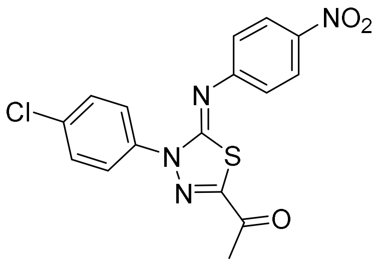

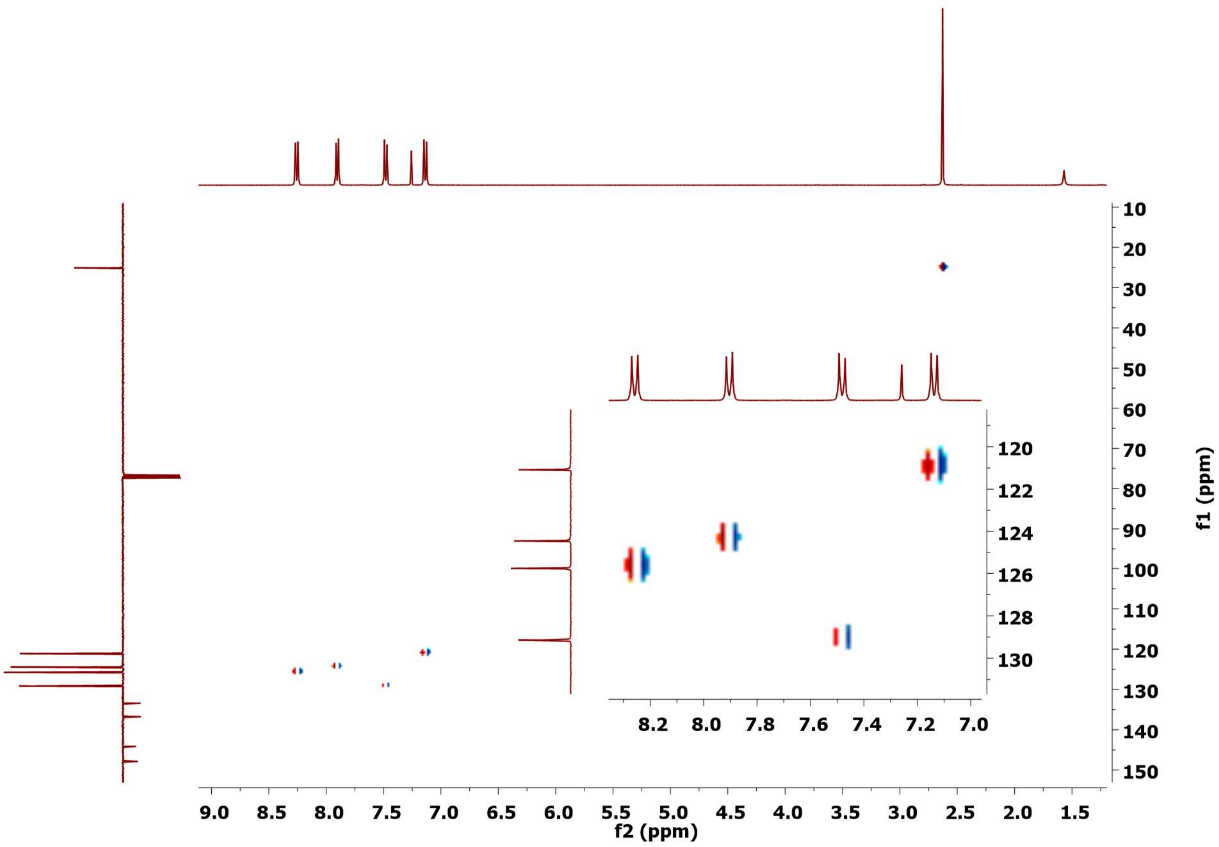

^{1}H-^{13}C-gHMBC NMR ($CDCl_3$) spectrum of (Z)-1-(4-(4-chlorophenyl)-5-((4-nitrophenyl)imino)-4,5-dihydro-1,3,4-thiadiazol-2-yl)ethan-1-one

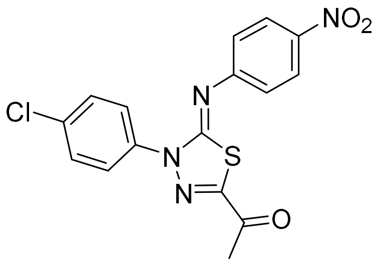

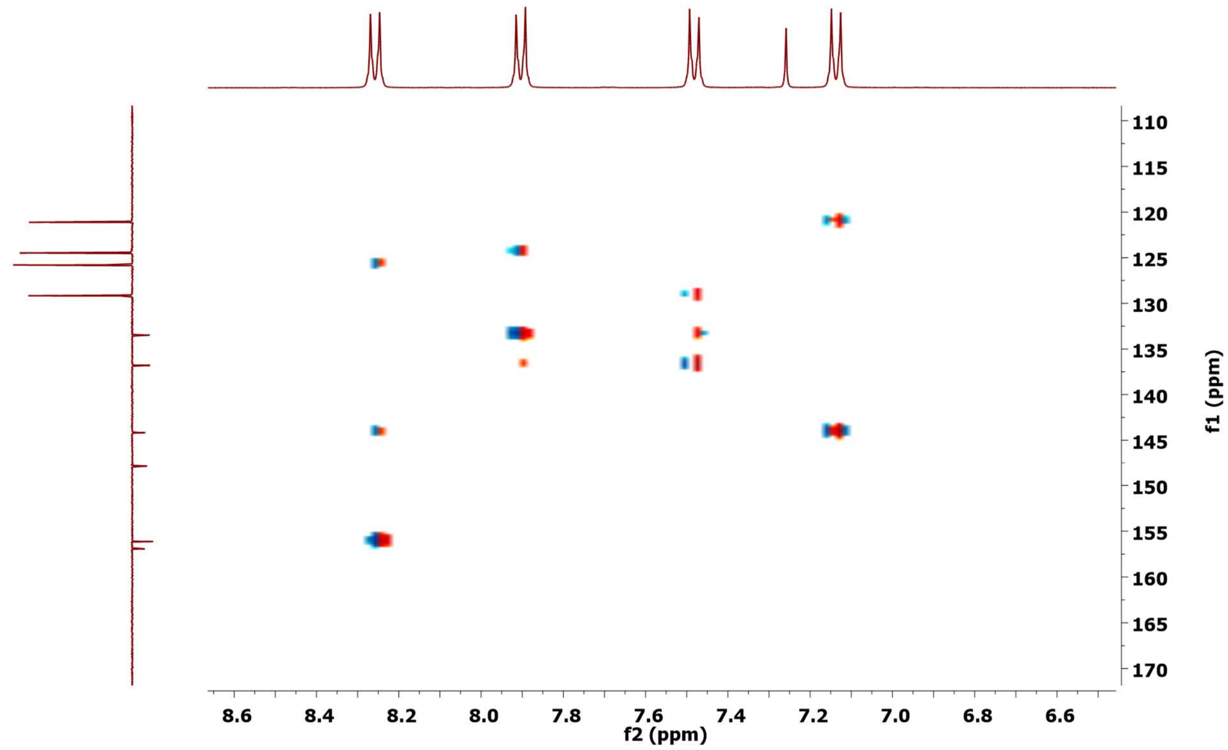

^{1}H NMR ($CDCl_3$) spectrum of (Z)-1-(4-(4-bromophenyl)-5-((4-nitrophenyl)imino)-4,5-dihydro-1,3,4-thiadiazol-2-yl)ethan-1-one

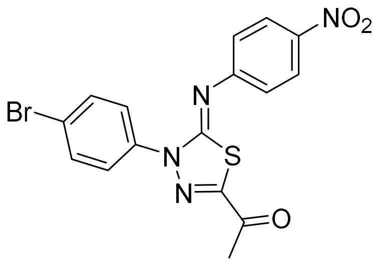

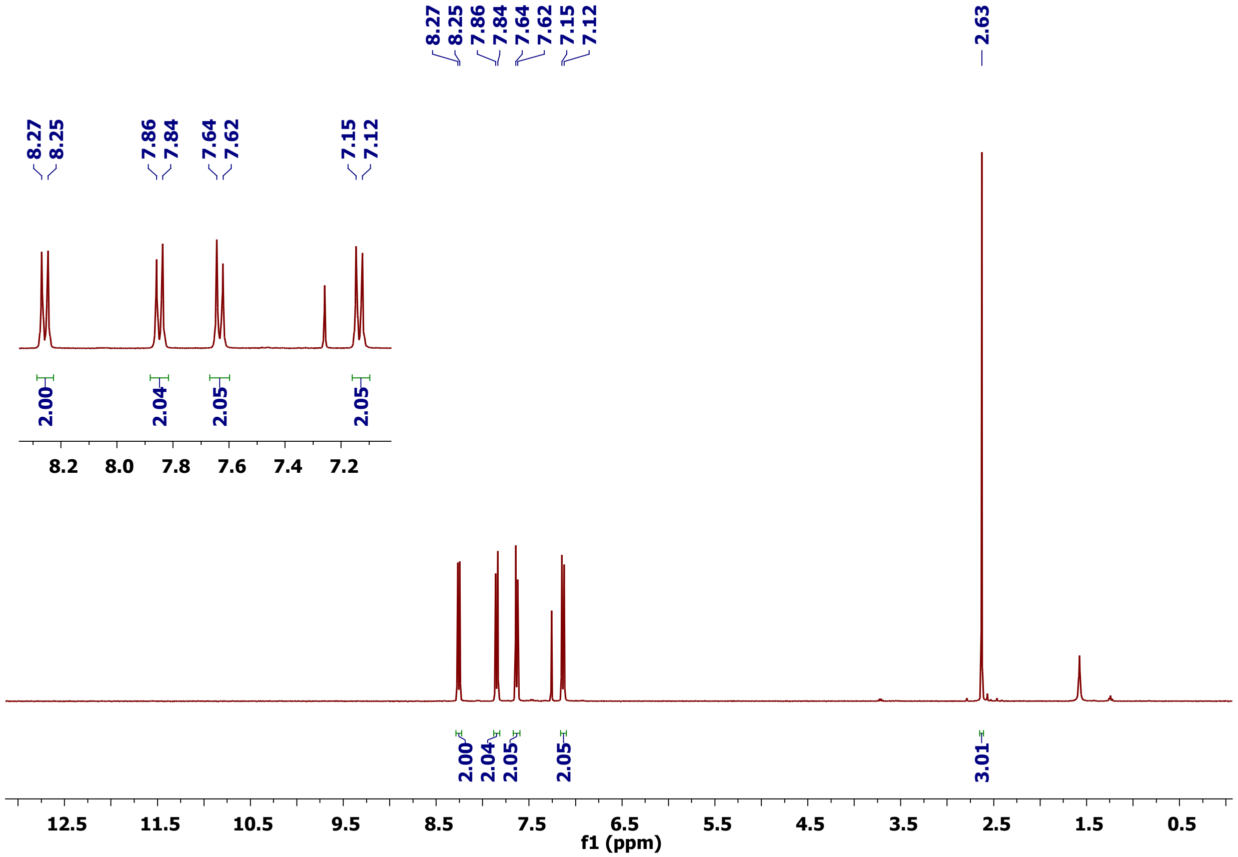

^{13}C NMR ($CDCl_3$) spectrum of (Z)-1-(4-(4-bromophenyl)-5-((4-nitrophenyl)imino)-4,5-dihydro-1,3,4-thiadiazol-2-yl)ethan-1-one

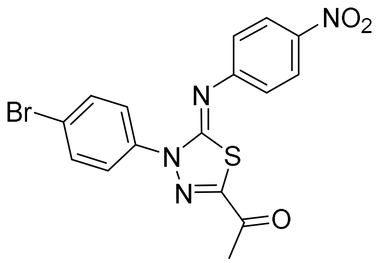

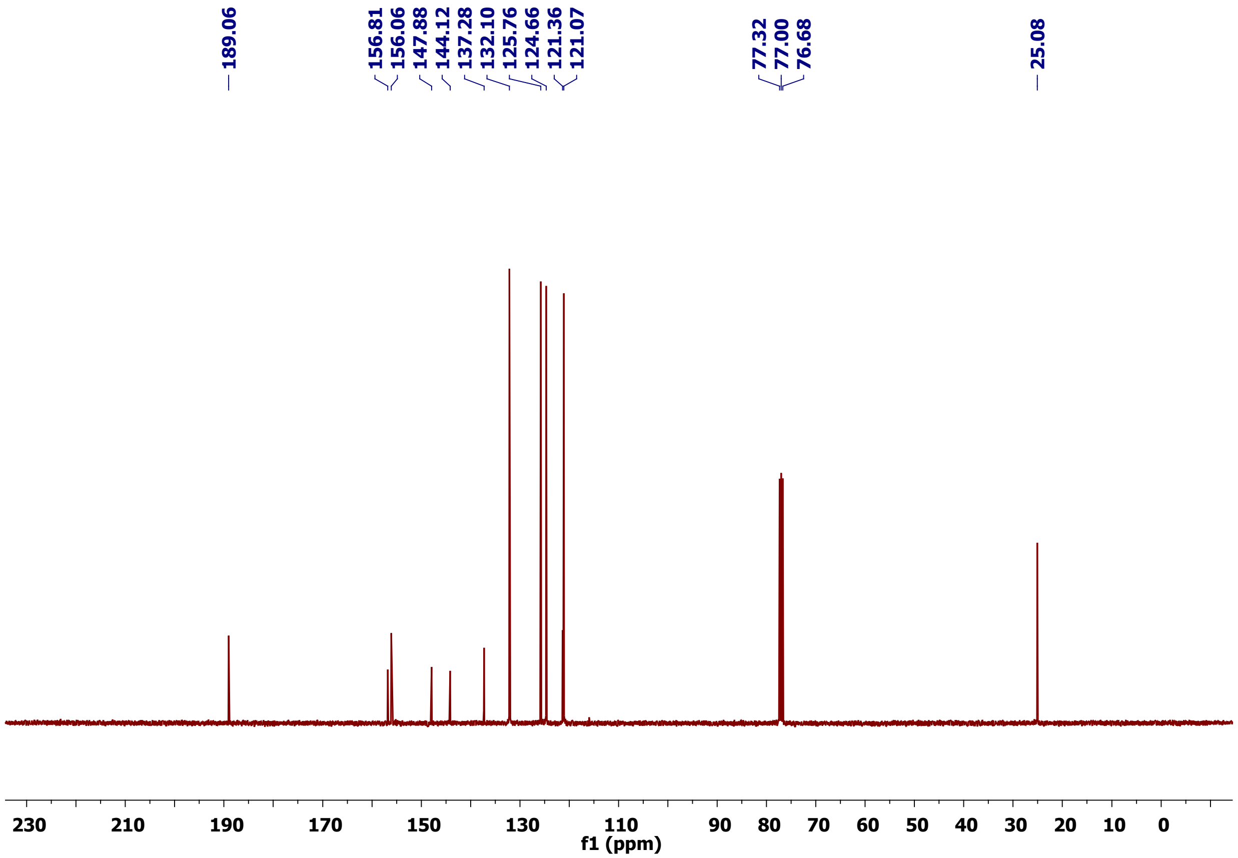

^{13}C CRAPT NMR ($CDCl_3$) spectrum of (Z)-1-(4-(4-bromophenyl)-5-((4-nitrophenyl)imino)-4,5-dihydro-1,3,4-thiadiazol-2-yl)ethan-1-one

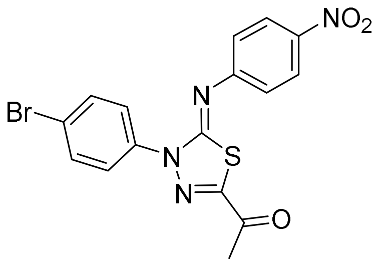

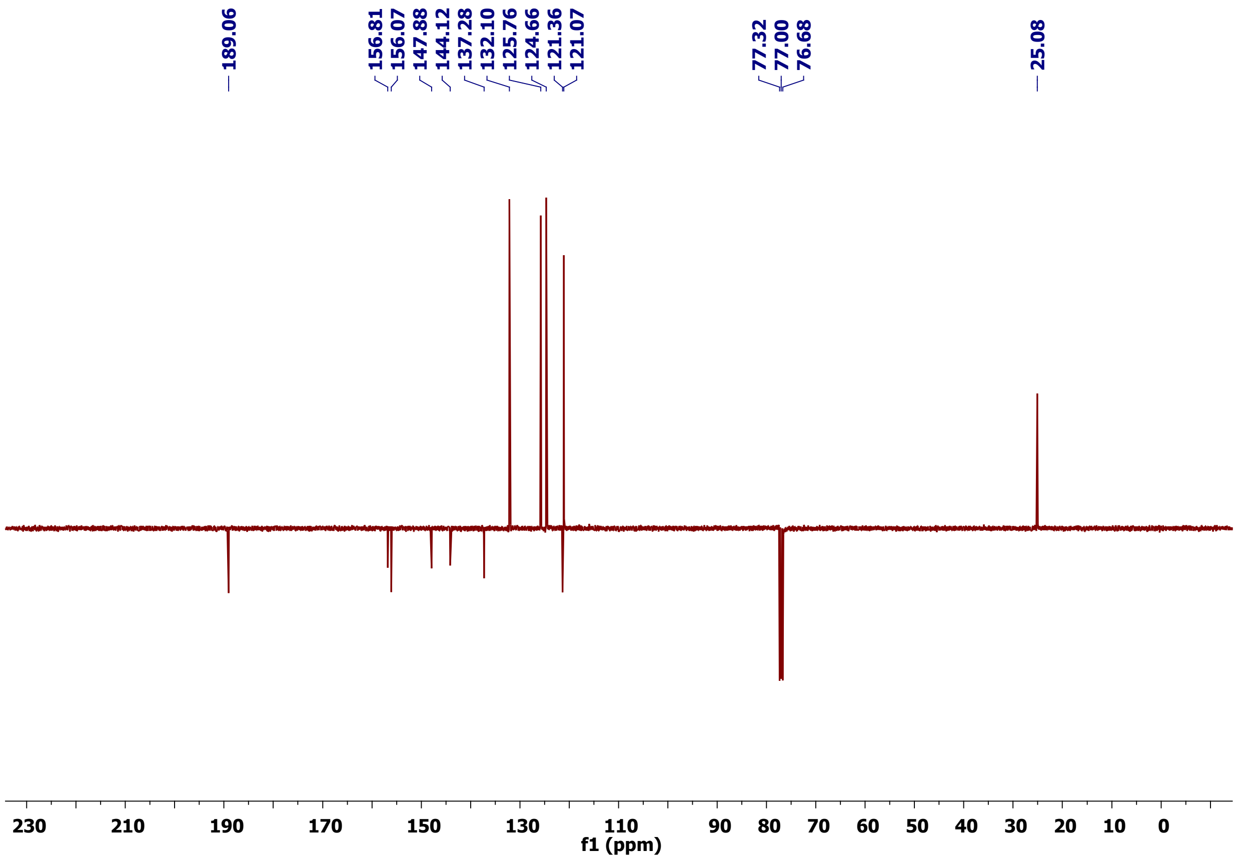

^{1}H-^{1}H gDQCOSY NMR ($CDCl_3$) spectrum of (Z)-1-(4-(4-bromophenyl)-5-((4-nitrophenyl)imino)-4,5-dihydro-1,3,4-thiadiazol-2-yl)ethan-1-one

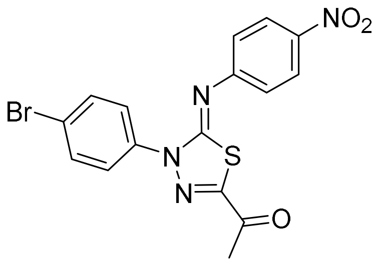

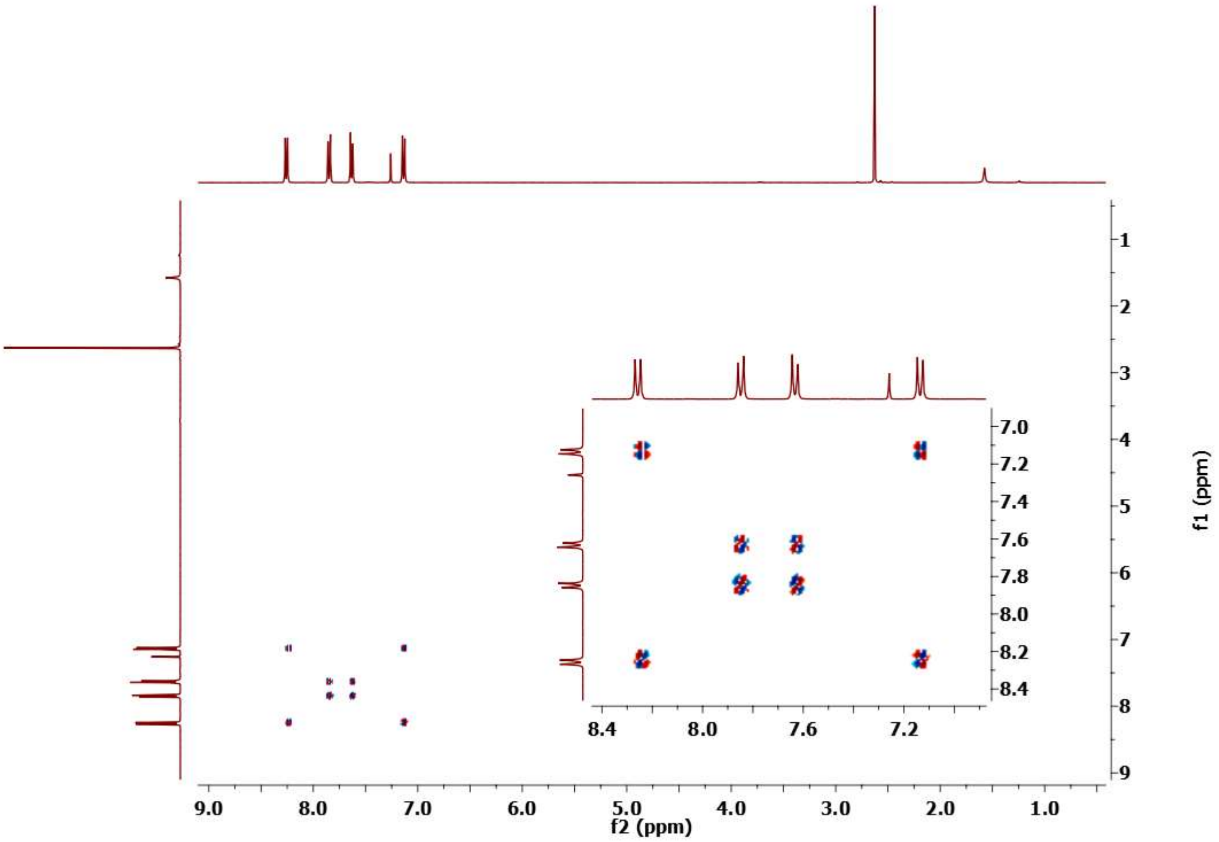

^{1}H-^{13}C-HSQC NMR ($CDCl_3$) spectrum of (Z)-1-(4-(4-bromophenyl)-5-((4-nitrophenyl)imino)-4,5-dihydro-1,3,4-thiadiazol-2-yl)ethan-1-one

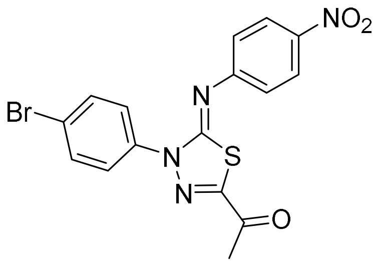

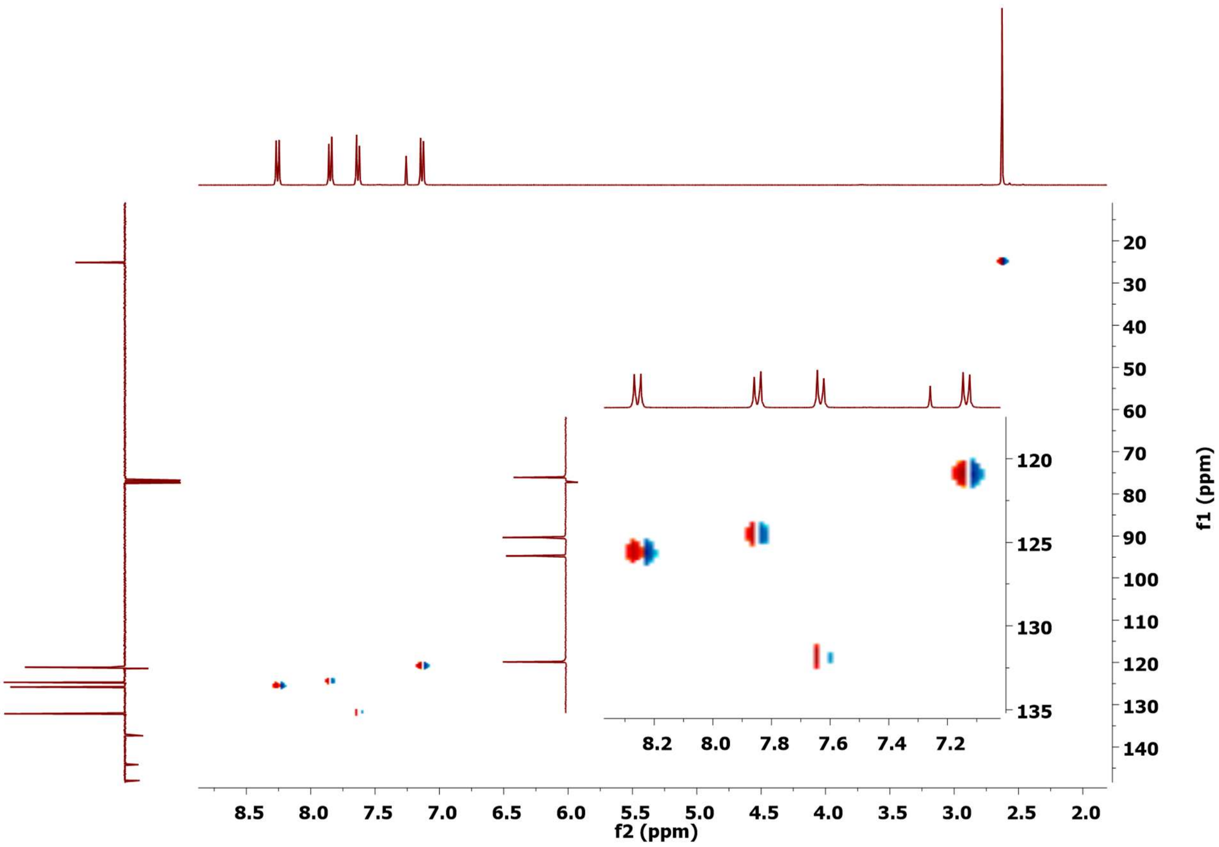

^{1}H-^{13}C-gHMBC NMR ($CDCl_3$) spectrum of (Z)-1-(4-(4-bromophenyl)-5-((4-nitrophenyl)imino)-4,5-dihydro-1,3,4-thiadiazol-2-yl)ethan-1-one

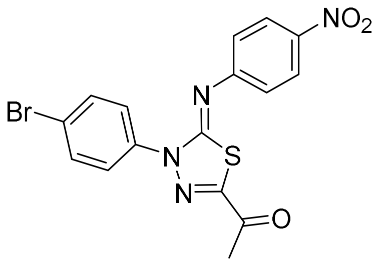

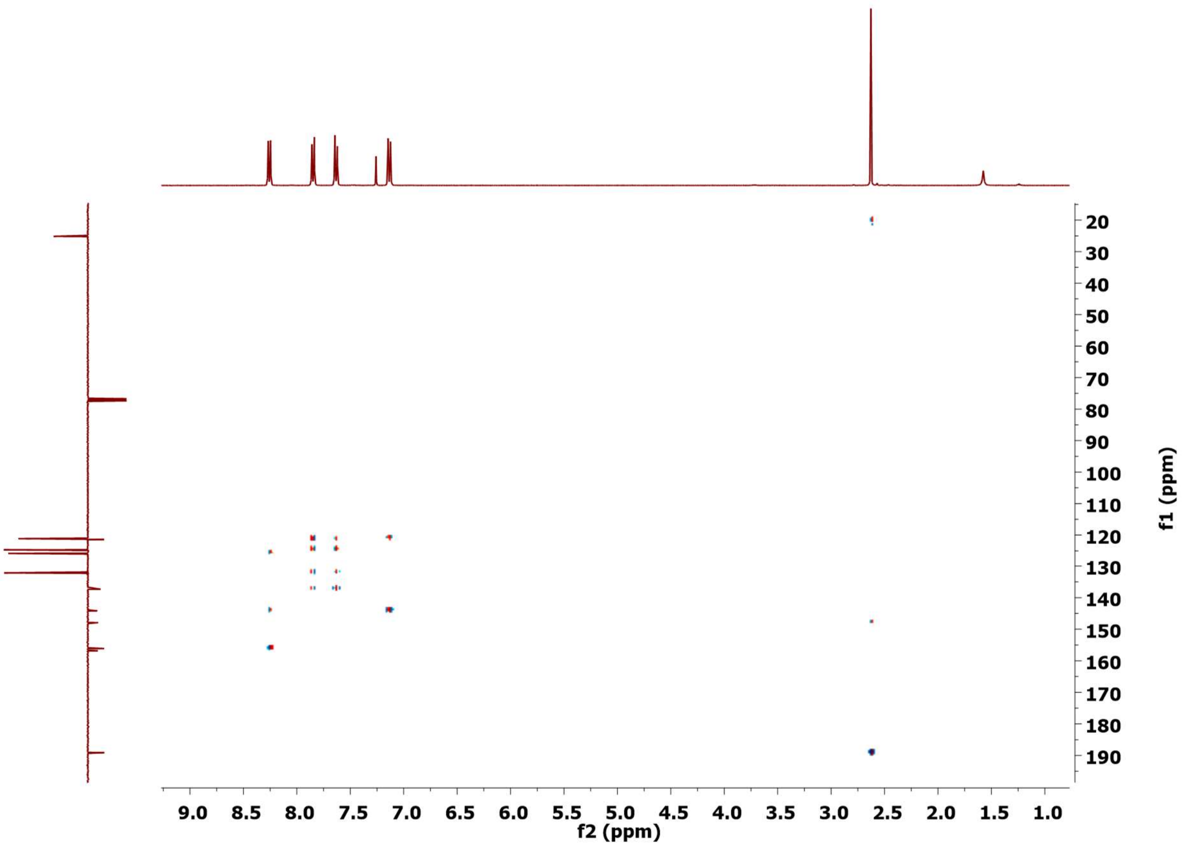

^{1}H NMR ($CDCl_3$) spectrum of (Z)-1-(4-(4-iodophenyl)-5-((4-nitrophenyl)imino)-4,5-dihydro-1,3,4-thiadiazol-2-yl)ethan-1-one

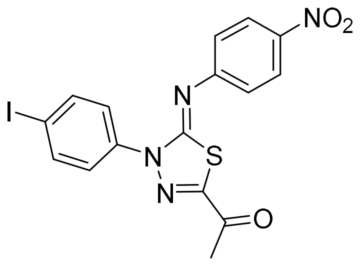

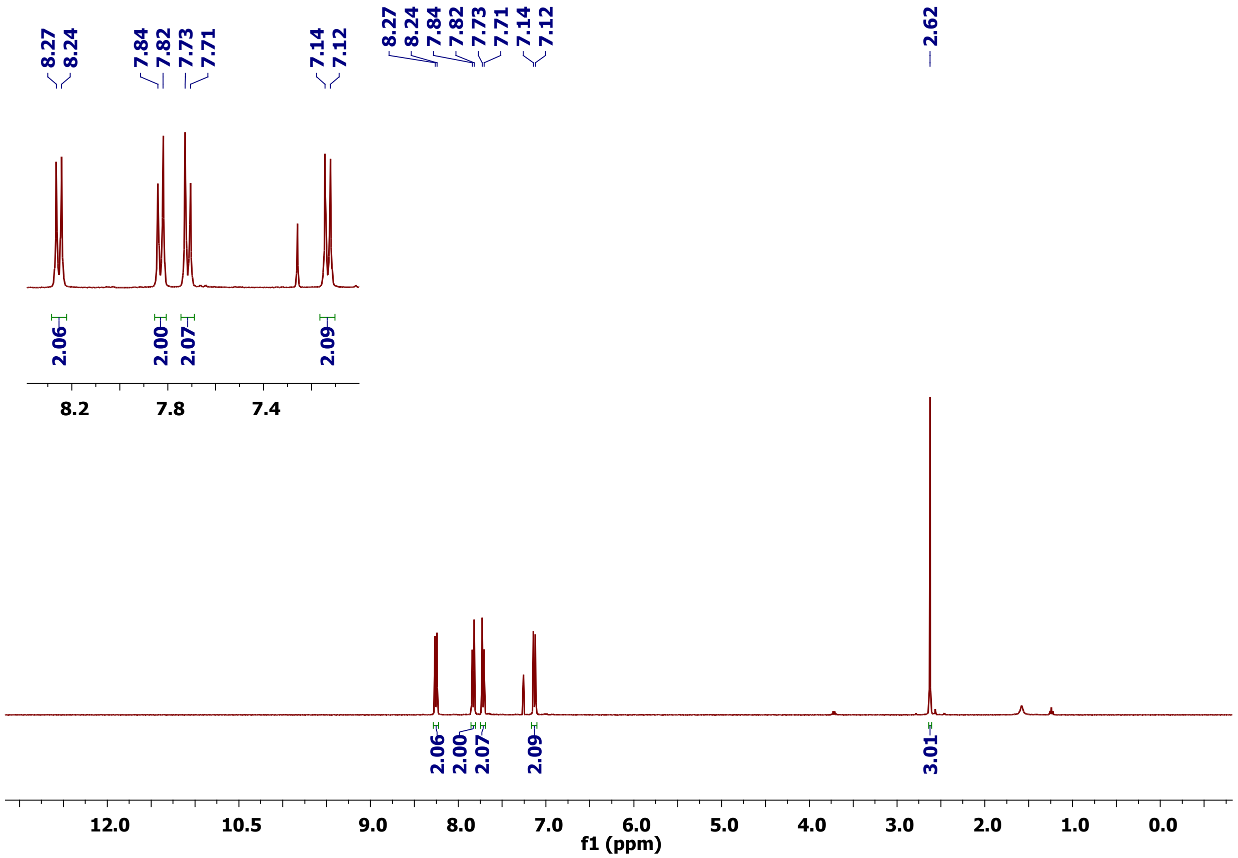

^{13}C NMR ($CDCl_3$) spectrum of (Z)-1-(4-(4-iodophenyl)-5-((4-nitrophenyl)imino)-4,5-dihydro-1,3,4-thiadiazol-2-yl)ethan-1-one

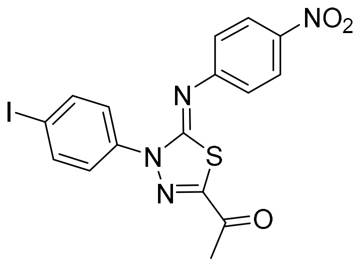

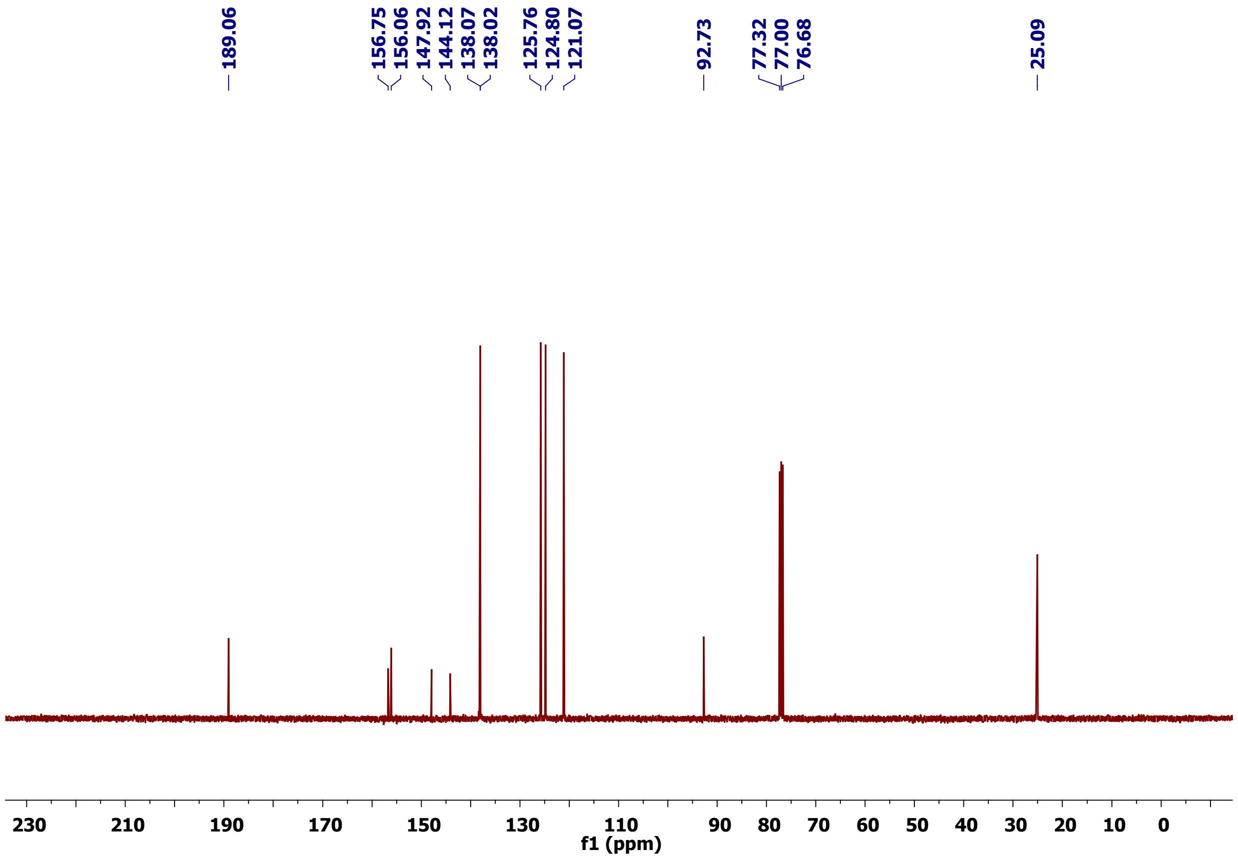

^{13}C CRAPT NMR ($CDCl_3$) spectrum of (Z)-1-(4-(4-iodophenyl)-5-((4-nitrophenyl)imino)-4,5-dihydro-1,3,4-thiadiazol-2-yl)ethan-1-one

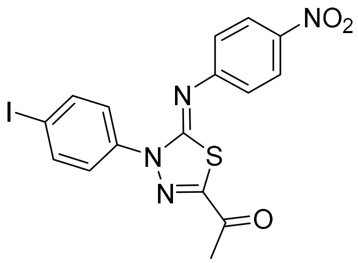

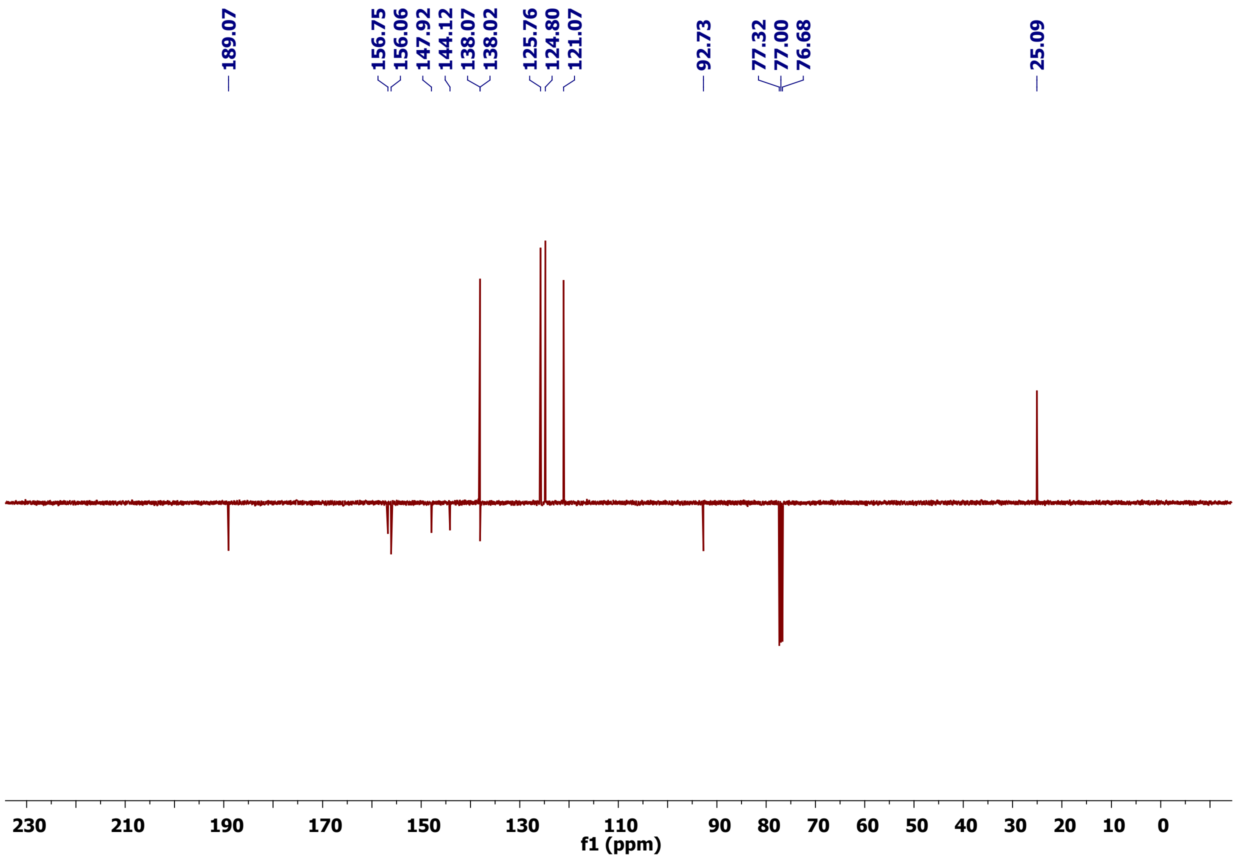

^{1}H-^{1}H gDQCOSY NMR ($CDCl_3$) spectrum of (Z)-1-(4-(4-iodophenyl)-5-((4-nitrophenyl)imino)-4,5-dihydro-1,3,4-thiadiazol-2-yl)ethan-1-one

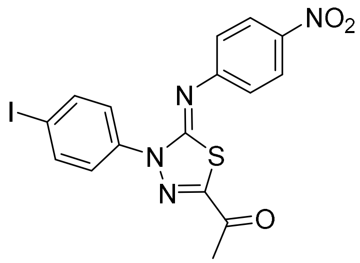

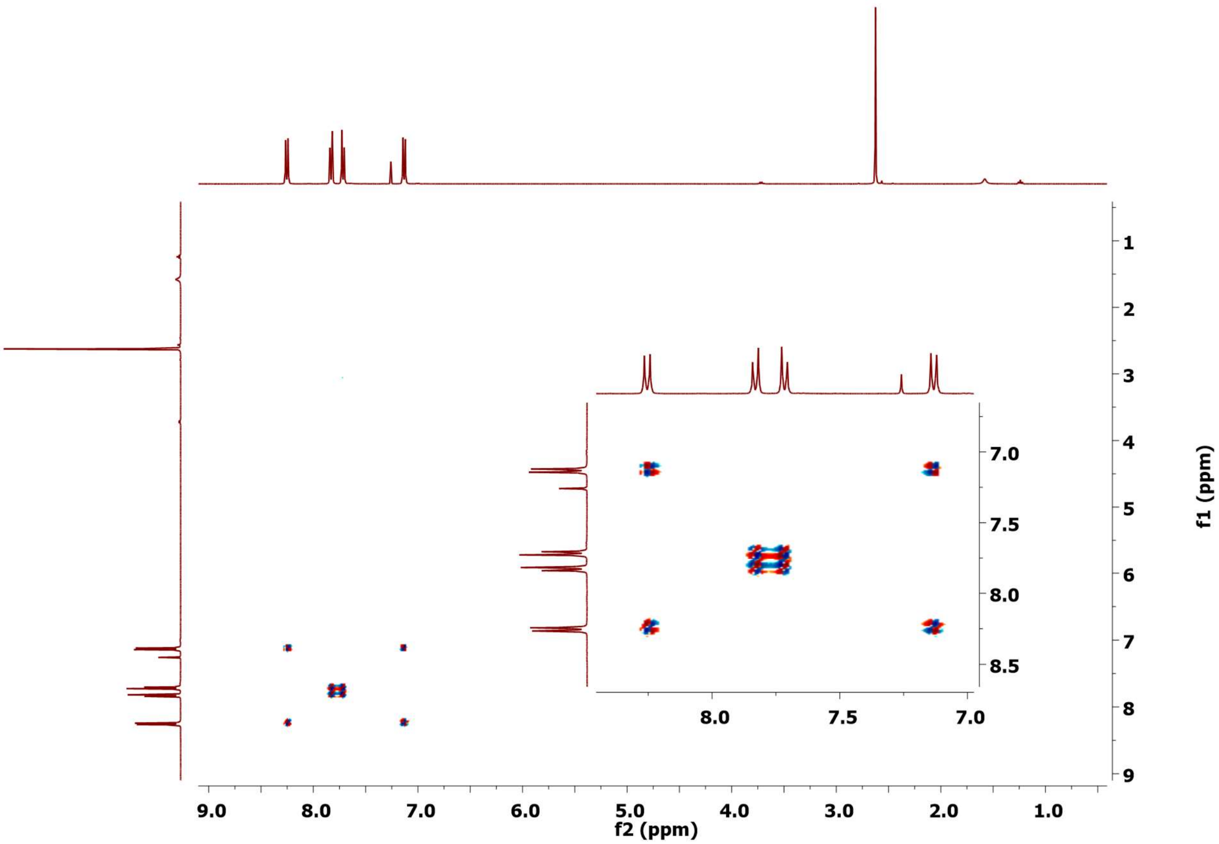

^{1}H-^{13}C-HSQC NMR ($CDCl_3$) spectrum of (Z)-1-(4-(4-iodophenyl)-5-((4-nitrophenyl)imino)-4,5-dihydro-1,3,4-thiadiazol-2-yl)ethan-1-one

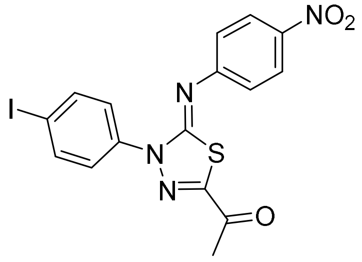

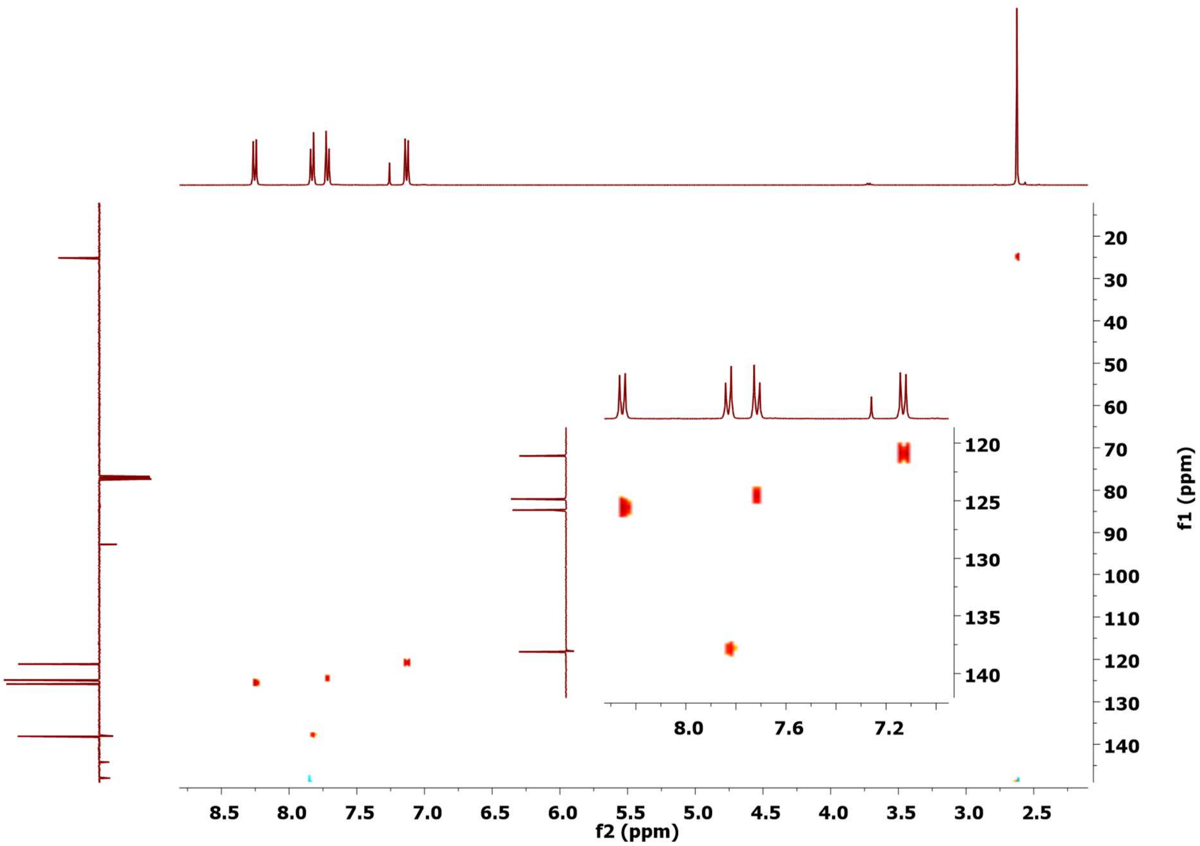

^{1}H-^{13}C-gHMBC NMR ($CDCl_3$) spectrum of (Z)-1-(4-(4-iodophenyl)-5-((4-nitrophenyl)imino)-4,5-dihydro-1,3,4-thiadiazol-2-yl)ethan-1-one

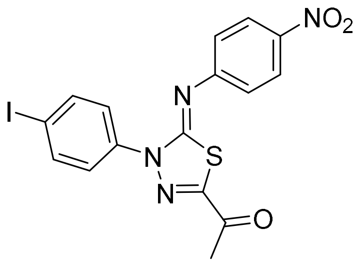

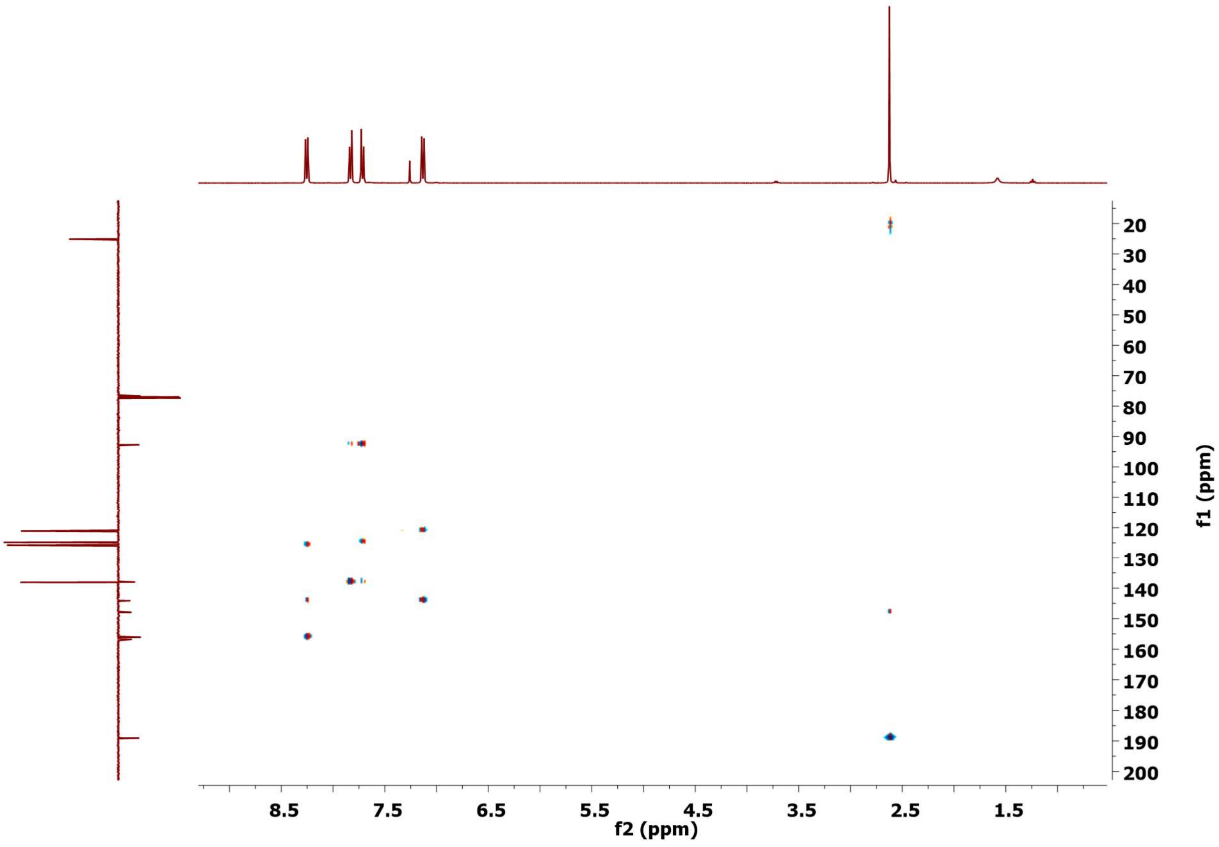

^{1}H NMR ($CDCl_3$) spectrum of (Z)-1-(5-((4-nitrophenyl)imino)-4-(p-tolyl)-4,5-dihydro-1,3,4-thiadiazol-2-yl)ethan-1-one

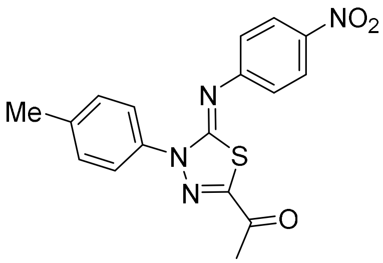

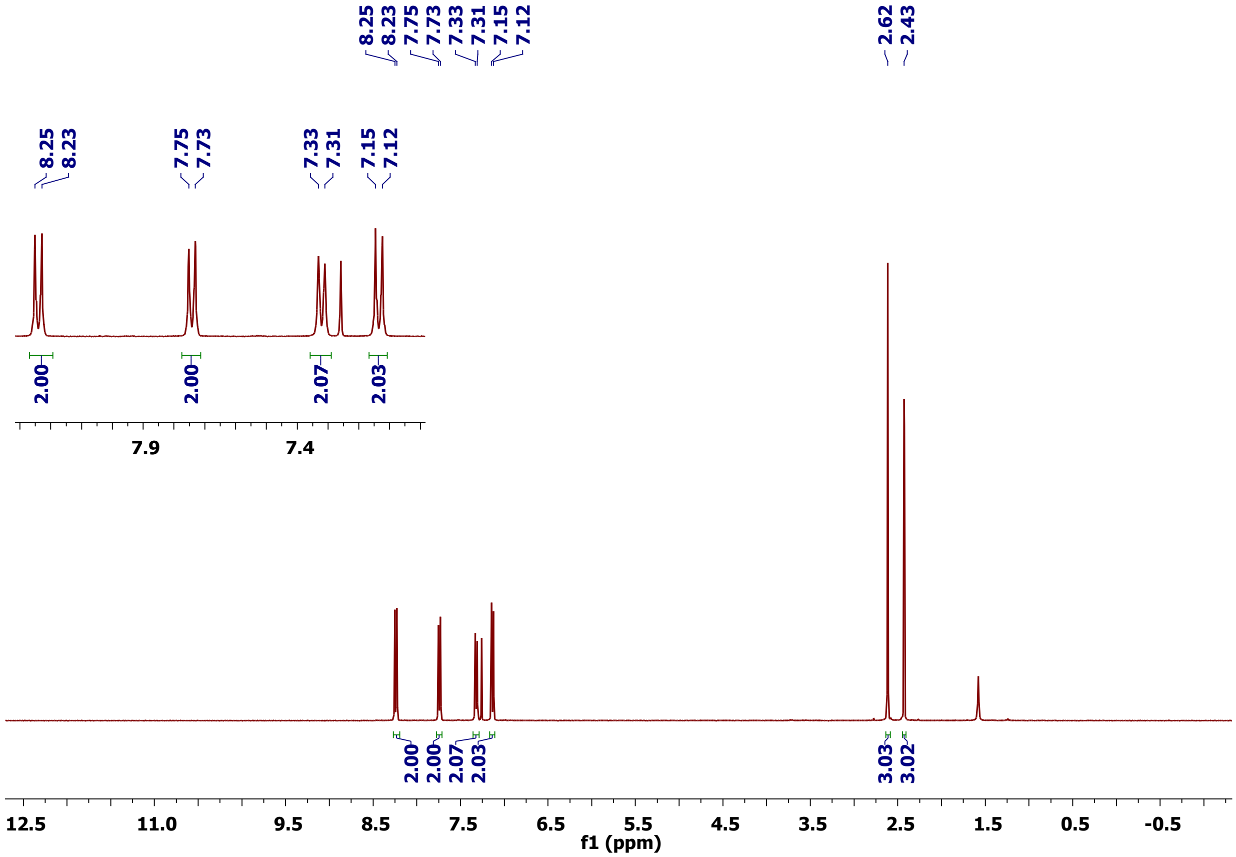

^{13}C NMR ($CDCl_3$) spectrum of (Z)-1-(5-((4-nitrophenyl)imino)-4-(p-tolyl)-4,5-dihydro-1,3,4-thiadiazol-2-yl)ethan-1-one

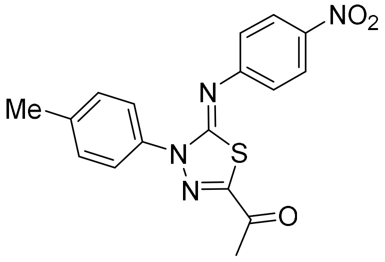

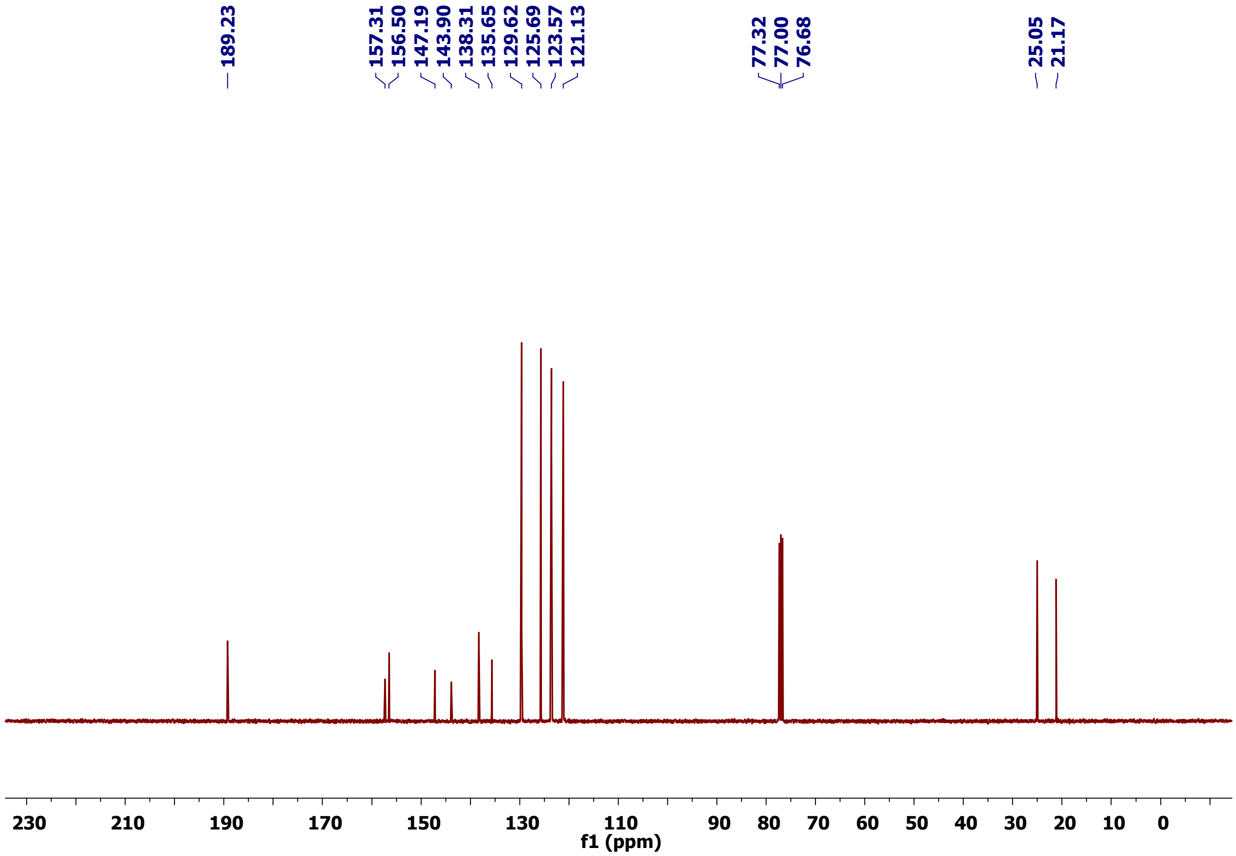

^{13}C CRAPT NMR ($CDCl_3$) spectrum of (Z)-1-(5-((4-nitrophenyl)imino)-4-(p-tolyl)-4,5-dihydro-1,3,4-thiadiazol-2-yl)ethan-1-one

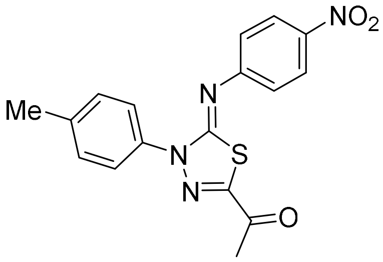

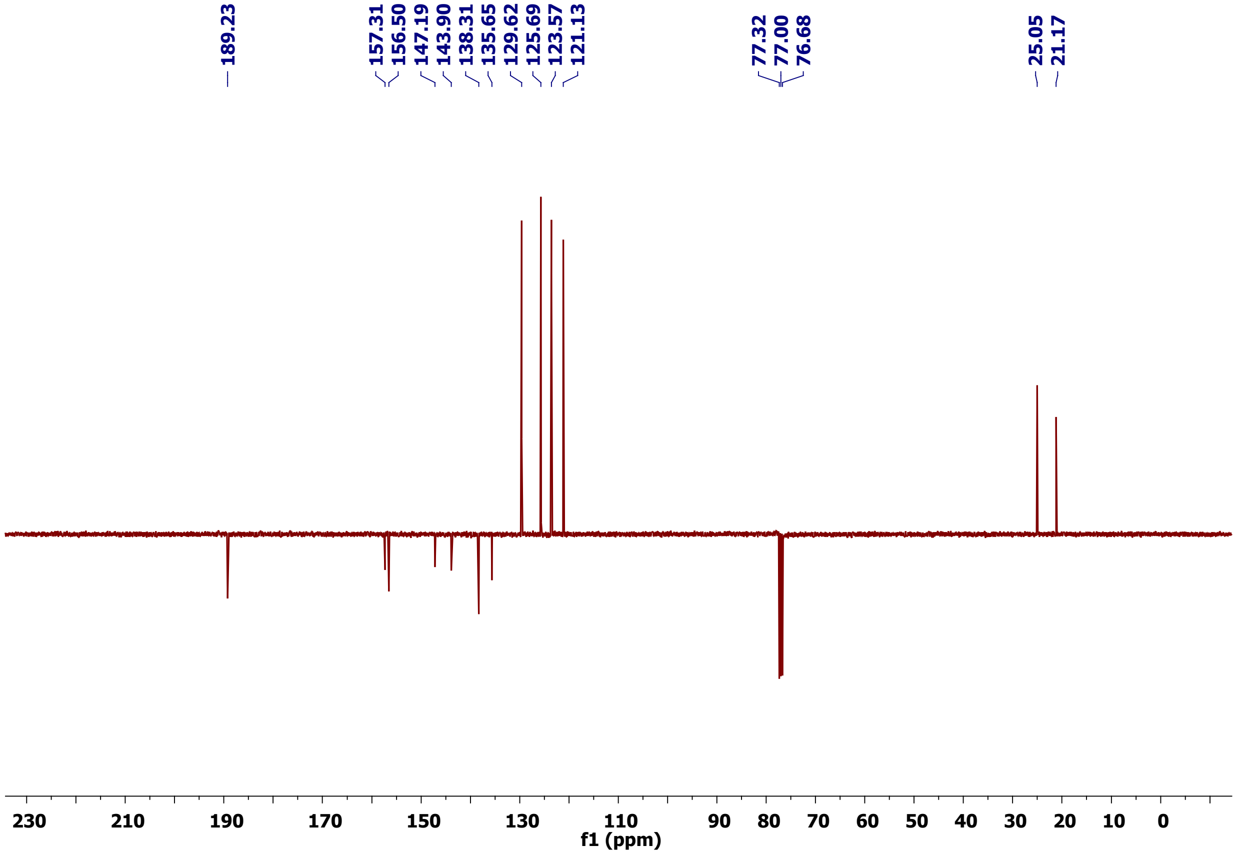

^{1}H-^{1}H gDQCOSY NMR ($CDCl_3$) spectrum of (Z)-1-(5-((4-nitrophenyl)imino)-4-(p-tolyl)-4,5-dihydro-1,3,4-thiadiazol-2-yl)ethan-1-one

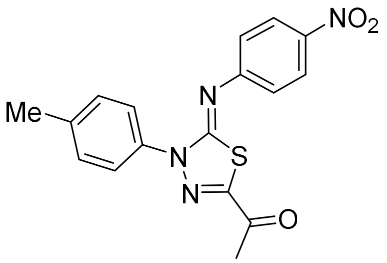

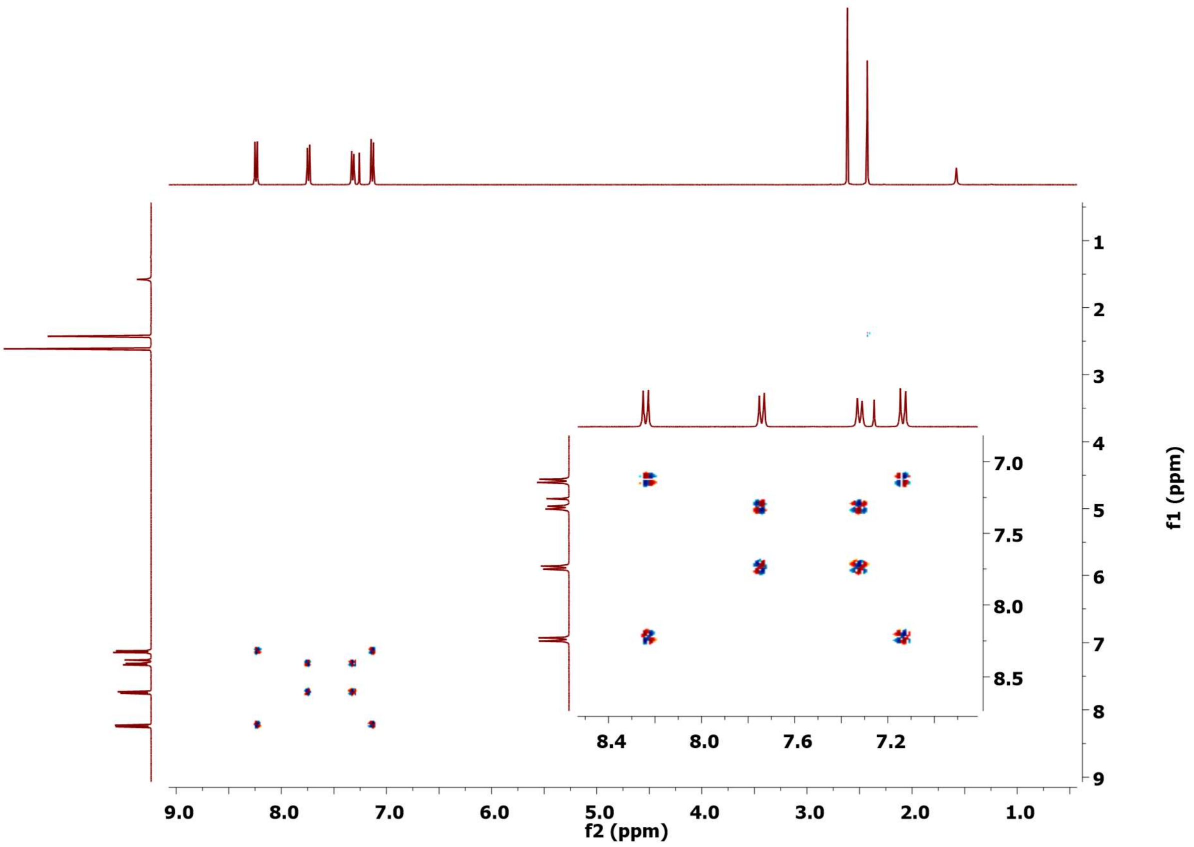

^{1}H-^{13}C-HSQC NMR ($CDCl_3$) spectrum of (Z)-1-(5-((4-nitrophenyl)imino)-4-(p-tolyl)-4,5-dihydro-1,3,4-thiadiazol-2-yl)ethan-1-one

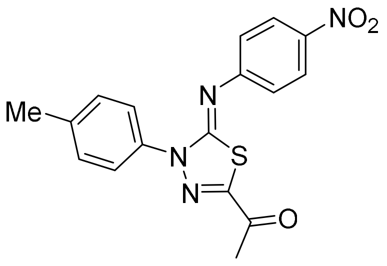

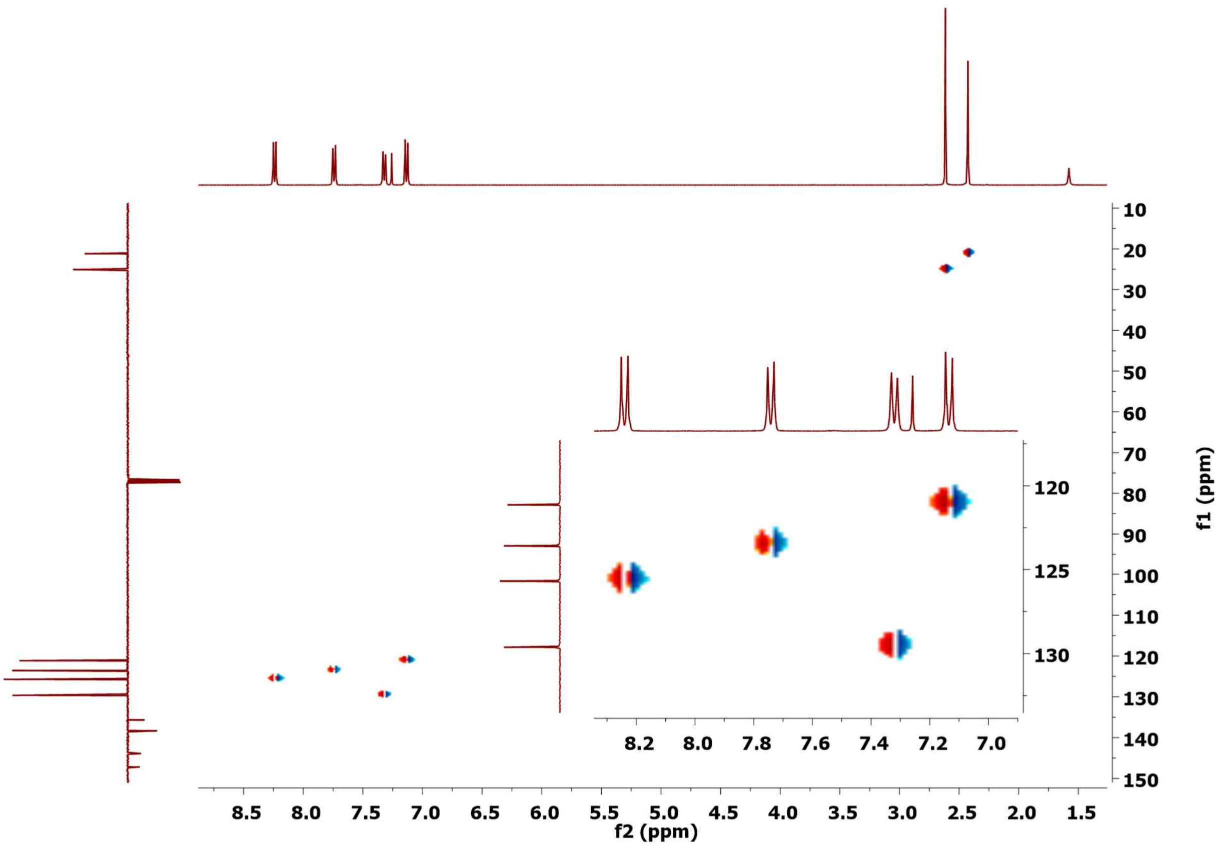

^{1}H-^{13}C-gHMBC NMR ($CDCl_3$) spectrum of (Z)-1-(5-((4-nitrophenyl)imino)-4-(p-tolyl)-4,5-dihydro-1,3,4-thiadiazol-2-yl)ethan-1-one

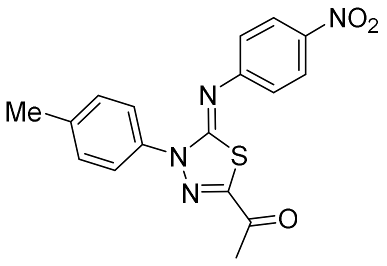

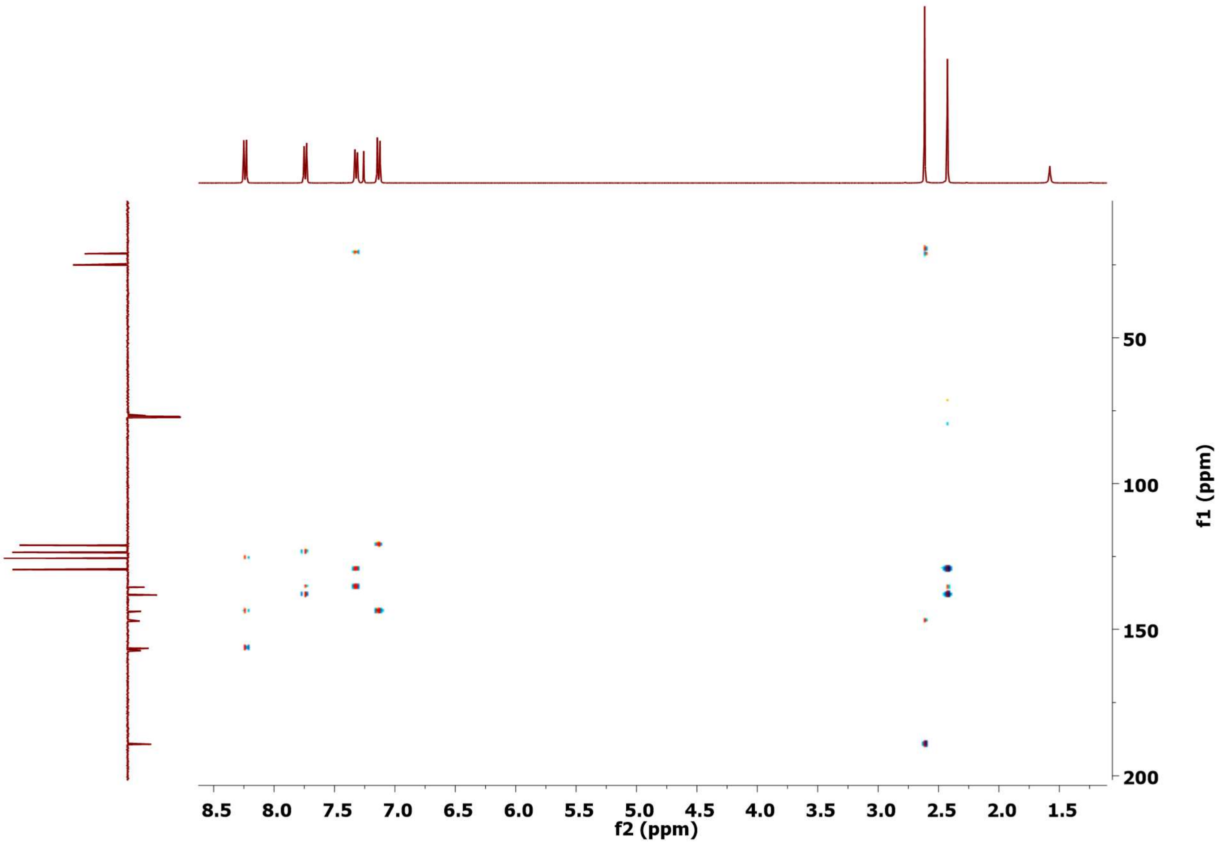

^{1}H NMR ($CDCl_3$) spectrum of (Z)-1-(5-((4-nitrophenyl)imino)-4-phenyl-4,5-dihydro-1,3,4-thiadiazol-2-yl)ethan-1-one

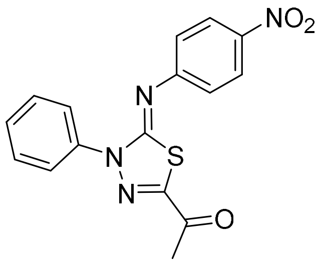

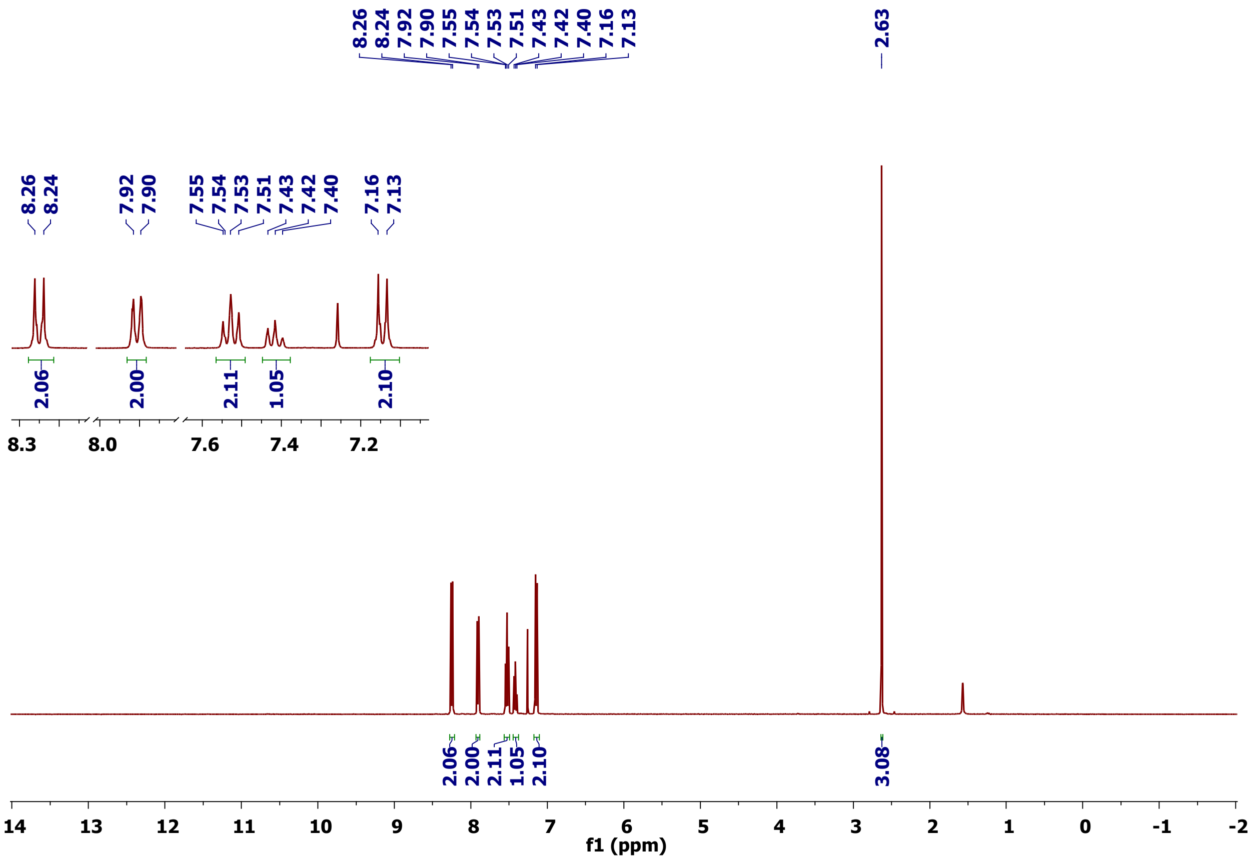

^{13}C NMR ($CDCl_3$) spectrum of (Z)-1-(5-((4-nitrophenyl)imino)-4-phenyl-4,5-dihydro-1,3,4-thiadiazol-2-yl)ethan-1-one

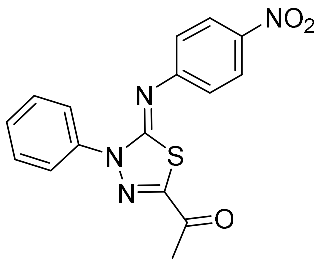

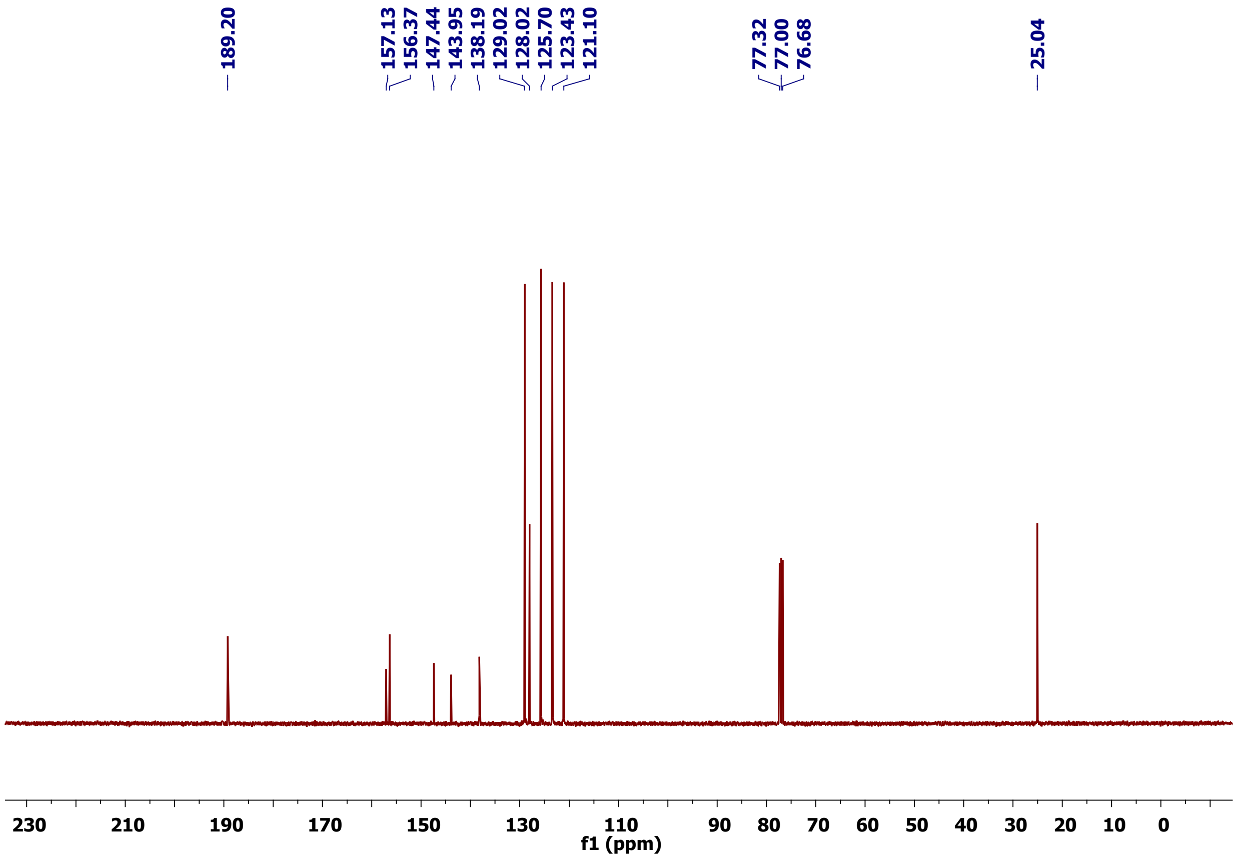

^{13}C CRAPT NMR ($CDCl_3$) spectrum of (Z)-1-(5-((4-nitrophenyl)imino)-4-phenyl-4,5-dihydro-1,3,4-thiadiazol-2-yl)ethan-1-one

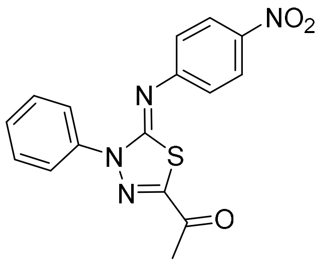

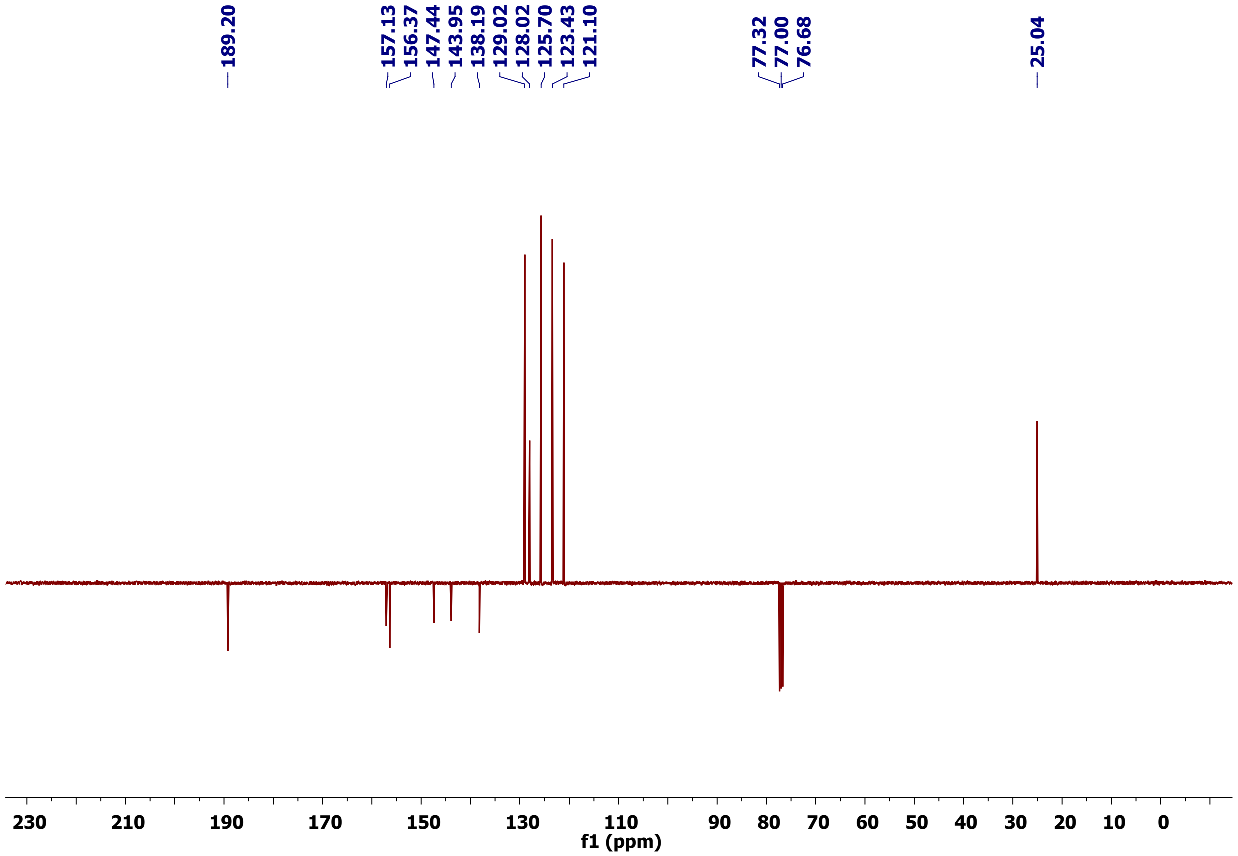

^{1}H-^{1}H gDQCOSY NMR ($CDCl_3$) spectrum of (Z)-1-(5-((4-nitrophenyl)imino)-4-phenyl-4,5-dihydro-1,3,4-thiadiazol-2-yl)ethan-1-one

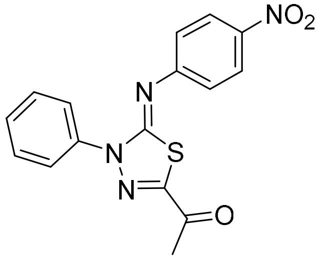

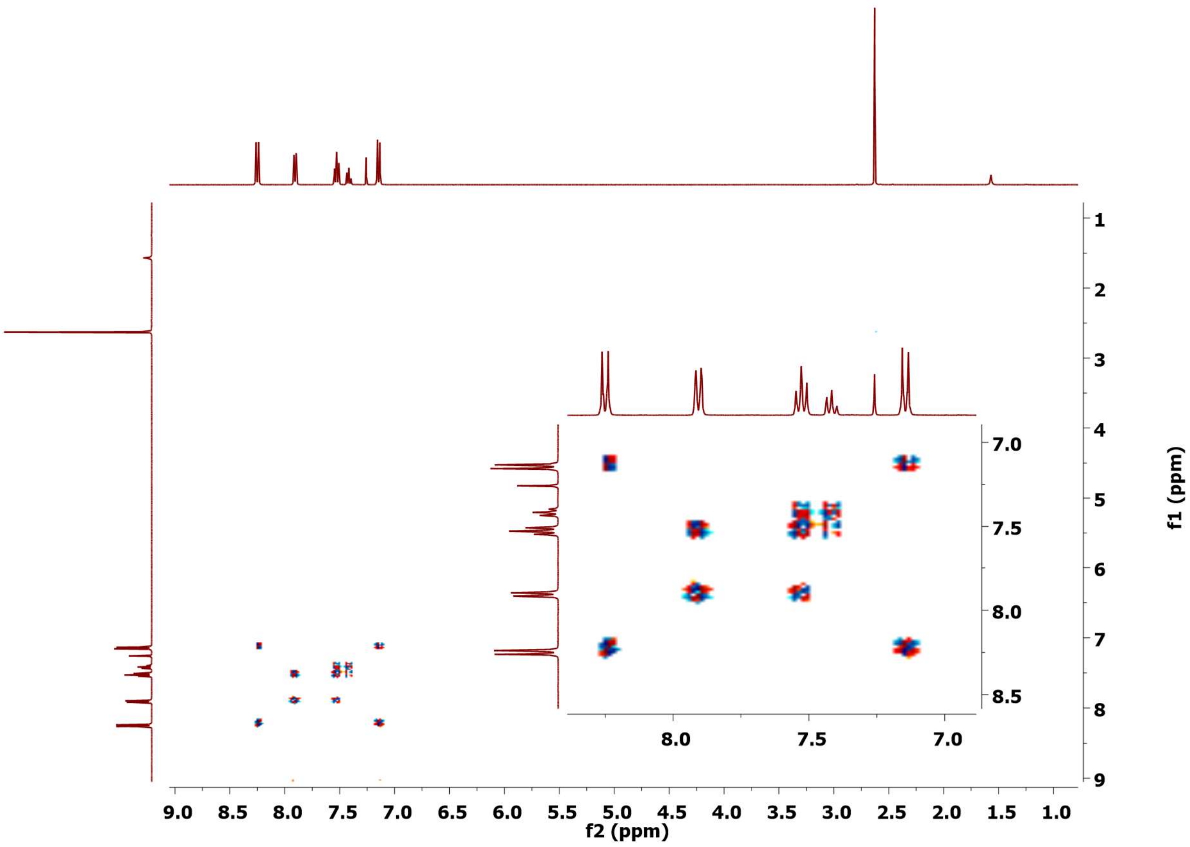

^{1}H-^{13}C-HSQC NMR ($CDCl_3$) spectrum of (Z)-1-(5-((4-nitrophenyl)imino)-4-phenyl-4,5-dihydro-1,3,4-thiadiazol-2-yl)ethan-1-one

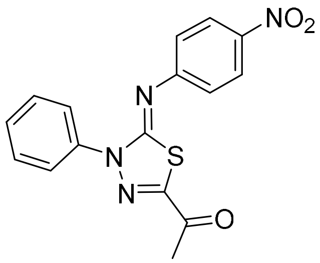

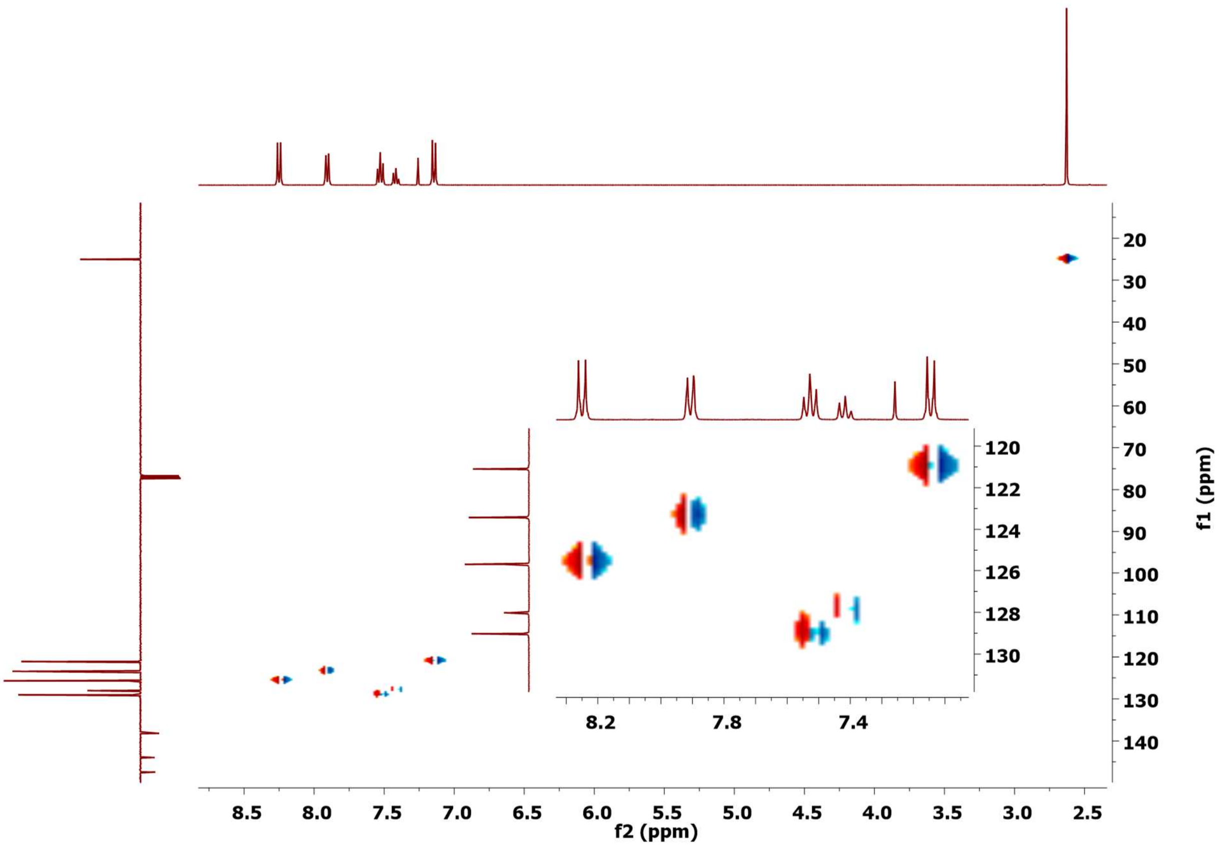

^1H-^{13}C-gHMBC NMR ($CDCl_3$) spectrum of (Z)-1-(5-((4-nitrophenyl)imino)-4-phenyl-4,5-dihydro-1,3,4-thiadiazol-2-yl)ethan-1-one

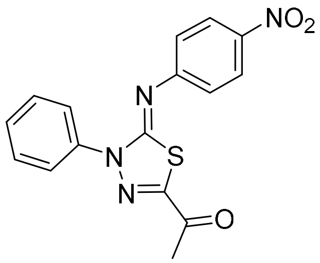

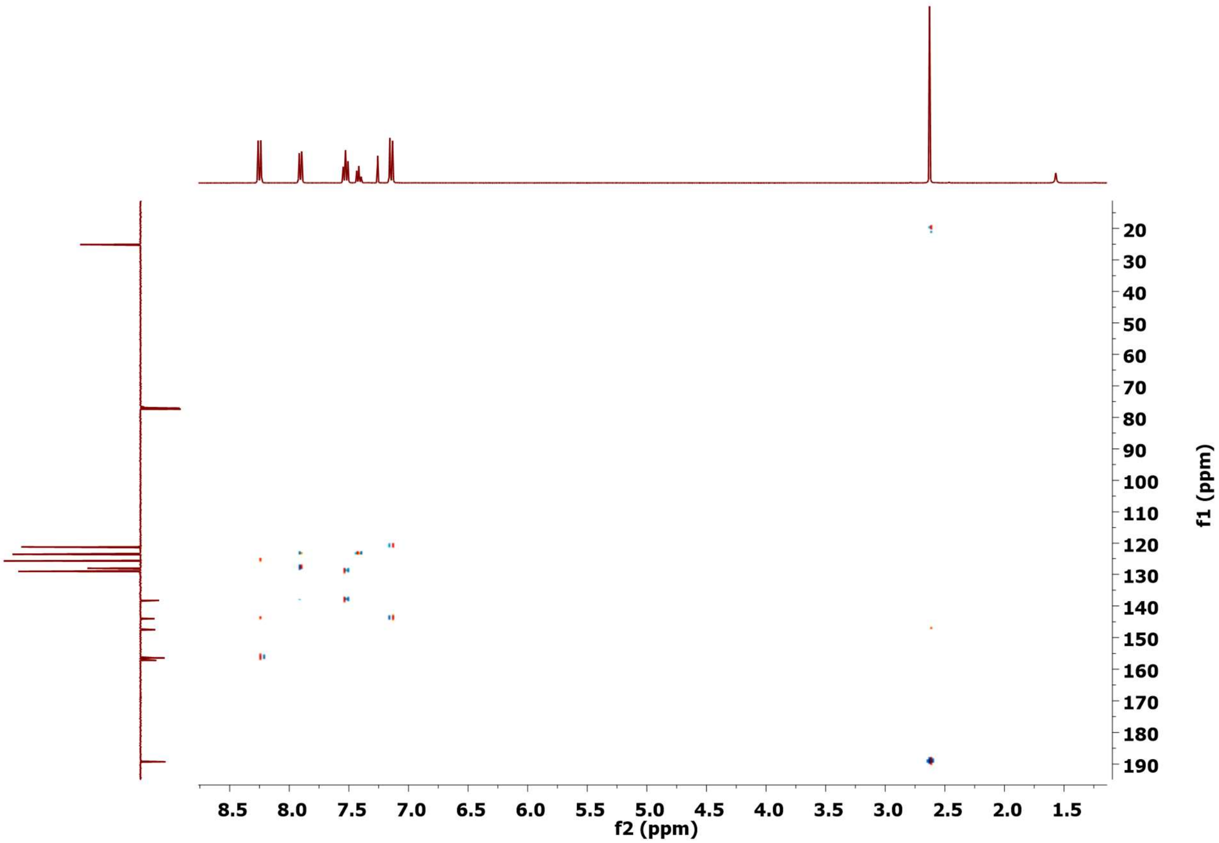

^{1}H NMR ($CDCl_3$) spectrum of (Z)-1-(4-(4-(methylthio)phenyl)-5-((4-nitrophenyl)imino)-4,5-dihydro-1,3,4-thiadiazol-2-yl)ethan-1-one

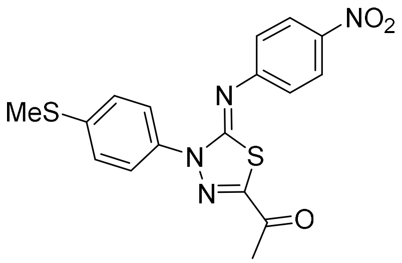

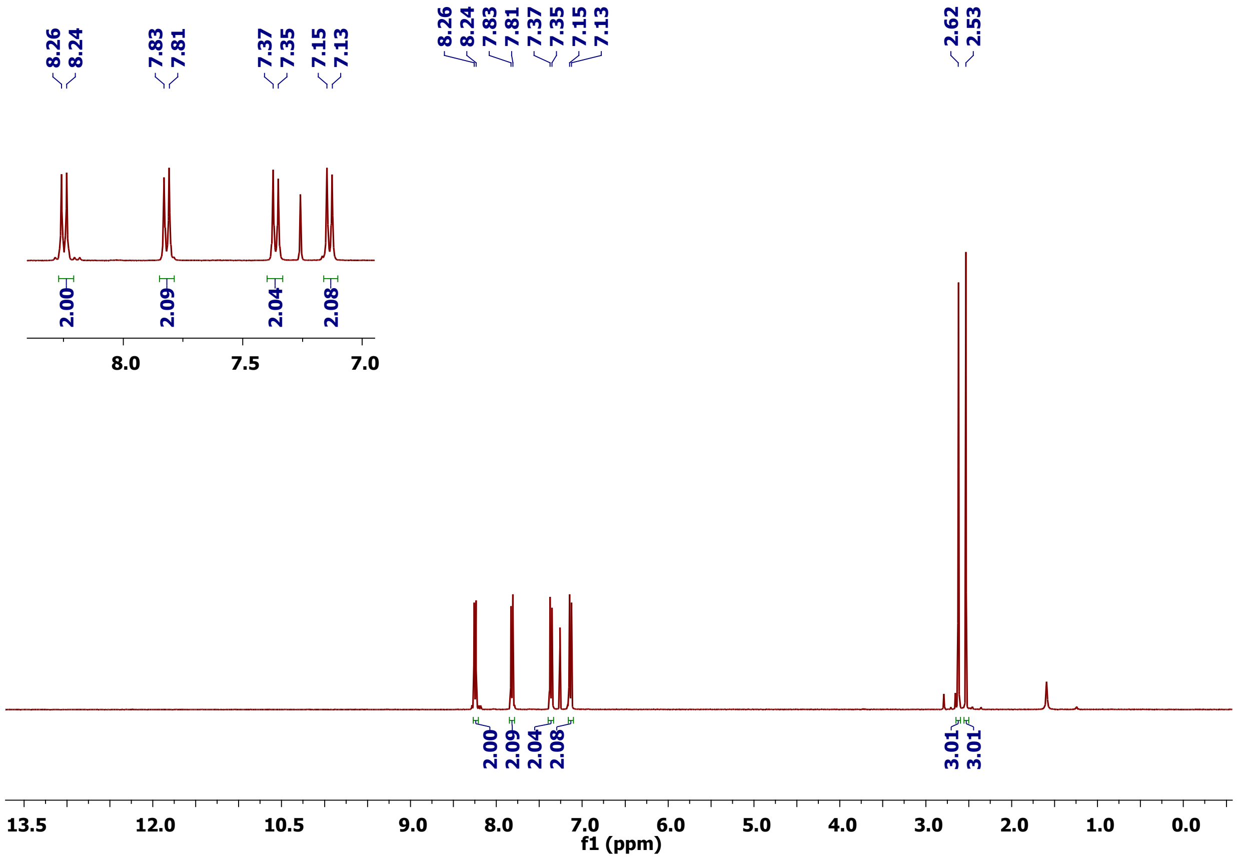

^{13}C NMR ($CDCl_3$) spectrum of (Z)-1-(4-(4-(methylthio)phenyl)-5-((4-nitrophenyl)imino)-4,5-dihydro-1,3,4-thiadiazol-2-yl)ethan-1-one

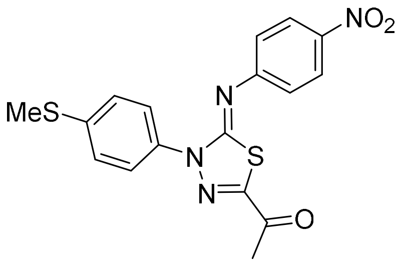

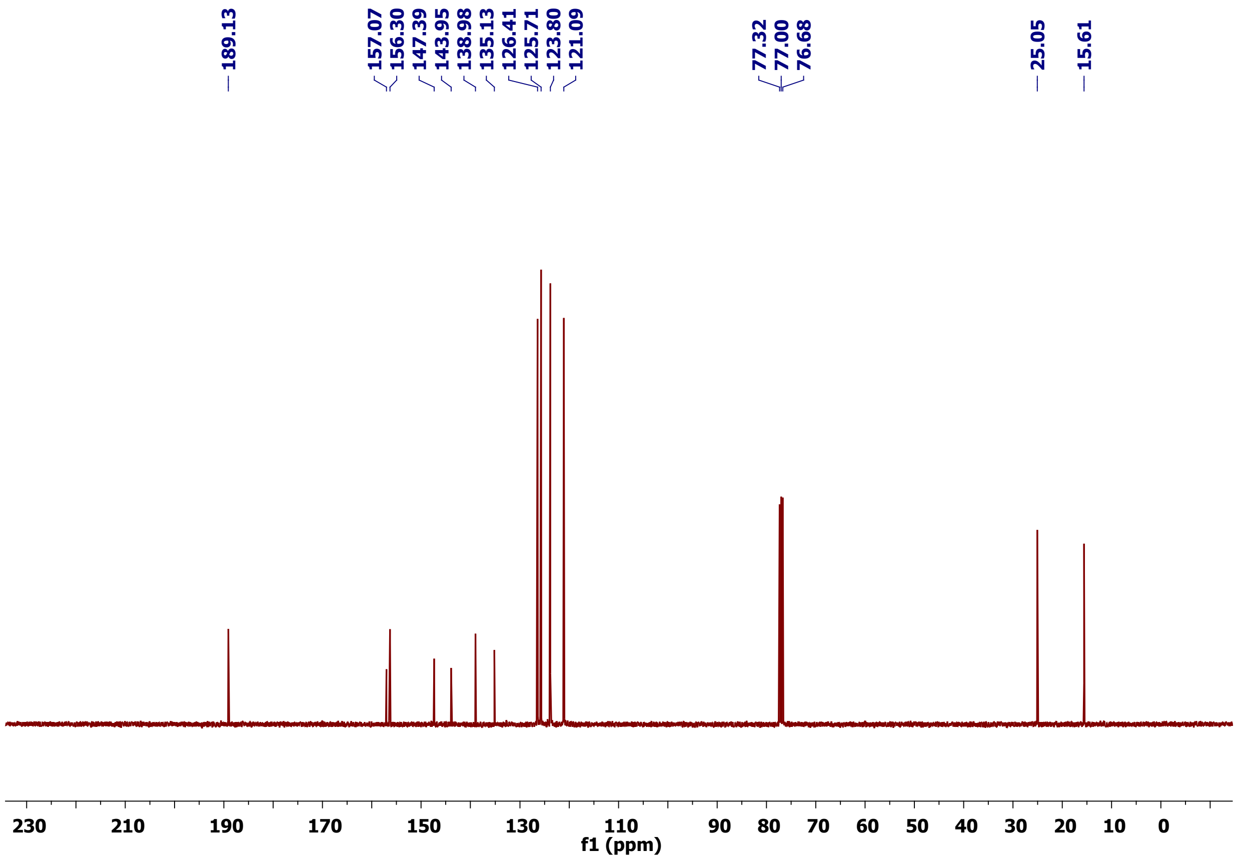

^{13}C CRAPT NMR ($CDCl_3$) spectrum of (Z)-1-(4-(4-(methylthio)phenyl)-5-((4-nitrophenyl)imino)-4,5-dihydro-1,3,4-thiadiazol-2-yl)ethan-1-one

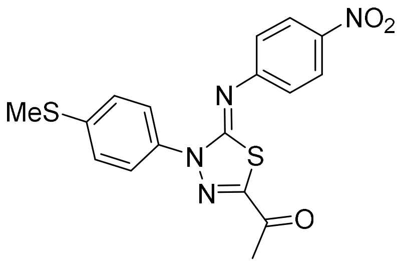

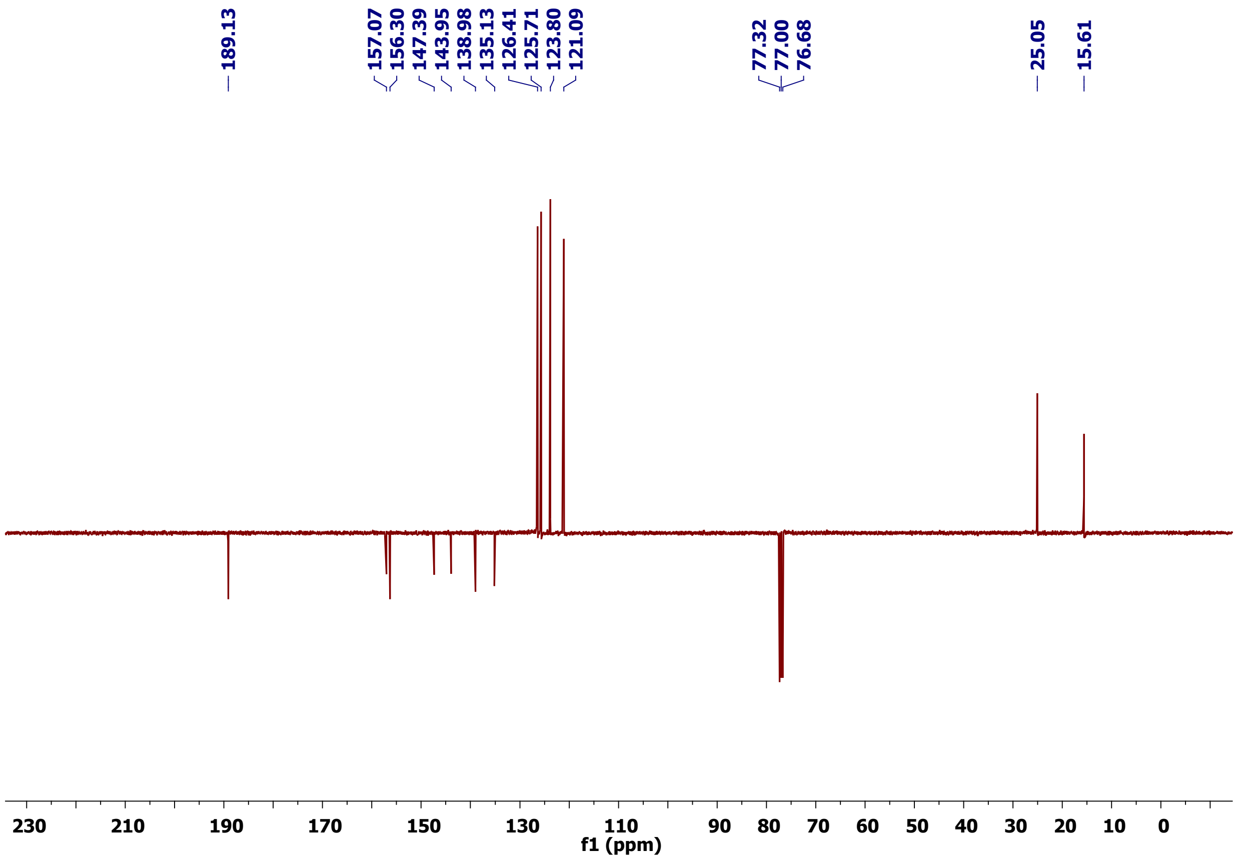

^1H-^1H gDQCOSY NMR ($CDCl_3$) spectrum of (Z)-1-(4-(4-(methylthio)phenyl)-5-((4-nitrophenyl)imino)-4,5-dihydro-1,3,4-thiadiazol-2-yl)ethan-1-one

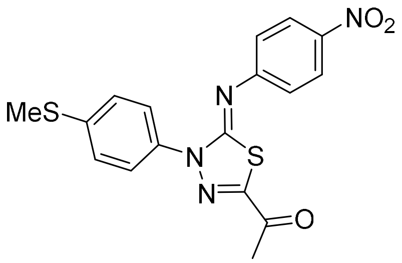

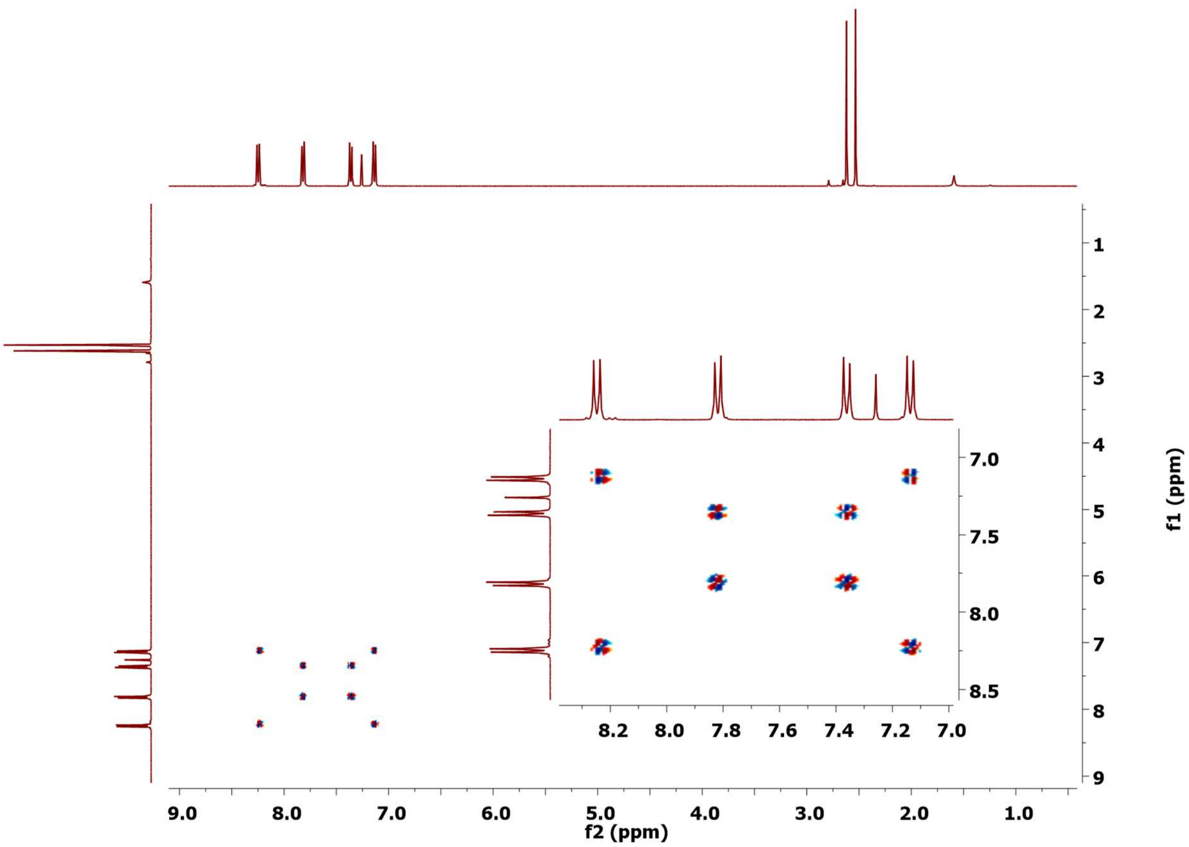

^{1}H-^{13}C-HSQC NMR ($CDCl_3$) spectrum of (Z)-1-(4-(4-(methylthio)phenyl)-5-((4-nitrophenyl)imino)-4,5-dihydro-1,3,4-thiadiazol-2-yl)ethan-1-one

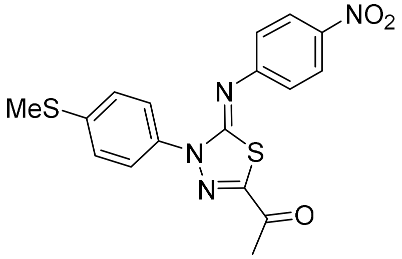

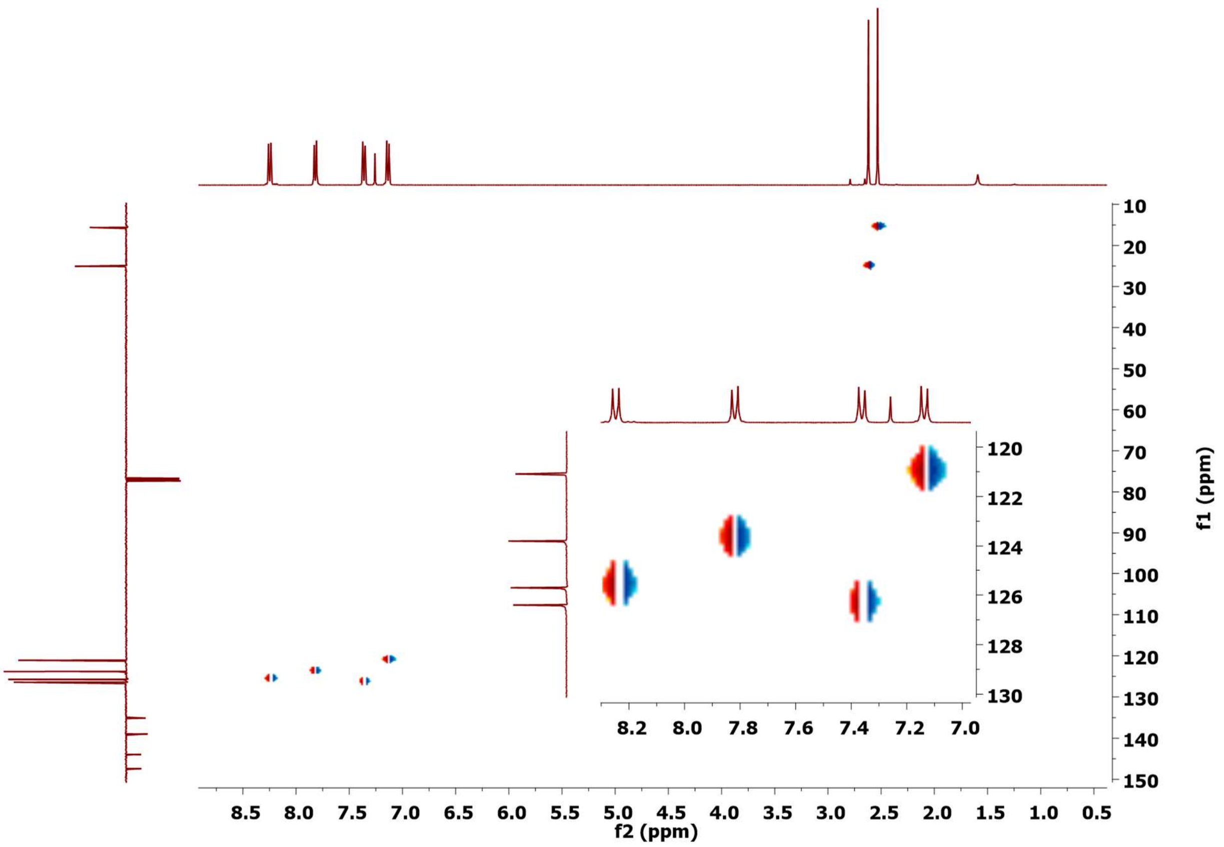

^{1}H-^{13}C-gHMBC NMR ($CDCl_3$) spectrum of (Z)-1-(4-(4-(methylthio)phenyl)-5-((4-nitrophenyl)imino)-4,5-dihydro-1,3,4-thiadiazol-2-yl)ethan-1-one

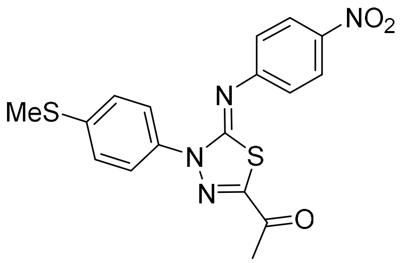

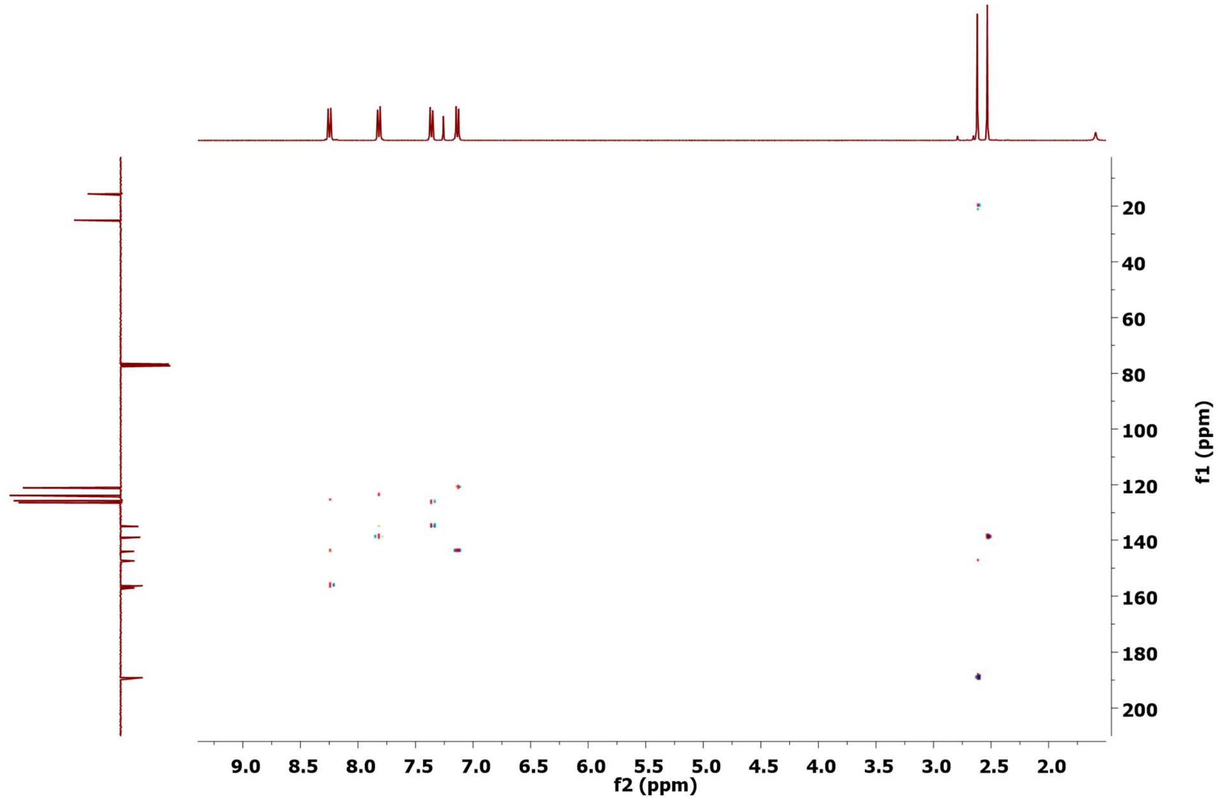

^1H NMR ($CDCl_3$) spectrum of (Z)-1-(4-(4-methoxyphenyl)-5-((4-nitrophenyl)imino)-4,5-dihydro-1,3,4-thiadiazol-2-yl)ethan-1-one

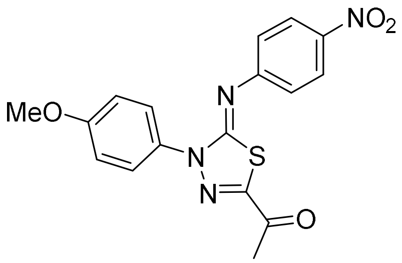

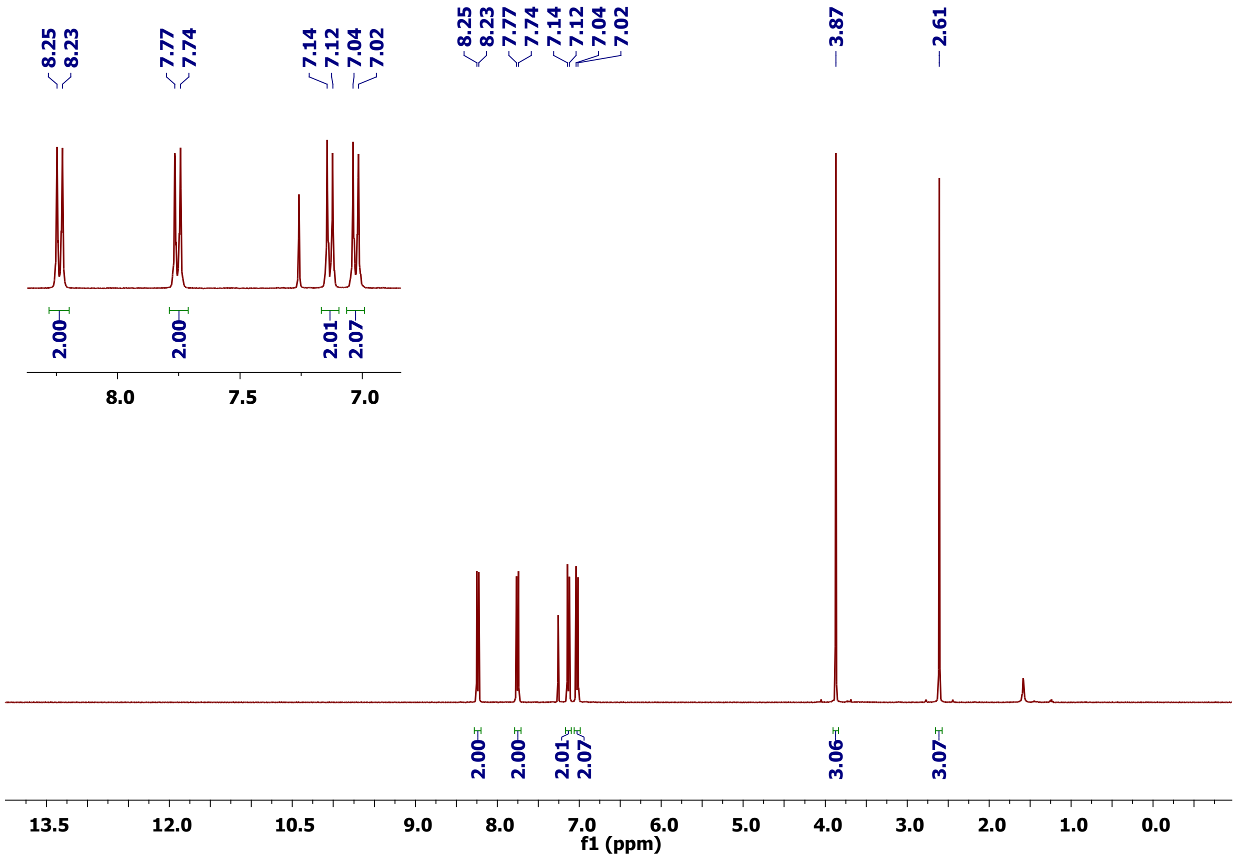

^{13}C NMR ($CDCl_3$) spectrum of (Z)-1-(4-(4-methoxyphenyl)-5-((4-nitrophenyl)imino)-4,5-dihydro-1,3,4-thiadiazol-2-yl)ethan-1-one

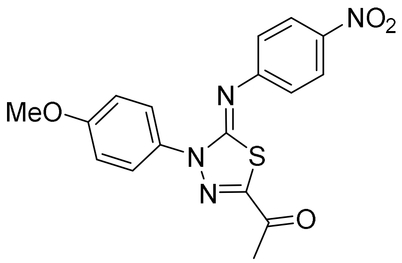

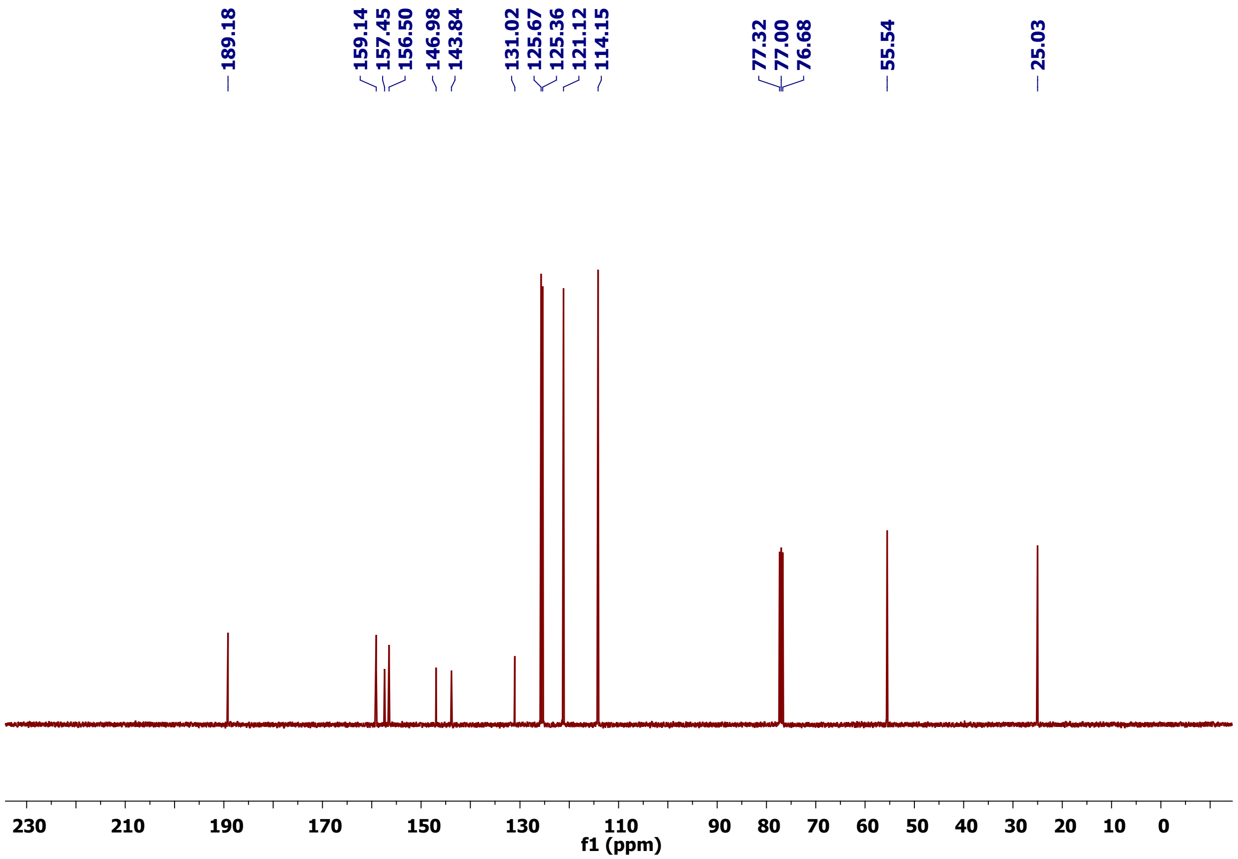

^{13}C CRAPT NMR ($CDCl_3$) spectrum of (Z)-1-(4-(4-methoxyphenyl)-5-((4-nitrophenyl)imino)-4,5-dihydro-1,3,4-thiadiazol-2-yl)ethan-1-one

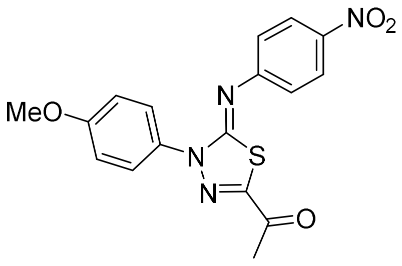

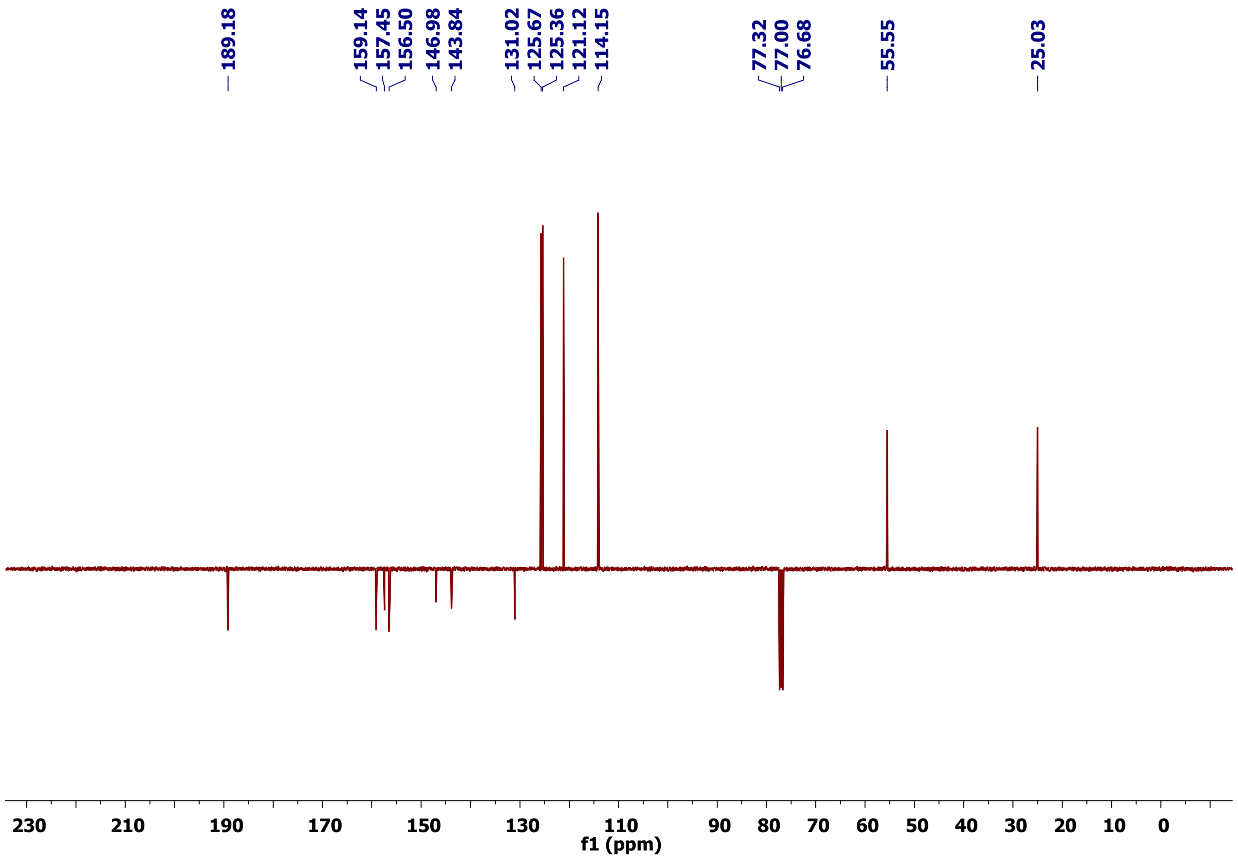

^{1}H-^{1}H gDQCOSY NMR ($CDCl_3$) spectrum of (Z)-1-(4-(4-methoxyphenyl)-5-((4-nitrophenyl)imino)-4,5-dihydro-1,3,4-thiadiazol-2-yl)ethan-1-one

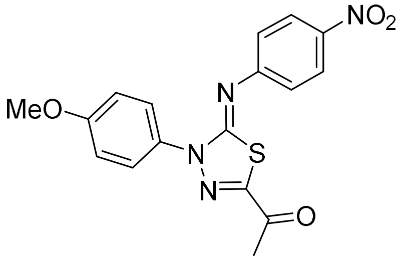

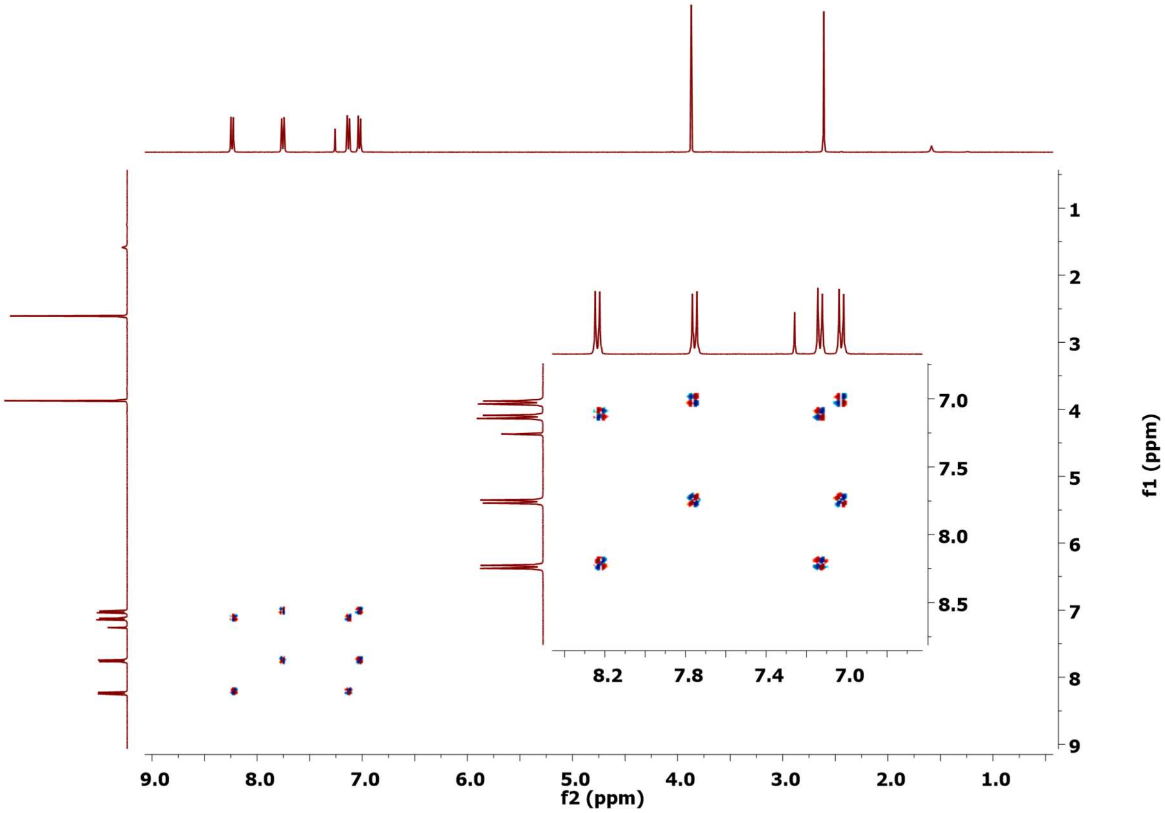

^{1}H-^{13}C-HSQC NMR ($CDCl_3$) spectrum of (Z)-1-(4-(4-methoxyphenyl)-5-((4-nitrophenyl)imino)-4,5-dihydro-1,3,4-thiadiazol-2-yl)ethan-1-one

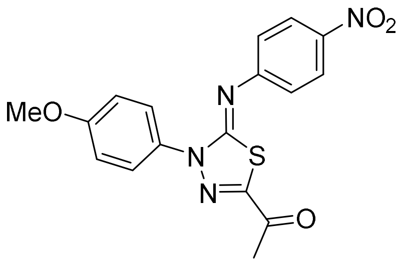

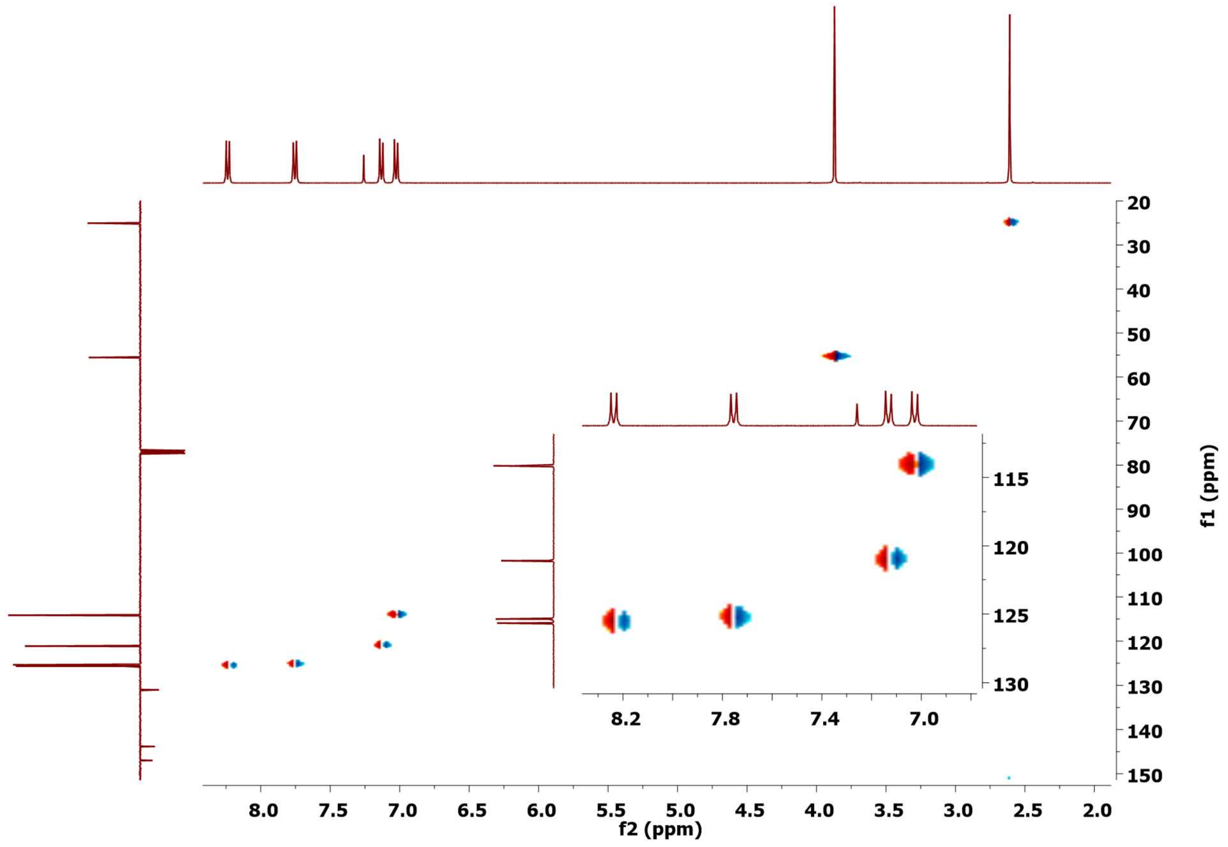

^{1}H-^{13}C-gHMBC NMR ($CDCl_3$) spectrum of (Z)-1-(4-(4-methoxyphenyl)-5-((4-nitrophenyl)imino)-4,5-dihydro-1,3,4-thiadiazol-2-yl)ethan-1-one

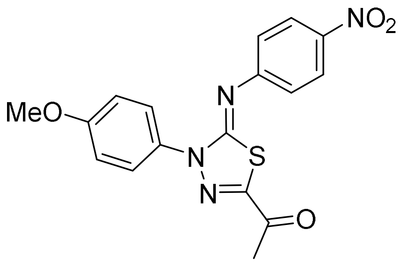

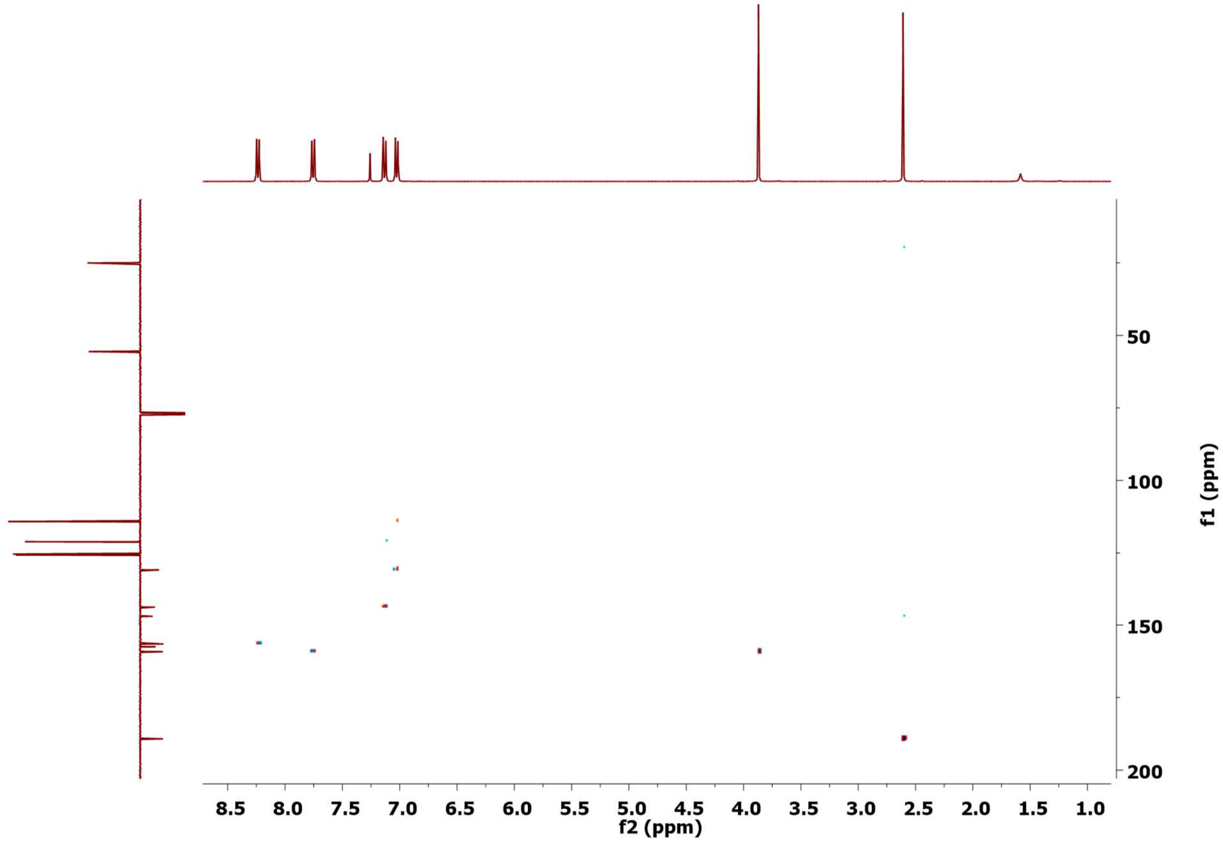

^{1}H NMR ($CDCl_3$) spectrum of (Z)-1-(5-((4-nitrophenyl)imino)-4-(m-tolyl)-4,5-dihydro-1,3,4-thiadiazol-2-yl)ethan-1-one

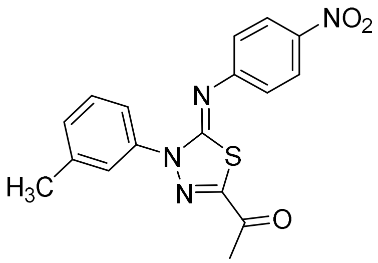

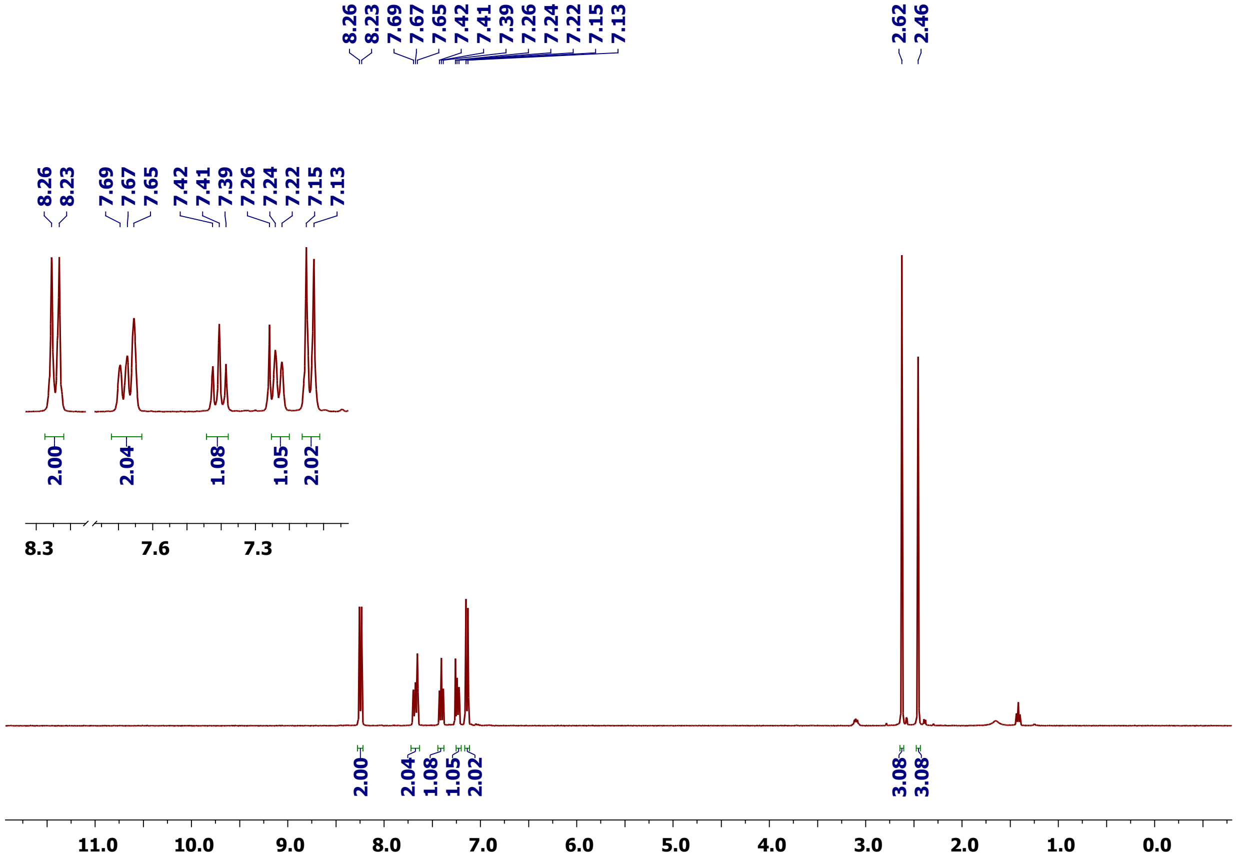

^{13}C NMR ($CDCl_3$) spectrum of (Z)-1-(5-((4-nitrophenyl)imino)-4-(m-tolyl)-4,5-dihydro-1,3,4-thiadiazol-2-yl)ethan-1-one

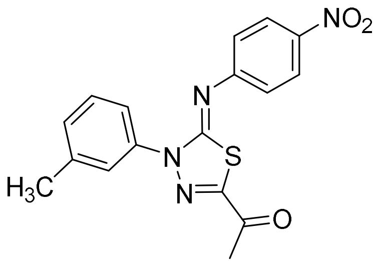

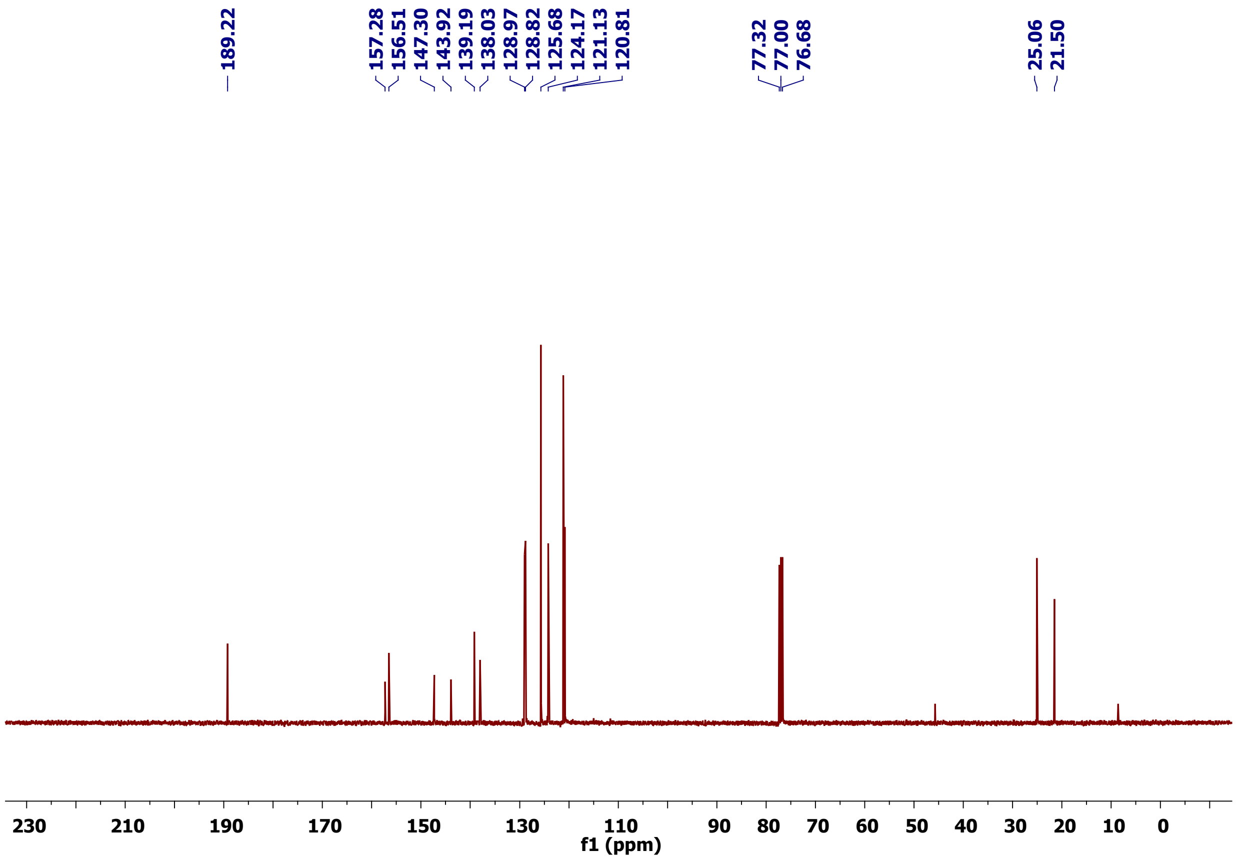

^{13}C CRAPT NMR ($CDCl_3$) spectrum of (Z)-1-(5-((4-nitrophenyl)imino)-4-(m-tolyl)-4,5-dihydro-1,3,4-thiadiazol-2-yl)ethan-1-one

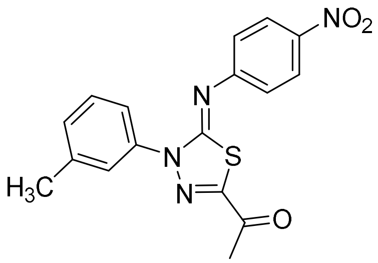

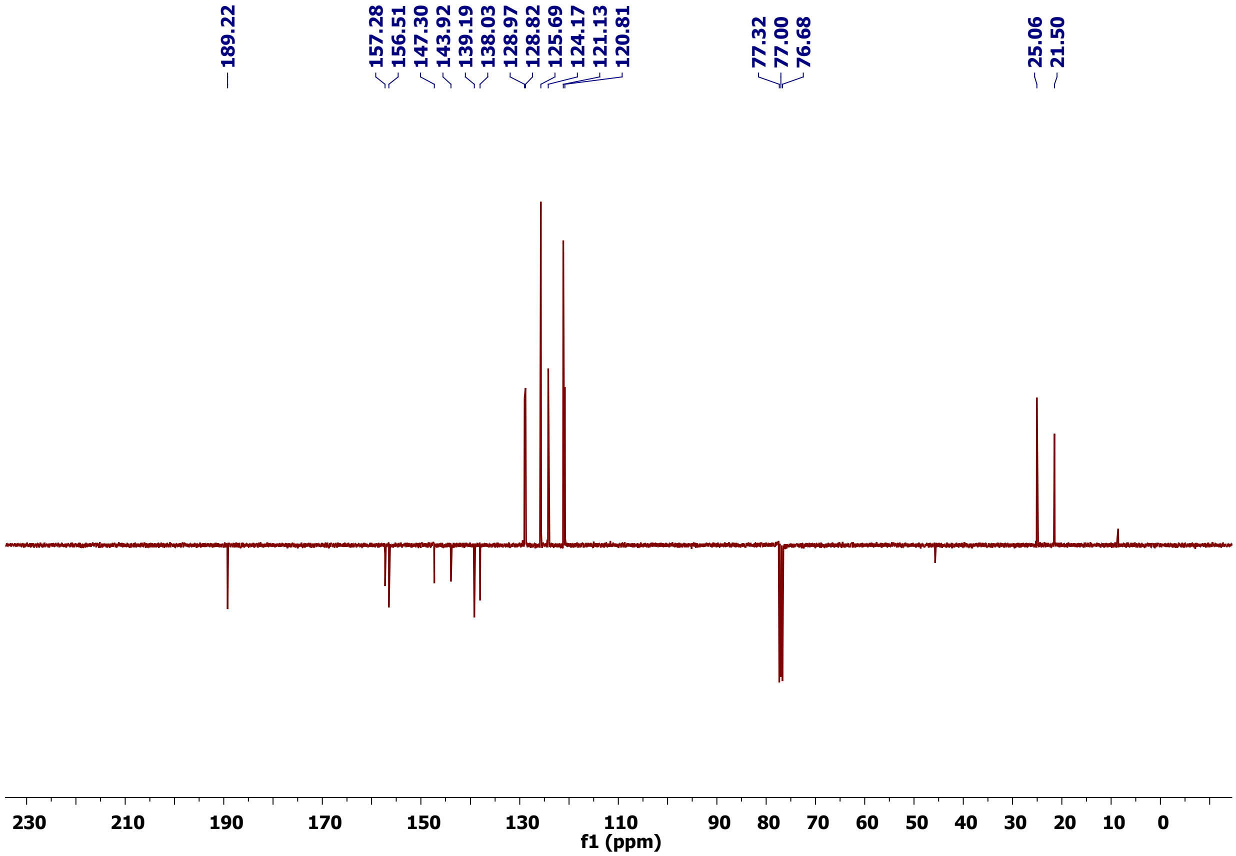

^{1}H-^{1}H gDQCOSY NMR ($CDCl_3$) spectrum of (Z)-1-(5-((4-nitrophenyl)imino)-4-(m-tolyl)-4,5-dihydro-1,3,4-thiadiazol-2-yl)ethan-1-one

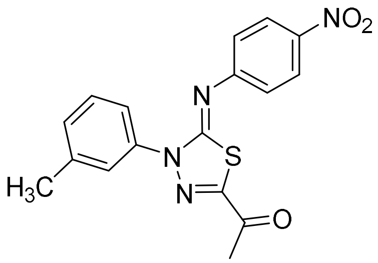

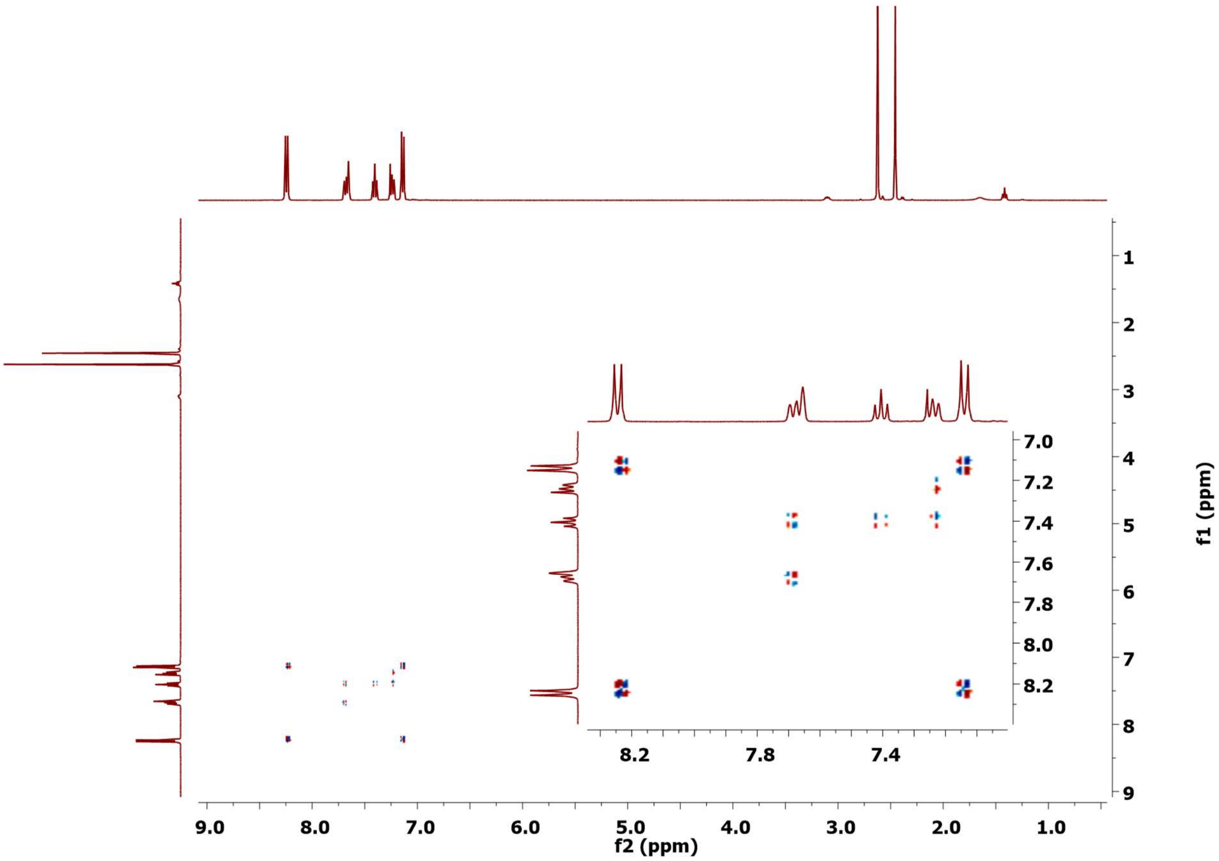

^{1}H-^{13}C-HSQC NMR ($CDCl_3$) spectrum of (Z)-1-(5-((4-nitrophenyl)imino)-4-(m-tolyl)-4,5-dihydro-1,3,4-thiadiazol-2-yl)ethan-1-one

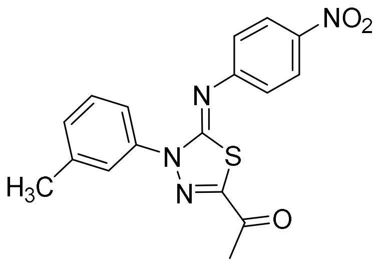

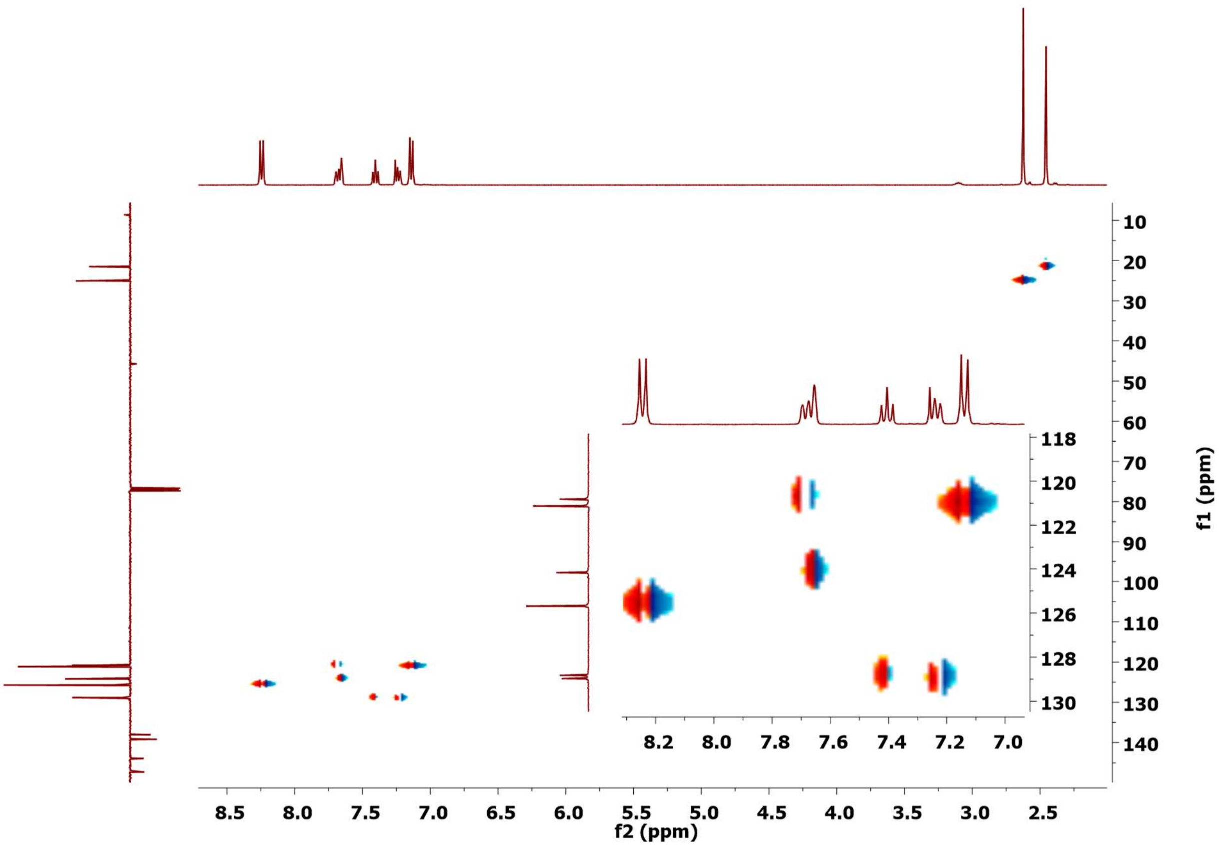

^{1}H-^{13}C-gHMBC NMR ($CDCl_3$) spectrum of (Z)-1-(5-((4-nitrophenyl)imino)-4-(m-tolyl)-4,5-dihydro-1,3,4-thiadiazol-2-yl)ethan-1-one

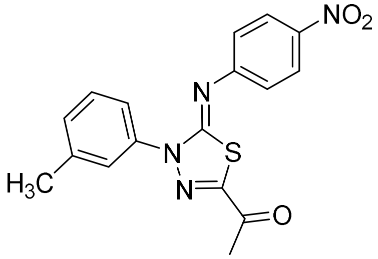

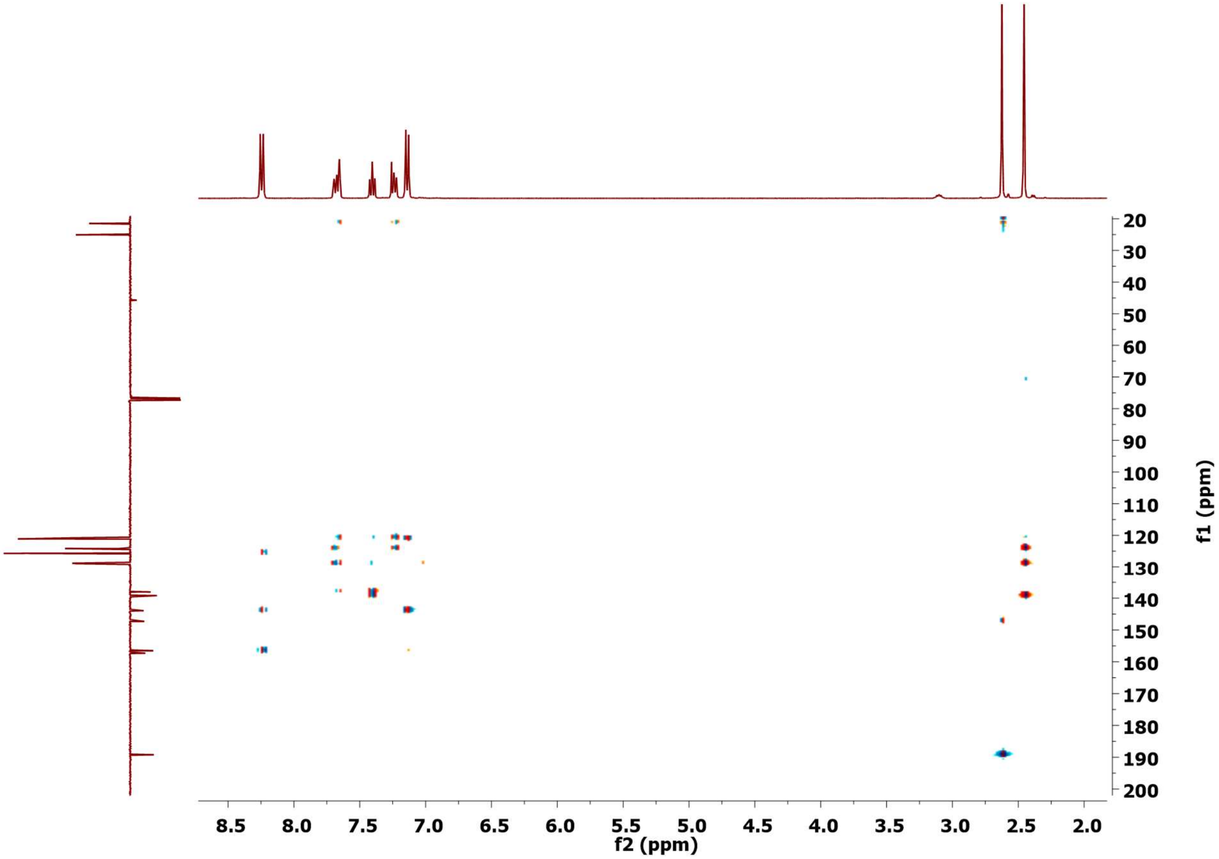

^{1}H NMR ($CDCl_3$) spectrum of (Z)-1-(4-(benzo[d][1,3]dioxol-5-yl)-5-((4-nitrophenyl)imino)-4,5-dihydro-1,3,4-thiadiazol-2-yl)ethan-1-one

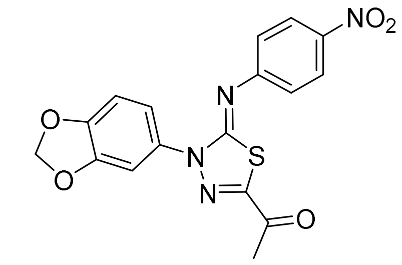

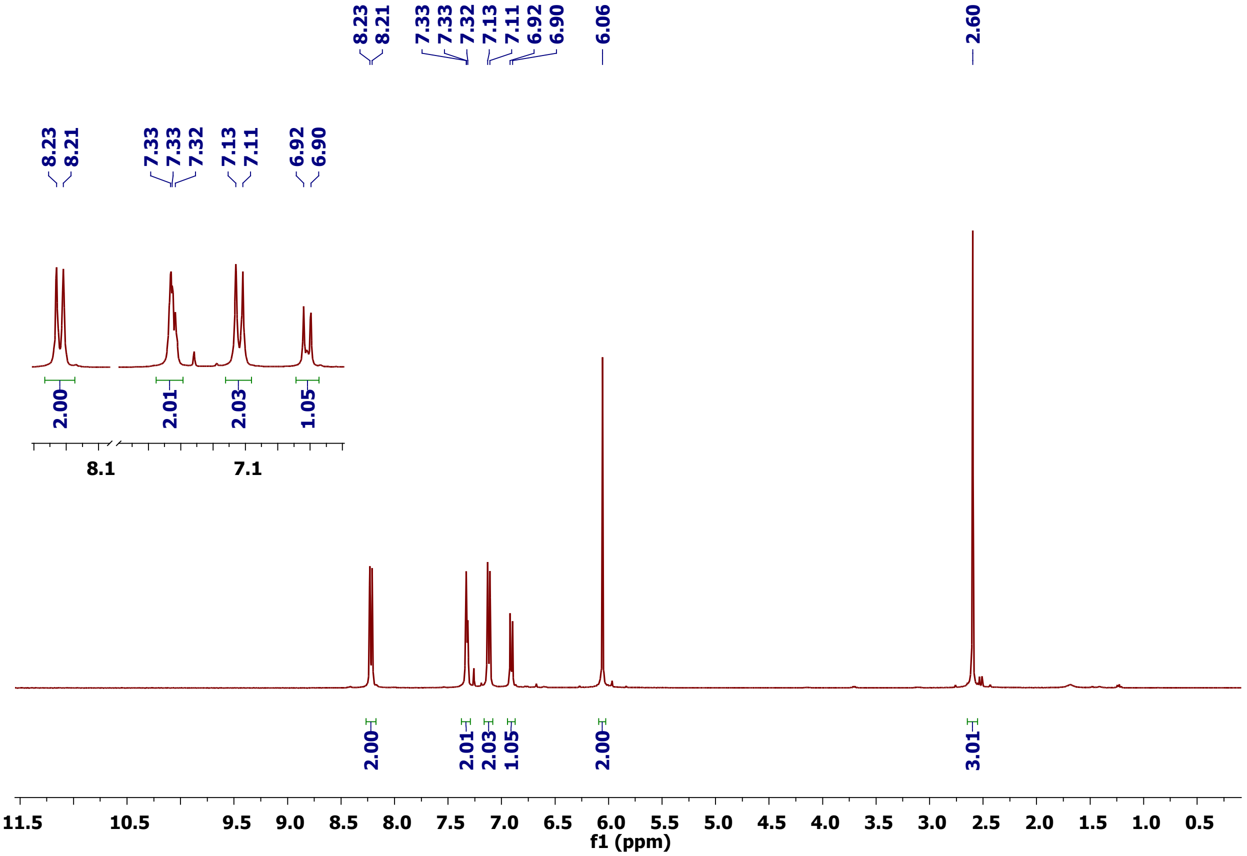

^{13}C NMR ($CDCl_3$) spectrum of (Z)-1-(4-(benzo[d][1,3]dioxol-5-yl)-5-((4-nitrophenyl)imino)-4,5-dihydro-1,3,4-thiadiazol-2-yl)ethan-1-one

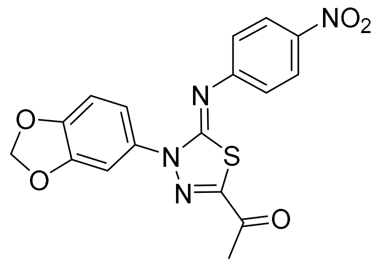

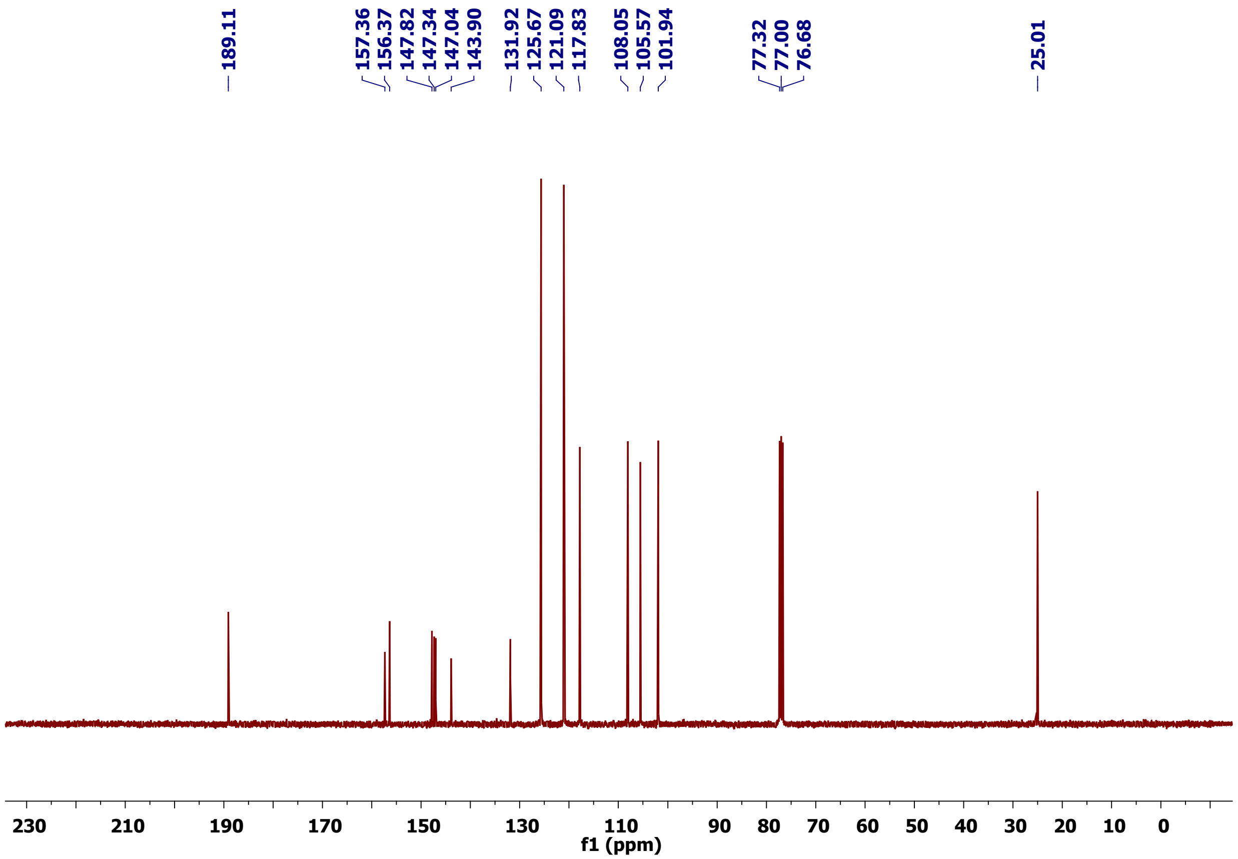

^{13}C CRAPT NMR ($CDCl_3$) spectrum of (Z)-1-(4-(benzo[d][1,3]dioxol-5-yl)-5-((4-nitrophenyl)imino)-4,5-dihydro-1,3,4-thiadiazol-2-yl)ethan-1-one

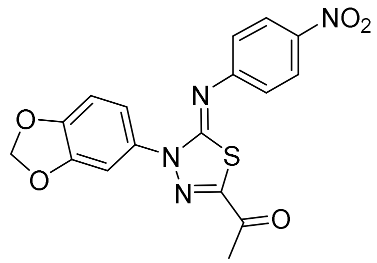

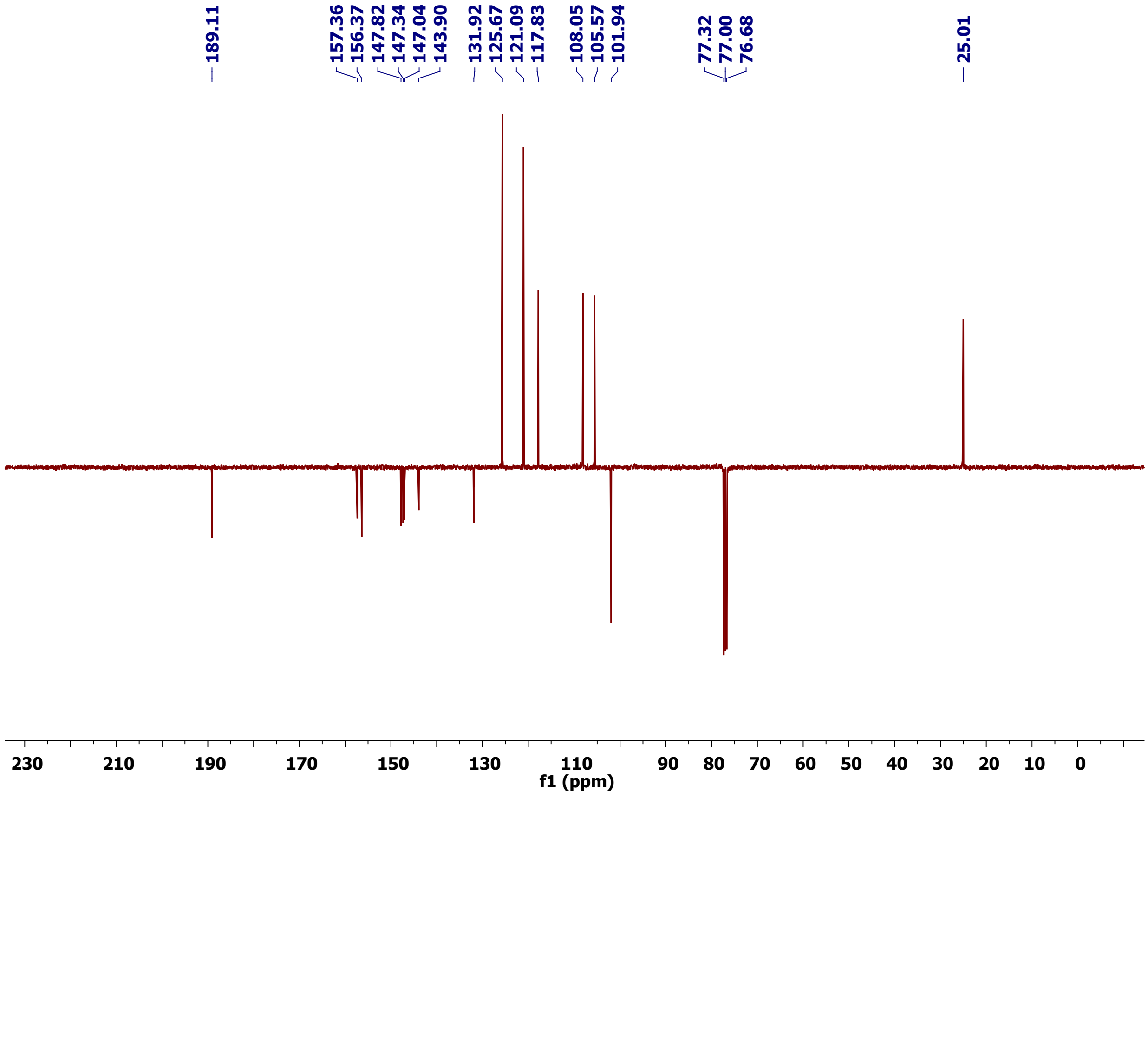

^1H-^1H gDQCOSY NMR ($CDCl_3$) spectrum of (Z)-1-(4-(benzo[d][1,3]dioxol-5-yl)-5-((4-nitrophenyl)imino)-4,5-dihydro-1,3,4-thiadiazol-2-yl)ethan-1-one

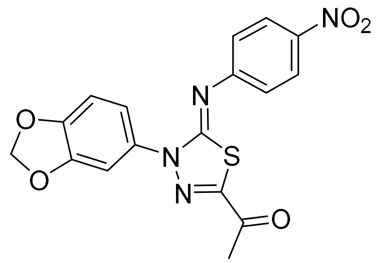

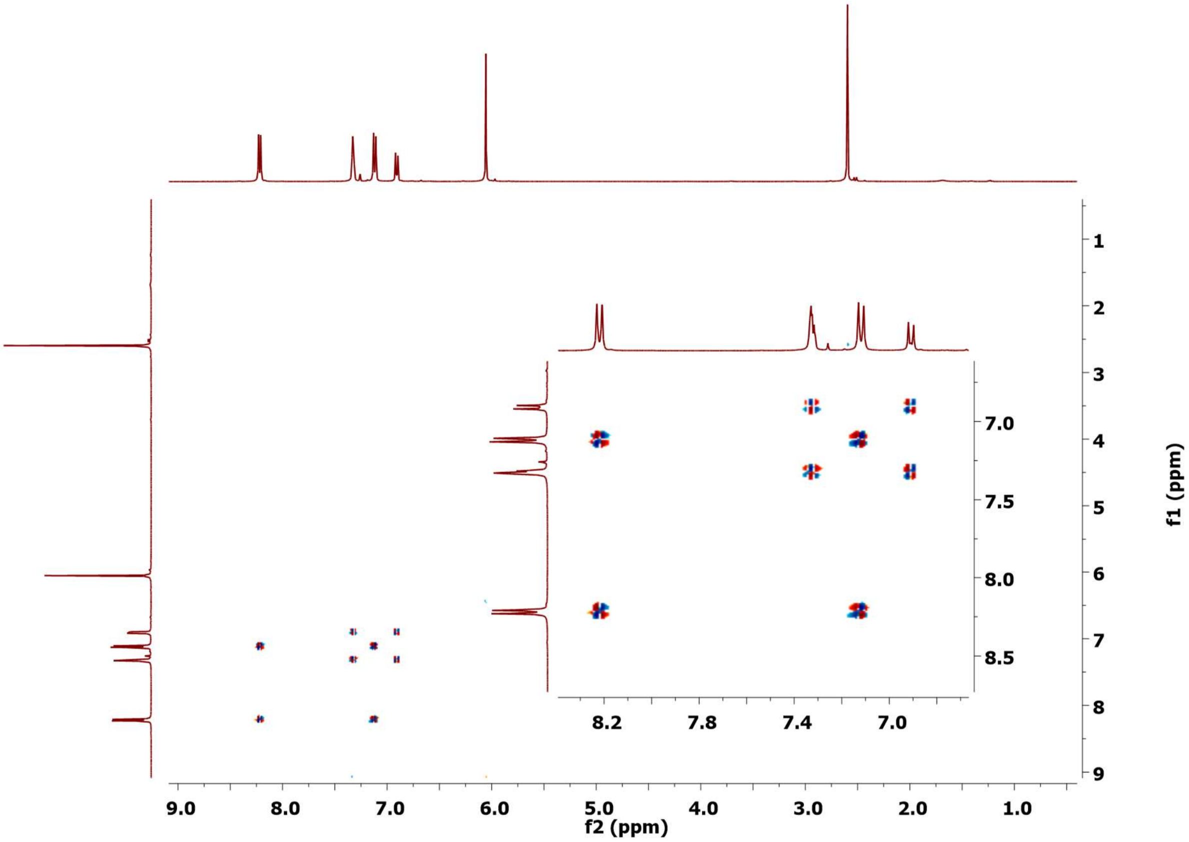

^{1}H-^{13}C-HSQC NMR ($CDCl_3$) spectrum of (Z)-1-(4-(benzo[d][1,3]dioxol-5-yl)-5-((4-nitrophenyl)imino)-4,5-dihydro-1,3,4-thiadiazol-2-yl)ethan-1-one

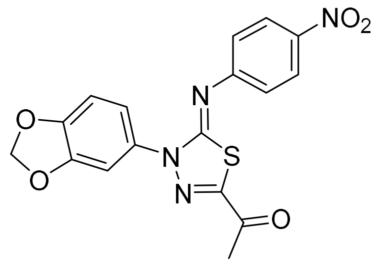

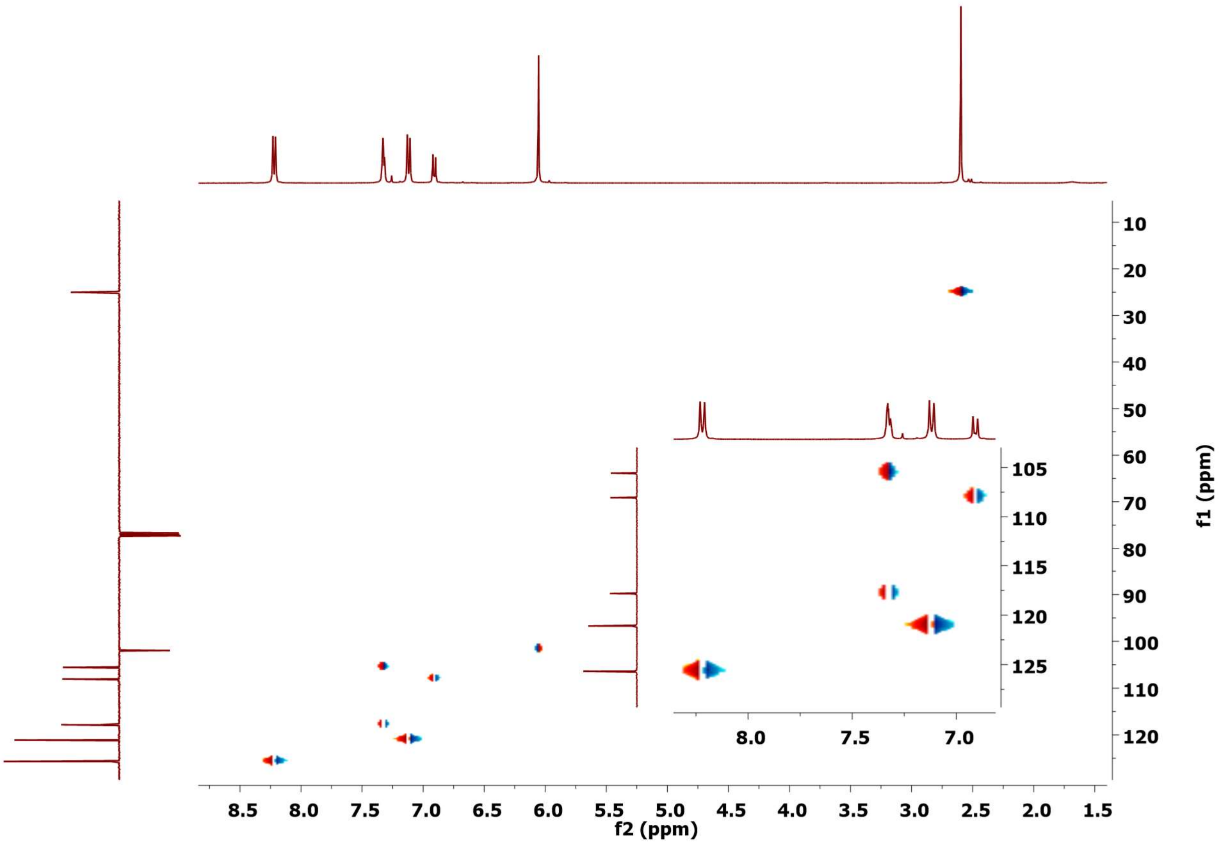

^{1}H-^{13}C-gHMBC NMR ($CDCl_3$) spectrum of (Z)-1-(4-(benzo[d][1,3]dioxol-5-yl)-5-((4-nitrophenyl)imino)-4,5-dihydro-1,3,4-thiadiazol-2-yl)ethan-1-one

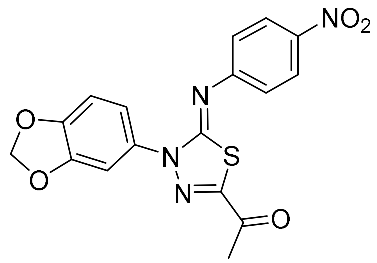

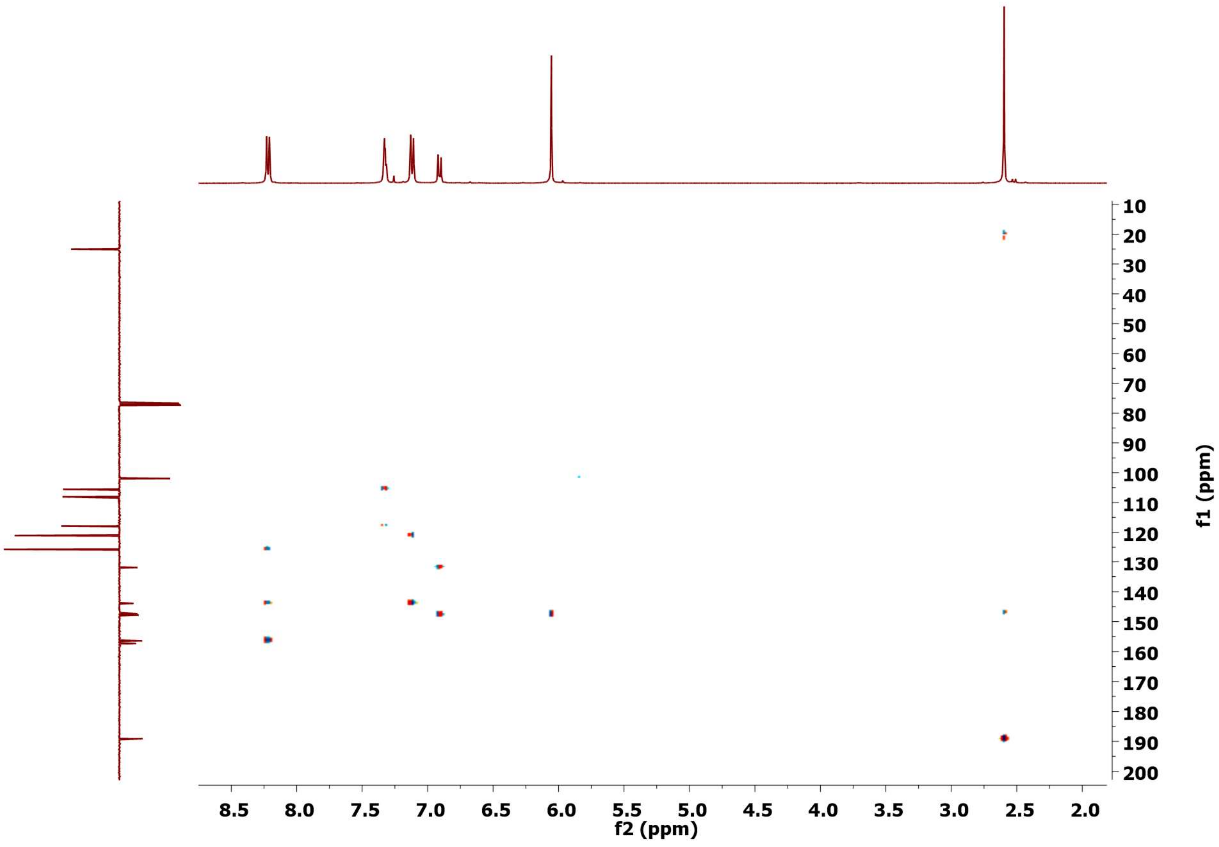

^{1}H NMR ($CDCl_3$) spectrum of (Z)-1-(5-((4-nitrophenyl)imino)-4-(4-(trifluoromethyl)phenyl)-4,5-dihydro-1,3,4-thiadiazol-2-yl)ethan-1-one

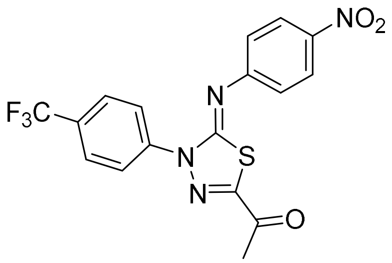

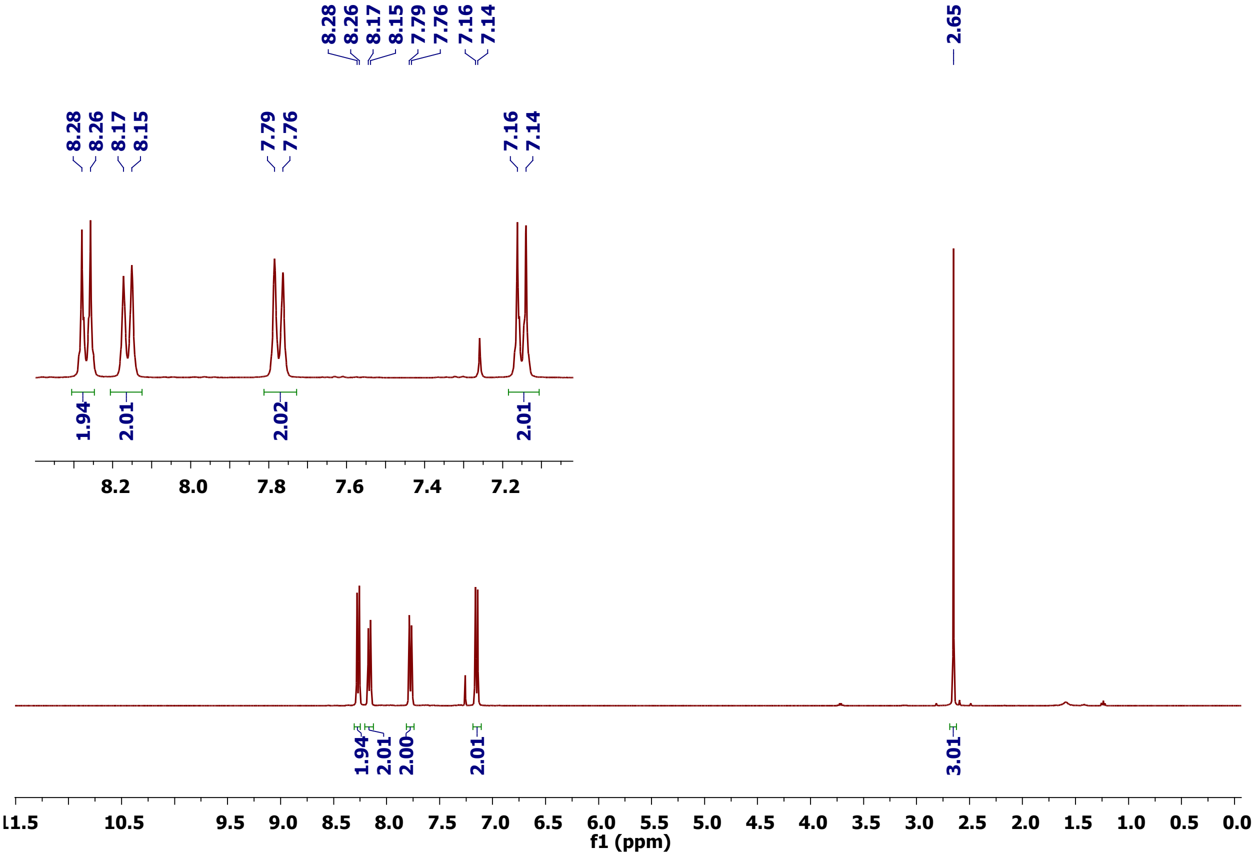

^{13}C NMR ($CDCl_3$) spectrum of (Z)-1-(5-((4-nitrophenyl)imino)-4-(4-(trifluoromethyl)phenyl)-4,5-dihydro-1,3,4-thiadiazol-2-yl)ethan-1-one

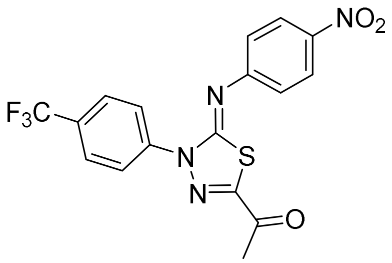

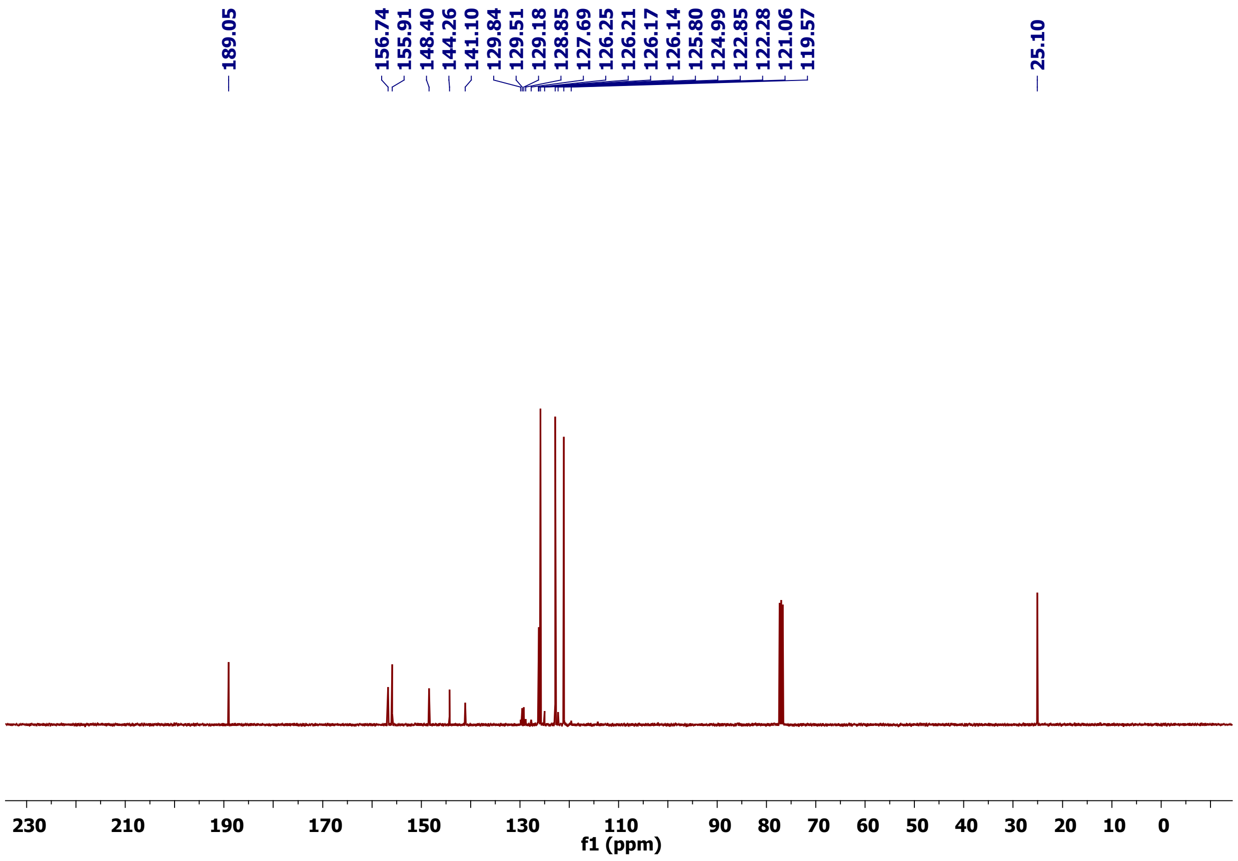

^{13}C CRAPT NMR ($CDCl_3$) spectrum of (Z)-1-(5-((4-nitrophenyl)imino)-4-(4-(trifluoromethyl)phenyl)-4,5-dihydro-1,3,4-thiadiazol-2-yl)ethan-1-one

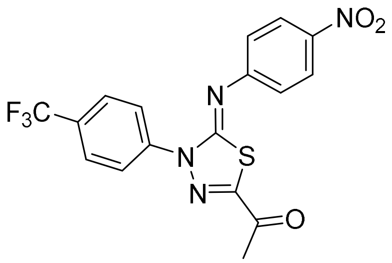

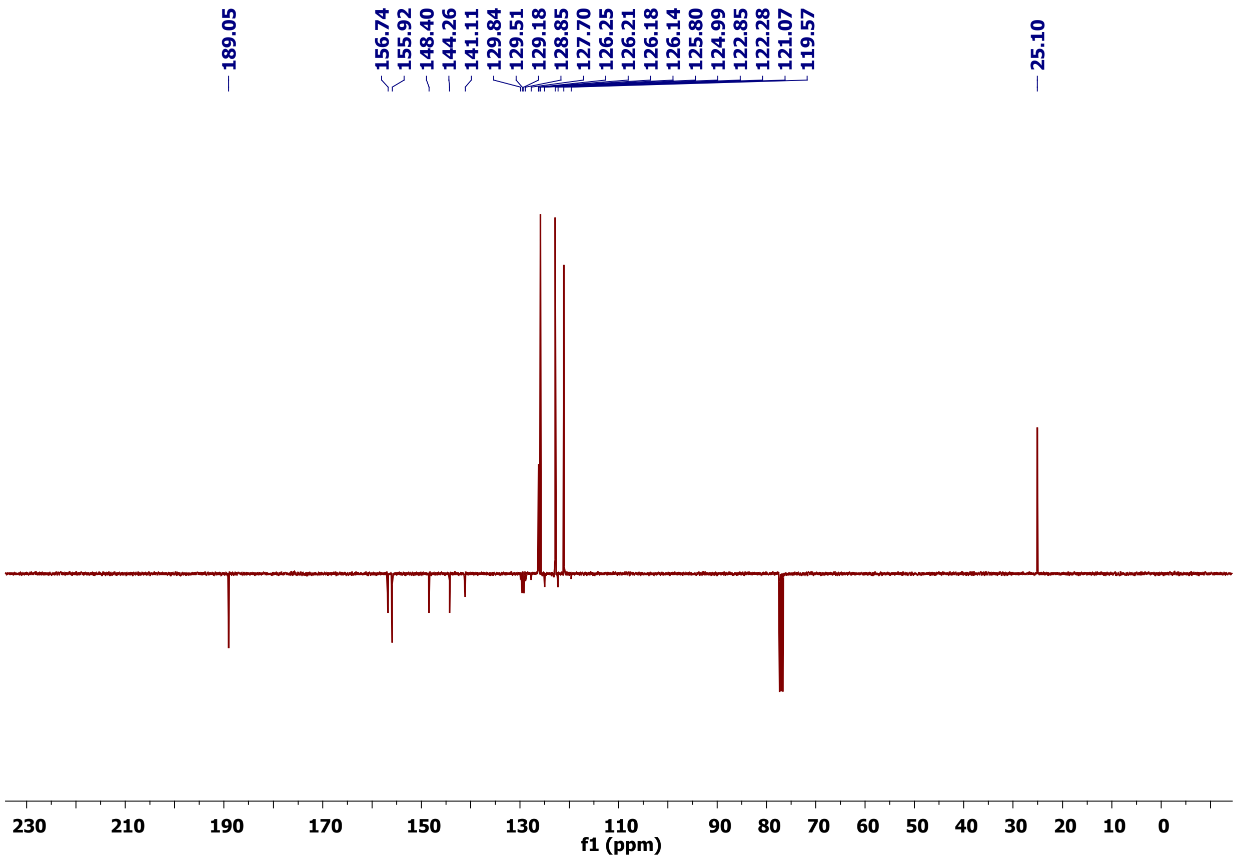

^1H-^1H gDQCOSY NMR ($CDCl_3$) spectrum of (Z)-1-(5-((4-nitrophenyl)imino)-4-(4-(trifluoromethyl)phenyl)-4,5-dihydro-1,3,4-thiadiazol-2-yl)ethan-1-one

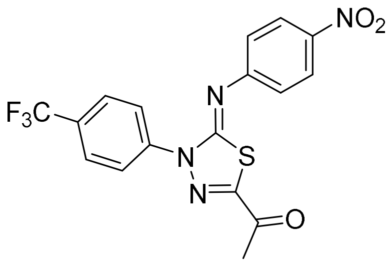

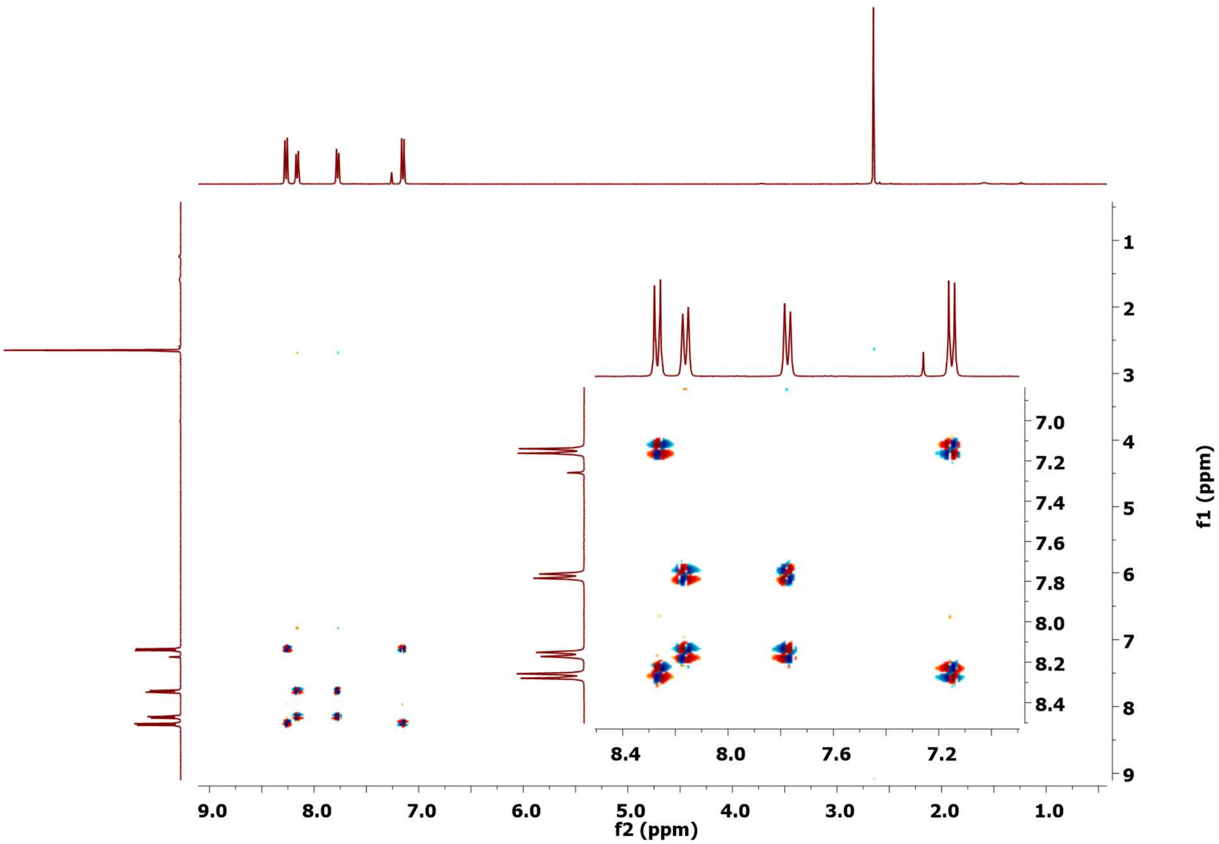

^{1}H-^{13}C-HSQC NMR ($CDCl_3$) spectrum of (Z)-1-(5-((4-nitrophenyl)imino)-4-(4-(trifluoromethyl)phenyl)-4,5-dihydro-1,3,4-thiadiazol-2-yl)ethan-1-one

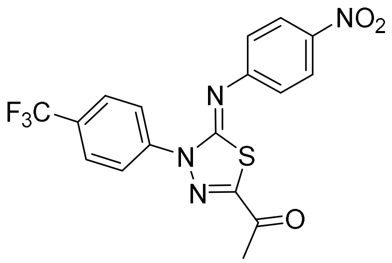

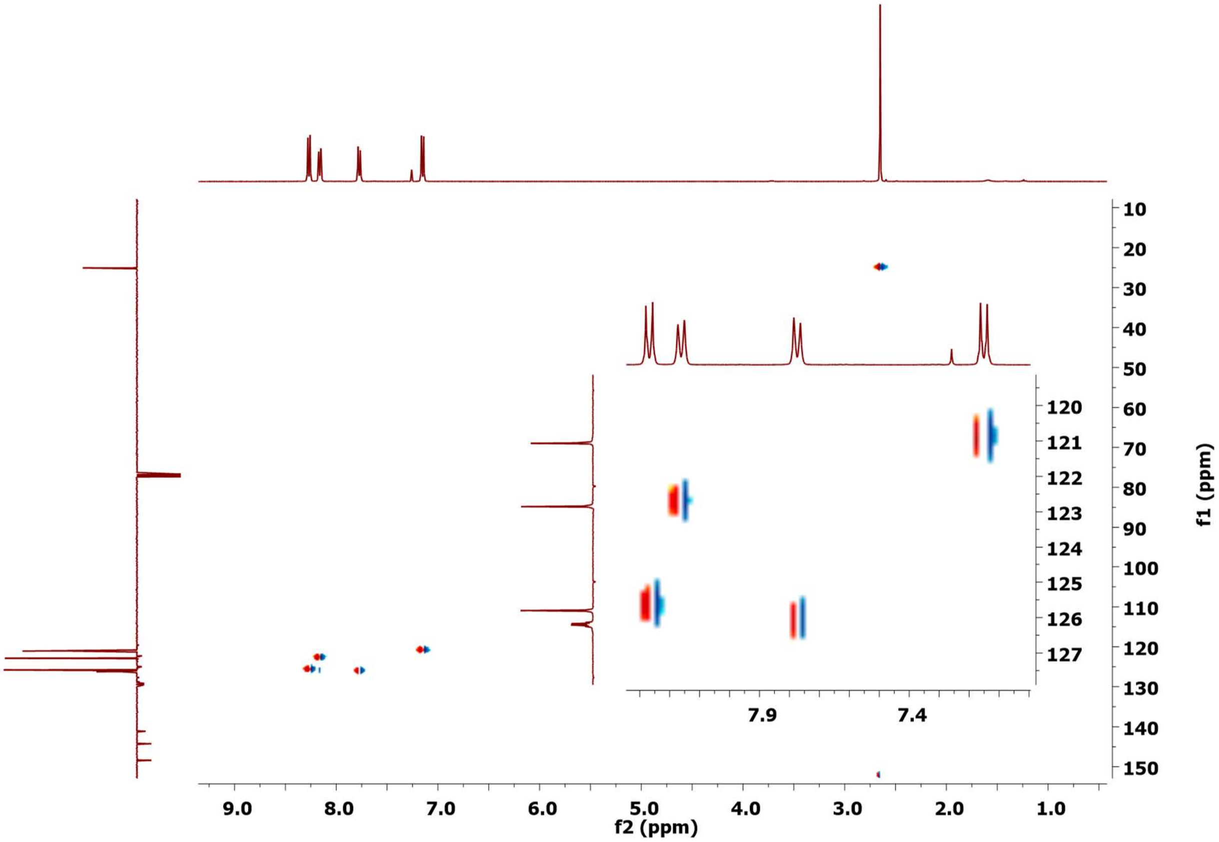

^{1}H-^{13}C-gHMBC NMR ($CDCl_3$) spectrum of (Z)-1-(5-((4-nitrophenyl)imino)-4-(4-(trifluoromethyl)phenyl)-4,5-dihydro-1,3,4-thiadiazol-2-yl)ethan-1-one

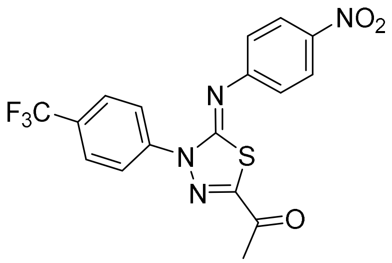

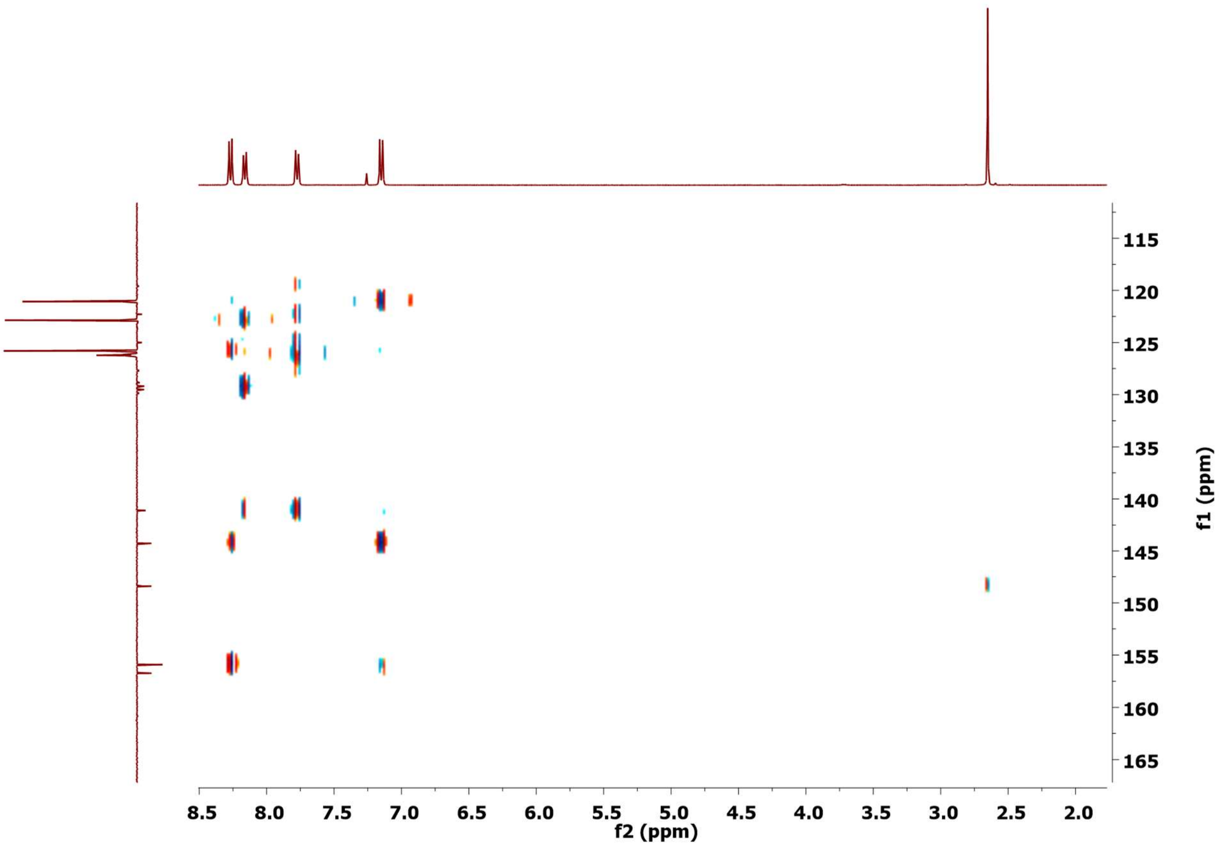

^{1}H NMR (CDCl3) spectrum of (Z)-1-(5-((4-nitrophenyl)imino)-4-(3-(trifluoromethyl)phenyl)-4,5-dihydro-1,3,4-thiadiazol-2-yl)ethan-1-one

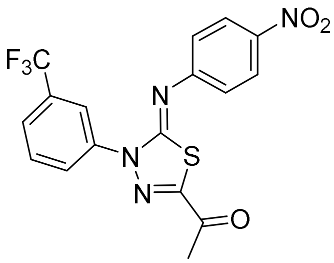

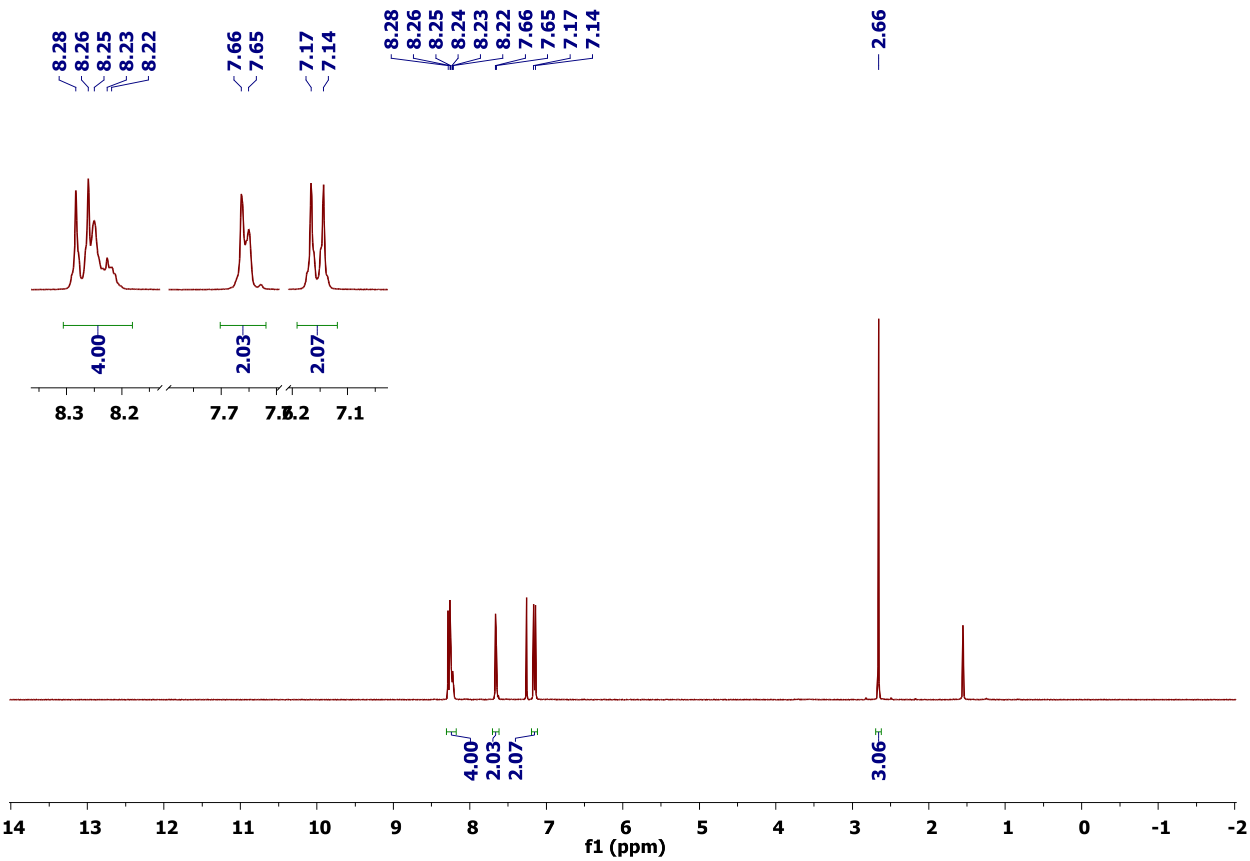

^{13}C NMR ($CDCl_3$) spectrum of (Z)-1-(5-((4-nitrophenyl)imino)-4-(3-(trifluoromethyl)phenyl)-4,5-dihydro-1,3,4-thiadiazol-2-yl)ethan-1-one

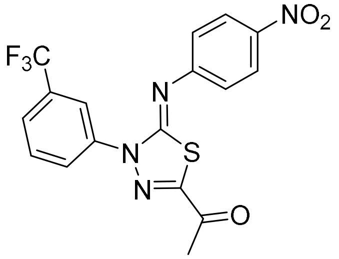

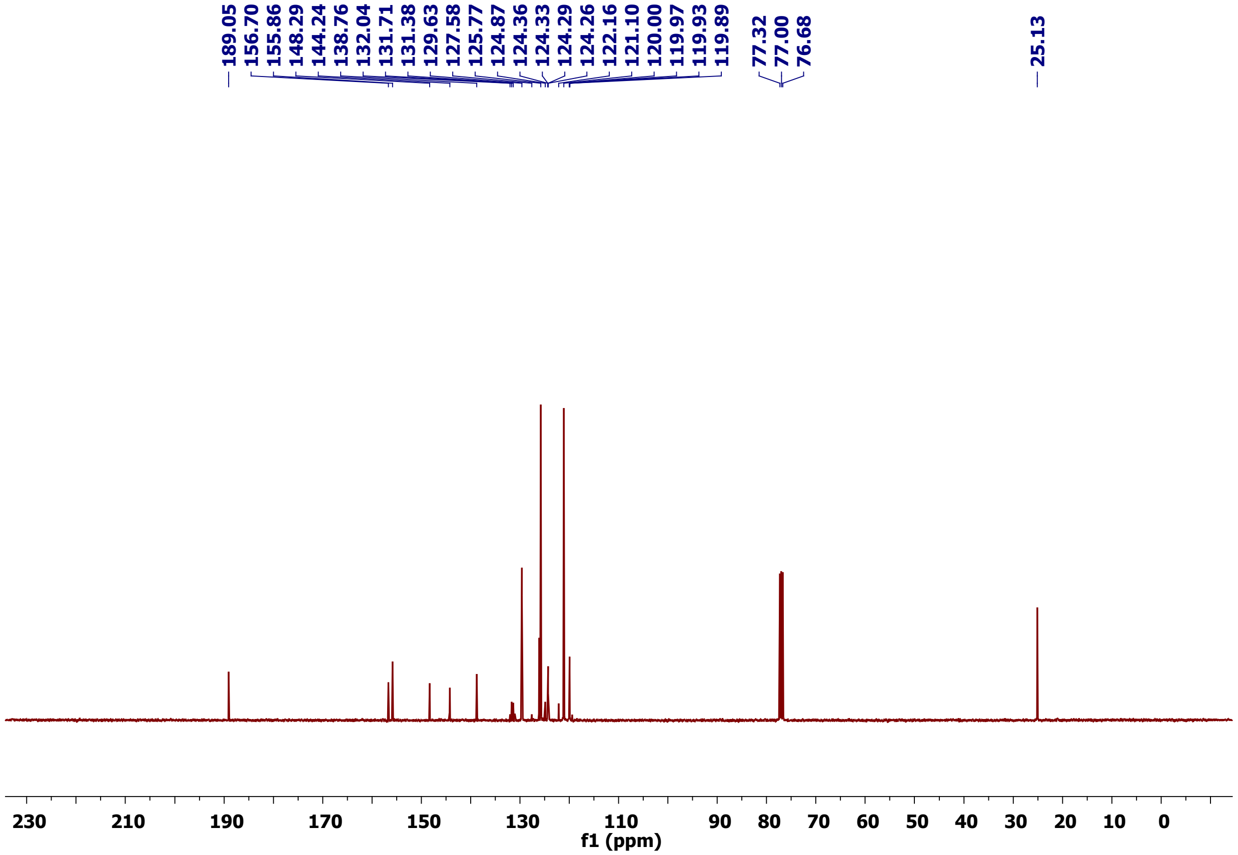

^{13}C CRAPT NMR ($CDCl_3$) spectrum of (Z)-1-(5-((4-nitrophenyl)imino)-4-(3-(trifluoromethyl)phenyl)-4,5-dihydro-1,3,4-thiadiazol-2-yl)ethan-1-one

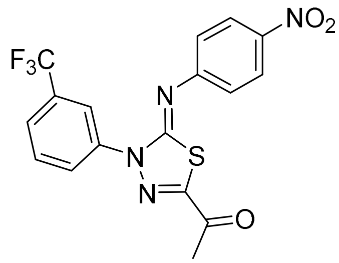

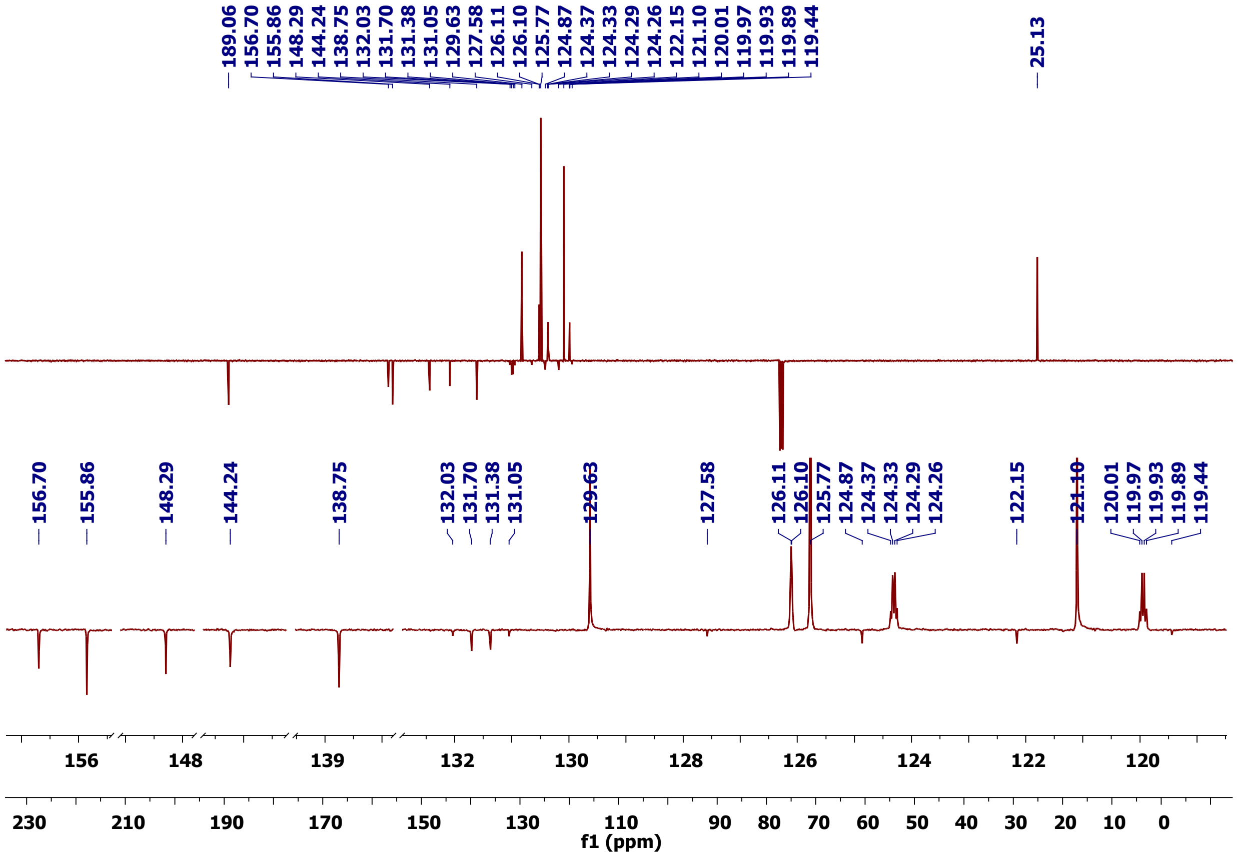

^{1}H-^{1}H gDQCOSY NMR ($CDCl_3$) spectrum of (Z)-1-(5-((4-nitrophenyl)imino)-4-(3-(trifluoromethyl)phenyl)-4,5-dihydro-1,3,4-thiadiazol-2-yl)ethan-1-one

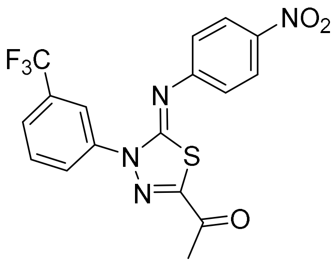

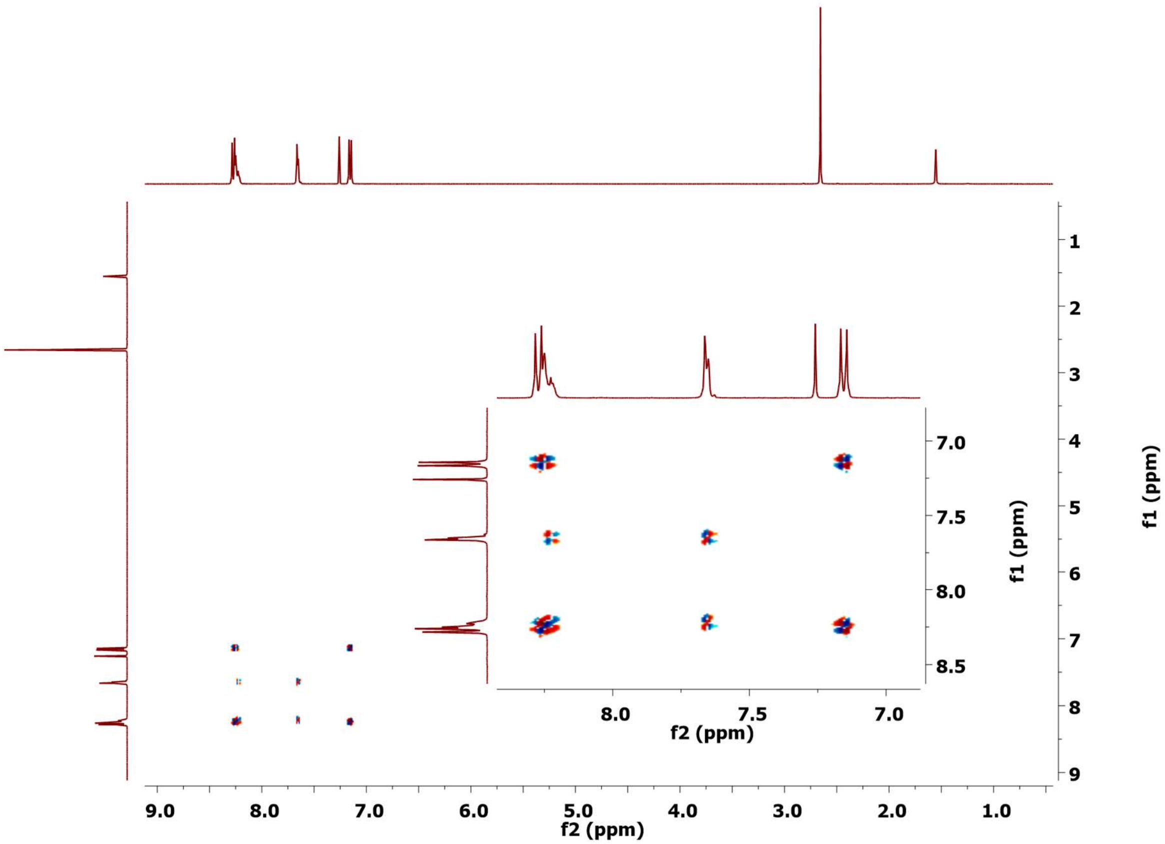

^{1}H-^{13}C-HSQC NMR ($CDCl_3$) spectrum of (Z)-1-(5-((4-nitrophenyl)imino)-4-(3-(trifluoromethyl)phenyl)-4,5-dihydro-1,3,4-thiadiazol-2-yl)ethan-1-one

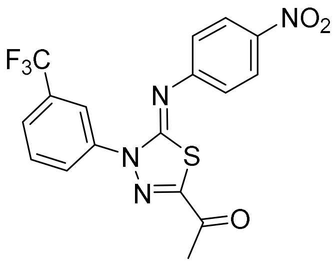

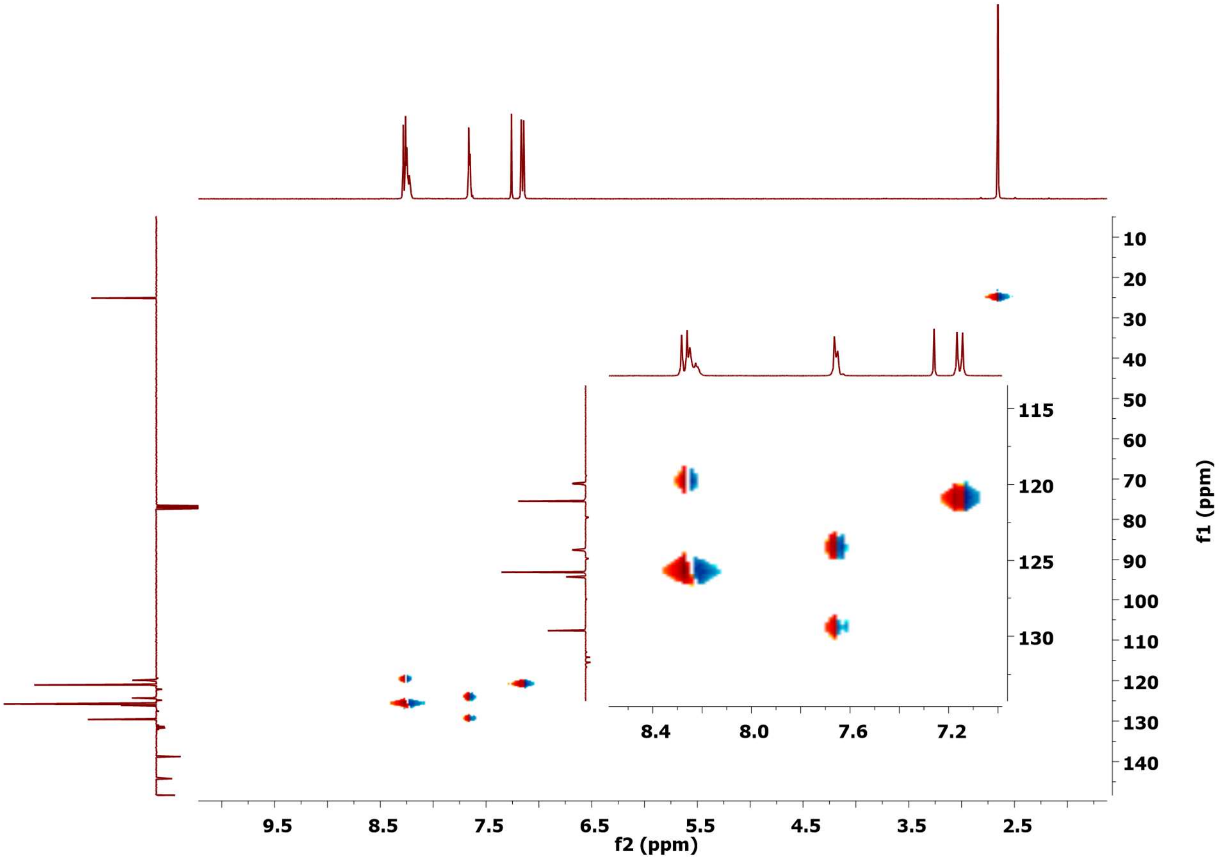

^{1}H-^{13}C-gHMBC NMR ($CDCl_3$) spectrum of (Z)-1-(5-((4-nitrophenyl)imino)-4-(3-(trifluoromethyl)phenyl)-4,5-dihydro-1,3,4-thiadiazol-2-yl)ethan-1-one

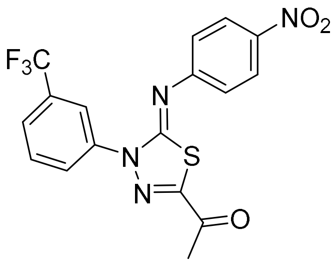

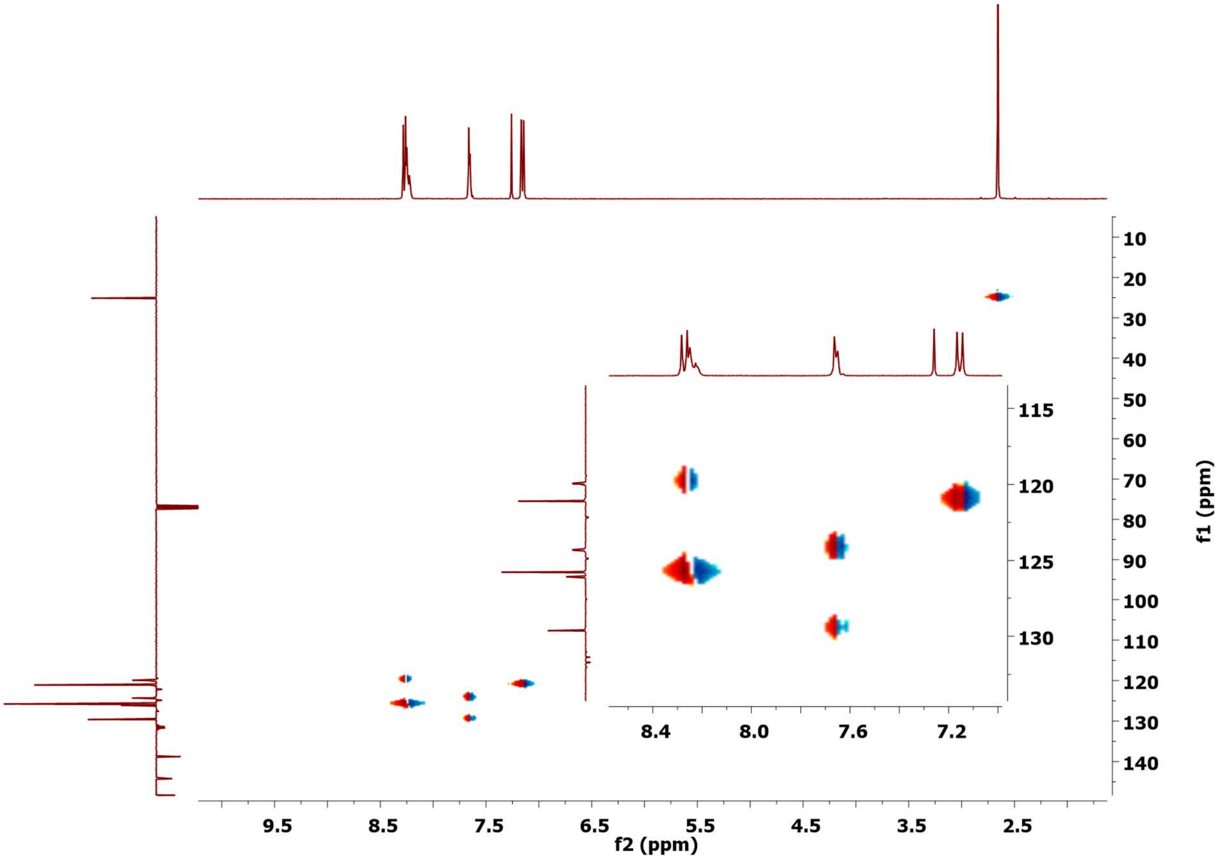

^{1}H NMR ($CDCl_3$) spectrum of (Z)-1-(4-(naphthalen-1-yl)-5-((4-nitrophenyl)imino)-4,5-dihydro-1,3,4-thiadiazol-2-yl)ethan-1-one

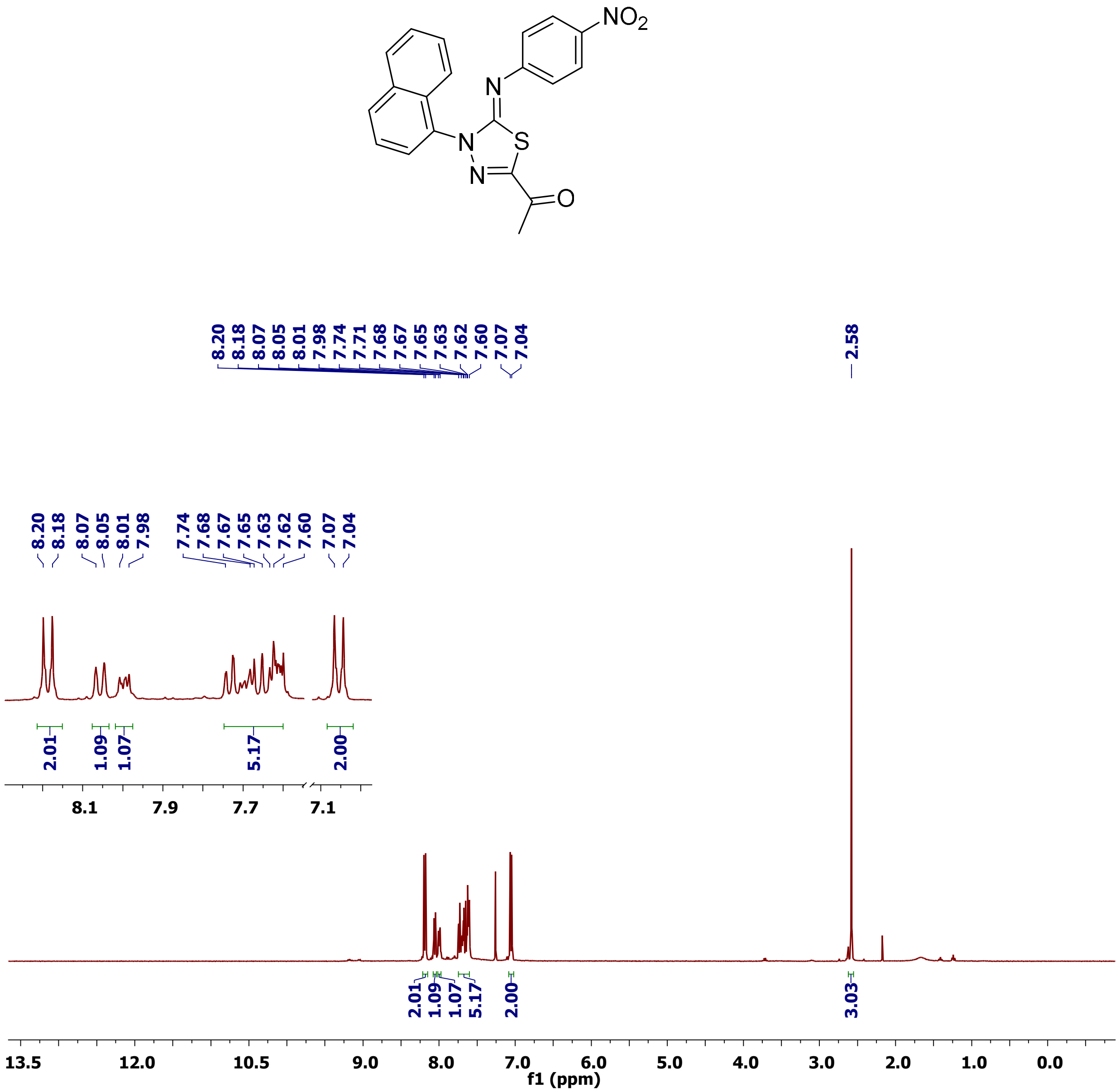

^{13}C NMR ($CDCl_3$) spectrum of (Z)-1-(4-(naphthalen-1-yl)-5-((4-nitrophenyl)imino)-4,5-dihydro-1,3,4-thiadiazol-2-yl)ethan-1-one

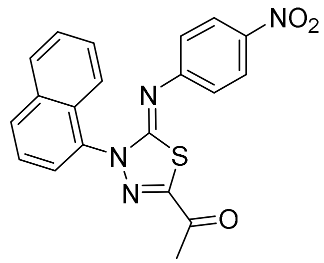

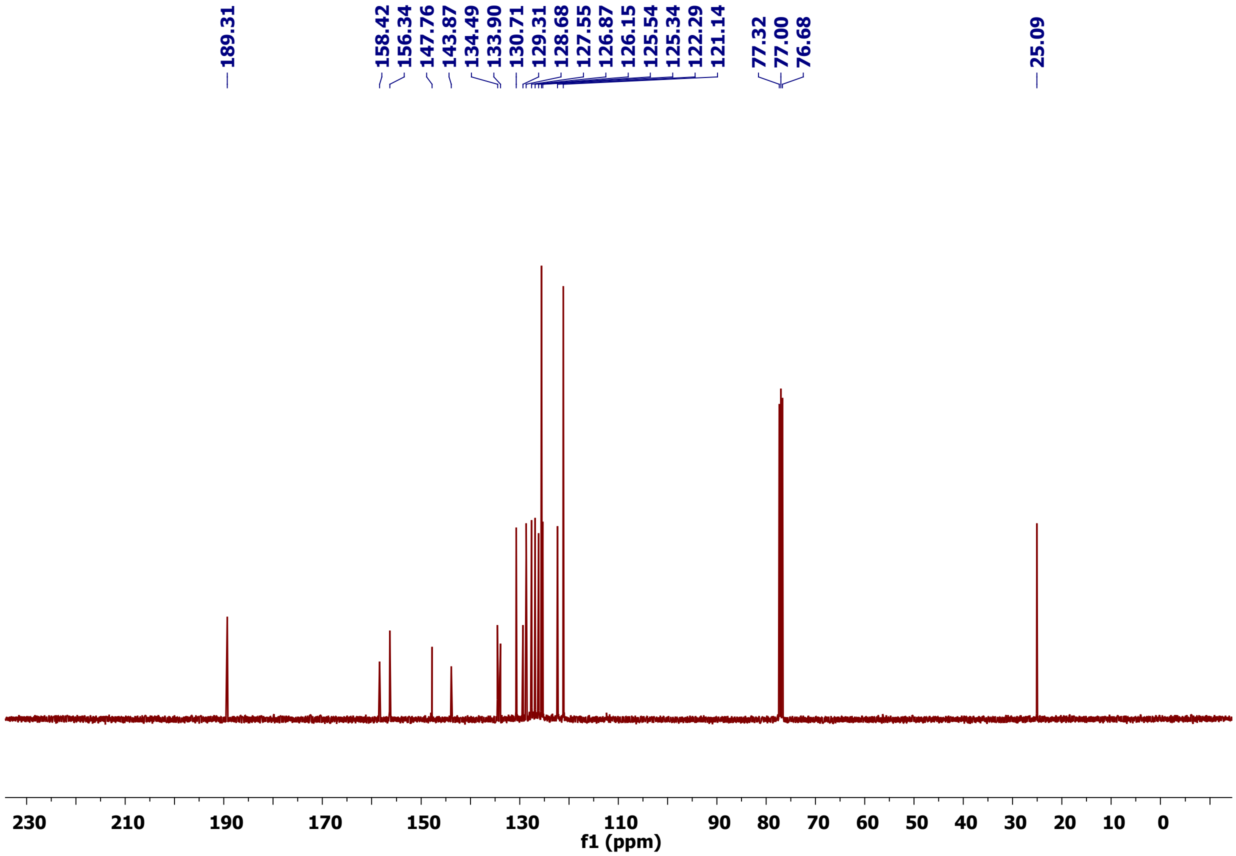

^{13}C CRAPT NMR ($CDCl_3$) spectrum of (Z)-1-(4-(naphthalen-1-yl)-5-((4-nitrophenyl)imino)-4,5-dihydro-1,3,4-thiadiazol-2-yl)ethan-1-one

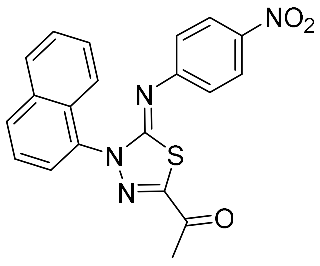

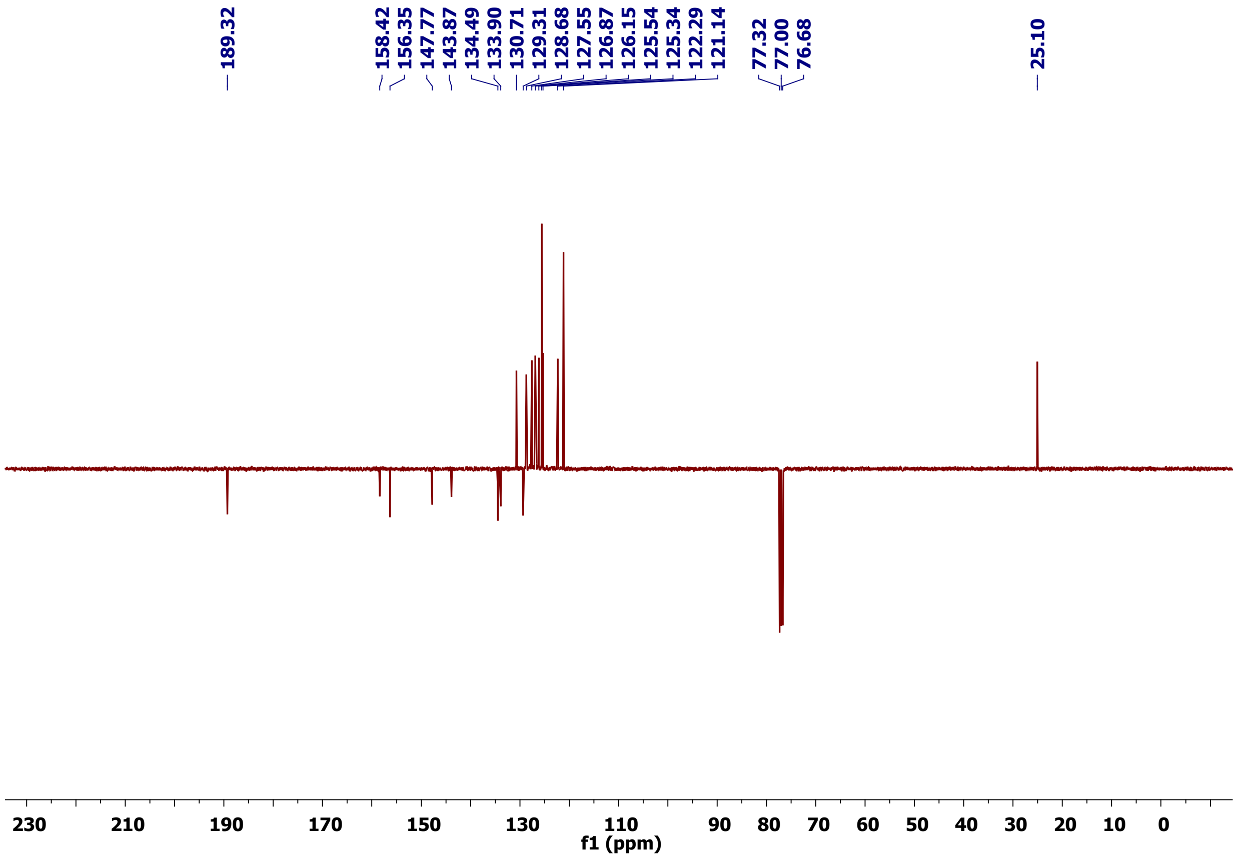

^1H-^1H gDQCOSY NMR ($CDCl_3$) spectrum of (Z)-1-(4-(naphthalen-1-yl)-5-((4-nitrophenyl)imino)-4,5-dihydro-1,3,4-thiadiazol-2-yl)ethan-1-one

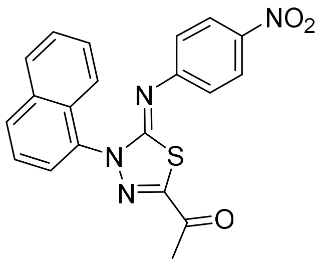

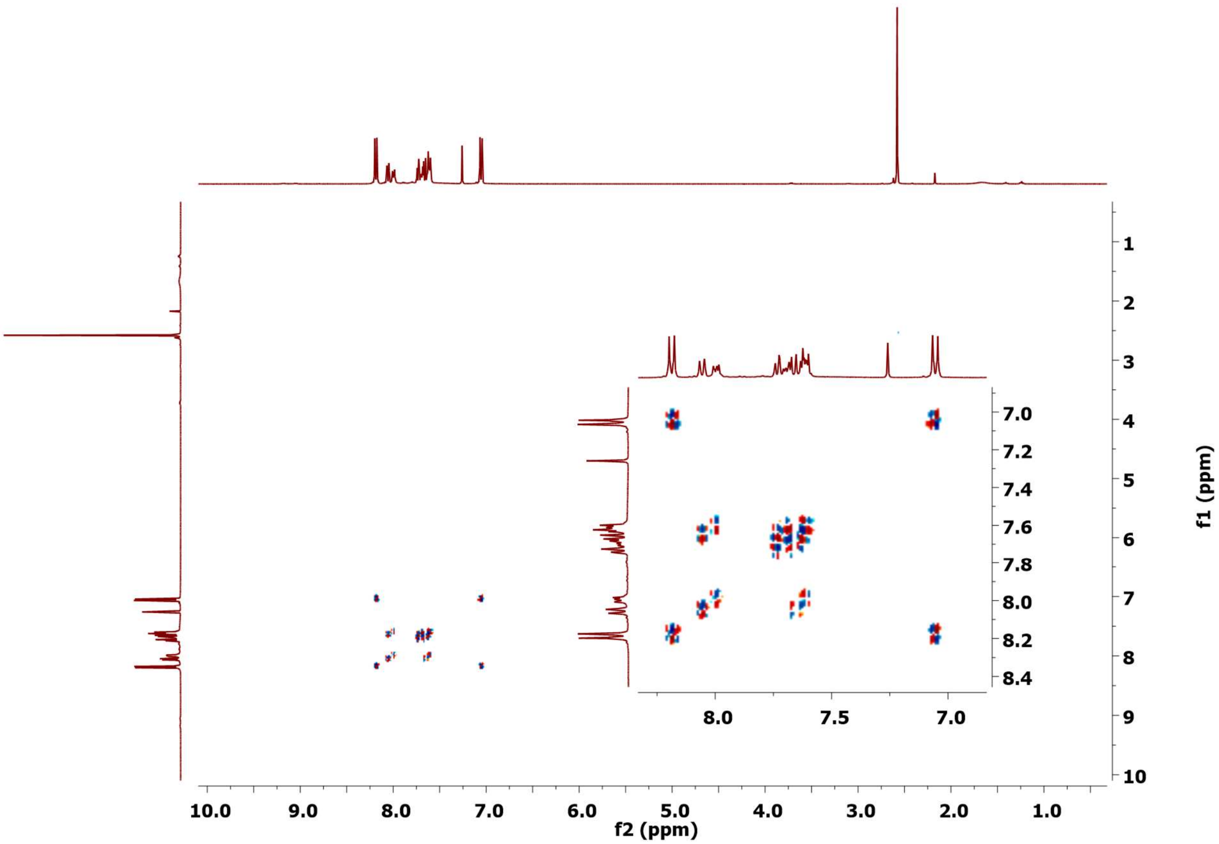

^{1}H-^{13}C-HSQC NMR ($CDCl_3$) spectrum of (Z)-1-(4-(naphthalen-1-yl)-5-((4-nitrophenyl)imino)-4,5-dihydro-1,3,4-thiadiazol-2-yl)ethan-1-one

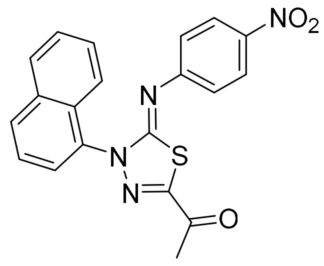

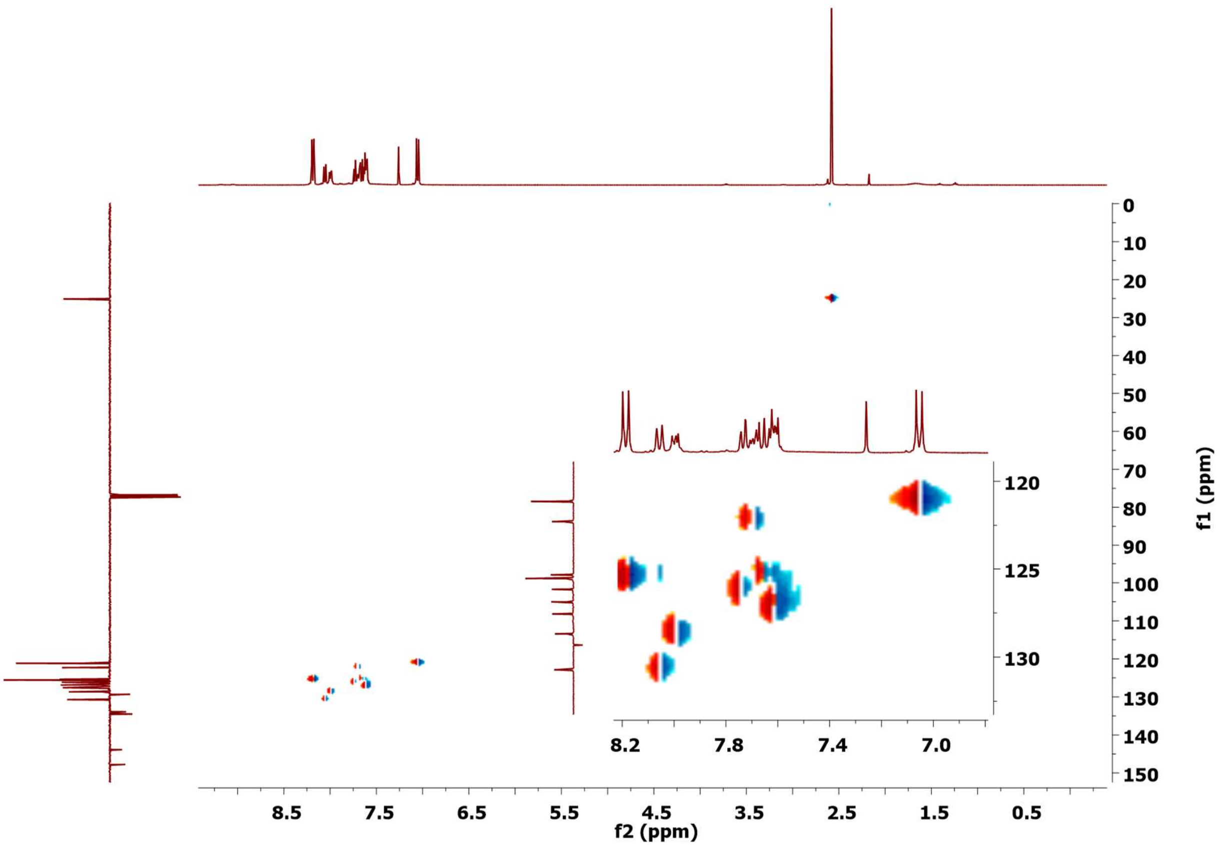

^{1}H-^{13}C-gHMBC NMR ($CDCl_3$) spectrum of (Z)-1-(4-(naphthalen-1-yl)-5-((4-nitrophenyl)imino)-4,5-dihydro-1,3,4-thiadiazol-2-yl)ethan-1-one

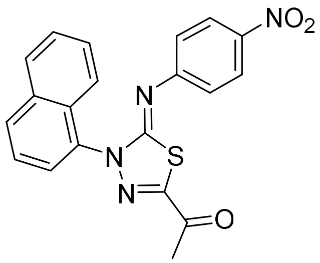

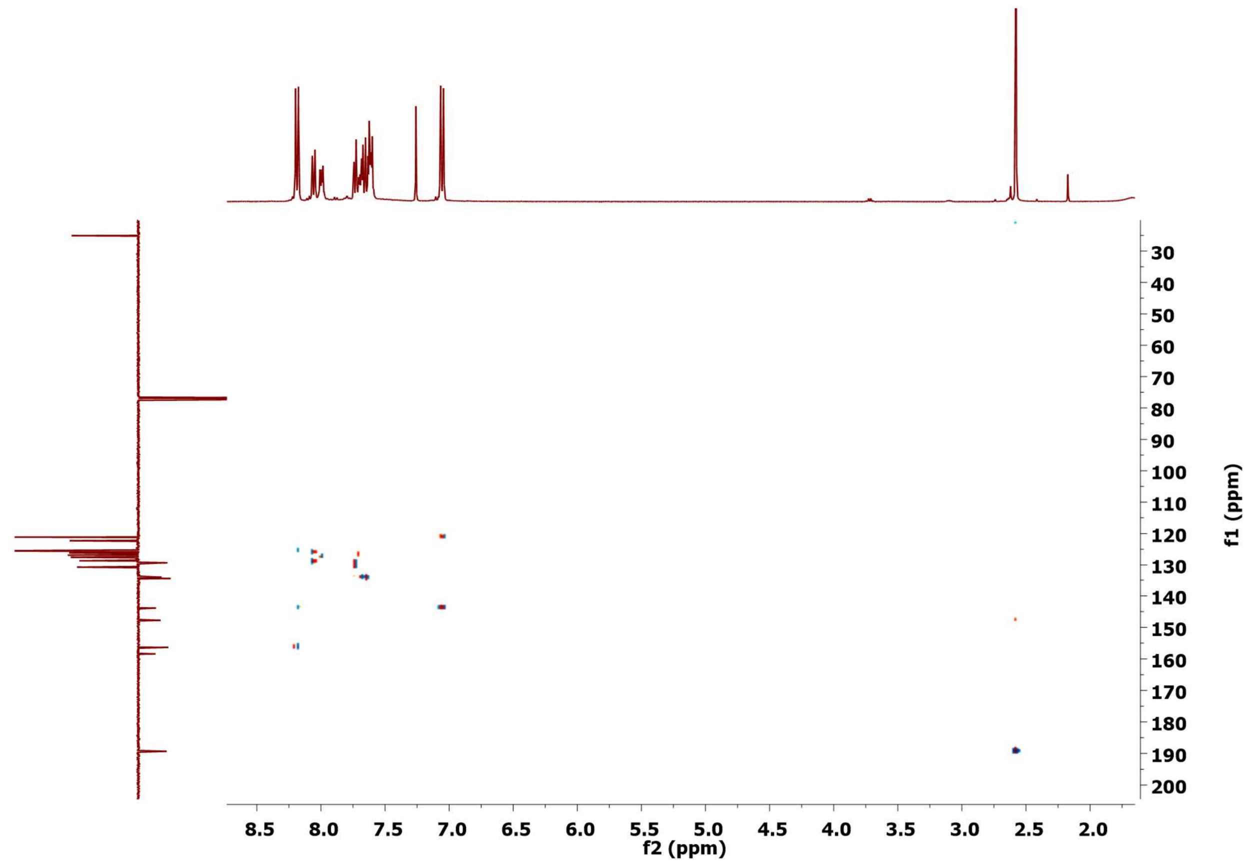

^{1}H NMR ($CDCl_3$) spectrum of (Z)-1-(4-(naphthalen-1-yl)-5-((4-nitrophenyl)imino)-4,5-dihydro-1,3,4-thiadiazol-2-yl)ethan-1-one

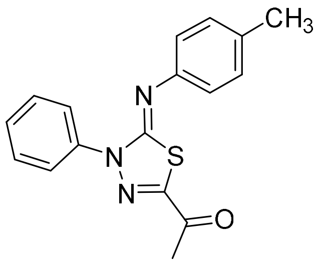

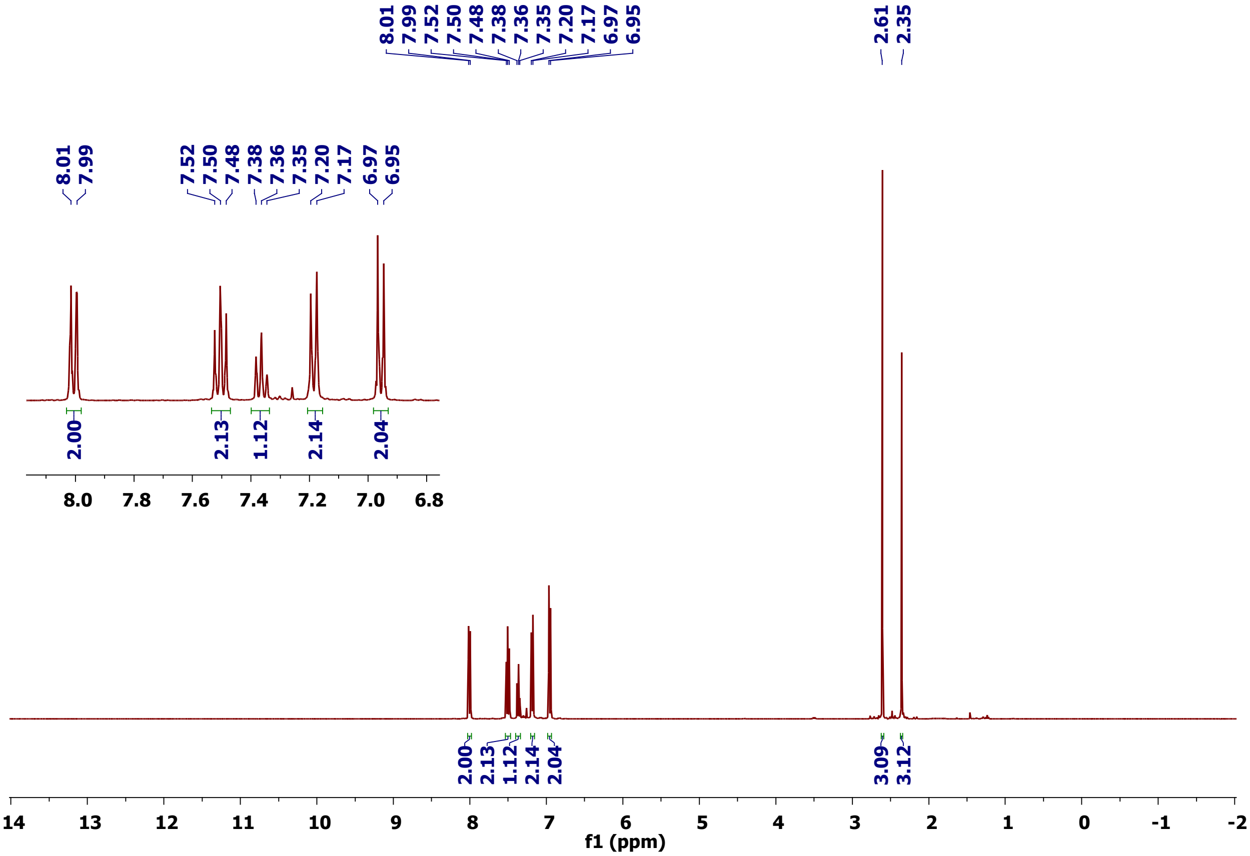

^{13}C NMR ($CDCl_3$) spectrum of (Z)-1-(4-(naphthalen-1-yl)-5-((4-nitrophenyl)imino)-4,5-dihydro-1,3,4-thiadiazol-2-yl)ethan-1-one

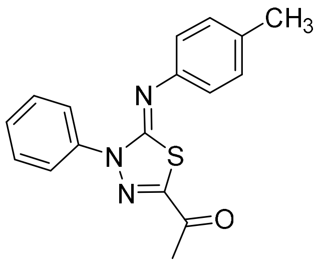

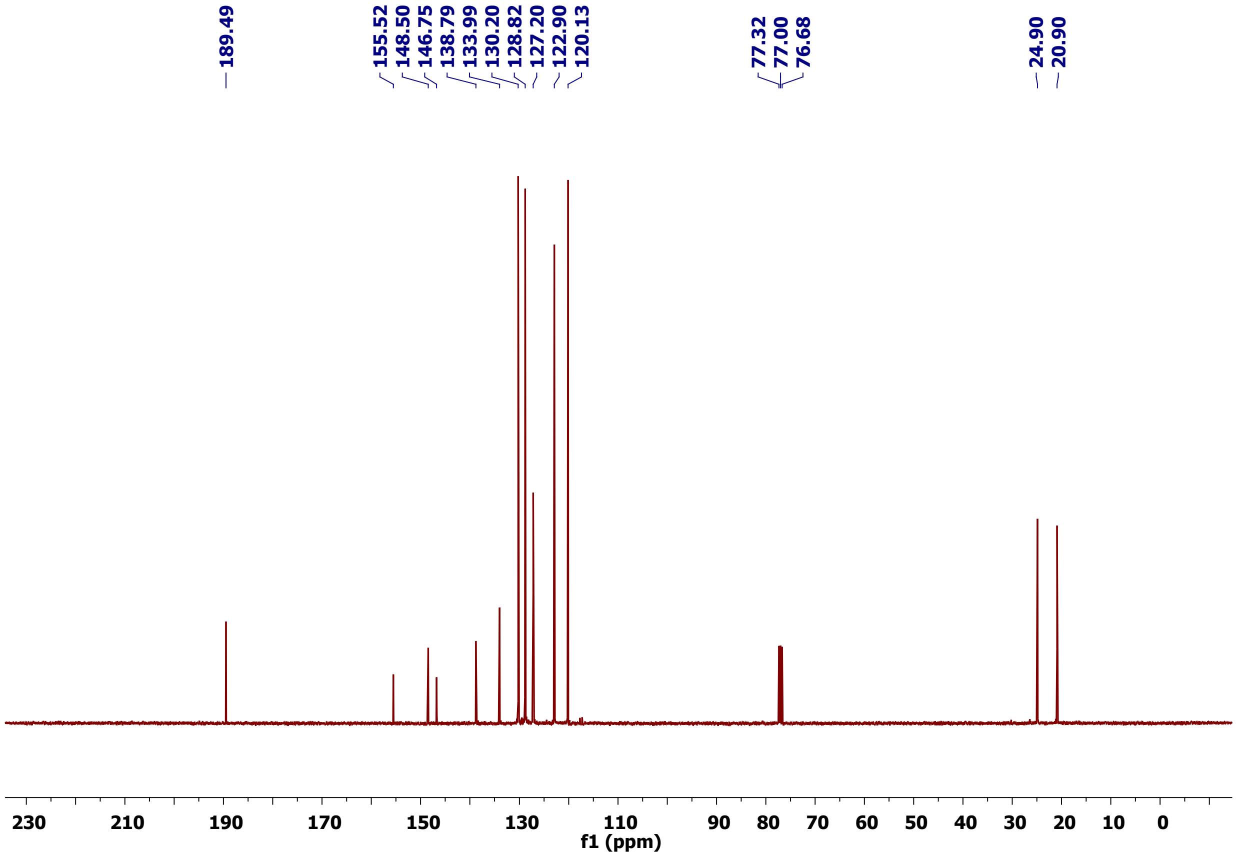

^{13}C CRAPT NMR ($CDCl_3$) spectrum of (Z)-1-(4-(naphthalen-1-yl)-5-((4-nitrophenyl)imino)-4,5-dihydro-1,3,4-thiadiazol-2-yl)ethan-1-one

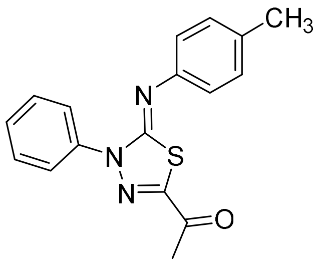

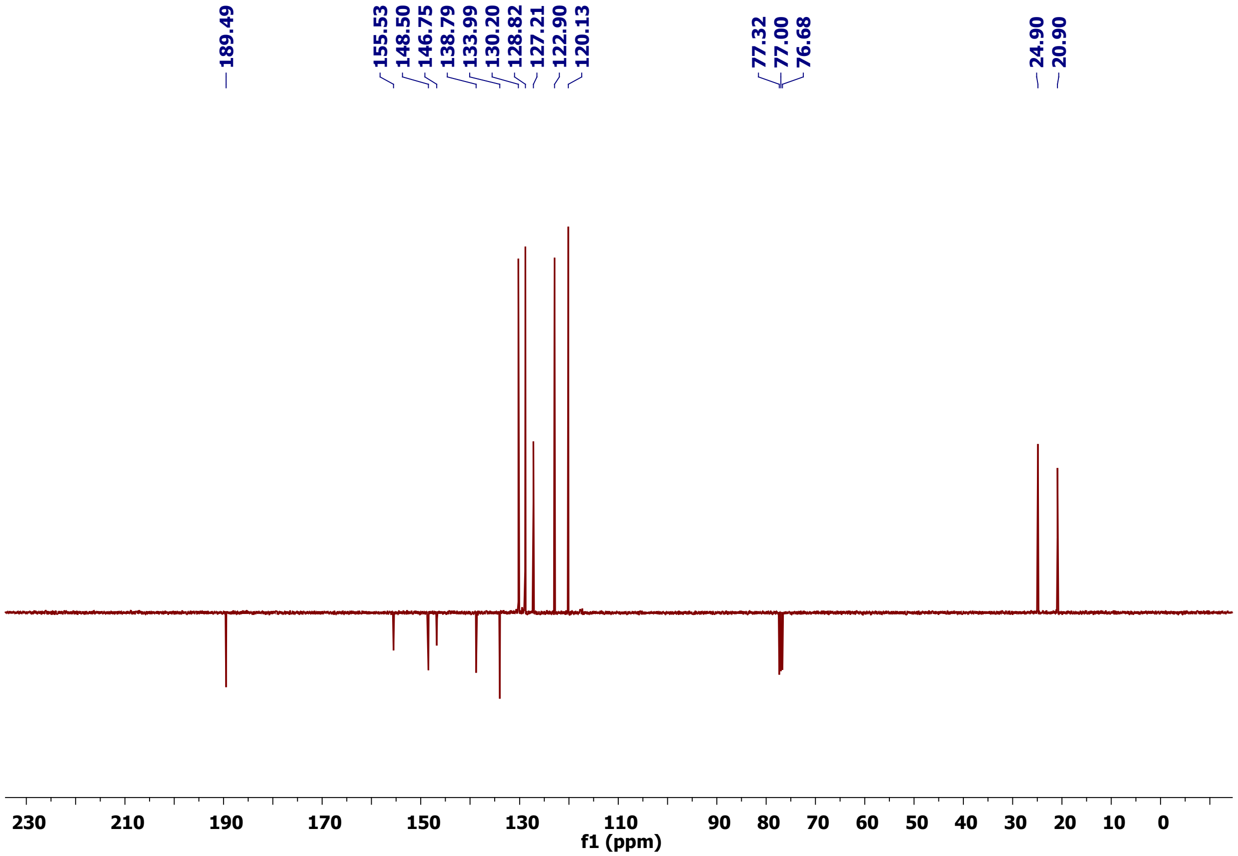

^1H-^1H gDQCOSY NMR ($CDCl_3$) spectrum of (Z)-1-(4-(naphthalen-1-yl)-5-((4-nitrophenyl)imino)-4,5-dihydro-1,3,4-thiadiazol-2-yl)ethan-1-one

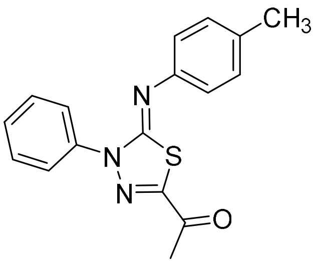

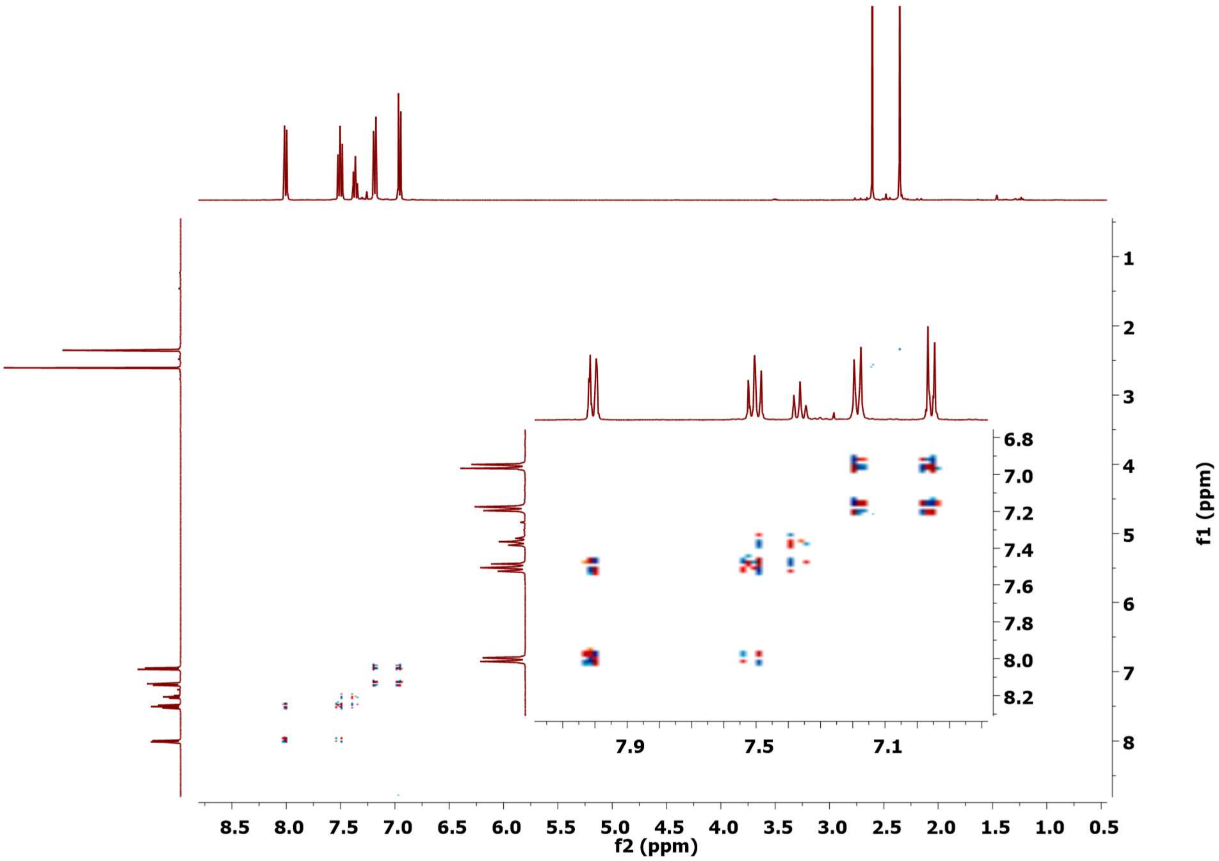

^{1}H-^{13}C-HSQC NMR ($CDCl_3$) spectrum of (Z)-1-(4-(naphthalen-1-yl)-5-((4-nitrophenyl)imino)-4,5-dihydro-1,3,4-thiadiazol-2-yl)ethan-1-one

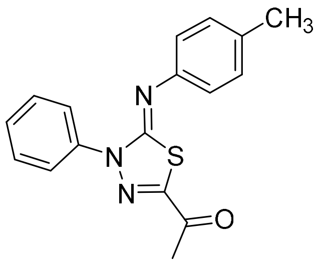

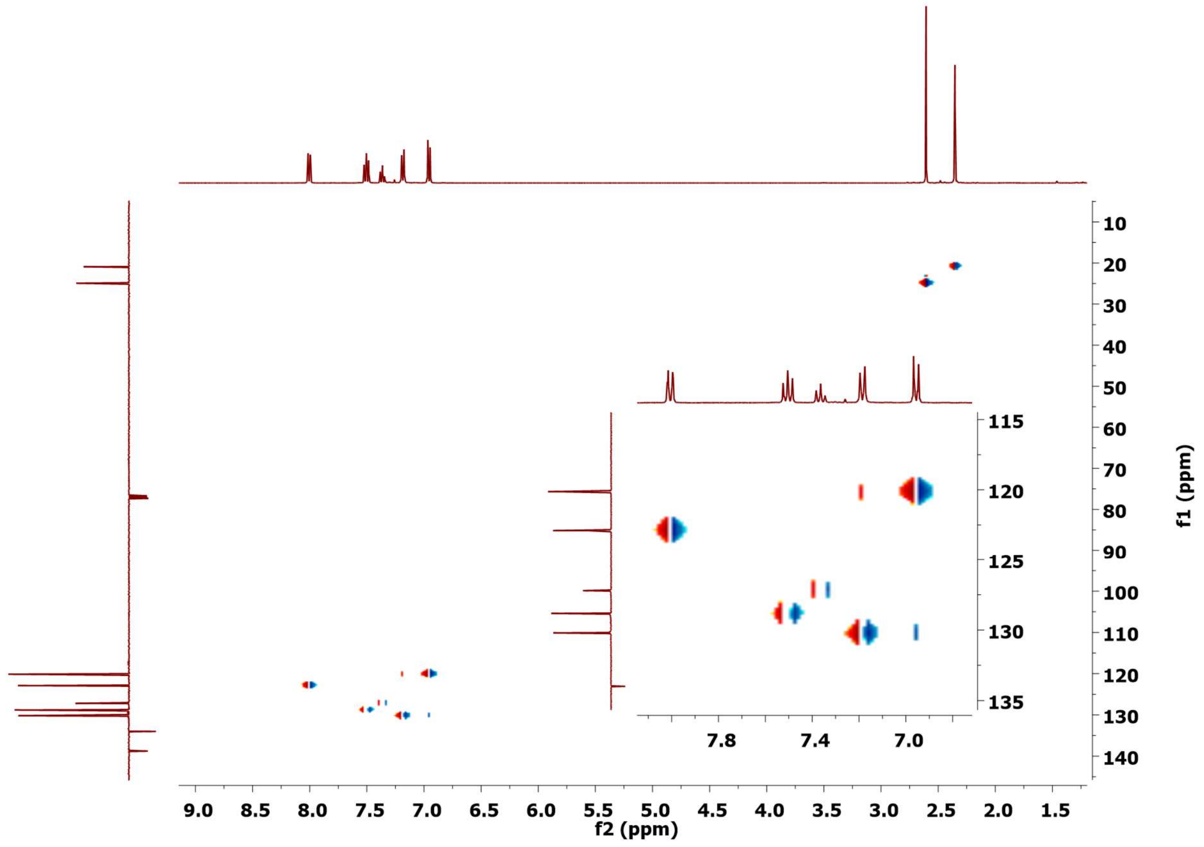

^{1}H-^{13}C-gHMBC NMR ($CDCl_3$) spectrum of (Z)-1-(4-(naphthalen-1-yl)-5-((4-nitrophenyl)imino)-4,5-dihydro-1,3,4-thiadiazol-2-yl)ethan-1-one

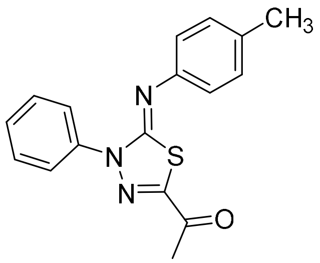

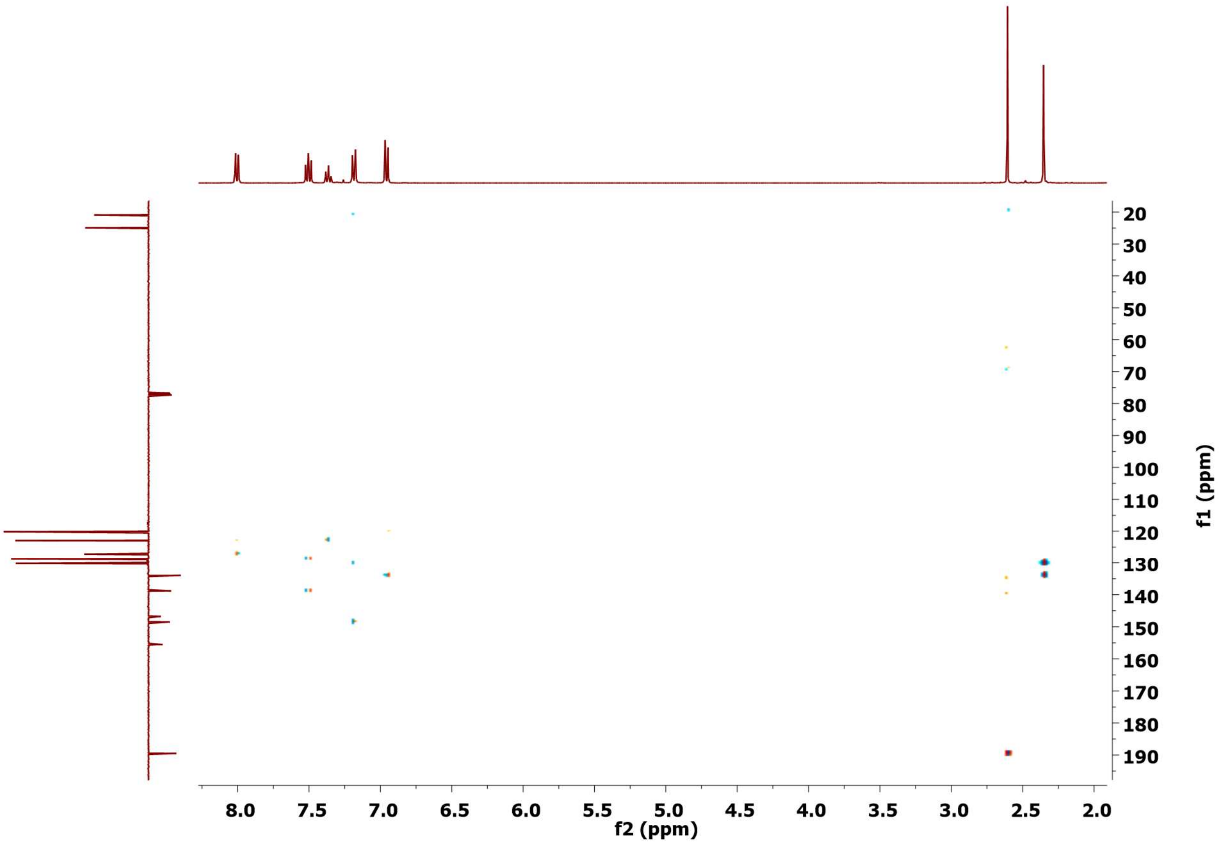

^{1}H NMR ($CDCl_3$) spectrum of (Z)-1-(5-(phenylimino)-4-(p-tolyl)-4,5-dihydro-1,3,4-thiadiazol-2-yl)ethan-1-one

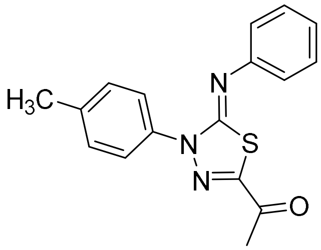

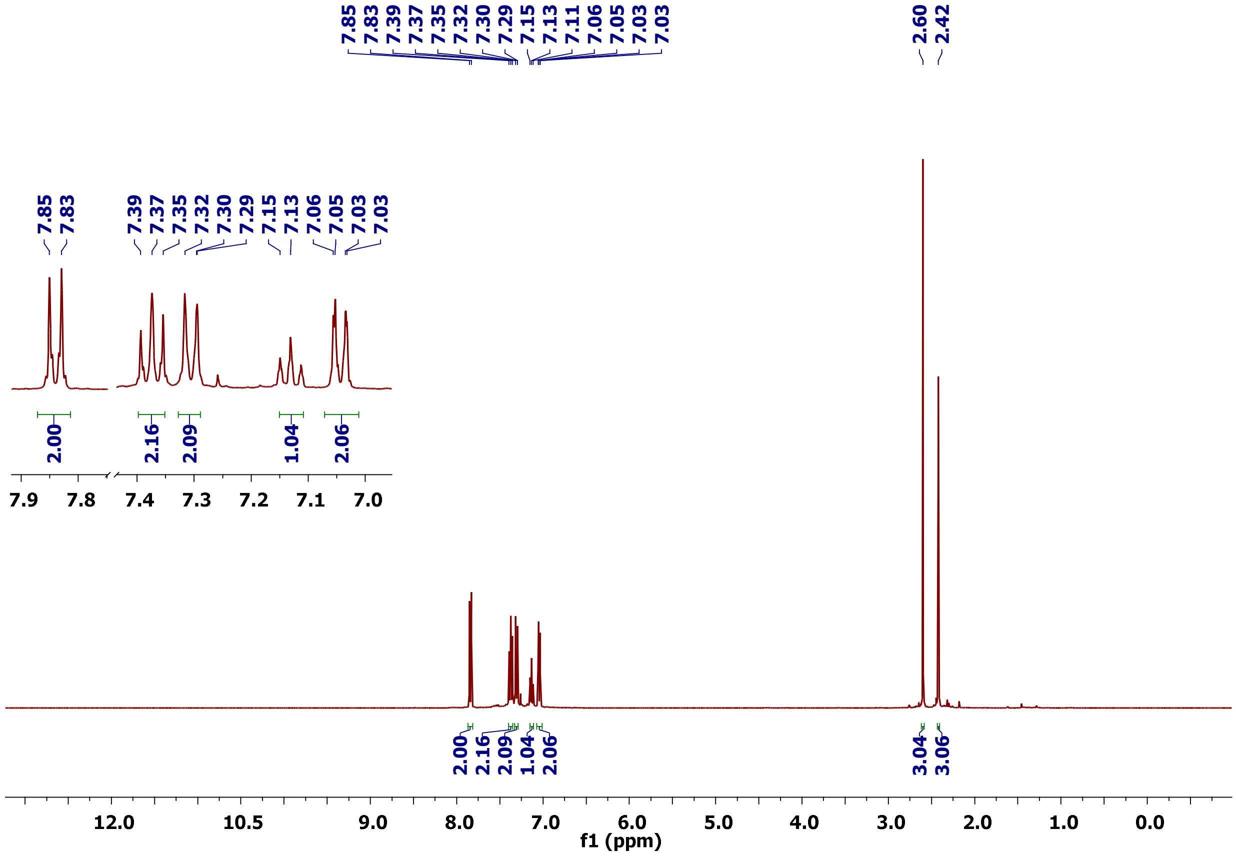

^{13}C NMR ($CDCl_3$) spectrum of (Z)-1-(5-(phenylimino)-4-(p-tolyl)-4,5-dihydro-1,3,4-thiadiazol-2-yl)ethan-1-one

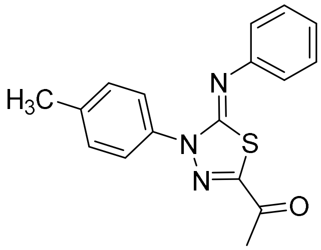

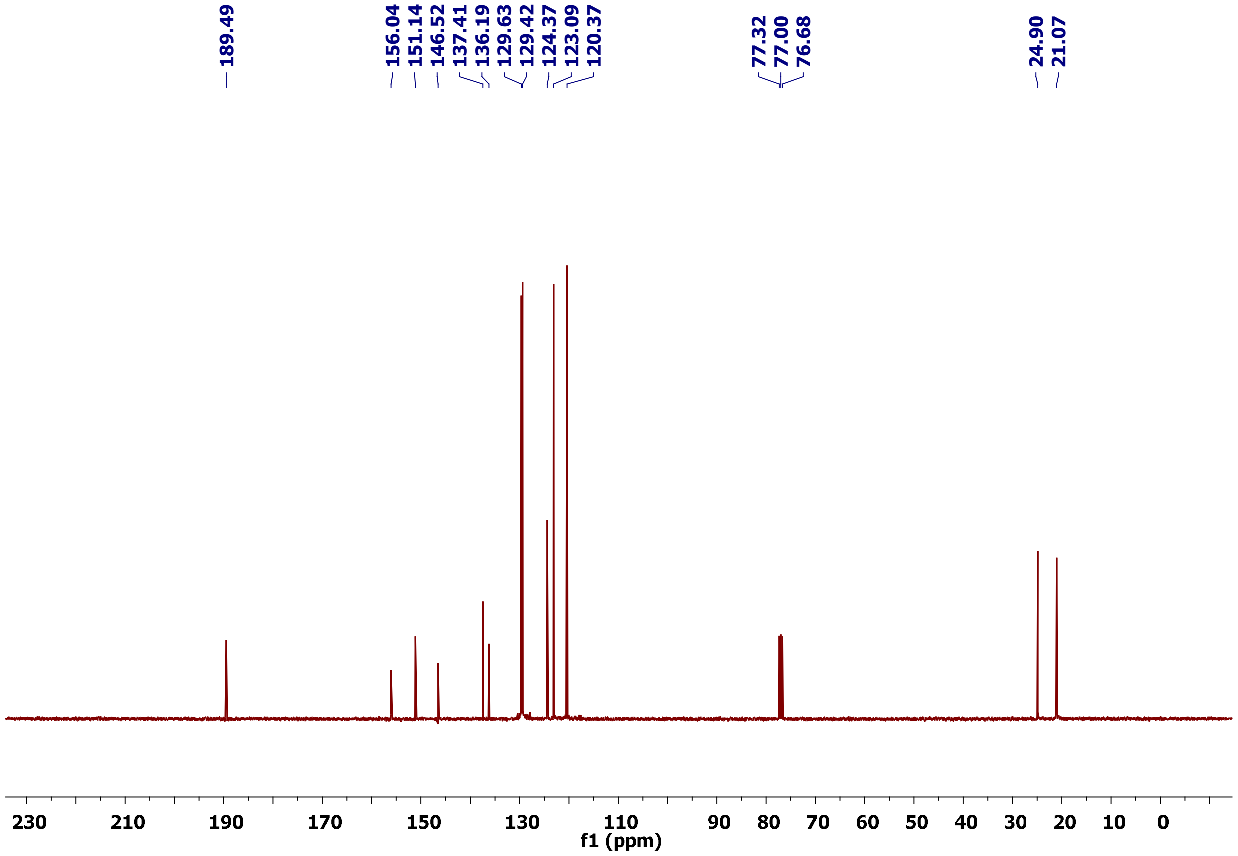

^{13}C CRAPT NMR ($CDCl_3$) spectrum of (Z)-1-(5-(phenylimino)-4-(p-tolyl)-4,5-dihydro-1,3,4-thiadiazol-2-yl)ethan-1-one

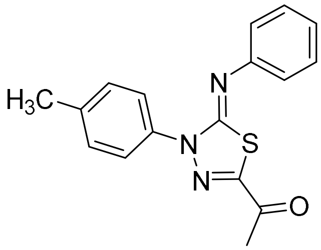

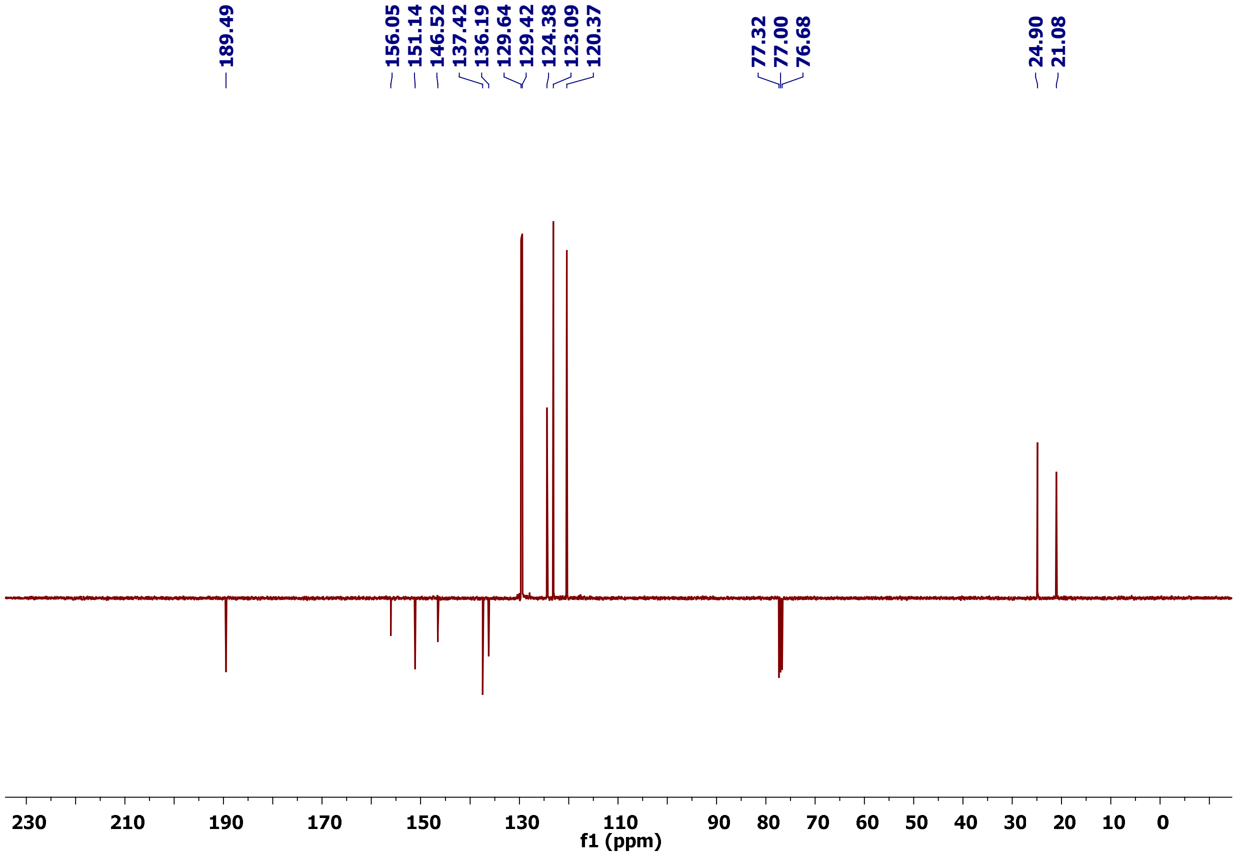

^{1}H-^{1}H gDQCOSY NMR ($CDCl_3$) spectrum of (Z)-1-(5-(phenylimino)-4-(p-tolyl)-4,5-dihydro-1,3,4-thiadiazol-2-yl)ethan-1-one

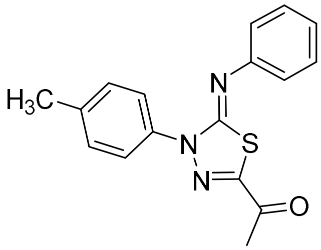

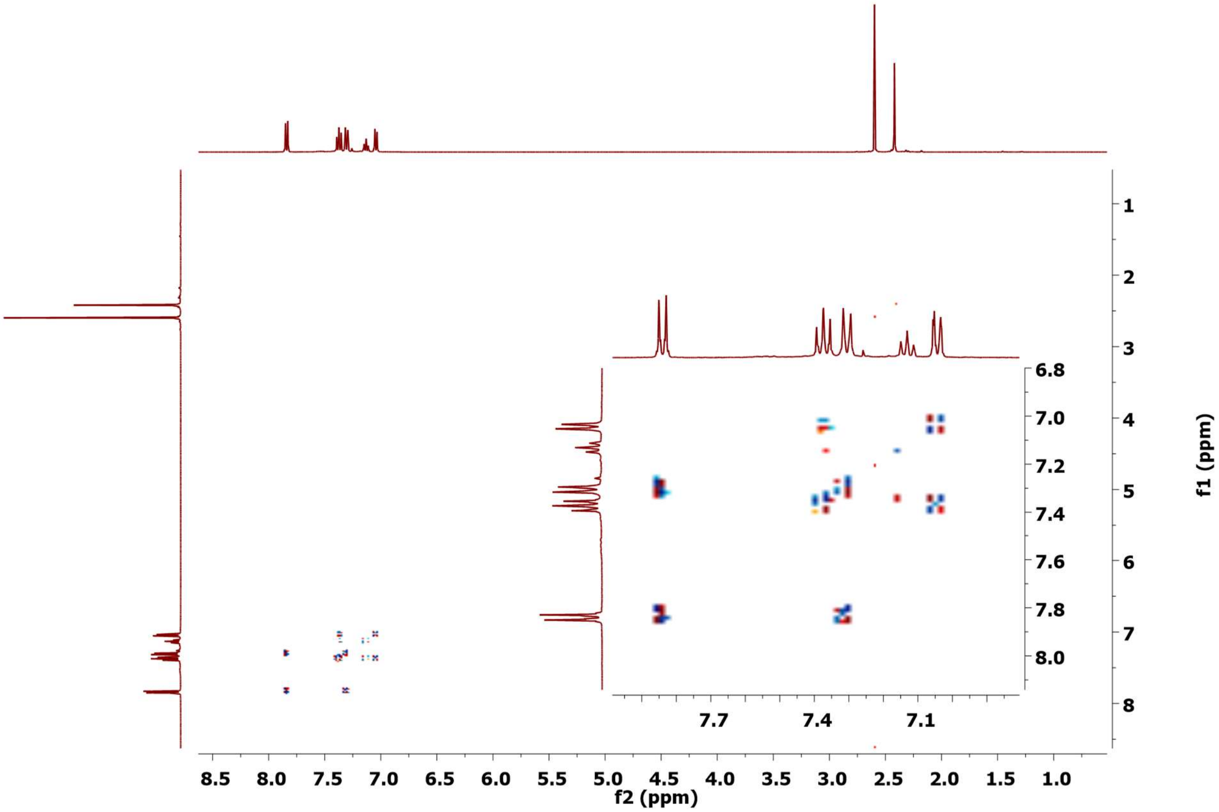

^{1}H-^{13}C-HSQC NMR ($CDCl_3$) spectrum of (Z)-1-(5-(phenylimino)-4-(p-tolyl)-4,5-dihydro-1,3,4-thiadiazol-2-yl)ethan-1-one

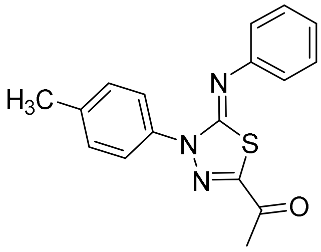

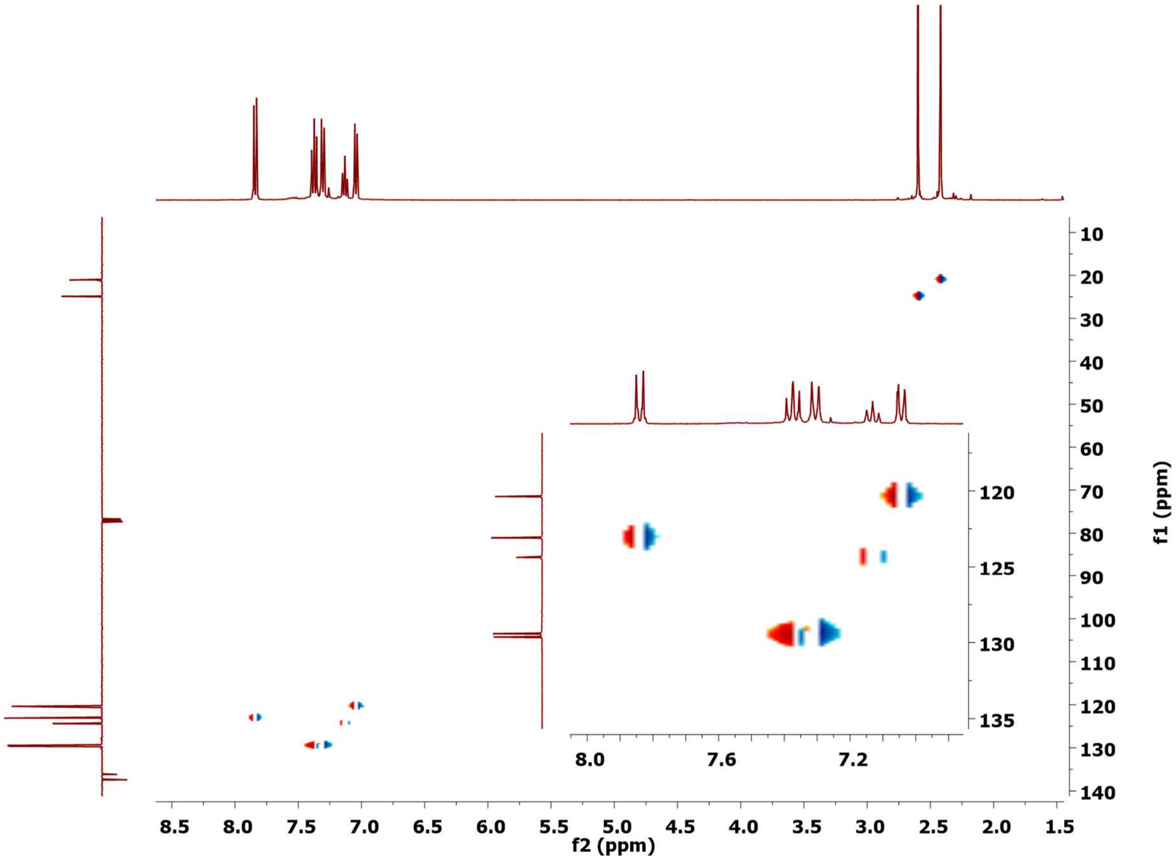

^{1}H-^{13}C-gHMBC NMR ($CDCl_3$) spectrum of (Z)-1-(5-(phenylimino)-4-(p-tolyl)-4,5-dihydro-1,3,4-thiadiazol-2-yl)ethan-1-one

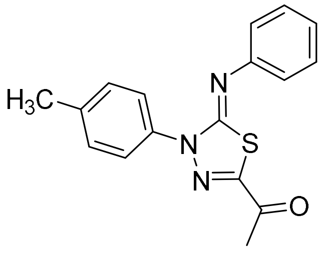

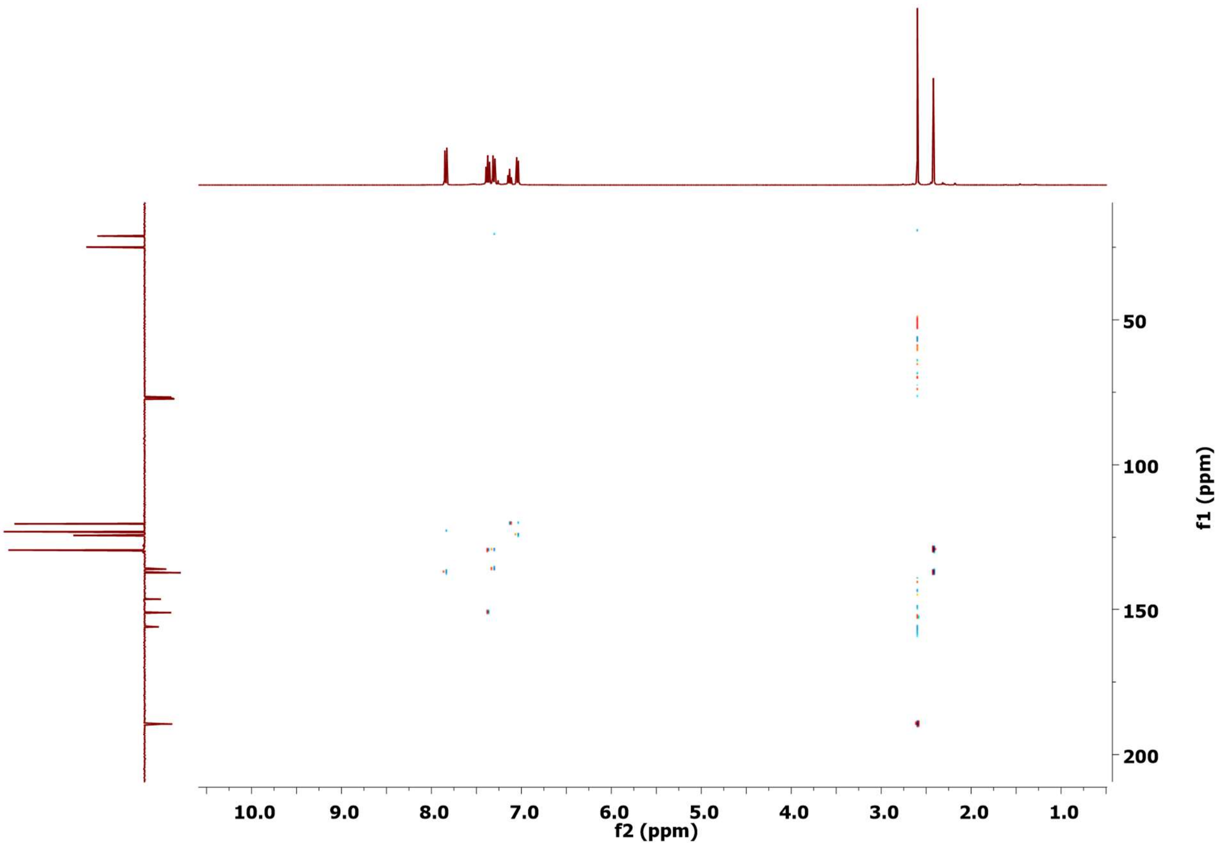

^{1}H NMR ($CDCl_3$) spectrum of (Z)-1-(4-phenyl-5-(phenylimino)-4,5-dihydro-1,3,4-thiadiazol-2-yl)ethan-1-one

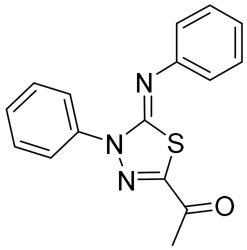

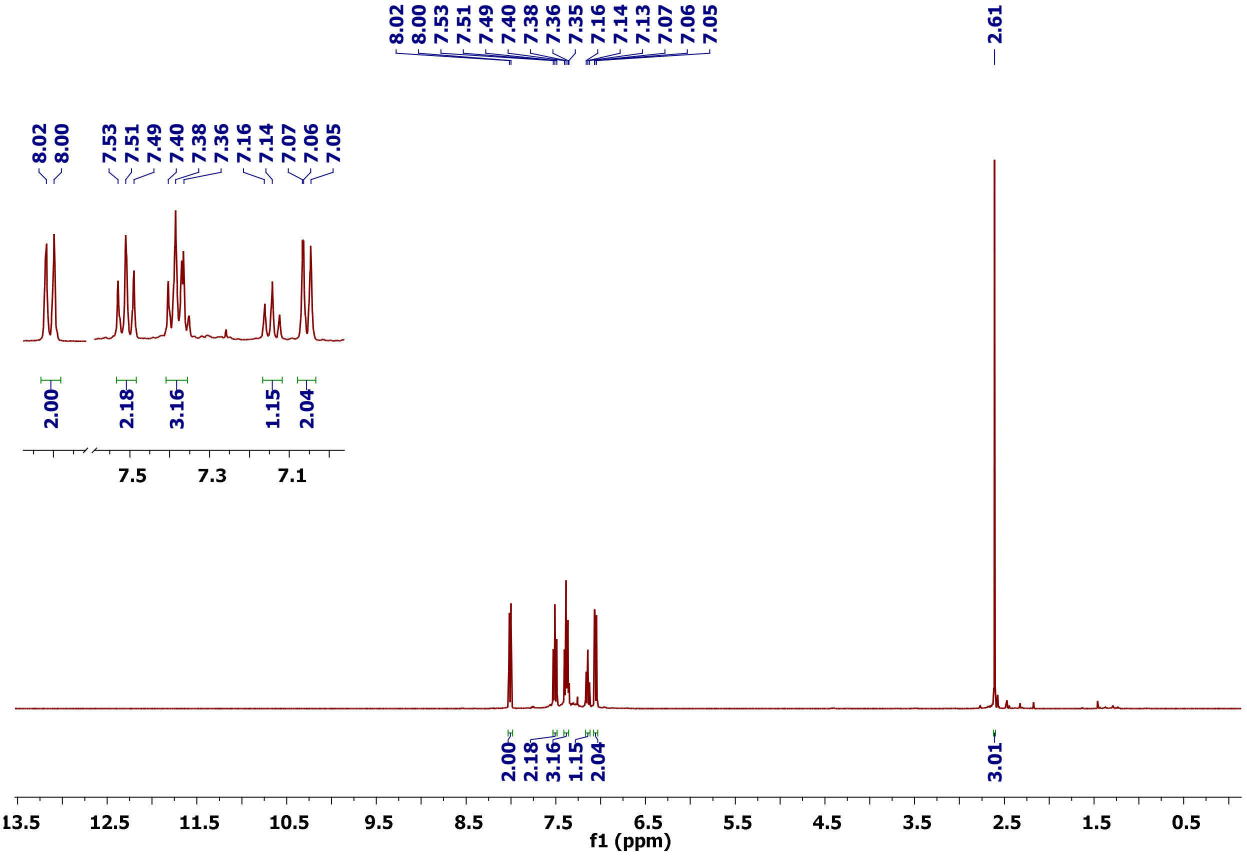

^{13}C NMR ($CDCl_3$) spectrum of (Z)-1-(4-phenyl-5-(phenylimino)-4,5-dihydro-1,3,4-thiadiazol-2-yl)ethan-1-one

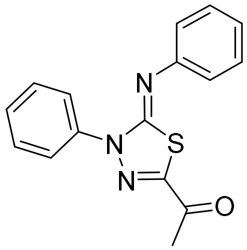

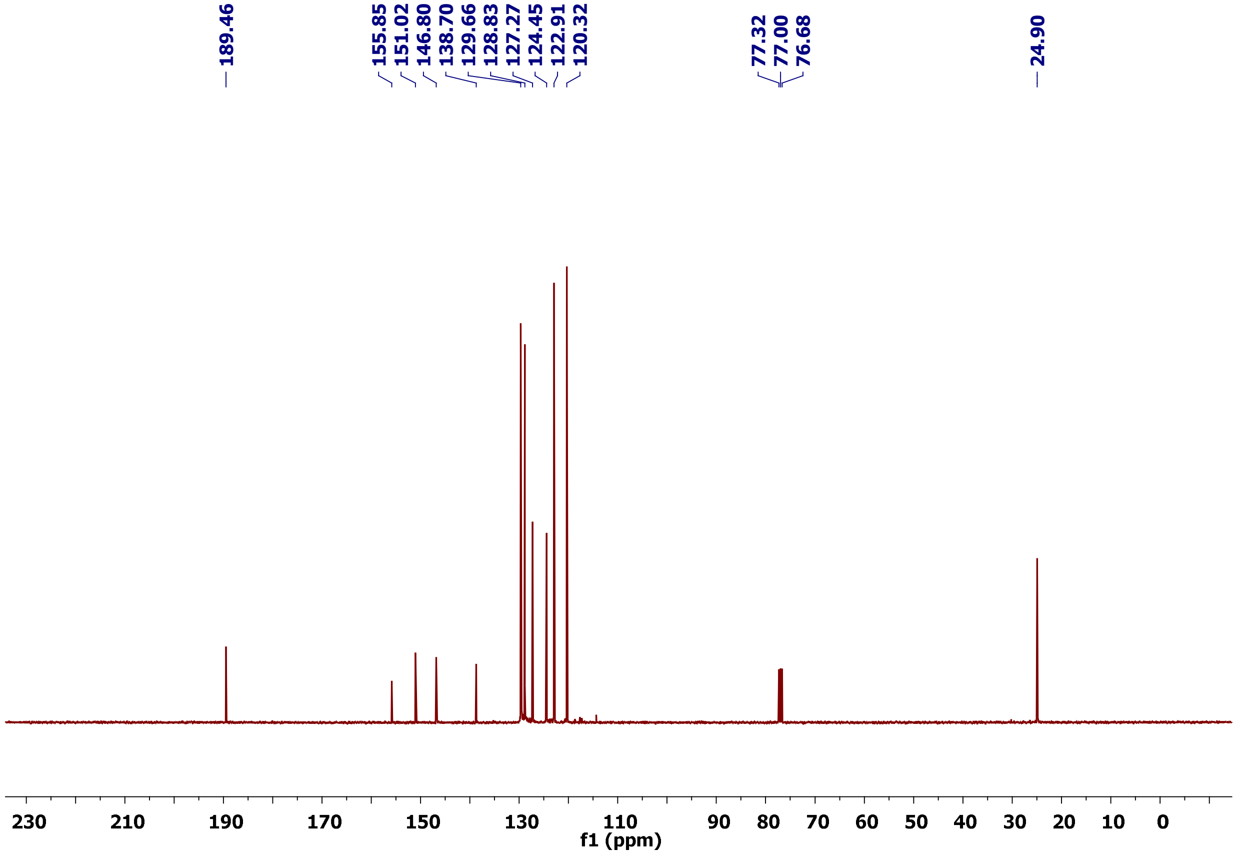

^{13}C CRAPT NMR ($CDCl_3$) spectrum of (Z)-1-(4-phenyl-5-(phenylimino)-4,5-dihydro-1,3,4-thiadiazol-2-yl)ethan-1-one

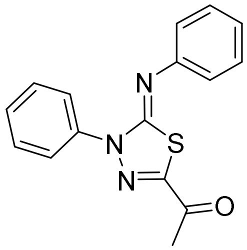

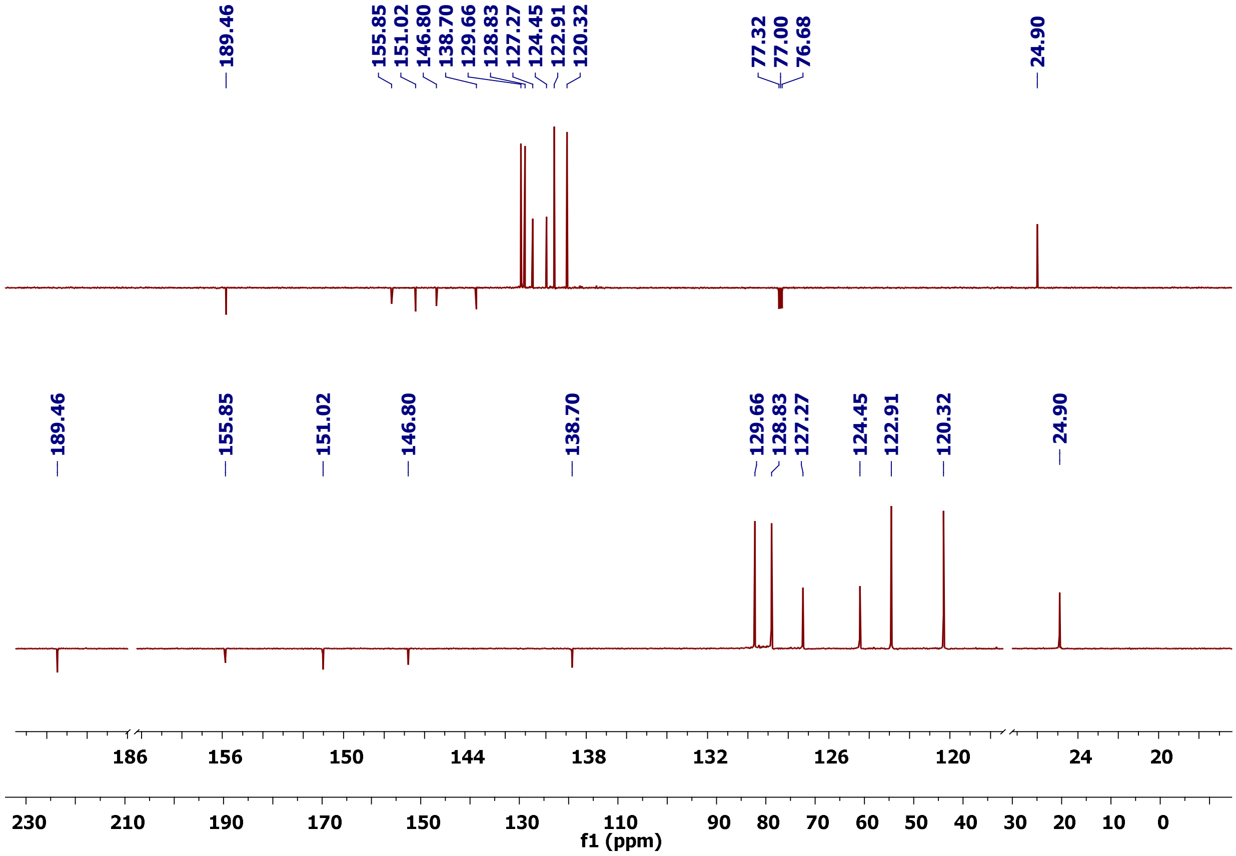

^{1}H-^{1}H gDQCOSY NMR ($CDCl_3$) spectrum of (Z)-1-(4-phenyl-5-(phenylimino)-4,5-dihydro-1,3,4-thiadiazol-2-yl)ethan-1-one

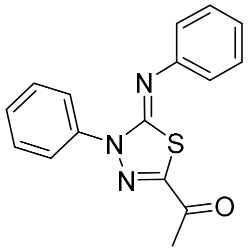

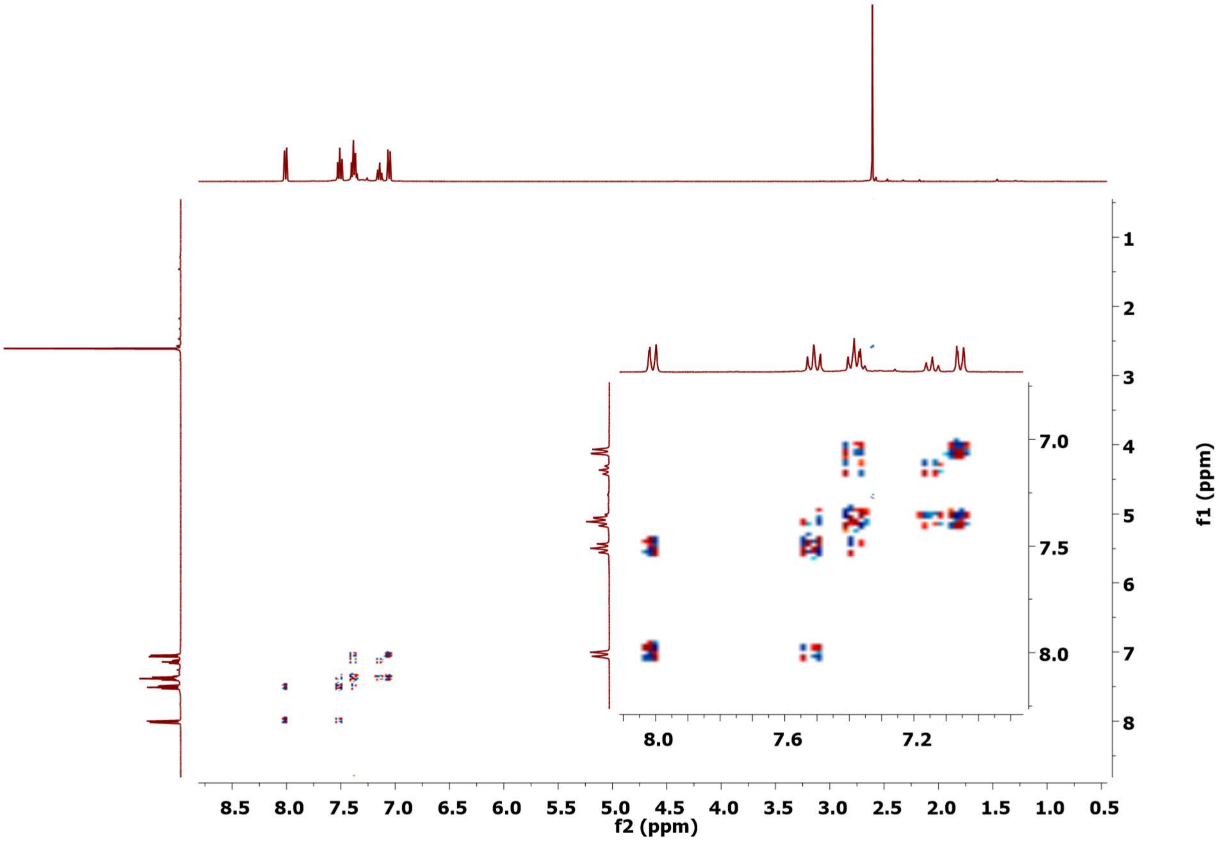

^{1}H-^{13}C-HSQC NMR ($CDCl_3$) spectrum of (Z)-1-(4-phenyl-5-(phenylimino)-4,5-dihydro-1,3,4-thiadiazol-2-yl)ethan-1-one

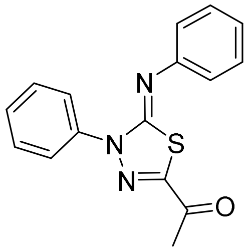

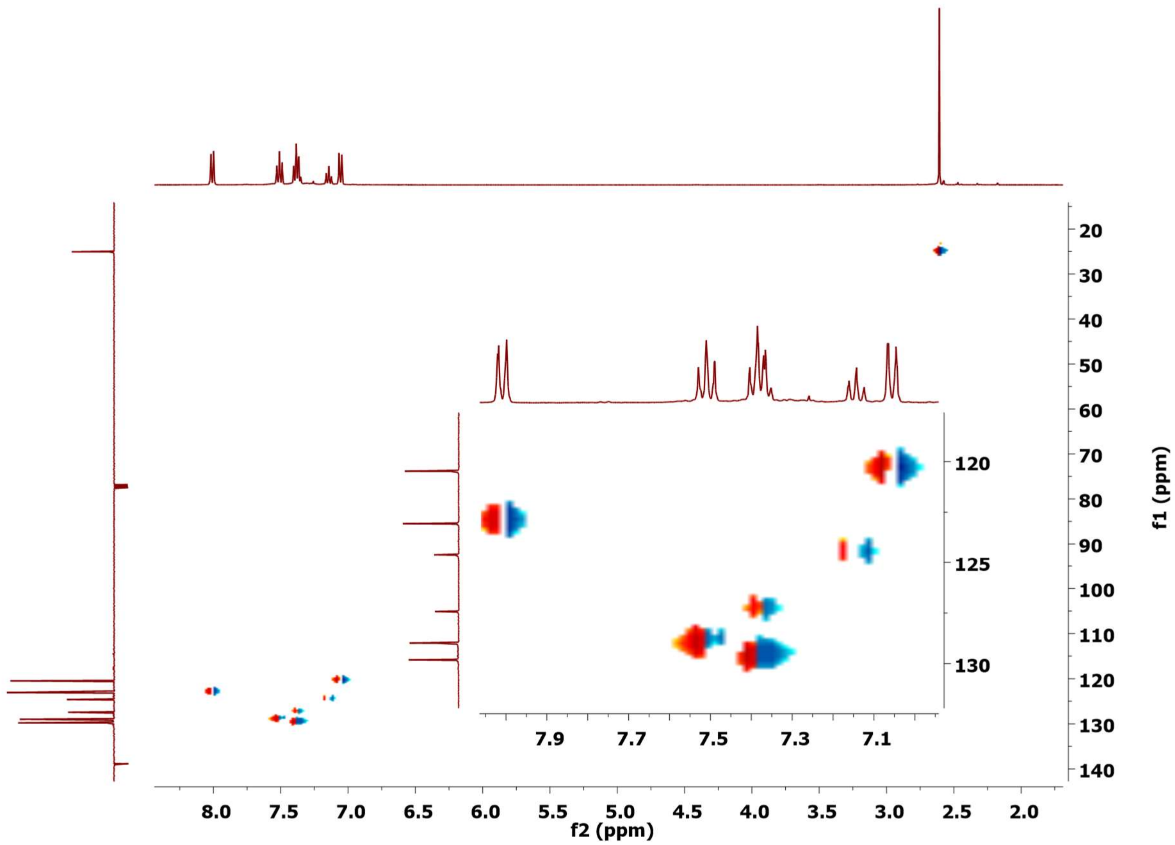

^{1}H-^{13}C-gHMBC NMR ($CDCl_3$) spectrum of (Z)-1-(4-phenyl-5-(phenylimino)-4,5-dihydro-1,3,4-thiadiazol-2-yl)ethan-1-one

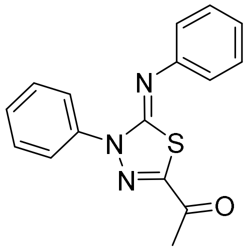

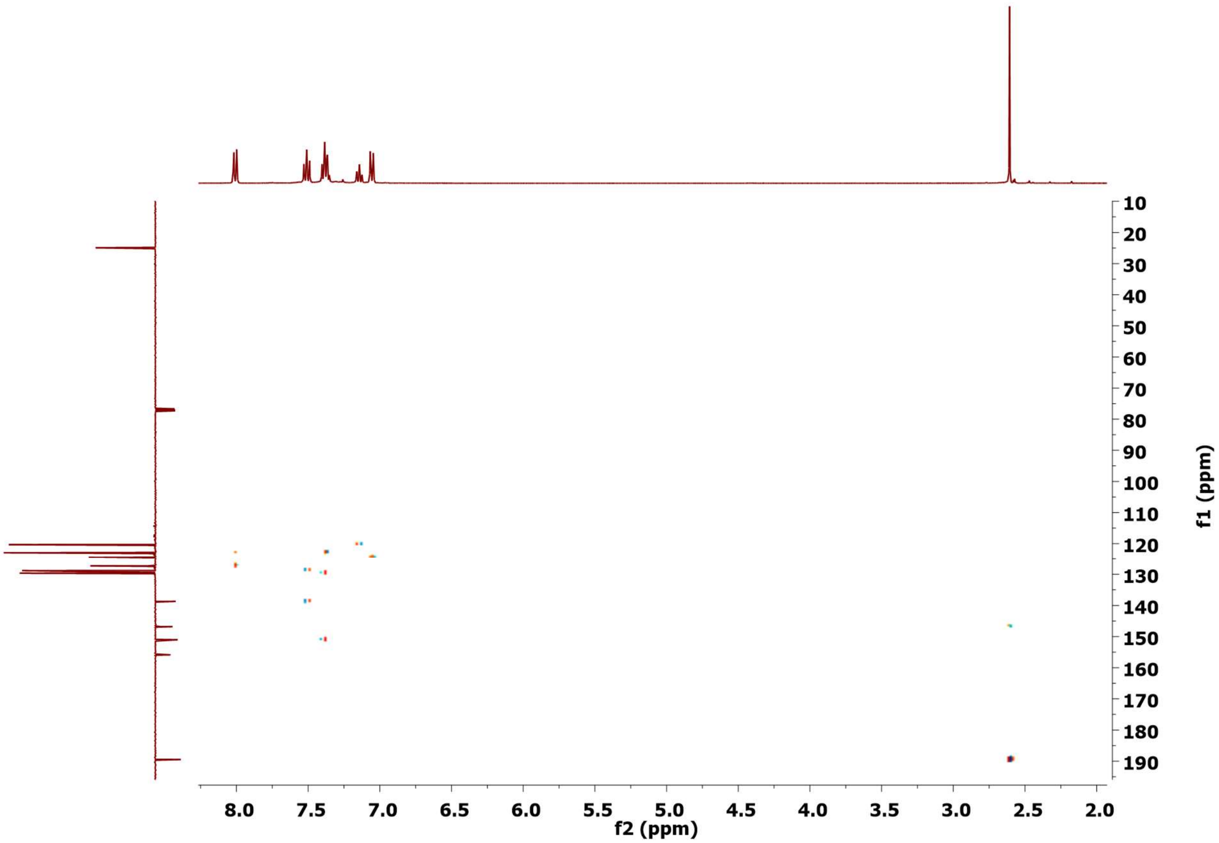

^{1}H NMR ($CDCl_3$) spectrum of (Z)-1-(5-((4-ethylphenyl)imino)-4-(p-tolyl)-4,5-dihydro-1,3,4-thiadiazol-2-yl)ethan-1-one

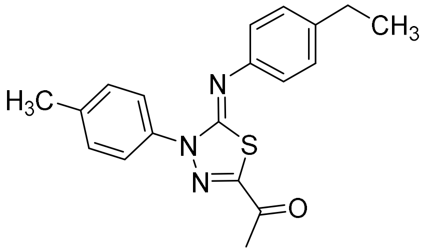

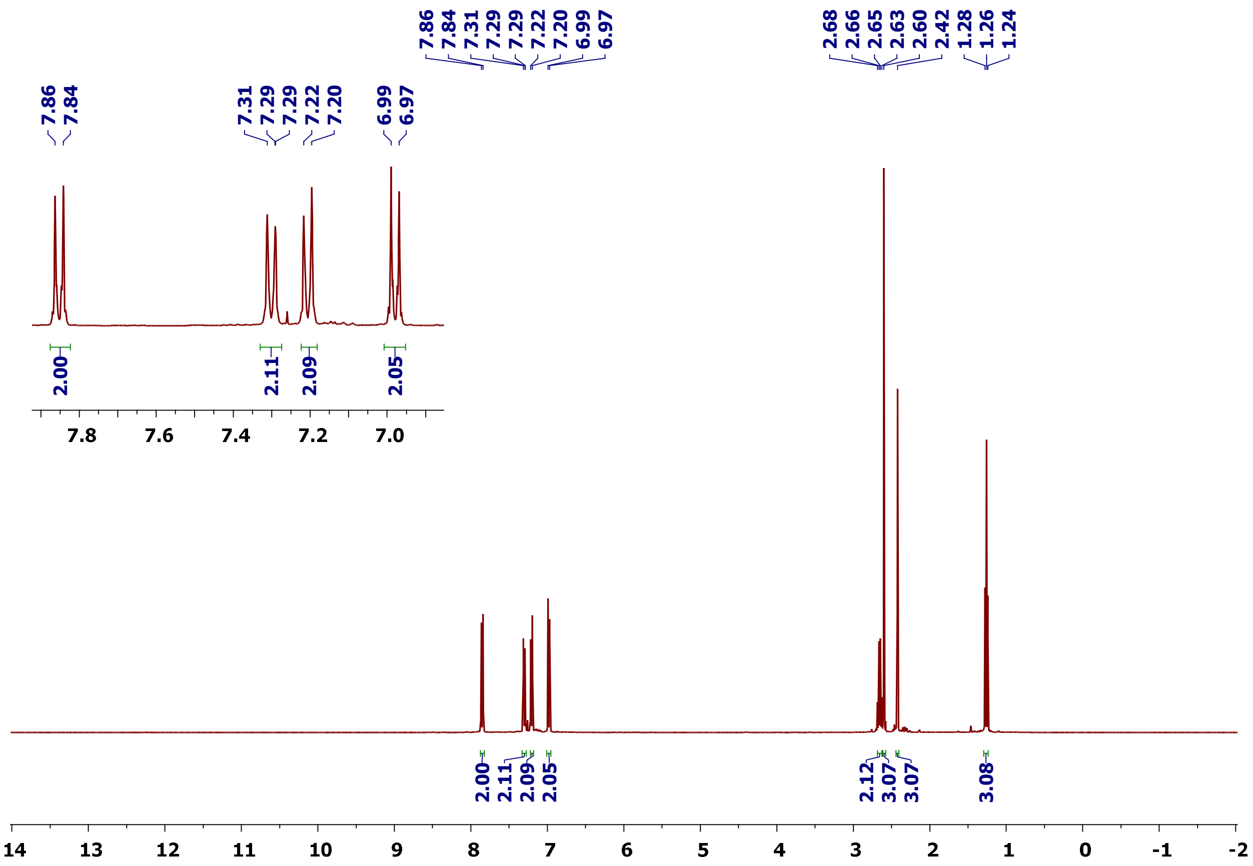

^{13}C NMR ($CDCl_3$) spectrum of (Z)-1-(5-((4-ethylphenyl)imino)-4-(p-tolyl)-4,5-dihydro-1,3,4-thiadiazol-2-yl)ethan-1-one

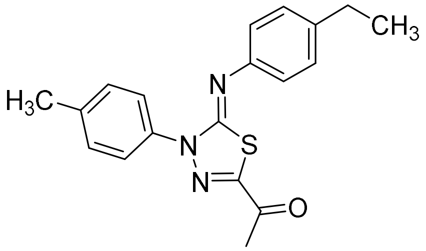

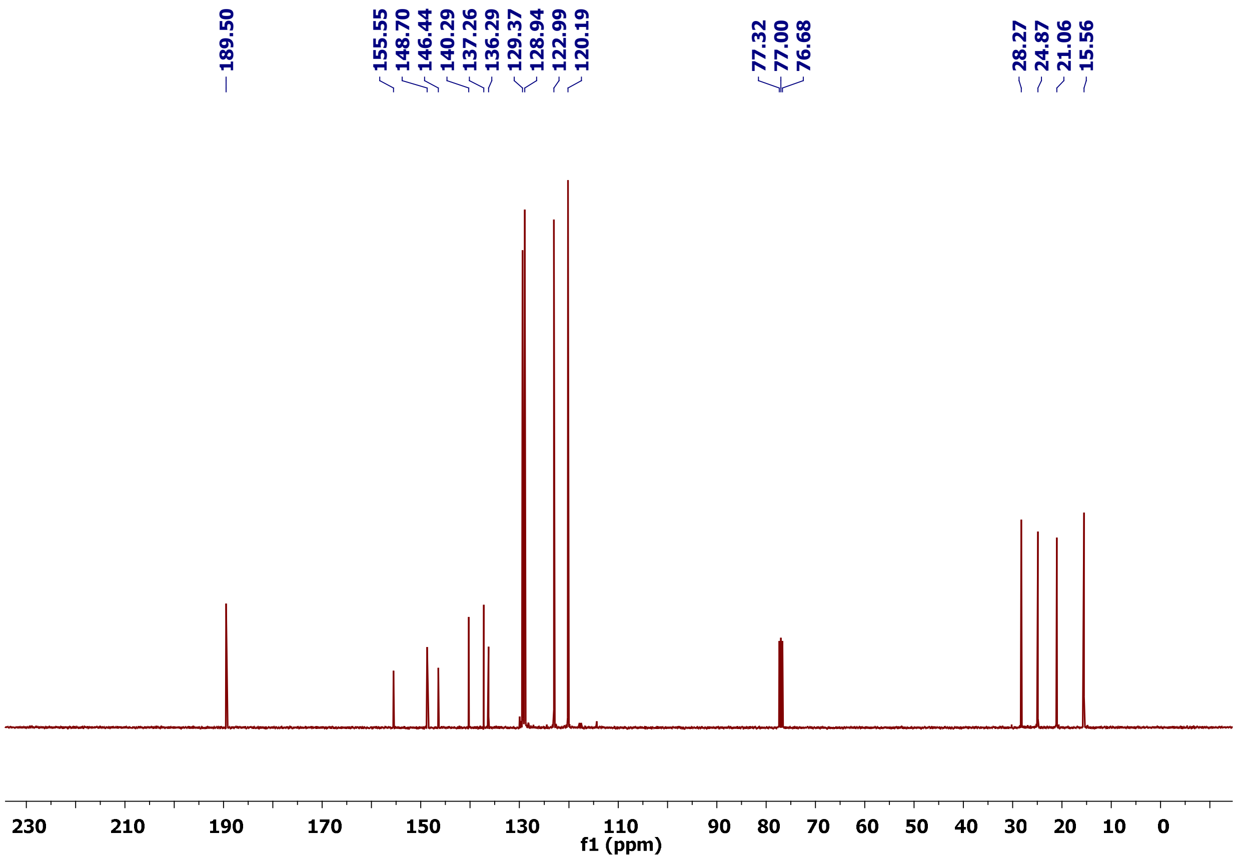

^{13}C CRAPT NMR ($CDCl_3$) spectrum of (Z)-1-(5-((4-ethylphenyl)imino)-4-(p-tolyl)-4,5-dihydro-1,3,4-thiadiazol-2-yl)ethan-1-one

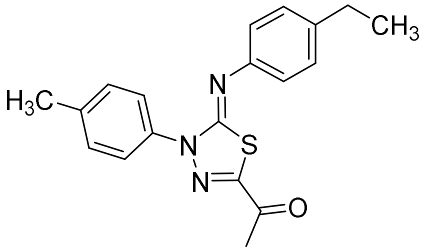

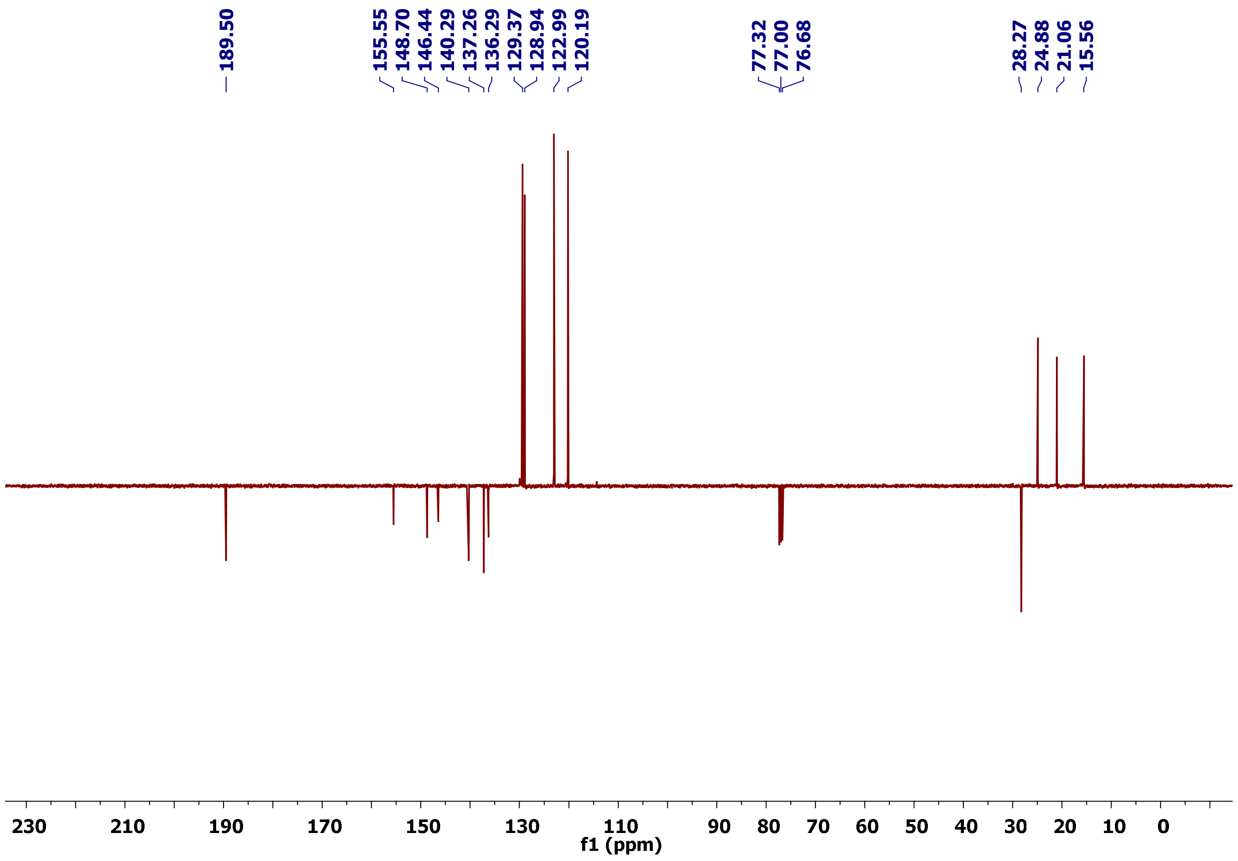

^{1}H-^{1}H gDQCOSY NMR ($CDCl_3$) spectrum of (Z)-1-(5-((4-ethylphenyl)imino)-4-(p-tolyl)-4,5-dihydro-1,3,4-thiadiazol-2-yl)ethan-1-one

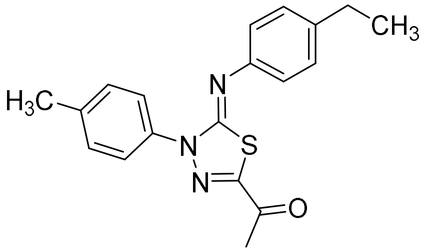

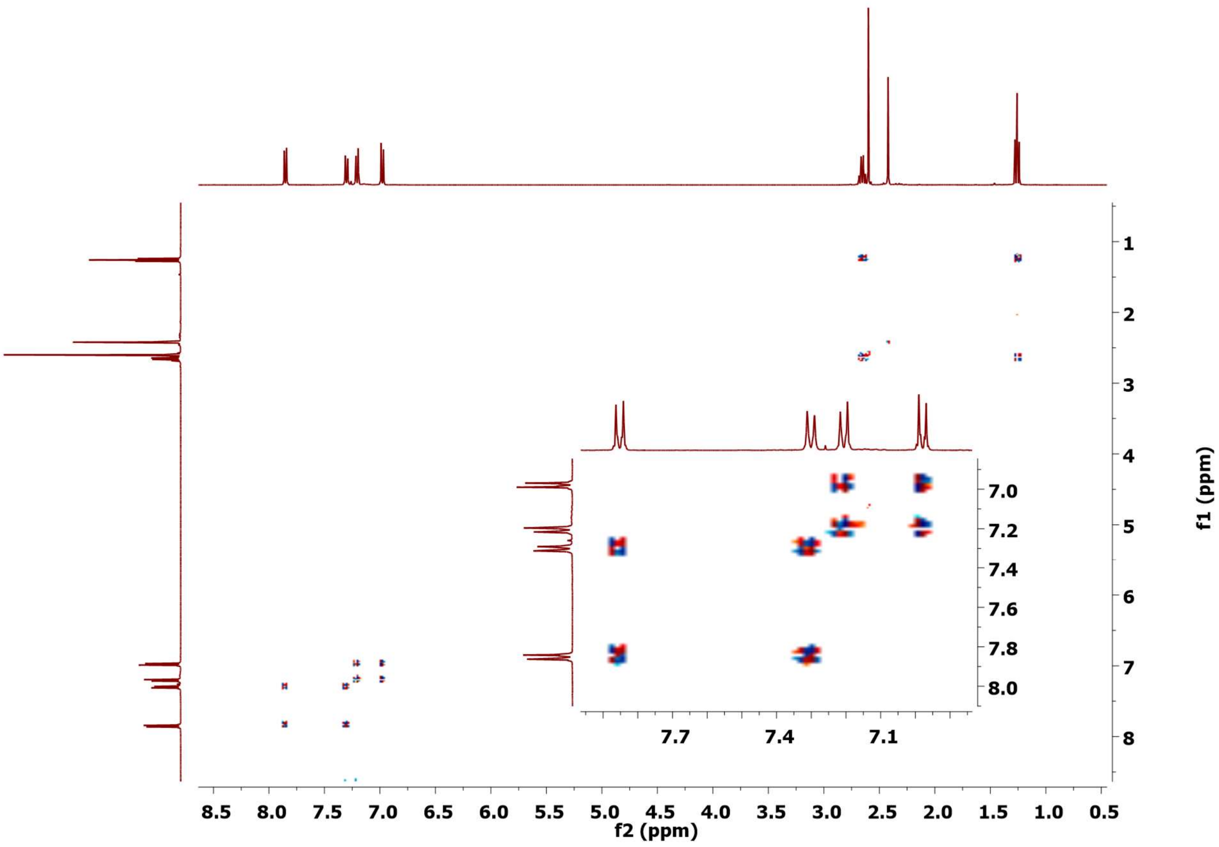

^{1}H-^{13}C-HSQC NMR ($CDCl_3$) spectrum of (Z)-1-(5-((4-ethylphenyl)imino)-4-(p-tolyl)-4,5-dihydro-1,3,4-thiadiazol-2-yl)ethan-1-one

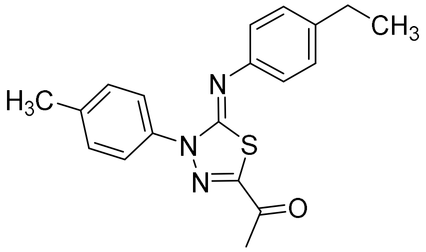

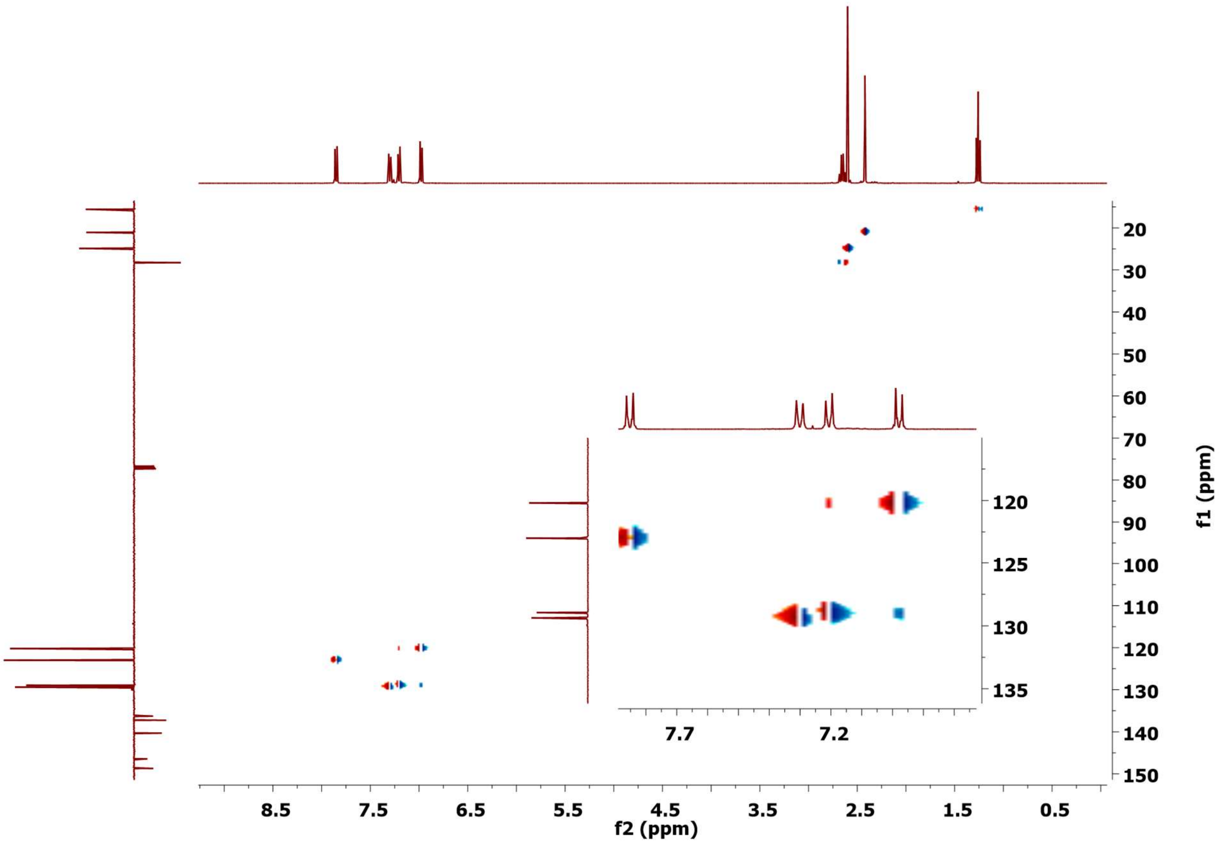

^{1}H-^{13}C-gHMBC NMR ($CDCl_3$) spectrum of (Z)-1-(5-((4-ethylphenyl)imino)-4-(p-tolyl)-4,5-dihydro-1,3,4-thiadiazol-2-yl)ethan-1-one

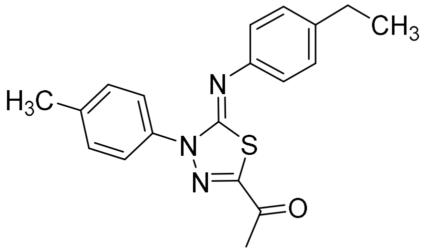

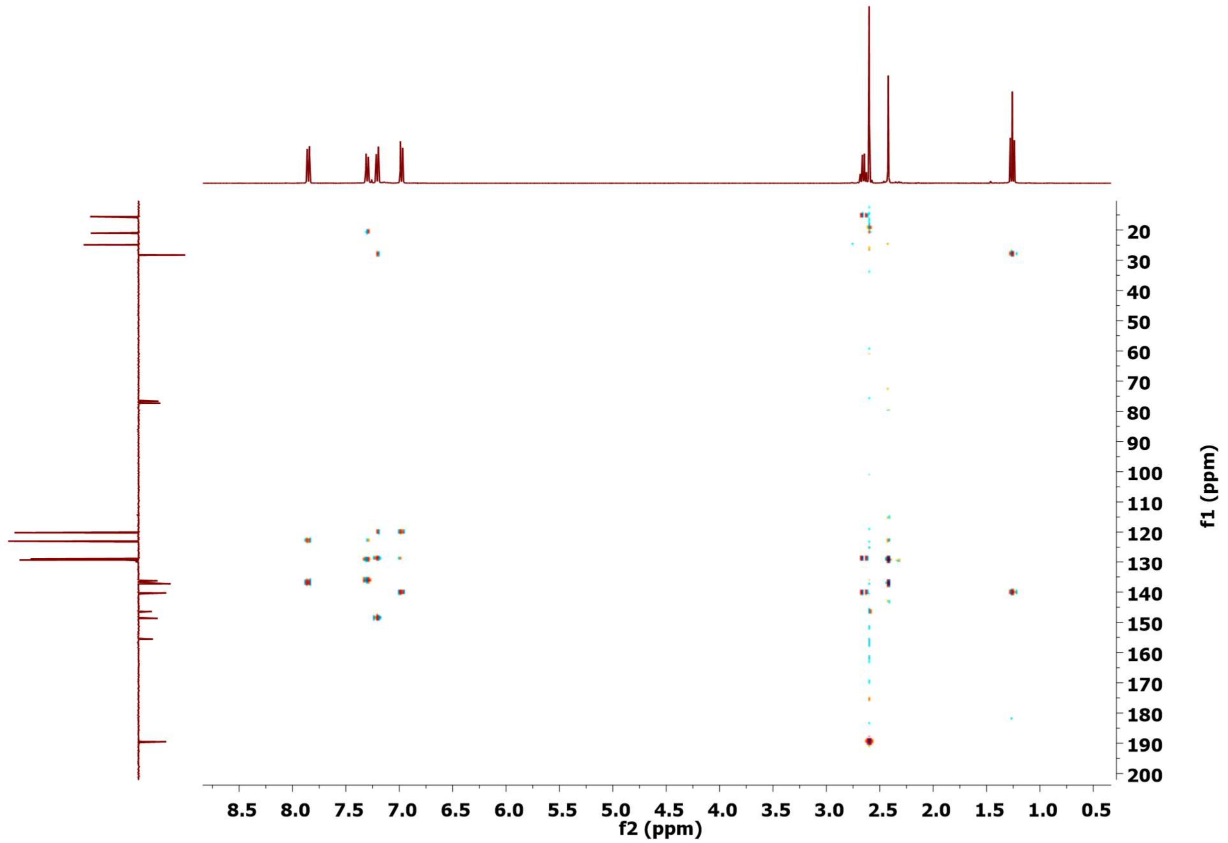

^{1}H NMR ($CDCl_3$) spectrum of (Z)-1-(4-(naphthalen-1-yl)-5-(naphthalen-1-ylimino)-4,5-dihydro-1,3,4-thiadiazol-2-yl)ethan-1-one

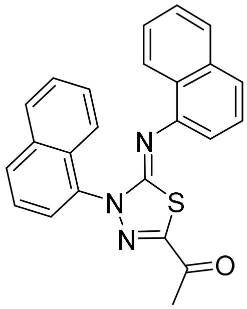

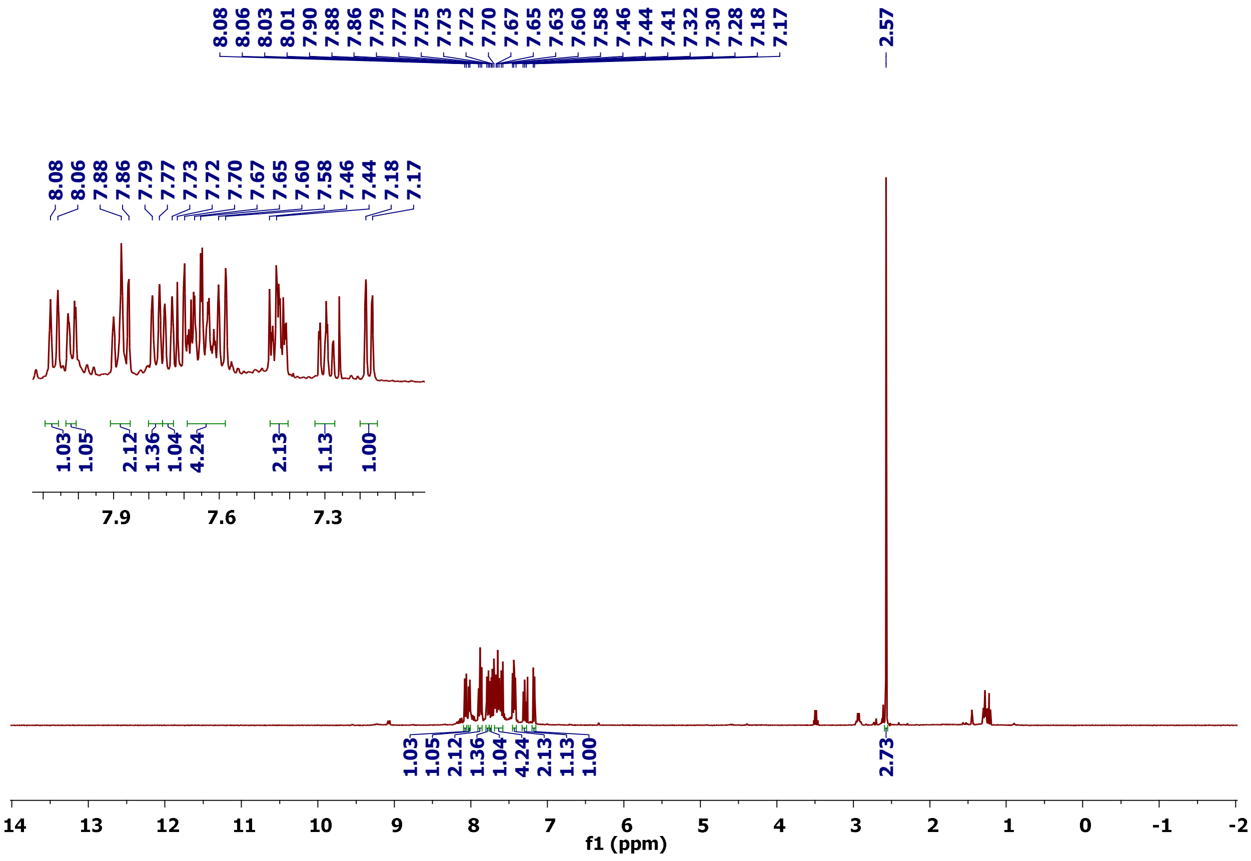

^{13}C NMR ($CDCl_3$) spectrum of (Z)-1-(4-(naphthalen-1-yl)-5-(naphthalen-1-ylimino)-4,5-dihydro-1,3,4-thiadiazol-2-yl)ethan-1-one

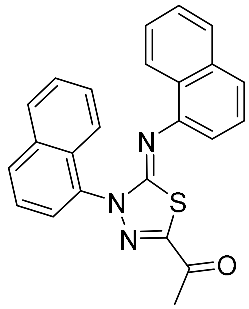

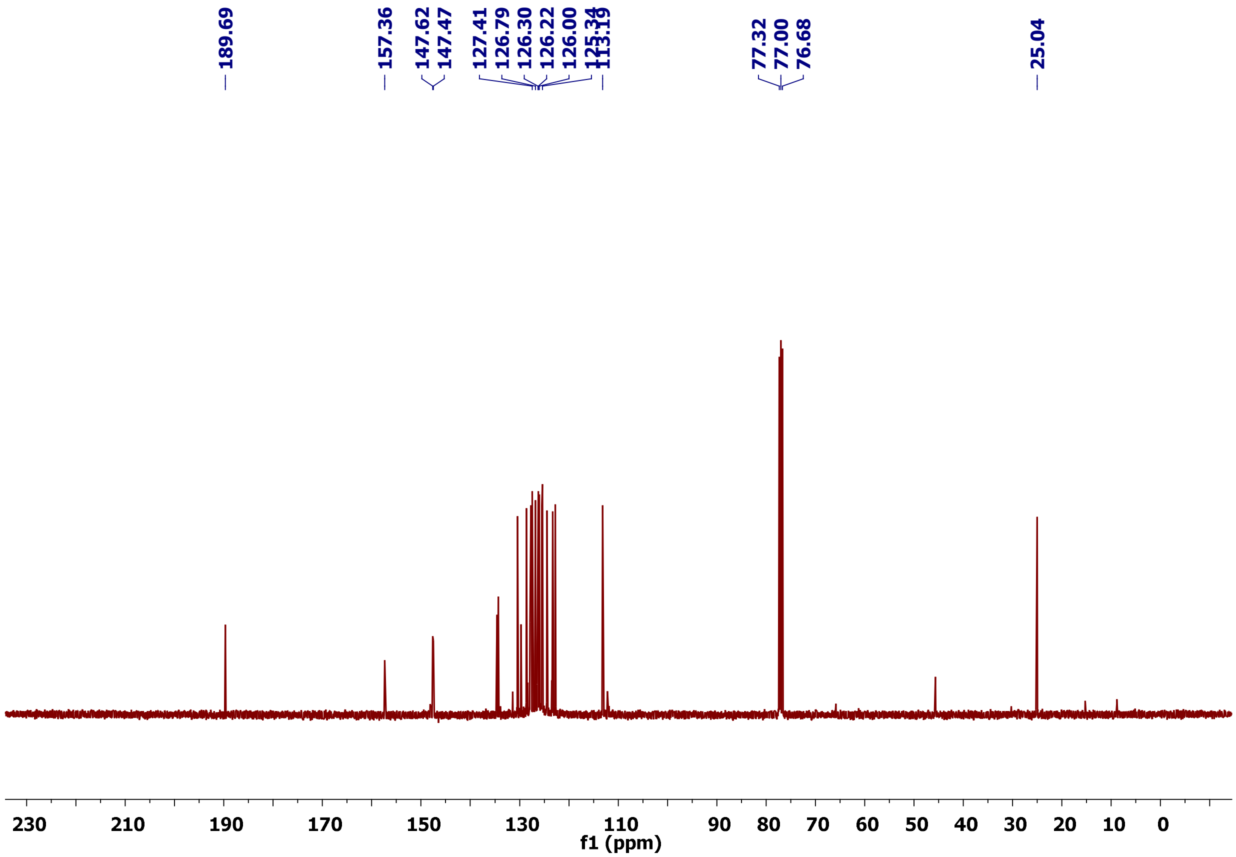

^{13}C CRAPT NMR ($CDCl_3$) spectrum of (Z)-1-(4-(naphthalen-1-yl)-5-(naphthalen-1-ylimino)-4,5-dihydro-1,3,4-thiadiazol-2-yl)ethan-1-one

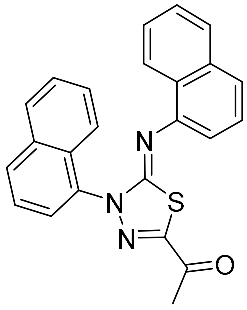

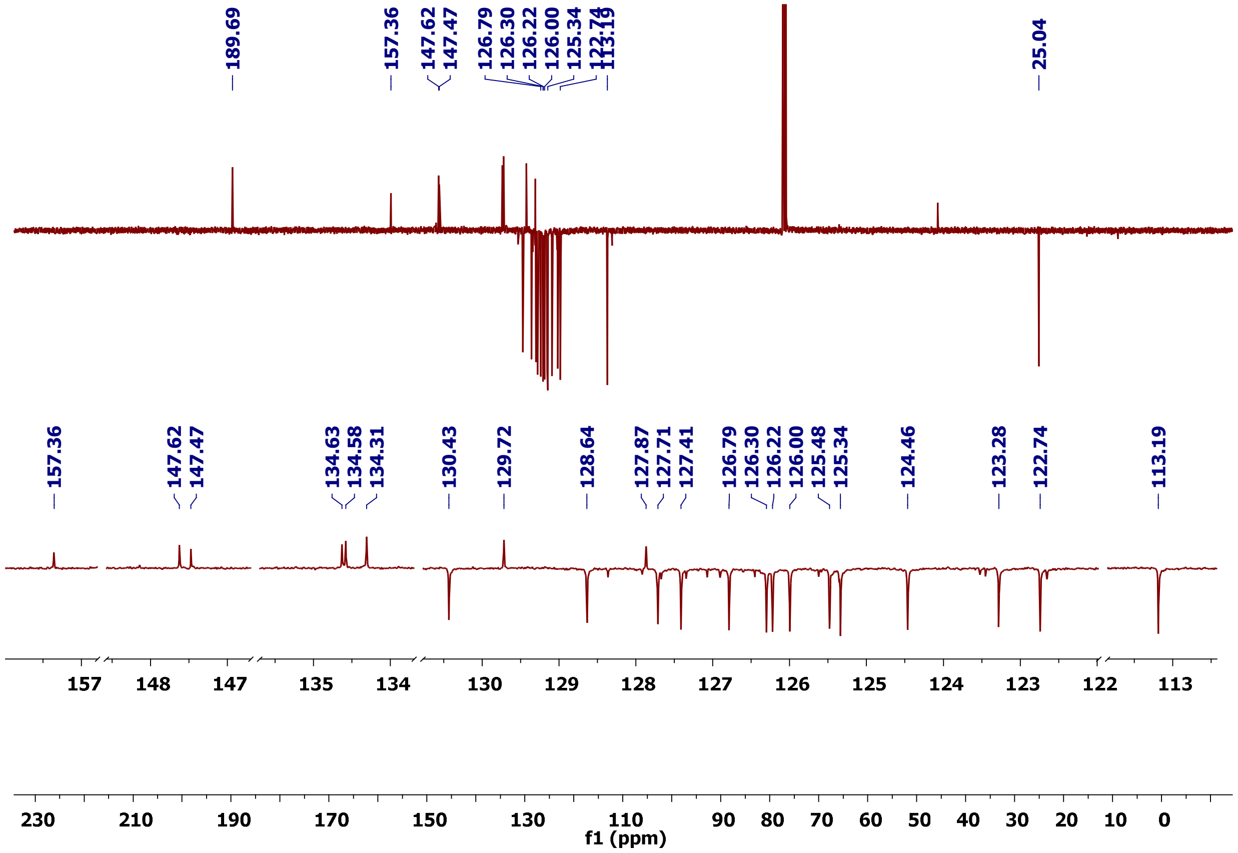

^{1}H-^{1}H gDQCOSY NMR ($CDCl_3$) spectrum of (Z)-1-(4-(naphthalen-1-yl)-5-(naphthalen-1-ylimino)-4,5-dihydro-1,3,4-thiadiazol-2-yl)ethan-1-one

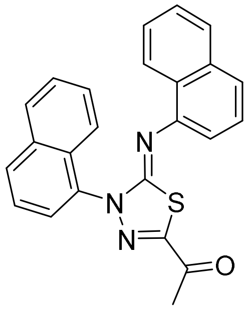

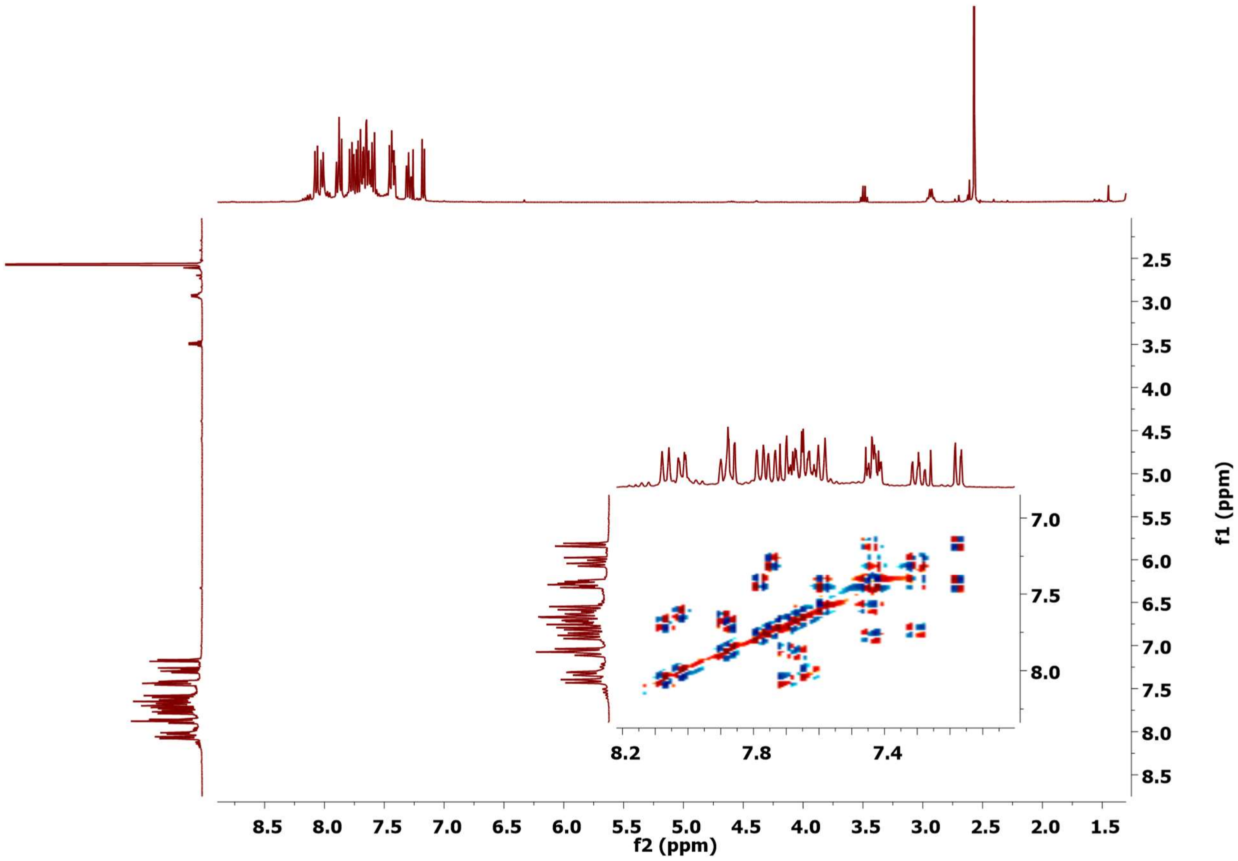

^{1}H-^{13}C-HSQC NMR ($CDCl_3$) spectrum of (Z)-1-(4-(naphthalen-1-yl)-5-(naphthalen-1-ylimino)-4,5-dihydro-1,3,4-thiadiazol-2-yl)ethan-1-one

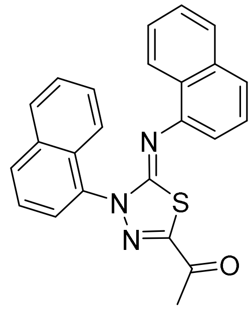

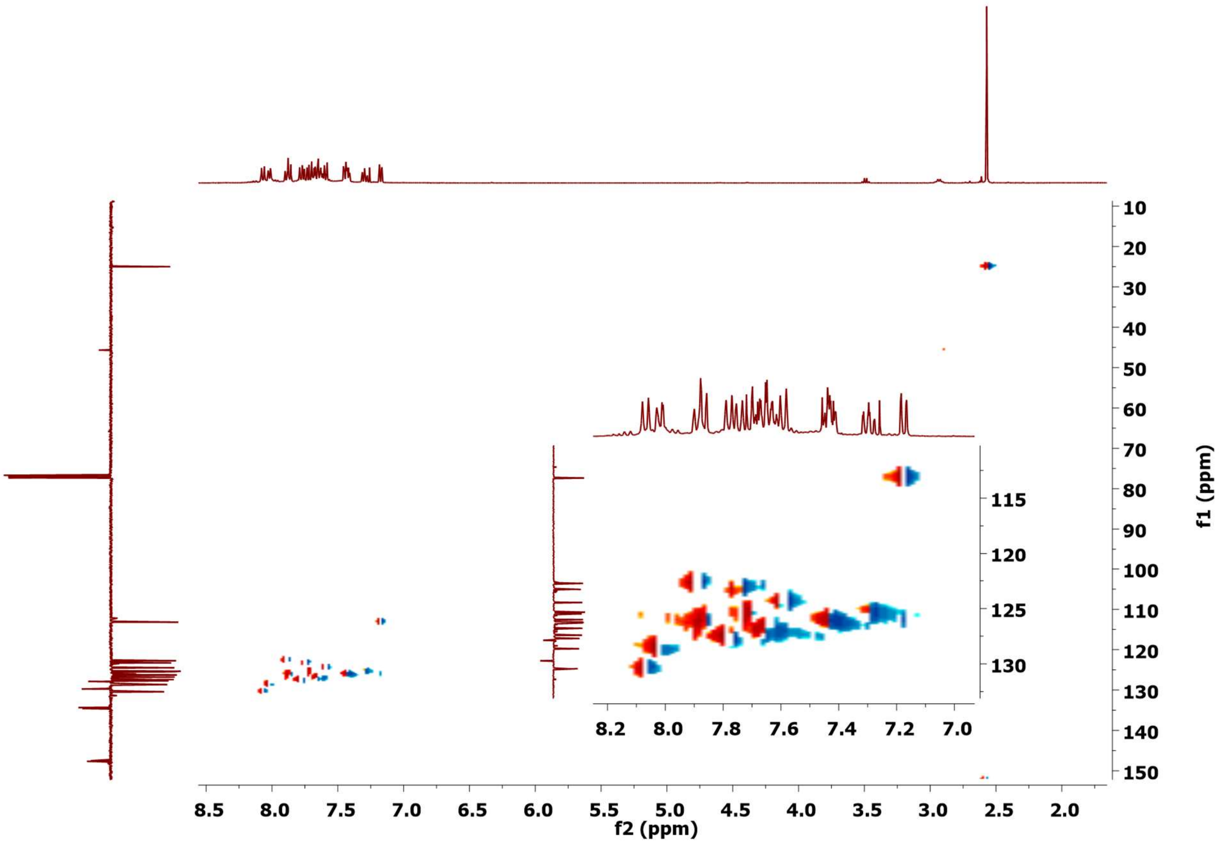

^{1}H-^{13}C-gHMBC NMR ($CDCl_3$) spectrum of (Z)-1-(4-(naphthalen-1-yl)-5-(naphthalen-1-ylimino)-4,5-dihydro-1,3,4-thiadiazol-2-yl)ethan-1-one

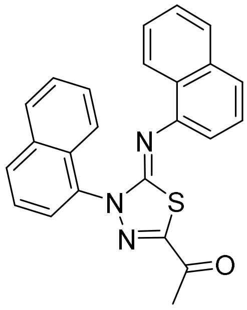

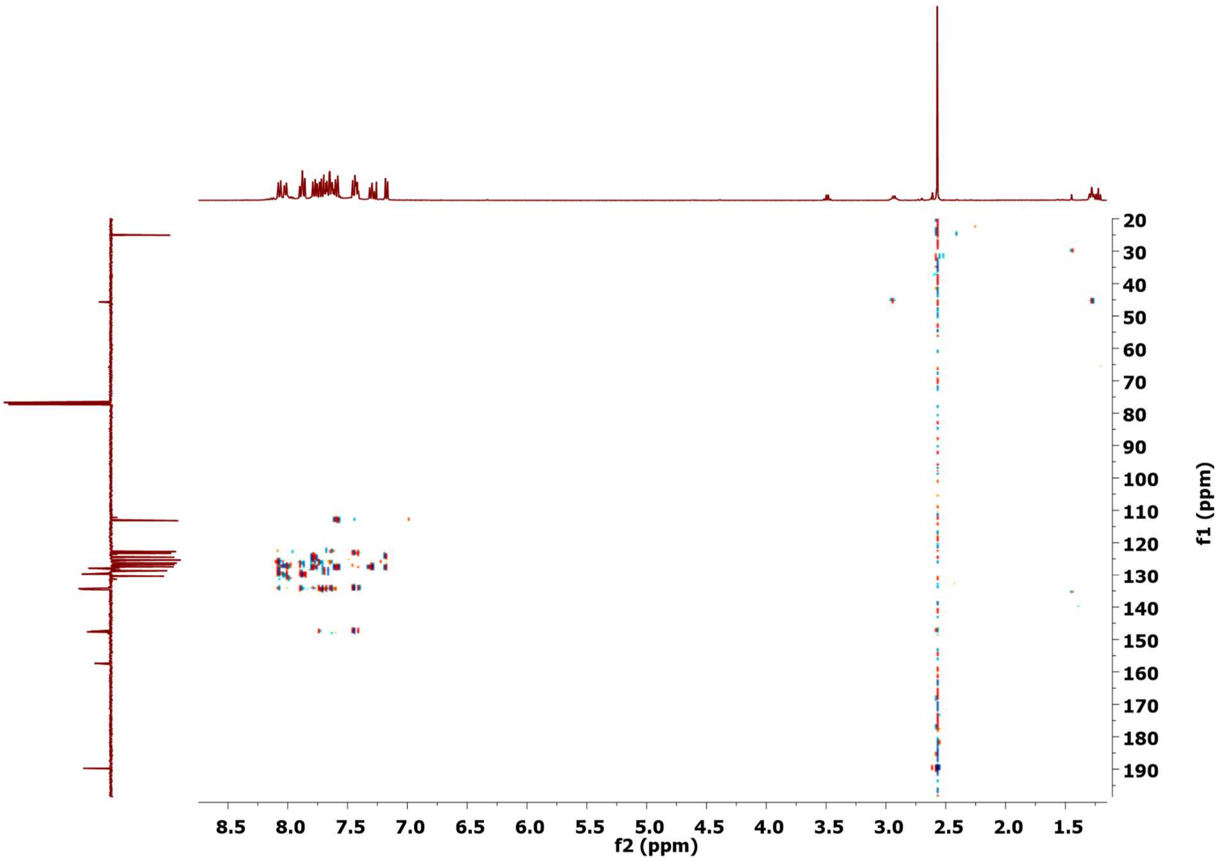

^1H NMR ($CDCl_3$) spectrum of (Z)-1-(4-(3,5-bis(trifluoromethyl)phenyl)-5-((4-nitrophenyl)imino)-4,5-dihydro-1,3,4-thiadiazol-2-yl)ethan-1-one

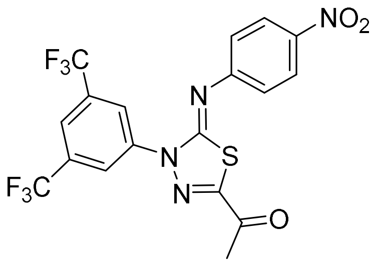

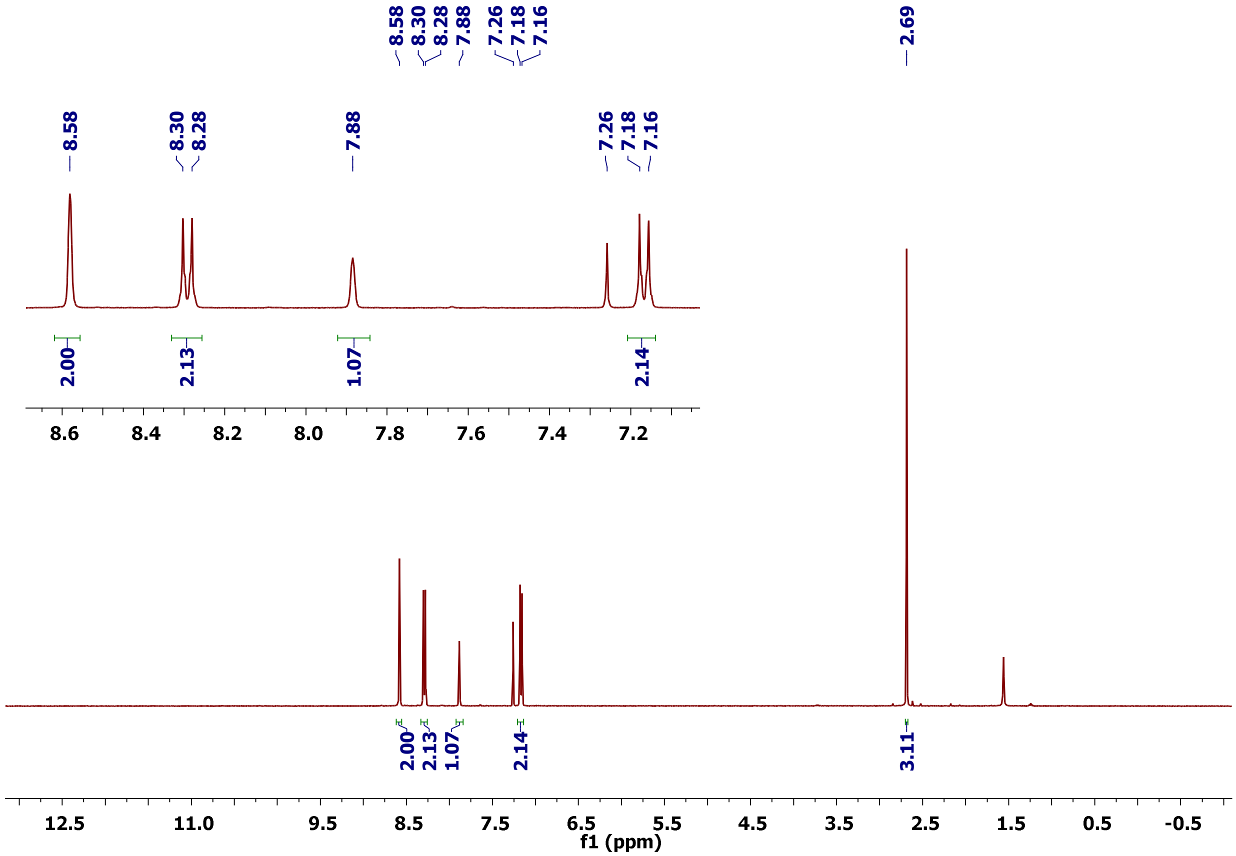

^{13}C NMR ($CDCl_3$) spectrum of (Z)-1-(4-(3,5-bis(trifluoromethyl)phenyl)-5-((4-nitrophenyl)imino)-4,5-dihydro-1,3,4-thiadiazol-2-yl)ethan-1-one

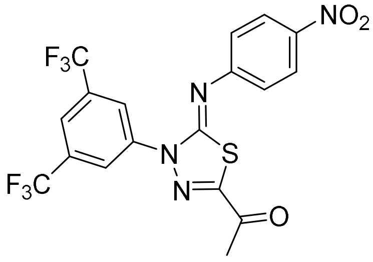

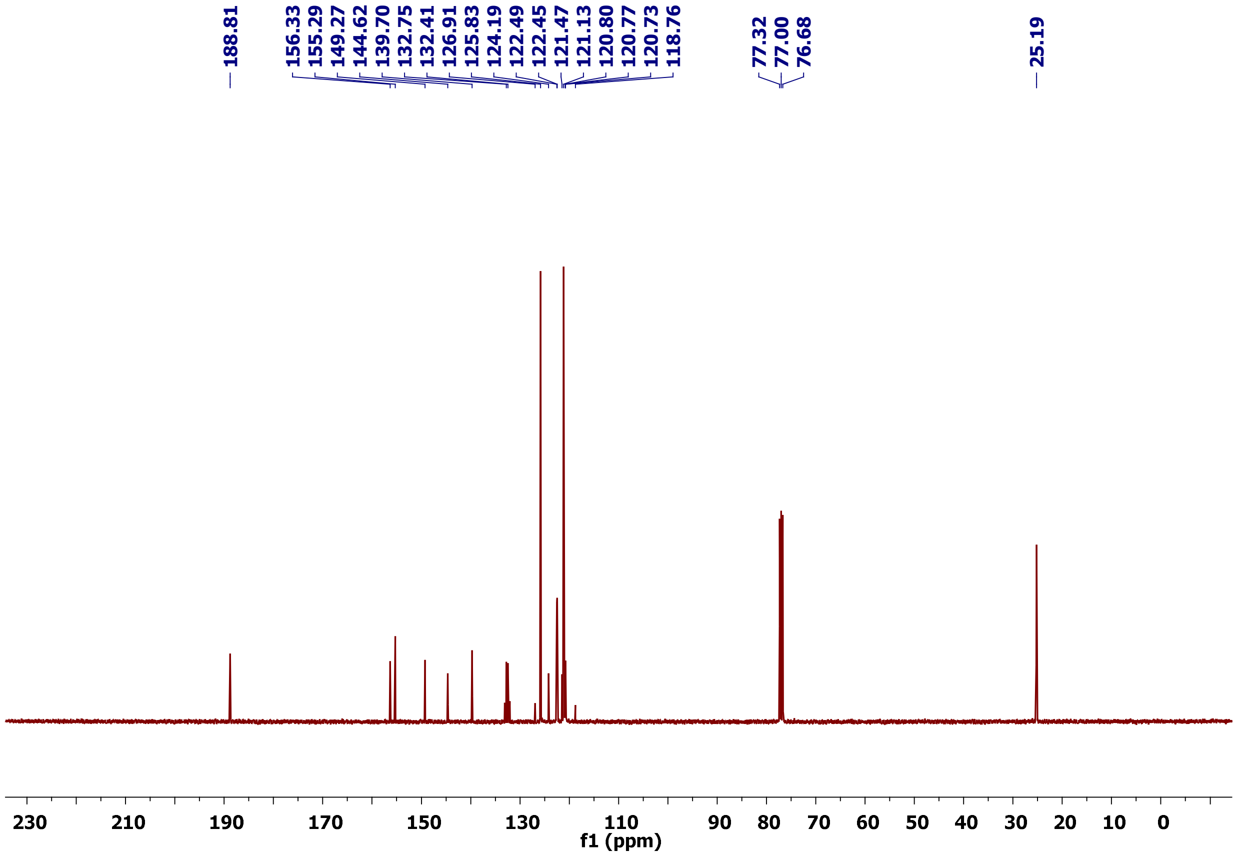

^{13}C CRAPT NMR ($CDCl_3$) spectrum of (Z)-1-(4-(3,5-bis(trifluoromethyl)phenyl)-5-((4-nitrophenyl)imino)-4,5-dihydro-1,3,4-thiadiazol-2-yl)ethan-1-one

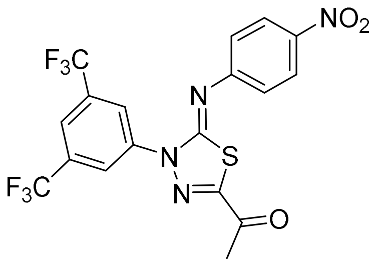

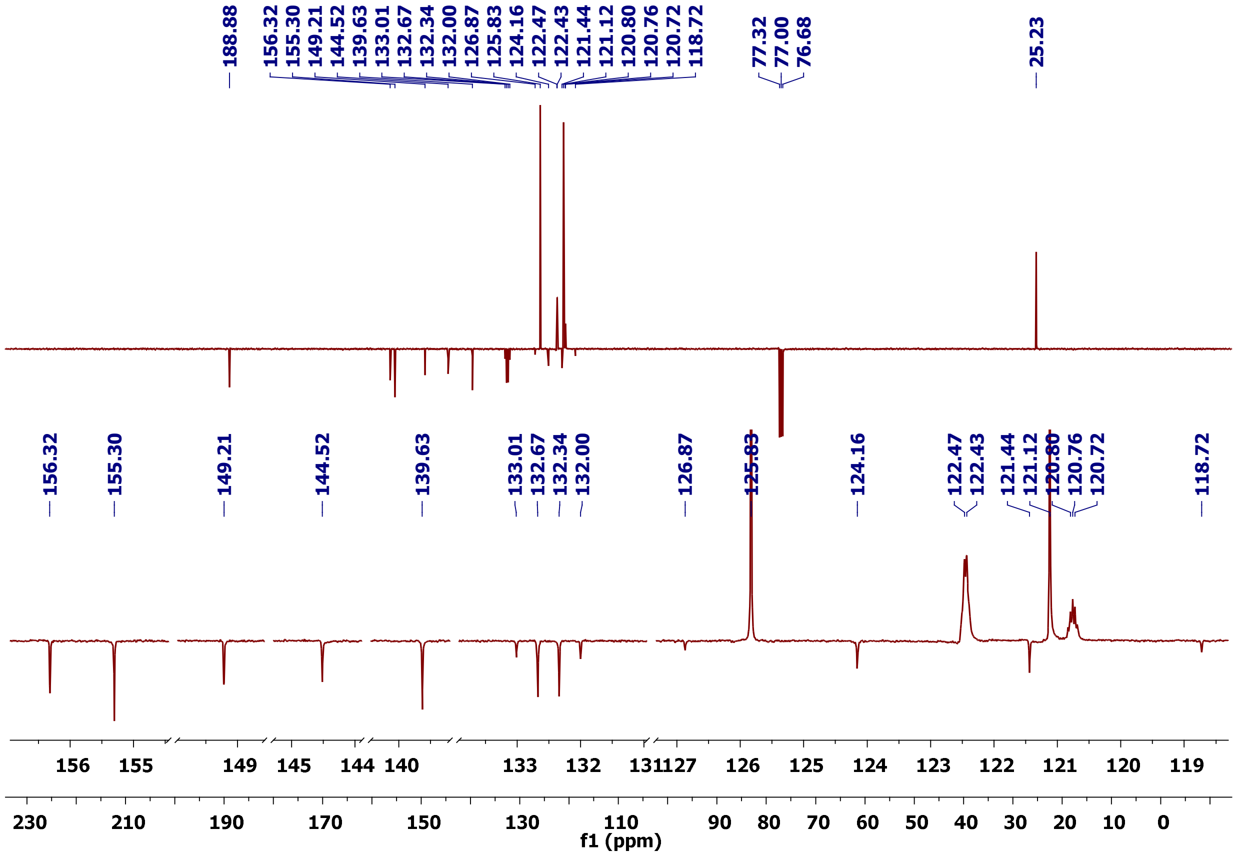

^{1}H-^{1}H gDQCOSY NMR ($CDCl_3$) spectrum of (Z)-1-(4-(3,5-bis(trifluoromethyl)phenyl)-5-((4-nitrophenyl)imino)-4,5-dihydro-1,3,4-thiadiazol-2-yl)ethan-1-one

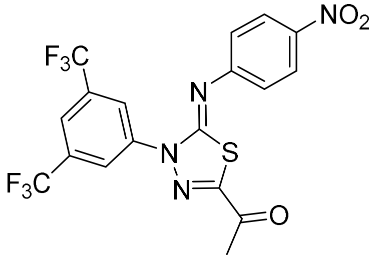

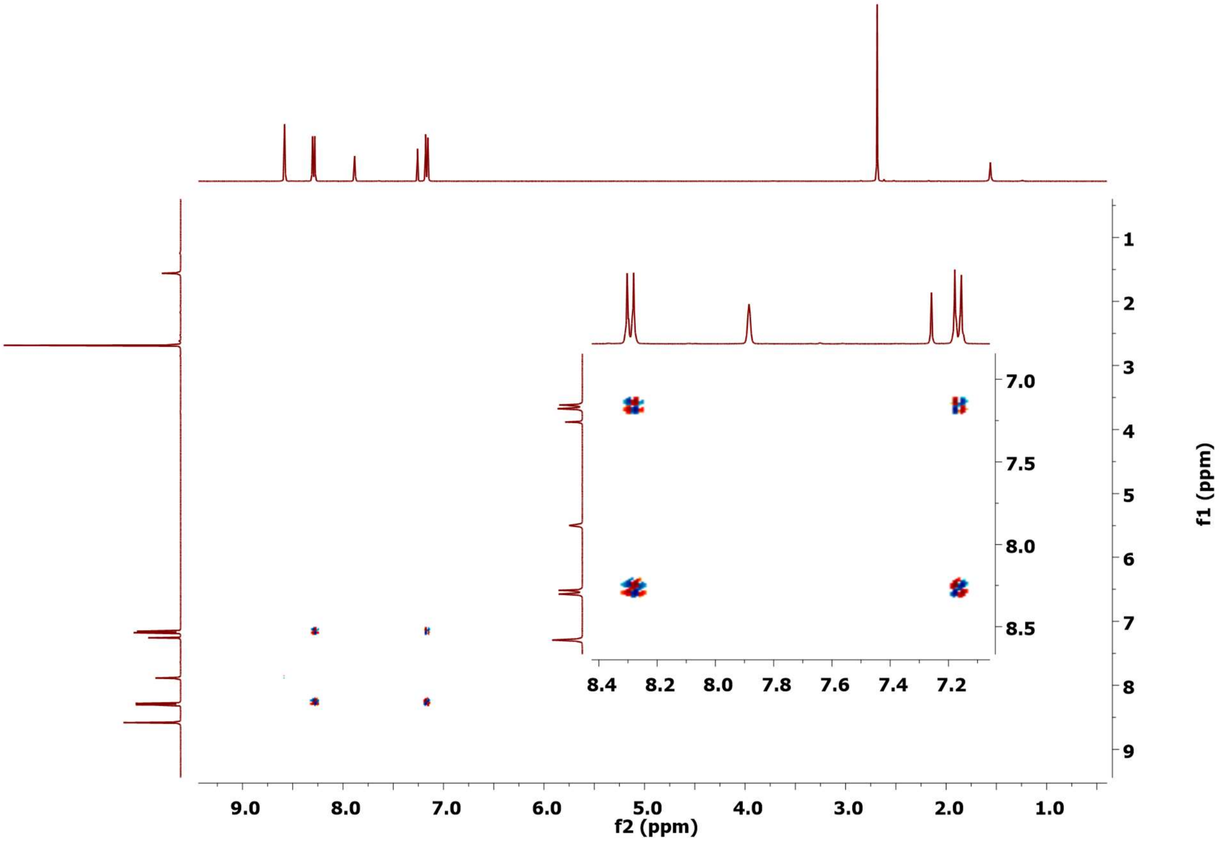

^{1}H-^{13}C-HSQC NMR ($CDCl_3$) spectrum of (Z)-1-(4-(3,5-bis(trifluoromethyl)phenyl)-5-((4-nitrophenyl)imino)-4,5-dihydro-1,3,4-thiadiazol-2-yl)ethan-1-one

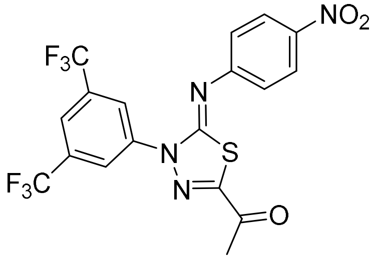

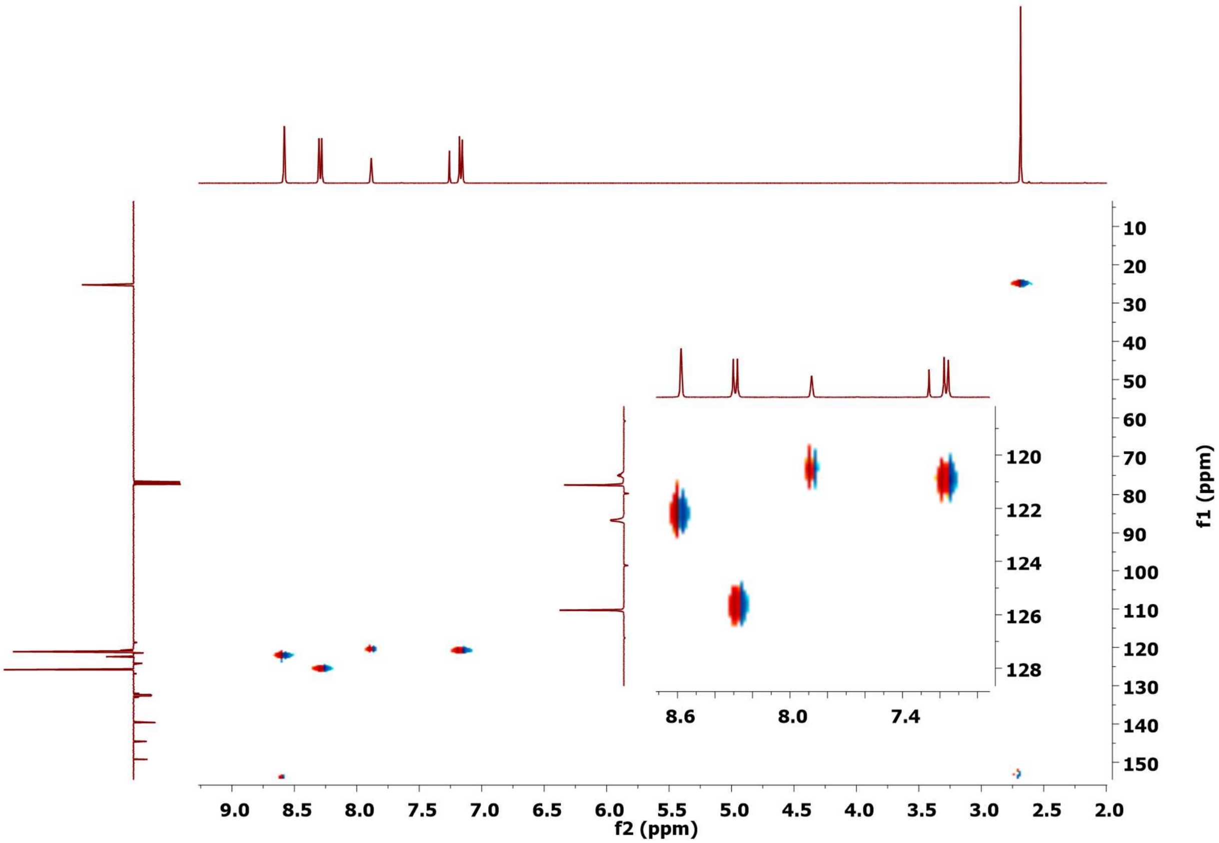

^1H-^{13}C-gHMBC NMR ($CDCl_3$) spectrum of (Z)-1-(4-(3,5-bis(trifluoromethyl)phenyl)-5-((4-nitrophenyl)imino)-4,5-dihydro-1,3,4-thiadiazol-2-yl)ethan-1-one

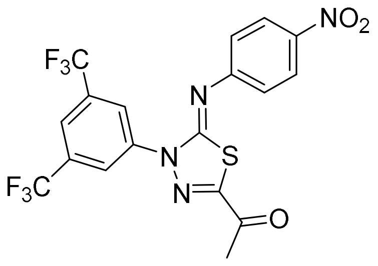

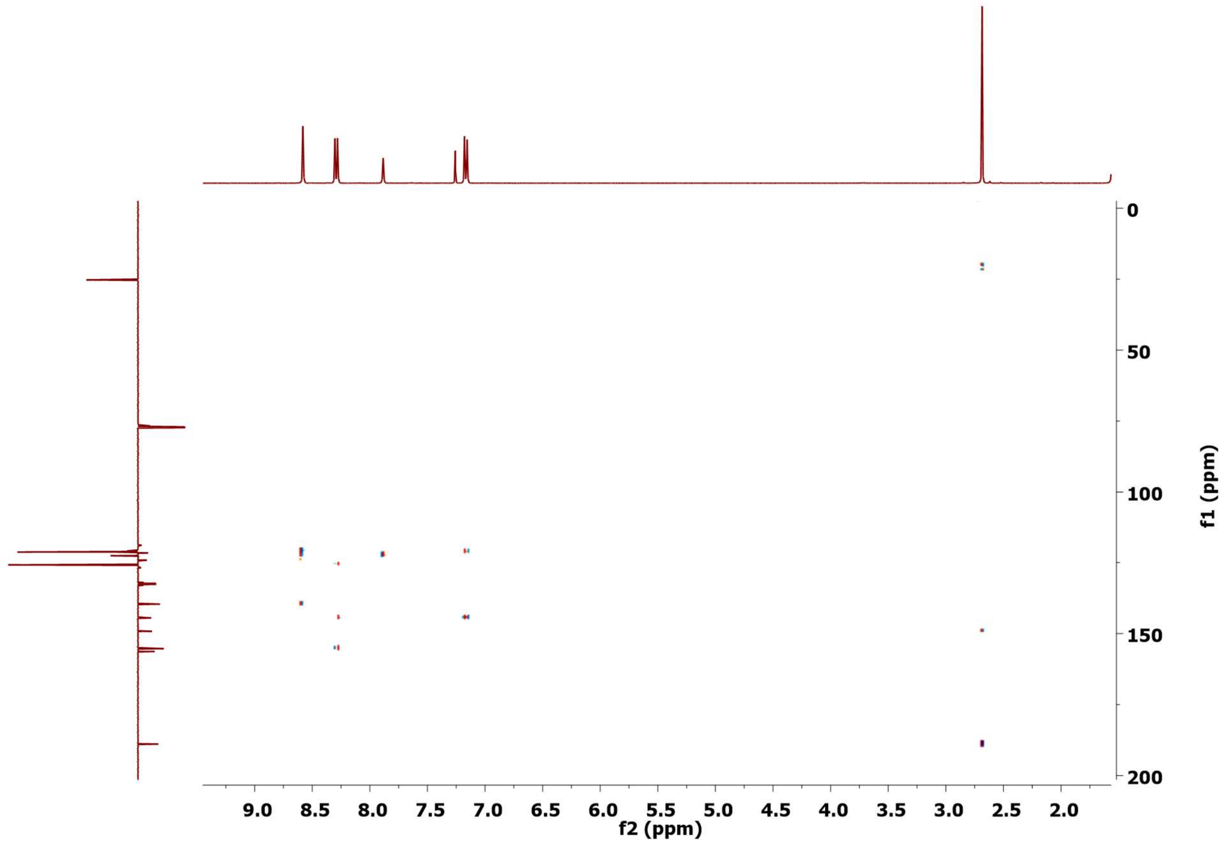

^{1}H NMR ($CDCl_3$) spectrum of (Z)-1-(4-(3,5-bis(trifluoromethyl)phenyl)-5-((4-methoxyphenyl)imino)-4,5-dihydro-1,3,4-thiadiazol-2-yl)ethan-1-one

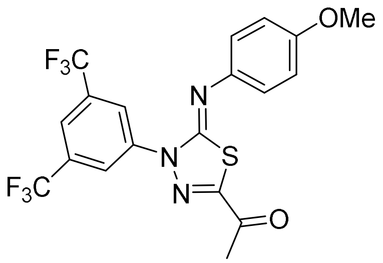

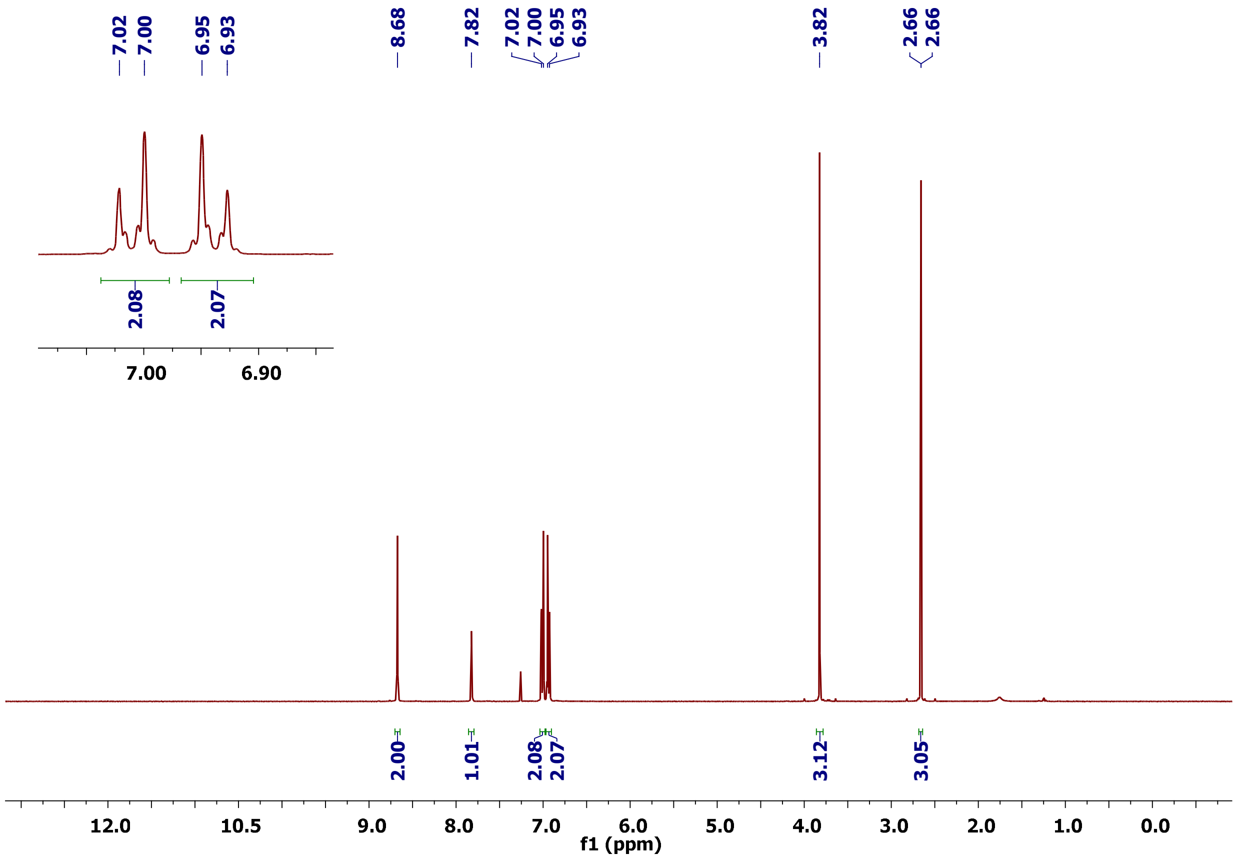

^{13}C NMR ($CDCl_3$) spectrum of (Z)-1-(4-(3,5-bis(trifluoromethyl)phenyl)-5-((4-methoxyphenyl)imino)-4,5-dihydro-1,3,4-thiadiazol-2-yl)ethan-1-one

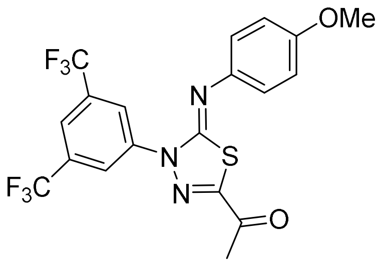

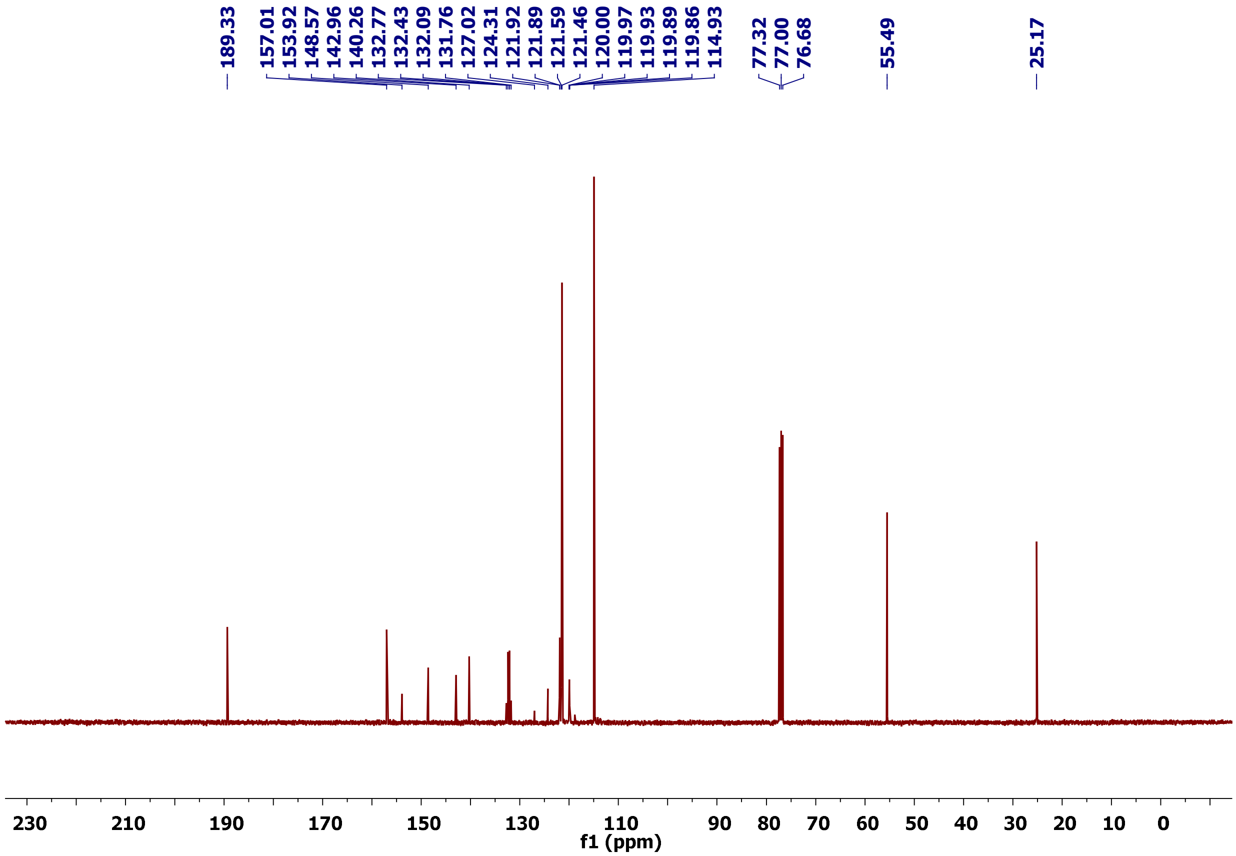

^{13}C CRAPT NMR ($CDCl_3$) spectrum of (Z)-1-(4-(3,5-bis(trifluoromethyl)phenyl)-5-((4-methoxyphenyl)imino)-4,5-dihydro-1,3,4-thiadiazol-2-yl)ethan-1-one

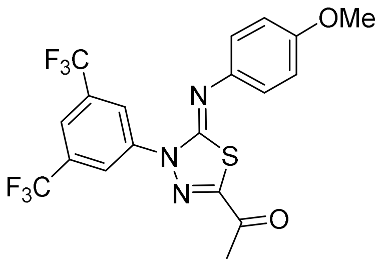

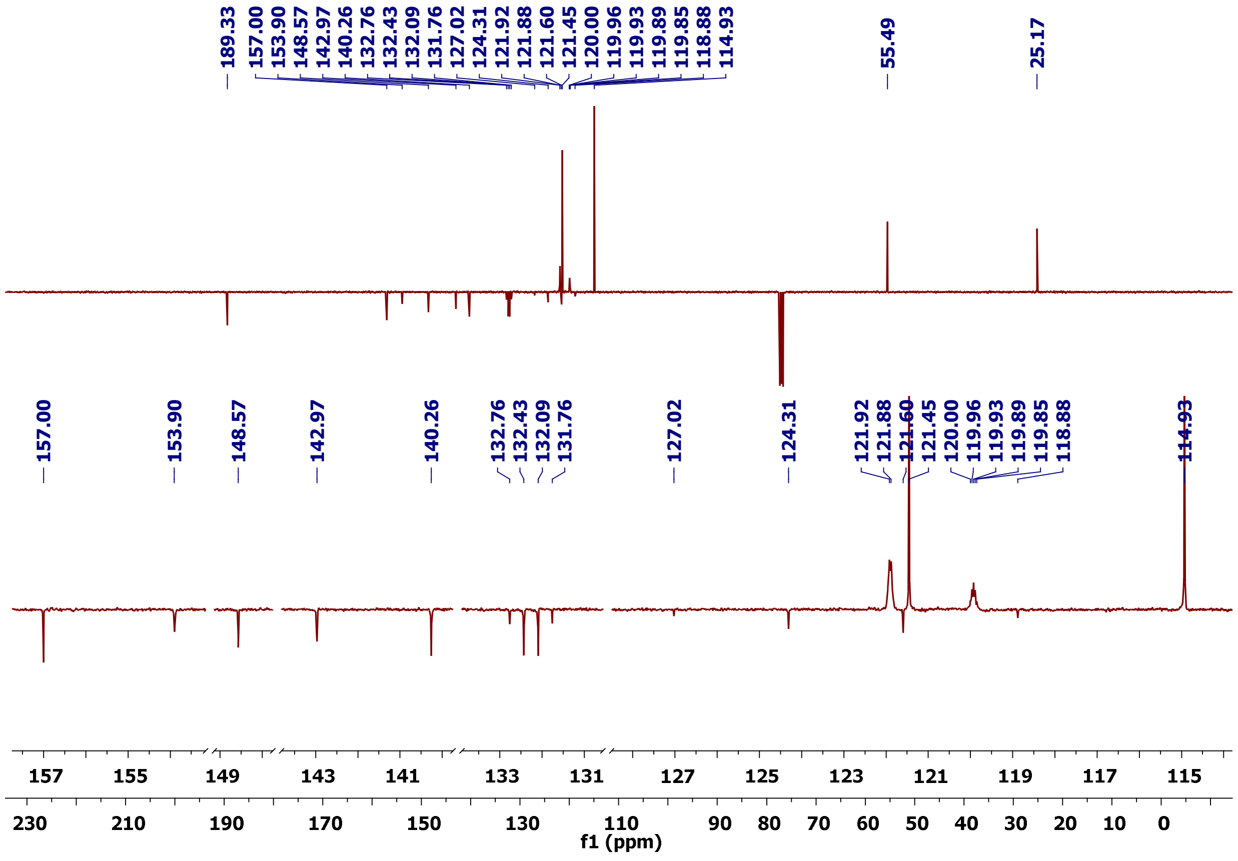

^1H-^1H gDQCOSY NMR ($CDCl_3$) spectrum of (Z)-1-(4-(3,5-bis(trifluoromethyl)phenyl)-5-((4-methoxyphenyl)imino)-4,5-dihydro-1,3,4-thiadiazol-2-yl)ethan-1-one

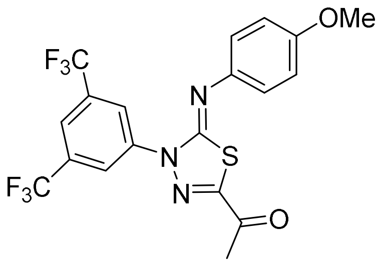

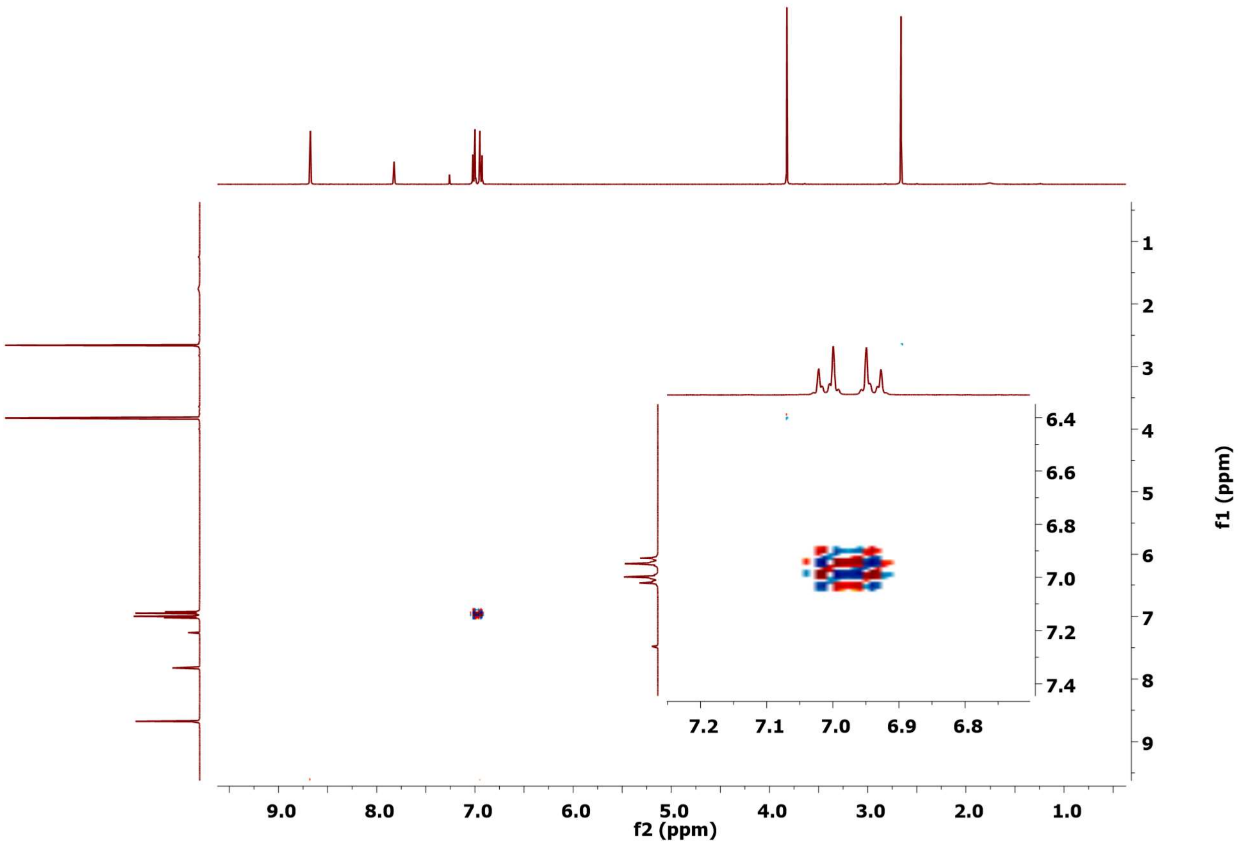

^{1}H-^{13}C-HSQC NMR ($CDCl_3$) spectrum of (Z)-1-(4-(3,5-bis(trifluoromethyl)phenyl)-5-((4-methoxyphenyl)imino)-4,5-dihydro-1,3,4-thiadiazol-2-yl)ethan-1-one

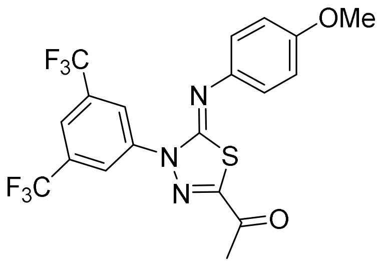

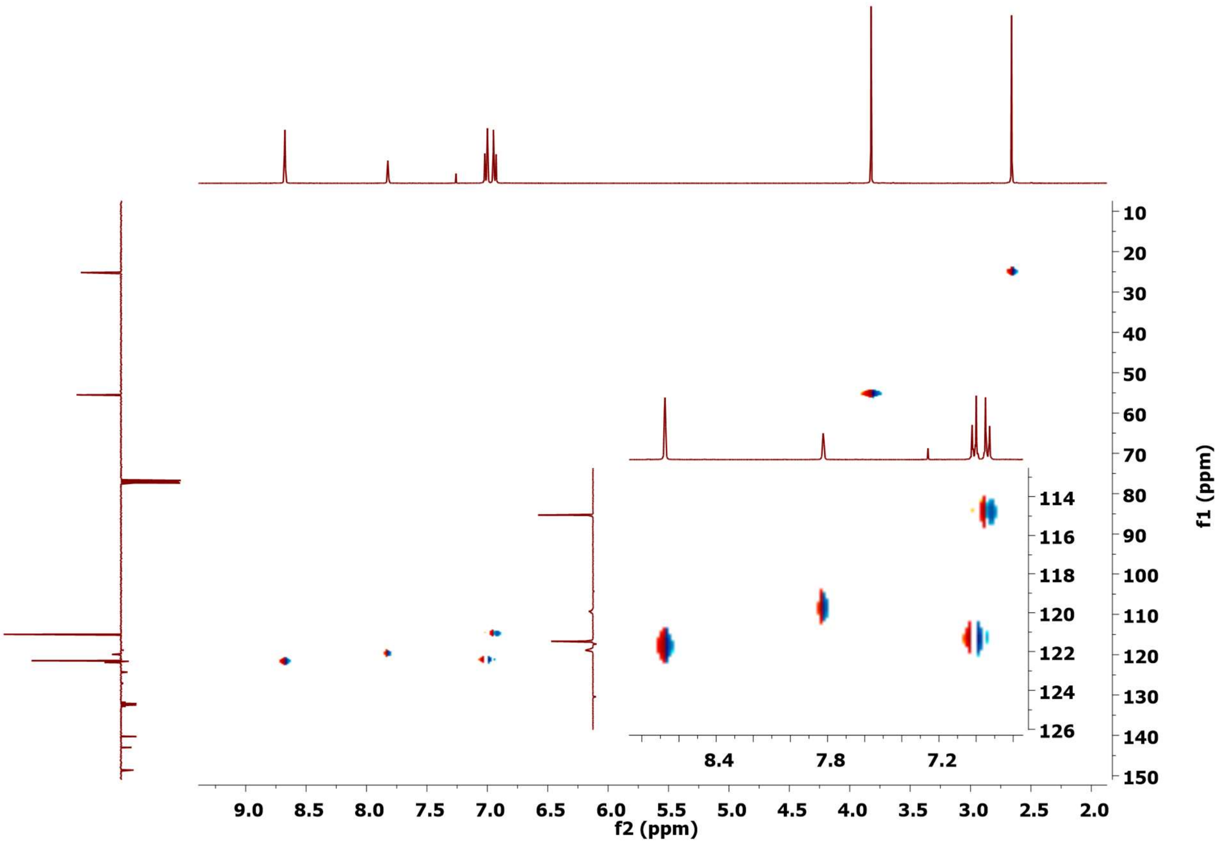

^{1}H-^{13}C-gHMBC NMR ($CDCl_3$) spectrum of (Z)-1-(4-(3,5-bis(trifluoromethyl)phenyl)-5-((4-methoxyphenyl)imino)-4,5-dihydro-1,3,4-thiadiazol-2-yl)ethan-1-one

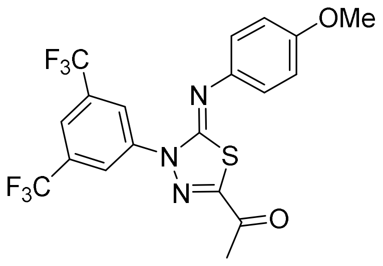

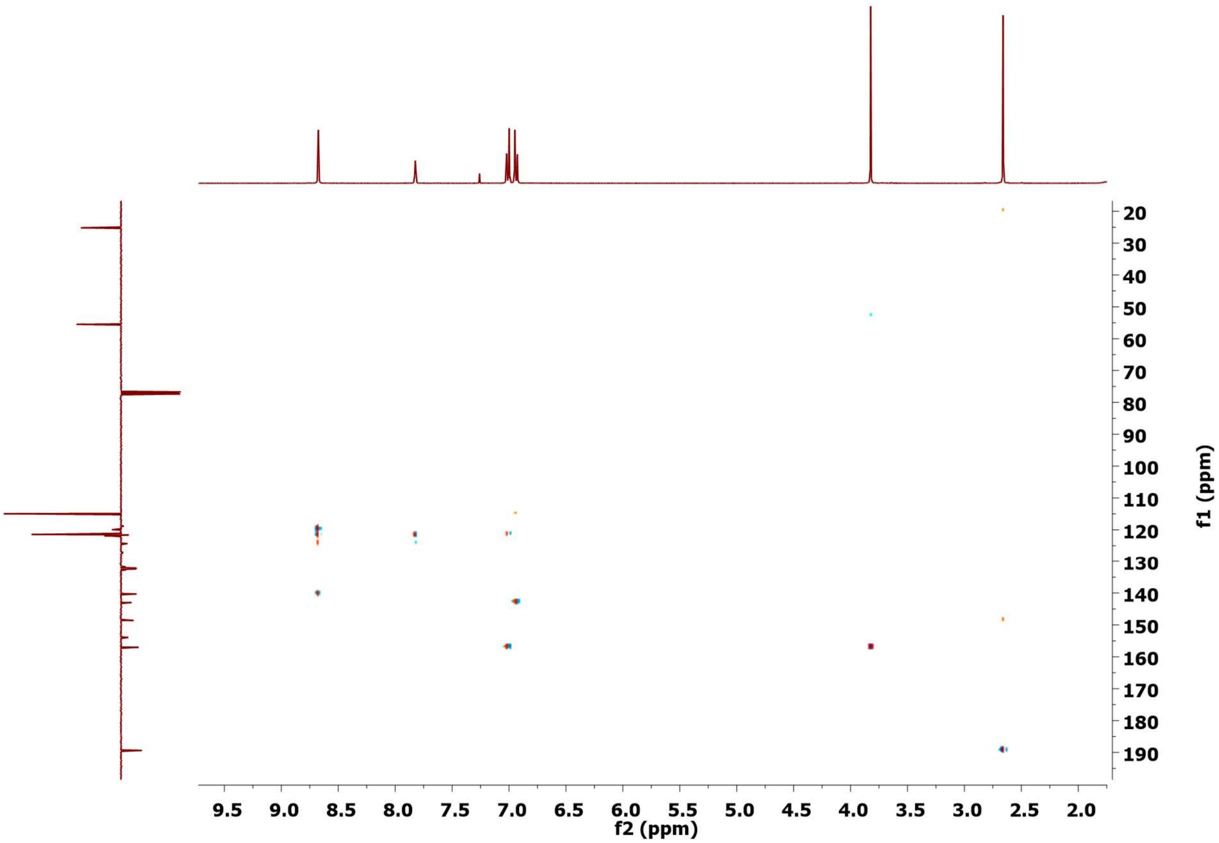

^{1}H NMR ($CDCl_3$) spectrum of (Z)-1-(4-(3,5-bis(trifluoromethyl)phenyl)-5-((4-bromophenyl)imino)-4,5-dihydro-1,3,4-thiadiazol-2-yl)ethan-1-one

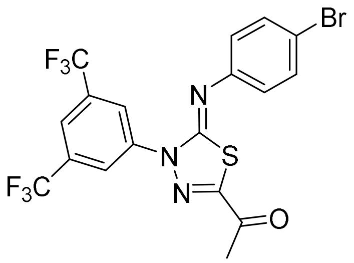

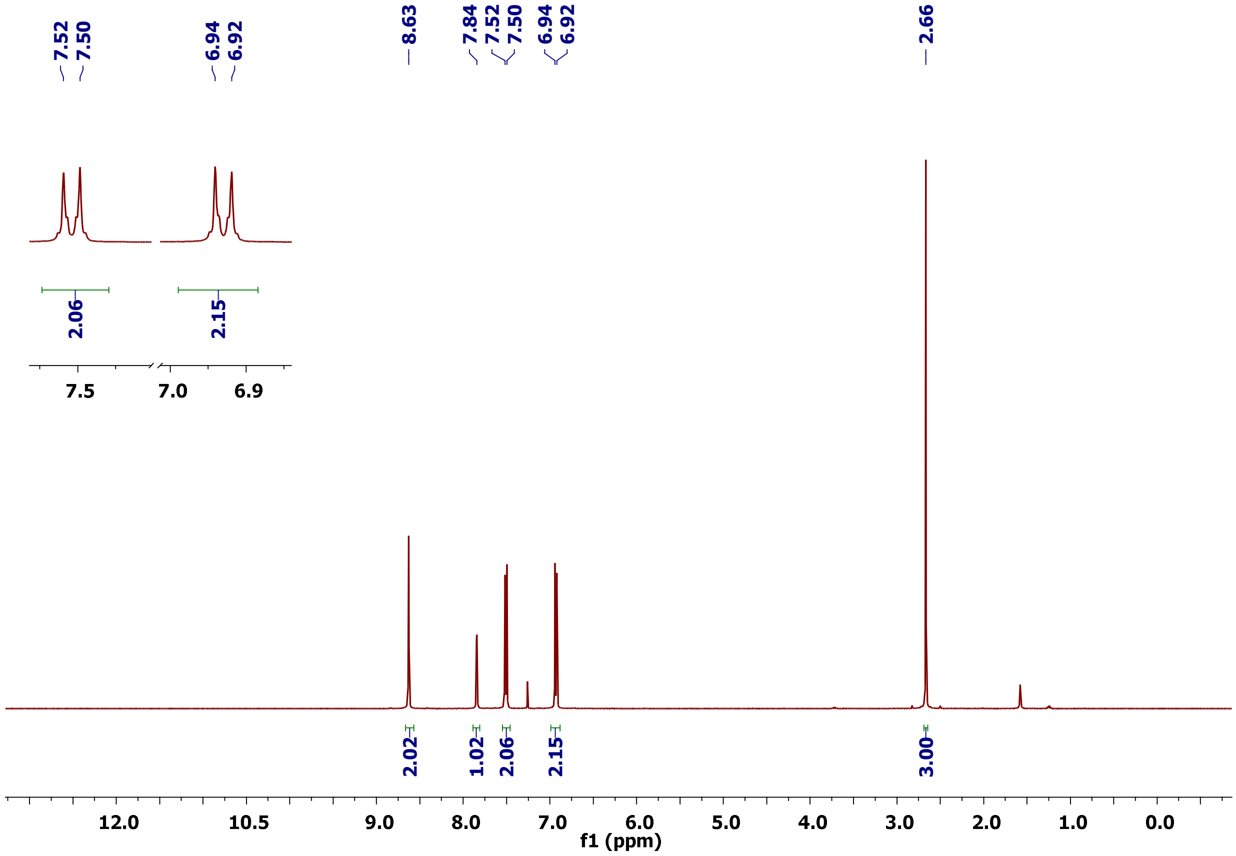

^{13}C NMR ($CDCl_3$) spectrum of (Z)-1-(4-(3,5-bis(trifluoromethyl)phenyl)-5-((4-bromophenyl)imino)-4,5-dihydro-1,3,4-thiadiazol-2-yl)ethan-1-one

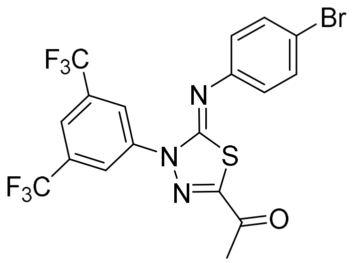

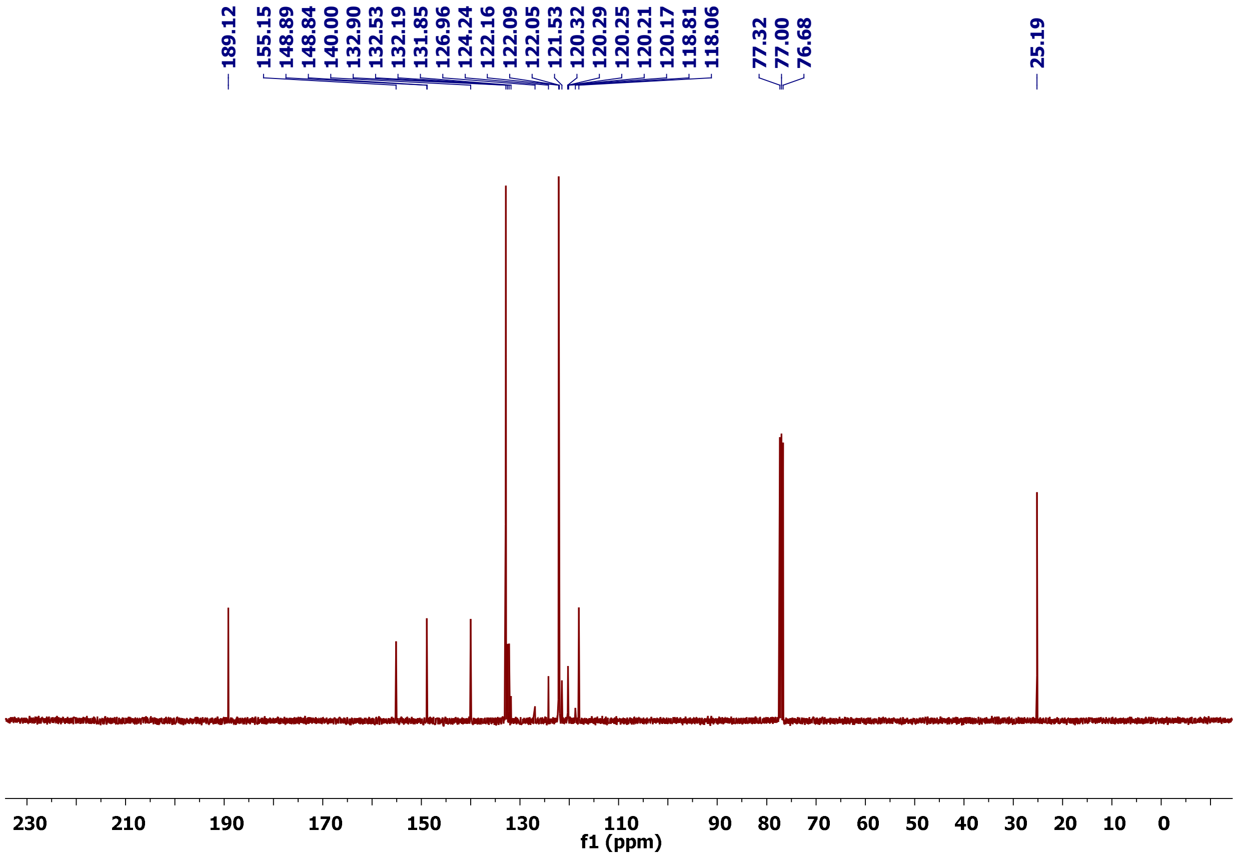

^{13}C CRAPT NMR ($CDCl_3$) spectrum of (Z)-1-(4-(3,5-bis(trifluoromethyl)phenyl)-5-((4-bromophenyl)imino)-4,5-dihydro-1,3,4-thiadiazol-2-yl)ethan-1-one

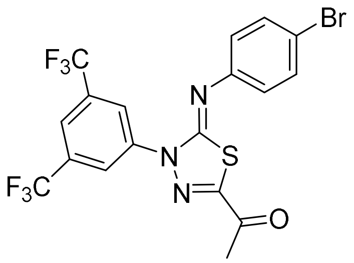

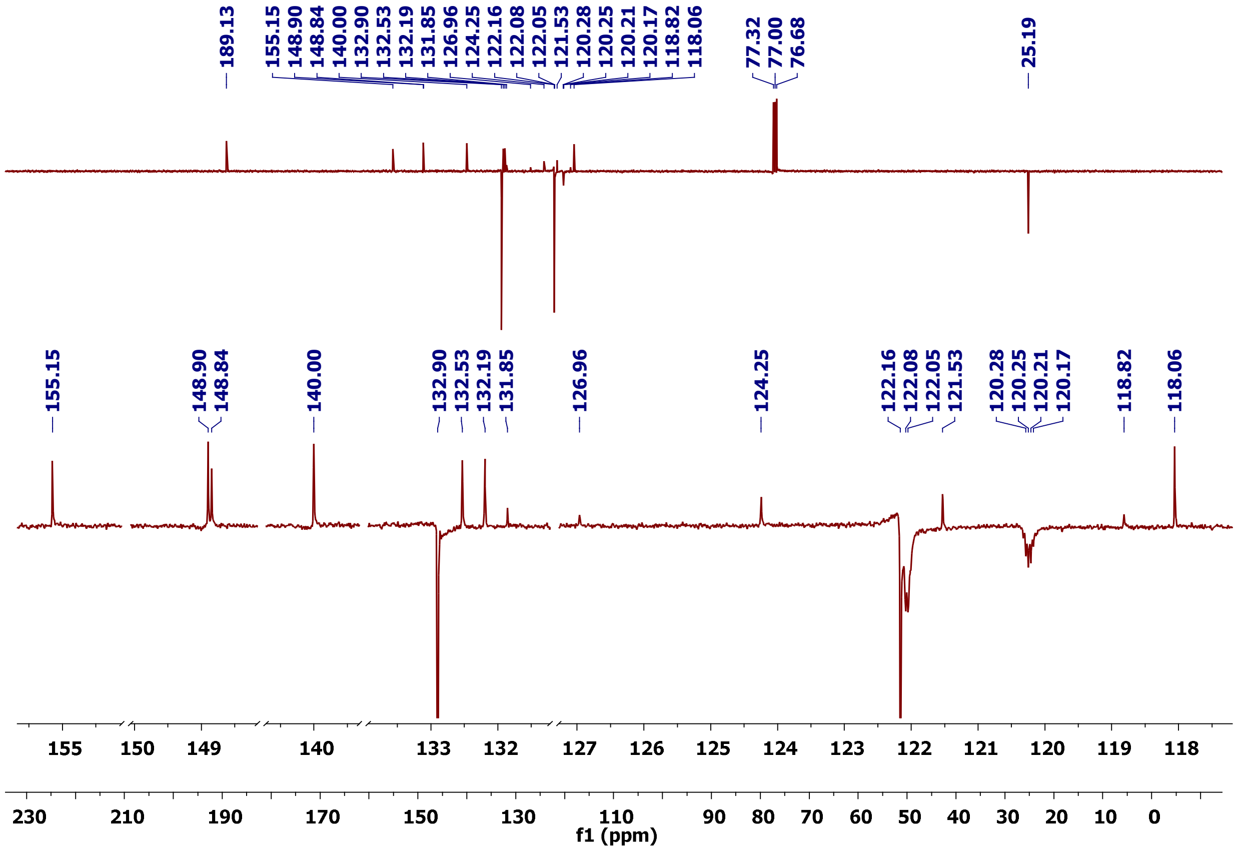

^{1}H-^{1}H gDQCOSY NMR ($CDCl_3$) spectrum of (Z)-1-(4-(3,5-bis(trifluoromethyl)phenyl)-5-((4-bromophenyl)imino)-4,5-dihydro-1,3,4-thiadiazol-2-yl)ethan-1-one

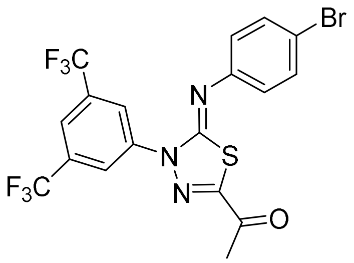

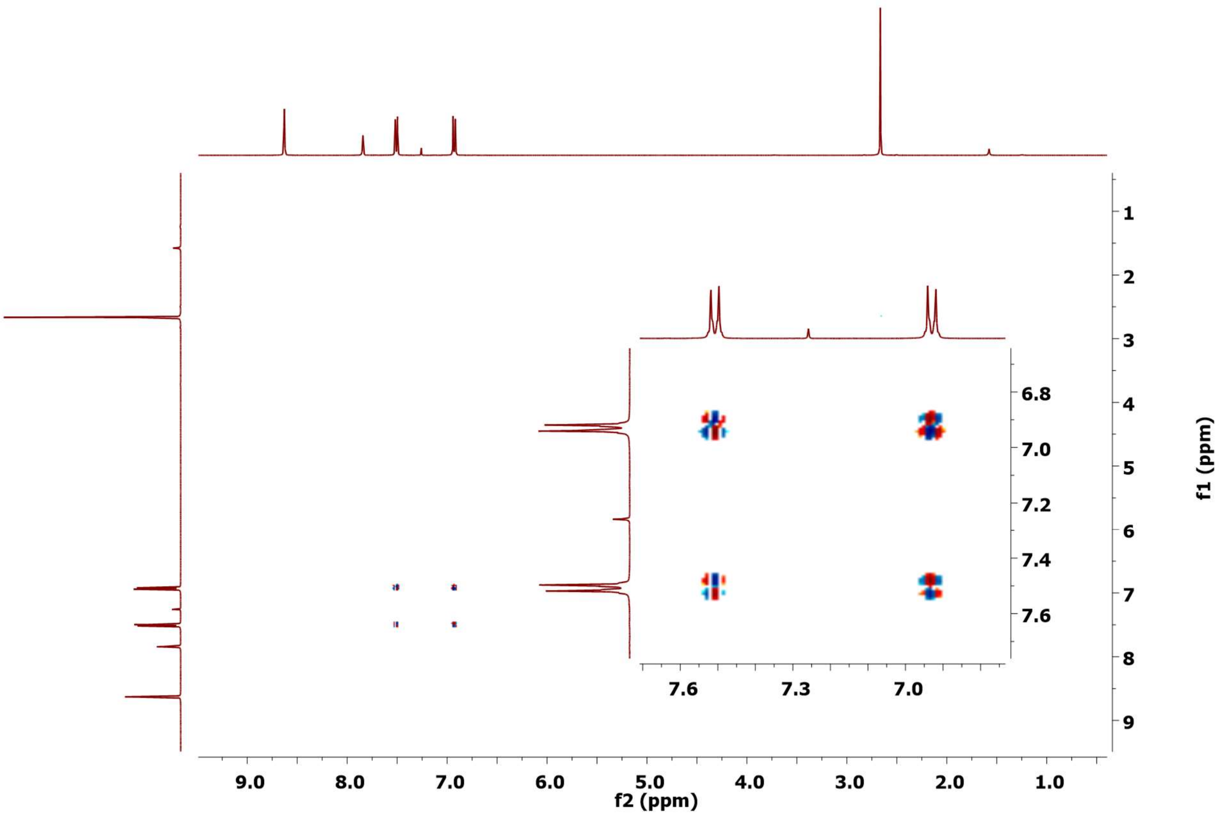

^{1}H-^{13}C-HSQC NMR ($CDCl_3$) spectrum of (Z)-1-(4-(3,5-bis(trifluoromethyl)phenyl)-5-((4-bromophenyl)imino)-4,5-dihydro-1,3,4-thiadiazol-2-yl)ethan-1-one

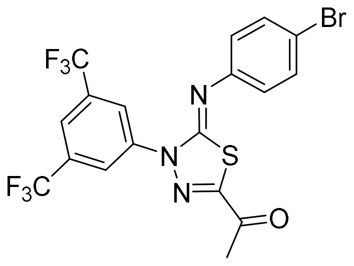

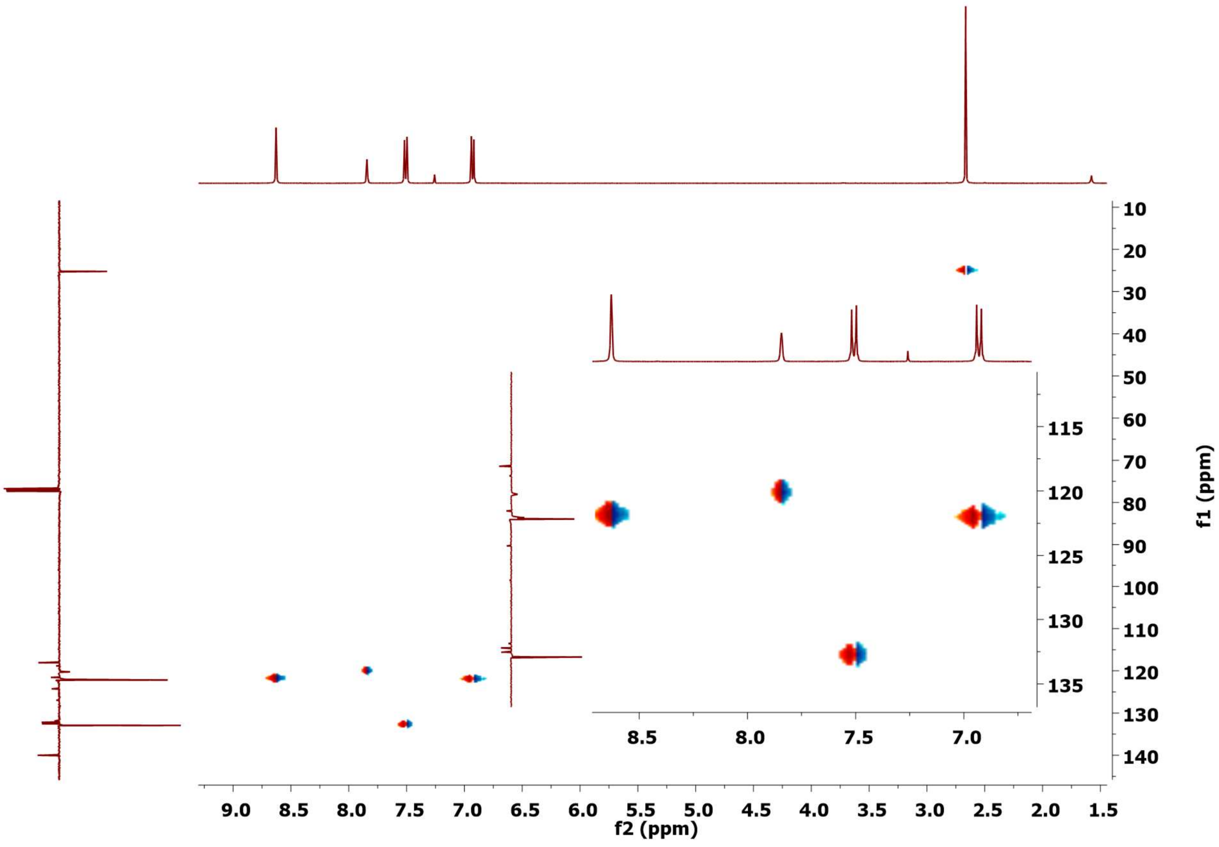

^{1}H-^{13}C-gHMBC NMR ($CDCl_3$) spectrum of (Z)-1-(4-(3,5-bis(trifluoromethyl)phenyl)-5-((4-bromophenyl)imino)-4,5-dihydro-1,3,4-thiadiazol-2-yl)ethan-1-one

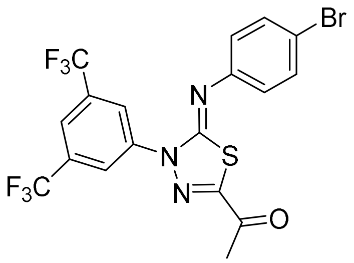

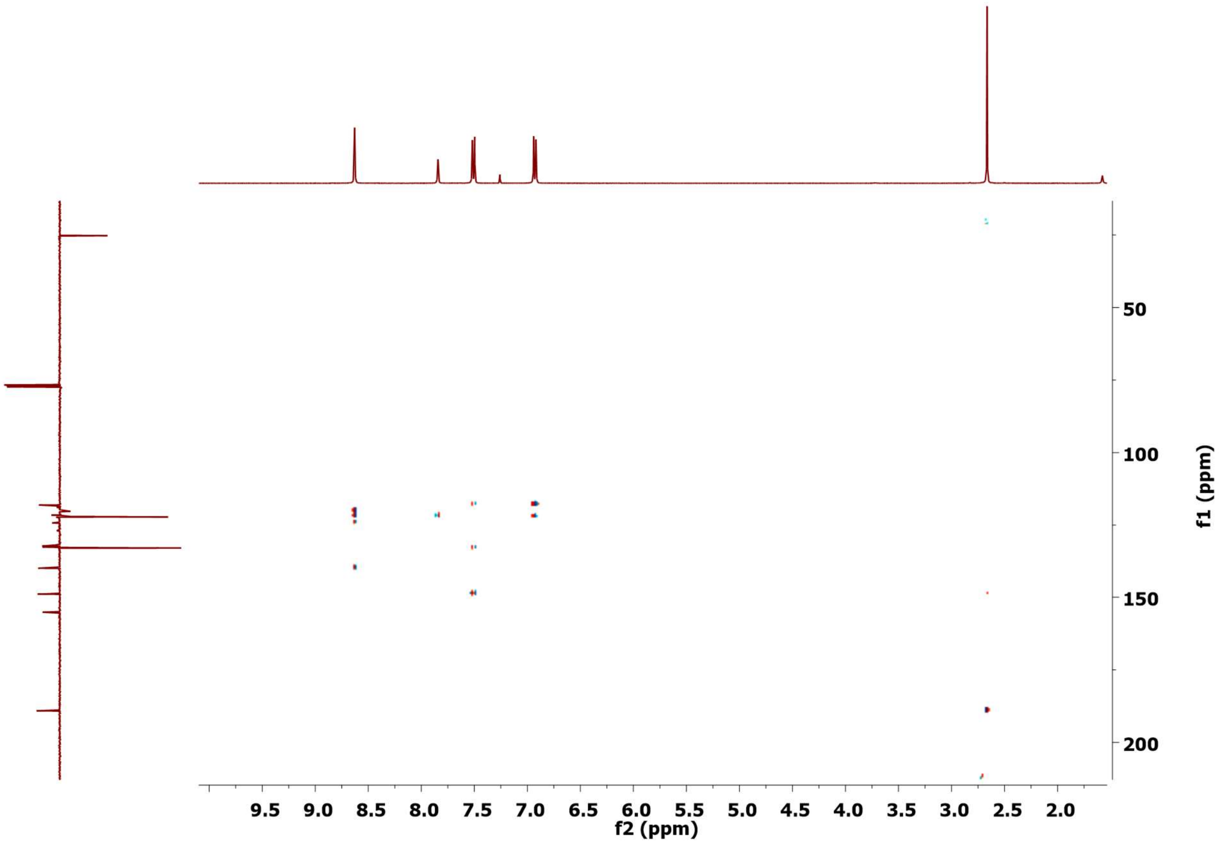

^{1}H NMR ($CDCl_3$) spectrum of (Z)-1-(4-(3,5-bis(trifluoromethyl)phenyl)-5-((4-fluorophenyl)imino)-4,5-dihydro-1,3,4-thiadiazol-2-yl)ethan-1-one

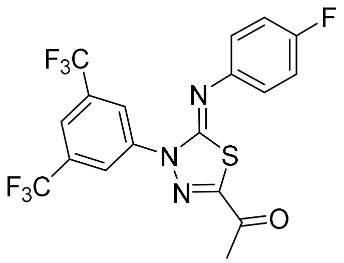

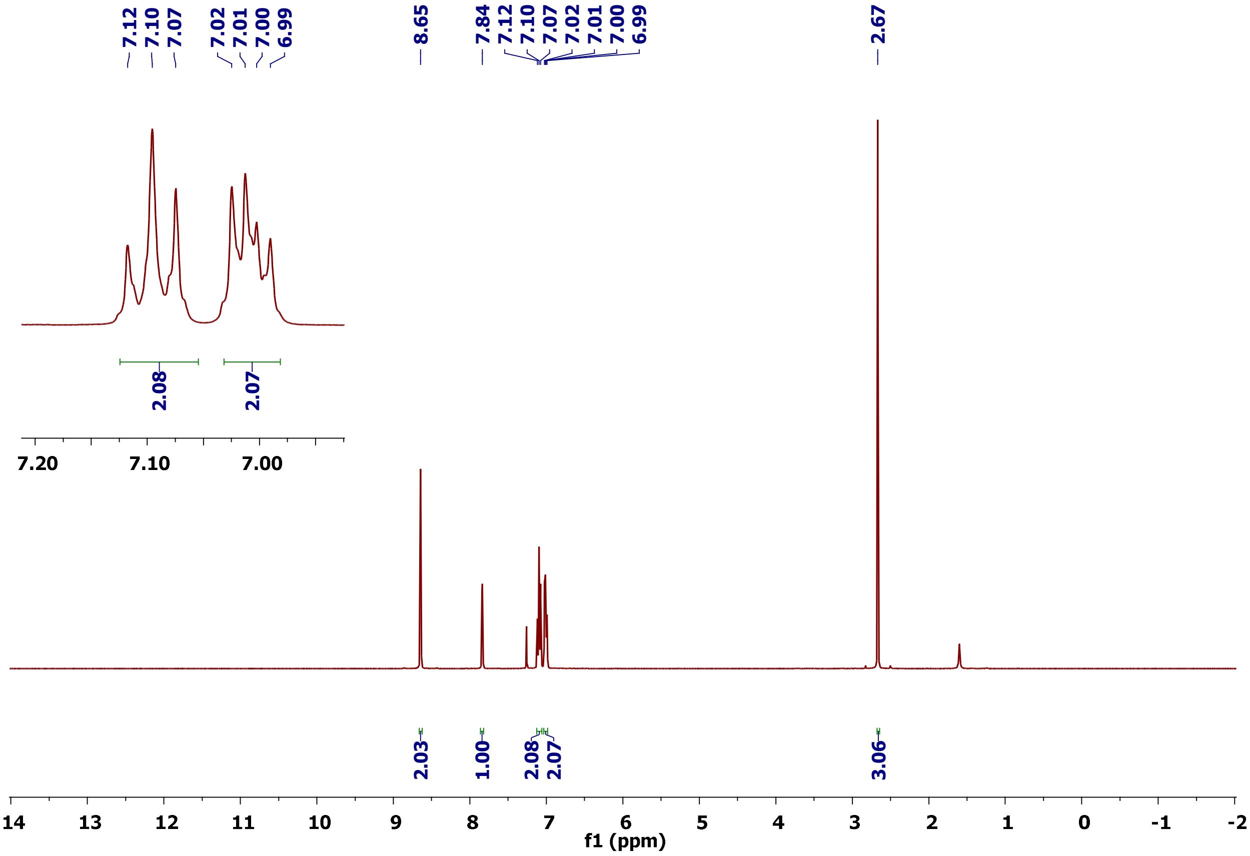

^{13}C NMR ($CDCl_3$) spectrum of (Z)-1-(4-(3,5-bis(trifluoromethyl)phenyl)-5-((4-fluorophenyl)imino)-4,5-dihydro-1,3,4-thiadiazol-2-yl)ethan-1-one

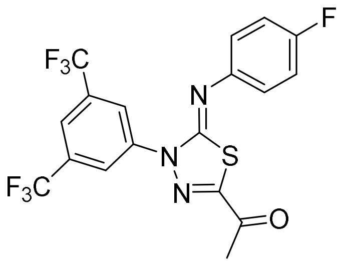

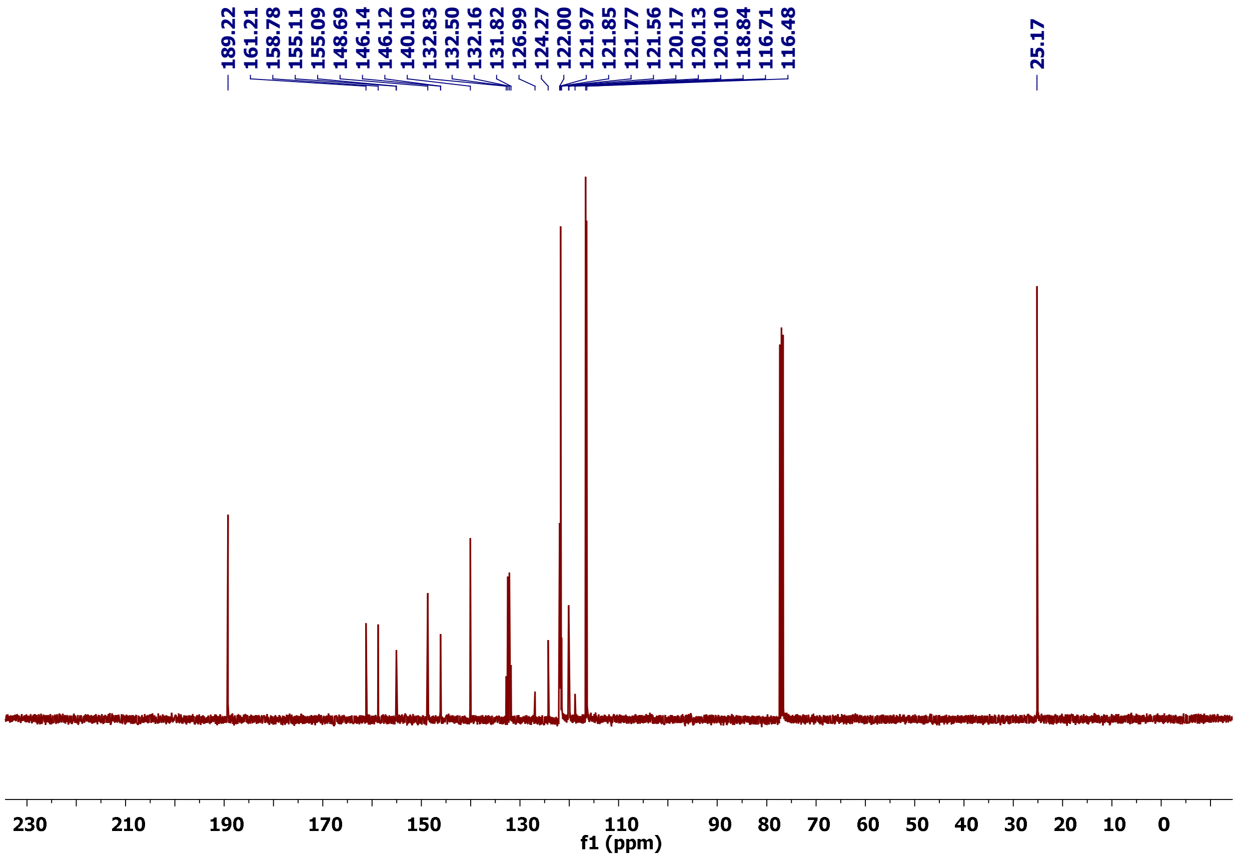

^{13}C CRAPT NMR ($CDCl_3$) spectrum of (Z)-1-(4-(3,5-bis(trifluoromethyl)phenyl)-5-((4-fluorophenyl)imino)-4,5-dihydro-1,3,4-thiadiazol-2-yl)ethan-1-one

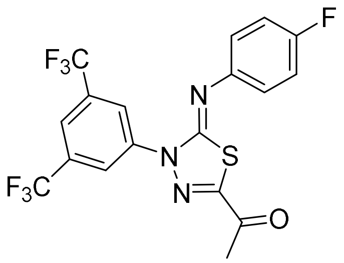

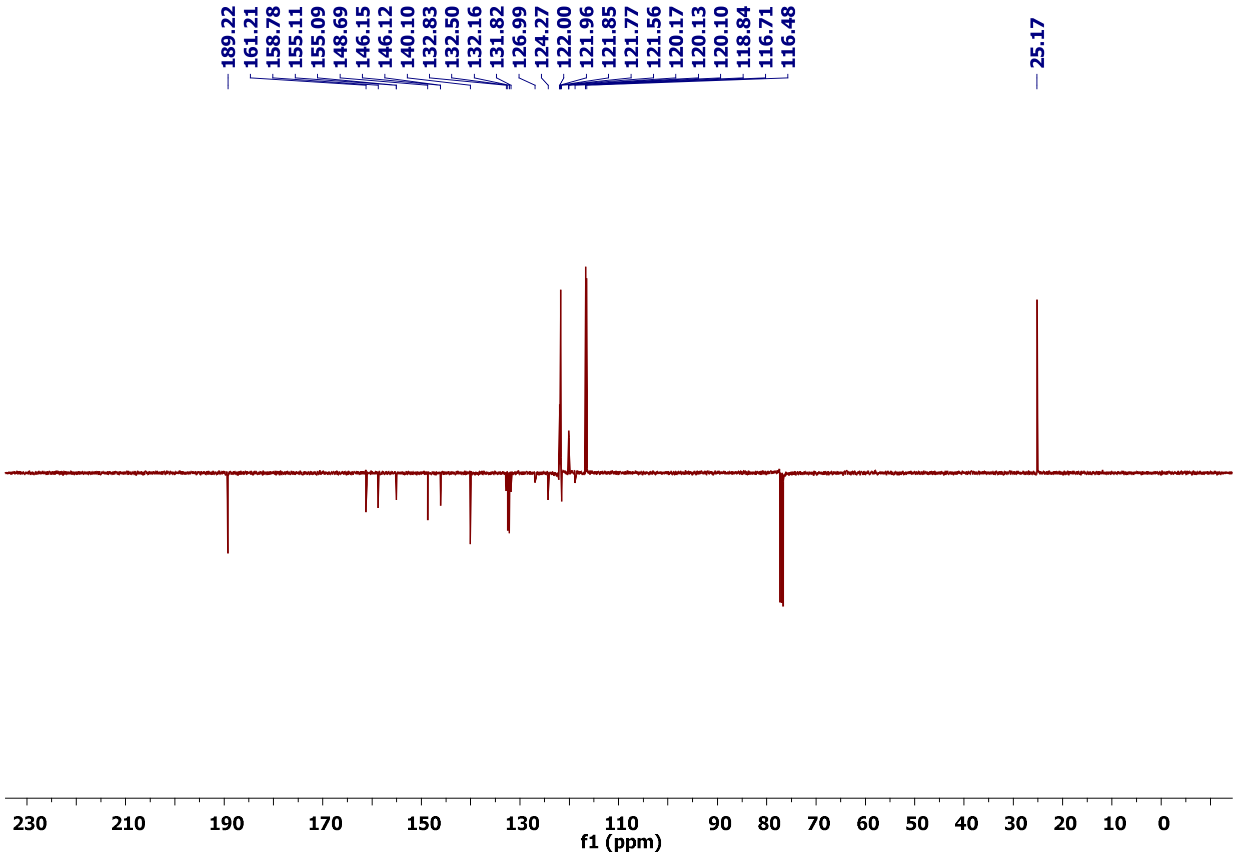

^{1}H-^{1}H gDQCOSY NMR ($CDCl_3$) spectrum of (Z)-1-(4-(3,5-bis(trifluoromethyl)phenyl)-5-((4-fluorophenyl)imino)-4,5-dihydro-1,3,4-thiadiazol-2-yl)ethan-1-one

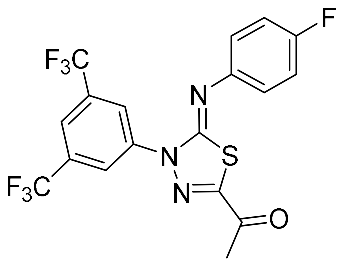

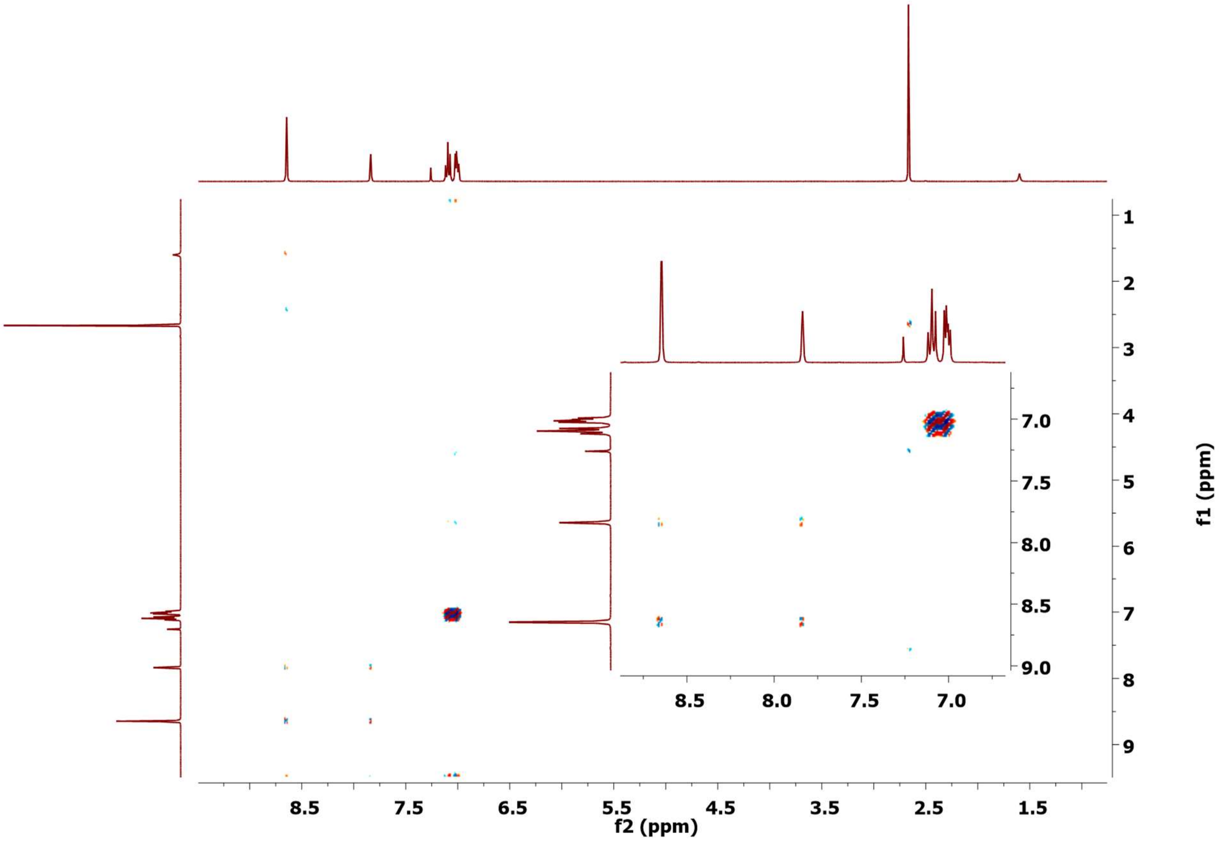

^{1}H-^{13}C-HSQC NMR ($CDCl_3$) spectrum of (Z)-1-(4-(3,5-bis(trifluoromethyl)phenyl)-5-((4-fluorophenyl)imino)-4,5-dihydro-1,3,4-thiadiazol-2-yl)ethan-1-one

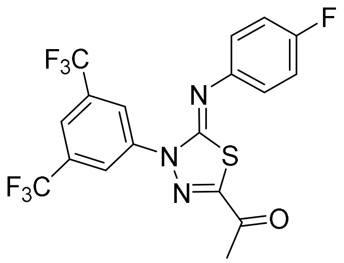

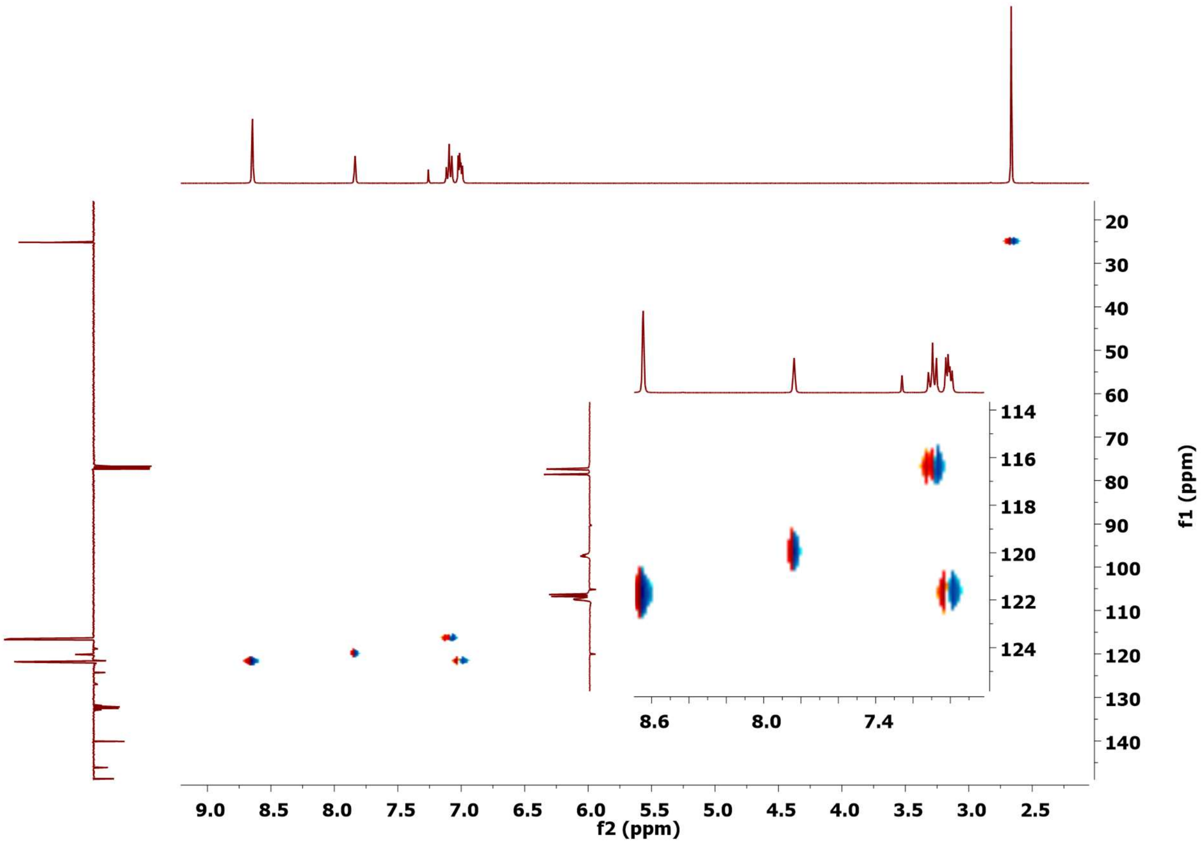

^{1}H-^{13}C-gHMBC NMR ($CDCl_3$) spectrum of (Z)-1-(4-(3,5-bis(trifluoromethyl)phenyl)-5-((4-fluorophenyl)imino)-4,5-dihydro-1,3,4-thiadiazol-2-yl)ethan-1-one

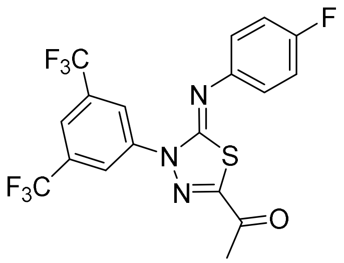

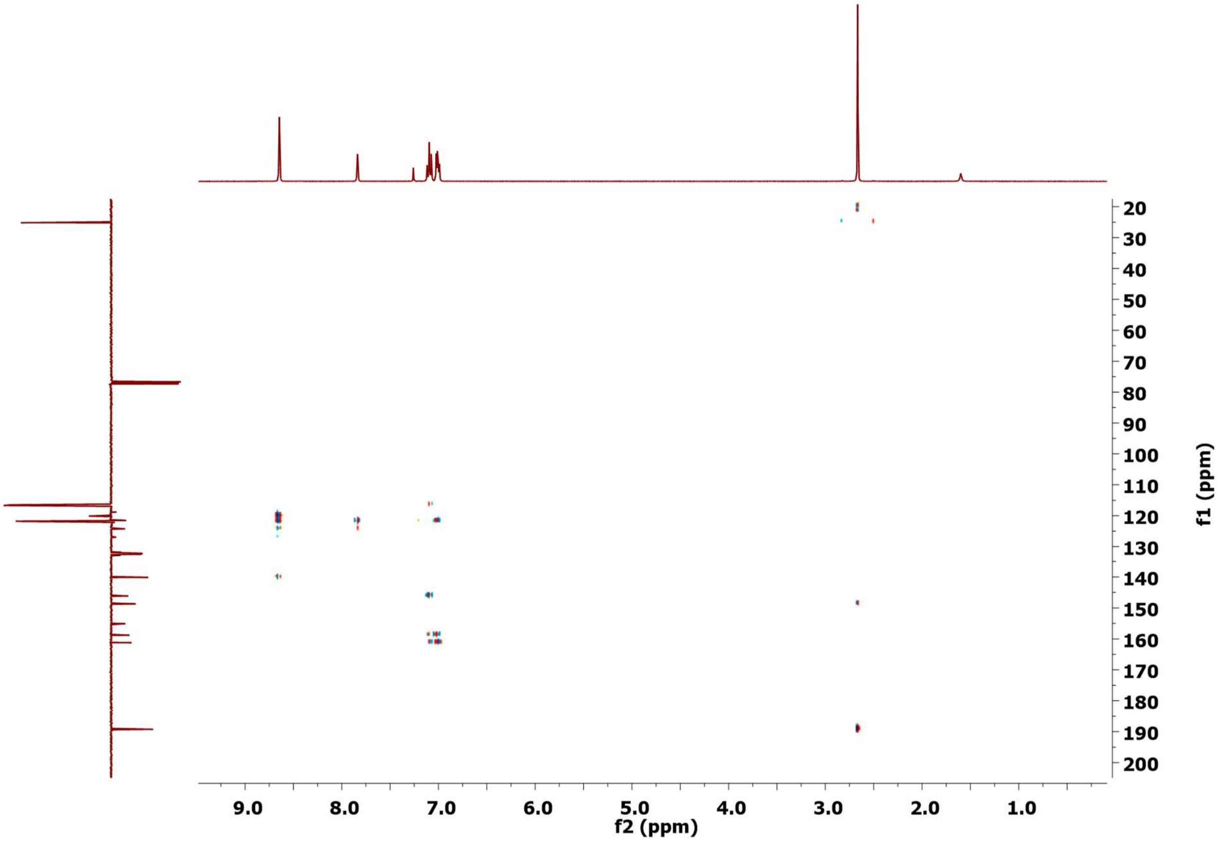

^{1}H NMR ($CDCl_3$) spectrum of (Z)-1-(5-((4-fluorophenyl)imino)-4-(4-iodophenyl)-4,5-dihydro-1,3,4-thiadiazol-2-yl)ethan-1-one

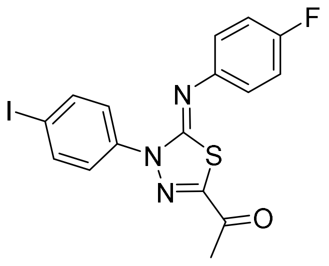

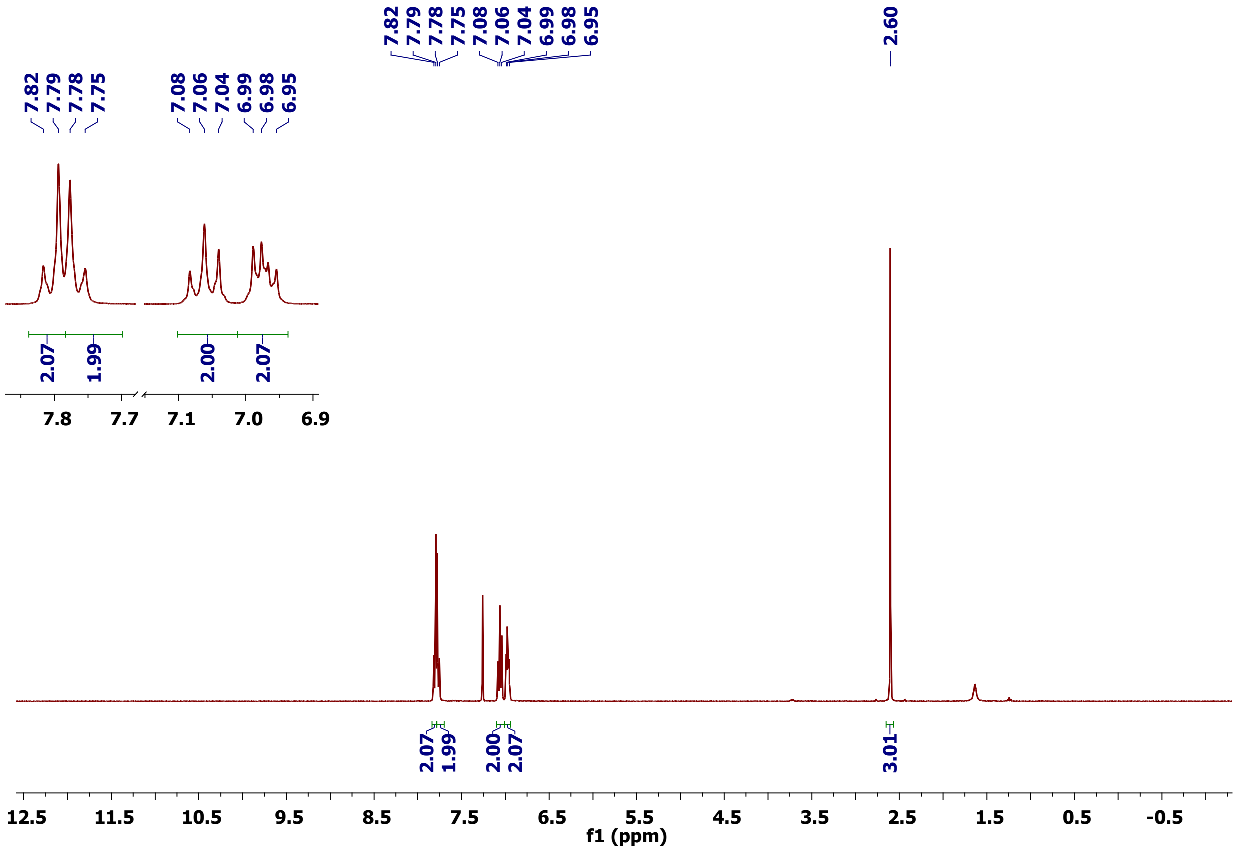

^{13}C NMR ($CDCl_3$) spectrum of (Z)-1-(5-((4-fluorophenyl)imino)-4-(4-iodophenyl)-4,5-dihydro-1,3,4-thiadiazol-2-yl)ethan-1-one

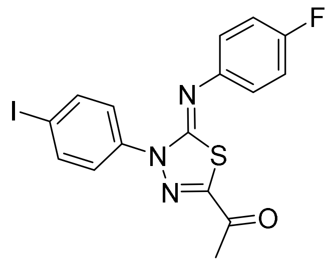

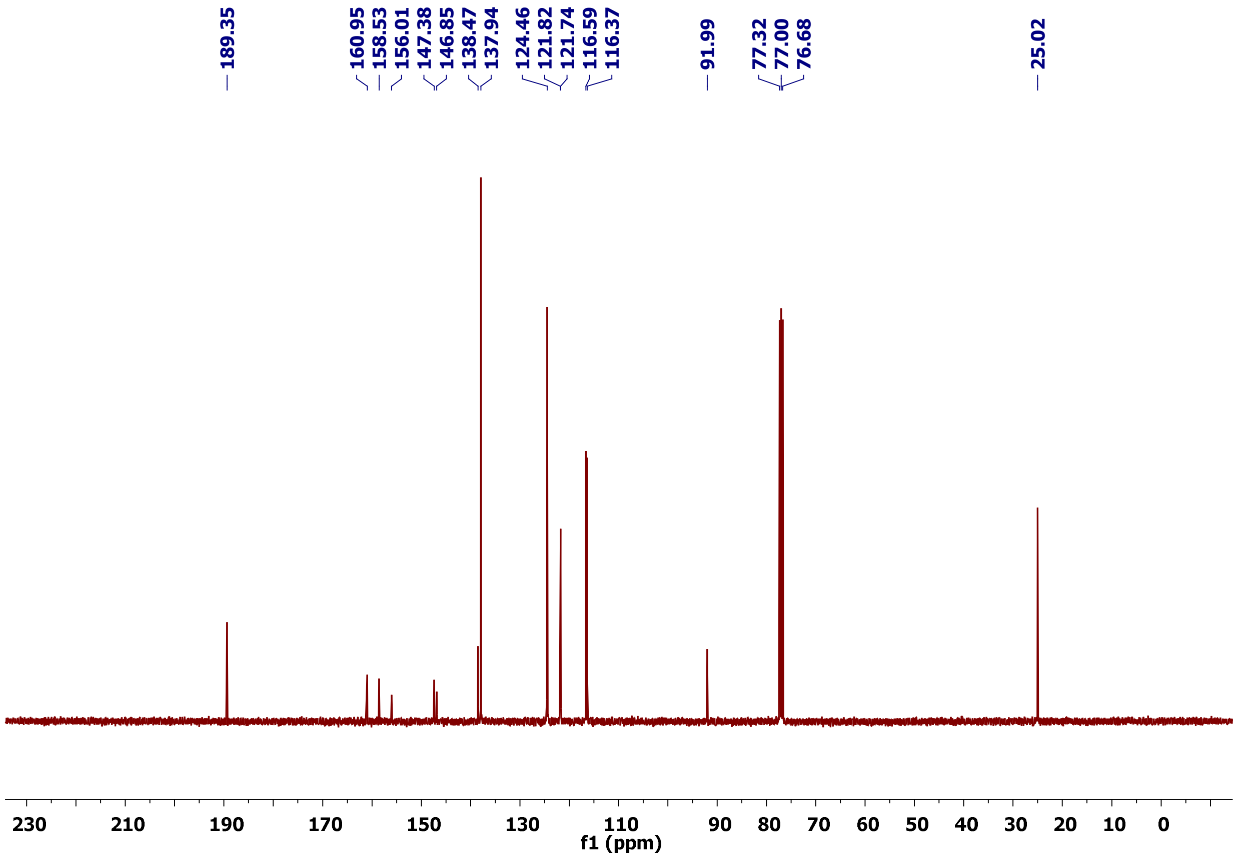

^{13}C CRAPT NMR ($CDCl_3$) spectrum of (Z)-1-(5-((4-fluorophenyl)imino)-4-(4-iodophenyl)-4,5-dihydro-1,3,4-thiadiazol-2-yl)ethan-1-one

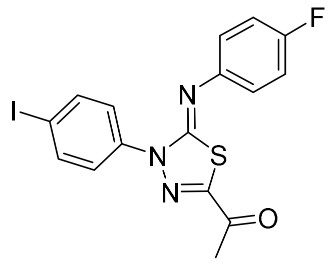

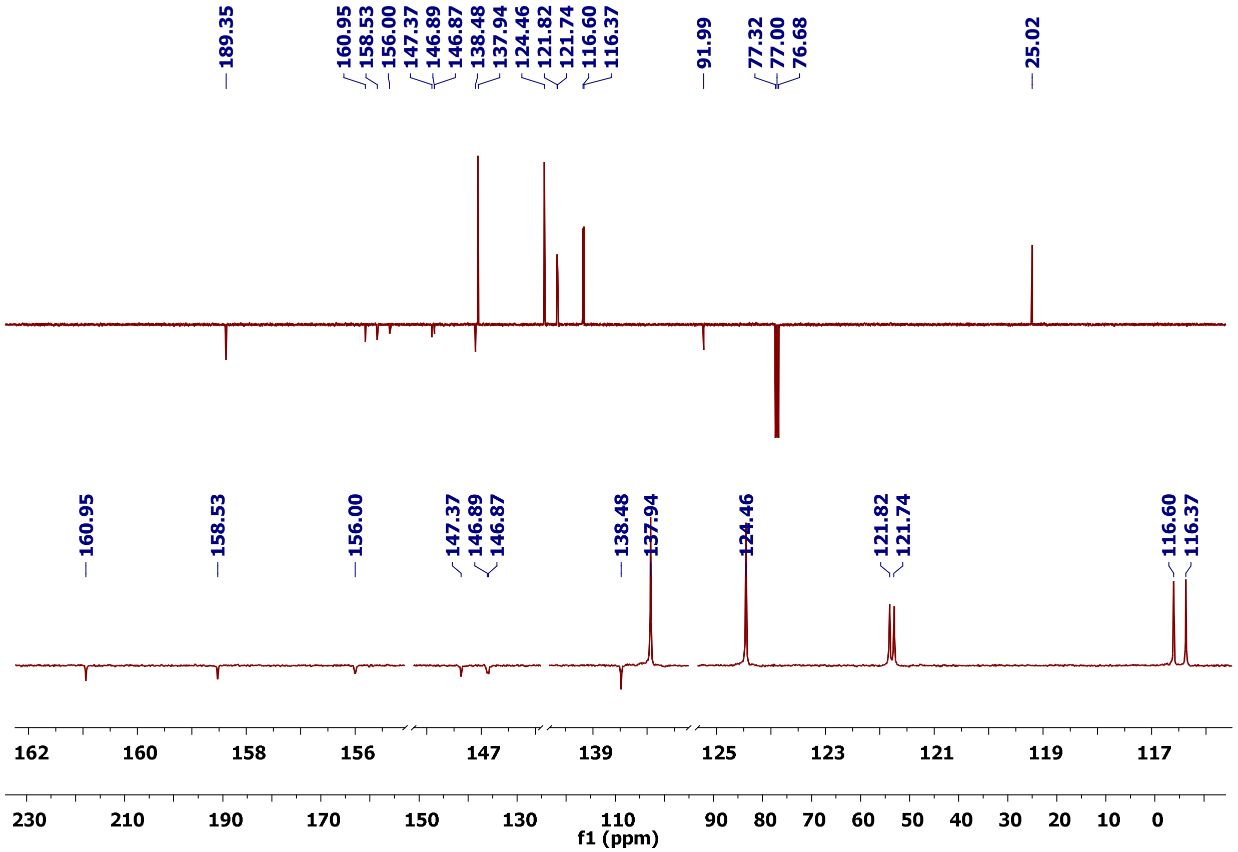

^1H-^1H gDQCOSY NMR ($CDCl_3$) spectrum of (Z)-1-(5-((4-fluorophenyl)imino)-4-(4-iodophenyl)-4,5-dihydro-1,3,4-thiadiazol-2-yl)ethan-1-one

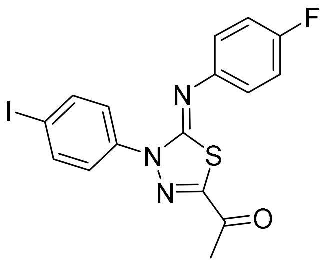

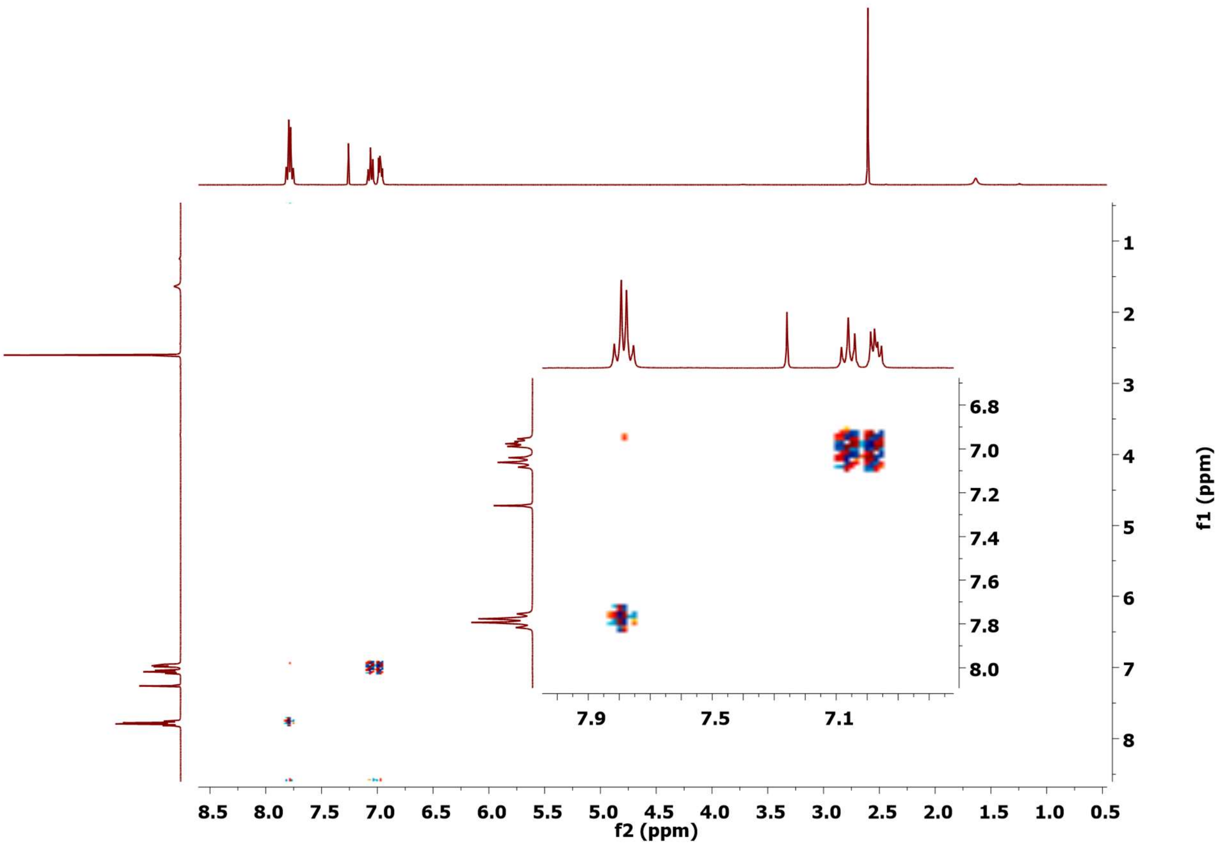

^{1}H-^{13}C-HSQC NMR ($CDCl_3$) spectrum of (Z)-1-(5-((4-fluorophenyl)imino)-4-(4-iodophenyl)-4,5-dihydro-1,3,4-thiadiazol-2-yl)ethan-1-one

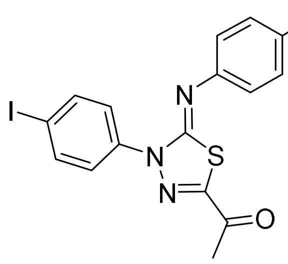

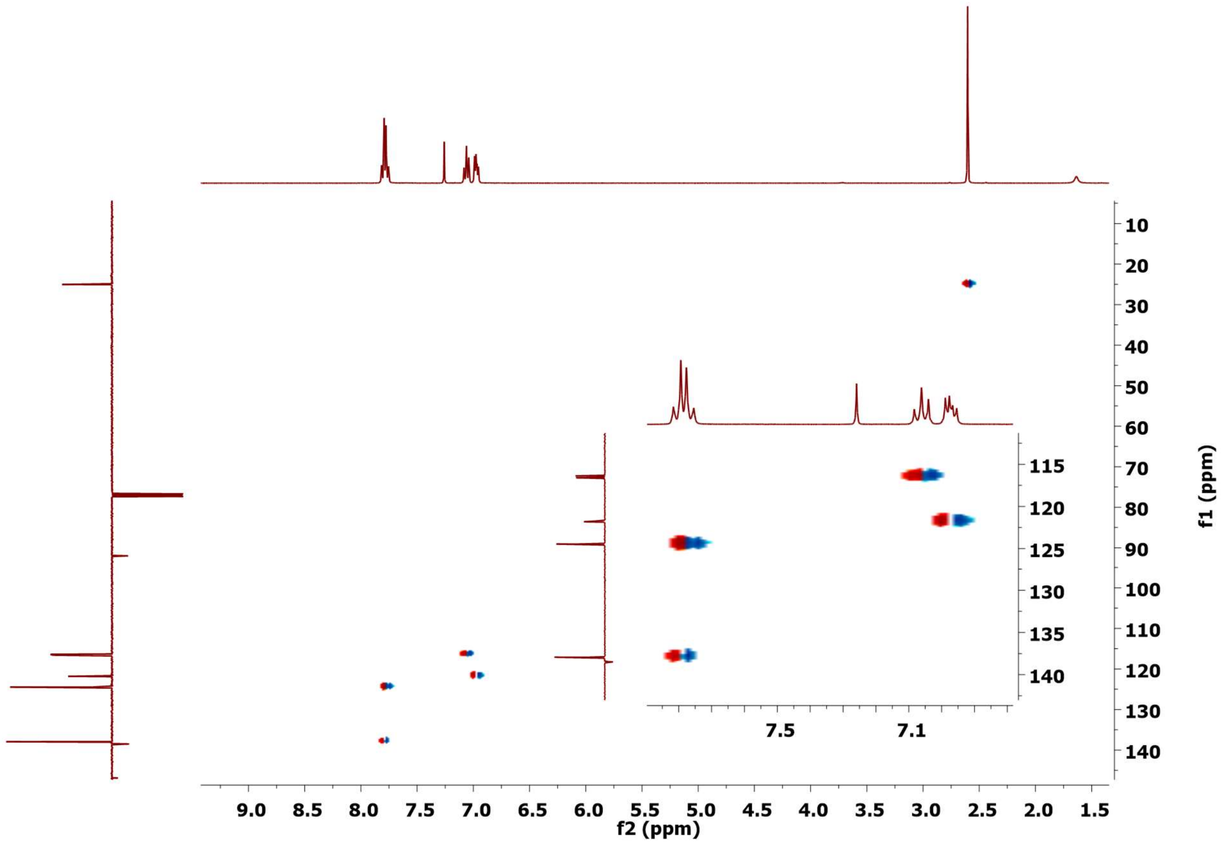

^{1}H-^{13}C-gHMBC NMR ($CDCl_3$) spectrum of (Z)-1-(5-((4-fluorophenyl)imino)-4-(4-iodophenyl)-4,5-dihydro-1,3,4-thiadiazol-2-yl)ethan-1-one

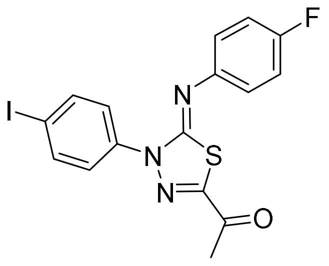

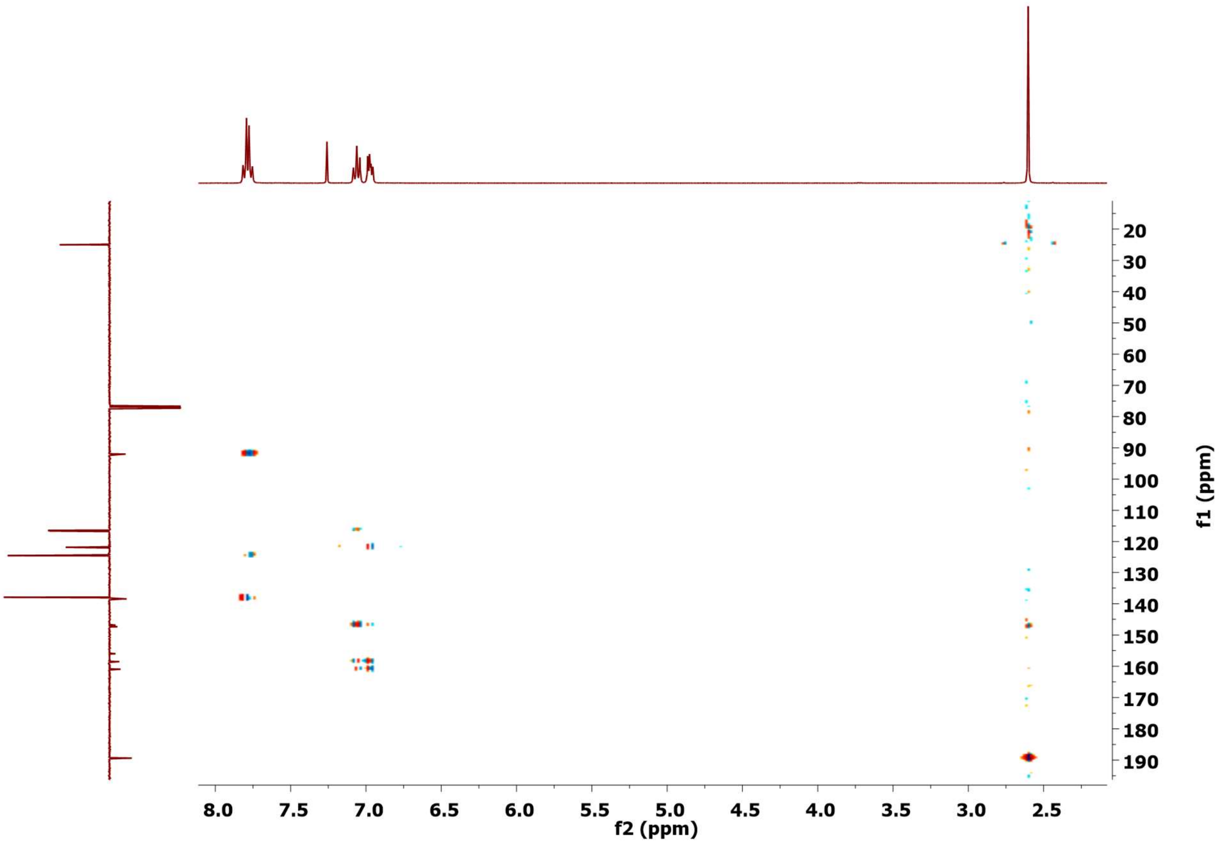

^{1}H NMR ($CDCl_3$) spectrum of (Z)-1-(4-(4-bromophenyl)-5-((4-fluorophenyl)imino)-4,5-dihydro-1,3,4-thiadiazol-2-yl)ethan-1-one

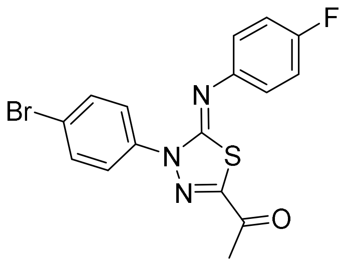

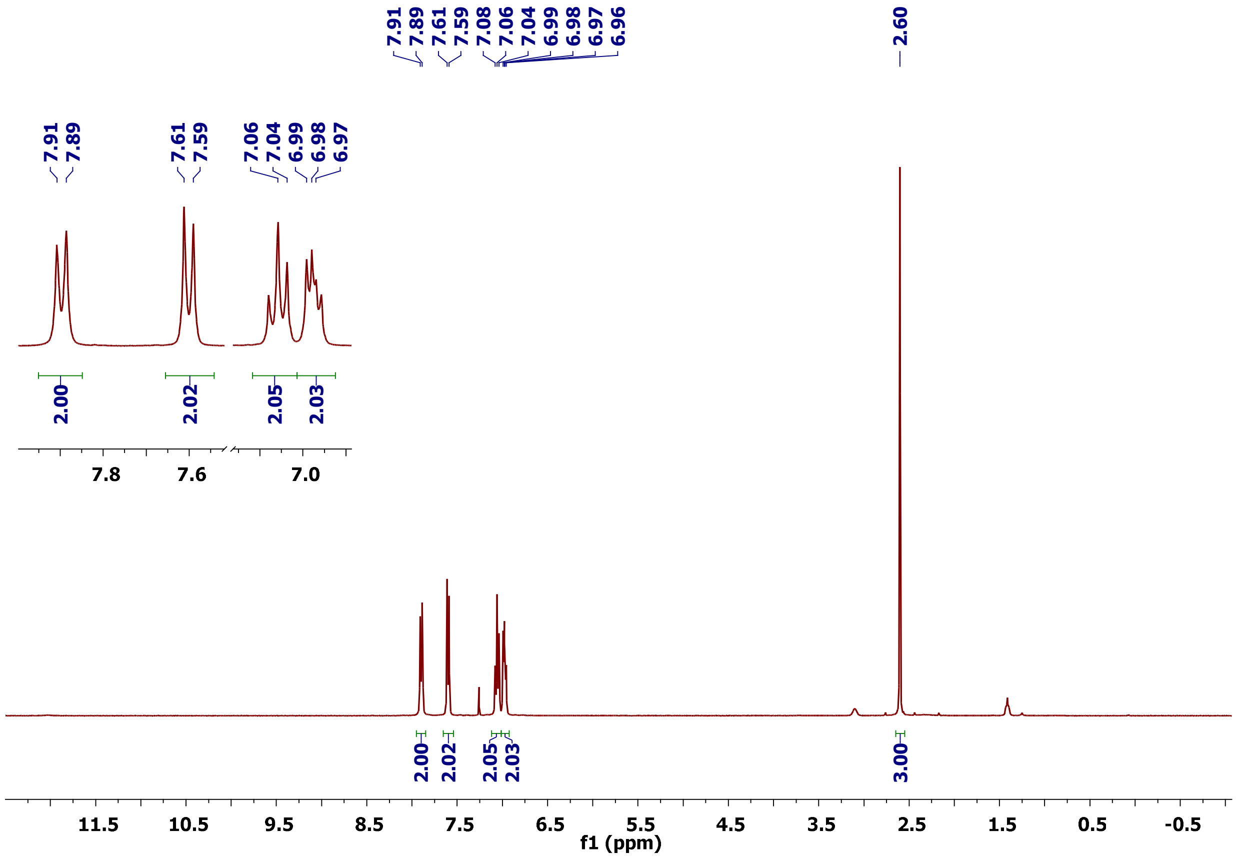

^{13}C NMR ($CDCl_3$) spectrum of (Z)-1-(4-(4-bromophenyl)-5-((4-fluorophenyl)imino)-4,5-dihydro-1,3,4-thiadiazol-2-yl)ethan-1-one

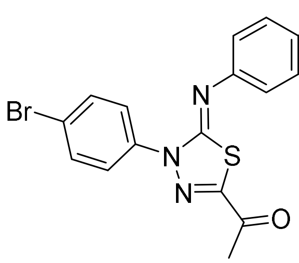

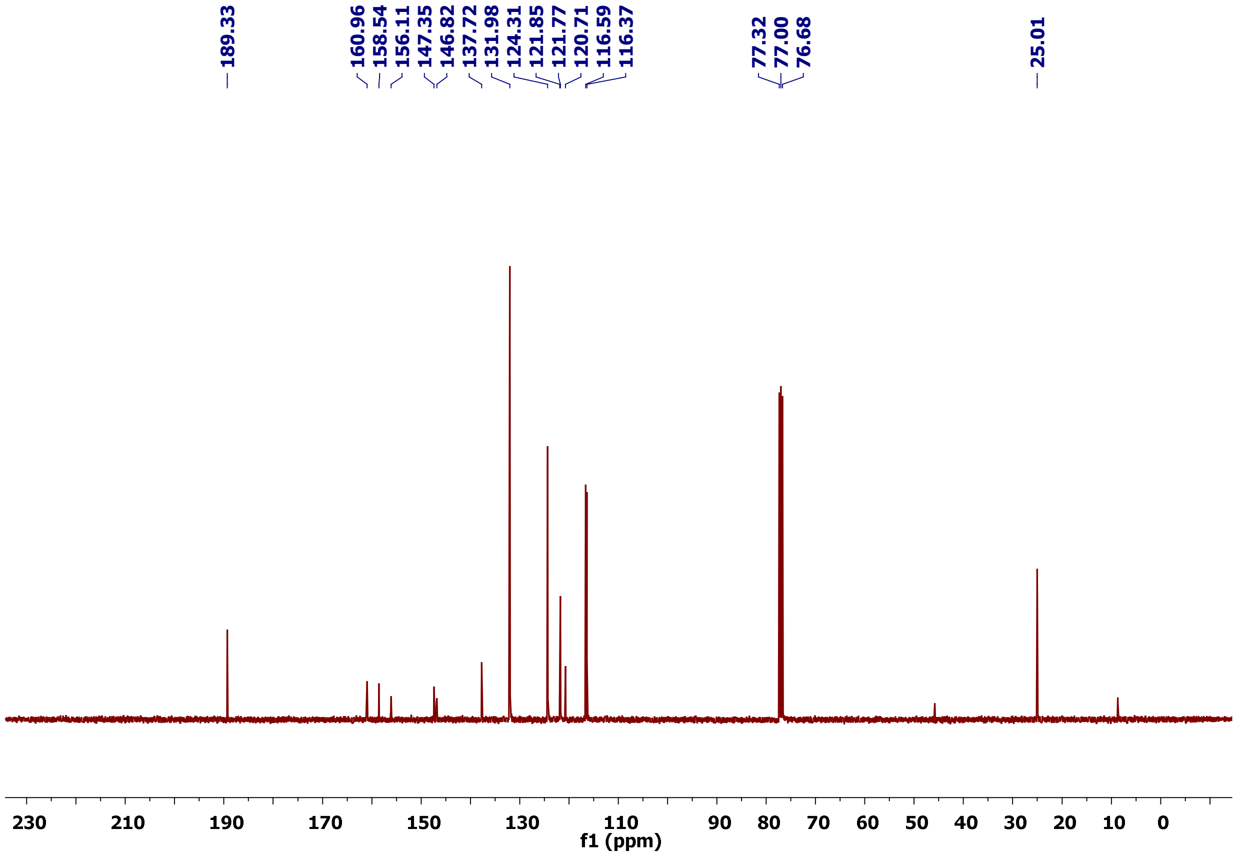

^{13}C CRAPT NMR ($CDCl_3$) spectrum of (Z)-1-(4-(4-bromophenyl)-5-((4-fluorophenyl)imino)-4,5-dihydro-1,3,4-thiadiazol-2-yl)ethan-1-one

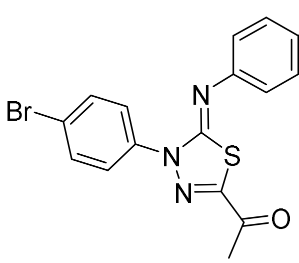

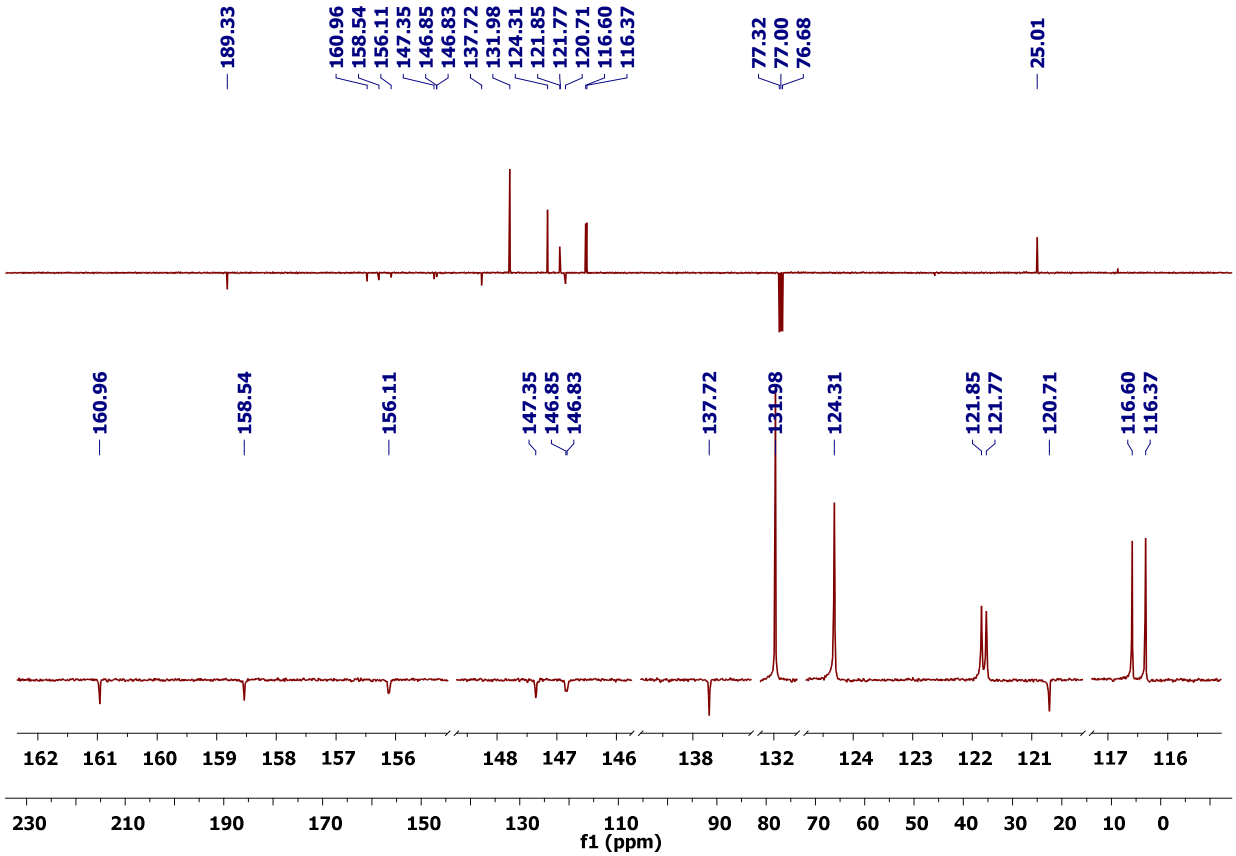

^{1}H-^{1}H gDQCOSY NMR ($CDCl_3$) spectrum of (Z)-1-(4-(4-bromophenyl)-5-((4-fluorophenyl)imino)-4,5-dihydro-1,3,4-thiadiazol-2-yl)ethan-1-one

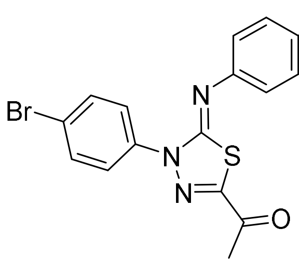

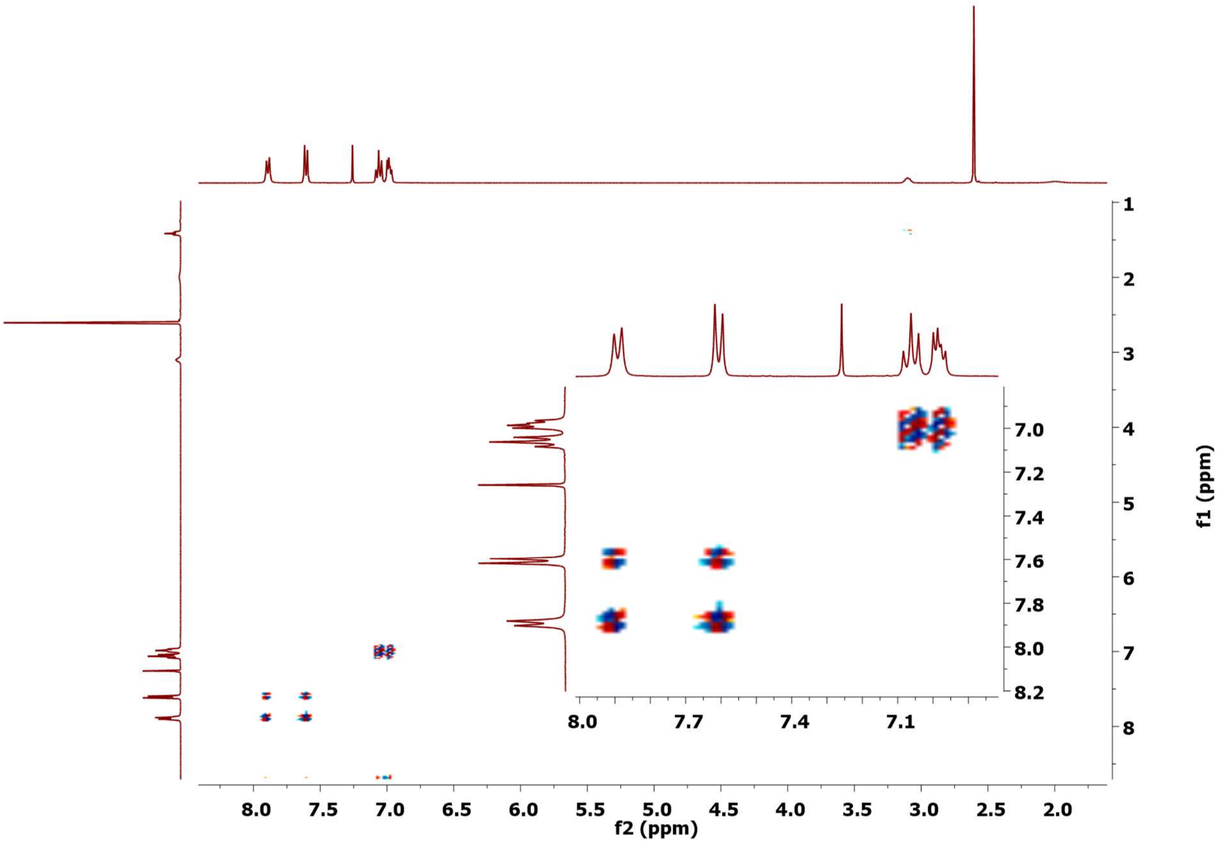

^{1}H-^{13}C-HSQC NMR ($CDCl_3$) spectrum of (Z)-1-(4-(4-bromophenyl)-5-((4-fluorophenyl)imino)-4,5-dihydro-1,3,4-thiadiazol-2-yl)ethan-1-one

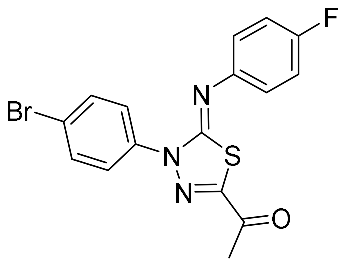

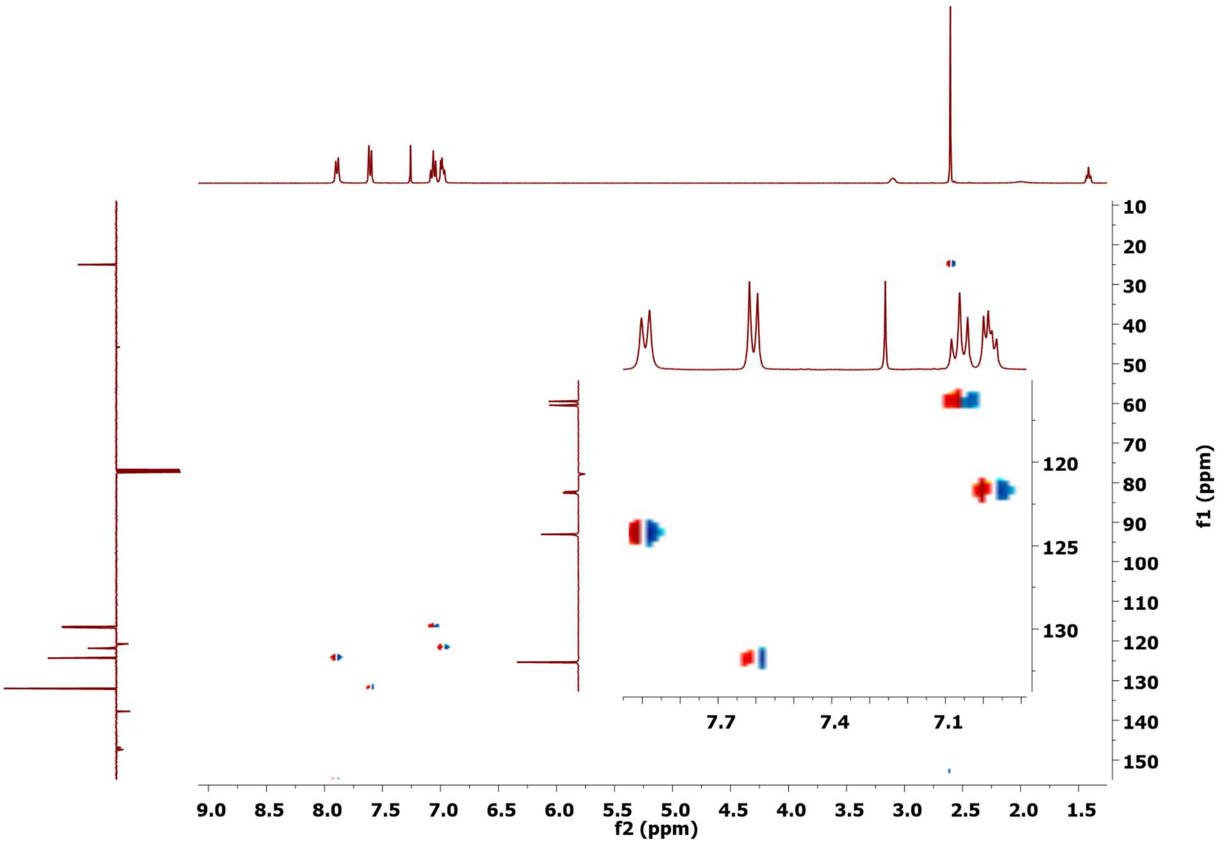

^{1}H-^{13}C-gHMBC NMR ($CDCl_3$) spectrum of (Z)-1-(4-(4-bromophenyl)-5-((4-fluorophenyl)imino)-4,5-dihydro-1,3,4-thiadiazol-2-yl)ethan-1-one

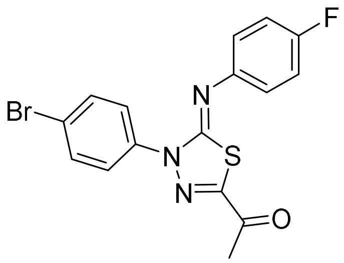

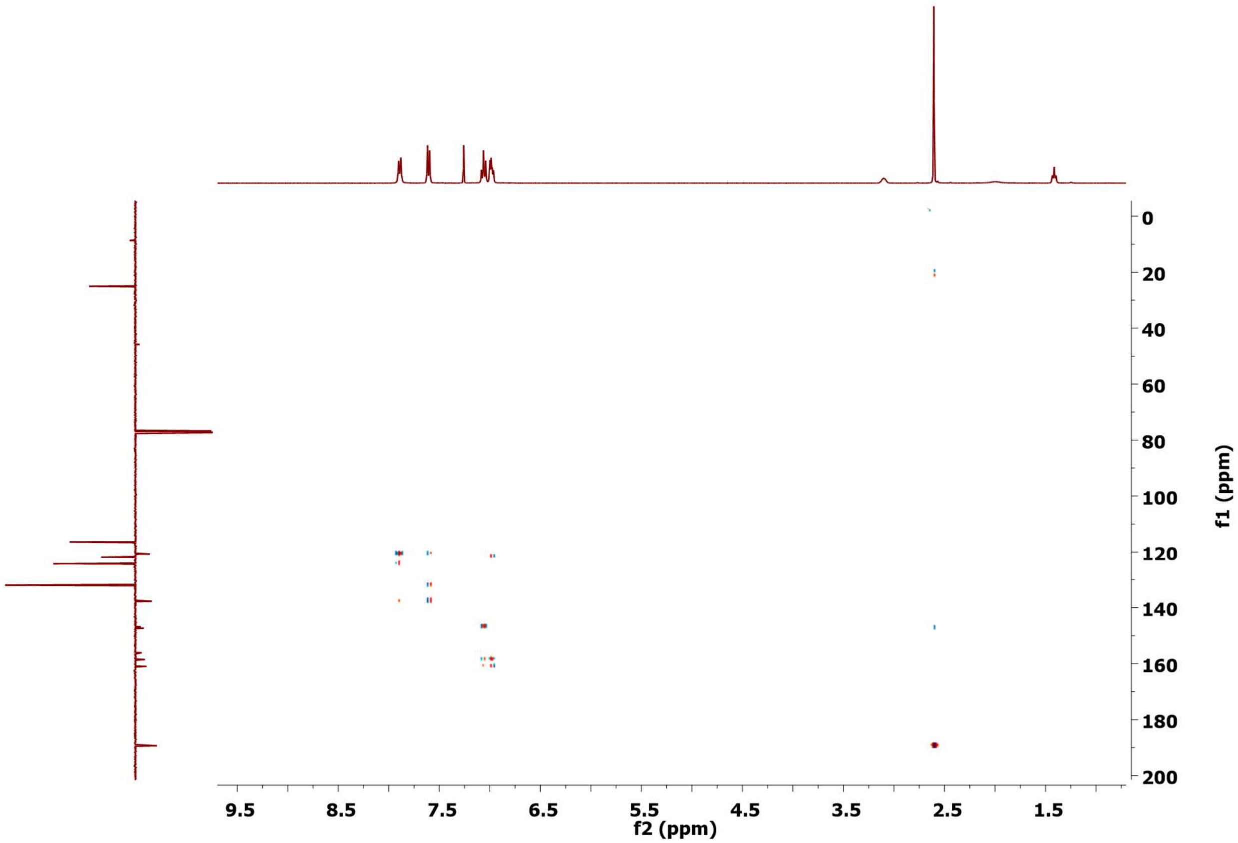

^{1}H NMR ($CDCl_3$) spectrum of (Z)-1-(4-(4-chlorophenyl)-5-((4-fluorophenyl)imino)-4,5-dihydro-1,3,4-thiadiazol-2-yl)ethan-1-one

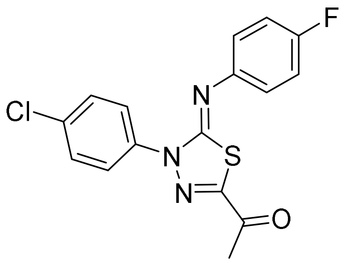

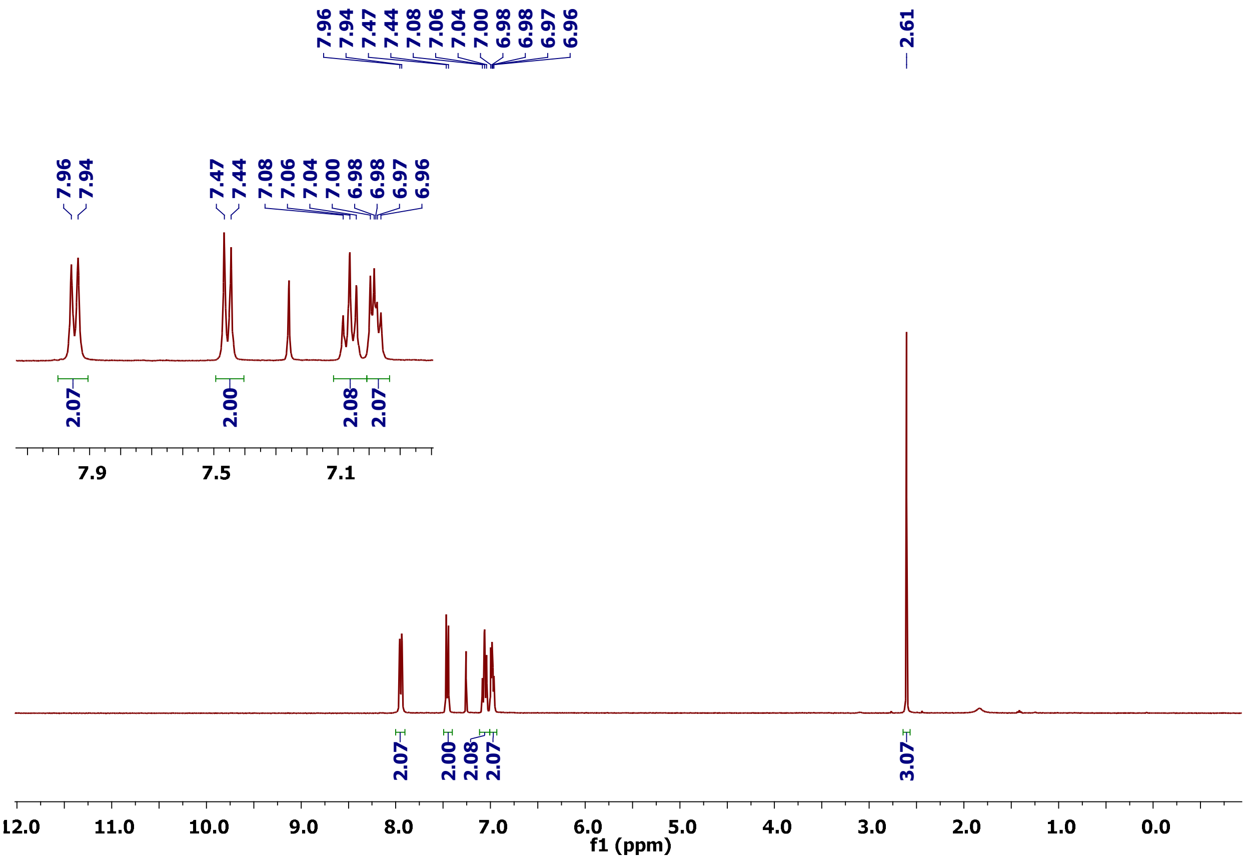

^{13}C NMR ($CDCl_3$) spectrum of (Z)-1-(4-(4-chlorophenyl)-5-((4-fluorophenyl)imino)-4,5-dihydro-1,3,4-thiadiazol-2-yl)ethan-1-one

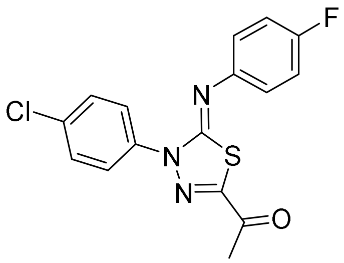

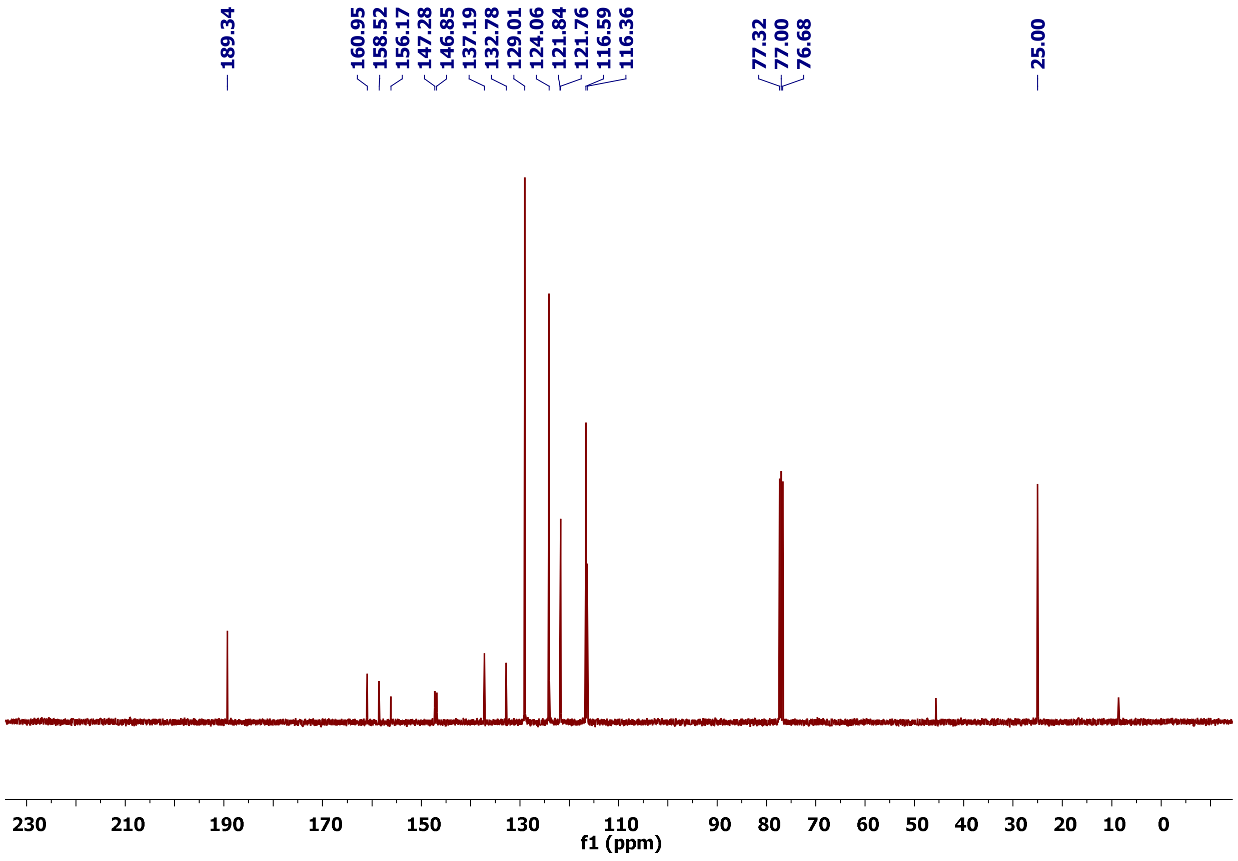

^{13}C CRAPT NMR ($CDCl_3$) spectrum of (Z)-1-(4-(4-chlorophenyl)-5-((4-fluorophenyl)imino)-4,5-dihydro-1,3,4-thiadiazol-2-yl)ethan-1-one

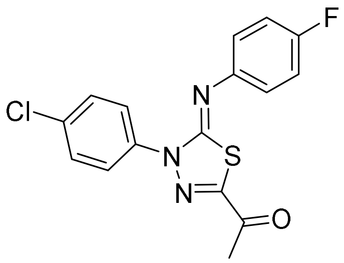

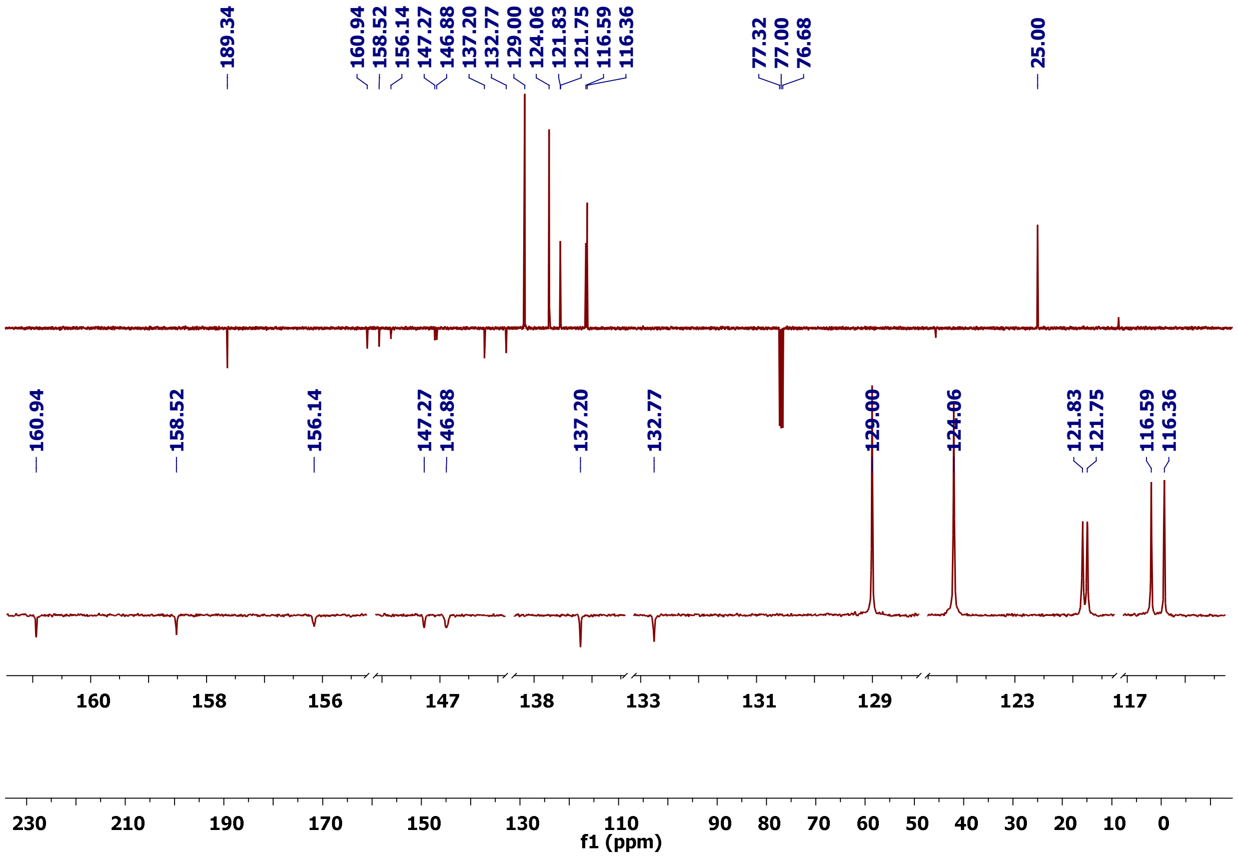

^{1}H-^{1}H gDQCOSY NMR ($CDCl_3$) spectrum of (Z)-1-(4-(4-chlorophenyl)-5-((4-fluorophenyl)imino)-4,5-dihydro-1,3,4-thiadiazol-2-yl)ethan-1-one

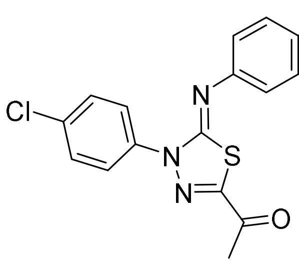

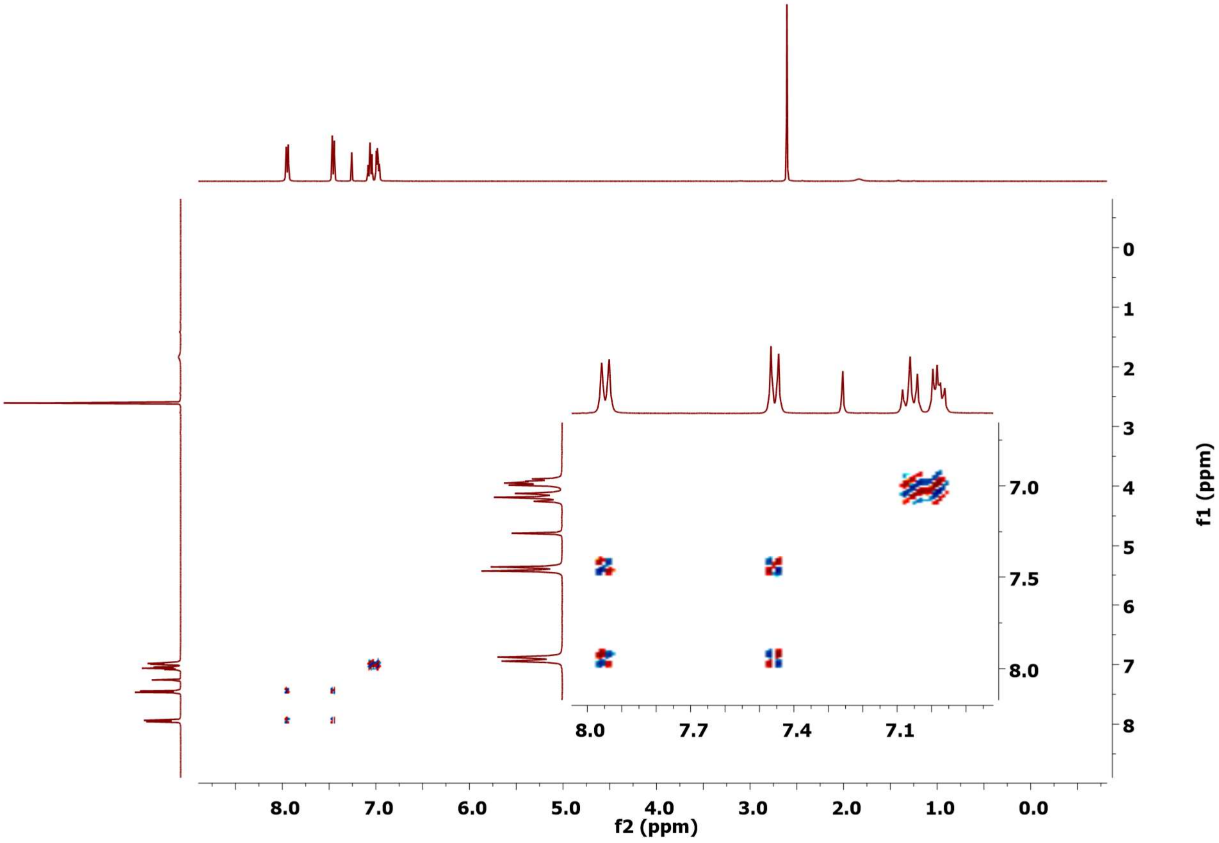

^{1}H-^{13}C-HSQC NMR ($CDCl_3$) spectrum of (Z)-1-(4-(4-chlorophenyl)-5-((4-fluorophenyl)imino)-4,5-dihydro-1,3,4-thiadiazol-2-yl)ethan-1-one

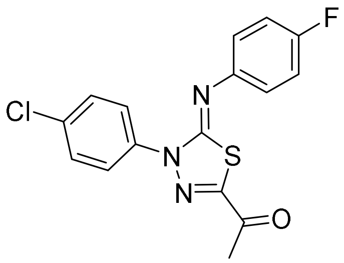

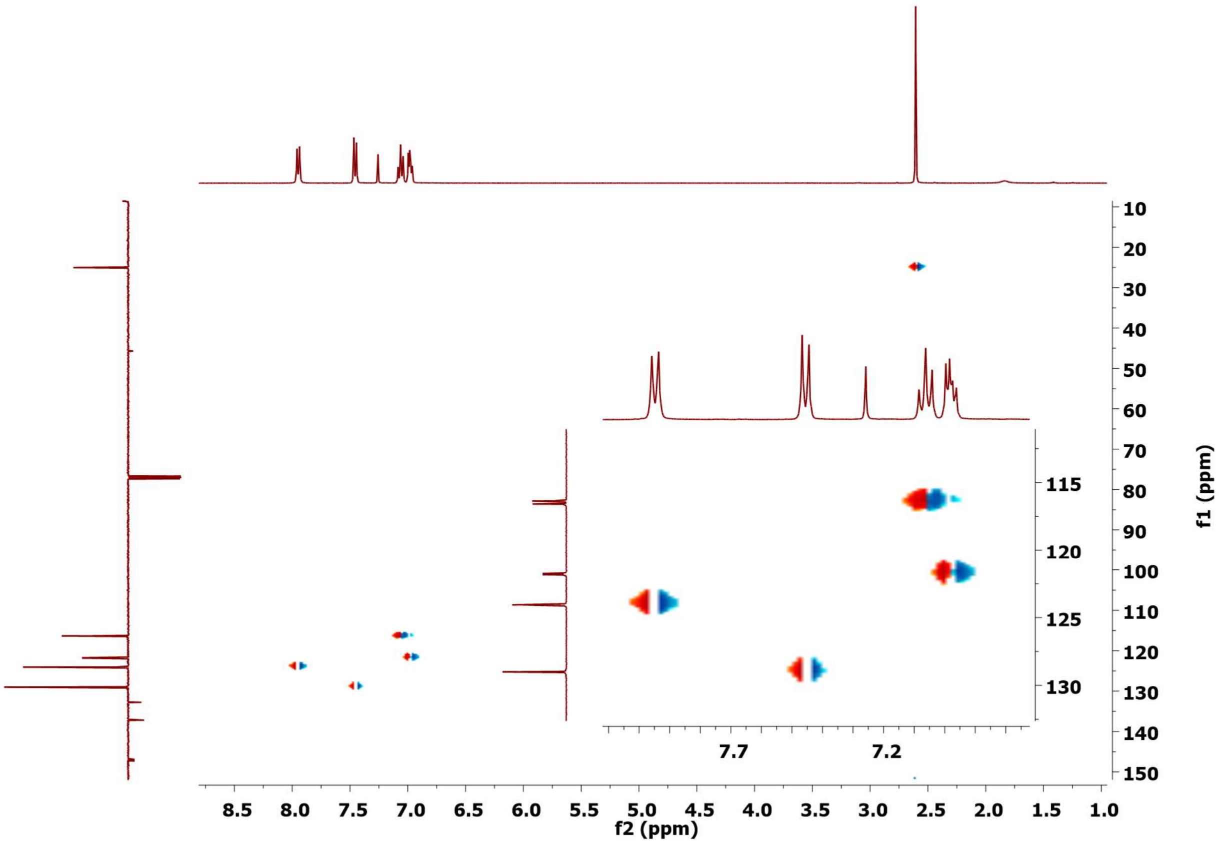

^{1}H-^{13}C-gHMBC NMR ($CDCl_3$) spectrum of (Z)-1-(4-(4-chlorophenyl)-5-((4-fluorophenyl)imino)-4,5-dihydro-1,3,4-thiadiazol-2-yl)ethan-1-one

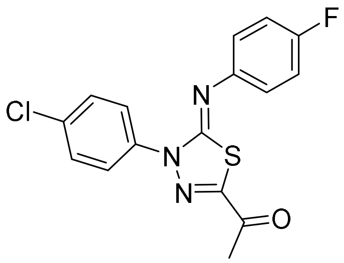

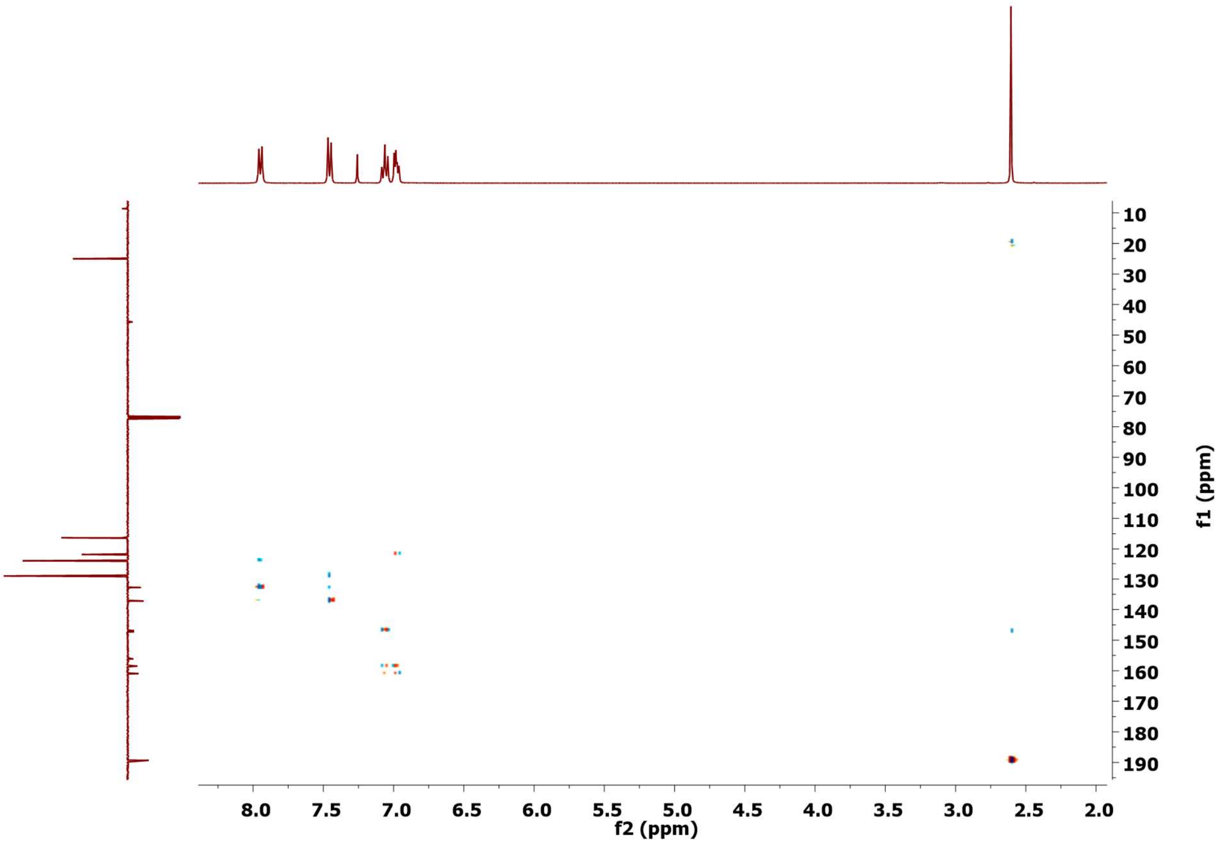

^{1}H NMR ($CDCl_3$) spectrum of (Z)-1-(5-((4-fluorophenyl)imino)-4-phenyl-4,5-dihydro-1,3,4-thiadiazol-2-yl)ethan-1-one

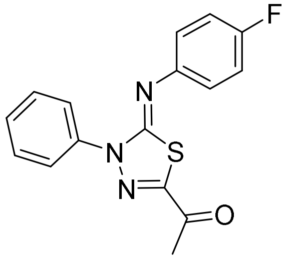

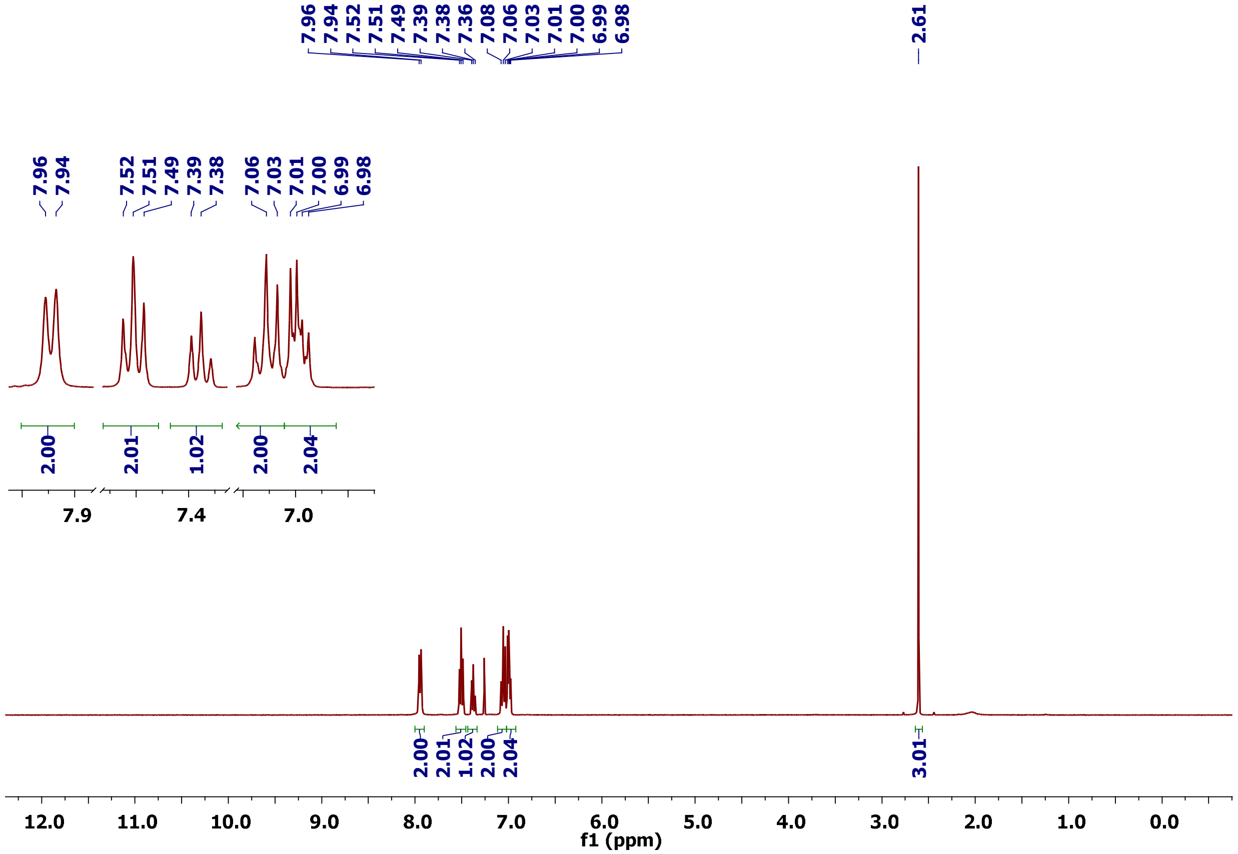

^{13}C NMR ($CDCl_3$) spectrum of (Z)-1-(5-((4-fluorophenyl)imino)-4-phenyl-4,5-dihydro-1,3,4-thiadiazol-2-yl)ethan-1-one

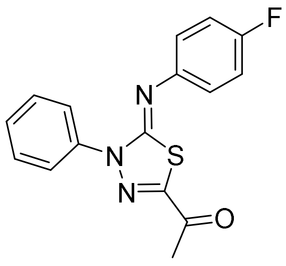

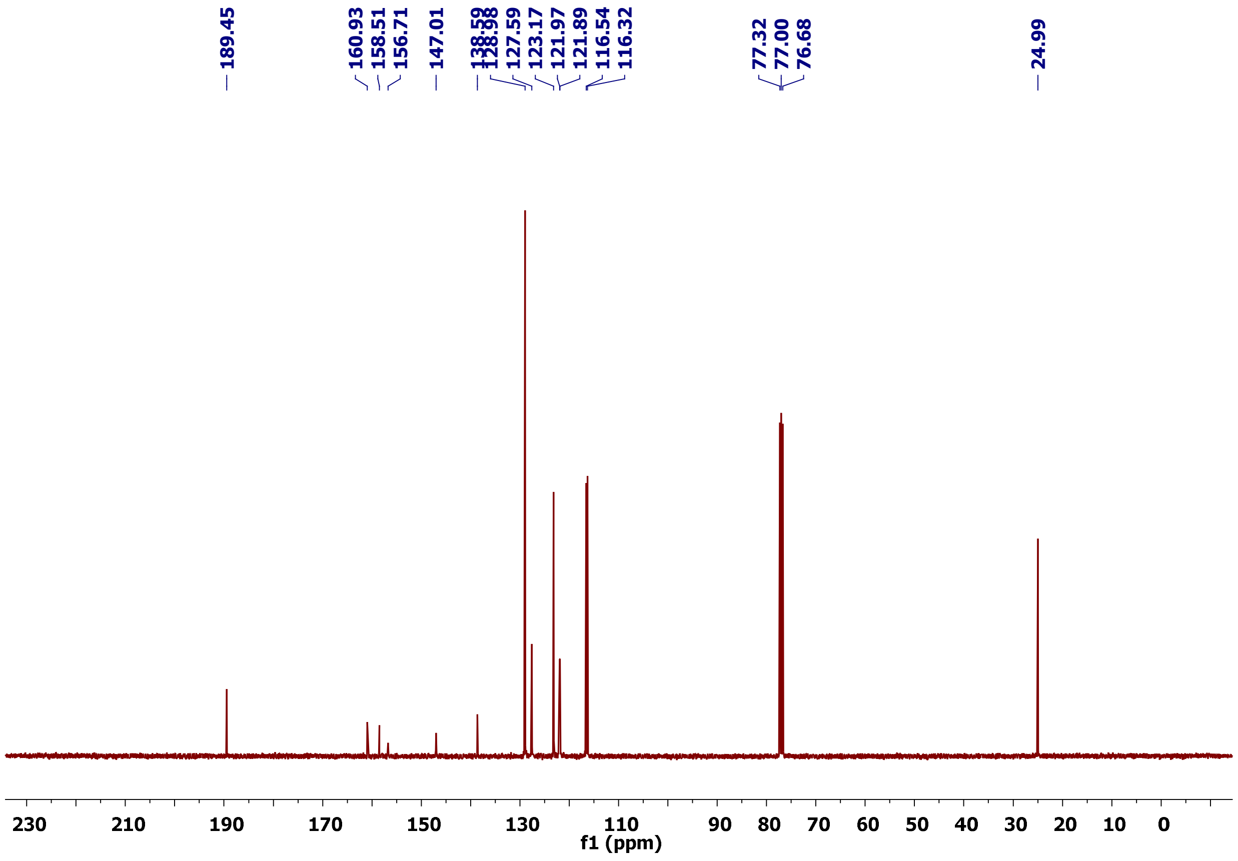

^{13}C CRAPT NMR ($CDCl_3$) spectrum of (Z)-1-(5-((4-fluorophenyl)imino)-4-phenyl-4,5-dihydro-1,3,4-thiadiazol-2-yl)ethan-1-one

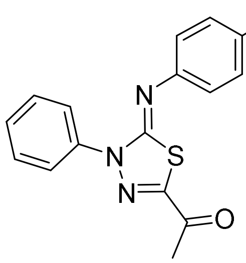

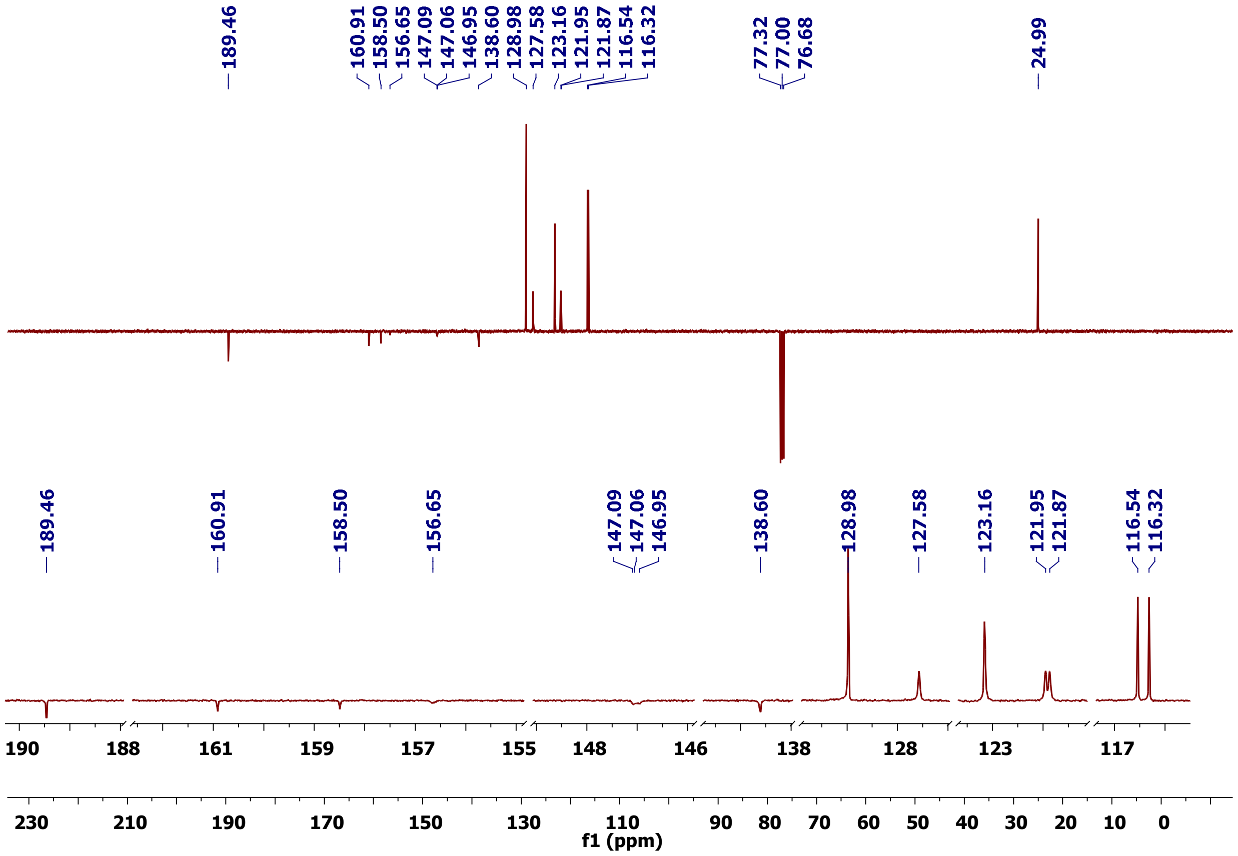

^1H-^1H gDQCOSY NMR ($CDCl_3$) spectrum of (Z)-1-(5-((4-fluorophenyl)imino)-4-phenyl-4,5-dihydro-1,3,4-thiadiazol-2-yl)ethan-1-one

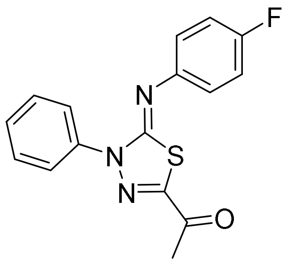

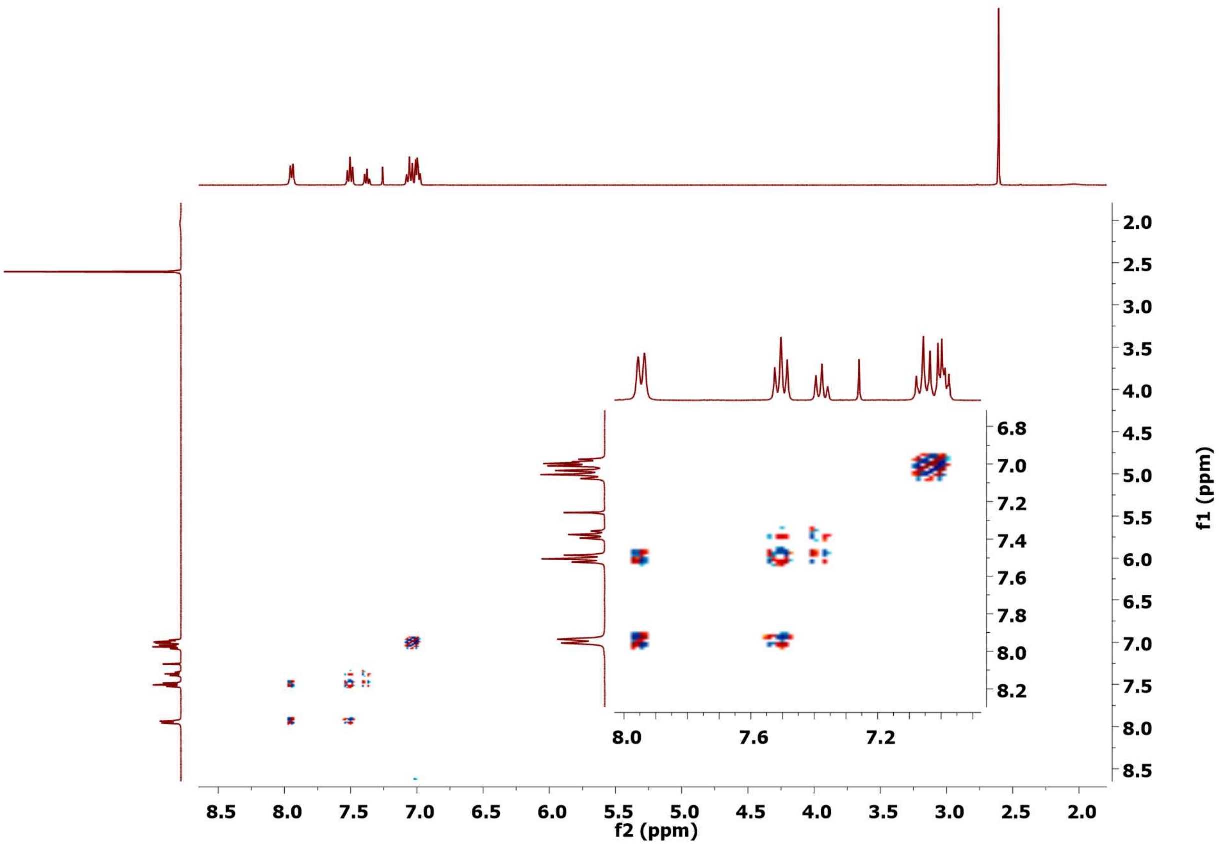

^{1}H-^{13}C-HSQC NMR ($CDCl_3$) spectrum of (Z)-1-(5-((4-fluorophenyl)imino)-4-phenyl-4,5-dihydro-1,3,4-thiadiazol-2-yl)ethan-1-one

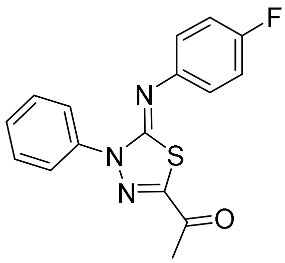

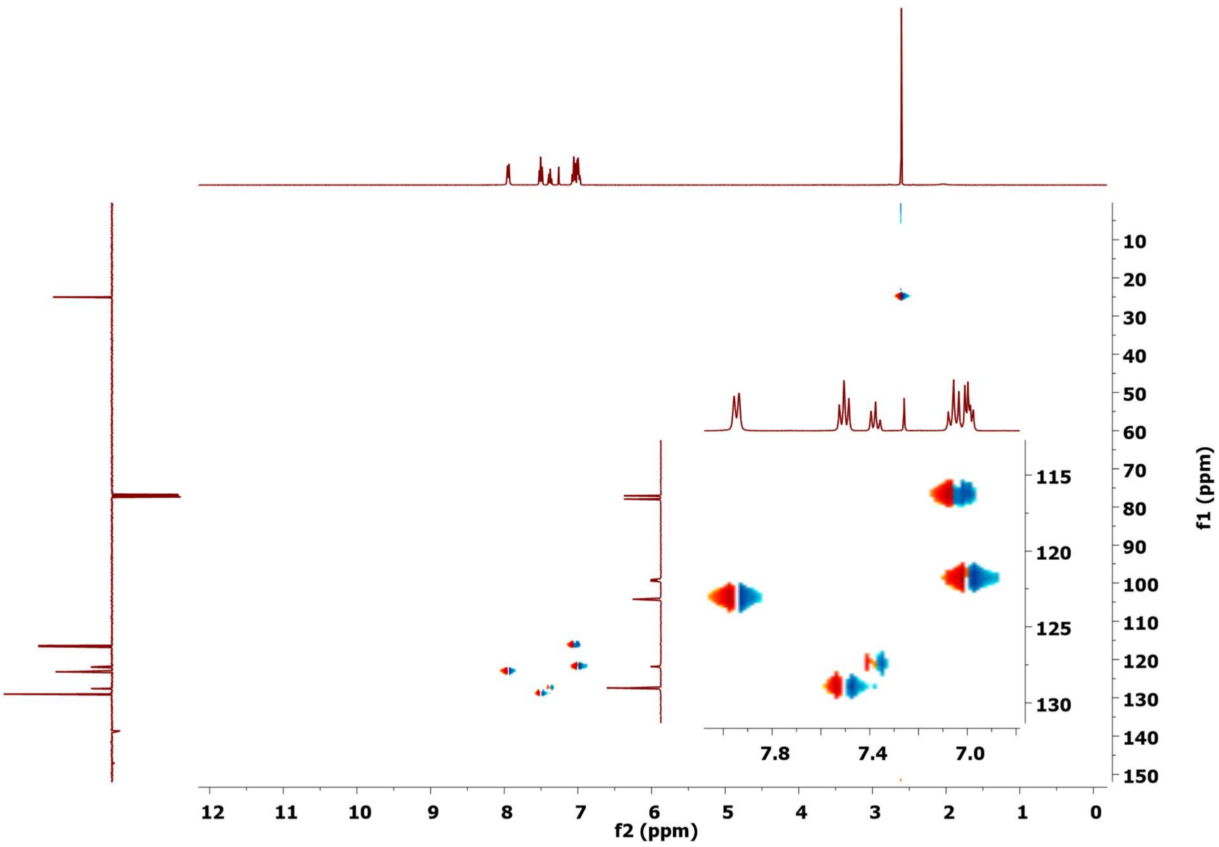

^{1}H-^{13}C-gHMBC NMR ($CDCl_3$) spectrum of (Z)-1-(5-((4-fluorophenyl)imino)-4-phenyl-4,5-dihydro-1,3,4-thiadiazol-2-yl)ethan-1-one

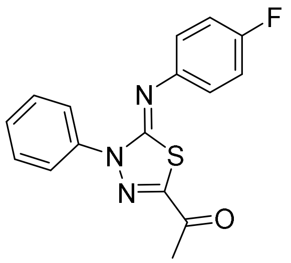

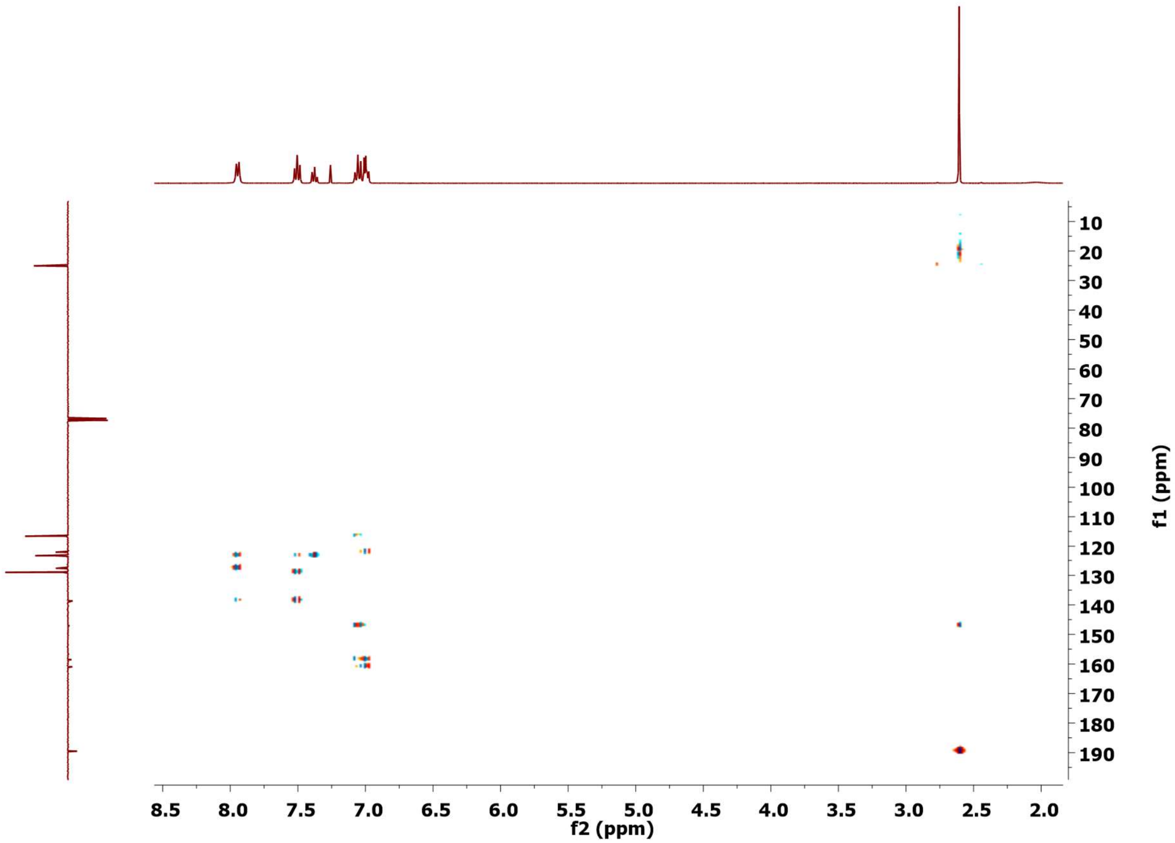

^{1}H NMR ($CDCl_3$) spectrum of (Z)-1-(5-((4-fluorophenyl)imino)-4-(p-tolyl)-4,5-dihydro-1,3,4-thiadiazol-2-yl)ethan-1-one

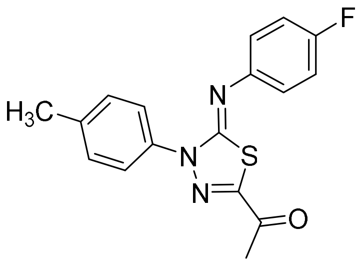

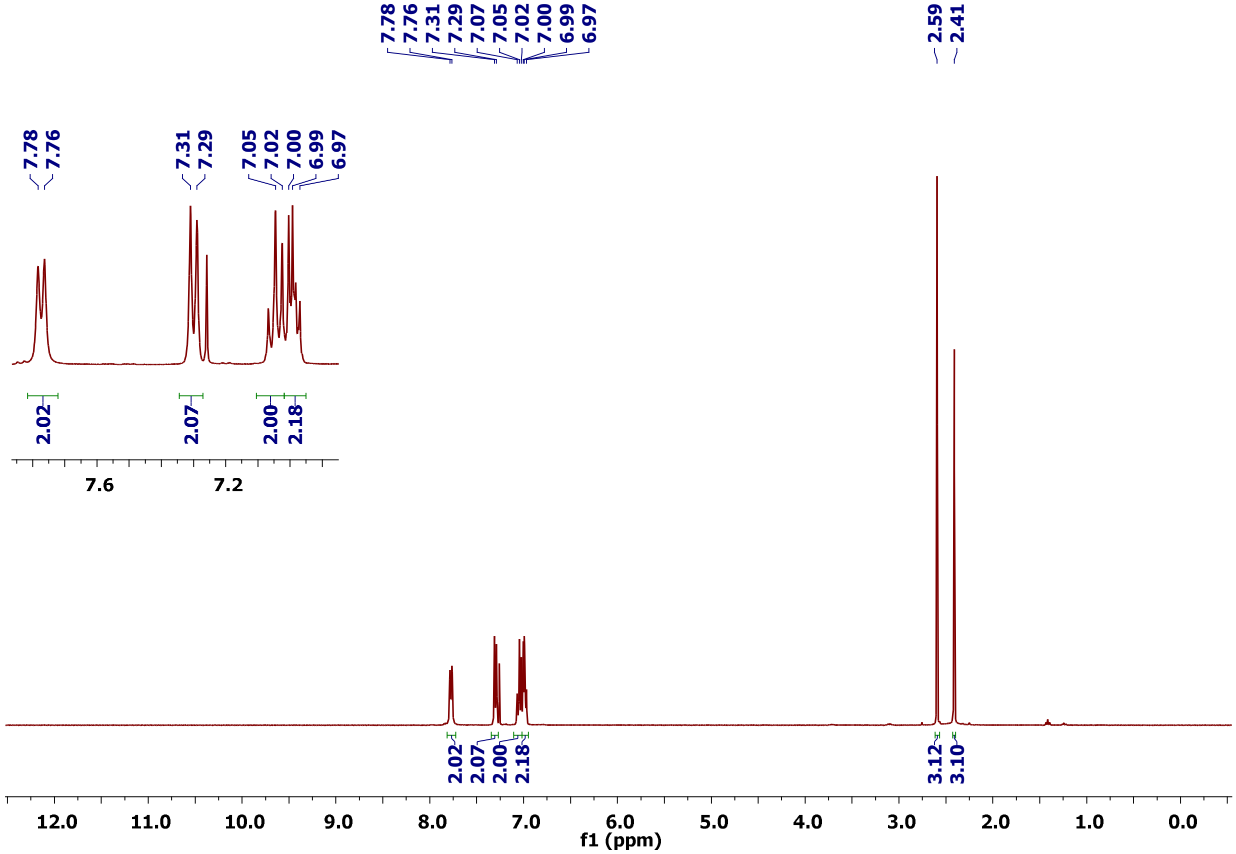

^{13}C NMR ($CDCl_3$) spectrum of (Z)-1-(5-((4-fluorophenyl)imino)-4-(p-tolyl)-4,5-dihydro-1,3,4-thiadiazol-2-yl)ethan-1-one

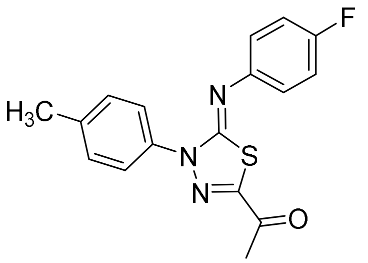

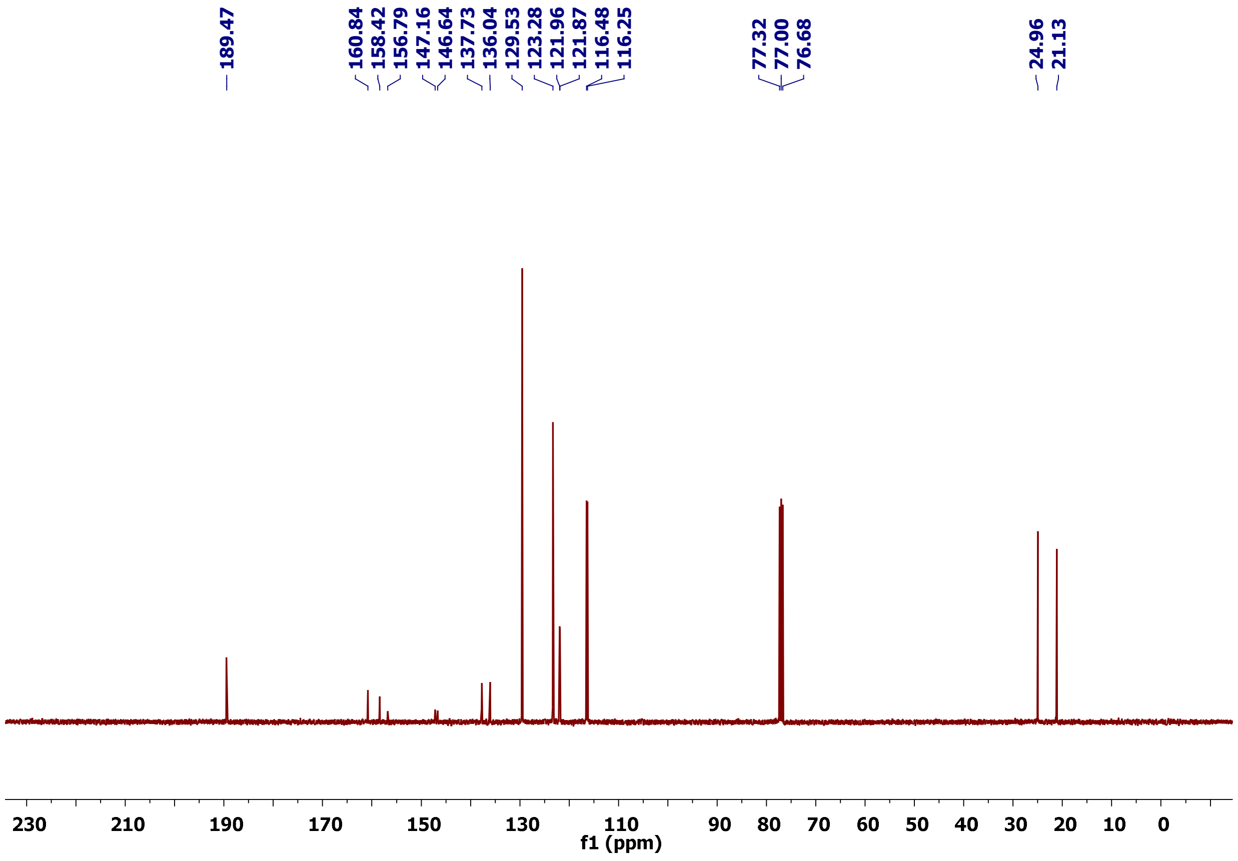

^{13}C CRAPT NMR ($CDCl_3$) spectrum of (Z)-1-(5-((4-fluorophenyl)imino)-4-(p-tolyl)-4,5-dihydro-1,3,4-thiadiazol-2-yl)ethan-1-one

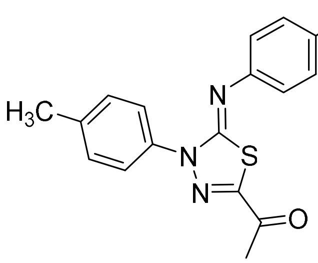

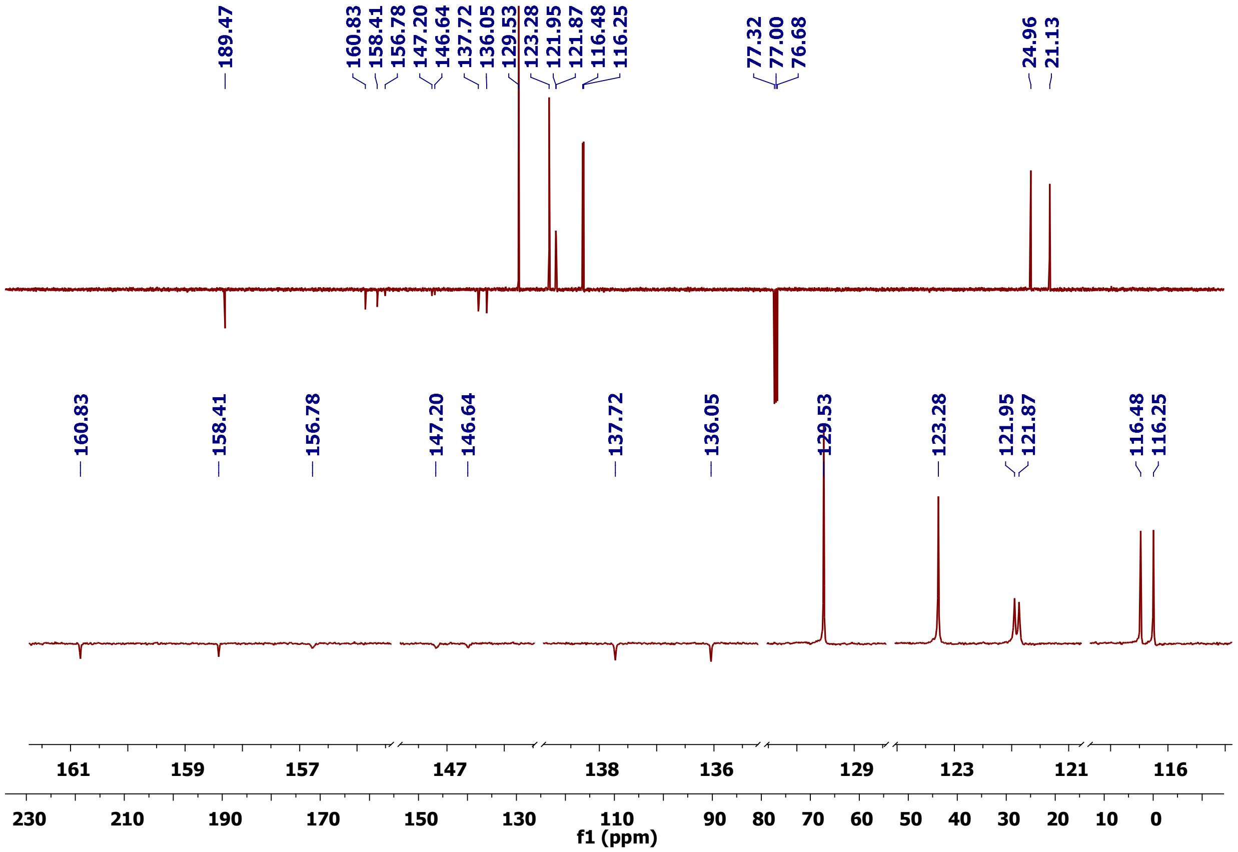

^{1}H-^{1}H gDQCOSY NMR ($CDCl_3$) spectrum of (Z)-1-(5-((4-fluorophenyl)imino)-4-(p-tolyl)-4,5-dihydro-1,3,4-thiadiazol-2-yl)ethan-1-one

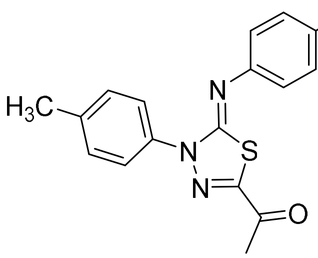

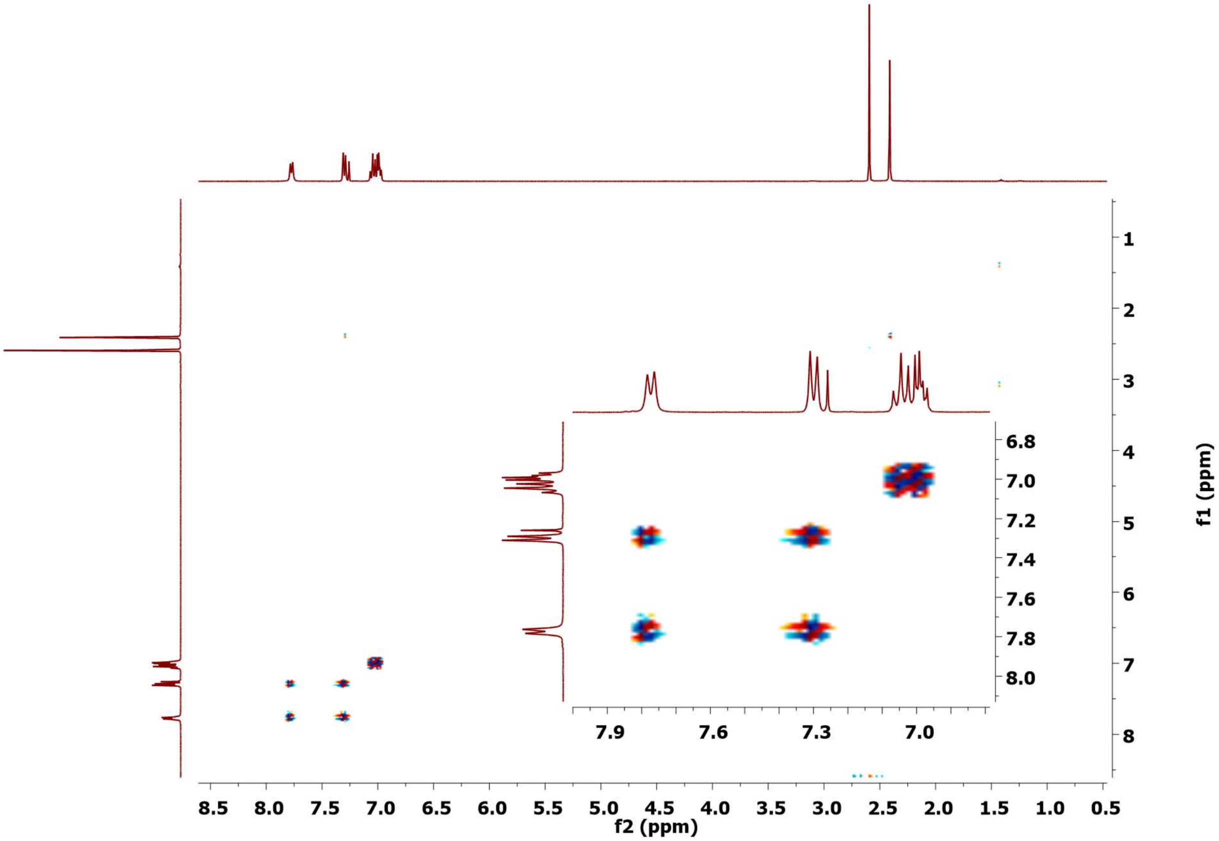

^{1}H-^{13}C-HSQC NMR ($CDCl_3$) spectrum of (Z)-1-(5-((4-fluorophenyl)imino)-4-(p-tolyl)-4,5-dihydro-1,3,4-thiadiazol-2-yl)ethan-1-one

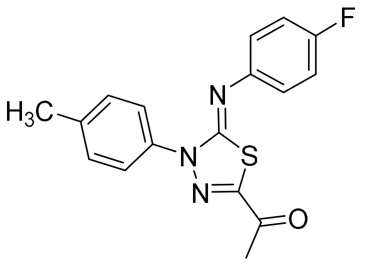

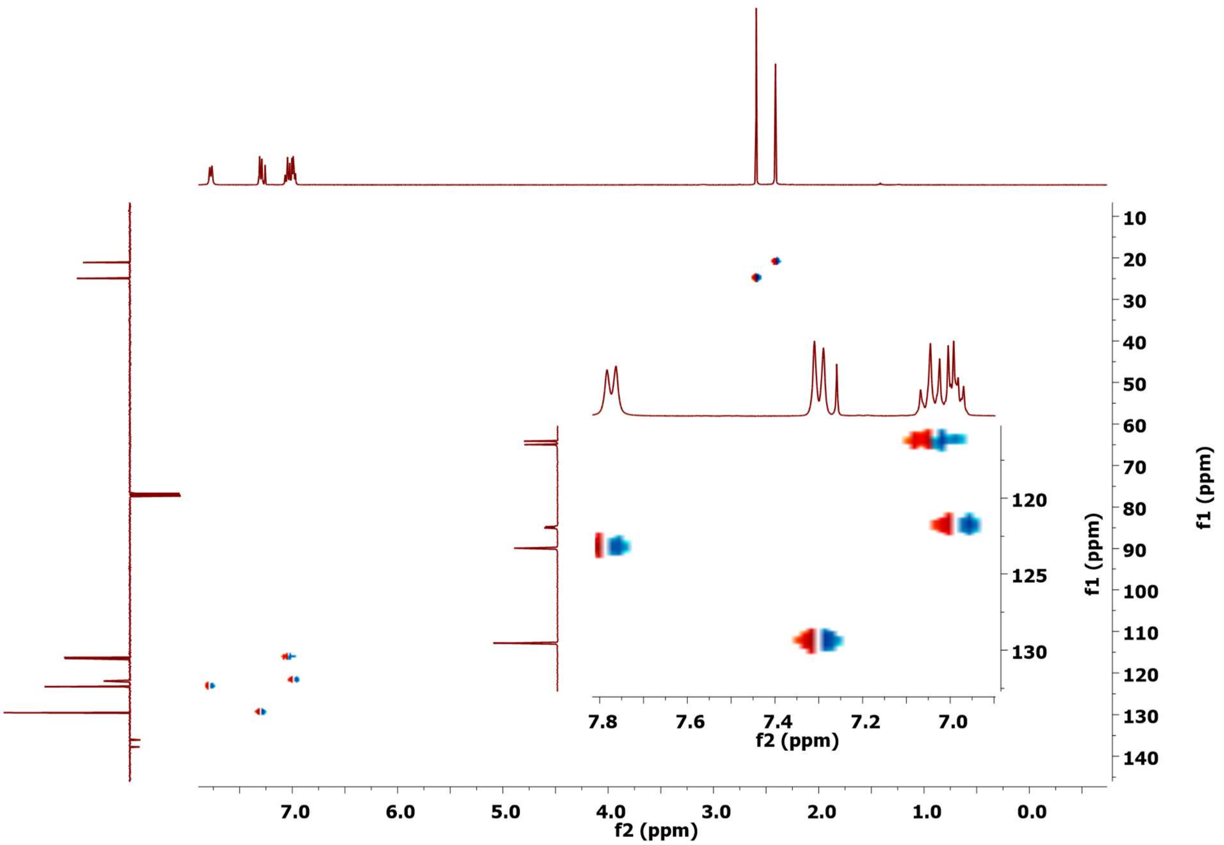

^{1}H-^{13}C-gHMBC NMR ($CDCl_3$) spectrum of (Z)-1-(5-((4-fluorophenyl)imino)-4-(p-tolyl)-4,5-dihydro-1,3,4-thiadiazol-2-yl)ethan-1-one

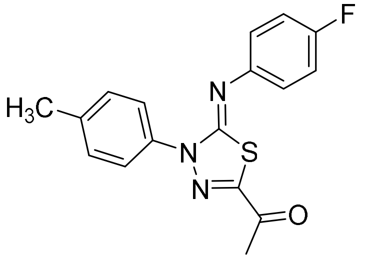

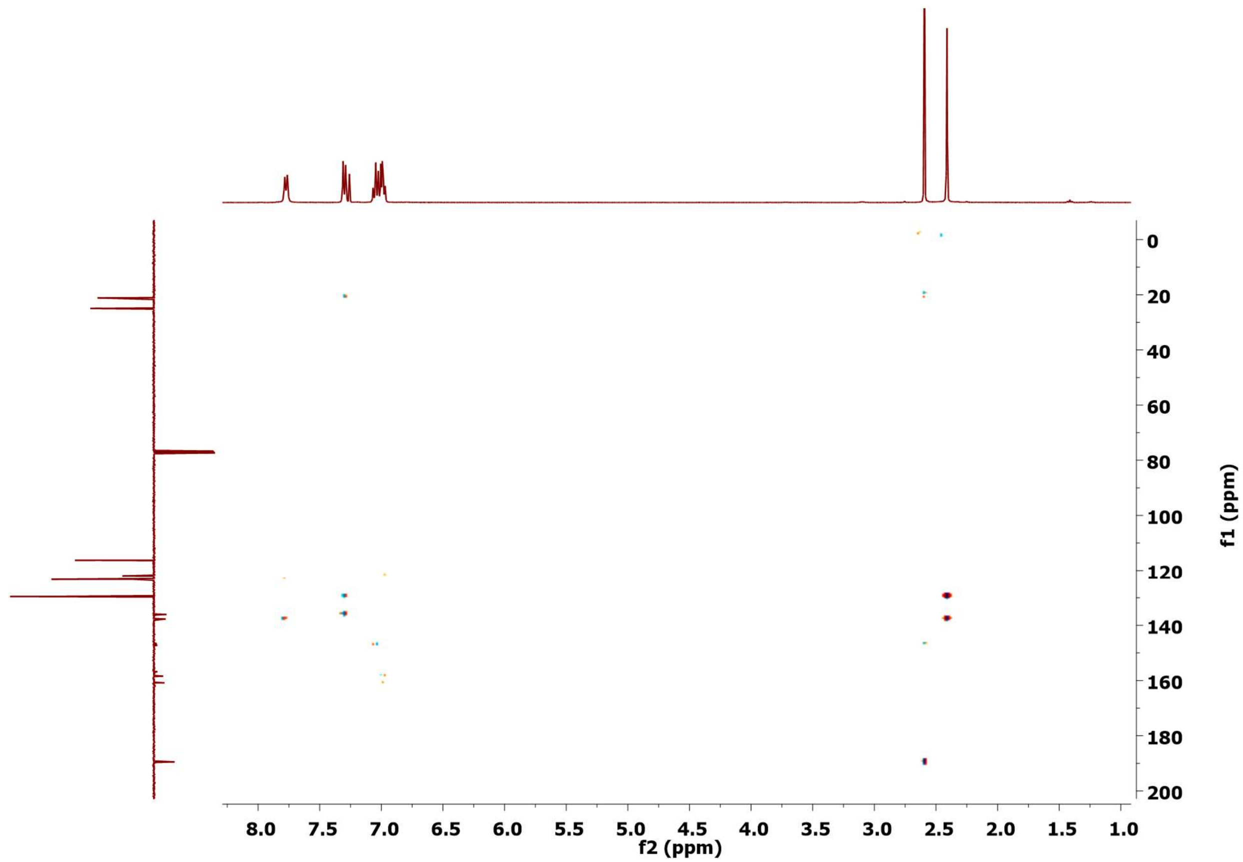

^{1}H NMR ($CDCl_3$) spectrum of (Z)-1-(5-((4-fluorophenyl)imino)-4-(4-(methylthio)phenyl)-4,5-dihydro-1,3,4-thiadiazol-2-yl)ethan-1-one

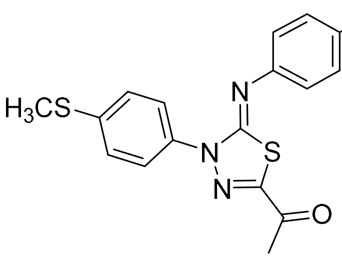

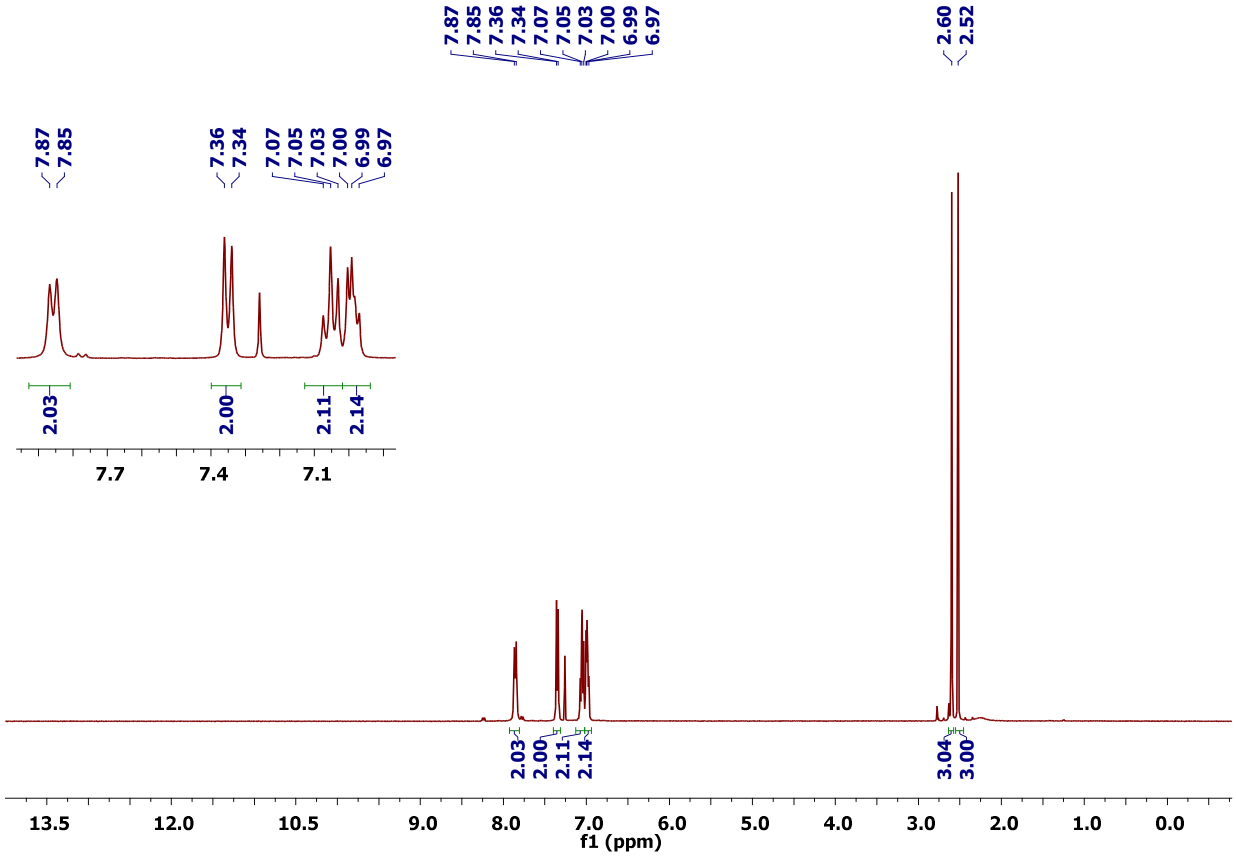

^{13}C NMR ($CDCl_3$) spectrum of (Z)-1-(5-((4-fluorophenyl)imino)-4-(4-(methylthio)phenyl)-4,5-dihydro-1,3,4-thiadiazol-2-yl)ethan-1-one

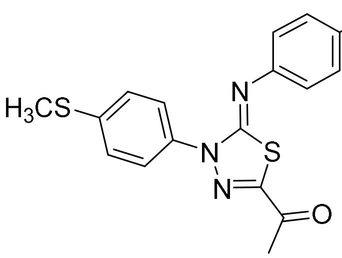

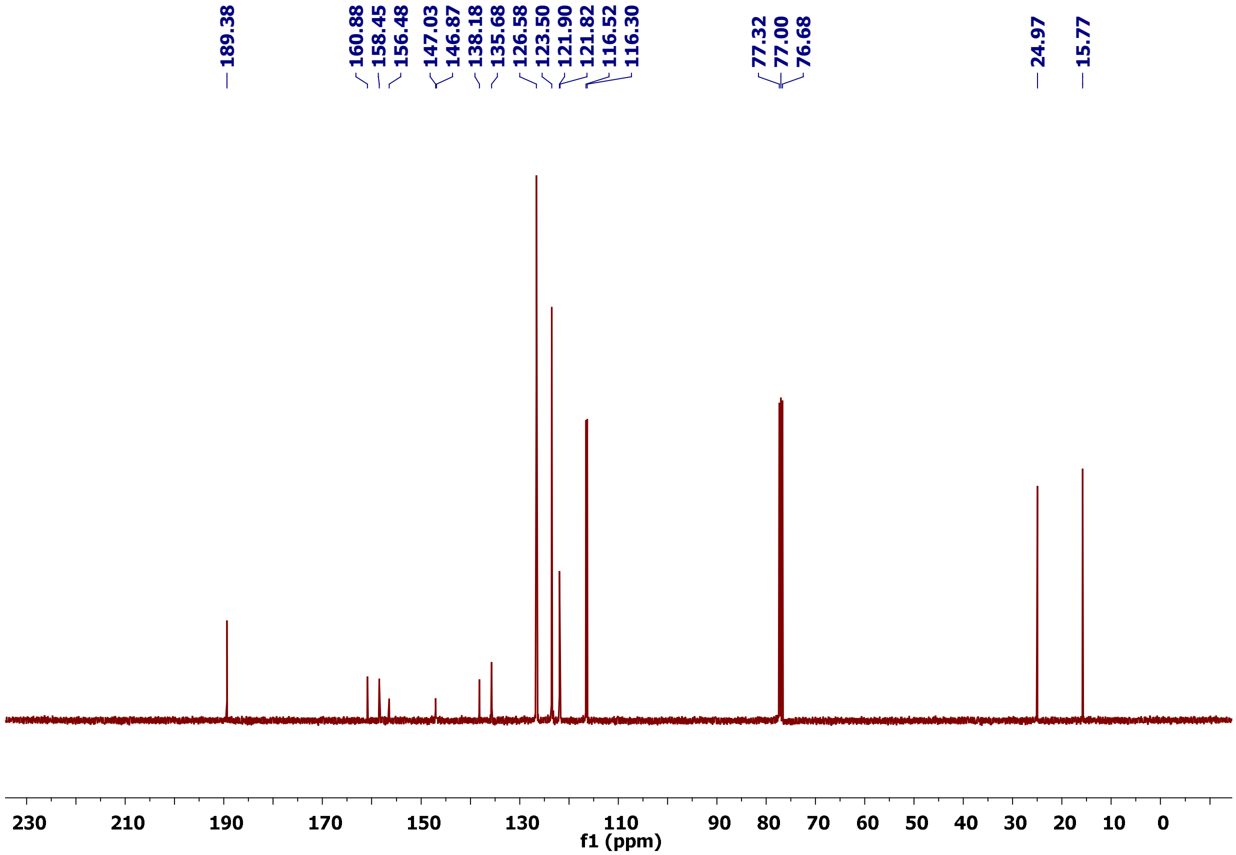

^{13}C CRAPT NMR ($CDCl_3$) spectrum of (Z)-1-(5-((4-fluorophenyl)imino)-4-(4-(methylthio)phenyl)-4,5-dihydro-1,3,4-thiadiazol-2-yl)ethan-1-one

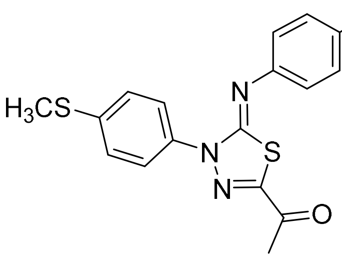

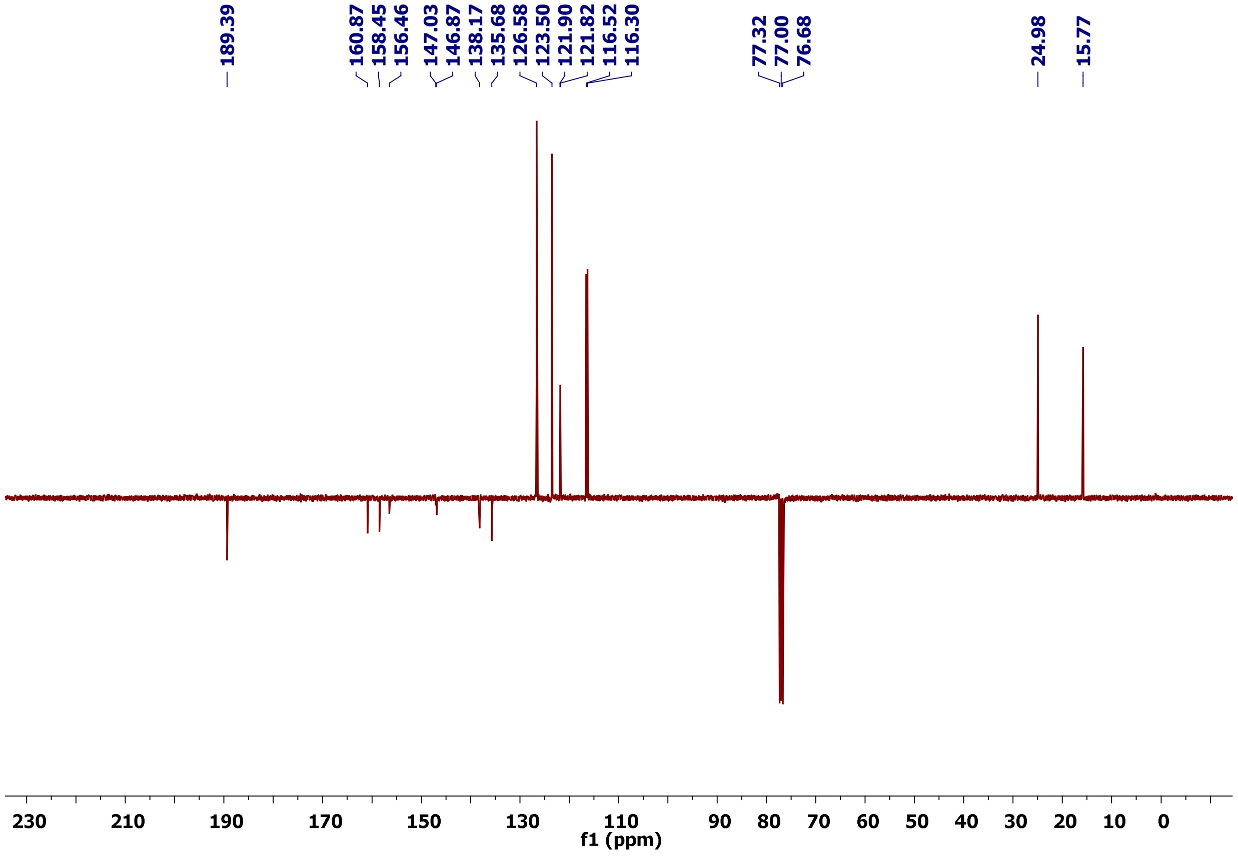

^{1}H-^{1}H gDQCOSY NMR ($CDCl_3$) spectrum of (Z)-1-(5-((4-fluorophenyl)imino)-4-(4-(methylthio)phenyl)-4,5-dihydro-1,3,4-thiadiazol-2-yl)ethan-1-one

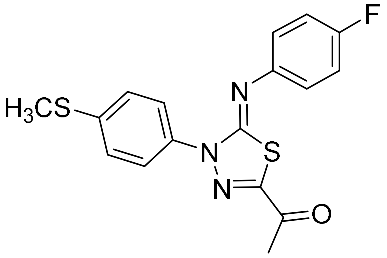

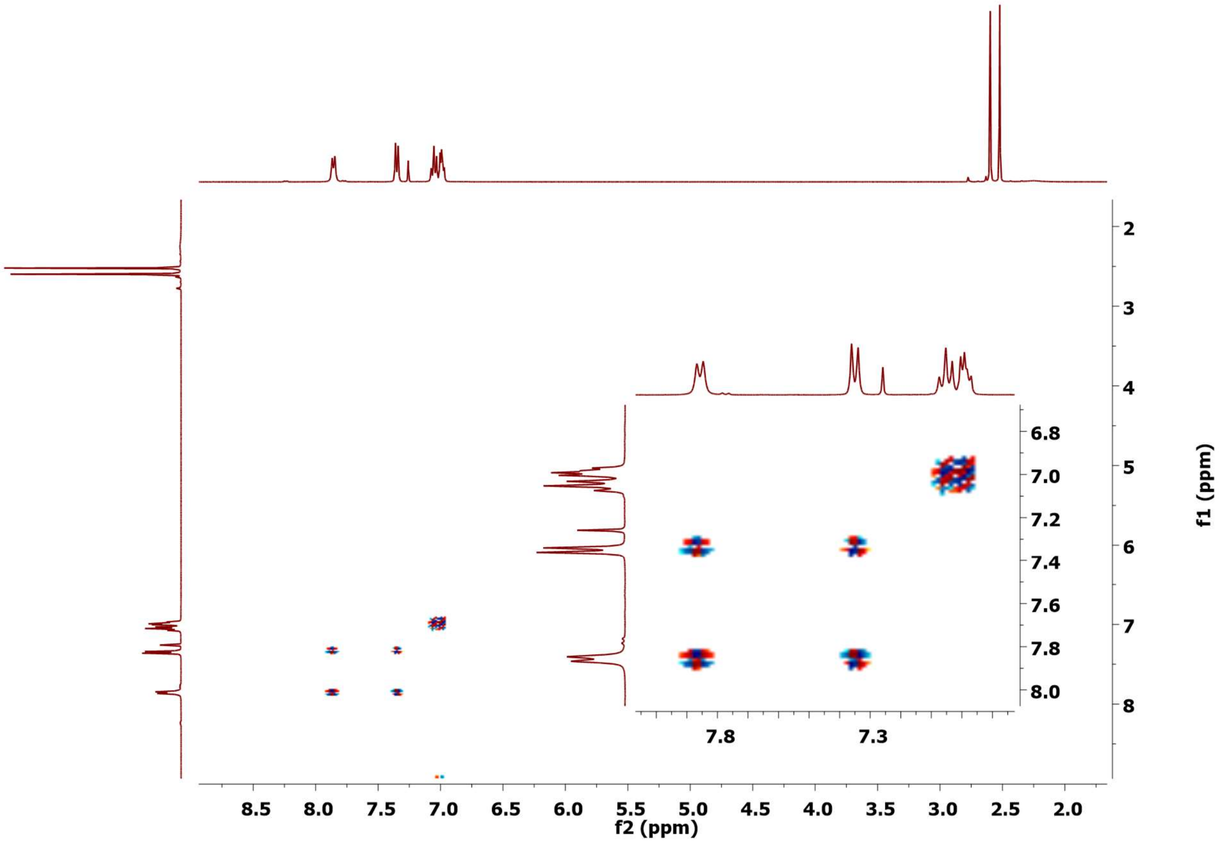

^{1}H-^{13}C-HSQC NMR ($CDCl_3$) spectrum of (Z)-1-(5-((4-fluorophenyl)imino)-4-(4-(methylthio)phenyl)-4,5-dihydro-1,3,4-thiadiazol-2-yl)ethan-1-one

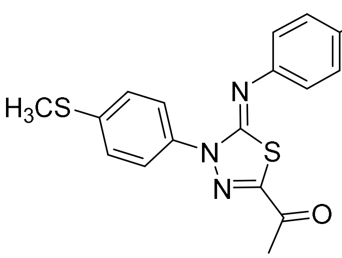

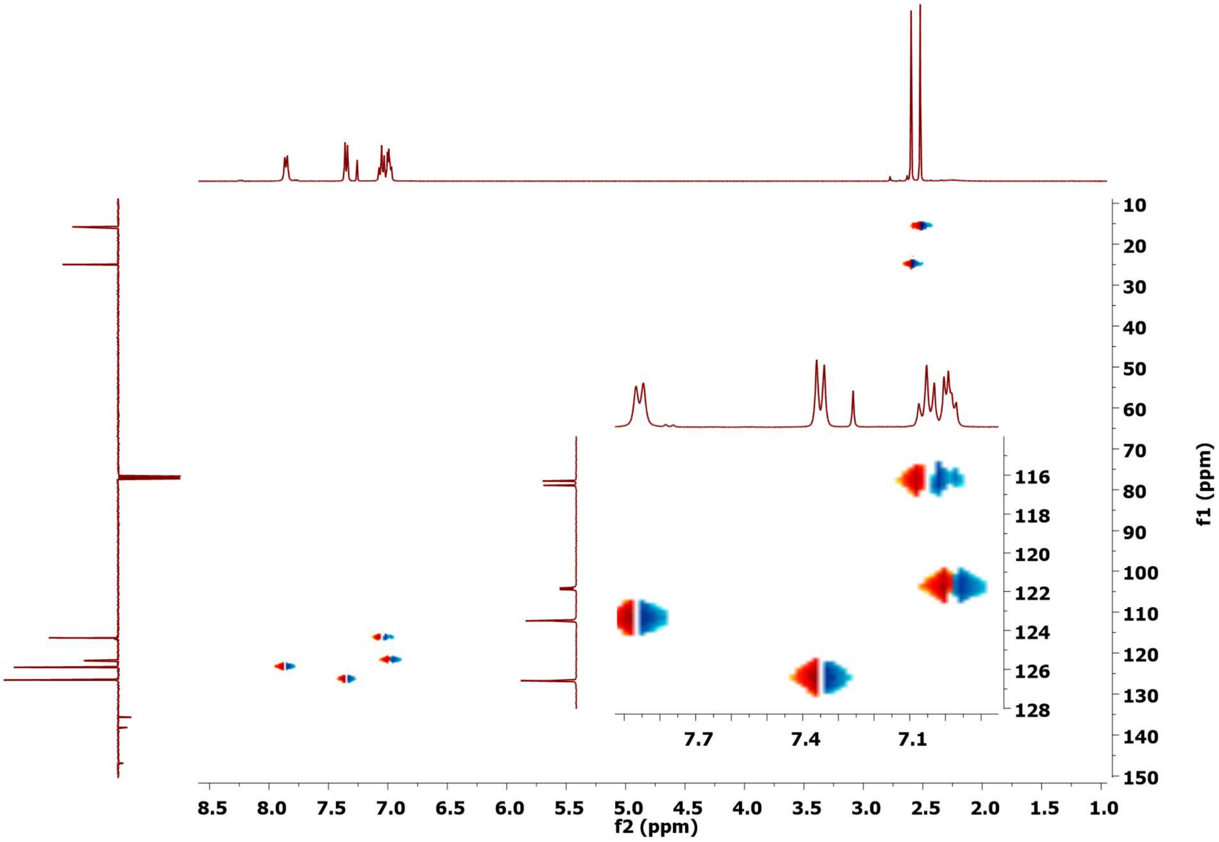

^{1}H-^{13}C-gHMBC NMR ($CDCl_3$) spectrum of (Z)-1-(5-((4-fluorophenyl)imino)-4-(4-(methylthio)phenyl)-4,5-dihydro-1,3,4-thiadiazol-2-yl)ethan-1-one

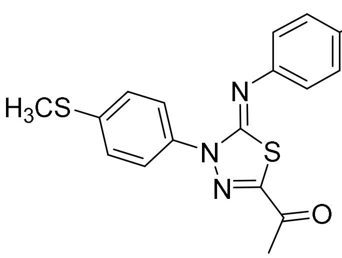

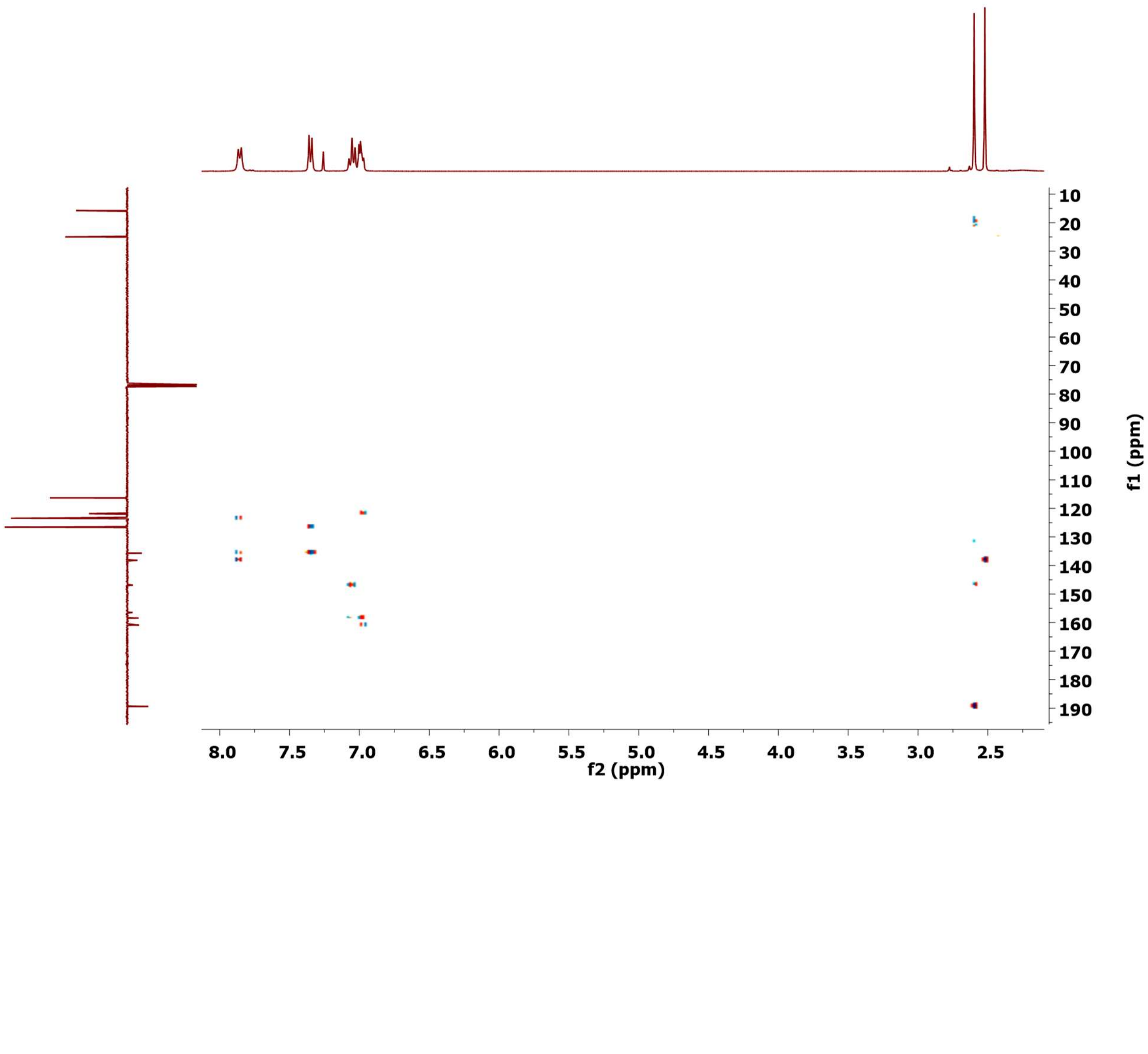

^{1}H NMR ($CDCl_3$) spectrum of (Z)-1-(5-((4-fluorophenyl)imino)-4-(4-methoxyphenyl)-4,5-dihydro-1,3,4-thiadiazol-2-yl)ethan-1-one

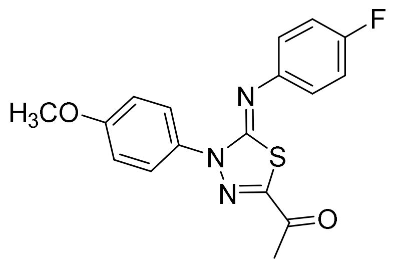

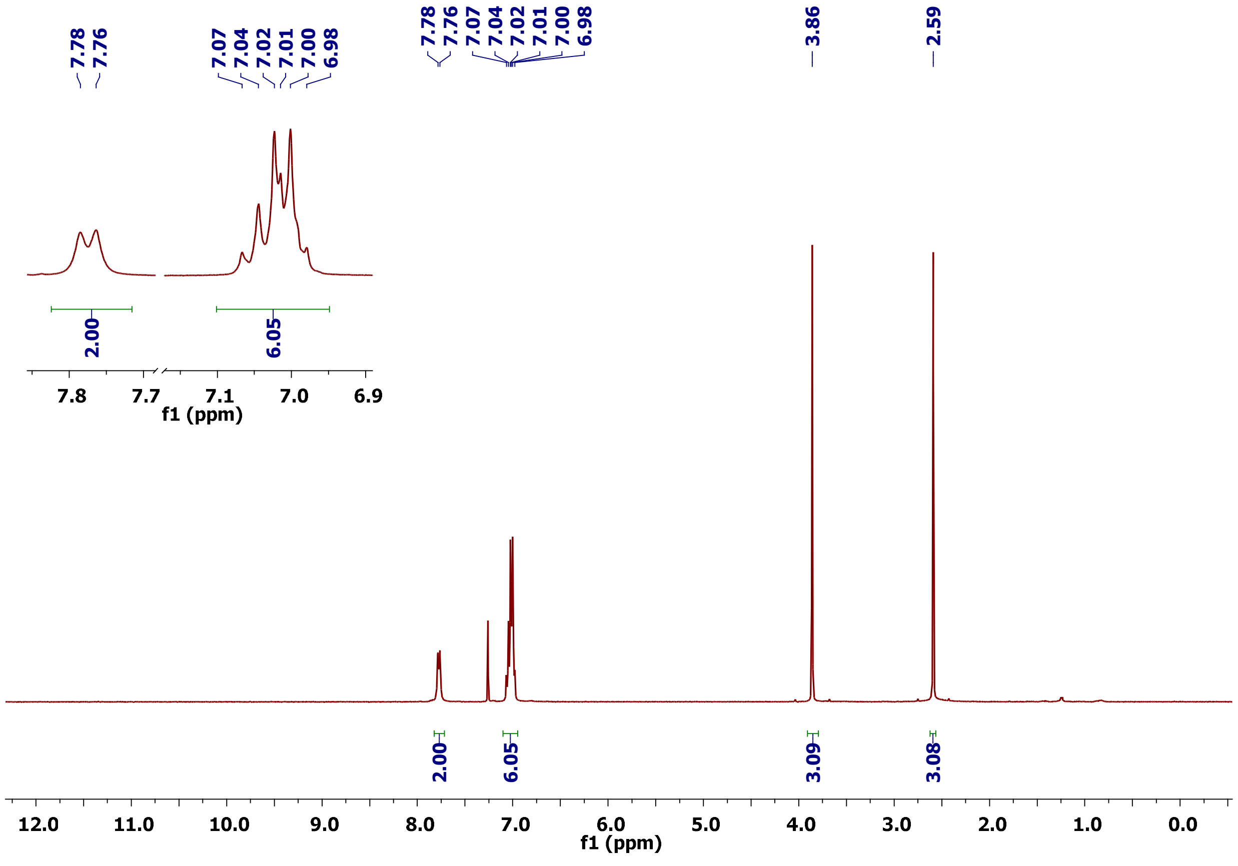

^{13}C NMR ($CDCl_3$) spectrum of (Z)-1-(5-((4-fluorophenyl)imino)-4-(4-methoxyphenyl)-4,5-dihydro-1,3,4-thiadiazol-2-yl)ethan-1-one

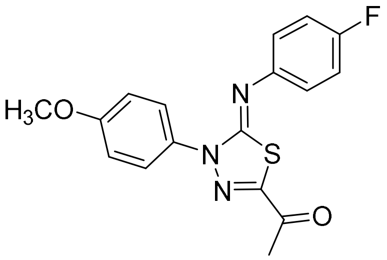

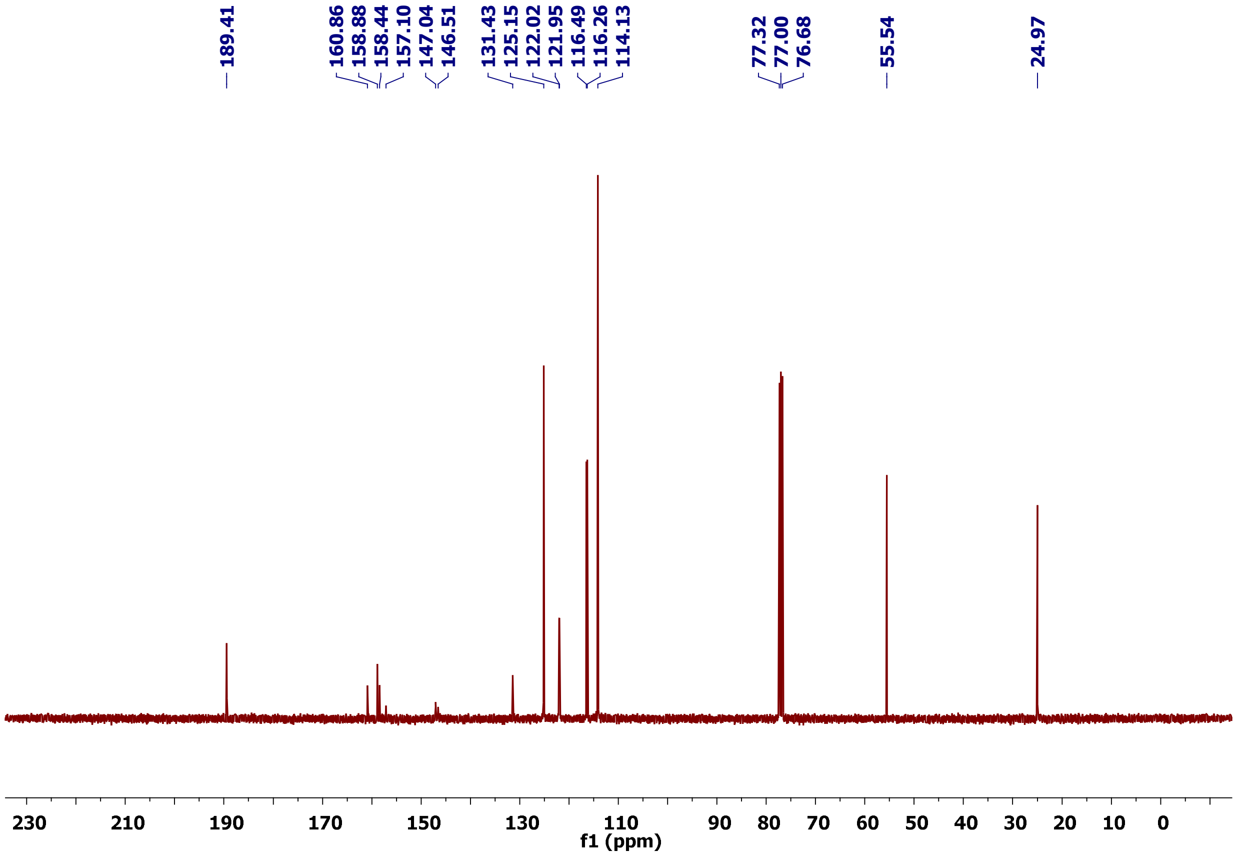

^{13}C CRAPT NMR ($CDCl_3$) spectrum of (Z)-1-(5-((4-fluorophenyl)imino)-4-(4-methoxyphenyl)-4,5-dihydro-1,3,4-thiadiazol-2-yl)ethan-1-one

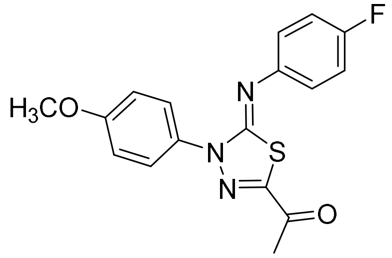

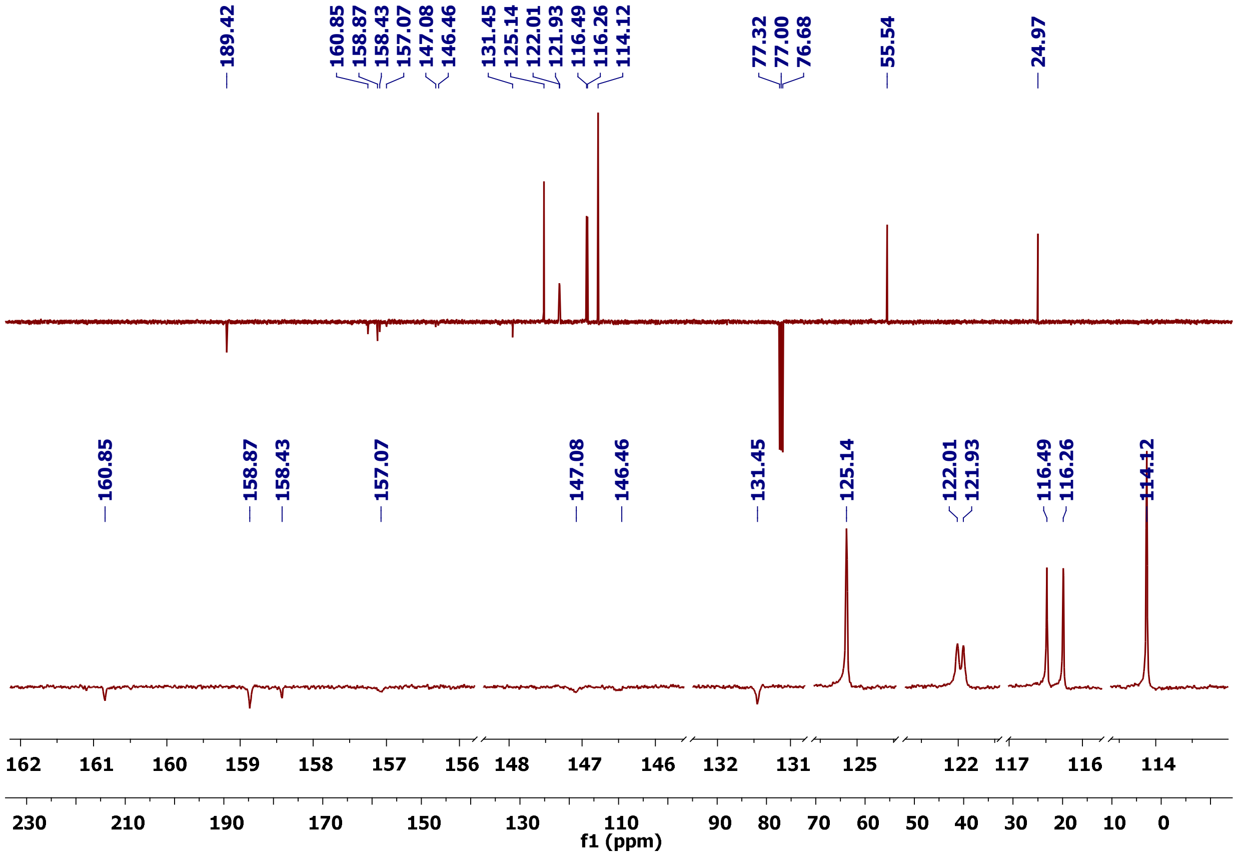

^{1}H-^{1}H gDQCOSY NMR ($CDCl_3$) spectrum of (Z)-1-(5-((4-fluorophenyl)imino)-4-(4-methoxyphenyl)-4,5-dihydro-1,3,4-thiadiazol-2-yl)ethan-1-one

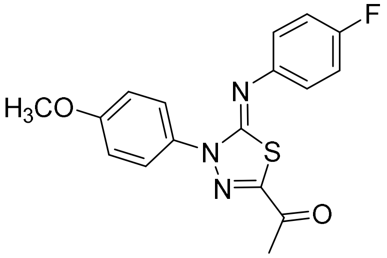

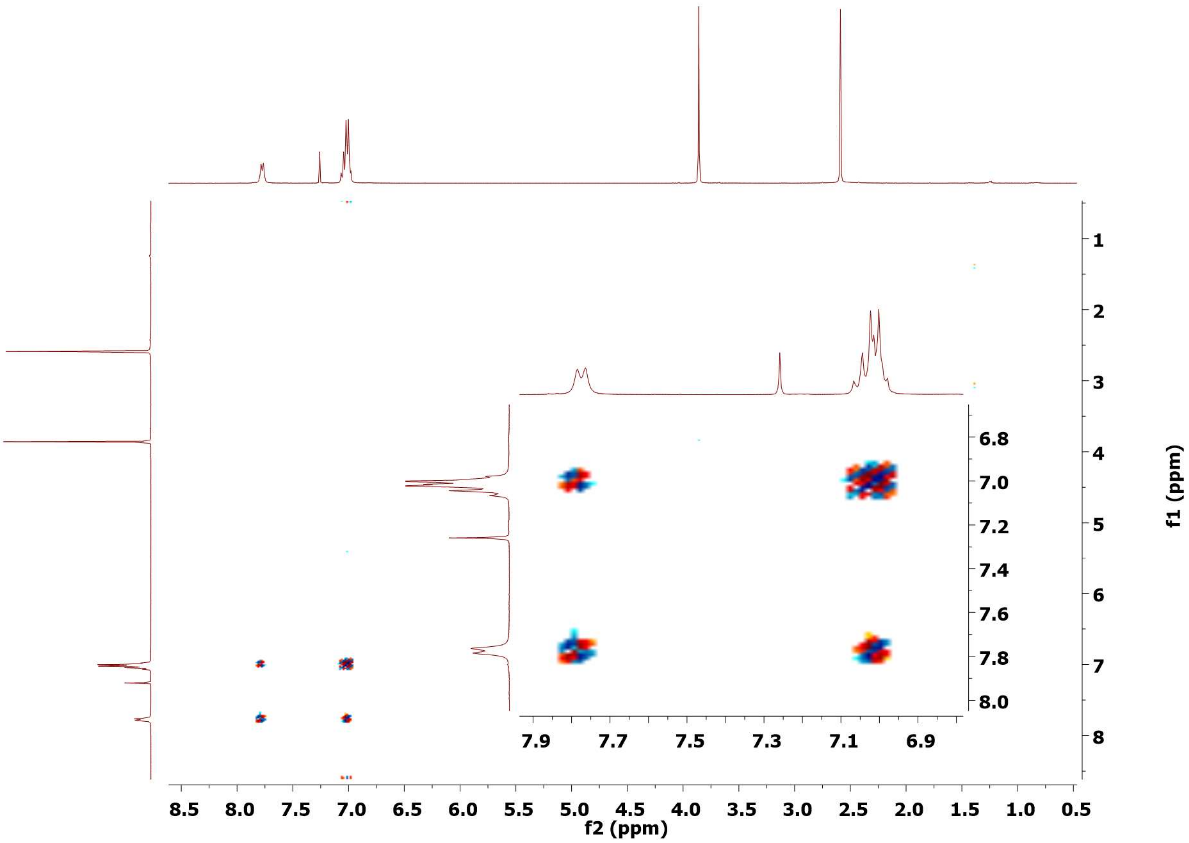

^{1}H-^{13}C-HSQC NMR ($CDCl_3$) spectrum of (Z)-1-(5-((4-fluorophenyl)imino)-4-(4-methoxyphenyl)-4,5-dihydro-1,3,4-thiadiazol-2-yl)ethan-1-one

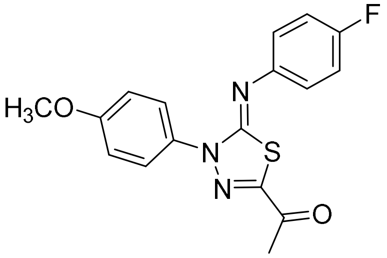

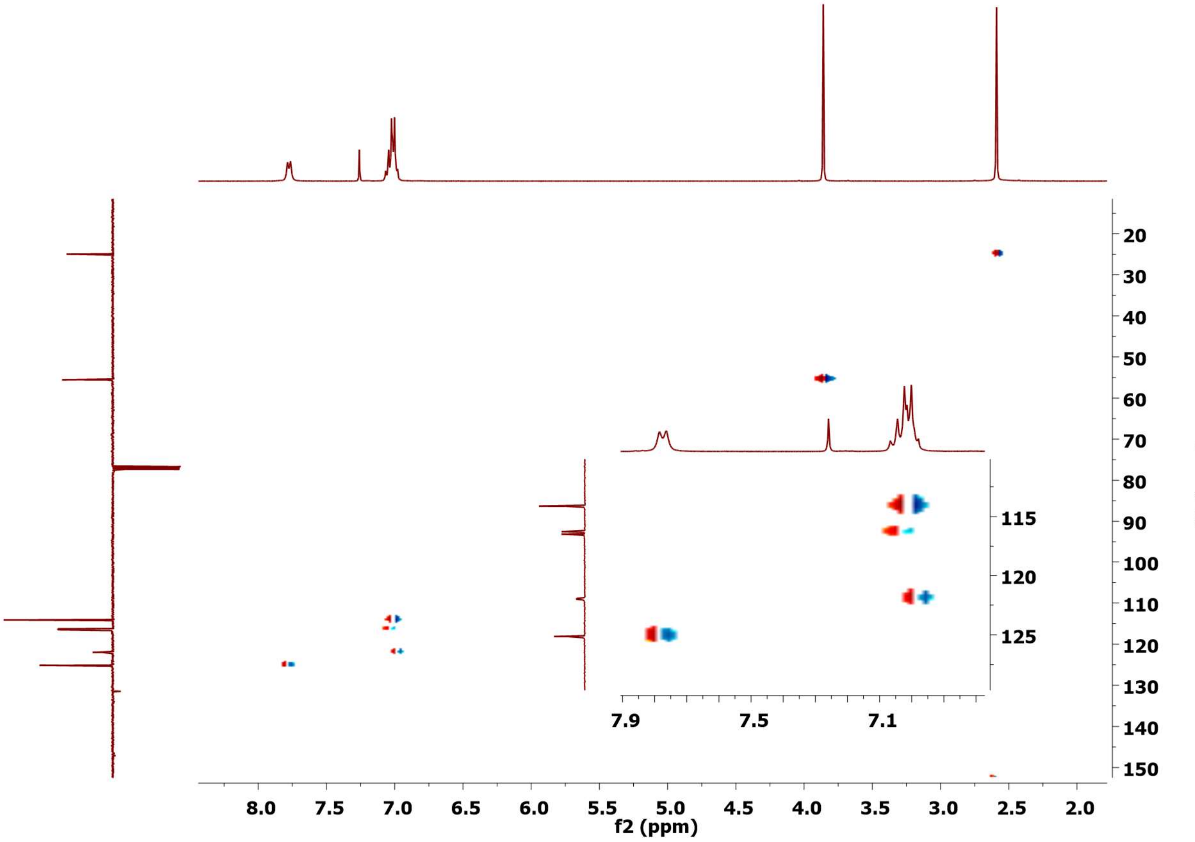

^{1}H-^{13}C-gHMBC NMR ($CDCl_3$) spectrum of (Z)-1-(5-((4-fluorophenyl)imino)-4-(4-methoxyphenyl)-4,5-dihydro-1,3,4-thiadiazol-2-yl)ethan-1-one

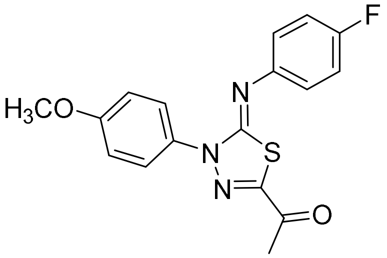

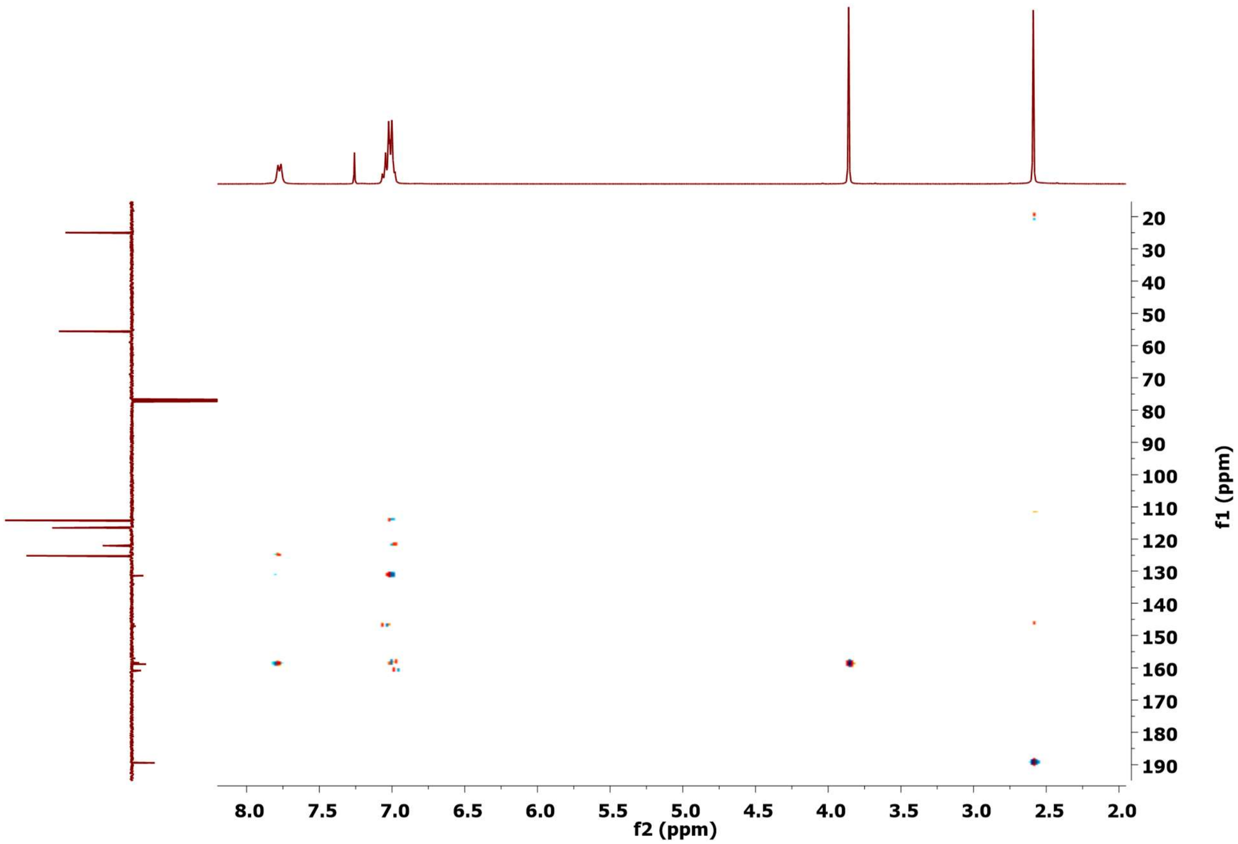

^{1}H NMR ($CDCl_3$) spectrum of (Z)-1-(5-((4-fluorophenyl)imino)-4-(m-tolyl)-4,5-dihydro-1,3,4-thiadiazol-2-yl)ethan-1-one

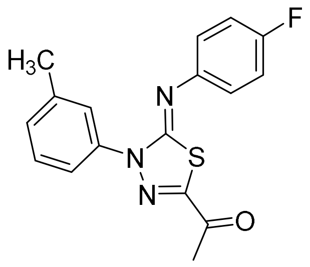

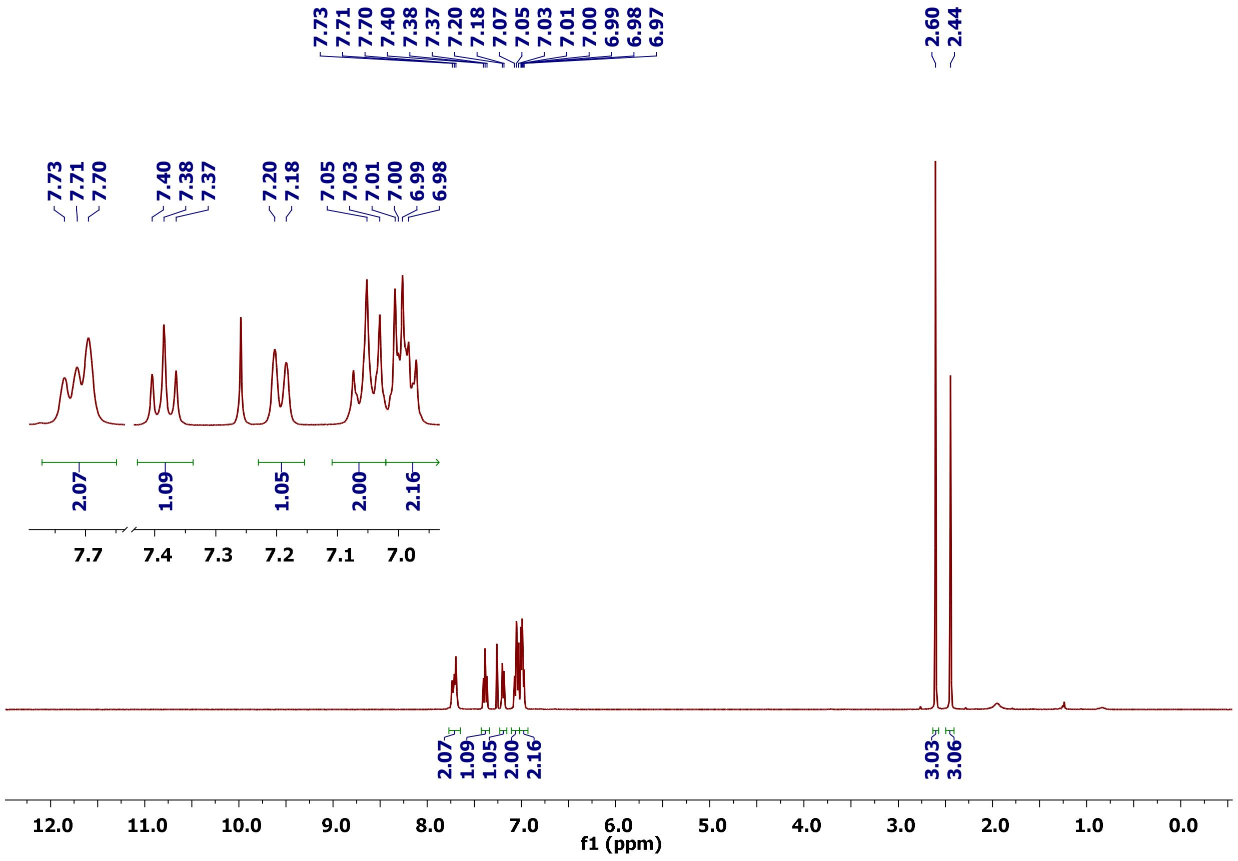

^{13}C NMR ($CDCl_3$) spectrum of (Z)-1-(5-((4-fluorophenyl)imino)-4-(m-tolyl)-4,5-dihydro-1,3,4-thiadiazol-2-yl)ethan-1-one

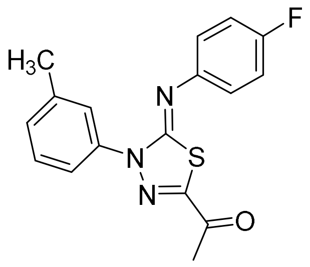

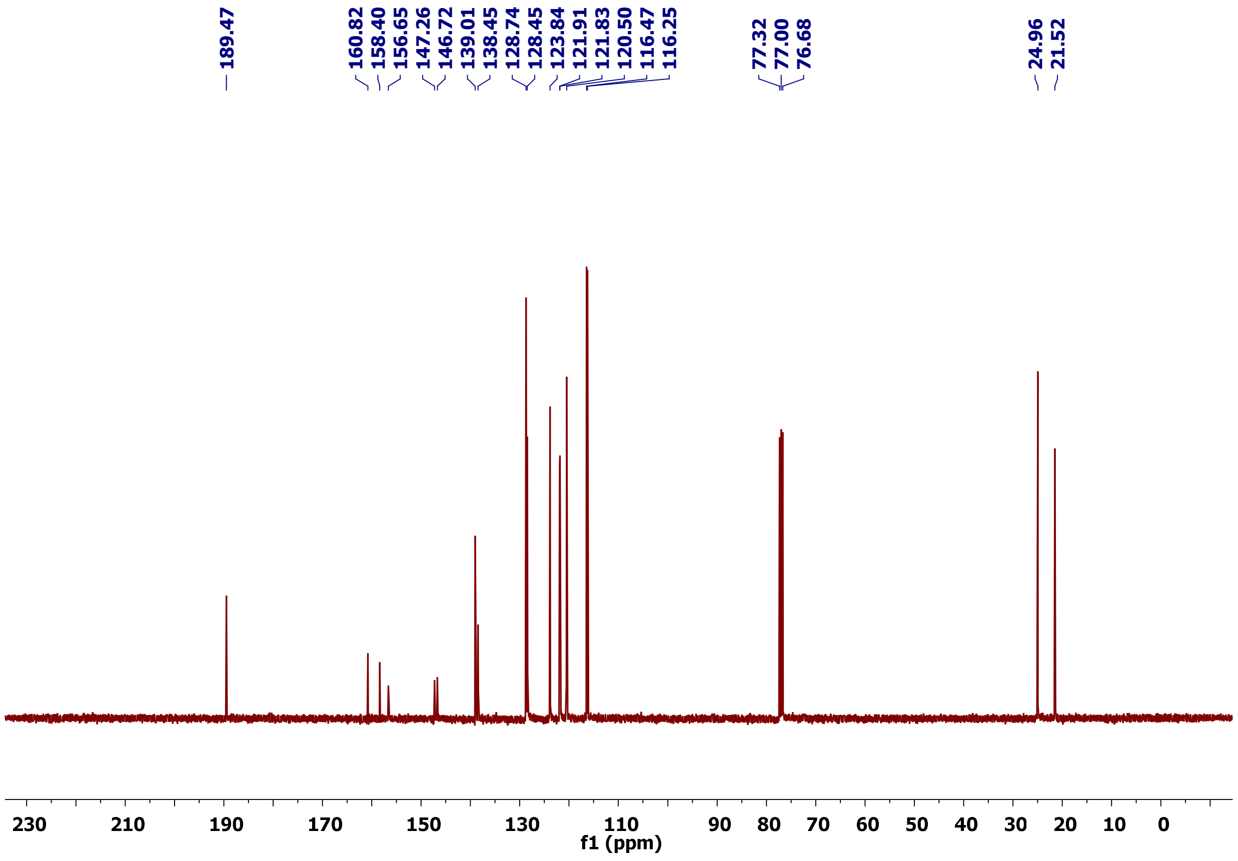

^{13}C CRAPT NMR ($CDCl_3$) spectrum of (Z)-1-(5-((4-fluorophenyl)imino)-4-(m-tolyl)-4,5-dihydro-1,3,4-thiadiazol-2-yl)ethan-1-one

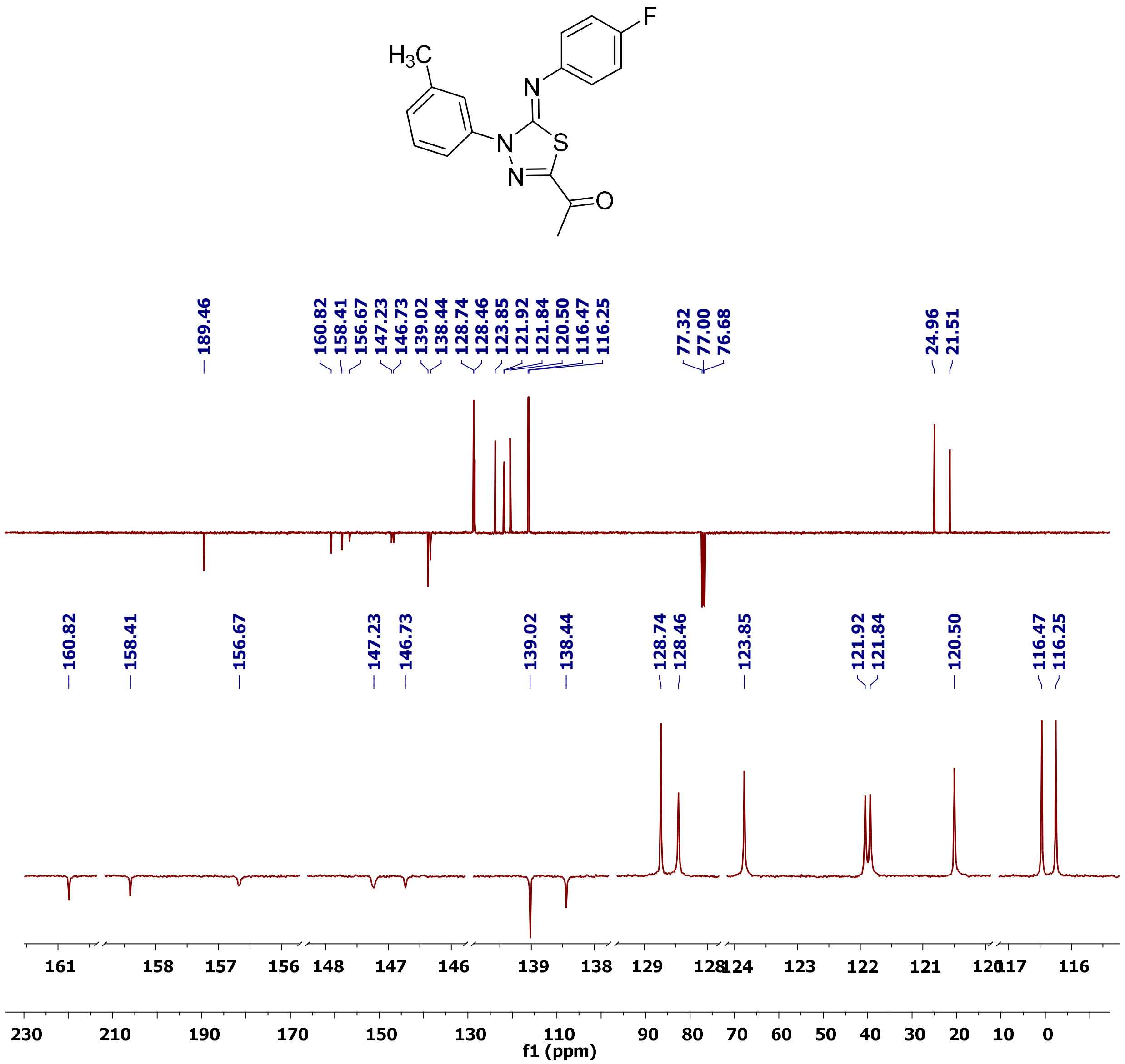

^{1}H-^{1}H gDQCOSY NMR ($CDCl_3$) spectrum of (Z)-1-(5-((4-fluorophenyl)imino)-4-(m-tolyl)-4,5-dihydro-1,3,4-thiadiazol-2-yl)ethan-1-one

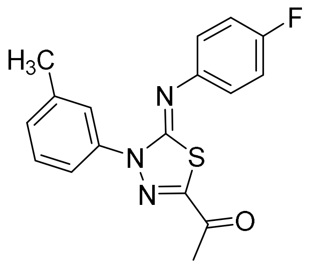

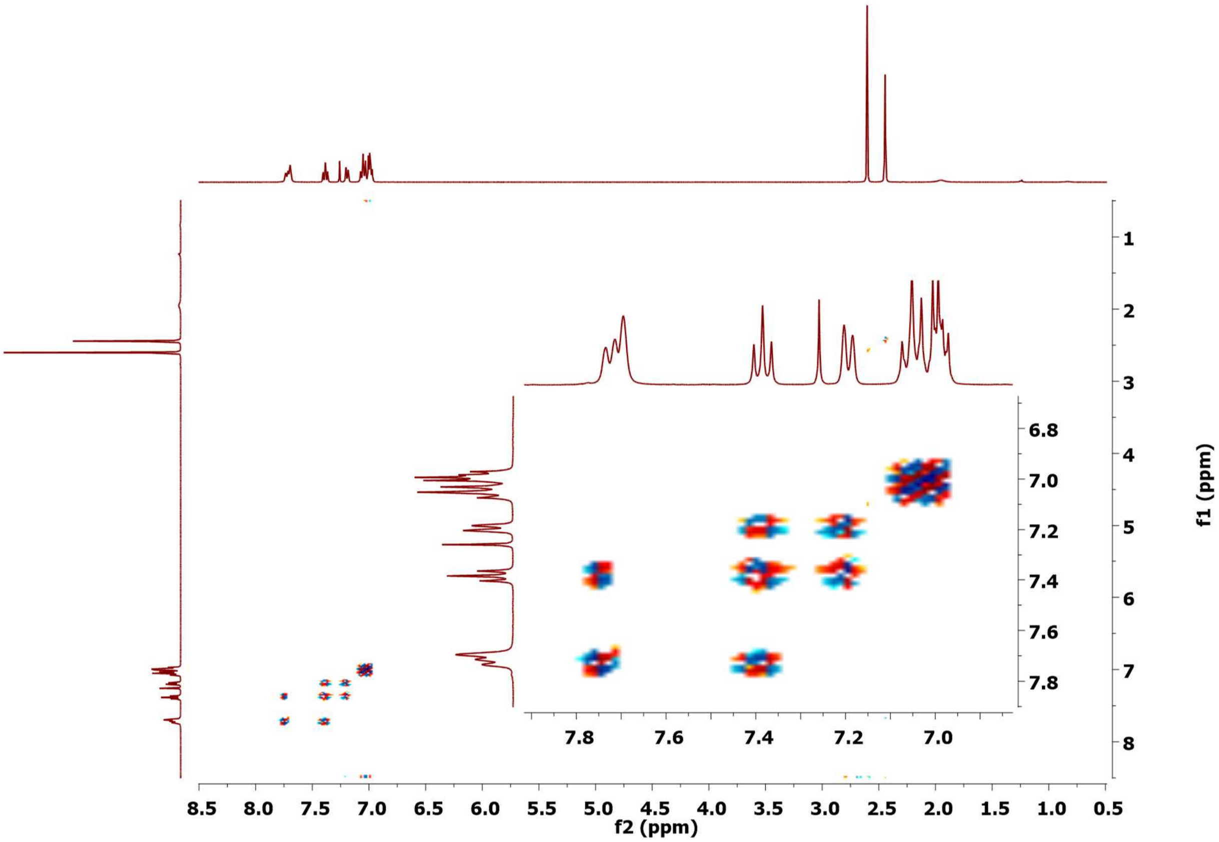

^{1}H-^{13}C-HSQC NMR ($CDCl_3$) spectrum of (Z)-1-(5-((4-fluorophenyl)imino)-4-(m-tolyl)-4,5-dihydro-1,3,4-thiadiazol-2-yl)ethan-1-one

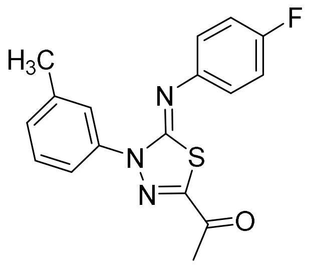

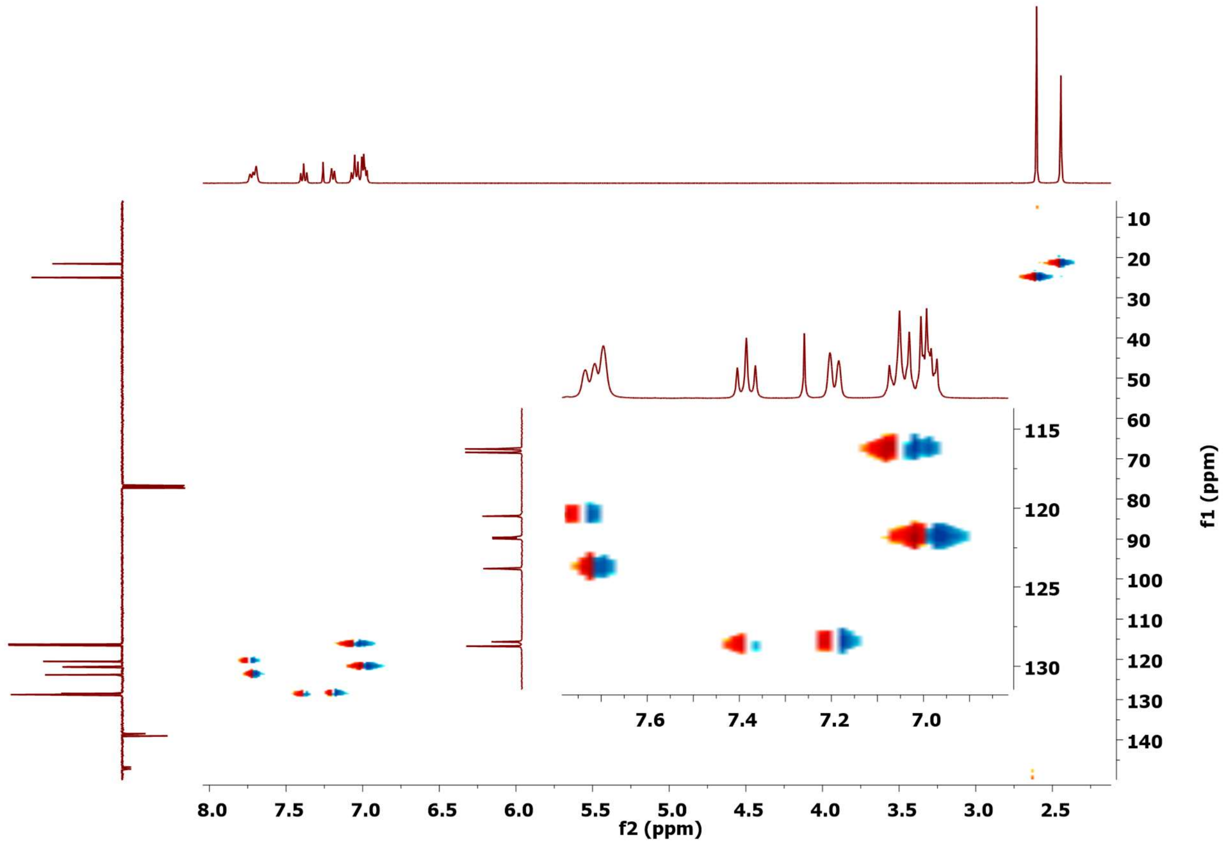

^{1}H-^{13}C-gHMBC NMR ($CDCl_3$) spectrum of (Z)-1-(5-((4-fluorophenyl)imino)-4-(m-tolyl)-4,5-dihydro-1,3,4-thiadiazol-2-yl)ethan-1-one

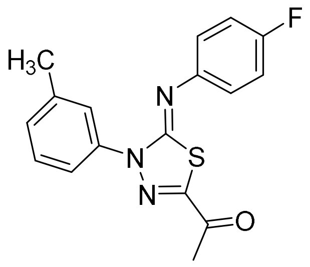

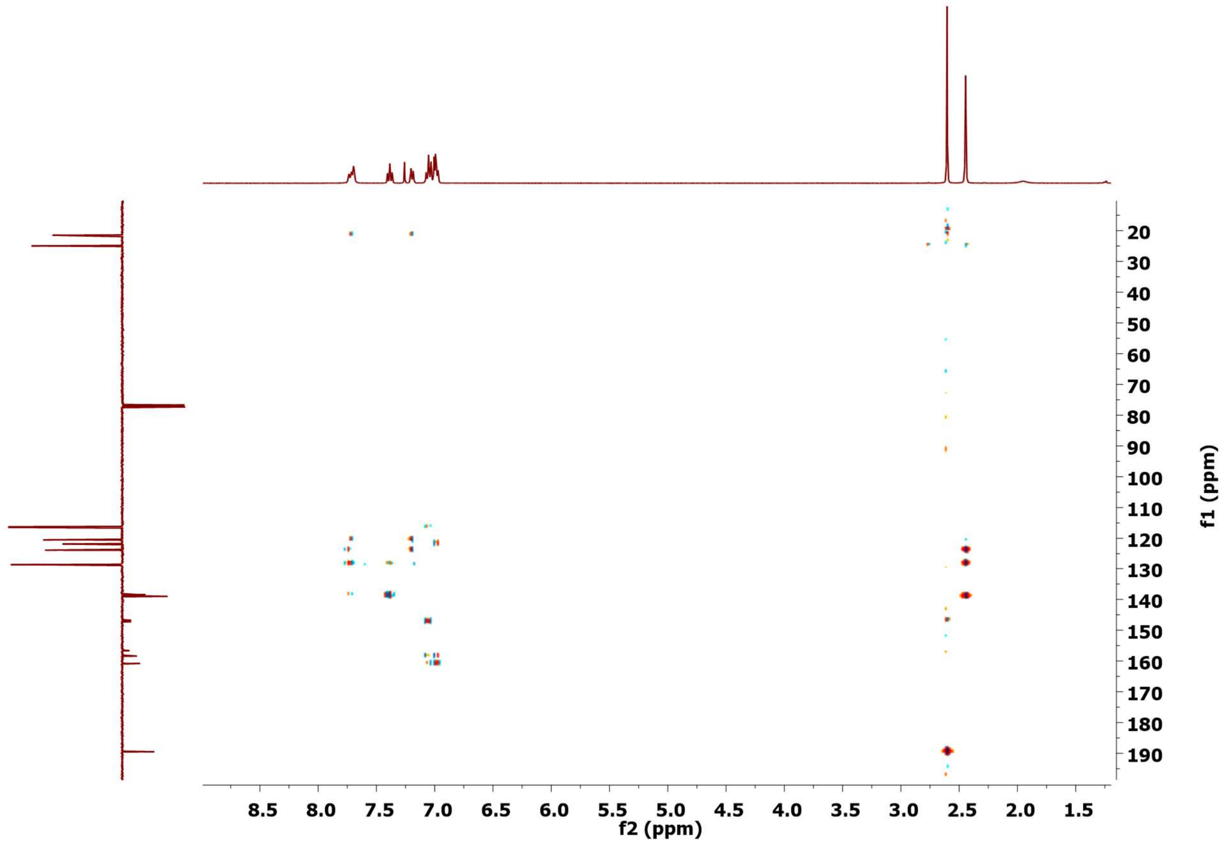

^{1}H NMR (CDCl$_3$) spectrum of (Z)-1-(4-(4-bromophenyl)-5-((4-isopropylphenyl)imino)-4,5-dihydro-1,3,4-thiadiazol-2-yl)ethan-1-one

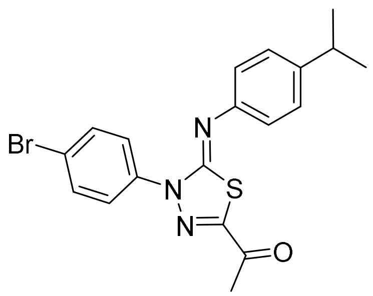

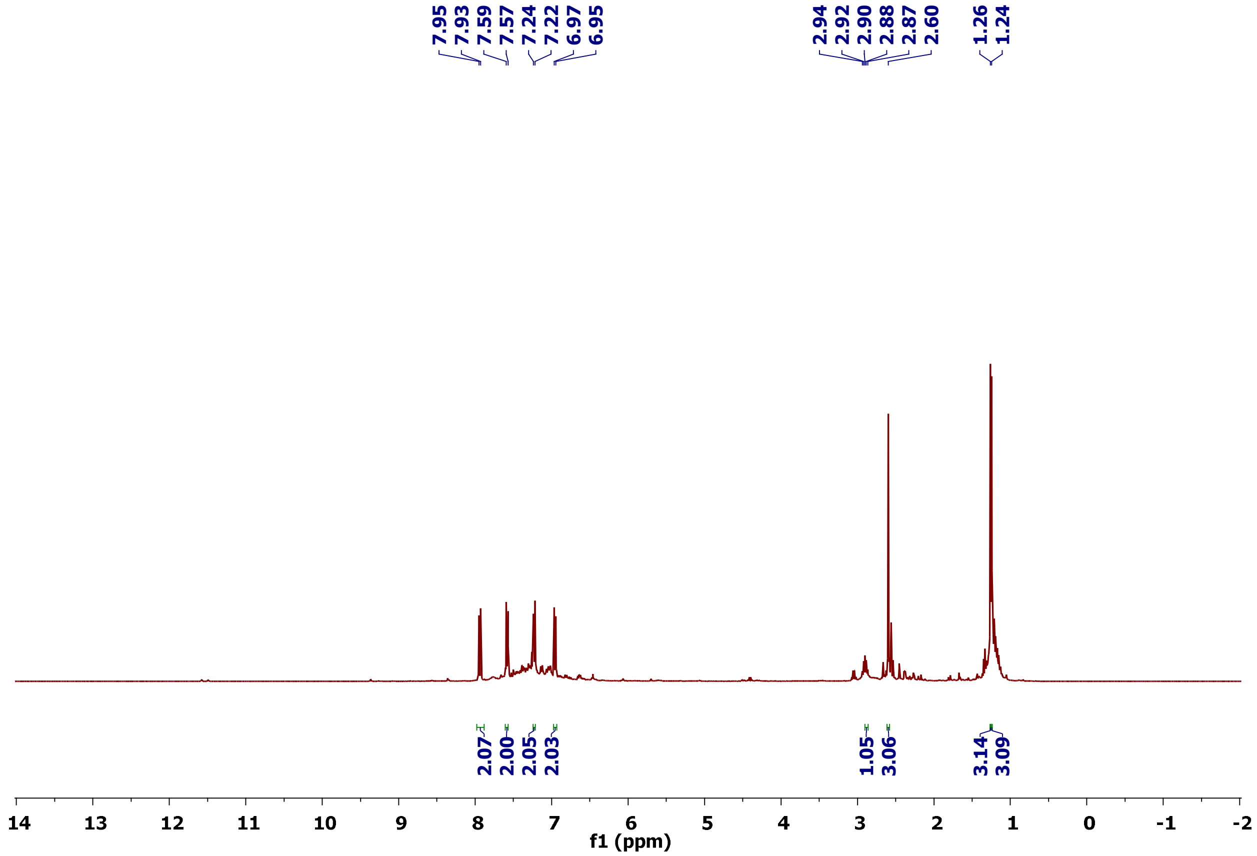

^{13}C NMR ($CDCl_3$) spectrum of (Z)-1-(4-(4-bromophenyl)-5-((4-isopropylphenyl)imino)-4,5-dihydro-1,3,4-thiadiazol-2-yl)ethan-1-one

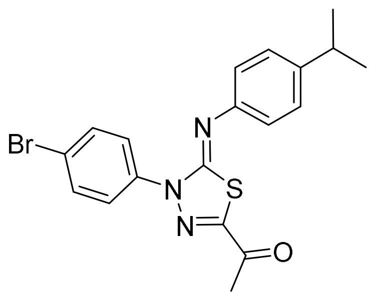

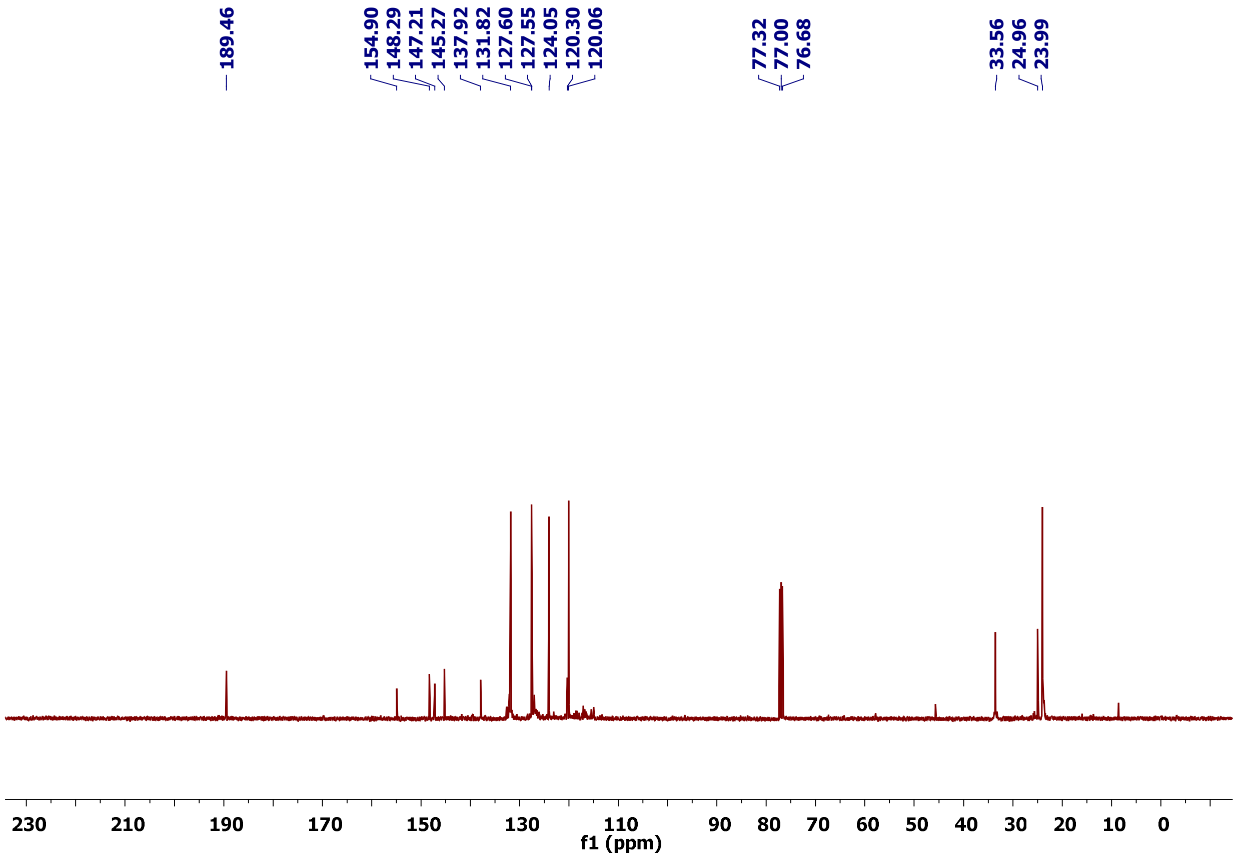

^{13}C CRAPT NMR ($CDCl_3$) spectrum of (Z)-1-(4-(4-bromophenyl)-5-((4-isopropylphenyl)imino)-4,5-dihydro-1,3,4-thiadiazol-2-yl)ethan-1-one

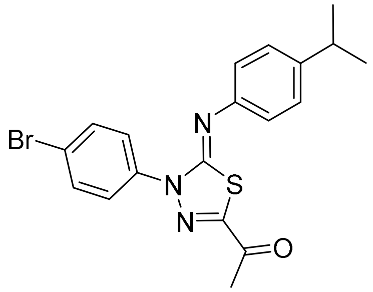

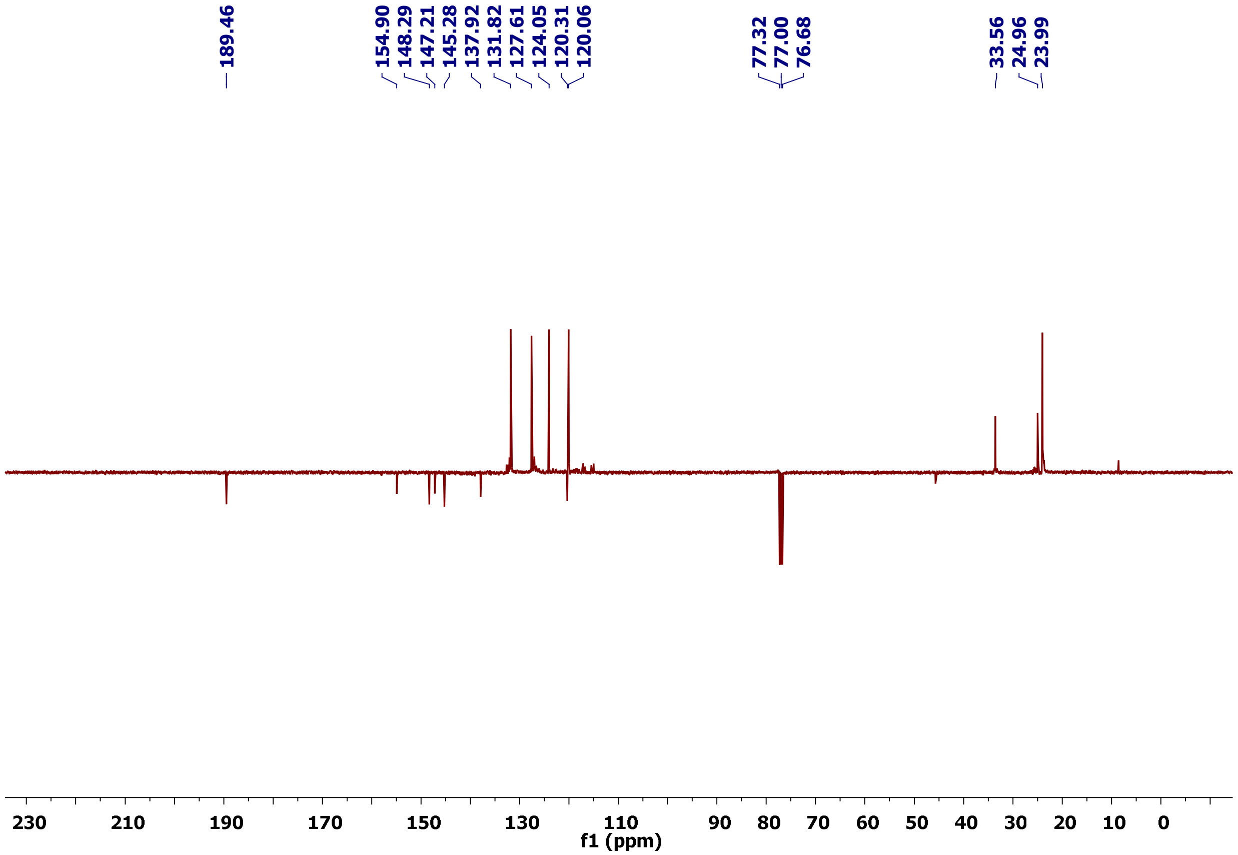

^{1}H-^{1}H gDQCOSY NMR ($CDCl_3$) spectrum of (Z)-1-(4-(4-bromophenyl)-5-((4-isopropylphenyl)imino)-4,5-dihydro-1,3,4-thiadiazol-2-yl)ethan-1-one

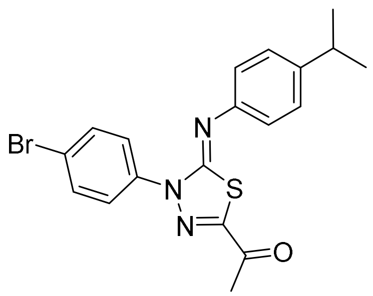

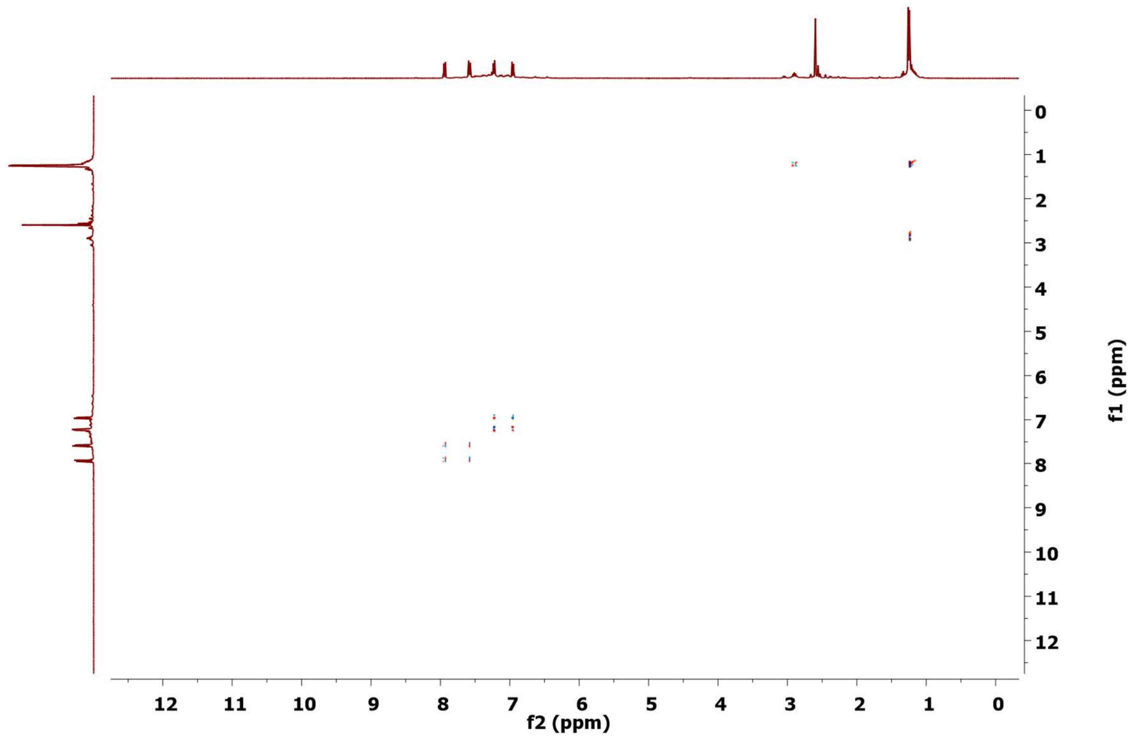

^{1}H-^{13}C-HSQC NMR ($CDCl_3$) spectrum of (Z)-1-(4-(4-bromophenyl)-5-((4-isopropylphenyl)imino)-4,5-dihydro-1,3,4-thiadiazol-2-yl)ethan-1-one

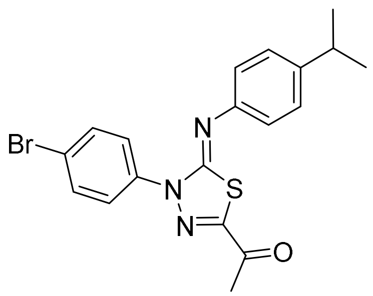

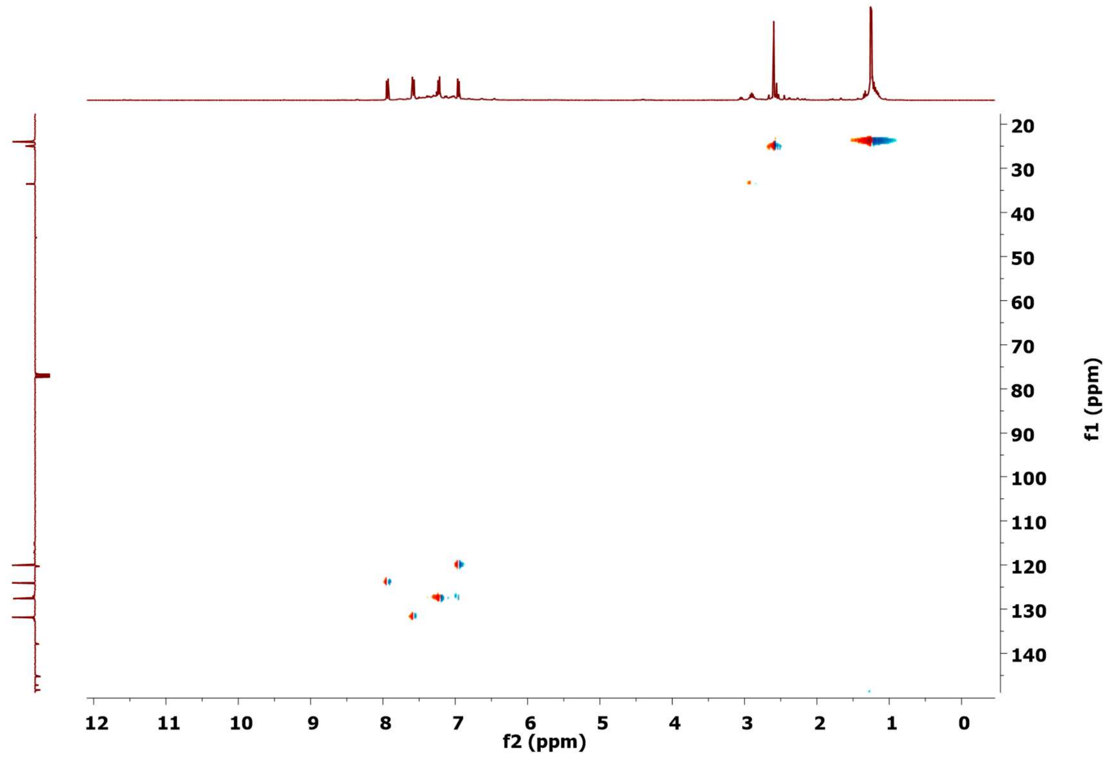

^{1}H-^{13}C-gHMBC NMR ($CDCl_3$) spectrum of (Z)-1-(4-(4-bromophenyl)-5-((4-isopropylphenyl)imino)-4,5-dihydro-1,3,4-thiadiazol-2-yl)ethan-1-one

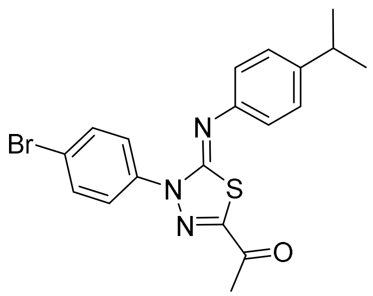

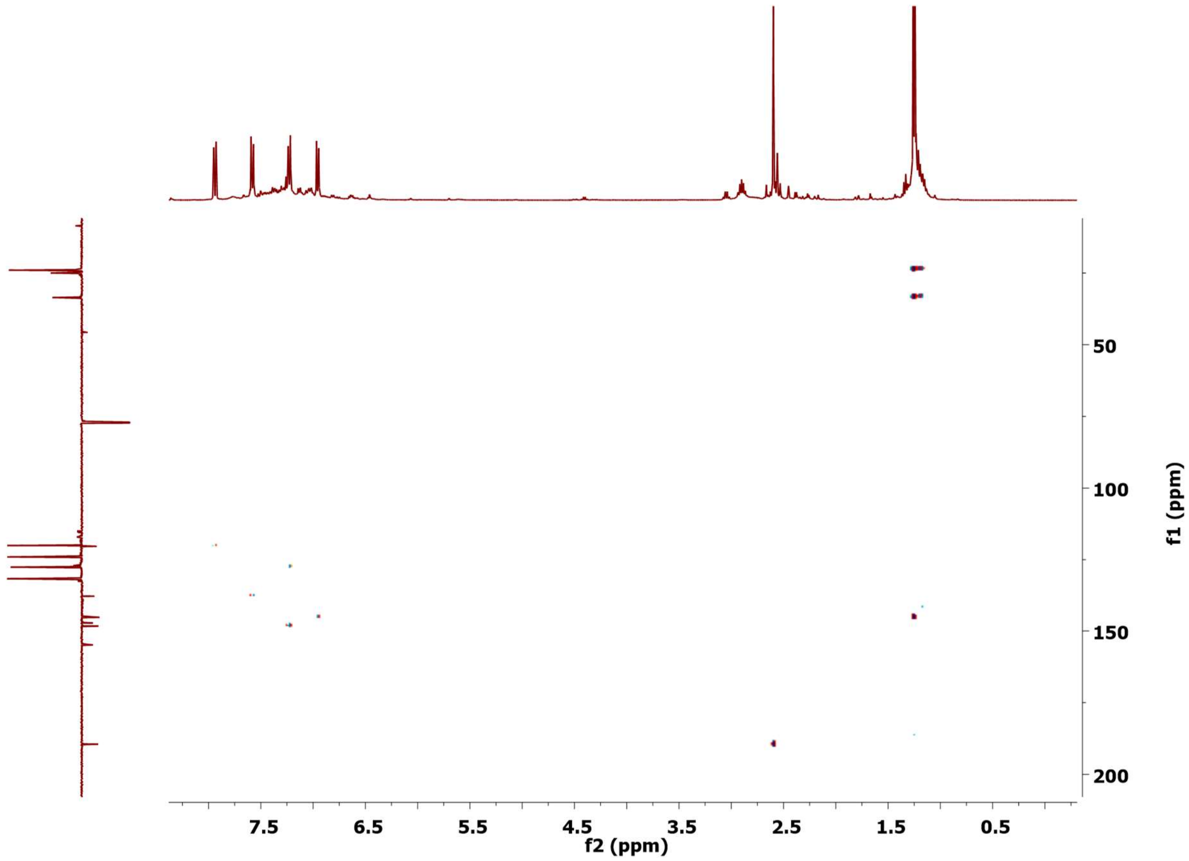

^{1}H NMR ($CDCl_3$) spectrum of (Z)-1-(4-(2,3-difluorophenyl)-5-((4-nitrophenyl)imino)-4,5-dihydro-1,3,4-thiadiazol-2-yl)ethan-1-one

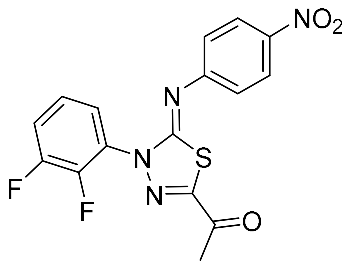

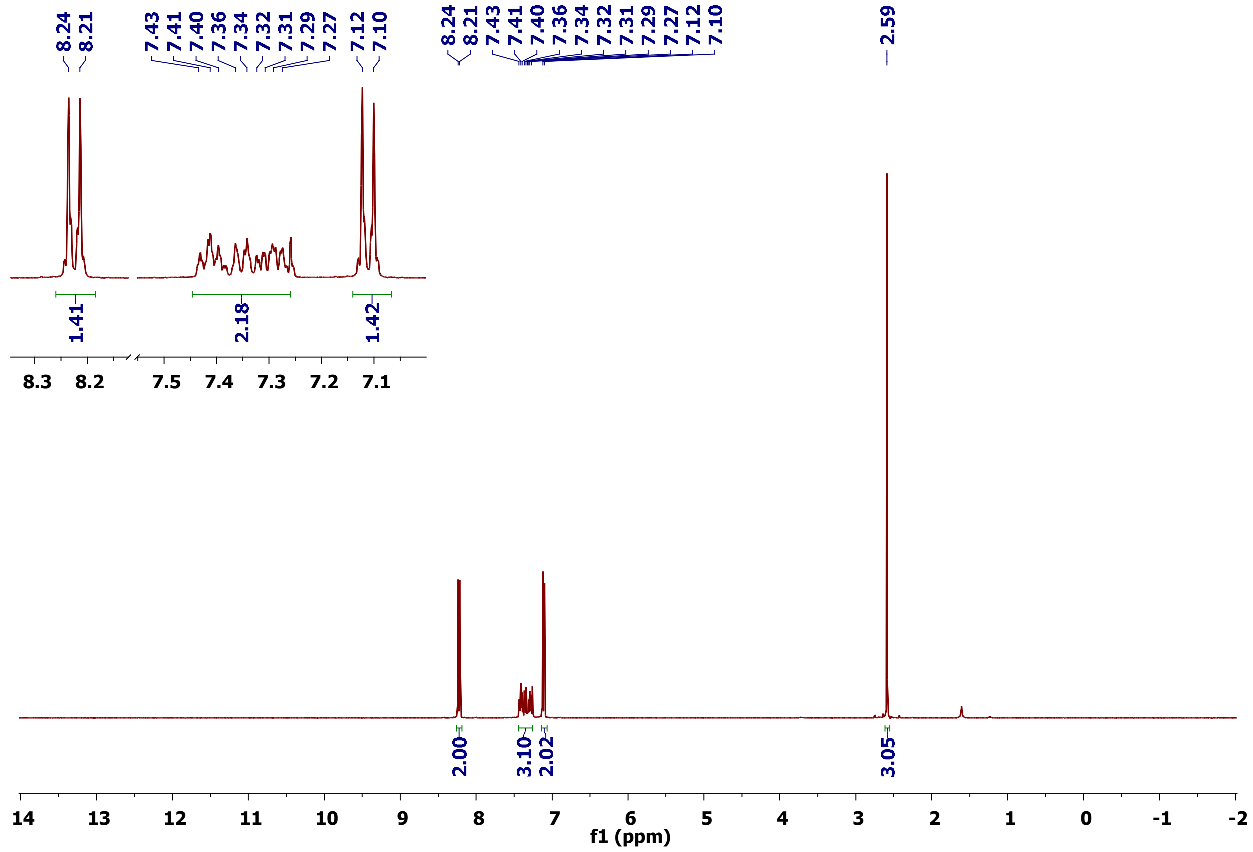

^{13}C NMR ($CDCl_3$) spectrum of (Z)-1-(4-(2,3-difluorophenyl)-5-((4-nitrophenyl)imino)-4,5-dihydro-1,3,4-thiadiazol-2-yl)ethan-1-one

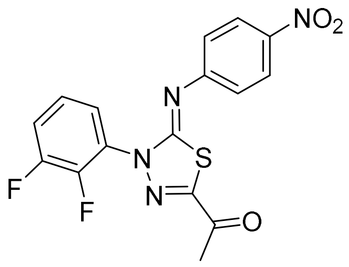

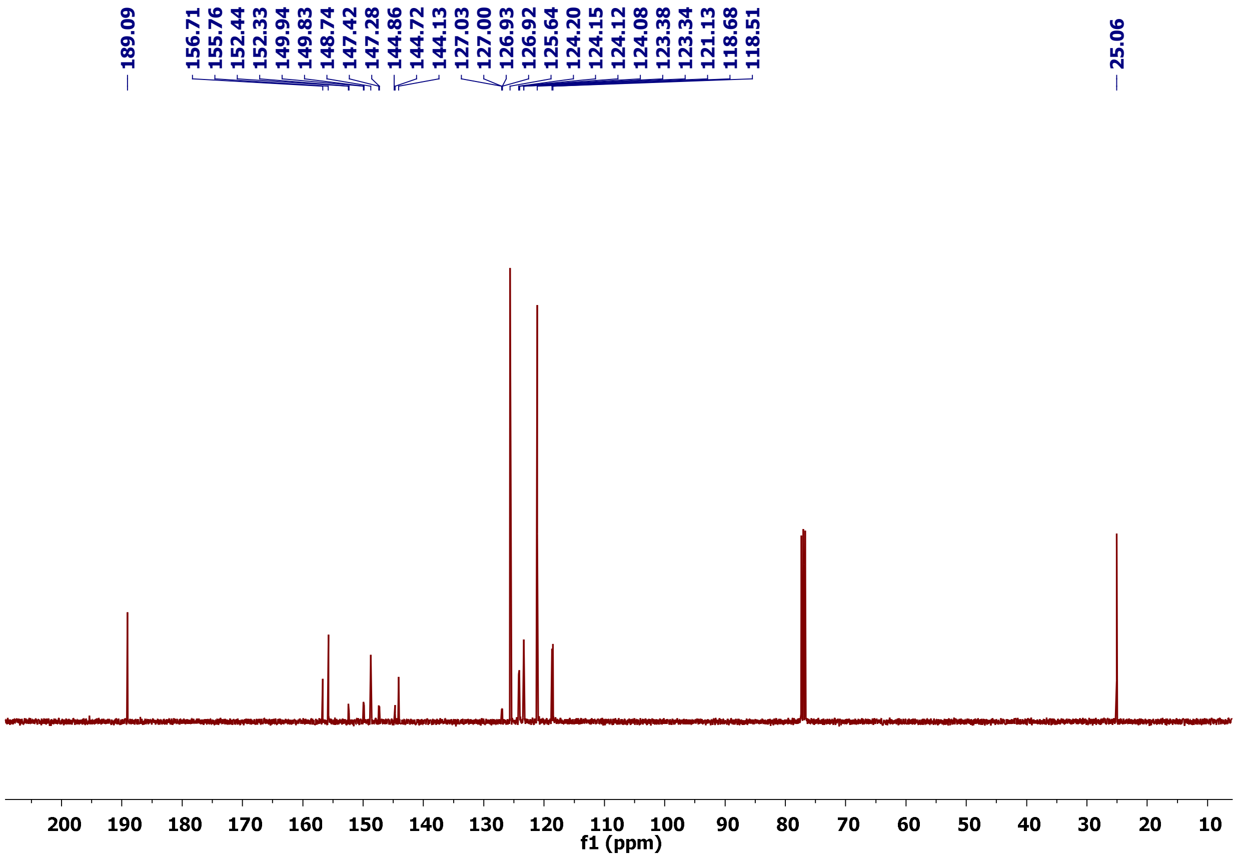

^{13}C CRAPT NMR ($CDCl_3$) spectrum of (Z)-1-(4-(2,3-difluorophenyl)-5-((4-nitrophenyl)imino)-4,5-dihydro-1,3,4-thiadiazol-2-yl)ethan-1-one

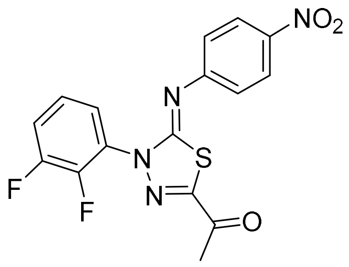

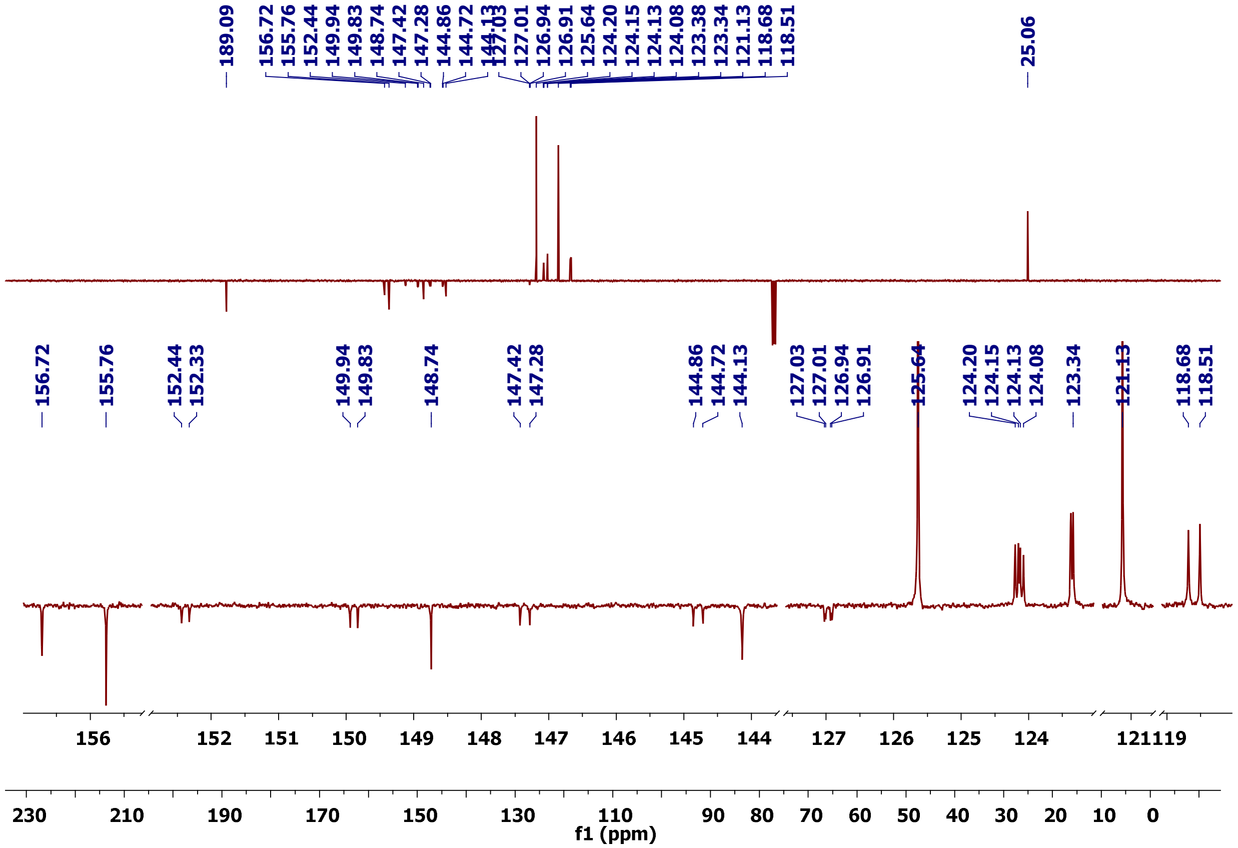

^{1}H-^{1}H gDQCOSY NMR ($CDCl_3$) spectrum of (Z)-1-(4-(2,3-difluorophenyl)-5-((4-nitrophenyl)imino)-4,5-dihydro-1,3,4-thiadiazol-2-yl)ethan-1-one

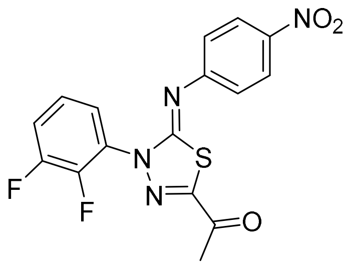

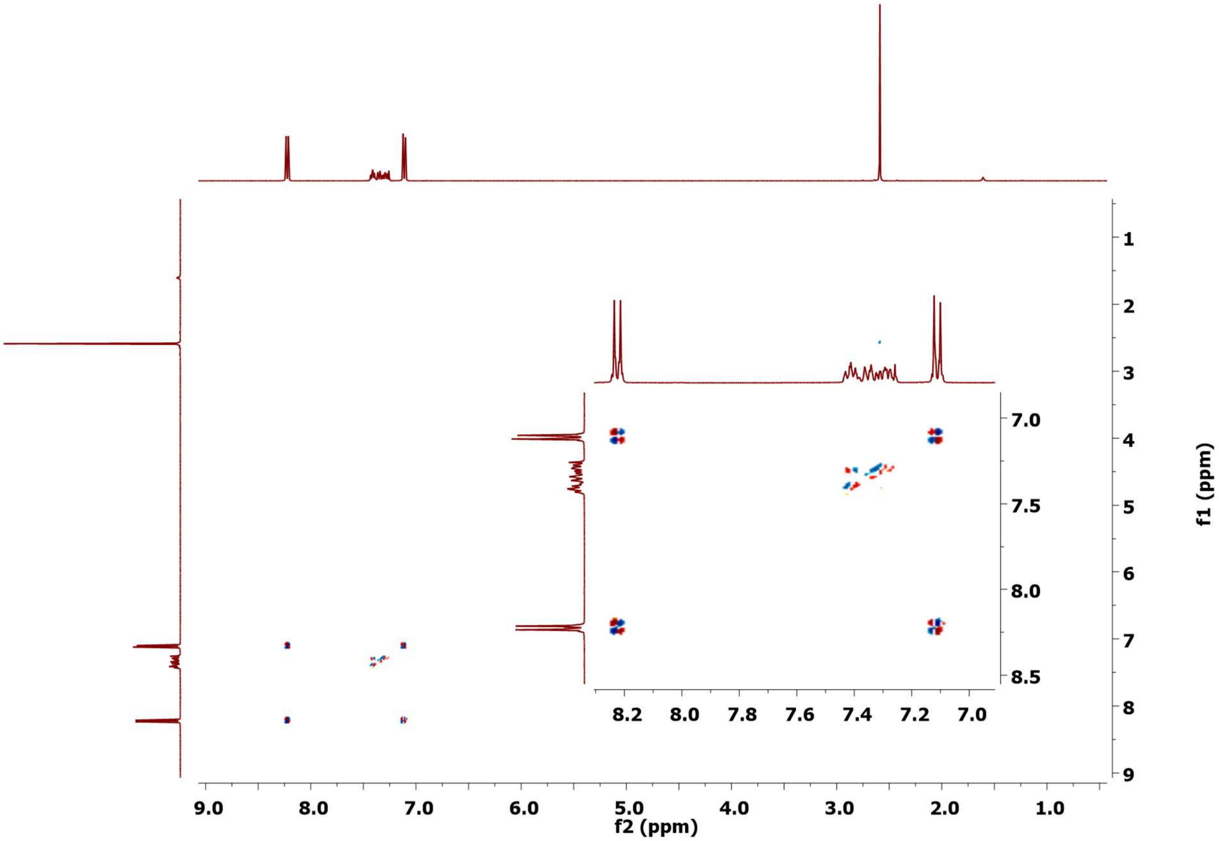

^{1}H-^{13}C-HSQC NMR ($CDCl_3$) spectrum of (Z)-1-(4-(2,3-difluorophenyl)-5-((4-nitrophenyl)imino)-4,5-dihydro-1,3,4-thiadiazol-2-yl)ethan-1-one

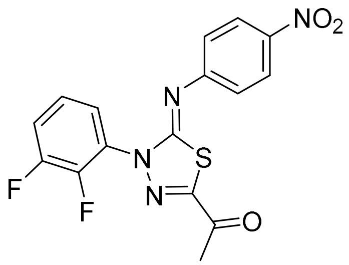

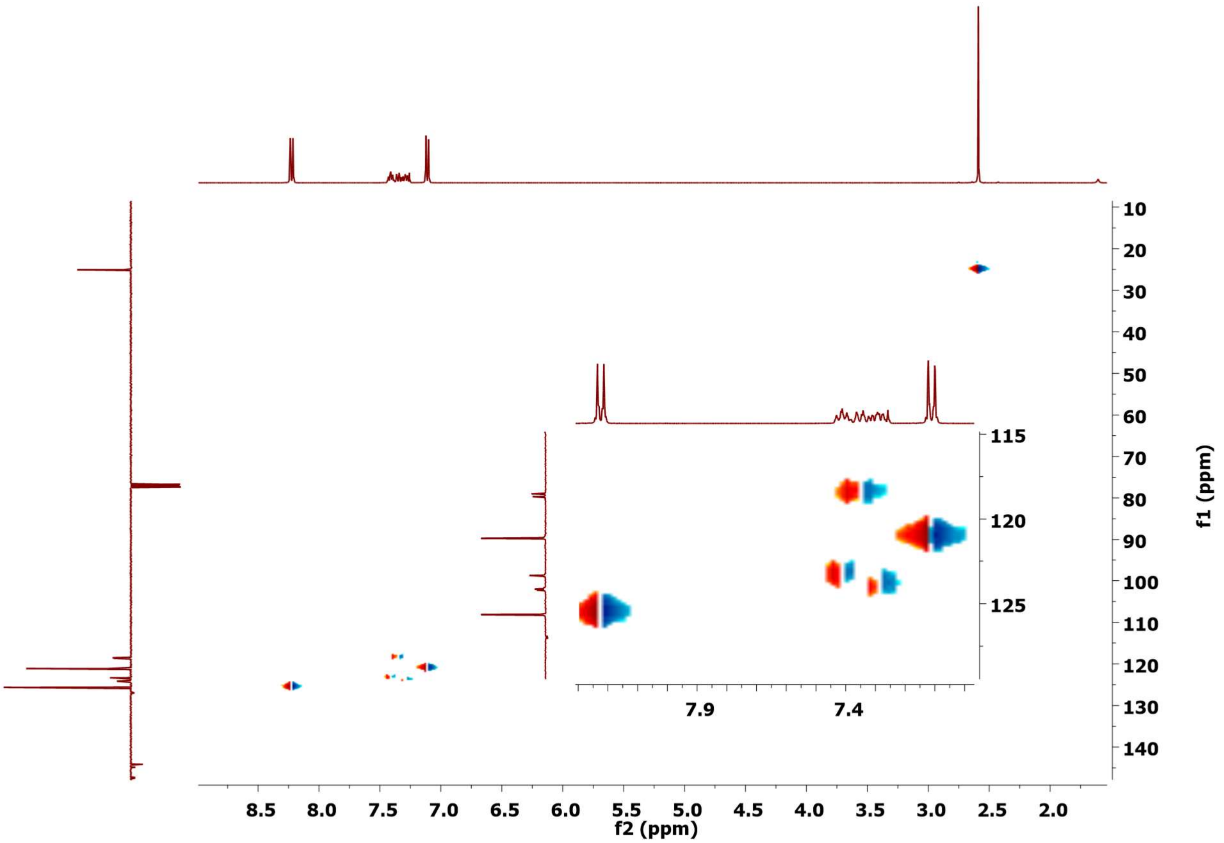

^{1}H-^{13}C-gHMBC NMR ($CDCl_3$) spectrum of (Z)-1-(4-(2,3-difluorophenyl)-5-((4-nitrophenyl)imino)-4,5-dihydro-1,3,4-thiadiazol-2-yl)ethan-1-one

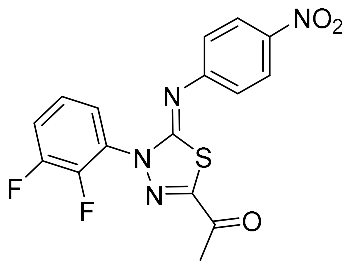

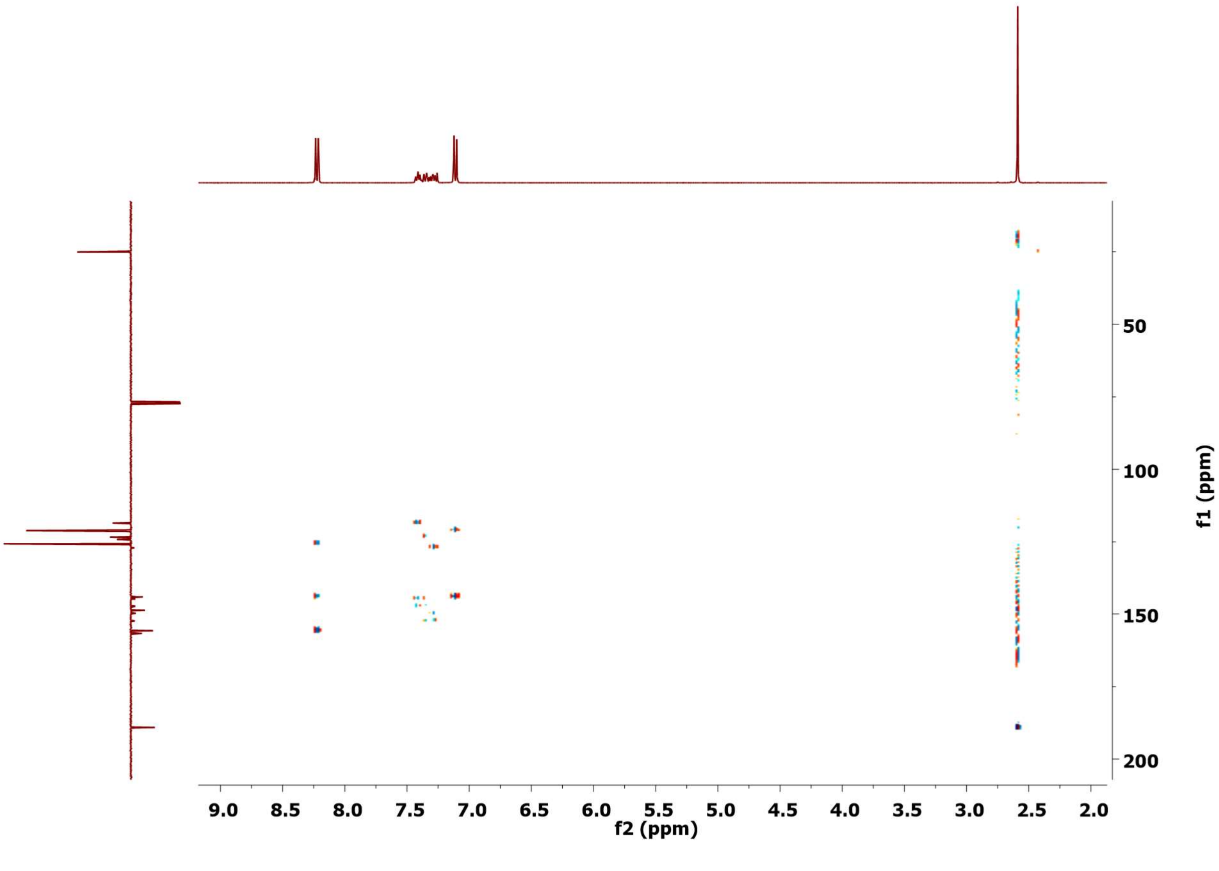

^1H NMR ($CDCl_3$) spectrum of (Z)-1-(5-((4-chlorophenyl)imino)-4-(2,3-difluorophenyl)-4,5-dihydro-1,3,4-thiadiazol-2-yl)ethan-1-one

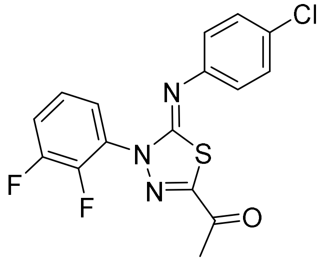

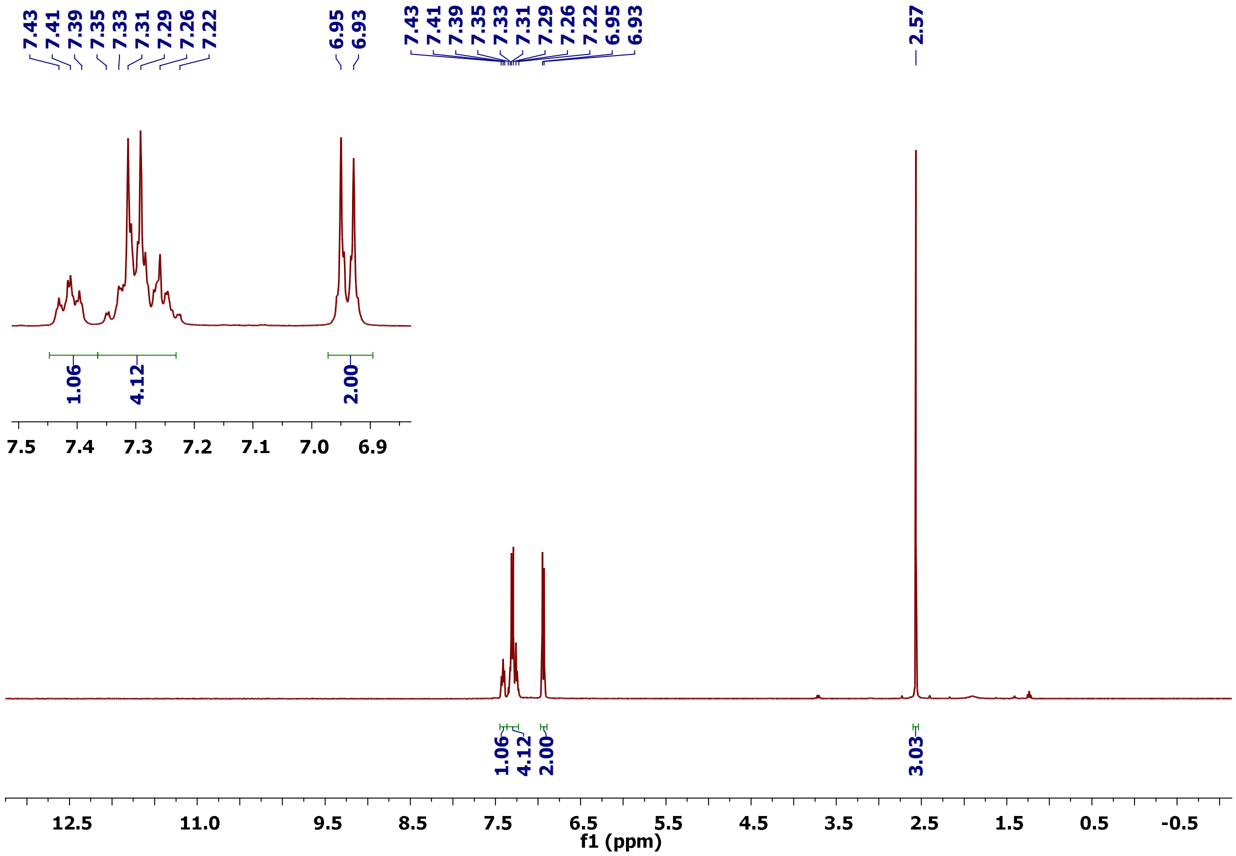

^{13}C NMR ($CDCl_3$) spectrum of (Z)-1-(5-((4-chlorophenyl)imino)-4-(2,3-difluorophenyl)-4,5-dihydro-1,3,4-thiadiazol-2-yl)ethan-1-one

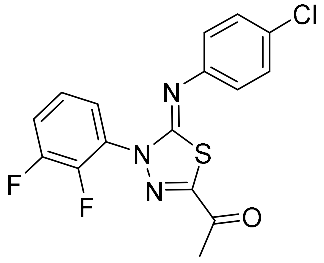

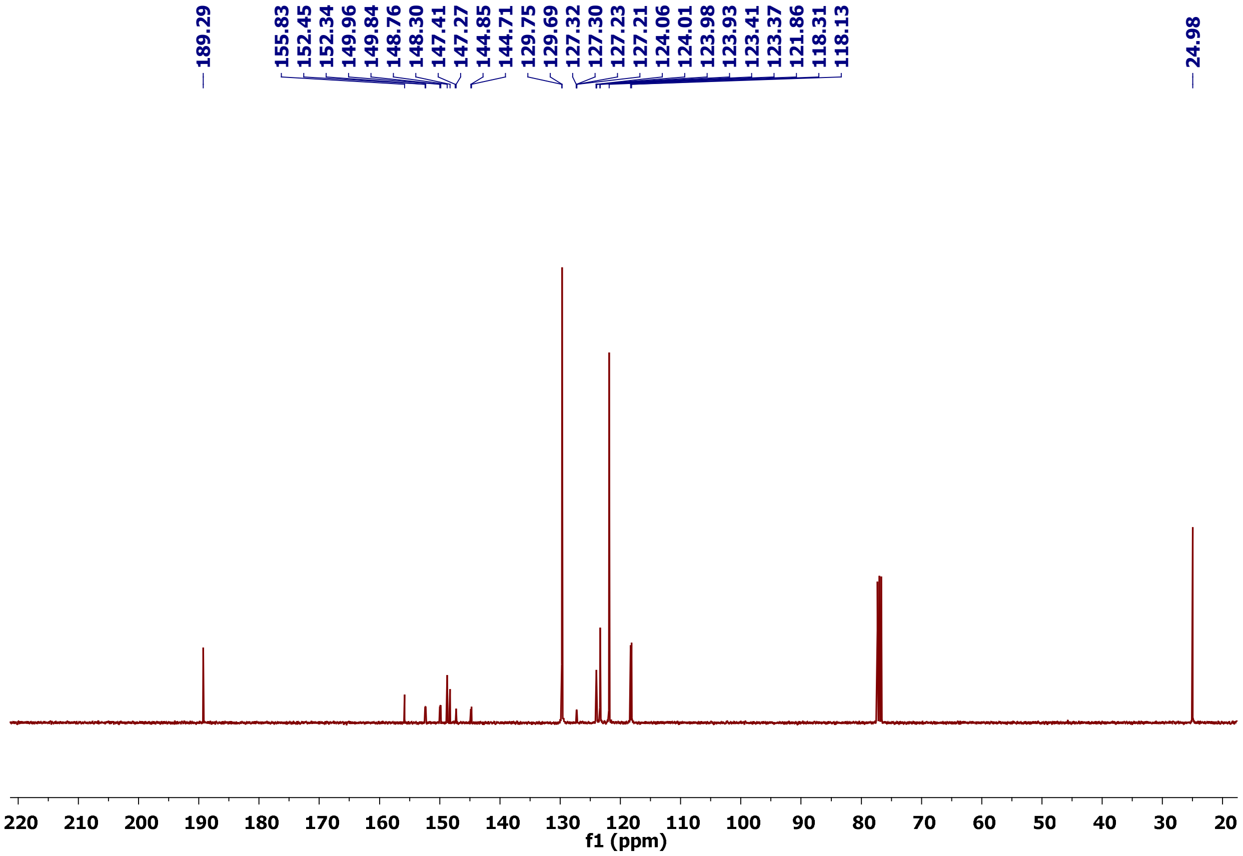

^{13}C CRAPT NMR ($CDCl_3$) spectrum of (Z)-1-(5-((4-chlorophenyl)imino)-4-(2,3-difluorophenyl)-4,5-dihydro-1,3,4-thiadiazol-2-yl)ethan-1-one

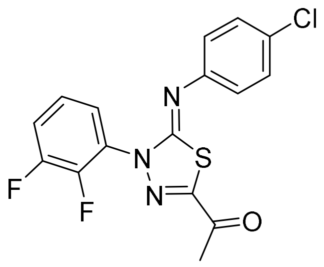

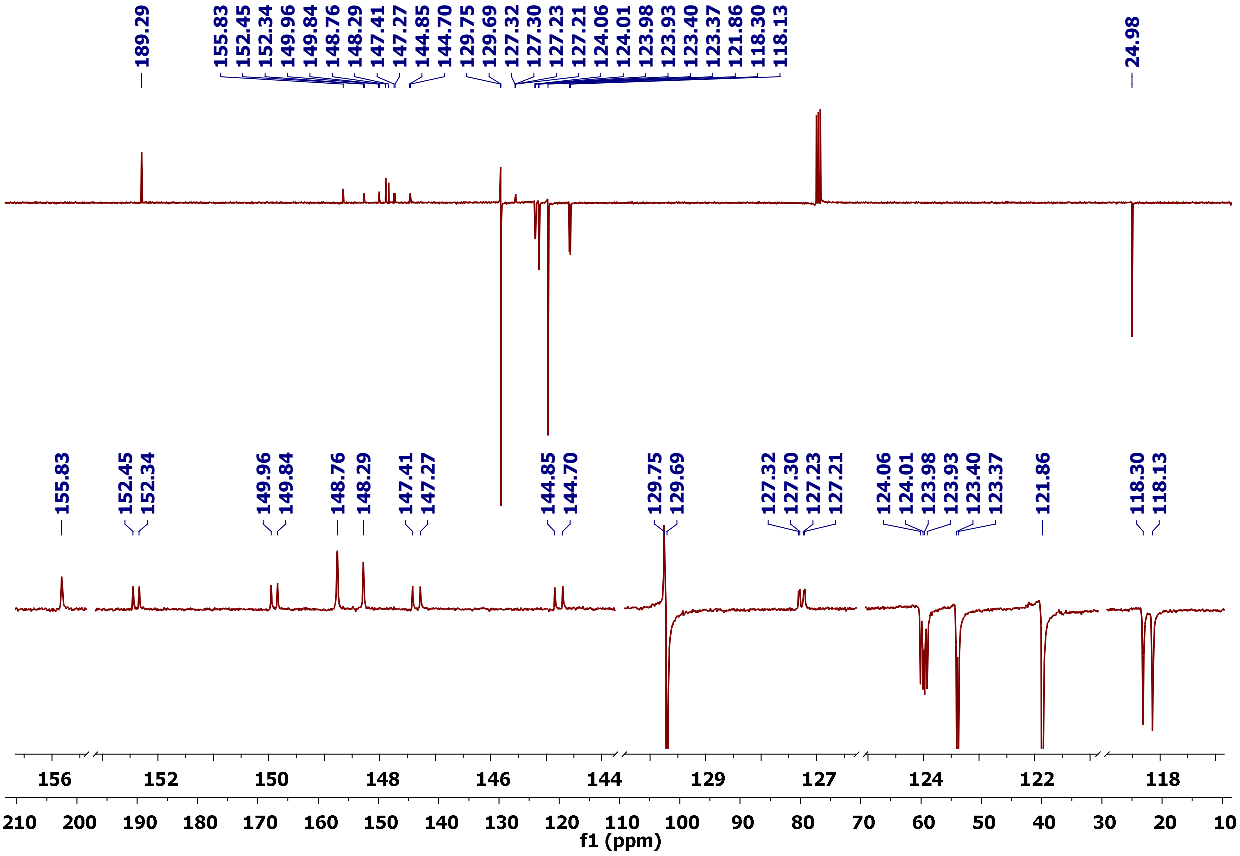

^1H-^1H gDQCOSY NMR ($CDCl_3$) spectrum of (Z)-1-(5-((4-chlorophenyl)imino)-4-(2,3-difluorophenyl)-4,5-dihydro-1,3,4-thiadiazol-2-yl)ethan-1-one

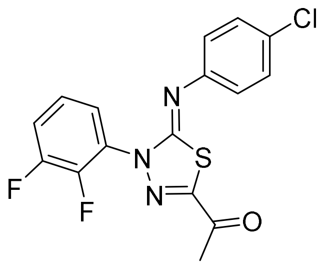

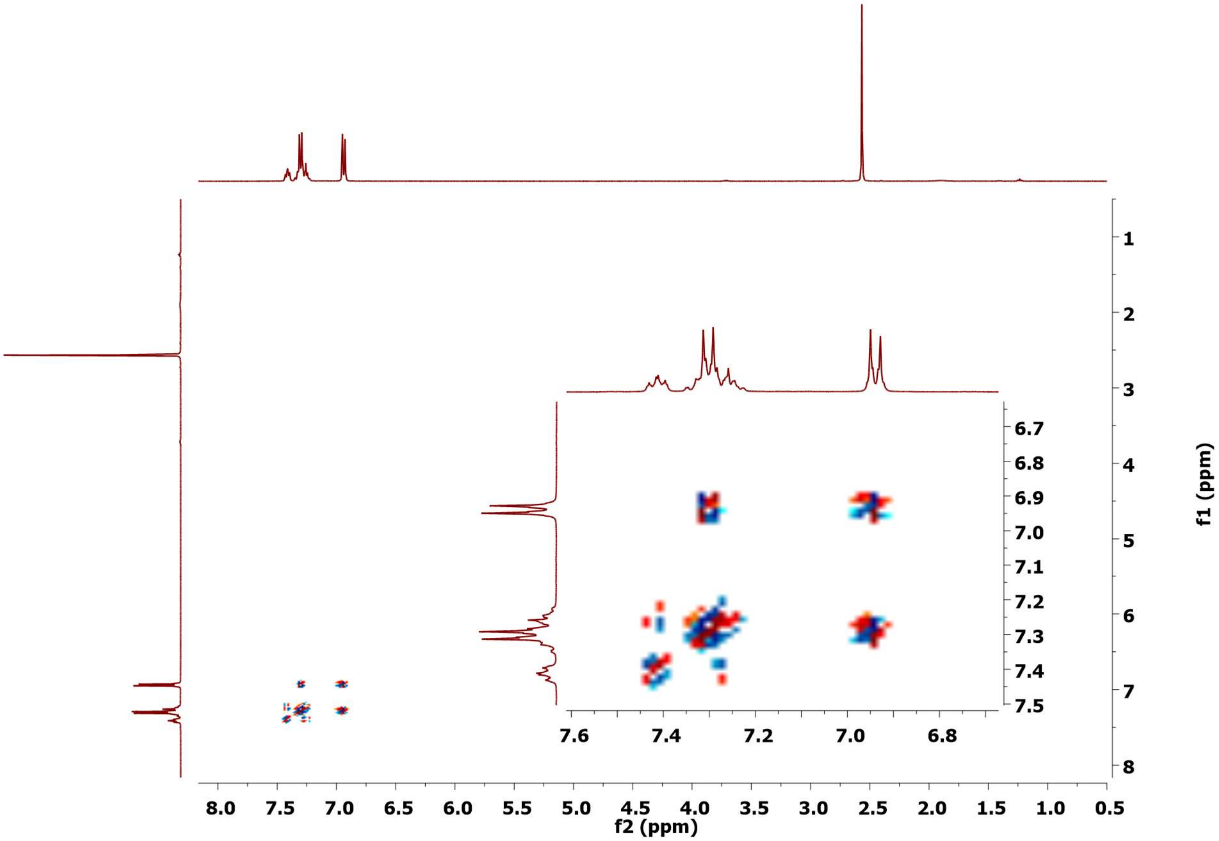

^{1}H-^{13}C-HSQC NMR ($CDCl_3$) spectrum of (Z)-1-(5-((4-chlorophenyl)imino)-4-(2,3-difluorophenyl)-4,5-dihydro-1,3,4-thiadiazol-2-yl)ethan-1-one

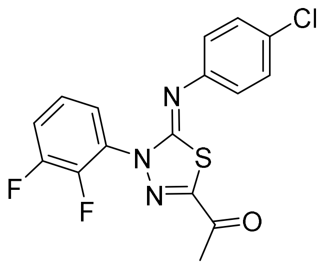

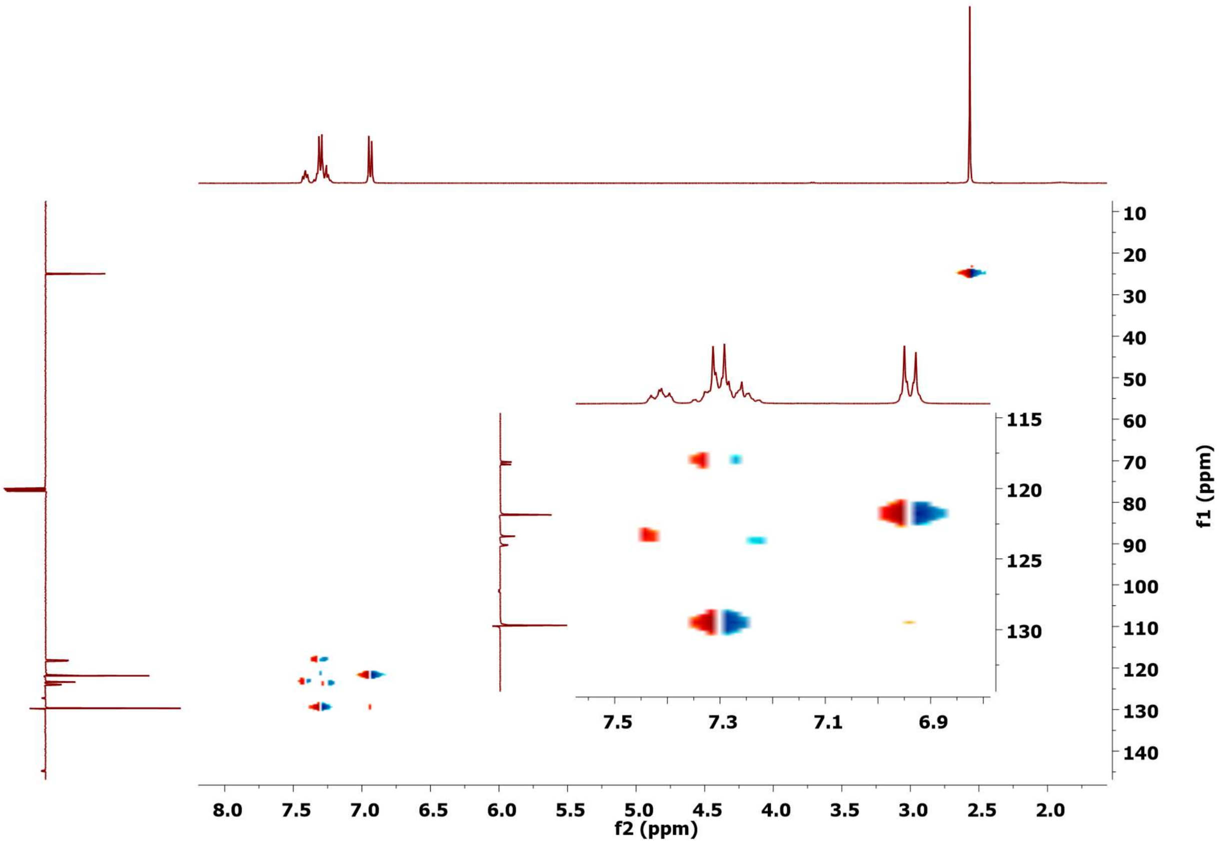

^{1}H-^{13}C-gHMBC NMR ($CDCl_3$) spectrum of (Z)-1-(5-((4-chlorophenyl)imino)-4-(2,3-difluorophenyl)-4,5-dihydro-1,3,4-thiadiazol-2-yl)ethan-1-one

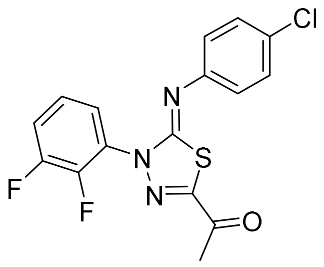

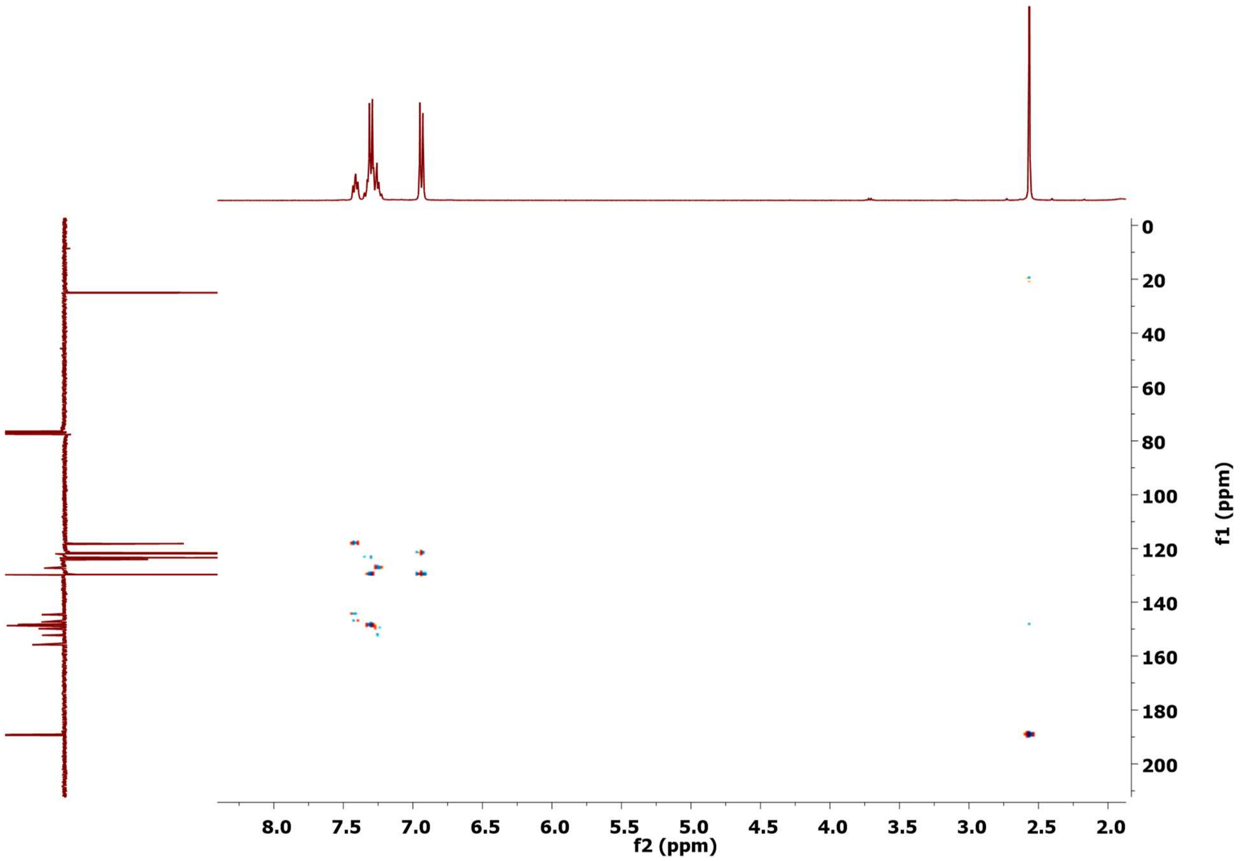

^{1}H NMR ($CDCl_3$) spectrum of (Z)-1-(4-(4-bromophenyl)-5-((3-fluorophenyl)imino)-4,5-dihydro-1,3,4-thiadiazol-2-yl)ethan-1-one

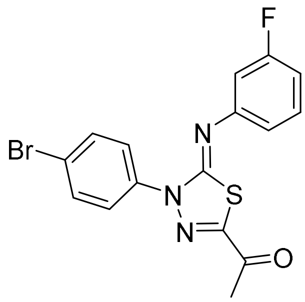

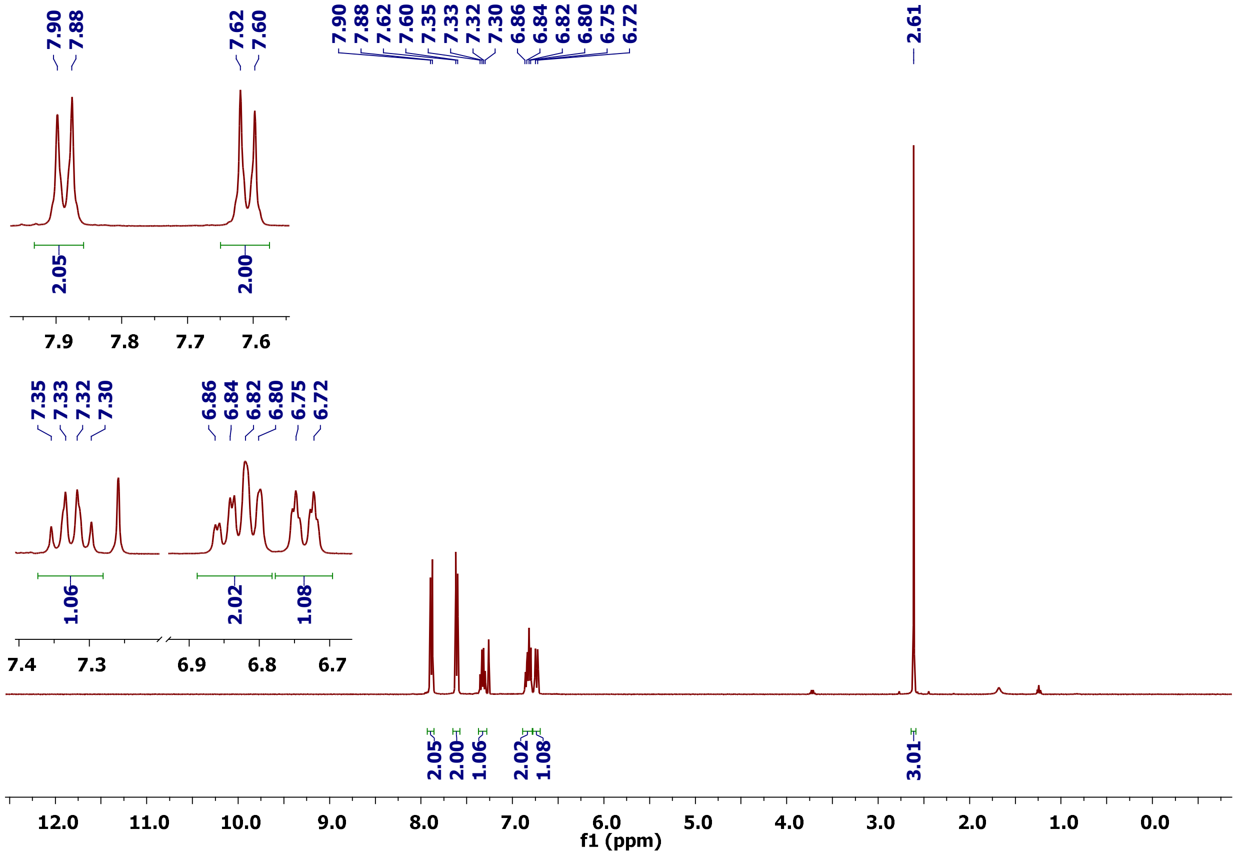

^{13}C NMR ($CDCl_3$) spectrum of (Z)-1-(4-(4-bromophenyl)-5-((3-fluorophenyl)imino)-4,5-dihydro-1,3,4-thiadiazol-2-yl)ethan-1-one

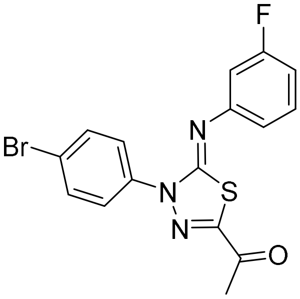

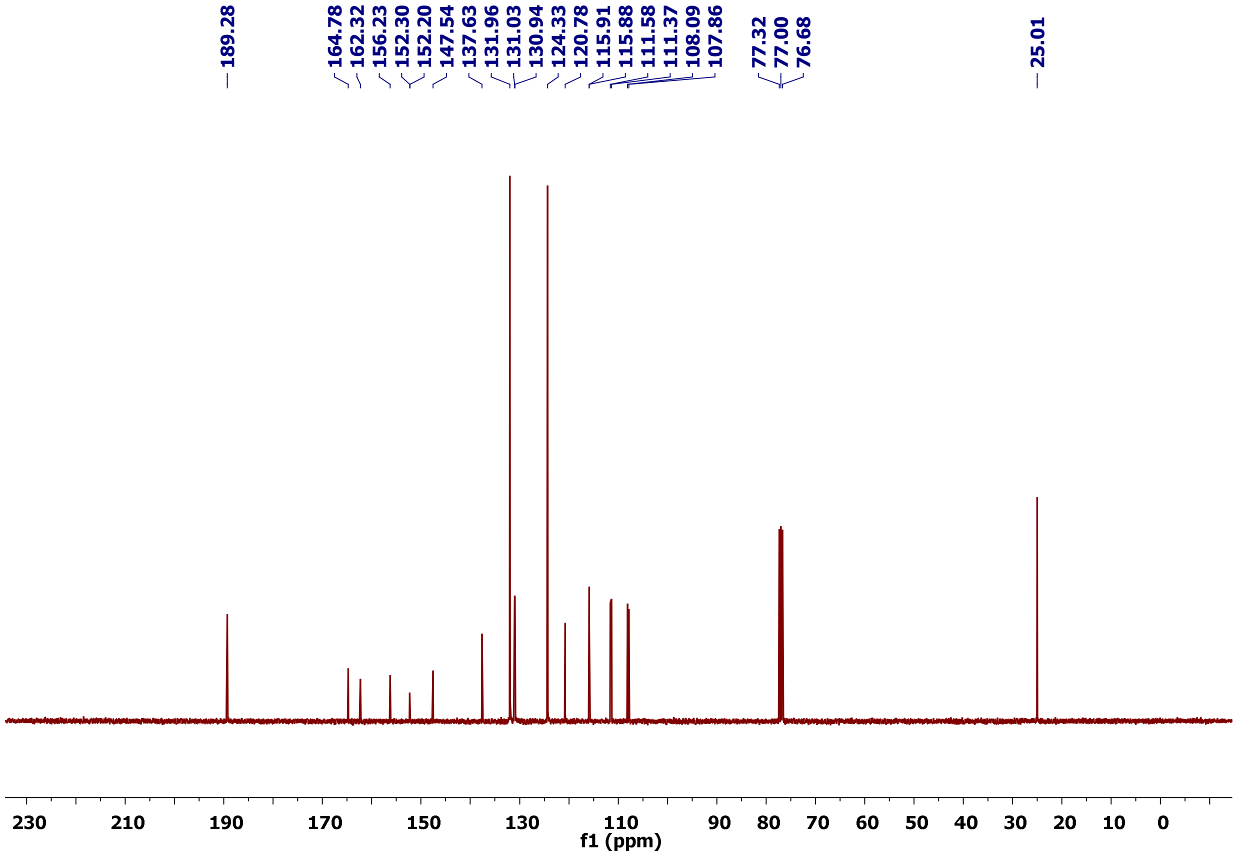

^{13}C CRAPT NMR ($CDCl_3$) spectrum of (Z)-1-(4-(4-bromophenyl)-5-((3-fluorophenyl)imino)-4,5-dihydro-1,3,4-thiadiazol-2-yl)ethan-1-one

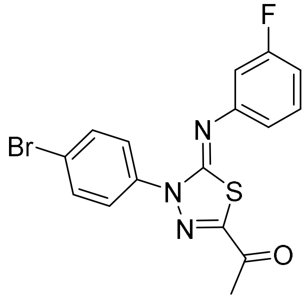

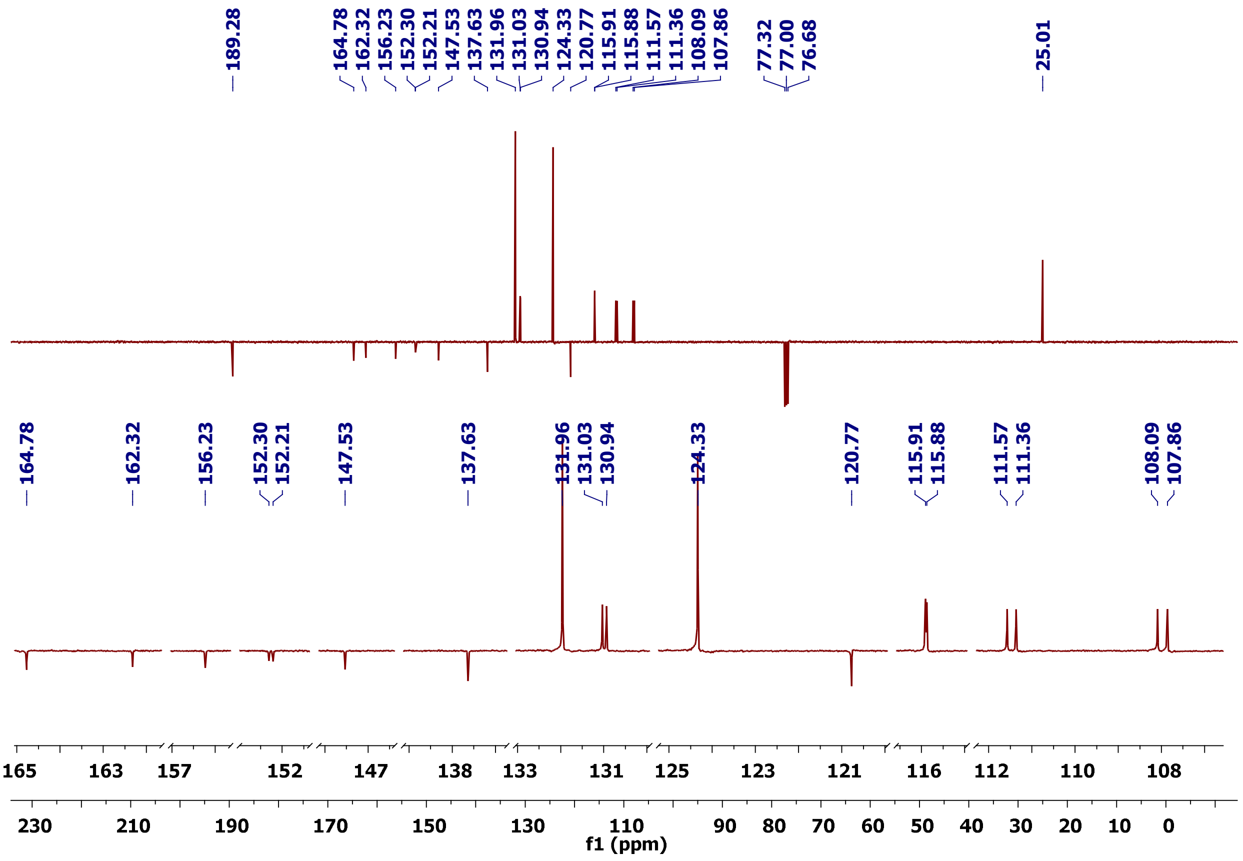

^{1}H-^{1}H gDQCOSY NMR ($CDCl_3$) spectrum of (Z)-1-(4-(4-bromophenyl)-5-((3-fluorophenyl)imino)-4,5-dihydro-1,3,4-thiadiazol-2-yl)ethan-1-one

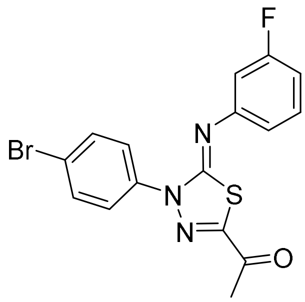

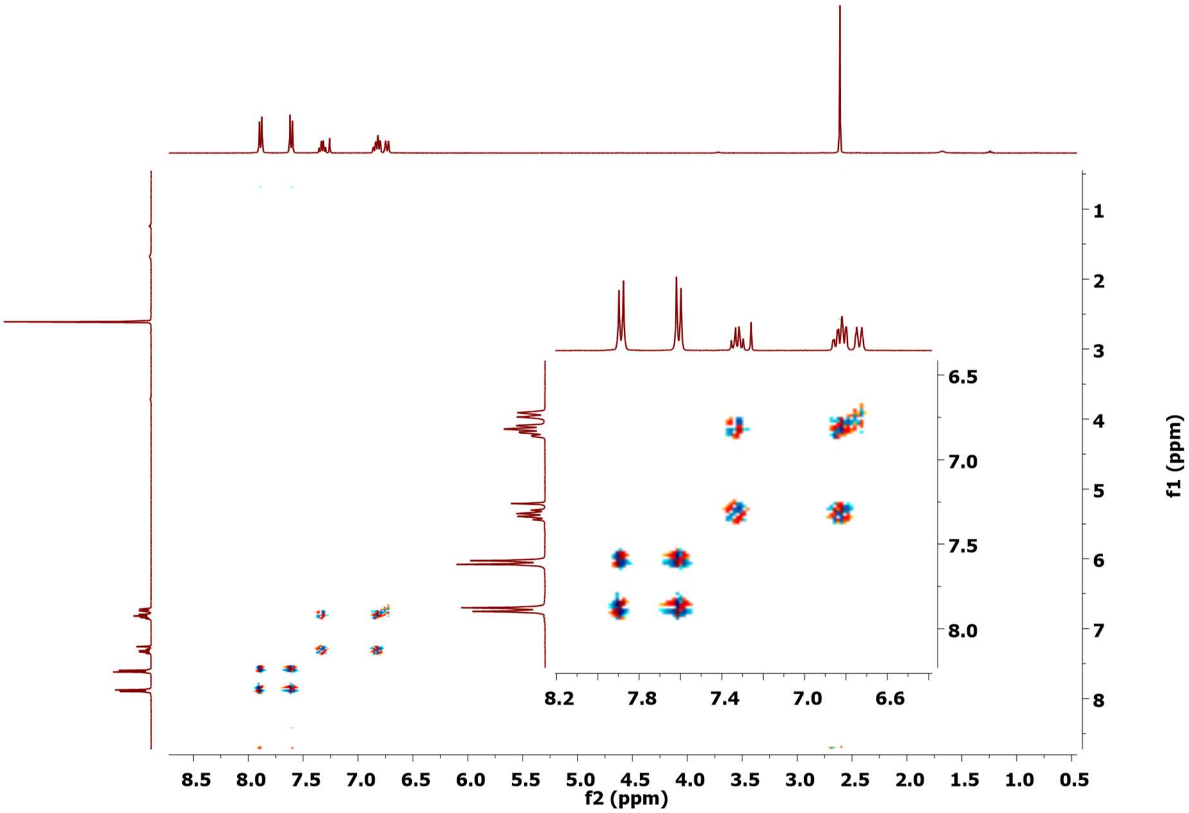

^{1}H-^{13}C-HSQC NMR ($CDCl_3$) spectrum of (Z)-1-(4-(4-bromophenyl)-5-((3-fluorophenyl)imino)-4,5-dihydro-1,3,4-thiadiazol-2-yl)ethan-1-one

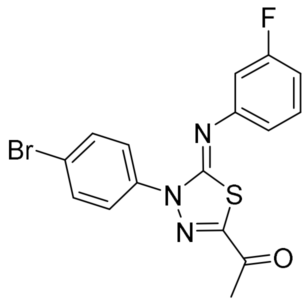

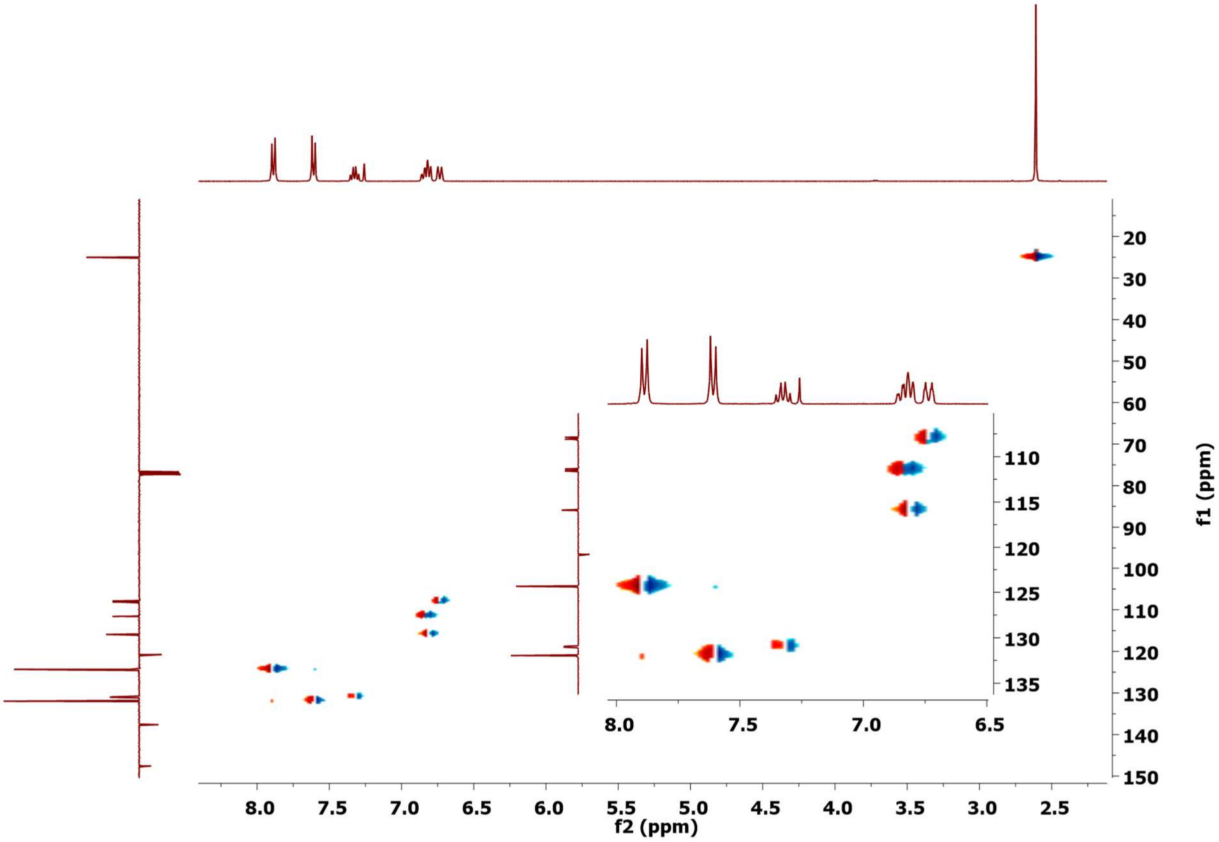

^{1}H-^{13}C-gHMBC NMR ($CDCl_3$) spectrum of (Z)-1-(4-(4-bromophenyl)-5-((3-fluorophenyl)imino)-4,5-dihydro-1,3,4-thiadiazol-2-yl)ethan-1-one

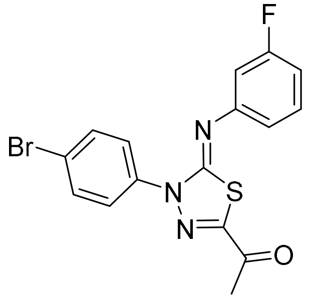

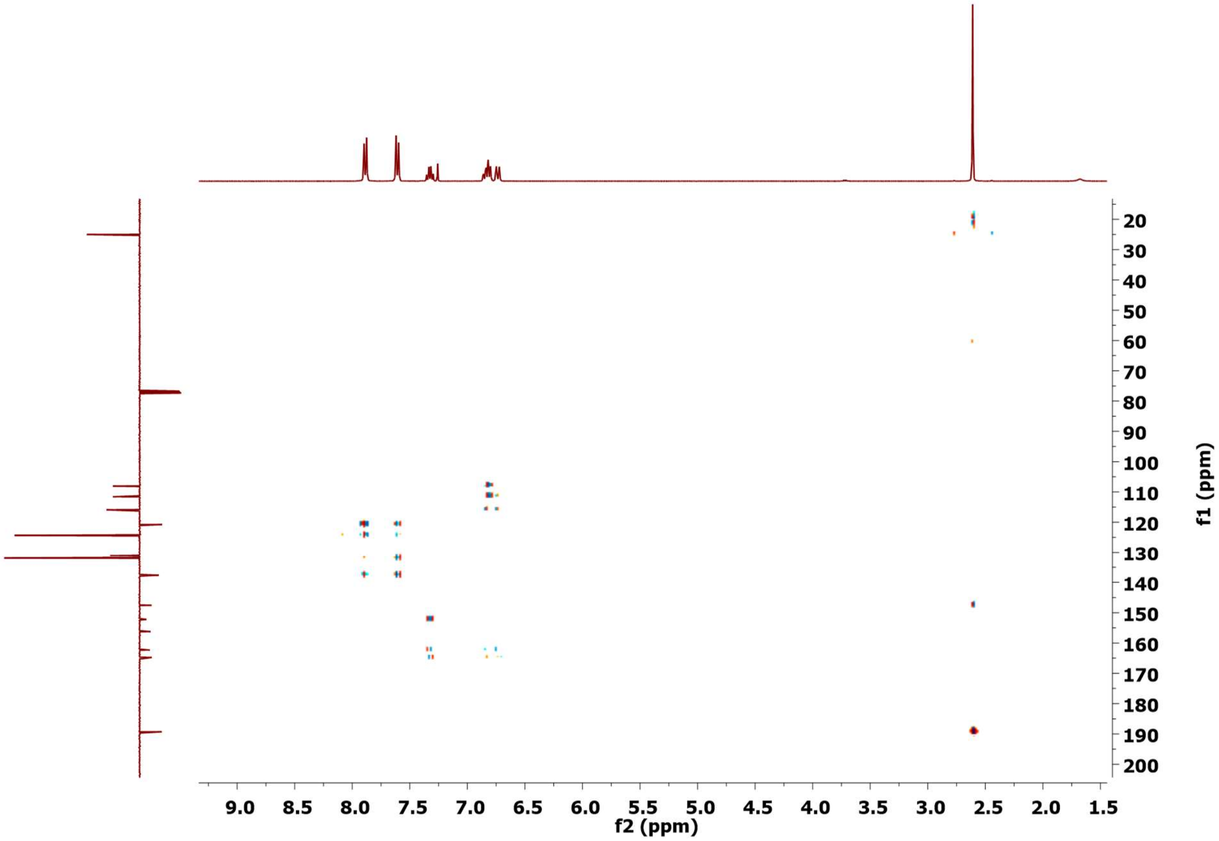

^{1}H NMR ($CDCl_3$) spectrum of (Z)-1-(4-(4-chlorophenyl)-5-((3-fluorophenyl)imino)-4,5-dihydro-1,3,4-thiadiazol-2-yl)ethan-1-one

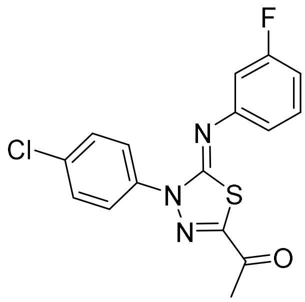

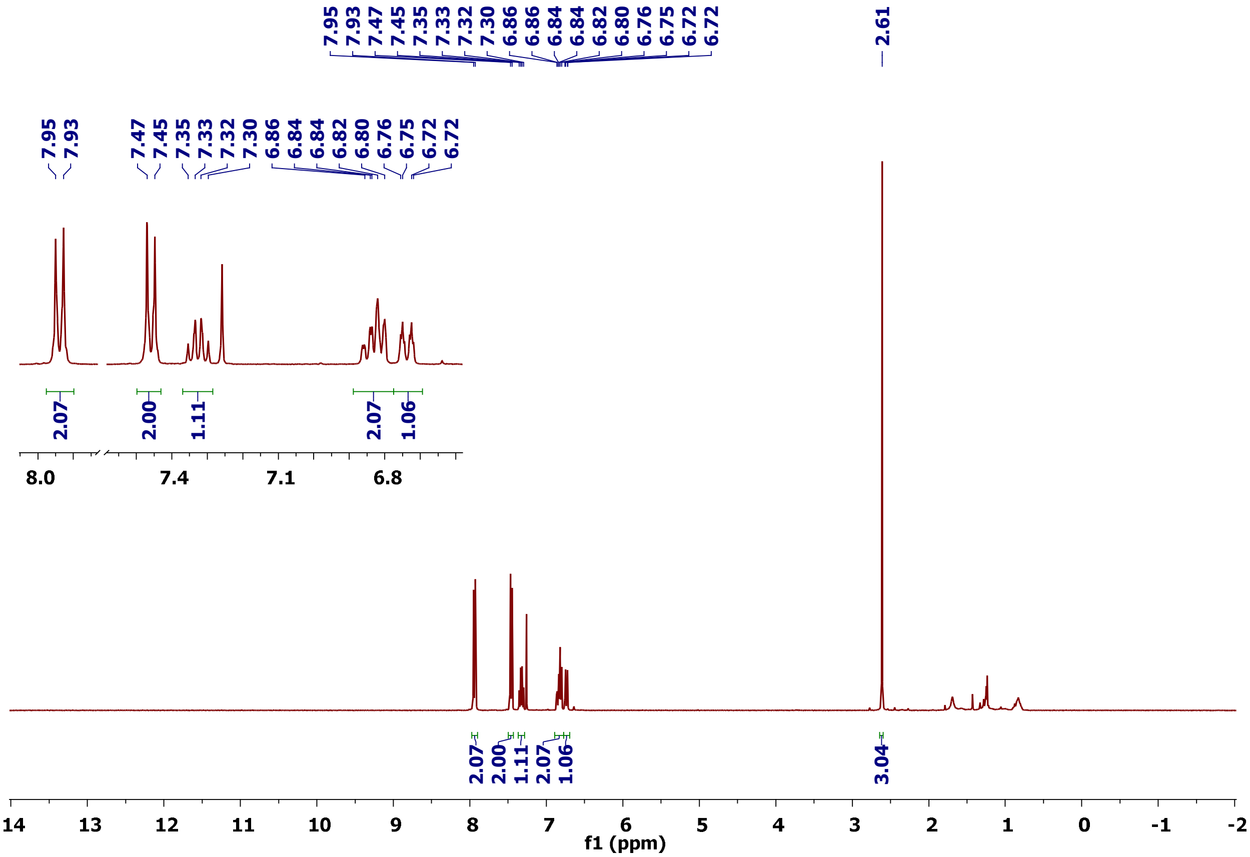

^{13}C NMR ($CDCl_3$) spectrum of (Z)-1-(4-(4-chlorophenyl)-5-((3-fluorophenyl)imino)-4,5-dihydro-1,3,4-thiadiazol-2-yl)ethan-1-one

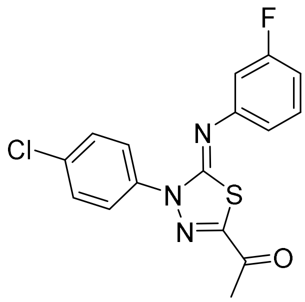

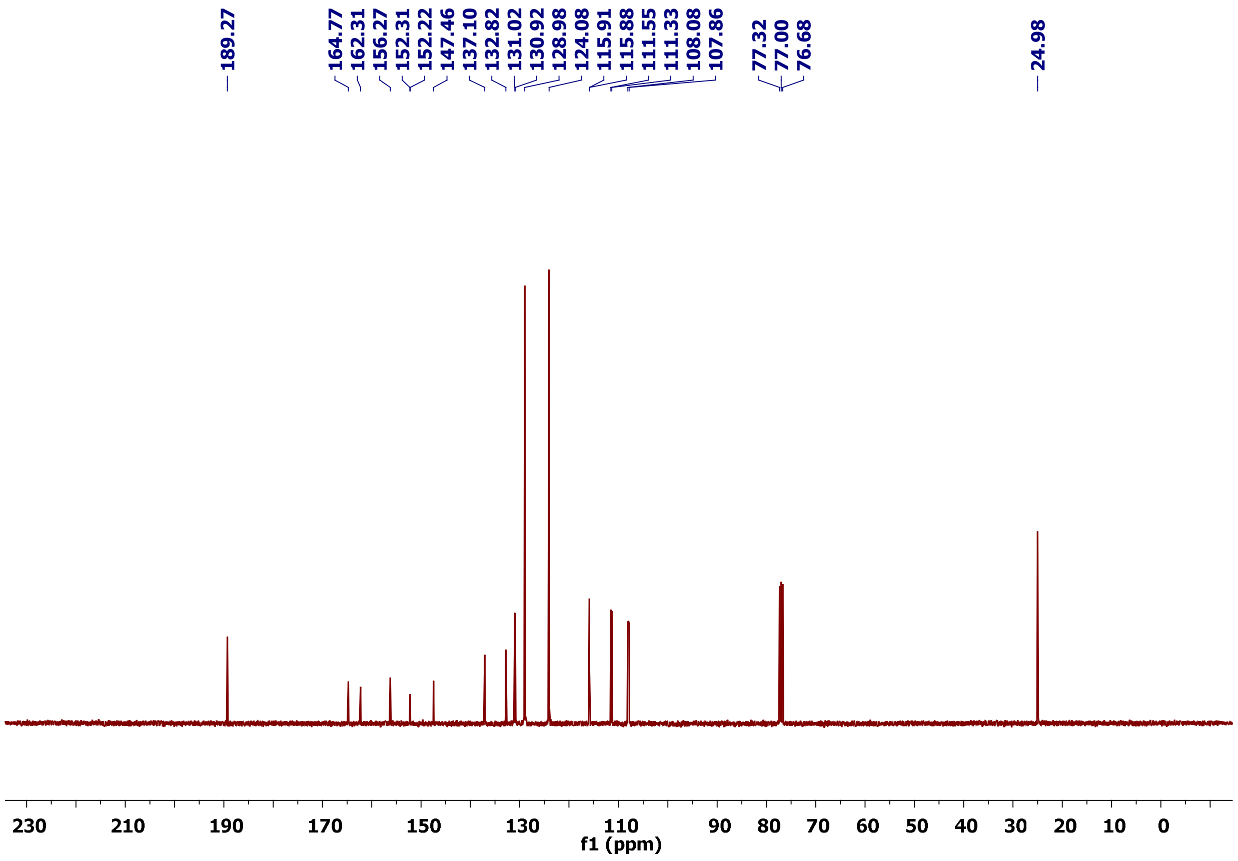

^{13}C CRAPT NMR ($CDCl_3$) spectrum of (Z)-1-(4-(4-chlorophenyl)-5-((3-fluorophenyl)imino)-4,5-dihydro-1,3,4-thiadiazol-2-yl)ethan-1-one

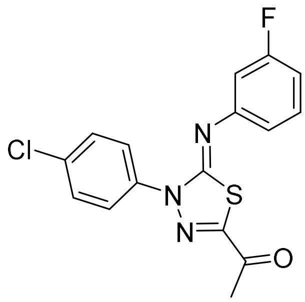

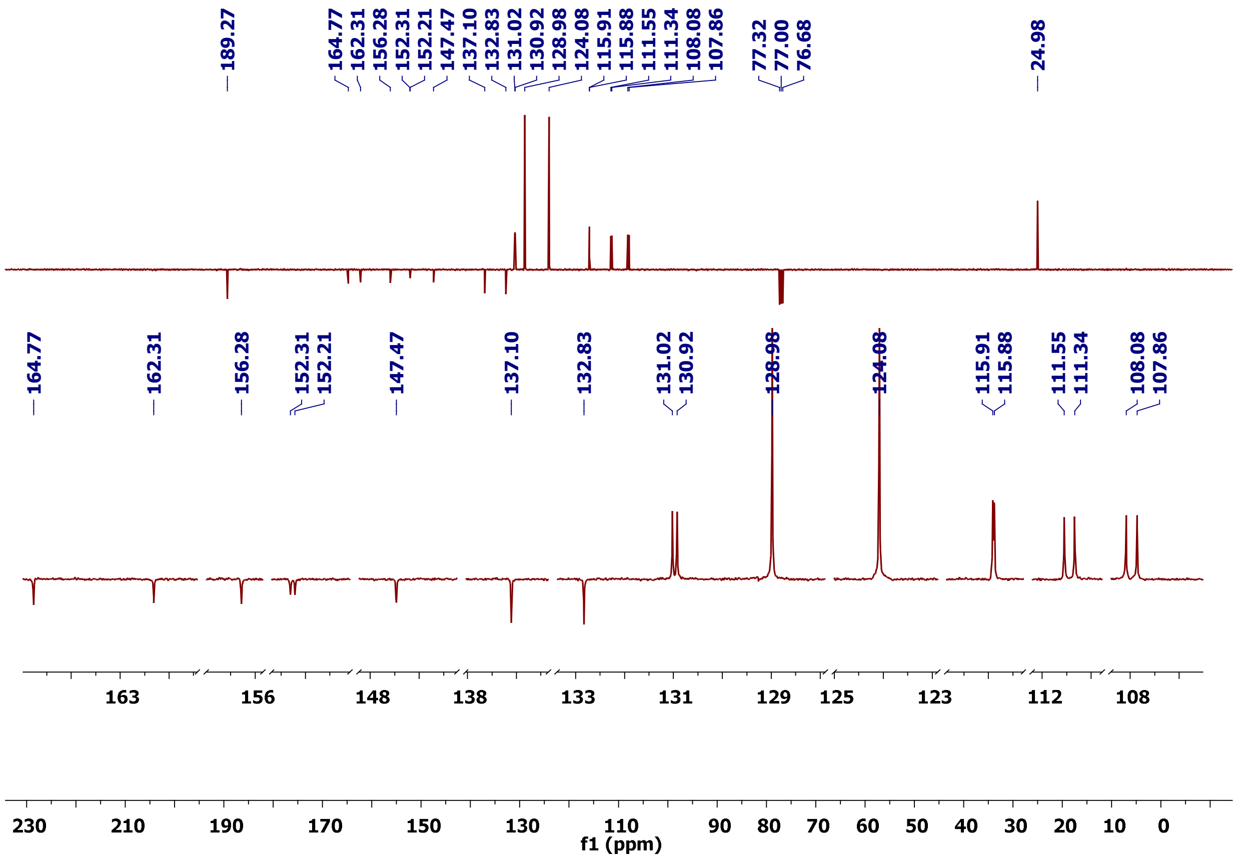

^{1}H-^{1}H gDQCOSY NMR ($CDCl_3$) spectrum of (Z)-1-(4-(4-chlorophenyl)-5-((3-fluorophenyl)imino)-4,5-dihydro-1,3,4-thiadiazol-2-yl)ethan-1-one

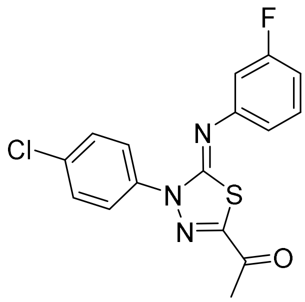

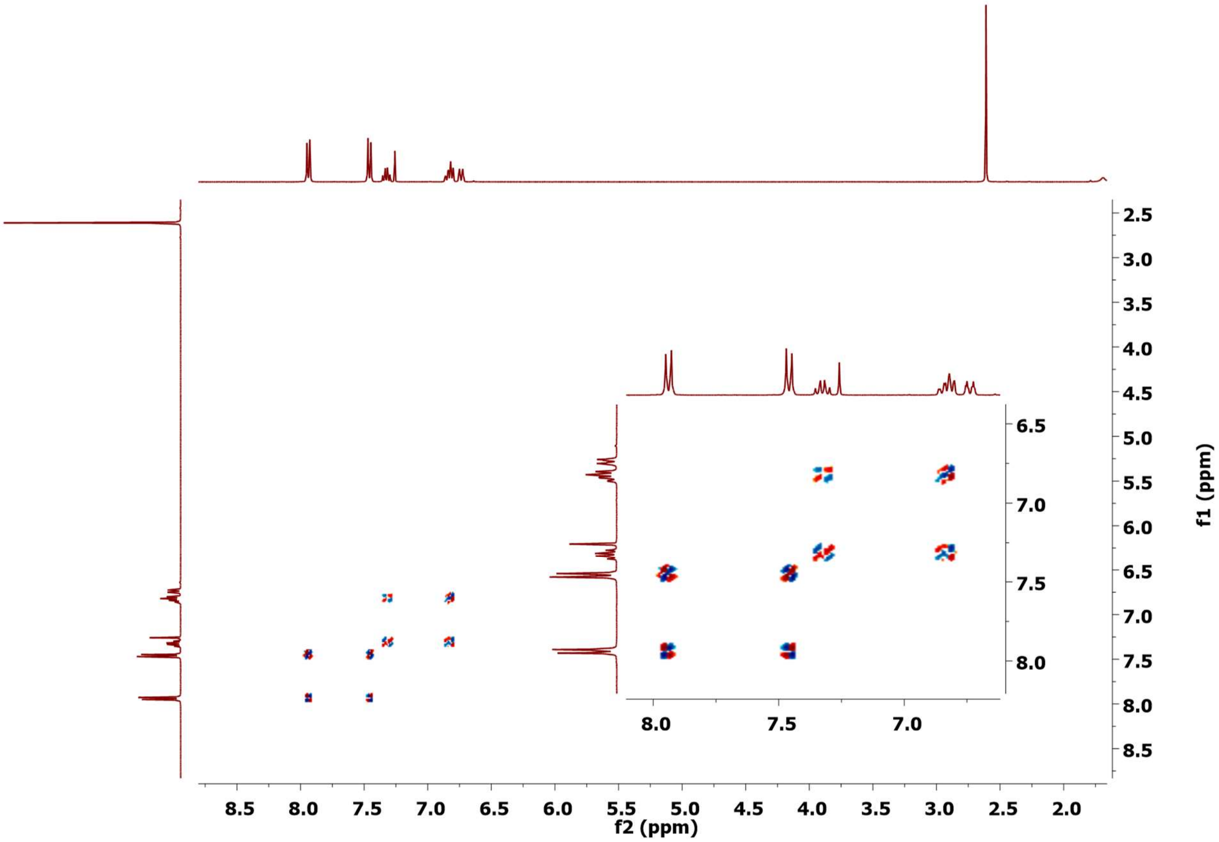

^{1}H-^{13}C-HSQC NMR ($CDCl_3$) spectrum of (Z)-1-(4-(4-chlorophenyl)-5-((3-fluorophenyl)imino)-4,5-dihydro-1,3,4-thiadiazol-2-yl)ethan-1-one

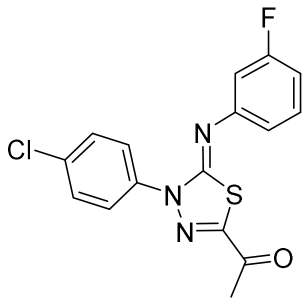

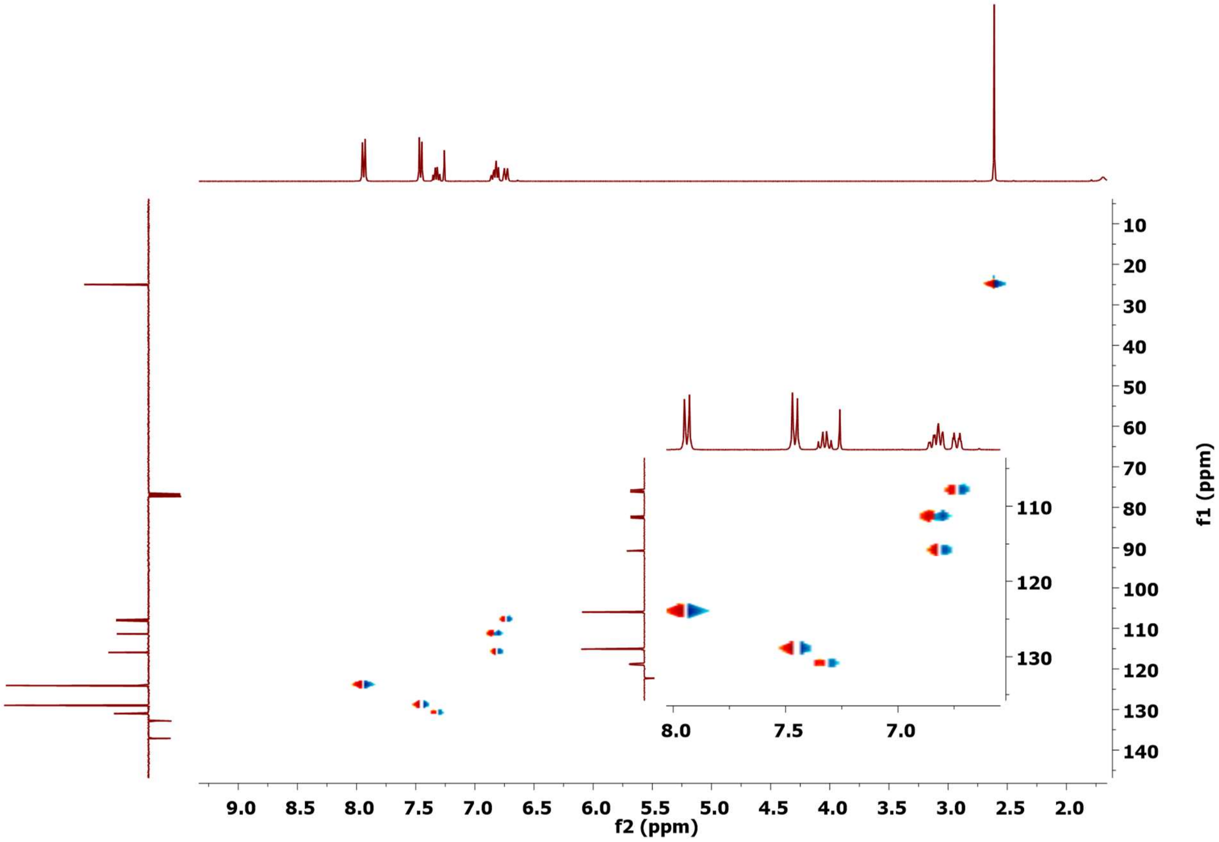

^{1}H-^{13}C-gHMBC NMR ($CDCl_3$) spectrum of (Z)-1-(4-(4-chlorophenyl)-5-((3-fluorophenyl)imino)-4,5-dihydro-1,3,4-thiadiazol-2-yl)ethan-1-one

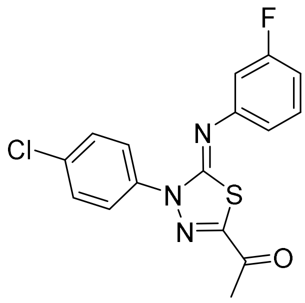

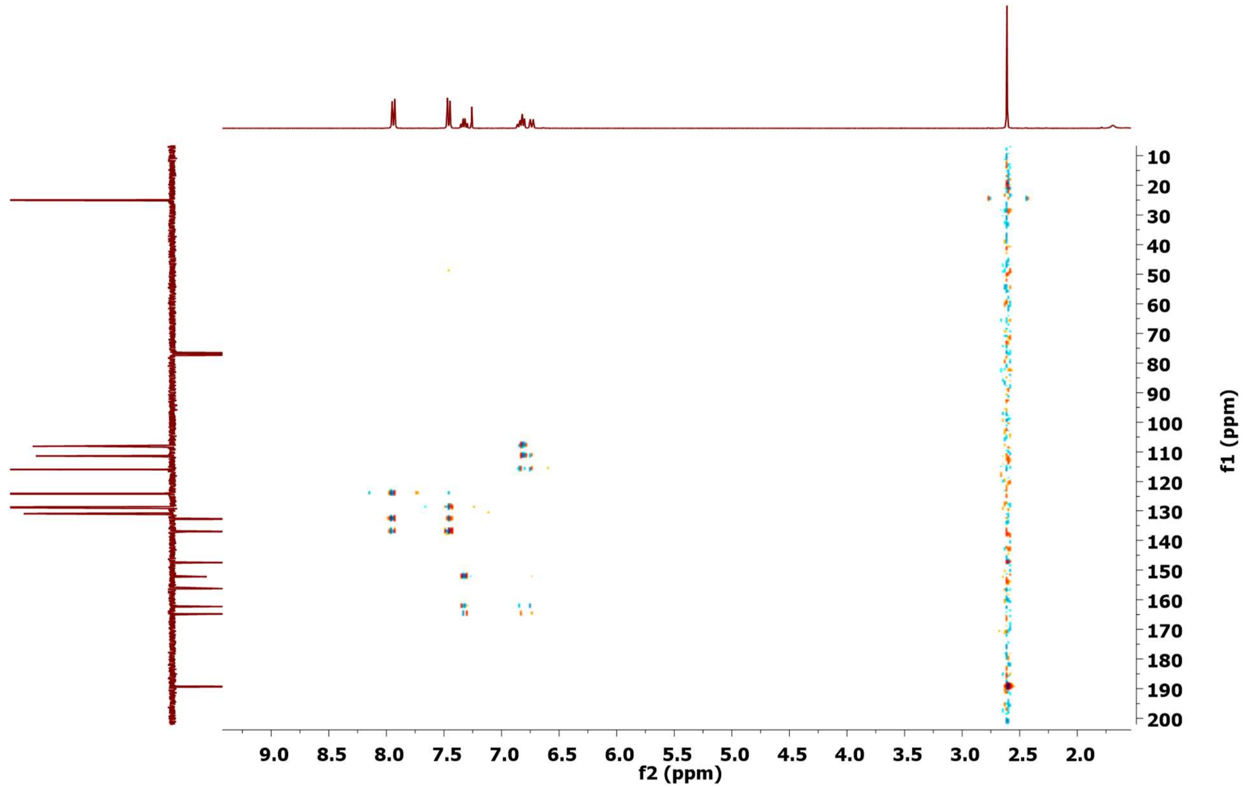

^{1}H NMR ($CDCl_3$) spectrum of (Z)-1-(5-((3-fluorophenyl)imino)-4-(4-iodophenyl)-4,5-dihydro-1,3,4-thiadiazol-2-yl)ethan-1-one

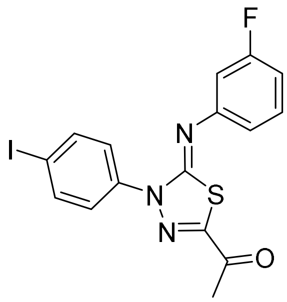

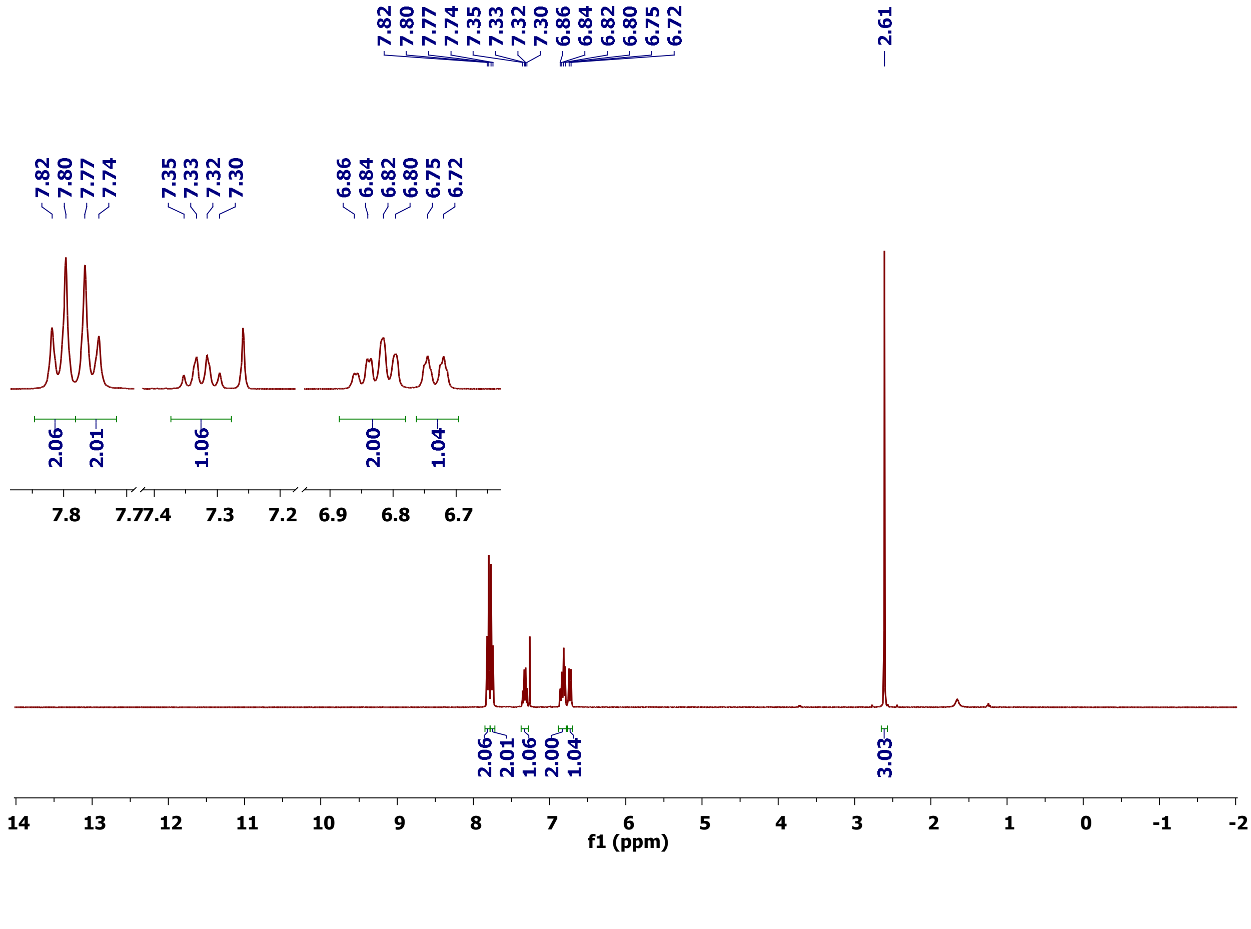

^{13}C NMR ($CDCl_3$) spectrum of (Z)-1-(5-((3-fluorophenyl)imino)-4-(4-iodophenyl)-4,5-dihydro-1,3,4-thiadiazol-2-yl)ethan-1-one

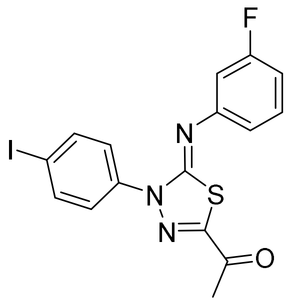

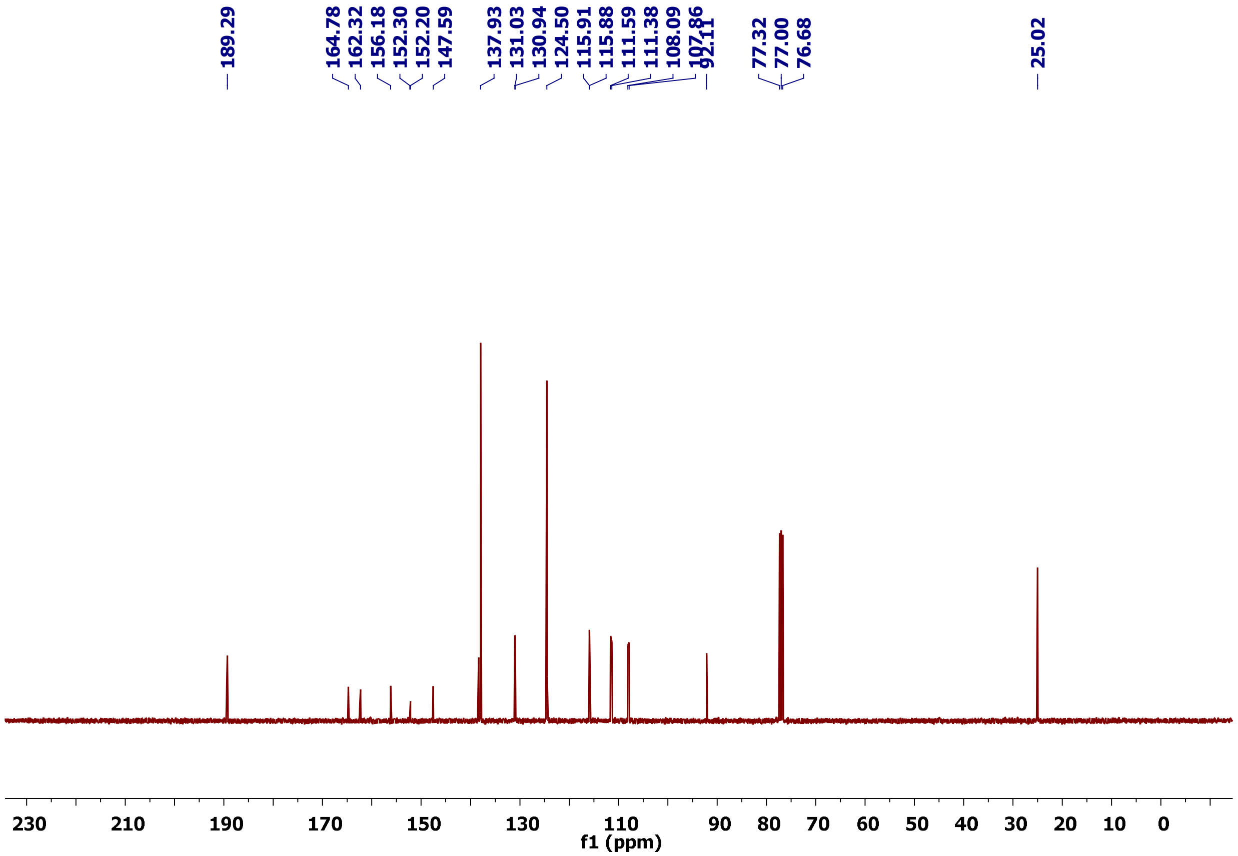

^{13}C CRAPT NMR ($CDCl_3$) spectrum of (Z)-1-(5-((3-fluorophenyl)imino)-4-(4-iodophenyl)-4,5-dihydro-1,3,4-thiadiazol-2-yl)ethan-1-one

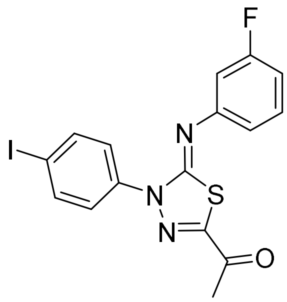

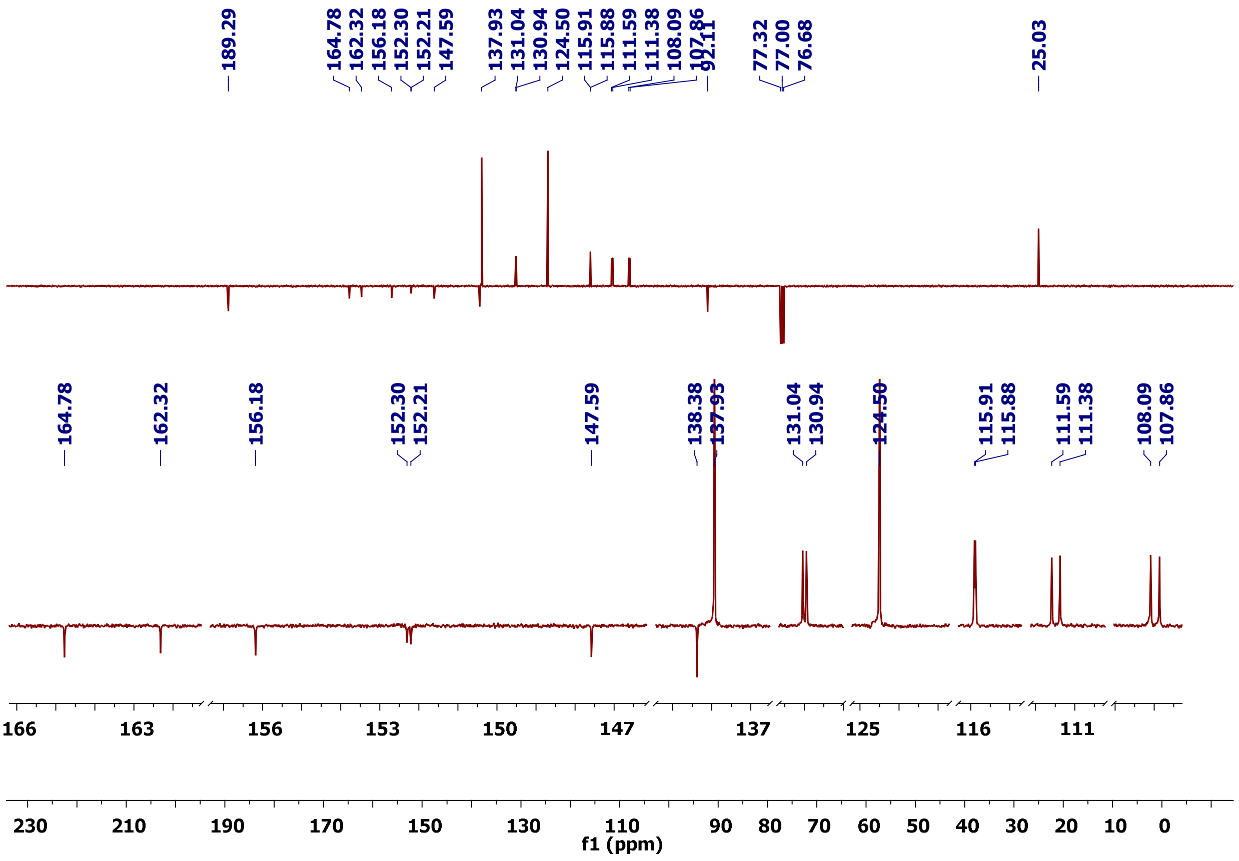

^{1}H-^{1}H gDQCOSY NMR ($CDCl_3$) spectrum of (Z)-1-(5-((3-fluorophenyl)imino)-4-(4-iodophenyl)-4,5-dihydro-1,3,4-thiadiazol-2-yl)ethan-1-one

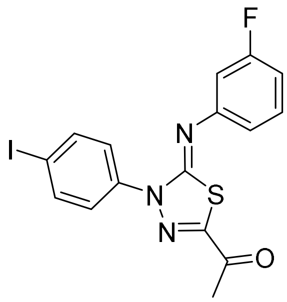

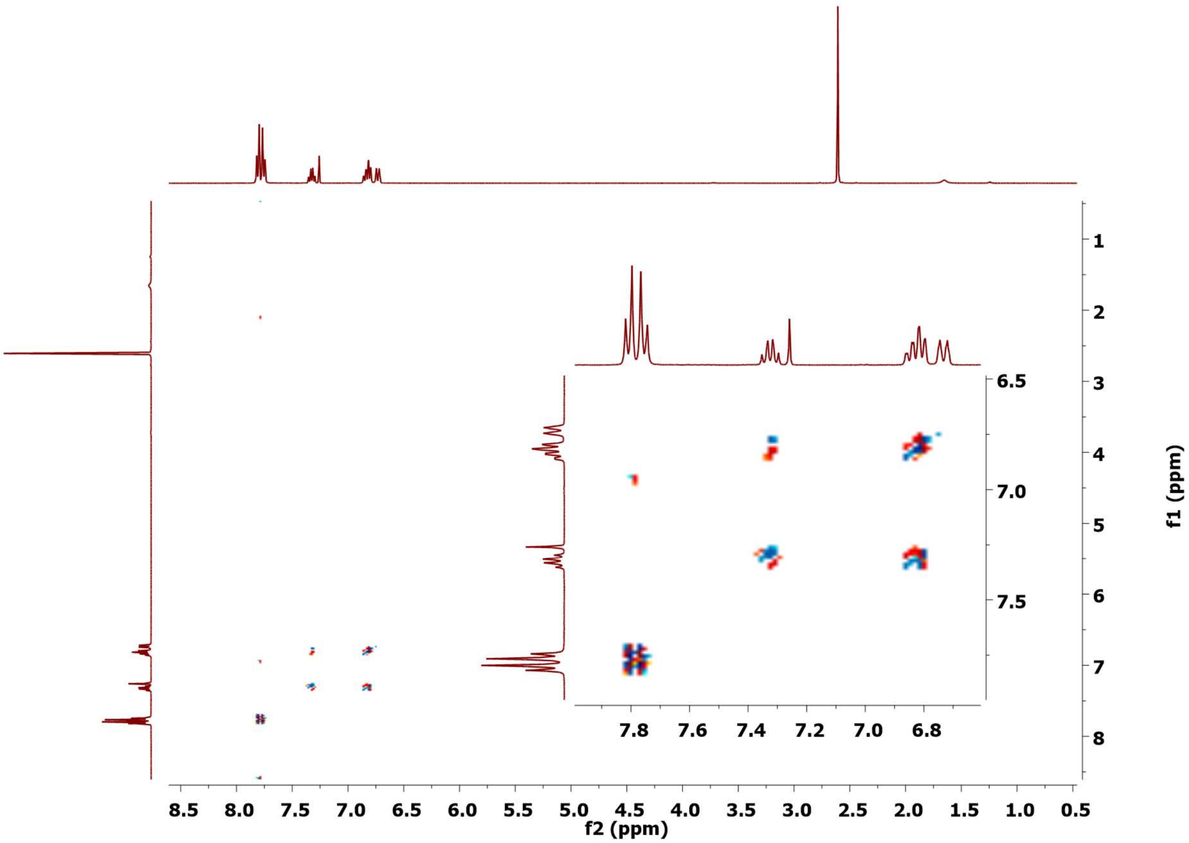

^{1}H-^{13}C-HSQC NMR ($CDCl_3$) spectrum of (Z)-1-(5-((3-fluorophenyl)imino)-4-(4-iodophenyl)-4,5-dihydro-1,3,4-thiadiazol-2-yl)ethan-1-one

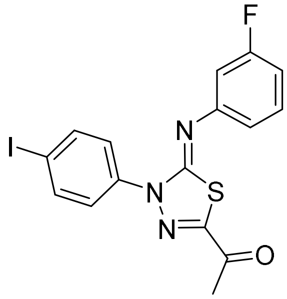

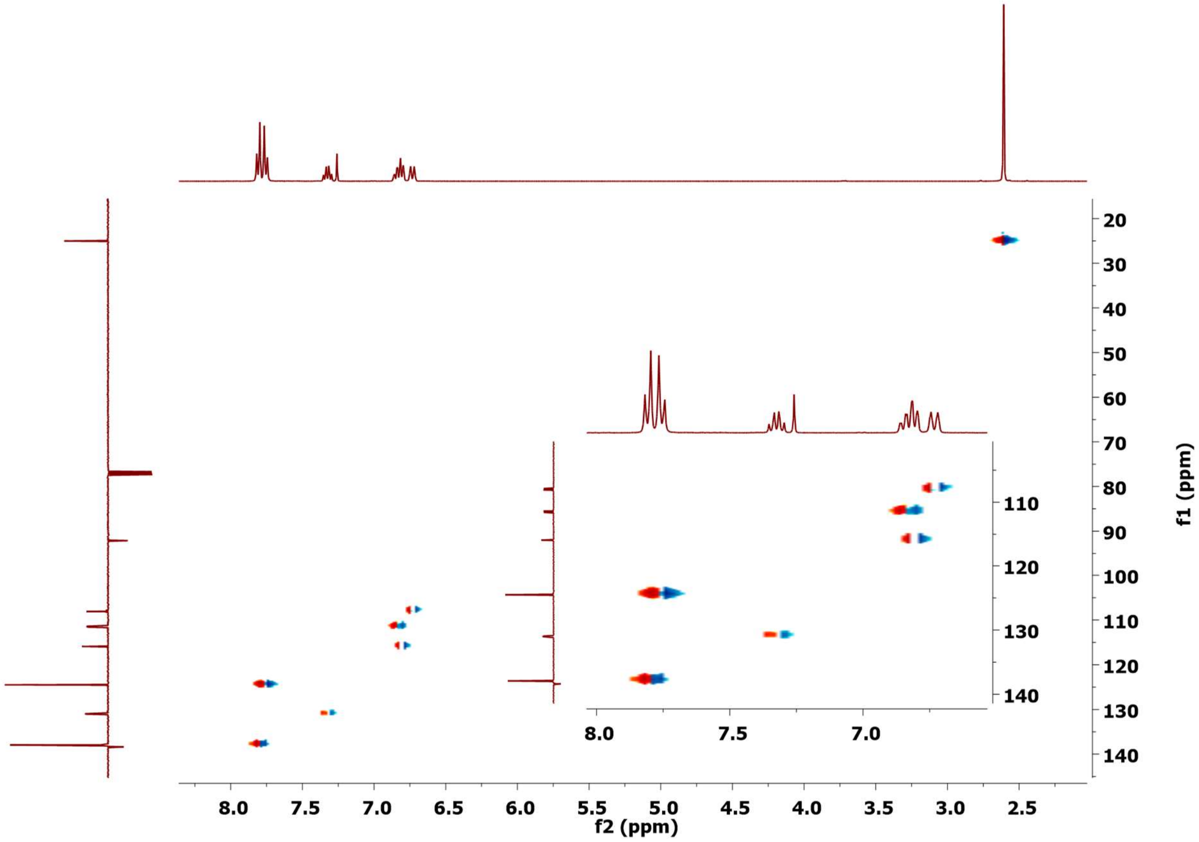

^1H-^{13}C-gHMBC NMR ($CDCl_3$) spectrum of (Z)-1-(5-((3-fluorophenyl)imino)-4-(4-iodophenyl)-4,5-dihydro-1,3,4-thiadiazol-2-yl)ethan-1-one

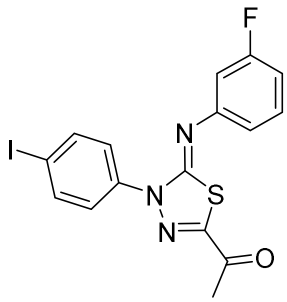

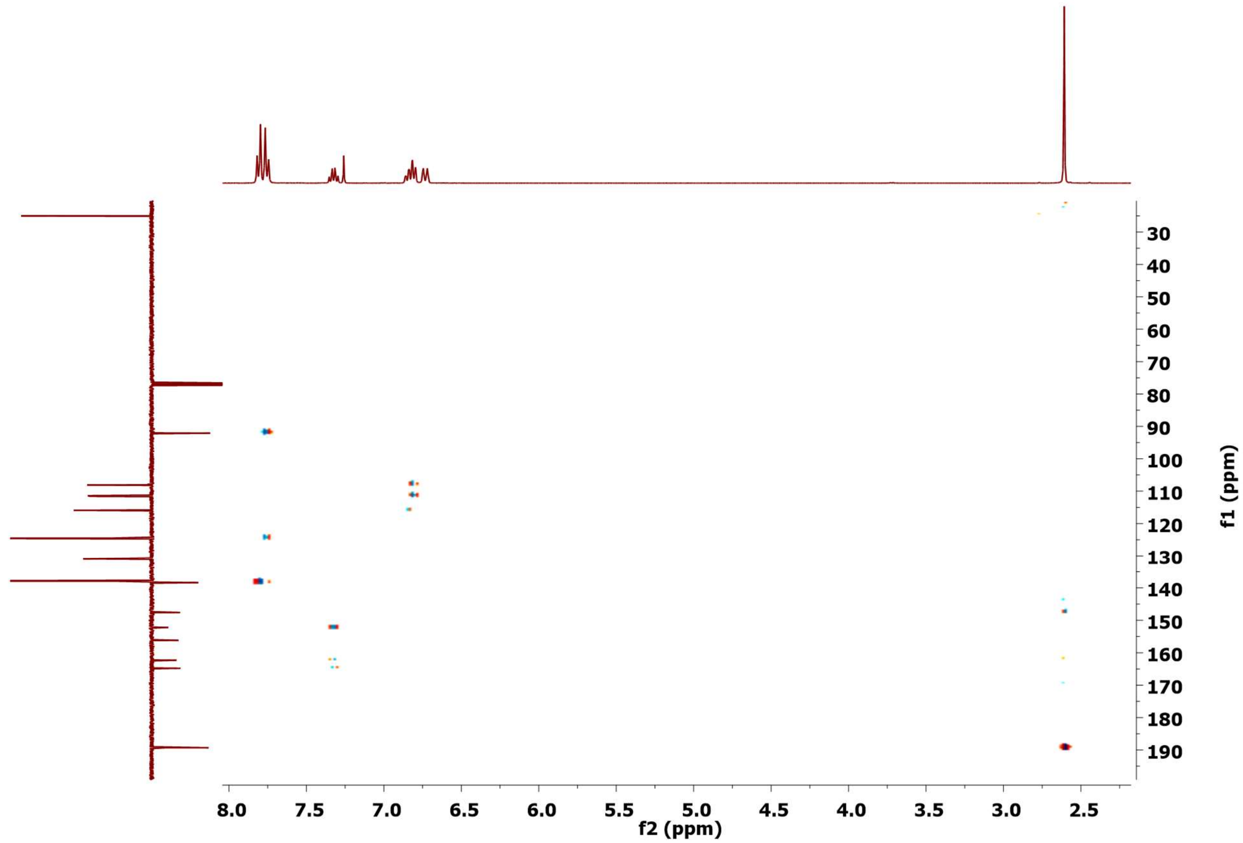

^{1}H NMR ($CDCl_3$) spectrum of (Z)-1-(5-((3-fluorophenyl)imino)-4-(p-tolyl)-4,5-dihydro-1,3,4-thiadiazol-2-yl)ethan-1-one

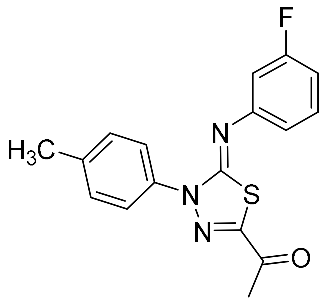

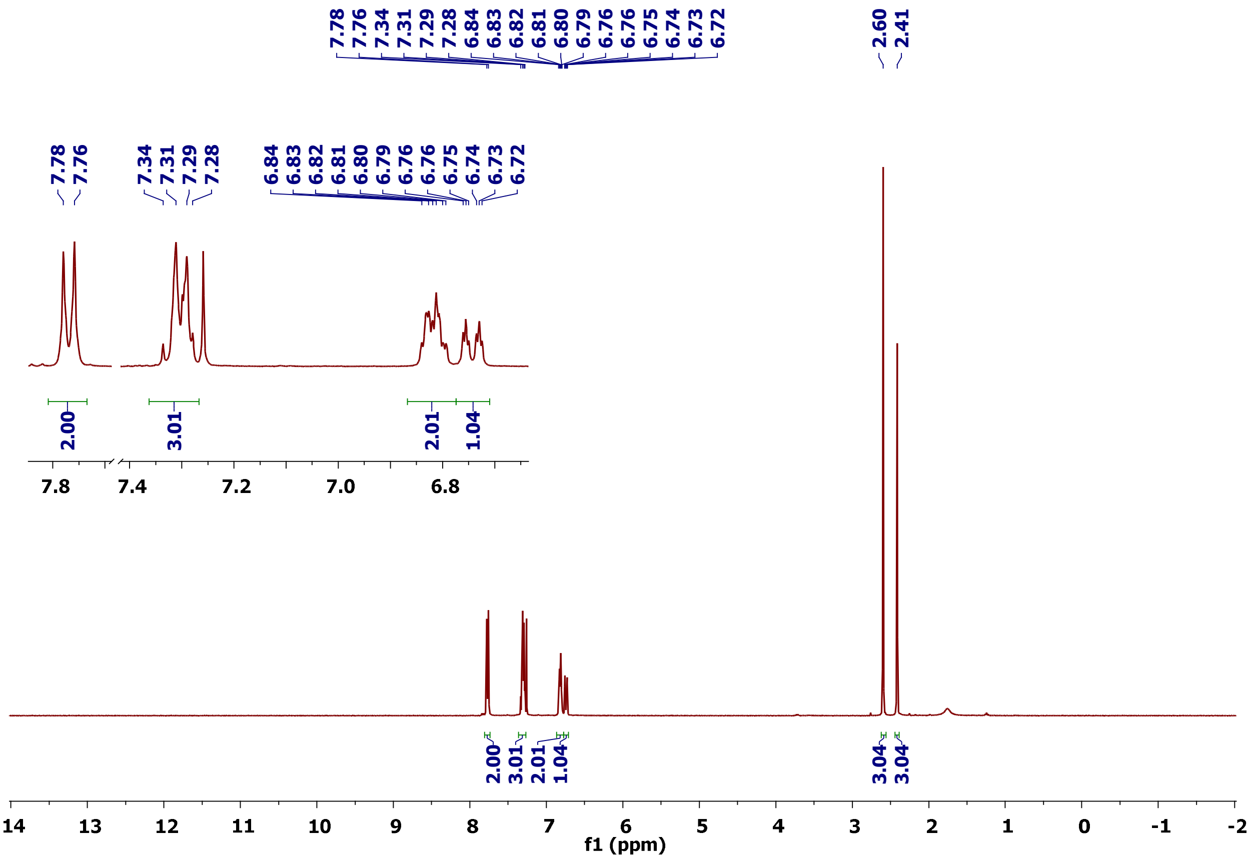

^{13}C NMR ($CDCl_3$) spectrum of (Z)-1-(5-((3-fluorophenyl)imino)-4-(p-tolyl)-4,5-dihydro-1,3,4-thiadiazol-2-yl)ethan-1-one

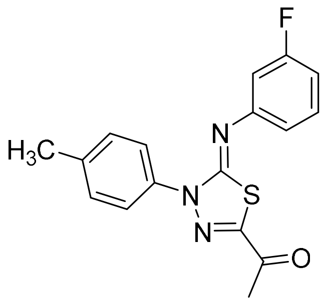

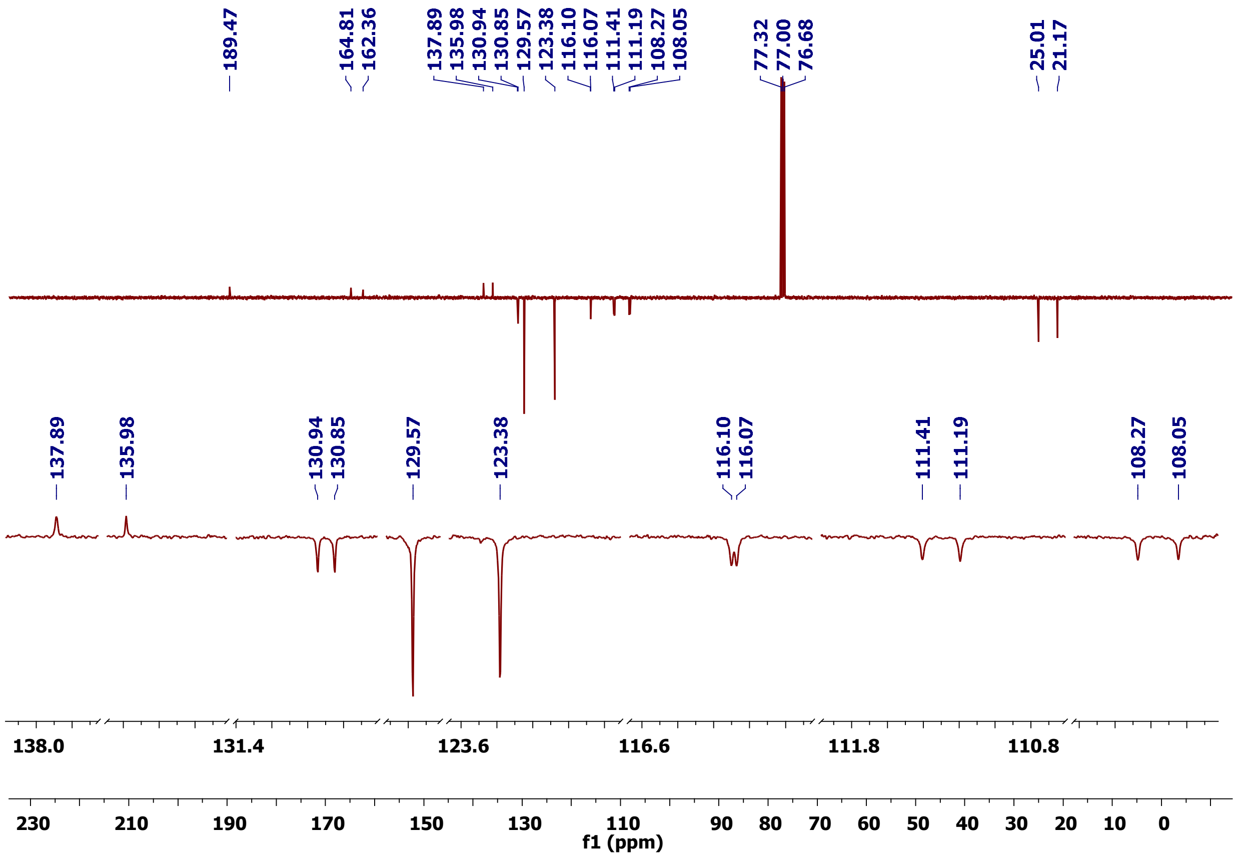

^{13}C CRAPT NMR ($CDCl_3$) spectrum of (Z)-1-(5-((3-fluorophenyl)imino)-4-(p-tolyl)-4,5-dihydro-1,3,4-thiadiazol-2-yl)ethan-1-one

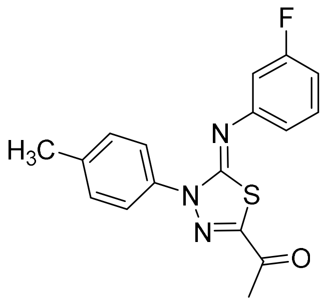

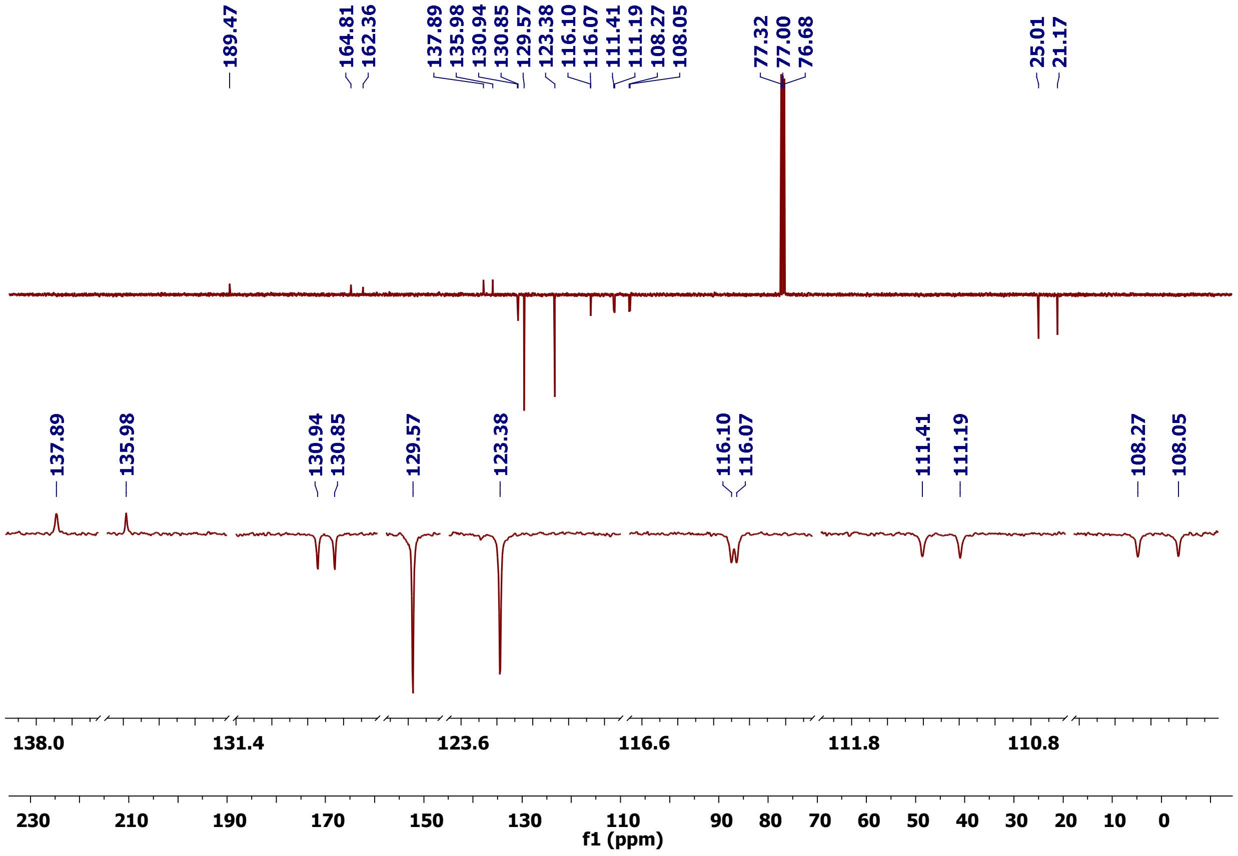

^{1}H-^{1}H gDQCOSY NMR ($CDCl_3$) spectrum of (Z)-1-(5-((3-fluorophenyl)imino)-4-(p-tolyl)-4,5-dihydro-1,3,4-thiadiazol-2-yl)ethan-1-one

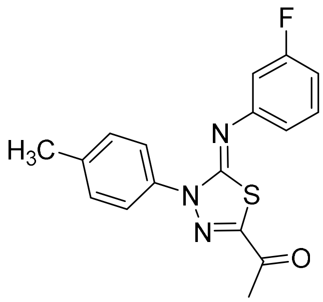

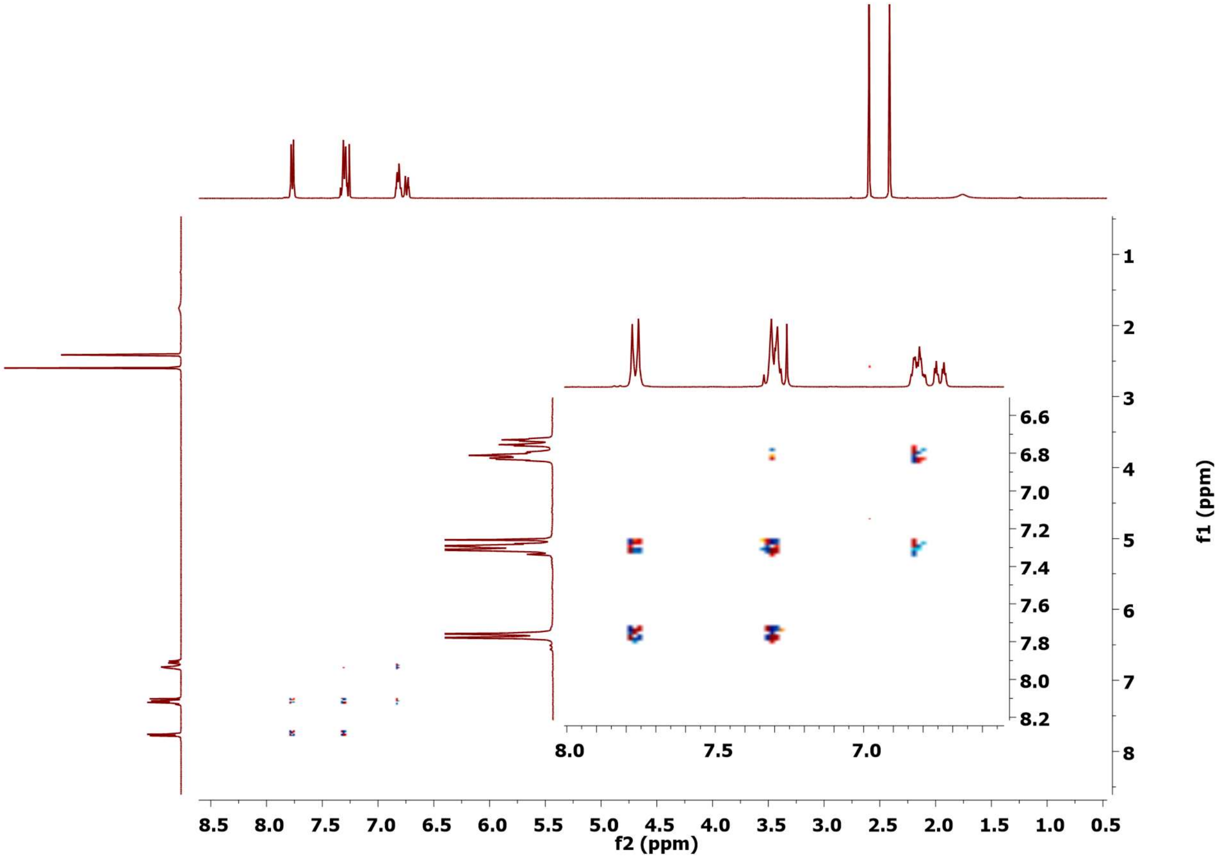

^{1}H NMR ($CDCl_3$) spectrum of (Z)-1-(4-(4-bromophenyl)-5-((2-fluorophenyl)imino)-4,5-dihydro-1,3,4-thiadiazol-2-yl)ethan-1-one

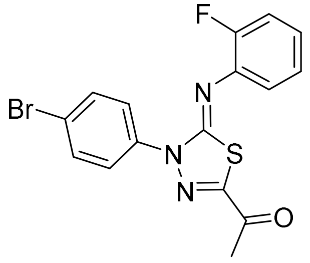

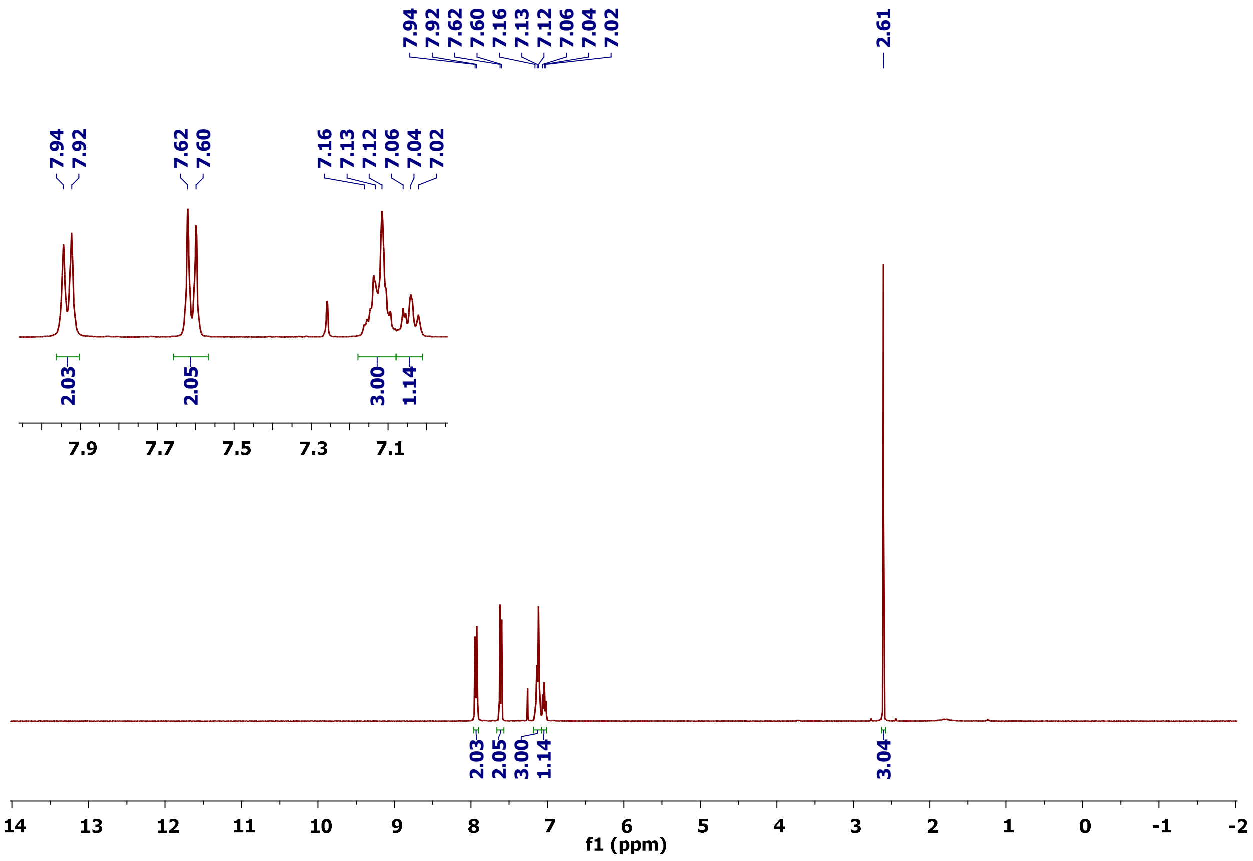

^{13}C NMR ($CDCl_3$) spectrum of (Z)-1-(4-(4-bromophenyl)-5-((2-fluorophenyl)imino)-4,5-dihydro-1,3,4-thiadiazol-2-yl)ethan-1-one

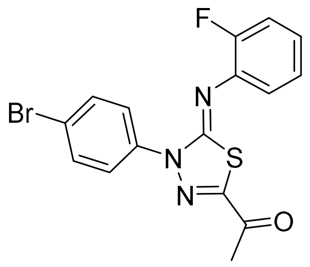

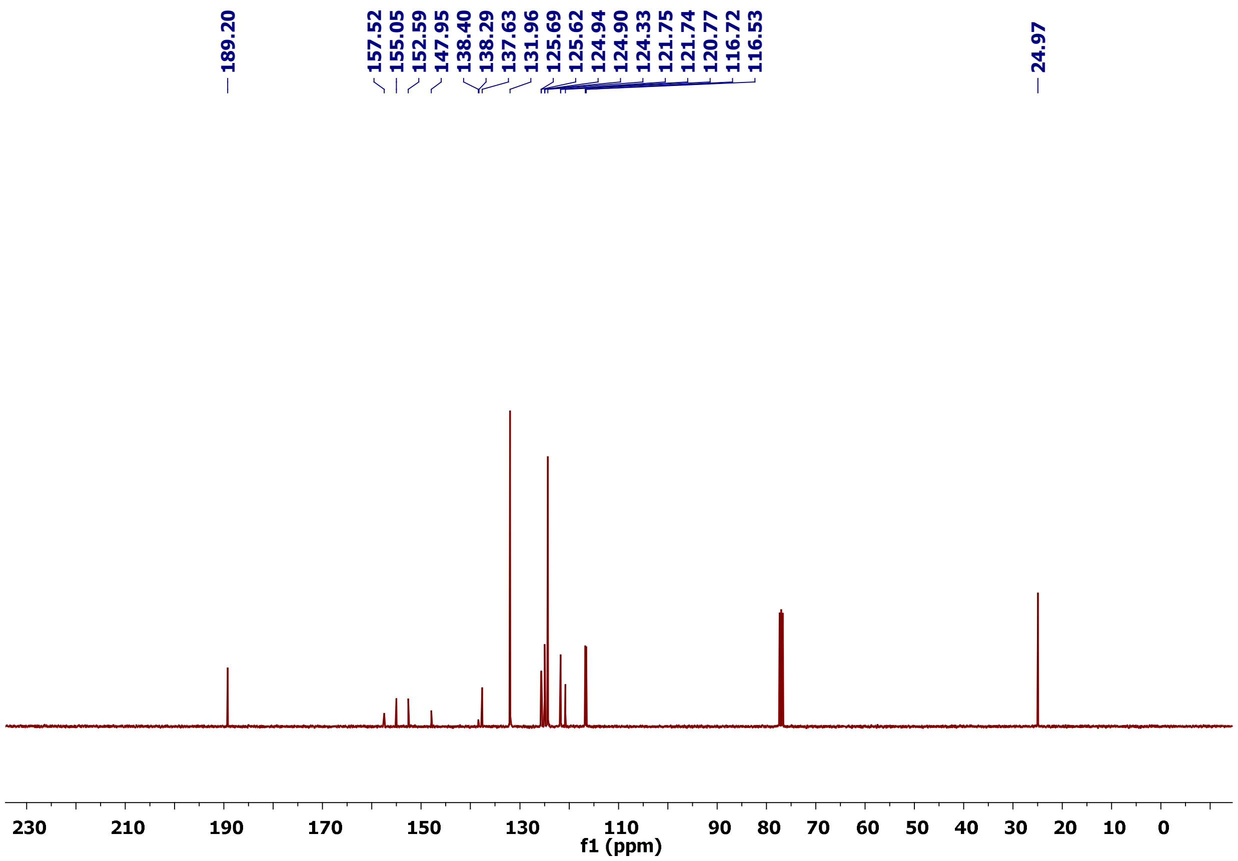

^{13}C CRAPT NMR ($CDCl_3$) spectrum of (Z)-1-(4-(4-bromophenyl)-5-((2-fluorophenyl)imino)-4,5-dihydro-1,3,4-thiadiazol-2-yl)ethan-1-one

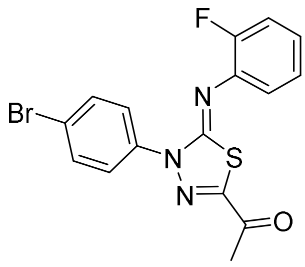

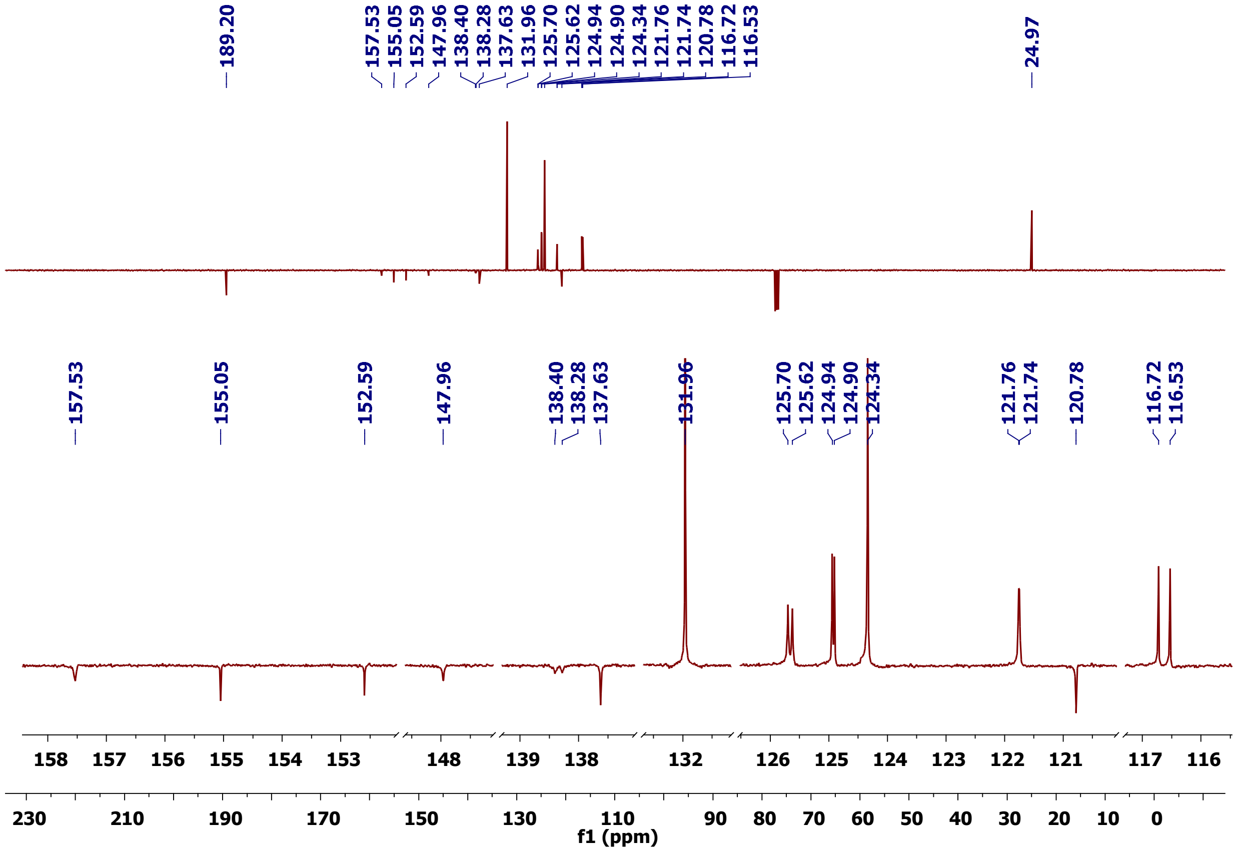

^1H-^1H gDQCOSY NMR ($CDCl_3$) spectrum of (Z)-1-(4-(4-bromophenyl)-5-((2-fluorophenyl)imino)-4,5-dihydro-1,3,4-thiadiazol-2-yl)ethan-1-one

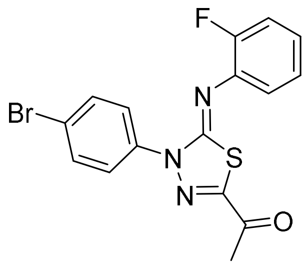

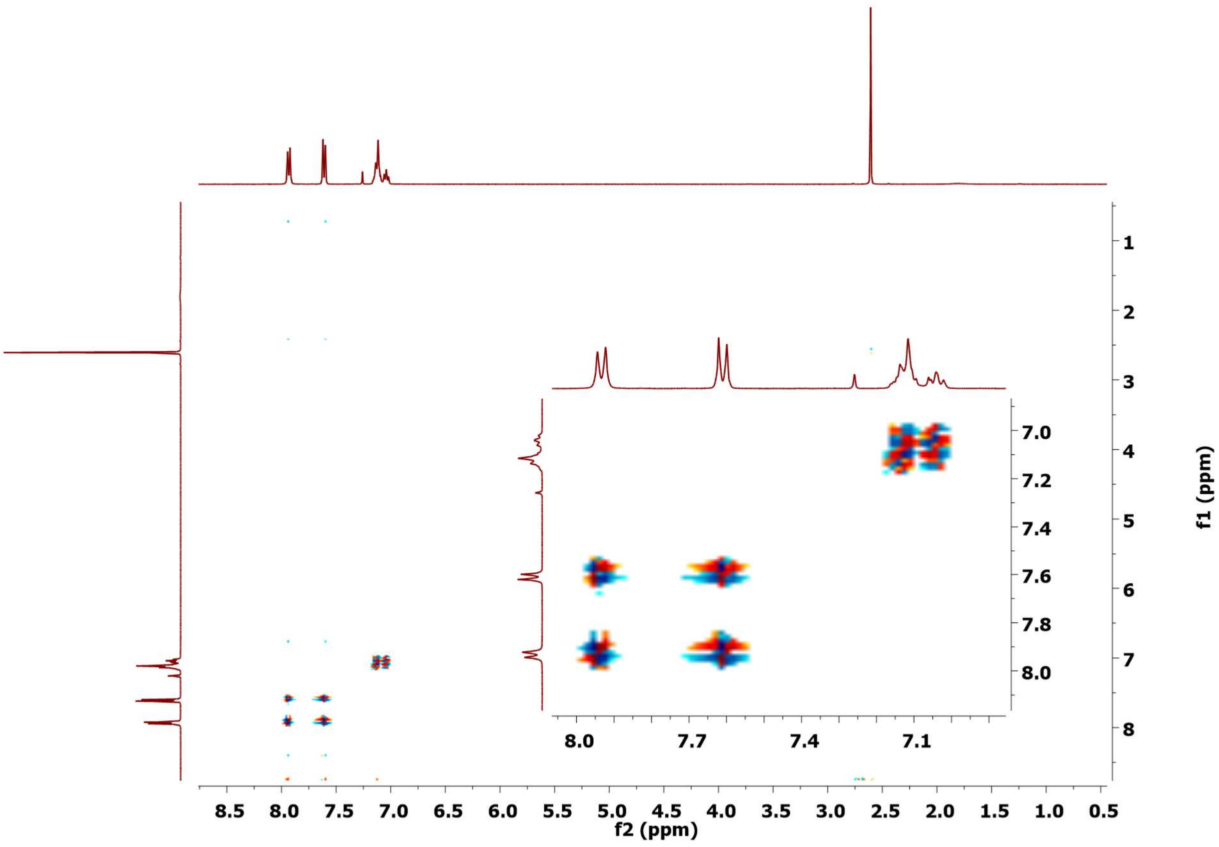

^{1}H-^{13}C-HSQC NMR ($CDCl_3$) spectrum of (Z)-1-(4-(4-bromophenyl)-5-((2-fluorophenyl)imino)-4,5-dihydro-1,3,4-thiadiazol-2-yl)ethan-1-one

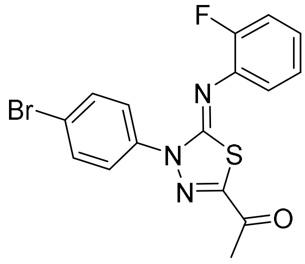

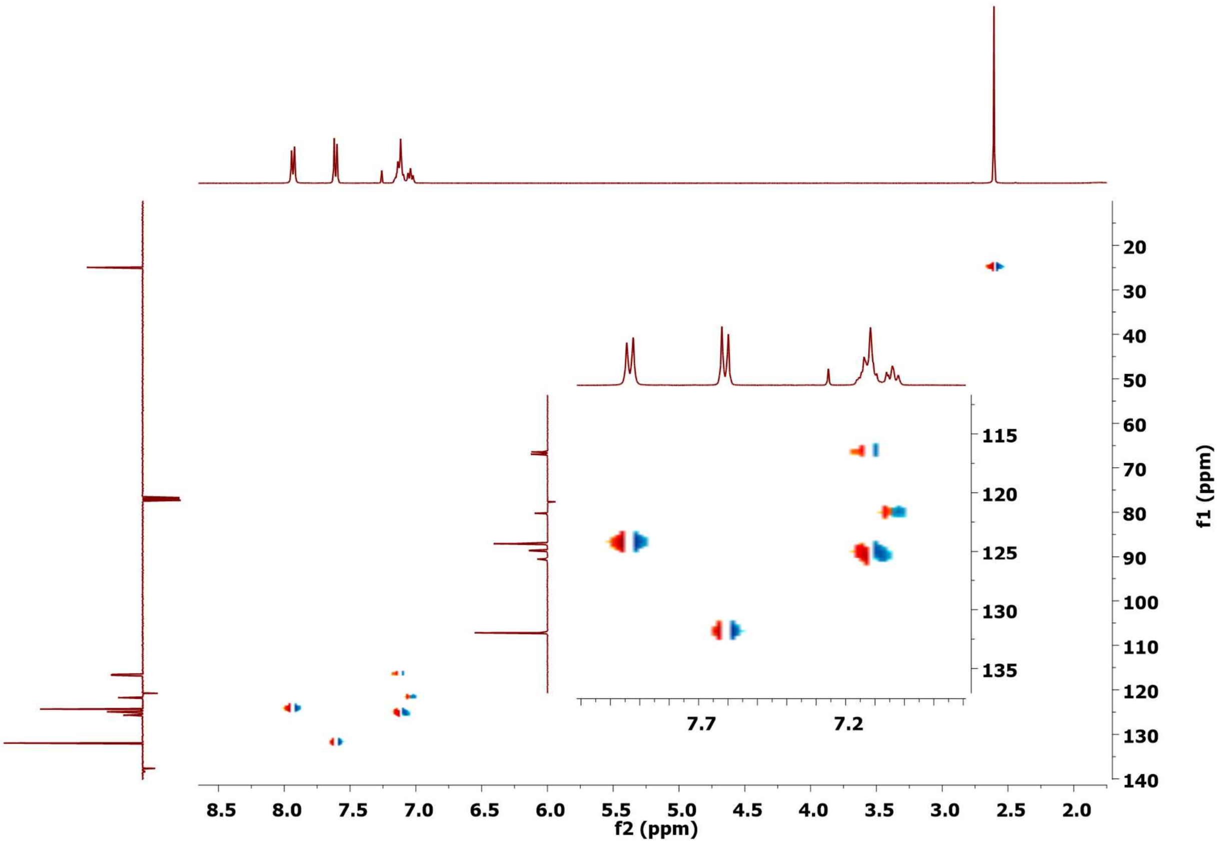

^{1}H-^{13}C-gHMBC NMR ($CDCl_3$) spectrum of (Z)-1-(4-(4-bromophenyl)-5-((2-fluorophenyl)imino)-4,5-dihydro-1,3,4-thiadiazol-2-yl)ethan-1-one

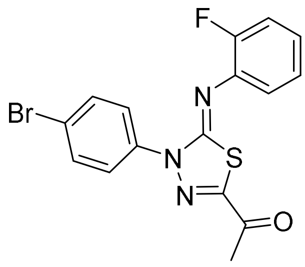

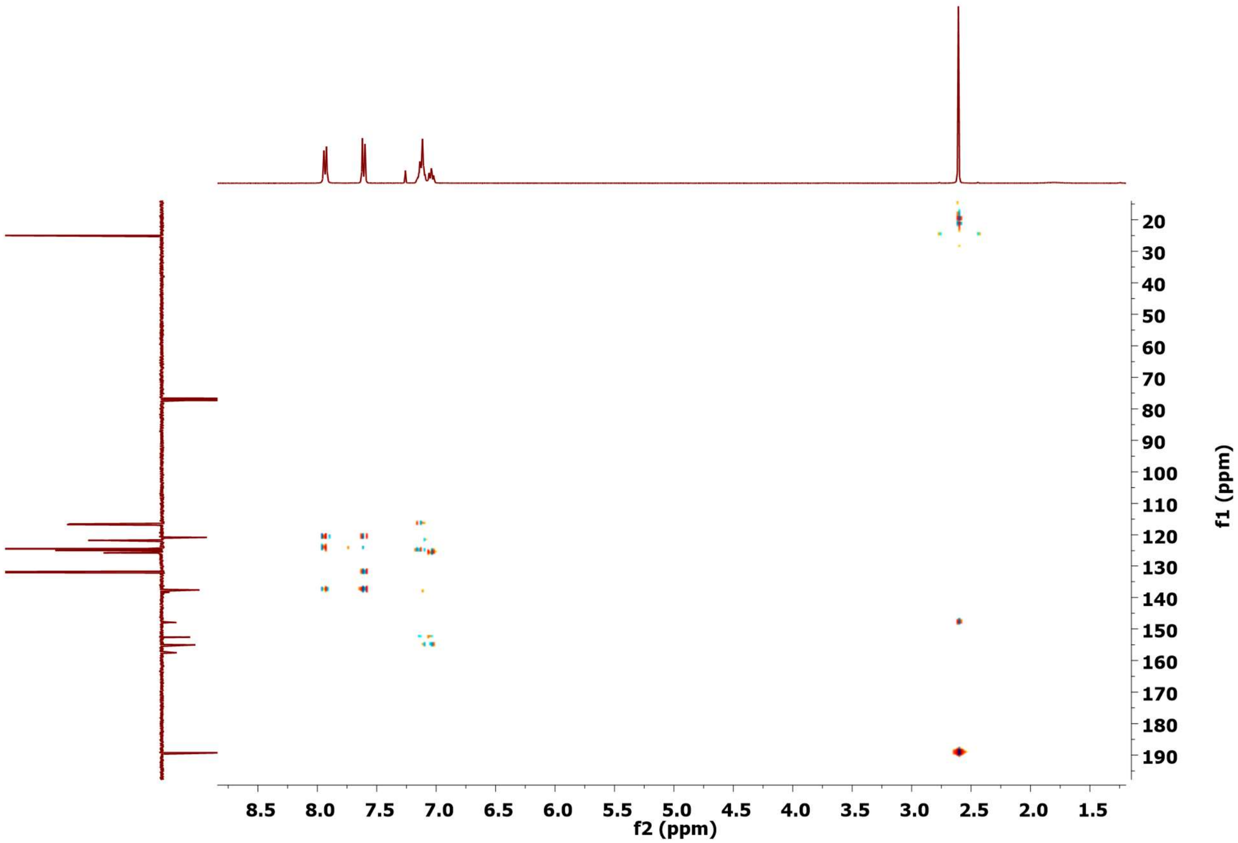

^{1}H NMR ($CDCl_3$) spectrum of (Z)-1-(4-(4-chlorophenyl)-5-((2-fluorophenyl)imino)-4,5-dihydro-1,3,4-thiadiazol-2-yl)ethan-1-one

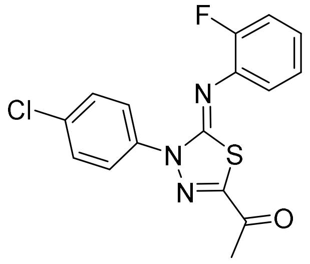

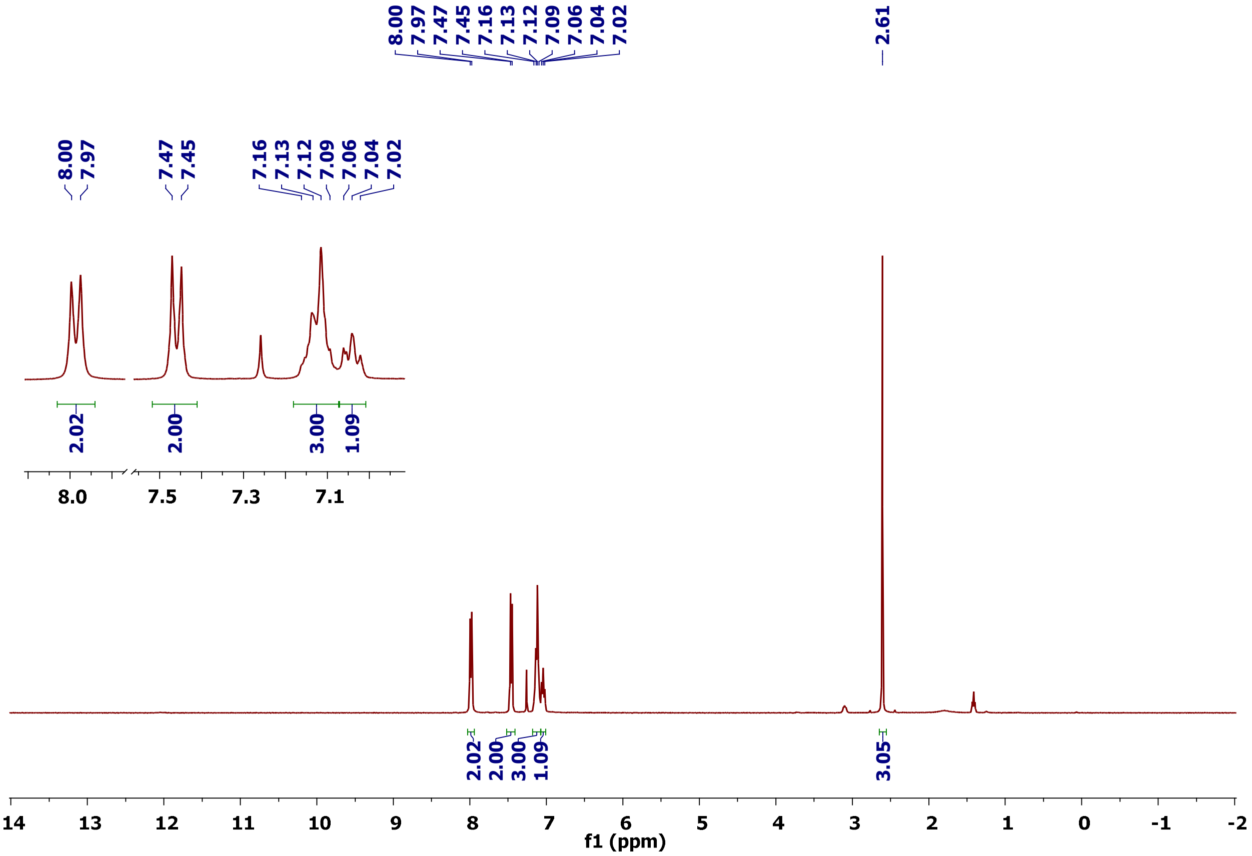

^{13}C NMR ($CDCl_3$) spectrum of (Z)-1-(4-(4-chlorophenyl)-5-((2-fluorophenyl)imino)-4,5-dihydro-1,3,4-thiadiazol-2-yl)ethan-1-one

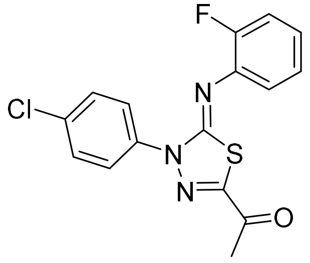

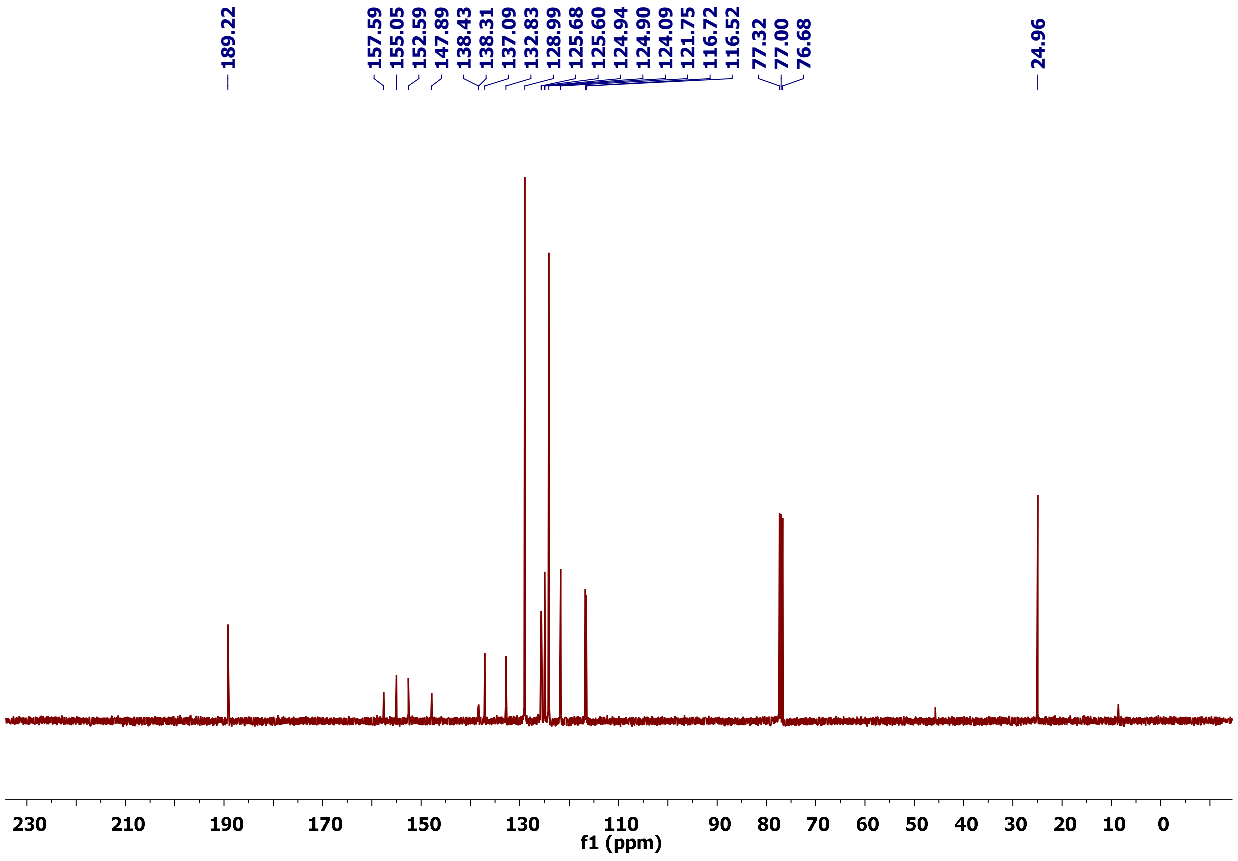

^{13}C CRAPT NMR ($CDCl_3$) spectrum of (Z)-1-(4-(4-chlorophenyl)-5-((2-fluorophenyl)imino)-4,5-dihydro-1,3,4-thiadiazol-2-yl)ethan-1-one

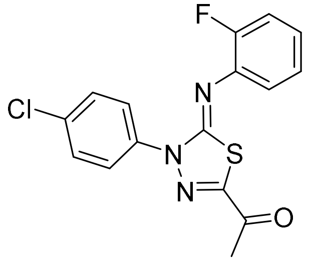

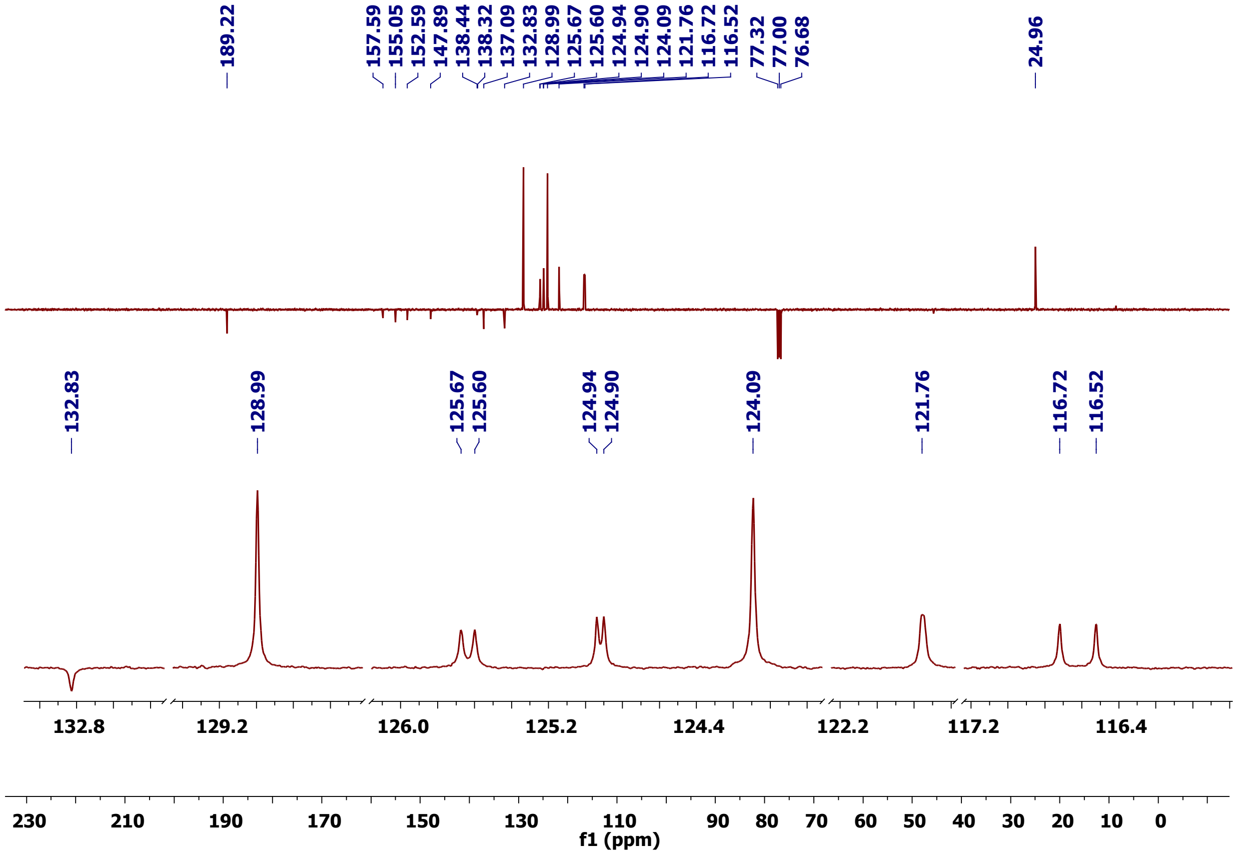

^1H-^1H gDQCOSY NMR ($CDCl_3$) spectrum of (Z)-1-(4-(4-chlorophenyl)-5-((2-fluorophenyl)imino)-4,5-dihydro-1,3,4-thiadiazol-2-yl)ethan-1-one

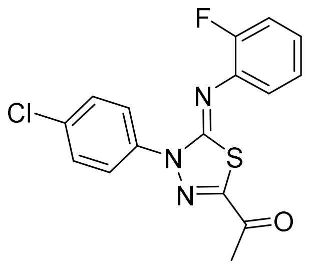

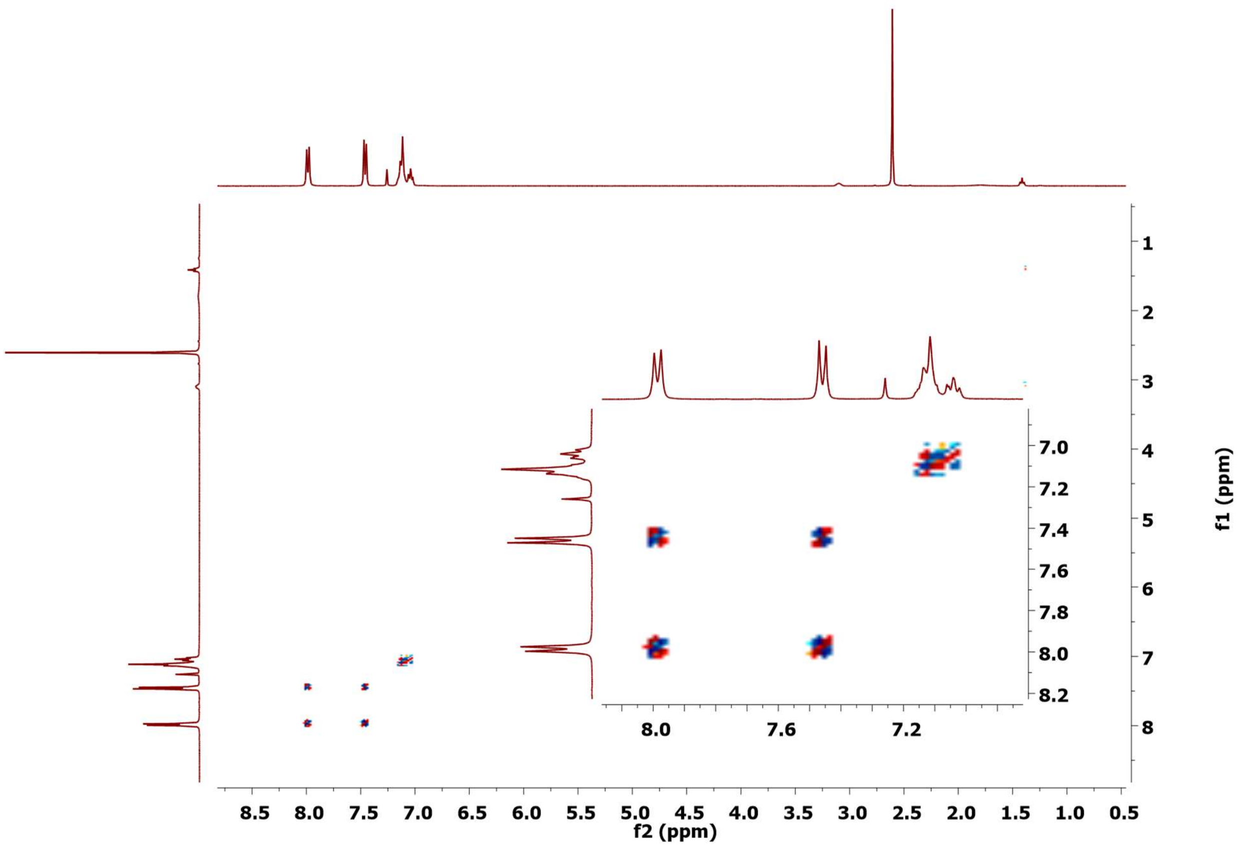

^{1}H-^{13}C-HSQC NMR ($CDCl_3$) spectrum of (Z)-1-(4-(4-chlorophenyl)-5-((2-fluorophenyl)imino)-4,5-dihydro-1,3,4-thiadiazol-2-yl)ethan-1-one

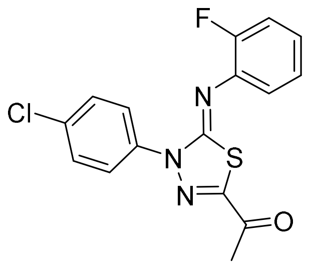

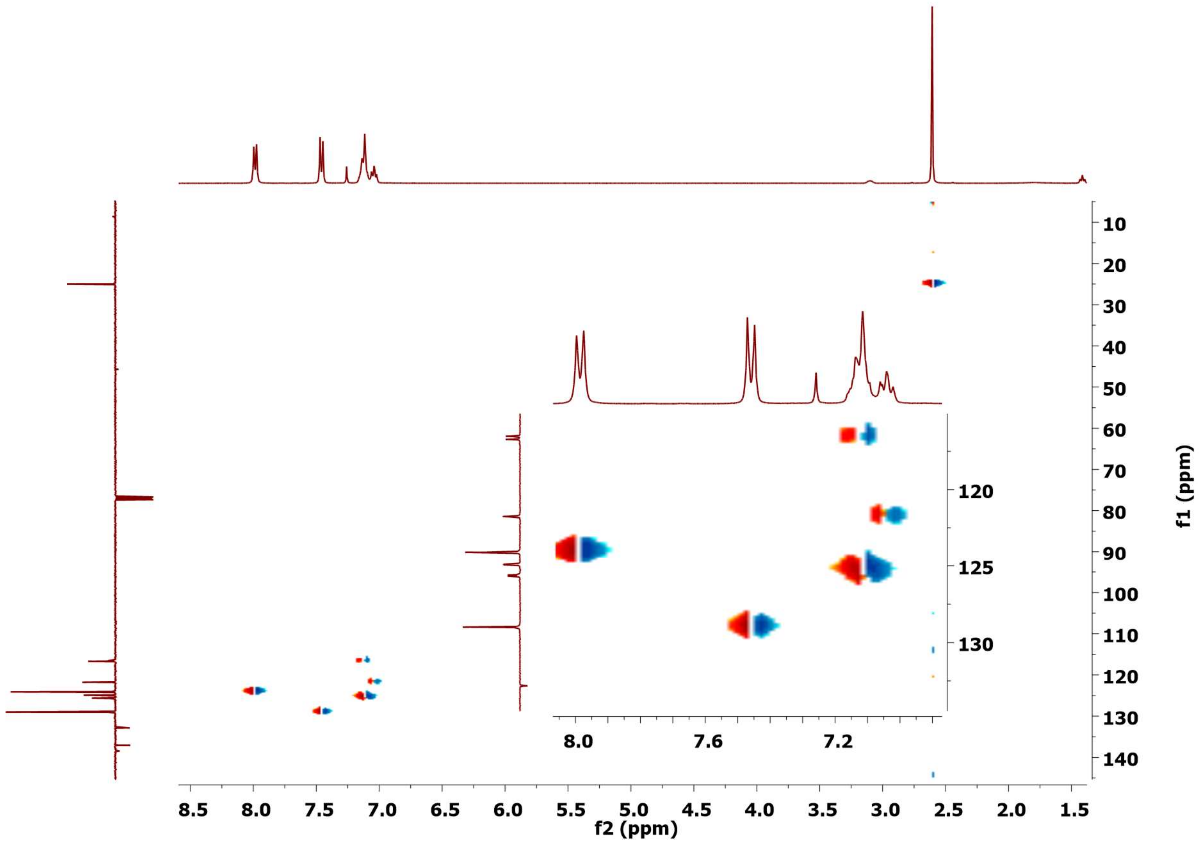

^{1}H-^{13}C-gHMBC NMR ($CDCl_3$) spectrum of (Z)-1-(4-(4-chlorophenyl)-5-((2-fluorophenyl)imino)-4,5-dihydro-1,3,4-thiadiazol-2-yl)ethan-1-one

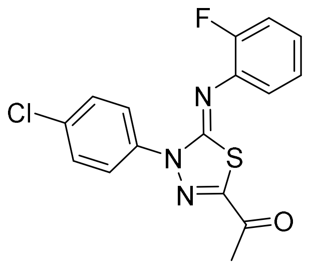

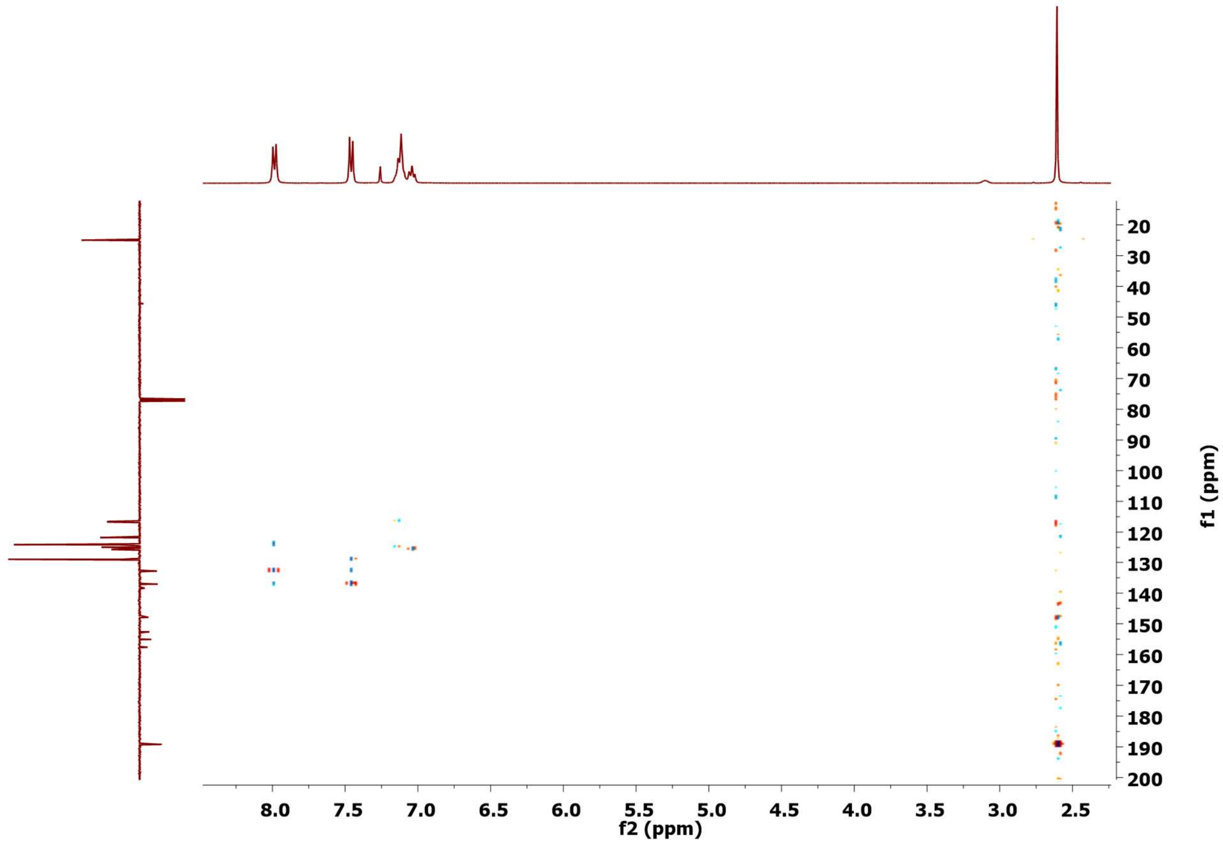

^{1}H NMR ($CDCl_3$) spectrum of (Z)-1-(5-((2-fluorophenyl)imino)-4-(4-iodophenyl)-4,5-dihydro-1,3,4-thiadiazol-2-yl)ethan-1-one

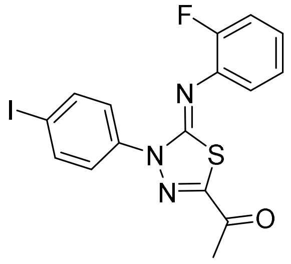

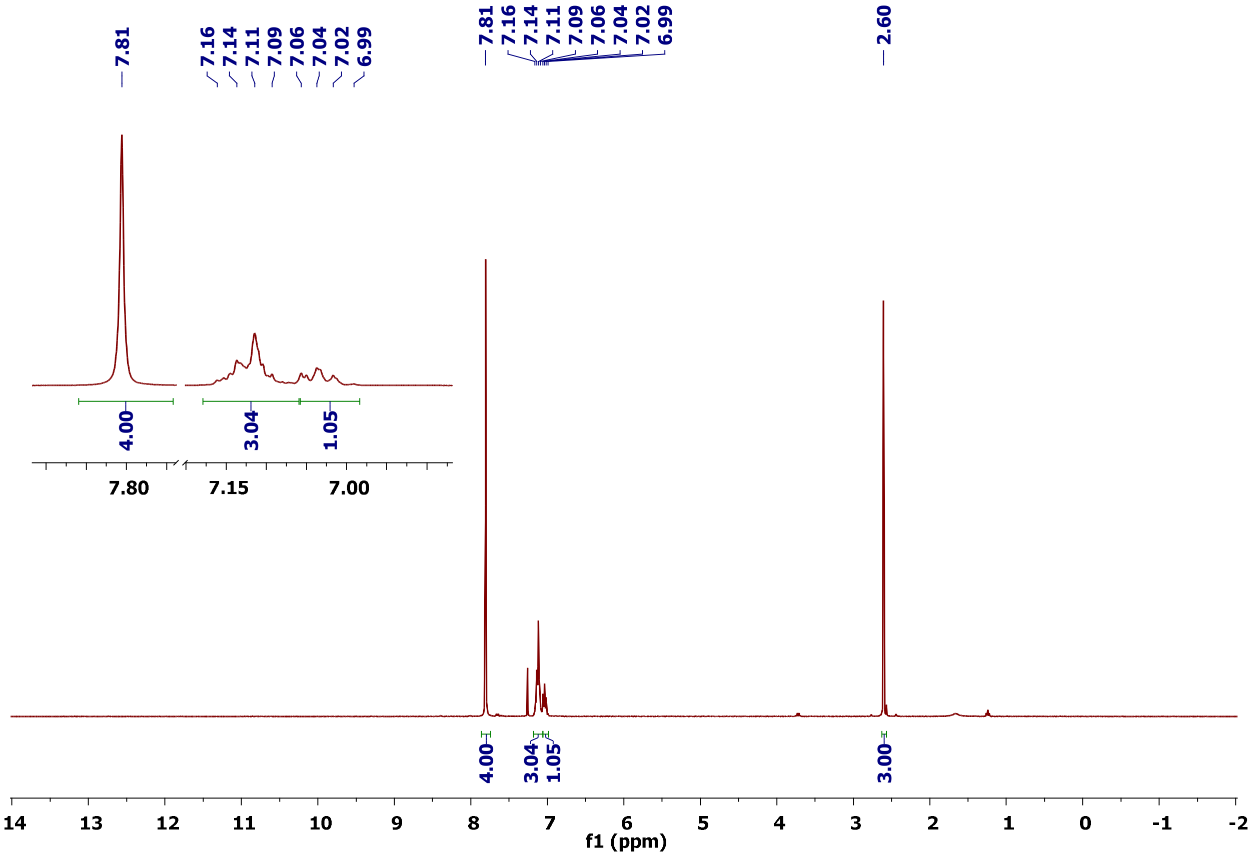

^{13}C NMR ($CDCl_3$) spectrum of (Z)-1-(5-((2-fluorophenyl)imino)-4-(4-iodophenyl)-4,5-dihydro-1,3,4-thiadiazol-2-yl)ethan-1-one

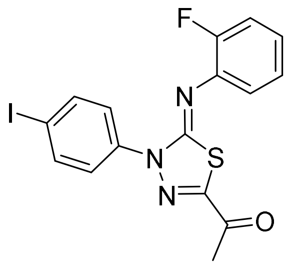

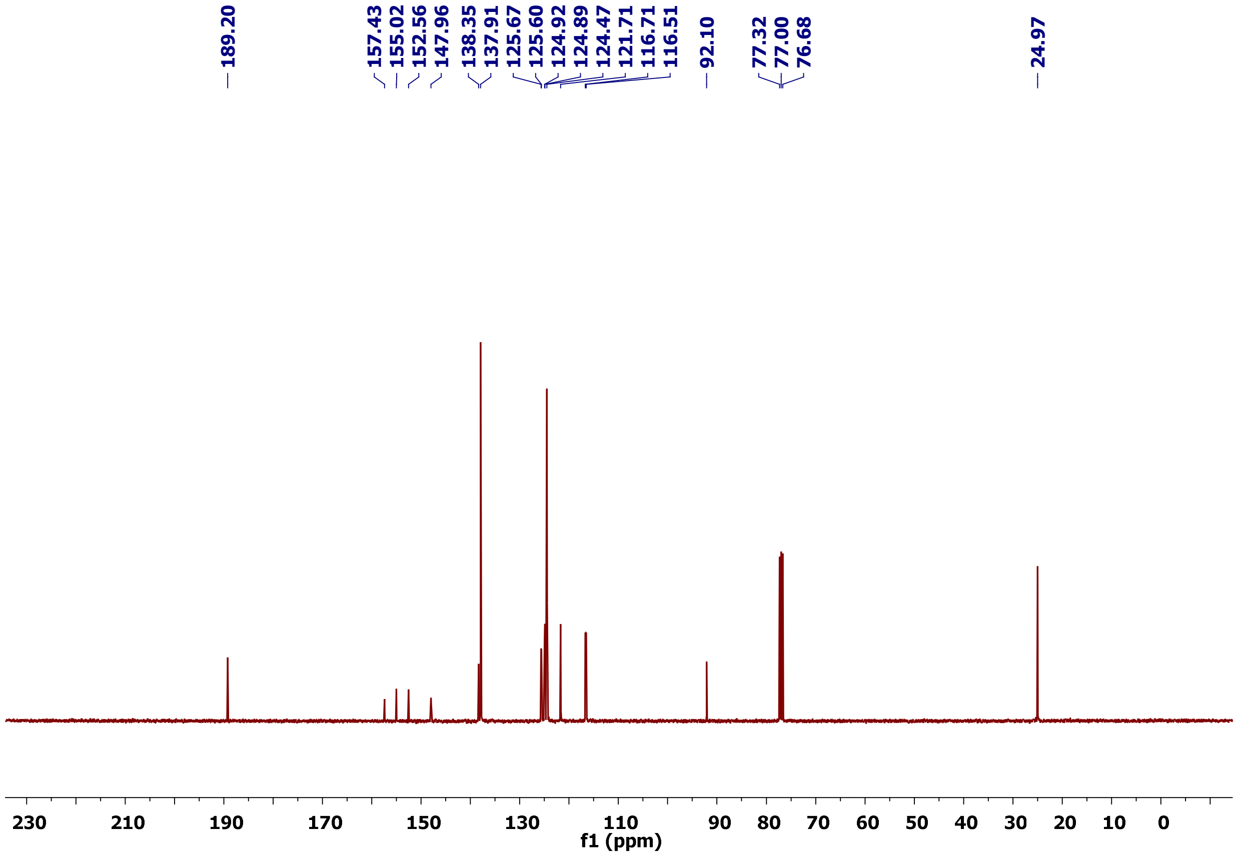

^{13}C CRAPT NMR ($CDCl_3$) spectrum of (Z)-1-(5-((2-fluorophenyl)imino)-4-(4-iodophenyl)-4,5-dihydro-1,3,4-thiadiazol-2-yl)ethan-1-one

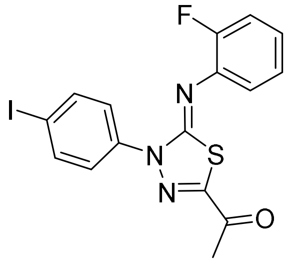

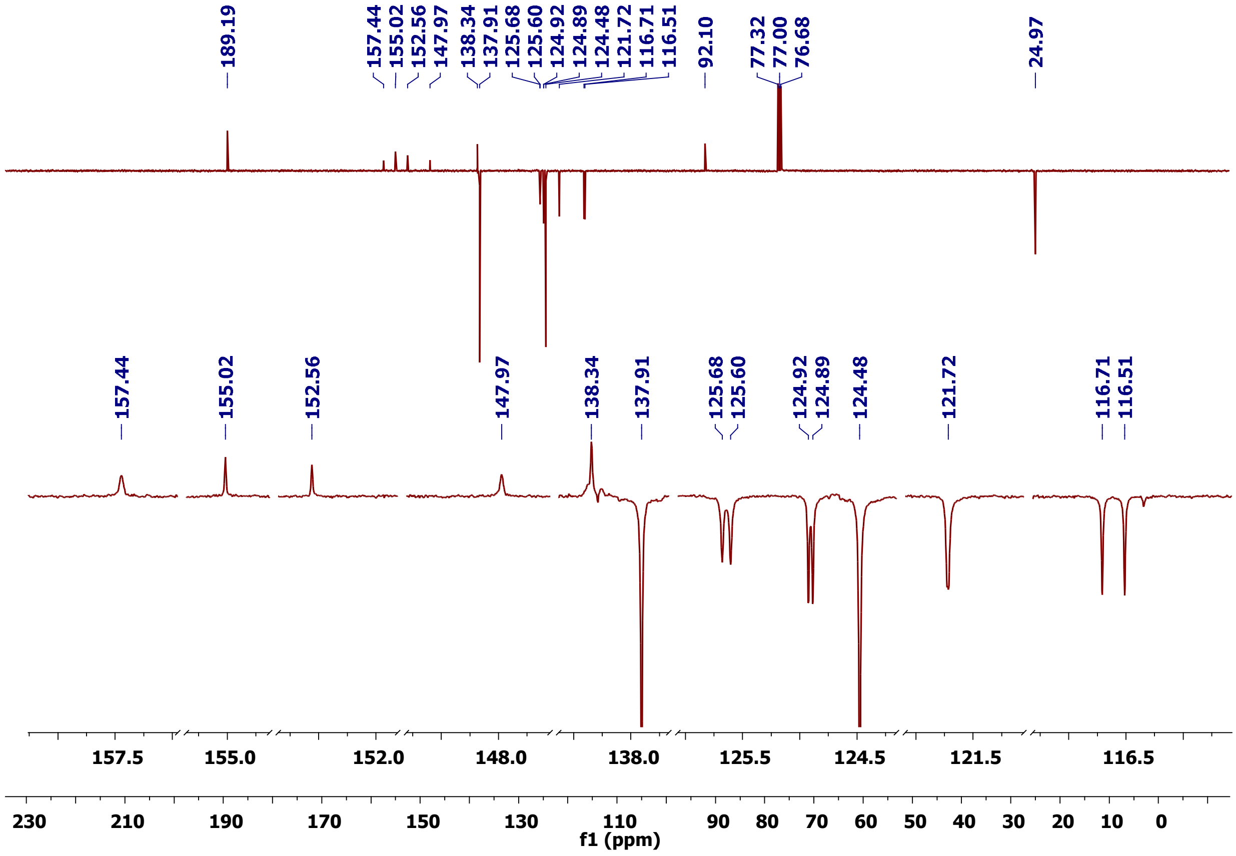

^1H-^1H gDQCOSY NMR ($CDCl_3$) spectrum of (Z)-1-(5-((2-fluorophenyl)imino)-4-(4-iodophenyl)-4,5-dihydro-1,3,4-thiadiazol-2-yl)ethan-1-one

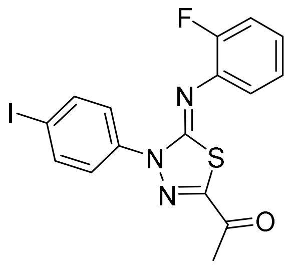

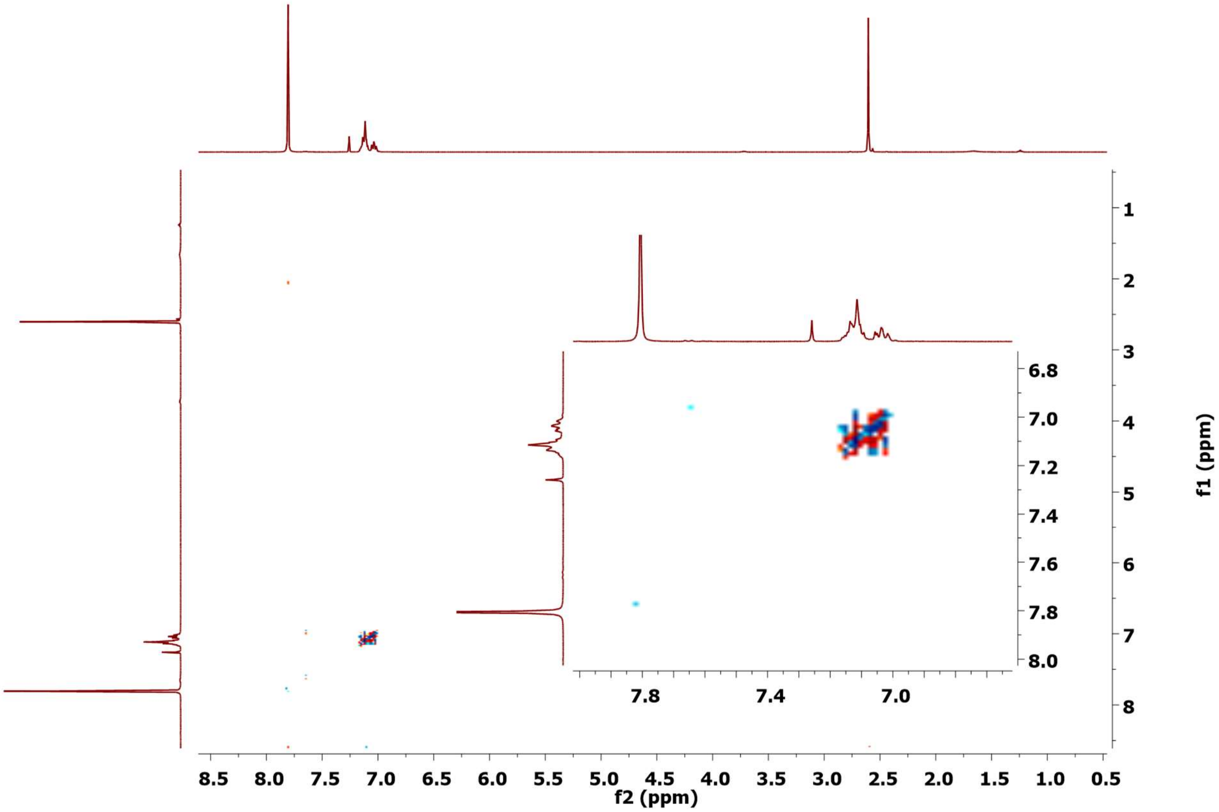

^{1}H-^{13}C-HSQC NMR ($CDCl_3$) spectrum of (Z)-1-(5-((2-fluorophenyl)imino)-4-(4-iodophenyl)-4,5-dihydro-1,3,4-thiadiazol-2-yl)ethan-1-one

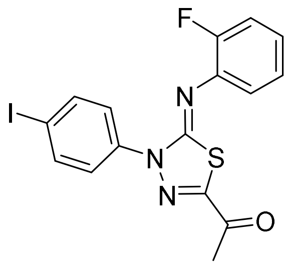

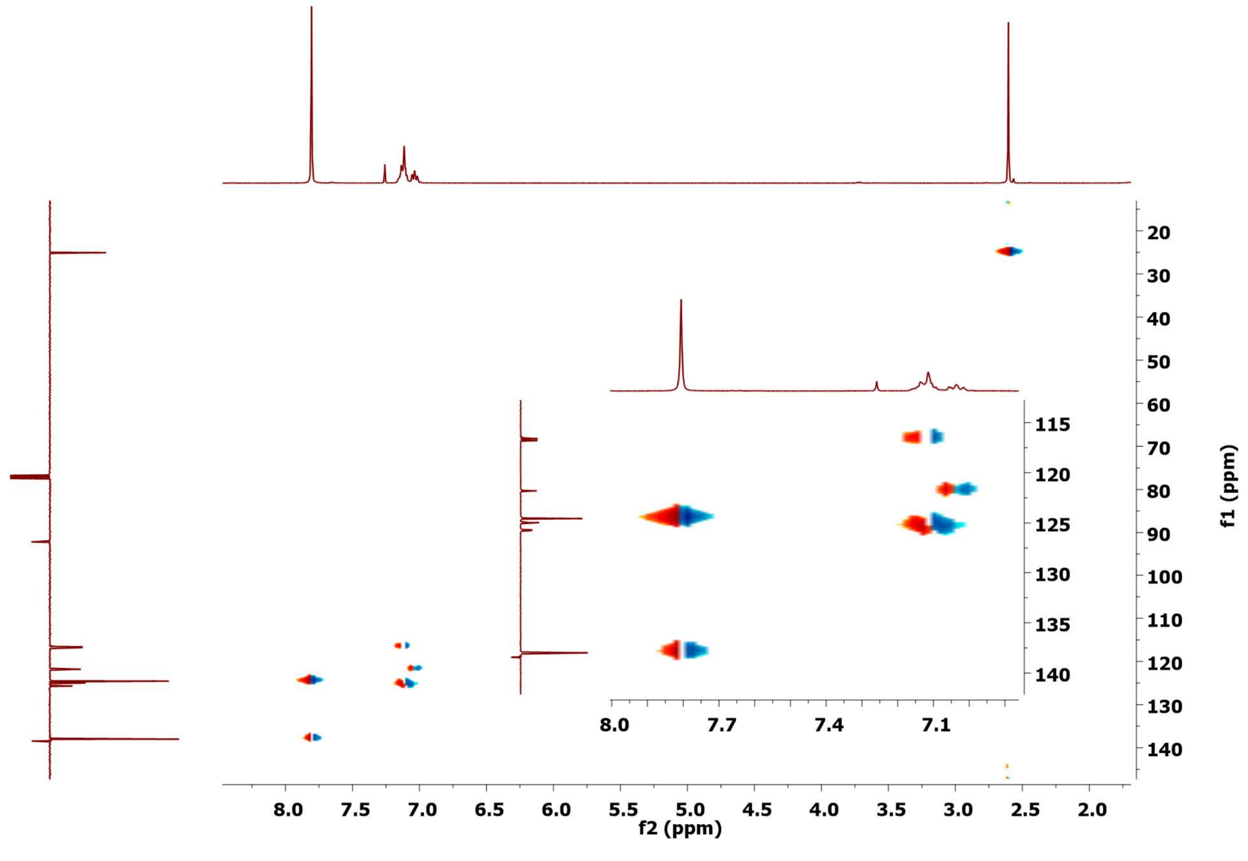

^{1}H-^{13}C-gHMBC NMR ($CDCl_3$) spectrum of (Z)-1-(5-((2-fluorophenyl)imino)-4-(4-iodophenyl)-4,5-dihydro-1,3,4-thiadiazol-2-yl)ethan-1-one

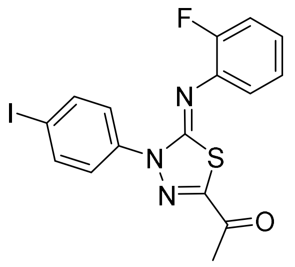

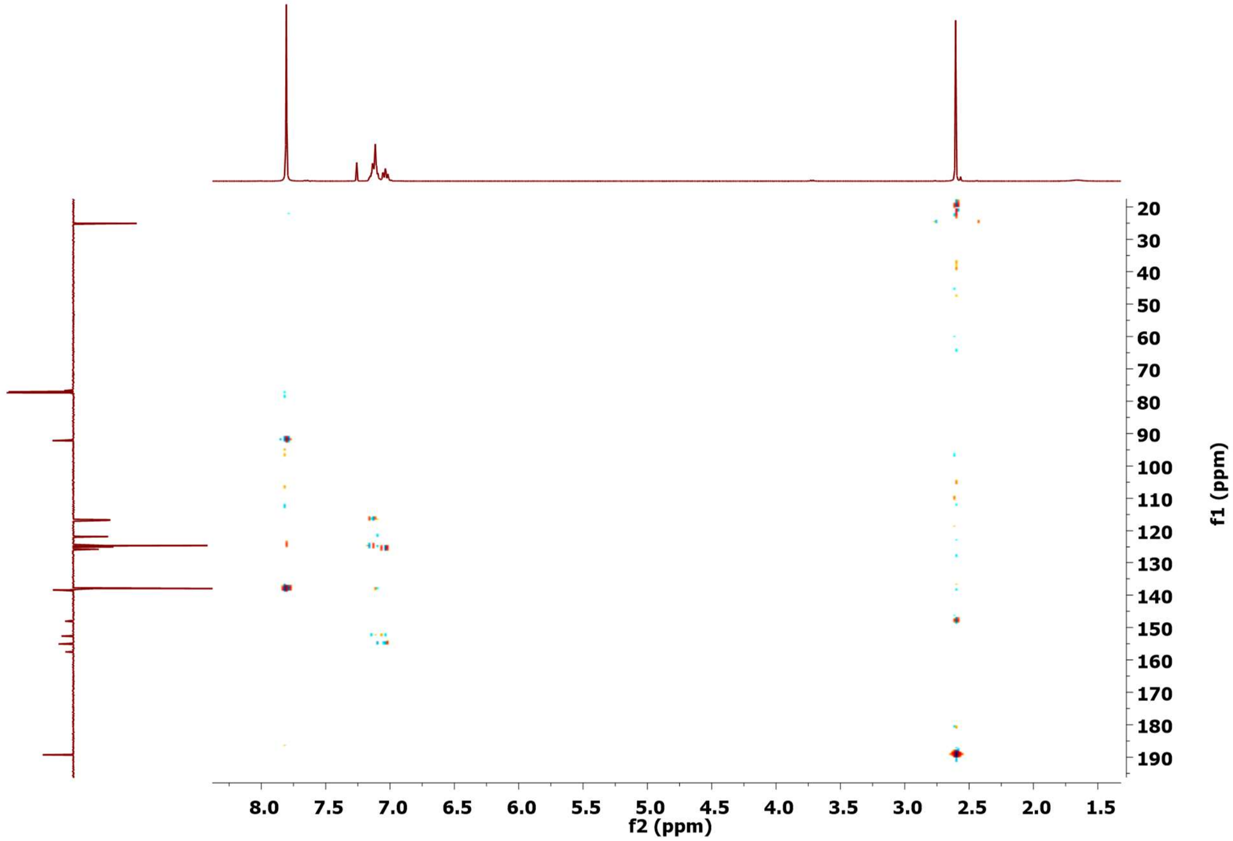

^{1}H NMR ($CDCl_3$) spectrum of (Z)-1-(5-((2-fluorophenyl)imino)-4-(p-tolyl)-4,5-dihydro-1,3,4-thiadiazol-2-yl)ethan-1-one

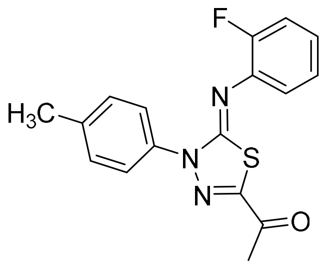

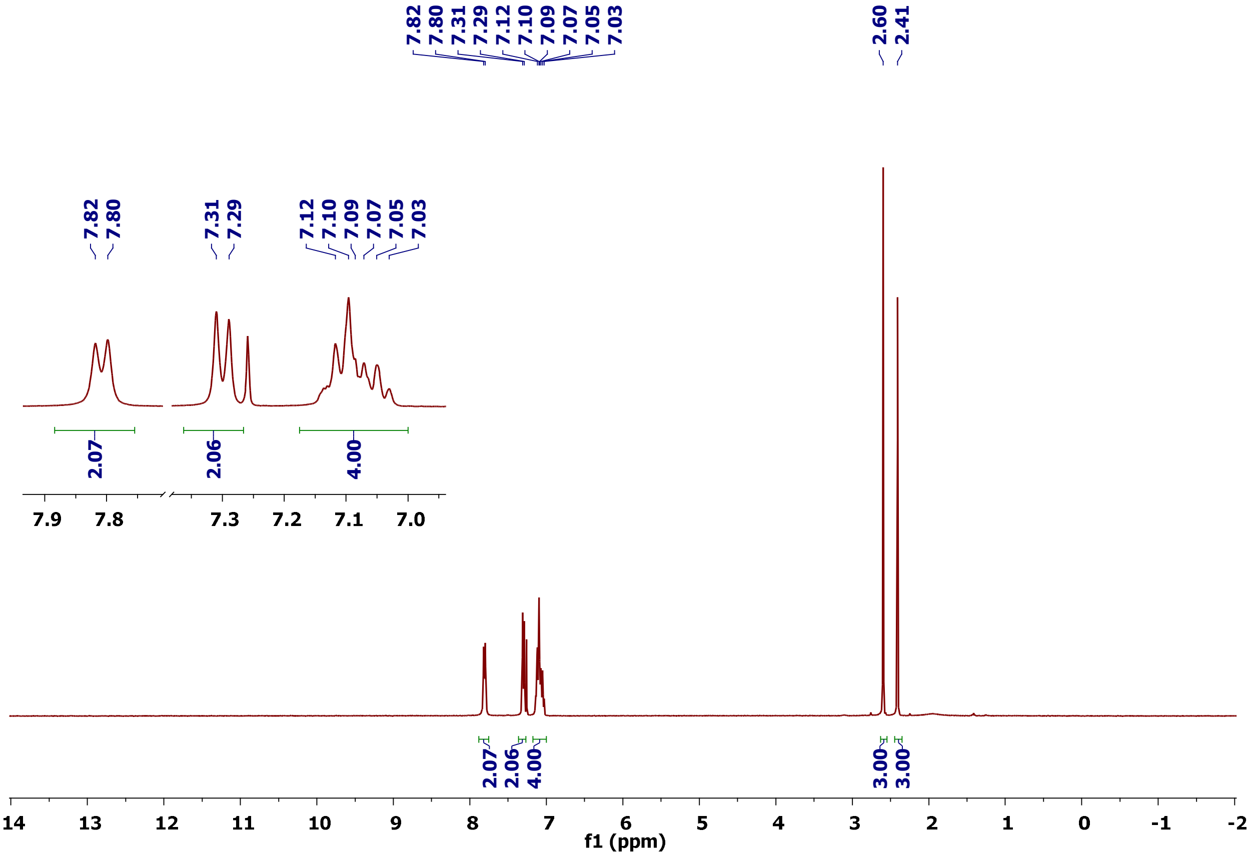

^{13}C NMR ($CDCl_3$) spectrum of (Z)-1-(5-((2-fluorophenyl)imino)-4-(p-tolyl)-4,5-dihydro-1,3,4-thiadiazol-2-yl)ethan-1-one

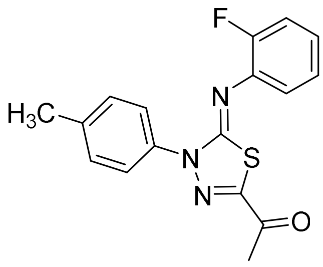

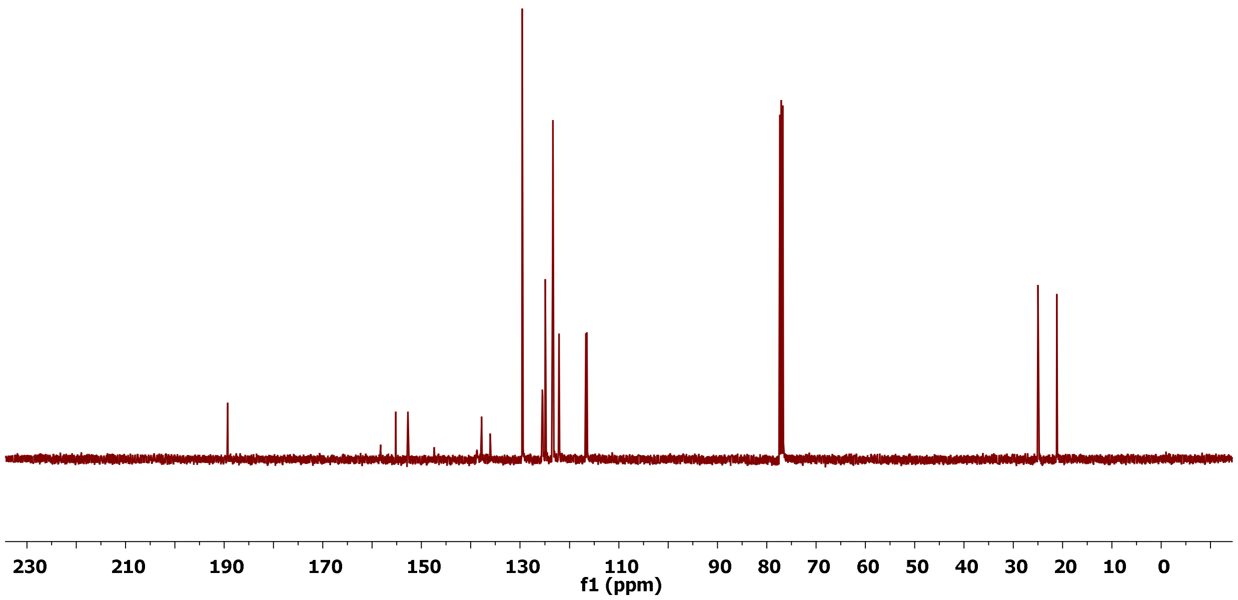

^{13}C CRAPT NMR ($CDCl_3$) spectrum of (Z)-1-(5-((2-fluorophenyl)imino)-4-(p-tolyl)-4,5-dihydro-1,3,4-thiadiazol-2-yl)ethan-1-one

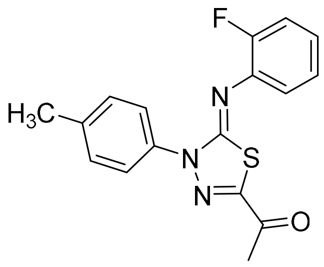

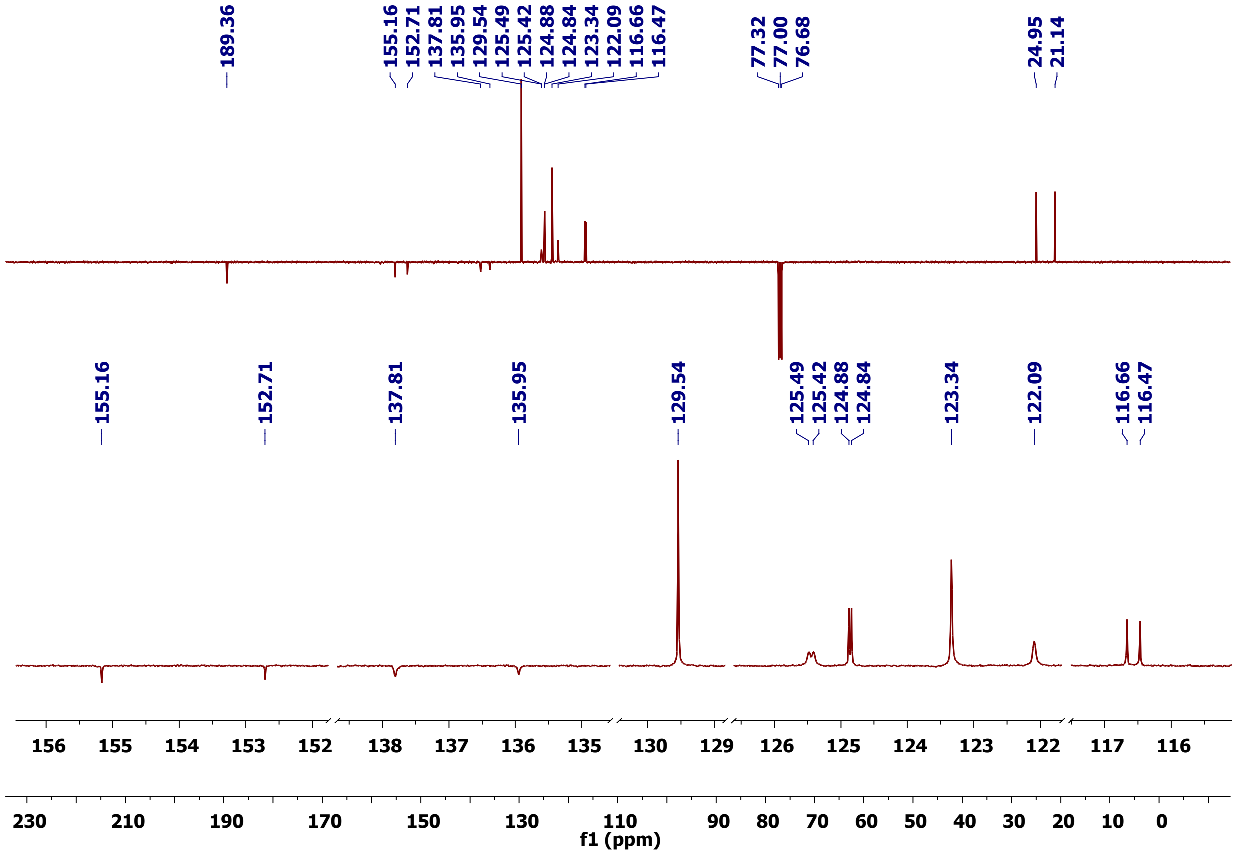

^{1}H-^{1}H gDQCOSY NMR ($CDCl_3$) spectrum of (Z)-1-(5-((2-fluorophenyl)imino)-4-(p-tolyl)-4,5-dihydro-1,3,4-thiadiazol-2-yl)ethan-1-one

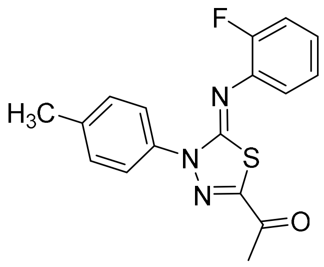

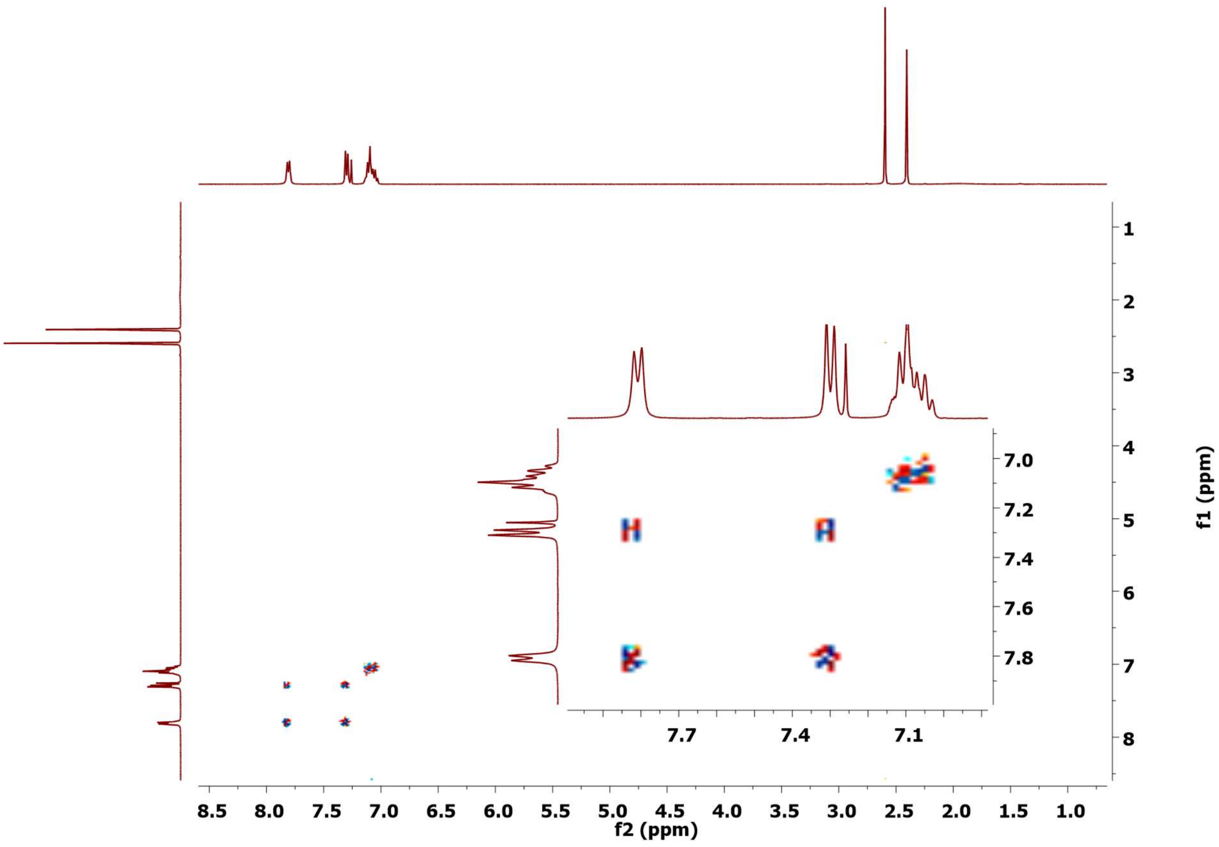

^{1}H-^{13}C-HSQC NMR ($CDCl_3$) spectrum of (Z)-1-(5-((2-fluorophenyl)imino)-4-(p-tolyl)-4,5-dihydro-1,3,4-thiadiazol-2-yl)ethan-1-one

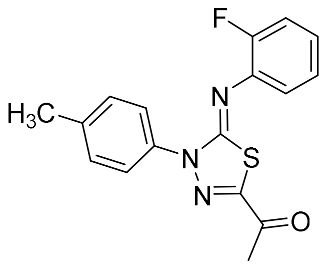

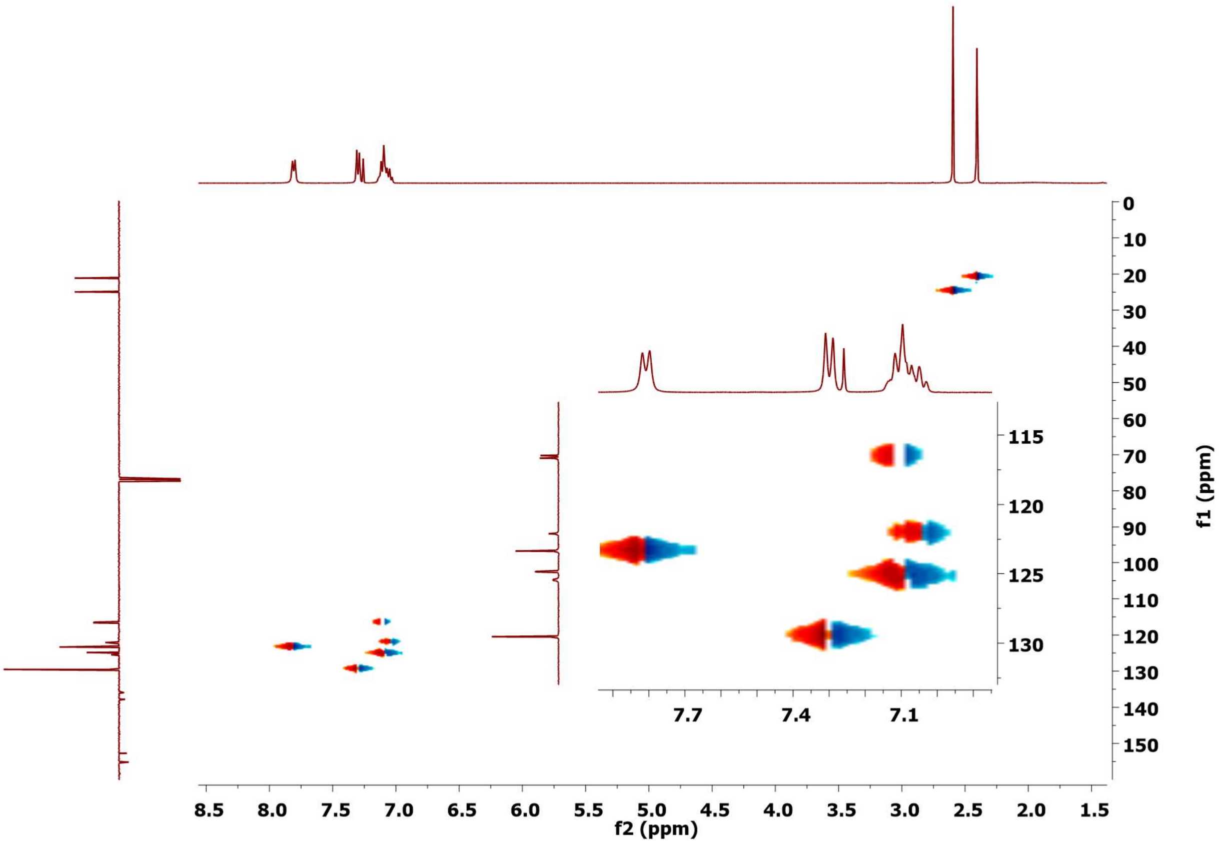

^{1}H-^{13}C-gHMBC NMR ($CDCl_3$) spectrum of (Z)-1-(5-((2-fluorophenyl)imino)-4-(p-tolyl)-4,5-dihydro-1,3,4-thiadiazol-2-yl)ethan-1-one

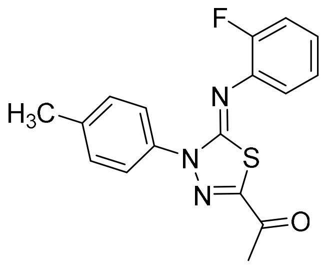

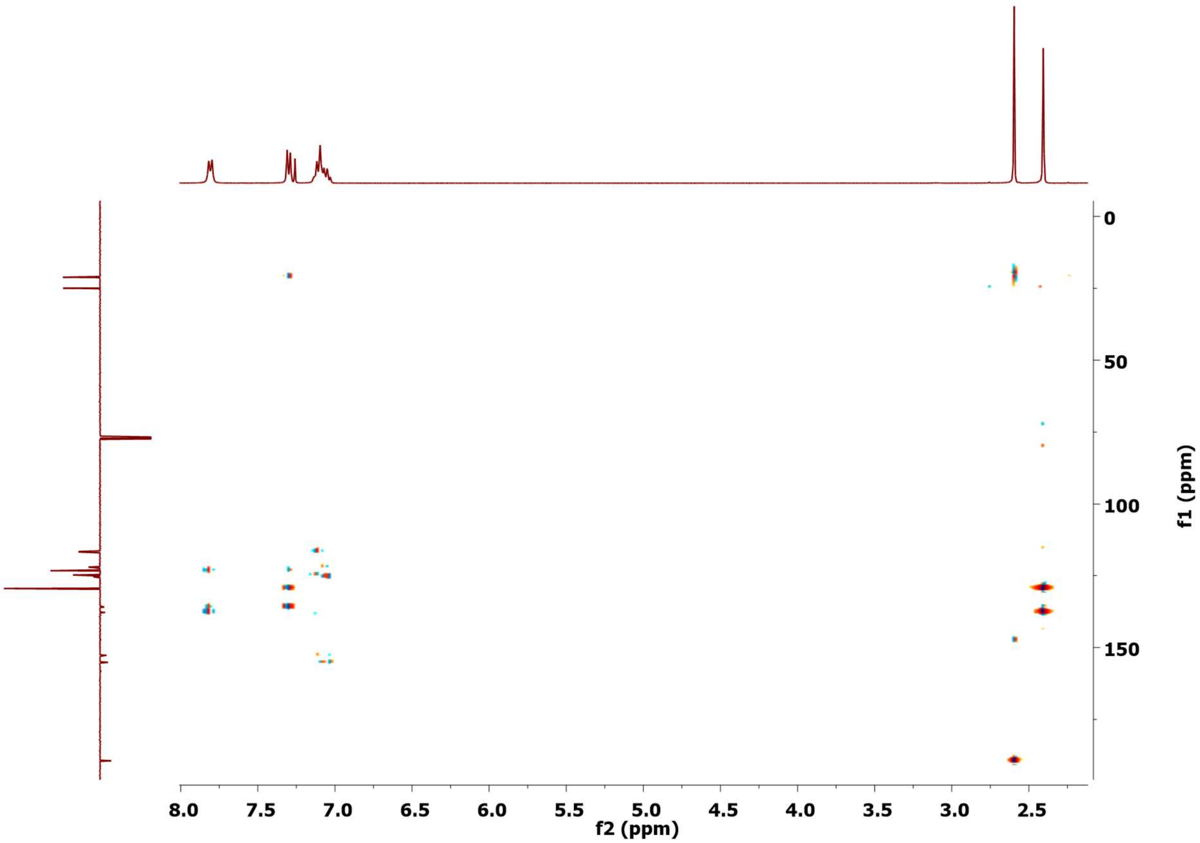

^{1}H NMR ($CDCl_3$) spectrum of (Z)-1-(5-((4-fluorophenyl)imino)-4-(3-(trifluoromethyl)phenyl)-4,5-dihydro-1,3,4-thiadiazol-2-yl)ethan-1-one

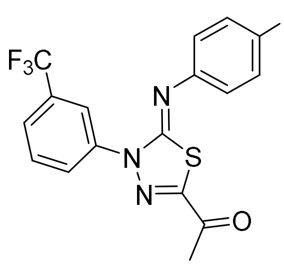

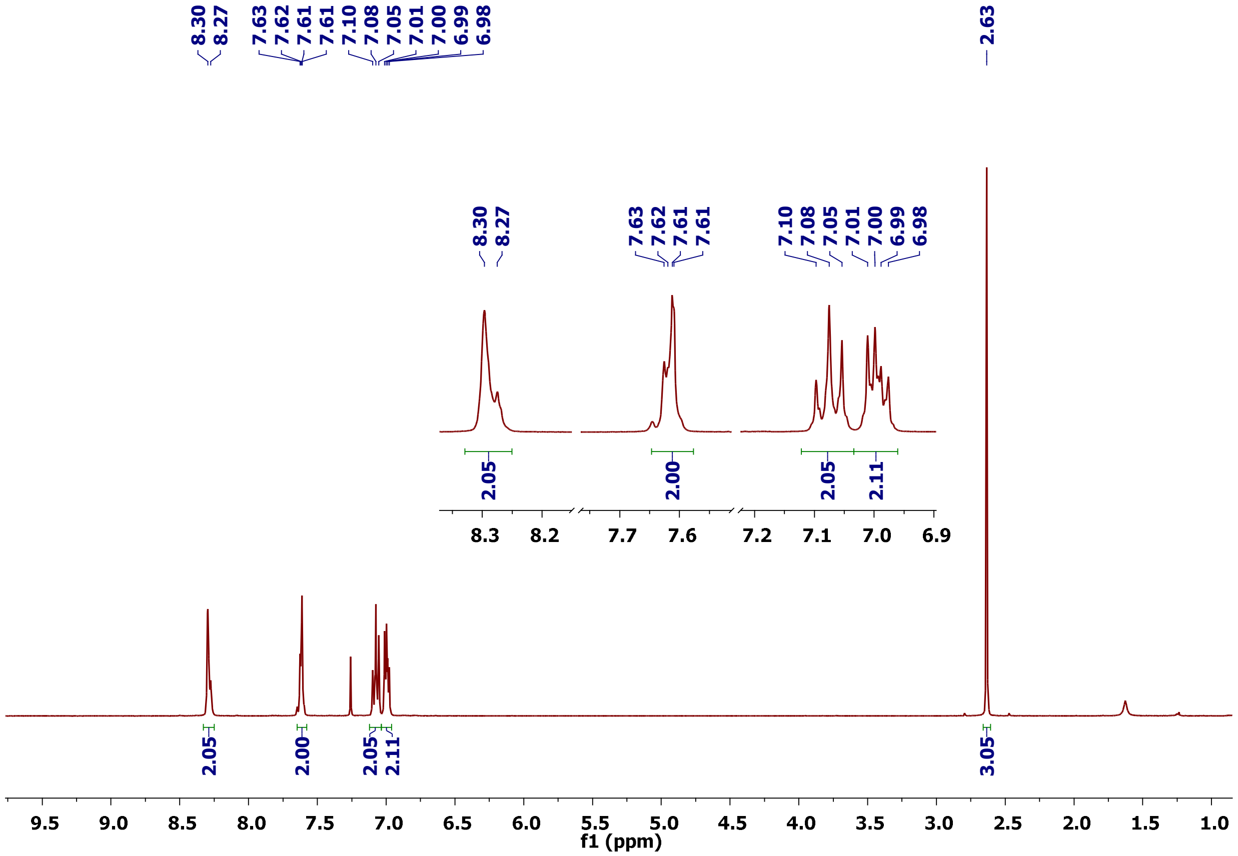

^{13}C NMR ($CDCl_3$) spectrum of (Z)-1-(5-((4-fluorophenyl)imino)-4-(3-(trifluoromethyl)phenyl)-4,5-dihydro-1,3,4-thiadiazol-2-yl)ethan-1-one

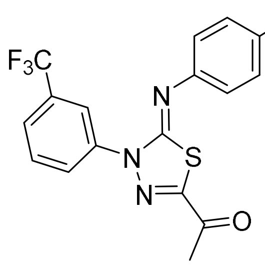

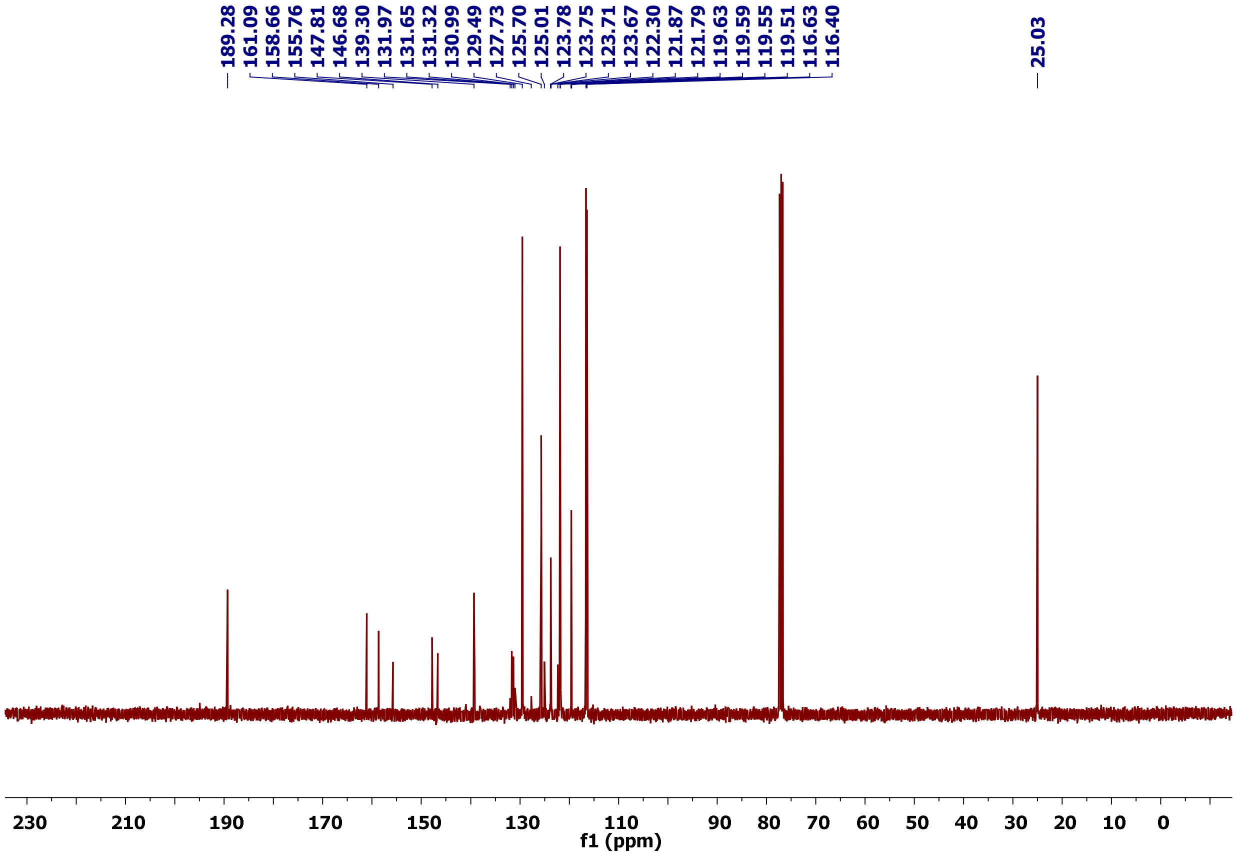

^{13}C CRAPT NMR ($CDCl_3$) spectrum of (Z)-1-(5-((4-fluorophenyl)imino)-4-(3-(trifluoromethyl)phenyl)-4,5-dihydro-1,3,4-thiadiazol-2-yl)ethan-1-one

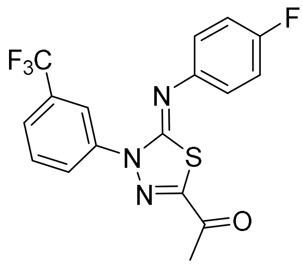

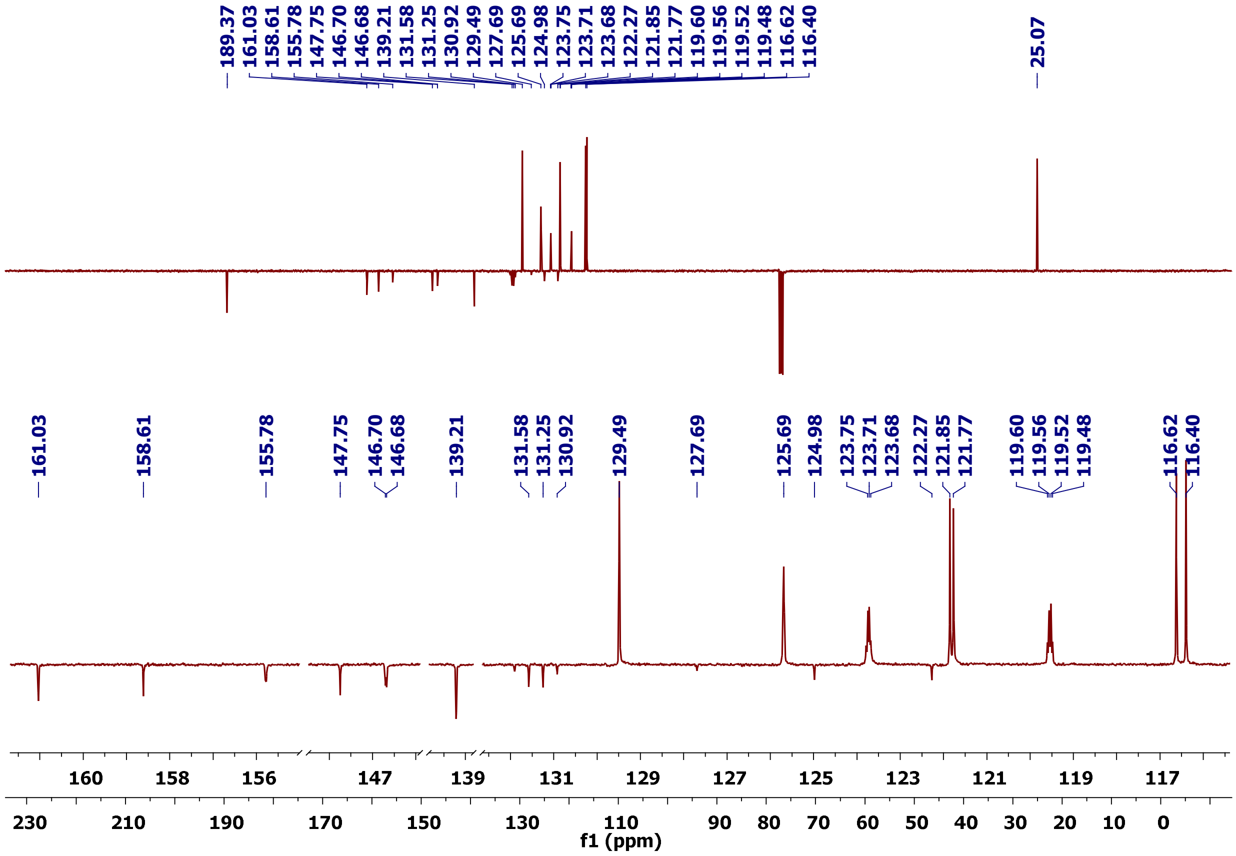

^1H-^1H gDQCOSY NMR ($CDCl_3$) spectrum of (Z)-1-(5-((4-fluorophenyl)imino)-4-(3-(trifluoromethyl)phenyl)-4,5-dihydro-1,3,4-thiadiazol-2-yl)ethan-1-one

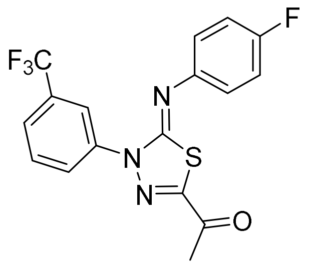

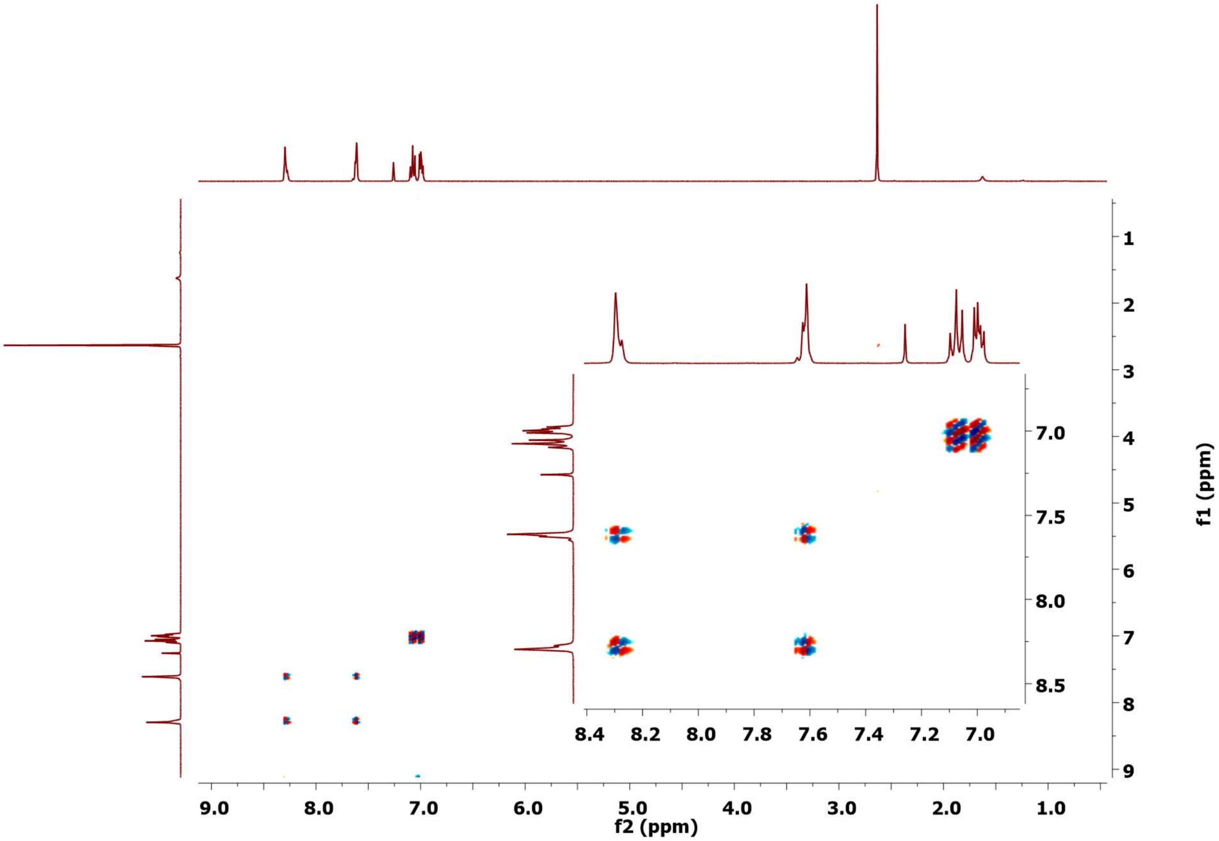

^{1}H-^{13}C-HSQC NMR ($CDCl_3$) spectrum of (Z)-1-(5-((4-fluorophenyl)imino)-4-(3-(trifluoromethyl)phenyl)-4,5-dihydro-1,3,4-thiadiazol-2-yl)ethan-1-one

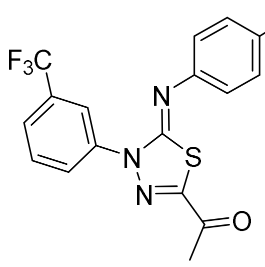

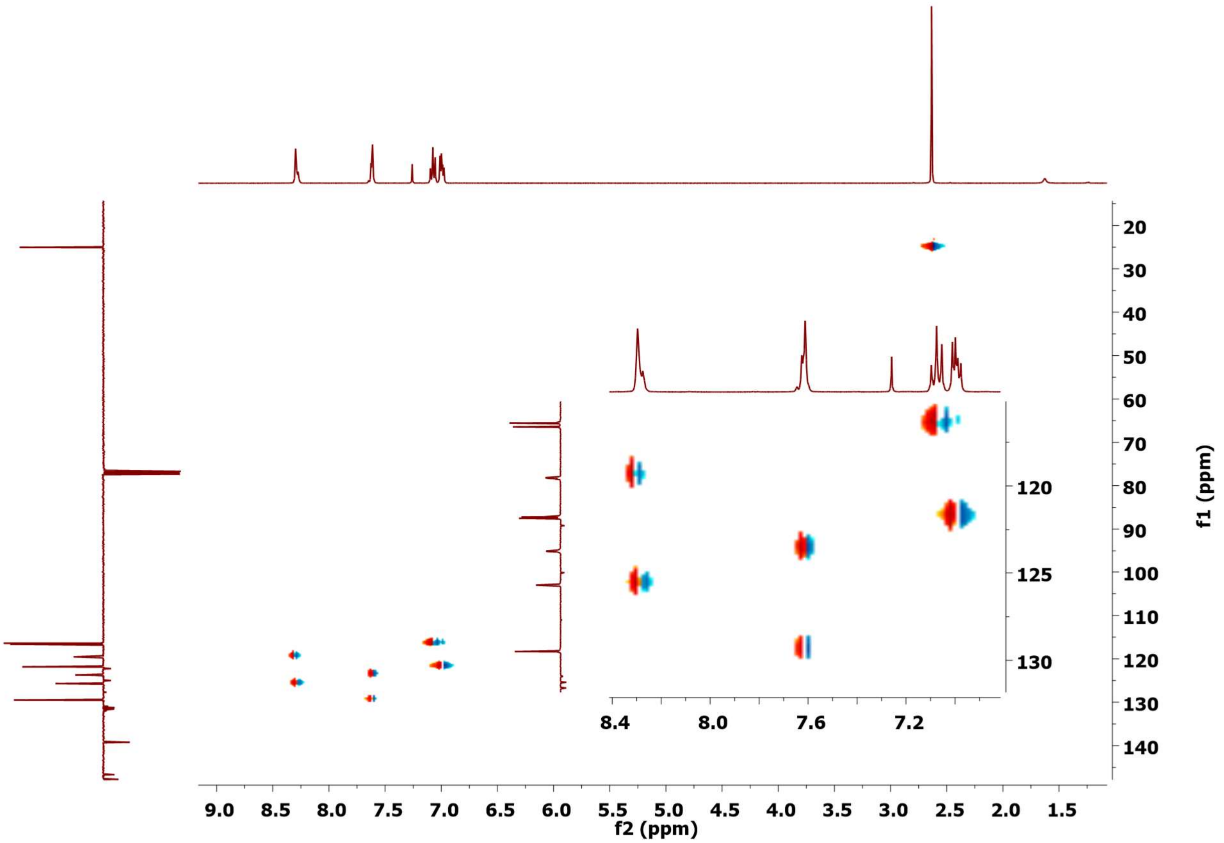

^{1}H-^{13}C-gHMBC NMR ($CDCl_3$) spectrum of (Z)-1-(5-((4-fluorophenyl)imino)-4-(3-(trifluoromethyl)phenyl)-4,5-dihydro-1,3,4-thiadiazol-2-yl)ethan-1-one

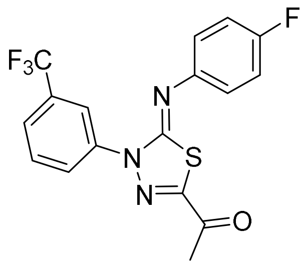

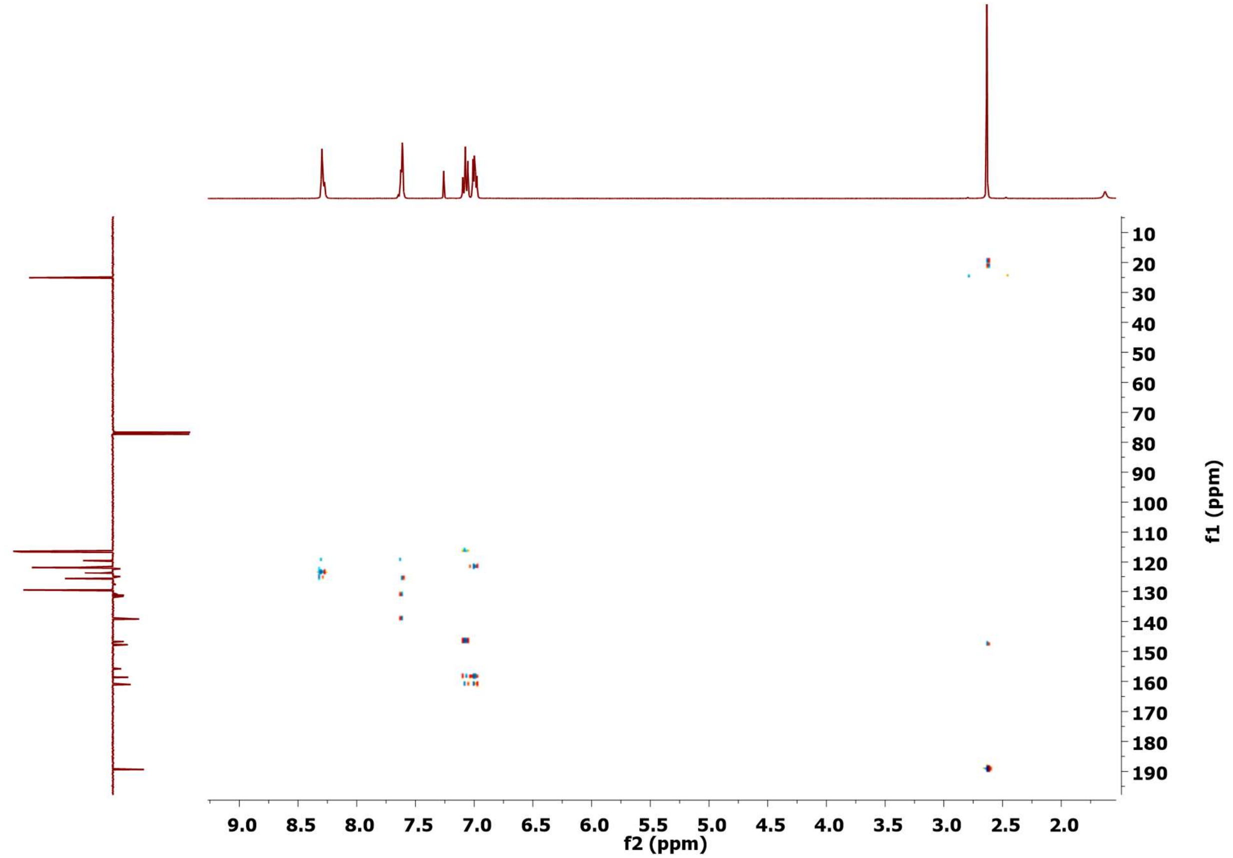

^{1}H NMR ($CDCl_3$) spectrum of (Z)-3-((5-acetyl-3-(4-bromophenyl)-1,3,4-thiadiazol-2(3H)-ylidene)amino)benzonitrile

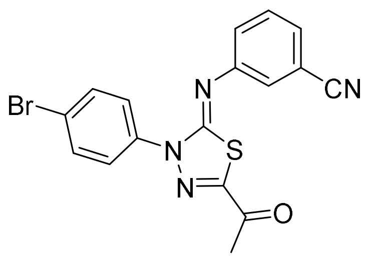

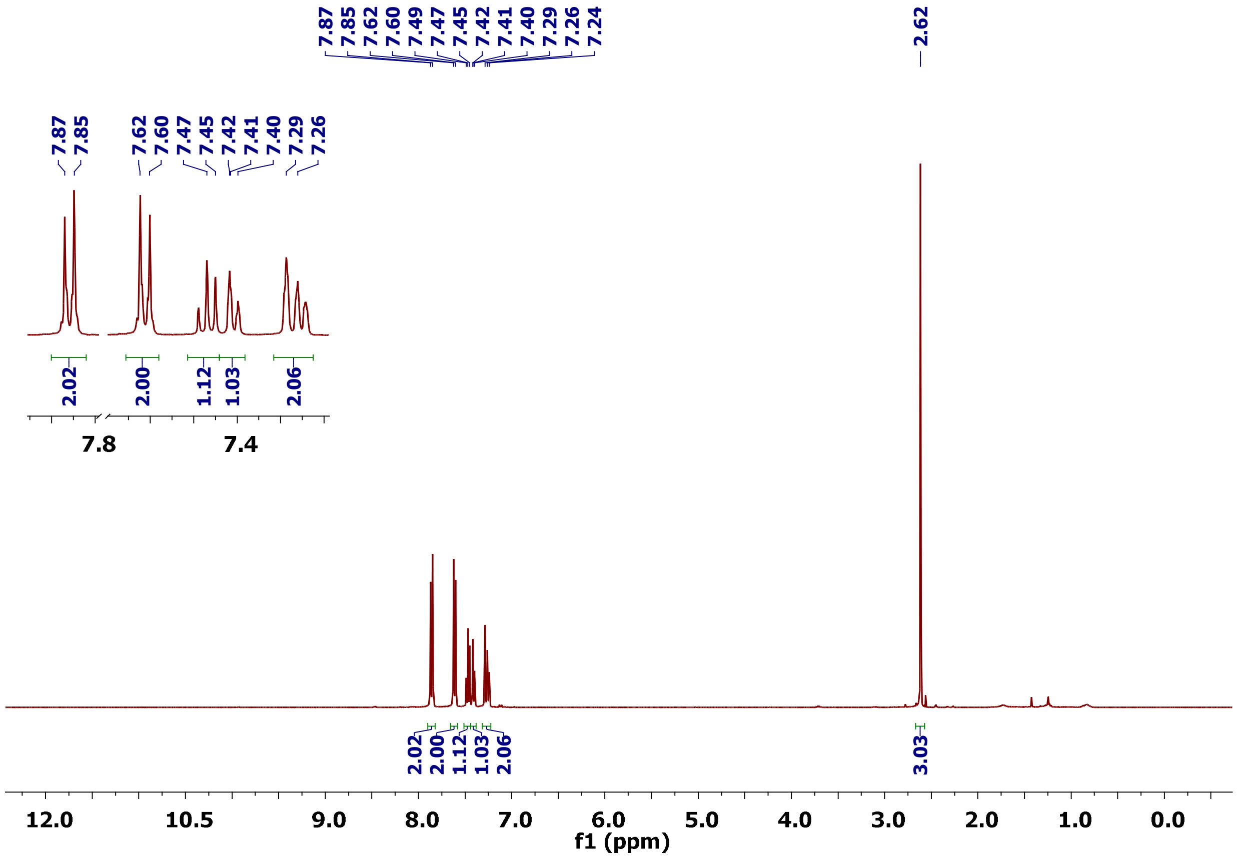

^{13}C NMR ($CDCl_3$) spectrum of (Z)-3-((5-acetyl-3-(4-bromophenyl)-1,3,4-thiadiazol-2(3H)-ylidene)amino)benzonitrile

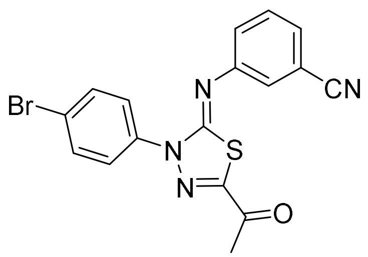

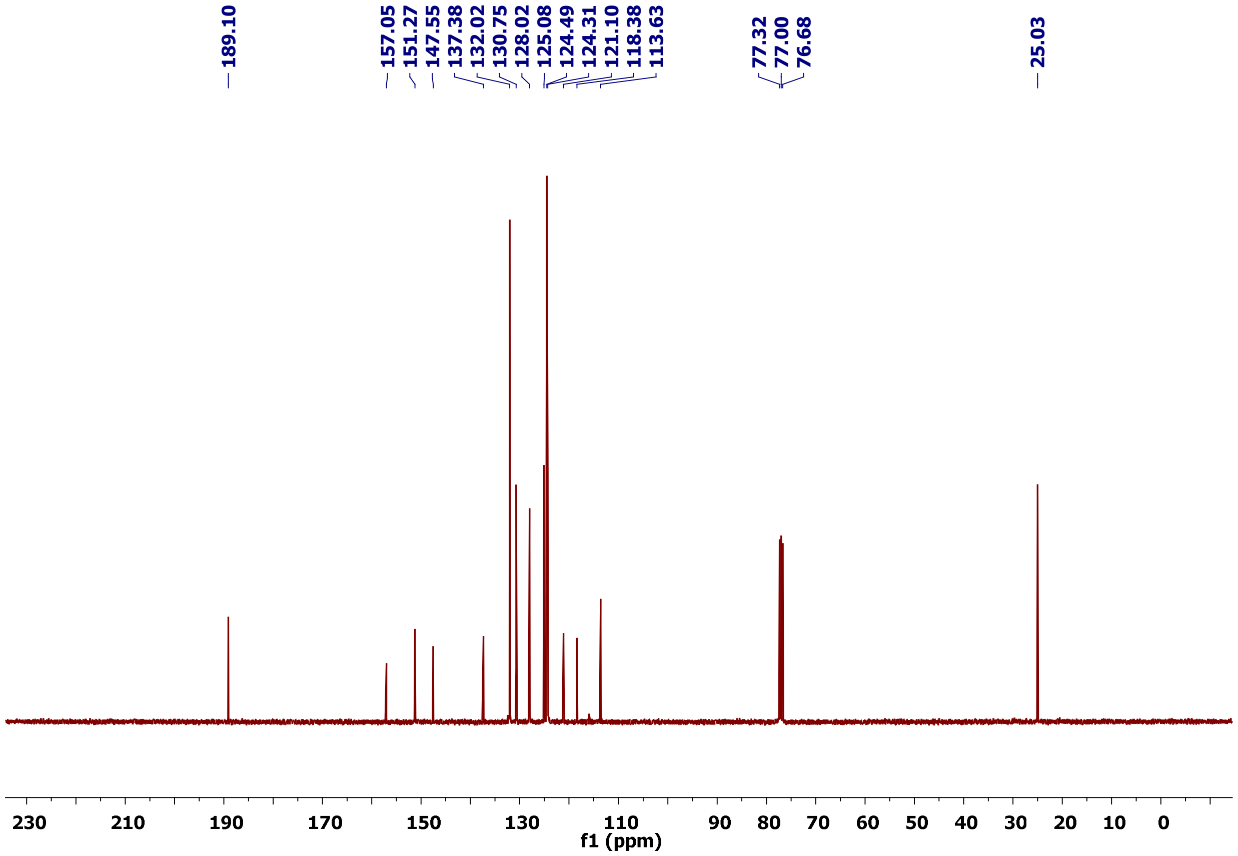

^{13}C CRAPT NMR ($CDCl_3$) spectrum of (Z)-3-((5-acetyl-3-(4-bromophenyl)-1,3,4-thiadiazol-2(3H)-ylidene)amino)benzonitrile

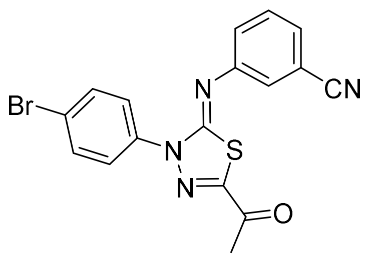

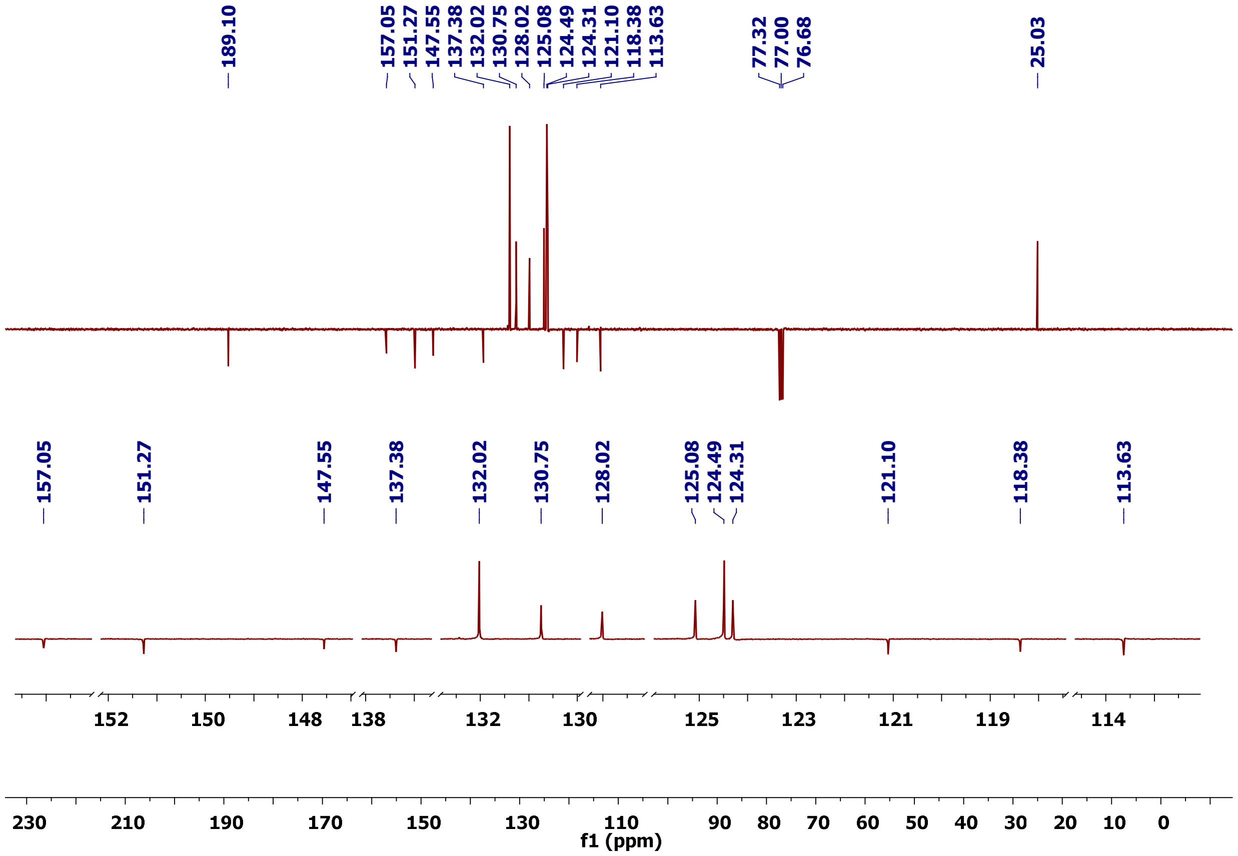

^{1}H-^{1}H gDQCOSY NMR ($CDCl_3$) spectrum of (Z)-3-((5-acetyl-3-(4-bromophenyl)-1,3,4-thiadiazol-2(3H)-ylidene)amino)benzonitrile

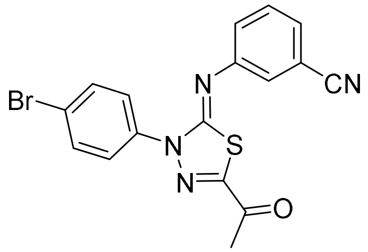

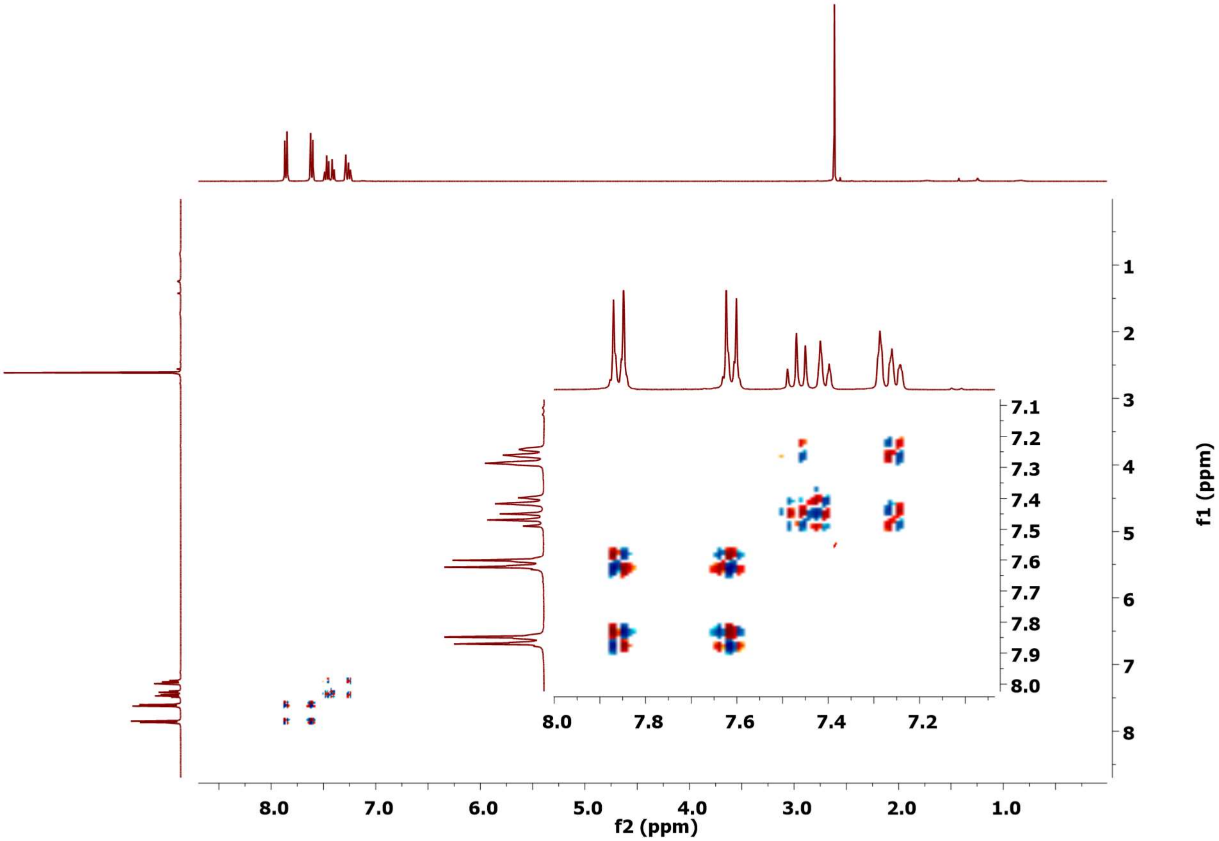

^{1}H-^{13}C-HSQC NMR ($CDCl_3$) spectrum of (Z)-3-((5-acetyl-3-(4-bromophenyl)-1,3,4-thiadiazol-2(3H)-ylidene)amino)benzonitrile

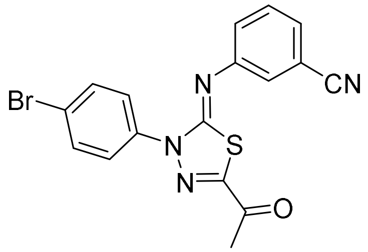

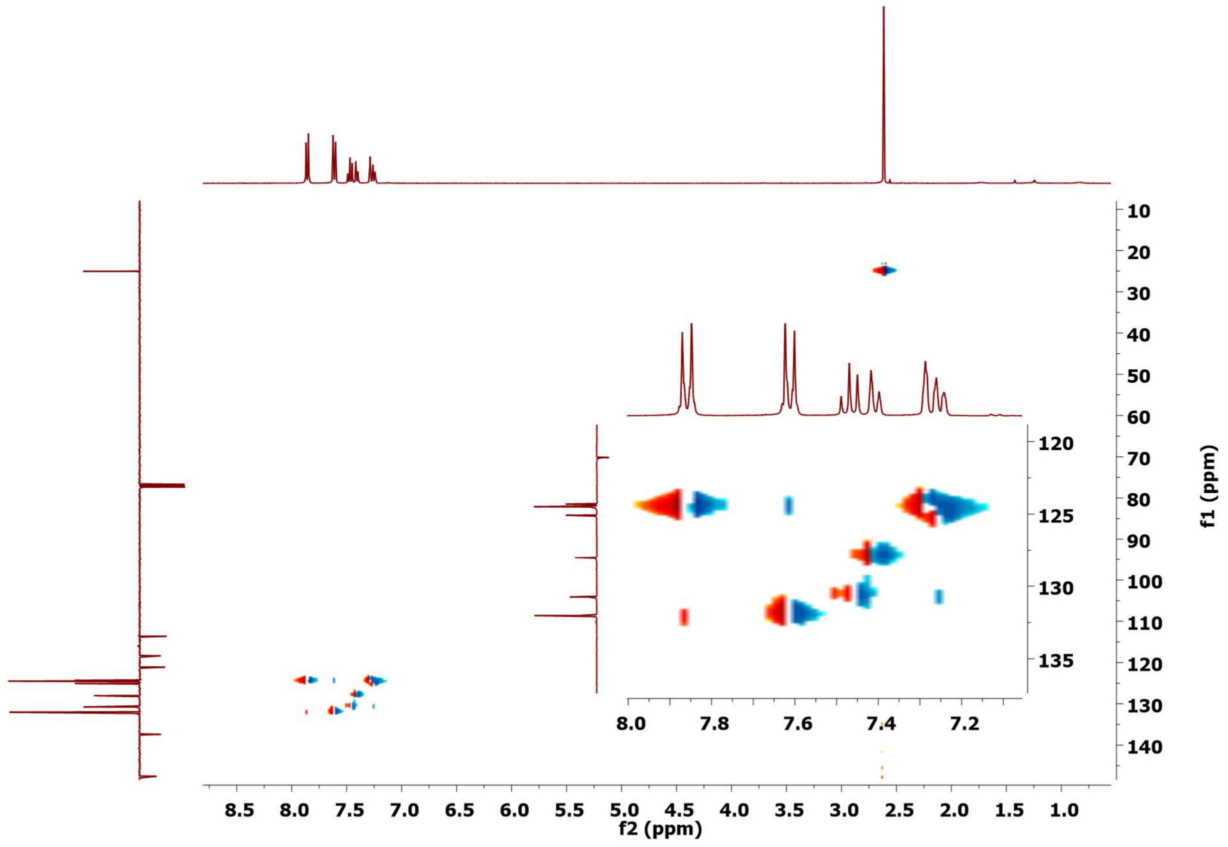

^{1}H-^{13}C-gHMBC NMR ($CDCl_3$) spectrum of (Z)-3-((5-acetyl-3-(4-bromophenyl)-1,3,4-thiadiazol-2(3H)-ylidene)amino)benzonitrile

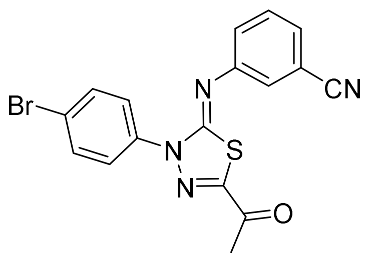

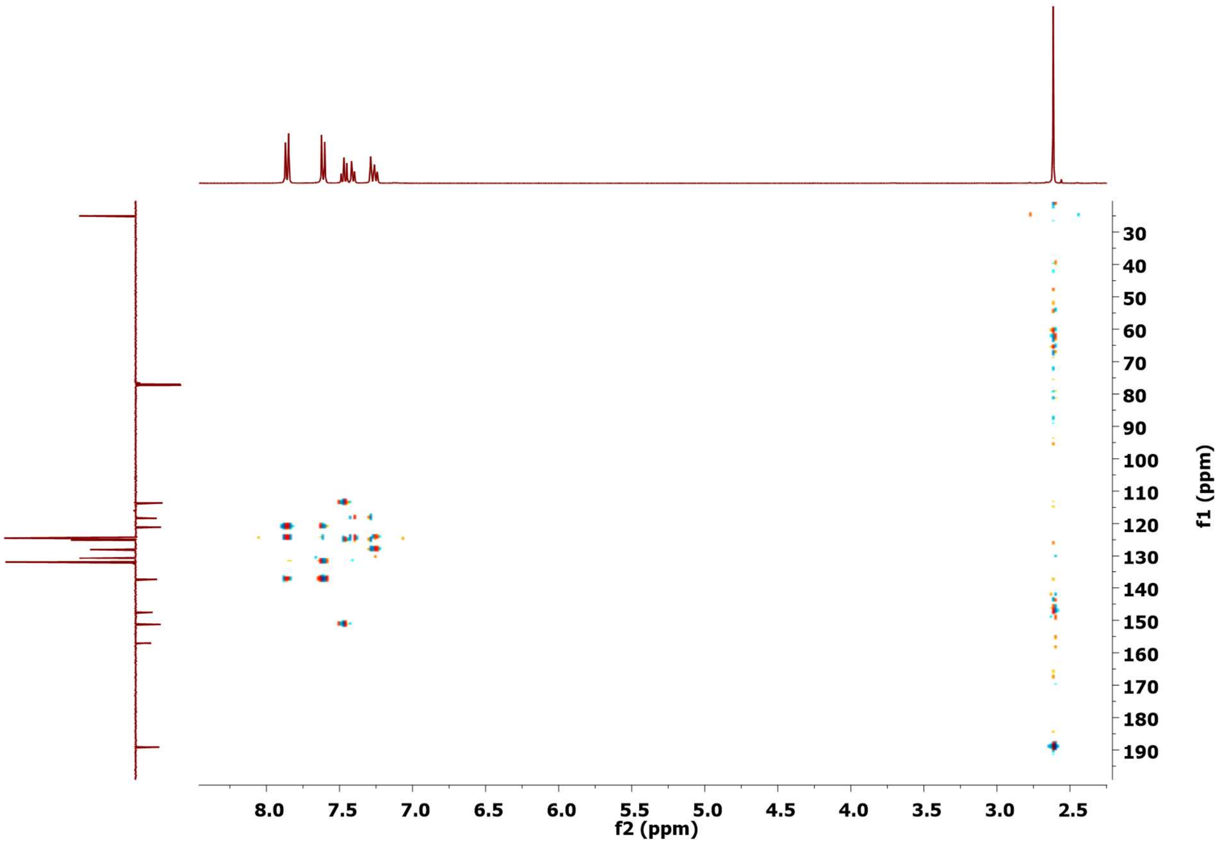

^{1}H NMR ($CDCl_3$) spectrum of (Z)-1-(5-((2-bromophenyl)imino)-4-(4-iodophenyl)-4,5-dihydro-1,3,4-thiadiazol-2-yl)ethan-1-one

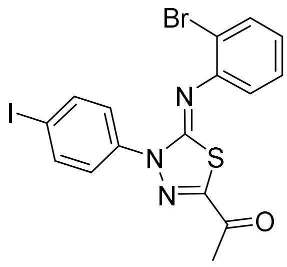

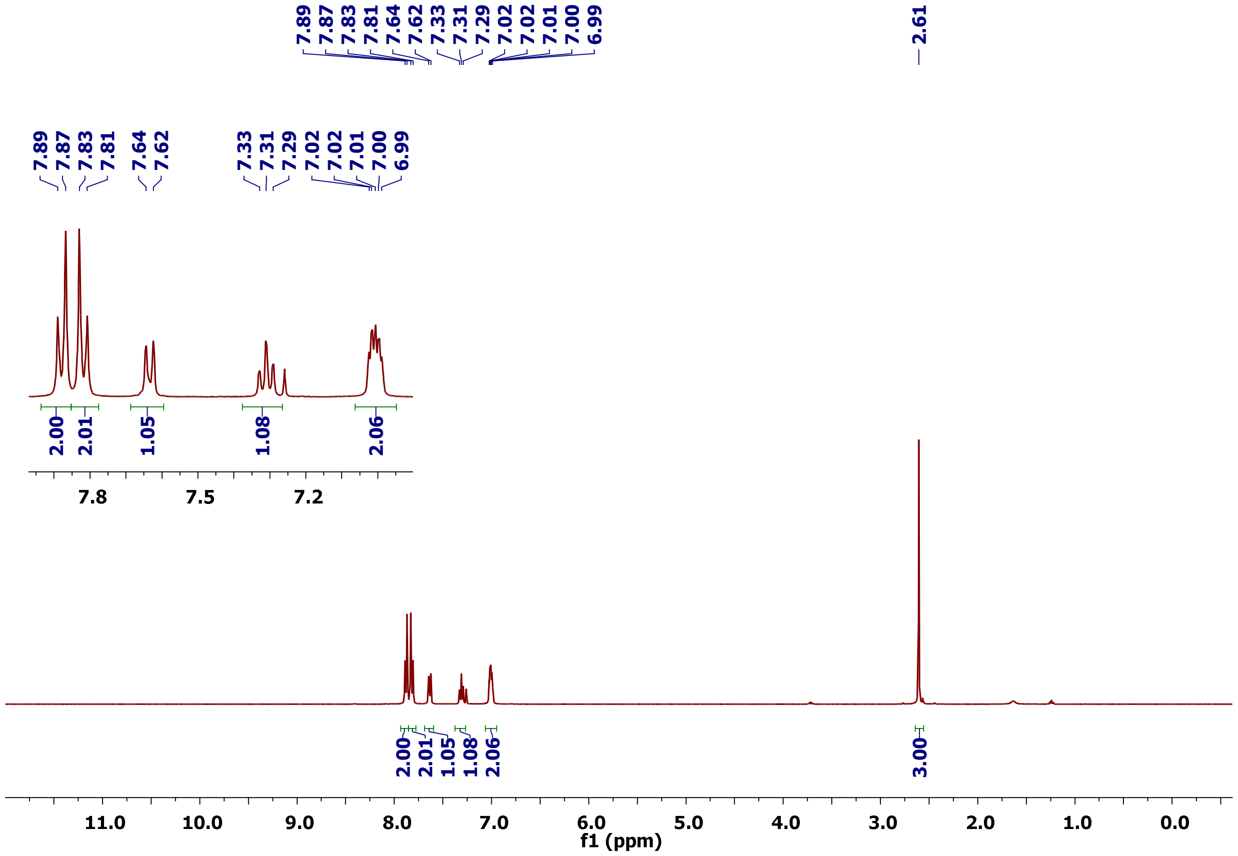

^{13}C NMR ($CDCl_3$) spectrum of (Z)-1-(5-((2-bromophenyl)imino)-4-(4-iodophenyl)-4,5-dihydro-1,3,4-thiadiazol-2-yl)ethan-1-one

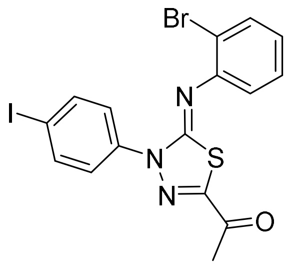

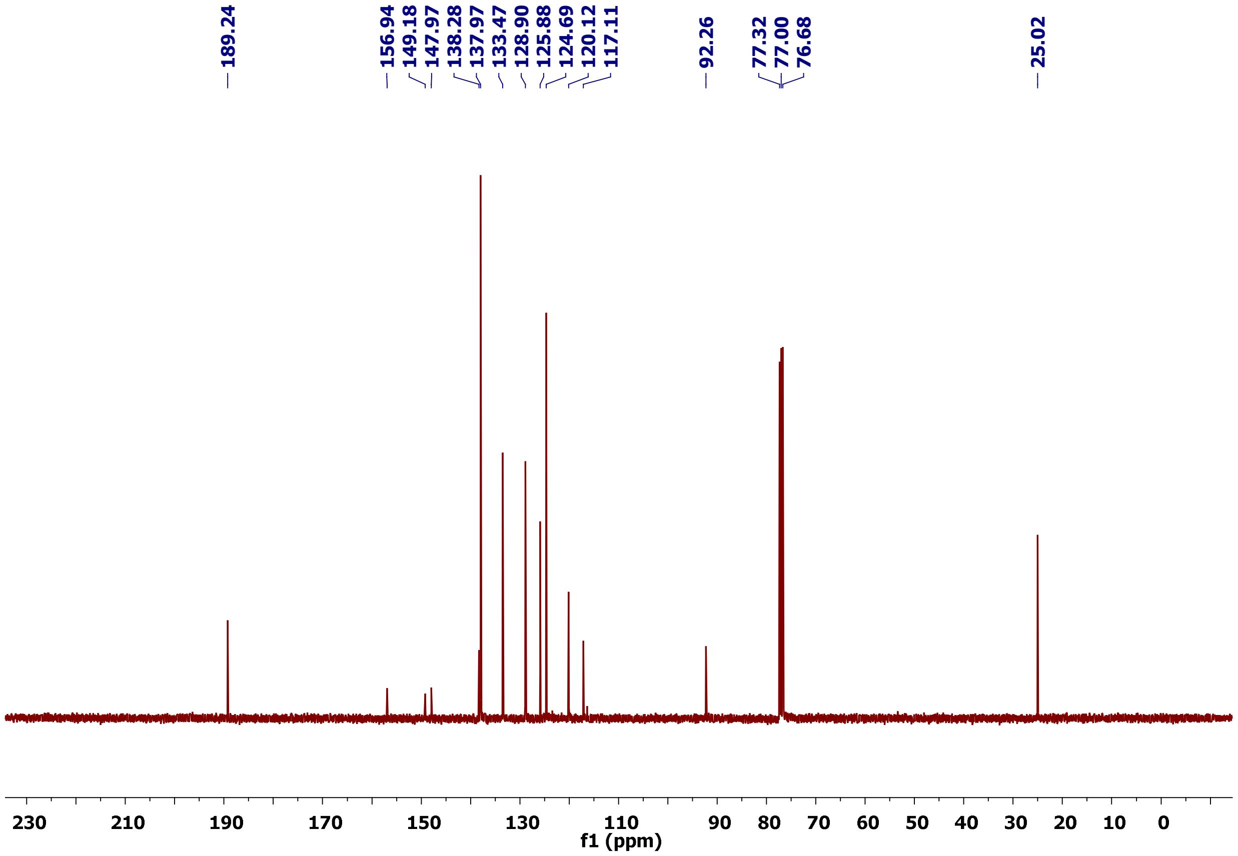

^{13}C CRAPT NMR ($CDCl_3$) spectrum of (Z)-1-(5-((2-bromophenyl)imino)-4-(4-iodophenyl)-4,5-dihydro-1,3,4-thiadiazol-2-yl)ethan-1-one

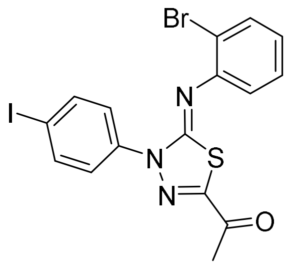

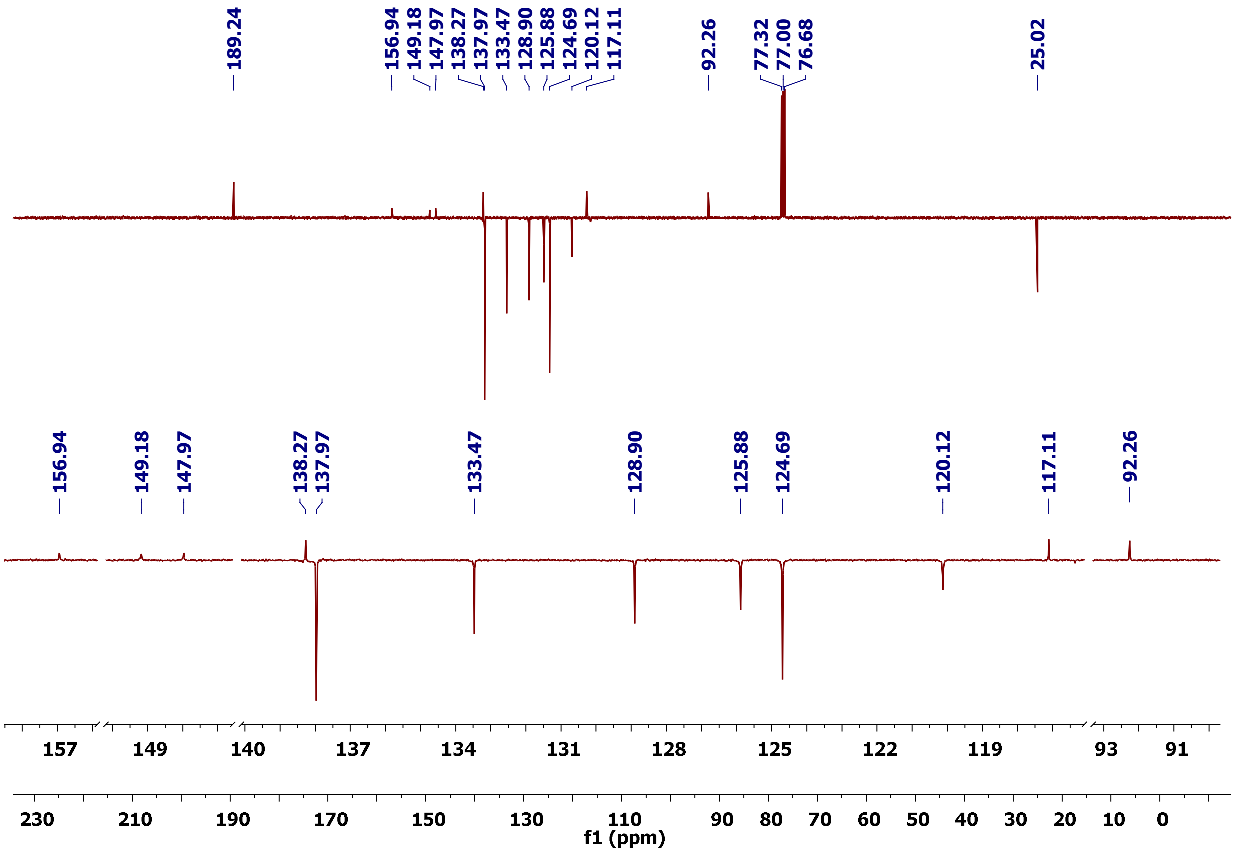

^{1}H-^{1}H gDQCOSY NMR ($CDCl_3$) spectrum of (Z)-1-(5-((2-bromophenyl)imino)-4-(4-iodophenyl)-4,5-dihydro-1,3,4-thiadiazol-2-yl)ethan-1-one

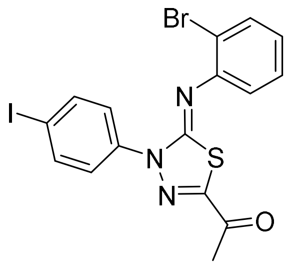

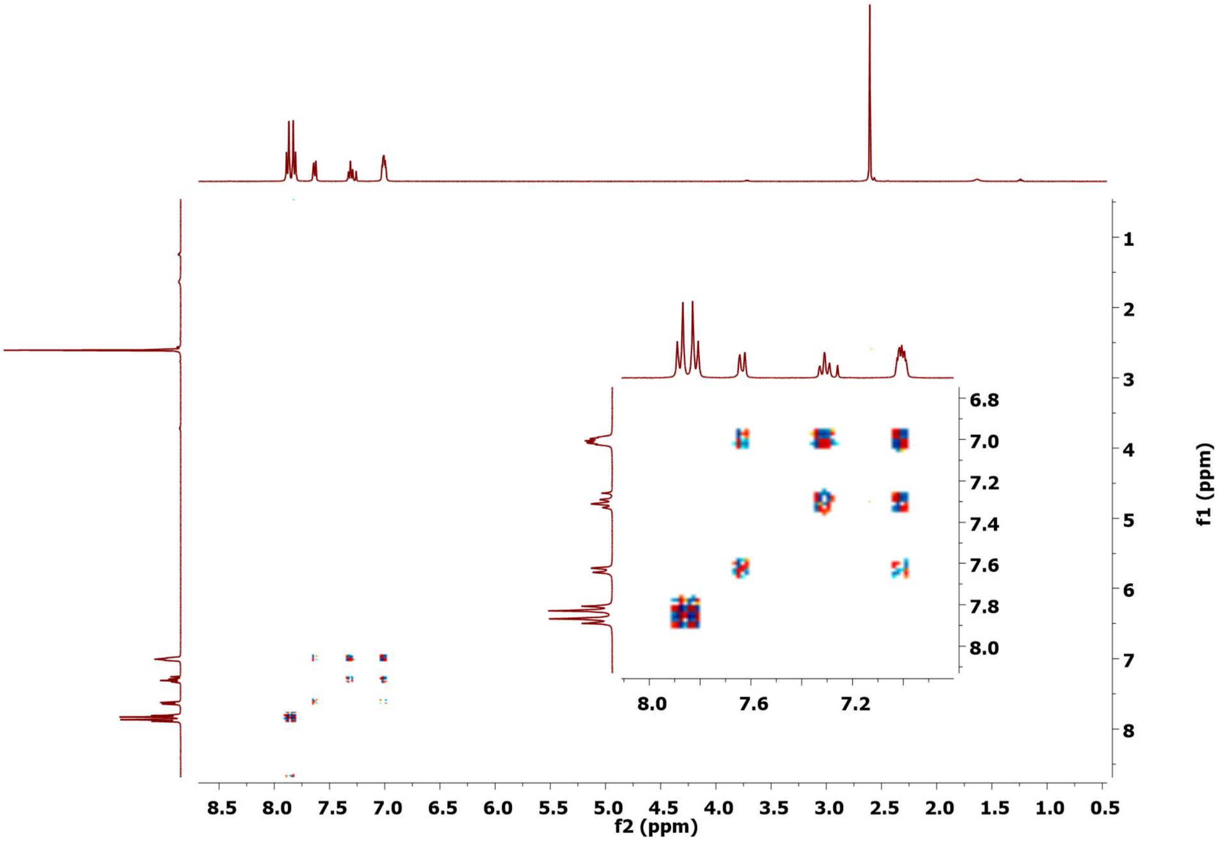

^{1}H-^{13}C-HSQC NMR ($CDCl_3$) spectrum of (Z)-1-(5-((2-bromophenyl)imino)-4-(4-iodophenyl)-4,5-dihydro-1,3,4-thiadiazol-2-yl)ethan-1-one

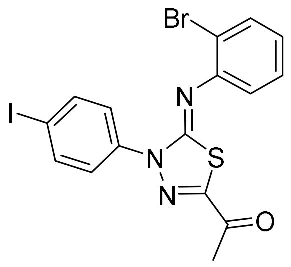

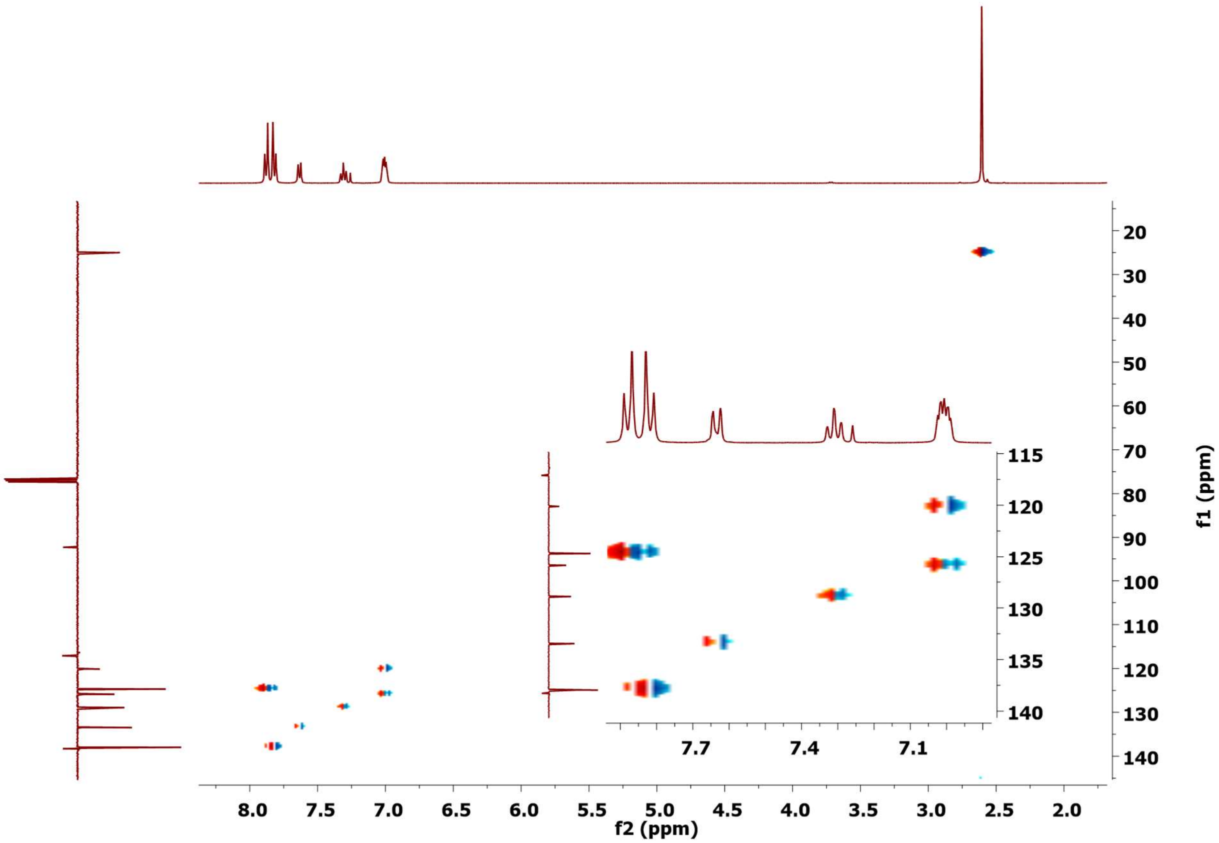

^{1}H-^{13}C-gHMBC NMR ($CDCl_3$) spectrum of (Z)-1-(5-((2-bromophenyl)imino)-4-(4-iodophenyl)-4,5-dihydro-1,3,4-thiadiazol-2-yl)ethan-1-one

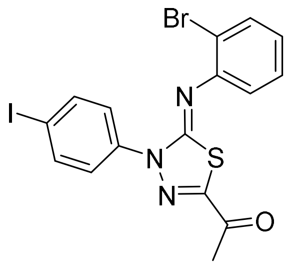

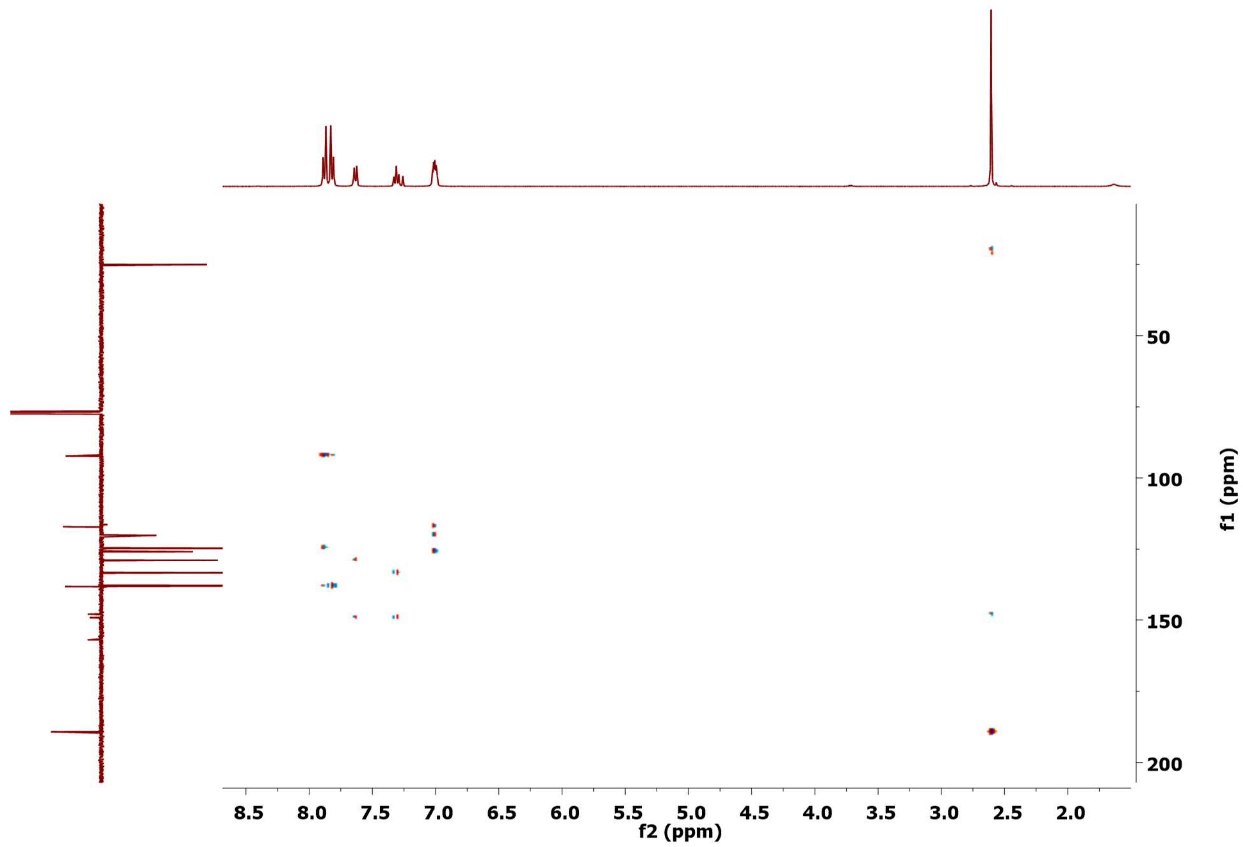

^{1}H NMR ($CDCl_3$) spectrum of (Z)-1-(4-(4-iodophenyl)-5-((2-(methylthio)phenyl)imino)-4,5-dihydro-1,3,4-thiadiazol-2-yl)ethan-1-one

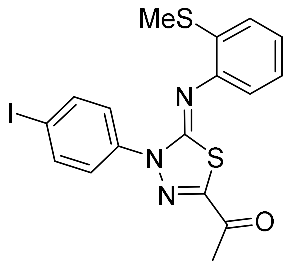

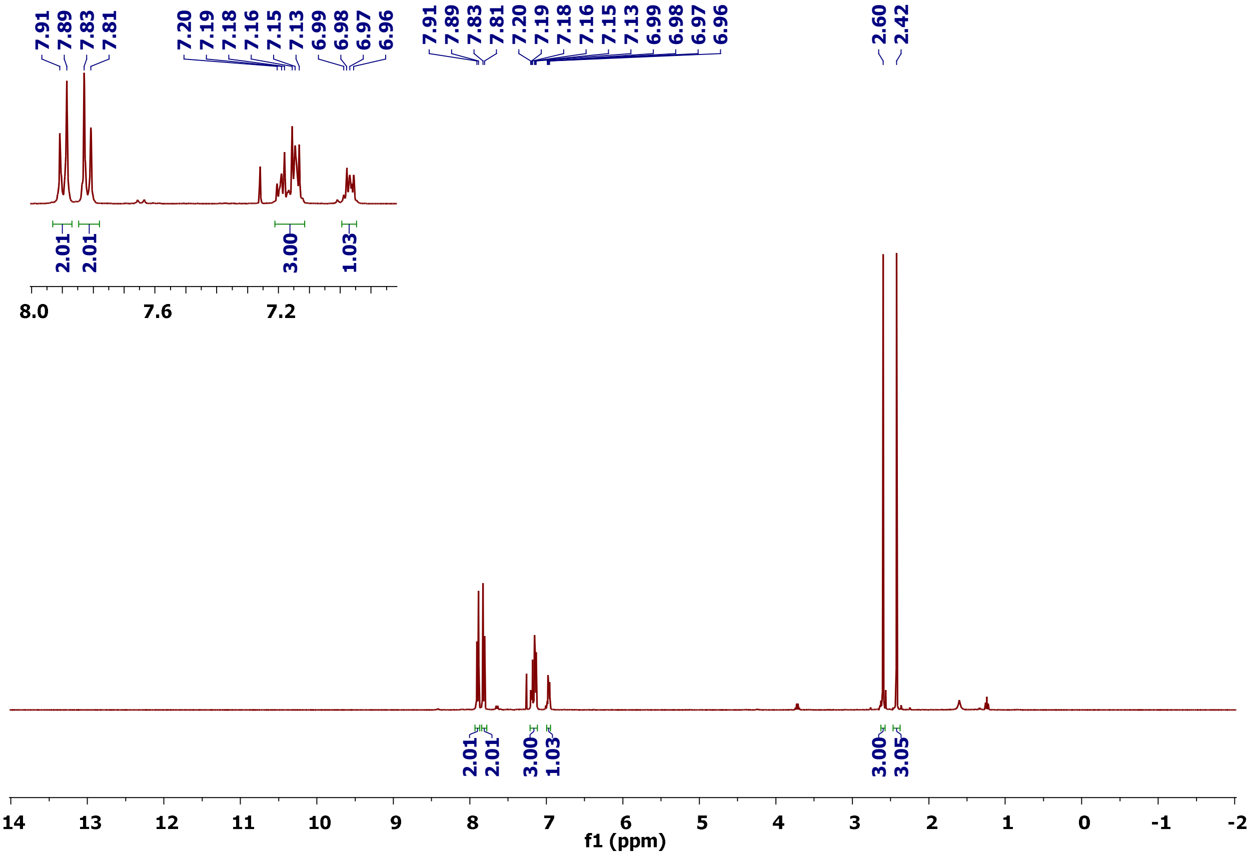

^{13}C NMR ($CDCl_3$) spectrum of (Z)-1-(4-(4-iodophenyl)-5-((2-(methylthio)phenyl)imino)-4,5-dihydro-1,3,4-thiadiazol-2-yl)ethan-1-one

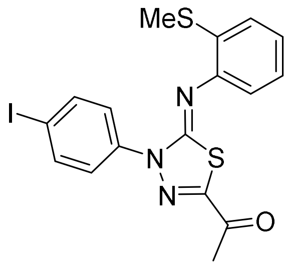

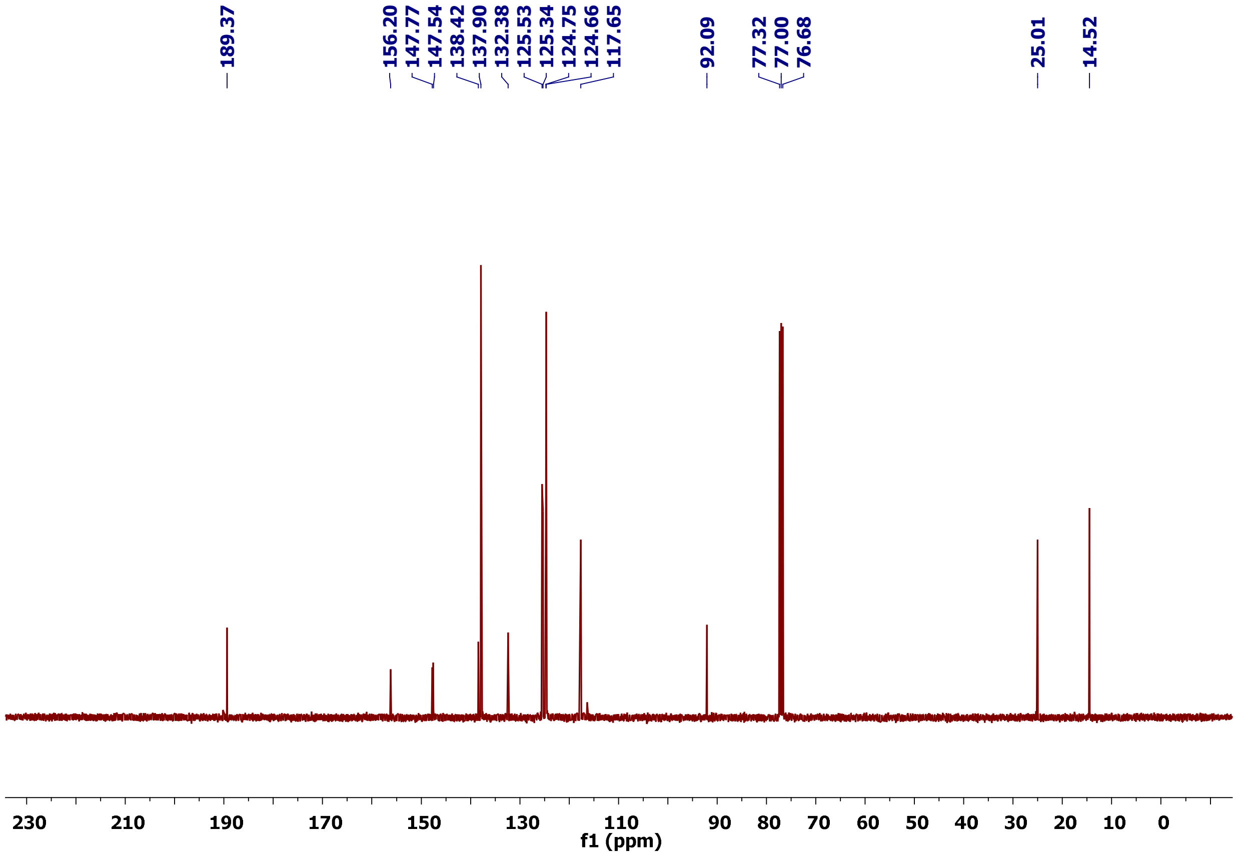

^{13}C CRAPT NMR ($CDCl_3$) spectrum of (Z)-1-(4-(4-iodophenyl)-5-((2-(methylthio)phenyl)imino)-4,5-dihydro-1,3,4-thiadiazol-2-yl)ethan-1-one

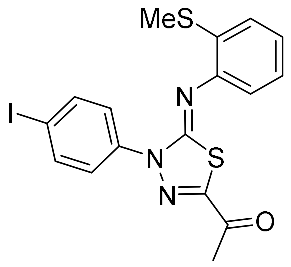

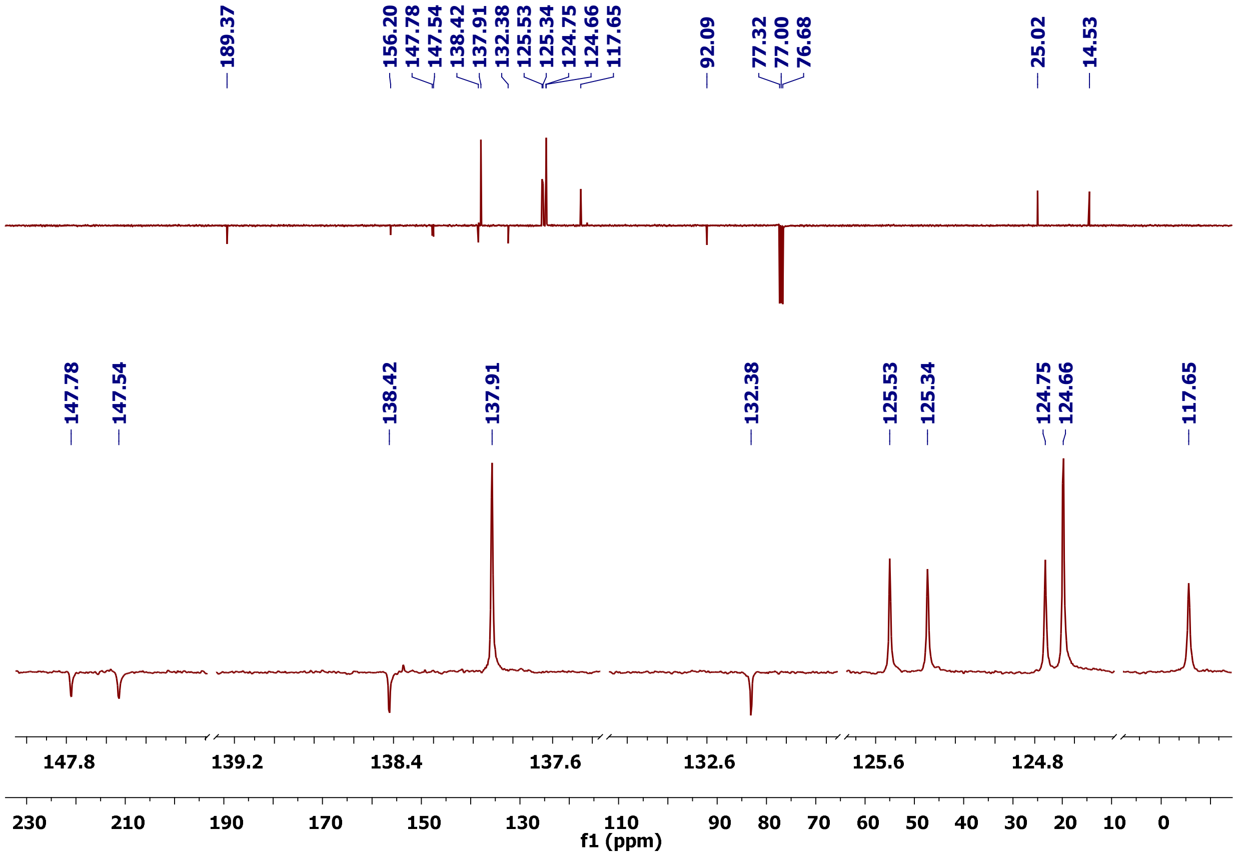

^1H-^1H gDQCOSY NMR ($CDCl_3$) spectrum of (Z)-1-(4-(4-iodophenyl)-5-((2-(methylthio)phenyl)imino)-4,5-dihydro-1,3,4-thiadiazol-2-yl)ethan-1-one

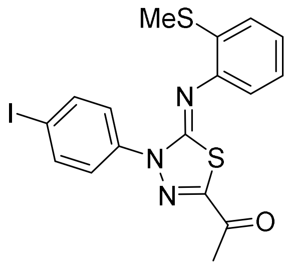

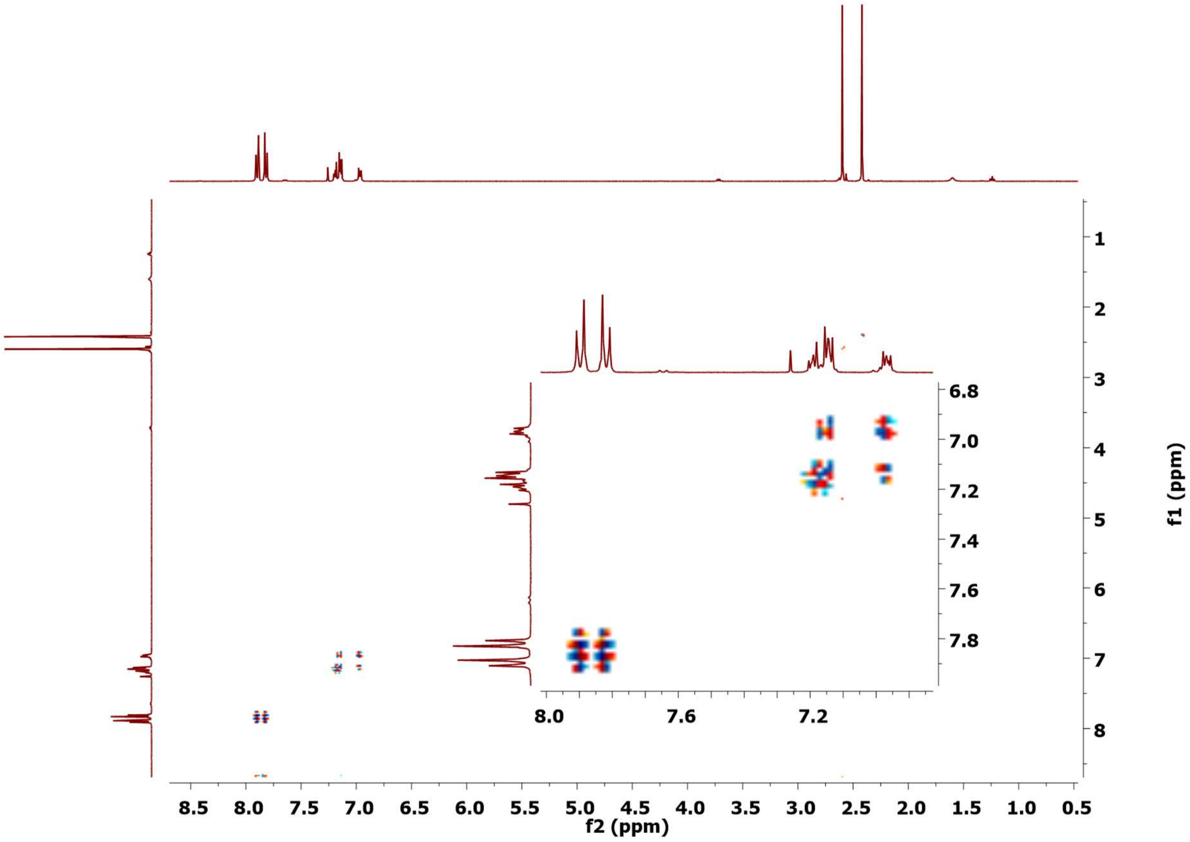

^{1}H-^{13}C-HSQC NMR ($CDCl_3$) spectrum of (Z)-1-(4-(4-iodophenyl)-5-((2-(methylthio)phenyl)imino)-4,5-dihydro-1,3,4-thiadiazol-2-yl)ethan-1-one

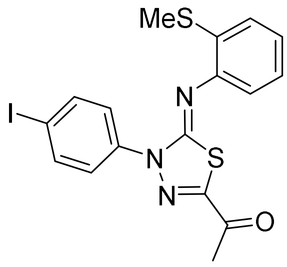

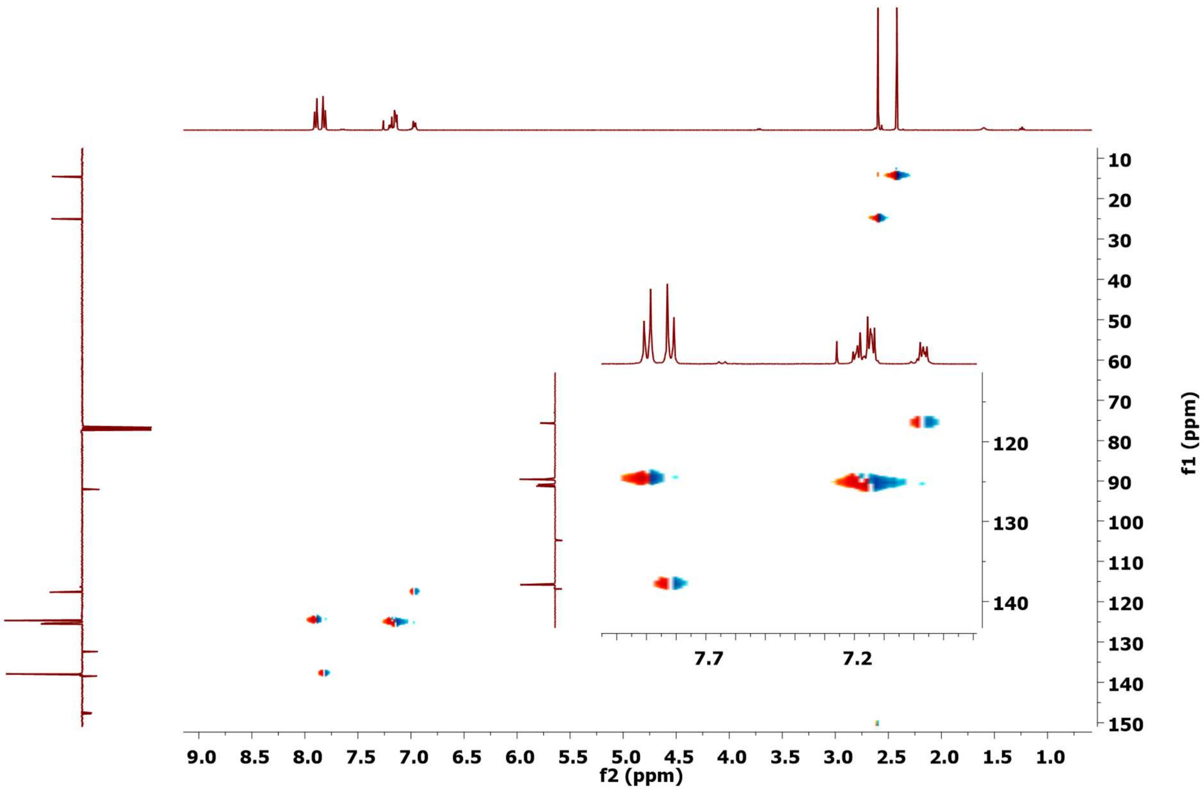

^{1}H-^{13}C-gHMBC NMR ($CDCl_3$) spectrum of (Z)-1-(4-(4-iodophenyl)-5-((2-(methylthio)phenyl)imino)-4,5-dihydro-1,3,4-thiadiazol-2-yl)ethan-1-one

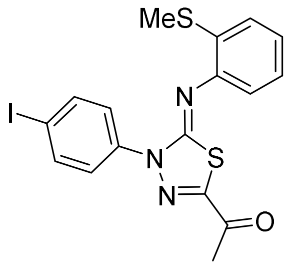

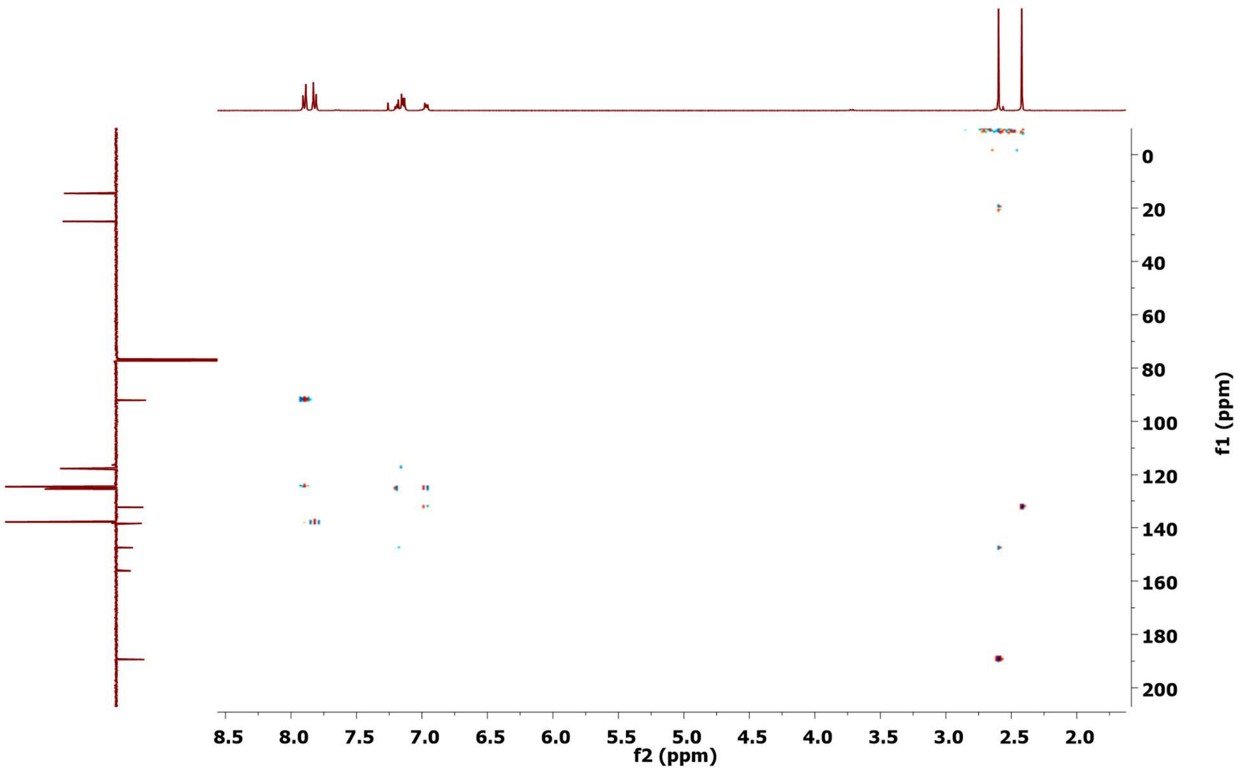

^{1}H NMR ($CDCl_3$) spectrum of (Z)-1-(4-(4-iodophenyl)-5-((2-(trifluoromethyl)phenyl)imino)-4,5-dihydro-1,3,4-thiadiazol-2-yl)ethan-1-one

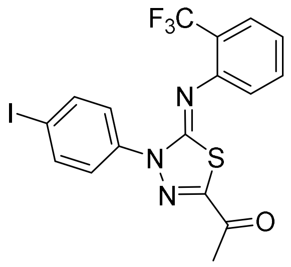

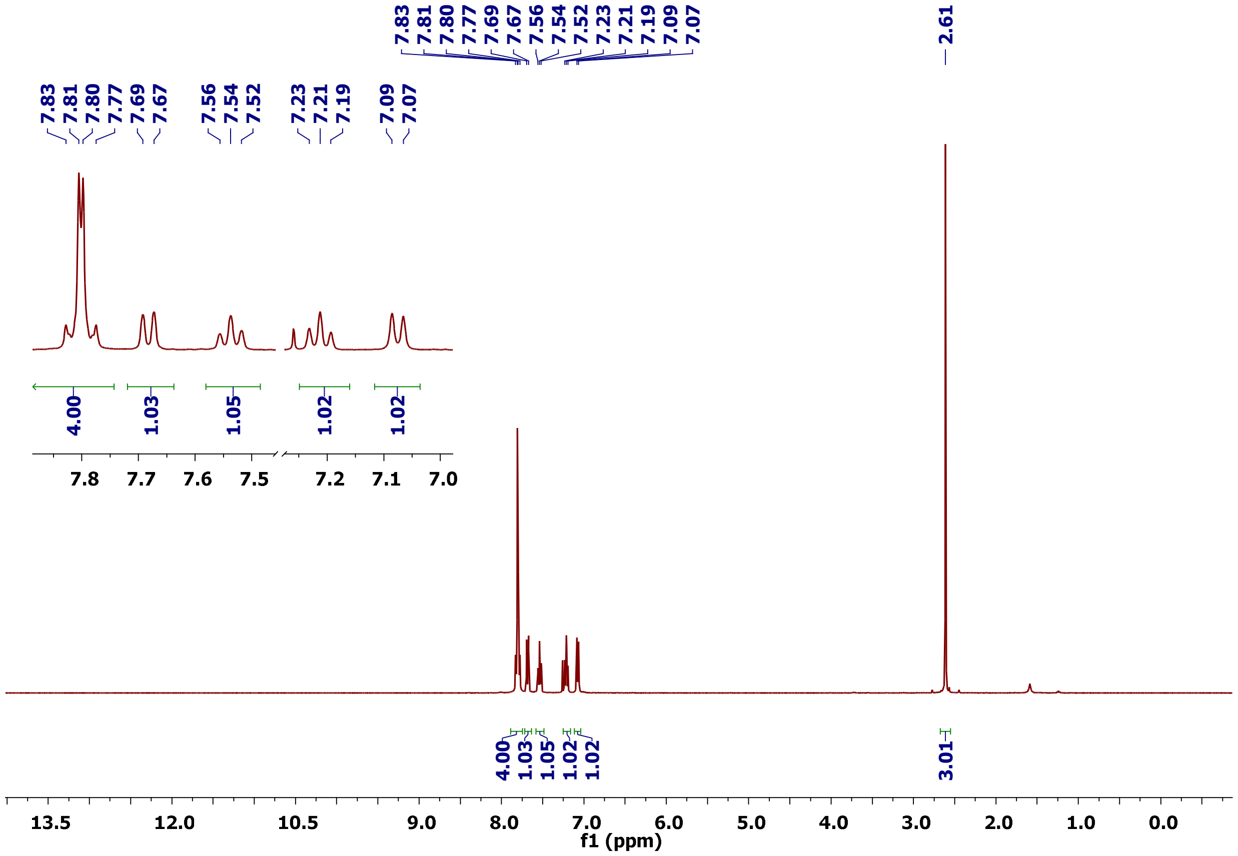

^{13}C NMR ($CDCl_3$) spectrum of (Z)-1-(4-(4-iodophenyl)-5-((2-(trifluoromethyl)phenyl)imino)-4,5-dihydro-1,3,4-thiadiazol-2-yl)ethan-1-one

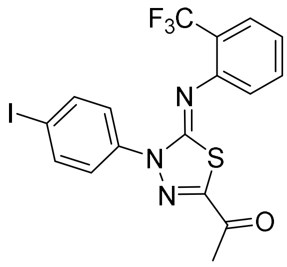

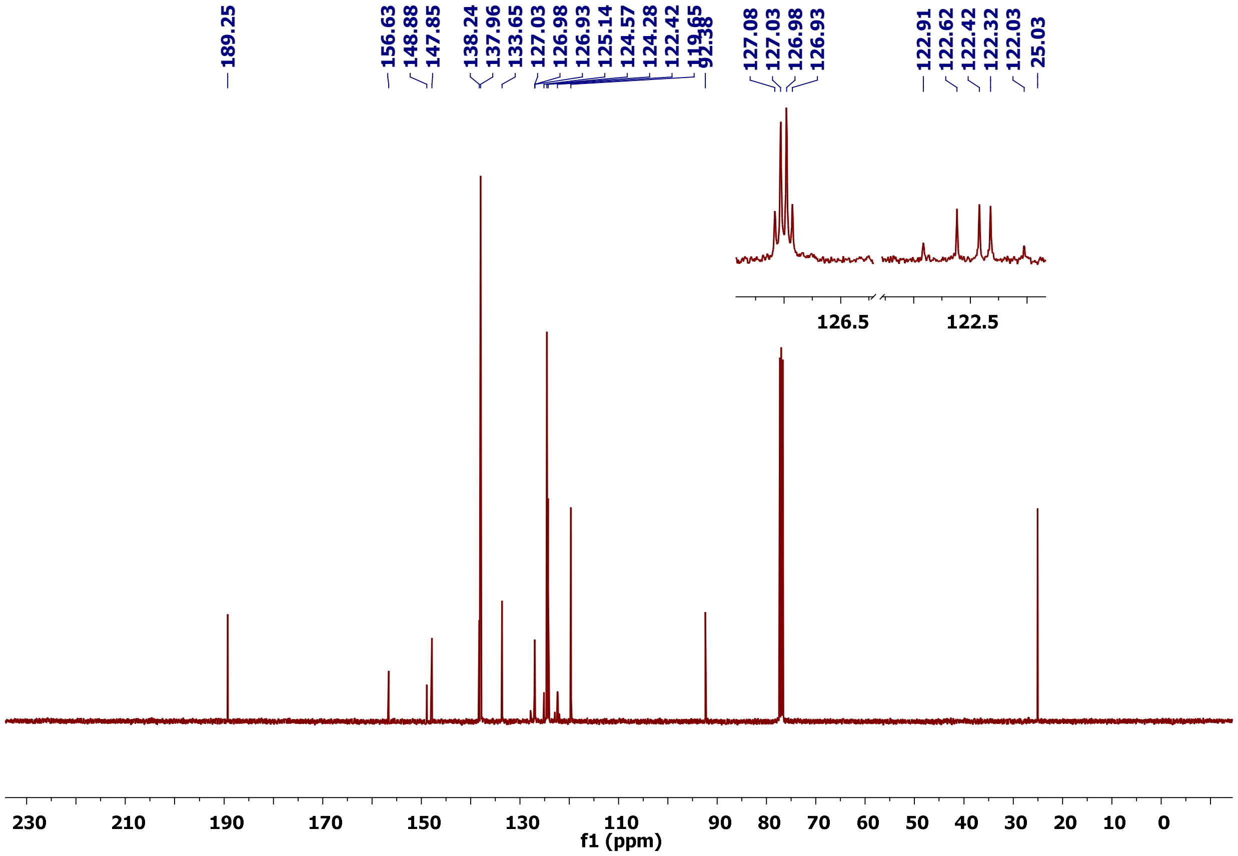

^{13}C CRAPT NMR ($CDCl_3$) spectrum of (Z)-1-(4-(4-iodophenyl)-5-((2-(trifluoromethyl)phenyl)imino)-4,5-dihydro-1,3,4-thiadiazol-2-yl)ethan-1-one

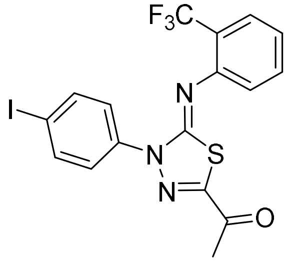

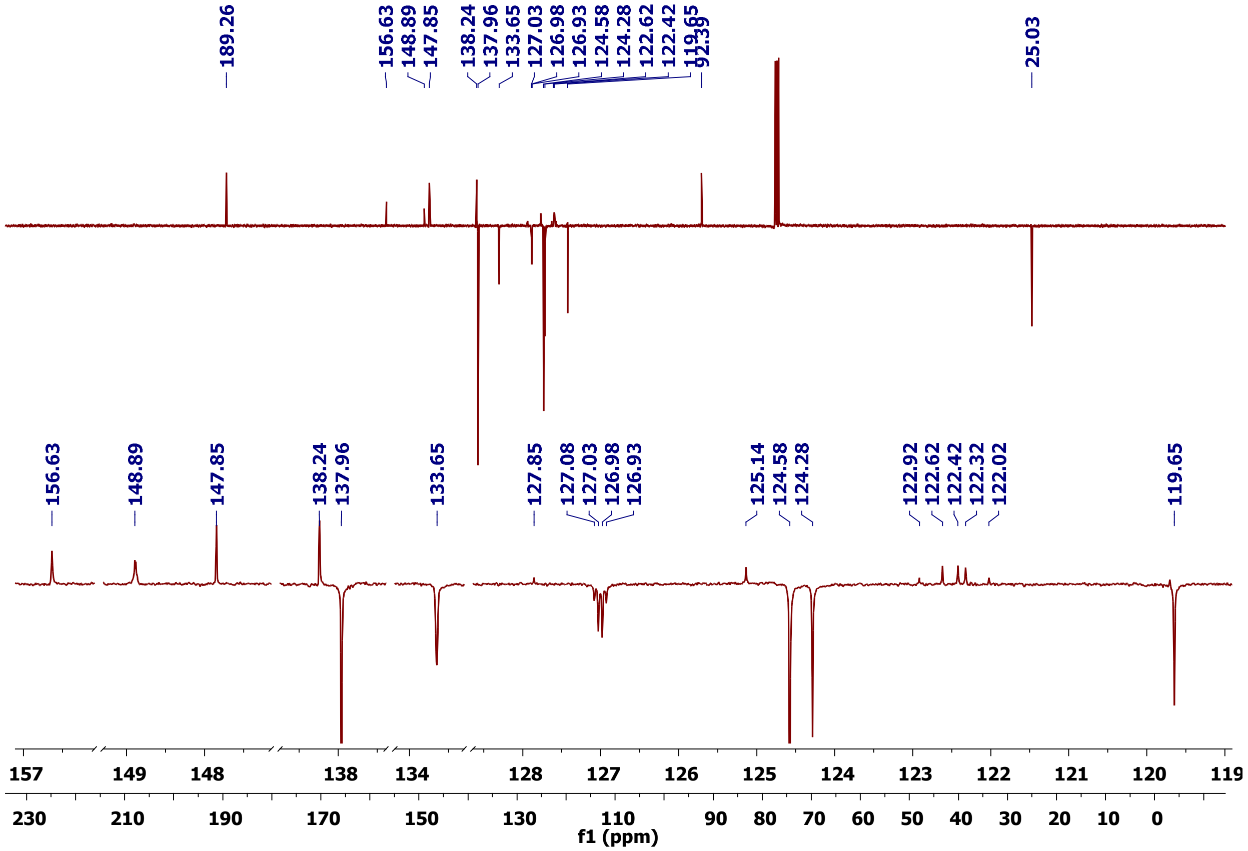

^1H-^1H gDQCOSY NMR ($CDCl_3$) spectrum of (Z)-1-(4-(4-iodophenyl)-5-((2-(trifluoromethyl)phenyl)imino)-4,5-dihydro-1,3,4-thiadiazol-2-yl)ethan-1-one

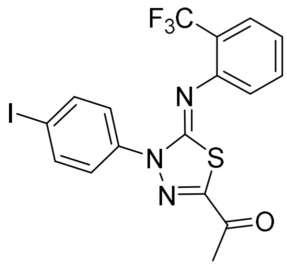

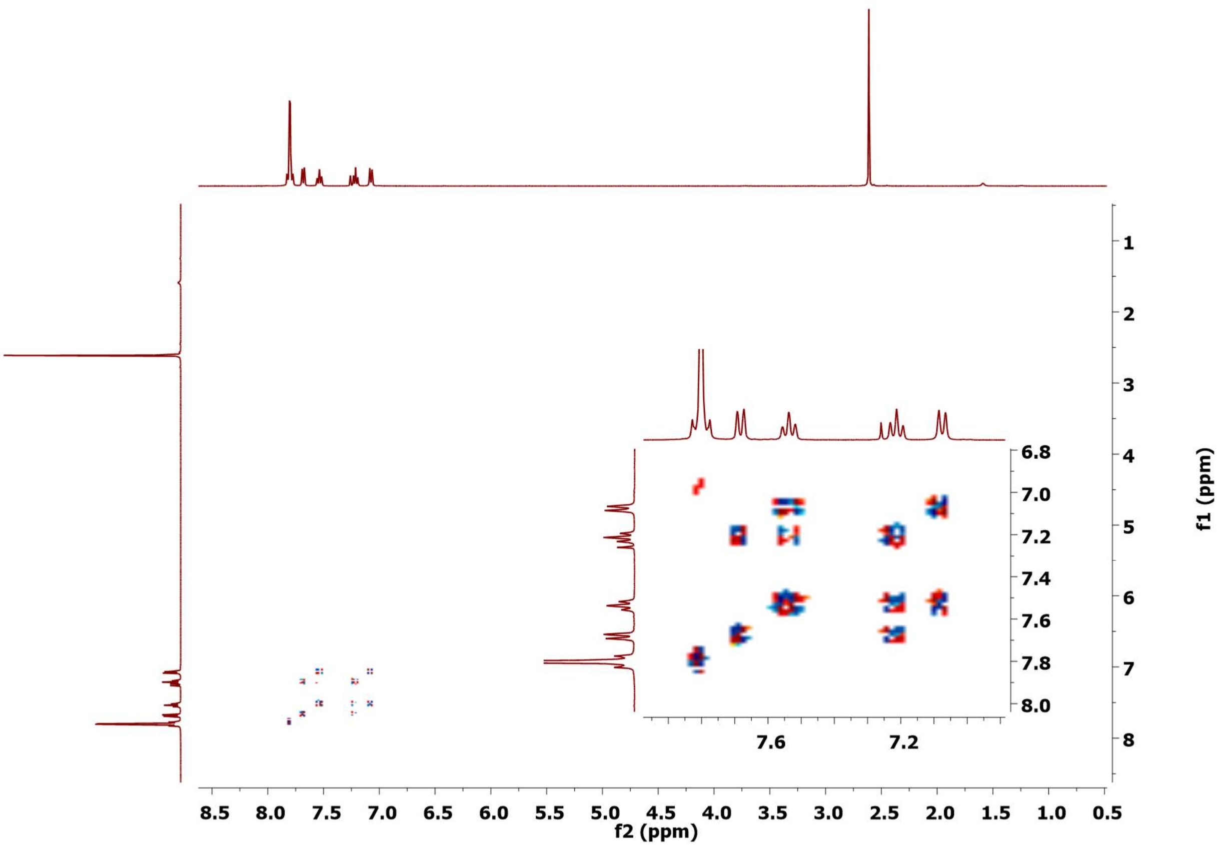

^{1}H-^{13}C-HSQC NMR ($CDCl_3$) spectrum of (Z)-1-(4-(4-iodophenyl)-5-((2-(trifluoromethyl)phenyl)imino)-4,5-dihydro-1,3,4-thiadiazol-2-yl)ethan-1-one

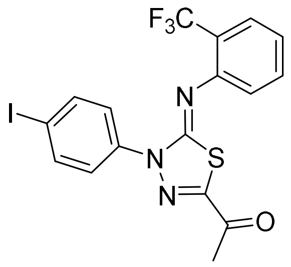

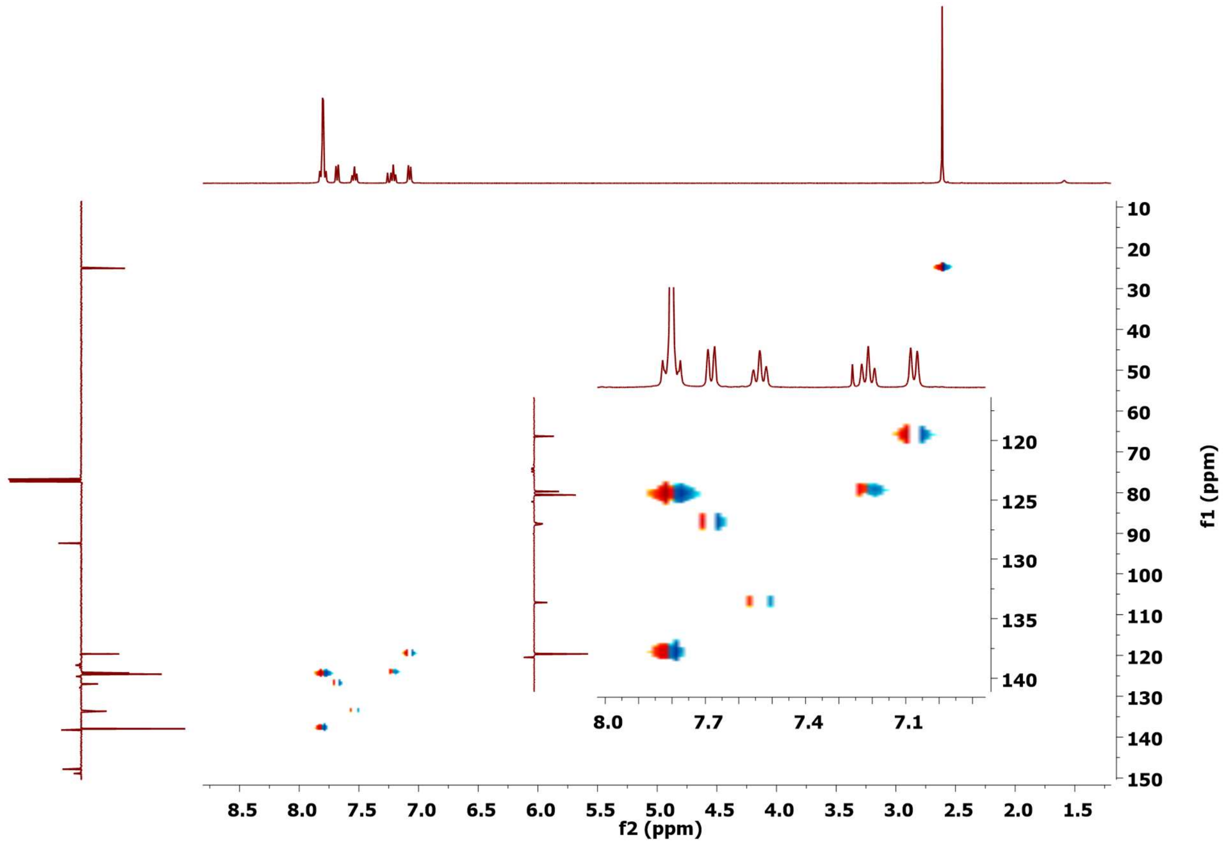

^{1}H-^{13}C-gHMBC NMR ($CDCl_3$) spectrum of (Z)-1-(4-(4-iodophenyl)-5-((2-(trifluoromethyl)phenyl)imino)-4,5-dihydro-1,3,4-thiadiazol-2-yl)ethan-1-one

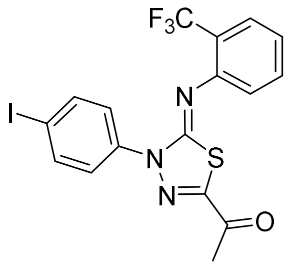

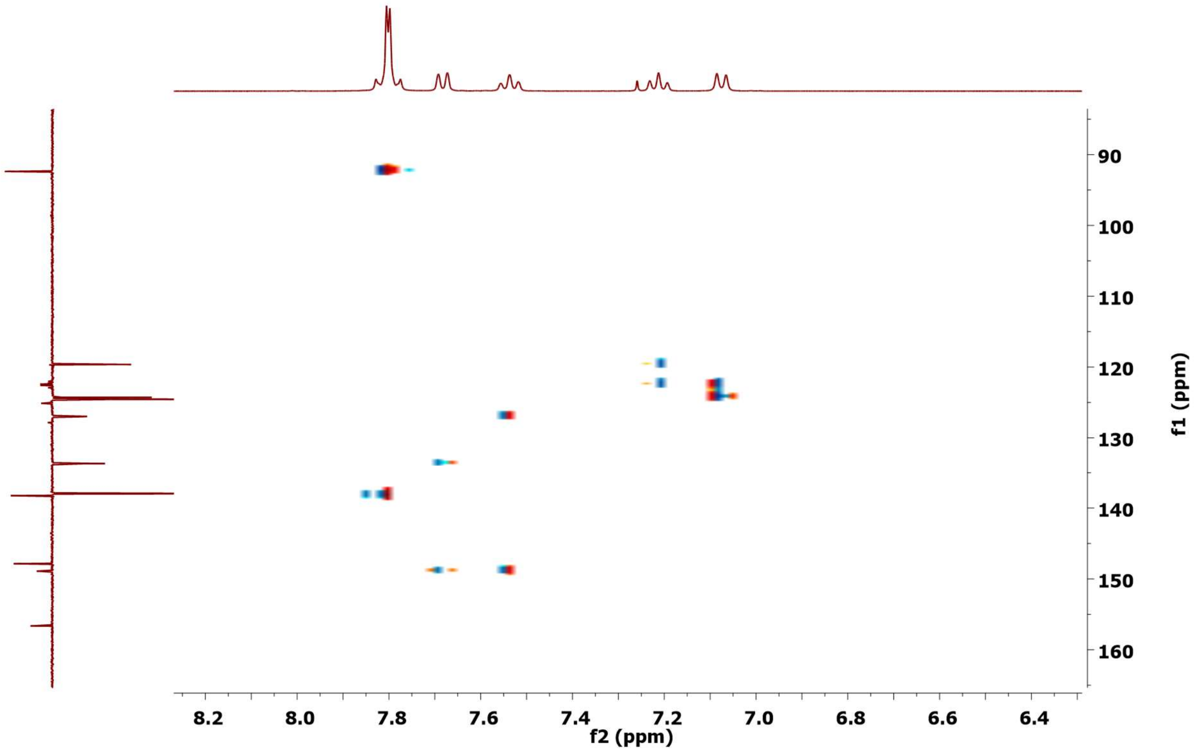

^{1}H NMR ($CDCl_3$) spectrum of (Z)-1-(5-((2,4-dimethylphenyl)imino)-4-(4-iodophenyl)-4,5-dihydro-1,3,4-thiadiazol-2-yl)ethan-1-one

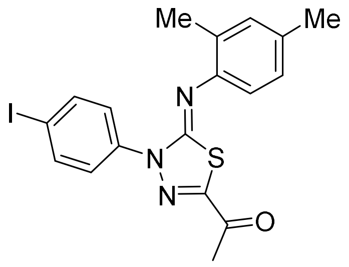

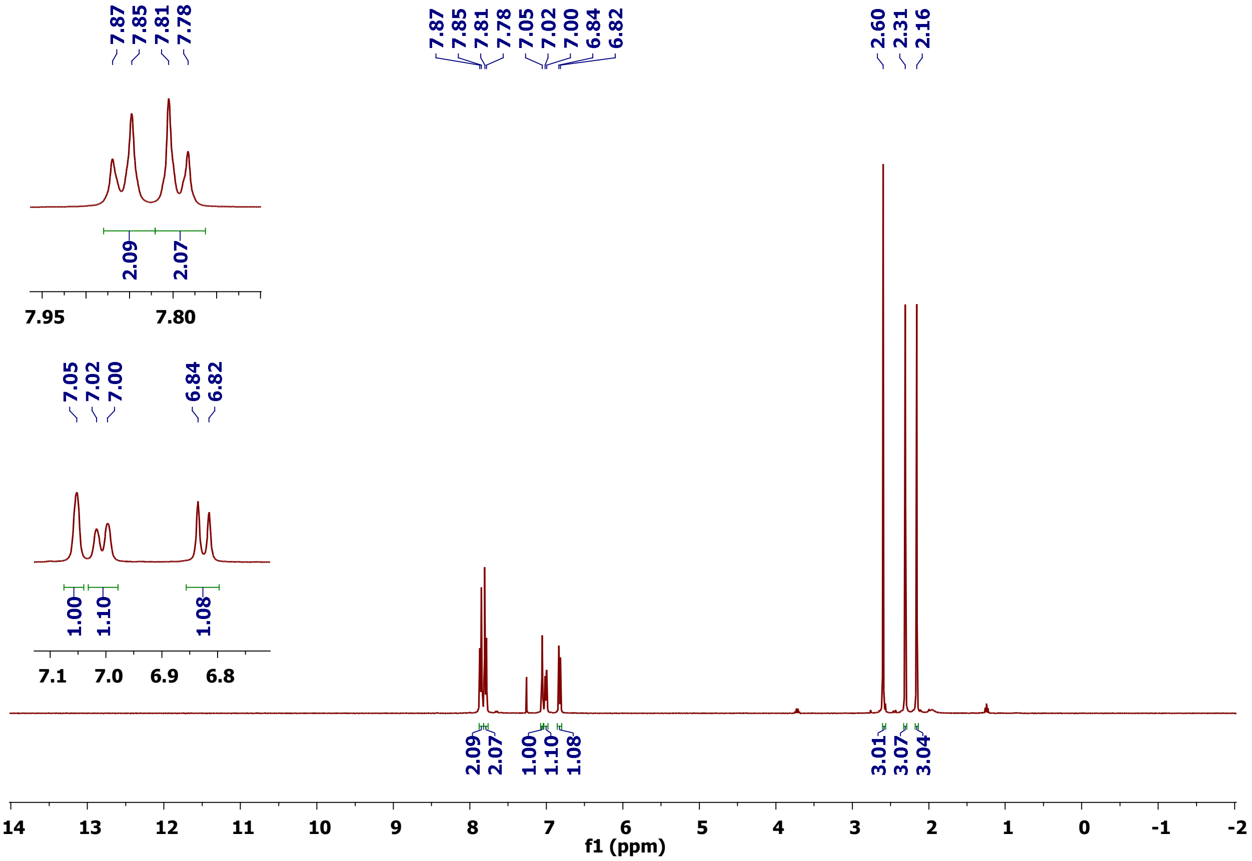

^{13}C NMR ($CDCl_3$) spectrum of (Z)-1-(5-((2,4-dimethylphenyl)imino)-4-(4-iodophenyl)-4,5-dihydro-1,3,4-thiadiazol-2-yl)ethan-1-one

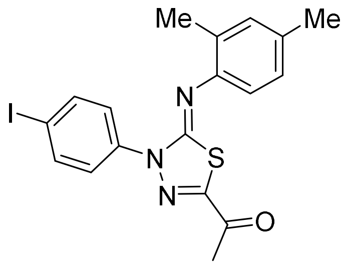

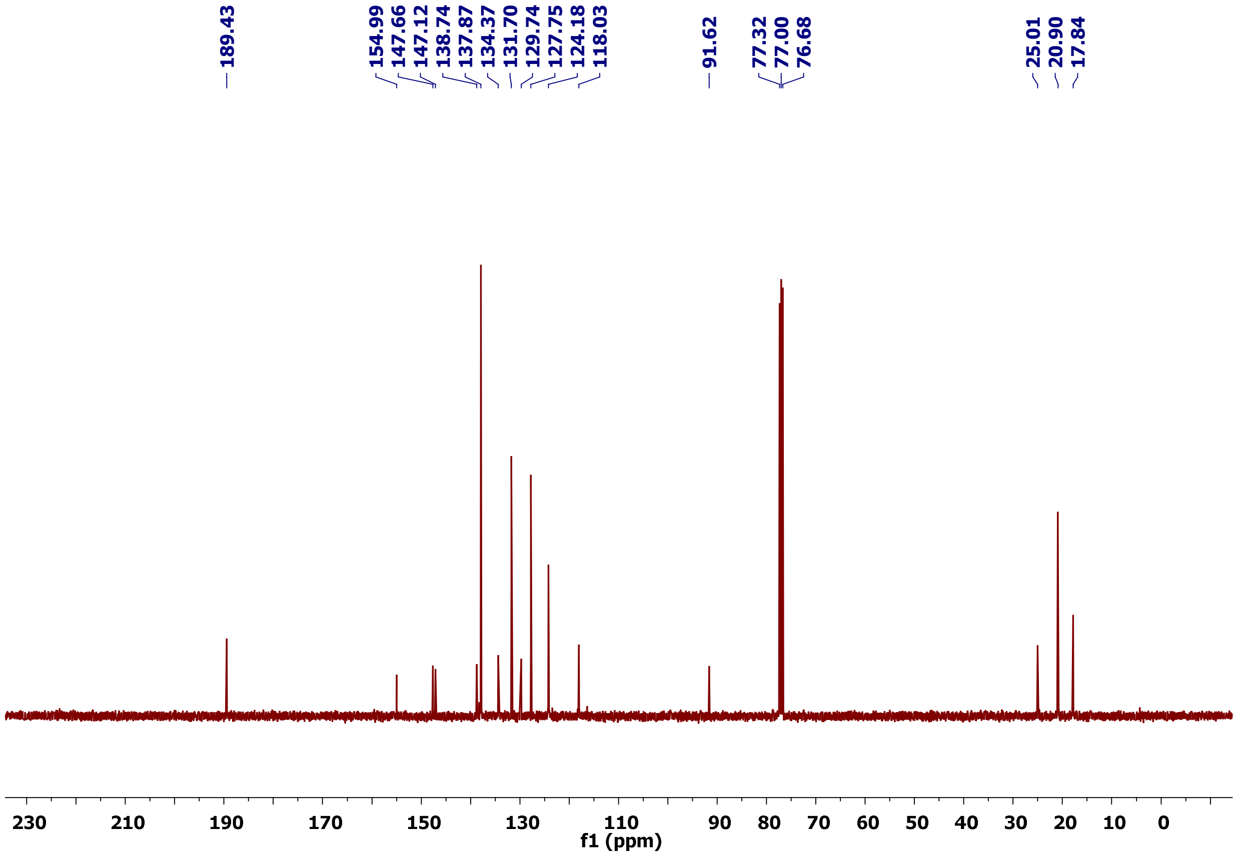

^{13}C CRAPT NMR ($CDCl_3$) spectrum of (Z)-1-(5-((2,4-dimethylphenyl)imino)-4-(4-iodophenyl)-4,5-dihydro-1,3,4-thiadiazol-2-yl)ethan-1-one

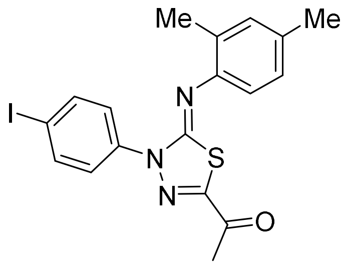

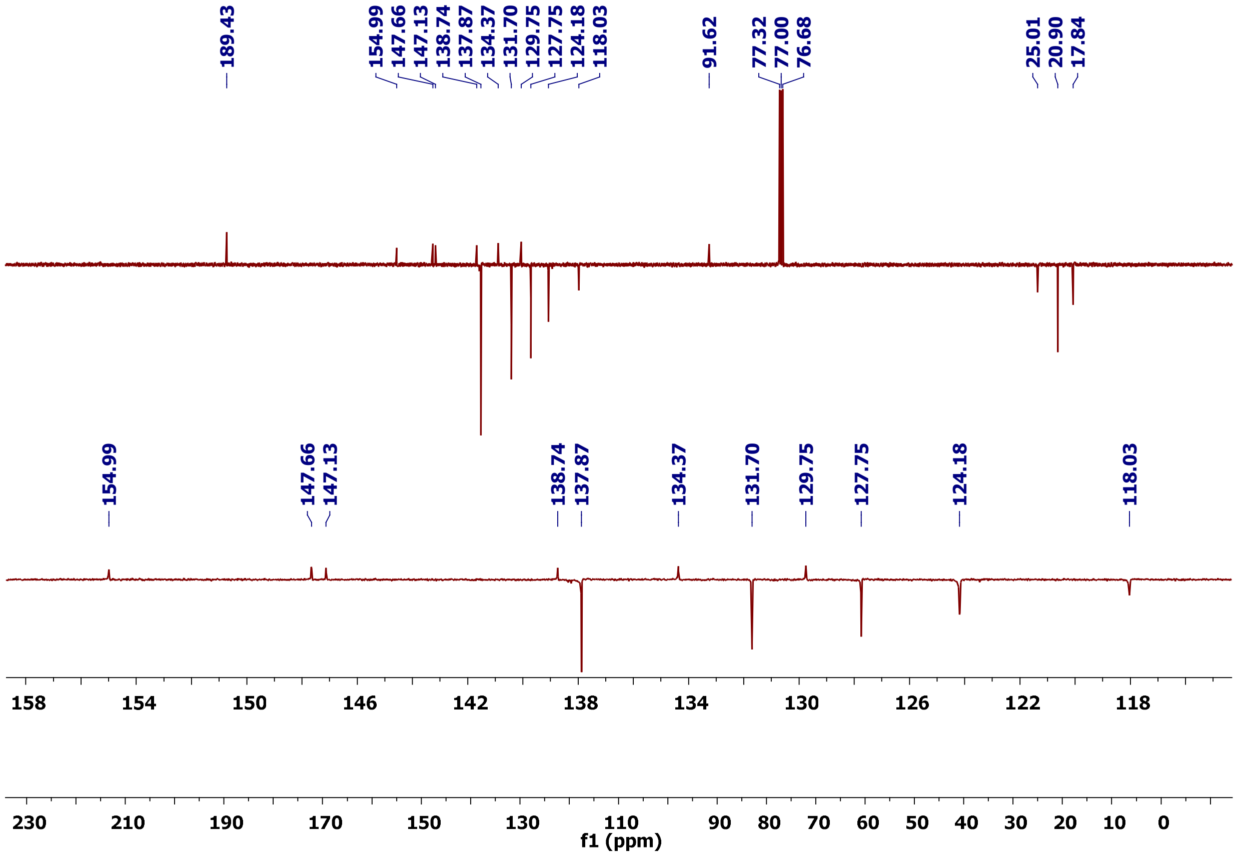

^1H-^1H gDQCOSY NMR ($CDCl_3$) spectrum of (Z)-1-(5-((2,4-dimethylphenyl)imino)-4-(4-iodophenyl)-4,5-dihydro-1,3,4-thiadiazol-2-yl)ethan-1-one

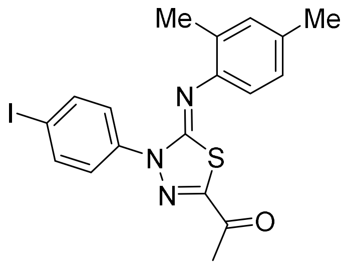

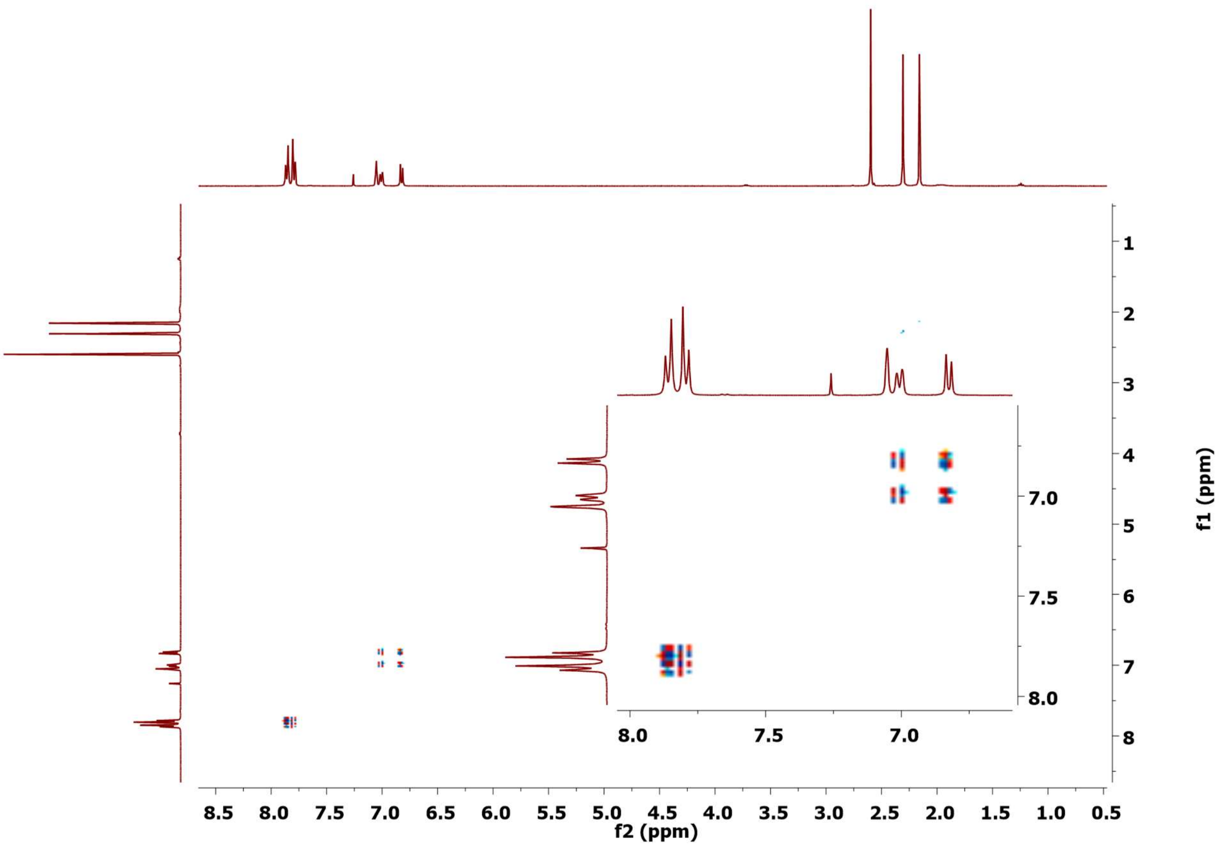

^1H-^{13}C-HSQC NMR ($CDCl_3$) spectrum of (Z)-1-(5-((2,4-dimethylphenyl)imino)-4-(4-iodophenyl)-4,5-dihydro-1,3,4-thiadiazol-2-yl)ethan-1-one

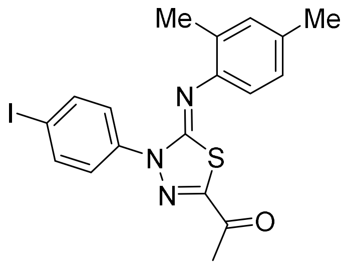

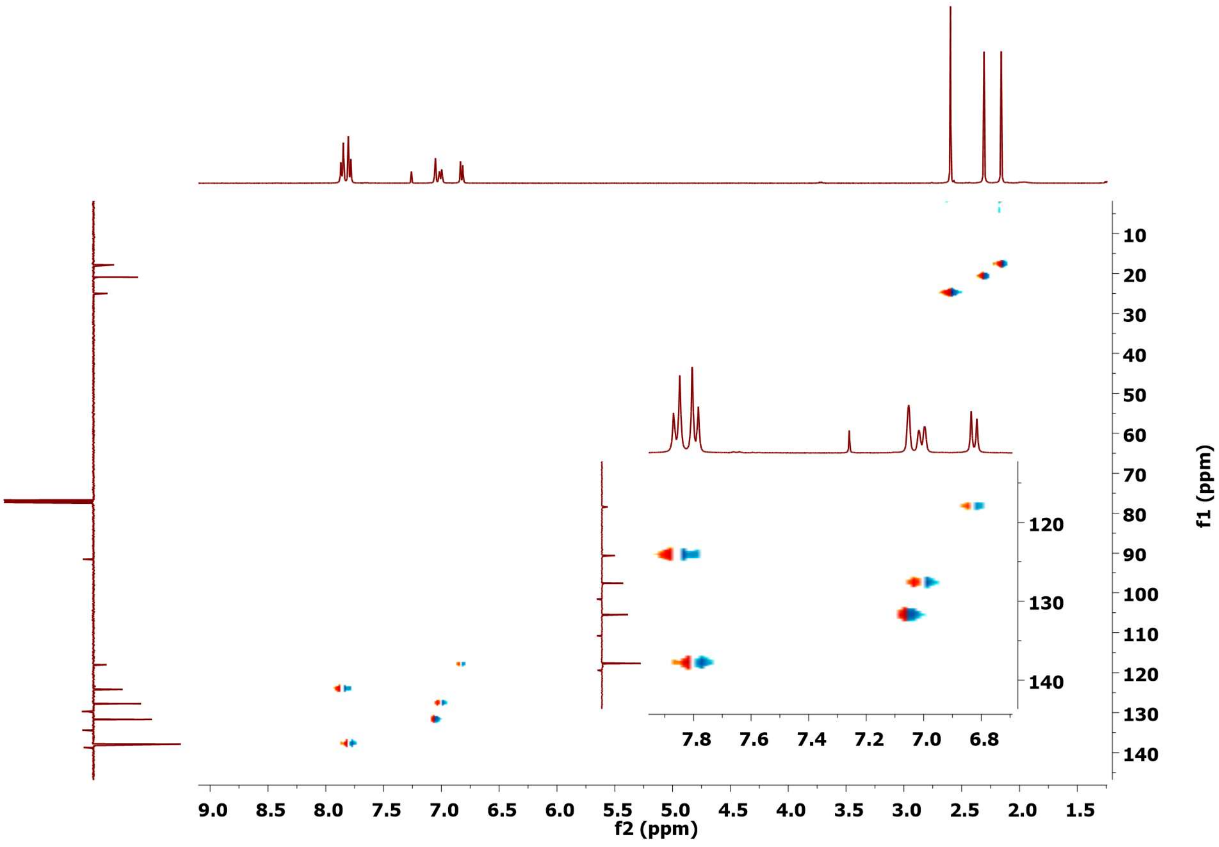

^{1}H-^{13}C-gHMBC NMR ($CDCl_3$) spectrum of (Z)-1-(5-((2,4-dimethylphenyl)imino)-4-(4-iodophenyl)-4,5-dihydro-1,3,4-thiadiazol-2-yl)ethan-1-one

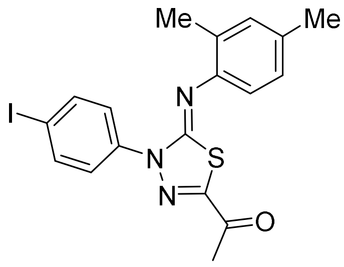

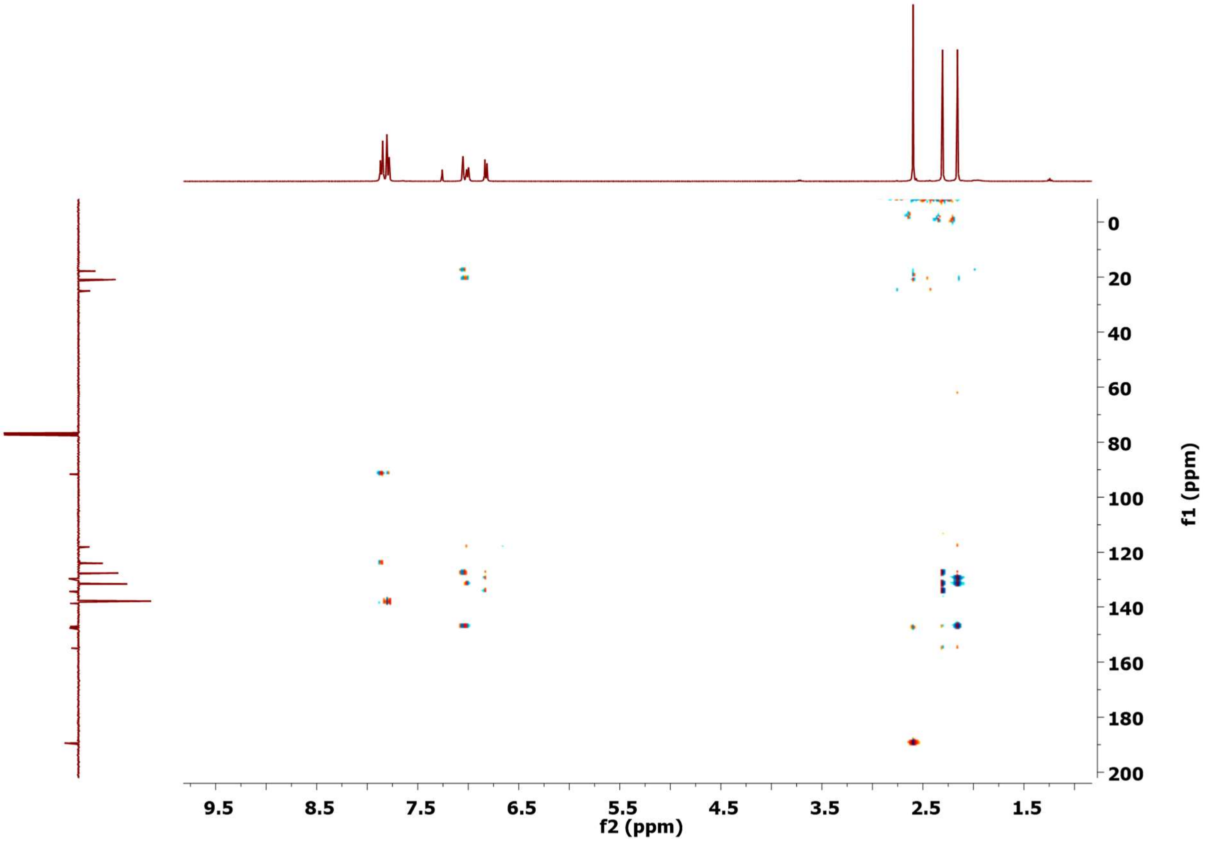

^{1}H NMR ($CDCl_3$) spectrum of (Z)-1-(3-((5-acetyl-3-(4-bromophenyl)-1,3,4-thiadiazol-2(3H)-ylidene)amino)phenyl)ethan-1-one

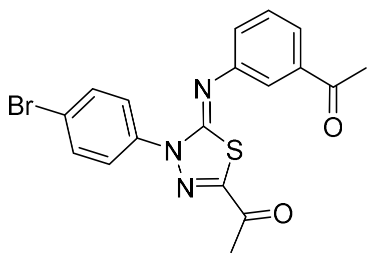

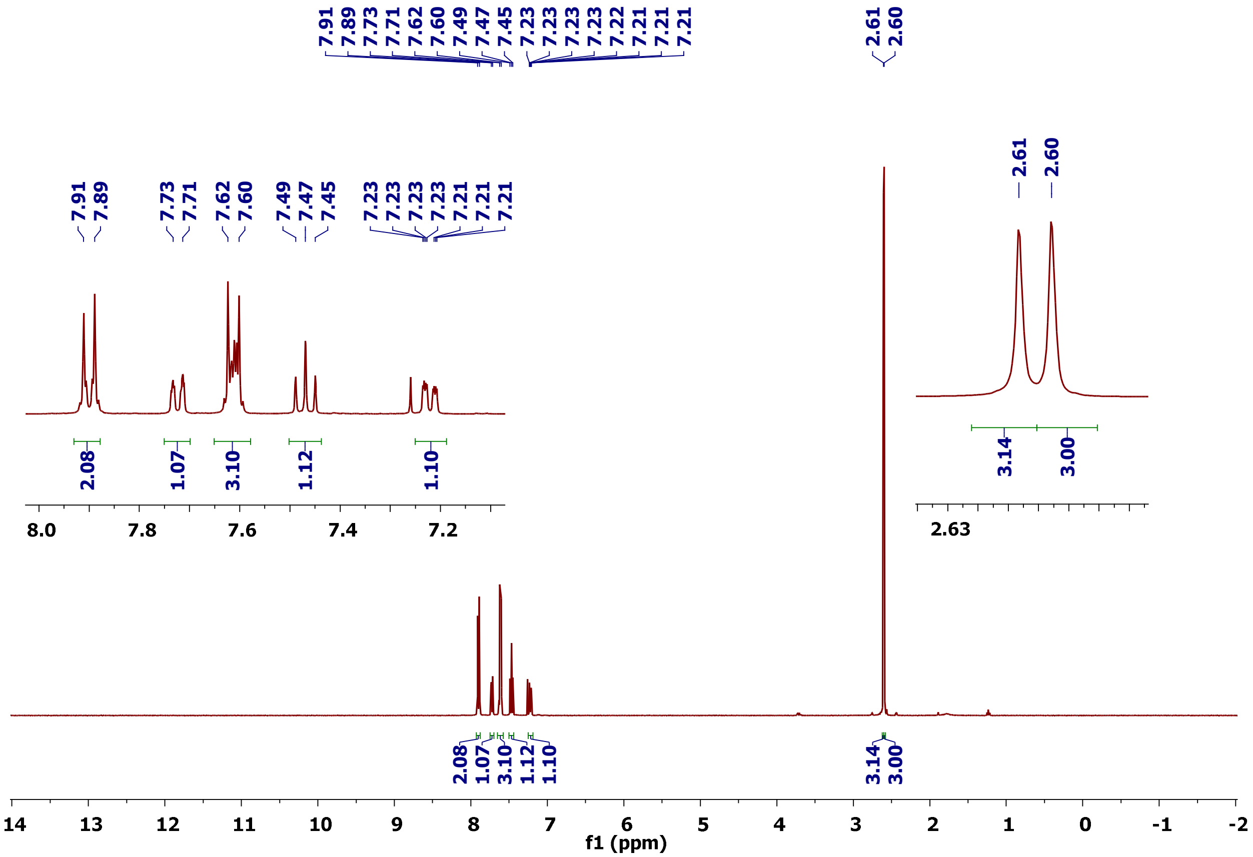

^{13}C NMR ($CDCl_3$) spectrum of (Z)-1-(3-((5-acetyl-3-(4-bromophenyl)-1,3,4-thiadiazol-2(3H)-ylidene)amino)phenyl)ethan-1-one

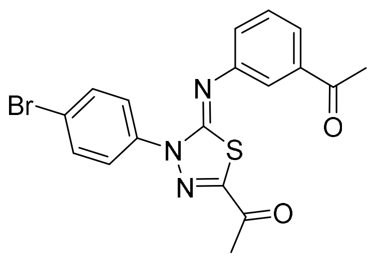

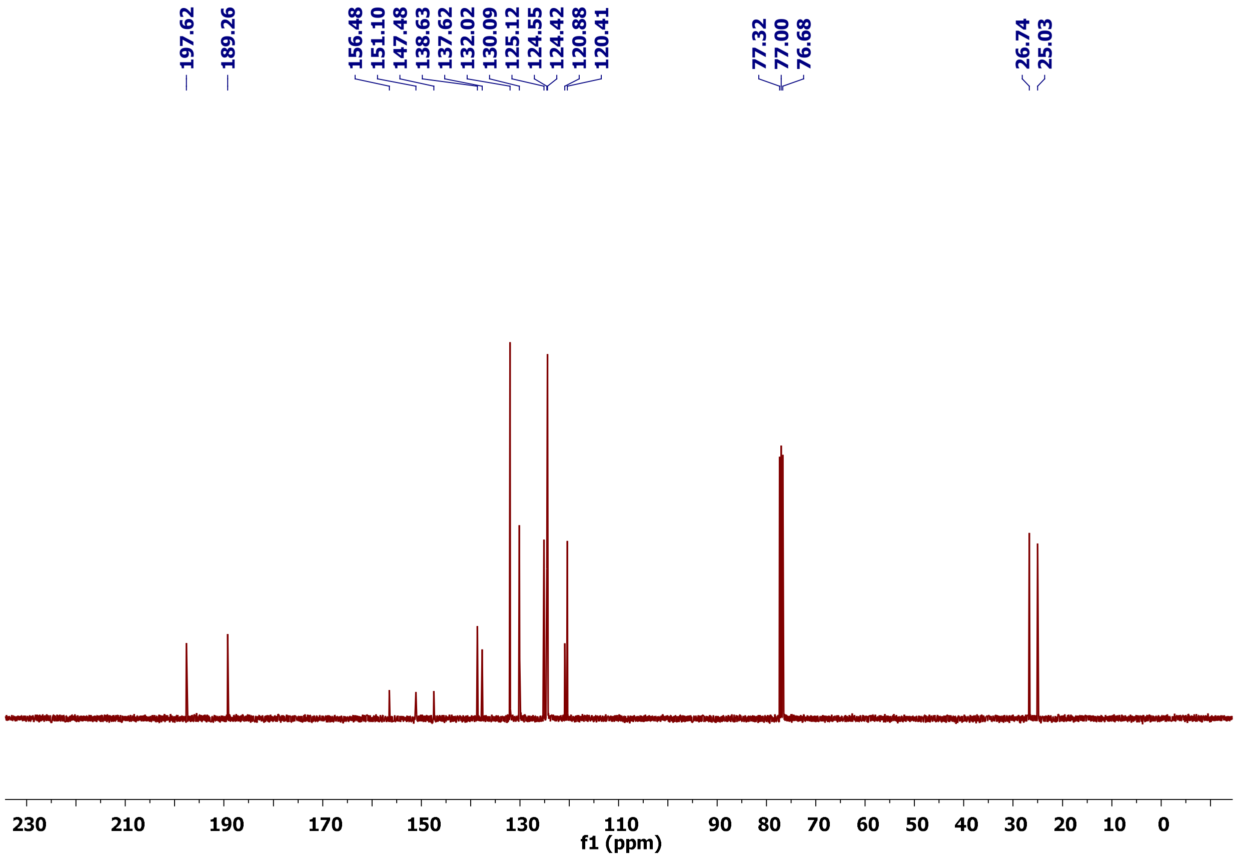

^{13}C CRAPT NMR ($CDCl_3$) spectrum of (Z)-1-(3-((5-acetyl-3-(4-bromophenyl)-1,3,4-thiadiazol-2(3H)-ylidene)amino)phenyl)ethan-1-one

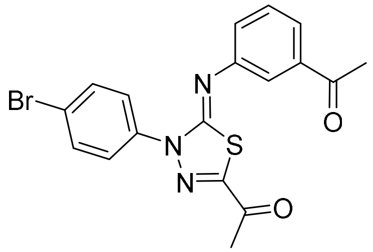

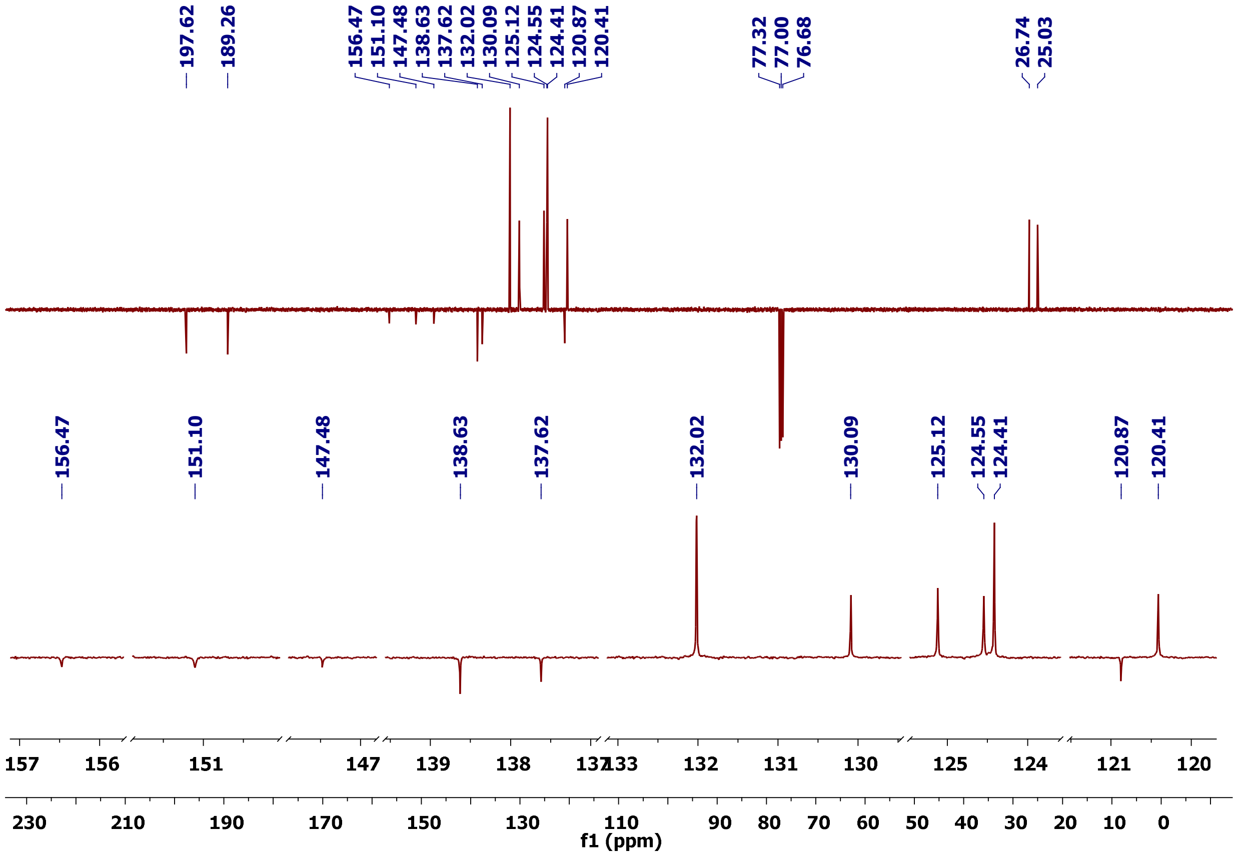

^{1}H-^{1}H gDQCOSY NMR ($CDCl_3$) spectrum of (Z)-1-(3-((5-acetyl-3-(4-bromophenyl)-1,3,4-thiadiazol-2(3H)-ylidene)amino)phenyl)ethan-1-one

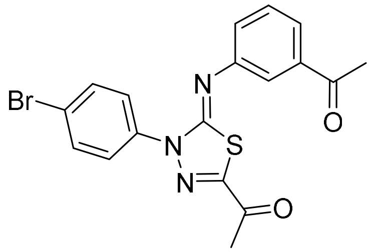

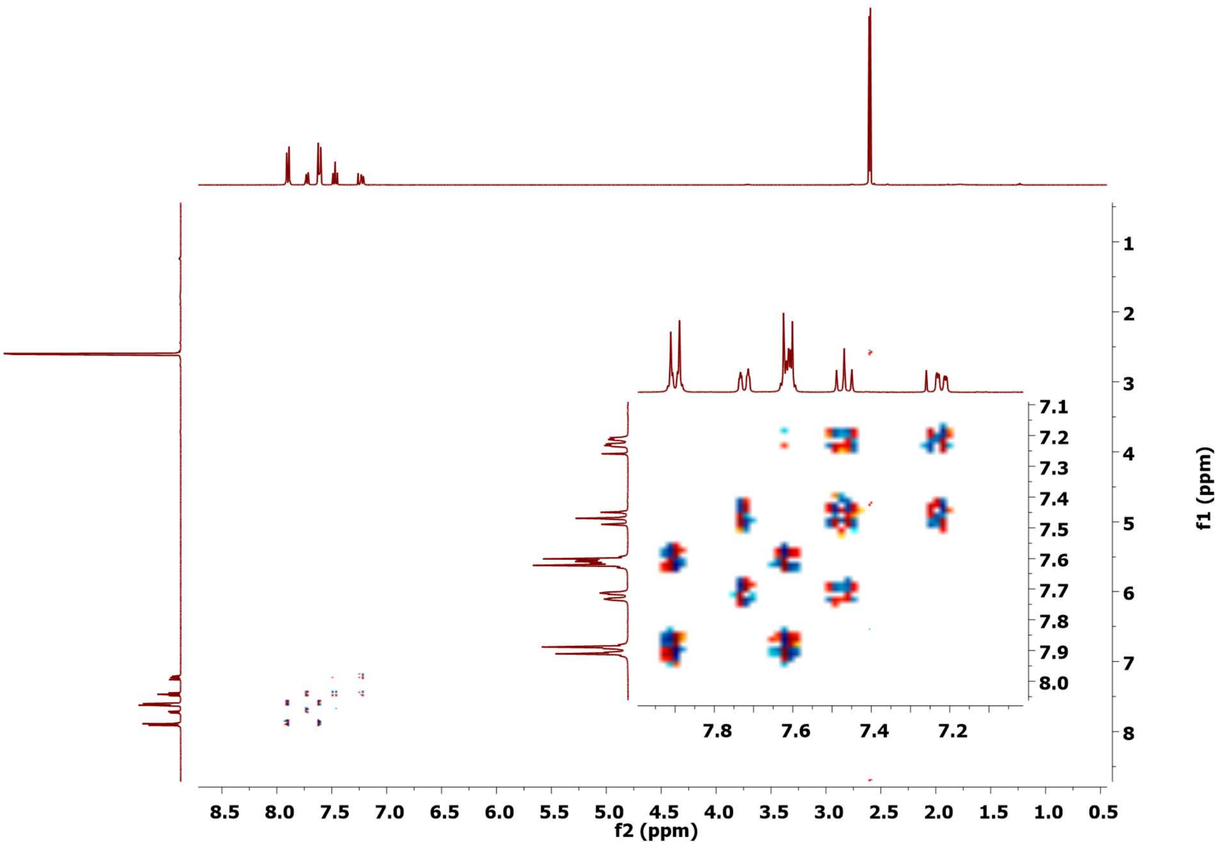

^1H-^{13}C-HSQC NMR ($CDCl_3$) spectrum of (Z)-1-(3-((5-acetyl-3-(4-bromophenyl)-1,3,4-thiadiazol-2(3H)-ylidene)amino)phenyl)ethan-1-one

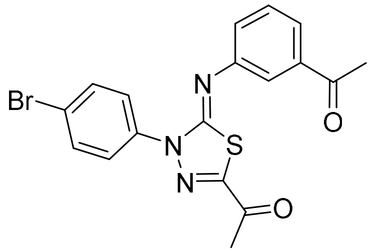

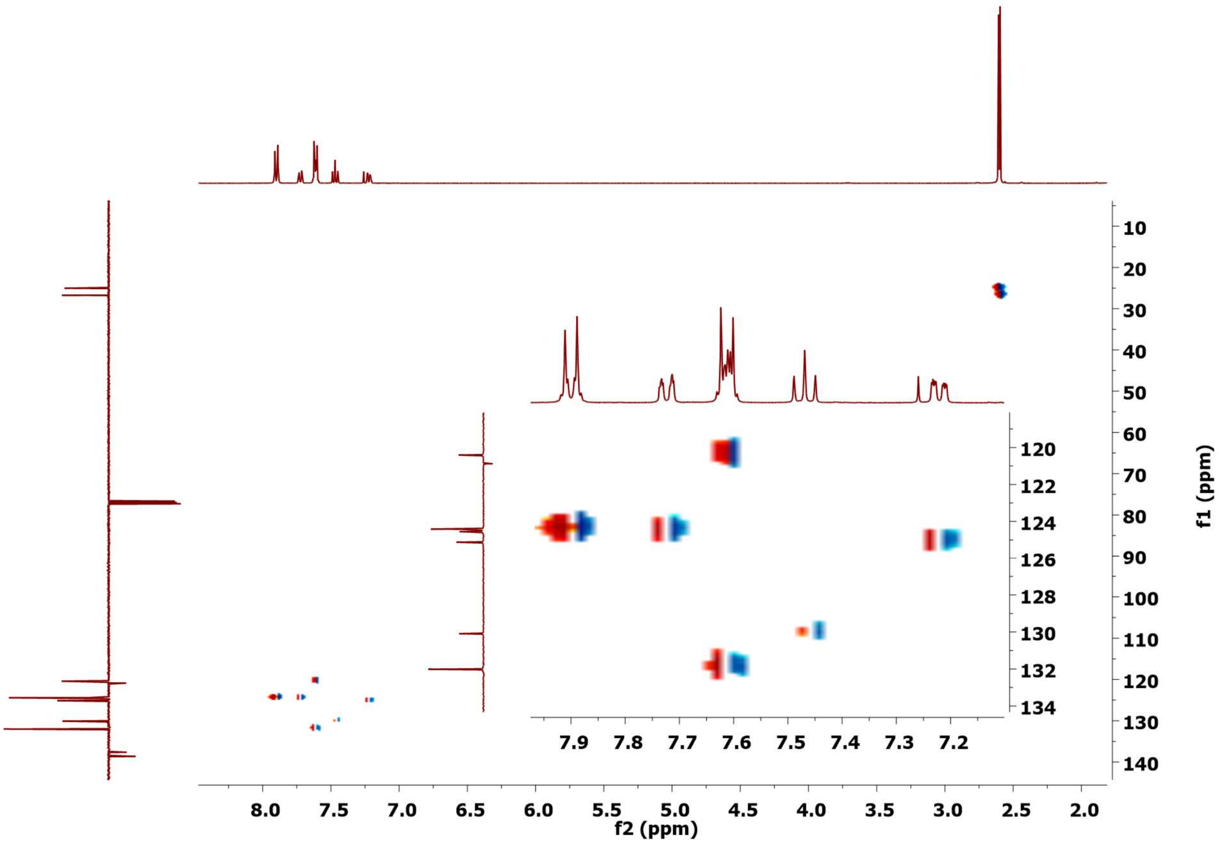

^{1}H-^{13}C-gHMBC NMR ($CDCl_3$) spectrum of (Z)-1-(3-((5-acetyl-3-(4-bromophenyl)-1,3,4-thiadiazol-2(3H)-ylidene)amino)phenyl)ethan-1-one

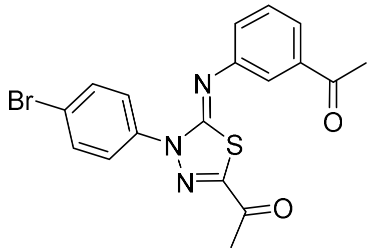

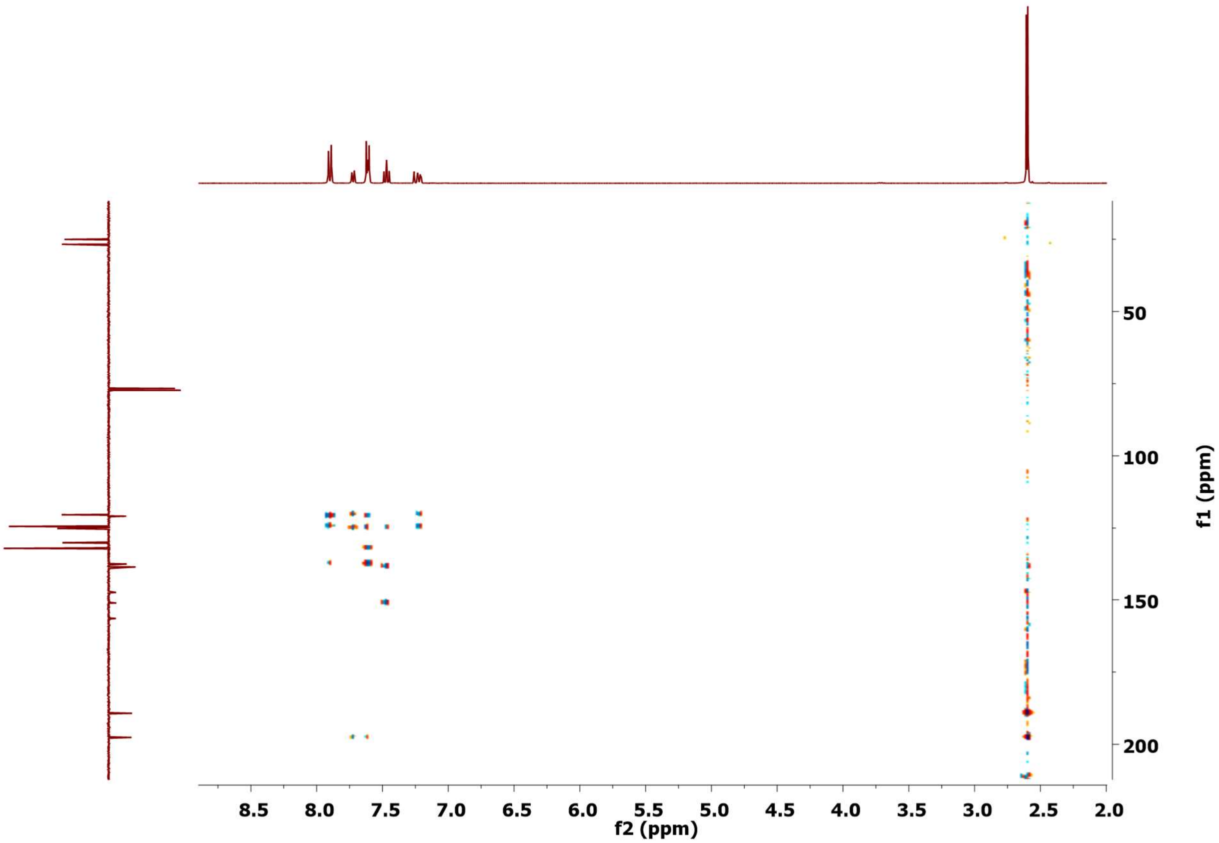

^{1}H NMR ($CDCl_3$) spectrum of (Z)-1-(5-((2,4-dichlorophenyl)imino)-4-(4-iodophenyl)-4,5-dihydro-1,3,4-thiadiazol-2-yl)ethan-1-one

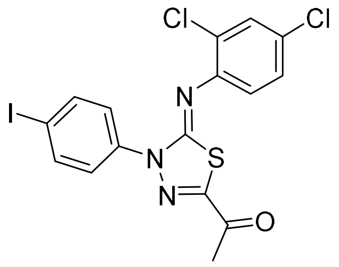

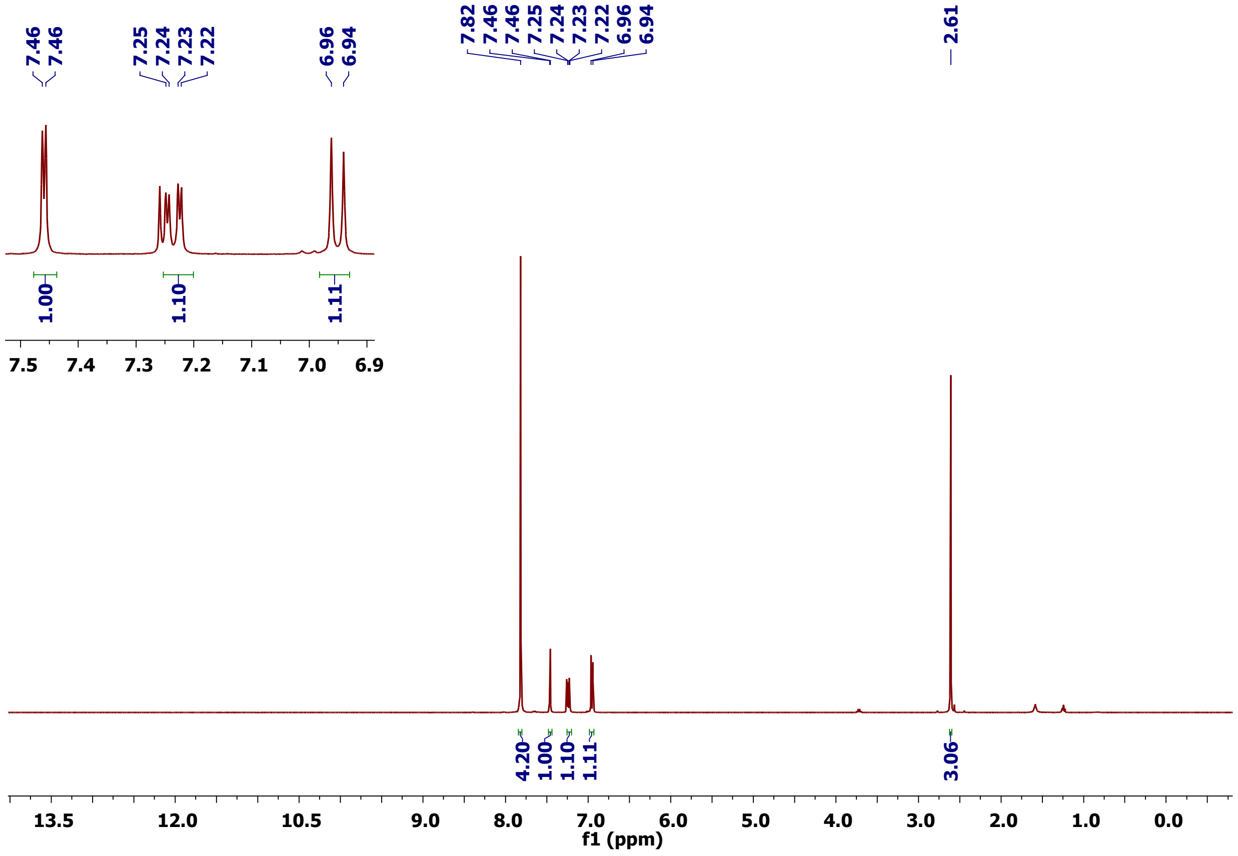

^{13}C NMR ($CDCl_3$) spectrum of (Z)-1-(5-((2,4-dichlorophenyl)imino)-4-(4-iodophenyl)-4,5-dihydro-1,3,4-thiadiazol-2-yl)ethan-1-one

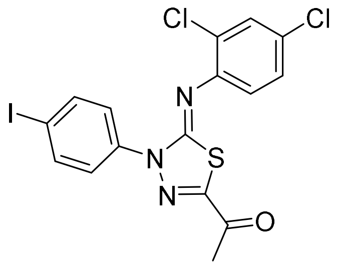

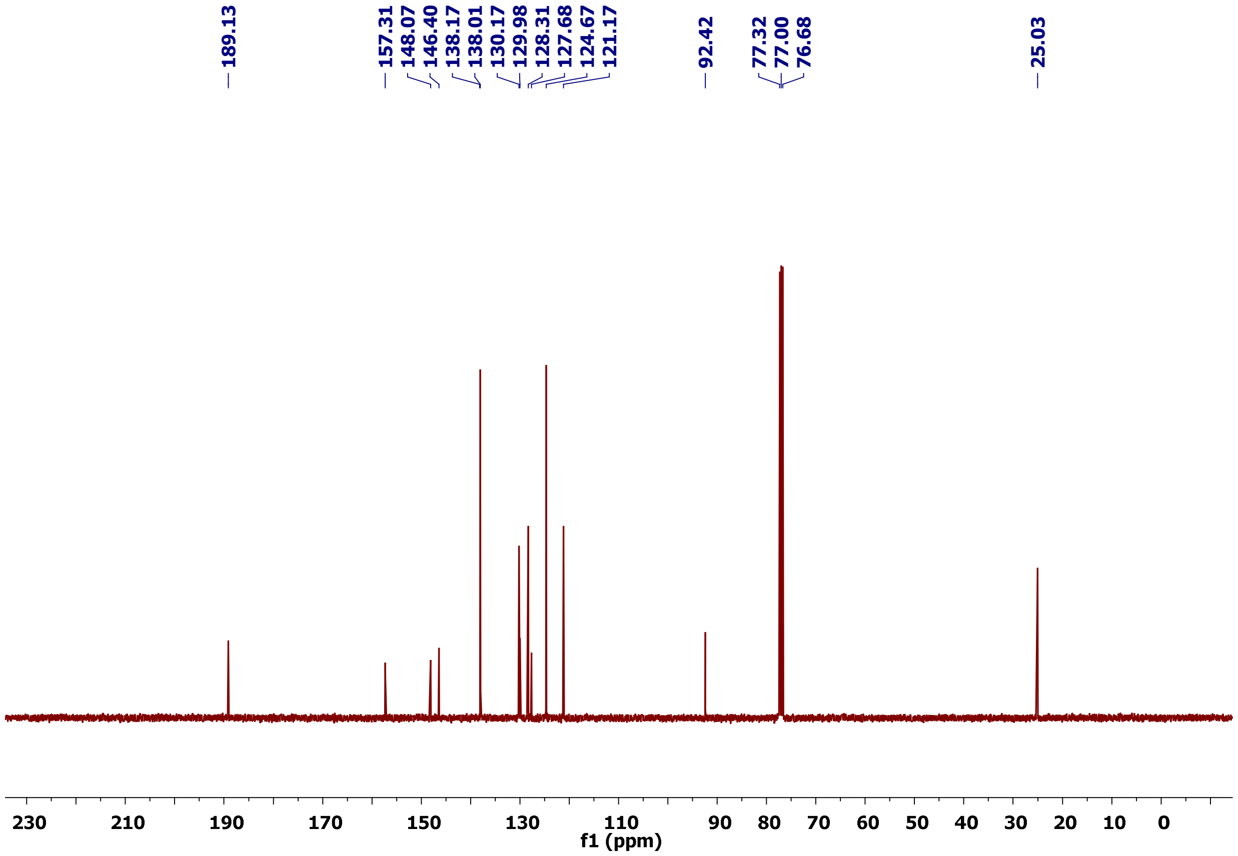

^{13}C CRAPT NMR ($CDCl_3$) spectrum of (Z)-1-(5-((2,4-dichlorophenyl)imino)-4-(4-iodophenyl)-4,5-dihydro-1,3,4-thiadiazol-2-yl)ethan-1-one

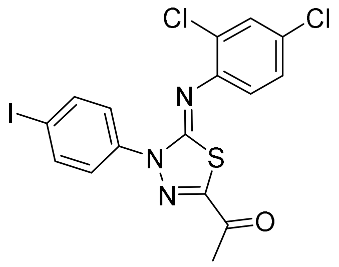

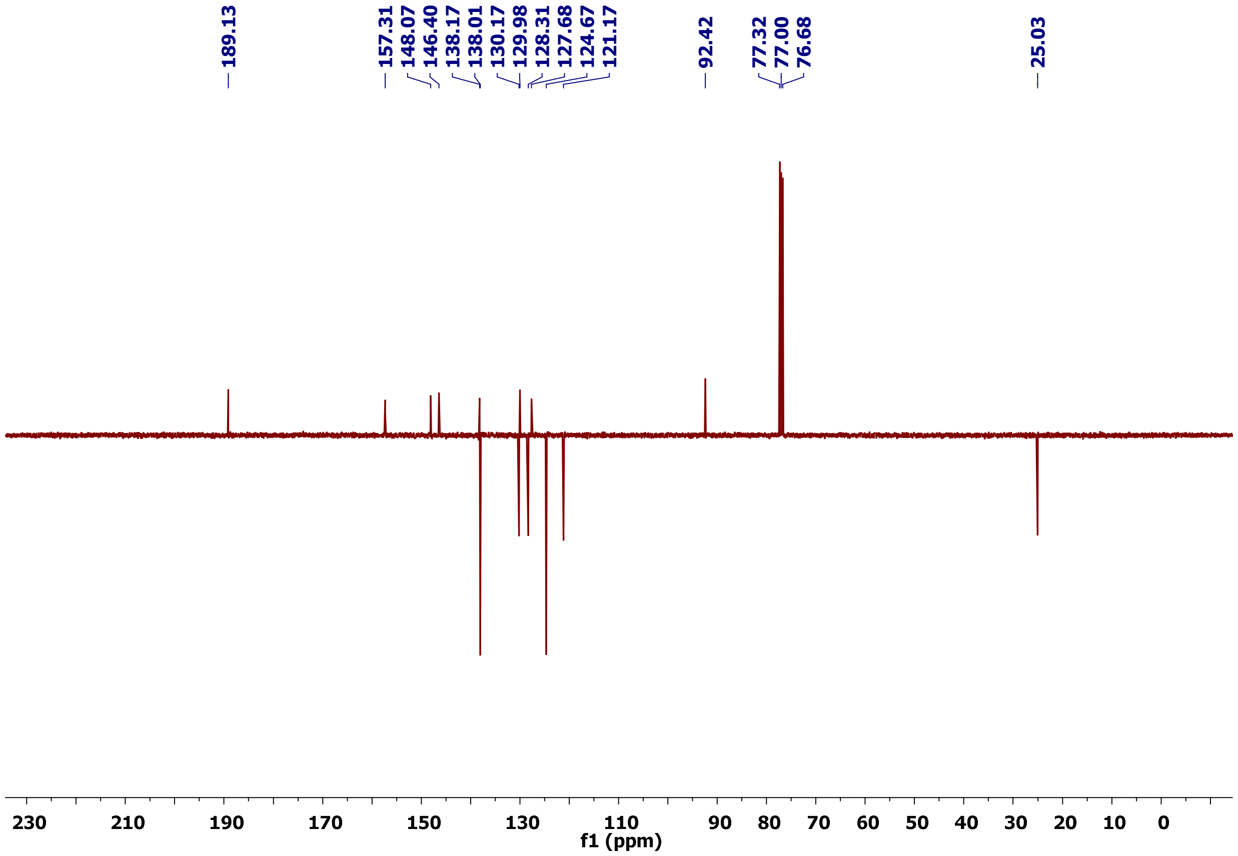

^{1}H-^{1}H gDQCOSY NMR ($CDCl_3$) spectrum of (Z)-1-(5-((2,4-dichlorophenyl)imino)-4-(4-iodophenyl)-4,5-dihydro-1,3,4-thiadiazol-2-yl)ethan-1-one

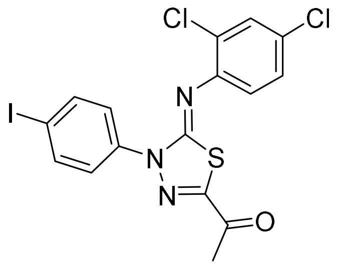

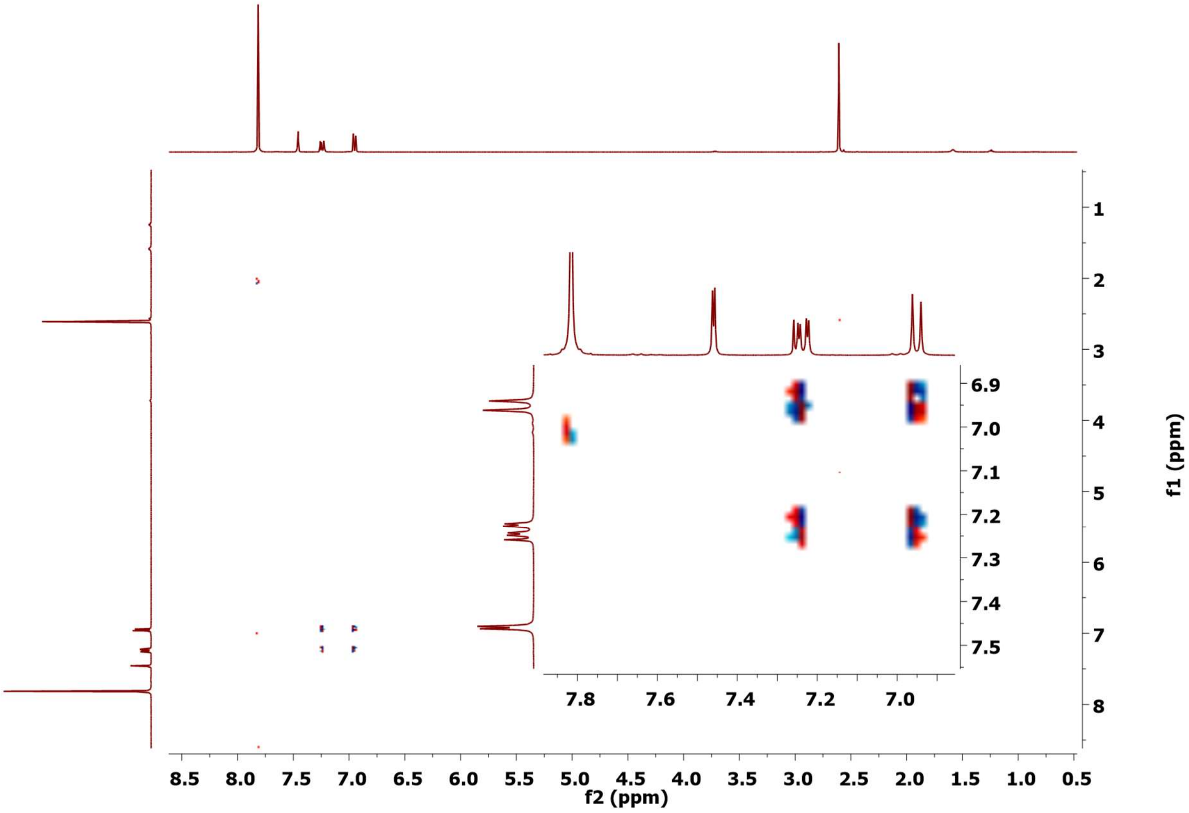

^{1}H-^{13}C-HSQC NMR ($CDCl_3$) spectrum of (Z)-1-(5-((2,4-dichlorophenyl)imino)-4-(4-iodophenyl)-4,5-dihydro-1,3,4-thiadiazol-2-yl)ethan-1-one

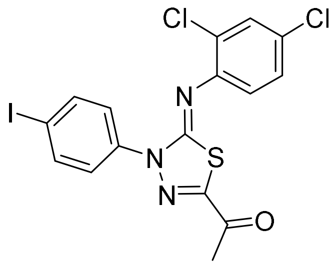

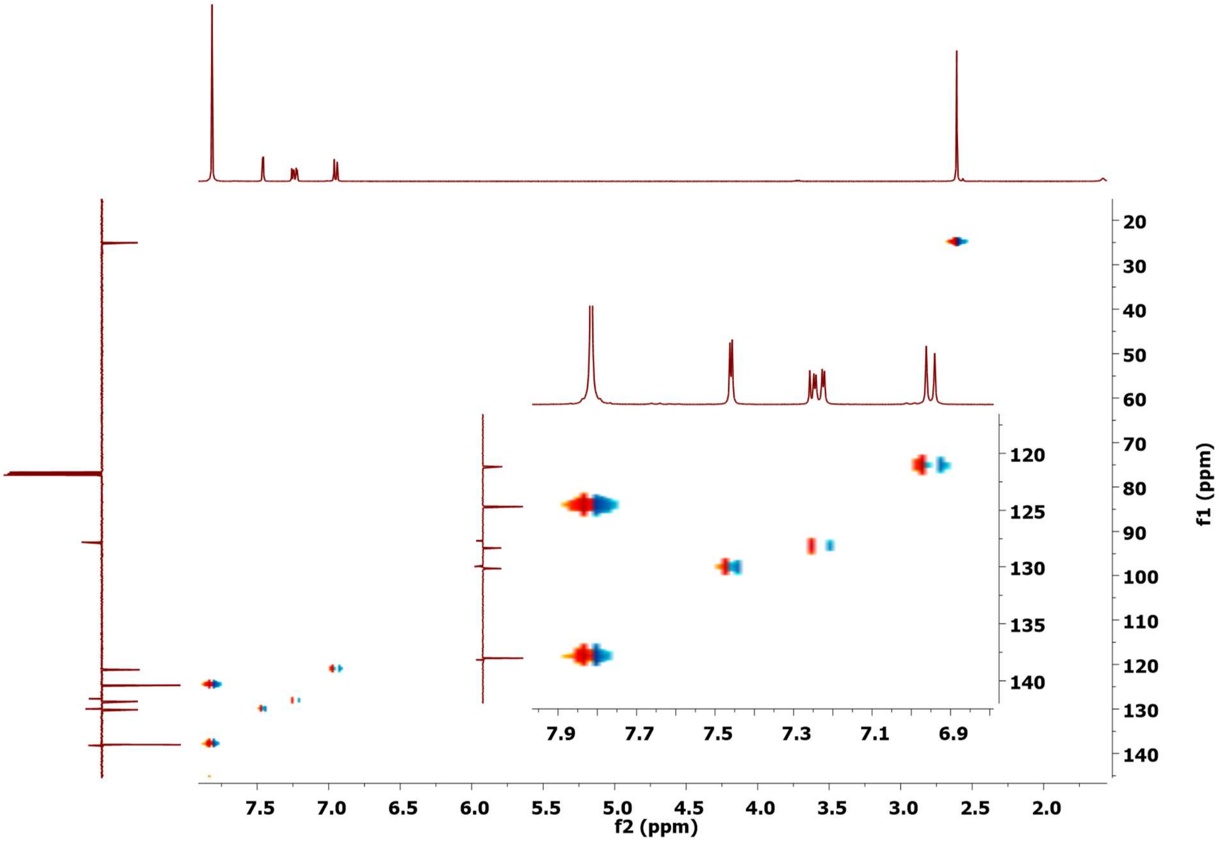

^{1}H-^{13}C-gHMBC NMR ($CDCl_3$) spectrum of (Z)-1-(5-((2,4-dichlorophenyl)imino)-4-(4-iodophenyl)-4,5-dihydro-1,3,4-thiadiazol-2-yl)ethan-1-one

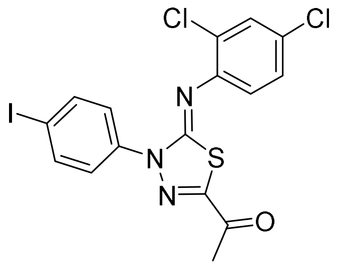

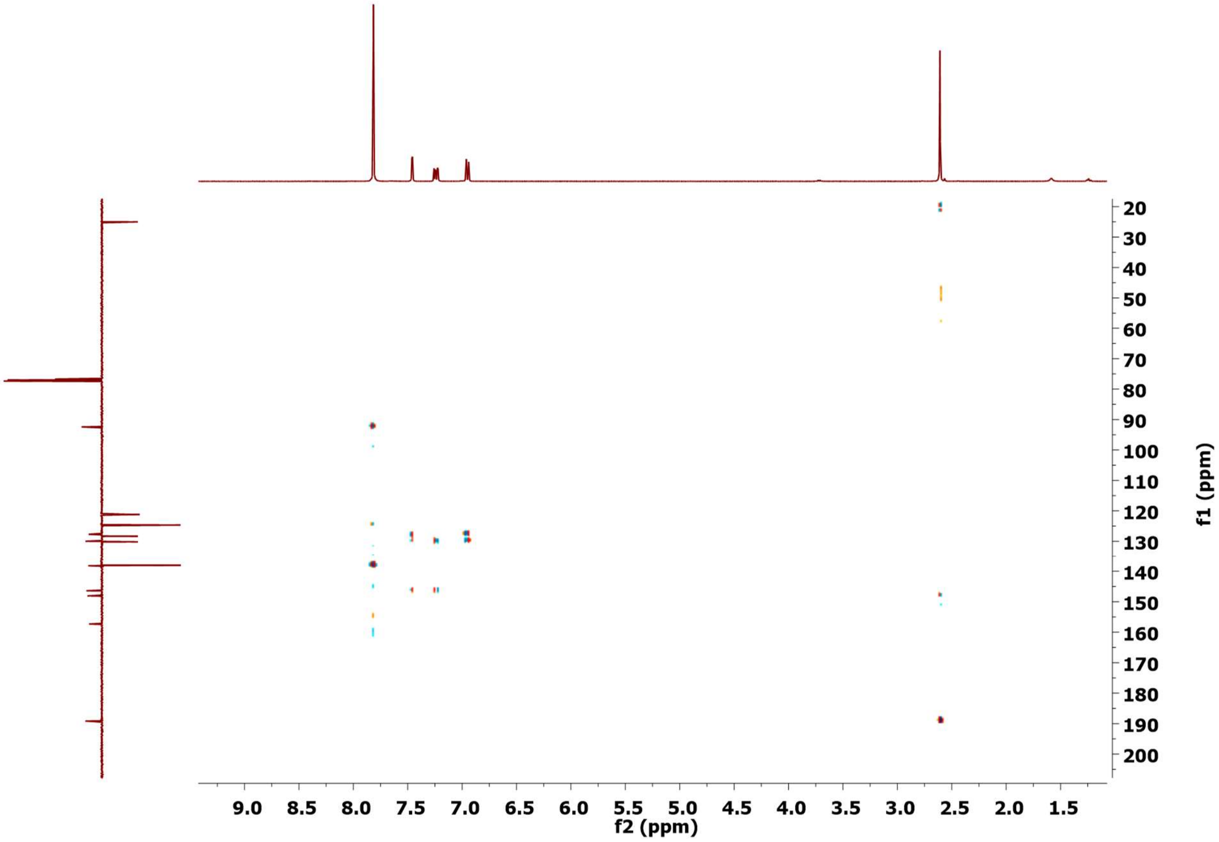

^{1}H NMR ($CDCl_3$) spectrum of (Z)-1-(4-(4-chloro-2-methylphenyl)-5-((2,4-dimethylphenyl)imino)-4,5-dihydro-1,3,4-thiadiazol-2-yl)ethan-1-one

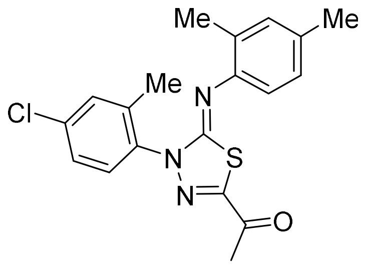

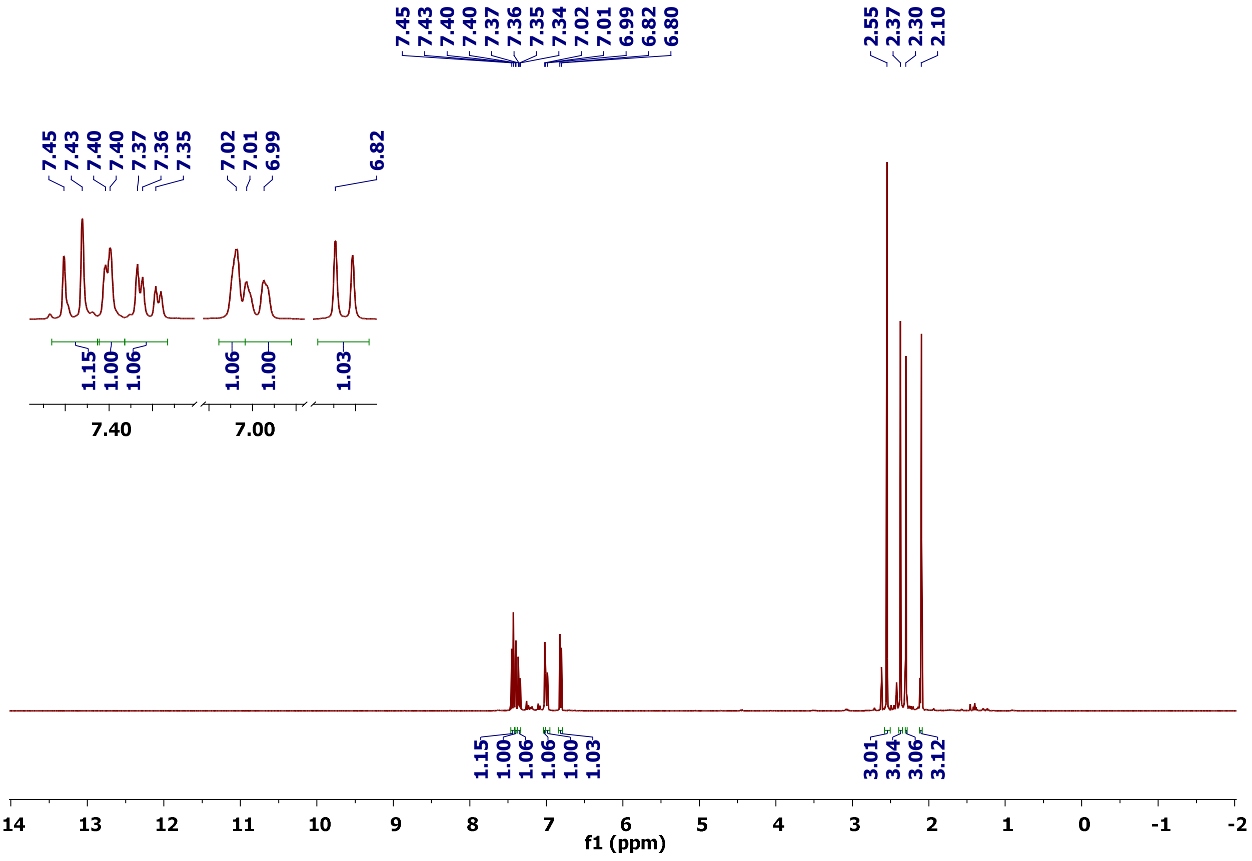

^{13}C NMR ($CDCl_3$) spectrum of (Z)-1-(4-(4-chloro-2-methylphenyl)-5-((2,4-dimethylphenyl)imino)-4,5-dihydro-1,3,4-thiadiazol-2-yl)ethan-1-one

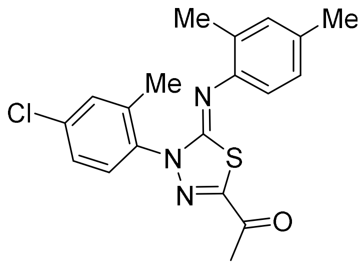

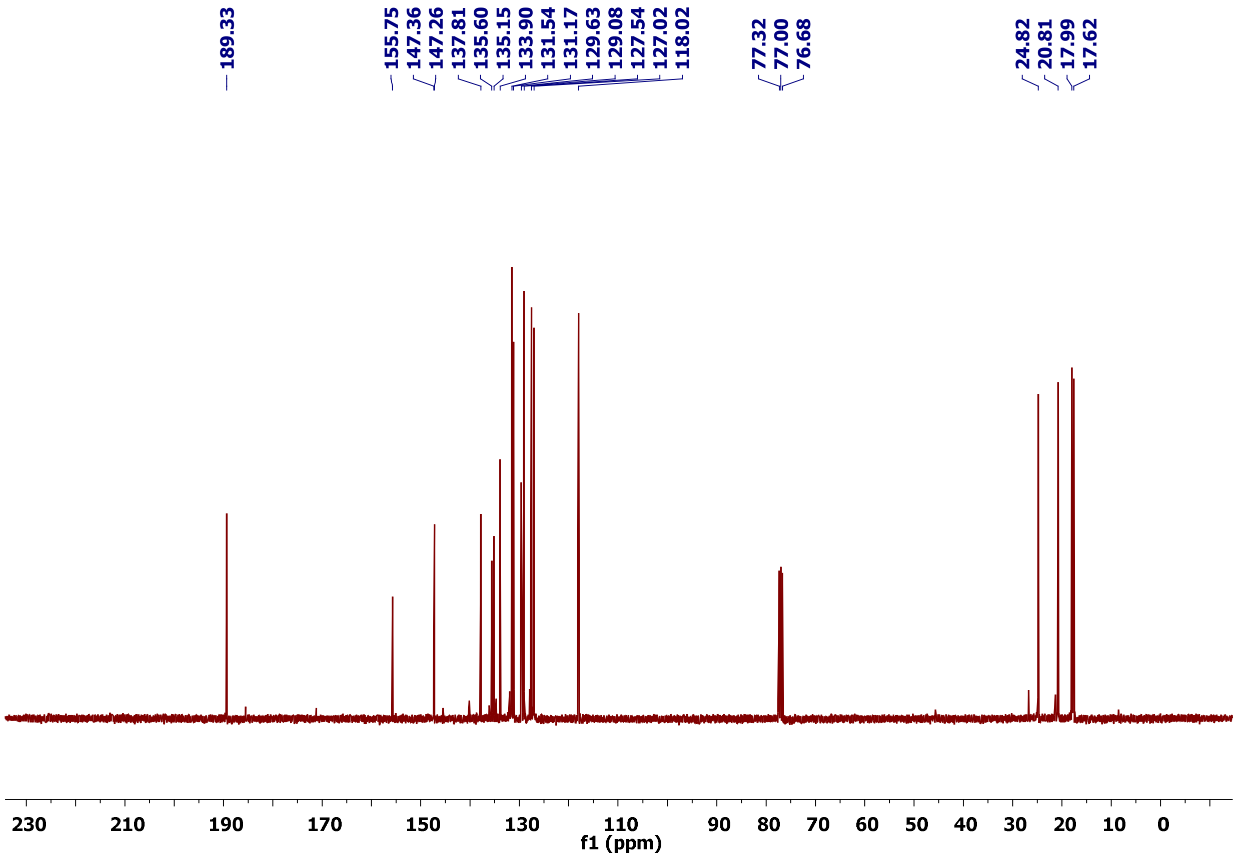

^{13}C CRAPT NMR ($CDCl_3$) spectrum of (Z)-1-(4-(4-chloro-2-methylphenyl)-5-((2,4-dimethylphenyl)imino)-4,5-dihydro-1,3,4-thiadiazol-2-yl)ethan-1-one

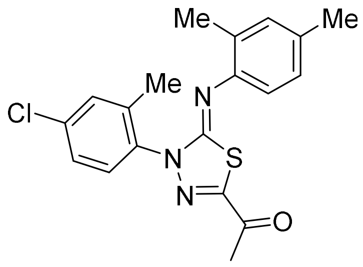

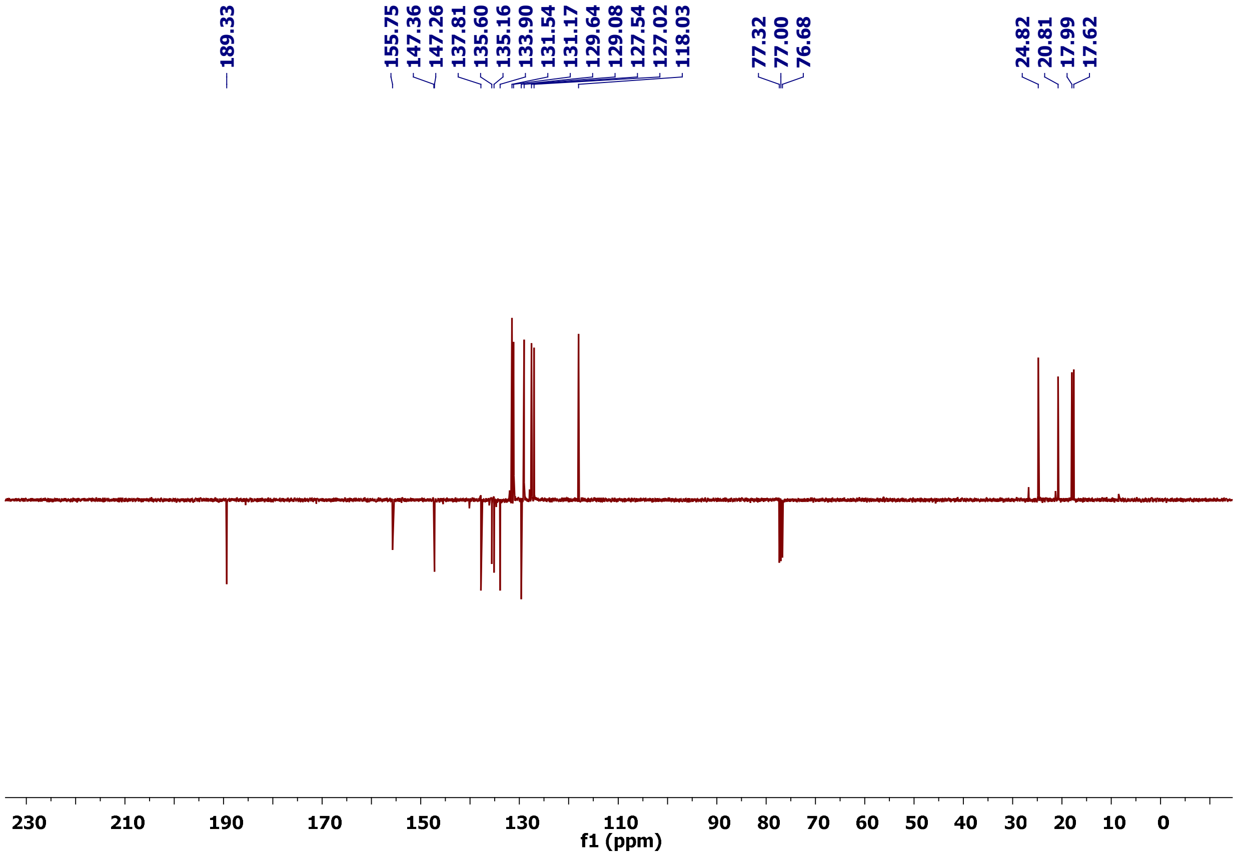

^{1}H-^{1}H gDQCOSY NMR ($CDCl_3$) spectrum of (Z)-1-(4-(4-chloro-2-methylphenyl)-5-((2,4-dimethylphenyl)imino)-4,5-dihydro-1,3,4-thiadiazol-2-yl)ethan-1-one

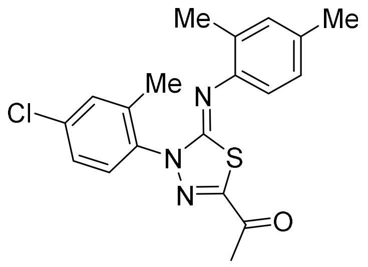

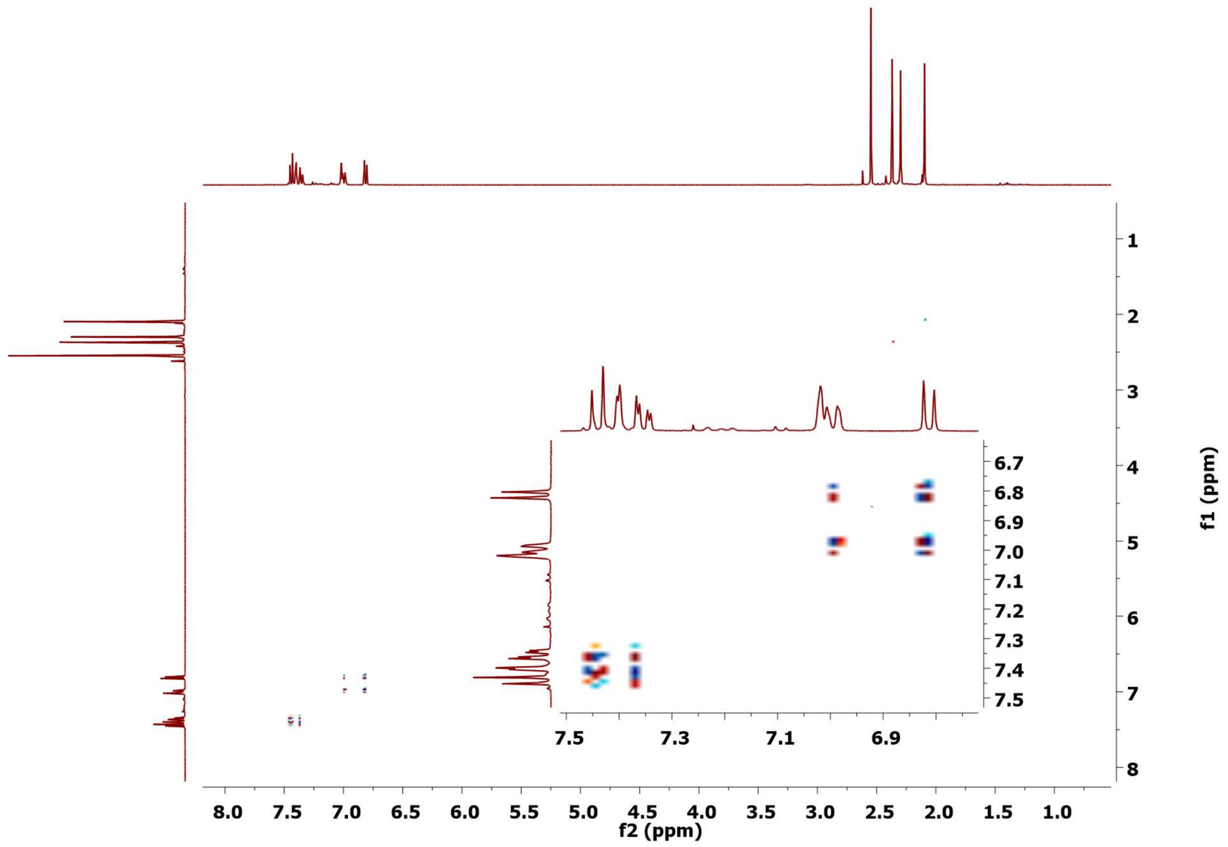

^{1}H-^{13}C-HSQC NMR ($CDCl_3$) spectrum of (Z)-1-(4-(4-chloro-2-methylphenyl)-5-((2,4-dimethylphenyl)imino)-4,5-dihydro-1,3,4-thiadiazol-2-yl)ethan-1-one

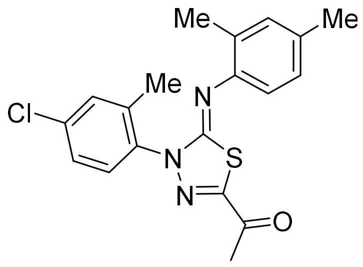

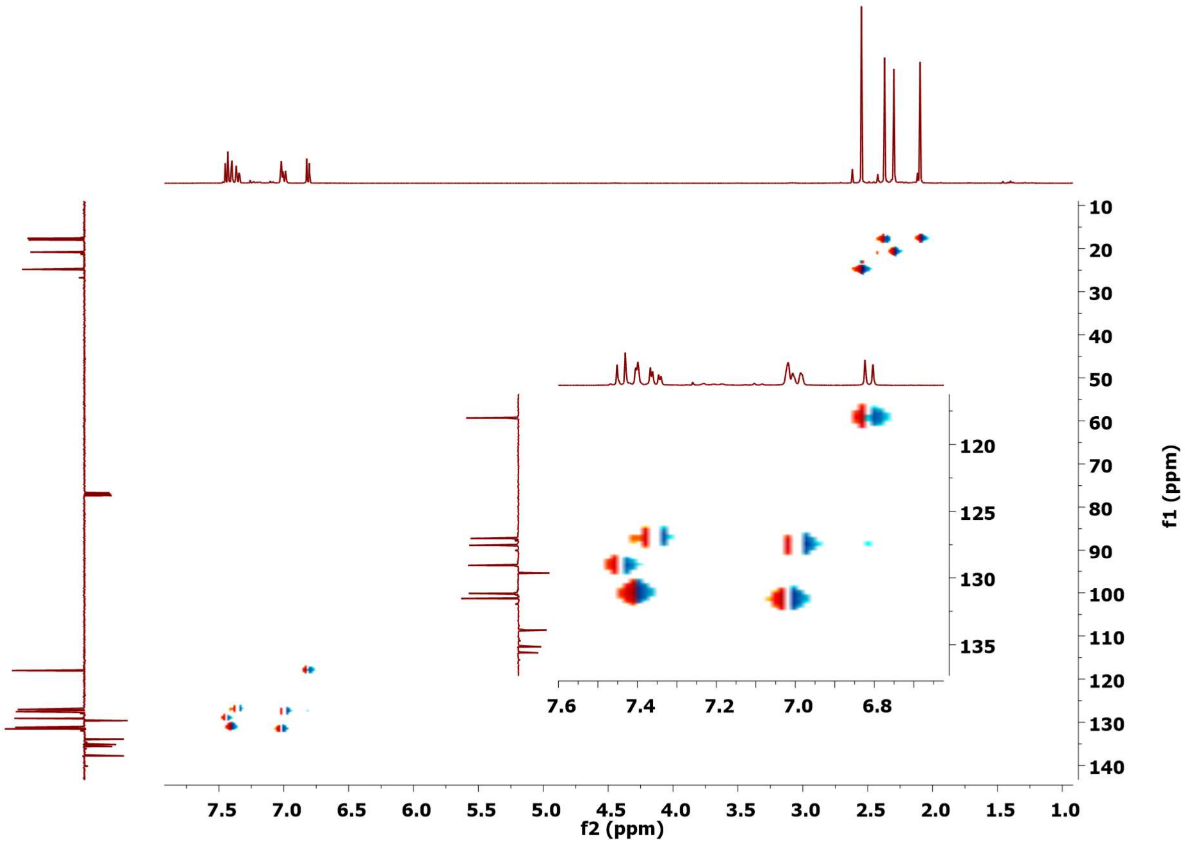

^{1}H-^{13}C-gHMBC NMR ($CDCl_3$) spectrum of (Z)-1-(4-(4-chloro-2-methylphenyl)-5-((2,4-dimethylphenyl)imino)-4,5-dihydro-1,3,4-thiadiazol-2-yl)ethan-1-one

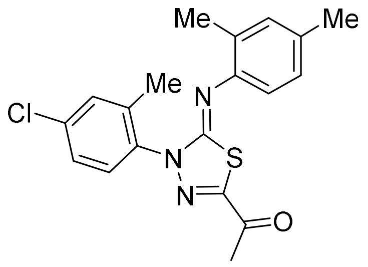

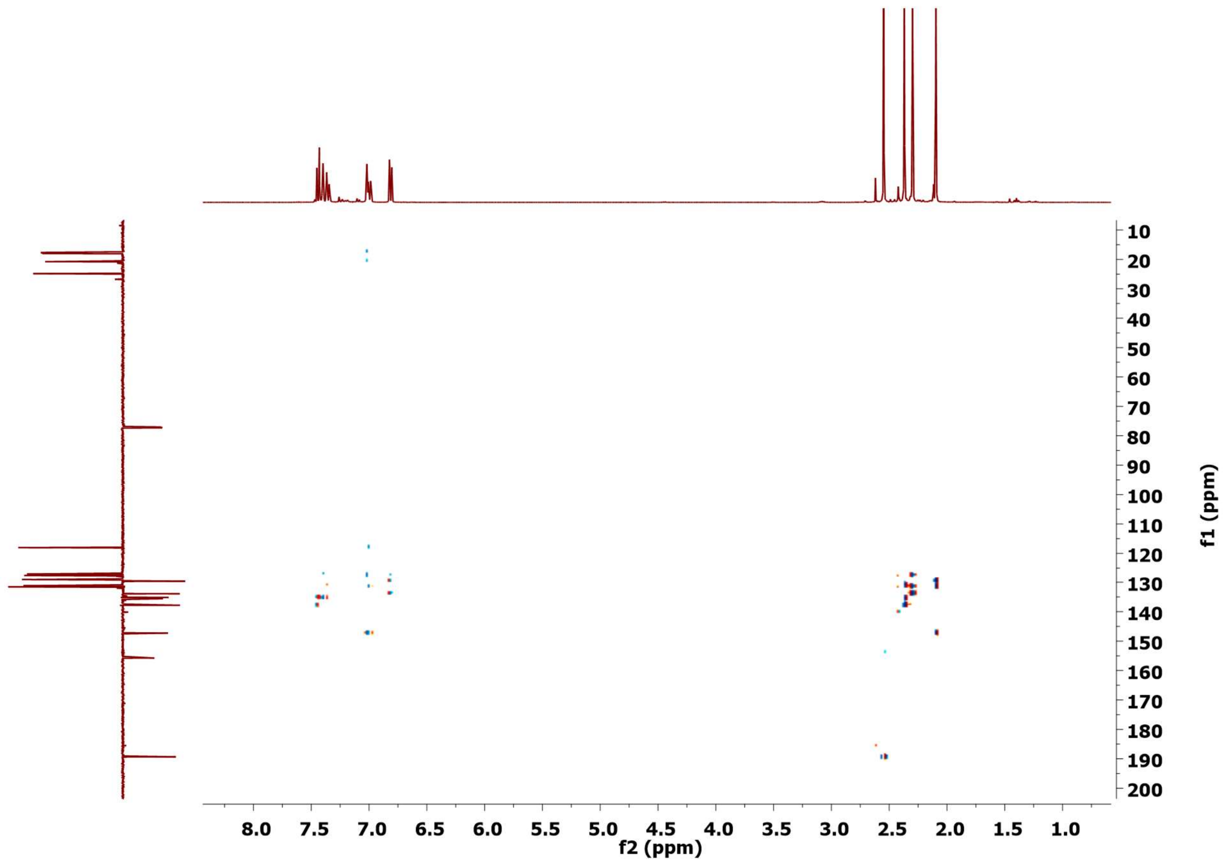

^1H NMR ($CDCl_3$) spectrum of (Z)-1-(4-(benzo[d][1,3]dioxol-5-yl)-5-((4-(benzyloxy)phenyl)imino)-4,5-dihydro-1,3,4-thiadiazol-2-yl)ethan-1-one

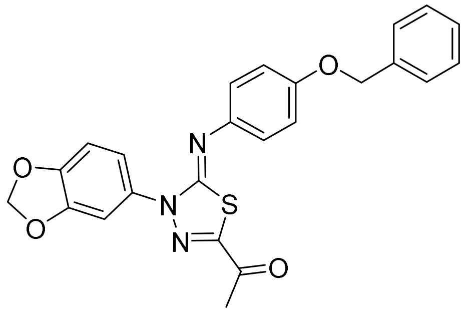

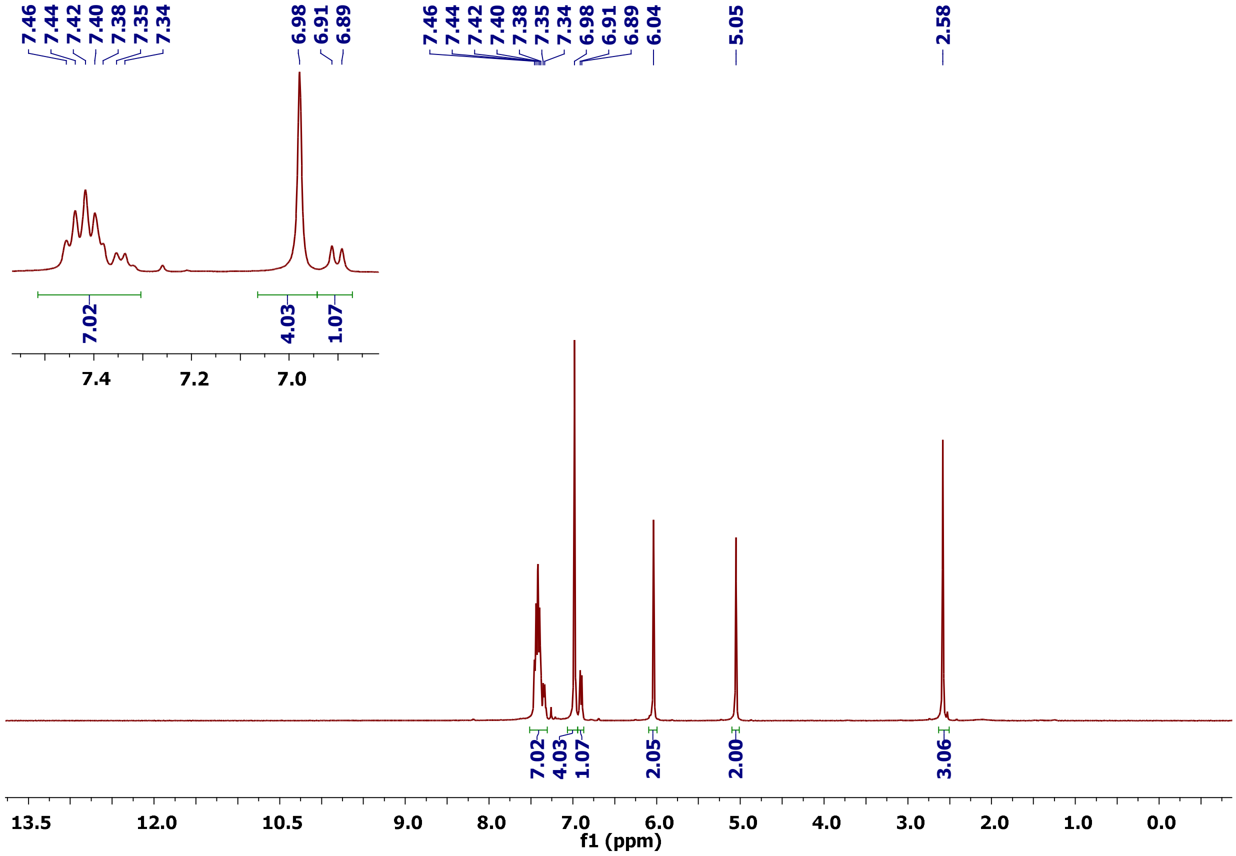

^{13}C NMR ($CDCl_3$) spectrum of (Z)-1-(4-(benzo[d][1,3]dioxol-5-yl)-5-((4-(benzyloxy)phenyl)imino)-4,5-dihydro-1,3,4-thiadiazol-2-yl)ethan-1-one

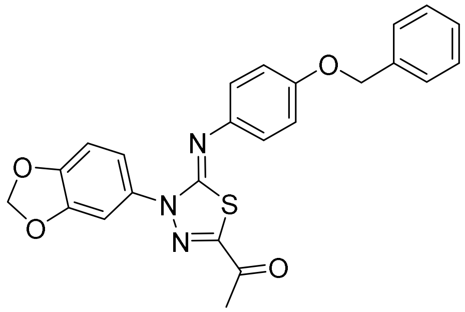

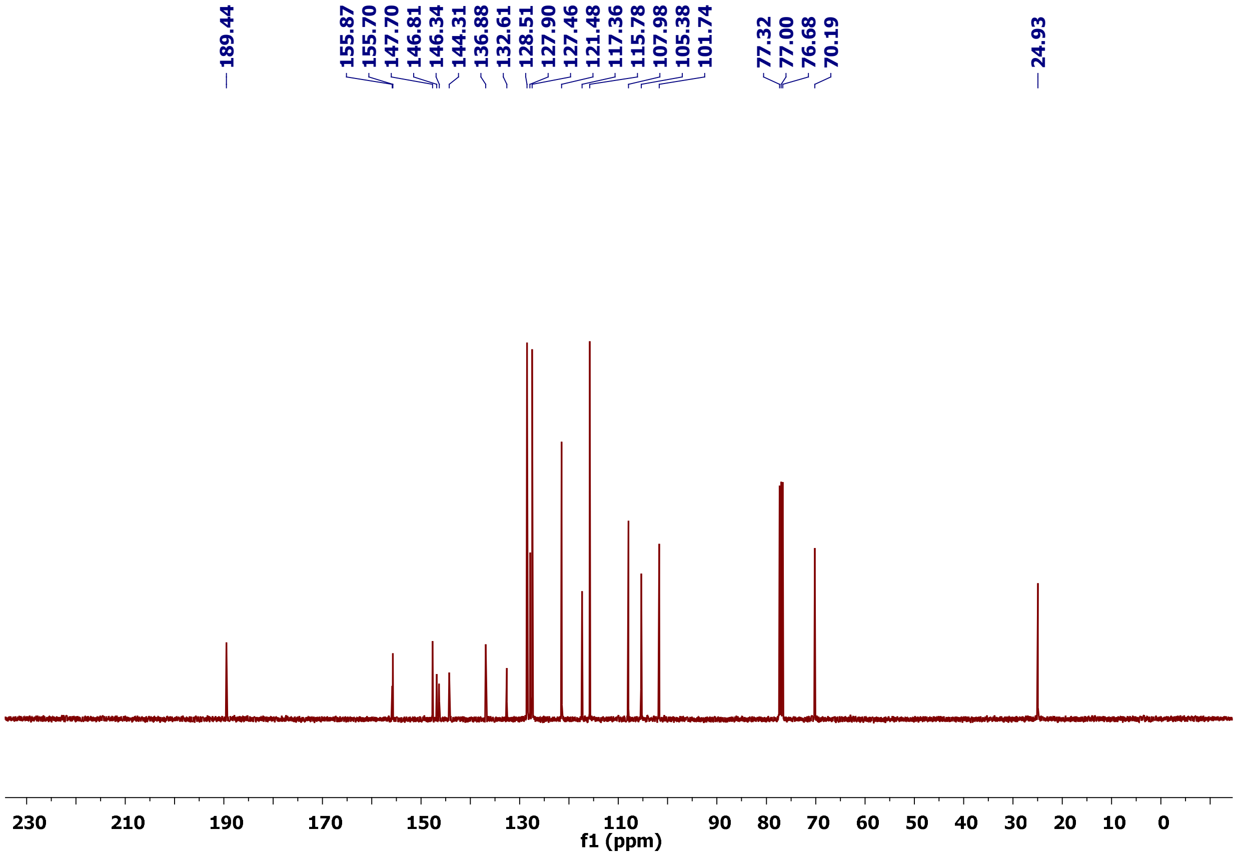

^{13}C CRAPT NMR ($CDCl_3$) spectrum of (Z)-1-(4-(benzo[d][1,3]dioxol-5-yl)-5-((4-(benzyloxy)phenyl)imino)-4,5-dihydro-1,3,4-thiadiazol-2-yl)ethan-1-one

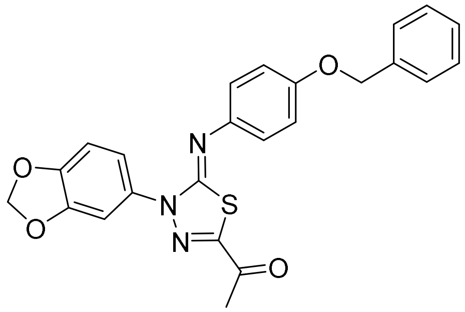

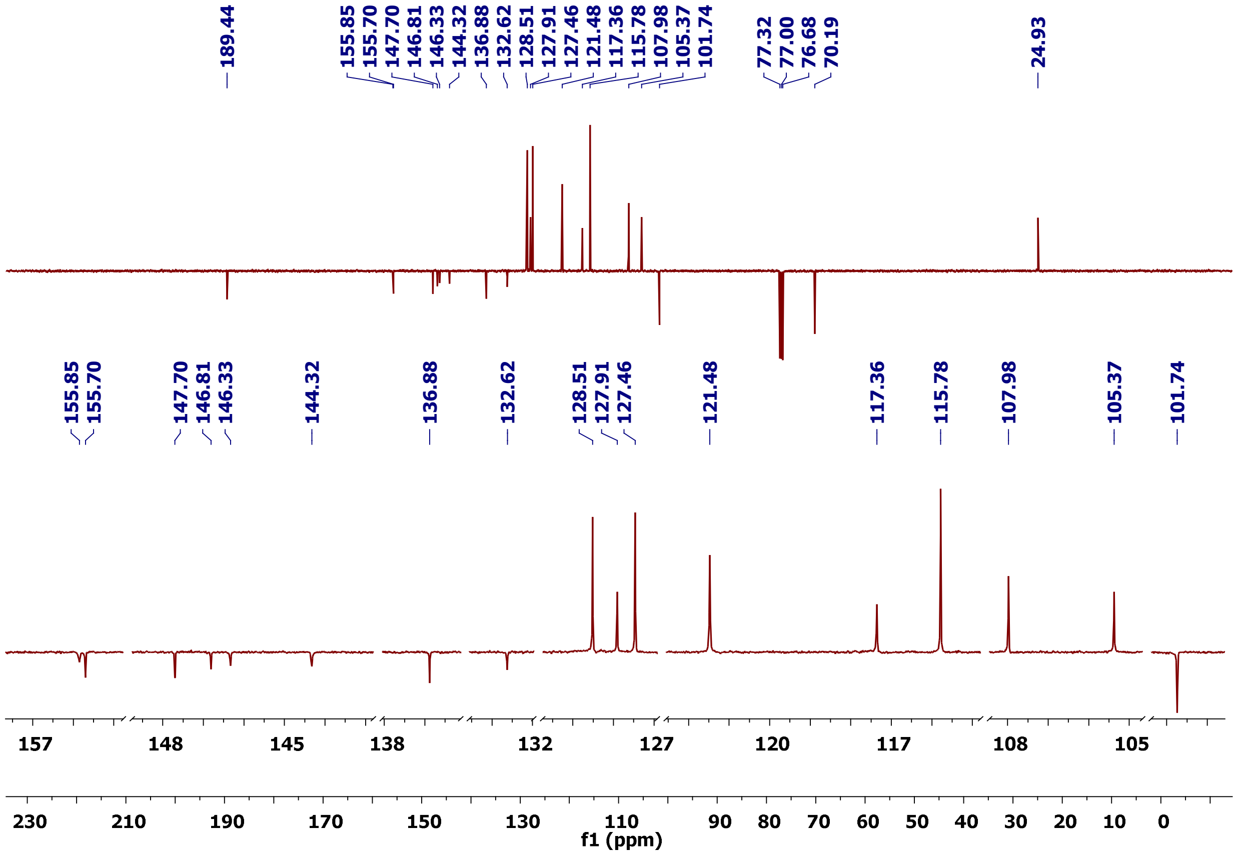

^{1}H-^{1}H gDQCOSY NMR ($CDCl_3$) spectrum of (Z)-1-(4-(benzo[d][1,3]dioxol-5-yl)-5-((4-(benzyloxy)phenyl)imino)-4,5-dihydro-1,3,4-thiadiazol-2-yl)ethan-1-one

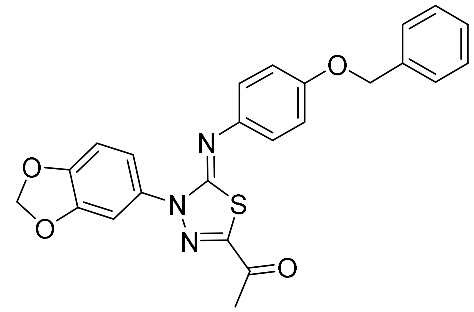

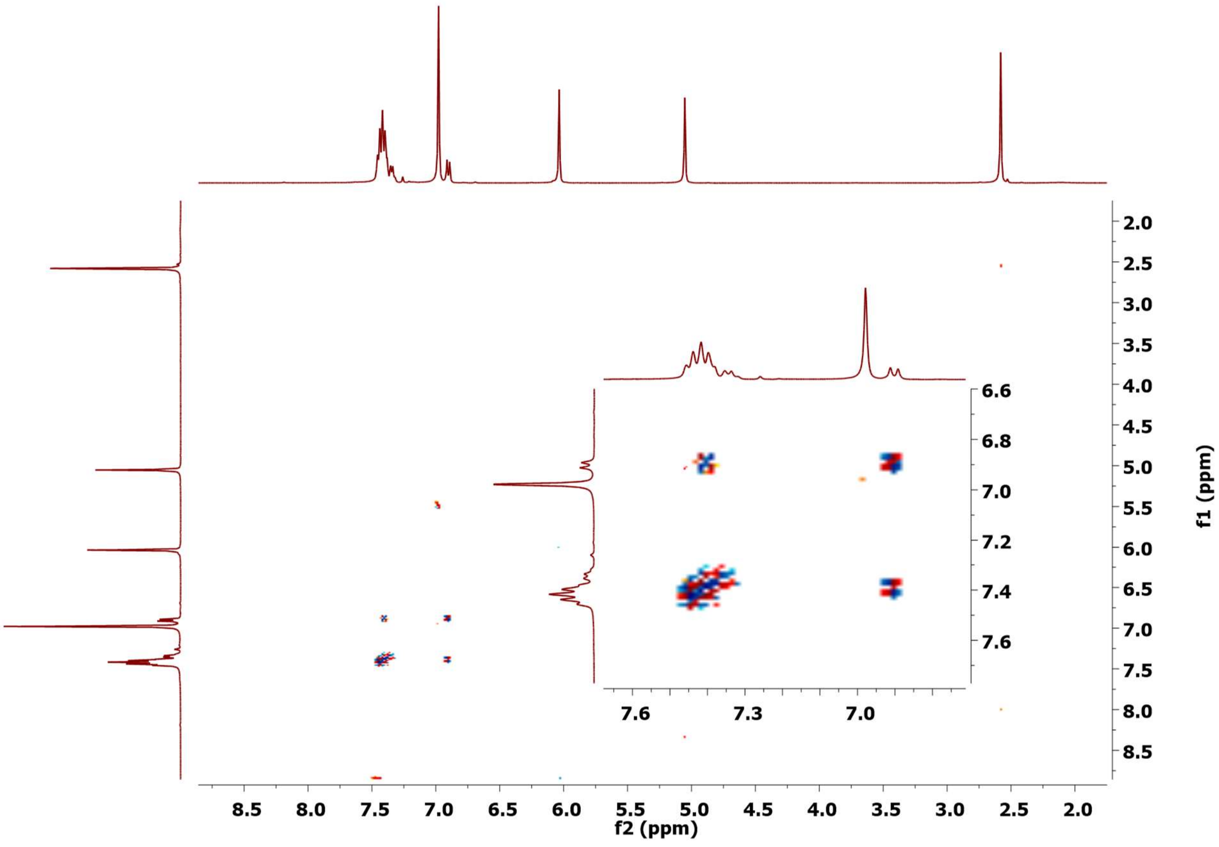

^{1}H-^{13}C-HSQC NMR ($CDCl_3$) spectrum of (Z)-1-(4-(benzo[d][1,3]dioxol-5-yl)-5-((4-(benzyloxy)phenyl)imino)-4,5-dihydro-1,3,4-thiadiazol-2-yl)ethan-1-one

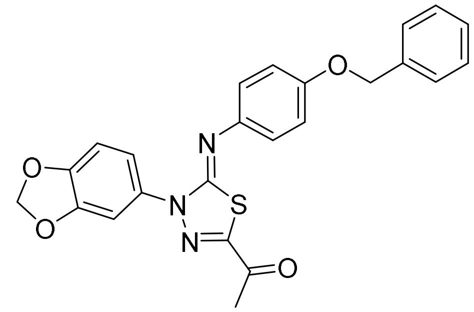

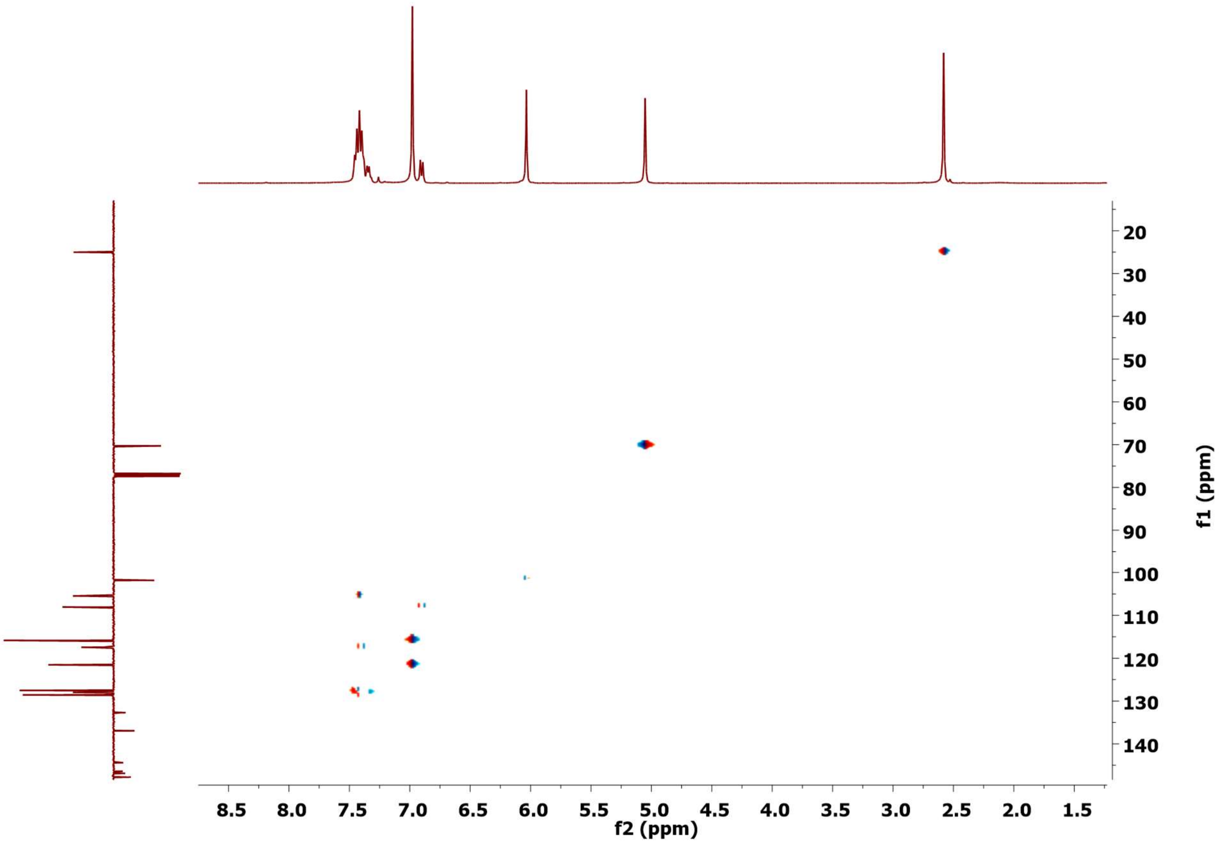

^{1}H-^{13}C-gHMBC NMR ($CDCl_3$) spectrum of (Z)-1-(4-(benzo[d][1,3]dioxol-5-yl)-5-((4-(benzyloxy)phenyl)imino)-4,5-dihydro-1,3,4-thiadiazol-2-yl)ethan-1-one

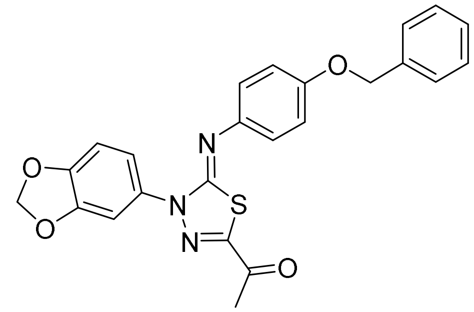

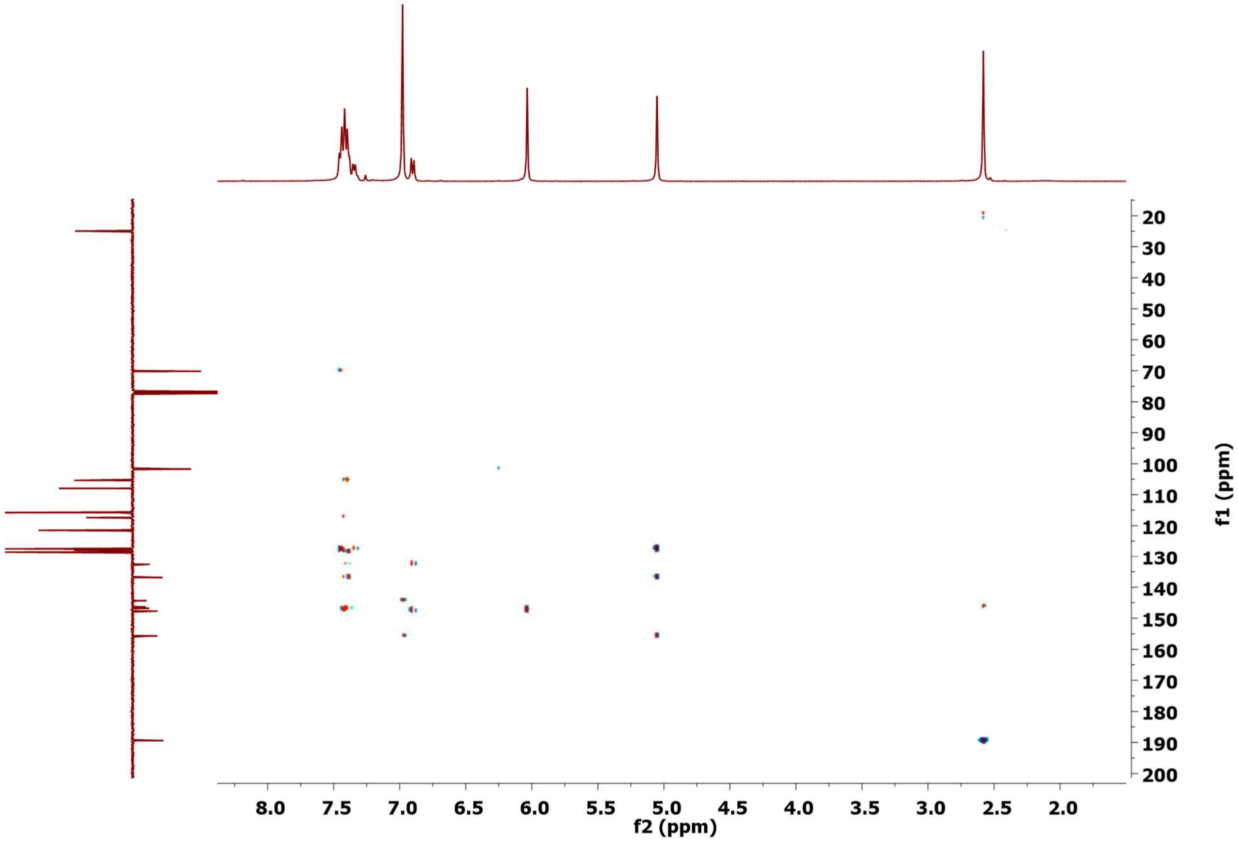

^{1}H NMR ($CDCl_3$) spectrum of (Z)-1-(4-(benzo[d][1,3]dioxol-5-yl)-5-((4-ethoxyphenyl)imino)-4,5-dihydro-1,3,4-thiadiazol-2-yl)ethan-1-one

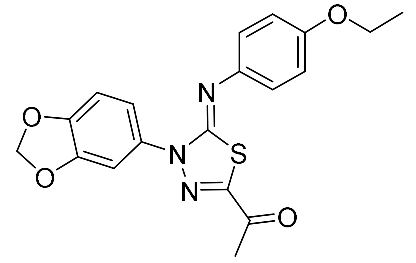

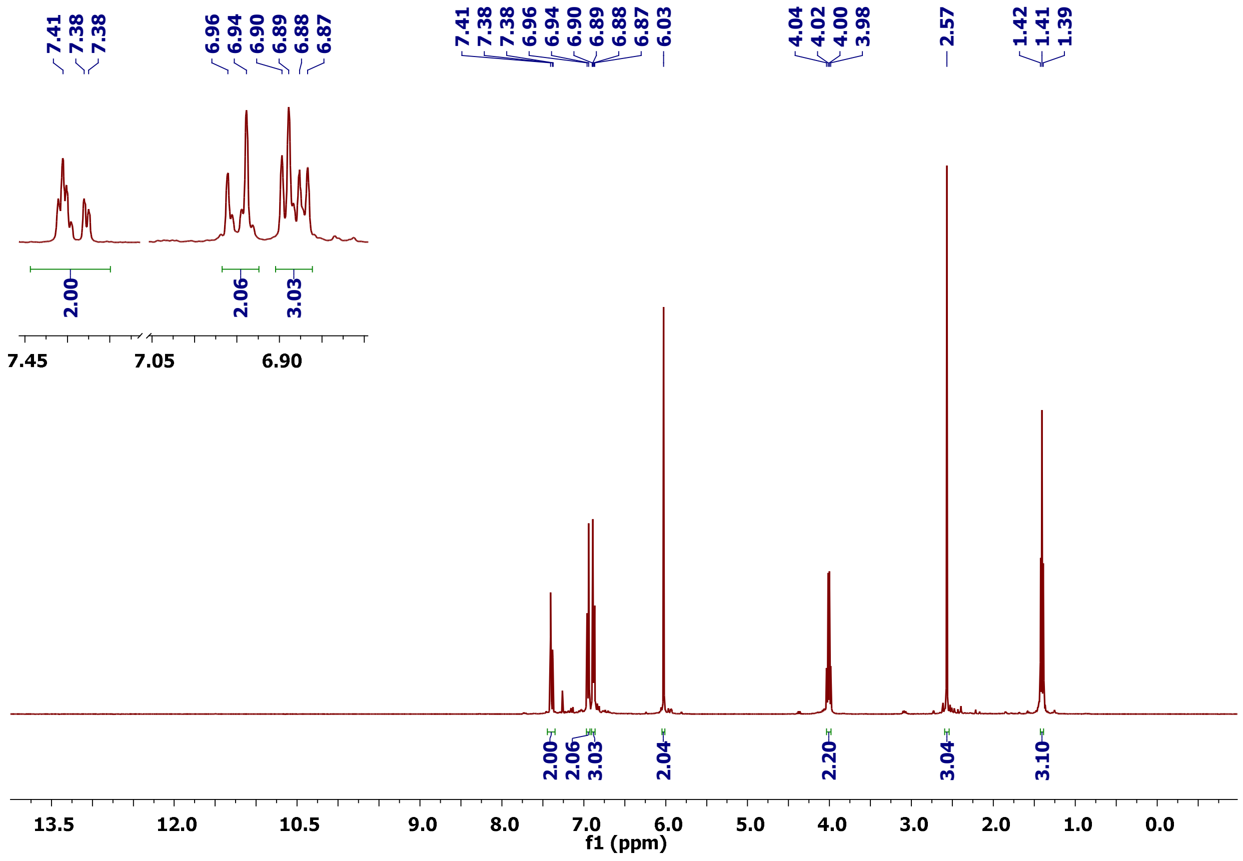

^{13}C NMR ($CDCl_3$) spectrum of (Z)-1-(4-(benzo[d][1,3]dioxol-5-yl)-5-((4-ethoxyphenyl)imino)-4,5-dihydro-1,3,4-thiadiazol-2-yl)ethan-1-one

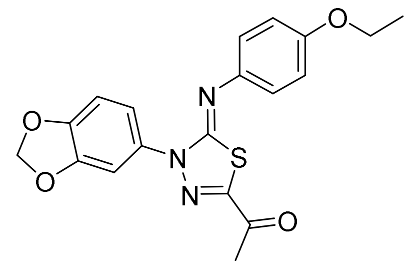

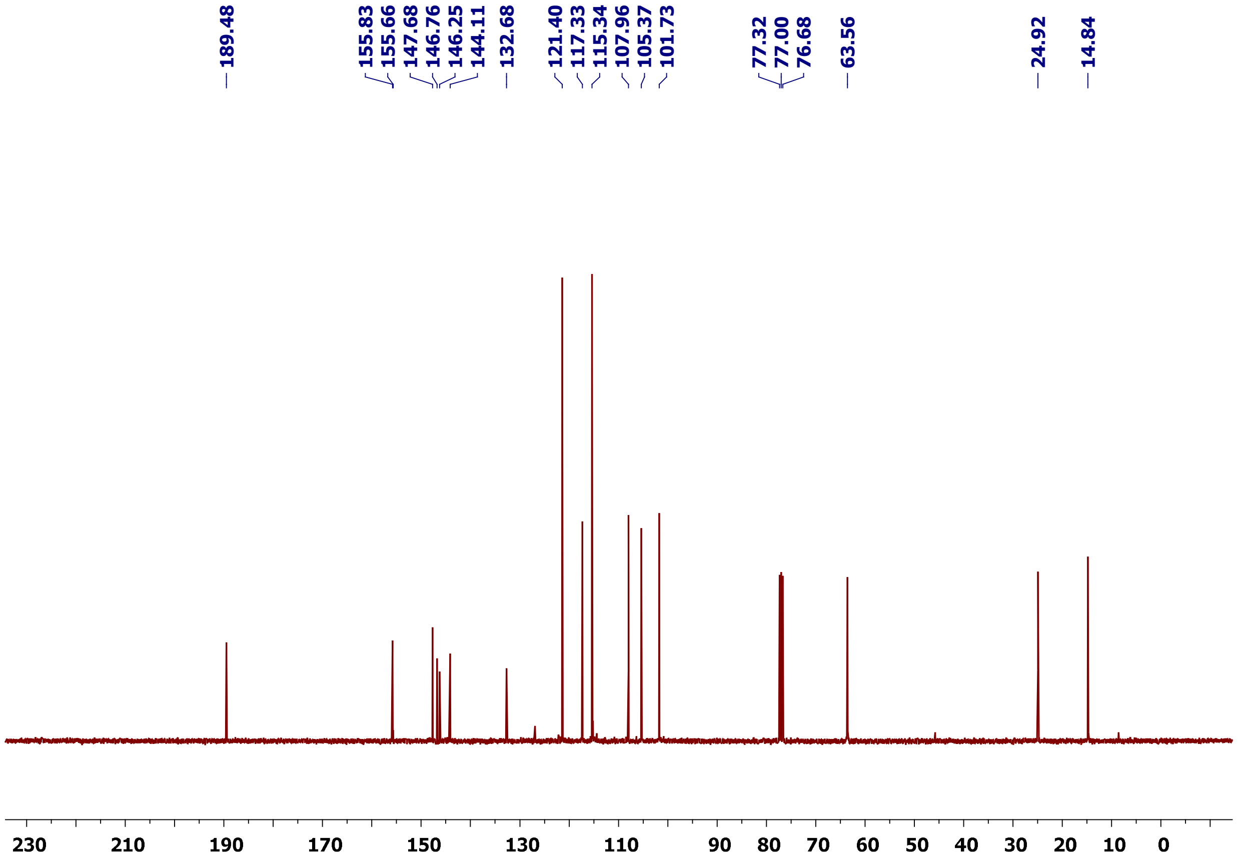

^{13}C CRAPT NMR ($CDCl_3$) spectrum of (Z)-1-(4-(benzo[d][1,3]dioxol-5-yl)-5-((4-ethoxyphenyl)imino)-4,5-dihydro-1,3,4-thiadiazol-2-yl)ethan-1-one

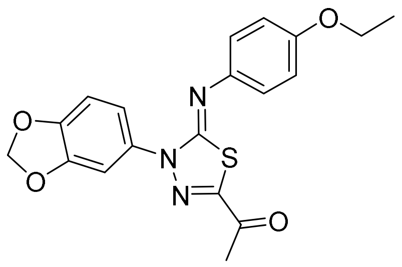

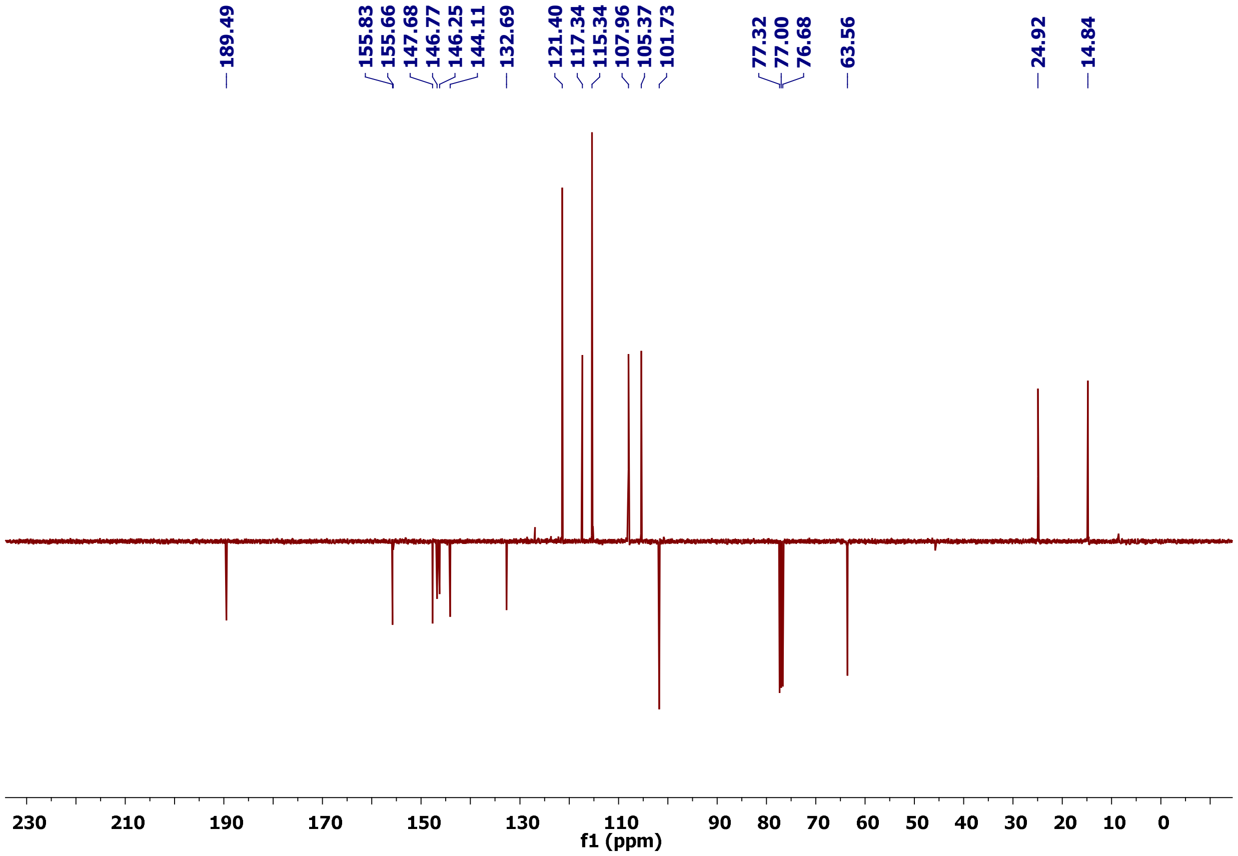

^{1}H-^{1}H gDQCOSY NMR ($CDCl_3$) spectrum of (Z)-1-(4-(benzo[d][1,3]dioxol-5-yl)-5-((4-ethoxyphenyl)imino)-4,5-dihydro-1,3,4-thiadiazol-2-yl)ethan-1-one

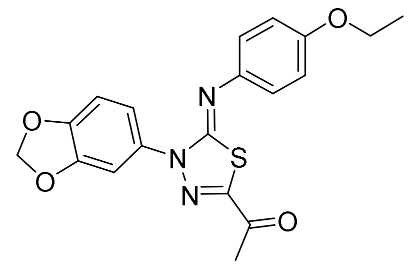

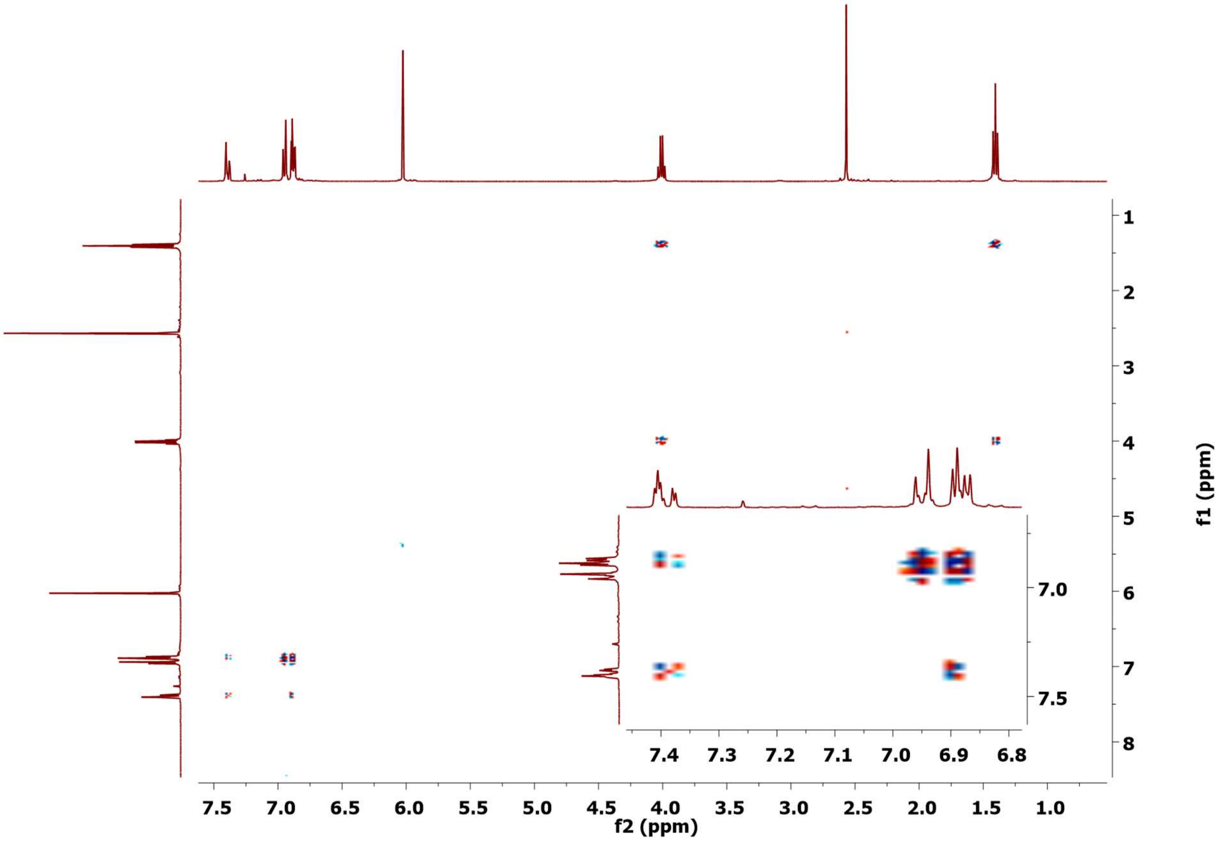

^{1}H-^{13}C-HSQC NMR ($CDCl_3$) spectrum of (Z)-1-(4-(benzo[d][1,3]dioxol-5-yl)-5-((4-ethoxyphenyl)imino)-4,5-dihydro-1,3,4-thiadiazol-2-yl)ethan-1-one

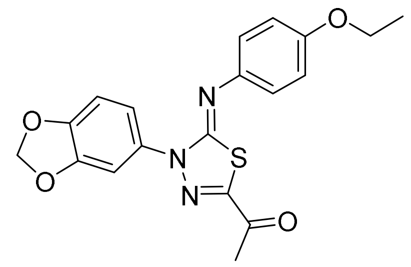

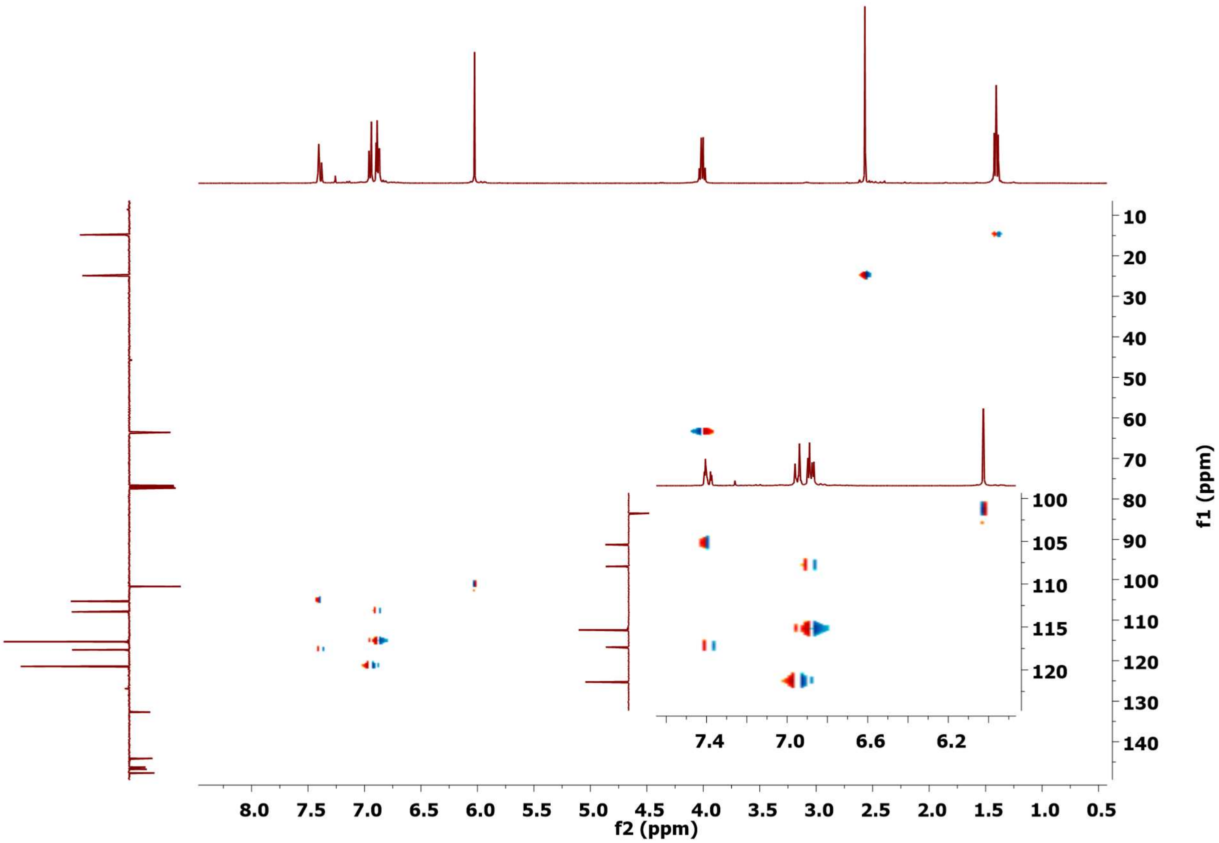

^{1}H-^{13}C-gHMBC NMR ($CDCl_3$) spectrum of (Z)-1-(4-(benzo[d][1,3]dioxol-5-yl)-5-((4-ethoxyphenyl)imino)-4,5-dihydro-1,3,4-thiadiazol-2-yl)ethan-1-one

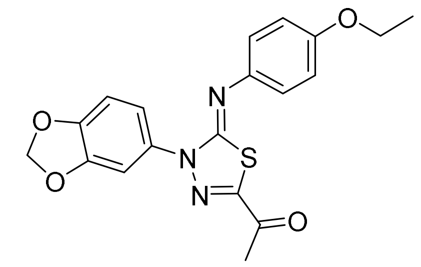

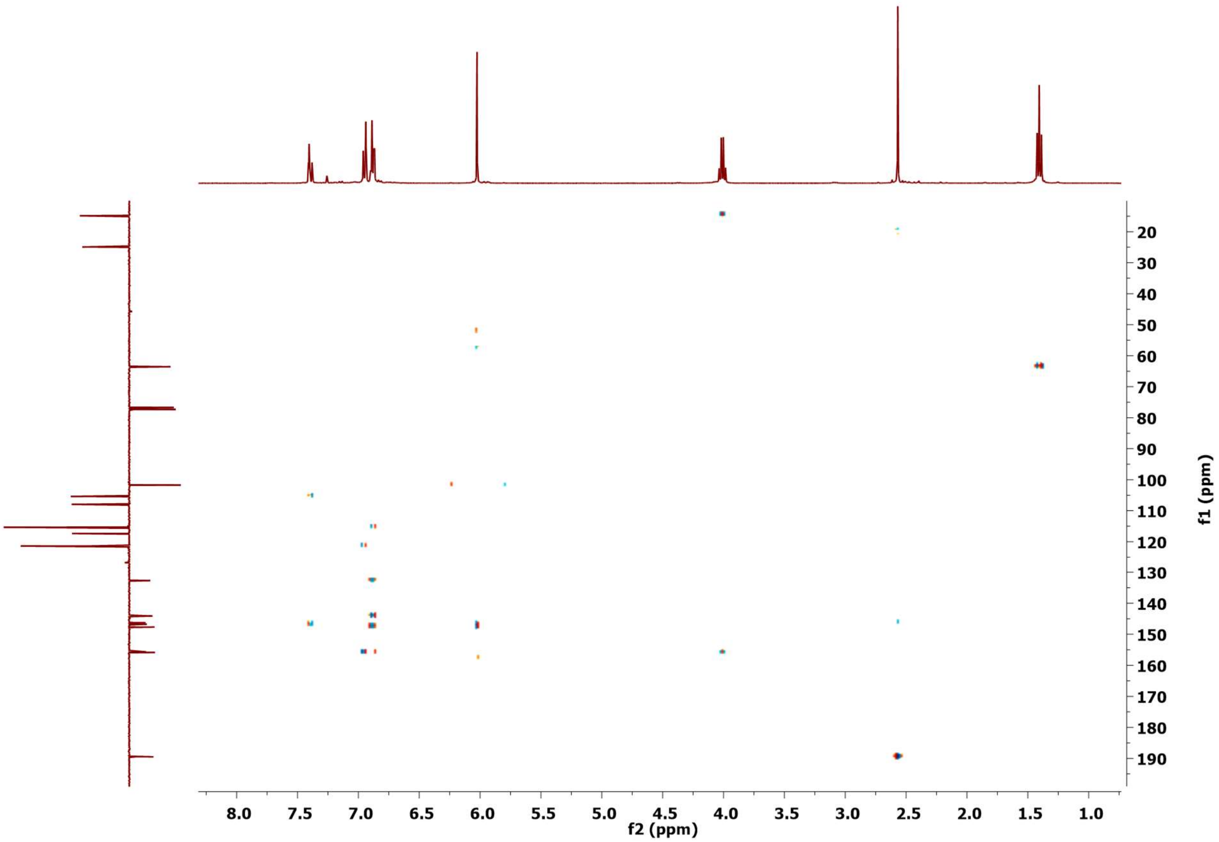

^{1}H NMR ($CDCl_3$) spectrum of (Z)-1-(4-(benzo[d][1,3]dioxol-5-yl)-5-((3,4-dimethoxyphenyl)imino)-4,5-dihydro-1,3,4-thiadiazol-2-yl)ethan-1-one

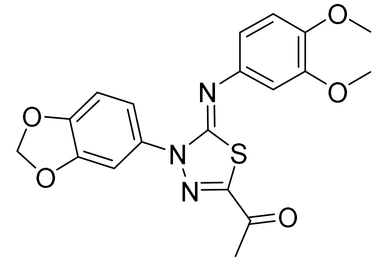

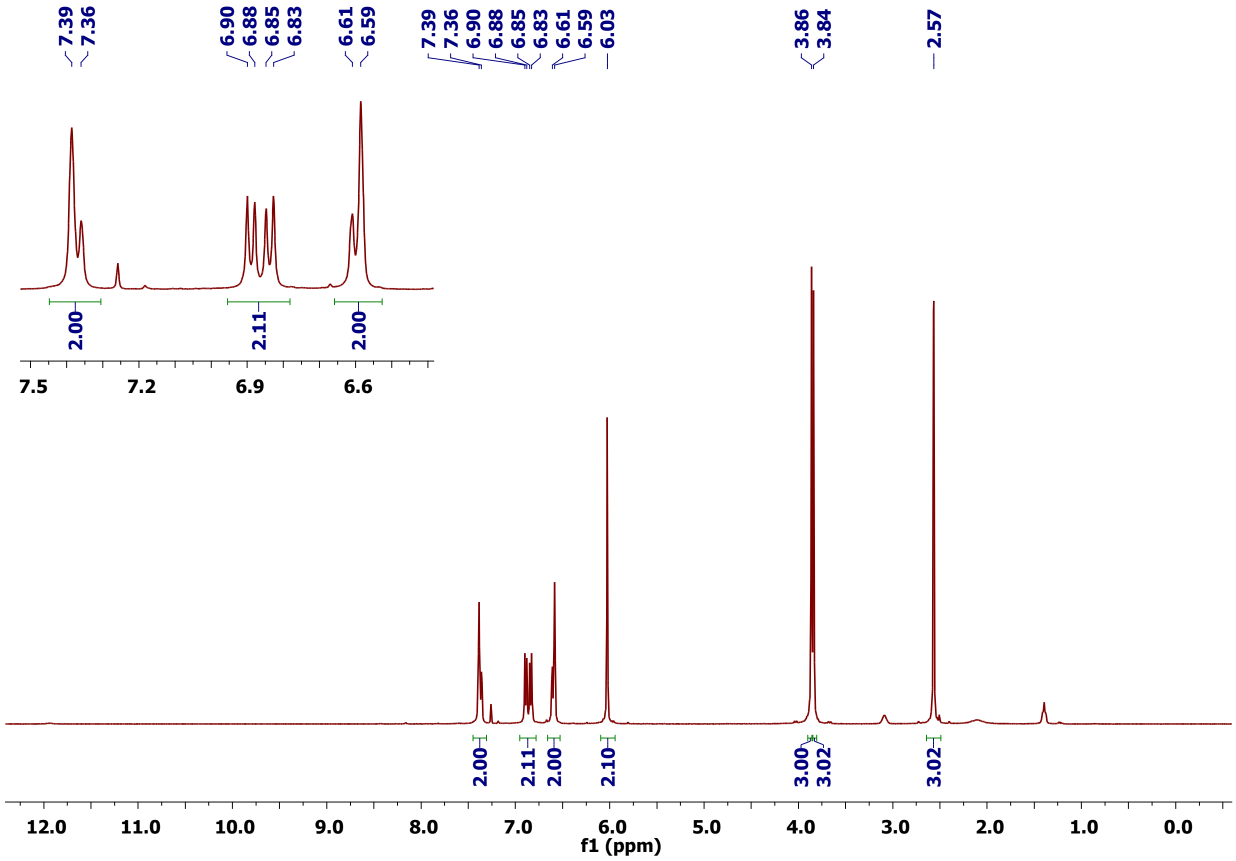

^{13}C NMR ($CDCl_3$) spectrum of (Z)-1-(4-(benzo[d][1,3]dioxol-5-yl)-5-((3,4-dimethoxyphenyl)imino)-4,5-dihydro-1,3,4-thiadiazol-2-yl)ethan-1-one

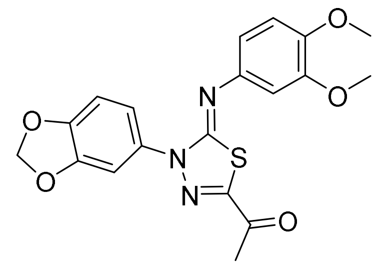

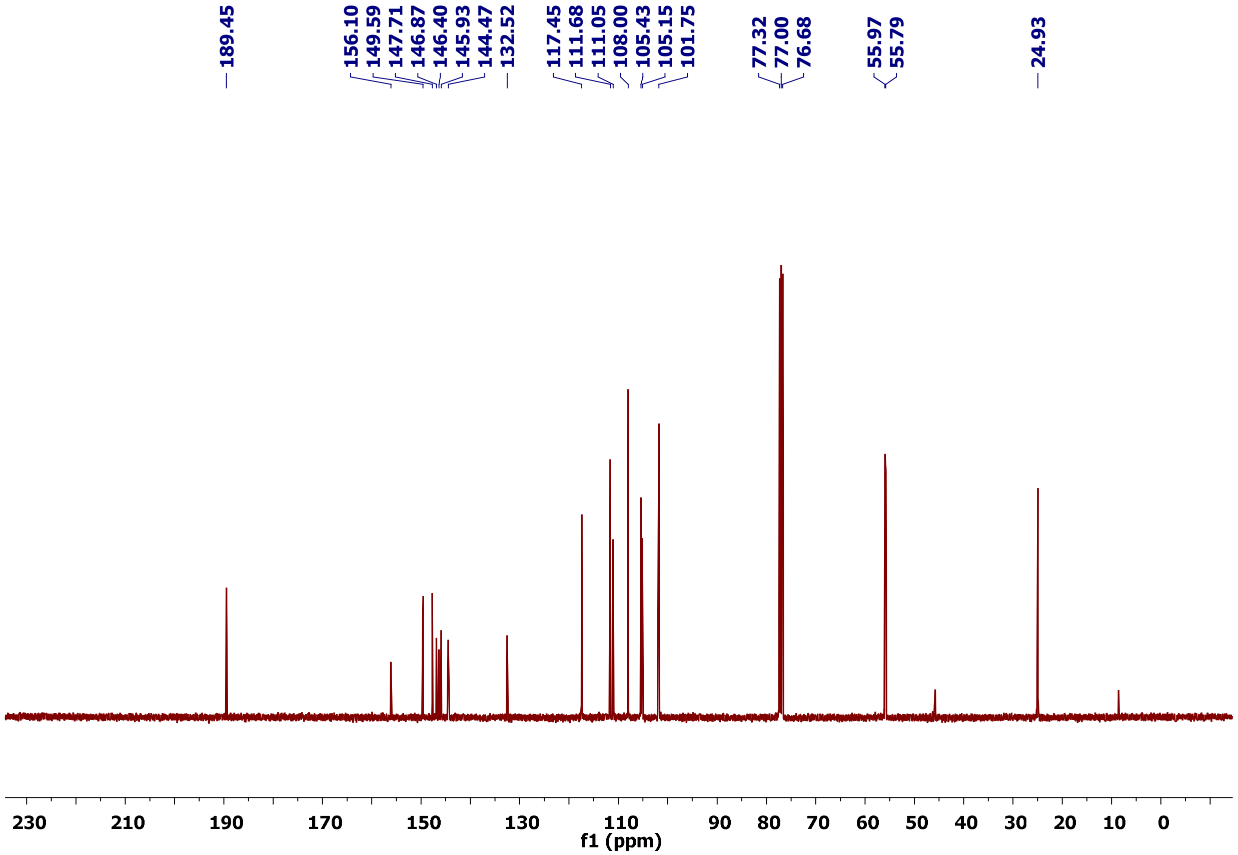

^{13}C CRAPT NMR ($CDCl_3$) spectrum of (Z)-1-(4-(benzo[d][1,3]dioxol-5-yl)-5-((3,4-dimethoxyphenyl)imino)-4,5-dihydro-1,3,4-thiadiazol-2-yl)ethan-1-one

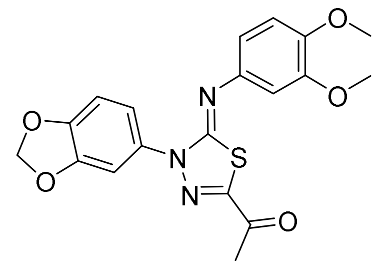

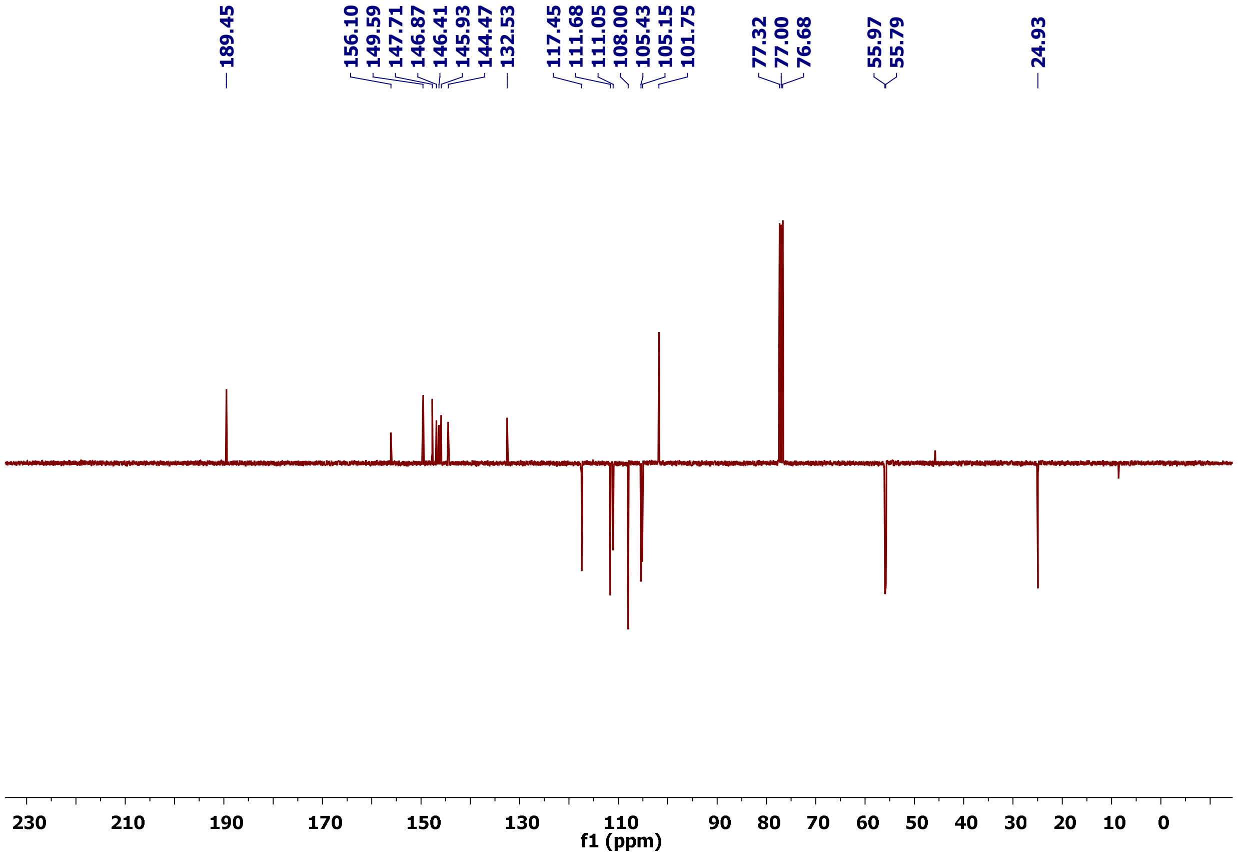

^{1}H-^{1}H gDQCOSY NMR ($CDCl_3$) spectrum of (Z)-1-(4-(benzo[d][1,3]dioxol-5-yl)-5-((3,4-dimethoxyphenyl)imino)-4,5-dihydro-1,3,4-thiadiazol-2-yl)ethan-1-one

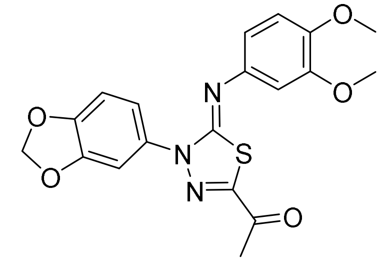

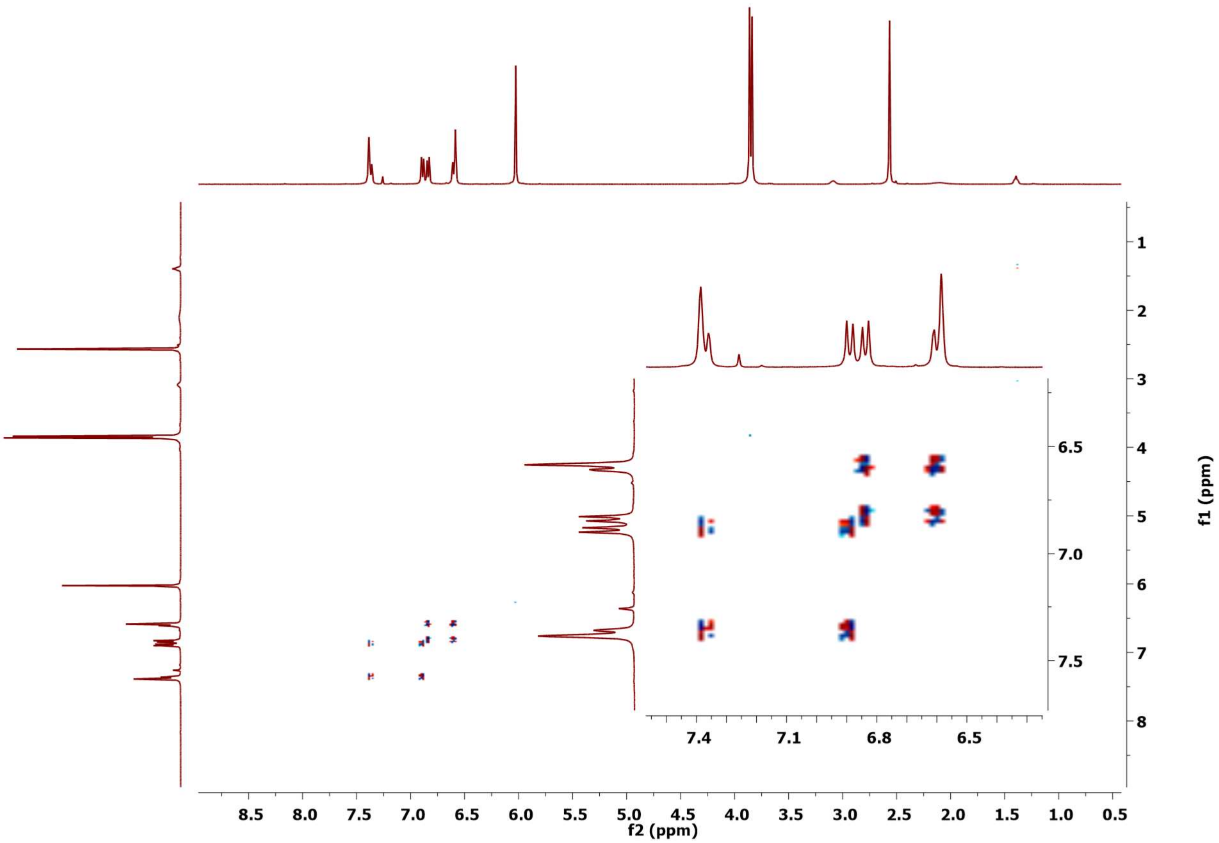

^{1}H-^{13}C-HSQC NMR ($CDCl_3$) spectrum of (Z)-1-(4-(benzo[d][1,3]dioxol-5-yl)-5-((3,4-dimethoxyphenyl)imino)-4,5-dihydro-1,3,4-thiadiazol-2-yl)ethan-1-one

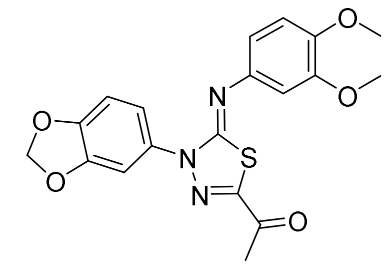

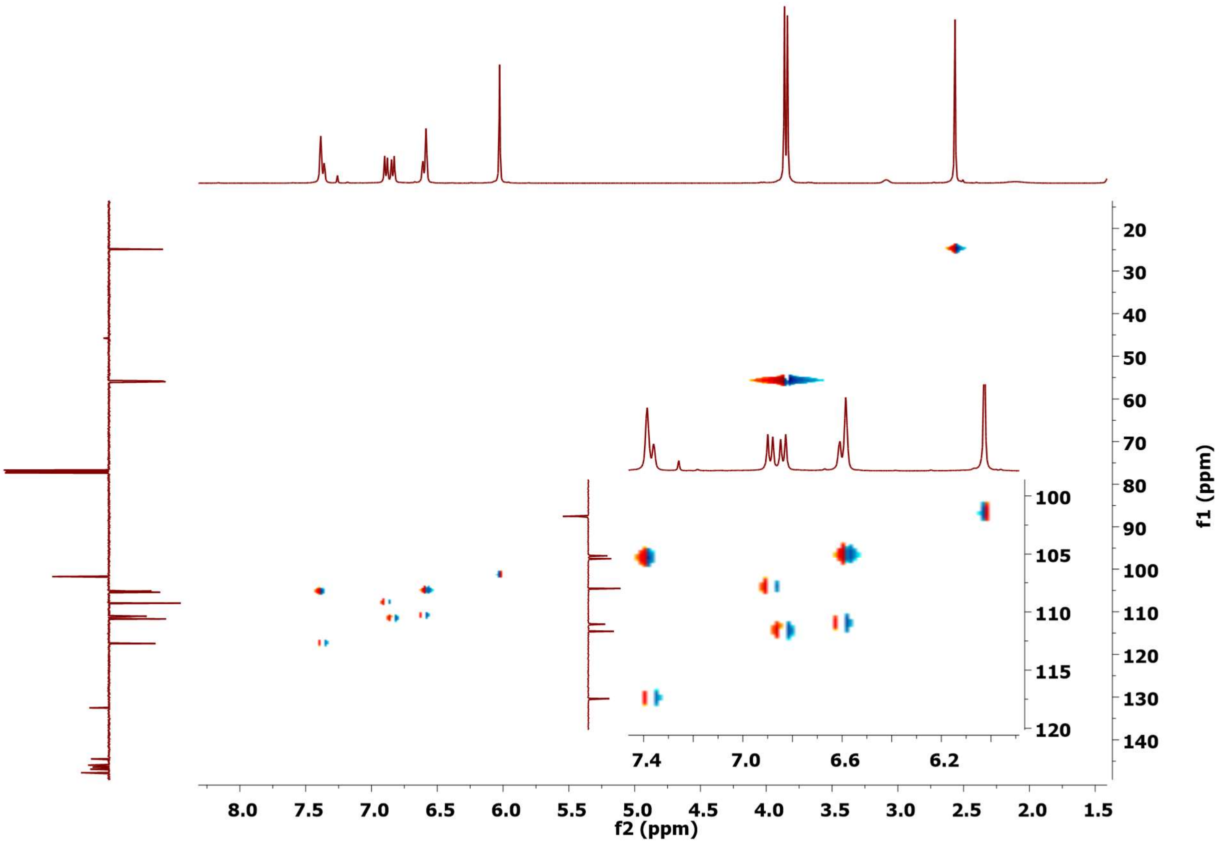

^1H-^{13}C-gHMBC NMR ($CDCl_3$) spectrum of (Z)-1-(4-(benzo[d][1,3]dioxol-5-yl)-5-((3,4-dimethoxyphenyl)imino)-4,5-dihydro-1,3,4-thiadiazol-2-yl)ethan-1-one

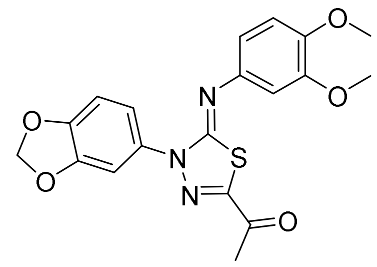

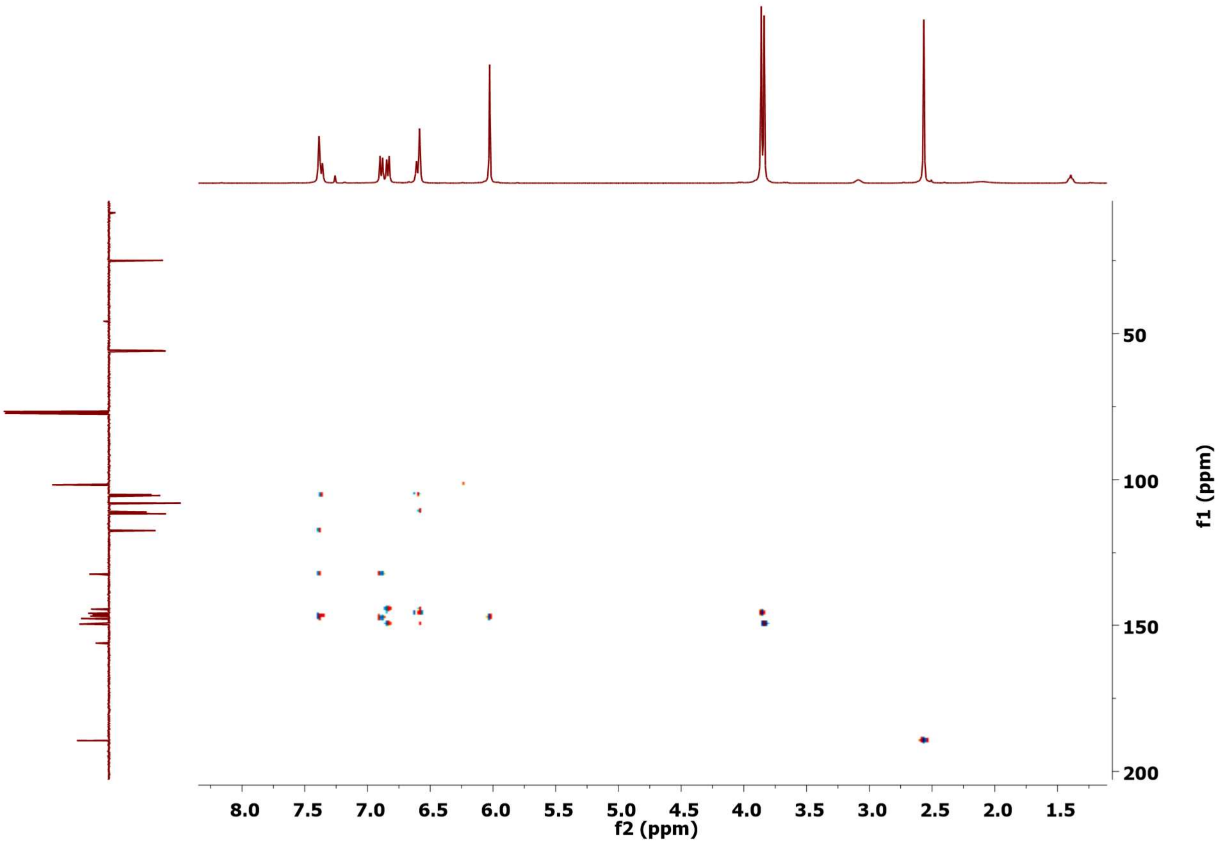

^{1}H NMR ($CDCl_3$) spectrum of (Z)-1-(5-((3,4-dimethoxyphenyl)imino)-4-(4-methoxyphenyl)-4,5-dihydro-1,3,4-thiadiazol-2-yl)ethan-1-one

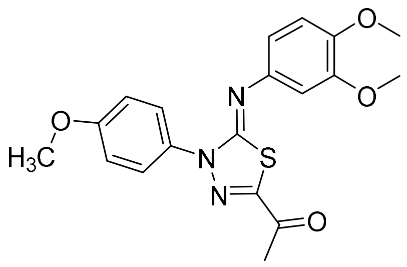

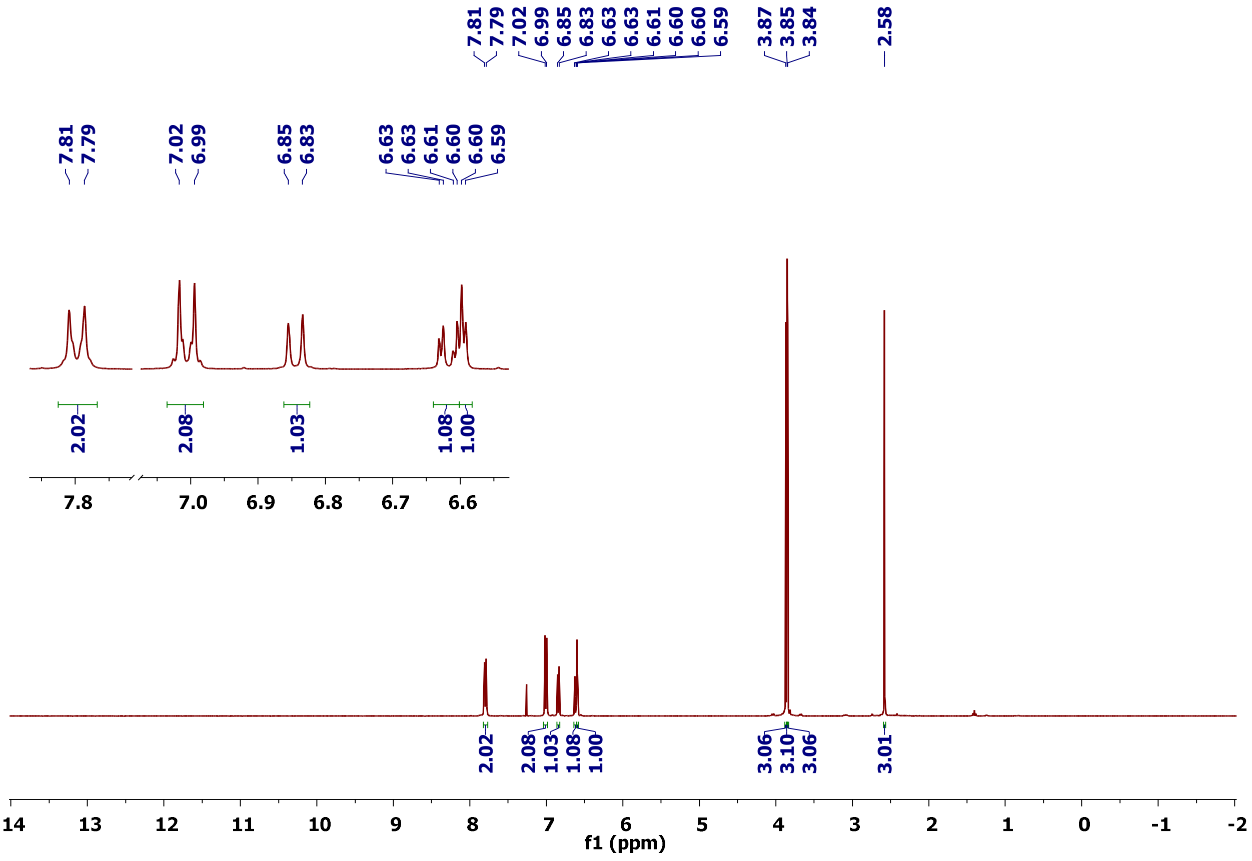

^{13}C NMR ($CDCl_3$) spectrum of (Z)-1-(5-((3,4-dimethoxyphenyl)imino)-4-(4-methoxyphenyl)-4,5-dihydro-1,3,4-thiadiazol-2-yl)ethan-1-one

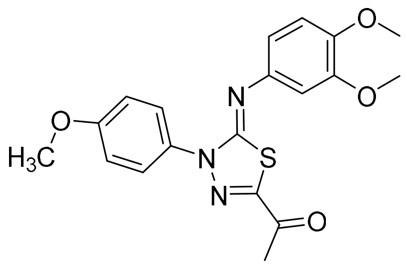

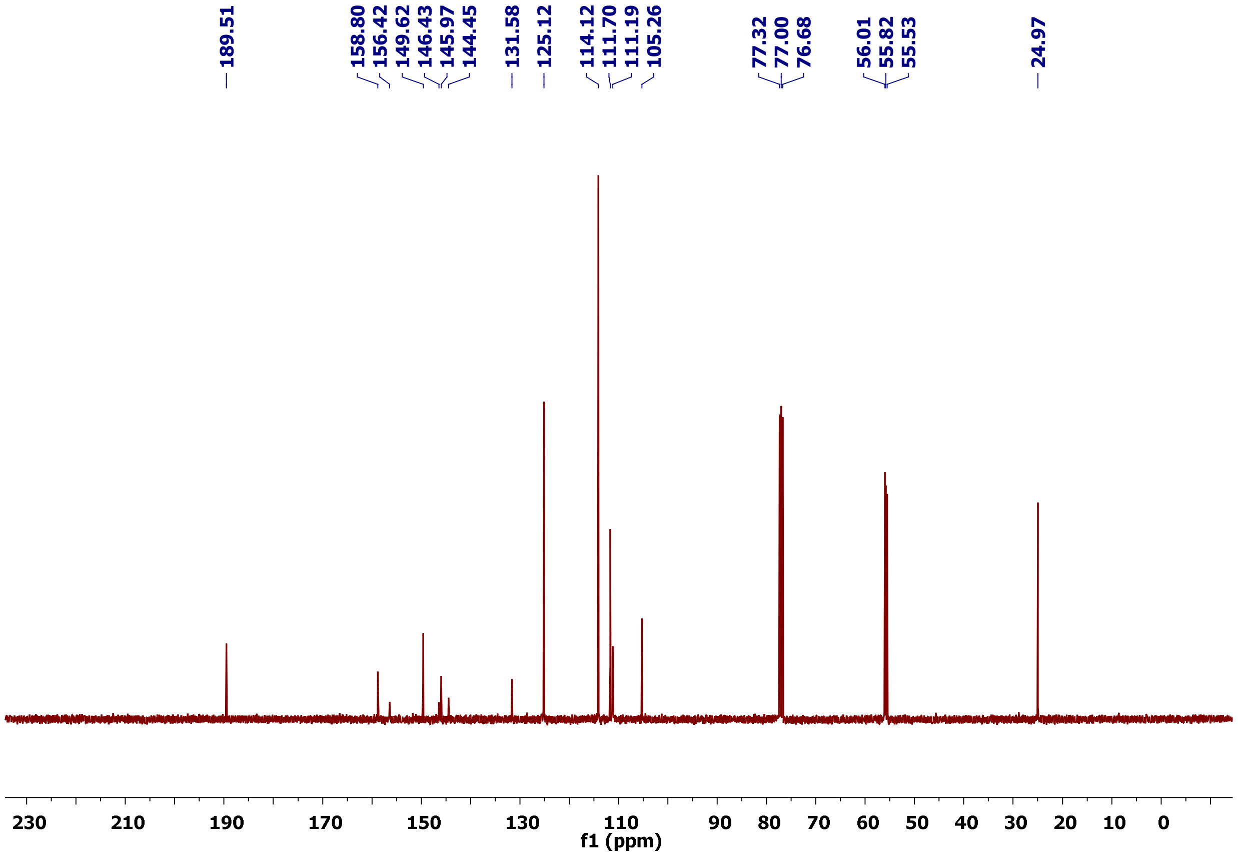

^{13}C CRAPT NMR ($CDCl_3$) spectrum of (Z)-1-(5-((3,4-dimethoxyphenyl)imino)-4-(4-methoxyphenyl)-4,5-dihydro-1,3,4-thiadiazol-2-yl)ethan-1-one

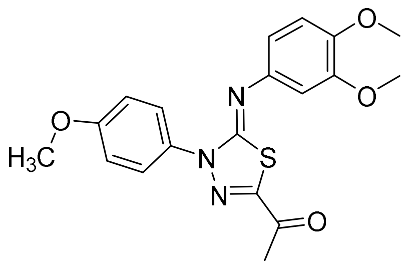

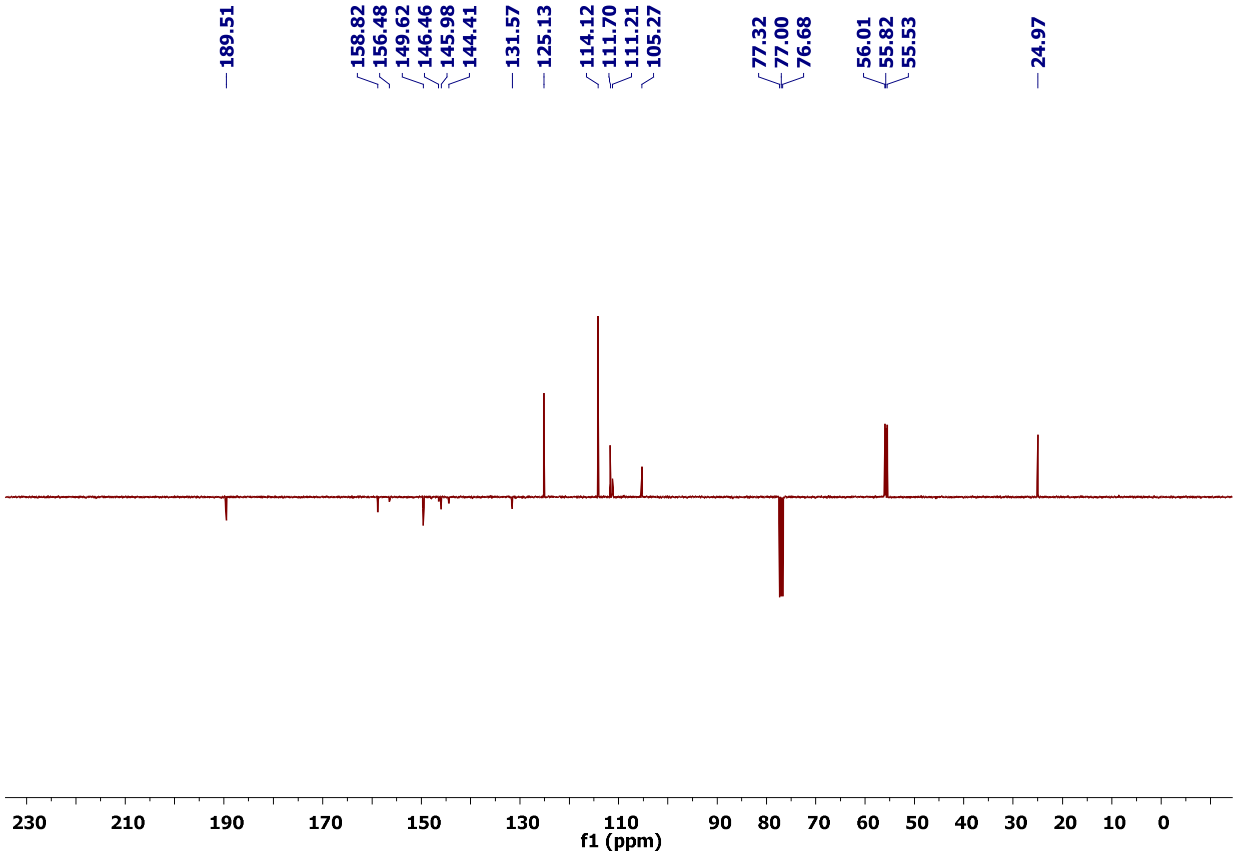

^{1}H-^{1}H gDQCOSY NMR ($CDCl_3$) spectrum of (Z)-1-(5-((3,4-dimethoxyphenyl)imino)-4-(4-methoxyphenyl)-4,5-dihydro-1,3,4-thiadiazol-2-yl)ethan-1-one

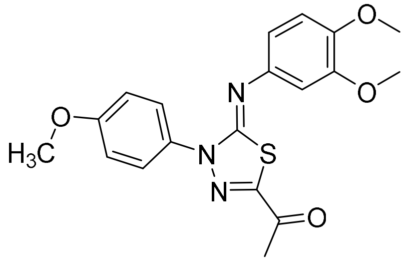

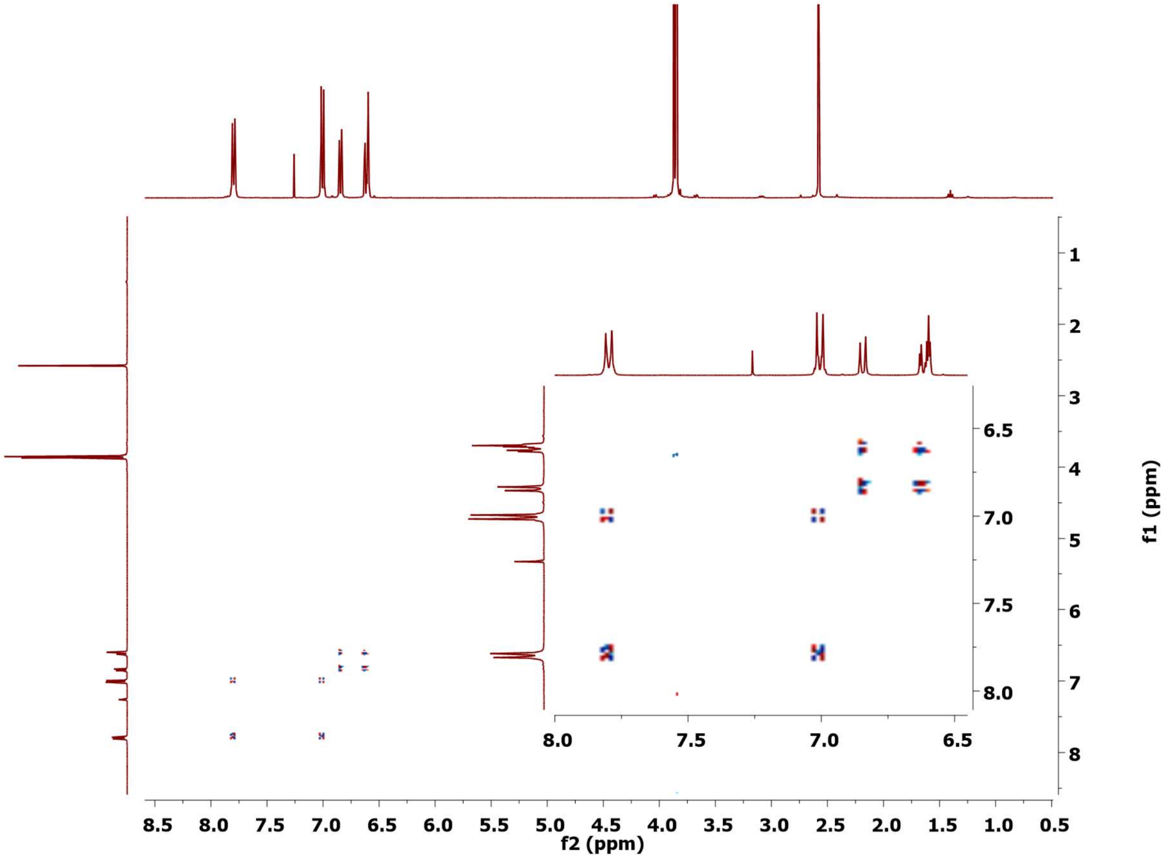

^{1}H-^{13}C-HSQC NMR ($CDCl_3$) spectrum of (Z)-1-(5-((3,4-dimethoxyphenyl)imino)-4-(4-methoxyphenyl)-4,5-dihydro-1,3,4-thiadiazol-2-yl)ethan-1-one

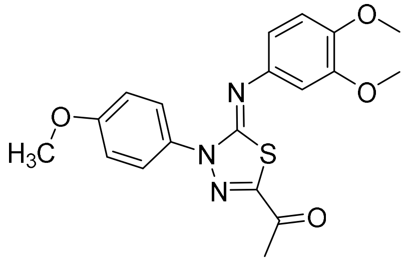

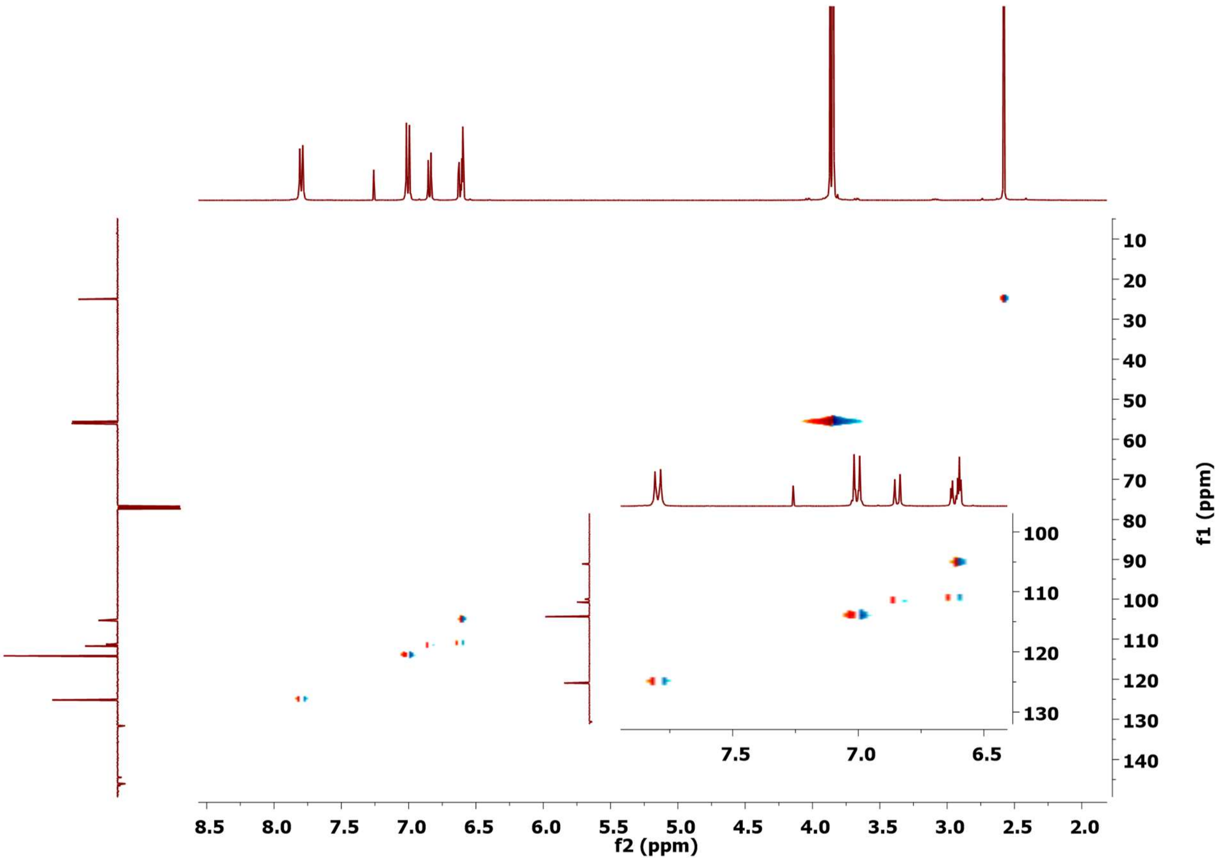

^{1}H-^{13}C-gHMBC NMR ($CDCl_3$) spectrum of (Z)-1-(5-((3,4-dimethoxyphenyl)imino)-4-(4-methoxyphenyl)-4,5-dihydro-1,3,4-thiadiazol-2-yl)ethan-1-one

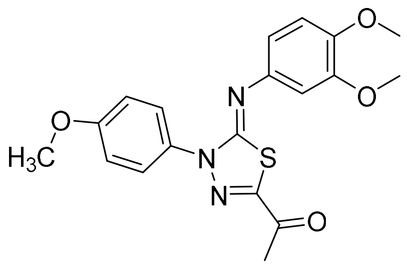

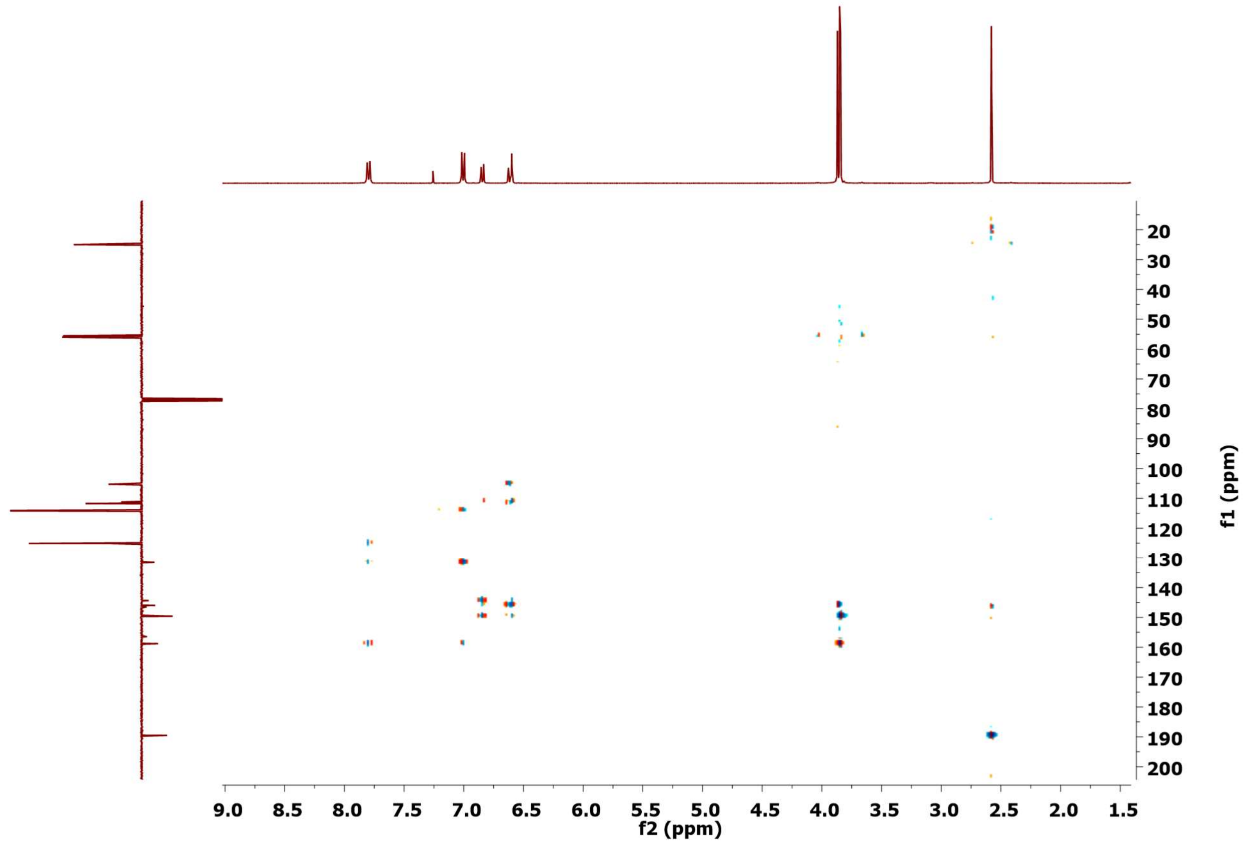

^{1}H NMR ($CDCl_3$) spectrum of (Z)-1-(4-(3,5-bis(trifluoromethyl)phenyl)-5-((2,5-dimethoxyphenyl)imino)-4,5-dihydro-1,3,4-thiadiazol-2-yl)ethan-1-one

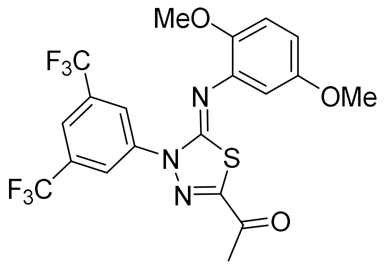

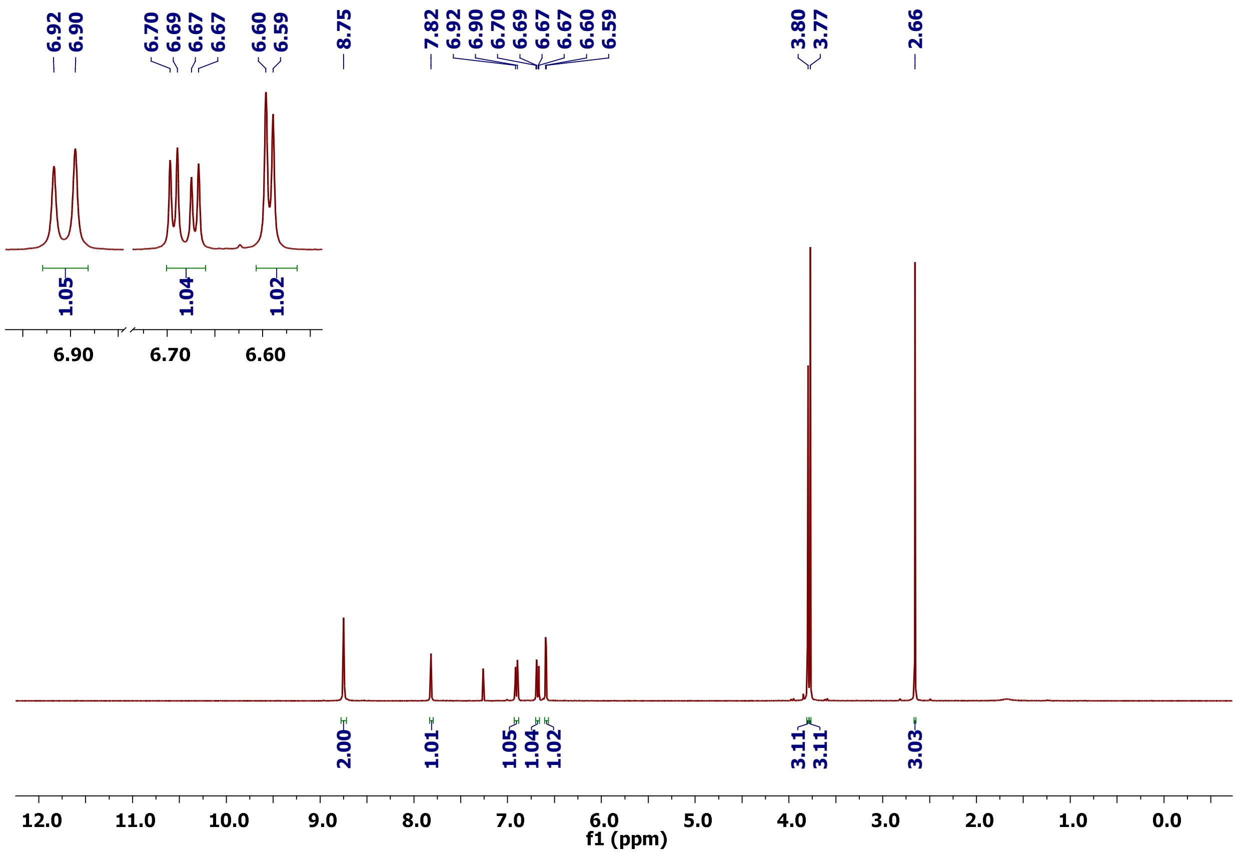

^{13}C CRAPT NMR ($CDCl_3$) spectrum of (Z)-1-(4-(3,5-bis(trifluoromethyl)phenyl)-5-((2,5-dimethoxyphenyl)imino)-4,5-dihydro-1,3,4-thiadiazol-2-yl)ethan-1-one

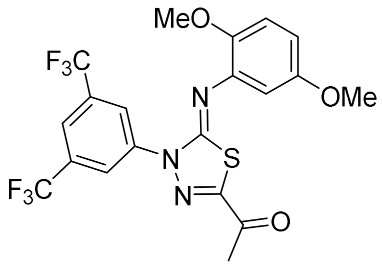

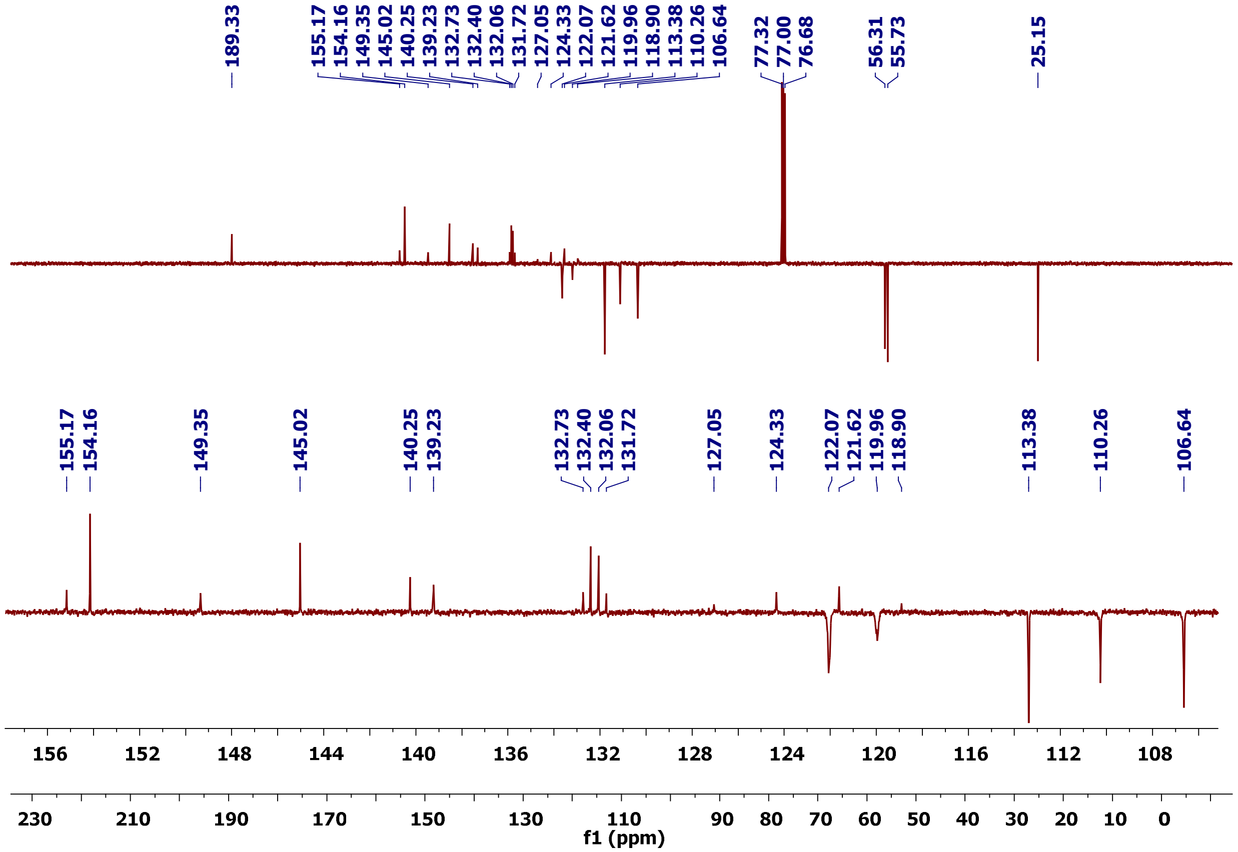

^{1}H-^{1}H gDQCOSY NMR ($CDCl_3$) spectrum of (Z)-1-(4-(3,5-bis(trifluoromethyl)phenyl)-5-((2,5-dimethoxyphenyl)imino)-4,5-dihydro-1,3,4-thiadiazol-2-yl)ethan-1-one

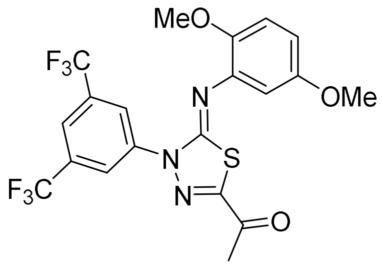

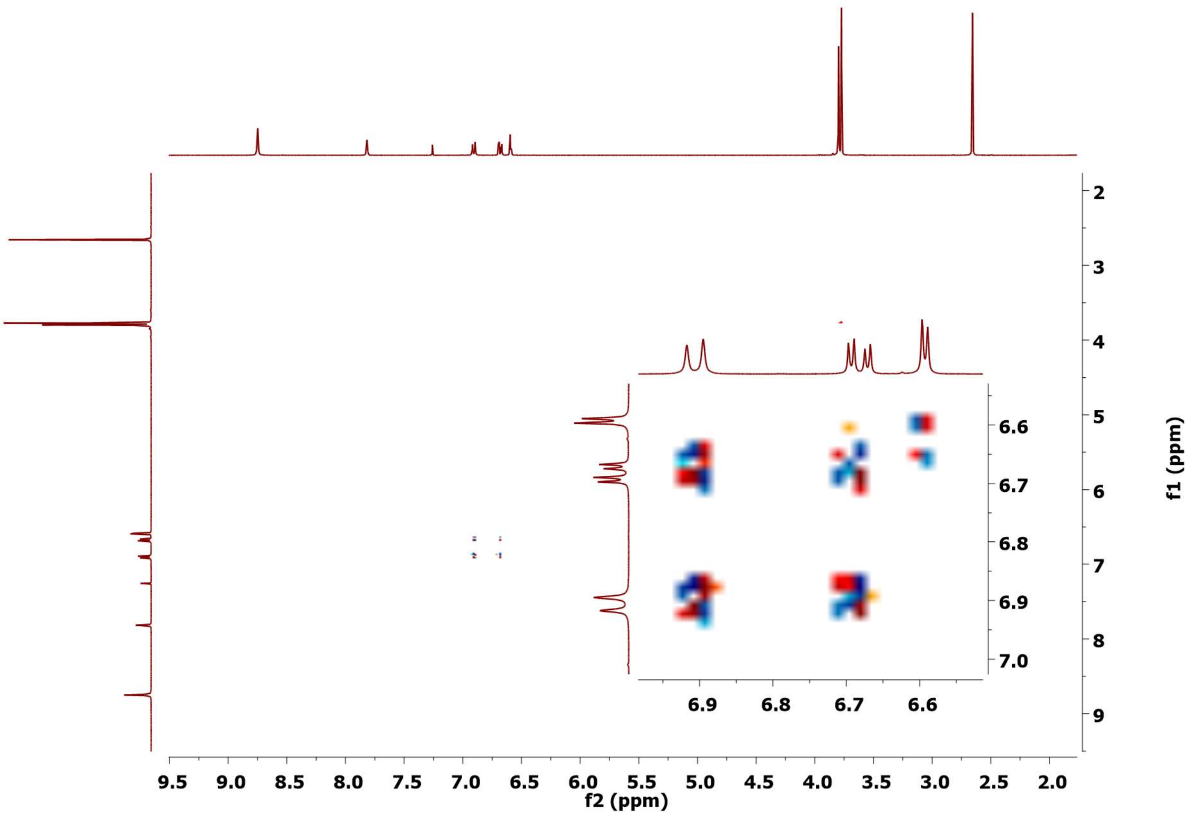

^{1}H-^{13}C-HSQC NMR ($CDCl_3$) spectrum of (Z)-1-(4-(3,5-bis(trifluoromethyl)phenyl)-5-((2,5-dimethoxyphenyl)imino)-4,5-dihydro-1,3,4-thiadiazol-2-yl)ethan-1-one

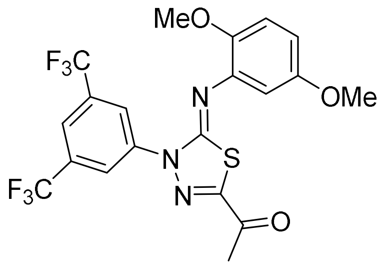

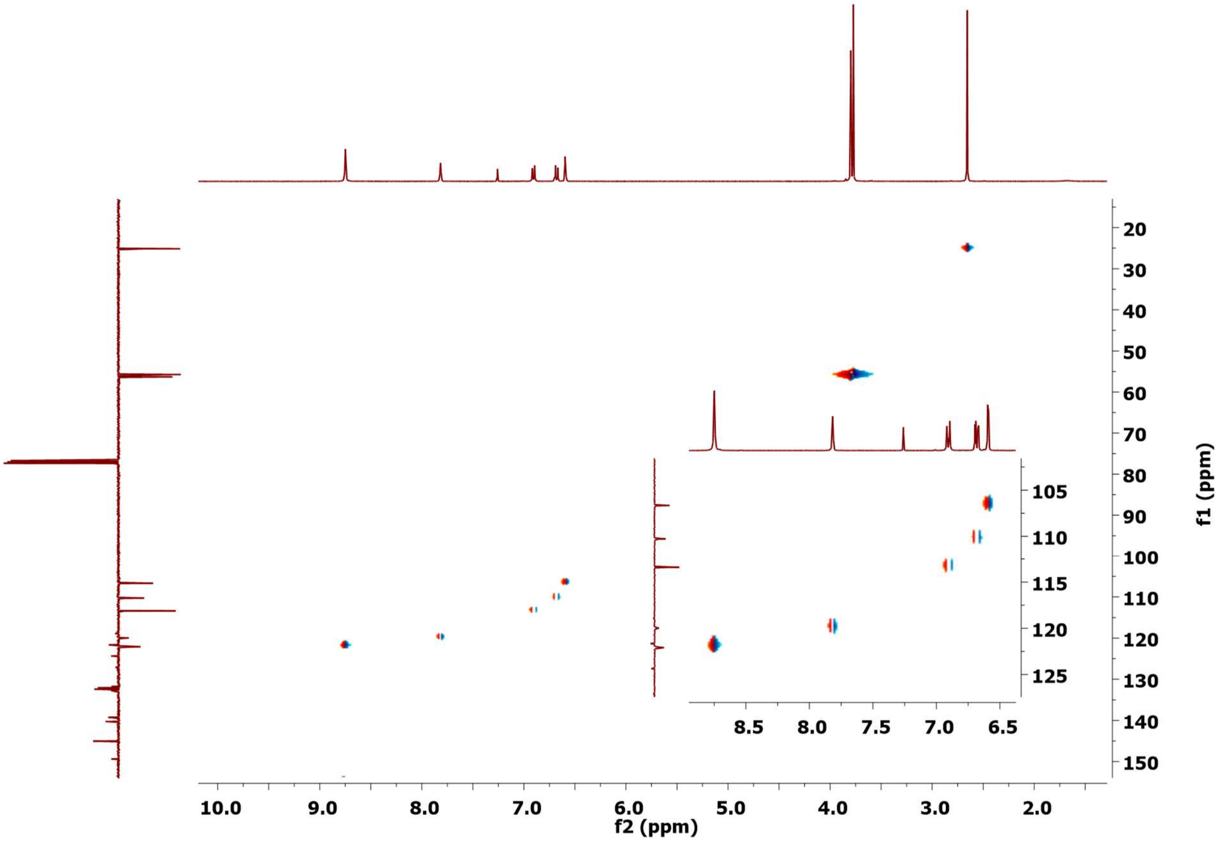

^{1}H-^{13}C-gHMBC NMR ($CDCl_3$) spectrum of (Z)-1-(4-(3,5-bis(trifluoromethyl)phenyl)-5-((2,5-dimethoxyphenyl)imino)-4,5-dihydro-1,3,4-thiadiazol-2-yl)ethan-1-one

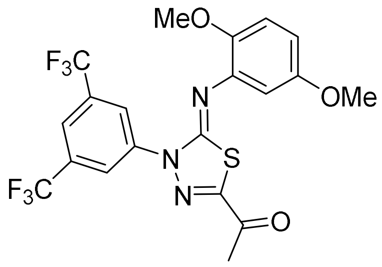

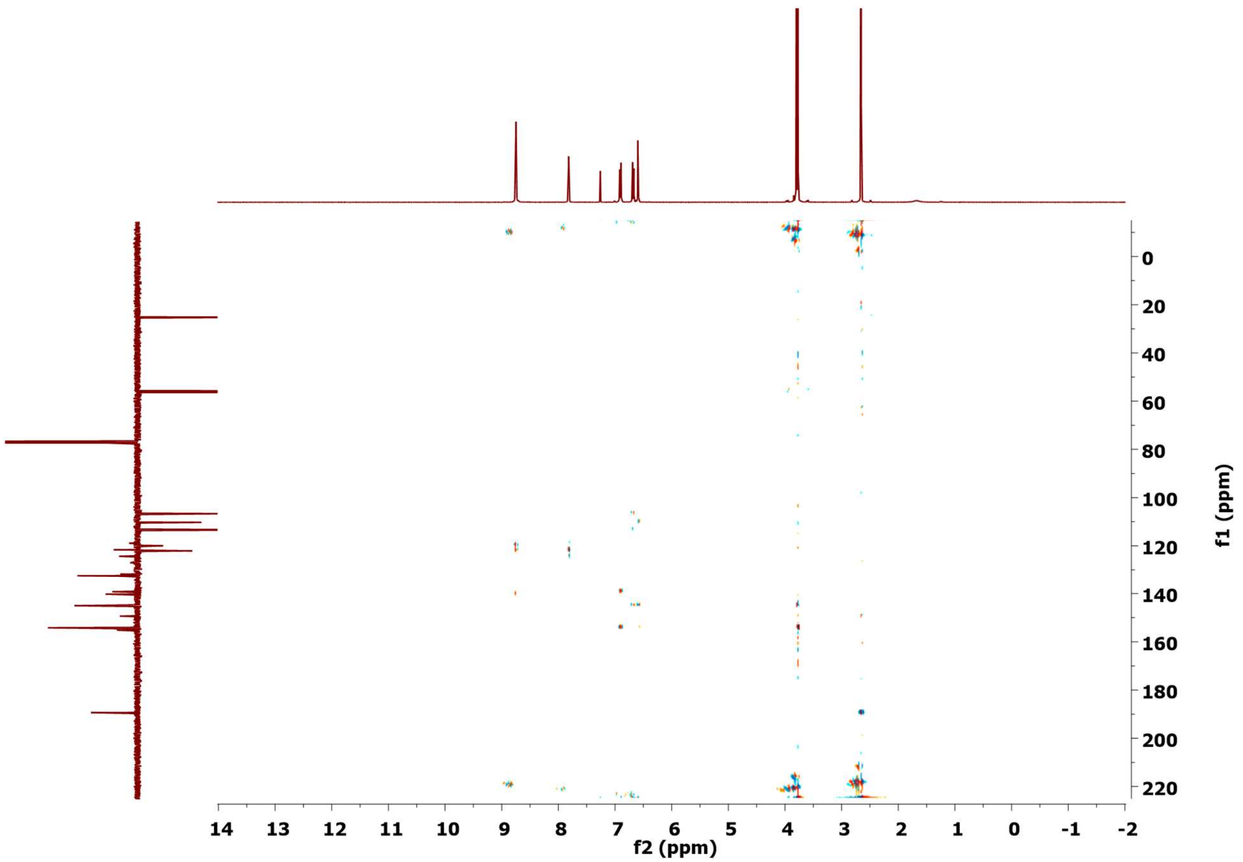

^{1}H NMR ($CDCl_3$) spectrum of ethyl (Z)-4-((5-acetyl-3-(4-bromophenyl)-1,3,4-thiadiazol-2(3H)-ylidene)amino)benzoate

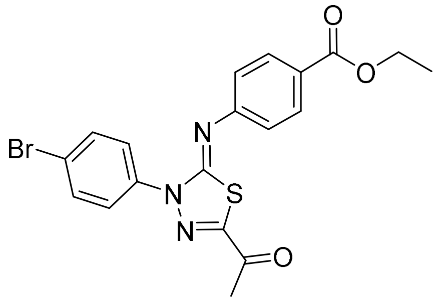

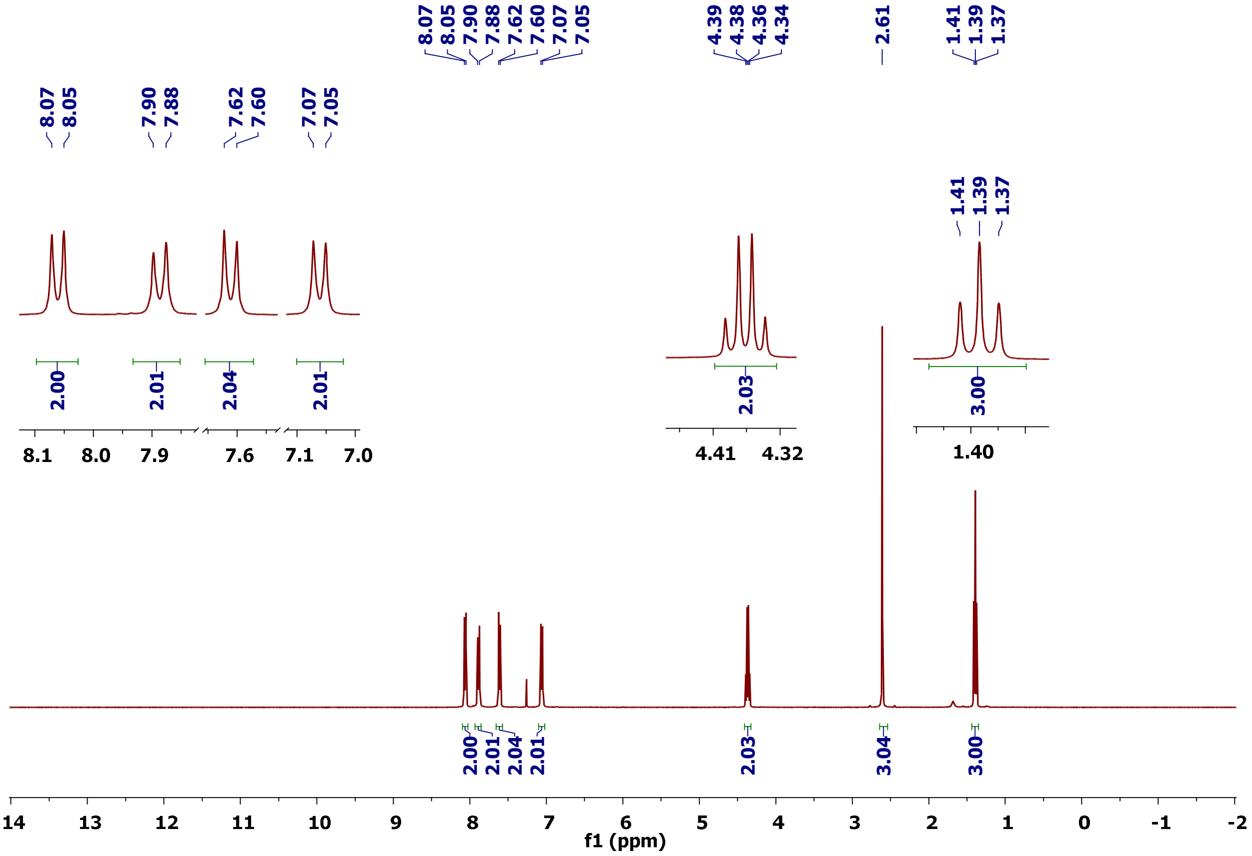

^{13}C NMR ($CDCl_3$) spectrum of ethyl (Z)-4-((5-acetyl-3-(4-bromophenyl)-1,3,4-thiadiazol-2(3H)-ylidene)amino)benzoate

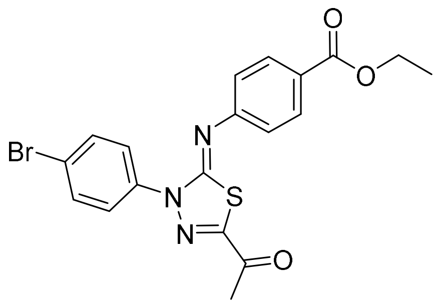

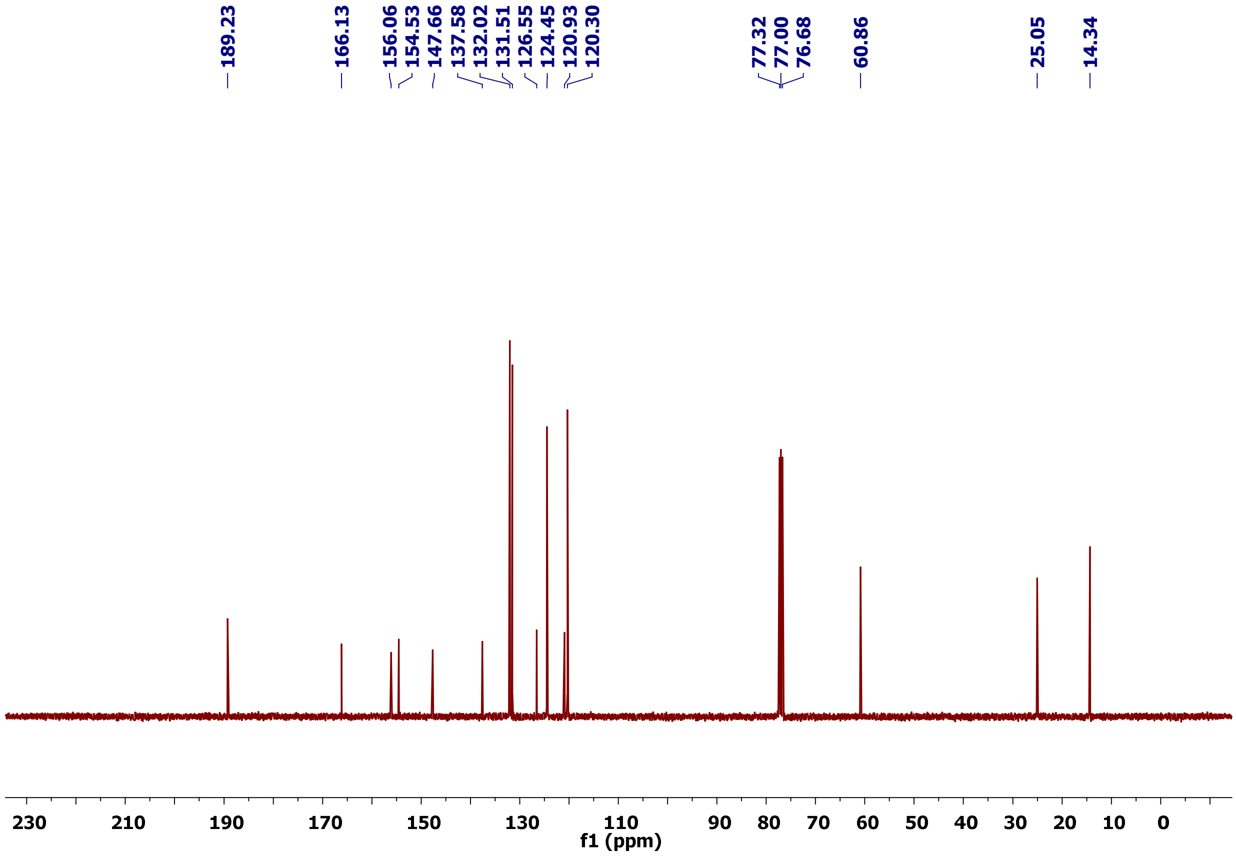

^{13}C CRAPT NMR ($CDCl_3$) spectrum of ethyl (Z)-4-((5-acetyl-3-(4-bromophenyl)-1,3,4-thiadiazol-2(3H)-ylidene)amino)benzoate

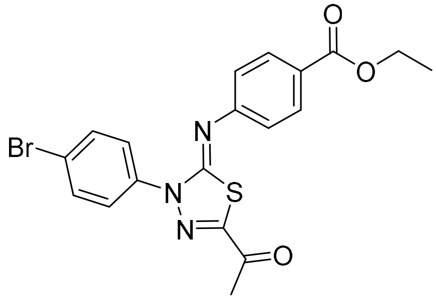

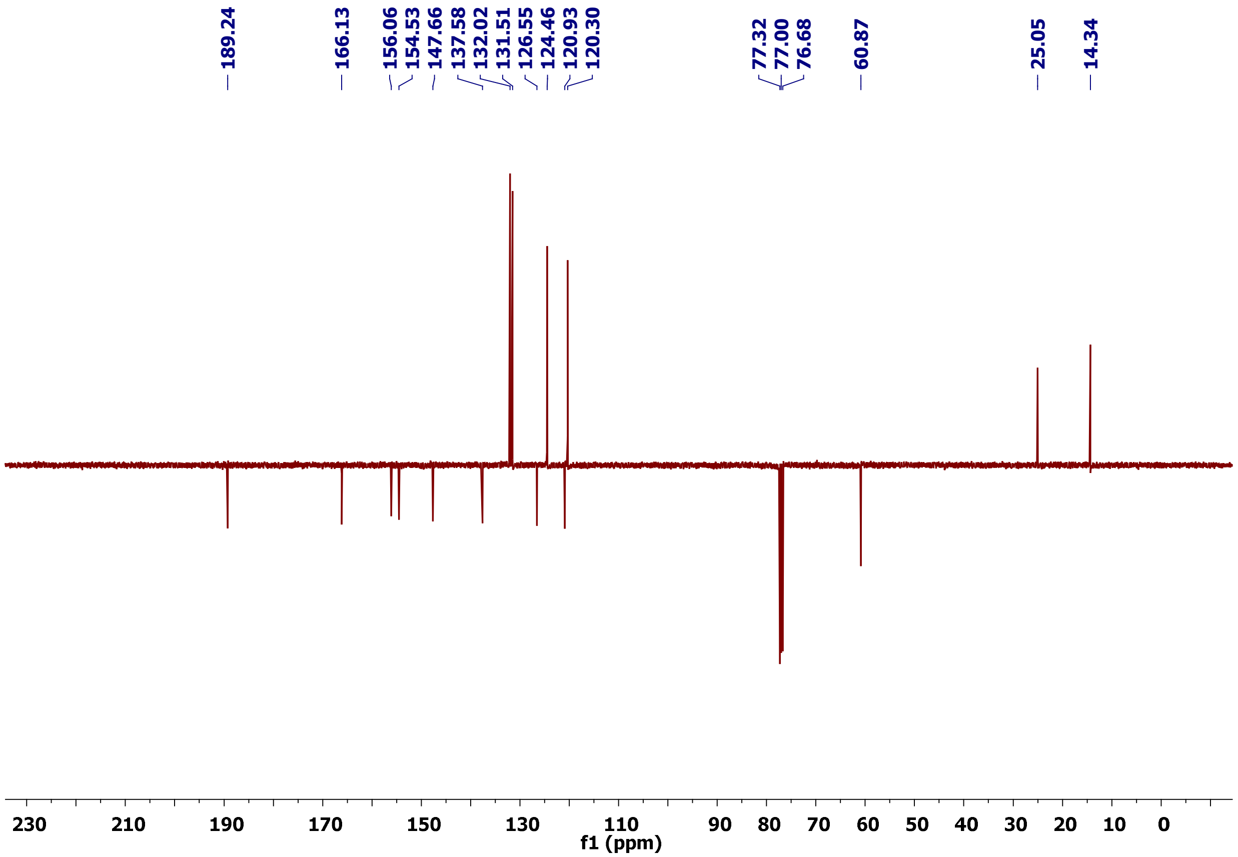

^1H-^1H gDQCOSY NMR ($CDCl_3$) spectrum of ethyl (Z)-4-((5-acetyl-3-(4-bromophenyl)-1,3,4-thiadiazol-2(3H)-ylidene)amino)benzoate

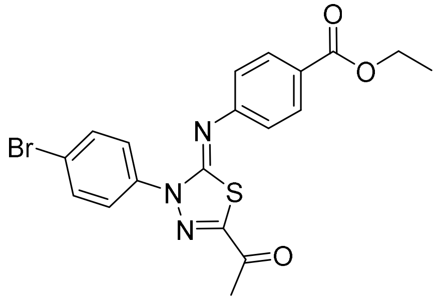

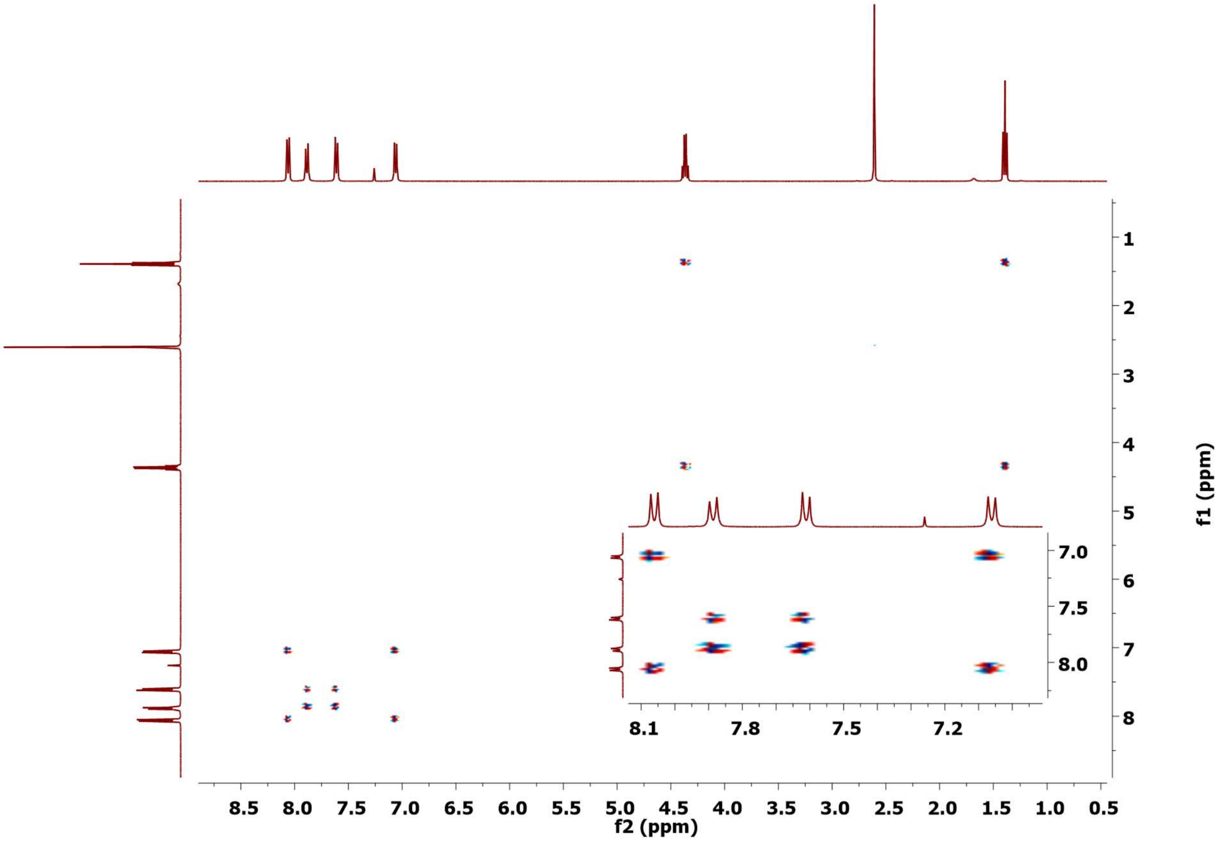

^{1}H-^{13}C-HSQC NMR ($CDCl_3$) spectrum of ethyl (Z)-4-((5-acetyl-3-(4-bromophenyl)-1,3,4-thiadiazol-2(3H)-ylidene)amino)benzoate

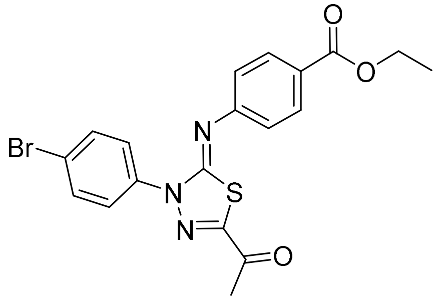

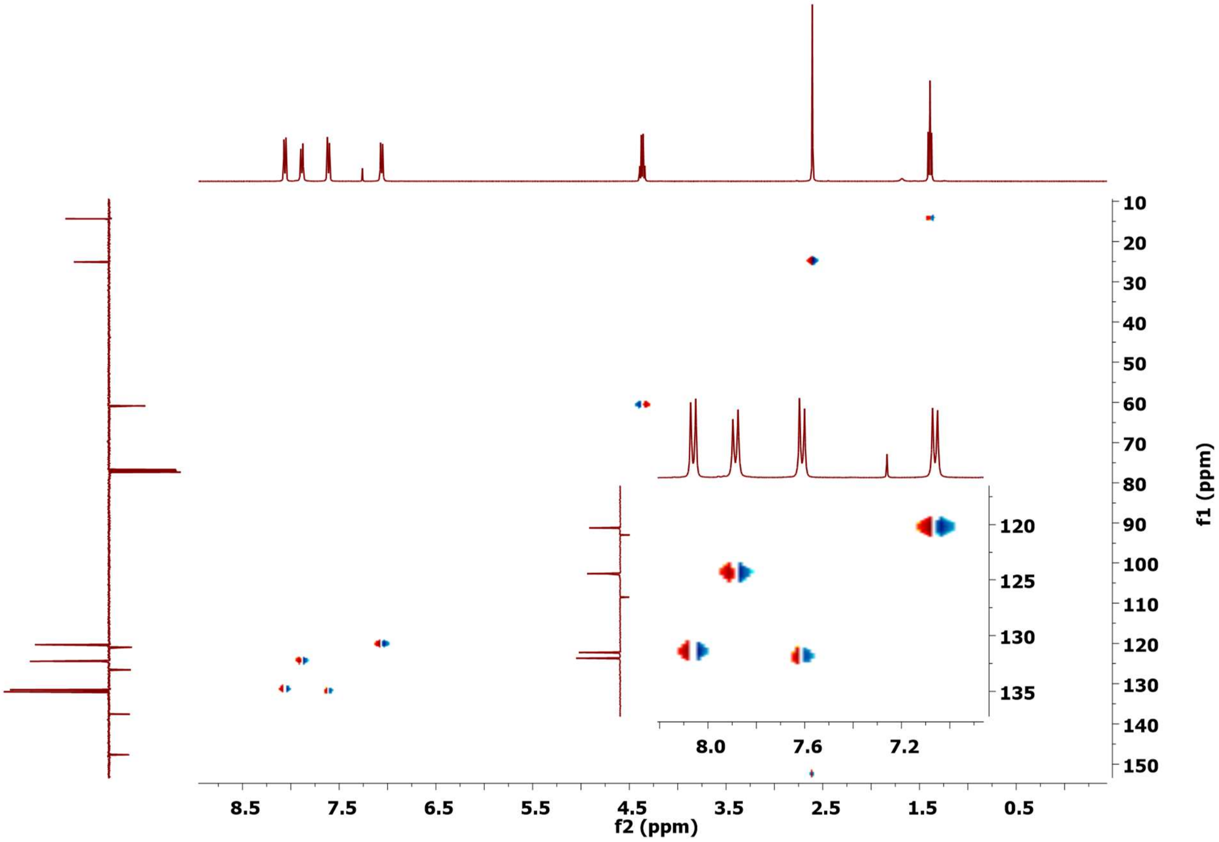

^{1}H-^{13}C-gHMBC NMR ($CDCl_3$) spectrum of ethyl (Z)-4-((5-acetyl-3-(4-bromophenyl)-1,3,4-thiadiazol-2(3H)-ylidene)amino)benzoate

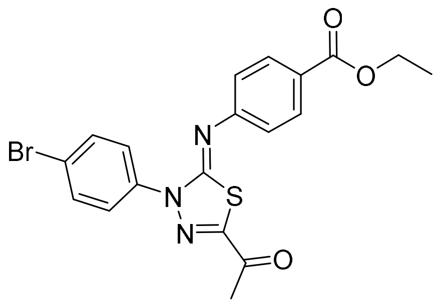

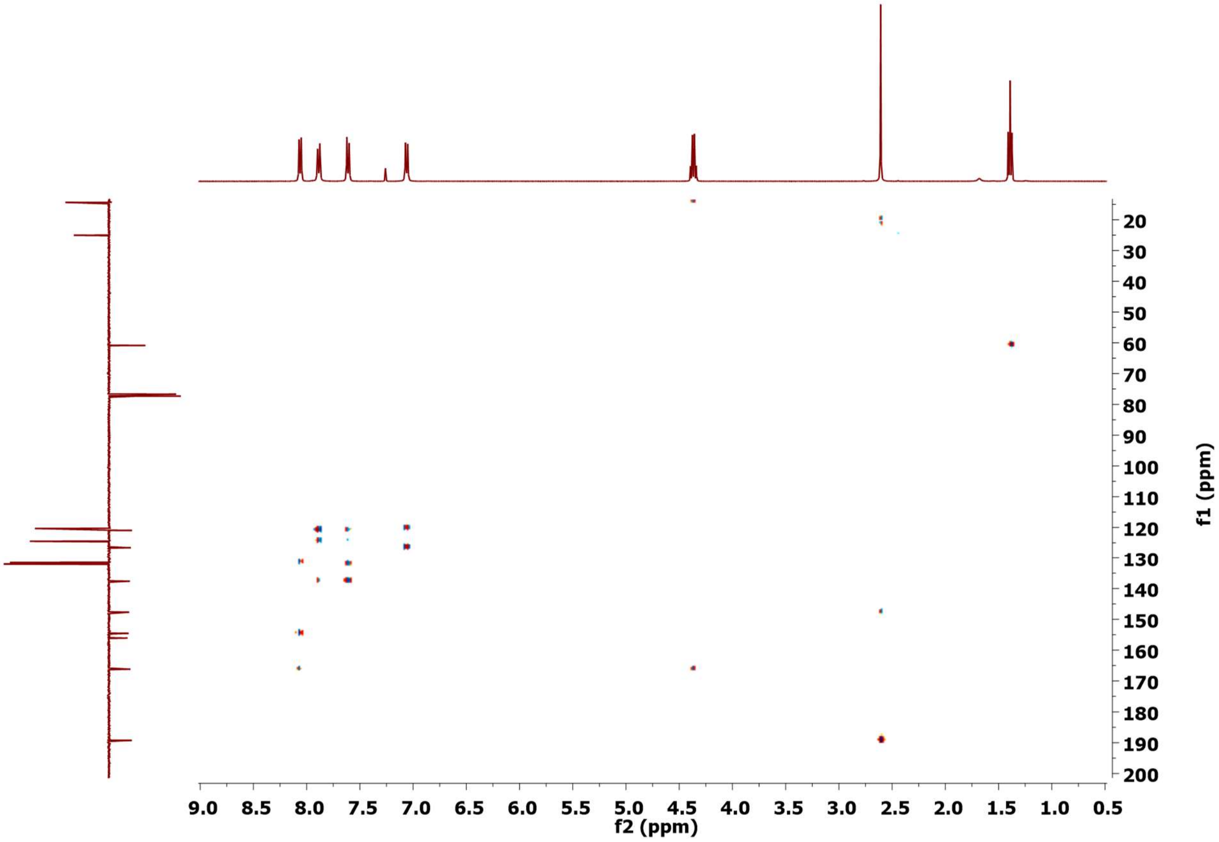

Crystal data for (Z)-N-(2,3-difluorophenyl)-2-oxopropanehydrazonoyl chloride

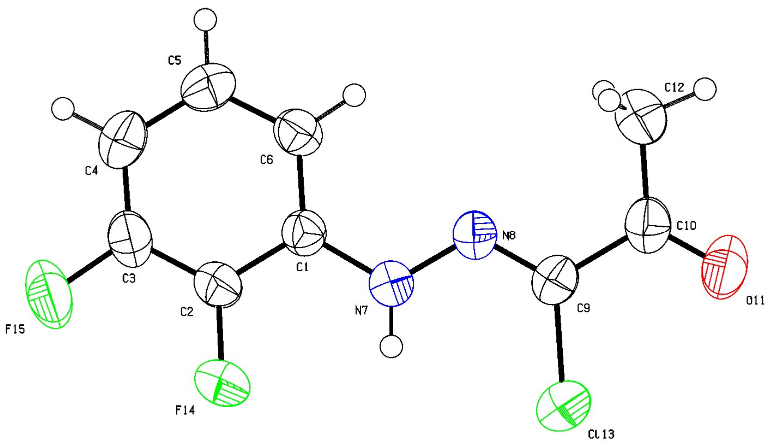

Table 1 Crystal data and structure refinement for exp_275_auto.

Identification code	exp_275_auto
Empirical formula	$C_9H_7ClF_2N_2O$
Formula weight	232.62
Temperature/K	297.00(10)
Crystal system	monoclinic
Space group	$P2_1/n$
a/Å	11.7982(4)
b/Å	7.1366(2)
c/Å	12.9116(5)
α/°	90
β/°	113.435(4)
γ/°	90
Volume/Å^3	997.47(7)
Z	4
ρ_{calc}g/cm^3	1.549
μ/mm^{-1}	3.489
F(000)	472.0
Crystal size/mm^3	0.6 × 0.6 × 0.4
Radiation	Cu Kα (λ = 1.54184)
2Θ range for data collection/°	8.598 to 155.1
Index ranges	$-14 \leq h \leq 13, -6 \leq k \leq 8, -16 \leq l \leq 15$
Reflections collected	9692
Independent reflections	2036 [R_{int} = 0.0546, R_{sigma} = 0.0348]
Data/restraints/parameters	2036/0/138
Goodness-of-fit on F^2	1.057
Final R indexes [I>=2σ (I)]	R_1 = 0.0408, wR_2 = 0.1087

Final R indexes [all data] R_1 = 0.0484, wR_2 = 0.1158
Largest diff. peak/hole / e Å^{-3} 0.20/-0.24

Table 2 Fractional Atomic Coordinates (×10^4) and Equivalent Isotropic Displacement Parameters (Å^2×10^3) for exp_275_auto. U_{eq} is defined as 1/3 of the trace of the orthogonalised U_{IJ} tensor.

Atom	*x*	*y*	*z*	U(eq)
Cl13	1709.2(4)	5065.4(8)	5934.8(5)	65.1(2)
F14	2417.8(10)	7352(2)	2818.5(10)	79.8(5)
F15	3740.2(13)	8700(2)	1710.1(10)	83.5(5)
N8	3893.4(13)	6409(2)	6171.2(12)	43.6(3)
N7	3417.7(13)	6666(2)	5058.4(12)	48.5(4)
O11	3169.5(15)	4768(3)	8362.9(14)	73.6(5)
C1	4171.0(14)	7307(2)	4535.2(13)	41.4(4)
C9	3238.7(16)	5731(3)	6656.3(15)	46.0(4)
C6	5426.1(15)	7639(3)	5100.6(15)	46.4(4)
C2	3639.4(15)	7669(3)	3385.5(15)	49.4(4)
C10	3778.6(18)	5450(3)	7898.9(16)	52.7(5)
C5	6100.2(16)	8333(3)	4521.6(17)	52.3(5)
C4	5560.8(18)	8715(3)	3381.1(17)	54.9(5)
C3	4323.2(18)	8364(3)	2831.8(15)	54.2(5)
C12	5090(2)	6051(4)	8523.7(18)	70.8(6)

Table 3 Anisotropic Displacement Parameters (Å^2×10^3) for exp_275_auto. The Anisotropic displacement factor exponent takes the form: $-2\pi^2[h^2a^{*2}U_{11}+2hka^*b^*U_{12}+\ldots]$.

Atom	U_{11}	U_{22}	U_{33}	U_{23}	U_{13}	U_{12}
Cl13	50.4(3)	84.0(4)	64.1(3)	-1.4(2)	26.3(2)	-11.5(2)
F14	45.4(6)	132.9(13)	48.6(6)	11.0(7)	5.5(5)	-7.4(7)
F15	89.7(9)	117.4(12)	44.5(6)	21.7(7)	27.9(6)	13.2(9)
N8	47.0(7)	45.7(8)	40.3(7)	1.9(6)	19.5(6)	0.8(6)
N7	41.7(7)	63.2(10)	39.8(7)	4.1(7)	15.4(6)	-3.3(7)
O11	74.0(10)	94.9(12)	65.3(9)	19.7(8)	41.8(8)	1.3(8)
C1	42.2(8)	43.6(9)	39.8(8)	0.7(7)	17.7(6)	2.3(7)
C9	46.3(8)	47.1(10)	49.5(9)	0.8(8)	24.1(7)	0.4(8)
C6	43.2(8)	51.2(10)	41.2(8)	0.2(7)	13.2(7)	4.1(8)
C2	41.2(8)	62.2(11)	40.5(8)	3.7(8)	11.8(7)	3.2(8)
C10	59.6(10)	55.7(11)	49.2(10)	5.7(8)	28.4(8)	6.3(9)
C5	42.3(8)	54.5(11)	62.6(11)	-3.3(9)	23.5(8)	-0.1(8)
C4	60.0(10)	54.0(11)	63.0(11)	3.7(9)	37.5(9)	3.4(9)
C3	61.9(10)	62.2(12)	41.6(9)	8.6(8)	24.0(8)	9.8(9)

Table 3 Anisotropic Displacement Parameters (Å^2×10^3) for exp_275_auto. The Anisotropic displacement factor exponent takes the form: $-2\pi^2[h^2a^{*2}U_{11}+2hka^*b^*U_{12}+\ldots]$.

Atom	U_{11}	U_{22}	U_{33}	U_{23}	U_{13}	U_{12}
C12	62.2(11)	99.6(19)	48.6(10)	3.0(11)	19.9(9)	-2.8(12)

Table 4 Bond Lengths for exp_275_auto.

Atom	Atom	Length/Å	Atom	Atom	Length/Å
Cl13	C9	1.7357(18)	C1	C2	1.387(2)
F14	C2	1.3509(19)	C9	C10	1.486(3)
F15	C3	1.355(2)	C6	C5	1.382(3)
N8	N7	1.331(2)	C2	C3	1.366(3)
N8	C9	1.268(2)	C10	C12	1.495(3)
N7	C1	1.390(2)	C5	C4	1.379(3)
O11	C10	1.206(2)	C4	C3	1.369(3)
C1	C6	1.386(2)			

Table 5 Bond Angles for exp_275_auto.

Atom	Atom	Atom	Angle/°	Atom	Atom	Atom	Angle/°
C9	N8	N7	120.50(15)	F14	C2	C3	120.12(16)
N8	N7	C1	119.55(14)	C3	C2	C1	121.19(16)
C6	C1	N7	123.77(15)	O11	C10	C9	120.44(18)
C6	C1	C2	117.73(15)	O11	C10	C12	122.75(19)
C2	C1	N7	118.49(14)	C9	C10	C12	116.81(17)
N8	C9	Cl13	123.16(14)	C4	C5	C6	121.88(17)
N8	C9	C10	120.07(16)	C3	C4	C5	117.45(17)
C10	C9	Cl13	116.77(13)	F15	C3	C2	117.98(17)
C5	C6	C1	120.04(16)	F15	C3	C4	120.31(17)
F14	C2	C1	118.67(15)	C2	C3	C4	121.70(17)

Table 6 Torsion Angles for exp_275_auto.

A	B	C	D	Angle/°	A	B	C	D	Angle/°
Cl13	C9	C10	O11	0.9(3)	N7	C1	C2	C3	177.65(18)
Cl13	C9	C10	C12	-179.06(16)	C1	C6	C5	C4	0.1(3)
F14	C2	C3	F15	-0.6(3)	C1	C2	C3	F15	-179.41(18)
F14	C2	C3	C4	179.47(19)	C1	C2	C3	C4	0.6(3)

Table 6 Torsion Angles for exp_275_auto.

A	B	C	D	Angle/°	A	B	C	D	Angle/°
N8	N7	C1	C6	2.6(3)	C9	N8	N7	C1	-176.19(17)
N8	N7	C1	C2	-176.24(17)	C6	C1	C2	F14	179.95(18)
N8	C9	C10	O11	-178.38(19)	C6	C1	C2	C3	-1.2(3)
N8	C9	C10	C12	1.6(3)	C6	C5	C4	C3	-0.7(3)
N7	N8	C9	Cl13	0.8(3)	C2	C1	C6	C5	0.8(3)
N7	N8	C9	C10	180.00(17)	C5	C4	C3	F15	-179.63(19)
N7	C1	C6	C5	-177.96(17)	C5	C4	C3	C2	0.3(3)
N7	C1	C2	F14	-1.2(3)					

Table 7 Hydrogen Atom Coordinates (Å×10^4) and Isotropic Displacement Parameters (Å^2×10^3) for exp_275_auto.

Atom	*x*	*y*	*z*	U(eq)
H7	2649.58	6436.85	4665.66	58
H6	5814.52	7396.07	5869.98	56
H5	6941.56	8547.22	4911.95	63
H4	6020.23	9194.05	2999.97	66
H12A	5158.84	7372.46	8421.82	106
H12B	5610.91	5383.07	8237.65	106
H12C	5342.84	5783.46	9312.7	106

Crystal data for (4-ethoxyphenyl)carbamothioyl cyanide

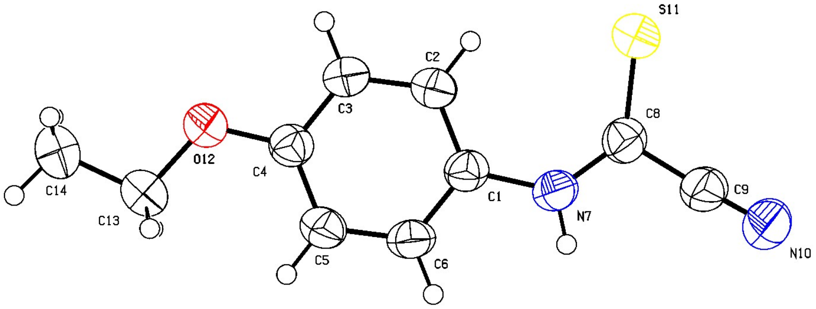

Table 1 Crystal data and structure refinement for exp_286_auto.

Identification code	exp_286_auto
Empirical formula	$C_{10}H_{10}N_2OS$
Formula weight	206.26
Temperature/K	297.00(10)
Crystal system	orthorhombic
Space group	$P2_12_12_1$
a/Å	4.0279(2)
b/Å	15.3918(8)
c/Å	16.6883(8)
α/°	90
β/°	90
γ/°	90
Volume/Å^3	1034.63(10)
Z	4
ρ_{calc}g/cm^3	1.324
μ/mm^{-1}	2.523
F(000)	432.0
Crystal size/mm^3	0.5 × 0.3 × 0.2
Radiation	Cu Kα (λ = 1.54184)
2Θ range for data collection/°	7.814 to 153.874
Index ranges	$-4 \leq h \leq 3$, $-18 \leq k \leq 18$, $-21 \leq l \leq 19$
Reflections collected	5226
Independent reflections	1818 [R_{int} = 0.0321, R_{sigma} = 0.0308]
Data/restraints/parameters	1818/0/130
Goodness-of-fit on F^2	1.096
Final R indexes [I>=2σ (I)]	R_1 = 0.0352, wR_2 = 0.0848

Final R indexes [all data]	R_1 = 0.0441, wR_2 = 0.0892
Largest diff. peak/hole / e Å^{-3}	0.14/-0.16
Flack parameter	0.28(3)

Table 2 Fractional Atomic Coordinates (×10^4) and Equivalent Isotropic Displacement Parameters (Å^2×10^3) for exp_286_auto. U_{eq} is defined as 1/3 of the trace of the orthogonalised U_{IJ} tensor.

Atom	*x*	*y*	*z*	U(eq)
S11	844(3)	5363.2(5)	6531.9(5)	74.7(3)
O12	8439(6)	2806.5(13)	3701.9(11)	59.5(6)
N7	3145(7)	5802.8(14)	5050.7(14)	54.0(7)
C4	7215(8)	3571.0(18)	4008.8(17)	48.7(7)
C1	4541(8)	5025.0(16)	4739.4(15)	47.7(7)
N10	-520(9)	7548.4(17)	5861.1(17)	79.6(10)
C6	5954(9)	5080.3(17)	3990.1(16)	55.4(7)
C8	1653(8)	5971.0(18)	5747.3(17)	52.5(8)
C5	7327(8)	4361.3(18)	3619.0(17)	54.4(8)
C3	5838(9)	3515.3(17)	4761.7(17)	55.2(7)
C2	4491(9)	4231.4(17)	5133.4(17)	55.3(8)
C13	9836(9)	2808(2)	2915.4(18)	60.9(9)
C9	490(10)	6868(2)	5800.4(18)	60.4(9)
C14	10876(12)	1892(2)	2741(2)	80.8(11)

Table 3 Anisotropic Displacement Parameters (Å^2×10^3) for exp_286_auto. The Anisotropic displacement factor exponent takes the form: $-2\pi^2[h^2a^{*2}U_{11}+2hka^*b^*U_{12}+\ldots]$.

Atom	U_{11}	U_{22}	U_{33}	U_{23}	U_{13}	U_{12}
S11	106.3(8)	61.4(5)	56.4(5)	6.8(4)	19.9(5)	9.9(5)
O12	76.9(15)	54.2(11)	47.5(11)	2.1(8)	7.2(11)	8.9(12)
N7	73.4(18)	42.5(12)	46.1(13)	3.4(10)	-0.9(13)	-0.8(12)
C4	52.3(18)	50.1(14)	43.7(14)	0.9(13)	-3.5(14)	-1.0(14)
C1	53.9(19)	44.2(13)	45.0(13)	0.8(11)	-3.7(14)	-4.2(14)
N10	115(3)	60.3(16)	63.5(16)	1.0(14)	1.2(19)	17.2(18)
C6	73(2)	46.8(14)	46.0(14)	9.3(12)	-1.2(18)	-3.2(16)
C8	60(2)	49.2(14)	48.4(15)	-3.2(12)	-5.9(15)	1.3(14)
C5	67(2)	56.1(16)	40.3(14)	3.3(12)	1.9(15)	-1.4(15)
C3	68(2)	45.9(14)	51.9(15)	7.7(13)	5.7(17)	-1.2(16)
C2	70(2)	51.7(15)	44.4(14)	4.9(12)	6.5(15)	-1.8(16)
C13	68(2)	65.0(17)	49.4(16)	0.4(14)	8.4(16)	1.2(17)
C9	81(2)	53.1(16)	47.6(15)	1.6(13)	-3.8(17)	1.2(17)
C14	96(3)	73(2)	74(2)	-3.7(18)	29(2)	14(2)

Table 4 Bond Lengths for exp_286_auto.

Atom	Atom	Length/Å	Atom	Atom	Length/Å
S11	C8	1.642(3)	C1	C6	1.377(4)
O12	C4	1.375(3)	C1	C2	1.387(3)
O12	C13	1.428(3)	N10	C9	1.128(4)
N7	C1	1.421(3)	C6	C5	1.384(4)
N7	C8	1.334(4)	C8	C9	1.461(4)
C4	C5	1.380(4)	C3	C2	1.376(4)
C4	C3	1.376(4)	C13	C14	1.499(4)

Table 5 Bond Angles for exp_286_auto.

Atom	Atom	Atom	Angle/°	Atom	Atom	Atom	Angle/°
C4	O12	C13	118.8(2)	N7	C8	S11	132.4(2)
C8	N7	C1	131.3(2)	N7	C8	C9	112.4(2)
O12	C4	C5	124.5(3)	C9	C8	S11	115.3(2)
O12	C4	C3	115.6(2)	C4	C5	C6	118.8(3)
C3	C4	C5	119.9(3)	C2	C3	C4	121.4(3)
C6	C1	N7	116.3(2)	C3	C2	C1	119.1(3)
C6	C1	C2	119.4(3)	O12	C13	C14	106.6(3)
C2	C1	N7	124.2(3)	N10	C9	C8	177.0(4)
C1	C6	C5	121.5(2)				

Table 6 Torsion Angles for exp_286_auto.

A	B	C	D	Angle/°	A	B	C	D	Angle/°
O12	C4	C5	C6	179.0(3)	C6	C1	C2	C3	-0.5(5)
O12	C4	C3	C2	-179.3(3)	C8	N7	C1	C6	-179.1(3)
N7	C1	C6	C5	-178.6(3)	C8	N7	C1	C2	2.3(5)
N7	C1	C2	C3	178.1(3)	C5	C4	C3	C2	1.4(5)
C4	O12	C13	C14	-178.8(3)	C3	C4	C5	C6	-1.8(5)
C4	C3	C2	C1	-0.2(5)	C2	C1	C6	C5	0.1(5)
C1	N7	C8	S11	0.8(5)	C13	O12	C4	C5	-2.2(5)
C1	N7	C8	C9	-178.0(3)	C13	O12	C4	C3	178.5(3)
C1	C6	C5	C4	1.1(5)					

Table 7 Hydrogen Atom Coordinates (Å×10^4) and Isotropic Displacement Parameters (Å^2×10^3) for exp_286_auto.

Atom	*x*	*y*	*z*	U(eq)
H7	3287.57	6242.39	4734.46	65
H6	5987.4	5613.13	3727.37	67
H5	8307.11	4409.56	3116.35	65
H3	5816.67	2982.64	5024.74	66
H2	3560.24	4183.98	5641.54	66
H13A	11736.45	3194.72	2892.38	73
H13B	8206.41	3002.04	2526.58	73
H14A	11830.21	1863.04	2214.79	121
H14B	8974.37	1517.49	2767.73	121
H14C	12489.7	1709.87	3129.75	121

Crystal data for (Z)-1-(5-((3-fluorophenyl)imino)-4-(4-iodophenyl)-4,5-dihydro-1,3,4-thiadiazol-2-yl)ethan-1-one

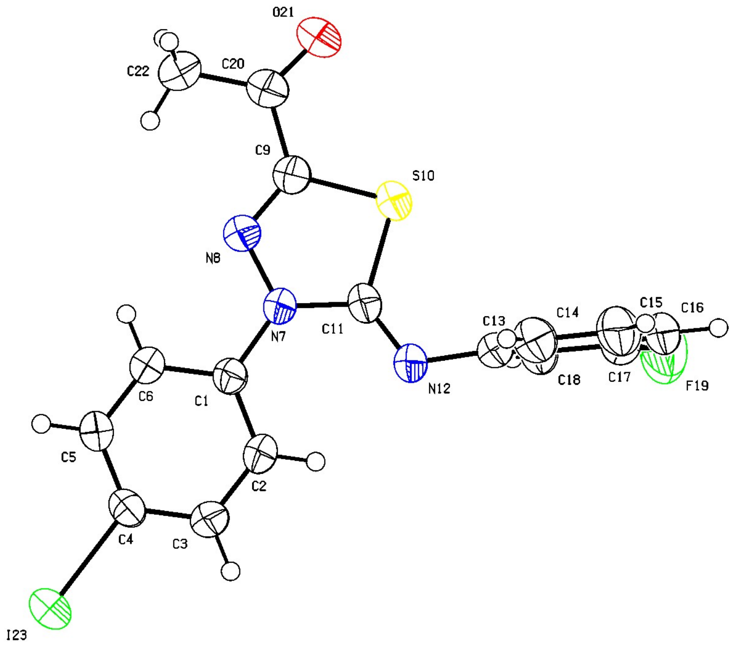

Table 1 Crystal data and structure refinement for exp_256_auto "Deposition Number 2178549".

Identification code	exp_256_auto
Empirical formula	$C_{16}H_{11}FIN_3OS$
Formula weight	439.24
Temperature/K	297.00(10)
Crystal system	monoclinic
Space group	$P2_1/c$
a/Å	11.2549(3)
b/Å	19.3773(4)
c/Å	7.6592(2)
α/°	90
β/°	102.691(3)
γ/°	90
Volume/Å^3	1629.58(7)
Z	4
ρ_{calc}g/cm^3	1.790
μ/mm^{-1}	16.799
F(000)	856.0
Crystal size/mm^3	0.8 × 0.5 × 0.5

Radiation	Cu Kα (λ = 1.54184)
2Θ range for data collection/°	8.052 to 155.282
Index ranges	-13 ≤ h ≤ 12, -24 ≤ k ≤ 16, -9 ≤ l ≤ 9
Reflections collected	14346
Independent reflections	3262 [R_{int} = 0.0593, R_{sigma} = 0.0439]
Data/restraints/parameters	3262/0/210
Goodness-of-fit on F^2	1.060
Final R indexes [I>=2σ (I)]	R_1 = 0.0383, wR_2 = 0.1031
Final R indexes [all data]	R_1 = 0.0434, wR_2 = 0.1077
Largest diff. peak/hole / e $Å^{-3}$	0.78/-1.06

Table 2 Fractional Atomic Coordinates (×10^4) and Equivalent Isotropic Displacement Parameters (Å^2×10^3) for exp_256_auto. U_{eq} is defined as 1/3 of the trace of the orthogonalised U_{IJ} tensor.

Atom	*x*	*y*	*z*	U(eq)
I23	1568.4(2)	366.8(2)	8208.9(4)	61.45(15)
S10	5372.6(8)	4305.7(5)	7366.3(15)	56.1(3)
N7	4073(2)	3250.3(15)	7805(4)	44.9(7)
N8	3370(3)	3810.0(15)	7949(4)	47.1(7)
O21	3963(3)	5569.4(17)	7787(6)	82.8(11)
F19	8868(3)	3465(2)	3856(5)	108.3(11)
N12	6065(3)	2958.3(17)	7516(5)	53.7(8)
C1	3555(3)	2585.6(17)	7940(4)	40.1(7)
C4	2419(3)	1327.0(19)	8105(5)	46.5(8)
C2	4112(3)	1978.9(19)	7596(5)	49.7(8)
C3	3556(3)	1354.3(19)	7691(6)	50.5(8)
C6	2420(3)	2554.6(19)	8392(5)	49.6(8)
C11	5251(3)	3399(2)	7560(5)	45.9(8)
C5	1859(3)	1927(2)	8460(5)	51.8(9)
C9	3912(3)	4375(2)	7773(5)	48.6(8)
C13	7226(3)	3190.6(19)	7326(6)	50.2(9)
C18	7468(4)	3253(2)	5644(6)	59.5(10)
C20	3378(4)	5064(2)	7943(6)	56.4(9)
C17	8619(4)	3436(2)	5499(7)	64.7(11)
C16	9540(4)	3575(2)	6923(7)	67.6(12)
C22	2138(4)	5084(2)	8332(7)	64.3(11)
C14	8138(4)	3320(2)	8814(7)	65.7(11)
C15	9283(4)	3516(3)	8584(8)	75.9(14)

Table 3 Anisotropic Displacement Parameters (Å^2×10^3) for exp_256_auto. The Anisotropic displacement factor exponent takes the form: $-2\pi^2[h^2a^{*2}U_{11}+2hka^*b^*U_{12}+…]$.

Atom	U_{11}	U_{22}	U_{33}	U_{23}	U_{13}	U_{12}

I23	57.4(2)	42.87(19)	87.1(2)	0.48(11)	22.50(15)	-11.59(10)
S10	39.4(4)	42.0(5)	88.9(7)	1.3(4)	18.5(4)	-6.1(4)
N7	31.6(13)	38.2(15)	66.7(18)	3.5(13)	14.5(12)	1.6(12)
N8	39.0(14)	37.5(15)	65.2(18)	0.8(13)	12.1(13)	1.2(12)
O21	65(2)	41.3(15)	148(3)	-5.2(19)	36(2)	-11.2(15)
F19	86(2)	126(3)	126(3)	18(2)	52(2)	-9(2)
N12	32.3(14)	41.7(16)	88(2)	1.3(16)	16.4(15)	-1.9(13)
C1	31.6(15)	37.2(16)	50.1(17)	3.6(13)	5.9(13)	0.4(13)
C4	42.1(17)	38.6(17)	59(2)	3.8(15)	11.6(15)	-7.0(15)
C2	32.6(16)	46(2)	72(2)	3.5(17)	14.7(15)	4.0(15)
C3	40.3(18)	38.3(18)	73(2)	-0.1(17)	11.8(16)	4.1(15)
C6	38.5(17)	39.9(18)	74(2)	-0.1(17)	20.6(16)	2.6(15)
C11	34.2(16)	44.4(18)	59(2)	0.9(15)	11.0(14)	-6.1(15)
C5	40.5(17)	45(2)	75(2)	-0.7(17)	24.3(17)	-4.3(16)
C9	39.3(17)	41.7(19)	64(2)	0.0(16)	9.7(16)	0.2(15)
C13	36.7(17)	35.8(18)	79(2)	-3.4(16)	14.4(17)	0.4(14)
C18	40.6(18)	56(2)	81(3)	1(2)	10.8(18)	-7.2(18)
C20	52(2)	41(2)	78(3)	-4.3(18)	15.0(18)	-3.5(18)
C17	53(2)	48(2)	100(3)	1(2)	31(2)	1.0(19)
C16	38.1(19)	50(2)	117(4)	-3(2)	21(2)	-6.6(18)
C22	59(2)	47(2)	93(3)	-9(2)	31(2)	4(2)
C14	53(2)	59(3)	83(3)	-7(2)	11(2)	-7(2)
C15	41(2)	69(3)	109(4)	-8(3)	-4(2)	-11(2)

Table 4 Bond Lengths for exp_256_auto.

Atom	Atom	Length/Å	Atom	Atom	Length/Å
I23	C4	2.102(3)	C4	C3	1.386(5)
S10	C11	1.771(4)	C4	C5	1.377(5)
S10	C9	1.744(4)	C2	C3	1.372(5)
N7	N8	1.361(4)	C6	C5	1.377(5)
N7	C1	1.427(4)	C9	C20	1.482(5)
N7	C11	1.409(4)	C13	C18	1.379(6)
N8	C9	1.275(5)	C13	C14	1.379(6)
O21	C20	1.200(5)	C18	C17	1.372(6)
F19	C17	1.349(6)	C20	C22	1.490(6)
N12	C11	1.258(5)	C17	C16	1.356(7)
N12	C13	1.419(5)	C16	C15	1.370(8)
C1	C2	1.385(5)	C14	C15	1.391(7)
C1	C6	1.395(5)			

Table 5 Bond Angles for exp_256_auto.

Atom	Atom	Atom	Angle/°	Atom	Atom	Atom	Angle/°

C9	S10	C11	88.21(17)	C4	C5	C6	120.3(3)
N8	N7	C1	117.3(3)	N8	C9	S10	116.3(3)
N8	N7	C11	115.4(3)	N8	C9	C20	123.5(3)
C11	N7	C1	127.3(3)	C20	C9	S10	120.1(3)
C9	N8	N7	112.1(3)	C18	C13	N12	119.9(4)
C11	N12	C13	118.7(3)	C14	C13	N12	120.6(4)
C2	C1	N7	122.9(3)	C14	C13	C18	119.5(4)
C2	C1	C6	119.2(3)	C17	C18	C13	118.8(4)
C6	C1	N7	117.9(3)	O21	C20	C9	118.9(4)
C3	C4	I23	119.6(3)	O21	C20	C22	123.9(4)
C5	C4	I23	120.5(3)	C9	C20	C22	117.1(3)
C5	C4	C3	119.9(3)	F19	C17	C18	118.7(5)
C3	C2	C1	120.5(3)	F19	C17	C16	117.6(4)
C2	C3	C4	120.1(3)	C16	C17	C18	123.7(5)
C5	C6	C1	120.0(3)	C17	C16	C15	116.8(4)
N7	C11	S10	107.8(3)	C13	C14	C15	119.2(5)
N12	C11	S10	126.9(3)	C16	C15	C14	122.1(4)
N12	C11	N7	125.2(3)				

Table 6 Torsion Angles for exp_256_auto.

A	B	C	D	Angle/°	A	B	C	D	Angle/°
I23	C4	C3	C2	179.1(3)	C2	C1	C6	C5	-1.1(6)
I23	C4	C5	C6	179.9(3)	C3	C4	C5	C6	0.5(6)
S10	C9	C20	O21	0.4(6)	C6	C1	C2	C3	0.2(6)
S10	C9	C20	C22	-178.6(3)	C11	S10	C9	N8	-2.8(3)
N7	N8	C9	S10	1.0(4)	C11	S10	C9	C20	175.7(3)
N7	N8	C9	C20	-177.4(3)	C11	N7	N8	C9	2.1(5)
N7	C1	C2	C3	-178.0(3)	C11	N7	C1	C2	-9.2(5)
N7	C1	C6	C5	177.1(3)	C11	N7	C1	C6	172.6(4)
N8	N7	C1	C2	171.7(3)	C11	N12	C13	C18	-91.6(5)
N8	N7	C1	C6	-6.5(5)	C11	N12	C13	C14	91.9(5)
N8	N7	C11	S10	-4.0(4)	C5	C4	C3	C2	-1.5(6)
N8	N7	C11	N12	175.1(4)	C9	S10	C11	N7	3.6(3)
N8	C9	C20	O21	178.7(4)	C9	S10	C11	N12	-175.5(4)
N8	C9	C20	C22	-0.3(6)	C13	N12	C11	S10	1.3(6)
F19	C17	C16	C15	-177.5(4)	C13	N12	C11	N7	-177.6(3)
N12	C13	C18	C17	-175.9(4)	C13	C18	C17	F19	177.0(4)
N12	C13	C14	C15	177.0(4)	C13	C18	C17	C16	-1.5(7)
C1	N7	N8	C9	-178.7(3)	C13	C14	C15	C16	-1.0(8)
C1	N7	C11	S10	176.9(3)	C18	C13	C14	C15	0.5(7)

Table 6 Torsion Angles for exp_256_auto.

A	B	C	D	Angle/°	A	B	C	D	Angle/°
C1	N7	C11	N12	-4.1(6)	C18	C17	C16	C15	1.0(7)
C1	C2	C3	C4	1.1(6)	C17	C16	C15	C14	0.3(7)
C1	C6	C5	C4	0.8(6)	C14	C13	C18	C17	0.7(6)

Table 7 Hydrogen Atom Coordinates (Å×10^4) and Isotropic Displacement Parameters (Å^2×10^3) for exp_256_auto.

Atom	*x*	*y*	*z*	U(eq)
H2	4869.27	1995.07	7296.98	60
H3	3943.26	948.84	7477.82	61
H6	2043.72	2957.77	8647.3	60
H5	1098.6	1907.45	8746.78	62
H18	6860.34	3172.37	4626.45	71
H16	10308.08	3704.07	6778.91	81
H22A	2177.78	5301.56	9468.53	97
H22B	1837.07	4621.26	8364.3	97
H22C	1600.55	5340.2	7412.68	97
H14	7990.42	3276.72	9956.74	79
H15	9892.5	3610.26	9588.59	91

Crystal data for (Z)-1-(4-phenyl-5-(p-tolylimino)-4,5-dihydro-1,3,4-thiadiazol-2-yl)ethan-1-one

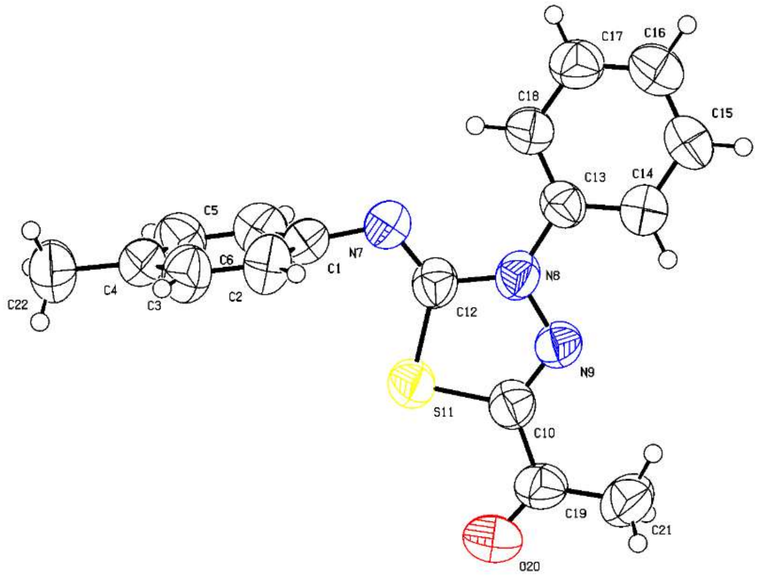

Table 1 Crystal data and structure refinement for exp_260_auto "Deposition Number 2178551".

Identification code	exp_260_auto
Empirical formula	$C_{17}H_{15}N_3OS$
Formula weight	309.38
Temperature/K	297.00(10)
Crystal system	orthorhombic
Space group	$P2_12_12_1$
a/Å	5.3058(4)
b/Å	8.6595(6)
c/Å	34.366(2)
α/°	90
β/°	90
γ/°	90
Volume/Å^3	1578.97(19)
Z	4
ρ_{calc}g/cm^3	1.301
μ/mm^{-1}	1.857
F(000)	648.0
Crystal size/mm^3	0.6 × 0.6 × 0.6
Radiation	Cu Kα (λ = 1.54184)

2Θ range for data collection/°	10.296 to 155.438
Index ranges	-5 ≤ h ≤ 6, -10 ≤ k ≤ 9, -41 ≤ l ≤ 41
Reflections collected	8336
Independent reflections	2872 [R_{int} = 0.0581, R_{sigma} = 0.0479]
Data/restraints/parameters	2872/0/201
Goodness-of-fit on F^2	0.746
Final R indexes [I>=2σ (I)]	R_1 = 0.0617, wR_2 = 0.1639
Final R indexes [all data]	R_1 = 0.0734, wR_2 = 0.1877
Largest diff. peak/hole / e $Å^{-3}$	0.44/-0.21
Flack parameter	0.02(3)

Table 2 Fractional Atomic Coordinates (×10^4) and Equivalent Isotropic Displacement Parameters ($Å^2$×10^3) for exp_260_auto. U_{eq} is defined as 1/3 of the trace of the orthogonalised U_{IJ} tensor.

Atom	*x*	*y*	*z*	U(eq)
S11	-95(2)	6332.2(12)	3448.6(3)	76.1(4)
N9	1584(7)	3868(4)	3115.5(9)	68.3(9)
N8	3005(7)	4046(4)	3443.3(9)	68.4(9)
O20	-3407(8)	6039(5)	2756.8(12)	103.3(12)
N7	3436(9)	5641(5)	3999.3(11)	81.0(11)
C13	4891(8)	2903(5)	3516.8(11)	64.6(9)
C1	2568(9)	6946(6)	4207.1(13)	72.2(11)
C12	2363(9)	5320(5)	3677.7(12)	67.9(10)
C18	6603(10)	3025(5)	3815.3(13)	75.6(12)
C10	-67(10)	4944(5)	3081.9(11)	69.6(10)
C19	-1907(10)	4988(6)	2761.9(14)	77.3(12)
C4	1070(11)	9460(6)	4663.2(14)	81.7(13)
C6	3917(11)	8301(6)	4206.3(16)	85.3(13)
C17	8426(11)	1903(6)	3864.9(15)	83.8(13)
C14	5028(10)	1634(6)	3272.8(15)	83.6(12)
C2	430(11)	6876(7)	4437.0(16)	88.3(14)
C5	3167(12)	9540(6)	4430.7(17)	88.9(14)
C15	6855(12)	531(6)	3334.3(18)	95.6(16)
C3	-257(12)	8124(7)	4661.0(15)	89.8(14)
C16	8543(12)	649(7)	3629.2(17)	87.2(14)
C21	-1832(14)	3752(7)	2464.5(15)	97.0(17)
C22	282(17)	10797(7)	4922.8(18)	118(2)

Table 3 Anisotropic Displacement Parameters (Å^2×10^3) for exp_260_auto. The Anisotropic displacement factor exponent takes the form: $-2\pi^2[h^2a^{*2}U_{11}+2hka^*b^*U_{12}+\ldots]$.

Atom	U_{11}	U_{22}	U_{33}	U_{23}	U_{13}	U_{12}
S11	95.7(8)	64.5(6)	68.2(6)	0.9(4)	-6.4(6)	10.4(6)
N9	81(2)	68.6(19)	55.4(17)	3.2(14)	-3.0(17)	4.4(18)
N8	83(2)	67.7(18)	54.2(16)	-3.5(15)	0.1(16)	0.9(17)
O20	111(3)	102(3)	97(2)	8(2)	-26(2)	27(2)
N7	89(2)	89(2)	66(2)	-12.4(19)	-10(2)	16(2)
C13	69(2)	61.7(19)	63(2)	4.4(14)	6(2)	7(2)
C1	81(3)	78(2)	58(2)	-4.1(19)	-9(2)	8(2)
C12	81(3)	67(2)	56(2)	0.5(17)	7.3(19)	1(2)
C18	87(3)	71(2)	69(2)	1.4(19)	-7(2)	12(2)
C10	82(3)	68(2)	58.4(19)	3.6(16)	5(2)	5(2)
C19	86(3)	76(2)	69(2)	12(2)	-11(2)	6(2)
C4	98(3)	79(3)	68(2)	-10(2)	-16(3)	14(3)
C6	85(3)	88(3)	83(3)	6(2)	7(3)	2(3)
C17	90(3)	83(3)	79(3)	9(2)	-9(3)	4(3)
C14	87(3)	82(3)	82(3)	-14(2)	-9(3)	9(3)
C2	95(3)	86(3)	84(3)	-18(2)	10(3)	-9(3)
C5	103(4)	69(3)	94(3)	2(2)	-3(3)	1(3)
C15	103(4)	73(3)	110(4)	-12(3)	-10(3)	25(3)
C3	96(3)	97(3)	77(3)	-13(2)	11(3)	2(3)
C16	87(3)	75(3)	99(3)	10(3)	-1(3)	9(3)
C21	125(5)	96(3)	69(3)	-4(3)	-21(3)	3(3)
C22	161(6)	92(3)	102(4)	-27(3)	-12(4)	32(4)

Table 4 Bond Lengths for exp_260_auto.

Atom	Atom	Length/Å	Atom	Atom	Length/Å
S11	C12	1.757(5)	C1	C2	1.384(7)
S11	C10	1.742(4)	C18	C17	1.382(7)
N9	N8	1.364(5)	C10	C19	1.471(7)
N9	C10	1.284(6)	C19	C21	1.481(7)
N8	C13	1.430(5)	C4	C5	1.372(9)
N8	C12	1.408(5)	C4	C3	1.354(8)
O20	C19	1.209(6)	C4	C22	1.520(7)
N7	C1	1.414(6)	C6	C5	1.380(8)
N7	C12	1.274(5)	C17	C16	1.356(8)
C13	C18	1.374(6)	C14	C15	1.377(7)
C13	C14	1.384(6)	C2	C3	1.376(7)
C1	C6	1.375(8)	C15	C16	1.356(8)

Table 5 Bond Angles for exp_260_auto.

Atom	Atom	Atom	Angle/°	Atom	Atom	Atom	Angle/°
C10	S11	C12	88.5(2)	N9	C10	C19	122.6(4)
C10	N9	N8	111.7(3)	C19	C10	S11	121.2(4)
N9	N8	C13	117.0(3)	O20	C19	C10	117.9(4)
N9	N8	C12	115.3(3)	O20	C19	C21	123.5(5)
C12	N8	C13	127.7(4)	C10	C19	C21	118.7(4)
C12	N7	C1	117.9(4)	C5	C4	C22	121.8(6)
C18	C13	N8	122.7(4)	C3	C4	C5	117.5(5)
C18	C13	C14	118.6(4)	C3	C4	C22	120.7(6)
C14	C13	N8	118.7(4)	C1	C6	C5	120.8(5)
C6	C1	N7	120.8(5)	C16	C17	C18	121.4(5)
C6	C1	C2	117.7(5)	C15	C14	C13	119.7(5)
C2	C1	N7	121.3(5)	C3	C2	C1	120.1(5)
N8	C12	S11	108.3(3)	C4	C5	C6	121.3(5)
N7	C12	S11	127.7(3)	C16	C15	C14	121.8(5)
N7	C12	N8	124.0(4)	C4	C3	C2	122.4(5)
C13	C18	C17	120.0(4)	C15	C16	C17	118.4(5)
N9	C10	S11	116.2(3)				

Table 6 Torsion Angles for exp_260_auto.

A	B	C	D	Angle/°	A	B	C	D	Angle/°
S11	C10	C19	O20	1.6(7)	C1	C2	C3	C4	1.0(9)
S11	C10	C19	C21	-178.4(4)	C12	S11	C10	N9	-1.2(4)
N9	N8	C13	C18	173.6(4)	C12	S11	C10	C19	176.6(4)
N9	N8	C13	C14	-5.1(6)	C12	N8	C13	C18	-8.9(7)
N9	N8	C12	S11	-2.1(4)	C12	N8	C13	C14	172.3(4)
N9	N8	C12	N7	178.0(4)	C12	N7	C1	C6	-103.6(6)
N9	C10	C19	O20	179.2(4)	C12	N7	C1	C2	80.6(6)
N9	C10	C19	C21	-0.7(7)	C18	C13	C14	C15	0.2(8)
N8	N9	C10	S11	0.2(5)	C18	C17	C16	C15	1.5(9)
N8	N9	C10	C19	-177.5(4)	C10	S11	C12	N8	1.8(3)
N8	C13	C18	C17	-178.2(4)	C10	S11	C12	N7	-178.3(5)
N8	C13	C14	C15	179.0(5)	C10	N9	N8	C13	179.0(4)
N7	C1	C6	C5	-175.4(5)	C10	N9	N8	C12	1.3(5)
N7	C1	C2	C3	174.7(5)	C6	C1	C2	C3	-1.3(8)
C13	N8	C12	S11	-179.6(3)	C14	C13	C18	C17	0.6(7)
C13	N8	C12	N7	0.5(7)	C14	C15	C16	C17	-0.8(9)

Table 6 Torsion Angles for exp_260_auto.

A	B	C	D	Angle/°	A	B	C	D	Angle/°
C13	C18	C17	C16	-1.4(8)	C2	C1	C6	C5	0.6(8)
C13	C14	C15	C16	0.0(9)	C5	C4	C3	C2	0.0(9)
C1	N7	C12	S11	1.0(7)	C3	C4	C5	C6	-0.7(8)
C1	N7	C12	N8	-179.1(4)	C22	C4	C5	C6	177.7(6)
C1	C6	C5	C4	0.4(9)	C22	C4	C3	C2	-178.5(6)

Table 7 Hydrogen Atom Coordinates (Å×10[4]) and Isotropic Displacement Parameters (Å[2]×10[3]) for exp_260_auto.

Atom	*x*	*y*	*z*	U(eq)
H18	6534.56	3864.28	3983.96	91
H6	5353.32	8383.85	4052.52	102
H17	9598.1	2009.46	4064.28	101
H14	3892.72	1526.95	3068.37	100
H2	-545.1	5983.52	4440.09	106
H5	4103.14	10448.06	4424.15	107
H15	6933.09	-318.7	3169.06	115
H3	-1684.93	8047.39	4816.66	108
H16	9752.77	-112.69	3668.77	105
H21A	-3520.89	3442.44	2401.25	145
H21B	-918.68	2881.69	2564.7	145
H21C	-1009.75	4130.19	2234.67	145
H22A	1302.18	11683.86	4865.32	177
H22B	503.94	10512.57	5190.58	177
H22C	-1457.92	11039.3	4876.37	177

Computational Details

We started our calculations with an exploratory conformational analysis of the reactants using the Grimme's XTB [1] and CREST [2,3] approaches using ethanol as implicit solvent [4]. The best structures from this conformational search were used as an initial geometry input for the more accurate DFT calculations. These DFT calculations were performed using the program ORCA (version 5.0.3) [5]. All geometry relaxations used the well-known hybrid exchange-correlation functional B3LYP, [6,7,8,9] supplemented with latest van der Waals (vdW) corrections due to Grimme et al. known as D4. [10,11] We used the triple-zeta polarized basis set (def2-TZVP) from Ahlrichs and coworkers. [12,13] The optimizations were speed up by using the RIJCOSX approximation. We used "TightSCF" keyword for all geometry relaxations for accurate evaluation of the ionic forces. Thus, the adopted computational protocol for the optimizations was B3LYP-D4/def2-TZVP. After geometry optimizations, we carried out a Hessian calculation and no imaginary frequencies were found in the vibrational analysis. This confirms that all the relaxed geometries presented here are true stationary points on the electronic ground state potential energy surface. Implicit solvent (ethanol) was considered at the SMD level to mimic the experimental conditions. An explicit ethanol molecule was used to further investigate the role of solvent. The typical ORCA keywords used in our input files were

!B3LYP D4 def2-TZVP def2/J RIJCOSX TightSCF Opt KeepDens NumFreq

%cpcm

smd true

smdsolvent "ethanol"

end

Wavefunction analysis and post-processing of the *ab initio* calculations were carried out with the Multiwfn (version 3.3.8) software [14]. Calculation of the average local ionization energies followed Ref. [15] while the local electron affinity followed Ref. [16]. Natural Population Analysis (NPA atomic charges) were calculated with the JANPA software [17,18]. The ADCH [19] atomic charges were calculated with Multiwfn. Visualization and rendering were done with Chemcraft [20] and Multiwfn codes.

REFERENCES:

[1] C. Bannwarth, E. Caldeweyher, S. Ehlert, A. Hansen, P. Pracht, J. Seibert, S. Spicher, S. Grimme *WIREs Comput. Mol. Sci.*, **2020**, 11, e01493. DOI: 10.1002/wcms.1493

[2] S. Grimme, *J. Chem. Theory Comput.*, **2019**, 155, 2847-2862. DOI: 10.1021/acs.jctc.9b00143

[3] P. Pracht, F. Bohle, S. Grimme, *Phys. Chem. Chem. Phys.*, **2020**, 22, 7169-7192. DOI: 10.1039/C9CP06869D

[4] S. Ehlert, M. Stahn, S. Spicher, S. Grimme, *J. Chem. Theory Comput.*, **2021**, 17, 4250-4261 DOI: 10.1021/acs.jctc.1c00471

[5] Neese, F. The ORCA Program System. *WIREs Comput Mol Sci* **2012**, *2* (1), 73–78. https://doi.org/10.1002/wcms.81.

[6] A.D. Becke, J.Chem.Phys. 98 (1993) 5648-5652

[7] C. Lee, W. Yang, R.G. Parr, Phys. Rev. B 37 (1988) 785-789

[8] S.H. Vosko, L. Wilk, M. Nusair, Can. J. Phys. 58 (1980) 1200-1211

[9] P.J. Stephens, F.J. Devlin, C.F. Chabalowski, M.J. Frisch, J.Phys.Chem. 98 (1994) 11623-11627

[10] E. Caldeweyher, C. Bannwarth and S. Grimme, *J. Chem. Phys.*, **2017**, 147, 034112. DOI: 10.1063/1.4993215

[11] C. Bannwarth, S. Ehlert and S. Grimme., *J. Chem. Theory Comput.*, **2019**, 15, 1652-1671. DOI: 10.1021/acs.jctc.8b01176

[12] Weigend, F.; Ahlrichs, R. Balanced Basis Sets of Split Valence, Triple Zeta Valence and Quadruple Zeta Valence Quality for H to Rn: Design and Assessment of Accuracy. *Phys. Chem. Chem. Phys.* **2005**, *7* (18), 3297. https://doi.org/10.1039/b508541a.

[13] Weigend, F. Accurate Coulomb-Fitting Basis Sets for H to Rn. *Phys. Chem. Chem. Phys.* **2006**, *8* (9), 1057. https://doi.org/10.1039/b515623h.

[14] Lu, T.; Chen, F. Multiwfn: A multifunctional wavefunction analyzer. Journal of Computational Chemistry 2012, 33, 580–592.

[15] Average local ionization energies on the molecular surfaces of aromatic systems as guides to chemical reactivity. Authors: Per Sjoberg, Jane S. Murray, Tore Brinck, and Peter Politzer. Canadian Journal of Chemistry, 68, 1440 (1990), https://doi.org/10.1139/v90-220

[16] J. Molecular Modelling, 9, 342-347 (2003). Local molecular properties and their use in predicting reactivity. Bernd Ehresmann 1, Bodo Martin, Anselm H C Horn, Timothy Clark. DOI: 10.1007/s00894-003-0153-x

[17] T. Yu. Nikolaienko, L. A. Bulavin, *Localized orbitals for optimal decomposition of molecular properties*, International Journal of Quantum Chemistry (2019), 119 : e25798, DOI: 10.1002/qua.25798

[18] T.Y.Nikolaienko, L.A.Bulavin, D.M.Hovorun, *JANPA: an open source cross-platform implementation of the Natural Population Analysis on the Java platform,* Computational and Theoretical Chemistry (2014), V.1050, P.15-22, DOI: 10.1016/j.comptc.2014.10.002, http://janpa.sourceforge.net

[19] Tian Lu and Feiwu Chen. Atomic dipole moment corrected Hirshfeld population method *J. Theor. Comput. Chem.*, 11, 163 (2012), https://doi.org/10.1142/S0219633612500113

[20] Chemcraft - graphical software for visualization of quantum chemistry computations. https://www.chemcraftprog.com
